# The extracellular Leucine-Rich Repeat superfamily; a comparative survey and analysis of evolutionary relationships and expression patterns

**DOI:** 10.1186/1471-2164-8-320

**Published:** 2007-09-14

**Authors:** Jackie Dolan, Karen Walshe, Samantha Alsbury, Karsten Hokamp, Sean O'Keeffe, Tatsuya Okafuji, Suzanne FC Miller, Guy Tear, Kevin J Mitchell

**Affiliations:** 1 Smurfit Institute of Genetics, Trinity College Dublin, Dublin 2, Ireland; 2 MRC Centre for Developmental Neurobiology, New Hunts House, Guys Campus, King's College London SE1 1UL, UK

## Abstract

**Background:**

Leucine-rich repeats (LRRs) are highly versatile and evolvable protein-ligand interaction motifs found in a large number of proteins with diverse functions, including innate immunity and nervous system development. Here we catalogue all of the extracellular LRR (eLRR) proteins in worms, flies, mice and humans. We use convergent evidence from several transmembrane-prediction and motif-detection programs, including a customised algorithm, LRRscan, to identify eLRR proteins, and a hierarchical clustering method based on TribeMCL to establish their evolutionary relationships.

**Results:**

This yields a total of 369 proteins (29 in worm, 66 in fly, 135 in mouse and 139 in human), many of them of unknown function. We group eLRR proteins into several classes: those with only LRRs, those that cluster with Toll-like receptors (Tlrs), those with immunoglobulin or fibronectin-type 3 (FN3) domains and those with some other domain. These groups show differential patterns of expansion and diversification across species. Our analyses reveal several clusters of novel genes, including two *Elfn *genes, encoding transmembrane proteins with eLRRs and an FN3 domain, and six genes encoding transmembrane proteins with eLRRs only (the Elron cluster). Many of these are expressed in discrete patterns in the developing mouse brain, notably in the thalamus and cortex. We have also identified a number of novel fly eLRR proteins with discrete expression in the embryonic nervous system.

**Conclusion:**

This study provides the necessary foundation for a systematic analysis of the functions of this class of genes, which are likely to include prominently innate immunity, inflammation and neural development, especially the specification of neuronal connectivity.

## Background

Leucine-rich repeats (LRRs) are protein-ligand interaction motifs found in a large number of proteins of diverse structure, localization and function in bacteria, fungi, plants and animals [1]. Many of these have well-known functions in the innate immune system [2]. Many others, especially those with extracellular LRRs (eLRRs), are involved in various aspects of nervous system development [3]. In both cases, the nature of the LRR motifs is important for generating a diversity of interactions, with exogenous factors in the immune system and with the huge number of different cell types in the developing nervous system. The structure of LRR motifs and their arrangement in repetitive stretches of variable length generate a versatile and highly evolvable framework for the binding of diverse proteins and non-protein ligands.

Seven classes of LRR have been defined [1]; (these have been referred to as LRR "subfamilies" [4]; we use the term subfamily here in the phylogenetic sense to refer to sets of closely-related genes). Within animals, four separate types are recognised, three typically intracellular and one extracellular. Whether all these different classes are evolutionarily related by descent or represent convergent evolution is open to debate [1] but they all share a characteristic structure. Each repeat is typically 19–29 amino acids long and has a well-conserved N-terminal stretch of 9–12 amino acids that is characterized by precisely-positioned hydrophobic residues (usually leucines) and that forms a β-strand and a C-terminal stretch of 10–19 amino acids that is more variable in length, sequence and structure. The arrangement of multiple repeats in tandem generates a horseshoe-shaped solenoidal structure, with the β-strands stacking to form the concave surface and the variable stretches forming the convex surface [1,5-7]. Most LRR regions typically also have both N-terminal and C-terminal cap regions, which shield the hydrophobic core of the LRR structure. In extracellular proteins these regions (LRR-NT and LRR-CT domains, of which several subtypes exist) are defined by precisely positioned cysteine residues [4].

LRR proteins, both intracellular and extracellular, have well-characterized functions in the innate immune system that are similar from plants to mammals [2]. The extracellular LRR (eLRR) proteins in animals include the Toll-like receptors (TLRs), a family of transmembrane proteins characterized by an LRR region, a transmembrane (TM) domain and a cytoplasmic Toll/IL-1 receptor (TIR) domain. This family has expanded in vertebrates to allow detection of a diverse set of antigens [8]. In flies, the TLR family has also expanded, where, in addition to roles in immunity for some of these proteins [9], many are required for various aspects of embryonic and nervous system development [10-13]. Tol-1 in worms is also important in development, possibly contributing to a code of molecules defining neuronal connectivity [14,15]. Recent reports indicate that some mammalian TLR genes may also be expressed and function in neurons [16,17].

A large number of other eLRR proteins have been implicated in various aspects of neural development, genetically in flies [18-20] and in mammals in assays of neurite outgrowth, [21-24], fasciculation [25] and/or synapse formation [26,27]. Some of these contain, in addition to the extracellular LRR domain, immunoglobulin (Ig) or fibronectin type-3 (FN3) domains (for review see [3]). In some cases, the functions of eLRR proteins are mediated by homophilic interactions [25,28-30]. In other cases they are mediated by the binding of other proteins *in cis *[31-33] and *in trans *[27,34-36]. Several eLRR proteins have been found to modulate the signaling of various growth factor pathways (e.g., [37-41]).

Surprisingly, apart from the TLR genes [42] and small secreted proteoglycans [43], relatively few eLRR genes have been studied genetically in mice. Among the ones that have, examples of phenotypic effects in the nervous system include increased plasticity, sprouting and nerve regeneration [44], and defects in axon guidance and cell migration [45], learning and memory [46], myelination [47,48] and neuronal survival [35].

The importance of this class of proteins for nervous system development in humans is apparent from the large number of examples implicated in neurological or psychiatric disorders (reviewed in [49]). These include epilepsy [50], Tourette's syndrome [51], night blindness [52], congenital insensitivity to pain (with mental retardation) [53], and possible links to Alzheimer's disease [54].

Despite the growing number of eLRR proteins implicated in nervous system development or disease this family of proteins has received far less attention as a class than other better characterized families like the immunoglobulin [55,56] and cadherin [57] superfamilies. In particular, there have been no systematic surveys of the genomic complement of these proteins or investigation of their evolutionary relationships. We therefore set out to catalogue the entire extracellular leucine-rich repeat proteome of four organisms: *Caenorhabditis elegans*, *Drosophila melanogaster*, *Mus musculus *and *Homo sapiens*. We used a hierarchical clustering system to analyse within and between-species relationships, revealing independent diversification and expansion of subfamilies in each species and rapid sequence divergence. These analyses highlight the large number of novel, uncharacterized eLRR proteins in each of these genomes, including several novel subfamilies. A number of these show highly restricted expression in the nervous system in mouse or fly.

## Results

### Bioinformatics strategy

**Figure 1:**
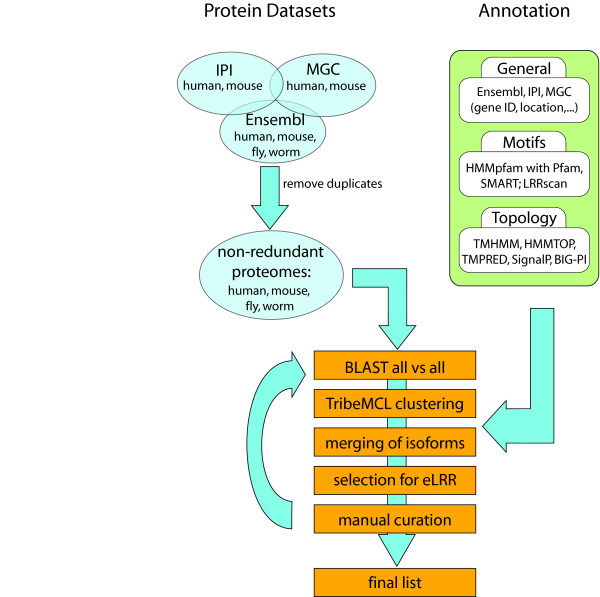
**Bioinformatics pipeline**. Figure shows starting datasets (blue), annotation programs (green) and clustering pipeline (orange) used to generate final eLRR dataset.

We began by obtaining whole proteome sets of known and predicted proteins from each of the four species, as described in Methods. Our initial approach was to filter the starting proteome datasets using transmembrane (TM) and signal peptide prediction programs (TMHMM [58] and SignalP [59]) to identify transmembrane or secreted proteins and then to filter that set using a motif recognition program (Pfam [60]) to identify the subset with motifs of interest. However, this approach using serial filters missed a number of known axon guidance molecules because TMHMM or SignalP misclassified them or Pfam did not detect specific motifs. We therefore included a number of other TM-prediction and motif-recognition programs, including a customised program to look for LRR domains (LRRscan, see below) in the pipeline. In addition, we first performed a clustering step using TribeMCL [61] on the entire proteome sets so as to identify related proteins even where these programs failed to detect specific motifs or architectures. Rather than using any of these programs or a combination of them as strict filters we generated a database containing all the results that could be browsed or searched using various criteria to extract particular gene families of interest (Figure 1).

### Clustering

TribeMCL is a program designed to cluster proteins into related families based on simultaneous examination of all pairwise BLAST similarity scores [61]. This program uses a Markov cluster algorithm that is particularly well suited to cluster highly divergent proteins with repeated domains into separate subfamilies, a task for which multiple alignment programs are not appropriate. The Markov cluster algorithm is based on simulated 'flow' through a network or graph, where each node is a gene and each connection is weighted by the symmetric pairwise BLAST score. A random walk of a certain length from node to node through this network, which begins within a small cluster of interconnected genes will have a higher probability of ending up within that cluster than crossing to a gene that is only related to one of them. The results of many simulated random walks thus define the clusters. Each time this process is reiterated the links within the clusters that emerge are given a higher weighting and the links that were not used are downgraded. Multiple rounds of this process eventually lead to completely separate clusters. There are two parameters that can be varied that affect the clustering; the first is the e-value cutoff of the BLAST scores that are used, which determines the initial set of connections considered. The second is the inflation parameter; this determines how strongly the links are upgraded with each iteration. A higher inflation parameter increases the 'granularity' of the output; i.e., it generates a larger number of smaller clusters. We used a number of e-value cutoffs (from e^-10 ^to e^-40^) and inflation parameters (1.2, 2, 3, 4 and 5) and compared the output produced.

The output from TribeMCL, for any given e-value cutoff and inflation parameter can be viewed as a list of genes organized into clusters with a number assigned to each cluster (lower numbers have more members). We reasoned that hierarchical sorting of genes across various inflation parameters should yield a tree-like structure, with larger clusters at low inflation parameters splitting into more discrete clusters at higher inflation parameters. At each e-value cutoff we therefore sorted the list of genes first at inflation parameter 1.2, then 2, then 3, 4 and 5. For the most part, increasing inflation parameter does lead to splitting of large clusters into smaller clusters and yields a tree-like arrangement of genes with relationships apparent across various levels (but see discussion on "LRR_Tollkin" group below).

### Identification of LRR motifs

To identify LRR proteins, the database was searched for all genes containing at least one LRR, LRR-NT or LRR-CT predicted by either SMART or Pfam. The cutoff values used were based on analysis of the results for proteins with known architecture (see Methods for details). This analysis yielded a total of 2,698 entries. These include both genes with intracellular LRRs and those with extracellular LRRs. It also contains isoforms for many genes. To screen out false positives we used the following criteria: if only one LRR was predicted in a gene and only by one of the programs and it was not predicted in either the mouse or human orthologue (for mammalian genes), or in other members of a closely-related cluster then it was considered a false positive and discarded.

### Identification of extracellular LRR proteins

Comparison of several TM-prediction programs suggests that TMHMM is the most reliable, although it is also the most selective [62]. A quick survey of some known TM receptors revealed that TMHMM failed to identify TM domains in several of them, including Robo2 in mammals and Kekkon2 and 3 in flies, for example. For that reason we also used two other programs, HMMTOP [63] and TMPred [64] to search for TM domains. At least one of these three programs successfully detected the TM domain in all the known TM receptors examined (while also increasing the number of false positives).

SignalP [59] was used to detect signal peptides. This suffered from poor prediction of 5' exons for many mammalian genes, which was solved by manual curation (see below). The GPI-prediction program BIG-PI [65] identified a small number of GPI-linked proteins, including all the known GPI-linked proteins such as Connectin, NgRs and Nyx (the latter in human but not mouse, as reported [66]). A number of other genes were tentatively assigned to the GPI-class by manual inspection based on the presence of a characteristic short C-terminal hydrophobic stretch (and a signal peptide).

In addition to examining the convergent evidence from these various programs to identify eLRR proteins we used three additional criteria. The first is the type of LRR predicted: extracellular proteins typically contain LRR types designated LRR_1, 2 or 3 by Pfam or LRR_typical by SMART, while the intracellular proteins have LRR_RI or LRR_sd22 (see [1]). Second, the prediction of an LRR-NT and/or LRR-CT domain was taken as evidence for extracellular localization. Third, especially at low e-value stringencies (e^-10^), the majority of extracellular LRR proteins cluster together with TribeMCL in one large group (and a few small ones), distinct from the intracellular proteins. Using these criteria in addition to the data from the prediction programs described above we collected what we believe is a comprehensive set of extracellular LRR proteins across worm, fly, mouse and human. We call these the eLRR proteome.

### Manual curation of extracellular sequences

To reduce the complexity of the final data set a single protein isoform was chosen for each gene and all others were removed (see Methods). Many peptides that we expected to be extracellular because of orthology, clustering or domain structure did not have a predicted signal peptide. Upon manual inspection of the sequences it was discovered that many gene predictions in Ensembl, especially for mammalian genes, were missing the 5'-most exon encoding the signal peptide. For many such genes we identified the 5' exon and the full coding sequence in a sequence from another database and/or by searching with an orthologous gene from mouse or human. In other cases the 5' predicted sequence extended past the apparent true methionine start codon, which could be recognised by conservation and the presence of the signal sequence.

We identified two fly genes that have been incorrectly annotated in Ensembl as two separate genes each. CG32637 and CG4187 represent the 5' and 3' ends of a cDNA encoded by AB134171, a new member of the Lgr3 family. Similarly, CG4054 and CG13487 represent the 5' and 3' ends of the fish-lips (fili) gene, encoded by AAV36870 [67] which is related to tartan and capricious [67,68]. We detected one similar mis-annotation in the worm database (pxn-1) and presume that this type of error may also have occurred for some mammalian sequences.

All the manually curated gene sequences are provided [see Additional File 1]. These curated sequences were fed back into the starting database and the BLAST and clustering analyses were re-performed to ensure that spurious results had not been generated by incorrect sequences.

### Defining consensus architectures

In order to derive a consensus architecture for each gene we compared the results of SMART and Pfam and the TM-prediction programs. Even at very low stringency some LRRs in proteins with known numbers of such repeats were missed by HMMpfam using the SMART and Pfam databases. This includes a number of somewhat degenerate LRRs in Lrrc8 proteins [69], for example, as well as atypical LRR-CT domains in small proteoglycans and G-protein-coupled receptors [4]. For this reason, using a similar strategy to Smits and colleagues [69], we wrote a customised program, LRRscan, to search for a more inclusive minimal consensus that defines LRRs as well as searching for consensus sequences derived from non-canonical LRR-CT domains (see Methods for details). LRRscan was successful in identifying all the predicted LRRs in Lrrc8 proteins, including atypical or degenerate ones [69], and additional LRRs in many other proteins that were not detected by HMMpfam with SMART or Pfam.

The output from LRRscan and HMMpfam was compared for all proteins [see Additional File 2] and a consensus architecture including number of LRRs and presence of LRR-NT and LRR-CT domains was derived by manual curation. The consensus matches the architecture of a number of eLRR proteins with published structures [5-7,70-72], allowing for semantic differences in how the LRRs are counted. The final LRR before the LRR-CT domain (CT1 subtype) often contains only the first subdomain of nine residues; following the convention of Matsushima and colleagues [49] we count this as one repeat rather than part of the LRR-CT domain, which in some cases may cause an apparent discrepancy with published reports. We also do not count in the total number of repeats putative LRRs which overlap with LRR-NT or LRR-CT domains, as has been done in some published cases [73,74].

**Figure 2:**
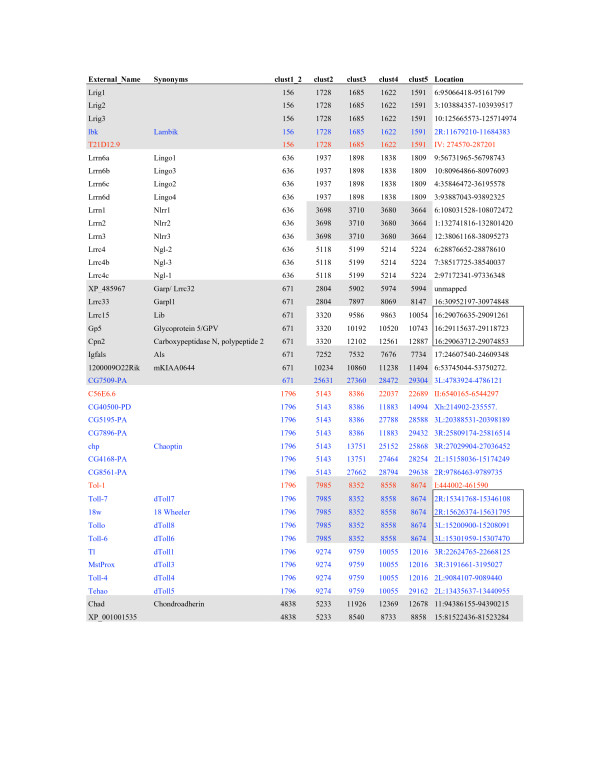
**Sample from list of all eLRR genes, hierarchically clustered at e^-40 ^cutoff**. Proteins have been sorted in this table based on the clustering output from TribeMCL. This has been done hierarchically across inflation parameters, starting at 1.2, then 2, 3, 4 and 5. For most proteins this yields a tree-like structure with cluster stringency increasing (and membership decreasing) from low inflation parameters to high. Numbers used to identify clusters are generated by TribeMCL with larger clusters having lower numbers. Proteins are colour-coded by species: black, mammalian; blue, fly; red, worm. For the mammalian proteins, only the mouse orthologue is listed. The table shows examples of clusters in the LRR_Ig/FN3 group with mouse, fly and worm orthologues (the Lrig subfamily) and with mouse paralogues only (the Lrrn6, Lrrn1–3 and Lrrc4 subfamilies, which cluster together at level 1.2). It also shows many of the proteins in the LRR_Tollkin group, with the hierarchical clustering apparent across inflation parameters and indicated by shading. One subfamily containing a known and novel member is shown at the bottom. Proteins encoded by genes located in tandem in the genome are boxed in the right-hand column. A complete list of all eLRR proteins is provided [see Additional File 3]. Lists clustered at the e^-25 ^and e^-10 ^cutoff levels are given [see Additional Files 4 and 5].

**Figure 3:**
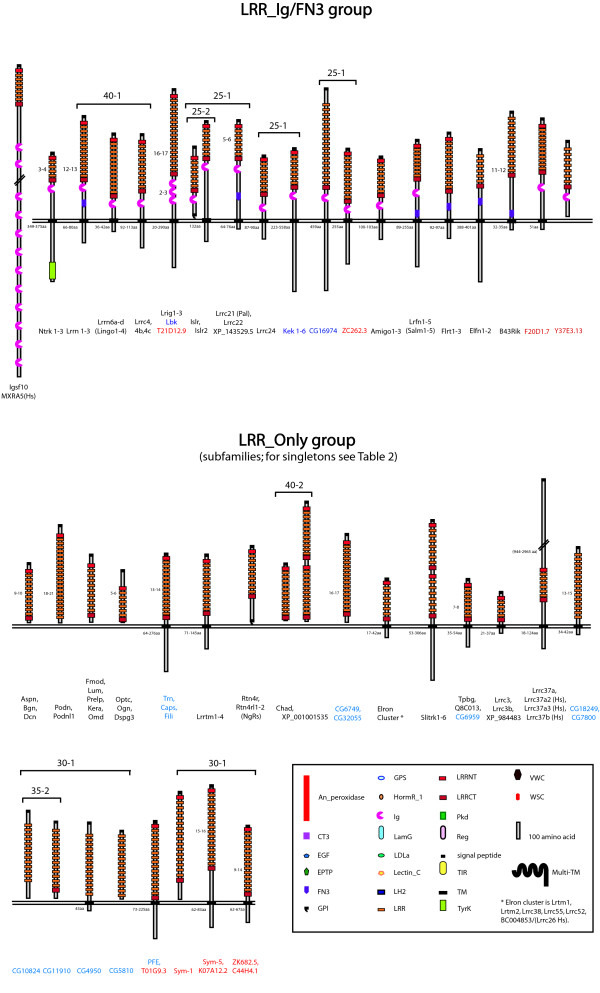
**eLRR protein predicted architectures (part 1)**. Consensus architectures are shown for all proteins in the LRR_Ig/FN3 group and for all proteins in subfamilies in the LRR_Only group. An additional set of LRR_Only singletons is listed separately in Table 1. Protein names are shown below the corresponding structures (black, mammalian; blue, fly; red, worm). All figures are drawn to scale (see Key). Consensus architectures were derived for single proteins and across subfamilies from convergent evidence from motif and topology prediction programmes. Where there is a range in number of predicted LRRs or other domains across members of a subfamily, this is indicated next to the domain. A range in length of the cytoplasmic domain is similarly indicated, where it exceeds 20 amino acids. Tightly clustered subfamilies (e.g., Slits, Amigos) are listed under a single consensus architecture. Clusters with more structurally diverse proteins are indicated by the brackets; the numbers refer to e-value and inflation parameter at which the proteins cluster in the MCL programme. See Key for more information.

**Figure 4:**
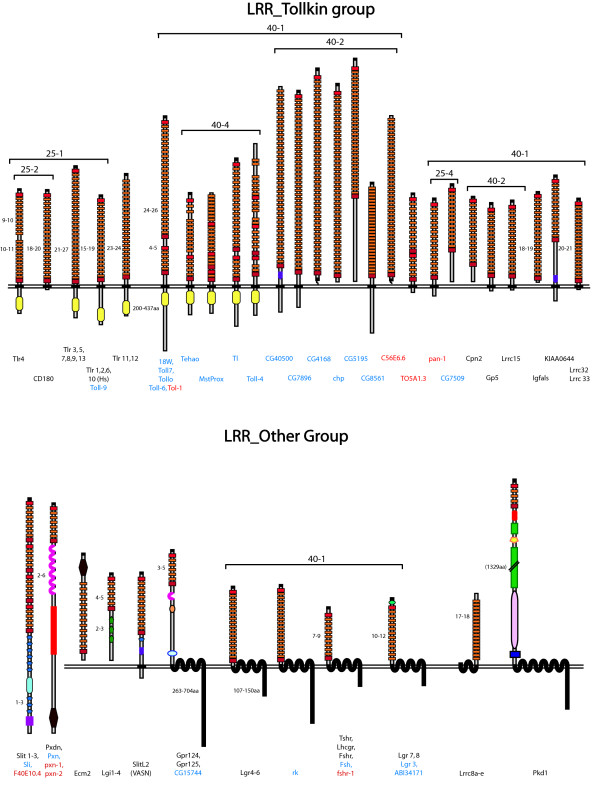
**eLRR protein predicted architectures (part 2)**. Consensus architectures are shown for all proteins in the LRR_Tollkin and LRR_Other groups. See Figure 3 legend for details.

A consensus topology for each protein was also derived by comparison of the signal peptide, GPI anchor and TM-prediction programs. A full list of all the eLRR proteins is provided [see Additional File 3] and a sample is shown in Figure 2. These are sorted hierarchically across inflation parameters at an e-value cutoff of -40. Clustering results at e-10 and e-25 are also presented [see Additional Files 4 and 5]. Figures 3 and 4 provide an overview of the consensus protein architectures of most of the eLRR proteins, arranged in subfamilies. A large number of singleton LRR_Only proteins are not shown in this diagram (these are listed separately in Table 1).

**Table 1:** List of LRR_Only singletons

**Symbol**	**Name/synonyms**	**Peptide length**	**Consensus architecture**
**Mammalian proteins**			
BC031901	novel	872	SS, 7LRR, TM
Cd14		366	SS, LRRNT, 11LRR, GPI
Gp1ba	Glycoprotein 1b, alpha polypeptide	734	SS, LRRNT, 8LRR, LRRCT1, TM
Gp1bb	Glycoprotein 1b, beta polypeptide	214	SS, LRRNT, 2LRR, LRRCT1, TM
Gp9	Glycoprotein 9	177	SS, LRRNT, 2LRR, LRRCT1, TM
Lrg1	Leucine-rich alpha-2-glycoprotein 1	342	SS, LRRNT, 9LRR, LRRCT2
Lrrc17		443	SS, LRRNT, 4LRR, LRRCT1, LRRNT, 3LRR, LRRCT1
Lrrc19		364	SS, LRRNT, 6LRR, LRRCT1, TM
Lrrc25		297	SS, 2LRR, LRRCT1, TM
Nepn	Nephrocan/5730521E12Rik	512	SS, LRRNT, 17LRR, LRRCT1
Nyx	Nyctalopin (mouse)	476	SS, LRRNT, 11LRR, LRRRCT1, TM
NYX	Nyctalopin (human)	481	SS, LRRNT, 12LRR, LRRRCT1, GPI
Omg	Oligodendrocyte myelin protein	440	SS, LRRNT, 7LRR, LRRCT2, GPI
Q7Z2Q7	Synleurin (human)	621	SS, LRRNT, 13LRR, LRRCT1, TM
Tsku	Tsukushi/Lrrc54	354	SS, LRRNT, 10LRR, LRRCT2

**Fly proteins**			
Con	Connectin	691	SS, LRRNT, 11LRR, LRRCT1, GPI
Gp150	Gp150	1051	SS, LRRNT, 15LRR, LRRCT2, TM
hfw	Halfway	611	SS, LRRNT, 4LRR, LRRNT, 2LRR, LRRCT1
wdp	windpipe	677	SS, LRRNT, 4LRR, LRRCT1, TM
CG1504		392	11LRR, LRRCT1, TM
CG4781		469	SS, LRRNT, 11LRR, LRRCT1, TM
CG5096		491	SS, LRRNT, 12LRR, LRRCT, TM
CG5541		463	SS, LRRNT, 6LRR, TM
CG5819		915	SS, LRRNT, 17LRR, LRRCT1, TM
CG5888		455	SS, LRRNT, 8LRR
CG7702		537	SS, LRRNT, 11LRR, LRRCT1, TM
CG8852		663	SS, 10LRR, LRRCT, TM
CG10148		329	SS, 9LRR
CG11136		799	SS, LRRNT, 13LRR, LRRCT1, TM
CG14351		1316	SS, LRRNT, 12LRR, LRRCT1, TM
CG14662		550	SS, 6LRR, TM
CG14762		470	SS, LRRNT, 14LRR, LRRCT1
CG15658		343	SS, LRRNT, 7LRR, LRRCT1, TM
CG17667		458	SS, LRRNT, 7LRR, TM
CG18095		548	SS, 18LRR, TM
CG18480		550	SS, LRRNT, 7LRR, LRRCT, TM
CG32372		817	SS, 23LRR

**Worm proteins**			
C02C6.3		369	SS, LRRNT, 8LRR, LRRCT1, GPI
C41C4.3		630	SS, 8LRR
F10F2.4		656	SS, LRRNT, 18LRR, LRRCT1, TM
F37E3.2		568	SS, LRRNT, 11LRR, TM
K03A1.2		586	SS, LRRNT, 9LRR, LRRCT1, TM
T22E7.1a		341	SS, 8LRR, LRRCT1, TM
T23G11.6		653	SS, LRRNT, 15LRR, LRRCT, TM
Y39A1A.7		187	SS, LRRNT, 4LRR
Y71F9B.8		542	SS, LRRNT, 14LRR, LRRCT1, TM
Y75B8A.5		448	SS, LRRNT, 6LRR, LRRCT1
Y76A2B.2		782	SS, LRRNT, 6LRR, GPI

List of singleton proteins in LRR_Only group not shown in Figure 3. For the mammalian proteins, only the mouse orthologue is listed, with the following exceptions: both human and mouse Nyctalopin (Nyx) are listed as they have different topologies (GPI-linked and TM, respectively) and *synleurin *is a human gene that has been pseudogenised in mouse.

### The eLRR superfamily

We categorized the eLRR proteins into four classes, based on their architecture and clustering. These are LRR_Ig/Fn3 (containing an Ig or FN3 domain but no other extracellular domains except LRRs), LRR_Tollkin (containing a cytoplasmic TIR domain or clustering with the Toll proteins), LRR_Other (containing some other domain, such as EGF repeats or a G-protein-coupled receptor domain) and LRR_Only (containing no other recognizable domain). These categories are broadly supported by the clustering results, although the LRR_Other group is clearly arbitrary and contains a number of unrelated subfamilies. The number of eLRR proteins in each of these classes in each of the four organisms studied is shown in Table 2. These are broken down into several categories, based on predicted localization: secreted, GPI-linked, type I transmembrane and multi-membrane spanning (all multi-membrane spanning proteins were classified into the LRR_Other group). Almost all of the LRR_Ig/FN3 group are associated with the plasma membrane, either as type I TM or GPI-linked proteins. In contrast, the LRR_Tollkin and LRR_Only groups contain a far higher percentage of secreted proteins. It is clear from an examination of these data that the eLRR superfamily has greatly expanded in mammals (>135 genes) and to a lesser extent, flies (66), compared to worms (29).

**Table 2:** Complement of eLRR proteins by group, localisation and species

**LRR_Ig/FN3**					
	Type I TM	GPI	Secreted	Multi-TM	Total

Worm	3	0	1	0	4
Fly	8	0	0	0	8
Mouse	35	1	1	0	37
Human	35	1	2	0	38
Total	81	2	4	0	87

**LRR_Tollkin**					

Worm	3	1	0	0	4
Fly	12	1	3	0	16
Mouse	17	0	2	0	19
Human	17	0	2	0	19
Total	49	2	7	0	58

**LRR_Other**					

Worm	0	0	3	1	4
Fly	0	0	2	5	7
Mouse	1	0	9	16	26
Human	1	0	9	16	26
Total	2	0	23	38	63

**LRR_Only**					

Worm	11	2	4	0	17
Fly	23	1	10	0	35*
Mouse	28	5	19	0	52
Human	32	6	19	0	57
Total	94	14	52	0	161*

The numbers of eLRR proteins in each of the four major groups is listed for each species, broken down by predicted protein localisation or topology: type I transmembrane, GPI-linked, secreted and multiple-membrane-spanning. *includes CG1504, unclassified localisation.

### Subfamily expansion and diversification

**Figure 5:**
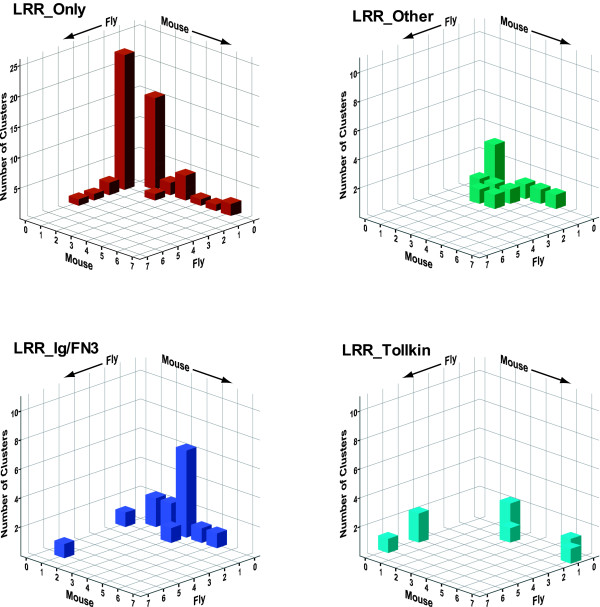
**Group-specific patterns of expansion and diversification**. The graphs depict three-dimensional histograms showing the number of clusters (on the *z *axis) having *x *members in the fly and *y *members in the mouse. The clusters used for this analysis are listed [see Additional File 6]. Different patterns of expansion (new members in one species of a conserved subfamily) and diversification (novel subfamilies in one species) are observed across the four major groups of eLRR proteins. Graphs were generated with the SPSS program.

In order to assess the extent of expansion (new members of existing subfamilies) and diversification (new subfamilies) across different organisms, we analysed the membership of clusters across mouse and fly. For this purpose, we defined clusters in such a way as to distinguish those with species-specific expansion from those with diversification [see Additional File 6]. For each cluster we counted the number of fly and mouse members and then generated histograms of the number of clusters with x fly members and y mouse members (Figure 5). For example, in the LRR_Ig/FN3 group there is one cluster with one fly gene and three mouse genes (Lrigs) and there are six clusters with no fly genes and three mouse genes (Ntrk, Lrrn1–3, Lrrc4, Amigo, FLRT and Lrrc21 groups). These graphs illustrate the different rates of expansion and diversification across these groups.

For the LRR_Ig-FN3 family there is a large number of clusters that have multiple mouse genes and no fly genes. These represent the diversification of new architectures and gene families in the mammalian lineage. There is only one case of expansion in the mouse within a conserved subfamily (the Lrig family which has three members in mouse and one, lambik, in fly (as well as one in worm)). Conversely, the kekkon family shows a specific expansion in flies compared to mammals, where there is only a single apparent closest orthologue, Lrrc24.

In contrast, in the LRR_Tollkin group there has been independent expansion of subfamilies in both flies and mammals (and even comparing mouse and human). Similar expansions are observed in the subfamilies of Toll-like receptor genes themselves and in the subfamilies of gene encoding proteins that do not have TIR domains but that cluster within this group (see below).

In the LRR_Only group there has also been independent expansion, apparently followed by rapid divergence, resulting in a very large number of singletons in each species. These are genes with no recognizable orthologue in the other species (fly or mouse) and no recognizable paralogue in their own species. The encoded proteins do not cluster at high stringency (e-value and inflation parameter) but many cluster into a very large group at lower stringency. This trend may reflect increased divergence rates of this class of proteins. There is only one case in this group of apparent orthology, between CG6959 in fly and Tpbg/5T4 [75] and a novel gene in mouse.

The LRR_Other group shows the opposite pattern with the largest number of clear orthologues between mouse and fly (clusters on the diagonal). This group also contains the most clusters with a clear worm orthologue [see Additional File 3 and Figure 4]. The members of this group include the Slit proteins, peroxidasins, and a number of G-protein coupled hormone receptors, which are all conserved, as well as a number of mammal-specific families including the Lgi proteins.

### Clustering of known proteins

These analyses provide an overview of relationships within the eLRR superfamily and highlight a number of previously unreported associations, allowing us to classify several novel proteins as paralogues of Lrrc21/Pal, Tpbg/5T4, Lrrc3 or Chad, for example. Conversely, it is clear that the recently named NLRR4 is not in fact a paralogue of the other NLRR proteins (-1, 2 and 3; also confusingly known as Lrrn1, 2 and 3). Also, the Lrig proteins in mammals are orthologous not to kekkon proteins in the fly, as has been suggested previously [76], but to the lambik protein in flies (and T21D12.9 in worms). The mammalian protein Lrrc24 appears to be the closest orthologue of the kekkon proteins.

A particularly interesting finding is of a number of LRR proteins which cluster with the Toll-like receptors in both flies and mammals but which do not have a characteristic TIR domain. One of these: CD180, also known as RP105, clusters specifically with Tlr4. This protein lacks a TIR domain and has recently been found to act as a negative regulator of Tlr4 [77]. Also in the LRR_Tollkin group in mammals is a subgroup of more distantly related proteins: carboxypeptidase N subunit 2 (Cpn2), glycoprotein V (Gp5) and leucine-rich repeat-containing protein 15 (Lrrc15, also known as Lib), (which form a sub-cluster), as well as insulin-growth factor acid labile subunit (Igfals) and KIAA0644 (which also has an FN3 domain). Lrrc32 (also known as GARP [78]) and the related protein Lrrc33 also fall into this cluster, along with the novel fly protein CG7509. In the fly there is also another subcluster that clusters with the Tlrs. This subcluster includes chaoptin, which is known to function as an adhesion molecule in neural development [18] and several other novel proteins, including one with an FN3 domain (CG40500-PD). The chaoptin cluster also contains the worm tol-1 protein and the novel worm protein C56E6.6. The large LRR_Tollkin group is one example where the expectation of hierarchical clustering does not hold; in many cases, individual proteins in this broad family cluster into different subfamilies at different e-values and inflation parameters [see Additional File 4 and discussion].

### Novel protein families

**Figure 6:**
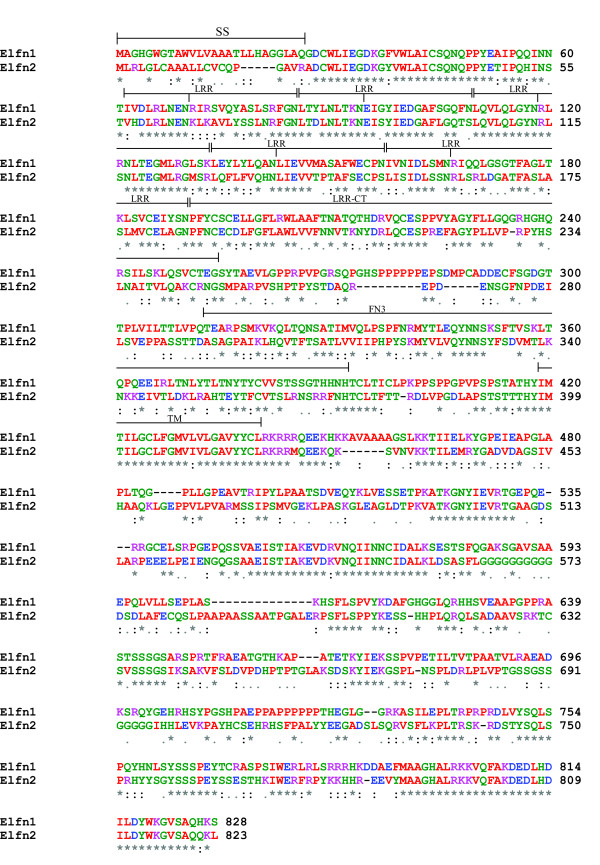
**Alignment of Elfn proteins**. Predicted amino acid sequences from Elfn1 (A930017N06Rik) and Elfn2 (Lrrc62) from the mouse were aligned with CLUSTALW. Amino acids are colour-coded by chemical properties: blue: acidic; green: hydroxyl/amine/basic/Q; magenta: basic; red: small, hydrophobic (including aliphatic Y). Brackets indicate the extent of predicted motifs, including signal sequence (SS), six LRRs (the notch under the bracket indicates the end of the conserved N-terminal portion of each LRR), LRR-CT domain, fibronectin type-3 (FN3) domain and a transmembrane domain (TM). No recognizable LRR-NT domain was predicted. Note that the final LRR comprises the highly conserved N-terminal half-repeat only (consensus: LxxLxxLxLxxN). Identical residues are indicated by an asterisk, highly conservative substitutions by two dots and conservative substitutions by a single dot.

These analyses have also catalogued a large number of novel proteins and subfamilies encoding eLRR proteins in worms, flies and mammals. Two novel clusters in mammals are of special interest due to their expression patterns in the nervous system (see below). One includes two closely related TM proteins currently identified as A930017N06Rik and Lrrc62 in the mouse. These proteins form a distinct subfamily at high stringency and are characterized by a signal peptide, 6 LRR repeats, an LRR-CT and an FN3 domain extracellularly, a TM domain and a long cytoplasmic tail (Figures 3, 6). The cytoplasmic tail contains a large number of tyrosines but no other detectable motifs. Both genes have two exons with the coding sequence entirely in the 3' exon. We propose to name these Elfn proteins, for extracellular-Leucine-rich repeat Fibronectin domain proteins. (A930017N06Rik is Elfn1 and Lrrc62 is Elfn2).

**Figure 7:**
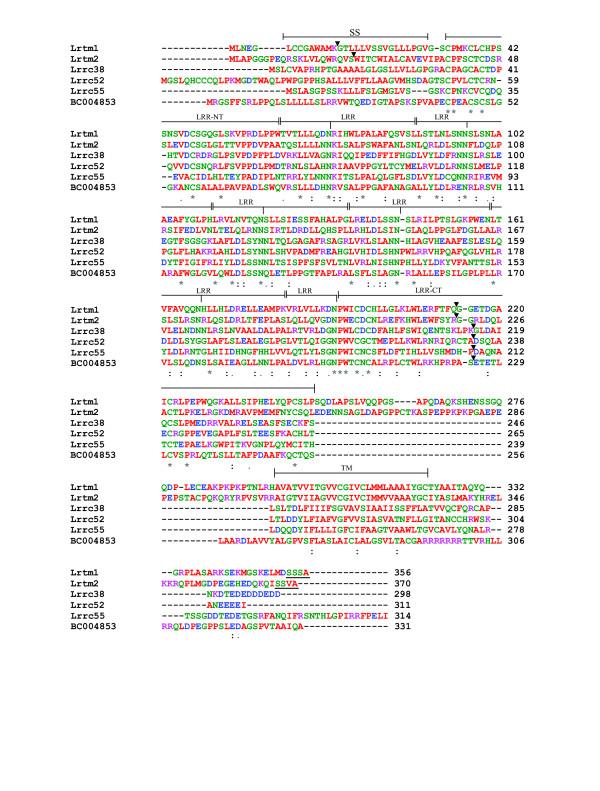
**Alignment of proteins in Elron cluster**. Predicted amino acid sequences from Lrtm1, Lrtm2, Lrrc38, Lrrc55, Lrrc52 and BC004853 from the mouse were aligned with CLUSTALW. Brackets indicate the extent of predicted motifs (consensus limits are shown); the notch under the bracket indicates the end of the conserved N-terminal portion of each LRR. Arrowheads denote exon-intron boundaries. The short cytoplasmic domain is poorly conserved, but does contain similarly positioned acidic residues (E/D) in all members. Lrtm1 and 2 end in consensus PDZ-binding domains (SSSA/SSVA), underlined. Abbreviations, amino acid colour-code and conservation symbols as in Figure 7.

Another cluster of related proteins comprises BC004853 (called LRRC26 in humans), Lrrc38, Lrrc52 and Lrrc55, Lrtm1 and Lrtm2 (names derived from sequencing projects [79]). These are all LRR_Only proteins with a signal peptide, an LRR-NT, 6 LRR repeats, an LRR-CT, a TM domain and a short cytoplasmic tail containing a short stretch of acidic residues (Figures 3, 7). Lrtm1 and 2 also contain conserved predicted PDZ-binding sequences at their C-termini, suggestive of synaptic localisation. These proteins cluster in a group of six at low stringency (e^-25^, level 1), but break into several subclusters at higher stringency (including Lrtm1 and 2 and Lrrc38 and 55). They are defined as paralogues in the Ensembl database but whether they represent a true "subfamily" is unclear. For convenience, we refer to this cluster as the "Elron" cluster, for extracellular-Leucine-Rich repeat-Only proteins, but have not renamed individual members.

In flies there are several subfamilies of novel proteins. These include CG7800 and CG18249, both LRR_Only TM proteins, CG32055 and CG6749, both secreted LRR_Only proteins and CG10824 and CG11910, which cluster as a pair at e^-35^, level 2 and in a group of four with CG4950 and CG5810 at e^-25^, level 1. CG4950 is a predicted TM protein while the others in this cluster are predicted secreted proteins.

In worms, a subfamily emerges comprising sym-1 (C44H4.3) and sym-5 (C44H4.2), both of which interact genetically with mec-8[80], along with C44H4.1 and two other predicted proteins K07A12.2 and ZK682.5. There are also several cases of apparent one-to-one worm-fly orthology of novel proteins, including CG16974 and ZC262.3a, CG7509 and M88.6a, CG15151 and T01G9.3 and CG5819 and K07A12.2.

### Genomic clustering

To assess the possibility that some related genes might occur in clusters in the genome we examined genomic locations for all genes in our eLRR dataset [see Figure 2 and Additional File 3]. Not surprisingly, many closely related genes occur in tandem: five of the six *Slitrk *genes occur in two clusters in the mouse, one on the X chromosome and one on chromosome 14. Other genes occurring in tandem include *Tlr7 *and *Tlr8*, *Islr1 *and *Islr2*, *Lrrc21*/*Pal *and *Lrrc22*, *Lrrc8b*, *c *and *d *and *Fshr *and *Lhcgr*. We also found a number of examples where more distantly related genes occur in tandem in the genome, lending further support to the clustering results presented above, including *Cpn2*, *Gp5 *and *Lrrc15*/*Lib*. In the fly, several Toll-related genes occur in adjacent pairs (*Tollo *with *Toll-6*, *Toll-7 *with *18w *and *Toll-9 *with *CG5195*, a novel non-TIR-containing member of the LRR_Tollkin group), as do *tartan *and *capricious*. Similarly, a number of the novel subfamilies identified above occur in tandem including *CG7800 *with *CG18249*, *CG32055 *with *CG6749 *and *CG10824 *with *CG5810*. In the worm genome, the C44H4 genes (.1, .2 (*sym-5*) and .3 (*sym-1*)) also occur in tandem.

We observed an interesting situation in the family of small secreted proteoglycans that includes decorin, biglycan, and related genes. Proteins in this family fall into several subclusters using TribeMCL, in agreement with previous analyses [81]. Interestingly, many of them are also grouped in tandem in the genome in several different loci but each locus contains a representative of two or three subclusters. This suggests two early duplications in tandem and a subsequent triplication of the entire locus, with some additional gene losses and duplications [82]. The *Ecm2 *gene is also located in tandem in one of these loci (with *Aspn*, *Omd *and *Ogn*) but it is highly divergent from the other proteins and whether it should be considered a member of this family is debatable [74].

### Expression analyses

**Figure 8:**
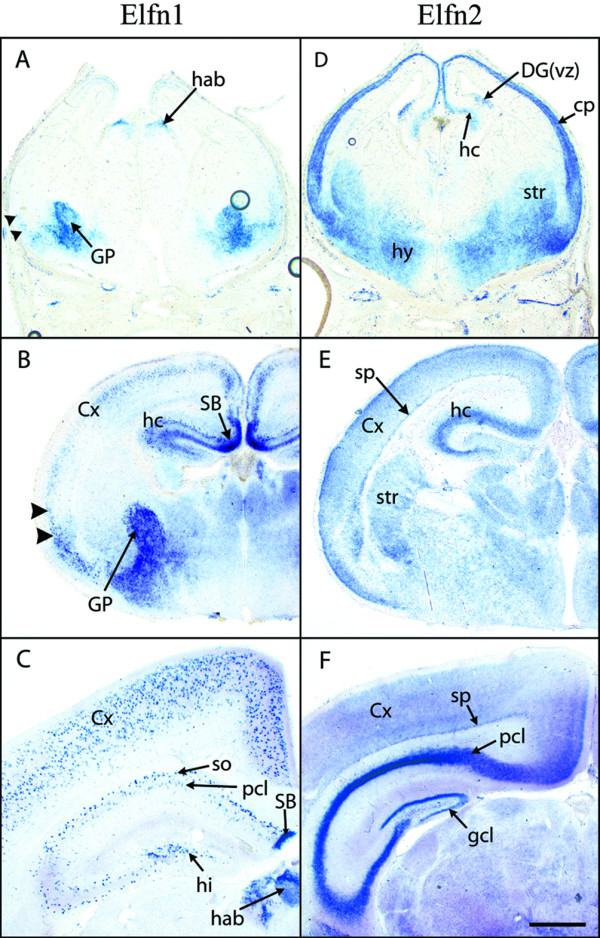
**Expression of *Elfn *genes in developing mouse brain**. Expression as defined by RNA *in situ *hybridisation is shown for *Elfn1 *(A-C) and *Elfn2 *(D-F) in coronal sections of mouse brain at three ages (embryonic day 15 (E15), A, D; postnatal day zero (P0), B, E; and postnatal day 9 (P9), C, F). *Elfn1 *is strongly expressed in globus pallidus and interneurons in cortex and hippocampus, while *Elfn2 *is expressed in striatum and in projection neurons in cortex and hippocampus. Arrowheads in A and B indicate presumed interneurons migrating towards cortex. Abbreviations: cp, cortical plate; Cx, cortex; DG(vz), ventricular zone of dentate gyrus; gcl, granule cell layer (of dentate gyrus); GP, globus pallidus; hab, habenula; hc, hippocampus; hi, hilus (of dentate gyrus); hy, hypothalamus; pcl; pyramidal cell layer (of hippocampus); SB, subiculum; sp, subplate; so, stratum oriens (of hippocampus), str, striatum. Scale bar: E15, 200 microns; P0 and P9, 500 microns.

In order to begin to assess the possible involvement of these novel genes and families in neural development we analysed the expression of a subset of them by *in situ *hybridisation in the mouse or fly developing nervous system. *Elfn1 *and *Elfn2 *show rather complementary expression patterns in the embryonic and postnatal mouse brain (Figure 8). *Elfn1 *is strongly expressed in interneurons in the hippocampus and cortex while *Elfn2 *is expressed more broadly in the cortex in presumed glutamatergic neurons and in the hippocampus in pyramidal and granule cells. In the basal ganglia, *Elfn1 *is expressed in the globus pallidus, while *Elfn2 *is expressed more strongly in the other major division, the striatum. These patterns are maintained in adults, according to the Allen Brain Atlas [83]. Based on abundance of cDNAs in the Unigene database, it appears that expression of *Elfn2 *is quite restricted to the nervous system (Unigene reference: Mm.323188), while *Elfn1 *is also expressed in endocrine and reproductive tissues (Unigene reference: Mm.237102).

**Figure 9:**
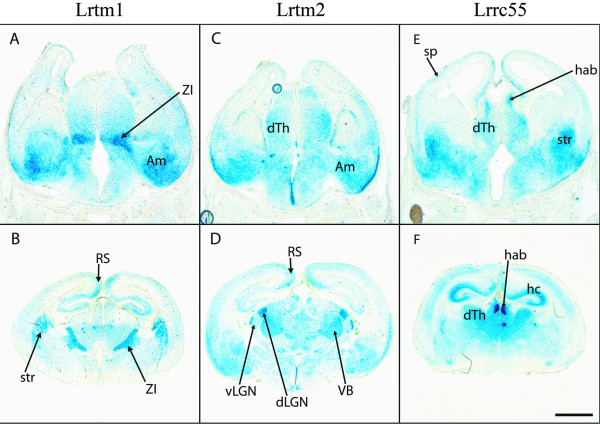
**Expression of Elron cluster genes in developing mouse brain**. Expression as defined by RNA *in situ *hybridisation is shown for *Lrtm1 *(A, B), *Lrtm2 *(C, D) and *Lrrc55 *(E, F) in coronal sections of mouse brain at two ages (E15, A, C, E and P0, B, D, F). Differential staining in subsets of thalamic nuclei and across cortex is observed. Abbreviations: Am, amygdala; dLGN, dorsal lateral geniculate nucleus; dTh, dorsal thalamus; hab, habenula; hc, hippocampus; RS, retrosplenial cortex; sp, subplate; str, striatum; vLGN, ventral lateral geniculate nucleus; ZI, zona incerta. Scale bar: E15, 200 microns; P0, 500 microns.

Among the genes in the Elron cluster, three (*Lrtm1*, *Lrtm2 *and *Lrrc55*) are expressed in discrete regions of the developing mouse brain, in particular marking different nuclei in the developing thalamus as well as a number of other areas (Figure 9). According to the Allen Brain Atlas, the expression of *Lrtm1 *declines after development and is practically undetectable in adults. In contrast, *Lrtm2 *is maintained at high levels in adults in a number of discrete regions including the granule cell layer in the olfactory bulb, the basal ganglia, dorsal thalamus, dentate gyrus, layers 2/3 and 5 in the cortex and Purkinje cells in the cerebellum. *Lrrc55 *is also maintained at high levels in mitral cells in the olfactory bulb, in the habenula and in layers 4 and 6a in the cortex. *Lrrc38 *is expressed at lower levels during development (data not shown) but is expressed in a specific pattern in the adult brain, including the CA3 region of the hippocampus and the zona incerta [83]. *BC004853 *and *Lrrc52 *do not appear to be expressed in the embryonic or postnatal brain. This result is confirmed by the absence of expression in the Allen Brain Atlas and by analysis of cDNA abundance in the Unigene database, which show that *Lrrc52 *(Mm.159799) is specific to muscle and testis and that *BC004853 *(Mm.275228) is almost exclusively expressed by the vesicular organ in the male reproductive system. Similar cDNA abundance data for the other four genes show that *LRTM2 *(in this case human, Hs.585579) is almost brain-specific, while *Lrtm1*(Mm.95780), *Lrrc38 *(Mm.94020) and *Lrrc55 *(Mm.291095) are also expressed in a small number of other tissues.

The expression of the *Elfn *genes and of several genes in the Elron cluster is thus consistent with a possible role in specifying neuronal connectivity, especially thalamic and cortical connectivity.

**Figure 10:**
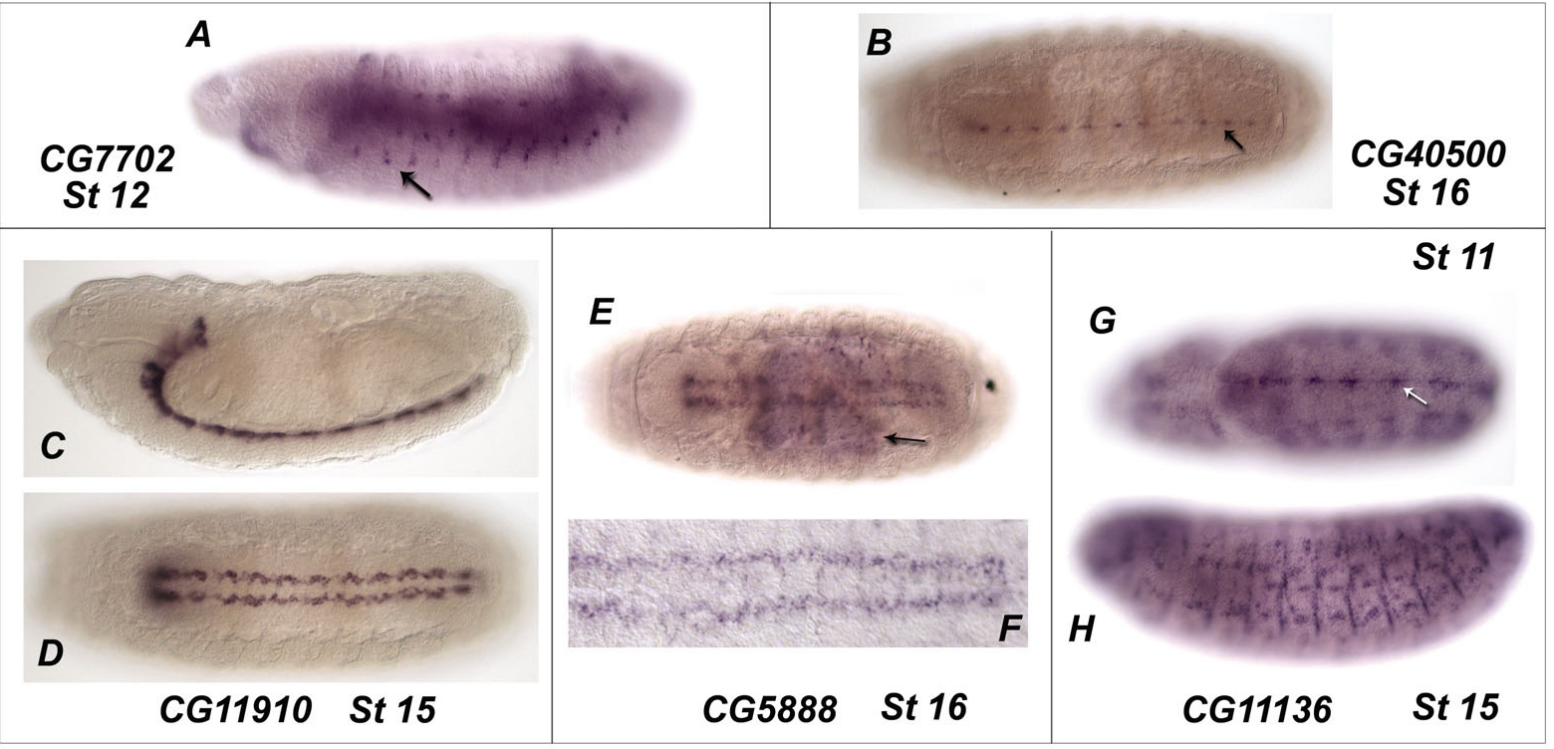
**Expression of novel eLRR genes in the *Drosophila *embryo**. (A) A lateral view of a stage 12 embryo showing expression of *CG7702 *in the midgut and the peripheral nervous system, PNS expression is indicated by a black arrow. (B) *CG40500 *expression in a stage 16 embryo, expression can be seen at the midline (indicated by a black arrow). (C and D) Lateral and ventral views, respectively, of a stage 15 embryo showing *CG11910 *expression in the central nervous system. (E) A stage 16 embryo with *CG5888 *expression in the CNS and midgut chamber, midgut chamber is indicated by a black arrow. (F) A dissected ventral nerve cord fillet with *CG5888 *expression (shown at 400× magnification). (G) A stage 11 embryo showing *CG11136 *expression at the midline, indicated by a white arrow and (H) a stage 15 embryo showing expression of *CG11136 *in the somatic musculature. All whole embryos are shown at 200× magnification. In all views anterior is to the left, in all lateral views dorsal is at the top, B, D and E show ventral views and G shows a dorsal view.

The expression patterns of many of the *Drosophila *eLRR genes identified in the bioinformatic screen were also examined in the embryo by *in situ *hybridisation. A summary of the expression patterns we identified and those previously described is presented [see Additional File 7]. We describe here the expression patterns of those novel eLRR genes identified in our survey that include expression in the nervous system (Figure 10). *CG7702 *is expressed dynamically in the peripheral nervous system (PNS), appearing at stage 11 and disappearing during stage 15. *CG40500 *is exclusively expressed in the CNS and is restricted to a subset of cells at the ventral midline, beginning during stage 14 and remaining into stage 17. *CG11910 *expression is restricted to the most dorsal layer of the CNS in a position consistent with the longitudinal glia. This expression begins at stage 12 and continues throughout embryonic development. *CG5888 *is expressed from stage 5 throughout the embryo with exception of the anterior tip (data not shown). At stage 15 expression of *CG5888 *is initiated in a subset of cells in the CNS. *CG11136 *is expressed in an anteroposterior stripe within the neurogenic region and in the prospective brain lobes during stages 8–10 (data not shown) and in discrete cells at the midline of the CNS during stages 11 and 12. From stage 11 onwards *CG11136 *expression is seen predominantly in the somatic musculature.

## Discussion

This study aimed to catalogue the full repertoire of eLRR proteins in the proteomes of worms, flies, mice and humans, to examine their evolutionary relationships and to identify novel proteins and subfamilies that may have important roles in nervous system development.

### Methodological issues

Generating this dataset required identifying all LRR proteins, distinguishing eLRR proteins among this set (i.e., correctly predicting cellular localisation) and analyzing evolutionary relationships across a large set of highly divergent, multi-domain, repetitive proteins in four distantly related species. For all of these tasks we found the use of single programs only partly reliable. It was especially difficult to derive a single set of parameters for any program that would reliably predict the presence of a particular motif or correctly identify orthologues and paralogues for all proteins in the dataset. To overcome this problem we developed an approach of parallel annotation with many different programs, followed by manual curation to arrive at a consensus architecture for each gene, along with a hierarchical clustering method designed to reveal relationships at multiple levels. This is in contrast to the automated one-size-fits-all approaches currently used by some of the large genome databases.

### A comprehensive, curated dataset

Our bioinformatics searches and exhaustive manual curation have yielded what we are confident should be an extremely comprehensive set of eLRR proteins across the four species examined. Rather than employing a series of strict filters we used the combined evidence from a variety of prediction programs and from clustering to distinguish eLRR proteins from intracellular LRR proteins. We think it is therefore unlikely that we have missed many true eLRR genes in any of the organisms. This obviously depends however on the quality and comprehensiveness of the gene predictions in our starting datasets. There may in the first instance be cases of genes that have simply not been predicted at all yet. We also came across numerous cases of mispredicted genes where only a fragment was predicted or where a single ORF was split into two predicted genes, for example. In most of these cases the fragments still clustered with other eLRR genes and a full-length sequence was often identifiable from one of the starting datasets. It is difficult to estimate how common such annotation errors are but it is reasonable to expect that they may have caused us to miss a small number of additional eLRR genes or to misclassify some as cytoplasmic.

The manual curation of these sequences has added substantially to the value of this dataset. An appreciable percentage of predicted protein sequences had to be amended in some way to yield what we consider to be the "correct" predicted full-length protein. These corrections were based on various factors including comparison of architectures across orthologues or paralogues, the absence of an expected signal peptide or the location of the predicted start codon with respect to the signal peptide.

Because degenerate or atypical LRRs have been described [4,49,69] that do not match the consensus motifs defined by SMART and Pfam we designed a customised program, LRRscan, to search for a minimal consensus that defines animal extracellular LRRs. We also searched for minimal consensus motifs that define LRR-NT and several varieties of LRR-CT domains found in different types of proteins, including small proteoglycans and G-protein-coupled receptors [4]. These predictions were compared with the results of SMART and Pfam [see Additional File 2] and a consensus architecture was predicted by manual inspection, based on converging evidence. The results match those of proteins with known structures [5-7,70-73,84], significantly better than a combination of SMART and Pfam alone (allowing for semantic differences in whether the final half repeat is counted as one and whether putative LRRs overlapping with NT or CT domains are counted). The predicted transmembrane topologies are also based on converging evidence from multiple programs and have also been subject to expert evaluation. Nevertheless, the architectures presented should be viewed as predictions that will require experimental verification. In particular, the absence of a predicted LRR-NT or LRR-CT domain does not mean there is no domain present that is performing a capping function; there may be additional varieties of such domains that have not yet been defined. In addition, we have chosen a representative isoform for each gene; the database thus contains no information on alternative splice forms or other isoforms that may have differing architectures.

### Hierarchical clustering

The hierarchical clustering method we used gets around the problem of defining a unique set of parameters that is suitable to all proteins and levels of inter-relationship. In most cases, it generates a tree-like structure that reveals relationships across many different levels at once. This is a difficult problem for multiple alignment programs such as CLUSTALW or T-COFFEE, which work well for closely related proteins but which are not designed to compare highly divergent proteins with differing architectures. Previous attempts using multiple alignment programs to derive a phylogenetic tree across many eLRR subfamilies at once contain numerous differences from our results and from known relationships [3,85].

In some cases, the results of TribeMCL depart from the expected hierarchical relationship. This is the case for the extended LRR_Tollkin group of proteins, including many proteins characterized by an eLRR domain but lacking an obvious TIR domain. While the clustering of these proteins with the TLR group is quite convincing, based on direct inspection of the BLAST results, it is extremely difficult, indeed impossible with these data, to discern more discrete relationships within this large family. The reasons for the anomalous hierarchical clustering results with these genes may relate to the large number of LRRs present in these proteins and the very slight differences in pairwise similarities across the group. At different levels of stringency small differences in BLAST scores may be amplified by the TribeMCL algorithm to result in membership of different clusters that do not share the expected hierarchical relationship. Attempts to resolve the phylogeny of all the genes in this group using the T-COFFEE multiple alignment program were no more enlightening, resulting in a starburst pattern where the roots of each subfamily are too close to each other to resolve (data not shown). Despite these limitations, the TribeMCL analysis has revealed a group of eLRR proteins that are clearly more related to the TLR proteins than to other LRR_Only proteins.

### Nomenclature

The current nomenclature of eLRR proteins is very confusing, with multiple synonyms for many genes [see Additional File 3], many of which do not give accurate information on relationships. For example, there is a large number of proteins designated LrrcX, where X is a number. These names were apparently derived from large-scale genome projects and do not represent a specific subfamily of related proteins. For that reason we have proposed the names Elfn1 and 2 for one novel subfamily. We also identify another discrete cluster of six "novel" proteins (which we refer to as the Elron cluster), although whether they represent a true subfamily is open to debate. In addition, some novel proteins that group into small subfamilies with Lrrc21/Pal, Chad, Lrrc3 and Tpbg/5T4 [see Additional File 3, Figures 3 and 4] could be given names to reflect that fact. Finally, while Lrrn1, 2 and 3 (also known as NLRR1, 5 and 3) form a subfamily, the recently named NLRR4 [46] is not in fact a member of this subfamily. It does not have the Ig domain present in these genes and does not cluster with those genes at any parameters.

### Comparative analyses of major groups

For the purposes of some of the analyses we split the eLRR proteins into four groups, based on architecture and clustering results. The LRR_Ig/FN3 group includes the largest percentage of mammal-specific subfamilies, many with multiple members. Almost all of the proteins in this group are associated with the membrane, either type I TM or GPI-linked. The majority of these subfamilies (including Ntrks, Lrfns/Salms, Flrts, Lrigs, Netrin-G ligands, and Lingo proteins) show discrete expression in the nervous system and many of them have been shown to have functions in neural development [3] and/or have been implicated in neurological or psychiatric disease [49]. Expansion of this class of proteins is thus correlated with the evolution of the complex mammalian brain and plausibly contributed to it by providing the requisite specificity of cellular interactions to mediate a large number of selective connectivity decisions. We have identified a novel mammal-specific LRR_FN3 subfamily, the Elfn proteins, with discrete nervous system expression.

The LRR_Only group shows independent diversification in flies and mammals, with a large number of singletons (unclustered proteins), suggesting rapid sequence divergence. This group also contains a number of proteins implicated in nervous system development or function including the Nogo-receptor, Lrrtm and Slitrk families, and connectin, Gp150, Tpbg/5T4 and Nyx for example. We have identified six proteins in another novel mammal-specific cluster, the Elron cluster, several of which show highly suggestive expression patterns in the developing nervous system. We have also discovered a number of novel fly proteins in this class that are similarly discretely expressed in the embryonic nervous system.

The LRR_Tollkin group shows a different pattern of evolution, with parallel expansions in flies and mammals, of both the Toll-like receptor genes and of the genes that cluster with them. In mammals, the latter include Cpn2, Gp5 and Lrrc15 (Lib), which form a subcluster and which are also arrayed in tandem on chromosome 3 in humans (16 in mouse). These proteins have diverse binding partners and biochemical functions but are all involved in inflammation in some way: as a regulatory subunit of carboxypeptidase [86], as a component of the platelet glycoprotein complex (which also contains the eLRR proteins GP1bα and GP1bβ[87]), and as a mediator of the glial response to β-amyloid [88], respectively. They form a slightly larger cluster with Igfals, the acid-labile subunit of insulin growth factor, which regulates IGF signaling [89] and with the novel gene KIAA0644, which has an FN3 domain in addition to the LRR domain. Lrrc32/GARP [90] and its paralogue Lrrc33 also cluster with this group at e^-40^, level 1, but not at some lower levels. The novel fly gene CG7509 also clusters with this group at e^-40^, level 1 and with other fly genes including chaoptin at some other levels. Whether it can be said to be directly orthologous to any (or all) of these mammalian proteins is hard to determine. The other fly genes in this group are mostly novel and include CG40500-PD, which has an FN3 domain and which shows very discrete expression in the midline of the embryonic nervous system.

The LRR_Other group is an arbitrary default group as it contains many unrelated genes or subfamilies. Nevertheless, it is interesting to note that this group contains the highest percentage of genes with orthologues across all species, including worms (e.g., the slits and peroxidasins and some of the seven-transmembrane hormone receptors). This group also includes the mammalian Lgi subfamily, recently implicated in epilepsy and myelination.

### Human-mouse differences

Only a small number of proteins are specific to either human or mouse. There are two cases where there are genes in humans that are not represented in mouse that both seem to be caused by specific loss in mice, rather than representing human-specific genes. The human gene synleurin appears to have been pseudogenised in rodents, although it is present in many other species besides humans (including dog, cow and chick, for example). Also, MXRA5 (or adlican), a paralogue of the large secreted protein Igsf10, is not detectable in the mouse genome (but is present in cow, dog and opossum, for example).

There is also a small number of examples where there has been independent expansion of subfamilies in either humans or mice. These include the Toll-like receptors TLR10 in humans and TLR11, 12 and 13 in the mouse. They also include the unusual subfamily of LRRC37 genes, which is represented by a single gene in the mouse (called Lrrc37a) but multiple, highly related genes in humans (LRRC37A, A2 and A3 and LRRC37B, as well as a number of other partial duplicates lacking LRRs). These are located in tandem on chromosome 17 and have arisen from multiple duplications of the BRCA1 region in primates [91]. The extracellular domains of these TM proteins are characterised by six predicted LRRs but these make up only a small fraction of the overall protein, which is highly variable in length. The functions and expression patterns of these unusual proteins are unknown.

## Conclusion

This survey presents a comprehensive overview of the repertoire of eLRR proteins in various species and their inter-relationships. As such, it provides the necessary foundation for a systematic analysis of the functions of this class of genes, which are likely to include prominently neural development, innate immunity and inflammation. In particular, expansion of the eLRR proteome is correlated with increasing complexity of the nervous system. Given the functions and discrete expression patterns of many known members, it seems likely that this superfamily, including the novel proteins identified here, could provide the requisite specificity of cellular interactions to mediate a large number of selective connectivity decisions.

## Methods

### Database pipeline

Protein sequences for all four species were retrieved from the Ensembl FTP site: Mouse release 36 NCBI m34 assembly (36471 sequences); Human release 36, NCBI 35 assembly (33869 sequences); Worm release 37, Wormbase 150 dataset (26032 sequences); Fly release 37, BDGP assembly release 4 (19369 sequences). In addition, 68627 mouse and 57366 human protein sequences were downloaded from the International Protein Index, version 3.14. A further 24273 human and 19258 mouse protein sequences were retrieved through the web interface [92] from the August 2006 version of the Mammalian Gene Collection. We also included a further 879 sequences comprising many from an older version of the Mammalian Gene Collection (February 2006) that were absent from the August 2006 release as well as several more added manually. All sequences were stored for easy access in a MySQL database.

The data set was reduced through use of a small Perl script that filters out duplicate copies of sequences for each species and keeps either the Ensembl version or an entry with a flag indicating its preference after manual curation. The non-redundant data sets for mouse, human, worm, and fly contained 85991, 74866, 22698, and 16857 sequences, respectively.

These sequences were subjected to an all-against-all Blast search (NCBI BlastP, version 2.2.12) carried out on a high-performance Linux cluster. An expectation cut-off of 0.1 was specified, and the top 200 hits for each search in tab-delimited format (-m8) were reported. The Blast results were parsed with the mcxdeblast tool using expectation cut-offs from e^-10 ^to e^-40 ^and formatted for clustering with the mcxassemble tool (options -q -r max -map -b), both part of the MCL package (version 1.005, 05–272). Each output was then subjected to Markov clustering with the MCL program using inflation parameters ranging from 1.2 to 5. The program Tribe-families was then run to produce the final clusters.

For proteins from the IPI and MGC set that did not have gene IDs assigned, we produced alignments using T-Coffee (version 3.93) with their best Blast hits. If sequences with matching protein names were found that are fully contained in another one or showed identity over at least 95% and sequence difference of maximal 15% we transferred Ensembl gene ID annotation where available. Through this, 2490 sequences from mouse and 1458 sequences from human were assigned Ensembl gene IDs. The gene information was used to remove isoforms from the clusters: only the protein with the longest sequence was kept for each gene. In some cases, where dubious excessive amino acids seem to have been added to a sequence, manual curation was necessary to overwrite this behavior and select proteins that seemed biologically more plausible.

For prediction of architecture we used HMMpfam of the HMMER package (version 2.3.2) [93], together with the SMART (release 25 Nov. 2004) and Pfam (version 19.0) HMM libraries. Transmembrane predictions were produced by the programs TMHMM (version 2.0 [58]), HMMTOP (version 2.1 [63]), and TMPred [64]. Signal Sequence analysis was carried out using SignalP (version 3.0 [59]) and GPI-link results calculated by the BIG-PI program [65] were obtained for human [94] and fly [95]. In addition, information about the genomic location and synonyms for a gene were retrieved from Ensembl, MGI, Wormbase and Flybase. Clustering and annotation information were combined into a large spreadsheet for the final output.

### LRRscan

Based on a number of published studies [1,4,49,96] and our own inspection of the sequences in our dataset we defined the minimal N-terminal part of a single extracellular-type LRR as: LxxLxLxxN. This is followed by a C-terminal part of each LRR of typically 10–21 amino acids that are quite variable. Consensus sequences for the LRR capping domains (LRR-NT at the N-terminus and LRR-CT at the C-terminus) have been defined by [4], including three different consensus sequences for the LRR-CT domain, derived from different classes of proteins. LRR-CT1 is the most common type, LRR-CT2 is found in small proteoglycans and LRR-CT3 in G-protein-coupled receptors:

LRR-CT1 domain: P(w/f)xCxCxoxWLxxw(9–24)oxC(9–18)CxxP

LRR-CT2 domain: nI(s/t)xogxxdFCxoxxxxo(4–5)y(4)LxxNpo(6)PxxfxCo

LRR-CT3 domain: LxxAxL(s/t)YPSHCCAFxN(6–19)nosxCnxsxxR...

LRR-NT domain: (7–10)CP(2–5)CxC(4–17)oxC(2–4)oxxoPxxoP

"x" represents any residue and "o" a non-polar residue [4].

We derived a minimal consensus sequence from each of the above and designed a new program, LRRscan, to search for these sequences as well as the minimal LRR defined above. The search for LRR-NTs and LRR-CTs focuses exclusively on the cysteines, which are the most conserved amino acids in these motifs. The regular expressions applied are as follows:

LRR: L..L.L..N.{10,21}

LRR-NT: C.{2,8}C.C.{6,19}C.{11,15}

LRR-CT1: ...C.C.{19,34}C.{9,18}C.{3}

LRR-CT1_short: ...C.C.{19,34}C.{22}

LRR-CT2: .{10}C.{30,31}C.

LRR-CT3: ..CC.{14,27}C.{6}

(Each dot represents any single letter, numbers in curly brackets indicate a repeat frequency, either exact or as a range where two numbers are given. Dots at the beginning or end of a domain denote spacing from the start or end of other motifs, including LRRs).

LRRscan was written in Perl and has been especially designed for the detection of LRR motifs. Input consists of a sequence file in FASTA format as well as search parameters. Each sequence is scanned for patterns, specified as strings, and alternative amino acids for certain positions, specified as triplets comprising position, alternative amino acid and score. The LRR pattern used in our search was 'LxxLxLxxN', where a small 'x' acts as a placeholder for any amino acid. The alternative options were amino acids A, I, V, F, G, M, or W for any of the leucines and C, S, or T instead of the asparagine. Each exact match between an amino acid and the search pattern produces a score of 1, whereas a match to an alternative letter only scores 0.4. The scores are summed up over the length of the pattern and a minimum score of 2 would lead to further consideration of the sequence region. To allow for maximum sensitivity an exhaustive search is carried out, i.e., all patterns that match the search criteria are initially captured even if they overlap.

In the next step the regions are grouped into stretches of LRRs located within a specific distance from each other, in our case allowing for a gap of 20 to 30 amino acids between starts of pattern. Within a sequence of LRRs the overlaps are removed by only keeping the highest scoring regions. However, overlaps between sets of LRRs are allowed in the LRRscan output. Such occurrences are indicated in the output by a backward shift in the sequence location, i.e. the end of one stretch of LRRs might be printed again at the beginning of the next one. This is usually interpreted in the manual curation process as an insertion in the LRR domain. Each sequence is also scanned for a minimal LRR-NT pattern in the upstream sequence and for a minimal LRR-CT pattern (one of three possible types) in the downstream sequence. These are allowed to overlap with predicted LRRs to maximise the detection rate. The presence of additional elements from the more complete consensus sequences defined by Kajava was considered as supportive evidence in the manual curation process. Two alternative types of LRR-NT have been proposed, with different numbers of cysteines [97]. We found it difficult to ascertain whether these were really evolutionarily distinct or whether some cysteines were simply not well conserved and SMART and Pfam can detect both types. For these reasons we have not attempted to distinguish between these putative types of LRR-NT. Similarly, some LRR-CT domains could not be categorized definitively as CT1 or CT2 subtypes; these are denoted as LRR-CT in Table 1 and [see Additional File 3].

The output from LRRscan reports the sequence that was searched and the positions of the motifs found followed by the sequence of the motifs themselves. The LRRs are numbered sequentially (within a set of grouped LRRs) and spaced to easily distinguish the well-conserved N-terminal from the more variable C-terminal part [see Additional File 2]. We ran LRRscan on a set of protein sequences for which HMMpfam had predicted LRR motifs already (using the PFAM and SMART databases). The high sensitivity might result in an excessive number of false positives if applied to other sequences, but our goal was to further increase the detection rate of LRRs in sequences that showed an initial sign of LRR occurrence. A summary graphical output was generated for the output of each program for each sequence and aligned for easy comparison [see Additional File 2]. Through extensive manual curation a consensus predicted architecture was produced. It was found during manual curation that the cysteine residues in the NT and CT domains were not always positioned strictly according to the above consensuses and some flexibility was allowed for in these cases. We counted the final half repeat before the CT domain as one and did not include putative LRRs that overlapped with well-defined LRR-NT or LRR-CT domains in the total number of repeats.

### RNA *in situ *hybridization

Please [see Additional File 8] for details.

## Abbreviations

ELRR, extracellular leucine-rich repeat; FN3, fibronectin-type 3; GPI, glycosyl phosphatidyl inositol; Ig, immunoglobulin; LRR, leucine-rich repeat; LRR-CT, leucine-rich repeat C-terminal domain; LRR-NT, leucine-rich repeat N-terminal domain; TIR, Toll/IL-1 receptor; Tlr, Toll-like receptor; TM, transmembrane.

## Authors' contributions

The project was designed by KM and GT. SO'K and KH carried out bioinformatics analyses. JD, KW, TO and SM performed analyses on mouse genes. SA performed analyses on fly genes. The manuscript was prepared by KM, JD, KW, SA, KH and GT. All authors read and approved the final manuscript.

## Supplementary Material

Additional file 1 Curated sequences of eLRR proteins. List of curated sequences of eLRR proteins in FASTA format. Click here for file

Additional file 2 LRRscan_out.html. Graphical comparison of HMMpfam and LRRscan results. A compressed archive (lrr_plots.tar.gz) containing 372 images in Portable Network Graphics (PNG) format, an information file (00README.txt) and two HTML-formatted pages, one with output from LRRscan (LRRscan_out.html) and one that links all the images together (00plots.html). After downloading, the archive must be to uncompressed and unpacked. Most modern operating systems (e.g. Windows XP, Mac OS X) will do this automatically when double-clicking on the file. Alternatively, you can use the free tool 'Stuffit Expander' () or your favourite unpacker. On Linux or Unix systems apply the following command: tar zxf lrr_plots.tar.gz. Please note that some browsers might uncompress the file during download without changing the file ending. If you have trouble unpacking the file try renaming it to lrr_plots.tar and double-click on it again. Unpacking the archive creates a new folder (lrr_plots) in which you can find a file called '00plots.html'. Open this file in a web-browser, either by double-clicking onto it or by using the 'File->Open File' menu (or equivalent) of your browser. This will bring up a web-page with plots of LRR motifs for 372 proteins. If you click on an image you can see the text output from LRRscan in a new window. Click here for file

Additional file 3 Table S1. Complete list of genes, clustered at e^-40^. Click here for file

Additional file 4 Table S2. Complete list of genes, clustered at e^-25^. Click here for file

Additional file 5 Table S3. Complete list of genes, clustered at e^-10^. Click here for file

Additional file 6 Table S4. List of clusters used in Figure 5. Click here for file

Additional file 7 Table S5. Summary of fly gene expression Click here for file

Additional file 8 Additional methods. *In situ *hybridisation protocols Click here for file
