# The extracellular Leucine-Rich Repeat superfamily; a comparative survey and analysis of evolutionary relationships and expression patterns

**DOI:** 10.1186/1471-2164-10-230

**Published:** 2009-05-18

**Authors:** Jackie Dolan, Karen Walshe, Samantha Alsbury, Karsten Hokamp, Sean O'Keeffe, Tatsuya Okafuji, Suzanne FC Miller, Guy Tear, Kevin J Mitchell

**Affiliations:** 1 Smurfit Institute of Genetics, Trinity College Dublin, Dublin 2, Ireland; 2 MRC Centre for Developmental Neurobiology, New Hunts House, Guys Campus, King's College London, London, SE1 1UL, UK

## Abstract

Correction to Dolan J, Walshe K, Alsbury S, Hokamp K, O'Keeffe S, Okafuji T, Miller SF, Tear G, Mitchell KJ: The extracellular leucine-rich repeat superfamily; a comparative survey and analysis of evolutionary relationships and expression patterns. BMC Genomics 2007, 8:320.

## Correction

In our original article [1] a mistake was made in Figure Four in the depiction of the structure of the Drosophila gene CG8561, now called Als or dAls (Drosophila acid-labile subunit). The correct structure is shown here in Figure 1, based on a newly-available and slightly extended sequence of CG8561 from the Ensembl database (FBpp0086669). This gene should have been shown to encode a secreted protein with 29 LRR domains, an N-terminal LRR-NT and a C-terminal LRR-CT domain, based on the LRRscan programme output. Use of the new sequence does not alter the clustering relationships of the CG8561 protein, shown in Figure Two and in Additional Files Three-Five of the original paper. We note that these data, derived from TribeMCL, which simultaneously takes all pairwise relationships across all members of the proteomes of the four species used into account, do not provide strong support for direct one-to-one orthology between CG8561 and mammalian Igfals.

**Figure 1:**
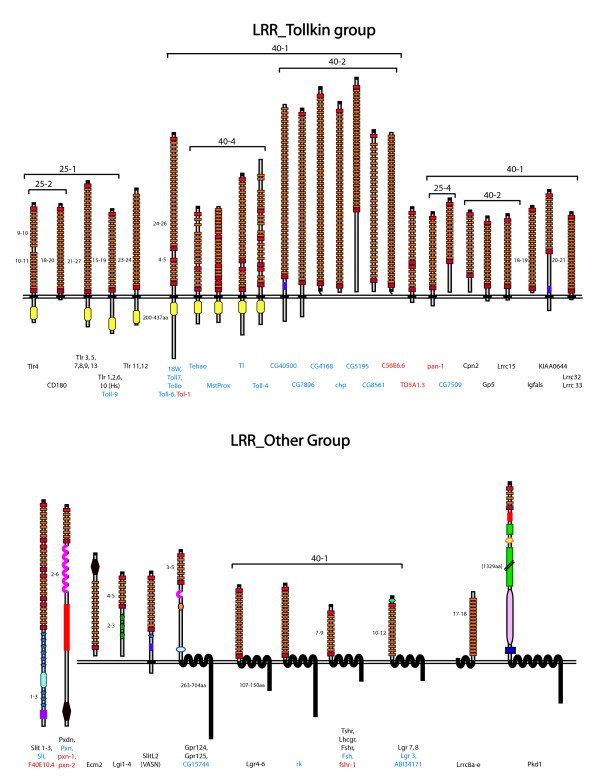
**eLRR protein predicted architectures (part 2)**. Consensus architectures are shown for all proteins in the LRR_Tollkin and LRR_Other groups. An additional set of LRR_Only singletons is listed separately in Table 1. Protein names are shown below the corresponding structures (black, mammalian; blue, fly; red, worm). All figures are drawn to scale (see Key). Consensus architectures were derived for single proteins and across subfamilies from convergent evidence from motif and topology prediction programmes. Where there is a range in number of predicted LRRs or other domains across members of a subfamily, this is indicated next to the domain. A range in length of the cytoplasmic domain is similarly indicated, where it exceeds 20 amino acids. Tightly clustered subfamilies (e.g., Slits, Amigos) are listed under a single consensus architecture. Clusters with more structurally diverse proteins are indicated by the brackets; the numbers refer to e-value and inflation parameter at which the proteins cluster in the MCL programme. See Key for more information.

The reassignment of structure also changes the figures in Table Two (shown here as Table 1). There should be 11 type I transmembrane and 4 secreted proteins in flies in the LRR-Tollkin subgroup.

**Table 1:** Complement of eLRR proteins by group, localisation and species

**LRR_Ig/FN3**					
	Type I TM	GPI	Secreted	Multi-TM	Total

Worm	3	0	1	0	4

Fly	8	0	0	0	8

Mouse	35	1	1	0	37

Human	35	1	2	0	38

Total	81	2	4	0	87

**LRR_Tollkin**					

Worm	3	1	0	0	4

Fly	11	1	4	0	16

Mouse	17	0	2	0	19

Human	17	0	2	0	19

Total	49	2	7	0	58

**LRR_Other**					

Worm	0	0	3	1	4

Fly	0	0	2	5	7

Mouse	1	0	9	16	26

Human	1	0	9	16	26

Total	2	0	23	38	63

**LRR_Only**					

Worm	11	2	4	0	17

Fly	23	1	10	0	35*'

Mouse	28	5	19	0	52

Human	32	6	19	0	57

Total	94	14	52	0	161*'

The numbers of eLRR proteins in each of the four major groups is listed for each species, broken down by predicted protein localisation or topology: type I transmembrane, GPI-linked, secreted and multiple-membrane-spanning. *'includes CG1504, unclassified localisation.
