# Supplementary material for: The extracellular Leucine-Rich Repeat superfamily; a comparative survey and analysis of evolutionary relationships and expression patterns
Source: BMC Genomics. 2007 Sep 14;8:320. doi: 10.1186/1471-2164-8-320 (PMC2235866; doi:10.1186/1471-2164-8-320)
Supplement: Additional file 1 — Curated sequences of eLRR proteins. List of curated sequences of eLRR proteins in FASTA format. [file 1471-2164-8-320-S1.doc]

>Peptide_ID; Gene name Other annotation (species, length, source – e.g., RefSeq, Ensembl release 40, etc. – default for Ensembl is release 38 or 37 or whatever it was)

>ENSMUSP00000025993; Slit1 Mm(1531aa) MALTPQRGSSSGLSRPELWLLLWAAAWRLGATACPALCTCTGTTVDCHGTGLQAIPKNIPRNTERLELNGNNITRIHKNDFAGLKQLRVLQLMENQIGAVERGAFDDMKELERLRLNRNQLQVLPELLFQNNQALSRLDLSENFLQAVPRKAFRGATDLKNLQLDKNRISCIEEGAFRALRGLEVLTLNNNNITTIPVSSFNHMPKLRTFRLHSNHLFCDCHLAWLSQWLRQRPTIGLFTQCSGPASLRGLNVAEVQKGEFSCSGQGEAAGAPACTLSSGSCPAMCSCSSGIVDCRGKGLTAIPANLPETMTEIRLELNGIKSIPPGAFSPYRKLRRIDLSNNQIAEIAPDAFQGLRSLNSLVLYGNKITDLPRGVFGGLYTLQLLLLNANKINCIRPDAFQDLQNLSLLSLYDNKIQSLAKGTFTSLRAIQTLHLAQNPFICDCNLKWLADFLRTNPIETTGARCASPRRLANKRIGQIKSKKFRCSAKEQYFIPGTEDYHLNSECTSDVACPHKCRCEASVVECSSLKLSKIPERIPQSTTELRLNNNEISILEATGLFKKLSHLKKINLSNNKVSEIEDGTFEGAASVSELHLTANQLESIRSGMFRGLDGLRTLMLRNNRISCIHNDSFTGLRNVRLLSLYDNHITTISPGAFDTLQALSTLNLLANPFNCNCHLSWLGDWLRKRKIVTGNPRCQNPDFLRQIPLQDVAFPDFRCEEGQEEVGCLPRPQCPQECACLDTVVRCSNKHLQALPKGIPKNVTELYLDGNQFTLVPGQLSTFKYLQLVDLSNNKISSLSNSSFTNMSQLTTLILSYNALQCIPPLAFQGLRSLRLLSLHGNDVSTLQEGIFADVTSLSHLAIGANPLYCDCRLRWLSSWVKTGYKEPGIARCAGPPEMEGKLLLTTPAKKFECQGPPSLAVQAKCDPCLSSPCQNQGTCHNDPLEVYRCTCPSGYKGRHCEVSLDGCSSNPCGNGGTCHAQEGEDAGFTCSCPSGFEGPTCGVDTDDCVKHACVNGGVCVDGVGNYTCQCPLQYTGRACEQLVDFCSPDMNPCQHEAQCVGTPDGPRCECMLGYTGDNCSENQDDCKDHKCQNGAQCVDEVNSYACLCVEGYSGQLCEIPPAPRSSCEGTECQNGANCVDQGSRPVCQCLPGFGGPECEKLLSVNFVDRDTYLQFTDLQNWPRANITLQVSTAEDNGILLYNGDNDHIAVELYQGHVRVSYDPGSYPSSAIYSAETINDGQFHTVELVTFDQMVNLSIDGGSPMTMDNFGKHYTLNSEAPLYVGGMPVDVNSAAFRLWQILNGTSFHGCIRNLYINNELQDFTKTQMKPGVVPGCEPCRKLYCLHGICQPNATPGPVCHCEAGWGGLHCDQPVDGPCHGHKCVHGKCVPLDALAYSCQCQDGYSGALCNQVGAVAEPCGGLQCLHGHCQASATKGAHCVCSPGFSGELCEQESECRGDPVRDFHRVQRGYAICQTTRPLSWVECRGACPGQGCCQGLRLKRRKLTFECSDGTSFAEEVEKPTKCGCAQCA

>ENSMUSP00000033967; Slit2 Mm(1521aa)

MSGIGWQTLSLSLGLVLSILNKVAPQACPAQCSCSGSTVDCHGLALRSVPRNIPRNTERLDLNGNNITRITKIDFAGLRHLRVLQLMENRISTIERGAFQDLKELERLRLNRNNLQLFPELLFLGTAKLYRLDLSENQIQAIPRKAFRGAVDIKNLQLDYNQISCIEDGAFRALRDLEVLTLNNNNITRLSVASFNHMPKLRTFRLHSNNLYCDCHLAWLSDWLRQRPRVGLYTQCMGPSHLRGHNVAEVQKREFVCSGHQSFMAPSCSVLHCPAACTCSNNIVDCRGKGLTEIPTNLPETITEIRLEQNSIRVIPPGAFSPYKKLRRLDLSNNQISELAPDAFQGLRSLNSLVLYGNKITELPKSLFEGLFSLQLLLLNANKINCLRVDAFQDLHNLNLLSLYDNKLQTVAKGTFSALRAIQTMHLAQNPFICDCHLKWLADYLHTNPIETSGARCTSPRRLANKRIGQIKSKKFRCSGTEDYRSKLSGDCFADLACPEKCRCEGTTVDCSNQRLNKIPDHIPQYTAELRLNNNEFTVLEATGIFKKLPQLRKINFSNNKITDIEEGAFEGASGVNEILLTSNRLENVQHKMFKGLESLKTLMLRSNRISCVGNDSFIGLGSVRLLSLYDNQITTVAPGAFDSLHSLSTLNLLANPFNCNCHLAWLGEWLRRKRIVTGNPRCQKPYFLKEIPIQDVAIQDFTCDDGNDDNSCSPLSRCPSECTCLDTVVRCSNKGLKVLPKGIPKDVTELYLDGNQFTLVPKELSNYKHLTLIDLSNNRISTLSNQSFSNMTQLLTLILSYNRLRCIPPRTFDGLKSLRLLSLHGNDISVVPEGAFNDLSALSHLAIGANPLYCDCNMQWLSDWVKSEYKEPGIARCAGPGEMADKLLLTTPSKKFTCQGPVDITIQAKCNPCLSNPCKNDGTCNNDPVDFYRCTCPYGFKGQDCDVPIHACISNPCKHGGTCHLKEGENAGFWCTCADGFEGENCEVNIDDCEDNDCENNSTCVDGINNYTCLCPPEYTGELCEEKLDFCAQDLNPCQHDSKCILTPKGFKCDCTPGYIGEHCDIDFDDCQDNKCKNGAHCTDAVNGYTCVCPEGYSGLFCEFSPPMVLPRTSPCDNFDCQNGAQCIIRINEPICQCLPGYLGEKCEKLVSVNFVNKESYLQIPSAKVRPQTNITLQIATDEDSGILLYKGDKDHIAVELYRGRVRASYDTGSHPASAIYSVETINDGNFHIVELLTLDSSLSLSVDGGSPKVITNLSKQSTLNFDSPLYVGGMPGKNNVASLRQAPGQNGTSFHGCIRNLYINSELQDFRKMPMQTGILPGCEPCHKKVCAHGMCQPSSQSGFTCECEEGWMGPLCDQRTNDPCLGNKCVHGTCLPINAFSYSCKCLEGHGGVLCDEEEDLFNPCQMIKCKHGKCRLSGVGQPYCECNSGFTGDSCDREISCRGERIRDYYQKQQGYAACQTTKKVSRLECRGGCAGGQCCGPLRSKRRKYSFECTDGSSFVDEVEKVVKCGCARCAS

> ENSMUSP00000066857; Mm Slit3 (1523aa)

MALGRTGAGAAVRARLALGLALASILSGPPAAACPTKCTCSAASVDCHGLGLRAVPRGIPRNAERLDLDRNNITRITKMDFAGLKNLRVLHLEDNQVSIIERGAFQDLKQLERLRLNKNKLQVLPELLFQSTPKLTRLDLSENQIQGIPRKAFRGVTGVKNLQLDNNHISCIEDGAFRALRDLEILTLNNNNISRILVTSFNHMPKIRTLRLHSNHLYCDCHLAWLSDWLRQRRTIGQFTLCMAPVHLRGFSVADVQKKEYVCPGPHSEAPACNANSLSCPSACSCSNNIVDCRGKGLTEIPANLPEGIVEIRLEQNSIKSIPAGAFTQYKKLKRIDISKNQISDIAPDAFQGLKSLTSLVLYGNKITEIPKGLFDGLVSLQLLLLNANKINCLRVNTFQDLQNLNLLSLYDNKLQTISKGLFVPLQSIQTLHLAQNPFVCDCHLKWLADYLQDNPIETSGARCSSPRRLANKRISQIKSKKFRCSGSEDYRNRFSSECFMDLVCPEKCRCEGTIVDCSNQKLARIPSHLPEYTTDLRLNDNDISVLEATGIFKKLPNLRKINLSNNRIKEVREGAFDGAAGVQELMLTGNQLETMHGRMFRGLSSLKTLMLRSNLISCVSNDTFAGLSSVRLLSLYDNRITTITPGAFTTLVSLSTINLLSNPFNCNCHMAWLGRWLRKRRIVSGNPRCQKPFFLKEIPIQDVAIQDFTCDGNEESSCQLSPRCPEQCTCVETVVRCSNRGLHALPKGMPKDVTELYLEGNHLTAVPKELSAFRQLTLIDLSNNSISMLTNHTFSNMSHLSTLILSYNRLRCIPVHAFNGLRSLRVLTLHGNDISSVPEGSFNDLTSLSHLALGTNPLHCDCSLRWLSEWVKAGYKEPGIARCSSPESMADRLLLTTPTHRFQCKGPVDINIVAKCNACLSSPCKNNGTCSQDPVEQYRCTCPYSYKGKDCTVPINTCVQNPCEHGGTCHLSENLRDGFSCSCPLGFEGQRCEINPDDCEDNDCENSATCVDGINNYACLCPPNYTGELCDEVIDYCVPEMNLCQHEAKCISLDKGFRCECVPGYSGKLCETNNDDCVAHKCRHGAQCVDEVNGYTCICPQGFSGLFCEHPPPMVLLQTSPCDQYECQNGAQCIVVQQEPTCRCPPGFAGPRCEKLITVNFVGKDSYVELASAKVRPQANISLQVATDKDNGILLYKGDNDPLALELYQGHVRLVYDSLSSPPTTVYSVETVNDGQFHSVELVMLNQTLNLVVDKGAPKSLGKLQKQPAVGSNSPLYLGGIPTSTGLSALRQGADRPLGGFHGCIHEVRINNELQDFKALPPQSLGVSPGCKSCTVCRHGLCRSVEKDSVVCECHPGWTGPLCDQEARDPCLGHSCRHGTCMATGDSYVCKCAEGYGGALCDQKNDSASACSAFKCHHGQCHISDRGEPYCLCQPGFSGHHCEQENPCMGEIVREAIRRQKDYASCATASKVPIMECRGGCGSQCCQPIRSKRRKYVFQCTDGSSFVEEVERHLECGCRACS

>OTTMUSP00000016116; Mm Lgr4 (951aa) (Vega sequence)

MPGPLRLLCFFALGLLGSAGPSGAAPPLCAAPCSCDGDRRVDCSGKGLTAVPEGLSAFTQALDISMNNITQLPEDAFKNFPFLEELQLAGNDLSFIHPKALSGLKELKVLTLQNNQLKTVPSEAIRGLSALQSLRLDANHITSVPEDSFEGLVQLRHLWLDDNILTEVPVRPLSNLPTLQALTLALNNISSIPDFAFTNLSSLVVLHLHNNKIKSLSQHCFDGLDNLETLDLNYNNLDEFPQAIKALPSLKELGFHSNSISVIPDGAFAGNPLLRTIHLYDNPLSFVGNSAFHNLSDLHSLVIRGASLVQWFPNLAGTVHLESLTLTGTKISSIPDDLCQNQKMLRTLDLSYNDIRDLPSFNGCRALEEISLQRNQISLIKETTFQGLTSLRILDLSRNLIREIHSGAFAKLGTITNLDVSFNELTSFPTEGLNGLNQLKLVGNFQLKDALAARDFANLRSLSVPYAYQCCAFWGCDSYANLNTEDNSPQDHSVTKEKGATDAANATSTAESEEHSQIIIHCTPSTGAFKPCEYLLGSWMIRLTVWFIFLVALLFNLLVILTVFASCSSLPASKLFIGLISVSNLLMGIYTGILTFLDAVSWGRFAEFGIWWETGSGCKVAGSLAVFSSESAVFLLTLAAVERSVFAKDVMKNGKSSHLRQFQVAALVALLGAAIAGCFPLFHGGQYSASPLCLPFPTGETPSLGFTVTLVLLNSLAFLLMAIIYTKLYCNLEKEDPSENSQSSMIKHVAWLIFTNCIFFCPVAFFSFAPLITAISISPEIMKSVTLIFFPLPACLNPVLYVFFNPKFKDDWKLLKRRVTRKHGSVSVSISSQGGCGEQDFYYDCGMYSHLQGNLTVCDCCESFLLTKPVSCKHLIKSHSCPVLTVASCQRPEAYWSDCGTQSAHSDYADEEDSFVSDSSDQVQACGRACFYQSRGFPLVRYAYNLPRVRD

>ENSMUSP00000020350; Mm Lgr5 (907aa)

MDTSCVHMLLSLLALLQLVAAGSSPGPDAIPRGCPSHCHCELDGRMLLRVDCSDLGLSELPSNLSVFTSYLDLSMNNISQLPASLLHRLCFLEELRLAGNALTHIPKGAFTGLHSLKVLMLQNNQLRQVPEEALQNLRSLQSLRLDANHISYVPPSCFSGLHSLRHLWLDDNALTDVPVQAFRSLSALQAMTLALNKIHHIADYAFGNLSSLVVLHLHNNRIHSLGKKCFDGLHSLETLDLNYNNLDEFPTAIKTLSNLKELGFHSNNIRSIPERAFVGNPSLITIHFYDNPIQFVGVSAFQHLPELRTLTLNGASHITEFPHLTGTATLESLTLTGAKISSLPQAVCDQLPNLQVLDLSYNLLEDLPSLSGCQKLQKIDLRHNEIYEIKGSTFQQLFNLRSLNLAWNKIAIIHPNAFSTLPSLIKLDLSSNLLSSFPVTGLHGLTHLKLTGNRALQSLIPSANFPELKIIEMPSAYQCCAFGGCENVYKISNQWNKDDGNSVDDLHKKDAGLFQVQDERDLEDFLLDFEEDLKALHSVQCSPSPGPFKPCEHLFGSWLIRIGVWTTAVLALSCNALVALTVFRTPLYISSIKLLIGVIAVVDILMGVSSAVLAAVDAFTFGRFAQHGAWWEDGIGCQIVGFLSIFASESSIFLLTLAALERGFSVKCSSKFEVKAPLFSLRAIVLLCVLLALTIATIPLLGGSKYNASPLCLPLPFGEPSTTGYMVALVLLNSLCFLIMTIAYTKLYCSLEKGELENLWDCSMVKHIALLLFANCILYCPVAFLSFSSLLNLTFISPDVIKFILLVIVPLPSCLNPLLYIVFNPHFKEDMGSLGKHTRFWMRSKHASLLSINSDDVEKRSCESTQALVSFTHASIAYDLPSTSGASPAYPMTESCHLSSVAFVPCL

>ENSMUSP00000035444; Mm Lgr6 (967aa)

MHSPPGLLALWLCAVLCASARAGSDPQPGPGRPACPAPCHCQEDGIMLSADCSELGLSVVPADLDPLTAYLDLSMNNLTELQPGLFHHLRFLEELRLSGNHLSHIPGQAFSGLHSLKILMLQSNQLRGIPAEALWELPSLQSLRLDANLISLVPERSFEGLSSLRHLWLDDNALTEIPVRALNNLPALQAMTLALNHIRHIPDYAFQNLTSLVVLHLHNNRIQHVGTHSFEGLHNLETLDLNYNELQEFPLAIRTLGRLQELGFHNNNIKAIPEKAFMGSPLLQTIHFYDNPIQFVGRSAFQYLSKLHTLSLNGATDIQEFPDLKGTTSLEILTLTRAGIRLLPPGVCQQLPRLRILELSHNQIEELPSLHRCQKLEEIGLRHNRIKEIGADTFSQLGSLQALDLSWNAIRAIHPEAFSTLRSLVKLDLTDNQLTTLPLAGLGGLMHLKLKGNLALSQAFSKDSFPKLRILEVPYAYQCCAYGICASFFKTSGQWQAEDFHPEEEEAPKRPLGLLAGQAENHYDLDLDELQMGTEDSKPHPSVQCSPVPGPFKPCEHLFESWGIRLAVWAIVLLSVLCNGLVLLTVFASGPSPLSPVKLVVGAMAGANALSGISCGLLASVDALTYGQFAEYGARWESGLGCQATGFLAVLGSEASVLLLTLAAVQCSISVTCVRAYGKAPSPGSVRAGALGCLALAGLAAALPLASVGEYGASPLCLPYAPPEGRPAALGFAVALVMMNSLCFLVVAGAYIKLYCDLPRGDFEAVWDCAMVRHVAWLIFADGLLYCPVAFLSFASMLGLFPVTPEAVKSVLLVVLPLPACLNPLLYLLFNPHFRDDLRRLWPSPRSPGPLAYAAAGELEKSSCDSTQALVAFSDVDLILEASEAGQPPGLETYGFPSVTLISRHQPGATRLEGNHFVESDGTKFGNPQPPMKGELLLKAEGATLAGCGSSVGGALWPSGSLFASHL

>ENSMUSP00000040477; Mm Fshr (692aa)

MALLLVSLLAFLGSGSGCHHWLCHCSNRVFLCQDSKVTEIPPDLPRNAIELRFVLTKLRVIPKGSFSGFGDLEKIEISQNDVLEVIEADVFSNLPNLHEIRIEKANNLLYINPEAFQNLPSLRYLLISNTGIKHLPAFHKIQSLQKVLLDIQDNINIHIIARNSFMGLSFESVILWLNKNGIQEIHNCAFNGTQLDELNLSDNNNLEELPDDVFQGASGPVVLDISRTKVYSLPNHGLENLKKLRARSTYRLKKLPSLDKFVMLIEASLTYPSHCCAFANWRRQTSELHPICNKSISRQDIDDMTQPGDQRVSLVDDEPSYGKGSDMLYSEFDYDLCNEFVDVTCSPKPDAFNPCEDIMGYNILRVLIWFISILAITGNTTVLVVLTTSQYKLTVPRFLMCNLAFADLCIGIYLLLIASVDIHTKSQYHNYAIDWQTGAGCDAAGFFTVFASELSVYTLAAITLERWHTITHAMQLECKVQLCHAASIMVLGWAFAFAAALFPIFGISSYMKVSICLPMDIDSPLSQLYVMALLVLNALAFVVICGCYTHIYLTVRNPNIVSSSRDTKIAKRMATLIFTDFLCMAPILFFAISASLKVPLITVSKAKILLVLFYPINSCANPFLYAIFTKNFRRDFFVLMSKFGCYEVQAQIYKTETSSITHNFHSRKNPCSSAPRVTNSYVLVPLNHSVQN

>ENSMUSP00000024916, Mm Lhcgr (700aa)

MGRRVPALRQLLVLAMLVLKQSQLHSPELSGSRCPEPCDCAPDGALRCPGPRAGLARLSLTYLPVKVIPSQAFRGLNEVVKIEISQSDSLERIEANAFDNLLNLSEILIQNTKNLLYIEPGAFTNLPRLKYLSICNTGIRTLPDVSKISSSEFNFILEICDNLYITTIPGNAFQGMNNESITLKLYGNGFEEVQSHAFNGTTLISLELKENIYLEKMHSGTFQGATGPSILDVSSTKLQALPSHGLESIQTLIATSSYSLKTLPSREKFTSLLVATLTYPSHCCAFRNLPKKEQNFSFSIFENFSKQCESTVREANNETLYSAIFEENELSGWDYDYDFCSPKTLQCTPEPDAFNPCEDIMGYAFLRVLIWLINILAIFGNLTVLFVLLTSRYKLTVPRFLMCNLSFADFCMGLYLLLIASVDSQTKGQYYNHAIDWQTGSGCSAAGFFTVFASELSVYTLTVITLERWHTITYAVQLDQKLRLRHAIPIMLGGWIFSTLMATLPLVGVSSYMKVSICLPMDVESTLSQVYILSILLLNAVAFVVICACYVRIYFAVQNPELTAPNKDTKIAKKMAILIFTDFTCMAPISFFAISAAFKVPLITVTNSKVLLVLFYPVNSCANPFLYAVFTKAFQRDFFLLLSRFGCCKHRAELYRRKEFSACTFNSKNGFPRSSKPSQAALKLSIVHCQQPTPPRVLIQ

>ENSMUSP00000021346; Mm Tshr (764aa)

MRPGSLLLLVLLLALSRSLRGKECASPPCECHQEDDFRVTCKELHRIPSLPPSTQTLKLIETHLKTIPSLAFSSLPNISRIYLSIDATLQRLEPHSFYNLSKMTHIEIRNTRSLTYIDPDALTELPLLKFLGIFNTGLRIFPDLTKIYSTDIFFILEITDNPYMTSVPENAFQGLCNETLTLKLYNNGFTSVQGHAFNGTKLDAVYLNKNKYLTAIDNDAFGGVYSGPTLLDVSSTSVTALPSKGLEHLKELIAKDTWTLKKLPLSLSFLHLTRADLSYPSHCCAFKNQKKIRGILESLMCNESSIRNLRQRKSVNILRGPIYQEYEEDPGDNSVGYKQNSKFQESPSNSHYYVFFEEQEDEVVGFGQELKNPQEETLQAFESHYDYTVCGDNEDMVCTPKSDEFNPCEDIMGYRFLRIVVWFVSLLALLGNIFVLLILLTSHYKLTVPRFLMCNLAFADFCMGVYLLLIASVDLYTHSEYYNHAIDWQTGPGCNTAGFFTVFASELSVYTLTVITLERWYAITFAMRLDRKIRLRHAYTIMAGGWVSCFLLALLPMVGISSYAKVSICLPMDTDTPLALAYIVLVLLLNVVAFVVVCSCYVKIYITVRNPQYNPRDKDTKIAKRMAVLIFTDFMCMAPISFYALSALMNKPLITVTNSKILLVLFYPLNSCANPFLYAIFTKAFQRDVFILLSKFGICKRQAQAYQGQRVCPNNSTGIQIQKIPQDTRQSLPNMQDTYELLGNSQLAPKLQGQISEEYKQTAL

>ENSMUSP00000077611; Mm Lgr7=Rxfp1(758aa)

MTSGPFFFCIFIIGKYFTLGSAQDVSCPLGSFPCGNMSRCLPQLLHCNGVDDCGNRADEDHCGDNNGWSLQLDKYFANYYKLASTNSFEAETSECLVGSVPMHCLCRDLELDCDEANLRAVPSVSSNVTVMSLQRNFIRTLPPNGFRKYHELQKLCLQNNRIHSVSVSAFRGLRSLTKLYLSHNRITFLKPGVFEDLHRLEWLIIEDNHLSRISPLTFYGLNSLILLVLMNNALTRLPDKPLCQHMPRLHWLDFEGNRIHNLRNLTFISCNNLTVLVMRKNKINYLNEHAFTHLQKLDELDLGSNKIENLPPNIFKDLKELSQLNISYNPIQKIEVNQFDCLAKLKSLSLEGIEISNIQQRMFRPLINLSHIYFKKFQYCGYAPHVRSCKPNTDGISSLENLLASIIQRVFVWVVSAITCFGNIFVICMRPYIRSENKLHAMSIISLCCADCLMGVYLFVIGAFDLKFRGEYNKHAQPWMESVHCQFMGSLAILSTEVSVLLLTFLTLEKYICIVYPFRCLRPRKCRTITVLIFIWIIGFIVAFAPLGNKEFFKNYYGTNGVCFPLHSEDTGSTGAQIYSVVIFLGINLVAFIIIVFSYGSMFYSVHQSSVTVTEIQKQVKKEVVLAKRFFFIVFTDALCWIPIFILKFLSLLQVEIPDSITSWVVIFILPINSALNPIIYTLTTRPFKEMIHQLWHNYRQRRSVDRKETQKAYAPSFIWVEMWPLQEMSSGFMKPGAFTDPCDLSLVSQSSRLNSYS

>ENSMUSP00000067897; Mm Lgr8=Rxfp2 (737aa)

MWLLLHVILLTEVKDFALADSSMVAPLCPKGYFPCGNLTKCLPRAFHCDGVDDCGNGADEDNCGDTSGWTTIFGTVHGNVNKVTLTQECFLSQYPQHCYCRENELECVKADLKAVPKVSSNVTLLSLKKNKIHRLPVKVFSRYTELRKIYLQHNCITHISRRAFLGLHNLQILYLSHNCITSLRPGIFKDLHQLAWLILDDNPITRISQKSFMGLNSLFFLSMVGNRLEALPETLCAQMPQLNWVDLANNGIKYITNSTFLTCDSLTVLFLPRNQIGFVPEKTFSSLKNLGELDLSSNMITKLPVHLFSDLHLLQKLNLSSNPLLYVHKNQFGSLKQLQSLDLERIEIPNISTGMFQPMKNLSHIYLKTFRYCSYVPHVRICMPSTDGISSSEDLLANGILRVSVWVIAFITCVGNFLVIAVRSLIKAENTTHAMSIKILCCADCLMGVYLFSVGVFDIKYRGQYQKYALLWMESVPCRLLGFLATLSTEVSVLLLTFLTLEKFLVIVFPFSNLRLGKRQTAVALASIWVVGFLIAAVPFTREDYFGNFYGKNGVCFPLHYDQAEDFGSRGYSLGIFLGVNLLAFLVIVISYVTMFCSIHKTALQTAEVRSHIGKEVAVANRFFFIVFSDAICWIPVFVVKILSLLQVEIPGTITSWIVVFFLPVNSALNPILYTLTTSFFKDKLKQLLHKHRRKPIFKVKKKSLSASIVWTDESSLKLGVLSKIALGDSIMKPVSP

>ENSMUSP00000049296; Mm Pkd1 (4293aa)

MPLGAPALLALALGLGLWLGALAGDPGRGCGPCPLPCFCGPAPDAACRVNCSGRWLQTLGPSLRIPADATALDLSHNLLQTLDIGLLVNLSALVELDLSNNRISTLEEGVFANLFNLSEINLSGNPFECNCGLAWLPRWAKEHQVHVVQSEATTCRGPIPLAGQPLLSIPLLDNACGEEYVACLPDNSSGAVAAVPFYFAHEGPLETEACSAFCFSAGEGLAALSEQNQCLCGAGQASNSSAACSSWCSSISLSLNSACGGPTLLQHTFPASPGATLVGPHGPLASGQPADFHITSSLPISSTRWNFGDGSPEVDMASPAATHFYVLPGSYHMTVVLALGAGSALLETEVQVEATPTVLELVCPSFVHSNESLELGIRHRGGSALEVTYSILALDKEPAQVVHPLCPLDTEIFPGNGHCYRLVAEKAPWLQAQEQCRTWAGAALAMVDSPAIQHFLVSKVTRSLDVWIGFSSVEGTEGLDPRGEAFSLESCQNWLPGEPHPATAEHCVRLGPAGQCNTDLCSAPHSYVCELRPGGPVWDTENFVMGMSGGGLSGPLHPLAQQETVQGPLRPVEVMVFPGLSPSREAFLTAAEFSTQKLEEPAQMRLQVYRPSGGAAAVPEGSSEPDNRTEPAPKCVPEELWCPGANVCIPFDASCNSHVCINGSVSRLGLSRASYTLWKEFFFSVPAGPPTQYLVTLHSQDVPMLPGDLIGLQHDAGPGTLLQCPLASSCPGQALYLSTNASDWMTNLPVHLEEAWAGPVCSLQLLLVTERLTPLLGLGPNPGLQHPGHYEVRATVGNSVSRQNLSCSFSVVSPIAGLRVIHPIPLDGHIYVPTNGSVLVLQVDSGANATATAQWFGGNISAPFEDACPPEVDFLKQDCTEEANGTLFSVLMLPRLKEGDHTVEIVAQNGASQANLSLRVTAEEPICGLRAVPSPEARVLQGILVRYSPMVEAGSDVAFRWTIDDKQSLTFHNTVFNVIYQSAAIFKLSLTASNHVSNITVNYNVTVERMNKMHGLWVSAVPTVLPPNATLALTGGVLVDSAVEVAFLWNFGDGEQVLRQFKPPYDESFQVPDPTVAQVLVEHNTTHIYTTPGEYNLTVLVSNTYENLTQQVTVSVRTVLPNVAIGMSSNVLVAGQPITFSPYPLPSTDGVLYTWDFGDGSPVLIQSQPVLNHTYSMTGAYRITLEVNNTVSSVTAHADIRVFQELHGLTVYLSPSVEQGAPMVVSASVESGDNITWTFDMGDGTVFTGPEATVQHVYLRAQNFTVTVEAANPAGHLSQSLHVQVFVLEVLHIEPSTCIPTQPSAQLMAHVTGDPVHYLFDWTFGDGSSNVTVHGHPSVTHNFTRSGIFPLALVLSSHVNKAHYFTSICVEPEIRNITLQPERQFVKLGDEARLVAYSWPPFPYRYTWDFGTEDTTHTQTGGSEVKFIYREPGSYLVIVTVSNNISSTNDSAFVEVQEPVLVTGIRINGSHVLELQQPYLLSAMGSGSPATYLWELGDGSQSEGPEVTHIYSSTGDFTVRVSGWNEVSRSEAQLNITVKQRVRGLTINASRTVVPLNGSVSFSTLLEVGSDVHYSWVLCDRCTPIPGGPTISYTFRSVGTFNIIVTAENEVGSAQDSIFIYVLQFIEGLQVAGGDNGCCFPTNYTLQLQAAVRDGTNISYSWTAQQEGSLITLFGSGKCFSLTSLKASTYYVHLRATNMLGSAAANRTIDFVEPVESLILSASPNPAAVNMSLTLCAELAGGSGVVYTWYLEEGLSWKTSMPSTTHTFAAPGLHLVRVTAENQLGSVNATVEVAIQVPVGGLSIRTSEPDSIFVAAGSTLPFWGQLAEGTNVTWCWTLPGGSKDSQYIAVRFSTAGSFSLQLNASNAVSWVSAMYNLTVEEPIVNLMLWASSKVVAPGQPVHFEILLAAGSALTFRLQVGGSVPEVLPSPHFSHSFFRVGDHLVNVQAENHVSHAQAQVRILVLEAVVGLQVPNCCEPGMATGTEKNFTARVQRGSRVAYAWYFSLQKVQGDSLVILSGRDVTYTPVAAGLLEIHVRAFNELGGVNLTLMVEVQDIIQYVTLQSGRCFTNRSARFEAATSPSPRRVTYHWDFGDGTPVQKTEEFWADHYYLRPGDYHVEVNATNLVSFFVAQATVTVQVLACREPEVEVALPLQVLMRRSQRNYLEAHVDLRNCVSYQTEYRWEIYRTASCQRPGRMAQMVLPGVDVSRPQLVVPRLALPVGHYCFVFVVSFGDTPLARSIQANVTVAAERLVPIIEGGSYRVWSDTQDLVLDGSKSYDPNLEDGDQTPLNFHWACVASTQSETGGCVLNFGPRGSSVVTIPLERLEAGVEYTFNLIVWKAGRKEEATNQTVLIRSGRVPIVSLECVSCKAQAVYEVSRSSYVYLEGHCHNCSRGYKQGCWAARTFSNKTLVLNETTTSTGSTGMNLVVRPGALRDGEGYIFTLTVLGHSGEEEGCASIRLSPNRPPLGGSCRLFPLDSVRGLTTKVHFECTGWRDAEDGGAPLVYALLLKRCRQSYCENFCIYKGSLSTYGAVLPPGFQPLFVVSLAVVVQDQLGAAVVALNRSLTIVLPEPSGNPADLVPWLHSLTASVLPGLLKQADPQHVIEYSLALITVLNEYEQAPDVSEPNVEQQLRAQMRKNITETLISLRVNTVDDIQQITAALAQCMVSSRELMCRSCLKKMLQKLEGMMRILQAETTEGTLTPTTIADSILNITGDLIHLASLDMQGPQPLELGVEPPSLMVASKAYNLSSALMRILMRSRVLNEEPLTLAGEEIVALGKRSDPLSLLCYGKALGPSCHFSIPEAFSGALSNLSDVVQLIFLVDSNPFPFGYISNYTVSTKVASMAFQTQTGTQIPIEQLAAERAITVKVPNNSDQAAQSSHNPVGSTIVQPQTSVSAVVTADNSNPQAGLHLRITYTVLNERYLSAEPEPYLAVYLHSVSQPNEYNCSASRRISLEVLEGADHRLYTFFIAPGTGTLDRSYYLNLTSHFHWSALEVSVGLYTSLCQYFSEEMMMWRTEGIVPLEETSPSQAVCLTRHLTAFGASLFVPPSHVQFIFPEPSASINYIVLLTCVICLVTYVVMAMILRKLDQLDVSRVRVIPFCGKGGRFKYEILVKTGWSRGSGTTAHVGIMLYGEDNRSGHRHLDGDRAFHRNSLDIFQIATPHSLGSVWKIRVWHDNKGLSPAWFLQHIIVRDLQSARSTFFLVNDWLSVETEANGGLVEKEVLAANEAALWQFQRLLVAELQRGFFDKHIWLSIWDRPPRSRFTRVQRVTCCVLLLCLFLAANAVWYGVVRDTTYSMGPVSSLISPGVDTVAIGLVSSVVVYPVYLAVLFLFRMSRSKVSGDQNPTPTGQQALDVDSYLDPSVLDSSLLTLSGLTEAFAGQVKNDLFLEDAKSLVCWPSSEGTLSWPDLLSDPSVVSSTLQRLTQGRPGCMLGSEEDGASLVSPSLPAKYLSASDEDLIHQVLADGANNLVPTQDTLLETDLLTSLSSVPGEKTETLILQTVGEERPASMGLSWEQSPVTRLSRTGLVEGFQKRLLPAWCAPLAHGLSLLLVAVAVAVSGWIGASFPPSVSVMWLLSSSSSFLASFLGWEPLKVLLEALYFSLVAKRLHPDEDDTLVESPAVTPVSERVPRVRPPHGFALFLAKEEARKVKRLHDMLKRLLVYMLFLLVTLLANYGDASCHGHAYRLQSAIKQELDSQAFLAITRSDEFWPWMSHVFLPYVHGNQSSPELGPPRLRQVRLQEAFCPDPSSSEHMCSAAGSLSTSDYGIGWQSVVQNGSETWAYSAPDLLGAWYWGYCAVYDSGGYIQELGLSLEESRARLGFLQLHNWLDSRSRAVFVELTRYSPAVGLHAAVTLRLEFPVAGHALAAFSVRPFALRRLSTGLSLPLLTSVCLLLFALYFSMAEVQTWRKDGCACTARPDTWARCLLVILTAATGLVRLAQLGIADRQWTHFVQDHPRHFTSFDQVAQLGSVARGLAASLLFLLLVKAAQQLRFVRQWSVFGKTLCRALPELMGATLGLVLLGVAYAQMAILLISSGADTLYNMARAFLVLCPGARVPTLCPSESWYLSPLLCVGLWALRVWGALRLGAILLRWRYHALRGELYRPAWEPQDYEMVELFLRRLRLWMGFSKVKEFRHKVRFEGMDPLPSRSSRGSKSSPVVLPPSSGSEASHPSTSSSQPDGPSASLSRSTLKLEPEPSRLHAVFESLLVQFDRLNQATEDVYQLEQQLQSLQGHGHNGPPSSPSPGCFPGSQPALPSRLSRASQGLDQTVGPNRVSLWPNNKVHPSST

>ENSMUSP00000060402; Mm Ecm2 (670aa)

MKLAVLFCFILLIVLQTDCERGTRRQRRRMHQRRLRKSSSFHLRANRQLEVQQTTAAPDARLPTANSDYSVEENIESLLSNLGVESSYSVLPGKKGYCFVKGMIMYNKAVWSPEPCTTCLCSNGRVLCDETECHPKACPYTIKPEGECCPICSDAEQESINKLHKQVPPPQMEMDQVAIKEALQSEEDEEIAEGHKEHKKETSVPTKIHGDGERTERKLRPEKEGRSAHQPLYHGRREEEESKEETEREGEEEEEEEEEEEEDAIRGDVFRMSSRVIPGTPRGRPRLPRSCSLSYRTISCVHADFTEIPPITAPEVTNLELVGNSIISIPDEAFNGLPNLERLDLSRNNITSSGIGPKAFKSLKKLMRLNMDGNNLVHIPSDLPSTLEELKINDNNLQAIDEKSLSDLNQLVTLELEGNNLSEINVDPLAFQSLESLSYLRLGRNKFRIIPQGLPASTEELYLENNQIEEITEICFNHTRKITMIILRYNKIEESRIAPLAWINQENLESIDLSYNKLYHVPSYLPKSLLHLVLIGNQIDRIPGYVFGHMQPGLEYLYLSFNRLSDDGVDLVSFYGAYHSLRELFLDHNDFKSIPPGIQDMKALHFLRLNNNKIRNIHPEQICNAEEDEDSALEHLHLENNYIRTREISSYAFSCIRLYSSIVLKPQHIK

>ENSMUSP00000033876; Mm Gpr124 (1336aa)

MGAGGRRMPVPPARLLLLPLLPCLLLLAPGTRGAPGCPVPIRGCKCSGERPKGLSGGAHNPARRRVVCGGGDLPEPPDPGLLPNGTITLLLSNNKITGLRNGSFLGLSLLEKLDLRSNVISTVQPGAFLGLGELKRLDLSNNRIGCLTSETFQGLPRLLRLNISGNIYSSLQPGVFDELPALKIVDFGTEFLTCDCRLRWLLPWARNHSLQLSERTLCAYPSALHAHALSSLQESQLRCEGALELHTHYLIPSLRQVVFQGDRLPFQCSASYLGNDTRIHWYHNGAPMESDEQAGIVLAENLIHDCTFITSELTLSHIGVWASGEWECSVSTVQGNTSKKVEIVVLETSASYCPAERVTNNRGDFRWPRTLAGITAYQSCLQYPFTSVPLSGGAPGTRASRRCDRAGRWEPGDYSHCLYTNDITRVLYTFVLMPINASNALTLAHQLRVYTAEAASFSDMMDVVYVAQMIQKFLGYVDQIKELVEVMVDMASNLMLVDEHLLWLAQREDKACSGIVGALERIGGAALSPHAQHISVNSRNVALEAYLIKPHSYVGLTCTAFQRREVGVSGAQPSSVGQDAPVEPEPLADQQLRFRCTTGRPNISLSSFHIKNSVALASIQLPPSLFSTLPAALAPPVPPDCTLQLLVFRNGRLFRSHGNNTSRPGAAGPGKRRGVATPVIFAGTSGCGVGNLTEPVAVSLRHWAEGADPMAAWWNQDGPGGWSSEGCRLRYSQPNVSSLYCQHLGNVAVLMELNAFPREAGGSGAGLHPVVYPCTALLLLCLFSTIITYILNHSSIHVSRKGWHMLLNLCFHMAMTSAVFVGGVTLTNYQMVCQAVGITLHYSSLSSLLWMGVKARVLHKELSWRAPPLEEGEAAPPGPRPMLRFYLIAGGIPLIICGITAAVNIHNYRDHSPYCWLVWRPSLGAFYIPVALILPITWIYFLCAGLHLRSHVAQNPKQGNRISLEPGEELRGSTRLRSSGVLLNDSGSLLATVSAGVGTPAPPEDGDGVYSPGVQLGALMTTHFLYLAMWACGALAVSQRWLPRVVCSCLYGVAASALGLFVFTHHCARRRDVRASWRACCPPASPSASHVPARALPTATEDGSPVLGEGPASLKSSPSGSSGRAPPPPCKLTNLQVAQSQVCEASVAARGDGEPEPTGSRGSLAPRHHNNLHHGRRVHKSRAKGHRAGETGGKSRLKALRAGTSPGAPELLSSESGSLHNSPSDSYPGSSRNSPGDGLPLEGEPMLTPSEGSDTSAAPIAETGRPGQRRSASRDNLKGSGSALERESKRRSYPLNTTSLNGAPKGGKYEDASVTGAEAIAGGSMKTGLWKSETTV

>ENSMUSP00000030971; Mm Gpr125 (1310aa)

MEPPPPLLLLPLALLALLWGGERGAAALPAGCKHDGRARGTGRAAAAAEGKVVCSSLELAQVLPPDTLPNRTVTLILSNNKISELKNGSFSGLSLLERLDLRNNLISRIAPGAFWGLSSLKRLDLTNNRIGCLNADVFRGLTNLVRLNLSGNLFTSLSQGTFDYLGSLRSLEFQTEYLLCDCNILWMHRWVKERNITVRDTRCVYPKSLQAQPVTGVKQELLTCDPPLELPSFYMTPSHRQVVFEGDSLPFQCMASYIDQDMQVLWYQDGRIVETDESQGIFVEKSMIHNCSLIASALTISNIQAGSTGNWGCHVQTKRGNNTRTVDIVVLESSAQYCPPERVVNNKGDFRWPRTLAGITAYLQCTRNTHSSGIYPGSAQDERKAWRRCDRGGFWADDDYSRCQYANDVTRVLYMFNQMPLNLTNAVATARQLLAYTVEAANFSDKMDVIFVAEMIEKFGRFTREEKSKELGDVMVDVASNIMLADERVLWLAQREAKACSRIVQCLQRIATHRLASGAHVYSTYSPNIALEAYVIKAAGFTGMTCSVFQKVAASDRAGLSDYGRRDPDGNLDKQLSFKCNVSSTFSSLALKNTIMEASIQLPSSLLSPKHKREARAADDALYKLQLIAFRNGKLFPATGNSTKLADDGKRRTVVTPVILTKIDGATVDTHHIPVNVTLRRIAHGADAVAAQWDFDLLNGQGGWKSDGCCILYSDENITTIQCGSLGNYAVLMDLTGTELYTPAASLLHPVVYTTAITLLLCLLAVIISYMYHHSLIRISLKSWHMLVNLCFHILLTCVVFVGGITQTRNASVCQAVGIILHYSTLATVLWVGVTARNIYKQVTKKAKRCQDPDEPPAPPRPMLRFYLIGGGIPIIVCGITAAANIKNYGSRPSAPYCWMAWEPSLGAFYGPASFITFVNCMYFLSIFIQLKRHPERKYELKEPTEEQQRLAANENGEINHQDSMSLSLISTSTLENEHSFQSQLLGASLTLLLYVILWMFGAMAVSLYYPLDLVFSFFFGATCLSFSAFMMVHHCINREDVRLAWIMMCCPGRSSYSVQVNVQPPNSSATNGEAPKCTNSSAESSCTNKSASSFKNSSQGCKLTNLQAAAAQYHSNALPVNATPQLDNSLTEHSMDNDIKMHVAPLDVQFRTNVHPSRHHKNRSKGHRASRLTVLREYAYDVPTSVEGSVQNGLPKSRPGSNEGHSRSRRAYLAYRERQYNPPQQDSSDACSTLPKSSRNVEKPVSTSSKKDAPRKPAAADLESQQKSYGLNLAVQNGPVKSNGQEGPLLATDVTGNVRTGLWKHETTV

>ENSMUSP00000007620; Mm Lrrc8=Lrrc8a (810aa)

MIPVTELRYFADTQPAYRILKPWWDVFTDYISIVMLMIAVFGGTLQVTQDKMICLPCKWVTKDSCNDSFRGWAASSPEPTYPNSTVLPTPDTGPTGIKYDLDRHQYNYVDAVCYENRLHWFAKYFPYLVLLHTLIFLACSNFWFKFPRTSSKLEHFVSILLKCFDSPWTTRALSETVVEESDPKPAFSKMNGSMDKKSSTVSEDVEATVPMLQRTKSRIEQGIVDRSETGVLDKKEGEQAKALFEKVKKFRTHVEEGDIVYRLYMRQTIIKVIKFALIICYTVYYVHNIKFDVDCTVDIESLTGYRTYRCAHPLATLFKILASFYISLVIFYGLICMYTLWWMLRRSLKKYSFESIREESSYSDIPDVKNDFAFMLHLIDQYDPLYSKRFAVFLSEVSENKLRQLNLNNEWTLDKLRQRLTKNAQDKLELHLFMLSGIPDTVFDLVELEVLKLELIPDVTIPPSIAQLTGLKELWLYHTAAKIEAPALAFLRENLRALHIKFTDIKEIPLWIYSLKTLEELHLTGNLSAENNRYIVIDGLRELKRLKVLRLKSNLSKLPQVVTDVGVHLQKLSINNEGTKLIVLNSLKKMVNLTELELIRCDLERIPHSIFSLHNLQEIDLKDNNLKTIEEIISFQHLHRLTCLKLWYNHIAYIPIQIGNLTNLERLYLNRNKIEKIPTQLFYCRKLRYLDLSHNNLTFLPADIGLLQNLQNLAVTANRIEALPPELFQCRKLRALHLGNNVLQSLPSRVGELTNLTQIELRGNRLECLPVELGECPLLKRSGLVVEEDLFSTLPPEVKERLWRADKEQA

>NP_001028722; Mm Lrrc8b (803aa)

MITLTELKCLADAQSSYHILKPWWDVFWYYITLIMLLVAVLAGALQLTQSRVLCCLPCKVEFDNQCAVPWDLLKGSENASSNSGLLLPLPLRIQNDLHRQQYSYIDAVCYEKQLHWFAKFFPYLVLLHTLIFAACSNFWLHYPSTSSRLEHFVSILHKCFDSPWTTRALPLKLSKSKTLLSTSGGSADIDASKQSLPYPQPGLESPGIESPTSSVLDKKEGEQAKAIFEKVKRFRLHVEQRDIIYRVYLKQIIVKVILFVLIITYVPYFLSYITLEIDCSIDVQAFTGYKRYQCVYSLAEIFKVLASFYVILVMLYGLTSSYSLWWMLRSSLKQYSFEALREKSNYSDIPDVKNDFAFILHLADQYDPLYSKRFSIFLSEVSENKLKQINLNNEWTVERLKSKLVKNSQDKVELHLFMLNGLPDNVFELTEMEVLSLELIPEVKLPAAVAQLVNLRELHVYHSSLVVDHPALAFLEENLRILRLKFTEMGKIPRWVFHLKNLKELYLSGCVLPEQLSSLHLEGFQDLKNLRTLYLKSSLSRIPQVVTDLLPSLQKLSLDNEGSKLVVLNNLKKMVNLKSLELLSCDLERIPHSIFSLNNLHELDLKENNLKTVEEIISFQHLPSLSCLKLWHNNIAYIPAQIGALSNLEQLFLGHNNIESLPLQLFLCTKLHYLDLSYNHLTFIPEEIQYLTNLQYFAVTNNNIEMLPDGLFQCKKLQCLLLGRNSLTDLSPLVGELSNLTHLELTGNYLETLPVELEGCQSLKRSCLIVEDSLLNSLPLPVAERLQTCLDKC

>ENSMUSP00000066015; Mm Lrrc8c (803aa)

MIPVTEFRQFSEQQPAFRVLKPWWDVFTDYLSVAMLMIGVFGCTLQVMQDKIICLPKRVQPAQNHSSVPNVSQAVISTTPLPPPKPSPTNPATVEMKGLKTDLDLQQYSFINQMCYERALHWYAKYFPYLVLIHTLVFMLCSNFWFKFPGSSSKIEHFISILGKCFDSPWTTRALSEVSGEDSEEKDNRKNNMNRSGTIQSGPEGNLVRSQSLKSIPEKFVVDKSAAGALDKKEGEQAKALFEKVKKFRLHVEEGDILYAMYVRQTVLKVIKFLIIIAYNSALVSKVQFTVDCNVDIQDMTGYKNFSCNHTMAHLFSKLSFCYLCFVSIYGLTCLYTLYWLFYRSLREYSFEYVRQETGIDDIPDVKNDFAFMLHMIDQYDPLYSKRFAVFLSEVSENKLKQLNLNNEWTPDKLRQKLQTNAHNRLELPLIMLSGLPDTVFEITELQSLKLEIIKNVMIPATIAQLDNLQELCLHQCSVKIHSAALSFLKENLKVLSVKFDDMRELPPWMYGLRNLEELYLVGSLSHDISKNVTLESLRDLKSLKILSIKSNVSKIPQAVVDVSSHLQKMCVHNDGTKLVMLNNLKKMTNLTELELVHCDLERIPHAVFSLLSLQELDLKENNLKSIEEIVSFQHLRKLTVLKLWYNSIAYIPEHIKKLTSLERLFFSHNKVEVLPSHLFLCNKIRYLDLSYNDIRFIPPEIGVLQSLQYFSITCNKVESLPDELYFCKKLKTLKIGKNSLSVLSPKIGNLLFLSYLDIKGNHFEVLPPELGDCRALKRAGLVVEDALFETLPSDVREQMKAD

>ENSMUSP00000057293; Mm Lrrc5=Lrrc8d (859aa)

MFTLAEVASLNDIQPTYRILKPWWDVFMDYLAVVMLMVAIFAGTMQLTKDQVVCLPVLPSPANSKAHTPPGNADITTEVPRMETATHQDQNGQTTTNDVAFGTSAVTPDIPLQATHPHAESTLPNQEAKKEKRDPTGRKTNLDFQQYVFINQMCYHLALPWYSKYFPYLALIHTIILMVSSNFWFKYPKTCSKVEHFVSILGKCFESPWTTKALSETACEDSEENKQRITGAQTLPKHVSTSSDEGSPSASTPMINKTGFKFSAEKPVIEVPSMTILDKKDGEQAKALFEKVRKFRAHVEDSDLIYKLYVVQTLIKTAKFIFILCYTANFVNAISFEHVCKPKVEHLTGYEVFECTHNMAYMLKKLLISYISIICVYGFICLYTLFWLFRIPLKEYSFEKVREESSFSDIPDVKNDFAFLLHMVDQYDQLYSKRFGVFLSEVSENKLREISLNHEWTFEKLRQHVSRNAQDKQELHLFMLSGVPDAVFDLTDLDVLKLELIPEAKIPAKISQMTNLQELHLCHCPAKVEQTAFSFLRDHLRCLHVKFTDVAEIPAWVYLLKNLRELYLIGNLNSENNKMIGLESLRELRHLKILHVKSNLTKVPSNITDVAPHLTKLVIHNDGTKLLVLNSLKKMMNVAELELQNCELERIPHAIFSLSNLQELDLKSNNIRTIEEIISFQHLKRLTCLKLWHNKIVAIPPSITHVKNLESLYFSNNKLESLPTAVFSLQKLRCLDVSYNNISTIPIEIGLLQNLQHLHITGNKVDILPKQLFKCVKLRTLNLGQNCIASLPEKISQLTQLTQLELKGNCLDRLPAQLGQCRMLKKSGLVVEDQLFDTLPLEVKEALNQDVNVPFANGI

>ENSMUSP00000052055; Mm Lrrc8e (795aa)

MIPVAEFKQFTEQQPAFKVLKPWWDVLAEYLTVAMLMIGVFGCTLQVTQDKIICLPSHESRENISGAPCQQLLPQGISEQMGGLRELSGLKNNLDLQQYSFINQLCYETALHWYAKYFPYLVVIHTLIFMVCTSFWFKFPGTSSKIEHFISILGKCFDSPWTTRALSEVSGENHKGPASGRAMVTTVTTTGAGSGKVGEGEKEKVLIEPEKVVSEPPVVTLLDKKEGEQAKALFEKVKKFRVHVEEGDILYSMYIRQTVLKVCKFFAILVYNLIYVEKISFLVACRVETSEITGYASFCCNHTKAHLFSKLAFCYISFVCVYGITCLYTLYWLFHRPLKEYSFRSVREETGMNDIPDVKNDFAFMLHLIDQYDSLYSKRFAVFLSEVSESRLKQLNLNHEWTPEKLRQKLQRNMRGRLELSLCMLPGLPDTVFELSEVEALRLEAICDISFPPGLSQLVNLQELSLLHSPARLPFSSQIFLRDRLKVICVKFEELREVPLWVFGLRGLEELHLEGLFPPEMARGATLESLRELKQLKVLSLRSNAGKVPASVTDVAGHLQRLSLHNDGARLLALNSLKKLAVLRELELVACGLERIPHAIFSLGALQELDLKDNHLRSIEEILSFQHCRKLVTLRLWHNQIAYVPEHVRKLRSLEQLYLSHNKLETLPTQLGQCFGLRLLDLSHNGLRSLPPELGLLQSLQHLALSYNALESLPDELFFCHKLRTLLLGYNHLTQLSPDVAALQALSRLELKGNRLETLPEELGDCKGLKKSGLLVEDTLYEGLPAEVREKMEEE

>ENSMUSP00000084507; Mm Lgi1 (557aa)

MESESSRRMGNACIPLKRIAYFLCLFSVVLLTEGKKPAKPKCPAVCTCSKDNALCENARSIPRTVPPDVISLSFVRSGFTEISEGSFLFTPSLQLLLFTSNSFDVISDDAFIGLPHLEYLFIENNNIKSISRHTFRGLKSLIHLSLANNNLQTLPKDIFKGLDSLTNVDLRGNAFNCDCKLKWLVEWLGHTNATVEDIYCEGPPEYKKRKINSLSPKDFDCIITEFAKSQDLPYQSLSIDTFSYLNDEYVVIAQPFTGKCIFLEWDHVEKTFRNYDNITGTSTVVCKPIVIDTQLYVIVAQLFGGSHIYKRDGFANKFIKIQDIEVLKIRKPNDIETFKIEDNWYFVVADSSKAGFTTIYKWNGNGFYSHQSLHAWYRDTDVEYLEIARPPLALRTPHLILSSSSQRPVIYQWSKATQLFTNQTDIPNMEDVYAVKHFSVKGDVYICLTRFIGDSKVMKWGGSSFQDIQRMPSRGSMVFQPLQINNYQYAILGSDYSFTQVYNWDAEKAKFVKFQELNVQAPRSFTHVSINKRNFLFASSFKGNTQIYKHVIVDLSA

>ENSMUSP00000040436; Mm Lgi2 (550aa)

MALWRGGGALGLLLLSAACLIPPSAQVRRLARCPATCSCTKESIICVGSSWVPRIVPGDISSLSLVNGTFLEIKDRMFSHLPSLQLLLLNSNSFTVIRDDAFAGLFHLEYLFIEGNKIETISRNAFRGLRDLTHLDLRGNKFECDCKAKWLYLWLKMTNSTVSDVLCIGPPEYQEKKLNEVTSFDYECTTTGPQTDEAKQRGWQLELSLGFCELIFVFQHPLSDFVVHQTLPYQSVSVDTFNSKNDVYVAIAQPSMENCMVLEWDHIEMNFRSYDNITGQSIVGCKAILIDDQVFVVVAQLFGGSHIYKYDESWTKFVKFQDIEVSRISKPNDIELFEIDDETFFIIADSSKAGLSTVYKWNSKGFYSYQSLHEWFRDTDAEFVDIDGKSHLILSSRSQVPIILQWNKSSKKFVPHGDIPNMEDVLAVKSFRMQNTLYLSLTRFIGDSRVMRWNSKQFVEVQALPSRGAMTLQPFSFKDNHYLALGSDYTFSQIYQWDKEKQQFKKFKEIYVQAPRSFTAVSTDRRDFFFASSFKGKTKIFEHIIVDLSL

>ENSMUSP00000046705; Mm Lgi3 (548aa)

MAGLRARRGPGRRLLVLSTLGFCLMLQVSAKRPPKTPPCPPSCSCTRDTAFCVDSKSVPKNLPSEVISLTLVNAAFSEIQDGAFSHLPLLQFLLLNSNKFTLIGDNAFIGLSHLQYLFIENNDIWALSKFTFRGLKSLTHLSLANNNLQTLPRDIFRPLDILSDLDLRGNALNCDCKVKWLVEWLAHTNTTVAPIYCASPPRFQEHKVQDLPLREFDCITTDFVLYQTLSFPAVSAEPFLYSSDLYLALAQPGASACTILKWDYVERQLRDYDRIPAPSAVHCKPMVVDGQLYVVVAQLFGGSYIYHWDPNTTRFTKLQDIDPQRVRKPNDLEAFRIDGDWFFAVADSSKAGATSLYRWHQNGFYSHQALHAWHRDTDLEFVDGEGKPRLIVSSSSQAPVIYQWSRSQKQFVAQGEVTQVPDAQAVKHFRAGRDSYLCLSRYIGDSKILRWEGTRFSEVQALPSRGSLALQPFLVGGHRYLALGSDFSFTQIYQWDEGRQKFVRFQELAVQAPRAFCYMPAGDAQLLLAPSFKGQTLVYRHVVVDLSA

>ENSMUSP00000041579; Mm Lgi4 (537aa)

MGGAGILLFLLAWAGAGVAWSPPKGKCPPHCSCSKENTLCEGSPELPESFSTTLLSLSLVRMGVSRLKAGSFLKMPSLHLLLFTSNTFSVIEGDAFIGLSYLQYLFIEDNKIGSISKNALRGLRSLTHLSLANNHLEALPRFLFRGLETLTHVDLRGNPFQCDCRVLWLLQWMPTVNASVGTGACAGPPAVAQIQLNHLDPKKFKCRATELSWLQTVGESALSVESFSYQGEPHMVLAQPFAGRCLILVWDYSLQRFRPEEELSAPSVVSCKPLVLGPRLFILAARLWGGSQLWSRSSPDLRLTPVQVLAPQRLLRPNDAELLWLDGQPCFVVADASKAGSTTLLCRDGPGFYPRQSLHAWHRDTDAEALELDGRPHLLLASASQRPVLFHWVGGRFERRTDIPEAEDVYATKHFQAGGDVFLCLTRYIGDSMVMRWDGSMFRLLQQLPSRGSHVFQPLLIARDQLAILGSDFAFSQVFRFESDKGILEPLQELGPPALVAPRAFAQVTVAGRRFLFAACFKGPTQIYQHHELDLSA

>ENSMUSP00000045162; Mm Slitl2=Vasn (673aa)

MHSRSCLPPLLLLLLVLLGSGVQGCPSGCQCNQPQTVFCTARQGTTVPRDVPPDTVGLYIFENGITTLDVGCFAGLPGLQLLDLSQNQITSLPGGIFQPLVNLSNLDLTANKLHEISNETFRGLRRLERLYLGKNRIRHIQPGAFDALDRLLELKLPDNELRVLPPLHLPRLLLLDLSHNSIPALEAGILDTANVEALRLAGLGLRQLDEGLFGRLLNLHDLDVSDNQLEHMPSVIQGLRGLTRLRLAGNTRIAQIRPEDLAGLTALQELDVSNLSLQALPSDLSSLFPRLRLLAAARNPFNCLCPLSWFGPWVRENHVVLASPEETRCHFPPKNAGRLLLDLDYADFGCPVTTTTATVPTIRSTIREPTLSTSSQAPTWPSLTEPTTQASTVLSTAPPTMRPAPQPQDCPASICLNGGSCRLGARHHWECLCPEGFIGLYCESPVEQGMKPSSIPDTPRPPPLLPLSIEPVSPTSLRVKLQRYLQGNTVQLRSLRLTYRNLSGPDKRLVTLRLPASLAEYTVTQLRPNATYSICVTPLGAGRTPEGEEACGEANTSQAVRSNHAPVTQAREGNLPLLIAPALAAVLLAVLAAAGAAYCVRRARATSTAQDKGQVGPGTGPLELEGVKAPLEPGSKATEGGGEALSGGPECEVPLMGYPGPSLQGVLPAKHYI

>ENSP00000266058; Hs SLIT1 (1534aa)

MALTPGWGSSAGPVRPELWLLLWAAAWRLGASACPALCTCTGTTVDCHGTGLQAIPKNIPRNTERLELNGNNITRIHKNDFAGLKQLRVLQLMENQIGAVERGAFDDMKELERLRLNRNQLHMLPELLFQNNQALSRLDLSENAIQAIPRKAFRGATDLKNLQLDKNQISCIEEGAFRALRGLEVLTLNNNNITTIPVSSFNHMPKLRTFRLHSNHLFCDCHLAWLSQWLRQRPTIGLFTQCSGPASLRGLNVAEVQKSEFSCSGQGEAGRVPTCTLSSGSCPAMCTCSNGIVDCRGKGLTAIPANLPETMTEIRLELNGIKSIPPGAFSPYRKLRRIDLSNNQIAEIAPDAFQGLRSLNSLVLYGNKITDLPRGVFGGLYTLQLLLLNANKINCIRPDAFQDLQNLSLLSLYDNKIQSLAKGTFTSLRAIQTLHLAQNPFICDCNLKWLADFLRTNPIETSGARCASPRRLANKRIGQIKSKKFRCSAKEQYFIPGTEDYQLNSECNSDVVCPHKCRCEANVVECSSLKLTKIPERIPQSTAELRLNNNEISILEATGMFKKLTHLKKINLSNNKVSEIEDGAFEGAASVSELHLTANQLESIRSGMFRGLDGLRTLMLRNNRISCIHNDSFTGLRNVRLLSLYDNQITTVSPGAFDTLQSLSTLNLLANPFNCNCQLAWLGGWLRKRKIVTGNPRCQNPDFLRQIPLQDVAFPDFRCEEGQEEGGCLPRPQCPQECACLDTVVRCSNKHLRALPKGIPKNVTELYLDGNQFTLVPGQLSTFKYLQLVDLSNNKISSLSNSSFTNMSQLTTLILSYNALQCIPPLAFQGLRSLRLLSLHGNDISTLQEGIFADVTSLSHLAIGANPLYCDCHLRWLSSWVKTGYKEPGIARCAGPQDMEGKLLLTTPAKKFECQGPPTLAVQAKCDLCLSSPCQNQGTCHNDPLEVYRCACPSGYKGRDCEVSLDSCSSGPCENGGTCHAQEGEDAPFTCSCPTGFEGPTCGVNTDDCVDHACANGGVCVDGVGNYTCQCPLQYEGKACEQLVDLCSPDLNPCQHEAQCVGTPDGPRCECMPGYAGDNCSENQDDCRDHRCQNGAQCMDEVNSYSCLCAEGYSGQLCEIPPHLPAPKSPCEGTECQNGANCVDQGNRPVCQCLPGFGGPECEKLLSVNFVDRDTYLQFTDLQNWPRANITLQVSTAEDNGILLYNGDNDHIAVELYQGHVRVSYDPGSYPSSAIYSAETINDGQFHTVELVAFDQMVNLSIDGGSPMTMDNFGKHYTLNSEAPLYVGGMPVDVNSAAFRLWQILNGTGFHGCIRNLYINNELQDFTKTQMKPGVVPGCEPCRKLYCLHGICQPNATPGPMCHCEAGWVGLHCDQPADGPCHGHKCVHGQCVPLDALSYSCQCQDGYSGALCNQAGALAEPCRGLQCLHGHCQASGTKGAHCVCDPGFSGELCEQESECRGDPVRDFHQVQRGYAICQTTRPLSWVECRGSCPGQGCCQGLRLKRRKFTFECSDGTSFAEEVEKPTKCGCALCA

>ENSP00000273739; Hs SLIT2 (1529aa)

MRGVGWQMLSLSLGLVLAILNKVAPQACPAQCSCSGSTVDCHGLALRSVPRNIPRNTERLDLNGNNITRITKTDFAGLRHLRVLQLMENKISTIERGAFQDLKELERLRLNRNHLQLFPELLFLGTAKLYRLDLSENQIQAIPRKAFRGAVDIKNLQLDYNQISCIEDGAFRALRDLEVLTLNNNNITRLSVASFNHMPKLRTFRLHSNNLYCDCHLAWLSDWLRQRPRVGLYTQCMGPSHLRGHNVAEVQKREFVCSGHQSFMAPSCSVLHCPAACTCSNNIVDCRGKGLTEIPTNLPETITEIRLEQNTIKVIPPGAFSPYKKLRRIDLSNNQISELAPDAFQGLRSLNSLVLYGNKITELPKSLFEGLFSLQLLLLNANKINCLRVDAFQDLHNLNLLSLYDNKLQTIAKGTFSPLRAIQTMHLAQNPFICDCHLKWLADYLHTNPIETSGARCTSPRRLANKRIGQIKSKKFRCSAKEQYFIPGTEDYRSKLSGDCFADLACPEKCRCEGTTVDCSNQKLNKIPEHIPQYTAELRLNNNEFTVLEATGIFKKLPQLRKINFSNNKITDIEEGAFEGASGVNEILLTSNRLENVQHKMFKGLESLKTLMLRSNRITCVGNDSFIGLSSVRLLSLYDNQITTVAPGAFDTLHSLSTLNLLANPFNCNCYLAWLGEWLRKKRIVTGNPRCQKPYFLKEIPIQDVAIQDFTCDDGNDDNSCSPLSRCPTECTCLDTVVRCSNKGLKVLPKGIPRDVTELYLDGNQFTLVPKELSNYKHLTLIDLSNNRISTLSNQSFSNMTQLLTLILSYNRLRCIPPRTFDGLKSLRLLSLHGNDISVVPEGAFNDLSALSHLAIGANPLYCDCNMQWLSDWVKSEYKEPGIARCAGPGEMADKLLLTTPSKKFTCQGPVDVNILAKCNPCLSNPCKNDGTCNSDPVDFYRCTCPYGFKGQDCDVPIHACISNPCKHGGTCHLKEGEEDGFWCICADGFEGENCEVNVDDCEDNDCENNSTCVDGINNYTCLCPPEYTGELCEEKLDFCAQDLNPCQHDSKCILTPKGFKCDCTPGYVGEHCDIDFDDCQDNKCKNGAHCTDAVNGYTCICPEGYSGLFCEFSPPMVLPRTSPCDNFDCQNGAQCIVRINEPICQCLPGYQGEKCEKLVSVNFINKESYLQIPSAKVRPQTNITLQIATDEDSGILLYKGDKDHIAVELYRGRVRASYDTGSHPASAIYSVETINDGNFHIVELLALDQSLSLSVDGGNPKIITNLSKQSTLNFDSPLYVGGMPGKSNVASLRQAPGQNGTSFHGCIRNLYINSELQDFQKVPMQTGILPGCEPCHKKVCAHGTCQPSSQAGFTCECQEGWMGPLCDQRTNDPCLGNKCVHGTCLPINAFSYSCKCLEGHGGVLCDEEEDLFNPCQAIKCKHGKCRLSGLGQPYCECSSGYTGDSCDREISCRGERIRDYYQKQQGYAACQTTKKVSRLECRGGCAGGQCCGPLRSKRRKYSFECTDGSSFVDEVEKVVKCGCTRCVS

>ENSP00000332164; Hs SLIT3 (1523aa)

MAPGWAGVGAAVRARLALALALASVLSGPPAVACPTKCTCSAASVDCHGLGLRAVPRGIPRNAERLDLDRNNITRITKMDFAGLKNLRVLHLEDNQVSVIERGAFQDLKQLERLRLNKNKLQVLPELLFQSTPKLTRLDLSENQIQGIPRKAFRGITDVKNLQLDNNHISCIEDGAFRALRDLEILTLNNNNISRILVTSFNHMPKIRTLRLHSNHLYCDCHLAWLSDWLRQRRTVGQFTLCMAPVHLRGFNVADVQKKEYVCPAPHSEPPSCNANSISCPSPCTCSNNIVDCRGKGLMEIPANLPEGIVEIRLEQNSIKAIPAGAFTQYKKLKRIDISKNQISDIAPDAFQGLKSLTSLVLYGNKITEIVKGLFDGLVSLQLLLLNANKINCLRVNTFQDLQNLNLLSLYDNKLQTISKGLFAPLQSIQTLHLAQNPFVCDCHLKWLADYLQDNPIETSGARCSSPRRLANKRISQIKSKKFRCSGSEDYRSRFSSECFMDLVCPEKCRCEGTIVDCSNQKLVRIPSHLPEYVTDLRLNDNEVSVLEATGIFKKLPNLRKINLSNNKIKEVREGAFDGAASVQELMLTGNQLETVHGRVFRGLSGLKTLMLRSNLIGCVSNDTFAGLSSVRLLSLYDNRITTITPGAFTTLVSLSTINLLSNPFNCNCHLAWLGKWLRKRRIVSGNPRCQKPFFLKEIPIQDVAIQDFTCDGNEESSCQLSPRCPEQCTCMETVVRCSNKGLRALPRGMPKDVTELYLEGNHLTAVPRELSALRHLTLIDLSNNSISMLTNYTFSNMSHLSTLILSYNRLRCIPVHAFNGLRSLRVLTLHGNDISSVPEGSFNDLTSLSHLALGTNPLHCDCSLRWLSEWVKAGYKEPGIARCSSPEPMADRLLLTTPTHRFQCKGPVDINIVAKCNACLSSPCKNNGTCTQDPVELYRCACPYSYKGKDCTVPINTCIQNPCQHGGTCHLSDSHKDGFSCSCPLGFEGQRCEINPDDCEDNDCENNATCVDGINNYVCICPPNYTGELCDEVIDHCVPELNLCQHEAKCIPLDKGFSCECVPGYSGKLCETDNDDCVAHKCRHGAQCVDTINGYTCTCPQGFSGPFCEHPPPMVLLQTSPCDQYECQNGAQCIVVQQEPTCRCPPGFAGPRCEKLITVNFVGKDSYVELASAKVRPQANISLQVATDKDNGILLYKGDNDPLALELYQGHVRLVYDSLSSPPTTVYSVETVNDGQFHSVELVTLNQTLNLVVDKGTPKSLGKLQKQPAVGINSPLYLGGIPTSTGLSALRQGTDRPLGGFHGCIHEVRINNELQDFKALPPQSLGVSPGCKSCTVCKHGLCRSVEKDSVVCECRPGWTGPLCDQEARDPCLGHRCHHGKCVATGTSYMCKCAEGYGGDLCDNKNDSANACSAFKCHHGQCHISDQGEPYCLCQPGFSGEHCQQENPCLGQVVREVIRRQKGYASCATASKVPIMECRGGCGPQCCQPTRSKRRKYVFQCTDGSSFVEEVERHLECGCLACS

>ENSP00000374508; Hs LGR4 (951aa)

MPGPLGLLCFLALGLLGSAGPSGAAPPLCAAPCSCDGDRRVDCSGKGLTAVPEGLSAFTQALDISMNNITQLPEDAFKNFPFLEELQLAGNDLSFIHPKALSGLKELKVLTLQNNQLKTVPSEAIRGLSALQSLRLDANHITSVPEDSFEGLVQLRHLWLDDNSLTEVPVHPLSNLPTLQALTLALNKISSIPDFAFTNLSSLVVLHLHNNKIRGLSQHCFDGLDNLETLDLSYNNLGEFPQAIKARPSLKELGFHSNSISVIPDGAFDGNPLLRTIHLYDNPLSFVGNSASHNLSDLHSLVIRGASMVQQFPNLTGTVHLESLTLTGTKISSIPNNLCQEQKMLRTLDLSYNNIRDLPSFNGCHALEEISLQRNQIYQIKEGTFQGLISLRILDLSRNLIHEIHSRAFATLGPITNLDVSFNELTSFPTEGPNGLNQLKLVGNFKLKEALAAKDFVNLRSLSVPYAYQCCAFWGCDSYANLNTEDNSLQDHSVAQEKGTADAANVTSTLENEEHSQIIIHCTPSTGAFKPCEYLLGSWMIRLTVWFIFLVALFFNLLVILTTFASCTSLPSSKLFIGLISVSNLFMGIYTGILTFLDAVSWGRFAEFGIWWETGSGCKVAGFLAVFSSESAIFLLMLATVERSLSAKDIMKNGKSNHLKQFRVAALSAFLGATVAGCFPLFHRGEYSASPLCLPFPTGETPSLGFTVTLVLLNSLAFLLMAVIYTKLYCNLEKEDLSENSQSSMIKHVAWLIFTNCIFFCPVAFFSFAPLITAISISPEIMKSVTLIFFPLPACLNPVLYVFFNPKFKEDWKLLKRRVTKKSGSVSVSISSQGGCLEQDFYYDCGMYSHLQGNLTVCDCCESFLLTKPVSCKHLIKSHSCPALAVASCQRPEGYWSDCGTQSAHSDYADEEDSFVSDSSDQVQACGRACFYQSRGFPLVRYAYNLPRVKD

>ENSP00000266674; Hs LGR5 (907aa)

MDTSRLGVLLSLPVLLQLATGGSSPRSGVLLRGCPTHCHCEPDGRMLLRVDCSDLGLSELPSNLSVFTSYLDLSMNNISQLLPNPLPSLRFLEELRLAGNALTYIPKGAFTGLYSLKVLMLQNNQLRHVPTEALQNLRSLQSLRLDANHISYVPPSCFSGLHSLRHLWLDDNALTEIPVQAFRSLSALQAMTLALNKIHHIPDYAFGNLSSLVVLHLHNNRIHSLGKKCFDGLHSLETLDLNYNNLDEFPTAIRTLSNLKELGFHSNNIRSIPEKAFVGNPSLITIHFYDNPIQFVGRSAFQHLPELRTLTLNGASQITEFPDLTGTANLESLTLTGAQISSLPQTVCNQLPNLQVLDLSYNLLEDLPSFSVCQKLQKIDLRHNEIYEIKVDTFQQLLSLRSLNLAWNKIAIIHPNAFSTLPSLIKLDLSSNLLSSFPITGLHGLTHLKLTGNHALQSLISSENFPELKVIEMPYAYQCCAFGVCENAYKISNQWNKGDNSSMDDLHKKDAGMFQAQDERDLEDFLLDFEEDLKALHSVQCSPSPGPFKPCEHLLDGWLIRIGVWTIAVLALTCNALVTSTVFRSPLYISPIKLLIGVIAAVNMLTGVSSAVLAGVDAFTFGSFARHGAWWENGVGCHVIGFLSIFASESSVFLLTLAALERGFSVKYSAKFETKAPFSSLKVIILLCALLALTMAAVPLLGGSKYGASPLCLPLPFGEPSTMGYMVALILLNSLCFLMMTIAYTKLYCNLDKGDLENIWDCSMVKHIALLLFTNCILNCPVAFLSFSSLINLTFISPEVIKFILLVVVPLPACLNPLLYILFNPHFKEDLVSLRKQTYVWTRSKHPSLMSINSDDVEKQSCDSTQALVTFTSSSITYDLPPSSVPSPAYPVTESCHLSSVAFVPCL

>ENSP00000354333; Hs LGR6 (967aa)

MPSPPGLRALWLCAALCASRRAGGAPQPGPGPTACPAPCHCQEDGIMLSADCSELGLSAVPGDLDPLTAYLDLSMNNLTELQPGLFHHLRFLEELRLSGNHLSHIPGQAFSGLYSLKILMLQNNQLGGIPAEALWELPSLQSLRLDANLISLVPERSFEGLSSLRHLWLDDNALTEIPVRALNNLPALQAMTLALNRISHIPDYAFQNLTSLVVLHLHNNRIQHLGTHSFEGLHNLETLDLNYNKLQEFPVAIRTLGRLQELGFHNNNIKAIPEKAFMGNPLLQTIHFYDNPIQFVGRSAFQYLPKLHTLSLNGAMDIQEFPDLKGTTSLEILTLTRAGIRLLPSGMCQQLPRLRVLELSHNQIEELPSLHRCQKLEEIGLQHNRIWEIGADTFSQLSSLQALDLSWNAIRSIHPEAFSTLHSLVKLDLTDNQLTTLPLAGLGGLMHLKLKGNLALSQAFSKDSFPKLRILEVPYAYQCCPYGMCASFFKASGQWEAEDLHLDDEESSKRPLGLLARQAENHYDQDLDELQLEMEDSKPHPSVQCSPTPGPFKPCEYLFESWGIRLAVWAIVLLSVLCNGLVLLTVFAGGPVPLPPVKFVVGAIAGANTLTGISCGLLASVDALTFGQFSEYGARWETGLGCRATGFLAVLGSEASVLLLTLAAVQCSVSVSCVRAYGKSPSLGSVRAGVLGCLALAGLAAALPLASVGEYGASPLCLPYAPPEGQPAALGFTVALVMMNSFCFLVVAGAYIKLYCDLPRGDFEAVWDCAMVRHVAWLIFADGLLYCPVAFLSFASMLGLFPVTPEAVKSVLLVVLPLPACLNPLLYLLFNPHFRDDLRRLRPRAGDSGPLAYAAAGELEKSSCDSTQALVAFSDVDLILEASEAGRPPGLETYGFPSVTLISCQQPGAPRLEGSHCVEPEGNHFGNPQPSMDGELLLRAEGSTPAGGGLSGGGGFQPSGLAFASHV

>ENSP00000306780; Hs FSHR (695aa)

MALLLVSLLAFLSLGSGCHHRICHCSNRVFLCQESKVTEIPSDLPRNAIELRFVLTKLRVIQKGAFSGFGDLEKIEISQNDVLEVIEADVFSNLPKLHEIRIEKANNLLYINPEAFQNLPNLQYLLISNTGIKHLPDVHKIHSLQKVLLDIQDNINIHTIERNSFVGLSFESVILWLNKNGIQEIHNCAFNGTQLDELNLSDNNNLEELPNDVFHGASGPVILDISRTRIHSLPSYGLENLKKLRARSTYNLKKLPTLEKLVALMEASLTYPSHCCAFANWRRQISELHPICNKSILRQEVDYMTQARGQRSSLAEDNESSYSRGFDMTYTEFDYDLCNEVVDVTCSPKPDAFNPCEDIMGYNILRVLIWFISILAITGNIIVLVILTTSQYKLTVPRFLMCNLAFADLCIGIYLLLIASVDIHTKSQYHNYAIDWQTGAGCDAAGFFTVFASELSVYTLTAITLERWHTITHAMQLDCKVQLRHAASVMVMGWIFAFAAALFPIFGISSYMKVSICLPMDIDSPLSQLYVMSLLVLNVLAFVVICGCYIHIYLTVRNPNIVSSSSDTRIAKRMAMLIFTDFLCMAPISFFAISASLKVPLITVSKAKILLVLFHPINSCANPFLYAIFTKNFRRDFFILLSKCGCYEMQAQIYRTETSSTVHNTHPRNGHCSSAPRVTSGSTYILVPLSHLAQN

>ENSP00000294954; Hs LHCGR (699aa)

MKQRFSALQLLKLLLLLQPPLPRALREALCPEPCNCVPDGALRCPGPTAGLTRLSLAYLPVKVIPSQAFRGLNEVIKIEISQIDSLERIEANAFDNLLNLSEILIQNTKNLRYIEPGAFINLPRLKYLSICNTGIRKFPDVTKVFSSESNFILEICDNLHITTIPGNAFQGMNNESVTLKLYGNGFEEVQSHAFNGTTLTSLELKENVHLEKMHNGAFRGATGPKTLDISSTKLQALPSYGLESIQRLIATSSYSLKKLPSRETFVNLLEATLTYPSHCCAFRNLPTKEQNFSHSISENFSKQCESTVRKVNNKTLYSSMLAESELSGWDYEYGFCLPKTPRCAPEPDAFNPCEDIMGYDFLRVLIWLINILAIMGNMTVLFVLLTSRYKLTVPRFLMCNLSFADFCMGLYLLLIASVDSQTKGQYYNHAIDWQTGSGCSTAGFFTVFASELSVYTLTVITLERWHTITYAIHLDQKLRLRHAILIMLGGWLFSSLIAMLPLVGVSNYMKVSICFPMDVETTLSQVYILTILILNVVAFFIICACYIKIYFAVRNPELMATNKDTKIAKKMAILIFTDFTCMAPISFFAISAAFKVPLITVTNSKVLLVLFYPINSCANPFLYAIFTKTFQRDFFLLLSKFGCCKRRAELYRRKDFSAYTSNCKNGFTGSNKPSQSTLKLSTLHCQGTALLDKTRYTEC

>ENSP00000298171; Hs TSHR (764aa)

MRPADLLQLVLLLDLPRDLGGMGCSSPPCECHQEEDFRVTCKDIQRIPSLPPSTQTLKLIETHLRTIPSHAFSNLPNISRIYVSIDVTLQQLESHSFYNLSKVTHIEIRNTRNLTYIDPDALKELPLLKFLGIFNTGLKMFPDLTKVYSTDIFFILEITDNPYMTSIPVNAFQGLCNETLTLKLYNNGFTSVQGYAFNGTKLDAVYLNKNKYLTVIDKDAFGGVYSGPSLLDVSQTSVTALPSKGLEHLKELIARNTWTLKKLPLSLSFLHLTRADLSYPSHCCAFKNQKKIRGILESLMCNESSMQSLRQRKSVNALNSPLHQEYEENLGDSIVGYKEKSKFQDTHNNAHYYVFFEEQEDEIIGFGQELKNPQEETLQAFDSHYDYTICGDSEDMVCTPKSDEFNPCEDIMGYKFLRIVVWFVSLLALLGNVFVLLILLTSHYKLNVPRFLMCNLAFADFCMGMYLLLIASVDLYTHSEYYNHAIDWQTGPGCNTAGFFTVFASELSVYTLTVITLERWYAITFAMRLDRKIRLRHACAIMVGGWVCCFLLALLPLVGISSYAKVSICLPMDTETPLALAYIVFVLTLNIVAFVIVCCCYVKIYITVRNPQYNPGDKDTKIAKRMAVLIFTDFICMAPISFYALSAILNKPLITVSNSKILLVLFYPLNSCANPFLYAIFTKAFQRDVFILLSKFGICKRQAQAYRGQRVPPKNSTDIQVQKVTHEMRQGLHNMEDVYELIENSHLTPKKQGQISEEYMQTVL

>NP_067647; Hs LGR7=RXFP1 (757aa)

MTSGSVFFYILIFGKYFSHGGGQDVKCSLGYFPCGNITKCLPQLLHCNGVDDCGNQADEDNCGDNNGWSLQFDKYFASYYKMTSQYPFEAETPECLVGSVPVQCLCQGLELDCDETNLRAVPSVSSNVTAMSLQWNLIRKLPPDCFKNYHDLQKLYLQNNKITSISIYAFRGLNSLTKLYLSHNRITFLKPGVFEDLHRLEWLIIEDNHLSRISPPTFYGLNSLILLVLMNNVLTRLPDKPLCQHMPRLHWLDLEGNHIHNLRNLTFISCSNLTVLVMRKNKINHLNENTFAPLQKLDELDLGSNKIENLPPLIFKDLKELSQLNLSYNPIQKIQANQFDYLVKLKSLSLEGIEISNIQQRMFRPLMNLSHIYFKKFQYCGYAPHVRSCKPNTDGISSLENLLASIIQRVFVWVVSAVTCFGNIFVICMRPYIRSENKLYAMSIISLCCADCLMGIYLFVIGGFDLKFRGEYNKHAQLWMESTHCQLVGSLAILSTEVSVLLLTFLTLEKYICIVYPFRCVRPGKCRTITVLILIWITGFIVAFIPLSNKEFFKNYYGTNGVCFPLHSEDTESIGAQIYSVAIFLGINLAAFIIIVFSYGSMFYSVHQSAITATEIRNQVKKEMILAKRFFFIVFTDALCWIPIFVVKFLSLLQVEIPGTITSWVVIFILPINSALNPILYTLTTRPFKEMIHRFWYNYRQRKSMDSKGQKTYAPSFIWVEMWPLQEMPPELMKPDLFTYPCEMSLISQSTRLNSYS

>ENSP00000298386; Hs RXFP2=LGR8 (754aa)

MIVFLVFKHLFSLRLITMFFLLHFIVLINVKDFALTQGSMITPSCQKGYFPCGNLTKCLPRAFHCDGKDDCGNGADEENCGDTSGWATIFGTVHGNANSVALTQECFLKQYPQCCDCKETELECVNGDLKSVPMISNNVTLLSLKKNKIHSLPDKVFIKYTKLKKIFLQHNCIRHISRKAFFGLCNLQILYLNHNCITTLRPGIFKDLHQLTWLILDDNPITRISQRLFTGLNSLFFLSMVNNYLEALPKQMCAQMPQLNWVDLEGNRIKYLTNSTFLSCDSLTVLFLPRNQIGFVPEKTFSSLKNLGELDLSSNTITELSPHLFKDLKLLQKLNLSSNPLMYLHKNQFESLKQLQSLDLERIEIPNINTRMFQPMKNLSHIYFKNFRYCSYAPHVRICMPLTDGISSFEDLLANNILRIFVWVIAFITCFGNLFVIGMRSFIKAENTTHAMSIKILCCADCLMGVYLFFVGIFDIKYRGQYQKYALLWMESVQCRLMGFLAMLSTEVSVLLLTYLTLEKFLVIVFPFSNIRPGKRQTSVILICIWMAGFLIAVIPFWNKDYFGNFYGKNGVCFPLYYDQTEDIGSKGYSLGIFLGVNLLAFLIIVFSYITMFCSIQKTALQTTEVRNCFGREVAVANRFFFIVFSDAICWIPVFVVKILSLFRVEIPDTMTSWIVIFFLPVNSALNPILYTLTTNFFKDKLKQLLHKHQRKSIFKIKKKSLSTSIVWIEDSSSLKLGVLNKITLGDSIMKPVS

>ENSP00000262304; Hs PKD1 (4303aa)

MPPAAPARLALALGLGLWLGALAGGPGRGCGPCEPPCLCGPAPGAACRVNCSGRGLRTLGPALRIPADATALDVSHNLLRALDVGLLANLSALAELDISNNKISTLEEGIFANLFNLSEINLSGNPFECDCGLAWLPRWAEEQQVRVVQPEAATCAGPGSLAGQPLLGIPLLDSGCGEEYVACLPDNSSGTVAAVSFSAAHEGLLQPEACSAFCFSTGQGLAALSEQGWCLCGAAQPSSASFACLSLCSGPPPPPAPTCRGPTLLQHVFPASPGATLVGPHGPLASGQLAAFHIAAPLPVTATRWDFGDGSAEVDAAGPAASHRYVLPGRYHVTAVLALGAGSALLGTDVQVEAAPAALELVCPSSVQSDESLDLSIQNRGGSGLEAAYSIVALGEEPARAVHPLCPSDTEIFPGNGHCYRLVVEKAAWLQAQEQCQAWAGAALAMVDSPAVQRFLVSRVTRSLDVWIGFSTVQGVEVGPAPQGEAFSLESCQNWLPGEPHPATAEHCVRLGPTGWCNTDLCSAPHSYVCELQPGGPVQDAENLLVGAPSGDLQGPLTPLAQQDGLSAPHEPVEVMVFPGLRLSREAFLTTAEFGTQELRRPAQLRLQVYRLLSTAGTPENGSEPESRSPDNRTQLAPACMPGGRWCPGANICLPLDASCHPQACANGCTSGPGLPGAPYALWREFLFSVPAGPPAQYSVTLHGQDVLMLPGDLVGLQHDAGPGALLHCSPAPGHPGPRAPYLSANASSWLPHLPAQLEGTWACPACALRLLAATEQLTVLLGLRPNPGLRLPGRYEVRAEVGNGVSRHNLSCSFDVVSPVAGLRVIYPAPRDGRLYVPTNGSALVLQVDSGANATATARWPGGSVSARFENVCPALVATFVPGCPWETNDTLFSVVALPWLSEGEHVVDVVVENSASRANLSLRVTAEEPICGLRATPSPEARVLQGVLVRYSPVVEAGSDMVFRWTINDKQSLTFQNVVFNVIYQSAAVFKLSLTASNHVSNVTVNYNVTVERMNRMQGLQVSTVPAVLSPNATLALTAGVLVDSAVEVAFLWTFGDGEQALHQFQPPYNESFPVPDPSVAQVLVEHNVMHTYAAPGEYLLTVLASNAFENLTQQVPVSVRASLPSVAVGVSDGVLVAGRPVTFYPHPLPSPGGVLYTWDFGDGSPVLTQSQPAANHTYASRGTYHVRLEVNNTVSGAAAQADVRVFEELRGLSVDMSLAVEQGAPVVVSAAVQTGDNITWTFDMGDGTVLSGPEATVEHVYLRAQNCTVTVGAASPAGHLARSLHVLVFVLEVLRVEPAACIPTQPDARLTAYVTGNPAHYLFDWTFGDGSSNTTVRGCPTVTHNFTRSGTFPLALVLSSRVNRAHYFTSICVEPEVGNVTLQPERQFVQLGDEAWLVACAWPPFPYRYTWDFGTEEAAPTRARGPEVTFIYRDPGSYLVTVTASNNISAANDSALVEVQEPVLVTSIKVNGSLGLELQQPYLFSAVGRGRPASYLWDLGDGGWLEGPEVTHAYNSTGDFTVRVAGWNEVSRSEAWLNVTVKRRVRGLVVNASRTVVPLNGSVSFSTSLEAGSDVRYSWVLCDRCTPIPGGPTISYTFRSVGTFNIIVTAENEVGSAQDSIFVYVLQLIEGLQVVGGGRYFPTNHTVQLQAVVRDGTNVSYSWTAWRDRGPALAGSGKGFSLTVLEAGTYHVQLRATNMLGSAWADCTMDFVEPVGWLMVAASPNPAAVNTSVTLSAELAGGSGVVYTWSLEEGLSWETSEPFTTHSFPTPGLHLVTMTAGNPLGSANATVEVDVQVPVSGLSIRASEPGGSFVAAGSSVPFWGQLATGTNVSWCWAVPGGSSKRGPHVTMVFPDAGTFSIRLNASNAVSWVSATYNLTAEEPIVGLVLWASSKVVAPGQLVHFQILLAAGSAVTFRLQVGGANPEVLPGPRFSHSFPRVGDHVVSVRGKNHVSWAQAQVRIVVLEAVSGLQVPNCCEPGIATGTERNFTARVQRGSRVAYAWYFSLQKVQGDSLVILSGRDVTYTPVAAGLLEIQVRAFNALGSENRTLVLEVQDAVQYVALQSGPCFTNRSAQFEAATSPSPRRVAYHWDFGDGSPGQDTDEPRAEHSYLRPGDYRVQVNASNLVSFFVAQATVTVQVLACREPEVDVVLPLQVLMRRSQRNYLEAHVDLRDCVTYQTEYRWEVYRTASCQRPGRPARVALPGVDVSRPRLVLPRLALPVGHYCFVFVVSFGDTPLTQSIQANVTVAPERLVPIIEGGSYRVWSDTRDLVLDGSESYDPNLEDGDQTPLSFHWACVASTQREAGGCALNFGPRGSSTVTIPRERLAAGVEYTFSLTVWKAGRKEEATNQTVLIRSGRVPIVSLECVSCKAQAVYEVSRSSYVYLEGRCLNCSSGSKRGRWAARTFSNKTLVLDETTTSTGSAGMRLVLRRGVLRDGEGYTFTLTVLGRSGEEEGCASIRLSPNRPPLGGSCRLFPLGAVHALTTKVHFECTGWHDAEDAGAPLVYALLLRRCRQGHCEEFCVYKGSLSSYGAVLPPGFRPHFEVGLAVVVQDQLGAAVVALNRSLAITLPEPNGSATGLTVWLHGLTASVLPGLLRQADPQHVIEYSLALVTVLNEYERALDVAAEPKHERQHRAQIRKNITETLVSLRVHTVDDIQQIAAALAQCMGPSRELVCRSCLKQTLHKLEAMMLILQAETTAGTVTPTAIGDSILNITGDLIHLASSDVRAPQPSELGAESPSRMVASQAYNLTSALMRILMRSRVLNEEPLTLAGEEIVAQGKRSDPRSLLCYGGAPGPGCHFSIPEAFSGALANLSDVVQLIFLVDSNPFPFGYISNYTVSTKVASMAFQTQAGAQIPIERLASERAITVKVPNNSDWAARGHRSSANSANSVVVQPQASVGAVVTLDSSNPAAGLHLQLNYTLLDGHYLSEEPEPYLAVYLHSEPRPNEHNCSASRRIRPESLQGADHRPYTFFISPGSRDPAGSYHLNLSSHFRWSALQVSVGLYTSLCQYFSEEDMVWRTEGLLPLEETSPRQAVCLTRHLTAFGASLFVPPSHVRFVFPEPTADVNYIVMLTCAVCLVTYMVMAAILHKLDQLDASRGRAIPFCGQRGRFKYEILVKTGWGRGSGTTAHVGIMLYGVDSRSGHRHLDGDRAFHRNSLDIFRIATPHSLGSVWKIRVWHDNKGLSPAWFLQHVIVRDLQTARSAFFLVNDWLSVETEANGGLVEKEVLAASDAALLRFRRLLVAELQRGFFDKHIWLSIWDRPPRSRFTRIQRATCCVLLICLFLGANAVWYGAVGDSAYSTGHVSRLSPLSVDTVAVGLVSSVVVYPVYLAILFLFRMSRSKVAGSPSPTPAGQQVLDIDSCLDSSVLDSSFLTFSGLHAEQAFVGQMKSDLFLDDSKSLVCWPSGEGTLSWPDLLSDPSIVGSNLRQLARGQAGHGLGPEEDGFSLASPYSPAKSFSASDEDLIQQVLAEGVSSPAPTQDTHMETDLLSSLSSTPGEKTETLALQRLGELGPPSPGLNWEQPQAARLSRTGLVEGLRKRLLPAWCASLAHGLSLLLVAVAVAVSGWVGASFPPGVSVAWLLSSSASFLASFLGWEPLKVLLEALYFSLVAKRLHPDEDDTLVESPAVTPVSARVPRVRPPHGFALFLAKEEARKVKRLHGMLRSLLVYMLFLLVTLLASYGDASCHGHAYRLQSAIKQELHSRAFLAITRSEELWPWMAHVLLPYVHGNQSSPELGPPRLRQVRLQEALYPDPPGPRVHTCSAAGGFSTSDYDVGWESPHNGSGTWAYSAPDLLGAWSWGSCAVYDSGGYVQELGLSLEESRDRLRFLQLHNWLDNRSRAVFLELTRYSPAVGLHAAVTLRLEFPAAGRALAALSVRPFALRRLSAGLSLPLLTSVCLLLFAVHFAVAEARTWHREGRWRVLRLGAWARWLLVALTAATALVRLAQLGAADRQWTRFVRGRPRRFTSFDQVAQLSSAARGLAASLLFLLLVKAAQQLRFVRQWSVFGKTLCRALPELLGVTLGLVVLGVAYAQLAILLVSSCVDSLWSVAQALLVLCPGTGLSTLCPAESWHLSPLLCVGLWALRLWGALRLGAVILRWRYHALRGELYRPAWEPQDYEMVELFLRRLRLWMGLSKVKEFRHKVRFEGMEPLPSRSSRGSKVSPDVPPPSAGSDASHPSTSSSQLDGLSVSLGRLGTRCEPEPSRLQAVFEALLTQFDRLNQATEDVYQLEQQLHSLQGRRSSRAPAGSSRGPSPGLRPALPSRLARASRGVDLATGPSRTPLRAKNKVHPSST

>ENSP00000223661; Hs ECM2 (699aa)

MKIAVLFCFFLLIIFQTDFGKNEEIPRKQRRKIYHRRLRKSSTSHKHRSNRQLGIQQTTVFTPVARLPIVNFDYSMEEKFESFSSFPGVESSYNVLPGKKGHCLVKGITMYNKAVWSPEPCTTCLCSDGRVLCDETMCHPQRCPQTVIPEGECCPVCSATVSYSLLSGIALNDRNEFSGDSSEQREPTNLLHKQLPPPQVGMDRIVRKEALQSEEDEEVKEEDTEQKRETPESRNQGQLYSEGDSRGGDRKQRPGEERRLAHQQQRQGREEEEDEEEEGEEGEEDEEDEEDPVRGDMFRMPSRSPLPAPPRGTLRLPSGCSLSYRTISCINAMLTQIPPLTAPQITSLELTGNSIASIPDEAFNGLPNLERLDLSKNNITSSGIGPKAFKLLKKLMRLNMDGNNLIQIPSQLPSTLEELKVNENNLQAIDEESLSDLNQLVTLELEGNNLSEANVNPLAFKPLKSLAYLRLGKNKFRIIPQGLPGSIEELYLENNQIEEITEICFNHTRKINVIVLRYNKIEENRIAPLAWINQENLESIDLSYNKLYHVPSYLPKSLLHLVLLGNQIERIPGYVFGHMEPGLEYLYLSFNKLADDGMDRVSFYGAYHSLRELFLDHNDLKSIPPGIQEMKALHFLRLNNNKIRNILPEEICNAEEDDDSNLEHLHLENNYIKIREIPSYTFSCIRSYSSIVLKPQNIK

>ENSP00000021763; Hs GPR124 (1331aa)

MGAGGRRMRGAPARLLLPLLPWLLLLLAPEARGAPGCPLSIRSCKCSGERPKGLSGGVPGPARRRVVCSGGDLPEPPEPGLLPNGTVTLLLSNNKITGLRNGSFLGLSLLEKLDLRNNIISTVQPGAFLGLGELKRLDLSNNRIGCLTSETFQGLPRLLRLNISGNIFSSLQPGVFDELPALKVVDLGTEFLTCDCHLRWLLPWAQNRSLQLSEHTLCAYPSALHAQALGSLQEAQLCCEGALELHTHHLIPSLRQVVFQGDRLPFQCSASYLGNDTRIRWYHNRAPVEGDEQAGILLAESLIHDCTFITSELTLSHIGVWASGEWECTVSMAQGNASKKVEIVVLETSASYCPAERVANNRGDFRWPRTLAGITAYQSCLQYPFTSVPLGGGAPGTRASRRCDRAGRWEPGDYSHCLYTNDITRVLYTFVLMPINASNALTLAHQLRVYTAEAASFSDMMDVVYVAQMIQKFLGYVDQIKELVEVMVDMASNLMLVDEHLLWLAQREDKACSRIVGALERIGGAALSPHAQHISVNARNVALEAYLIKPHSYVGLTCTAFQRREGGVPGTRPGSPGQNPPPEPEPPADQQLRFRCTTGRPNVSLSSFHIKNSVALASIQLPPSLFSSLPAALAPPVPPDCTLQLLVFRNGRLFHSHSNTSRPGAAGPGKRRGVATPVIFAGTSGCGVGNLTEPVAVSLRHWAEGAEPVAAWWSQEGPGEAGGWTSEGCQLRSSQPNVSALHCQHLGNVAVLMELSAFPREVGGAGAGLHPVVYPCTALLLLCLFATIITYILNHSSIRVSRKGWHMLLNLCFHIAMTSAVFAGGITLTNYQMVCQAVGITLHYSSLSTLLWMGVKARVLHKELTWRAPPPQEGDPALPTPSPMLRFYLIAGGIPLIICGITAAVNIHNYRDHSPYCWLVWRPSLGAFYIPVALILLITWIYFLCAGLRLRGPLAQNPKAGNSRASLEAGEELRGSTRLRGSGPLLSDSGSLLATGSARVGTPGPPEDGDSLYSPGVQLGALVTTHFLYLAMWACGALAVSQRWLPRVVCSCLYGVAASALGLFVFTHHCARRRDVRASWRACCPPASPAAPHAPPRALPAAAEDGSPVFGEGPPSLKSSPSGSSGHPLALGPCKLTNLQLAQSQVCEAGAAAGGEGEPEPAGTRGNLAHRHPNNVHHGRRAHKSRAKGHRAGEACGKNRLKALRGGAAGALELLSSESGSLHNSPTDSYLGSSRNSPGAGLQLEGEPMLTPSEGSDTSAAPLSEAGRAGQRRSASRDSLKGGGALEKESHRRSYPLNAASLNGAPKGGKYDDVTLMGAEVASGGCMKTGLWKSETTV

>ENSP00000334952; Hs GPR125 (1321aa)

MEPPGRRRGRAQPPLLLPLSLLALLALLGGGGGGGAAALPAGCKHDGRPRGAGRAAGAAEGKVVCSSLELAQVLPPDTLPNRTVTLILSNNKISELKNGSFSGLSLLERLDLRNNLISSIDPGAFWGLSSLKRLDLTNNRIGCLNADIFRGLTNLVRLNLSGNLFSSLSQGTFDYLASLRSLEFQTEYLLCDCNILWMHRWVKEKNITVRDTRCVYPKSLQAQPVTGVKQELLTCDPPLELPSFYMTPSHRQVVFEGDSLPFQCMASYIDQDMQVLWYQDGRIVETDESQGIFVEKNMIHNCSLIASALTISNIQAGSTGNWGCHVQTKRGNNTRTVDIVVLESSAQYCPPERVVNNKGDFRWPRTLAGITAYLQCTRNTHGSGIYPGNPQDERKAWRRCDRGGFWADDDYSRCQYANDVTRVLYMFNQMPLNLTNAVATARQLLAYTVEAANFSDKMDVIFVAEMIEKFGRFTKEEKSKELGDVMVDIASNIMLADERVLWLAQREAKACSRIVQCLQRIATYRLAGGAHVYSTYSPNIALEAYVIKSTGFTGMTCTVFQKVAASDRTGLSDYGRRDPEGNLDKQLSFKCNVSNTFSSLALKNTIVEASIQLPPSLFSPKQKRELRPTDDSLYKLQLIAFRNGKLFPATGNSTNLADDGKRRTVVTPVILTKIDGVNVDTHHIPVNVTLRRIAHGADAVAARWDFDLLNGQGGWKSDGCHILYSDENITTIQCYSLSNYAVLMDLTGSELYTQAASLLHPVVYTTAIILLLCLLAVIVSYIYHHSLIRISLKSWHMLVNLCFHIFLTCVVFVGGITQTRNASICQAVGIILHYSTLATVLWVGVTARNIYKQVTKKAKRCQDPDEPPPPPRPMLRFYLIGGGIPIIVCGITAAANIKNYGSRPNAPYCWMAWEPSLGAFYGPASFITFVNCMYFLSIFIQLKRHPERKYELKEPTEEQQRLAANENGEINHQDSMSLSLISTSALENEHTFHSQLLGASLTLLLYVALWMFGALAVSLYYPLDLVFSFVFGATSLSFSAFFVVHHCVNREDVRLAWIMTCCPGRSSYSVQVNVQPPNSNGTNGEAPKCPNSSAESSCTNKSASSFKNSSQGCKLTNLQAAAAQCHANSLPLNSTPQLDNSLTEHSMDNDIKMHVAPLEVQFRTNVHSSRHHKNRSKGHRASRLTVLREYAYDVPTSVEGSVQNGLPKSRLGNNEGHSRSRRAYLAYRERQYNPPQQDSSDACSTLPKSSRNFEKPVSTTSKKDALRKPAVVELENQQKSYGLNLAIQNGPIKSNGQEGPLLGTDSTGNVRTGLWKHETTV

>ENSP00000259324; Hs LRRC8A (810aa)

MIPVTELRYFADTQPAYRILKPWWDVFTDYISIVMLMIAVFGGTLQVTQDKMICLPCKWVTKDSCNDSFRGWAAPGPEPTYPNSTILPTPDTGPTGIKYDLDRHQYNYVDAVCYENRLHWFAKYFPYLVLLHTLIFLACSNFWFKFPRTSSKLEHFVSILLKCFDSPWTTRALSETVVEESDPKPAFSKMNGSMDKKSSTVSEDVEATVPMLQRTKSRIEQGIVDRSETGVLDKKEGEQAKALFEKVKKFRTHVEEGDIVYRLYMRQTIIKVIKFILIICYTVYYVHNIKFDVDCTVDIESLTGYRTYRCAHPLATLFKILASFYISLVIFYGLICMYTLWWMLRRSLKKYSFESIREESSYSDIPDVKNDFAFMLHLIDQYDPLYSKRFAVFLSEVSENKLRQLNLNNEWTLDKLRQRLTKNAQDKLELHLFMLSGIPDTVFDLVELEVLKLELIPDVTIPPSIAQLTGLKELWLYHTAAKIEAPALAFLRENLRALHIKFTDIKEIPLWIYSLKTLEELHLTGNLSAENNRYIVIDGLRELKRLKVLRLKSNLSKLPQVVTDVGVHLQKLSINNEGTKLIVLNSLKKMANLTELELIRCDLERIPHSIFSLHNLQEIDLKDNNLKTIEEIISFQHLHRLTCLKLWYNHIAYIPIQIGNLTNLERLYLNRNKIEKIPTQLFYCRKLRYLDLSHNNLTFLPADIGLLQNLQNLAITANRIETLPPELFQCRKLRALHLGNNVLQSLPSRVGELTNLTQIELRGNRLECLPVELGECPLLKRSGLVVEEDLFNTLPPEVKERLWRADKEQA

>ENSP00000350933; Hs LRRC8B (803aa)

MITLTELKCLADAQSSYHILKPWWDVFWYYITLIMLLVAVLAGALQLTQSRVLCCLPCKVEFDNHCAVPWDILKASMNTSSNPGTPLPLPLRIQNDLHRQQYSYIDAVCYEKQLHWFAKFFPYLVLLHTLIFAACSNFWLHYPSTSSRLEHFVAILHKCFDSPWTTRALSETVAEQSVRPLKLSKSKILLSSSGCSADIDSGKQSLPYPQPGLESAGIESPTSSVLDKKEGEQAKAIFEKVKRFRMHVEQKDIIYRVYLKQIIVKVILFVLIITYVPYFLTHITLEIDCSVDVQAFTGYKRYQCVYSLAEIFKVLASFYVILVILYGLTSSYSLWWMLRSSLKQYSFEALREKSNYSDIPDVKNDFAFILHLADQYDPLYSKRFSIFLSEVSENKLKQINLNNEWTVEKLKSKLVKNAQDKIELHLFMLNGLPDNVFELTEMEVLSLELIPEVKLPSAVSQLVNLKELRVYHSSLVVDHPALAFLEENLKILRLKFTEMGKIPRWVFHLKNLKELYLSGCVLPEQLSTMQLEGFQDLKNLRTLYLKSSLSRIPQVVTDLLPSLQKLSLDNEGSKLVVLNNLKKMVNLKSLELISCDLERIPHSIFSLNNLHELDLRENNLKTVEEIISFQHLQNLSCLKLWHNNIAYIPAQIGALSNLEQLSLDHNNIENLPLQLFLCTKLHYLDLSYNHLTFIPEEIQYLSNLQYFAVTNNNIEMLPDGLFQCKKLQCLLLGKNSLMNLSPHVGELSNLTHLELIGNYLETLPPELEGCQSLKRNCLIVEENLLNTLPLPVTERLQTCLDKC

>ENSP00000304477; Hs LRRC8C (803aa)

MIPVTEFRQFSEQQPAFRVLKPWWDVFTDYLSVAMLMIGVFGCTLQVMQDKIICLPKRVQPAQNHSSLSNVSQAVASTTPLPPPKPSPANPITVEMKGLKTDLDLQQYSFINQMCYERALHWYAKYFPYLVLIHTLVFMLCSNFWFKFPGSSSKIEHFISILGKCFDSPWTTRALSEVSGEDSEEKDNRKNNMNRSNTIQSGPEDSLVNSQSLKSIPEKFVVDKSTAGALDKKEGEQAKALFEKVKKFRLHVEEGDILYAMYVRQTVLKVIKFLIIIAYNSALVSKVQFTVDCNVDIQDMTGYKNFSCNHTMAHLFSKLSFCYLCFVSIYGLTCLYTLYWLFYRSLREYSFEYVRQETGIDDIPDVKNDFAFMLHMIDQYDPLYSKRFAVFLSEVSENKLKQLNLNNEWTPDKLRQKLQTNAHNRLELPLIMLSGLPDTVFEITELQSLKLEIIKNVMIPATIAQLDNLQELSLHQCSVKIHSAALSFLKENLKVLSVKFDDMRELPPWMYGLRNLEELYLVGSLSHDISRNVTLESLRDLKSLKILSIKSNVSKIPQAVVDVSSHLQKMCIHNDGTKLVMLNNLKKMTNLTELELVHCDLERIPHAVFSLLSLQELDLKENNLKSIEEIVSFQHLRKLTVLKLWHNSITYIPEHIKKLTSLERLSFSHNKIEVLPSHLFLCNKIRYLDLSYNDIRFIPPEIGVLQSLQYFSITCNKVESLPDELYFCKKLKTLKIGKNSLSVLSPKIGNLLFLSYLDVKGNHFEILPPELGDCRALKRAGLVVEDALFETLPSDVREQMKTE

>ENSP00000338887; Hs LRRC8D (858aa)

MFTLAEVASLNDIQPTYRILKPWWDVFMDYLAVVMLMVAIFAGTMQLTKDQVVCLPVLPSPVNSKAHTPPGNAEVTTNIPKMEAATNQDQDGRTTNDISFGTSAVTPDIPLRATYPRTDFALPNQEAKKEKKDPTGRKTNLDFQQYVFINQMCYHLALPWYSKYFPYLALIHTIILMVSSNFWFKYPKTCSKVEHFVSILGKCFESPWTTKALSETACEDSEENKQRITGAQTLPKHVSTSSDEGSPSASTPMINKTGFKFSAEKPVIEVPSMTILDKKDGEQAKALFEKVRKFRAHVEDSDLIYKLYVVQTVIKTAKFIFILCYTANFVNAISFEHVCKPKVEHLIGYEVFECTHNMAYMLKKLLISYISIICVYGFICLYTLFWLFRIPLKEYSFEKVREESSFSDIPDVKNDFAFLLHMVDQYDQLYSKRFGVFLSEVSENKLREISLNHEWTFEKLRQHISRNAQDKQELHLFMLSGVPDAVFDLTDLDVLKLELIPEAKIPAKISQMTNLQELHLCHCPAKVEQTAFSFLRDHLRCLHVKFTDVAEIPAWVYLLKNLRELYLIGNLNSENNKMIGLESLRELRHLKILHVKSNLTKVPSNITDVAPHLTKLVIHNDGTKLLVLNSLKKMMNVAELELQNCELERIPHAIFSLSNLQELDLKSNNIRTIEEIISFQHLKRLTCLKLWHNKIVTIPPSITHVKNLESLYFSNNKLESLPVAVFSLQKLRCLDVSYNNISMIPIEIGLLQNLQHLHITGNKVDILPKQLFKCIKLRTLNLGQNCITSLPEKVGQLSQLTQLELKGNCLDRLPAQLGQCRMLKKSGLVVEDHLFDTLPLEVKEALNQDINIPFANGI

>ENSP00000306524; Hs LRRC8E (796aa)

MIPVAEFKQFTEQQPAFKVLKPWWDVLAEYLTVAMLMIGVFGCTLQVTQDKIICLPNHELQENLSEAPCQQLLPRGIPEQIGALQEVKGLKNNLDLQQYSFINQLCYETALHWYAKYFPYLVVIHTLIFMVCTSFWFKFPGTSSKIEHFISILGKCFDSPWTTRALSEVSGENQKGPAATERAAATIVAMAGTGPGKAGEGEKEKVLAEPEKVVTEPPVVTLLDKKEGEQAKALFEKVKKFRMHVEEGDILYTMYIRQTVLKVCKFLAILVYNLVYVEKISFLVACRVETSEVTGYASFCCNHTKAHLFSKLAFCYISFVCIYGLTCIYTLYWLFHRPLKEYSFRSVREETGMGDIPDVKNDFAFMLHLIDQYDSLYSKRFAVFLSEVSESRLKQLNLNHEWTPEKLRQKLQRNAAGRLELALCMLPGLPDTVFELSEVESLRLEAICDITFPPGLSQLVHLQELSLLHSPARLPFSLQVFLRDHLKVMRVKCEELREVPLWVFGLRGLEELHLEGLFPQELARAATLESLRELKQLKVLSLRSNAGKVPASVTDVAGHLQRLSLHNDGARLVALNSLKKLAALRELELVACGLERIPHAVFSLGALQELDLKDNHLRSIEEILSFQHCRKLVTLRLWHNQIAYVPEHVRKLRSLEQLYLSYNKLETLPSQLGLCSGLRLLDVSHNGLHSLPPEVGLLQNLQHLALSYNALEALPEELFFCRKLRTLLLGDNQLSQLSPHVGALRALSRLELKGNRLEALPEELGNCGGLKKAGLLVEDTLYQGLPAEVRDKMEEE

>ENSP00000265991; Hs LGI1 (557aa)

MESERSKRMGNACIPLKRIAYFLCLLSALLLTEGKKPAKPKCPAVCTCTKDNALCENARSIPRTVPPDVISLSFVRSGFTEISEGSFLFTPSLQLLLFTSNSFDVISDDAFIGLPHLEYLFIENNNIKSISRHTFRGLKSLIHLSLANNNLQTLPKDIFKGLDSLTNVDLRGNSFNCDCKLKWLVEWLGHTNATVEDIYCEGPPEYKKRKINSLSSKDFDCIITEFAKSQDLPYQSLSIDTFSYLNDEYVVIAQPFTGKCIFLEWDHVEKTFRNYDNITGTSTVVCKPIVIETQLYVIVAQLFGGSHIYKRDSFANKFIKIQDIEILKIRKPNDIETFKIENNWYFVVADSSKAGFTTIYKWNGNGFYSHQSLHAWYRDTDVEYLEIVRTPQTLRTPHLILSSSSQRPVIYQWNKATQLFTNQTDIPNMEDVYAVKHFSVKGDVYICLTRFIGDSKVMKWGGSSFQDIQRMPSRGSMVFQPLQINNYQYAILGSDYSFTQVYNWDAEKAKFVKFQELNVQAPRSFTHVSINKRNFLFASSFKGNTQIYKHVIVDLSA

>ENSP00000282970; Hs LGI2 (545aa)

MALRRGGCGALGLLLLLLGAACLIPRSAQVRRLARCPATCSCTKESIICVGSSWVPRIVPGDISSLSLVNGTFSEIKDRMFSHLPSLQLLLLNSNSFTIIRDDAFAGLFHLEYLFIEGNKIETISRNAFRGLRDLTHLSLANNHIKALPRDVFSDLDSLIELDLRGNKFECDCKAKWLYLWLKMTNSTVSDVLCIGPPEYQEKKLNDVTSFDYECTTTDFVVHQTLPYQSVSVDTFNSKNDVYVAIAQPSMENCMVLEWDHIEMNFRSYDNITGQSIVGCKAILIDDQVFVVVAQLFGGSHIYKYDESWTKFVKFQDIEVSRISKPNDIELFQIDDETFFVIADSSKAGLSTVYKWNSKGFYSYQSLHEWFRDTDAEFVDIDGKSHLILSSRSQVPIILQWNKSSKKFVPHGDIPNMEDVLAVKSFRMQNTLYLSLTRFIGDSRVMRWNSKQFVEIQALPSRGAMTLQPFSFKDNHYLALGSDYTFSQIYQWDKEKQLFKKFKEIYVQAPRSFTAVSTDRRDFFFASSFKGKTKIFEHIIVDLSL

>ENSP00000302297; Hs LGI3 (548aa)

MAGLRARGGPGPGLLALSALGFCLMLQVSAKRPPKTPPCPPSCSCTRDTAFCVDSKAVPRNLPSEVISLTLVNAAFSEIQDGAFSHLPLLQFLLLNSNKFTLIGDNAFTGLSHLQYLFIENNDIWALSKFTFRGLKSLTHLSLANNNLQTLPRDIFRPLDILNDLDLRGNSLNCDCKVKWLVEWLAHTNTTVAPIYCASPPRFQEHKVQDLPLREFDCITTDFVLYQTLAFPAVSAEPFLYSSDLYLALAQPGVSACTILKWDYVERQLRDYDRIPAPSAVHCKPMVVDSQLYVVVAQLFGGSYIYHWDPNTTRFTRLQDIDPQRVRKPNDLEAFRIDGDWYFAVADSSKAGATSLYRWHQNGFYSHQALHPWHRDTDLEFVDGEGKPRLIVSSSSQAPVIYQWSRTQKQFVAQGEVTQVPDAQAVKHFRAGRDSYLCLSRYIGDSKILRWEGTRFSEVQALPSRGSLALQPFLVGGRRYLALGSDFSFTQIYQWDEGRQKFVRFQELAVQAPRAFCYMPAGDAQLLLAPSFKGQTLVYRHIVVDLSA

>ENSP00000312273; Hs LGI4 (537aa)

MGGAGILLLLLAGAGVVVAWRPPKGKCPLRCSCSKDSALCEGSPDLPVSFSPTLLSLSLVRTGVTQLKAGSFLRIPSLHLLLFTSNSFSVIEDDAFAGLSHLQYLFIEDNEIGSISKNALRGLRSLTHLSLANNHLETLPRFLFRGLDTLTHVDLRGNPFQCDCRVLWLLQWMPTVNASVGTGACAGPASLSHMQLHHLDPKTFKCRAIELSWFQTVGESALSVEPFSYQGEPHIVLAQPFAGRCLILSWDYSLQRFRPEEELPAASVVSCKPLVLGPSLFVLAARLWGGSQLWARPSPGLRLAPTQTLAPRRLLRPNDAELLWLEGQPCFVVADASKAGSTTLLCRDGPGFYPHQSLHAWHRDTDAEALELDGRPHLLLASASQRPVLFHWTGGRFERRTDIPEAEDVYATRHFQAGGDVFLCLTRYIGDSMVMRWDGSMFRLLQQLPSRGAHVFQPLLIARDQLAILGSDFAFSQVLRLEPDKGLLEPLQELGPPALVAPRAFAHITMAGRRFLFAACFKGPTQIYQHHEIDLSA

>ENSP00000306864; Hs VASN=SLITL2 (673aa)

MCSRVPLLLPLLLLLALGPGVQGCPSGCQCSQPQTVFCTARQGTTVPRDVPPDTVGLYVFENGITMLDAGSFAGLPGLQLLDLSQNQIASLPSGVFQPLANLSNLDLTANRLHEITNETFRGLRRLERLYLGKNRIRHIQPGAFDTLDRLLELKLQDNELRALPPLRLPRLLLLDLSHNSLLALEPGILDTANVEALRLAGLGLQQLDEGLFSRLRNLHDLDVSDNQLERVPPVIRGLRGLTRLRLAGNTRIAQLRPEDLAGLAALQELDVSNLSLQALPGDLSGLFPRLRLLAAARNPFNCVCPLSWFGPWVRESHVTLASPEETRCHFPPKNAGRLLLELDYADFGCPATTTTATVPTTRPVVREPTALSSSLAPTWLSPTEPATEAPSPPSTAPPTVGPVPQPQDCPPSTCLNGGTCHLGTRHHLACLCPEGFTGLYCESQMGQGTRPSPTPVTPRPPRSLTLGIEPVSPTSLRVGLQRYLQGSSVQLRSLRLTYRNLSGPDKRLVTLRLPASLAEYTVTQLRPNATYSVCVMPLGPGRVPEGEEACGEAHTPPAVHSNHAPVTQAREGNLPLLIAPALAAVLLAALAAVGAAYCVRRGRAMAAAAQDKGQVGPGAGPLELEGVKVPLEPGPKATEGGGEALPSGSECEVPLMGFPGPGLQSPLHAKPYI

>AK004926.1; Igfals (seq from RIKEN BAB23677)

1 malrtgspal vvllafwval gpcylqgtdp gasadaegpq cpvtctcsyd dytdelsvfc

61 ssrnltqlpd sipvstralw ldgnnlssip saafqnlssl dflnlqgswl rslepqallg

121 lqnlyhlhle rnllrslaag lfrhtpslas lslgnnllgr leeglfrgls hlwdlnlgwn

181 slvvlpdtvf qglgnlhelv lagnkltylq pallcglgel reldlsrnal rsvkanvfih

241 lprlqklyld rnlitavapr aflgmkalrw ldlshnrvag lledtfpgll glhvlrlahn

301 aitslrprtf kdlhfleelq lghnrirqlg ektfeglgql evltlndnqi hevkvgaffg

361 lfnvavmnls gnclrslpeh vfqglgrlhs lhlehsclgr irlhtfagls glrrlflrdn

421 sissieeqsl aglselleld ltanqlthlp rqlfqglgql eylllsnnql tmlsedvlgp

481 lqrafwldls hnrletpaeg lfsslgrlry lnlrnnslqt fvpqpglerl wldanpwdcs

541 cplkalrdfa lqnpgvvprf vqtvcegddc qpvytynnit cagpanvsgl dlrdisetlf

601 vhc

>ENSP00000319464;CPN2 (Ensembl update; release 40)

MLPGAWLLWTSLLLLARPAQPCPMGCDCFVQEVFCSDEELATVPL

DIPPYTKNIIFVETSFTTLETRAFGSNPNLTKVVFLNTQLCQFRPDAFGGLPRLEDLEVT

GSSFLNLSTNIFSNLTSLGKLTLNFNMLEALPEGLFQHLAALESLHLQGNQLQALPRRLF

QPLTHLKTLNLAQNLLAQLPEELFHPLTSLQTLKLSNNALSGLPQGVFGKLGSLQELFLD

SNNISELPPQVFSQLFCLERLWLQRNAITHLPLSIFASLGNLTFLSLQWNMLRVLPAGLF

AHTPCLVGLSLTHNQLETVAEGTFAHLSNLRSLMLSYNAITHLPAGIFRDLEELVKLYLG

SNNLTALHPALFQNLSKLELLSLSKNQLTTLPEGIFDTNYNLFNLALHGNPWQCDCHLAY

LFNWLQQYTDRLLNIQTYCAGPAYLKGQVVPALNEKQLVCPVTRDHLGFQVTWPDESKAG

GSWDLAVQERAARSQCTYSNPEGTVVLACDQAQCRWLNVQLSPWQGSLGLQYNASQEWDL

RSSCGSLRLTVSIEARAAGP

>ENSMUSP00000069318;Cpn2 (Ensembl update; release 40)

MFPGAWLCWVSLLLLARLTQPCPVGCDCFGREVFCSD

EQLADIPPDIPPHITDIVFVETAFTTVRTRAFSGSPNLTKVVFLNTQVRHLEPDAFGGLP

RLQDLEITGSPVSNLSAHIFSNLSSLEKLTLDFDRLAGLPEDLFCHMDILESLQLQGNQL

RTLPGRLFQSLRDLRTLNLAQNLLTQLPKGAFQSLTGLQMLKLSNNMLARLPEGALGSLS

SLQELFLDGNAITELSPHLFSQLFSLEMLWLQHNAICHLPVSLFSSLHNLTFLSLKDNAL

RTLPEGLFAHNQGLLHLSLSYNQLETIPEGAFTNLSRLVSLTLSHNAITDLPEHVFRNLE

# QLVKLSLDSNNLTALHPALFHNLSRLQLLNLSRNQLTTLPGGIFDTNYDLFNLALLGNPW

QCDCHLSYLTSWLRLYNNQISNTHTFCAGPAYLKGQLVPNLKQEQLICPVNPGHLSFRAL

GLDEGEPAGSWDLTVEGRAAHSQCAYSNPEGTVLLACEESRCRWLNIQLSSRDGSDSAAM

VYNSSQEWGLRSSCGLLRVTVSIEAPAAGP

>BC036337; mKIAA0644

MEAA RALRLLLVVC GCLALPPLAE PVCPERCDCQ HPQHLLCTNR

GLRVVPKTSS LPSPHDVLTY SLGGNFITNI TAFDFHRLGQ LRRLDLQYNQ IRSLHPKTFE

KLSRLEELYL GNNLLQALAP GTLAPLRKLR ILYANGNEIS RLSRGSFEGL ESLVKLRLDG

NALGALPDAV FAPLGNLLYL HLESNRIRFL GKNAFAQLGK LRFLNLSANE LQPSLRHAAT

FAPLRSLSSL ILSANSLQHL GPRIFQHLPR LGLLSLRGNQ LTHLAPEAFW GLEALRELRL

EGNRLSQLPT ALLEPLHSLE ALDLSGNELS ALHPATFGHL GRLRELSLRN NALSALSGDI

FAASPALYRL DLDGNGWTCD CRLRGLKRWM GDWHSQGRLL TVFVQCRHPP ALRGKYLDYL

DDQQLQNGSC ADPSPSASLT ADRRRQPLPT AAGEEMTPPA GLAEELPPQP QLQQQGRFLA

GVAWDGAARE LVGNRSALRL SRRGPGLQQP SPSVAAAAGP APQSLDLHKK PQRGRPTRAD

PALAEPTPTA SPGSAPSPAG DPWQRATKHR LGTEHQERAA QSDGGAGLPP LVSDPCDFNK

FILCNLTVEA VGADSASVRW AVREHRSPRP LGGARFRLLF DRFGQQPKFH RFVYLPESSD

SATLRELRGD TPYLVCVEGV LGGRVCPVAP RDHCAGLVTL PEAGSRGGVD YQLLTLALLT

VNALLVLLAL AAWASRWLRR KLRARRKGGA PVHVRHMYST RRPLRSMGTG VSADFSGFQS

HRPRTTVCAL SEADLIEFPC DRFMDSAGGG AGGSLRREDR LLQRFAD

>BC043099; KIAA0644

megvgavrfw lvvcgclafp praesvcper cdcqhpqhll ctnrglravp ktsslpspqd

vltyslggnf itnitafdfh rlgqlrrldl qynqirslhp ktfeklsrle elylgnnllq

alvpgtlapl rklrilyang neigrlsrgs fegleslvkl rldgnvlgal pdavfaplgn

llylhlesnr irflgknafs qlgklrflnl sanelqpslr haatfvplrs lstlilsans

lqhlgprvfq hlprlgllsl sgnqlthlap eafwglealr elrlegnrln qlpltllepl

hslealdlsg nelsalhpat fghqgrlrel slrdnalsal sgdifaaspa lyrldldgng

wtcdcrlrgl krwmgnwhsq grlltvfvqc rhppalrgky ldylddqllq ngscvdpsps

ptagsrqwpl ptsseegmtp paglsqelpl qpqpqpqqrg rllpgvawgg aakelvgnrs

alrlsrrgpg phqgpsaaap gsapqsldlh ekpgrgrhtr anlsqteptp tsepasgtps

ardswqraak qrlaseqqes avqsvsgvgl pplvsdpcdf nkfilcnltv eavsansasv

rwavrehrsp rpqggarfrl lfdrfgqqpk fqrfvylper sdsatlhelr gdtpylvcve

gvlggrvcpv aprdhcaglv tlpeaggrgg vdyqlltlvl lavnallvll alaawgsrwl

rrklrarrkg gapvhvrhmy strrplrsmg tgvsadfsgf qshrprttvc alseadlief

pcdrfmdstg ggtsgslrre dhllqrfad

>Q9BXB1; LGR4 (Hs) (SwissProt)

1 mpgplgllcf lalgllgsag psgaapplca apcscdgdrr vdcsgkglta vpeglsaftq

61 aldismnnit qlpedafknf pfleelqlag ndlsfihpka lsglkelkvl tlqnnqlktv

121 pseairglsa lqslrldanh itsvpedsfe glvqlrhlwl ddnsltevpv hplsnlptlq

181 altlalnkis sipdfaftnl sslvvlhlhn nkirglsqhc fdgldnletl dlsynnlgef

241 pqaikarpsl kelgfhsnsi svipdgafdg npllrtihly dnplsfvgns afhnlsdlhs

301 lvirgasmvq qfpnltgtvh lesltltgtk issipnnlcq eqkmlrtldl synnirdlps

361 fngchaleei slqrnqiyqi kegtfqglis lrildlsrnl iheihsrafa tlgpitnldv

421 sfneltsfpt eglnglnqlk lvgnfklkea laakdfvnlr slsvpyayqc cafwgcdsya

481 nlntednslq dhsvaqekgt adaanvtstl eneehsqiii hctpstgafk pceyllgswm

541 irltvwfifl valffnllvi lttfasctsl pssklfigli svsnlfmgiy tgiltfldav

601 swgrfaefgi wwetgsgckv agflavfsse saifllmlat verslsakdi mkngksnhlk

661 qfrvaallaf lgatvagcfp lfhrgeysas plclpfptge tpslgftvtl vllnslafll

721 maviytklyc nlekedlsen sqssmikhva wliftnciff cpvaffsfap litaisispe

781 imksvtliff plpaclnpvl yvffnpkfke dwkllkrrvt kksgsvsvsi ssqggcleqd

841 fyydcgmysh lqgnltvcdc cesflltkpv sckhlikshs cpalavascq rpegywsdcg

901 tqsahsdyad eedsfvsdss dqvqacgrac fyqsrgfplv ryaynlprvk d

>CG8930-PA; CG8930-PA, rk

MAARCRWSWRLALCPLLLQLLLQLLLLPPSAMGHDETKENPAPDMQNSQEQEPYVHLQHL

QQQQQQNPQTVQQLSQITVNRTSKSASVTPTGIRENVMLPSADPEKEAQILYEKSLQEYH

GSQLSTASTATDVIAGKRTLHSICERWLQKHCHCTGSLEVLRLSCRGIGILAVPVNLPNE

VVVLDLGNNNLTKLEANSFFMAPNLEELTLSDNSIINMDPNAFYGLAKLKRLSLQNCGLK

SLPPQSFQGLAQLTSLQLNGNALVSLDGDCLGHLQKLRTLRLEGNLFYRIPTNALAGLRT

LEALNLGSNLLTIINDEDFPRMPNLIVLLLKRNQIMKISAGALKNLTALKVLELDDNLIS

SLPEGLSKLSQLQELSITSNRLRWINDTELPRSMQMLDMRANPLSTISPGAFRGMSKLRK

LILSDVRTLRSFPELEACHALEILKLDRAGIQEVPANLCRQTPRLKSLELKTNSLKRIPN

LSSCRDLRLLDLSSNQIEKIQGKPFNGLKQLNDLLLSYNRIKALPQDAFQGIPKLQLLDL

EGNEISYIHKEAFSGFTALEDLNLGNNIFPELPESGLRALLHLKTFNNPKLREFPPPDTF

PRIQTLILSYAYHCCAFLPLVAMSSQKKTSQVQEAVLFPSDAEFDMTLWNNSMMNIWPQM

HNLSKQLGASMHDPWETAINFNEEQLQTQTGGQIATSYMEEYFEEHDVSGPATGYGFGTG

LFSGMSTEDFQPGSVQCLPMPGPFLPCADLFDWWTLRCGVWVVFLLSLLGNGTVVFVLLC

SRSKMDVPRFLVCNLAAADFFMGIYLGILAIVDAATLGEFRMFAIPWQMSVLCQLSGFLA

VLSSELSVYTLAVITLERNYAITHAIHLNKRLSLKQAGYIMSVGWVFALIMALMPLVGVS

DYRKFAVCLPFETTTGPASLTYVISLMFINGCAFLTLMGCYLKMYWAIRGSQAWNTNDSR

IAKRMALLVFTDFLCWSPIAFFSITAIFGLQLISLEQAKIFTVFVLPLNSCCNPFLYAIM

TKQFKKDCVTLCKHFEESRVVGGGGPGGRGAVARTKRGDLPPPLLPAAAVAHPPGCRCLR

MLPSEMPNWHKMEQTPSMWQRLRTFCCGENRRRRKQRRQPQQRRQRAYTAAAANPYQYQF

AELRQQRQNRASSISSENFCSSRSSSWRHGPPSSAPVPPGNCSMPLKMLEPHAHPHGHGR

RRHSAWLITRKTSQDSNLSSSRNDSSASATTASTSTFRLSRSSAGSSTPLPSIIAHNGKA

QLDAVKPRLVRQEAVQEEEDSSPPRLGVRFLPTIPSAADSSVVMEDGDSANTGVASFLGM

PLPGASSGFLIAPTTAATTSPPPVVLQPAKPPPDPNDAPL

>CG8595-PA;Toll-7

MAAILLLLLGFSWSLAVESALAPKESESSASAMLGAGTGAAATVSLSGDYSSLLSNVPAA

SPVPANPSQPSGPANQCSWSYNGTSSVHCALRLIERQPGLDLQGADGSSQLTIQCSELYL

FESTLPVAVFARLQTLEALRLDSCKLLQLPNNAFEGLATLKSLRLSTHNSEWGPTRTLEL

FPDSLGGLKQLTDLDLGDNNLRQLPSGFLCPVGNLQVLNLTRNRIRTAEQMGFADMNCGA

GSGSAGSELQVLDASHNELRSISESWGISRLRRLQHLNLAYNNLSELSGEALAGLASLRI

VNLSNNHLETLPEGLFAGSKELREIHLQQNELYELPKGLFHRLEQLLVVDLSGNQLTSNH

VDNTTFAGLIRLIVLNLAHNALTRIDYRTFKELYFLQILNLRNNSIGHIEDNAFLPLYNL

HTLNLAENRLHTLDDKLFNGLYVLSKLTLNNNLISVVEPAVFKNCSDLKELDLSSNQLNE

VPRALQDLAMLRTLDLGENQIRTFDNQSFKNLHQLTGLRLIDNQIGNITVGMFQDLPRLS

VLNLAKNRIQSIERGSFDKNFELEAIRLDRNFLADINGVFATLVSLLWLNLSENHLVWFD

YAFIPSNLKWLDIHGNYIEALGNYYKLQEEIRVKTLDASHNRITEIGPMSIPNTIELLFI

NNNLIGNVQPNAFVDKANLARVDLYANQLSKLQLQQLRVAPVVAPKPLPEFYLGGNPFEC

DCTMDWLQRINNLTTRQHPRVMDMANIECVMPHARGAAVRPLSGLRPQDFLCRYESHCFA

LCHCCDFDACDCEMTCPSNCTCYHDQIWSTNVVDCGGQQTTELPRRVPMDSSVVYLDGNN

FPVLKNHAFIGRKNLRALYVNGSQVAAIQNRTFASLASLQLLHLADNKLRTLHGYEFEQL

SALRELYLQNNQLTTIENATLAPLAALELIRIDGNRLVTLPIWQMHATHFGTRLKSISLG

RNQWSCRCQFLQALTSYVADNALIVQDAQDIYCMAASSGTGSAALEDSSSNSGSLEKREL

DFNATGAACTDYYSGGSMLQHGIPESYIPLLAAALALLFLLVVIAMVFAFRESLRIWLFA

HYGVRVFGPRCEESEKLYDAVLLHSAKDSEFVCQHLAAQLETGRPPLRVCLQHRDLAHDA

THYQLLEATRVSRRVVILLTRNFLQTEWARCELRRSVHDALRGRPQKLVIIEEPEVAFEA

ESDIELLPYLKTSAVHRIRRSDRHFWEKLRYALPVDYPTFRGNNYTLELDHHNHERVKQP

ASPGLLYRQAPPPAYCGPADAVGIGAVPQVVPVNASVPAEQNYSTATTATPSPRPQRRGE

QPGSGSGGNHHLHAQYYQHHGMRPPSEHIYSSIDSDYSTLDNEQHMLMMPGAPGGLAMEA

AQRAQTWRPKREQLHLQQAQAGTLGSKASQAAHQQQQQQQQQQQQQPNPTAVSGQQQGPH

VQAYLV

>ENSP00000215539;IGFALS

MALRKGGLALALLLLSWVALGPRSLEGADPGTPGEAEGPACPAACVCSYDDDADELSVFC

SSRNLTRLPDGVPGGTQALWLDGNNLSSVPPAAFQNLSSLGFLNLQGGQLGSLEPQALLG

LENLCHLHLERNQLRSLALGTFAHTPALASLGLSNNRLSRLEDGLFEGLGSLWDLNLGWN

SLAVLPDAAFRGLGSLRELVLAGNRLAYLQPALFSGLAELRELDLSRNALRAIKANVFVQ

LPRLQKLYLDRNLIAAVAPGAFLGLKALRWLDLSHNRVAGLLEDTFPGLLGLRVLRLSHN

AIASLRPRTFKDLHFLEELQLGHNRIRQLAERSFEGLGQLEVLTLDHNQLQEVKAGAFLG

LTNVAVMNLSGNCLRNLPEQVFRGLGKLHSLHLEGSCLGRIRPHTFTGLSGLRRLFLKDN

GLVGIEEQSLWGLAELLELDLTSNQLTHLPHRLFQGLGKLEYLLLSRNRLAELPADALGP

LQRAFWLDVSHNRLEALPNSLLAPLGRLRYLSLRNNSLRTFTPQPPGLERLWLEGNPWDC

GCPLKALRDFALQNPSAVPRFVQAICEGDDCQPPAYTYNNITCASPPEVVGLDLRDLSEA

HFAPC

>ENSMUSP00000051895;Gp5

MLRSALLSAVLALLRAQPFPCPKTCKCVVRDAAQCSGGSVAHIAELGLPTNLTHILLFRM

DQGILRNHSFSGMTVLQRLMLSDSHISAIDPGTFNDLVKLKTLRLTRNKISRLPRAILDK

MVLLEQLFLDHNALRDLDQNLFQQLRNLQELGLNQNQLSFLPANLFSSLRELKLLDLSRN

NLTHLPKGLLGAQVKLEKLLLYSNQLTSVDSGLLSNLGALTELRLERNHLRSVAPGAFDR

LGNLSSLTLSGNLLESLPPALFLHVSSVSRLTLFENPLEELPDVLFGEMAGLRELWLNGT

HLSTLPAAAFRNLSGLQTLGLTRNPRLSALPRGVFQGLRELRVLALHTNALAELRDDALR

GLGHLRQVSLRHNRLRALPRTLFRNLSSLESVQLEHNQLETLPGDVFAALPQLTQVLLGH

NPWLCDCGLWPFLQWLRHHPDILGRDEPPQCRGPEPRASLSFWELLQGDPWCPDPRSLPL

DPPTENALEAPVPSWLPNSWQSQTWAQLVARGESPNNRLYWGLYILLLVAQAIIAAFIVF

AMIKIGQLFRTLIREKLLLEAMGKSCN

>ENSP00000319286;GP5

MLRGTLLCAVLGLLRAQPFPCPPACKCVFRDAAQCSGGDVARISALGLPTNLTHILLFGM

GRGVLQSQSFSGMTVLQRLMISDSHISAVAPGTFSDLIKLKTLRLSRNKITHLPGALLDK

MVLLEQLFLDHNALRGIDQNMFQKLVNLQELALNQNQLDFLPASLFTNLENLKLLDLSGN

NLTHLPKGLLGAQAKLERLLLHSNRLVSLDSGLLNSLGALTELQFHRNHIRSIAPGAFDR

LPNLSSLTLSRNHLAFLPSALFLHSHNLTLLTLFENPLAELPGVLFGEMGGLQELWLNRT

QLRTLPAAAFRNLSRLRYLGVTLSPRLSALPQGAFQGLGELQVLALHSNGLTALPDGLLR

GLGKLRQVSLRRNRLRALPRALFRNLSSLESVQLDHNQLETLPGDVFGALPRLTEVLLGH

NSWRCDCGLGPFLGWLRQHLGLVGGEEPPRCAGPGAHAGLPLWALPGGDAECPGPRGPPP

RPAADSSSEAPVHPALAPNSSEPWVWAQPVTTGKGQDHSPFWGFYFLLLAVQAMITVIIV

FAMIKIGQLFRKLIRERALG

>CG1744-PA;chp

MGLEFFFKFGYAFLTITLMIMIWMSLARASMFDREMEETHYPPCTYNVMCTCSKSSTDLG

IVHCKNVPFPALPRMVNQSKVFMLHMENTGLREIEPYFLQSTGMYRLKISGNHLTEIPDD

AFTGLERSLWELILPQNDLVEIPSKSLRHLQKLRHLDLGYNHITHIQHDSFRGLEDSLQT

LILRENCISQLMSHSFSGLLILETLDLSGNNLFEIDPNVFVDGMPRLTRLLLTDNILSEI

PYDALGPLKSLRTLDISHNVIWSLSGNETYEIKASTKLNLDNLHLEYNHIEVLPPNSFKY

FDTVNRTFFDGNPIHTLREDAFKPARIREIYMRYCGLTNISPVAFDSLVNSLQILDLSGN

NLTKLHHKLFNNFDVLRVISMRDNKIKIQKPTETFNAVHYTLLKLDLSGDRNDPTNLQTL

RNMTRMRNMRSLSISRLGSSSVGPEDFKDFGVELEDLQITRASLSGIQSHAFKHVRGLKR

LDFSENGISSIENDAFHEIGHSLISLKMSHGYSGSALPAEPLRHLTSLQELDFSNNHISS

MSDTSFHFLKNLRLLELHDNRIEQVLKGTFQGDIHSKLEEISLRFNHLTSISQHTFFDLE

ALRKLHLDDNKIDKIERRAFMNLDELEYLSLRGNKINNLADESFQNLPKLEILDMAFNQL

PNFNFDYFDQVGTLSNLNVNVSHNQIRQLMYNSSWSGRNEHGGMYHSNIKILDLSHNNIS

IIHPGYFRPAEISLTHLHLGYNSLMNTTRDVFGNMPHLQWLDLSYNWIHELDFDAFKNTK

QLQLVFFGHNYLSDIPQDIFKPVQGLRIVDFSHNHLRGLPDNLFYNGGMEKLDVSHNMML

KIPSSSLSSLAALTLCELHLSNNFISTIHSMDLSNKFRSLRYLDISYNYLLRIDDAVFAT

MPKLAVLDLSHNRDLKVMDKSFMGLENSLIKLGLENISLSTVPEIRLKYLREFRLGYNEL

PSIPQELAHNMSNLRMLDLSNNDLTNVPLMTQALPHLRRLMLSGNPITSLNNNSFDGVNE

DLEMLDISNFRLHYFEYGCLDSLPHLRSLKLTAYSHLEHFNIPHLLRHHYNIRQLWIEAP

QPFTRIVKKGSGPTQEMQTLQLGNPTDLQREMEGHLPSKLTNITFSGPQFTNLNERILRG

MRSPYLYMQLFNTSLQALPPNFFKYMGRVRNISLDIRYHNRNLKKIPNPNTGAVPYLPNS

VFLTDLKMSHTDLNCDCDLGWVEFWQRKRRQYICSSQTWTDTVFRTFMNSPCQVYGRHNC

DEHDDDLRETRCENKGGQQLMEALKFDLECGWDNANCREAAFVVVMVCVAMVFWM

>ENSMUSP00000066777;Lrrc15

MPLKHYLLLLVSCQAWAAGLAYYGCPSECTCSRASQVECTGAQIVAMPSPLPWNAMSLQI

LNTHITELPEDKFLNISALIALKMEKNELANIMPGAFRNLGSLRHLSLANNKLKNLPVRL

FQDVNNLETLLLSNNQLVQIQPAQFSQFSNLKELQLYGNNLEYIPEGVFDHLVGLTKLNL

GNNGFTHLSPRVFQHLGNLQVLRLYENRLSDIPMGTFDALGNLQELALQENQIGTLSPGL

FHNNRNLQRLYLSNNHISHLPPGIFMQLPHLNKLTLFGNSLKELSPGVFGPMPNLRELWL

YNNHITSLPDNAFSHLNQLQVLILSHNQLSYISPGAFNGLTNLRELSLHTNALQDLDGNV

FRSLANLRNVSLQNNRLRQLPGSIFANVNGLMTIQLQNNNLENLPLGIFDHLGNLCELRL

YDNPWRCDSNILPLHDWLILNRARLGTDTLPVCSSPASVRGQSLVIINVNFPGPSVQGPE

TPEVSSYPDTSSYPDSTSISSTTEITRSTDDDYTDLNTIEPIDDRNTWGMTDAQSGLAIA

AIVIGIIALACSLAACICCCCCKKRSQAVLMQMKAPNEC

>CG8896-PA;18w

MPATSSIITIIAVAACLLLLVADAHAQQQCNWQYGLTTMDIRCSVRALESGTGTPLDLQV

AEAAGRLDLQCSQELLHASELAPGLFRQLQKLSELRIDACKLQRVPPNAFEGLMSLKRLT

LESHNAVWGPGKTLELHGQSFQGLKELSELHLGDNNIRQLPEGVWCSMPSLQLLNLTQNR

IRSAEFLGFSEKLCAGSALSNANGAVSGGSELQTLDVSFNELRSLPDAWGASRLRRLQTL

SLQHNNISTLAPNALAGLSSLRVLNISYNHLVSLPSEAFAGNKELRELHLQGNDLYELPK

GLLHRLEQLLVLDLSGNQLTSHHVDNSTFAGLIRLIVLNLSNNALTRIGSKTFKELYFLQ

ILDMRNNSIGHIEEGAFLPLYNLHTLNLAENRLHTLDNRIFNGLYVLTKLTLNNNLVSIV

ESQAFRNCSDLKELDLSSNQLTEVPEAVQDLSMLKTLDLGENQISEFKNNTFRNLNQLTG

LRLIDNRIGNITVGMFQDLPRLSVLNLAKNRIQSIERGAFDKNTEIEAIRLDKNFLTDIN

GIFATLASLLWLNLSENHLVWFDYAFIPSNLKWLDIHGNYIEALGNYYKLQEEIRVTTLD

ASHNRITEIGAMSVPNSIELLFINNNIIGQIQANTFVDKTRLARVDLYANVLSKISLNAL

RVAPVSAEKPVPEFYLGGNPFECDCSMEWLQRINNLTTRQHPHVVDLGNIECLMPHSRSA

PLRPLASLSASDFVCKYESHCPPTCHCCEYEQCECEVICPGNCSCFHDATWATNIVDCGR

QDLAALPNRIPQDVSDLYLDGNNMPELEVGHLTGRRNLRALYLNASNLMTLQNGSLAQLV

NLRVLHLENNKLTALEGTEFRSLGLLRELYLHNNMLTHISNATFEPLVSLEVLRLDNNRL

SSLPHLQYRHSLQGLTLGRNAWSCRCQQLRELAQFVSDNAMVVRDAHDIYCLDAGIKREL

ELIGNLANGPDCSDLLDASASNISSSQDLAGGYRLPLLAAVLVLIFLVVVLIIVFVFRES

VRMWLFAHYGVRVCEPRFEDAGKLYDAIILHSEKDYEFVCRNIAAELEHGRPPFRLCIQQ

RDLPPQASHLQLVEGARASRKIILVLTRNLLATEWNRIEFRNAFHESLRGLAQKLVIIEE

TSVSAEAEDVAELSPYLKSVPSNRLLTCDRYFWEKLRYAIPIELSPRGNNYTLDHHERFK

QPVSPGMIFRQAPPPPAYYCTEEMEANYSSATTATPSPRPTRPGGAARIVDSMPMPMRPP

SEHIYHSIESEYSAYDQHEALSMIPTGLMHQHQQQQLRLHQQQQQQQQQRLLQPQFRAMP

QQAIPAPSAPVHLRSGSGLSQASTSTQSTAQASTSAAAAQQQQQQQQQQAAGSEAANKNG

QAFLV

>C07F11.1;C07F11.1

MRRKMKLFLFLLLVINICRSAAANGDECPKFCKCAPDPVQPTSKLLLCDYSSKNTTITPI

ASSNYDQVANIRSLFISCDNNNFQFPDAYFKSLTALHHLRIVGCETTHFSVKLFEDLAAL

RRLELDQISTASTSFEMTEDVLMPLARLEKFSLTRSRNIELPQRLLCSLPHLQVLNISSN

ELPSLRREESCVAQQLLIVDLSRNRLTNIEQFLRGIPAIRQISVAYNSIAELDLSLATPF

LQQLDAEANRIVDLTSLPGTVVHVNLAGNALKRVPDAVAELASLVALNVSRNEIEAGNSS

VFSSPELEMLDASYNKLDSLPVEWLQKCEKRIAHLHLEHNSIEQLTGGVLANATNLQTLD

LSSNQLRVFRDEVLPENSKIGNLRLSNNSLELLEPSSLSGLKLESLDLSHNKLTEVPAAI

GKVEQLKKVDLSHNRIAKVYQYVLNKIKQLHTVDLSNNQLQSIGPYIFSDSSELHSLDVS

NNEISLLFKDAFARCPKLRKISMKMNKIKSLDEGLTEASGLRRLDVSHNEILVLKWSALP

ENLEILNADNNDINLLTAASMSPSTANLKSVSLSNNGITIMNADQIPNSLESLDVSNNRL

AKLGKTALAAKSQLRRLNLKGNLLTVVATESMKVVEAVHPLKVEISENPLICDCQMGWMI

GGAKPKVLIQDSETASCSHAVDGHQIQIQSLSKKDLLCPYKSVCEPECICCQYGNCDCKS

VCPANCRCFRDDQFNINIVRCHGNSSMVPKREFVVSELPVSATEIILSGVTLPQLRTHSF

IGRLRLQRLHINGTGLRSIQPKAFHTLPALKTLDLSDNSLISLSGEEFLKCGEVSQLFLN

GNRFSTLSRGIFEKLPNLKYLTLHNNSLEDIPQVLHSTALSKISLSSNPLRCDCSGGSQQ

HLHHRRDPKAHPFWEHNAAEWFSLHRHLVVDFPKVECWENVTKAFLTNDTTVLSAYPPNM

GNDVFVMPIEEFLRDYNSTICVPFSSGFFGQDPQNSILFVIITISIAVLLCVLVILAISF

IRKSHDAINQRRYKASSLNCSTSAGSSPLPVPLLSYHAFVSYSKKDEKMVIDQLCRPLED

EDYQLCLLHRDGPTYCSNLHAISDELIAQMDSSQCLILVLTKHFLENEWKTLQIKTSHQL

FAKNRAKRVIAVLGDGVDANLLDDELGQILRKHTRIEMRSHLFWTLLHSSLPSRLPLPSN

SGDDSSQLYSDIYGIVPSDVV

>C56E6.6;C56E6.6

MKLTQLDLSHNNLSVIPTWALTYLHSLQILHLENNRIDVLRSNTFDETQLNNLQFLYLDN

NQLRIIPNLAFNHLRLVVLMLANNRITEIQKMSLPQTLKFLVLRNNLLTQIPYVALNDLK

TLQSIDLEGNNITHLMDTNEVTFESEMKVILRNNKVRRLDKHSFRSFRKIRELDISYNQI

QTVEDSSFETVGHMQSLDLSYNRIAYLPRGMLKNFAKTLKTLKLAENMIHATPEALRDLR

NLTHLNLNGNKLNRIDGDVLKGCTDTLVELFIANNYLEHIPHGVLSGMKQLEHLDISKNK

IMSLKKPTSLLSITKEETSTVRRLNLAGNRINNMSDYLIFEHMPLLTYVDVSFNRIRFIS

PRVFEKLKNLESLFLQNNQLAHFPSLFRLDKLRHLMLDNNQIQKIDNFSLADLPKLQHLS

LAGNQLDIITENMFGSSSSSELKSLNLAHNKIHSISSRSFSDLDNLQQLRLSHNNIRTIT

SMTFSNLRNLRYLDLSHNRIIKILPSALYQLPALDVLHLDHNNLNEIDRDAFRSFSDLQS

LKLSHNAFRRFSCEFLGSISQVHQLDLSSNQINEIDIFCIARGIRKLSLASNSVEKINRK

LLQDATELTSIDISHNGIIDVDSDAFCECRKLSHIKLSHNYIRNLWKGTFQYQESLHTLD

ISFNDILFLHQGTFGKNNILQLHVNNNKLSRIPLEALSSTMASLHLLDLAHNNIKIVDSS

QLTSFGNLSILSFANNKVDSIEDGAFENLLSLKILDLSNNPVTSWSPTAFRDLSHSISSI

NMANTGLFSMPKFSHRSIQSLNISCNKIYELSEKDLAPLTKVVALDISHNNLKQISSMAF

EPLIHLKQLNVSANPITHLTNEHIQQLYKLYNIPDMARPYQISSILSNLPPLHTIYVDIK

ESALDRQFYTADTRLLRHLVVAGRNLTKIEVGAFATIRGFRVRIEIHNSSIEEFPSRIFD

TLTGISILSLSLTDNKLTTFNPFQSTVAPAVNQHGTILHSLELKNNPIKCDCQFKWMDDF

IRVTKFLSDHHISHDFDKVECADAQSSNLESFASAANELFSYKTKMTLLSKSEDFGLECA

VKYSNPLKPLIYVLMLVCLSYL

>CG7896-PA; CG7896-PA

MEAWKQLPNGGRFLLLLLACIAHLGHPILAWRPCPELSPALRLPCRCNVVPFAATGQLGA

VAMDCDRVVFHGDAPQLPYGAPIVAYTQRHSGQQVLPAQTFGQLKLTIEELDLSYNLIRR

IPEKAFDGLKDSLNELRLANNLLGDNLNPIFSTAELHVLKNLRLLDLSGNKIKLIEEGLL

KGCMDLKEFYIDRNSLTSVPTNSLNGPSALRHLSLRQNQIGSLLADSFNAQRQLEIIDLR

HNVIRSIDSLAFKGLQKIREIKLAGNRISHLNSDVFEKLQSLQKLDLSENFFGQFPTVAL

AAVPGLKHLNLSSNMLQQLDYTHMQVVRSLESLDISRNTITTITPGTFREMGALKYLDLS

LNSLRTIEDDALEGLDSLQTLIIKDNNILLVPGSALGRLPQLTSLQLDYNRVAALSAEIL

GSLQAGDITTLSLSRNVIRELPPGSFQMFSSLHTLDLSGNSLAVINADTFAGLESTLMAL

KLSQNRLTGLGGAPWVLPELRSLDLSGNTLTELPSTIFEELENVQSLNLSGNHLTPLTGA

LFKPLDRLQVIDLSGCNIRQISGDLLAGLQDLKHIYLNDNQLQELQDGSFVNLWNISSID

LSNNRIGSIRSGAFVNVMKLQKLDLHGNQLSAFKGEYFNTGTGIEELDISDNQLSYLFPS

SFRIHPRLREIRAANNKFSFFPAELISTLQYLEHIDLSHNQLKTIEELDFARLPRLRVLL

VANNQLDMVSEMAFHNSTQLQILDLAHNNLDRIGERTFEGLVRLEQLNLEGNRLSELSDG

VFERTKLQMLENINLAHNRFEYAPLNALQRQFFFVSSVDLSHNKIKELPGDDSIMVNIKR

IDLSFNPLSSKAVHNVLNEPKTVRELSLAGTGIENLELLETPFLQFLNLSHNKLKNVKPE

VFQRVTLLETLDLSSNQLESLEDLSMAWPQLQVLQSLDVSNNSFEIVSQSNFGKLEMLRS

LRLSHLPQCTRIEKNAFKQLPNLVSLEAYDLPLLGYLDLQGILELLPGLEVLDIEVKDSS

IGSEQIQPLKHPRLKSLGIRGDRLKSISSGTLAGLKSNDLSVQLRNTSLNALPPALLFPV

PRSSHLSLNVEGSKITVLVPQFLNALEDRRASLQLQGLASNPIVCDCNARALRRWLPSSG

MPDVTCASPAYLLNRKLIEVGDDELTCDARRMTSSTSRPTASVPHLLKTSSQLVTRSSST

TEEPLIIWSLEPTQPPSLKKMKTKAPLMKAQSPIISNDDTLIIGIVGGVVAFIAILIIII

CIIRLRMSNAEYQQNATMIGIPAGMQMGAHNAAYNYKNGAGAALYAVPPYHATLPHKAAS

IHQSSQNLSQRQQQQQQQQQVAAAAAAYSTMSRMSYFSGAGGGNGDGAESLTHQHPHQHQ

PYIIYSDDKAYR

>ENSMUSP00000036762;Tlr8

MENMPPQSWILTCFCLLSSGTSAIFHKANYSRSYPCDEIRHNSLVIAECNHRQLHEVPQT

IGKYVTNIDLSDNAITHITKESFQKLQNLTKIDLNHNAKQQHPNENKNGMNITEGALLSL

RNLTVLLLEDNQLYTIPAGLPESLKELSLIQNNIFQVTKNNTFGLRNLERLYLGWNCYFK

CNQTFKVEDGAFKNLIHLKVLSLSFNNLFYVPPKLPSSLRKLFLSNAKIMNITQEDFKGL

ENLTLLDLSGNCPRCYNAPFPCTPCKENSSIHIHPLAFQSLTQLLYLNLSSTSLRTIPST

WFENLSNLKELHLEFNYLVQEIASGAFLTKLPSLQILDLSFNFQYKEYLQFINISSNFSK

LRSLKKLHLRGYVFRELKKKHFEHLQSLPNLATINLGINFIEKIDFKAFQNFSKLDVIYL

SGNRIASVLDGTDYSSWRNRLRKPLSTDDDEFDPHVNFYHSTKPLIKPQCTAYGKALDLS

LNNIFIIGKSQFEGFQDIACLNLSFNANTQVFNGTEFSSMPHIKYLDLTNNRLDFDDNNA

FSDLHDLEVLDLSHNAHYFSIAGVTHRLGFIQNLINLRVLNLSHNGIYTLTEESELKSIS

LKELVFSGNRLDRLWNANDGKYWSIFKSLQNLIRLDLSYNNLQQIPNGAFLNLPQSLQEL

LISGNKLRFFNWTLLQYFPHLHLLDLSRNELYFLPNCLSKFAHSLETLLLSHNHFSHLPS

GFLSEARNLVHLDLSFNTIKMINKSSLQTKMKTNLSILELHGNYFDCTCDISDFRSWLDE

NLNITIPKLVNVICSNPGDQKSKSIMSLDLTTCVSDTTAAVLFFLTFLTTSMVMLAALVH

HLFYWDVWFIYHMCSAKLKGYRTSSTSQTFYDAYISYDTKDASVTDWVINELRYHLEESE

DKSVLLCLEERDWDPGLPIIDNLMQSINQSKKTIFVLTKKYAKSWNFKTAFYLALQRLMD

ENMDVIIFILLEPVLQYSQYLRLRQRICKSSILQWPNNPKAENLFWQSLKNVVLTENDSR

YDDLYIDSIRQY

>ENSMUSP00000043101;Tlr13

MSGLYRILVQLEQSPYVKTVPLNMRRDFFFLVVTWMPKTVKMNGSSFVPSLQLLLMLVGF

SLPPVAETYGFNKCTQYEFDIHHVLCIRKKITNLTEAISDIPRYTTHLNLTHNEIQVLPP

WSFTNLSALVDLRLEWNSIWKIDEGAFRGLENLTLLNLVENKIQSVNNSFEGLSSLKTLL

LSHNQITHIHKDAFTPLIKLKYLSLSRNNISDFSGILEAVQHLPCLERLDLTNNSIMYLD

HSPRSLVSLTHLSFEGNKLRELNFSALSLPNLTNLSASRNGNKVIQNVYLKTLPQLKSLN

LSGTVIKLENLSAKHLQNLRAMDLSNWELRHGHLDMKTVCHLLGNLPKLETLVFQKNVTN

AEGIKQLAKCTRLLFLDLGQNSDLIYLNDSEFNALPSLQKLNLNKCQLSFINNRTWSSLQ

NLTSLDLSHNKFKSFPDFAFSPLKHLEFLSLSRNPITELNNLAFSGLFALKELNLAACWI

VTIDRYSFTQFPNLEVLDLGDNNIRTLNHGTFRPLKKLQSLILSHNCLKILEPNSFSGLT

NLRSLDLMYNSLSYFHEHLFSGLEKLLILKLGFNKITYETTRTLQYPPFIKLKSLKQLNL

EGQRHGIQVVPSNFFQGLGSLQELLLGKNPSVFLDHHQFDPLINLTKLDISGTKDGDRSL

YLNASLFQNLKRLKILRLENNNLESLVPDMFSSLQSLQVFSLRFNNLKVINQSHLKNLKS

LMFFDVYGNKLQCTCDNLWFKNWSMNTEEVHIPFLRSYPCQQPGSQSLLIDFDDAMCNFD

LGKVYFLCSFSMVLSTMVFSWFSTKMIASLWYGLYICRAWYLTKWHKTEKKFLYDAFVSF

SATDEAWVYKELVPALEQGSQTTFKLCLHQRDFEPGIDIFENIQNAINTSRKTLCVVSNH

YLHSEWCRLEVQLASMKMFYEHKDVIILIFLEEIPNYKLSSYHRLRKLINKQTFITWPDS

VHQQPLFWARIRNALGKETVEKENTHLIVVE

>ENSMUSP00000061853;Tlr7

MVFSMWTRKRQILIFLNMLLVSRVFGFRWFPKTLPCEVKVNIPEAHVIVDCTDKHLTEIP

EGIPTNTTNLTLTINHIPSISPDSFRRLNHLEEIDLRCNCVPVLLGSKANVCTKRLQIRP

GSFSGLSDLKALYLDGNQLLEIPQDLPSSLHLLSLEANNIFSITKENLTELVNIETLYLG

QNCYYRNPCNVSYSIEKDAFLVMRNLKVLSLKDNNVTAVPTTLPPNLLELYLYNNIIKKI

QENDFNNLNELQVLDLSGNCPRCYNVPYPCTPCENNSPLQIHDNAFNSLTELKVLRLHSN

SLQHVPPTWFKNMRNLQELDLSQNYLAREIEEAKFLHFLPNLVELDFSFNYELQVYHASI

TLPHSLSSLENLKILRVKGYVFKELKNSSLSVLHKLPRLEVLDLGTNFIKIADLNIFKHF

ENLKLIDLSVNKISPSEESREVGFCPNAQTSVDRHGPQVLEALHYFRYDEYARSCRFKNK

EPPSFLPLNADCHIYGQTLDLSRNNIFFIKPSDFQHLSFLKCLNLSGNTIGQTLNGSELW

PLRELRYLDFSNNRLDLLYSTAFEELQSLEVLDLSSNSHYFQAEGITHMLNFTKKLRLLD

KLMMNDNDISTSASRTMESDSLRILEFRGNHLDVLWRAGDNRYLDFFKNLFNLEVLDISR

NSLNSLPPEVFEGMPPNLKNLSLAKNGLKSFFWDRLQLLKHLEILDLSHNQLTKVPERLA

NCSKSLTTLILKHNQIRQLTKYFLEDALQLRYLDISSNKIQVIQKTSFPENVLNNLEMLV

LHHNRFLCNCDAVWFVWWVNHTDVTIPYLATDVTCVGPGAHKGQSVISLDLYTCELDLTN

LILFSVSISSVLFLMVVMTTSHLFFWDMWYIYYFWKAKIKGYQHLQSMESCYDAFIVYDT

KNSAVTEWVLQELVAKLEDPREKHFNLCLEERDWLPGQPVLENLSQSIQLSKKTVFVMTQ

KYAKTESFKMAFYLSHQRLLDEKVDVIILIFLEKPLQKSKFLQLRKRLCRSSVLEWPANP

QAHPYFWQCLKNALTTDNHVAYSQMFKETV

>ENSMUSP00000082207;Tlr9

MVLRRRTLHPLSLLVQAAVLAETLALGTLPAFLPCELKPHGLVDCNWLFLKSVPRFSAAA

SCSNITRLSLISNRIHHLHNSDFVHLSNLRQLNLKWNCPPTGLSPLHFSCHMTIEPRTFL

AMRTLEELNLSYNGITTVPRLPSSLVNLSLSHTNILVLDANSLAGLYSLRVLFMDGNCYY

KNPCTGAVKVTPGALLGLSNLTHLSLKYNNLTKVPRQLPPSLEYLLVSYNLIVKLGPEDL

ANLTSLRVLDVGGNCRRCDHAPNPCIECGQKSLHLHPETFHHLSHLEGLVLKDSSLHTLN

SSWFQGLVNLSVLDLSENFLYESITHTNAFQNLTRLRKLNLSFNYRKKVSFARLHLASSF

KNLVSLQELNMNGIFFRLLNKYTLRWLADLPKLHTLHLQMNFINQAQLSIFGTFRALRFV

DLSDNRISGPSTLSEATPEEADDAEQEELLSADPHPAPLSTPASKNFMDRCKNFKFTMDL

SRNNLVTIKPEMFVNLSRLQCLSLSHNSIAQAVNGSQFLPLTNLQVLDLSHNKLDLYHWK

SFSELPQLQALDLSYNSQPFSMKGIGHNFSFVTHLSMLQSLSLAHNDIHTRVSSHLNSNS

VRFLDFSGNGMGRMWDEGGLYLHFFQGLSGLLKLDLSQNNLHILRPQNLDNLPKSLKLLS

LRDNYLSFFNWTSLSFLPNLEVLDLAGNQLKALTNGTLPNGTLLQKLDVSSNSIVSVVPA

FFALAVELKEVNLSHNILKTVDRSWFGPIVMNLTVLDVRSNPLHCACGAAFVDLLLEVQT

KVPGLANGVKCGSPGQLQGRSIFAQDLRLCLDEVLSWDCFGLSLLAVAVGMVVPILHHLC

GWDVWYCFHLCLAWLPLLARSRRSAQTLPYDAFVVFDKAQSAVADWVYNELRVRLEERRG

RRALRLCLEDRDWLPGQTLFENLWASIYGSRKTLFVLAHTDRVSGLLRTSFLLAQQRLLE

DRKDVVVLVILRPDAHRSRYVRLRQRLCRQSVLFWPQQPNGQGGFWAQLSTALTRDNRHF

YNQNFCRGPTAE

>ENSP00000311174;PTK9L

MLYSSCKSRLLDSVEQDFHLEIAKKGFCRSALHPLSLLVQAIMLAMTLALGTLPAFLPCE

LQPHGLVNCNWLFLKSVPHFSMAAPRGNVTSLSLSSNRIHHLHDSDFAHLPSLRHLNLKW

NCPPVGLSPMHFPCHMTIEPSTFLAVPTLEELNLSYNNIMTVPALPKSLISLSLSHTNIL

MLDSASLAGLHALRFLFMDGNCYYKNPCRQALEVAPGALLGLGNLTHLSLKYNNLTVVPR

NLPSSLEYLLLSYNRIVKLAPEDLANLTALRVLDVGGNCRRCDHAPNPCMECPRHFPQLH

PDTFSHLSRLEGLVLKDSSLSWLNASWFRGLGNLRVLDLSENFLYKCITKTKAFQGLTQL

RKLNLSFNYQKRVSFAHLSLAPSFGSLVALKELDMHGIFFRSLDETTLRPLARLPMLQTL

RLQMNFINQAQLGIFRAFPGLRYVDLSDNRISGASELTATMGEADGGEKVWLQPGDLAPA

PVDTPSSEDFRPNCSTLNFTLDLSRNNLVTVQPEMFAQLSHLQCLRLSHNCISQAVNGSQ

FLPLTGLQVLDLSHNKLDLYHEHSFTELPRLEALDLSYNSQPFGMQGVGHNFSFVAHLRT

LRHLSLAHNNIHSQVSQQLCSTSLRALDFSGNALGHMWAEGDLYLHFFQGLSGLIWLDLS

QNRLHTLLPQTLRNLPKSLQVLRLRDNYLAFFKWWSLHFLPKLEVLDLAGNQLKALTNGS

LPAGTRLRRLDVSCNSISFVAPGFFSKAKELRELNLSANALKTVDHSWFGPLASALQILD

VSANPLHCACGAAFMDFLLEVQAAVPGLPSRVKCGSPGQLQGLSIFAQDLRLCLDEALSW

DCFALSLLAVALGLGVPMLHHLCGWDLWYCFHLCLAWLPWRGRQSGRDEDALPYDAFVVF

DKTQSAVADWVYNELRGQLEECRGRWALRLCLEERDWLPGKTLFENLWASVYGSRKTLFV

LAHTDRVSGLLRASFLLAQQRLLEDRKDVVVLVILSPDGRRSRYVRLRQRLCRQSVLLWP

HQPSGQRSFWAQLGMALTRDNHHFYNRNFCQGPTAE

>ENSP00000312082;TLR8

MKESSLQNSSCSLGKETKKENMFLQSSMLTCIFLLISGSCELCAEENFSRSYPCDEKKQN

DSVIAECSNRRLQEVPQTVGKYVTELDLSDNFITHITNESFQGLQNLTKINLNHNPNVQH

QNGNPGIQSNGLNITDGAFLNLKNLRELLLEDNQLPQIPSGLPESLTELSLIQNNIYNIT

KEGISRLINLKNLYLAWNCYFNKVCEKTNIEDGVFETLTNLELLSLSFNSLSHVPPKLPS

SLRKLFLSNTQIKYISEEDFKGLINLTLLDLSGNCPRCFNAPFPCVPCDGGASINIDRFA

FQNLTQLRYLNLSSTSLRKINAAWFKNMPHLKVLDLEFNYLVGEIASGAFLTMLPRLEIL

DLSFNYIKGSYPQHINISRNFSKLLSLRALHLRGYVFQELREDDFQPLMQLPNLSTINLG

INFIKQIDFKLFQNFSNLEIIYLSENRISPLVKDTRQSYANSSSFQRHIRKRRSTDFEFD

PHSNFYHFTRPLIKPQCAAYGKALDLSLNSIFFIGPNQFENLPDIACLNLSANSNAQVLS

GTEFSAIPHVKYLDLTNNRLDFDNASALTELSDLEVLDLSYNSHYFRIAGVTHHLEFIQN

FTNLKVLNLSHNNIYTLTDKYNLESKSLVELVFSGNRLDILWNDDDNRYISIFKGLKNLT

RLDLSLNRLKHIPNEAFLNLPASLTELHINDNMLKFFNWTLLQQFPRLELLDLRGNKLLF

LTDSLSDFTSSLRTLLLSHNRISHLPSGFLSEVSSLKHLDLSSNLLKTINKSALETKTTT

KLSMLELHGNPFECTCDIGDFRRWMDEHLNVKIPRLVDVICASPGDQRGKSIVSLELTTC

VSDVTAVILFFFTFFITTMVMLAALAHHLFYWDVWFIYNVCLAKVKGYRSLSTSQTFYDA

YISYDTKDASVTDWVINELRYHLEESRDKNVLLCLEERDWDPGLAIIDNLMQSINQSKKT

VFVLTKKYAKSWNFKTAFYLALQRLMDENMDVIIFILLEPVLQHSQYLRLRQRICKSSIL

QWPDNPKAEGLFWQTLRNVVLTENDSRYNNMYVDSIKQY

>ENSP00000349736;TLR7

MVFPMWTLKRQILILFNIILISKLLGARWFPKTLPCDVTLDVPKNHVIVDCTDKHLTEIP

GGIPTNTTNLTLTINHIPDISPASFHRLDHLVEIDFRCNCVPIPLGSKNNMCIKRLQIKP

RSFSGLTYLKSLYLDGNQLLEIPQGLPPSLQLLSLEANNIFSIRKENLTELANIEILYLG

QNCYYRNPCYVSYSIEKDAFLNLTKLKVLSLKDNNVTAVPTVLPSTLTELYLYNNMIAKI

QEDDFNNLNQLQILDLSGNCPRCYNAPFPCAPCKNNSPLQIPVNAFDALTELKVLRLHSN

SLQHVPPRWFKNINKLQELDLSQNFLAKEIGDAKFLHFLPSLIQLDLSFNFELQVYRASM

NLSQAFSSLKSLKILRIRGYVFKELKSFNLSPLHNLQNLEVLDLGTNFIKIANLSMFKQF

KRLKVIDLSVNKISPSGDSSEVGFCSNARTSVESYEPQVLEQLHYFRYDKYARSCRFKNK

EASFMSVNESCYKYGQTLDLSKNSIFFVKSSDFQHLSFLKCLNLSGNLISQTLNGSEFQP

LAELRYLDFSNNRLDLLHSTAFEELHKLEVLDISSNSHYFQSEGITHMLNFTKNLKVLQK

LMMNDNDISSSTSRTMESESLRTLEFRGNHLDVLWREGDNRYLQLFKNLLKLEELDISKN

SLSFLPSGVFDGMPPNLKNLSLAKNGLKSFSWKKLQCLKNLETLDLSHNQLTTVPERLSN

CSRSLKNLILKNNQIRSLTKYFLQDAFQLRYLDLSSNKIQMIQKTSFPENVLNNLKMLLL

HHNRFLCTCDAVWFVWWVNHTEVTIPYLATDVTCVGPGAHKGQSVISLDLYTCELDLTNL

ILFSLSISVSLFLMVMMTASHLYFWDVWYIYHFCKAKIKGYQRLISPDCCYDAFIVYDTK

DPAVTEWVLAELVAKLEDPREKHFNLCLEERDWLPGQPVLENLSQSIQLSKKTVFVMTDK

YAKTENFKIAFYLSHQRLMDEKVDVIILIFLEKPFQKSKFLQLRKRLCGSSVLEWPTNPQ

AHPYFWQCLKNALATDNHVAYSQVFKETV

>ENSMUSP00000057529;Tlr5

MACQLDLLIGVIFMASPVLVISPCSSDGRIAFFRGCNLTQIPWILNTTTERLLLSFNYIS

MVVATSFPLLERLQLLELGTQYANLTIGPGAFRNLPNLRILDLGQSQIEVLNRDAFQGLP

HLLELRLFSCGLSSAVLSDGYFRNLYSLARLDLSGNQIHSLRLHSSFRELNSLSDVNFAF

NQIFTICEDELEPLQGKTLSFFGLKLTKLFSRVSVGWETCRNPFRGVRLETLDLSENGWT

VDITRNFSNIIQGSQISSLILKHHIMGPGFGFQNIRDPDQSTFASLARSSVLQLDLSHGF

IFSLNPRLFGTLKDLKMLNLAFNKINKIGENAFYGLDSLQVLNLSYNLLGELYNSNFYGL

PRVAYVDLQRNHIGIIQDQTFRLLKTLQTLDLRDNALKAIGFIPSIQMVLLGGNKLVHLP

HIHFTANFLELSENRLENLSDLYFLLRVPQLQFLILNQNRLSSCKAAHTPSENPSLEQLF

LTENMLQLAWETGLCWDVFQGLSRLQILYLSNNYLNFLPPGIFNDLVALRMLSLSANKLT

VLSPGSLPANLEILDISRNQLFSPDPALFSSLRVLDITHNEFVCNCELSTFISWLNQTNV

TLFGSPADVYCMYPNSLLGGSLYNISTEDCDEEEAMRSLKFSLFILCTVTLTLFLVITLV

VIKFRGICFLCYKTIQKLVFKDKVWSLEPGAYRYDAYFCFSSKDFEWAQNALLKHLDAHY

SSRNRLRLCFEERDFIPGENHISNIQAAVWGSRKTVCLVSRHFLKDGWCLEAFRYAQSRS

LSDLKSILIVVVVGSLSQYQLMRHETIRGFLQKQQYLRWPEDLQDVGWFLDKLSGCILKE

EKGKKRSSSIQLRTIATIS

>ENSP00000340089;TLR5

MGDHLDLLLGVVLMAGPVFGIPSCSFDGRIAFYRFCNLTQVPQVLNTTERLLLSFNYIRT

VTASSFPFLEQLQLLELGSQYTPLTIDKEAFRNLPNLRILDLGSSKIYFLHPDAFQGLFH

LFELRLYFCGLSDAVLKDGYFRNLKALTRLDLSKNQIRSLYLHPSFGKLNSLKSIDFSSN

QIFLVCEHELEPLQGKTLSFFSLAANSLYSRVSVDWGKCMNPFRNMVLEILDVSGNGWTV

DITGNFSNAISKSQAFSLILAHHIMGAGFGFHNIKDPDQNTFAGLARSSVRHLDLSHGFV

FSLNSRVFETLKDLKVLNLAYNKINKIADEAFYGLDNLQVLNLSYNLLGELYSSNFYGLP

KVAYIDLQKNHIAIIQDQTFKFLEKLQTLDLRDNALTTIHFIPSIPDIFLSGNKLVTLPK

INLTANLIHLSENRLENLDILYFLLRVPHLQILILNQNRFSSCSGDQTPSENPSLEQLFL

GENMLQLAWETELCWDVFEGLSHLQVLYLNHNYLNSLPPGVFSHLTALRGLSLNSNRLTV

LSHNDLPANLEILDISRNQLLAPNPDVFVSLSVLDITHNKFICECELSTFINWLNHTNVT

IAGPPADIYCVYPDSFSGVSLFSLSTEGCDEEEVLKSLKFSLFIVCTVTLTLFLMTILTV

TKFRGFCFICYKTAQRLVFKDHPQGTEPDMYKYDAYLCFSSKDFTWVQNALLKHLDTQYS

DQNRFNLCFEERDFVPGENRIANIQDAIWNSRKIVCLVSRHFLRDGWCLEAFSYAQGRCL

SDLNSALIMVVVGSLSQYQLMKHQSIRGFVQKQQYLRWPEDFQDVGWFLHKLSQQILKKE

KEKKKDNNIPLQTVATIS

>ENSMUSP00000034056;Tlr3

MKGCSSYLMYSFGGLLSLWILLVSSTNQCTVRYNVADCSHLKLTHIPDDLPSNITVLNLT

HNQLRRLPPTNFTRYSQLAILDAGFNSISKLEPELCQILPLLKVLNLQHNELSQISDQTF

VFCTNLTELDLMSNSIHKIKSNPFKNQKNLIKLDLSHNGLSSTKLGTGVQLENLQELLLA

KNKILALRSEELEFLGNSSLRKLDLSSNPLKEFSPGCFQTIGKLFALLLNNAQLNPHLTE

KLCWELSNTSIQNLSLANNQLLATSESTFSGLKWTNLTQLDLSYNNLHDVGNGSFSYLPS

LRYLSLEYNNIQRLSPRSFYGLSNLRYLSLKRAFTKQSVSLASHPNIDDFSFQWLKYLEY

LNMDDNNIPSTKSNTFTGLVSLKYLSLSKTFTSLQTLTNETFVSLAHSPLLTLNLTKNHI

SKIANGTFSWLGQLRILDLGLNEIEQKLSGQEWRGLRNIFEIYLSYNKYLQLSTSSFALV

PSLQRLMLRRVALKNVDISPSPFRPLRNLTILDLSNNNIANINEDLLEGLENLEILDFQH

NNLARLWKRANPGGPVNFLKGLSHLHILNLESNGLDEIPVGVFKNLFELKSINLGLNNLN

KLEPFIFDDQTSLRSLNLQKNLITSVEKDVFGPPFQNLNSLDMRFNPFDCTCESISWFVN

WINQTHTNISELSTHYLCNTPHHYYGFPLKLFDTSSCKDSAPFELLFIISTSMLLVFILV

VLLIHIEGWRISFYWNVSVHRILGFKEIDTQAEQFEYTAYIIHAHKDRDWVWEHFSPMEE

QDQSLKFCLEERDFEAGVLGLEAIVNSIKRSRKIIFVITHHLLKDPLCRRFKVHHAVQQA

IEQNLDSIILIFLQNIPDYKLNHALCLRRGMFKSHCILNWPVQKERINAFHHKLQVALGS

RNSAH

>ENSP00000296795;TLR3

MRQTLPCIYFWGGLLPFGMLCASSTTKCTVSHEVADCSHLKLTQVPDDLPTNITVLNLTH

NQLRRLPAANFTRYSQLTSLDVGFNTISKLEPELCQKLPMLKVLNLQHNELSQLSDKTFA

FCTNLTELHLMSNSIQKIKNNPFVKQKNLITLDLSHNGLSSTKLGTQVQLENLQELLLSN

NKIQALKSEELDIFANSSLKKLELSSNQIKEFSPGCFHAIGRLFGLFLNNVQLGPSLTEK

LCLELANTSIRNLSLSNSQLSTTSNTTFLGLKWTNLTMLDLSYNNLNVVGNDSFAWLPQL

EYFFLEYNNIQHLFSHSLHGLFNVRYLNLKRSFTKQSISLASLPKIDDFSFQWLKCLEHL

NMEDNDIPGIKSNMFTGLINLKYLSLSNSFTSLRTLTNETFVSLAHSPLHILNLTKNKIS

KIESDAFSWLGHLEVLDLGLNEIGQELTGQEWRGLENIFEIYLSYNKYLQLTRNSFALVP

SLQRLMLRRVALKNVDSSPSPFQPLRNLTILDLSNNNIANINDDMLEGLEKLEILDLQHN

NLARLWKHANPGGPIYFLKGLSHLHILNLESNGFDEIPVEVFKDLFELKIIDLGLNNLNT

LPASVFNNQVSLKSLNLQKNLITSVEKKVFGPAFRNLTELDMRFNPFDCTCESIAWFVNW

INETHTNIPELSSHYLCNTPPHYHGFPVRLFDTSSCKDSAPFELFFMINTSILLIFIFIV

LLIHFEGWRISFYWNVSVHRVLGFKEIDRQTEQFEYAAYIIHAYKDKDWVWEHFSSMEKE

DQSLKFCLEERDFEAGVFELEAIVNSIKRSRKIIFVITHHLLKDPLCKRFKVHHAVQQAI

EQNLDSIILVFLEEIPDYKLNHALCLRRGMFKSHCILNWPVQKERIGAFRHKLQVALGSK

NSVH

>ENSMUSP00000029623;Tlr2

MLRALWLFWILVAITVLFSKRCSAQESLSCDASGVCDGRSRSFTSIPSGLTAAMKSLDLS

FNKITYIGHGDLRACANLQVLMLKSSRINTIEGDAFYSLGSLEHLDLSDNHLSSLSSSWF

GPLSSLKYLNLMGNPYQTLGVTSLFPNLTNLQTLRIGNVETFSEIRRIDFAGLTSLNELE

IKALSLRNYQSQSLKSIRDIHHLTLHLSESAFLLEIFADILSSVRYLELRDTNLARFQFS

PLPVDEVSSPMKKLAFRGSVLTDESFNELLKLLRYILELSEVEFDDCTLNGLGDFNPSES

DVVSELGKVETVTIRRLHIPQFYLFYDLSTVYSLLEKVKRITVENSKVFLVPCSFSQHLK

SLEFLDLSENLMVEEYLKNSACKGAWPSLQTLVLSQNHLRSMQKTGEILLTLKNLTSLDI

SRNTFHPMPDSCQWPEKMRFLNLSSTGIRVVKTCIPQTLEVLDVSNNNLDSFSLFLPRLQ

ELYISRNKLKTLPDASLFPVLLVMKIRENAVSTFSKDQLGSFPKLETLEAGDNHFVCSCE

LLSFTMETPALAQILVDWPDSYLCDSPPRLHGHRLQDARPSVLECHQAALVSGVCCALLL

LILLVGALCHHFHGLWYLRMMWAWLQAKRKPKKAPCRDVCYDAFVSYSEQDSHWVENLMV

QQLENSDPPFKLCLHKRDFVPGKWIIDNIIDSIEKSHKTVFVLSENFVRSEWCKYELDFS

HFRLFDENNDAAILVLLEPIERKAIPQRFCKLRKIMNTKTYLEWPLDEGQQEVFWVNLRT

AIKS

>ENSMUSP00000060793;Tlr1

MTKPNSLIFYCIIVLGLTLMKIQLSEECELIIKRPNANLTRVPKDLPLQTTTLDLSQNNI

SELQTSDILSLSKLRVLIMSYNRLQYLNISVFKFNTELEYLDLSHNELKVILCHPTVSLK

HLDLSFNAFDALPICKEFGNMSQLQFLGLSGSRVQSSSVQLIAHLNISKVLLVLGDAYGE

KEDPESLRHVSTETLHIVFPSKREFRFLLDVSVSTTIGLELSNIKCVLEDQGCSYFLRAL

SKLGKNLKLSNLTLNNVETTWNSFINILQIVWHTPVKYFSISNVKLQGQLAFRMFNYSDT

SLKALSIHQVVTDVFSFPQSYIYSIFANMNIQNFTMSGTHMVHMLCPSQVSPFLHVDFTD

NLLTDMVFKDCRNLVRLKTLSLQKNQLKNLENIILTSAKMTSLQKLDISQNSLRYSDGGI

PCAWTQSLLVLNLSSNMLTGSVFRCLPPKVKVLDLHNNRIMSIPKDVTHLQALQELNVAS

NSLTDLPGCGAFSSLSVLVIDHNSVSHPSEDFFQSCQNIRSLTAGNNPFQCTCELRDFVK

NIGWVAREVVEGWPDSYRCDYPESSRGTALRDFHMSPLSCDTVLLTVTIGATMLVLAVTG

AFLCLYFDLPWYVRMLCQWTQTRHRARHIPLEELQRNLQFHAFVSYSGHDSAWVKNELLP

NLEKDDIQICLHERNFVPGKSIVENIINFIEKSYKSIFVLSPHFIQSEWCHYELYFAHHN

LFHEGSDNLILILLAPIPQYSIPTNYHKLKTLMSRRTYLEWPTEKNKHGLFWANLRASIN

VKLVNQAEGTCYTQQ

>ENSMUSP00000062096;Tlr6

MVKSLWDSLCNMSQDRKPIVGSFHFVCALALIVGSMTPFSNELESMVDYSNRNLTHVPKD

LPPRTKALSLSQNSISELRMPDISFLSELRVLRLSHNRIRSLDFHVFLFNQDLEYLDVSH

NRLQNISCCPMASLRHLDLSFNDFDVLPVCKEFGNLTKLTFLGLSAAKFRQLDLLPVAHL

HLSCILLDLVSYHIKGGETESLQIPNTTVLHLVFHPNSLFSVQVNMSVNALGHLQLSNIK

LNDENCQRLMTFLSELTRGPTLLNVTLQHIETTWKCSVKLFQFFWPRPVEYLNIYNLTIT

ERIDREEFTYSETALKSLMIEHVKNQVFLFSKEALYSVFAEMNIKMLSISDTPFIHMVCP

PSPSSFTFLNFTQNVFTDSVFQGCSTLKRLQTLILQRNGLKNFFKVALMTKNMSSLETLD

VSLNSLNSHAYDRTCAWAESILVLNLSSNMLTGSVFRCLPPKVKVLDLHNNRIMSIPKDV

THLQALQELNVASNSLTDLPGCGAFSSLSVLVIDHNSVSHPSEDFFQSCQNIRSLTAGNN

PFQCTCELRDFVKNIGWVAREVVEGWPDSYRCDYPESSKGTALRDFHMSPLSCDTVLLTV

TIGATMLVLAVTGAFLCLYFDLPWYVRMLCQWTQTRHRARHIPLEELQRNLQFHAFVSYS

EHDSAWVKNELLPNLEKDDIRVCLHERNFVPGKSIVENIINFIEKSYKAIFVLSPHFIQS

EWCHYELYFAHHNLFHEGSDNLILILLEPILQNNIPSRYHKLRALMAQRTYLEWPTEKGK

RGLFWANLRASFIMKLALVNEDDVKT

>ENSP00000260010;TLR2

MPHTLWMVWVLGVIISLSKEESSNQASLSCDRNGICKGSSGSLNSIPSGLTEAVKSLDLS

NNRITYISNSDLQRCVNLQALVLTSNGINTIEEDSFSSLGSLEHLDLSYNYLSNLSSSWF

KPLSSLTFLNLLGNPYKTLGETSLFSHLTKLQILRVGNMDTFTKIQRKDFAGLTFLEELE

IDASDLQSYEPKSLKSIQNVSHLILHMKQHILLLEIFVDVTSSVECLELRDTDLDTFHFS

ELSTGETNSLIKKFTFRNVKITDESLFQVMKLLNQISGLLELEFDDCTLNGVGNFRASDN

DRVIDPGKVETLTIRRLHIPRFYLFYDLSTLYSLTERVKRITVENSKVFLVPCLLSQHLK

SLEYLDLSENLMVEEYLKNSACEDAWPSLQTLILRQNHLASLEKTGETLLTLKNLTNIDI

SKNSFHSMPETCQWPEKMKYLNLSSTRIHSVTGCIPKTLEILDVSNNNLNLFSLNLPQLK

ELYISRNKLMTLPDASLLPMLLVLKISRNAITTFSKEQLDSFHTLKTLEAGGNNFICSCE

FLSFTQEQQALAKVLIDWPANYLCDSPSHVRGQQVQDVRLSVSECHRTALVSGMCCALFL

LILLTGVLCHRFHGLWYMKMMWAWLQAKRKPRKAPSRNICYDAFVSYSERDAYWVENLMV

QELENFNPPFKLCLHKRDFIPGKWIIDNIIDSIEKSHKTVFVLSENFVKSEWCKYELDFS

HFRLFDENNDAAILILLEPIEKKAIPQRFCKLRKIMNTKTYLEWPMDEAQREGFWVNLRA

AIKS

>ENSP00000309253;TLR6

MTKDKEPIVKSFHFVCLMIIIVGTRIQFSDGNEFAVDKSKRGLIHVPKDLPLKTKVLDMS

QNYIAELQVSDMSFLSELTVLRLSHNRIQLLDLSVFKFNQDLEYLDLSHNQLQKISCHPI

VSFRHLDLSFNDFKALPICKEFGNLSQLNFLGLSAMKLQKLDLLPIAHLHLSYILLDLRN

YYIKENETESLQILNAKTLHLVFHPTSLFAIQVNISVNTLGCLQLTNIKLNDDNCQVFIK

FLSELTRGSTLLNFTLNHIETTWKCLVRVFQFLWPKPVEYLNIYNLTIIESIREEDFTYS

KTTLKALTIEHITNQVFLFSQTALYTVFSEMNIMMLTISDTPFIHMLCPHAPSTFKFLNF

TQNVFTDSIFEKCSTLVKLETLILQKNGLKDLFKVGLMTKDMPSLEILDVSWNSLESGRH

KENCTWVESIVVLNLSSNMLTDSVFRCLPPRIKVLDLHSNKIKSVPKQVVKLEALQELNV

AFNSLTDLPGCGSFSSLSVLIIDHNSVSHPSADFFQSCQKMRSIKAGDNPFQCTCELREF

VKNIDQVSSEVLEGWPDSYKCDYPESYRGSPLKDFHMSELSCNITLLIVTIGATMLVLAV

TVTSLCIYLDLPWYLRMVCQWTQTRRRARNIPLEELQRNLQFHAFISYSEHDSAWVKSEL

VPYLEKEDIQICLHERNFVPGKSIVENIINCIEKSYKSIFVLSPNFVQSEWCHYELYFAH

HNLFHEGSNNLILILLEPIPQNSIPNKYHKLKALMTQRTYLQWPKEKSKRGLFWANIRAA

FNMKLTLVTENNDVKS

>ENSP00000354459;TLR10

MRLIRNIYIFCSIVMTAEGDAPELPEERELMTNCSNMSLRKVPADLTPATTTLDLSYNLL

FQLQSSDFHSVSKLRVLILCHNRIQQLDLKTFEFNKELRYLDLSNNRLKSVTWYLLAGLR

YLDLSFNDFDTMPICEEAGNMSHLEILGLSGAKIQKSDFQKIAHLHLNTVFLGFRTLPHY

EEGSLPILNTTKLHIVLPMDTNFWVLLRDGIKTSKILEMTNIDGKSQFVSYEMQRNLSLE

NAKTSVLLLNKVDLLWDDLFLILQFVWHTSVEHFQIRNVTFGGKAYLDHNSFDYSNTVMR

TIKLEHVHFRVFYIQQDKIYLLLTKMDIENLTISNAQMPHMLFPNYPTKFQYLNFANNIL

TDELFKRTIQLPHLKTLILNGNKLETLSLVSCFANNTPLEHLDLSQNLLQHKNDENCSWP

ETVVNMNLSYNKLSDSVFRCLPKSIQILDLNNNQIQTVPKETIHLMALRELNIAFNFLTD

LPGCSHFSRLSVLNIEMNFILSPSLDFVQSCQEVKTLNAGRNPFRCTCELKNFIQLETYS

EVMMVGWSDSYTCEYPLNLRGTRLKDVHLHELSCNTALLIVTIVVIMLVLGLAVAFCCLH

FDLPWYLRMLGQCTQTWHRVRKTTQEQLKRNVRFHAFISYSEHDSLWVKNELIPNLEKED

GSILICLYESYFDPGKSISENIVSFIEKSYKSIFVLSPNFVQNEWCHYEFYFAHHNLFHE

NSDHIILILLEPIPFYCIPTRYHKLKALLEKKAYLEWPKDRRKCGLFWANLRAAINVNVL

ATREMYELQTFTELNEESRGSTISLMRTDCL

>ENSP00000354932;TLR1

MTSIFHFAIIFMLILQIRIQLSEESEFLVDRSKNGLIHVPKDLSQKTTILNISQNYISEL

WTSDILSLSKLRILIISHNRIQYLDISVFKFNQELEYLDLSHNKLVKISCHPTVNLKHLD

LSFNAFDALPICKEFGNMSQLKFLGLSTTHLEKSSVLPIAHLNISKVLLVLGETYGEKED

PEGLQDFNTESLHIVFPTNKEFHFILDVSVKTVANLELSNIKCVLEDNKCSYFLSILAKL

QTNPKLSNLTLNNIETTWNSFIRILQLVWHTTVWYFSISNVKLQGQLDFRDFDYSGTSLK

ALSIHQVVSDVFGFPQSYIYEIFSNMNIKNFTVSGTRMVHMLCPSKISPFLHLDFSNNLL

TDTVFENCGHLTELETLILQMNQLKELSKIAEMTTQMKSLQQLDISQNSVSYDEKKGDCS

WTKSLLSLNMSSNILTDTIFRCLPPRIKVLDLHSNKIKSIPKQVVKLEALQELNVAFNSL

TDLPGCGSFSSLSVLIIDHNSVSHPSADFFQSCQKMRSIKAGDNPFQCTCELGEFVKNID

QVSSEVLEGWPDSYKCDYPESYRGTLLKDFHMSELSCNITLLIVTIVATMLVLAVTVTSL

CSYLDLPWYLRMVCQWTQTRRRARNIPLEELQRNLQFHAFISYSGHDSFWVKNELLPNLE

KEGMQICLHERNFVPGKSIVENIITCIEKSYKSIFVLSPNFVQSEWCHYELYFAHHNLFH

EGSNSLILILLEPIPQYSIPSSYHKLKSLMARRTYLEWPKEKSKRGLFWANLRAAINIKL

TEQAKK

>CG5528-PA;Toll-9

MCPKYIWDVIVLVCLFLGNVREAYTEFSIQDGLIIEPDSATTSSEEAEEVSKERTDLKSL

MLKYESDDGNSCLLDLIKDEVIWWQFPNGTLRDSTKKYAHKLYLDLSHGNLKDDSDLFRE

AKLSRKVTIWRTEVFSAAFNTLTAAPFRTLYSMRESLKLLSLRGNNFAELIPDAEDFARF

VNESRLEASNSVPHHCELLLLHNTTDLYDRECYLYFNNNTNMGQSITTGRNYTNFIKVLK

DRFDQHGSSSQSIAWATFPKMPRLVELDISNCSIEYVSKEAFRNVSNLRRLFMSDNKIMT

ISHDTFYYVQGVQYLDLSFTNFLTYSYQLQLPTLEMALSLIYGLKIQQNVFKYLPELIYL

DLSHSKMTRNSAVAFAHLGDKLKFLSLCYTAIPMVSSTIFKNTVLEGLDLSGNPYLSYNI

IDDAFDGIANTLKYLYFERSNIKDLEWSKSLKNLQVLGLAGNNINALTPAMFQSLESLEI

LDLSSNHVGNWYRSAFHNNSALRVLNLRSNTINMLSNEMLKDFERLDYLSLGDNDFICDC

HLRAVVEVAAANNKDADCSYRLLNYSQNAVGEEVISLAESLIIDRKLWQSRYIPWLQRSY

SNIREFNRANHIIKLRFSSEDYMVAKCSAAQPYHLGDLDGDLTLKFQLLDYEASQYYCFN

NTDQLQVDELNCQIRSMSDLAEELHHVTNTVIAVMGSLVGACILGFIIYLKRWHIHYYYS

SLKSAALLSSASKESVNKFTNISQRDPSAVYDIFISYCQNDRTWVLNELLPNVEETGDVS

ICLHERDFQIGVTILDNIISCMDRSYSLMLIISSKFLLSHWCQFEMYLAQHRIFEVSKEH

LILVFLEDIPRRKRPKTLQYLMDVKTYIKWPTAKEDRKLFWKRLKRSLEVIGINSREISV

>ENSMUSP00000045770;Tlr4

MMPPWLLARTLIMALFFSCLTPGSLNPCIEVVPNITYQCMDQKLSKVPDDIPSSTKNIDL

SFNPLKILKSYSFSNFSELQWLDLSRCEIETIEDKAWHGLHHLSNLILTGNPIQSFSPGS

FSGLTSLENLVAVETKLASLESFPIGQLITLKKLNVAHNFIHSCKLPAYFSNLTNLVHVD

LSYNYIQTITVNDLQFLRENPQVNLSLDMSLNPIDFIQDQAFQGIKLHELTLRGNFNSSN

IMKTCLQNLAGLHVHRLILGEFKDERNLEIFEPSIMEGLCDVTIDEFRLTYTNDFSDDIV

KFHCLANVSAMSLAGVSIKYLEDVPKHFKWQSLSIIRCQLKQFPTLDLPFLKSLTLTMNK

GSISFKKVALPSLSYLDLSRNALSFSGCCSYSDLGTNSLRHLDLSFNGAIIMSANFMGLE

ELQHLDFQHSTLKRVTEFSAFLSLEKLLYLDISYTNTKIDFDGIFLGLTSLNTLKMAGNS

FKDNTLSNVFANTTNLTFLDLSKCQLEQISWGVFDTLHRLQLLNMSHNNLLFLDSSHYNQ

LYSLSTLDCSFNRIETSKGILQHFPKSLAFFNLTNNSVACICEHQKFLQWVKEQKQFLVN

VEQMTCATPVEMNTSLVLDFNNSTCYMYKTIISVSVVSVIVVSTVAFLIYHFYFHLILIA

GCKKYSRGESIYDAFVIYSSQNEDWVRNELVKNLEEGVPRFHLCLHYRDFIPGVAIAANI

IQEGFHKSRKVIVVVSRHFIQSRWCIFEYEIAQTWQFLSSRSGIIFIVLEKVEKSLLRQQ

VELYRLLSRNTYLEWEDNPLGRHIFWRRLKNALLDGKASNPEQTAEEEQETATWT

>ENSP00000346893;TLR4

MMSASRLAGTLIPAMAFLSCVRPESWEPCVEVVPNITYQCMELNFYKIPDNLPFSTKNLD

LSFNPLRHLGSYSFFSFPELQVLDLSRCEIQTIEDGAYQSLSHLSTLILTGNPIQSLALG

AFSGLSSLQKLVAVETNLASLENFPIGHLKTLKELNVAHNLIQSFKLPEYFSNLTNLEHL

DLSSNKIQSIYCTDLRVLHQMPLLNLSLDLSLNPMNFIQPGAFKEIRLHKLTLRNNFDSL

NVMKTCIQGLAGLEVHRLVLGEFRNEGNLEKFDKSALEGLCNLTIEEFRLAYLDYYLDDI

IDLFNCLTNVSSFSLVSVTIERVKDFSYNFGWQHLELVNCKFGQFPTLKLKSLKRLTFTS

NKGGNAFSEVDLPSLEFLDLSRNGLSFKGCCSQSDFGTTSLKYLDLSFNGVITMSSNFLG

LEQLEHLDFQHSNLKQMSEFSVFLSLRNLIYLDISHTHTRVAFNGIFNGLSSLEVLKMAG

NSFQENFLPDIFTELRNLTFLDLSQCQLEQLSPTAFNSLSSLQVLNMSHNNFFSLDTFPY

KCLNSLQVLDYSLNHIMTSKKQELQHFPSSLAFLNLTQNDFACTCEHQSFLQWIKDQRQL

LVEVERMECATPSDKQGMPVLSLNITCQMNKTIIGVSVLSVLVVSVVAVLVYKFYFHLML

LAGCIKYGRGENIYDAFVIYSSQDEDWVRNELVKNLEEGVPPFQLCLHYRDFIPGVAIAA

NIIHEGFHKSRKVIVVVSQHFIQSRWCIFEYEIAQTWQFLSSRAGIIFIVLQKVEKTLLR

QQVELYRLLSRNTYLEWEDSVLGRHIFWRRLRKALLDGKSWNPEGTVGTGCNWQEATSI

>ENSMUSP00000022124;Cd180

MAPDISCFFLVALFLASCRATTSSDQKCIEKEVNKTYNCENLGLNEIPGTLPNSTECLEF

SFNVLPTIQNTTFSRLINLTFLDLTRCQIYWIHEDTFQSQHRLDTLVLTANPLIFMAETA

LSGPKALKHLFFIQTGISSIDFIPLHNQKTLESLYLGSNHISSIKLPKGFPTEKLKVLDF

QNNAIHYLSKEDMSSLQQATNLSLNLNGNDIAGIELGAFDSAVFQSLNFGGTQNLLVIFK

GLKNSTIQSLWLGTFEDMDDEDISPAVFEGLCEMSVESINLQKHYFFNISSNTFHCFSGL

QELDLTATHLSELPSGLVGLSTLKKLVLSANKFENLCQISASNFPSLTHLSIKGNTKRLE

LGTGCLENLENLRELDLSHDDIETSDCCNLQLRNLSHLQSLNLSYNEPLSLKTEAFKECP

QLELLDLAFTRLKVKDAQSPFQNLHLLKVLNLSHSLLDISSEQLFDGLPALQHLNLQGNH

FPKGNIQKTNSLQTLGRLEILVLSFCDLSSIDQHAFTSLKMMNHVDLSHNRLTSSSIEAL

SHLKGIYLNLASNRISIILPSLLPILSQQRTINLRQNPLDCTCSNIYFLEWYKENMQKLE

DTEDTLCENPPLLRGVRLSDVTLSCSMAAVGIFFLIVFLLVFAILLIFAVKYFLRWKYQH

I

>ENSP00000256447;CD180

MAFDVSCFFWVVLFSAGCKVITSWDQMCIEKEANKTYNCENLGLSEIPDTLPNTTEFLEF

SFNFLPTIHNRTFSRLMNLTFLDLTRCQINWIHEDTFQSHHQLSTLVLTGNPLIFMAETS

LNGPKSLKHLFLIQTGISNLEFIPVHNLENLESLYLGSNHISSIKFPKDFPARNLKVLDF

QNNAIHYISREDMRSLEQAINLSLNFNGNNVKGIELGAFDSTIFQSLNFGGTPNLSVIFN

GLQNSTTQSLWLGTFEDIDDEDISSAMLKGLCEMSVESLNLQEHRFSDISSTTFQCFTQL

QELDLTATHLKGLPSGMKGLNLLKKLVLSVNHFDQLCQISAANFPSLTHLYIRGNVKKLH

LGVGCLEKLGNLQTLDLSHNDIEASDCCSLQLKNLSHLQTLNLSHNEPLGLQSQAFKECP

QLELLDLAFTRLHINAPQSPFQNLHFLQVLNLTYCFLDTSNQHLLAGLPVLRHLNLKGNH

FQDGTITKTNLLQTVGSLEVLILSSCGLLSIDQQAFHSLGKMSHVDLSHNSLTCDSIDSL

SHLKGIYLNLAANSINIISPRLLPILSQQSTINLSHNPLDCTCSNIHFLTWYKENLHKLE

GSEETTCANPPSLRGVKLSDVKLSCGITAIGIFFLIVFLLLLAILLFFAVKYLLRWKYQH

I

>CG4168-PA; CG4168-PA

MHLYLVLISCLVSSSHCWQFDGGGGHRRPRNSGRGPPSSSGGRGSAALLGSQQQDILYAC

PLNSMCQCAGLPNETSTLIEINCNEVALYKFPAWSPHRTDFLPGMSPCGREARKNSQNYA

PRNFKRIKMGACDGGRGFDNCNWGHKLEIHLISQNFQQCPDPVPLARPENEHRIESVASS

PAGNWFGLRAMDDVPWTWSSLVHRFEFMHSSVRYIEMSNTHLQSVDDETFQGLRLKTLKL

IDNELQDISERSFSTMTHSLMTLDISGNKMQHLPLDALQRLHSLSRLVAQRNHITTLDGN

WDAQHDTLRSLHLSDNDITEVAPGGGSIEVVSQNDNSSQPSSLAAVQTRLETSNSIYPPS

PSSGDRTTGGRPFEQLQKLLWLDLSNNRIYHVAGNYLPRSLVTMDLSSNLLTVFPQQLFE

QLPELRIVSLRDNLIRSVQWKELQVRPLRMHLERLDLGQNCIENLESDYFQQNYSDVHLR

ALNLEQNFVTQLPEAVFKATGIAHLVLAFNAISRVHPSAFEGLTETLEYLDLERNRLTTV

PVALSSLHHLKYLYLTSNQISQLNNLPSFTENLRVLSLSGNNFSMIPVLGLKNYTQLSYL

NMGYNSITDIPEGIFAVDSWGSNLQTILLRNNKITHLHLGSFAGLEQIQEISLSFNDITI

HHPLVFENVSRTLKILELSFAVFPARSLESLDPLDALLPLSQLIWLGLDNNNLKQVSNES

FAQMRELSYINLSFNQLKTLPRGLFQSDAHSHLVEIDLSYNGLERLEAQTFHSLGDLQTL

NLQSNRLRTIARHAFHNLEFLRYLDLSYNRLVNISHGAFTVLPNLAALDLMHNQLCSLSL

KSFLYVSNTTTPLRLNVSHNHIASFYDELSSYMYIYQLDISHNHVTKSDSFTNLANTLRF

LNLAHNQLGSLQSHAFGDLEFLEILNVAHNNLTSLRRRSFQGLNSLQELDLSHNQLDQLQ

VEQFSNLRKLRILRINSNRLRALPREVFMNTRLEFLDIAENQLSVWPVPAFTDIGFTLRS

IQMSHNNLEYLDASMFINSQFLYDISLARNRITILPDNTFSFLNNLTNLDLSQNPLVTTN

LREVFVHTPRLRKLSLHHMGLYVLPPLKLPLLSYLDVSGNYLQELSPLGSLRHLRHVNVS

HNKLTNASCAAEHLPPSVRVLDLAHNPLRRITLHDLASLRHLAELNILDVKVTNPQAFSK

LRSLRKLHASSHANLGEIVARIPGLQQLRVHCLEPNIGQQLFAKLANNTKIRLLELYGSN

VQTIAPDVFTGLSRSQRLQVKISHTRISDLPPGIFYALREVPHLSIDISHNRINALAADS

FYPNKSYWDAVGTRSIMGGLITSHNPLECECGLVWFGHWLRRWLRESAQIKVIQKDDLKR

MVQRARANTCHDPTSGRHLPILEIFPEDLLCQASALSSSGQRIFLLSFAMALLIPIVMTT

MTL

>CG6890-PA;Tollo

MLATTHMLYVLIATCVIPIFGAALSKTVLYQAPDECRWSGGGEHDITLVCHLRTINSELE

NTNFSVIQPQNTVRLRLECNDALFFQSSLSPDSFRSLVELRDLTIEYCKLGNLTDGSFRG

LQELRNLTIRTHNGDWSTMSLEMASNSFVEFRQLERLDLSLNNIWLIPDGMVCPLKSLQH

LNASYNKIQDISNFYFSASLSSRKARVCGSTLQSLDLSANKMVSLPTAMLSALGRLTHLN

MAKNSMSFLADRAFEGLLSLRVVDLSANRLTSLPPELFAETKQLQEIYLRNNSINVLAPG

IFGELAELLVLDLASNELNSQWINAATFVGLKRLMMLDLSANKISRLEAHIFRPLASLQI

LKLEDNYIDQLPGGIFADLTNLHTLILSRNRISVIEQRTLQGLKNLLVLSLDFNRISRMD

QRSLVNCSQLQDLHLNDNKLQAVPEALAHVQLLKTLDVGENMISQIENTSITQLESLYGL

RMTENSLTHIRRGVFDRMSSLQILNLSQNKLKSIEAGSLQRNSQLQAIRLDGNQLKSIAG

LFTELPNLVWLNISGNRLEKFDYSHIPIGLQWLDVRANRITQLGNYFEIESELSLSTFDA

SYNLLTEITASSIPNSVEVLYLNDNQISKIQPYTFFKKPNLTRVDLVRNRLTTLEPNALR

LSPIAEDREIPEFYIGHNAYECDCNLDWLQKVNRESRTQPQLMDLDQIHCRLAYARGSSH

VSLIEAKSDDFLCKYASHCFALCHCCDFQACDCKMECPDRCSCYHDQSWTSNVVDCSRAS

YEQTLPSHIPMDSTQLYLDGNNFRELQSHAFIGRKRLKVLHLNHSRIEVLHNRTFYGLLE

LEVLQLQSNQLKALNGNEFQGLDNLQELYLQHNAIATIDTLTFTHLYHLKILRLDHNAIT

SFAVWNFLPSYLNELRLASNPWTCSCEFIDKLRDYINRHEYVVDKLKMKCDVISGNSTQQ

MVIYPGSGEPASLPVVQCSQTLPLGLDNNFNYAEQAGGENASNATSTKMILNQPPKLDYI

PILVAILTAFIFVMICISLVFIFRQEMRVWCHSRFGVRLFYNAQKDVDKNEREKLFDAFV

SYSSKDELFVNEELAPMLEMGEHRYKLCLHQRDFPVGGYLPETIVQAIDSSRRTIMVVSE

NFIKSEWCRFEFKSAHQSVLRDRRRRLIVIVLGEVPQKELDPDLRLYLKTNTYLQWGDKL

FWQKLRFALPDVSSSQRSNVAGQSCHVPINHASYHHHHHVHQQAMPLPHSVHHHQQQFML

PPPPQQPGSFRRQPSLHQQQQQQQQIRGNNNTTQQQQQQQAALLMGGGSVGGPAPQMIPL

AGGIQQQSLPLPPNQQPTPASRNLHM

>CG7250-PA;Toll-6

MIYYMLLILPVVLAQDQQHTTESLSTKHHQQQQLSHSNAIMGEAGVSNSQLMQPSTPART

LRPLTAGAGGDPSLYDAPDDCHFMPAAGLDQPEIALTCNLRTVNSEFDTTNFSVIPAEHT

IALHILCNDEIMAKSRLEAQSFAHLVRLQQLSIQYCKLGRLGRQVLDGLEQLRNLTLRTH

NILWPALNFEIEADAFSVTRRLERLDLSSNNIWSLPDNIFCTLSELSALNMSENRLQDVN

ELGFRDRSKEPTNGSTESTSTTESAKKSSSSSTSCSLDLEYLDVSHNDFVVLPANGFGTL

RRLRVLSVNNNGISMIADKALSGLKNLQILNLSSNKIVALPTELFAEQAKIIQEVYLQNN

SISVLNPQLFSNLDQLQALDLSMNQITSTWIDKNTFVGLIRLVLLNLSHNKLTKLEPEIF

SDLYTLQILNLRHNQLENIAADTFAPMNNLHTLLLSHNKLKYLDAYALNGLYVLSLLSLD

NNALIGVHPDAFRNCSALQDLNLNGNQLKTVPLALRNMRHLRTVDLGENMITVMEDSAFK

GLGNLYGLRLIGNYLENITMHTFRDLPNLQILNLARNRIAVVEPGAFEMTSSIQAVRLDG

NELNDINGLFSNMPSLLWLNISDNRLESFDYGHVPSTLQWLDLHKNRLSSLSNRFGLDSE

LKLQTLDVSFNQLQRIGPSSIPNSIELLFLNDNLITTVDPDTFMHKTNLTRVDLYANQIT

TLDIKSLRILPVWEHRALPEFYIGGNPFTCDCNIDWLQKINHITSRQYPRIMDLETIYCK

LLNNRERAYIPLIEAEPKHFLCTYKTHCFAVCHCCEFDACDCEMTCPTNCTCFHDQTWST

NIVECSGAAYSEMPRRVPMDTSELYIDGNNFVELAGHSFLGRKNLAVLYANNSNVAHIYN

TTFSGLKRLLILHLEDNHIISLEGNEFHNLENLRELYLQSNKIASIANGSFQMLRKLEVL

RLDGNRLMHFEVWQLSANPYLVEISLADNQWSCECGYLARFRNYLGQSSEKIIDASRVSC

IYNNATSVLREKNGTKCTLRDGVAHYMHTNEIEGLLPLLLVATCAFVAFFGLIFGLFCYR

HELKIWAHSTNCLMNFCYKSPRFVDQLDKERPNDAYFAYSLQDEHFVNQILAQTLENDIG

YRLCLHYRDVNINAYITDALIEAAESAKQFVLVLSKNFLYNEWSRFEYKSALHELVKRRK

RVVFILYGDLPQRDIDMDMRHYLRTSTCIEWDDKKFWQKLRLALPLPNGRGNNNKRVVSG

CLSGRTPSVNMYATSHEYQAGNGGVIPPPSARYADCGSNNYATINECAAAGGGRGYKPIP

TSASAAAAACKFNTMNQLSKKQQRDLSVAGMAKTLEHQHHHNHQANRRSQHEYAVPSYLP

SAAPAYDSVDYAKQQIRNNANCECVNLGTAKRAAGKNPASGLPSSFSSNFVPPGGASYNC

KKSCSCIGDDELLCSCGGGGGIGVNLLESGTQSSVTMSSSSNNSRQPELTHYESNLSLND

DEDEDHDQQKNLWA

>CG1149-PA;MstProx

MKLLLTIPDDYCETYCGGICNDTVASKTCDREAYDYIATNEYHLIMRDGHLEVNWKIPDP

NIFIISPISKENRLKLNELIVSDTSYPIRAVDYLRQLGVETVTRFENQINSFKVIERDVH

YINGPKSLKIIIQSNLYLNEIIEYINKTTDNVNEIIINAYKTVENQQIALDNLIFNGKSH

LRSLTFIGFQIENLSTKPFAQFINLKRMVLTNCTVRNLTFLRTLQKSLEHLELDIDNEVD

LKYFTNFSSLKFMKVRNYIPNKNFTALICTHKNCNFIRGINGLECPKLCQCLYIIDDLEL

NIDCSNLGLLQIPPLPIPSYGGVKLNFSNNSLSQLPTMTLPGYKLVKRLDVSRNRLTNLS

INHLPAKLDYLDVSFNEIINMGNDVIKYLRTVPIFKQTGNQWTIHCDDKPLLNFFRHLKL

IIRMKSAEMKPMFLHSLTELPKGFLKFLGKHFIWLGVRKQEYYLINEEQLLQSMHRKLNN

LNTIMSIYKYMEWLHRKLIFVNREYDLFYIRQMAAPCPHKCECCYSRDSLILKIDCRNKF

VYNFPDIVARNSRLMUKQNMSSPMELHLSKNNISNITIAMLPKELRFLDLRFNNLVTLDD

KVLSYLKKNSIKTKLSGNPWNCDCKSRSVLSILRDHEPLEYDVTLKRCNISPTDCPDVCV

CCLDNLTWPSFIVDCRGEGLLQMPSLSSRVTYVDLRNNNLTALSQKNRSSIENRSLKLHL

LDNPWSCSCNDIEKINFMKSVSSSIVDFTEIKCSNGEKLVSINQHIVCPSDLFYYLALAI

SLVATIIALNFLIWFRQPVLVWFYEHGVCLSLSAKRELDKDKRFDAFLAFTHKDEALLEE

FVDRLERGRPRFQLCFYLRDWLAGESIPDCIGQSIKDSRRIIVLMTENFMNSTWGRLEFR

LALHATSRDRCKRLIVVLYPNVKNFDSLDSELRTYMAFNTYLERSHPNFWNKLIYSMPLL

PSYVDUD

>CG18241-PA;Toll-4

MEHSKLWDLRPEVRERRFKWTSDGQQQQQQQLGWCNNKDDPPNSHQKSKSNNDARNLNSR

VRVRARVRVVPGEMRMGDINCSNGLGNIREDYCEIYLDELGENGTCSIANNEVTTEDYQM

KLVFLKLEINWTSPVFHGWNIFKICNETDYELVIISVLGIRSEVDMRISPAVQYLSLLGI

REISGYDIYLPSVLITEMDVHHANGPKMVTFKYLYDSTVNSVITNNYIRKTMNNTEKIKI

YYHNTFEKTTLTMEKNIFHGKNKMSALIFNGLKIKGLTNNTFENLTSLNTLIFDNVFLKD

LSFLRSSTLQSSLTYCIMKVDNMVDLKSFEKFTNLEIIEVSQYKGFKNFTAFICEPYKSH

CKFTLGINEVACPLKCNCSYNRDKSQLEIDCWQKNLTTIPSLPVPKKGSSALVFQSNLLA

ELPDNSLEGYHNLKSLDVSYNQLTSLSVSQLPESLHYLDIRHNKITTLSPQVVEYLYSVN

VFNQYGNKWSIYCDEYHLQEFFWYKAKLLRIKTSKFQTIMEYIELSSKGSFVENFFVQNI

DQLYLEANEDEIIDAFGPSDKYFNLKLMEALNHAIWLFSGEFDEIILHHLNSPCPYRCSC

CFEWHTGEFLINCRNLSLDIYPRLPNSIPYKTTLYLDRNEIRKLTNTESLVVAGHASIHK

LHMSQNLLRELPLHLLPENITYLDVRNNLLKYLDDGVIAFLEYRENITKIELSGNPWECN

CKAKAFLSFLRRHEPMEYETVLRRVEITDDKCPEDCICCVDTSNSDSLAYVVDCSGKELS

EIPQLPTPTYGQTTLVFERNSLKKWPSSLLPGYSSVTRFYLAHNRLSDIDQLPDKLEYLD

ISNNNFSALDDRVRGFLQKRMNSSQLQLSLFGNPWTCRCEDKDFLVFVKEQAKNIANASA

IQCIDTGRSLIEVEETDICPSVLIYYTSLAVSLLIIALSINVFICFRQPIMIWFYEHEIC

LSLAARRELDEDKKYDAFLSFTHKDEDLIEEFVDRLENGRHKFRLCFYLRDWLVGESIPD

CINQSVKGSRRIIILMTKNFLKSTWGRLEFRLALHATSRDRCKRLIVVLYPDVEHFDDLD

SELRAYMVLNTYLDRNNPNFWNKLMYSMPHASHLKRSRSDAETKV

>CG5490-PA;Tl

MSRLKAASELALLVIILQLLQWPGSEASFGRDACSEMSIDGLCQCAPIMSEYEIICPANA

ENPTFRLTIQPKDYVQIMCNLTDTTDYQQLPKKLRIGEVDRVQMRRCMLPGHTPIASILD

YLGIVSPTTLIFESDNLGMNITRQHLDRLHGLKRFRFTTRRLTHIPANLLTDMRNLSHLE

LRANIEEMPSHLFDDLENLESIEFGSNKLRQMPRGIFGKMPKLKQLNLWSNQLHNLTKHD

FEGATSVLGIDIHDNGIEQLPHDVFAHLTNVTDINLSANLFRSLPQGLFDHNKHLNEVRL

MNNRVPLATLPSRLFANQPELQILRLRAELQSLPGDLFEHSTQITNISLGDNLLKTLPAT

LLEHQVNLLSLDLSNNRLTHLPDSLFAHTTNLTDLRLEDNLLTGISGDIFSNLGNLVTLV

MSRNRLRTIDSRAFVSTNGLRHLHLDHNDIDLQQPLLDIMLQTQINSPFGYMHGLLTLNL

RNNSIIFVYNDWKNTMLQLRELDLSYNNISSLGYEDLAFLSQNRLHVNMTHNKIRRIALP

EDVHLGEGYNNNLVHVDLNDNPLVCDCTILWFIQLVRGVHKPQYSRQFKLRTDRLVCSQP

NVLEGTPVRQIEPQTLICPLDFSDDPRERKCPRGCNCHVRTYDKALVINCHSGNLTHVPR

LPNLHKNMQLMELHLENNTLLRLPSANTPGYESVTSLHLAGNNLTSIDVDQLPTNLTHLD

ISWNHLQMLNATVLGFLNRTMKWRSVKLSGNPWMCDCTAKPLLLFTQDNFERIGDRNEMM

CVNAEMPTRMVELSTNDICPAEKGVFIALAVVIALTGLLAGFTAALYYKFQTEIKIWLYA

HNLLLWFVTEEDLDKDKKFDAFISYSHKDQSFIEDYLVPQLEHGPQKFQLCVHERDWLVG

GHIPENIMRSVADSRRTIIVLSQNFIKSEWARLEFRAAHRSALNEGRSRIIVIIYSDIGD

VEKLDEELKAYLKMNTYLKWGDPWFWDKLRFALPHRRPVGNIGNGALIKTALKGSTDDKL

ELIKPSPVTPPLTTPPAEATKNPLVAQLNGVTPHQAIMIANGKNGLTNLYTPNGKSHGNG

HINGAFIINTNAKQSDV

>CG7121-PA;Tehao

MLTYLPVVWLFFALLVLRSATGQIIPLPTFCLGLSPQCTCAAEGNVVRFHCPDEYAMLLE

VSEPGASLYMSYYASTELQWLPRFNISSLVKIEFDAYIFWPEKFLSDLLKTLGVQTVKTI

IFRDRTLETVVTRDVLNSGNGYMETSQPENITTWHFGSVPGLKKFKFFSHVPELQESIFH

GFDTLRDLHLSVNVTTLPGNMLSTVNGTLKTLTIESPGIVSFGNPLLRELQQLRNLSLAL

IHPFHERDKQLQPHFFGSMTNLEEVRLASATSSVNRSMFKGTNKLQLIKMNGNDDLMELP

GEIFLDQVNLKTLDLSCNAIVTLHEDVFKGLGNLTLLDLSKNRLTNLSSTIFAPLTSLNV

LRLNKNSLTAMSPSVFQDVVSLNYIEMVNTQFYGATLLMNYEAVVCTNDEACQYKSAEWQ

CDPRCICWVQRSVGSLIVDCRGTSLEELPDLPRTTLLSTVLKVGNNSLTSLPTVSEHSGY

ANVSGLFLSDNNLTSLGSGDQLPDNLTHLDVRGNQIQSLSDEFLLFLQEPNNTMTLSLSG

NPITCGCESLSLLFFVRTNPQRVRDIADIVCTKQKKSFQQMEAFELCPSYVLLISCVVGG

LVIVICLLTVFYLMFQQELKIWLYNNNLCLWWVSEEELDKDKTYDAFISYSHKDEELISK

LLPKLESGPHPFRLCLHDRDWLVGDCIPEQIVRTVDDSKRVIIVLSQHFIDSVWARMEFR

IAYQATLQDKRKRIIIILYRELEHMNGIDSELRAYLKLNTYLKWGDPLFWSKLYYAMPHN

RRVLKGQKKHAGPLI

>T05A1.3;T05A1.3

MSVLPLLHLFLISSLQIAPTATSSPSQFPCPTRCQCYADNEQNQQVHLICKWEQLNVTTL

QLARPDLVRTLTIKCPYHSPKVSTPPHSLFQGFRNLDRLELDRCLIDTVPEGLFAGLGQL

YSLIVKNAKITDFPREIFAHVPNLMTLDLSGNRLRIEPYSLRSLQNLIHLDVSDNDIGFL

TNTLISLTKLKVITMNNNKITNIDFRRFPENLTDLSIRHNLVSTIHYVPASARNLKRLDL

AGNRMEFVGGLTTGAVNVLPAELKHVDLSNNKLTYLHDHAFEHLTNLILLDLKNNSLKEV

KSSSFAGSKFQVKLFLSENPLLCHCNHKWLMDSSAKNISIGDLQTIECLNILKPEKKMLL

TLAHSRNQLLCKYSNMCEADCECCQKKECECRFECPPTCKCLRSADVSIVRTSQNIMVCD

KLRWDKLGKFPSPLTQLHMNQTDWKIFETEKLKELSNLRALKITNSKMTSVEMEKLTDLA

NLTHLEITSSAITSIPETITKLPSLTHLYLSDNPLDQLSSTSLNHLDGLKKIRLGGNFSR

FACDCESPSDFQRWLMRRINRAKIDDIDDLYCDLNGHGTVWMLEALPGTNESVCLDPTEE

TKQWMTFVENAQKGIMERVYTSTESTTKSEKTMMTTTLKKSILDTMDELEGVKSTEKISN

KVISTTTVPTTTPRYRKKYKNYDNEPHKFVNVLIFLLFLCVIILIISIGVTVYLKFWHPE

ELLMKRRKKERLRPEIQREEEPLQHLD

>ENSMUSP00000068906;Tlr11

MPRMERHQFCSVLLILILLTLVSLTLTGWAWTIPDCIIADSLLFPNLSYYIPFCTSAPGL

HLLASCSNVKNLNQTLKRVPRNTEVLCLQGMVPTLPAKAFIRFHSLQLLRLQLRTTSVTS

RTFQGLDQLQYLFFDHHAPCCLSLFLSPNCFESLRSLSSLSFQGYCLTYSQSIYLPTSLR

HLTLRNSCLTKFQDLQRLFPDLLLSTSSTPNIKPGAPFLETLDLSYNLQLKQAGVRDLYG

LTLHSLILDGTPLKALDLTDSGLLHLHFLSLVGTGIEKVPASLTGYSELRALDLGKNQIQ

NILENGEIPGYKALEFLSLHDNHLQTLPTRFLHTLPQLQKLNLSMNKLGPILELPEGLFS

TNLKVLDLSYNQLCDVPHGALSLLSQLQELWLSGNNISSLSNESLQGLRQLRTLDLSWNQ

IKVLKPGWLSHLPALTTLNLLGTYLEYILGIQLQGPKMLRHLQLGSYPILDIYPPWPPTL

LSLEIQAESCIQFMIHSGQPFLFLENLTLETSILLLKPDNITIHFPSLRRLTLRGYSFIF

STSQLQRFFPQQLPLLEHFFIWCENSYAVDLYLFGMPRLRVLELGYLNFFYESSTMKLEM

LLKEVPQLQVLALSHLNLRNLSVSSFKSLQDLKLLLFNSERALEMNSNLQEFIPQMPQYV

YFSDVTFTCQCEASWLESWATRAPNTFVYGLEKSICIANASDYSKTLLFSFLATNCPHGT

EFWGFLTSFILLLLLIILPLISCPKWSWLHHLWTLFHTCWWKLCGHRLRGQFNYDVFISY

CEEDQAWVLEELVPVLEKAPPEGEGLRLCLPARDFGIGNDRMESMIASMGKSRATLCVLT

GQALASPWCNLELRLATYHLVARPGTTHLLLLFLEPLDRQRLHSYHRLSRWLQKEDYFDL

SQGKVEWNSFCEQLKRRLSKAGQERD

>ENSMUSP00000074381;Tlr12

MGRYWLLPGLLLSLPLVTGWSTSNCLVTEGSRLPLVSRYFTFCRHSKLSFLAACLSVSNL

TQTLEVVPRTVEGLCLGGTVSTLLPDAFSAFPGLKVLALSLHLTQLLPGALRGLGQLQSL

SFFDSPLRRSLFLPPDAFSDLISLQRLHISGPCLDKKAGIRLPPGLQWLGVTLSCIQDVG

ELAGMFPDLVQGSSSRVSWTLQKLDLSSNWKLKMASPGSLQGLQVEILDLTRTPLDAVWL

KGLGLQKLDVLYAQTATAELAAEAVAHFELQGLIVKESKIGSISQEALASCHSLKTLGLS

STGLTKLPPGFLTAMPRLQRLELSGNQLQSAVLCMNETGDVSGLTTLDLSGNRLRILPPA

AFSCLPHLRELLLRYNQLLSLEGYLFQELQQLETLKLDGNPLLHLGKNWLAALPALTTLS

LLDTQIRMSPEPGFWGAKNLHTLSLKLPALPAPAVLFLPMYLTSLELHIASGTTEHWTLS

PAIFPSLETLTISGGGLKLKLGSQNASGVFPALQKLSLLKNSLDAFCSQGTSNLFLWQLP

KLQSLRVWGAGNSSRPCLITGLPSLRELKLASLQSITQPRSVQLEELVGDLPQLQALVLS

STGLKSLSAAAFQRLHSLQVLVLEYEKDLMLQDSLREYSPQMPHYIYILESNLACHCANA

WMEPWVKRSTKTYIYIRDNRLCPGQDRLSARGSLPSFLWDHCPQTLELKLFLASSALVFM

LIALPLLQEARNSWIPYLQALFRVWLQGLRGKGDKGKRFLFDVFVSHCRQDQGWVIEELL

PALEGFLPAGLGLRLCLPERDFEPGKDVVDNVVDSMLSSRTTLCVLSGQALCNPRCRLEL

RLATSLLLAAPSPPVLLLVFLEPISRHQLPGYHRLARLLRRGDYCLWPEEEERKSGFWTW

LRSRLG

>CG40500-PD; gi|113193636|gb|ABI31006.1|

MQKLTALDFDYNEIVRVDDYSFYGLRISKLNMKGNRLQGMPEHAFAGLEECMQEIDVSENGLRTFPLMAL

RKLDHLRILRLSNNRIPTFYGDIQLATNNASAAAAAVGAFQLPSLIFLDLSSNQFAEIGPDCFRAFPQLK

TLSFYANQIELVQPEAFKSLRELMSLDMSHNRIIGLDPKVFEKNKRLQTVDLSHNHIHTIGGVFSNLPQL

REVFLSENNILELPADAFTNSTNVDVIYLESNAIAHIDPNVFSTLVNLDHLYLRSNFIPLLPVTLFDKST

KLTSLSLDNNEIQDLEIGMFRKLEHLREVRLHNNRIRRVRRGVFEPLPSLQELHIQKNSIEDIEPQAFHT

LENMQHINLQDNQLTVLEDIFPDENSSLLSVQLEANYLHKVHPRTFSRQQKVQIMWLKDNQLTRVERSFF

ADTPQLGRLYLSDNKIRDIEKDTFVNLLLLQFLDLSGNQLRQLRRDYFAPLQDLEELSLARNHIEAIEGY

AFAKLKNLKSLDLSHNPLVQLTRDIFSNEFPLNSLNLGNCSLRKLEQHAFKSLTNLNELNLERNQLNPAD

IQTLDIPNLRRLLLSHNNFSYAGSVGIMAGMLDRLRSLQQLSMSNCSLGQIPDLLFAKNTNLVRLDLCDN

RLTQINRNIFSGLNVFKELRLCRNELSDFPHIALYNLSTLESLDLARNQLASIDFFKLSGTLNLRQLILR

DNKITALSGFNAVNLTQLDSVDLSGNLLLSLPANFLRHSINLQKVHLSNNRFLQIPSSALSDVSIPRLSW

LNLTGNPINRIYTVKEERYPYLKELYICQTNLSILTSKDFEAFQALQHLHLVNNRITRISPGAFKSLTNL

LTLDLSVNELEMLPKERLQGLRLLRFLNISHNTLKDLEEFSVDLLEMQTLDLSFNQLDRISKKTFRNLHG

LLELFLMGNRMTVLSNDAFRFLRKLHVLDLRKNYFELVPLEPLRPLETNLRTLRLEENPLHCSCDAQKLW

EWLRDHRKWSLSMGSGAGRGIGGLTGGLGGDSINYLRCEHPTELRGKVFGRMEPQQFCDAPLIPKMAIQD

IQPYSVVVSWQSRDHLGLNGFEIVYHATGDGLVTAGSPTSRERDSLRDIHDPERQRTRDQARERSRERDR

DRERDRERFSIATDEIHGKRLNGTASSTKLTKLSPNTRYHICVIGSGNWLSEPLANALQRLHTNESLREE

EAVMMSAPLPESFSPYQHQHNRNSNGNANPAMVQDHNKKQNPDQDLDNVVGQDNALMEVLKNSHISACTD

VQTLDSTPSLVTDENGLSSNGFIHSILTRRLGLIVGCCLGIIVFIVMISVLSYVKLKKKRIENAKRQAAL

PPEYISYRHFSIPNEELTRTAANAGTVAAAAAANNSHNSNNSTDEGSSPHMSSVPSGISAHISGTSLSTG

GTGTGSTTTTATTPIGGVPLGKAGSTLVGVGLVVADSSVDVGYAHNHAHAHAHRHMAAGVVLNTNHLSVC

>IPI00662472.2; Mm NTRK1

MLRGQRLGQL GWHRPAAGLG SLMTSL MLAC ASAASCREVC CPVGPSGLRC TRAGSLDTLR

GLRGAGNLTE LYVENQQHLQ RLEFEDLQGL GELRSLTIVK SGLRFVAPDA FRFTPRLSHL

NLSSNALESL SWKTVQGLSL QDLTLSGNPL HCSCALFWLQ RWEQEGLCGV HTQTLHDSGP

GDQFLPLGHN TSCGVPTVKI QMPNDSVEVG DDVFLQCQVE GLALQQADWI LTELEGAATV

KKFGDLPSLG LILVNVTSDL NKKNVTCWAE NDVGRAEVSV QVSVSFPASV HLGLAVEQHH

WCIPFSVDGQ PAPSLRWLFN GSVLNETSFI FTQFLESALT NETMRHGCLR LNQPTHVNNG

NYTLLAANPY GQAAASVMAA FMDNPFEFNP EDPIPVSFSP VDGNSTSRDP VEKKDETPFG

VSVAVGLAVS AALFLSALLL VLNKCGQRSK FGINRPAVLA PEDGLAMSLH FMTLGGSSLS

PTEGKGSGLQ GHIMENPQYF SDTCVHHIKR QDIILKWELG EGAFGKVFLA ECYNLLNDQD

KMLVAVKALK EASENARQDF QREAELLTML QHQHIVRFFG VCTEGGPLLM VFEYMRHGDL

NRFLRSHGPD AKLLAGGEDV APGPLGLGQL LAVASQVAAG MVYLASLHFV HRDLATRNCL

VGQGLVVKIG DFGMSRDIYS TDYYRVGGRT MLPIRWMPPE SILYRKFSTE SDVWSFGVVL

WEIFTYGKQP WYQLSNTEAI ECITQGRELE RPRACPPDVY AIMRGCWQRE PQQRLSMKDV

HARLQALAQA PPSYLDVLG

>XP_892248; Mm IGSF10

MQKRGREVSCLLISLTAICLVVTPGSRVCPRRCACYVPTEVHCTFRYLTSIPDGIPANVE

RVNLGYNSLTRLTENDFSGLSRLELLMLHSNGIHRVSDKTFSGLQSLQVLKMSYNKVQII

EKDTLYGLRSLTRLHLDHNNIEFINPEAFYGLTLLRLVHLEGNRLTKLHPDTFVSLSYLQ

IFKTSFIKYLYLSDNFLTSLPKEMVSSMPNLESLYLHGNPWTCDCHLKWLSEWMQGNPDI

IKCKKERIPSSPQQCPLCMNPRISKGRSIAMVPSGSFLCTKPTIDPSLKSKSLGIQEDNG

SASVSPQDFIEPFGSLSLNMTDLSGNKANVICSIQKPSRTLPIAFTEENDYIMLNMSFST

NLVCSVNYNHIQPVWQLLALYSDSPLILERKPQHTETPLLSPKYQQVALRPEDTFTNIEA

DFKADPFWFQQEKISLQLNRTATTLSTLQIQFSTDAQITLPKAEMRPVKRKWTMILMMNN

TRLEHTVLVGGTIALDCPGKGDPSPHLEWVLADGSKVRAPYVSEDGRILIDKKGKLELQM

ADTFDAGLYHCISTNDADADILTYRITVVEPYVENKHENGALHTVIMGEILDLPCLSTGI

PDASISWILPRNTVFSQSSRDMQILNNGTLRILQATPKDQGHYRCVAANPSGADFSSFQV

SVQMKGQRTIEHDRDIDGSGLEEPKPSVLLKQPPSLKLPASSLTGTEAGKQVSGIHKKNK

HRDLTHRRRGDSTLRRFREHRRQLPLSARRIDPQHWAALLEKAKKNSVLRKQENTTVKPT

PLAIPLVELAGEEKDASGLTPPDEEFTVLKTKAFGVPERSPTADSRPVNHGFVTSSASGT

EVSSTVNPQTLLPTHLPDFKLFNVVDSAAVSKSMNRPVTSKIEDTTHQNPIIIFPSVAEI

QDSAQVGRTSSQSAHPATGGAMATYGYTTMLSSFTNKANTVLQSANPTESYGPQIPLTEV

SRVSSNNSLAHTTKDPGFSKRPSDSHTTAPSLFQTPRNNSTGNVGRERTIWSRGRAISPY

RTPVLRRHRHRIVRPALKGPANRNISQVSATEPPGMCRTCSSTERLTMATAALSVTGSSH

TTLPKANNVGIISEESTTVVKKPSLLLKNKQDVDIETITTTINYFRSESTHMTPTEASMI

SAPTSISLGKTPIDTSGHLSMPRTIQAGTDLVVTPPLSSPLSQPSIPTKATSTKLSRRKI

PWHPIFANNHNKEGMLKNLHQFGLQKNTATKPPEKAPLLPTDHGSSSPSTTLLASLTPAQ

SATMAATRRNGTEVQGARSLSAGKEQPFINSFLVLPSTTRKRSSTLSFLSVETPTVTTPP

VIASAIISETQEVRSKKAKDQTKGSLKNRKGPTITPRQISGYSTYSVPTTTDTPLAFSHS

PGKVTGRTVSTAAPHSAASLLGITELPQKCTHTSGNITASETTLLSKSQESTAMKRASAT

PPLLSSGAPRMPTPSPPPFTKVVVTDSEVPAVFKMMSNRMVTIYESSRHDIDLQQPSAEA

SPNPEILTGSTDFPLSSLLTSTPMPAPRVDKPQDSQWKPSPWPENKFQLRSYSETIEKGK

RPEISLSPHLSFPEASTHALHWNAQRHAEKSVFDKKPAQNPTSKHLPYDSLPKTILKKPR

IIGGKAASFTVPTNSDVLLPCEAVGDPKPTIHWTRVSSGREISRGIQKTRFHVLPNGTLS

IQRVSIQDRGQYLCAASNPLGVDHLHVTLSVVSYPARILESHVKEITAHSGSTVKLKCRV

EGMPRPTISWILANQTVVSETPEGSRKVWVTPDGTLIIHNLSLYDRGFYKCVANNPSGQD

SLLVKIQVITAPPVIIEQKRQAIVGVLGESLKLPCTAKGTPQPSVHWVLYDGTELKPLQL

THSRFFLYPNGTLYIRNIVSSVRGTYECIATSSSGSERRVVILRVEEQETVPRIETASQK

WTEVNLGEKLLLNCSATGDPKPTIIWKLPSKVVIDQWHRMGSRIHVYPNGSLVIGSVTEK

DGGDYLCVARNKMGDDLVLMHVRLRLTPAKIEHKQHFKKQVLHGKDFQVDCKASGSPVPE

VSWSLPDGTVVNNVAQADDSGYRTKRYTLFHNGTLYFNKVGMAEEGDYICSAQNTLGKDE

MKVHLTVLTAIPRIRQNYRSNVRIKAGDTAVLDCEVTGEPKPNVFWLLPSNNVISFSNDR

FIFHANGTLSINKVKPLDSGKYVCVAQNPSGDDTKTYKLDIVSRPPLINGLYANKTVIKA

TAIQHSKKHLDCRADGVPPPQITWIMPDNIFLTAPYYGGRITVHQNGTLEIRNIRLSDSA

DFTCVVRSEGGESVLVVQLKVLEMLRRPTFRNPFNEKVVAQVGKPVAMNCSVDGNPTPEI

IWILPDGTQFANGPQNSPYLMASNGSLIVYKATRNKSGKYRCTARNKVGYIEKLILLEIG

QKPVILTYEPGMIKSAGGESLSLHCVSDGIPKPNVKWTTPGGLVIDRPQVGGKYILHENG

TLVIKETTAHDRGNYICKAQNSVGQAVISVPVTIVAYPPRIINYLPRSMLRRTGEAMQLH

CVALGVPKPQITWETPGYSLLSTATERRPHRSEMLPLQGTLVIQNLRASDSGVYKCRAQN

VLGADYATTYIQVL

>ENSP00000314901; Hs Lrrn1 (release 40)

MARMSFVIAACQLVLGLLMTSLTESSIQNSECPQLCVCEIRPWFTPQSTYREATTVDCND

LRLTRIPSNLSSDTQVLLLQSNNIAKTVDELQQLFNLTELDFSQNNFTNIKEVGLANLTQ

LTTLHLEENQITEMTDYCLQDLSNLQELYINHNQISTISAHAFAGLKNLLRLHLNSNKLK

VIDSRWFDSTPNLEILMIGENPVIGILDMNFKPLANLRSLVLAGMYLTDIPGNALVGLDS

LESLSFYDNKLVKVPQLALQKVPNLKFLDLNKNPIHKIQEGDFKNMLRLKELGINNMGEL

VSVDRYALDNLPELTKLEATNNPKLSYIHRLAFRSVPALESLMLNNNALNAIYQKTVESL

PNLREISIHSNPLRCDCVIHWINSNKTNIRFMEPLSMFCAMPPEYKGHQVKEVLIQDSSE

QCLPMISHDSFPNRLNVDIGTTVFLDCRAMAEPEPEIYWVTPIGNKITVETLSDKYKLSS

EGTLEISNIQIEDSGRYTCVAQNVQGADTRVATIKVNGTLLDGTQVLKIYVKQTESHSIL

VSWKVNSNVMTSNLKWSSATMKIDNPHITYTARVPVDVHEYNLTHLQPSTDYEVCLTVSN

IHQQTQKSCVNVTTKNAAFAVDISDQETSTALAAVMGSMFAVISLASIAVYFAKRFKRKN

YHHSLKKYMQKTSSIPLNELYPPLINLWEGDSEKDKDGSADTKPTQVDTSRSYYMW

>AAH11057; Hs Lrrn6a

mqvskrmlag gvrsmpspll acwqpilllv lgsvlsgsat gcpprcecsa qdravlchrk

rfvavpegip tetrlldlgk nriktlnqde fasfphleel elnenivsav epgafnnlfn

lrtlglrsnr lkliplgvft glsnltkldi senkivilld ymfqdlynlk slevgdndlv

yishrafsgl nsleqltlek cnltsiptea lshlhglivl rlrhlninai rdysfkrlyr

lkvleishwp yldtmtpncl yglnltslsi thcnltavpy lavrhlvylr flnlsynpis

tiegsmlhel lrlqeiqlvg gqlavvepya frglnylrvl nvsgnqlttl eesvfhsvgn

letlildsnp lacdcrllwv frrrwrlnfn rqqptcatpe fvqgkefkdf pdvllpnyft

crrarirdrk aqqvfvdegh tvqfvcradg dpppailwls prkhlvsaks ngrltvfpdg

tlevryaqvq dngtylciaa naggndsmpa hlhvrsyspd wphqpnktfa fisnqpgege

anstratvpf pfdiktliia ttmgfisflg vvlfclvllf lwsrgkgntk hnieieyvpr

ksdagissad aprkfnmkmi

>XP_944870; Hs Lrrn6b

MTCWLCVLSLPLLLLPAAPPPAGGCPARCECTVQTRAV

ACTRRRLTAVPDGIPAETRLLELSRNRIRCLNPGDLAALPALEELDLSENAIAHVEPGAF

ANLPRLRVLRLRGNQLKLIPPGVFTRLDNLTLLDLSENKLVILLDYTFQDLHSLRRLEVG

DNDLVFVSRRAFAGLLALEELTLERCNLTALSGESLGHLRSLGALRLRHLAIASLEDQNF

RRLPGLLHLEIDNWPLLEEVAAGSLRGLNLTSLSVTHTNITAVPAAALRHQAHLTCLNLS

HNPISTVPRGSFRDLVRLRELHLAGALLAVVEPQAFLGLRQIRLLNLSNNLLSTLEESTF

HSVNTLETLRVDGNPLACDCRLLWIVQRRKTLNFDGRLPACATPAEVRGDALRNLPDSVL

FEYFVCRKPKIRERRLQRVTATAGEDVRFLCRAEGEPAPTVAWVTPQHRPVTATSAGRAR

VLPGGTLEIQDARPQDSGTYTCVASNAGGNDTYFATLTVRPEPAANRTPGEAHNETLAAL

RAPLDLTTILVSTAMGCITFLGVVLFCFVLLFVWSRGRGQHKNNFSVEYSFRKVDGPAAA

AGQGGARKFNMKMI

>**ENSMUSP00000058050;** Mm Lrrn6d (**release 40; curated)**

MDAATAPKQAWLPWSPLLFLLLLPGGSISSCPTVC

DCTSQTRAVFCAHRRLDTIPGGLPLDTELLDLSGNRLWGLQRGMLSRLGQLQELDLSYNQ

LSTLEPGAFHGLQSLLTLRLQGNRLRIVGPGIFSGLTALTLLDLRLNQIVLFLDGAFSEL

GSLQQLEVGDNHLVFVAPGAFAGLAKLSTITLERCNLSTVPGLALAQLPALVALRLRELD

IERLPAGALRGLGQLKELEIHHWPSLEALDPGSLVGLNLSSLAITRCNLSSVPFQALHHL

SFLRILDLSQNPISAIPARRLSPLVRLQELRLSGACLTSIAAHAFHGLTAFHLLDVADNA

LQTLEETAFPSPDKLVTLRLSGNPLTCDCRLLWLLRLRRRLDFGTSPPACAGPQHVQGKS

LREFSDILPPGHFTCKPALIRKSGPRWVIAEEGGHAVFSCSGDGDPAPTVSWMRPQGAWL

GRVGRVRVLEDGTLEIRSVQLRDRGAYVCVVSNVAGNDSLRTWLEVIQVEPPNGTLSDPN

ITMPGIPGPFFLDSRGVAMVLAVGFLPFLTSVTLCFGLIALWSKGKGRVKHHMTFDFVAP

RPSGDKNSGGNRVTAKLF

>ENSP00000333767; Hs Lrrn6d (release 40)

MDAATAPKQAWLPWSPLLFLLLLPGGSISSCPTVC

DCTSQTRAVFCAHRRLDTIPGGLPLDTELLDLSGNRLWGLQRGMLSRLGQLQELDLSYNQ

LSTLEPGAFHGLQSLLTLRLQGNRLRIVGPGIFSGLTALTLLDLRLNQIVLFLDGAFSEL

GSLQQLEVGDNHLVFVAPGAFAGLAKLSTITLERCNLSTVPGLALAQLPALVALRLRELD

IERLPAGALRGLGQLKELEIHHWPSLEALDPGSLVGLNLSSLAITRCNLSSVPFQALHHL

SFLRILDLSQNPISAIPARRLSPLVRLQELRLSGACLTSIAAHAFHGLTAFHLLDVADNA

LQTLEETAFPSPDKLVTLRLSGNPLTCDCRLLWLLRLRRRLDFGTSPPACAGPQHVQGKS

LREFSDILPPGHFTCKPALIRKSGPRWVIAEEGGHAVFSCSGDGDPAPTVSWMRPQGAWL

GRVGRVRVLEDGTLEIRSVQLRDRGAYVCVVSNVAGNDSLRTWLEVIQVEPPNGTLSDPN

ITMPGIPGPFFLDSRGVAMVLAVGFLPFLTSVTLCFGLIALWSKGKGRVKHHMTFDFVAP

RPSGDKNSGGNRVTAKLF

>ENSMUSP00000035999; Mm Lrig2 (release 40)

MAAAPRGIWEQRRLGCGLGPLARLLILAQALRLLPAARAGLCPAPCACRLPLLDCSRRKL

PAPSWRALSGPLPSDISSLDLSHNRLSNWNNTLESQTLQEVKMNYNELTEIPYFGEPTPN

ITLLSLVHNLIPEINAEAFELYSALESLDLSSNIISEIKTSSFPRMSLKYLNLSNNRIST

LEAGCFDNLSDSLLVVKLNRNRISMIPPKVFKLPHLQFLELKRNRIKIVEGLTFQGLDSL

RSLKMQRNGISKLKDGAFFGLNNMEELELEHNNLTGVNKGWLYGLRMLQQLYMSQNAIEK

ISPDAWEFCQRLSELDLSYNQLTRLDESAFVGLSLLERLNLGDNRVTHIADGVFRFLSNL

QTLDLRNNDISWAIEDASEAFSGLKSLTKLILQGNRIKSVTQKAFIGLESLEYLDLNNNA

IMSIQENAFSQTHLKGLVLNTSSLLCDCHLKWLLQWLVDNNFHHSVNVSCAHPEWLAGQS

ILNVDLKDFVCDDFLKPQIRTHPESTIALRGVNVTLTCTAVSSSDSPMSTIWRKDSEILY

DVDIENFVRYRQQDGEALEYTSVLRLFSVNFTDEGKYQCIVTNHFGSNYSQKAKLTVNEM

PSFLKTPMDLTIRTGAMARLECAAEGHPTPQISWQKDGGTDFPAARERRMHVMPEDDVFF

IANVKIEDMGIYSCMAQNIAGGLSANASLTVLETPSFIRPLEDKTVTRGETAVLQCIAGG

SPAPRLNWTKDDGPLLVTERHFFAAANQLLIIVDAGLEDAGKYTCLMSNTLGTERGHIYL

NVISSPNCDSSQSSIGHEDDGWTTVGIVIIVVVCCVVGTSLIWVIVIYHMRRKNEDYSIT

NTEELNLPADIPSYLSSQGTLSEPQEGYSNSEAGSHQQLMPPANGYTHRGTDGGAGTRVI

CSDCYDNANIYSRTREYCPYTYIAEEDVLDQALSSLMVQMPKETFLSHPPQDAANLESLI

PSAEREPAAFPTNHERMTENLPFSQRSSEIFQRPLWNMNRELGLLPFSQQPVLESPELTE

RDPNCSSPVTCRRLHDHAFDFSRTRIIQDGTEGT

>ENSP00000355396; Hs Lrig 2 (release 40)

MAPAPLGVPEEQLLGCRSRVLSRLLFIAQTALLLLPAAGAGLCPAPCSCRIPLLDCSRRK

LPAPSWRALSGLLPPDTAILDFSHNRLSNWNISLESQTLQEVKMNYNELTEIPYFGEPTS

NITLLSLVHNIIPEINAQALQFYPALESLDLSSNIISEIKTSSFPRMQLKYLNLSNNRIT

TLEAGCFDNLSSSLLVVKLNRNRMSMIPPKIFKLPHLQFLELKRNRIKIVEGLTFQGLDS

LRSLKMQRNGISKLKDGAFFGLNNMEELELEHNNLTRVNKGWLYGLRMLQQLYVSQNAIE

RISPDAWEFCQRLSELDLSYNQLTRLDESAFVGLSLLERLNLGDNRVTHIADGVFRFLSN

LQTLDLRNNEISWAIEDASEAFAGLTSLTKLILQGNQIKSITKKAFIGLESLEHLDLNNN

AIMSIQENAFSQTHLKELILNTSSLLCDCHLKWLLQWLVDNNFQHSVNVSCAHPEWLAGQ

SILNVDLKDFVCDDFLKPQIRTHPETIIALRGMNVTLTCTAVSSSDSPMSTVWRKDSEIL

YDVDTENFVRYWQQAGEALEYTSILHLFNVNFTDEGKYQCIVTNHFGSNYSQKAKLTVNE

MPSFLKTPMDLTIRTGAMARLECAAEGHPAPQISWQKDGGTDFPAARERRMHVMPEDDVF

FIANVKIEDMGIYSCMAQNTAGGLSANASLTVLETPSFIRPLEDKTVTRGETAVLQCIAG

GSPAPRLNWTKDDGPLLVTERHFFAAANQLLIIVDAGLEDAGKYTCIMSNTLGTERGHIY

LNVISSPNCDSSQSSIGHEDDGWTTVGIVIIVVVCCVVGTSLIWVIVIYHMRRKNEDYSI

TNTEELNLPADIPSYLSSQGTLSEPQEGYSNSEAGSHQQLMPPANGYIHKGTDGGTGTRV

ICSDCYDNANIYSRTREYCPYTYIAEEDVLDQTLSSLMVQMPKETYLVHPPQDTTALESL

IPSANREPSAFPTNHERISEKKLPSTQMSGETLQRPVWNINRELGLPHPPFSQQPVHESP

QLHQNEGLAGREPDCSASSMSCHRLQDHAFDFSRTRNIQDGSEGT

>T21D12.9; T21D12.9 C elegans

MIVYSLLLLILYDFLPKVVTFDTVCIGLCHCVGNVVDCSSLDLSEIPTTIPNNTRILLLS

DNEIESIDKSRLKGFYFLQTLDISNNIIRHIDFEFFYNLPNLKILNIRKNRLARIPRGSH

ELGHLEKLDLRSNLISTVTSEELSYLAAVRSVDLSRNLISYLPKPTTSAKVNIEKLDLAS

NSITDIGTDHFSSFNTLVTLKLARNHITTLNQFSFSRLRKLESLDLTRNMIREVRFLAFN

QLPSLQNVSLARNDVYRLDDGMFYACEGLKHLNLSTNRVQAVTEGWMFGLTSLEVLDLSY

NQIQSFHISSWSHTPKLKWLSLHSNRIQSLPSGSFRVLRQLEELILSANSIDSLHKFALV

GMSSLHKLDLSSNTLAVCVEDGAVLYNTSMPFLRSLRFTNNQLRVIPKRAFERFPALEEL

DLTDNPIATIHPEAFEPLELKRLVMNSSSILCDCQISWLASWIYRLKLDKSSIIAKCSYP

PPLADLYVVAIDTANLTCHNDSPRAKIVRQPVEVSTLIGEKARFTCNVYGASPLSIEWRV

MENGQPRVLVQDSATFLSINRTAVVNGTFDERELAAAELLLDNVAMTDNSEYQCVARNRF

GSDFSTHVKLQVYQAPKFTYTPEDMPLLVGQTAKFLCAATGTPRPEIKWAFEQIPFPAAE

ARRLYVTPNDDHIYIMNVTKEDQGAYTCHATNVAGQTQASANLIVFENFFHYPESPDLSP

MLIKKRDALKIDCSCDLISSRQRMVWKRQQQVILFLKKAKFSDNDQILTLTQTTFSDSGE

YSCELWVDDTILMRKITMVKVVNEDEFVSSEATLTQRVQRIRAMASKLVDNIKSTGNGIY

LMASCSAFGLMILGVITSICICIAKCSCNQNHVFNVLPVQV

>AAZ20639; Mm Lrfn1

mapgpfssgl fspppaalpf llllwagasr gqpcpgrcic qnvaptltml caktgllfvp

paidrrvvel rltdnfiaav rrrdfadmts lvhltlsrnt igqvaagafa dlralralhl

dsnrlaevrg dqlrglgnlr hlilgnnqir kvesaafdaf lstvedldls ynnlealpwe

avgqmvnlnt ltldhnlidh iaegtfvqlh klvrldmtsn rlhklppdgl flrsqgggpk

pptpltvsfg gnplhcncel lwlrrltred dletcatpeh ltdryfwsip eeeflceppl

itrqaggral vvegqavslr cravgdpepv vhwvapdgrl lgnssrtrvr gdgtldvtit

tlrdsgtftc iasnaageat apvevcvvpl plmapppaap ppltepgssd iatpgrpgan

dstserrlva aeltsssvli rwpaqrpvpg irmyqvqyns saddslvyrm ipstsqtflv

ndlaagrayd lcvlavyddg atalpatrvv gcvqfttagd papcrplrah flggtmiiai

ggvivasvlv fivllmiryk vygdgdsrri kgtsrtpprv shvcsqtnga gaqqasappa

pdryealrev avpaaieaka meaeatstel evvlgrslgg satslcllps eetsgeesra

mtgprrsrsg algpptsapp tlalvpggap arprpqqrys fdgdygalfq shsyprrarr

tkrhrstphl dgagggaage dgdlglgsar arlaftstew mlestv

>XP_945666; Hs Lrfn1

mapgpfssal lspppaalpf llllwagasr gqpcpgrcic qnvaptltml caktgllfvp

paidrrvvel rltdnfiaav rrrdfanmts lvhltlsrnt igqvaagafa dlralralhl

dsnrlaevrg dqlrglgnlr hlilgnnqir rvesaafdaf lstvedldls ynnlealpwe

avgqmvnlnt ltldhnlidh iaegtfvqlh klvrldmtsn rlhklppdgl flrsqgtgpk

pptpltvsfg gnplhcncel lwlrrltred dletcatpeh ltdryfwsip eeeflceppl

itrqaggral vvegqavslr cravgdpepv vhwvapdgrl lgnssrtrvr gdgtldvtit

tlrdsgtftc iasnaageat apvevcvvpl plmapppaap ppltepgssd iatpgrpgan

dsaaerrlva aeltsnsvli rwpaqrpvpg irmyqvqyns svddslvyrm ipstsqtflv

ndlaagrayd lcvlavyddg atalpatrvv gcvqfttagd papcrplrah flggtmiiai

ggvivasvlv fivllmiryk vygdgdsrrv kgsrslprvs hvcsqtngag tgaaqapalp

aqdhyealre vesqaapava veakameaet asaepevvlg rslggsatsl cllpseetsg

eesraavgpr rsrsgalepp tsapptlalv pggaaarprp qqrysfdgdy galfqshsyp

rrarrtkrhr stphldgagg gaagedgdlg lgsaraclaf tstewmlest v

>ENSMUSP00000047573; Mm Lrfn2 (release 40)

METLLGGLLAFGMAFAVVDACPKYCVCQNLSESLGTLCPSKGLLFVPPDIDRRTVELRLG

GNFIIHIGRQDFANMTGLVDLTLSRNTISHIQPFSFLDLESLRSLHLDSNRLPSLGEDTL

RGLVNLQHLIVNNNQLGGIADDAFEDFLLTLEDLDLSYNNLHGLPWDSVRRMVNLHQLSL

DHNLLDHIAEGTFADLQKLARLDLTSNRLQKLPPDPIFARSQASLLTATPFAPPLSFSFG

GNPLHCNCELLWLRRLERDDDLETCGSPGSLKGRYFWHIREEEFVCEPPLITQHTHKLLV

LEGQAATLKCKAIGDPSPLIHWVAPDDRLVGNSSRTAVYDNGTLDILITTSQDSGPFTCI

AANAAGEATATVEVSIVQLPHLSNSTSRMAPPKSRLSDITGSSKTSRGGGGSGAGEPPKS

TPERAVLVSDVTTTSALVKWSVSKSAPRVKMYQLQYNCSDDEVLIYRMIPASNKAFVVNN

LVSGTGYDLCVLAMWDDTATTLTATNIVGCAQFFTKADYPQCQSMHSQILGGTMILVIGG

IIVATLLVFIVILMVRYKVCNHDTPGKMAAATVSNVYSQTNGSQPPPLGGIPVGQLPQAP

PKVVVRNELMDFSTSLARACDSSSSSSLGSGEAAGLGRGPWRLPPPAPRPKPSLDRLMGA

FASLDLKSQRKEELLDSRTPAGRGAGTSSRGHHSDREPLLGPPATRARSLLPLPLEGKAK

RSHSFDMGDFAAAAAAVPGGYSPPRRVSNIWTKRSLSVNGMLLPFEESDLVGARGTFGSS

EWVMESTV

>ENSP00000345985; Hs Lrfn2 (release 40)

METLLGGLLAFGMAFAVVDACPKYCVCQNLSESLGTLCPSKGLLFVPPDIDRRTVELRLG

GNFIIHISRQDFANMTGLVDLTLSRNTISHIQPFSFLDLESLRSLHLDSNRLPSLGEDTL

RGLVNLQHLIVNNNQLGGIADEAFEDFLLTLEDLDLSYNNLHGLPWDSVRRMVNLHQLSL

DHNLLDHIAEGTFADLQKLARLDLTSNRLQKLPPDPIFARSQASALTATPFAPPLSFSFG

GNPLHCNCELLWLRRLERDDDLETCGSPGGLKGRYFWHVREEEFVCEPPLITQHTHKLLV

LEGQAATLKCKAIGDPSPLIHWVAPDDRLVGNSSRTAVYDNGTLDIFITTSQDSGAFTCI

AANAAGEATAMVEVSIVQLPHLSNSTSRTAPPKSRLSDITGSSKTSRGGGGSGGGEPPKS

PPERAVLVSEVTTTSALVKWSVSKSAPRVKMYQLQYNCSDDEVLIYRMIPASNKAFVVNN

LVSGTGYDLCVLAMWDDTATTLTATNIVGCAQFFTKADYPQCQSMHSQILGGTMILVIGG

IIVATLLVFIVILMVRYKVCNHEAPSKMAAAVSNVYSQTNGAQPPPPSSAPAGAPPQGPP

KVVVRNELLDFTASLARASDSSSSSSLGSGEAAGLGRAPWRIPPSAPRPKPSLDRLMGAF

ASLDLKSQRKEELLDSRTPAGRGAGTSARGHHSDREPLLGPPAARARSLLPLPLEGKAKR

SHSFDMGDFAAAAAGGVVPGGYSPPRKVSNIWTKRSLSVNGMLLPFEESDLVGARGTFGS

SEWVMESTV

>ENSMUSP00000053123; Mm Lrrc4b (release 40)

MAQAHIRGSPCPLLPPGRMSWPHGALLLLWLFSPPLRAGGGGVAVTSAAGGGSPPATSCP

AACSCSNQASRVICTRRELAEVPASIPVNTRYLNLQENSIQVIRTDTFKHLRHLEILQLS

KNLVRKIEVGAFNGLPSLNTLELFDNRLTTVPTQAFEYLSKLRELWLRNNPIESIPSYAF

NRVPSLRRLDLGELKRLEYISEAAFEGLVNLRYLNLGMCNLKDIPNLTALVRLEELELSG

NRLDLIRPGSFQGLTSLRKLWLMHAQVATIERNAFDDLKSLEELNLSHNNLMSLPHDLFT

PLHRLERVHLNHNPWHCNCDVLWLSWWLKETVPSNTTCCARCHAPAGLKGRYIGELDQSH

FTCYAPVIVEPPTDLNVTEGMAAELKCRTGTSMTSVNWLTPNGTLMTHGSYRVRISVLHD

GTLNFTNVTVQDTGQYTCMVTNSAGNTTASATLNVSAVDPVAAGGPGGGGPGGGGGAGGA

GGYTYFTTVTVETLETQPGEEAQQPRGTEKEPPGPTTDGAWGGGRPDAAAPASASTTAPA

PRSSRPTEKAFTVPITDVTENALKDLDDVMKTTKIIIGCFVAITFMAAVMLVAFYKLRKQ

HQLHKHHGPTRTVEIINVEDELPAASAVSVAAAAAVAGGAGVGGDSHLALPALERDHLNH

HHYVAAAFKAHYGGNPGGGCGAKGPGLNSIHEPLLFKSGSKENVQETQI

>**ENSP00000373853;** Hs Lrrc4b (**release 40 curated)**

MARARGSPCPPLPPGRMSWPHGALLFLWLFSPPLGAGGGGVAVTSAAGGGSPPATSCP

VACSCSNQASRVICTRRDLAEVPASIPVNTRYLNLQENGIQVIRTDTFKHLRHLEILQLS

KNLVRKIEVGAFNGLPSLNTLELFDNRLTTVPTQAFEYLSKLRELWLRNNPIESIPSYAF

NRVPSLRRLDLGELKRLEYISEAAFEGLVNLRYLNLGMCNLKDIPNLTALVRLEELELSG

NRLDLIRPGSFQGLTSLRKLWLMHAQVATIERNAFDDLKSLEELNLSHNNLMSLPHDLFT

PLHRLERVHLNHNPWHCNCDVLWLSWWLKETVPSNTTCCARCHAPAGLKGRYIGELDQSH

FTCYAPVIVEPPTDLNVTEGMAAELKCRTGTSMTSVNWLTPNGTLMTHGSYRVRISVLHD

GTLNFTNVTVQDTGQYTCMVTNSAGNTTASATLNVSAVDPVAAGGTGSGGGGPGGSGGVG

GGSGGYTYFTTVTVETLETQPGEEALQPRGTEKEPPGPTTDGVWGGGRPGDAAGPASSST

TAPAPRSSRPTEKAFTVPITDVTENALKDLDDVMKTTKIIIGCFVAITFMAAVMLVAFYK

LRKQHQLHKHHGPTRTVEIINVEDELPAASAVSVAAAAAVASGGGVGGDSHLALPALERD

HLNHHHYVAAAFKAHYSSNPSGGGCGGKGPPGLNSIHEPLLFKSGSKENVQETQI

>ENSMUSP00000021008; Mm PXDN (release 40)

MAVRPTRRCLLALLLCFAWWAMAVVASKQGAGCPSRCLCFRTTVRCMHLLLEAVPAVAPQ

TSILDLRFNRIREIQPGAFRRLRSLNTLLLNNNQIKKIPNGAFEDLENLKYLYLYKNEIQ

SIDRQAFKGLASLEQLYLHFNQIETLDPESFQHLPKLERLFLHNNRITHLVPGTFSQLES

MKRLRLDSNALHCDCEILWLADLLKTYAQSGNAQAAATCEYPRRIQGRSVATITPEELNC

ERPRITSEPQDADVTSGNTVYFTCRAEGNPKPEIIWLRNNNELSMKTDSRLNLLDDGTLM

IQNTQEADEGVYQCMAKNVAGEAKTQEVTLRYLGSPARPTFVIQPQNTEVLVGESVTLEC

SATGHPLPQITWTRGDRTPLPIDPRVNITPSGGLYIQNVAQSDSGEYTCFASNSVDSIHA

TAFIIVQALPQFTVTPQSRVVIEGQTVDFQCAAKGHPQPVIAWTKGGSQLSVDRRHLVLS

SGTLRISGVALHDQGQYECQAVNIIGSQKVVAHLTVQPRVTPVFASIPSDMTVEVGTNVQ

LPCSSQGEPEPAITWNKDGVQVTESGKFHISPEGFLTINDVGTADAGRYECVARNTIGYA

SVSMVLSVNVPDVSRNGDPYVATSIVEAIATVDRAINSTRTHLFDSRPRSPNDLLALFRY

PRDPYTVGQARAGEIFERTLQLIQEHVQHGLMVDLNGTSYHYNDLVSPQYLSLIANLSGC

TAHRRVNNCSDMCFHQKYRTHDGTCNNLQHPMWGASLTAFERLLKAVYENGFNTPRGINS

QRQYNGHVLPMPRLVSTTLIGTEVITPDEQFTHMLMQWGQFLDHDLDSTVVALSQARFSD

GQHCSSVCSNDPPCFSVMIPPNDPRVRSGARCMFFVRSSPVCGSGMTSLLMNSVYPREQI

NQLTSYIDASNVYGSTDHEARSIRDLASHRGLLRQGIVQRSGKPLLPFATGPPTECMRDE

NESPIPCFLAGDHRANEQLGLTSMHTLWFREHNRIAAELLKLNPHWDGDTVYHETRKIVG

AEIQHITYRHWLPKILGEVGMKMLGEYRGYDPSVNAGIFNAFATAAFRFGHTLINPLLYR

LDENFEPIPQGHVPLHKAFFSPFRIVNEGGIDPLLRGLFGVAGKMRIPSQLLNTELTERL

FSMAHTVALDLAAINIQRGRDHGIPPYHDYRVYCNLSAAYTFEDLKNEIKSPVIREKLQR

LYGSTLNIDLFPALMVEDLVPGSRLGPTLMCLLSTQFRRLRDGDRLWYENPGVFSPAQLT

QLKQTSLARILCDNSDNITRVQQDVFRVAEFPHGYSSCEDIPRVDLRVWQDCCEDCRTRG

QFNAFSYHFRGRRSLEFSYEDDKPTKRARWRKALSVKHGKHLSNATSATHEHLEGPATND

LKEFVLEMQKIITDLRKQINSLESRLSTTECVDDSGESHGGNTKWKKDPCTVCECKNGQI

TCFVEACQPAACPQPVKVEGACCPVCLKNTAEEKP

>**ENSMUSP00000053573;** Mm Flrt1 (**release 40 curated)**

MDLRDWLFLCYGLIAFLTEVIDSTTCPSVCRCDNGFIYCNDRGLTSIPSDIPDDATTLY

LQNNQINNAGIPQDLKTKVKVQVIYLYENDLDEFPINLPRSLRELHLQDNNVRTIARDSL

ARIPLLEKLHLDDNSVSTVSIEEDAFADSKQLKLLFLSRNHLSSIPSGLPHTLEELRLDD

NRISTIPLHAFKGLNSLRRLVLDGNLLANQRIADDTFSRLQNLTELSLVRNSLAAPPLNL

PSAHLQKLYLQDNAISHIPYNTLAKMRELERLDLSNNNLTTLPRGLFDDLGNLAQLLLRN

NPWFCGCNLMWLRDWVRARAAVVNVRGLMCQGPEKVRGMAIKDITSEMDECFEAGSQGGA

ANAAAKTTVSNHASATTPQGSLFTLKAKRPGLRLPDSNIDYPMATGDGAKTLVIQVKPLT

ADSIRITWKAMLPASSFRLSWLRLGHSPAVGSITETLVQGDKTEYLLTALEPKSTYIICM

VTMETGNTYVADETPVCAKAETADSYGPTTTLNQEQNAGPMAGLPLAGIIGGAVALVFLF

LVLGAICWYVHRAGELLTRERVYNRGSRRKDDYMESGTKKDNSILEIRGPGLQMLPINPY

RSKEEYVVHTIFPSNGSSLCKGAHTIGYGTTRGYREAGIPDVDYSYT

>**ENSP00000246841;** Hs Flrt1 (**release 40 curated)**

MDLRDWLFLCYGLIAFLTEVIDSTTCPSVCRC

DNGFIYCNDRGLTSIPADIPDDATTLYLQNNQINNAGIPQDLKTKVNVQVIYLYENDLDE

FPINLPRSLRELHLQDNNVRTIARDSLARIPLLEKLHLDDNSVSTVSIEEDAFADSKQLK

LLFLSRNHLSSIPSGLPHTLEELRLDDNRISTIPLHAFKGLNSLRRLVLDGNLLANQRIA

DDTFSRLQNLTELSLVRNSLAAPPLNLPSAHLQKLYLQDNAISHIPYNTLAKMRELERLD

LSNNNLTTLPRGLFDDLGNLAQLLLRNNPWFCGCNLMWLRDWVKARAAVVNVRGLMCQGP

EKVRGMAIKDITSEMDECFETGPQGGVANAAAKTTASNHASATTPQGSLFTLKAKRPGLR

LPDSNIDYPMATGDGAKTLAIHVKALTADSIRITWKATLPASSFRLSWLRLGHSPAVGSI

TETLVQGDKTEYLLTALEPKSTYIICMVTMETSNAYVADETPVCAKAETADSYGPTTTLN

QEQNAGPMASLPLAGIIGGAVALVFLFLVLGAICWYVHQAGELLTRERAYNRGSRKKDDY

MESGTKKDNSILEIRGPGLQMLPINPYRAKEEYVVHTIFPSNGSSLCKATHTIGYGTTRG

YRDGGIPDIDYSYT

>ENSMUSP00000053399; Mm Flrt3 (release 40)

MISPAWSLFLIGTKIGLFFQVAPLSVVAKSCPSVCRCDAGFIYCNDRSLTSIPVGIPEDA

TTLYLQNNQINNVGIPSDLKNLLKVQRIYLYHNSLDEFPTNLPKYVKELHLQENNIRTIT

YDSLSKIPYLEELHLDDNSVSAVSIEEGAFRDSNYLRLLFLSRNHLSTIPGGLPRTIEEL

RLDDNRISTISSPSLHGLTSLKRLVLDGNLLNNHGLGDKVFFNLVNLTELSLVRNSLTAA

PVNLPGTSLRKLYLQDNHINRVPPNAFSYLRQLYRLDMSNNNLSNLPQGIFDDLDNITQL

ILRNNPWYCGCKMKWVRDWLQSLPVKVNVRGLMCQAPEKVRGMAIKDLSAELFDCKDSGI

VSTIQITTAIPNTAYPAQGQWPAPVTKQPDIKNPKLIKDQRTTGSPSRKTILITVKSVTP

DTIHISWRLALPMTALRLSWLKLGHSPAFGSITETIVTGERSEYLVTALEPESPYRVCMV

PMETSNLYLFDETPVCIETQTAPLRMYNPTTTLNREQEKEPYKNPNLPLAAIIGGAVALV

SIALLALVCWYVHRNGSLFSRNCAYSKGRRRKDDYAEAGTKKDNSILEIRETSFQMLPIS

NEPISKEEFVIHTIFPPNGMNLYKNNLSESSSNRSYRDSGIPDSDHSHS

>ENSMUSP00000061244; Mm Amigo1 (release 40)

MQPQRDLRGLWLLLLSVFLLLFEVARAGRSVVSCPANCLCASNILSCSKQQLPNVPQSLP

SYTALLDLSHNNLSRLRAEWTPTRLTNLHSLLLSHNHLNFISSEAFVPVPNLRYLDLSSN

HLHTLDEFLFSDLQALEVLLLYNNHIVVVDRNAFEDMAQLQKLYLSQNQISRFPVELIKD

GNKLPKLMLLDLSSNKLKKLPLTDLQKLPAWVKNGLYLHNNPLECDCKLYQLFSHWQYRQ

LSSVMDFQEDLYCMHSKKLHNIFSLDFFNCSEYKESAWEAHLGDTLTIRCDTKQQGMTKV

WVSPSNEQVLSQGSNGSVSVRNGDLFFKKVQVEDGGVYTCYAMGETFNETLSVELKVYNF

TLHGHHDTLNTAYTTLVGCILSVVLVLIYLYLTPCRCWCRGVEKPSSHQGDSLSSSMLST

TPNHDPMAGGDKDDGFDRRVAFLEPAGPGQGQNGKLKPGNTLPVPEATGKGQRRMSDPES

VSSVFSDTPIVV

>**ENSMUSP00000082137;** Mm Amigo 3 (**release 40)**

MAWLVLSGILLCMLGAGLGTSDLEDVLPPAPHNCPDICICAADVLSCAGRGLQDLPVALP

TTAAELDLSHNALKRLHPGWLAPLSRLRALHLGYNKLEVLGHGAFTNASGLRTLDLSSNM

LRMLHTHDLDGLEELEKLLLFNNSLMHLDLDAFQGLRMLSHLYLSCNELSSFSFNHLHGL

GLTRLRTLDLSSNWLKHISIPELAALPTYLKNRLYLHNNPLPCDCSLYHLLRRWHQRGLS

ALHDFEREYTCLVFKVSESRVRFFEHSRVFKNCSVAAAPGLELPEEQLHAQVGQSLRLFC

NTSVPATRVAWVSPKNELLVAPASQDGSIAVLADGSLAIGRVQEQHAGVFVCLASGPRLH

HNQTLEYNVSVQKARPEPETFNTGFTTLLGCIVGLVLVLLYLFAPPCRGCCHCCQRACRN

RCWPRASSPLQELSAQSSMLSTTPPDAPSRKASVHKHVVFLEPGKKGLNGRVQLAVAEDF

DLCNPMGLQLKAGSESASSTGSEGLVMS

>ENSP00000323096; Hs Amigo 3 (release 40)

MTWLVLLGTLLCMLRVGLGTPDSEGFPPRALHNCPYKCICAADLLSCTGLGLQDVPAELP

AATADLDLSHNALQRLRPGWLAPLFQLRALHLDHNELDALGRGVFVNASGLRLLDLSSNT

LRALGRHDLDGLGALEKLLLFNNRLVHLDEHAFHGLRALSHLYLGCNELASFSFDHLHGL

SATHLLTLDLSSNRLGHISVPELAALPAFLKNGLYLHNNPLPCDCRLYHLLQRWHQRGLS

AVRDFAREYVCLAFKVPASRVRFFQHSRVFENCSSAPALGLERPEEHLYALVGRSLRLYC

NTSVPAMRIAWVSPQQELLRAPGSRDGSIAVLADGSLAIGNVQEQHAGLFVCLATGPRLH

HNQTHEYNVSVHFPRPEPEAFNTGFTTLLGCAVGLVLVLLYLFAPPCRCCRRACRCRRWP

QTPSPLQELSAQSSVLSTTPPDAPSRKASVHKHVVFLEPGRRGLNGRVQLAVAEEFDLYN

PGGLQLKAGSESASSIGSEGPMTT

>**ENSP00000361183;** Hs Lrrc22 (**release 40)**

MASVFHYFLLVLVFLDTHAAQPFCLPGCTCSEESFGRTLQCTSVSLGKIPGNLSEEFKQV

RIENSPLFEMPQGSFINMSTLEYLWLNFNNISVIHLGALEHLPELRELRLEGNKLCSVPW

TAFRATPLLRVLDLKRNKIDALPELALQFLVSLTYLDLSSNRLTVVSKSVFLNWPAYQKC

RQPDCGAEILSSLVVALHDNPWVCDCRLRGLVQFVKSITLPVILVNSYLICQGPLSKAGQ

LFHETELSACMKPQISTPSANITIRAGQNVTLRCLAQASPSPSIAWTYPLSMWREFDVLT

SSTGEDTALSELAIPAAHLVDSGNYTCMASNSIGKSNLVISLHVQPAQALHAPDSLSIPS

EGNAYIDLRVVKQTVHGILLEWLAVADTSKEEWFTLYIASDEAFRKEVVHIGPGINTYAV

DDLLPGTKYEACLSLEGQPPHQGQCVAFVTGRDAGGLEAREHLLHVTVVLCVVLLAVPVG

AYAWAAQGPCSCSKWVLRGCLHRRKAPSCTPAAPQSKDGSFREHPAVCDDGEGHIDTEGD

KEKGGTEDNS

>XP_143529; Mm XP_143529

mtaqdqgeap asscdsalkc lpeewtlpsf wcestrrlvl cndldmnevp anfpvdtskl

riektvvrrl paeafyylve lqylwlayns vasietssfy nlrqlhelrl dgnsltafpw

vslldmphlr tldlhnnria svpneavryl rnltcldlss nrlttlppdf ldswshlavt

psrspdfppr riilglqdnp wfcdchiskv ielskvtdha vvlldplmvc seperfqgil

fqrvelekcl kpsvmmsatk itsalgsnvl lrcdakghpt pqltwtrsdg stvnytviqe

spgegirwsi isltsishkd agdyrckakn lagiseavvt vtvvggvttt lspdssersp

geppeqhpqp glggstppsk swlspgltsa psyptpsaal ytstwsppps slppifsaas

attsvqtsis grtartshqp pllhpggksn akiekngrkf pplsaskkee lalldqaapm

etnvtikdlr varetgvsvt lmwnsssstq essvtvlysk ygekdlllvn addygknqat

inglepgsqy vacvcpkgvg predlcitfs tnrvegrgsq wslllvvtst acvivvplic

fllykvcklq ctsdpfweed lsketyiqfe tlsprsqsig elwtrrhrdd gerlllcsqs

svdsqmnlks dgcrteyyg

>[BC104037.1](http://www.ncbi.nlm.nih.gov/entrez/viewer.fcgi?val=BC104037.1); Hs [BC104037.1](http://www.ncbi.nlm.nih.gov/entrez/viewer.fcgi?val=BC104037.1)

mpllrtldlh nnkitsvpne alrylknlay ldlssnrltt ltpdflenwt hlvstpsgvl

dlspsriilg lqdnpwfcdc hiskmielsk vvdpaivlld plmtcseper ltgilfqrae

lehclkpsvm tsatkimsal gsnvllrcda tgfptpqitw trsdsspvny tviqespeeg

vrwsimsltg isskdagdyk ckaknlagms eavvtvtvlg itttpippdt sertgdhpew

dvqpgsgrst svssassylw sssfsptssf sastlsppst asfslspfss stvsstttls

tsisasttma nkrsfqlhqg gkrnlkvakn gsklppasts kkeelalldq tmltetnati

enlrvvsetk esvtlmwnmi ntthnsavtv lyskyggkdl lllnadsskn qvtidglepg

gqymacvcpk gvppqkdqci tfstervegd dsqwslllvv tstacvvilp licfllykvc

klqcksepfw eddlaketyi qfetlfprsq svgelwtrsh rddseklllc srssvesqvt

fksegsrpey yc

>NP_065902; Hs ISLR2

MFPLRALWLVWALLGVAGSCPEPCACVDKYAHQFADCAYKELREVPEGLPANVTTLSLSANKITVLRRGAFADVTQVTSLWLAHNEVRTVEPGALAVLSQLKNLDLSHNFISSFPWSDLRNLSALQLLKMNHNRLGSLPRD

ALGALPDLRSLRINNNRLRTLAPGTFDALSALSHLQLYHNPFHCGCGLVWLQAWAASTRVSLPEPDSIACASPPALQGVPVYRLPALPCAPPSVHLSAEPPLEAPGTPLRAGLAFVLHCIADGHPTPRLQWQLQIPGGTVVLEPPVLSGEDDGVGAEEGEGEGDGDLLTQTQAQTPTPAPAWPAPPATPRFLALANGSLLVPLLSAKEAGVYTCRAHNELGANSTSIRVAVAATGPPKHAPGAGGEPDGQAPTSERKSTAKGRGNSVLPSKPEGKIKGQGLAKVSILGETETEPEEDTSEGEEAEDQILADPAEEQRCGNGDPSRYVSNHAFNQSAELKPHVFELGVIALDVAEREARVQLTPLAARWGPGPGGAGGAPRPGRRPLRLLYLCPAGGGAAVQWSRVEEGVNAYWFRGLRPGTNYSVCLALAGEACHVQVVFSTKKELPSLLVIVAVSVFLLVLATVPLLGAACCHLLAKHPGKPYRLILRPQAPDPMEKRIAADFDPRASYLESEKSYPAGGEAGGEEPEDVQGEGLDEDAEQGDPSGDLQREESLAACSLVESQSKANQEEFEAGSEYSDRLPLGAEAVNIAQEINGNYRQTAG

>ENSP00000339255; Hs LRRC24 (release 40)

MALRAPALLPLLLLLLPLRAAGCPAACRCYSATVECGALRLRVVPLGIPPGTQTLFLQDN

NIARLEPGALAPLAALRRLYLHNNSLRALEAGAFRAQPRLLELALTSNRLRGLRSGAFVG

LAQLRVLYLAGNQLARLLDFTFLHLPRLQELHLQENSIELLEDQALAGLSSLALLDLSRN

QLGTISREALQPLASLQVLRLTENPWRCDCALHWLGAWIKEGGQRLLTSRDRKIMCAEPP

RLALQSLLDVSHSSLICIPPSVHVQPLELTANLGEDLRVACQASGYPQPLVTWRKVPQPR

EGRPRAQAQLEGGLLGLGGHSASDTGSGMLFLSNITLAHAGKYECEASNAGGAARVPFRL

LVNASRQQPQQPAQPPPPAARPAGSEPRPEAGSMAFRALGVATQTAIAAAIALLALTALL

LVAMICRRRRRRKKARGPPGEGALFVNDYLDGPCTFAQLEELRDERGHEMFVINRSKPLF

AEGPAEAPADCGPEQGAGPGLRVPPPVAYEIHC

>ENSP00000333227; Hs XP_294219.4; ELFN1 (release 40)

MAGRGWGALWVCVAAATLLHAGGLARADCWLIEGDKGFVWLAICSQNQPPYEAIPQQINS

TIVDLRLNENRIRSVQYASLSRFGNLTYLNLTKNEIGYIEDGAFSGQFNLQVLQLGYNRL

RNLTEGMLRGLGKLEYLYLQANLIEVVMASSFWECPNIVNIDLSMNRIQQLNSGTFAGLA

KLSVCELYSNPFYCSCELLGFLRWLAAFTNATQTYDRMQCESPPVYSGYYLLGQGRRGHR

SILSKLQSVCTEDSYAAEVVGPPRPASGRSQPGRSPPPPPPPEPSDMPCADDECFSGDGT

TPLVALPTLATQAEARPLIKVKQLTQNSATITVQLPSPFHRMYTLEHFNNSKASTVSRLT

KAQEEIRLTNLFTLTNYTYCVVSTSAGLRHNHTCLTICLPRLPSPPGPVPSPSTATHYIM

TILGCLFGMVLVLGAVYYCLRRRRRQEEKHKKAASAAAAGSLKKTIIELKYGPELEAPGL

APLSQGPLLGPEAVTRIPYLPAAGEVEQYKLVESADTPKASKGSYMEVRTGDPPERRDCE

LGRPGPDSQSSVAEISTIAKEVDKVNQIINNCIDALKSESTSFQGVKSGPVSVAEPPLVL

LSEPLAAKHGFLAPGYKDAFGHSLQRHHSVEAAGPPRASTSSSGSVRSPRAFRAEAVGVH

KAAAAEAKYIEKGSPAADAILTVTPAAAVLRAEAEKGRQYGEHRHSYPGSHPAEPPAPPG

PPPPPPHEGLGRKASILEPLTRPRPRDLAYSQLSPQYHSLSYSSSPEYTCRASQSIWERF

RLSRRRHKEEEEFMAAGHALRKKVQFAKDEDLHDILDYWKGVSAQHKS

>ENSP00000300147; Hs NP_443138.1; ELFN2 (release 40)

MLRLGLCAAALLCVCRPGAVRADCWLIEGDKGYVWLAICSQNQPPYETIPQHINSTVHDL

RLNENKLKAVLYSSLNRFGNLTDLNLTKNEISYIEDGAFLGQSSLQVLQLGYNKLSNLTE

GMLRGMSRLQFLFVQHNLIEVVTPTAFSECPSLISIDLSSNRLSRLDGATFASLASLMVC

ELAGNPFNCECDLFGFLAWLVVFNNVTKNYDRLQCESPREFAGYPLLVPRPYHSLNAITV

LQAKCRNGSLPARPVSHPTPYSTDAQREPDENSGFNPDEILSVEPPASSTTDASAGPAIK

LHHVTFTSATLVVIIPHPYSKMYILVQYNNSYFSDVMTLKNKKEIVTLDKLRAHTEYTFC

VTSLRNSRRFNHTCLTFTTRDPVPGDLAPSTSTTTHYIMTILGCLFGMVIVLGAVYYCLR

KRRMQEEKQKSVNVKKTILEMRYGADVDAGSIVHAAQKLGEPPVLPVSRMASIPSMIGEK

LPTAKGLEAGLDTPKVATKGNYIEVRTGAGGDGLARPEDDLPDLENGQGSAAEISTIAKE

VDKVNQIINNCIDALKLDSASFLGGGSSSGDPELAFECQSLPAAAAASSATGPGALERPS

FLSPPYKESSHHPLQRQLSADAAVTRKTCSVSSSGSIKSAKVFSLDVPDHPAATGLAKGD

SKYIEKGSPLNSPLDRLPLVPAGSGGGSGGGGGIHHLEVKPAYHCSEHRHSFPALYYEEG

ADSLSQRVSFLKPLTRSKRDSTYSQLSPRHYYSGYSSSPEYSSESTHKIWERFRPYKKHH

REEVYMAAGHALRKKVQFAKDEDLHDILDYWKGVSAQQKL

>ENSMUSP00000037909;Ntrk3

MDVSLCPAKCSFWRIFLLGSVWLDYVGSVLACPANCVCSKTEINCRRPDDGNLFPLLEGQ

DSGNSNGNASINITDISRNITSIHIENWRGLHTLNAVDMELYTGLQKLTIKNSGLRNIQP

RAFAKNPHLRYINLSSNRLTTLSWQLFQTLSLRELRLEQNFFNCSCDIRWMQLWQEQGEA

RLDSQSLYCISADGSQLPLFRMNISQCDLPEISVSHVNLTVREGDNAVITCNGSGSPLPD

VDWIVTGLQSINTHQTNLNWTNVHAINLTLVNVTSEDNGFTLTCIAENVVGMSNASVALT

VYYPPRVVSLVEPEVRLEHCIEFVVRGNPTPTLHWLYNGQPLRESKIIHMDYYQEGEVSE

GCLLFNKPTHYNNGNYTLIAKNALGTANQTINGHFLKEPFPESTDFFDFESDASPTPPIT

VTHKPEEDTFGVSIAVGLAAFACVLLVVLFIMINKYGRRSKFGMKGPVAVISGEEDSASP

LHHINHGITTPSSLDAGPDTVVIGMTRIPVIENPQYFRQGHNCHKPDTYVQHIKRRDIVL

KRELGEGAFGKVFLAECYNLSPTKDKMLVAVKALKDPTLAARKDFQREAELLTNLQHEHI

VKFYGVCGDGDPLIMVFEYMKHGDLNKFLRAHGPDAMILVDGQPRQAKGELGLSQMLHIA

SQIASGMVYLASQHFVHRDLATRNCLVGANLLVKIGDFGMSRDVYSTDYYRVGGHTMLPI

RWMPPESIMYRKFTTESDVWSFGVILWEIFTYGKQPWFQLSNTEVIECITQGRVLERPRV

CPKEVYDVMLGCWQREPQQRLNIKEIYKILHALGKATPIYLDILG

>ENSMUSP00000078757;Ntrk2

MSPWLKWHGPAMARLWGLCLLVLGFWRASLACPTSCKCSSARIWCTEPSPGIVAFPRLEP

NSVDPENITEILIANQKRLEIINEDDVEAYVGLRNLTIVDSGLKFVAYKAFLKNSNLRHI

NFTRNKLTSLSRRHFRHLDLSDLILTGNPFTCSCDIMWLKTLQETKSSPDTQDLYCLNES

SKNMPLANLQIPNCGLPSARLAAPNLTVEEGKSVTLSCSVGGDPLPTLYWDVGNLVSKHM

NETSHTQGSLRITNISSDDSGKQISCVAENLVGEDQDSVNLTVHFAPTITFLESPTSDHH

WCIPFTVRGNPKPALQWFYNGAILNESKYICTKIHVTNHTEYHGCLQLDNPTHMNNGDYT

LMAKNEYGKDERQISAHFMGRPGVDYETNPNYPEVLYEDWTTPTDIGDTTNKSNEIPSTD

VADQSNREHLSVYAVVVIASVVGFCLLVMLLLLKLARHSKFGMKGPASVISNDDDSASPL

HHISNGSNTPSSSEGGPDAVIIGMTKIPVIENPQYFGITNSQLKPDTFVQHIKRHNIVLK

RELGEGAFGKVFLAECYNLCPEQDKILVAVKTLKDASDNARKDFHREAELLTNLQHEHIV

KFYGVCVEGDPLIMVFEYMKHGDLNKFLRAHGPDAVLMAEGNPPTELTQSQMLHIAQQIA

AGMVYLASQHFVHRDLATRNCLVGENLLVKIGDFGMSRDVYSTDYYRVGGHTMLPIRWMP

PESIMYRKFTTESDVWSLGVVLWEIFTYGKQPWYQLSNNEVIECITQGRVLQRPRTCPQE

VYELMLGCWQREPHTRKNIKSIHTLLQNLAKASPVYLDILG

>ENSP00000277120;NTRK2

MSSWIRWHGPAMARLWGFCWLVVGFWRAAFACPTSCKCSASRIWCSDPSPGIVAFPRLEP

NSVDPENITEIFIANQKRLEIINEDDVEAYVGLRNLTIVDSGLKFVAHKAFLKNSNLQHI

NFTRNKLTSLSRKHFRHLDLSELILVGNPFTCSCDIMWIKTLQEAKSSPDTQDLYCLNES

SKNIPLANLQIPNCGLPSANLAAPNLTVEEGKSITLSCSVAGDPVPNMYWDVGNLVSKHM

NETSHTQGSLRITNISSDDSGKQISCVAENLVGEDQDSVNLTVHFAPTITFLESPTSDHH

WCIPFTVKGNPKPALQWFYNGAILNESKYICTKIHVTNHTEYHGCLQLDNPTHMNNGDYT

LIAKNEYGKDEKQISAHFMGWPGIDDGANPNYPDVIYEDYGTAANDIGDTTNRSNEIPST

DVTDKTGREHLSVYAVVVIASVVGFCLLVMLFLLKLARHSKFGMKDFSWFGFGKVKSRQG

VGPASVISNDDDSASPLHHISNGSNTPSSSEGGPDAVIIGMTKIPVIENPQYFGITNSQL

KPDTFVQHIKRHNIVLKRELGEGAFGKVFLAECYNLCPEQDKILVAVKTLKDASDNARKD

FHREAELLTNLQHEHIVKFYGVCVEGDPLIMVFEYMKHGDLNKFLRAHGPDAVLMAEGNP

PTELTQSQMLHIAQQIAAGMVYLASQHFVHRDLATRNCLVGENLLVKIGDFGMSRDVYST

DYYRVGGHTMLPIRWMPPESIMYRKFTTESDVWSLGVVLWEIFTYGKQPWYQLSNNEVIE

CITQGRVLQRPRTCPQEVYELMLGCWQREPHMRKNIKGIHTLLQNLAKASPVYLDILG

>ENSP00000351486;NTRK1

MLRGGRRGQLGWHSWAAGPGSLLAWLILASAGAAPCPDACCPHGSSGLRCTRDGALDSLH

HLPGAENLTELYIENQQHLQHLELRDLRGLGELRNLTIVKSGLRFVAPDAFHFTPRLSRL

NLSFNALESLSWKTVQGLSLQELVLSGNPLHCSCALRWLQRWEEEGLGGVPEQKLQCHGQ

GPLAHMPNASCGVPTLKVQVPNASVDVGDDVLLRCQVEGRGLEQAGWILTELEQSATVMK

SGGLPSLGLTLANVTSDLNRKNVTCWAENDVGRAEVSVQVNVSFPASVQLHTAVEMHHWC

IPFSVDGQPAPSLRWLFNGSVLNETSFIFTEFLEPAANETVRHGCLRLNQPTHVNNGNYT

LLAANPFGQASASIMAAFMDNPFEFNPEDPIPVSFSPVDTNSTSGDPVEKKDETPFGVSV

AVGLAVFACLFLSTLLLVLNKCGRRNKFGINRPAVLAPEDGLAMSLHFMTLGGSSLSPTE

GKGSGLQGHIIENPQYFSDACVHHIKRRDIVLKWELGEGAFGKVFLAECHNLLPEQDKML

VAVKALKEASESARQDFQREAELLTMLQHQHIVRFFGVCTEGRPLLMVFEYMRHGDLNRF

LRSHGPDAKLLAGGEDVAPGPLGLGQLLAVASQVAAGMVYLAGLHFVHRDLATRNCLVGQ

GLVVKIGDFGMSRDIYSTDYYRVGGRTMLPIRWMPPESILYRKFTTESDVWSFGVVLWEI

FTYGKQPWYQLSNTEAIDCITQGRELERPRACPPEVYAIMRGCWQREPQQRHSIKDVHAR

LQALAQAPPVYLDVLG

>ENSP00000354207;NTRK3

MDVSLCPAKCSFWRIFLLGSVWLDYVGSVLACPANCVCSKTEINCRRPDDGNLFPLLEGQ

DSGNSNGNASINITDISRNITSIHIENWRSLHTLNAVDMELYTGLQKLTIKNSGLRSIQP

RAFAKNPHLRYINLSSNRLTTLSWQLFQTLSLRELQLEQNFFNCSCDIRWMQLWQEQGEA

KLNSQNLYCINADGSQLPLFRMNISQCDLPEISVSHVNLTVREGDNAVITCNGSGSPLPD

VDWIVTGLQSINTHQTNLNWTNVHAINLTLVNVTSEDNGFTLTCIAENVVGMSNASVALT

VYYPPRVVSLEEPELRLEHCIEFVVRGNPPPTLHWLHNGQPLRESKIIHVEYYQEGEISE

GCLLFNKPTHYNNGNYTLIAKNPLGTANQTINGHFLKEPFPESTDNFILFDEVSPTPPIT

VTHKPEEDTFGVSIAVGLAAFACVLLVVLFVMINKYGRRSKFGMKGPVAVISGEEDSASP

LHHINHGITTPSSLDAGPDTVVIGMTRIPVIENPQYFRQGHNCHKPDTYVQHIKRRDIVL

KRELGEGAFGKVFLAECYNLSPTKDKMLVAVKALKDPTLAARKDFQREAELLTNLQHEHI

VKFYGVCGDGDPLIMVFEYMKHGDLNKFLRAHGPDAMILVDGQPRQAKGELGLSQMLHIA

SQIASGMVYLASQHFVHRDLATRNCLVGANLLVKIGDFGMSRDVYSTDYYRLFNPSGNDF

CIWCEVGGHTMLPIRWMPPESIMYRKFTTESDVWSFGVILWEIFTYGKQPWFQLSNTEVI

ECITQGRVLERPRVCPKEVYDVMLGCWQREPQQRLNIKEIYKILHALGKATPIYLDILG

>ENSP00000217939;MXRA5

MPKRAHWGALSVVLILLWGHPRVALACPHPCACYVPSEVHCTFRSLASVPAGIAKHVERI

NLGFNSIQALSETSFAGLTKLELLMIHGNEIPSIPDGALRDLSSLQVFKFSYNKLRVITG

QTLQGLSNLMRLHIDHNKIEFIHPQAFNGLTSLRLLHLEGNLLHQLHPSTFSTFTFLDYF

RLSTIRHLYLAENMVRTLPASMLRNMPLLENLYLQGNPWTCDCEMRWFLEWDAKSRGILK

CKKDKAYEGGQLCAMCFSPKKLYKHEIHKLKDMTCLKPSIESPLRQNRSRSIEEEQEQEE

DGGSQLILEKFQLPQWSISLNMTDEHGNMVNLVCDIKKPMDVYKIHLNQTDPPDIDINAT

VALDFECPMTRENYEKLWKLIAYYSEVPVKLHRELMLSKDPRVSYQYRQDADEEALYYTG

VRAQILAEPEWVMQPSIDIQLNRRQSTAKKVLLSYYTQYSQTISTKDTRQARGRSWVMIE

PSGAVQRDQTVLEGGPCQLSCNVKASESPSIFWVLPDGSILKAPMDDPDSKFSILSSGWL

RIKSMEPSDSGLYQCIAQVRDEMDRMVYRVLVQSPSTQPAEKDTVTIGKNPGESVTLPCN

ALAIPEAHLSWILPNRRIINDLANTSHVYMLPNGTLSIPKVQVSDSGYYRCVAVNQQGAD

HFTVGITVTKKGSGLPSKRGRRPGAKALSRVREDIVEDEGGSGMGDEENTSRRLLHPKDQ

EVFLKTKDDAINGDKKAKKGRRKLKLWKHSEKEPETNVAEGRRVFESRRRINMANKQINP

ERWADILAKVRGKNLPKGTEVPPLIKTTSPPSLSLEVTPPFPAISPPSASPVQTVTSAEE

SSADVPLLGEEEHVLGTISSASMGLEHNHNGVILVEPEVTSTPLEEVVDDLSEKTEEITS

TEGDLKGTAAPTLISEPYEPSPTLHTLDTVYEKPTHEETATEGWSAADVGSSPEPTSSEY

EPPLDAVSLAESEPMQYFDPDLETKSQPDEDKMKEDTFAHLTPTPTIWVNDSSTSQLFED

STIGEPGVPGQSHLQGLTDNIHLVKSSLSTQDTLLIKKGMKEMSQTLQGGNMLEGDPTHS

RSSESEGQESKSITLPDSTLGIMSSMSPVKKPAETTVGTLLDKDTTTATTTPRQKVAPSS

TMSTHPSRRRPNGRRRLRPNKFRHRHKQTPPTTFAPSETFSTQPTQAPDIKISSQVESSL

VPTAWVDNTVNTPKQLEMEKNAEPTSKGTPRRKHGKRPNKHRYTPSTVSSRASGSKPSPS

PENKHRNIVTPSSETILLPRTVSLKTEGPYDSLDYMTTTRKIYSSYPKVQETLPVTYKPT

SDGKEIKDDVATNVDKHKSDILVTGESITNAIPTSRSLVSTMGEFKEESSPVGFPGTPTW

NPSRTAQPGRLQTGIPVTTSGENLTDPPLLKELEDVDFTSEFLSSLTVSTPFHQEEAGSS

TTLSSIKVEVASSQAETTTLDQDHLETTVAILLSETRPQNHTPTAARMKEPASSSPSTIL

MSLGQTTTTKPALPSPRISQASRDSKENVFLNYVGNPETEATPVNNEGTQHMSGPNELST

PSSDQDAFNLSTKLELEKQVFGSRSLPRGPDSQRQDGRVHASHQLTRVPAKPILPTATVR

LPEMSTQSASRYFVTSQSPRHWTNKPEITTYPSGALPENKQFTTPRLSSTTIPLPLHMSK

PSIPSKFTDRRTDQFNGYSKVFGNNNIPEARNPVGKPPSPRIPHYSNGRLPFFTNKTLSF

PQLGVTRRPQIPTSPAPVMRERKVIPGSYNRIHSHSTFHLDFGPPAPPLLHTPQTTGSPS

TNLQNIPMVSSTQSSISFITSSVQSSGSFHQSSSKFFAGGPPASKFWSLGEKPQILTKSP

QTVSVTAETDTVFPCEATGKPKPFVTWTKVSTGALMTPNTRIQRFEVLKNGTLVIRKVQV

QDRGQYMCTASNLHGLDRMVVLLSVTVQQPQILASHYQDVTVYLGDTIAMECLAKGTPAP

QISWIFPDRRVWQTVSPVEGRITLHENRTLSIKEASFSDRGVYKCVASNAAGADSLAIRL

HVAALPPVIHQEKLENISLPPGLSIHIHCTAKAAPLPSVRWVLGDGTQIRPSQFLHGNLF

VFPNGTLYIRNLAPKDSGRYECVAANLVGSARRTVQLNVQRAAANARITGTSPRRTDVRY

GGTLKLDCSASGDPWPRILWRLPSKRMIDALFSFDSRIKVFANGTLVVKSVTDKDAGDYL

CVARNKVGDDYVVLKVDVVMKPAKIEHKEENDHKVFYGGDLKVDCVATGLPNPEISWSLP

DGSLVNSFMQSDDSGGRTKRYVVFNNGTLYFNEVGMREEGDYTCFAENQVGKDEMRVRVK

VVTAPATIRNKTYLAVQVPYGDVVTVACEAKGEPMPKVTWLSPTNKVIPTSSEKYQIYQD

GTLLIQKAQRSDSGNYTCLVRNSAGEDRKTVWIHVNVQPPKINGNPNPITTVREIAAGGS

RKLIDCKAEGIPTPRVLWAFPEGVVLPAPYYGNRITVHGNGSLDIRSLRKSDSVQLVCMA

RNEGGEARLILQLTVLEPMEKPIFHDPISEKITAMAGHTISLNCSAAGTPTPSLVWVLPN

GTDLQSGQQLQRFYHKADGMLHISGLSSVDAGAYRCVARNAAGHTERLVSLKVGLKPEAN

KQYHNLVSIINGETLKLPCTPPGAGQGRFSWTLPNGMHLEGPQTLGRVSLLDNGTLTVRE

ASVFDRGTYVCRMETEYGPSVTSIPVIVIAYPPRITSEPTPVIYTRPGNTVKLNCMAMGI

PKADITWELPDKSHLKAGVQARLYGNRFLHPQGSLTIQHATQRDAGFYKCMAKNILGSDS

KTTYIHVF

>ENSP00000282466;IGSF10

MKVKGRGITCLLVSFAVICLVATPGGKACPRRCACYMPTEVHCTFRYLTSIPDSIPPNVE

RINLGYNSLVRLMETDFSGLTKLELLMLHSNGIHTIPDKTFSDLQALQVLKMSYNKVRKL

QKDTFYGLRSLTRLHMDHNNIEFINPEVFYGLNFLRLVHLEGNQLTKLHPDTFVSLSYLQ

IFKISFIKFLYLSDNFLTSLPQEMVSYMPDLDSLYLHGNPWTCDCHLKWLSDWIQEKPDV

IKCKKDRSPSSAQQCPLCMNPRTSKGKPLAMVSAAAFQCAKPTIDSSLKSKSLTILEDSS

SAFISPQGFMAPFGSLTLNMTDQSGNEANMVCSIQKPSRTSPIAFTEENDYIVLNTSFST

FLVCNIDYGHIQPVWQILALYSDSPLILERSHLLSETPQLYYKYKQVAPKPEDIFTNIEA

DLRADPSWLMQDQISLQLNRTATTFSTLQIQYSSDAQITLPRAEMRPVKHKWTMISRDNN

TKLEHTVLVGGTVGLNCPGQGDPTPHVDWLLADGSKVRAPYVSEDGRILIDKSGKLELQM

ADSFDTGVYHCISSNYDDADILTYRITVVEPLVEAYQENGIHHTVFIGETLDLPCHSTGI

PDASISWVIPGNNVLYQSSRDKKVLNNGTLRILQVTPKDQGYYRCVAANPSGVDFLIFQV

SVKMKGQRPLEHDGETEGSGLDESNPIAHLKEPPGAQLRTSALMEAEVGKHTSSTSKRHN

YRELTLQRRGDSTHRRFRENRRHFPPSARRIDPQHWAALLEKAKKNAMPDKRENTTVSPP

PVVTQLPNIPGEEDDSSGMLALHEEFMVPATKALNLPARTVTADSRTISDSPMTNINYGT

EFSPVVNSQILPPEEPTDFKLSTAIKTTAMSKNINPTMSSQIQGTTNQHSSTVFPLLLGA

TEFQDSDQMGRGREHFQSRPPITVRTMIKDVNVKMLSSTTNKLLLESVNTTNSHQTSVRE

VSEPRHNHFYSHTTQILSTSTFPSDPHTAAHSQFPIPRNSTVNIPLFRRFGRQRKIGGRG

RIISPYRTPVLRRHRYSIFRSTTRGSSEKSTTAFSATVLNVTCLSCLPRERLTTATAALS

FPSAAPITFPKADIARVPSEESTTLVQNPLLLLENKPSVEKTTPTIKYFRTEISQVTPTG

AVMTYAPTSIPMEKTHKVNASYPRVSSTNEAKRDSVITSSLSGAITKPPMTIIAITRFSR

RKIPWQQNFVNNHNPKGRLRNQHKVSLQKSTAVMLPKTSPALPRDKVSPFHFTTLSTSVM

QIPSNTLTTAHHTTTKTHNPGSLPTKKELPFPPLNPMLPSIISKDSSTKSIISTQTAIPA

TTPTFPASVITYETQTERSRAQTIQREQEPQKKNRTDPNISPDQSSGFTTPTAMTPPVLT

TAETSVKPSVSAFTHSPPENTTGISSTISFHSRTLNLTDVIEELAQASTQTLKSTIASET

TLSSKSHQSTTTRKAIIRHSTIPPFLSSSATLMPVPISPPFTQRAVTDNVATPISGLMTN

TVVKLHESSRHNAKPQQLVAEVATSPKVHPNAKFTIGTTHFIYSNLLHSTPMPALTTVKS

QNSKLTPSPWAENQFWHKPYSEIAEKGKKPEVSMLATTGLSEATTLVSDWDGQKNTKKSD

FDKKPVQEATTSKLLPFDSLSRYIFEKPRIVGGKAASFTIPANSDAFLPCEAVGNPLPTI

HWTRVPSGLDLSKRKQNSRVQVLPNGTLSIQRVEIQDRGQYLCSASNLFGTDHLHVTLSV

VSYPPRILERRTKEITVHSGSTVELKCRAEGRPSPTVTWILANQTVVSESSQGSRQAVVT

VDGTLVLHNLSIYDRGFYKCVASNPGGQDSLLVKIQVIAAPPVILEQRRQVIVGTWGESL

KLPCTAKGTPQPSVYWVLSDGTEVKPLQFTNSKLFLFSNGTLYIRNLASSDRGTYECIAT

SSTGSERRVVMLTMEERVTSPRIEAASQKRTEVNFGDKLLLNCSATGEPKPQIMWRLPSK

AVVDQQHRVGSWIHVYPNGSLFIGSVTEKDSGVYLCVARNKMGDDLILMHVSLRLKPAKI

DHKQYFRKQVLHGKDFQVDCKASGSPVPEISWSLPDGTMINNAMQADDSGHRTRRYTLFN

NGTLYFNKVGVAEEGDYTCYAQNTLGKDEMKVHLTVITAAPRIRQSNKTNKRIKAGDTAV

LDCEVTGDPKPKIFWLLPSNDMISFSIDRYTFHANGSLTINKVKLLDSGEYVCVARNPSG

DDTKMYKLDVVSKPPLINGLYTNRTVIKATAVRHSKKHFDCRAEGTPSPEVMWIMPDNIF

LTAPYYGSRITVHKNGTLEIRNVRLSDSADFICVARNEGGESVLVVQLEVLEMLRRPTFR

NPFNEKIVAQLGKSTALNCSVDGNPPPEIIWILPNGTRFSNGPQSYQYLIASNGSFIISK

TTREDAGKYRCAARNKVGYIEKLVILEIGQKPVILTYAPGTVKGISGESLSLHCVSDGIP

KPNIKWTMPSGYVVDRPQINGKYILHDNGTLVIKEATAYDRGNYICKAQNSVGHTLITVP

VMIVAYPPRITNRPPRSIVTRTGAAFQLHCVALGVPKPEITWEMPDHSLLSTASKERTHG

SEQLHLQGTLVIQNPQTSDSGIYKCTAKNPLGSDYAATYIQVI

>ENSMUSP00000037096;Lrrn1

MARLSTGKAACQVVLGLLITSLTESSILTSECPQLCVCEIRPWFTPQSTYREATTVDCND

LRLTRIPGNLSSDTQVLLLQSNNIAKTVDELQQLFNLTELDFSQNNFTNIKEVGLANLTQ

LTTLHLEENQISEMTDYCLQDLSNLQELYINHNQISTISANAFSGLKNLLRLHLNSNKLK

VIDSRWFDSTPNLEILMIGENPVIGILDMNFRPLSNLRSLVLAGMYLTDVPGNALVGLDS

LESLSFYDNKLIKVPQLALQKVPNLKFLDLNKNPIHKIQEGDFKNMLRLKELGINNMGEL

VSVDRYALDNLPELTKLEATNNPKLSYIHRLAFRSVPALESLMLNNNALNAVYQKTVESL

PNLREISIHSNPLRCDCVIHWINSNKTNIRFMEPLSMFCAMPPEYRGQQVKEVLIQDSSE

QCLPMISHDTFPNHLNMDIGTTLFLDCRAMAEPEPEIYWVTPIGNKITVETLSDKYKLSS

EGTLEIANIQIEDSGRYTCVAQNVQGADTRVATIKVNGTLLDGAQVLKIYVKQTESHSIL

VSWKVNSNVMTSNLKWSSATMKIDNPHITYTARVPVDVHEYNLTHLQPSTDYEVCLTVSN

IHQQTQKSCVNVTTKTAAFALDISDHETSTALAAVMGSMFAVISLASIAIYIAKRFKRKN

YHHSLKKYMQKTSSIPLNELYPPLINLWEADSDKDKDGSADTKPTQVDTSRSYYMW

>ENSMUSP00000027706;Lrrn2

MRLLVAALLLSWVAGTTAAAPVVPWRVPCPPQCACQIRPWYTPRSSYREATTVDCNDLFL

TAVPPRLPAGTQTLLLQSNSISRIDQTELAYLANLTELDLSQNSFSDARDCDFQALPQLL

SLHLEENRLNRLEDHSFAGLTSLQELYLNHNQLCRISPRAFAGLGNLLRLHLNSNLLRTI

DSRWFEMLPNLEILMIGGNKVDAILDMNFRPLANLRSLVLAGMSLREISDYALEGLQSLE

SLSFYDNQLAQVPKRALEQVPGLKFLDLNKNPLQRVGPGDFANMLHLKELGLNNMEELVS

IDKFALVNLPELTKLDITNNPRLSFIHPRAFHHLPQMETLMLNNNALSALHQQTVESLPN

LQEVGLHGNPIRCDCVIRWANATGTHVRFIEPQSTLCAEPPDLQRRPVREVPFREMTDHC

LPLISPRSFPSSLQIASGESTVLHCRALAEPEPEIYWVTPAGVRLTPARSGRRYRVFPEG

TLELRRVTAEEAGLYTCVAQNLVGADTKTVSVVVGHAPFQPGRDKGPGLKLHVQETHPYH

ILLFWAPPPNIVSTNLTWSSASSLRDHEAPALARLPRGTHRYNITRLLPATEYWACLQVA

FADAHTQLACVWARTKEASPCHRALGDRPGLIAILALAVLLLAAGLAAHLGRGQSKQGVV

GEKPLLPVWAFWGWSAPSVRVVSAPLVLPWNPGRKQPRCQMGRGCHHHCLNIAEVPPGLS

SREITRTTLH

>ENSMUSP00000043818;Lrrn3

MKDTPLQVHVLLGLAITTLVQAIDKKVDCPQLCTCEIRPWFTPRSIYMEASTVDCNDLGL

LNFPARLPADTQILLLQTNNIARIEHSTDFPVNLTGLDLSQNNLSSVTNINVQKMSQLLS

VYLEENKLTELPEKCLYGLSNLQELYVNHNLLSTISPGAFIGLHNLLRLHLNSNRLQMIN

SQWFDALPNLEILMLGDNPIIRIKDMNFQPLVKLRSLVIAGINLTEIPDDALAGLENLES

ISFYDNRLSKVPQVALQKAVNLKFLDLNKNPINRIRRGDFSNMLHLKELGINNMPELVSI

DSLAVDNLPDLRKIEATNNPRLSYIHPNAFFRLPKLESLMLNTNALSALYHGTIESLPNL

KEISIHSNPIRCDCVIRWINMNKTNIRFMEPDSLFCVDPPEFQGQNVRQVHFRDMMEICL

PLIAPESFPSDLDVEADSYVSLHCRATAEPQPEIYWITPSGKKLLPNTMREKFYVHSEGT

LEIRGITPKEGGLYTCIATNLVGADLKSIMIKVGGSVPQDNNGSLNIKIRDIRANSVLVS

WKASSKILKSSVKWTAFVKTEDSHAAQSARIPFDVKVYNLTHLKPSTEYKICIDIPTVYQ

KSRKQCVNVTTKSLEHDGKEYGKNHTVFVACVGGLLGIIGVMCLFSCVSQEGSSEGEHSY

AVNHCHKPALAFSELYPPLINLWESSKEKRATLEVKATAIGVPTNMS

>ENSP00000312001;LRRN3

MKDMPLRIHVLLGLAITTLVQAVDKKVDCPRLCTCEIRPWFTPRSIYMEASTVDCNDLGL

LTFPARLPANTQILLLQTNNIAKIEYSTDFPVNLTGLDLSQNNLSSVTNINVKKMPQLLS

VYLEENKLTELPEKCLSELSNLQELYINHNLLSTISPGAFIGLHNLLRLHLNSNRLQMIN

SKWFDALPNLEILMIGENPIIRIKDMNFKPLINLRSLVIAGINLTEIPDNALVGLENLES

ISFYDNRLIKVPHVALQKVVNLKFLDLNKNPINRIRRGDFSNMLHLKELGINNMPELISI

DSLAVDNLPDLRKIEATNNPRLSYIHPNAFFRLPKLESLMLNSNALSALYHGTIESLPNL

KEISIHSNPIRCDCVIRWMNMNKTNIRFMEPDSLFCVDPPEFQGQNVRQVHFRDMMEICL

PLIAPESFPSNLNVEAGSYVSFHCRATAEPQPEIYWITPSGQKLLPNTLTDKFYVHSEGT

LDINGVTPKEGGLYTCIATNLVGADLKSVMIKVDGSFPQDNNGSLNIKIRDIQANSVLVS

WKASSKILKSSVKWTAFVKTENSHAAQSARIPSDVKVYNLTHLNPSTEYKICIDIPTIYQ

KNRKKCVNVTTKGLHPDQKEYEKNNTTTLMACLGGLLGIIGVICLISCLSPEMNCDGGHS

YVRNYLQKPTFALGELYPPLINLWEAGKEKSTSLKVKATVIGLPTNMS

>ENSP00000319223;LRRN5

MRLLVAPLLLAWVAGATAAVPVVPWHVPCPPQCACQIRPWYTPRSSYREATTVDCNDLFL

TAVPPALPAGTQTLLLQSNSIVRVDQSELGYLANLTELDLSQNSFSDARDCDFHALPQLL

SLHLEENQLTRLEDHSFAGLASLQELYLNHNQLYRIAPRAFSGLSNLLRLHLNSNLLRAI

DSRWFEMLPNLEILMIGGNKVDAILDMNFRPLANLRSLVLAGMNLREISDYALEGLQSLE

SLSFYDNQLARVPRRALEQVPGLKFLDLNKNPLQRVGPGDFANMLHLKELGLNNMEELVS

IDKFALVNLPELTKLDITNNPRLSFIHPRAFHHLPQMETLMLNNNALSALHQQTVESLPN

LQEVGLHGNPIRCDCVIRWANATGTRVRFIEPQSTLCAEPPDLQRLPVREVPFREMTDHC

LPLISPRSFPPSLQVASGESMVLHCRALAEPEPEIYWVTPAGLRLTPAHAGRRYRVYPEG

TLELRRVTAEEAGLYTCVAQNLVGADTKTVSVVVGRALLQPGRDEGQGLELRVQETHPYH

ILLSWVTPPNTVSTNLTWSSASSLRGQGATALARLPRGTHSYNITRLLQATEYWACLQVA

FADAHTQLACVWARTKEATSCHRALGDRPGLIAILALAVLLLAAGLAAHLGTGQPRKGVG

GRRPLPPAWAFWGWSAPSVRVVSAPLVLPWNPGRKLPRSSEGETLLPPLSQNS

>ENSMUSP00000059050;Lrrn6a

MLAGGMRSMPSPLLACWQPILLLVLGSVLSGSATGCPPRCECSAQDRAVLCHRKRFVAVP

EGIPTETRLLDLGKNRIKTLNQDEFASFPHLEELELNENIVSAVEPGAFNNLFNLRTLGL

RSNRLKLIPLGVFTGLSNLTKLDISENKIVILLDYMFQDLYNLKSLEVGDNDLVYISHRA

FSGLNSLEQLTLEKCNLTSIPTEALSHLHGLIVLRLRHLNINAIRDYSFKRLYRLKVLEI

SHWPYLDTMTPNCLYGLNLTSLSITHCNLTAVPYLAVRHLVYLRFLNLSYNPIGTIEGSM

LHELLRLQEIQLVGGQLAVVEPYAFRGLNYLRVLNVSGNQLTTLEESAFHSVGNLETLIL

DSNPLACDCRLLWVFRRRWRLNFNRQQPTCATPEFVQGKEFKDFPDVLLPNYFTCRRAHI

RDRKAQQVFVDEGHTVQFVCRADGDPPPAILWLSPRKHLVSAKSNGRLTVFPDGTLEVRY

AQVQDNGTYLCIAANAGGNDSMPAHLHVRSYSPDWPHQPNKTFAFISNQPGEGEANSTRA

TVPFPFDIKTLIIATTMGFISFLGVVLFCLVLLFLWSRGKGNTKHNIEIEYVPRKSDAGI

SSADAPRKFNMKMI

>ENSMUSP00000054960;Lrrn6b

MTCWLHMLGLHLLLLPTAPLAAGCPARCECSASTRTVACGRRRLTAIPEGIPAETRMLEL

SRNRIRCLNPGDLASLPTLEELDLNHNVIAHVEPGAFANLPRLRVLRLRGNQLKLIPPGV

FTHLDSLTLLDLSENKLVILLDFSFQDLRSLQRLEVGDNDLVFISRRAFAGLLGLAELTL

ERCNLTSLSPESLGHLRGLGALRLRHLAIAALEDQNFQKLPGLSHLEIDNWPLLEEVAPG

SLRGLNLTSLSITHTNITAVPAAALRQQAHLTCLNLSHNPISMVPRGSFRDLVRLRELHL

AGALLAVIEPQAFVGLRQIRLLNLSDNLLSTLEENTFHSVNTLETLRVDGNPLACDCRLL

WIVQRRKTLNFDGRLPACATPAEVRGDALHNLPDSVLFEYFVCRKPKIRERRLQHVTATE

GDDVRFLCRAEGEPAPTVAWVTPQHHSVTAASRGRARVLPGGTLTIADTRPQDSGTYTCV

ASNAGGNDTYFATLTVQPAANRTQGDGHNETQVGVRFPLDLTTILVSTAMGCITFLGVVL

FCFLLLFVWSRGRGQHKNNFSVEYSFRKVDGPAAAAGQGGARKFNMKMI

>ENSMUSP00000069772;Lrrn6c

MLHTAIPCWQPFLGLAVVLLLMGSTIGCPARCECSAQNKSVSCHRRRLLAIPEGIPIETK

ILDLSKNRLKSINPEEFISYPLLEEIDLSDNIIANVEPGAFNNLFNLRSLRLKGNRLKLV

PLGVFTGLSNLTKLDISENKIVILLDYMFQDLHNLKSLEVGDNDLVYISHRAFSGLLSLE

QLTLEKCNLTAVPTEALSHLRSLIALHLKHLNINNMPVYAFKRLFHLKNLEIDYWPLLDL

MPANSLYGLNLTSLSITNTNLSTVPFLAFKHLVYLTHLNLSYNPISTIEAGMFSDLIRLQ

ELHIVGAQLRTIEPHSFQGLRFLRVLNVSQNLLETLEENVFSSPRALEVLSINNNPLACD

CRLLWLLQRQPNLQFGGQQPMCAGPDTIRERSFKDFHSTALSFYFTCKKPKIREKKLQHL

LVDEGQTVQLECNADGDPQPVISWVTPRRRFITTKSNGRATVLGDGTLEIRFAQDQDSGM

YVCIASNAAGNDTFTASLTVKGFTSDRFLYANRTPMYMTDSNDTVSNGTNANTFSLDLKT

ILVSTAMGCFTFLGVVLFCFLLLFVWSRGKGKHKNSIDLEYVPRKNNGAVVEGEVAGPRR

FNMKMI

>ENSP00000310126;LRRN6C

MLHTAISCWQPFLGLAVVLIFMGSTIGCPARCECSAQNKSVSCHRRRLIAIPEGIPIETK

ILDLSKNRLKSVNPEEFISYPLLEEIDLSDNIIANVEPGAFNNLFNLRSLRLKGNRLKLV

PLGVFTGLSNLTKLDISENKIVILLDYMFQDLHNLKSLEVGDNDLVYISHRAFSGLLSLE

QLTLEKCNLTAVPTEALSHLRSLISLHLKHLNINNMPVYAFKRLFHLKHLEIDYWPLLDM

MPANSLYGLNLTSLSVTNTNLSTVPFLAFKHLVYLTHLNLSYNPISTIEAGMFSDLIRLQ

ELHIVGAQLRTIEPHSFQGLRFLRVLNVSQNLLETLEENVFSSPRALEVLSINNNPLACD

CRLLWILQRQPTLQFGGQQPMCAGPDTIRERSFKDFHSTALSFYFTCKKPKIREKKLQHL

LVDEGQTVQLECSADGDPQPVISWVTPRRRFITTKSNGRATVLGDGTLEIRFAQDQDSGM

YVCIASNAAGNDTFTASLTVKGFASDRFLYANRTPMYMTDSNDTISNGTNANTFSLDLKT

ILVSTAMGCFTFLGVVLFCFLLLFVWSRGKGKHKNSIDLEYVPRKNNGAVVEGEVAGPRR

FNMKMI

>ENSMUSP00000032105;Lrig1

MARPGPGVLGAPRLAPRLLLWLLLLLLQWPESAGAQAGPRAPCAAACTCAGDSLDCSGRG

LATLPRDLPSWTRSLNLSYNRLSEIDSAAFEDLTNLQEVYLNSNELTAIPSLGAASIGVV

SLFLQHNKILSVDGSQLKSYLSLEVLDLSSNNITEIRSSCFPNGLRIRELNLASNRISIL

ESGAFDGLSRSLLTLRLSKNRITQLPVKAFKLPRLTQLDLNRNRIRLIEGLTFQGLDSLE

VLRLQRNNISRLTDGAFWGLSKMHVLHLEYNSLVEVNSGSLYGLTALHQLHLSNNSISRI

QRDGWSFCQKLHELILSFNNLTRLDEESLAELSSLSILRLSHNAISHIAEGAFKGLKSLR

VLDLDHNEISGTIEDTSGAFTGLDNLSKLTLFGNKIKSVAKRAFSGLESLEHLNLGENAI

RSVQFDAFAKMKNLKELYISSESFLCDCQLKWLPPWLMGRMLQAFVTATCAHPESLKGQS

IFSVLPDSFVCDDFPKPQIITQPETTMAVVGKDIRFTCSAASSSSSPMTFAWKKDNEVLA

NADMENFAHVRAQDGEVMEYTTILHLRHVTFGHEGRYQCIITNHFGSTYSHKARLTVNVL

PSFTKIPHDIAIRTGTTARLECAATGHPNPQIAWQKDGGTDFPAARERRMHVMPDDDVFF

ITDVKIDDMGVYSCTAQNSAGSVSANATLTVLETPSLAVPLEDRVVTVGETVAFQCKATG

SPTPRITWLKGGRPLSLTERHHFTPGNQLLVVQNVMIDDAGRYTCEMSNPLGTERAHSQL

SILPTPGCRKDGTTVGIFTIAVVCSIVLTSLVWVCIIYQTRKKSEEYSVTNTDETIVPPD

VPSYLSSQGTLSDRQETVVRTEGGHQANGHIESNGVCLRDPSLFPEVDIHSTTCRQPKLC

VGYTREPWKVTEKADRTAAPHTTAHSGSAVCSDCSTDTAYHPQPVPRDSGQPGTASSQEL

RQHDREYSPHHPYSGTADGSHTLSGGSLYPSNHDRILPSLKNKAASADGNGDSSWTLAKL

HEADCIDLKPSPTLASGSPELMEDAISTEAQHLLVSNGHLPKACDSSPESVPLKGQITGK

RRGPLLLAPRS

>ENSP00000273261;LRIG1

MARPVRGGLGAPRRSPCLLLLWLLLLRLEPVTAAAGPRAPCAAACTCAGDSLDCGGRGLA

ALPGDLPSWTRSLNLSYNKLSEIDPAGFEDLPNLQEVYLNNNELTAVPSLGAASSHVVSL

FLQHNKIRSVEGSQLKAYLSLEVLDLSLNNITEVRNTCFPHGPPIKELNLAGNRIGTLEL

GAFDGLSRSLLTLRLSKNRITQLPVRAFKLPRLTQLDLNRNRIRLIEGLTFQGLNSLEVL

KLQRNNISKLTDGAFWGLSKMHVLHLEYNSLVEVNSGSLYGLTALHQLHLSNNSIARIHR

KGWSFCQKLHELVLSFNNLTRLDEESLAELSSLSVLRLSHNSISHIAEGAFKGLRSLRVL

DLDHNEISGTIEDTSGAFSGLDSLSKLTLFGNKIKSVAKRAFSGLEGLEHLNLGGNAIRS

VQFDAFVKMKNLKELHISSDSFLCDCQLKWLPPWLIGRMLQAFVTATCAHPESLKGQSIF

SVPPESFVCDDFLKPQIITQPETTMAMVGKDIRFTCSAASSSSSPMTFAWKKDNEVLTNA

DMENFVHVHAQDGEVMEYTTILHLRQVTFGHEGRYQCVITNHFGSTYSHKARLTVNVLPS

FTKTPHDITIRTTTMARLECAATGHPNPQIAWQKDGGTDFPAARERRMHVMPDDDVFFIT

DVKIDDAGVYSCTAQNSAGSISANATLTVLETPSLVVPLEDRVVSVGETVALQCKATGNP

PPRITWFKGDRPLSLTERHHLTPDNQLLVVQNVVAEDAGRYTCEMSNTLGTERAHSQLSV

LPAAGCRKDGTTVGIFTIAVVSSIVLTSLVWVCIIYQTRKKSEEYSVTNTDETVVPPDVP

SYLSSQGTLSDRQETVVRTEGGPQANGHIESNGVCPRDASHFPEPDTHSVACRQPKLCAG

SAYHKEPWKAMEKAEGTPGPHKMEHGGRVVCSDCNTEVDCYSRGQAFHPQPVSRDSAQPS

APNGPEPGGSDQEHSPHHQCSRTAAGSCPECQGSLYPSNHDRMLTAVKKKPMASLDGKGD

SSWTLARLYHPDSTELQPASSLTSGSPERAEAQYLLVSNGHLPKACDASPESTPLTGQLP

GKQRVPLLLAPKS

>ENSMUSP00000074360;Lrig3

MGAPGLRAATAALGLLLCAGLGRAGPAGSGGHGAPGQLLDDDAQRPCPAACHCLGDLLDC

SRRRLVRLPDPLPAWVTRLDLSHNRLSFIQTSSLSHLQSLQEVKLNNNELETIPNLGSIS

ANIRQLSLAGNAIDKILPEQLEAFQSLETLDLSNNNISELRTAFPPLQLKYLYINNNRVS

SMEPGYFDNLASTLLVLKLNRNRISAIPPKMFKLPQLQHLELNRNKIKNVDGLTFQGLGA

LKSLKMQRNGVTKLMDGAFWGLSNMEVLQLDHNNLTEITKGWLYGLLMLRELHLSQNAIN

RISPDAWEFCQKLSELDLTFNHLSRLDDSSFLGLSLLNALHIGNNKVSYIADCAFRGLTS

LKTLDLRNNEISWTIEDMSGAFSGLDRLRQLILQGNRIRSITKKAFAGLDTLEHLDLSGN

AIMSLQSNAFSQMKKLQQLHLNTSSLLCDCQLRWLPQWVAENNFQSLTKWICCLPLFTYA

QSFGCCSSLRQLLYDFPKPQITVQPETQSAIKGSDVSFTCSAASSSDSPMTFAWKKDNEA

LQDAEMENYAHLRAQGGELMEYTTILRLRNVEFTSEGKYQCVISNHFGSSYSVKAKLTIN

MLPSFTKTPMDLTIRAGAMARLECAAVGHPAPQIAWQKDGGTDFPAARERRMHVMPEDDV

FFIVDVKIEDIGVYSCTAQNSAGSVSANATLTVLETPSFLRPLLDRTVTKGETAVLQCIA

GGSPPPRLNWTKDDSPLVVTERHFFAAGNQLLIIVDSDVSDAGKYTCEMSNTLGTERGNV

RLSVIPTPTCDSPHMTAPSLDGDGWATVGVVIIAVVCCVVGTSLVWVVIIYHTRRRNEDC

SITNTDETNLPADIPSYLSSQGTLADRQDGYISSESGSHHQFVTSSGGGFFLPQHDGAGT

CHFDDSSEADVEAASDPFLCPFVGSTGPVYLQGNLYSPDPFEVYLPGCSSDPRTALMDHC

ESSYVKQDRFSCARPSEEPCERSLKSIPWPHSRKLTDSTYPPNEGHTVQTLCLNKSSVDF

STGPEPGSATSSNSFMGTFGKPLRRPHLDAFSSSAQPPDCQPRPCHGKSLSSPELDSESE

ENDKERTDFREENHRCTYQQIFHTYRTPDCQPCDSDT

>ENSP00000326759;LRIG3

MSAPSLRARAAGLGLLLCAVLGRAGRSDSGGRGELGQPSGVAAERPCPTTCRCLGDLLDC

SRKRLARLPEPLPSWVARLDLSHNRLSFIKASSMSHLQSLREVKLNNNELETIPNLGPVS

ANITLLSLAGNRIVEILPEHLKEFQSLETLDLSSNNISELQTAFPALQLKYLYLNSNRVT

SMEPGYFDNLANTLLVLKLNRNRISAIPPKMFKLPQLQHLELNRNKIKNVDGLTFQGLGA

LKSLKMQRNGVTKLMDGAFWGLSNMEILQLDHNNLTEITKGWLYGLLMLQELHLSQNAIN

RISPDAWEFCQKLSELDLTFNHLSRLDDSSFLGLSLLNTLHIGNNRVSYIADCAFRGLSS

LKTLDLKNNEISWTIEDMNGAFSGLDKLRRLILQGNRIRSITKKAFTGLDALEHLDLSDN

AIMSLQGNAFSQMKKLQQLHLNTSSLLCDCQLKWLPQWVAENNFQSFVNASCAHPQLLKG

RSIFAVSPDGFVCDDFPKPQITVQPETQSAIKGSNLSFICSAASSSDSPMTFAWKKDNEL

LHDAEMENYAHLRAQGGEVMEYTTILRLREVEFASEGKYQCVISNHFGSSYSVKAKLTVN

MLPSFTKTPMDLTIRAGAMARLECAAVGHPAPQIAWQKDGGTDFPAARERRMHVMPEDDV

FFIVDVKIEDIGVYSCTAQNSAGSISANATLTVLETPSFLRPLLDRTVTKGETAVLQCIA

GGSPPPKLNWTKDDSPLVVTERHFFAAGNQLLIIVDSDVSDAGKYTCEMSNTLGTERGNV

RLSVIPTPTCDSPQMTAPSLDDDGWATVGVVIIAVVCCVVGTSLVWVVIIYHTRRRNEDC

SITNTDETNLPADIPSYLSSQGTLADRQDGYVSSESGSHHQFVTSSGAGFFLPQHDSSGT

CHIDNSSEADVEAATDLFLCPFLGSTGPMYLKGNVYGSDPFETYHTGCSPDPRTVLMDHY

EPSYIKKKECYPCSHPSEESCERSFSNISWPSHVRKLLNTSYSHNEGPGMKNLCLNKSSL

DFSANPEPASVASSNSFMGTFGKALRRPHLDAYSSFGQPSDCQPRAFYLKAHSSPDLDSG

SEEDGKERTDFQEENHICTFKQTLENYRTPNFQSYDLDT

>CG8434-PA;lbk

MSWSRRRRRNNNNNNNSSSNNNNAASESRSNDNCNYSTTNDFNKINKLLLITYMLLITAA

TICQSAAQSPSSSNGNGVGGSGSTGGSPLSALLKSGGASYNQQLPQQQLFAMLTRNGGGS

SGPRSSGGNRGYTSARQHFMALSEDYSDADVDEEDMAALHYPLSASHPPAGVQGSSSSSG

ISIQSGSLETNGFVADDGGQYEHAQKALEAAAAAAKASNKYNIDCPKDCKCLNVLFDCDK

LHLERVPVLPSYVQTLHLANNKLNDTTVLEIRNLLNLTKVSLKRNLLEVIPKFIGLSGLK

HLVLANNHITSISSESLAALPLLRTLDLSRNKLHTIELNSFPKSNNLVHLILSFNEITNV

NEHSFATLNNLTDLELSNNRLSTLPIRVFKNLNQLKKLALNFNQLEINWSTFRGLESMKN

LQLKSNKIRALQDGVFYVMHKIETIDLAMNQISSLSRQGLFNLTKLRHLNLSFNAISRIE

VDTWEFTQSLEVLDLSNNAINEFKPQHLDCLHRLKTLNLAHNRLQYLQENTFDCVKNLEE

LNLRRNRLSWIIEDQSAAAPFKGLRKLRRLDLHGNNLKQISTKAMSGLNNLEILNLGSNA

LASIQVNAFEHMLRLNKLVFKSLNFICDCDLVWFQQWLKNRFPQQAEHAVCGYPEHLLDR

HLKSLSSSELVCVDSPKPRVEQEPDDMLAVNAANITLECIASSPTAASLAAADELKIKWR

HDNQHVQERPAVHDGASTETQIRHDLSTNQTSIYGYLRLTNVTYESAGRYQCVVSNAFGT

TYAQKFKISIGIHPTFLQVPSNLTLDAGEMARLVCSASGDPTPEIALQKFGGSEFPAATE

RRLQVIREENAFLITNAKPSDSGIYTCTALSAAGEIKVNATLVVNDKPQPSIPLVHQEVV

VGRTCVLQCLSETANADFELEHPHREWFKENKPIHISPTAPDGDQLVVVKENLNWVVLLV

GIITVTVICVVVDCCIIWCTLRYQKKKLRMSLAAERHSTQLRPRSLADLGCSYDEANHRR

LTVMSTPPSEQRCLEQGLTLSYLRQTDLEAQQDHLSSKDSGTGSDAAVKRTLDDFEVAMP

SHKHHTDDEEEEEVEEVYEENEPPYIKHTYEVNYAEPEQHLFLQNNNHNYDGGGIGVAGG

VNKMVPNALLRKCSAGASGSNYSSIQQCTTVDI

>ENSMUSP00000037616;Lrfn3

MAVLPLLLCLLPLAPASSPPQPAISSPCPRRCRCQTQSMPLSVLCPGAGLLFVPPSLDRR

AAELRLADNFIAAVRRRDLANMTGLLHLSLSRNTIRHVAAGAFADLRALRALHLDGNRLT

SLGEGQLRGLVNLRHLILSNNQLAALAAGALDDCAETLEDLDLSYNNLEQLPWEALGRLG

NVNTLGLDHNLLASVPAGAFSRLHKLARLDMTSNRLTTIPPDPLFSRLPLLARPRGSPAS

ALVLAFGGNPLHCNCELVWLRRLAREDDLEACASPPALGGRYFWAVGEEEFVCEPPVVTH

RSPPLAVPAGRPAALRCRAVGDPEPRVRWVSPQGRLLGNSSRARAFPNGTLELLVTEPED

GGTFTCIAANAAGEATAAVELTVGPPPPPQLANSTSCDPPRDGEPDALTPPSAASASAKV

ADTVAPTDRGVQVTEHGATAALVQWPDQRPVPGIRMYQIQYNSSADDILVYRMIPADSRS

FLLTDLASGRTYDLCVLAVYEDSATGLTATRPVGCARFSTEPALRPCAAPHAPFLGGTMI

IALGGVIVASVLVFIFVLLLRYKVHGGQPPGKAKATAPVSSVCSQTNGALGPVPSAPAPE

PAAPRAHTVVQLDCEPWGPSHEPAGP

>ENSP00000246529;LRFN3

MAILPLLLCLLPLAPASSPPQSATPSPCPRRCRCQTQSLPLSVLCPGAGLLFVPPSLDRR

AAELRLADNFIASVRRRDLANMTGLLHLSLSRNTIRHVAAGAFADLRALRALHLDGNRLT

SLGEGQLRGLVNLRHLILSNNQLAALAAGALDDCAETLEDLDLSYNNLEQLPWEALGRLG

NVNTLGLDHNLLASVPAGAFSRLHKLARLDMTSNRLTTIPPDPLFSRLPLLARPRGSPAS

ALVLAFGGNPLHCNCELVWLRRLAREDDLEACASPPALGGRYFWAVGEEEFVCEPPVVTH

RSPPLAVPAGRPAALRCRAVGDPEPRVRWVSPQGRLLGNSSRARAFPNGTLELLVTEPGD

GGIFTCIAANAAGEATAAVELTVGPPPPPQLANSTSCDPPRDGDPDALTPPSAASASAKV

ADTGPPTDRGVQVTEHGATAALVQWPDQRPIPGIRMYQIQYNSSADDILVYRMIPAESRS

FLLTDLASGRTYDLCVLAVYEDSATGLTATRPVGCARFSTEPALRPCGAPHAPFLGGTMI

IALGGVIVASVLVFIFVLLMRYKVHGGQPPGKAKIPAPVSSVCSQTNGALGPTPTPAPPA

PEPAALRAHTVVQLDCEPWGPGHEPVGP

>ENSMUSP00000050039; LRFN4

MAPPLLLLLLASGAAACPLPCVCQNLSESLSTLCAHRGLLFVPPNVDRRTVELRLADNFI

QALGPPDFRNMTGLVDLTLSRNAITRIGARSFGDLESLRSLHLDGNRLVELGSSSLRGPV

NLQHLILSGNQLGRIAPGAFDDFLDSLEDLDVSYNNLRQVPWAGIGSMPALHTLNLDHNL

IDALPPGVFAQLSQLSRLDLTSNRLATLAPDPLFSRGRDAEASPSPLVLSFSGNPLHCNC

ELLWLRRLARPDDLETCASPPTLAGRYFWAVPEGEFSCEPPLIARHTQRLWVLEGQRATL

RCRALGDPVPTMHWVGPDDRLVGNSSRAWAFPNGTLEIGVTGAGDAGAYTCIATNPAGEA

TARVELRVLALPHGGNTSAEGGRPGPSDIAASARTAAEGEGTLESEPAVQVTEVTATSGL

VSWGLGRPADPVWMFQIQYNSSEDETLIYRIVPASSHHFLLKHLVPGADYDLCLLALSPA

AGPSDLTATRLLGCAHFSTLPATPLCHALQAHVLGGTLTVAVGGVLVAALLVFTVALLVR

GRGAGNGRLPLKLSHVQSQTNGGTSPMPKSHPPRSPPPRPQRSCSLDLGDTGGCYGYARR

LGGAWARRSHSVHGGLLGAGCRGVGGSAERLEESVV

>ENSP00000312535;LRFN4

MAPPLLLLLLASGAAACPLPCVCQNLSESLSTLCAHRGLLFVPPNVDRRTVELRLADNFI

QALGPPDFRNMTGLVDLTLSRNAITRIGARAFGDLESLRSLHLDGNRLVELGTGSLRGPV

NLQHLILSGNQLGRIAPGAFDDFLESLEDLDLSYNNLRQVPWAGIGAMPALHTLNLDHNL

IDALPPGAFAQLGQLSRLDLTSNRLATLAPDPLFSRGRDAEASPAPLVLSFSGNPLHCNC

ELLWLRRLARPDDLETCASPPGLAGRYFWAVPEGEFSCEPPLIARHTQRLWVLEGQRATL

RCRALGDPAPTMHWVGPDDRLVGNSSRARAFPNGTLEIGVTGAGDAGGYTCIATNPAGEA

TARVELRVLALPHGGNSSAEGGRPGPSDIAASARTAAEGEGTLESEPAVQVTEVTATSGL

VSWGPGRPADPVWMFQIQYNSSEDETLIYRIVPASSHHFLLKHLVPGADYDLCLLALSPA

AGPSDLTATRLLGCAHFSTLPASPLCHALQAHVLGGTLTVAVGGVLVAALLVFTVALLVR

GRGAGNGRLPLKLSHVQSQTNGGPSPTPKAHPPRSPPPRPQRSCSLDLGDAGCYGYARRL

GGAWARRSHSVHGGLLGAGCRGVGGSAERLEESVV

>ENSMUSP00000051546;Lrfn5

MEKFLFYLFLIGIAVRAQICPKRCVCQILSPNLATLCAKKGLLFVPPNIDRRTVELRLAD

NFVTNIKRKDFANMTSLVDLTLSRNTISFITPHAFADLRNLRALHLNSNRLTKITNDMFS

GLSNLHHLILNNNQLTLISSTAFDDVFALEELDLSYNNLETIPWDAVEKMVSLHTLSLDH

NMIDNIPKGTFSHLHKMTRLDVTSNKLQKLPPDPLFQRAQVLATSGIISPSTFALSFGGN

PLHCNCELLWLRRLSREDDLETCASPALLTGRYFWSIPEEEFLCEPPLITRHTHEMRVLE

GQRATLRCKARGDPEPAIHWISPEGKLISNATRSLVYDNGTLDILITTVKDTGAFTCIAS

NPAGEATQTVDLHIIKLPHLLNSTNHIHEPDPGSSDISTSTKSGSNASSSNGDTKMSQDK

IVVAEATSSTALLKFNFQRNIPGIRMFQIQYNGTYDDTLVYRMIPPTSKTFLVNNLASGT

MYDLCVLAIYDDGITSLTATRVVGCIQFTTEQDYVRCHFMQSQFLGGTMIIIIGGIIVAS

VLVFIIILMIRYKVCNNNGQHKVTKVSNVYSQTNGAQMQGCSVTLPQSMSKQAMGHEENA

QCCKVASDNAIQSSETCSSQDSSTTTSALPPTWTSSAPVSQKQKRKTGTKPSAEPQSEAV

TNVESQNTNRNNSTALQLASCPPDSVTEGPTSQRAHTKPSKFLTVPAEGSRARHRASLSG

GLKDSFHYGNSQLSLKRSMSMNAMWT

>ENSP00000298119;LRFN5

MEKILFYLFLIGIAVKAQICPKRCVCQILSPNLATLCAKKGLLFVPPNIDRRTVELRLAD

NFVTNIKRKDFANMTSLVDLTLSRNTISFITPHAFADLRNLRALHLNSNRLTKITNDMFS

GLSNLHHLILNNNQLTLISSTAFDDVFALEELDLSYNNLETIPWDAVEKMVSLHTLSLDH

NMIDNIPKGTFSHLHKMTRLDVTSNKLQKLPPDPLFQRAQVLATSGIISPSTFALSFGGN

PLHCNCELLWLRRLSREDDLETCASPPLLTGRYFWSIPEEEFLCEPPLITRHTHEMRVLE

GQRATLRCKARGDPEPAIHWISPEGKLISNATRSLVYDNGTLDILITTVKDTGAFTCIAS

NPAGEATQIVDLHIIKLPHLLNSTNHIHEPDPGSSDISTSTKSGSNTSSSNGDTKLSQDK

IVVAEATSSTALLKFNFQRNIPGIRMFQIQYNGTYDDTLVYRMIPPTSKTFLVNNLAAGT

MYDLCVLAIYDDGITSLTATRVVGCIQFTTEQDYVRCHFMQSQFLGGTMIIIIGGIIVAS

VLVFIIILMIRYKVCNNNGQHKVTKVSNVYSQTNGAQIQGCSVTLPQSVSKQAVGHEENA

QCCKATSDNVIQSSETCSSQDSSTTTSALPPSWTSSTSVSQKQKRKTGTKPSTEPQNEAV

TNVESQNTNRNNSTALQLASRPPDSVTEGPTSKRAHIKPNALLTNVDQIVQETQRLELI

>ENSMUSP00000062158;Lrrc4

MKLLWQVTVHHTWNAVLLPVVYLTAQVWILCAAIAAAASAGPQNCPSVCSCSNQFSKVVC

TRRGLSEVPQGIPSNTRYLNLMENNIQMIQADTFRHLHHLEVLQLGRNSIRQIEVGAFNG

LASLNTLELFDNWLTVIPSGAFEYLSKLRELWLRNNPIESIPSYAFNRVPSLMRLDLGEL

KKLEYISEGAFEGLFNLKYLNLGMCNIKDMPNLTPLVGLEELEMSGNHFPEIRPGSFHGL

SSLKKLWVMNSQVSLIERNAFDGLASLVELNLAHNNLSSLPHDLFTPLRYLVELHLHHNP

WNCDCDILWLAWWLREYIPTNSTCCGRCHAPMHMRGRYLVEVDQAAFQCSAPFIMDAPRD

LNISEDRMAELKCRTPPMSSVKWLLPNGTVLSHASRHPRISVLNDGTLNFSRVLLIDTGV

YTCMVTNVAGNSNASAYLNVSSAELNTPNFSFFTTVTVETTEISPEDITRKYKPVPTTST

GYQPAYTTSTTVLIQTTRVPKQVPVPSTDTTDKMQTSLDEVMKTTKIIIGCFVAVTLLAA

AMLIVFYKLRKRHQQRSTVTAARTVEIIQVDEDIPAAAPAAATAAPSGVSGEGAVVLPTI

HDHINYNTYKPAHGAHWTENSLGNSLHPTVTTISEPYIIQTHTKDKVQETQI

>ENSP00000249363;LRRC4

MKLLWQVTVHHHTWNAILLPFVYLTAQVWILCAAIAAAASAGPQNCPSVCSCSNQFSKVV

CTRRGLSEVPQGIPSNTRYLNLMENNIQMIQADTFRHLHHLEVLQLGRNSIRQIEVGAFN

GLASLNTLELFDNWLTVIPSGAFEYLSKLRELWLRNNPIESIPSYAFNRVPSLMRLDLGE

LKKLEYISEGAFEGLFNLKYLNLGMCNIKDMPNLTPLVGLEELEMSGNHFPEIRPGSFHG

LSSLKKLWVMNSQVSLIERNAFDGLASLVELNLAHNNLSSLPHDLFTPLRYLVELHLHHN

PWNCDCDILWLAWWLREYIPTNSTCCGRCHAPMHMRGRYLVEVDQASFQCSAPFIMDAPR

DLNISEGRMAELKCRTPPMSSVKWLLPNGTVLSHASRHPRISVLNDGTLNFSHVLLSDTG

VYTCMVTNVAGNSNASAYLNVSTAELNTSNYSFFTTVTVETTEISPEDTTRKYKPVPTTS

TGYQPAYTTSTTVLIQTTRVPKQVAVPATDTTDKMQTSLDEVMKTTKIIIGCFVAVTLLA

AAMLIVFYKLRKRHQQRSTVTAARTVEIIQVDEDIPAATSAAATAAPSGVSGEGAVVLPT

IHDHINYNTYKPAHGAHWTENSLGNSLHPTVTTISEPYIIQTHTKDKVQETQI

>ENSMUSP00000053840;Lrrc4c

MLNKMTLHPQQIMIGPRFNRALFDPLLVVLLALQLLVVAGLVRAQTCPSVCSCSNQFSKV

ICVRKNLREVPDGISTNTRLLNLHENQIQIIKVNSFKHLRHLEILQLSRNHIRTIEIGAF

NGLANLNTLELFDNRLTTIPNGAFVYLSKLKELWLRNNPIESIPSYAFNRIPSLRRLDLG

ELKRLSYISEGAFEGLSNLRYLNLAMCNLREIPNLTPLIKLDELDLSGNHLSAIRPGSFQ

GLMHLQKLWMIQSQIQVIERNAFDNLQSLVEINLAHNNLTLLPHDLFTPLHHLERIHLHH

NPWNCNCDILWLSWWIRDMAPSNTACCARCNTPPNLKGRYIGELDQNYFTCYAPVIVEPP

ADLNVTEGMAAELKCRASTSLTSVSWITPNGTVMTHGAYKVRIAVLSDGTLNFTNVTVQD

TGMYTCMVSNSVGNTTASATLNVTAATTTPFSYFSTVTVETMEPSQDEARTTDNNVGPTP

VIDWETTNVTTSLTPQSTRSTEKTFTIPVTDINSGIPGIDEVMKTTKIIIGCFVAITLMA

AVMLVIFYKMRKQHHRQNHHAPTRTVEIINVDDEITGDTPMESHLPMPAIEHEHLNHYNS

YKSPFNHTTTVNTINSIHSSVHEPLLIRMNSKDNVQETQI

>ENSP00000278198;LRRC4C

MLNKMTLHPQQIMIGPRFNRALFDPLLVVLLALQLLVVAGLVRAQTCPSVCSCSNQFSKV

ICVRKNLREVPDGISTNTRLLNLHENQIQIIKVNSFKHLRHLEILQLSRNHIRTIEIGAF

NGLANLNTLELFDNRLTTIPNGAFVYLSKLKELWLRNNPIESIPSYAFNRIPSLRRLDLG

ELKRLSYISEGAFEGLSNLRYLNLAMCNLREIPNLTPLIKLDELDLSGNHLSAIRPGSFQ

GLMHLQKLWMIQSQIQVIERNAFDNLQSLVEINLAHNNLTLLPHDLFTPLHHLERIHLHH

NPWNCNCDILWLSWWIKDMAPSNTACCARCNTPPNLKGRYIGELDQNYFTCYAPVIVEPP

ADLNVTEGMAAELKCRASTSLTSVSWITPNGTVMTHGAYKVRIAVLSDGTLNFTNVTVQD

TGMYTCMVSNSVGNTTASATLNVTAATTTPFSYFSTVTVETMEPSQDEARTTDNNVGPTP

VVDWETTNVTTSLTPQSTRSTEKTFTIPVTDINSGIPGIDEVMKTTKIIIGCFVAITLMA

AVMLVIFYKMRKQHHRQNHHAPTRTVEIINVDDEITGDTPMESHLPMPAIEHEHLNHYNS

YKSPFNHTTTVNTINSIHSSVHEPLLIRMNSKDNVQETQI

>ENSP00000252804;PXDN

SRPWWLRASERPSAPSAMAKRSRGPGRRCLLALVLFCAWGTLAVVAQKPGAGCPSRCLCF

RTTVRCMHLLLEAVPAVAPQTSILDLRFNRIREIQPGAFRRLRNLNTLLLNNNQIKRIPS

GAFEDLENLKYLYLYKNEIQSIDRQAFKGLASLEQLYLHFNQIETLDPDSFQHLPKLERL

FLHNNRITHLVPGTFNHLESMKRLRLDSNTLHCDCEILWLADLLKTYAESGNAQAAAICE

YPRRIQGRSVATITPEELNCERPRITSEPQDADVTSGNTVYFTCRAEGNPKPEIIWLRNN

NELSMKTDSRLNLLDDGTLMIQNTQETDQGIYQCMAKNVAGEVKTQEVTLRYFGSPARPT

FVIQPQNTEVLVGESVTLECSATGHPPPRISWTRGDRTPLPVDPRVNITPSGGLYIQNVV

QGDSGEYACSATNNIDSVHATAFIIVQALPQFTVTPQDRVVIEGQTVDFQCEAKGNPPPV

IAWTKGGSQLSVDRRHLVLSSGTLRISGVALHDQGQYECQAVNIIGSQKVVAHLTVQPRV

TPVFASIPSDTTVEVGANVQLPCSSQGEPEPAITWNKDGVQVTESGKFHISPEGFLTIND

VGPADAGRYECVARNTIGSASVSMVLSVNVPDVSRNGDPFVATSIVEAIATVDRAINSTR

THLFDSRPRSPNDLLALFRYPRDPYTVEQARAGEIFERTLQLIQEHVQHGLMVDLNGTSY

HYNDLVSPQYLNLIANLSGCTAHRRVNNCSDMCFHQKYRTHDGTCNNLQHPMWGASLTAF

ERLLKSVYENGFNTPRGINPHRLYNGHALPMPRLVSTTLIGTETVTPDEQFTHMLMQWGQ

FLDHDLDSTVVALSQARFSDGQHCSNVCSNDPPCFSVMIPPNDSRARSGARCMFFVRSSP

VCGSGMTSLLMNSVYPREQINQLTSYIDASNVYGSTEHEARSIRDLASHRGLLRQGIVQR

SGKPLLPFATGPPTECMRDENESPIPCFLAGDHRANEQLGLTSMHTLWFREHNRIATELL

KLNPHWDGDTIYYETRKIVGAEIQHITYQHWLPKILGEVGMRTLGEYHGYDPGINAGIFN

AFATAAFRFGHTLVNPLLYRLDENFQPIAQDHLPLHKAFFSPFRIVNEGGIDPLLRGLFG

VAGKMRVPSQLLNTELTERLFSMAHTVALDLAAINIQRGRDHGIPPYHDYRVYCNLSAAH

TFEDLKNEIKNPEIREKLKRLYGSTLNIDLFPALVVEDLVPGSRLGPTLMCLLSTQFKRL

RDGDRLWYENPGVFSPAQLTQIKQTSLARILCDNADNITRVQSDVFRVAEFPHGYGSCDE

IPRVDLRVWQDCCEDCRTRGQFNAFSYHFRGRRSLEFSYQEDKPTKKTRPRKIPSVGRQG

EHLSNSTSAFSTRSDASGTNDFREFVLEMQKTITDLRTQIKKLESRLSTTECVDAGGESH

ANNTKWKKDACTICECKDGQVTCFVEACPPATCAVPVNIPGACCPVCLQKRAEEKP

>CG12002-PA;Pxn

MRFMLLMLQLLGLLLLLAGGVQSVYCPAGCTCLERTVRCIRAKLSAVPKLPQDTQTLDLR

FNHIEELPANAFSGLAQLTTLFLNDNELAYLQDGALNGLTALRFVYLNNNRLSRLPATIF

QRMPRLEAIFLENNDIWQLPAGLFDNLPRLNRLIMYNNKLTQLPVDGFNRLNNLKRLRLD

GNAIDCNCGVYSLWRRWHLDVQRQLVSISLTCAAPQMLQNQGFSSLGEHHFKCAKPQFLV

APQDAQVAAGEQVELSCEVTGLPRPQITWMHNTQELGLEEQAQAEILPSGSLLIRSADTS

DMGIYQCIARNEMGALRSQPVRLVVNGGNHPLDSPIDARSNQVWADAGTPMHGATPLPSP

LPSPPHFTHQPHDQIVALHGSGHVLLDCAASGWPQPDIQWFVNGRQLLQSTPSLQLQANG

SLILLQPNQLSAGTYRCEARNSLGSVQATARIELKELPEILTAPQSQTIKLGKAFVLECD

ADGNPLPTIDWQLNGVPLPGNTPDLQLENENTELVVGAARQEHAGVYRCTAHNENGETSV

EATIKVERSQSPPQLAIEPSNLVAITGTTIELPCQADQPEDGLQISWRHDGRLIDPNVQL

AEKYQISGAGSLFVKNVTIPDGGRYECQLKNQFGRASASALVTIRNNVDLAPGDRYVRIA

FAEAAKEIDLAINNTLDMLFSNRSDKAPPNYGELLRVFRFPTGEARQLARAAEIYERTLV

NIRKHVQEGDNLTMKSEEYEFRDLLSREHLHLVAELSGCMEHREMPNCTDMCFHSRYRSI

DGTCNNLQHPTWGASLTAFRRLAPPIYENGFSMPVGWTKGMLYSGHAKPSARLVSTSLVA

TKEITPDARITHMVMQWGQFLDHDLDHAIPSVSSESWDGIDCKKSCEMAPPCYPIEVPPN

DPRVRNRRCIDVVRSSAICGSGMTSLFFDSVQHREQINQLTSYIDASQVYGYSTAFAQEL

RNLTSQEGLLRVGVHFPRQKDMLPFAAPQDGMDCRRNLDENTMSCFVSGDIRVNEQVGLL

AMHTIWMREHNRIASKLKQINSHWDGDTLYQEARKIVGAQMQHITFKQWLPLIIGESGME

MMGEYQGYNPQLNPSIANEFATAALRFGHTIINPILHRLNETFQPIPQGHLLLHKAFFAP

WRLAYEGGVDPLMRGFLAVPAKLKTPDQNLNTELTEKLFQTAHAVALDLAAINIQRGRDH

GMPGYNVYRKLCNLTVAQDFEDLAGEISSAEIRQKMKELYGHPDNVDVWLGGILEDQVEG

GKVGPLFQCLLVEQFRRLRDGDRLYYENPGVFSPEQLTQIKQANFGRVLCDVGDNFDQVT

ENVFILAKHQGGYKKCEDIIGINLYLWQECGRCNSPPAIFDSYIPQTYTKRSNRQKRDLG

KENDEVATAESYDSPLESLYDVNEERVSGLEELIGSFQKELKKLHKKLRKLEDSCNSADS

EPVAQVVQLAAAPPQLVSKPKRSHCVDDKGTTRLNNEVWSPDVCTKCNCFHGQVNCLRER

CGEVSCPPGVDPLTPPEACCPHCPMVK

>K09C8.5;Pxn-2

MLLEFLLLIGISLSTACPSECRCAGLDVHCEGKNLTAIPGHIPIATTNLYFSNNLLNSLS

KSNFQALPNLQYLDLSNNSIRDIEETLLDSFPGLKYLDLSWNKIRYVPKLSTAPNALVSL

NLVHNEISRLDNDLVSHSPYMQTFLIQRNRIQSLPHDFFNSRMVPTLKTVKMAGNPWSCD

CRMVNVKQFADSLFAHSNQNIFIVGKCFFPKGLRNYVFRNLSIENLECEKPEYSKTDDGM

FKMSCPNNEMEGYHYDSIFLENNKEARHTAHFARDKDGSLLSNGQFTRNYQCAFYRQKQS

IHMQKKMQASSSTEPPITTTTMEPMTTSTMDSMDTTESVVTMTTMPEIDTKIVFEHKQLD

TTSRDGETLELKCEASGEPTPTITWLFEKQKLTESRKHKLTKNGSVLKIFPFLNTDIGQY

ECVASNGEESKSHIFSVSLKESEQPVIIDAPMDTNATIGQQVTLRCNAKGFPVPDVVWLF

EGIRIPRRNTRYTISDNNIELTIEKVTRHDSGVFTCQAVNSVGSAVATANLLVGAELTEK

VDKLLDDSTIEKIAKEAKQKVEKALSSTKDQQRMDKIESPNDLSKLFKFAINLKKVDLGK

AREIYEESIRLVQMHIDNGLAFESAMISPNVSYEAVLPVSYVQTLMEKSGCQTGQFAESC

EDHCFFSKYRSYDGQCNNHEHPWWGVSEMAFMRLLPPRYENGFNTPVGWEKGKRYNGYEV

PNARKVSRVLIGTDETTPHSHLSAMTMQWGQFIDHDLTLTAPALTRHSYKEGAFCNRTCE

NADPCFNIQLEADDPKLHTGLYQKHPCMEFERNGAACGSGETSPIFQRVTYRDQLNLLTS

YLDASGIYGNSEEQALELRDLYSDHGLLRFDIVSGANKPYMPFEKDSDMDCRRNFSRENP

IKCFLAGDVRANEQLGLMSMHTIFLREHNRIASRLLEVNENWDGETIFQETRKLIGAMLQ

HITYNAWLPKILGKATYNTIIGEYKGYNPDVNPTIANEFATAALRFAHTLINTHLFRFDK

DFKETKQGHLPLHNAFFAPERLVSEGGVDPLLRGLFAAPIKMPRPDQVLNKELTEKLFNR

FHEVALDLAALNIQRGRDHGLPSWTEYRKFCNLTVPKTWSDMKNIVQNDTVISKLQSLYG

VTENIDLWVGGVTEKRTADALMGPTLACIIADQFKRLRDGDRFWYENEEMFSKAQLRQIK

KVTLSKIICTNGDDIDRIQRDIFVYHGNSTQFYEPCESLPEINLNMWTTCCDAMCSSSST

LARNAIGGDEKAKRRKRRHHHSKKSCHDKGKRRKSGDRWNHSNDICVECMCHDGEVWCKT

NNFCKSQV

>ENSMUSP00000062171;Flrt2

MGLQTTKWPGRGAFILKFWLIISLGLYLQVSKLLACPSVCRCDRNFVYCNERSLTSVPLG

IPEGVTVLYLHNNQINNAGFPAELHNVQSVHTVYLYGNQLDEFPMNLPKNVRVLHLQENN

IQTISRAALAQLLKLEELHLDDNSISTVGVEDGAFREAISLKLLFLSKNHLSSVPVGLPV

DLQELRVDENRIAVISDMAFQNLTSLERLIVDGNLLTNKGIAEGTFSHLTKLKEFSIVRN

SLSHPPPDLPGTHLIRLYLQDNQINHIPLTAFANLRKLERLDISNNQLRMLTQGVFDHLS

NLKQLTARNNPWFCDCSIKWVTEWLKYIPSSLNVRGFMCQGPEQVRGMAVRELNMNLLSC

PTTTPGLPVFTPAPSTVSPTTQSPTLSVPSPSRGSVPPAPTPSKLPTIPDWDGRERVTPP

ISERIQLSIHFVNDTSIQVSWLSLFTVMAYKLTWVKMGHSLVGGIVQERIVSGEKQHLSL

VNLEPRSTYRICLVPLDAFNYRTVEDTICSEATTHASYLNNGSNTASSHEQTTSHSMGSP

FLLAGLIGGAVIFVLVVLLSVFCWHMHKKGRYTSQKWKYNRGRRKDDYCEAGTKKDNSIL

EMTETSFQIVSLNNDQLLKGDFRLQPIYTPNGGINYTDCHIPNNMRYCNSSVPDLEHCHT

>ENSP00000332879;FLRT2

MGLQTTKWPSHGAFFLKSWLIISLGLYSQVSKLLACPSVCRCDRNFVYCNERSLTSVPLG

IPEGVTVLYLHNNQINNAGFPAELHNVQSVHTVYLYGNQLDEFPMNLPKNVRVLHLQENN

IQTISRAALAQLLKLEELHLDDNSISTVGVEDGAFREAISLKLLFLSKNHLSSVPVGLPV

DLQELRVDENRIAVISDMAFQNLTSLERLIVDGNLLTNKGIAEGTFSHLTKLKEFSIVRN

SLSHPPPDLPGTHLIRLYLQDNQINHIPLTAFSNLRKLERLDISNNQLRMLTQGVFDNLS

NLKQLTARNNPWFCDCSIKWVTEWLKYIPSSLNVRGFMCQGPEQVRGMAVRELNMNLLSC

PTTTPGLPLFTPAPSTASPTTQPPTLSIPNPSRSYTPPTPTTSKLPTIPDWDGRERVTPP

ISERIQLSIHFVNDTSIQVSWLSLFTVMAYKLTWVKMGHSLVGGIVQERIVSGEKQHLSL

VNLEPRSTYRICLVPLDAFNYRAVEDTICSEATTHASYLNNGSNTASSHEQTTSHSMGSP

FLLAGLIGGAVIFVLVVLLSVFCWHMHKKGRYTSQKWKYNRGRRKDDYCEAGTKKDNSIL

EMTETSFQIVSLNNDQLLKGDFRLQPIYTPNGGINYTDCHIPNNMRYCNSSVPDLEHCHT

>ENSP00000246047;FLRT3

MISAAWSIFLIGTKIGLFLQVAPLSVMAKSCPSVCRCDAGFIYCNDRFLTSIPTGIPEDA

TTLYLQNNQINNAGIPSDLKNLLKVERIYLYHNSLDEFPTNLPKYVKELHLQENNIRTIT

YDSLSKIPYLEELHLDDNSVSAVSIEEGAFRDSNYLRLLFLSRNHLSTIPWGLPRTIEEL

RLDDNRISTISSPSLQGLTSLKRLVLDGNLLNNHGLGDKVFFNLVNLTELSLVRNSLTAA

PVNLPGTNLRKLYLQDNHINRVPPNAFSYLRQLYRLDMSNNNLSNLPQGIFDDLDNITQL

ILRNNPWYCGCKMKWVRDWLQSLPVKVNVRGLMCQAPEKVRGMAIKDLNAELFDCKDSGI

VSTIQITTAIPNTVYPAQGQWPAPVTKQPDIKNPKLTKDHQTTGSPSRKTITITVKSVTS

DTIHISWKLALPMTALRLSWLKLGHSPAFGSITETIVTGERSEYLVTALEPDSPYKVCMV

PMETSNLYLFDETPVCIETETAPLRMYNPTTTLNREQEKEPYKNPNLPLAAIIGGAVALV

TIALLALVCWYVHRNGSLFSRNCAYSKGRRRKDDYAEAGTKKDNSILEIRETSFQMLPIS

NEPISKEEFVIHTIFPPNGMNLYKNNHSESSSNRSYRDSGIPDSDHSHS

>CG15744-PA; CG15744-PA

MPTATATSTAAEGGQAVQVQTHQDTELPQNPETGIATGQGTASSCPRKCSCRSTAENIHS

LKIRCDEQQITNWRELDFGEDVTSIVSINASKNSIALITAEDFRNFTELKRLDLSFNLLT

ELDKDTFGDSLAHLEKLKLAGNAISHIYEGTFDQMPKLKLLDLSGNPLACDCGLIWLIAW

SSSREVRLQPPPKCESPGNFRGMPLKKLRVGKDFHCETLLQPLLELIPSQNQVAFEGDEL

QLKCHAPRVAIGVPRESEDLPTKAYVFWGWSEKIRAKNSTEDIIYQDPTKVFGDVNLETR

HSTDSGILQSILRIASLTQNHTGMWDCTLRSQQANLSQAIVLHVVAKGTLYCEARVVHTN

KGTYHWPRTMRGETVLQECVEEPSDATQARRASHECGPSGEWLNLDTESCVYVSETTRIL

EQFAKVNLTLTKGQNALEIARRLHNFTQAQTQLNRIRDPMDLEYIARTLVKYLDQLEQPQ

QQQEISHLLMDIVSQLLNLPAHLFRAAQSEQGTGQRLLHVVESSAMRLALASTQAEPLPA

EMIPWRGSLAQQRNLFVEFFNISLDAFVSLSCVWLEQSPRGFQCNSANDTIPMYEHGDID

AAIQLPYSVIGNSSTTLPATTTIRSLRLMISLHRNGKLLPNLRGSHNESLSSAIIGILAY

SSDGEALQFRADNELDPEEDVYQQRVTVMLRAHPYHNPLSAPQPAWWDADEQRWETSVCQ

QHYQHRTLVMFSCSRTGYYGLLQRSQYLNDFRSEESGARFRHPPAAVYAGCGLLFACCAF

NAVTFAVFGRAVRINRVQRHALVNTWLALGALALAFSLGIYQTASQPQCRLLGLLMHYLG

LCVLLWVCVSLSSMYKRLTKTTTSGQGQCPGQDMEPQRERERKPILGIYLVGWGIALLIC

GISSAVNLAEYATYDYCFLHSSTTLNALLVPAVILVIFCGILALCIYYQLSQQAVNVLQL

QMQHQQNRQYSDNNTQATEHIDLDWLDANGSATTAAIGGGSGNHGGRKEQDHMQEQYSTL

SNPLSSIVDDFERSNLSHLRGHFIFLVLYAGAWLSAAAYVNGGQELYVLSFAGCCSVLGI

FLLIFYNLSRNDARQAWSQGRDGRSIPAKLVTYNNGSQARGAHPSSMMPGPGTAMISNSI

VAYKANPGPGSLYEANNSAGSRSNSQCSRSMRSQTRSQTRSQQEQLLQANGAGGVTIINN

TSGQPGAGGGPAAGGVSGSTGGAPVPPHSLNALLHGSSHDLIPSAEIFYNPNQINVARKF

FKKQKRLAKRNNFELQRQTMPQMQLQMQMQMQLHSQHSLSDASSEQLYSRHHNAMTMLAG

GSKINNTNLHYKNQGSPMAGAPKEHGNMGAMSSGGDSMQFKRFVPASASSASKIMQANIY

TNIPETLTPQHEVIKLRANGRTRTPSLLDETLHELDDDEEDEEEEEHGQTPATMEEPEHE

LEEEDASSLDEHAPLYANTLPPASGMSSLFRNRGSSHQPMSSTPVKQPSLEAMNSLGLPE

VSLEEPLLQKHEIYVSNSLQVTTSNSIQLDDDFPSVLIRFSQQQQSKSLNNISEMLAGGG

GGGGNAPGLDSLGDQQESSQLSVNEGSTLEEQQLRQIYSCSSSNLSQLKGHHPTATVDTE

DDGRLLSGSPTNESDLNYQNSEISIRSHGLYAPQADNDLNLTLTDDFRCYQSSNASDADV

DVLNEFDDEFVAATGGERVVGDAEQDPHHDHDQDTSIDELYEAIKCRSPLRNKQEAVERF

ERERERDREKEMEMEAKPLSNSHNENLNETIEDDSSQSSVISYIDPRAANEPRPFPS

>ENSP00000316119;AMIGO1

MHPHRDPRGLWLLLPSLSLLLFEVARAGRAVVSCPAACLCASNILSCSKQQLPNVPHSLP

SYTALLDLSHNNLSRLRAEWTPTRLTQLHSLLLSHNHLNFISSEAFSPVPNLRYLDLSSN

QLRTLDEFLFSDLQVLEVLLLYNNHIMAVDRCAFDDMAQLQKLYLSQNQISRFPLELVKE

GAKLPKLTLLDLSSNKLKNLPLPDLQKLPAWIKNGLYLHNNPLNCDCELYQLFSHWQYRQ

LSSVMDFQEDLYCMNSKKLHNVFNLSFLNCGEYKERAWEAHLGDTLIIKCDTKQQGMTKV

WVTPSNERVLDEVTNGTVSVSKDGSLLFQQVQVEDGGVYTCYAMGETFNETLSVELKVHN

FTLHGHHDTLNTAYTTLVGCILSVVLVLIYLYLTPCRCWCRGVEKPSSHQGDSLSSSMLS

TTPNHDPMAGGDKDDGFDRRVAFLEPAGPGQGQNGKLKPGNTLPVPEATGKGQRRMSDPE

SVSSVFSDTPIVV

>ENSMUSP00000059913;Amigo2

MSLRFHTLPTLPRAVKPGCRELLCLLVIAVMVSPSASGMCPTACICATDIVSCTNKNLSK

VPGNLFRLIKRLDLSYNRIGLLDADWIPVSFVKLSTLILRHNNITSISTGSFSTTPNLKC

LDLSSNRLKSVKSATFQELKALEVLLLYNNHISYLDPAAFGGLSHLQKLYLSGNFLTQFP

MDLYTGRFKLADLTFLDVSYNRIPSIPMHHINLVPGRQLRGIYLHGNPFVCDCSLYSLLI

FWYRRHFSSVMDFKNDYTCRLWSDSRHSHQLQLLQESFLNCSYSVINGSFHALGFIHEAQ

VGERAIVHCDSKTGNGNTDFIWVGPDNRLLEPDKDMGNFRVFYNGSLVIENPGFEDAGVY

SCIAMNRQRLLNETVDIMINVSNFTINRSHAHEAFNTAFTTLAACVASIVLVLLYLYLTP

CPCKCKAKRQKNTLSQSSAHSSILSPGPTGDASADDRKAGKRVVFLEPLKDTAAGQNGKV

KLFPSETVIAEGILKSTRAKSDSDSVNSVFSDTPFVAST

>ENSP00000266581;AMIGO2

MSLRVHTLPTLLGAVVRPGCRELLCLLMITVTVGPGASGVCPTACICATDIVSCTNKNLS

KVPGNLFRLIKRLDLSYNRIGLLDSEWIPVSFAKLNTLILRHNNITSISTGSFSTTPNLK

CLDLSSNKLKTVKNAVFQELKVLEVLLLYNNHISYLDPSAFGGLSQLQKLYLSGNFLTQF

PMDLYVGRFKLAELMFLDVSYNRIPSMPMHHINLVPGKQLRGIYLHGNPFVCDCSLYSLL

VFWYRRHFSSVMDFKNDYTCRLWSDSRHSRQVLLLQDSFMNCSDSIINGSFRALGFIHEA

QVGERLMVHCDSKTGNANTDFIWVGPDNRLLEPDKEMENFYVFHNGSLVIESPRFEDAGV

YSCIAMNKQRLLNETVDVTINVSNFTVSRSHAHEAFNTAFTTLAACVASIVLVLLYLYLT

PCPCKCKTKRQKNMLHQSNAHSSILSPGPASDASADERKAGAGKRVVFLEPLKDTAAGQN

GKVRLFPSEAVIAEGILKSTRGKSDSDSVNSVFSDTPFVAST

>ENSMUSP00000035831;Lrrc21

MLWLLALGGPHQAWGFCPSECSCSLRILSDGSKARTVVCSDPDLTLPPASIPPDTCKLRL

ERTAIRRVPGETFRPLSRLEQLWLPYNALSELSALMLRGLRRLRELRLPGNRLVTFPWAA

LRDTPQLQLLDLQANRLSTLPPEAAHFLENLTFLDLSNNQLMRLPEELLDVWAHLKTGPF

LSGHHARLILGLQDNPWVCDCRLYDLVHLLDGWVSSNLIFIEARLRCASPRSLAGVAFSQ

LELRKCQSPELRPGVTSIISPLGSTVLLRCGATGIPGPEMSWRRANGRPLNGTVHQEVSS

DGSSWTLLDLPVVSLFDSGDYICQAKNFLGASETLISLIVTEPQTSTGYSGIPGVLWART

GEGAEAAAYNNKLVARHVPHMPEHVALATKPSMPSIKEELALQNFQMDVPGEFSREPSEH

QEAQMVRSLKVVGDTYHSVSLVWKAPQAGNTTAFSVLYAVFGHRDMRRMTVEPGKTSVTI

EGLAPKTKYVACVCVRGLVPTKEQCVIFSTDEVVDAEGTQRLINMVVISVAAIIALPPTL

LVCCGALRRRCHKCRTGGSAEASGAYVNLERLGHSEDSSEVLSRSSLSEGDRLLSARSSL

DSQVLGVRGGRRINEYFC

>ENSP00000277778;LRRC21

MRVALGMLWLLALAWPPQARGFCPSQCSCSLHIMGDGSKARTVVCNDPDMTLPPASIPPD

TSRLRLERTAIRRVPGEAFRPLGRLEQLWLPYNALSELNALMLRGLRRLRELRLPGNRLA

AFPWAALRDAPKLRLLDLQANRLSAVPAEAARFLENLTFLDLSSNQLMRLPQELIVSWAH

LETGIFPPGHHPRRVLGLQDNPWACDCRLYDLVHLLDGWAPNLAFIETELRCASPRSLAG

VAFSQLELRKCQGPELHPGVASIRSLLGGTALLRCGATGVPGPEMSWRRANGRPLNGTVH

QEVSSDGTSWTLLGLPAVSHLDSGDYICQAKNFLGASETVISLIVTEPPTSTEHSGSPGA

LWARTGGGGEAAAYNNKLVARHVPQIPKPAVLATGPSVPSTKEELTLEHFQMDALGELSD

GRAGPSEARMVRSVKVVGDTYHSVSLVWKAPQAKNTTAFSVLYAVFGQHSMRRVIVQPGK

TRVTITGLLPKTKYVACVCVQGLVPRKEQCVIFSTNEVVDAENTQQLINVVVISVAIVIA

LPLTLLVCCSALQKRCRKCFNKDSTEATVTYVNLERLGYSEDGLEELSRHSVSEADRLLS

ARSSVDFQAFGVKGGRRINEYFC

>ENSMUSP00000056642;Lrrc22

MAFVFYCFLQVLVSWVIHAVQPFCLPECTCSEESFGRSLQCMSMSLGKIPDNFPEELKQV

RIENSPLFELSQGFFTNMSSLEYLWLNFNNVTVIHLGALEDLPELRELRLEGNKLRSVPW

TAFRATPLLRVLDLKHNRIDSVPELALQFLTNLIYLDISSNRLTVVSKGVFLNWPAYQKR

QQLGCGAEFLSNMVLSLHNNPWLCDCRLRGLAQFVKSVGPPFILVNSYLVCQGPVSKAGQ

LLHETELGVCMKPTISTPSVNVTIQVGKNVTLQCFAQASPSPTIAWKYPLSTWREFDVLA

SPIAEGIILSQLVIPAAQLVDGGNYTCMAFNSIGRSSLVILLYVQPAQAMPGLHFLSTSS

EVSAYVDLRVVKQTVHGILLQWLTVTNLAEEQWFTLYITSDEALRKKVVHIGPGINTYAV

DDLLPATKYKACLSLRNQPPSQGQCVVFVTGKDSGGLEGREHLLHVTVVLCAVLLALPVG

AYVWVSQGPYNFSEWCWRRCPLHRKTLRCPQAVPQCKDNSFKDPSGVYEDGESHRVMEED

EEVEKEGIS

>ENSMUSP00000045142;Islr

MRALCLLCWAVLLNLVRACPEPCDCGEKYGFQIADCAYRDLEGVPPGFPANVTTLSLSAN

RLPGLPEGAFREVPLLQSLWLAHNEIRSVAIGALAPLSHLKSLDLSHNLLSEFAWSDLHN

LSALQLLKMDSNELAFIPRDAFSSLSALRSLQLNHNRLHALAEGTFAPLTALSHLQINDN

PFDCTCGIVWFKTWALASAVSIPEQDNIACTTPHVLKGIPLGRLPPLPCSAPSVQLSYQP

SQDGAELRPGFVLALHCDVDGQPVPQLHWHIHTPGGTVEIASPNVGTDGRALPGALATSG

QPRFQAFANGSLLIPDFGKLEEGTYSCLATNELGSAESSVNVALATPGEGGEDAVGHKFH

GKAVEGKGCYTVDNEVQPSGPEDNVVIIYLSRAGPPEAAIAADGRPAQQFSGILLLGQSL

LVLSFFYF

>ENSP00000249842;ISLR

MQELHLLWWALLLGLAQACPEPCDCGEKYGFQIADCAYRDLESVPPGFPANVTTLSLSAN

RLPGLPEGAFREVPLLQSLWLAHNEIRTVAAGALASLSHLKSLDLSHNLISDFAWSDLHN

LSALQLLKMDSNELTFIPRDAFRSLRALRSLQLNHNRLHTLAEGTFTPLTALSHLQINEN

PFDCTCGIVWLKTWALTTAVSIPEQDNIACTSPHVLKGTPLSRLPPLPCSAPSVQLSYQP

SQDGAELRPGFVLALHCDVDGQPAPQLHWHIQIPSGIVEITSPNVGTDGRALPGTPVASS

QPRFQAFANGSLLIPDFGKLEEGTYSCLATNELGSAESSVDVALATPGEGGEDTLGRRFH

GKAVEGKGCYTVDNEVQPSGPEDNVVIIYLSRAGNPEAAVAEGVPGQLPPGLLLLGQSLL

LFFFLTSF

>ENSMUSP00000055604;Islr2

MGPFGALCLAWALLGVVRACPEPCACVDKYAHQFADCAYKELREVPEGLPANVTTLSLSA

NKITVLRRGAFVNVTQVTSLWLAHSEVRTVESGALAVLSQLKNLDLSHNLISNFPWSDLR

NLSALQLLKMNHNRLGSLPRDALGALPDLRSLRINNNRLRTLEPGTFDALSALSHLQLYH

NPFHCSCGLVWLQAWAASTRVSLPEPDSIACASPPELQGVPVHRLPALPCAPPSVRLSAE

PPPEAPGTPLRAGLAFMLHCVAEGHPTPRLQWQLQIPGGTVVLVPPVLSKEEDGGDKVED

GEGDGDEDLPTQTEAPTPTPAPAWPAPPATPRFLALANGSLLVPLLSAKEAGIYTCRAHN

ELGTNSTSLRVTVAAAGPPKHAPGTGEEPDAQVPTSERKATTKGRSNSVLPFKPEGKTKG

QGLARVSVLGEIEAELEETDEGEQMEGQIPADPMGEKHCGHGDPSRYVSNHAFNQSSDLK

PHVFELGVIALDVAEREARVQLTPLAARWGPGPDGASGARRPGRRPLRLLYLCPAGGGTA

VQWSRVEEGVNAYWFRGLRPGTNYSVCLALAGEACHVQVVFSTKKELPSLLVIVTVSVFL

LVLATVPLLGAACCHLLAKHPGKPYRLILRPQAPDPMEKRIAADFDPRASYLESEKSYPA

RGEAGGEEPEEVPEEGLDEDVEQGDPSGDLQREESLAGCSLVESQSKANQEEFEAGSEYS

DRLPLGAEAVNIAQEINGNYRQTAG

>CG12199-PB;kek5

MMGNRTERSGRRLGMILLLLGVLVVLMALPPPTAGTTDWMQSCGTCHCQWNSGKKSADCK

NKALTKIPQDMSNEMQVLDFAHNQIPELRREEFLLAGLPNVHKIFLRNCTIQEVHREAFK

GLHILIELDLSGNRIRELHPGTFAGLEKLRNVIINNNEIEVLPNHLFVNLSFLSRIEFRN

NRLRQVQLHVFAGTMALSAISLEQNRLSHLHKETFKDLQKLMHLSLQGNAWNCSCELQDF

RDFAISKRLYTPPTDCQEPPQLRGKLWSEVPSENFACRPRILGSVRSFIEANHDNISLPC

RIVGSPRPNVTWVYNKRPLQQYDPRVRVLTSVEQMPEQPSQVLTSELRIVGVRASDKGAY

TCVADNRGGRAEAEFQLLVSGDYAGAVSASDGMGMGAIGAPTIDPQTNMFLIICLIITTL

LLLLLVAVLTLFWYCRRIKTYQKDTTMMSGDGLISSKMDKTHNGSMLEGSVIMEMQKSLL

NEVNPVEKPPRRTDIESVDGGDDVLEIKKTLLDDTVYVANHSRDEEAVSVAMSDTTTTPR

SRHTYVDDAYANSLPPDLLAFPARVPPTSPSMQSSQSNIPDQVIYGIRSPPSLTSPVYTH

MTPHGIYGTKTMTAPHNGFMTLQHPKSRNLALIATTNSSRQHQHHHQLQQQQQHHHHHQQ

QQQQQQQQQHPLATTSPFLPAPVVYSPATGVVMKQGYMTIPRKPRAPSWAPSTSGAAGHG

SIQLSEFQSPTSPNPSETGTATTAELQAEPVYDNLGLRTTAGGNSTLNLTKIAGSQGGAG

QQYSMRDRPLPATPSLTSVSSATNASKIYEPIHELIQQQQQLQQQQQQQQQRLGSMDTEP

LYGVRQQGITILPGSSISGAGLGHAAYLSPGSGAAVSPSHASSSGDSPKAAKIPPRPPPK

PKKKMSVTTTRSGQGSTSQLFDDEGEDGTEV

>CG12283-PA;kek1

MHIREAVFLVLTLLPGMILGTRYNQLHLYANGGASSSGPGGYRPAPSSQNEVYSIADSQP

MTEDGYMPPSQHFPPTHSDLDPPAQQQSTCQTVCACKWKGGKQTVECIDRHLIQIPEHID

PNTQVLDMSGNKLQTLSNEQFIRANLLNLQKLYLRNCKIGEIERETFKGLTNLVELDLSH

NLLVTVPSLALGHIPSLRELTLASNHIHKIESQAFGNTPSLHKLDLSHCDIQTISAQAFG

GLQGLTLLRLNGNKLSELLPKTIETLSRLHGIELHDNPWLCDCRLRDTKLWLMKRNIPYP

VAPVCSGGPERIIDRSFADLHVDEFACRPEMLPISHYVEAAMGENASITCRARAVPAANI

NWYWNGRLLANNSAFTAYQRIHMLEQVEGGFEKRSKLVLTNAQETDSSEFYCVAENRAGM

AEANFTLHVSMRAAGMASLGSGQIVGLSAALVALIVFALGVIMCLLLRVKRQPYVDSKTP

NHMEVITSVNHQNSITNKTQPATGNGSIGGVVIANGAVANIIDGGVVQGGTLERKSSGRG

GVPHGVHDQRSANPVQKPPRLTDLPYSTQGYDNNGSVLSTASCFISPSGSTGNGGNNPDL

INDTKRFGSDEFADLKIPPISGVGVGGSGEYSRANGCDSLYPSGLWEHGAPVGTTSADDL

FMKRYTDKTPIIDSTQLYDLHERTAATDYFSKTFPRSHLQQGMMTGGGGGTSTASTVTTN

LSGGSSSGYPNDYGLPLVPGAEHQHNHQLQMHPLQQLQQQLTSTLNHQKQEGSSTGSSPH

FSSRTLPRLHEGSGGGGSSRSNGEETKPRTD

>CG1804-PA;kek6

MHRSMDRRRSRTPRTLPVCWILLCLVAWTVADDWSLSCASNCTCKWTNGKKSAICSSLQL

TTIPNTLSTELQVLVLNDNHIPYLNREEFSTLGLLNLQRIYLKKSEVQYIHKESFRNLKI

LVEIDLSDNKLEMLDKDTFMGNDRLRILYLNGNPLKRLAAYQFPILPHLRTLDMHDCLIS

YIDPMSLANLNLLEFLNLKNNLLESLSEYVFQHMANLKTLSLEENPWQCNCKLRKFRGWY

VNSRLSSVSLVCKGPPAQKDRTWDSVDDELFGCPPRVEIFNNEEVQNIDIGSNTTFSCLV

YGDPLPEVAWELNGKILDNDNVLFESESIASDKLWSNLTVFNVTSLDAGTYACTGSNSIG

SMTQNISIYLSEIVQHVLEKTPETFWYFGLIMGIFGTVFLLISISFVVCLCKRTTRQHRH

ANKAGVKSSVSFNDQEKKLLDSSVTTTTNDRGDSYGIDNQPTSIGMNKGDSAGMGFNQIE

IHAVESHRHGSMLVQQQPQQQQVAGGGGMRQQLMQVKDSTCGMMSVPTSMAGHAHSHPAQ

ISEEFPLNVGVFPPPPEFCSNIVPNPAFGGNIFIRVSVTQDMLDGADLNMYPDLLNIPKR

MQDVQESGAGAVAVPEGQFATLPRHTARRGILKKDTSLQQQQQQHQQQHQHQQQQQQQQI

QQQQHQQLQQQHQPSGLYTHDEIVTYNLEASGYDPHQSGYHSNAMELPPPPPPPAVTAVV

QCHHPSPNNCASCINNAPPPPSACQSPPVEVTPMRPLDSSAYPKYDNMGRRITASGGLGG

SNLSLHDEERYENETLFGQAESQTKGMPEQSQDLHQPQEVTQGQDKGGGPGEFVSL

>CG4192-PA;kek3

MAAGRAAATLEAPGPPSGQDIASDNSAQRRTLATKVRRKGPRPQRRLHPPLRPRLPLHLH

LLLWLLCCCSQLGQLRAECPAVCECKWKSGKESVLCLNANLTHIPQPLDAGTQLLDLSGN

EIQLIPDDSFATAQLLNLQKVYLARCHLRLIERHAFRKLINLVELDLSQNLLSAIPSLAL

YHVSELRELRLSGNPILRVPDDAFGHVPQLVKLELSDCRLSHIAVRAFAGLESSLEWLKL

DGNRLSEVRSGTITSLASLHGLELARNTWNCSCSLRPLRAWMLQQNIPSGIPPTCESPPR

LSGRAWDKLDVDDFACVPQIVATDTTAHGVEGRNITMSCYVEGVPQPAVKWLLKNRLIAN

LSAGGDGDSDSEPRTAAATQGRKTYVVNMLRNASNLTILTADMQDAGIYTCAAENKAGKV

EASVTLAVSRRPPEAPWGVRIILLGAVAALLLVGGSSFAAICLCSLQRRRKLRLWNSVPP

VRRSESYEKIEMTARTRPDLGGGASCGGGSATGAGLFHDAEEQGYLRAAHTPLNDNDAGQ

AAAIVNPSAGSAQRRNGDYLHVSTHCDDEEEDQQLHHHPQQQPASQHHPHPNQQQHQQRK

GSQGHVVSASGANNSAPLEETDLHIPRLIDIGGTDSASSSISSQVDAAARLAGYAGHTWK

TTPIATTKINSPHSKPVTSAAPSSLNTQATPYAHYGNHPADEMATSVFCSEGQESDLFDS

NYPDLLDIAKYAVAQAQQEGRGQGYAQATTTPNGGLCTLPRKLKTSGKYFRNSSDSQSPL

LADNSSKYGSSTLGDGSFLNEAMGLGRRYSAESSYANYSSTATYTGGGQRANSFLNLVQS

GAHQGKLLPSHLGQKPSLPSSPVQHQRSLSSAATPLLDFSALASRAAGAANTSVAAYDYH

AAQLERFLEEYRNLQDQLCKMKETCDTIRKKETPLRVAIGQSAAQLADPVMYSAASHSPK

PPATSNLKTKTLLPGQPPDPPPYWLHRNAMLKRLNGDGSAGTNGSGGSPASPQPRQDIFK

S

>CG4977-PA;kek2

MSGLPIWIPLLALLAITAACPPEVCVCKWKGGKQTVECGGQQLSNLPEGMDPGTQVLNFS

GNALQVLQSERFLRMDLLNLQKIYLSRNQLIRIHEKAFRGLTNLVELDLSENALQNVPSE

TFQDYSSLMRLSLSGNPIRELKTSAFRHLSFLTTLELSNCQVERIENEAFVGMDNLEWLR

LDGNRIGFIQGTHILPKSLHGISLHSNRWNCDCRLLDIHFWLVNYNTPLAEEPKCMEPAR

LKGQVIKSLQREQLACLPEVSPQSSYTEVSEGRNMSITCLVRAIPEPKVLWLFNGQVMSN

DSLMDNLHMYYYIDETIGVSGAEEKRSEIFIYNVGAEDNGTFSCVGQNIAGTTFSNYTLR

VIIKEPPVVNEVSFPRDYMNYIVASSAGGGIIFVVLLCTIVVKCKKTSEPAKQRKKCDQV

TSIAGGTDSSTGSTQDTGMGMMKCASILNDGGDSMNGNPGLLLGDTLTPTKAANGAAGGG

IILGNQMKQNLLLYATPNSAQQQLQLNVNLMGTGPGSPPLLLSNGHGLAAAYCSPPASLR

NYQEKNPDLVNDAESVKHKLKTAVSLDGAGEYETQSDCGQYEGCYQLAAAPHPHQGHQHP

HPGHPLMGRFAQAMTTLPRGMQLKPAPHQVDVHLNPVCFLGQDGSFAYDYSSAHMVQQPP

QQQQQQQQVQPANNFYRTLPHNRLHKQQQFQAAAAAGGNVGVGGNPTLRYSLEAEFIQRG

PTVSYEKYQLPNVRFTAEGYPQQQQQQQQQLQQQQQLQLQQQHQFPSPPEGYKSDLAVMP

APFQQWPSCLPGYRFAQSPTSLPAVATPPPAAVVATPPPPTSAVSTQSTATSTIPELDES

EASSPRLEEAAGSAAPPAGEEESSDTAKLKQLNGPLADSPDEGYVGDGQETSDI

>CG9431-PA;kek4

MAIKLSFDPCSISLKHLSLFLFKIYCLALIFRSASADWLLDCGNCHCKWNSGKKTADCRN

LSLSGVPEYLSPEVQVLDLSHNHIFYLEENAFLTTHLQNLQKLLIRNGTLKYLNQRSFTQ

LQILIELDLSNNLLVDLLPNVFDCLSKVRAIFLNGNLLQALRHGVFRNLKYLHKIELKRN

RLVSIDAKAFVGVPLLSQIYLDNNELTKLRVESFQDLTKLTALSLVENPWNCTCDLQMFR

DFVIGMNLYTPPTSCHYPLQLRGRLWIEDQPEAFACKPKIVYPTLSTSINTSKENVTLIC

RVHGSPNTVIAWDYTNQVYESRSKPVKSLQKQRIYIELLREDESKIRKFGHDVFVSRLTI

VNARKSDEGVYTCLAENPGGKDSVHISVVVQKDMERISLIDSNFFAIVCLIAMGFLSMSI

LFSLVTCLIFKRFKQFHPGQHTYLQPTSLPVQSPGSEEATAISALSSGVIRESKIVLDPL

SAINEPSNKNYTLFKTSNSNGSEYMHTRNYKDVRLNSNTYTENLDNQAESISSRNRELYS

NIAGDREKEELKQKDELDKDSRQSSLQSTGCSRKKGQIDELQPDLLPSTQPTALKNINET

FGPSAKKAEVNPRSKYNTNVQKYLKEKYGSVRIKNISTKEPITGVDISI

>ENSMUSP00000061906;Lrrc24

MALRAPTLLLLLLGLLLLPLLPGLPPRATGCPAACRCYSATVECGALRLRVVPPGIPPGT

QTLFLQDNSIAHLEQGSLAPLAALRHLYLHNNTLRALESGAFRAQPRLLELALTGNRLRG

LRGGAFVGLVQLRVLYLAGNQLAKLLDFTFLHLPRLQELHLQENSIELLEDQALAGLSSL

ALLDLSRNQLGTISKEALQPLSSLQVLRLTENPWRCDCALHWLGSWIKEGGRRLLSSRDK

KITCAEPPRLALQSLLEVSGGSLICIPPSVNVEPPEFTANLGEDLQVACQASGYPQPLVV

WRKVPQPRDGKPQAQAQLEGGAPGLGGHGTRDTGSGMLFLTNITLAHAGKYECEAANAGG

KARVPFHLLVNASRQQSQQLPDPQAPATRPVGHEPQHEAGSMAFRALGLATQTAITAAIA

LLALTALLLAAMICRRRRRRKKVPAPSGEGTLFVNDYSDGPCTFAQLEELRDDHGHEMFV

IDRSKPLFPEVLPEEAPEHNPPDGLKSGLRLPTRVAYEIHC

>ENSMUSP00000053869;Elfn1

MAGHGWGTAWVLVAAATLLHAGGLAQGDCWLIEGDKGFVWLAICSQNQPPYEAIPQQINN

TIVDLRLNENRIRSVQYASLSRFGNLTYLNLTKNEIGYIEDGAFSGQFNLQVLQLGYNRL

RNLTEGMLRGLSKLEYLYLQANLIEVVMASAFWECPNIVNIDLSMNRIQQLGSGTFAGLT

KLSVCEIYSNPFYCSCELLGFLRWLAAFTNATQTHDRVQCESPPVYAGYFLLGQGRHGHQ

RSILSKLQSVCTEGSYTAEVLGPPRPVPGRSQPGHSPPPPPPEPSDMPCADDECFSGDGT

TPLVILTTLVPQTEARPSMKVKQLTQNSATIMVQLPSPFNRMYTLEQYNNSKSFTVSKLT

QPQEEIRLTNLYTLTNYTYCVVSTSSGTHHNHTCLTICLPKPPSPPGPVPSPSTATHYIM

TILGCLFGMVLVLGAVYYCLRKRRRQEEKHKKAVAAAAGSLKKTIIELKYGPEIEAPGLA

PLTQGPLLGPEAVTRIPYLPAATSDVEQYKLVESSETPKATKGNYIEVRTGEPQERRGCE

LSRPGEPQSSVAEISTIAKEVDRVNQIINNCIDALKSESTSFQGAKSGAVSAAEPQLVLL

SEPLASKHSFLSPVYKDAFGHGGLQRHHSVEAAPGPPRASTSSSGSARSPRTFRAEATGT

HKAPATETKYIEKSSPVPETILTVTPAATVLRAEADKSRQYGEHRHSYPGSHPAEPPAPP

PPPPTHEGLGGRKASILEPLTRPRPRDLVYSQLSPQYHNLSYSSSPEYTCRASPSIWERL

RLSRRRHKDDAEFMAAGHALRKKVQFAKDEDLHDILDYWKGVSAQHKS

>ENSMUSP00000060210;Elfn2

MLRLGLCAAALLCVCQPGAVRADCWLIEGDKGYVWLAICSQNQPPYETIPQHINSTVHDL

RLNENKLKAVLYSSLNRFGNLTDLNLTKNEISYIEDGAFLGQTSLQVLQLGYNRLSNLTE

GMLRGMSRLQFLFVQHNLIEVVTPTAFSECPSLISIDLSSNRLSRLDGATFASLASLMVC

ELAGNPFNCECDLFGFLAWLVVFNNVTKNYDRLQCESPREFAGYPLLVPRPYHSLNAITV

LQAKCRNGSMPARPVSHPTPYSTDAQREPDENSGFNPDEILSVEPPASSTTDASAGPAIK

LHQVTFTSATLVVIIPHPYSKMYVLVQYNNSYFSDVMTLKNKKEIVTLDKLRAHTEYTFC

VTSLRNSRRFNHTCLTFTTRDLVPGDLAPSTSTTTHYIMTILGCLFGMVIVLGAVYYCLR

KRRMQEEKQKSVNVKKTILEMRYGADVDAGSIVHAAQKLGEPPVLPVARMSSIPSMVGEK

LPASKGLEAGLDTPKVATKGNYIEVRTGAAGDSLARPEEELPEIENGQGSAAEISTIAKE

VDKVNQIINNCIDALKLDSASFLGGGGGGGGGGDSDLAFECQSLPAAPAASSAATPGALE

RPSFLSPPYKESSHHPLQRQLSADAAVSRKTCSVSSSGSIKSAKVFSLDVPDHPTPTGLA

KSDSKYIEKGSPLNSPLDRLPLVPTGSSGSSGGGGGIHHLEVKPAYHCSEHRHSFPALYY

EEGADSLSQRVSFLKPLTRSKRDSTYSQLSPRHYYSGYSSSPEYSSESTHKIWERFRPYK

KHHREEVYMAAGHALRKKVQFAKDEDLHDILDYWKGVSAQQKL

>ENSMUSP00000057005;B430119L13Rik

MRWTLMLQLLQLLLQLLMAQSQSLERISQDRIPLFRLTQQGDWDSLDRHPTDSLCVGLPA

AGVTTLNLANRSLESLPSCLPRTLRSLDGSHNLLRALSEPVLGRLPELRVLTLHHNRISV

LHWGRDTLAELRELDLSHNLLTELPPCAGPSGSSLRSLALAGNPLRALLPRTFACFPALR

LLNLSCSELGHIAQEAFAGVDGGPLAALELLDLSGTSLERVESGWIRNLPKLKSLFLRKM

PRLKTLEGDIFKMTPNLRQLDCGDSPALTSVHTEIFQDTPNLQVLQFQNCNLSSFGPWNS

SQVLSVSLFGNPLICSCELAWLLVDVNKTVLHRAADTMCEPALGSTGPFSGPLSLSHLSN

VCRSDQSTTLLPSNPGRFDHSVFAPRIQGPSIEQSTALSAQPGGSQQNITKVPSLTMTSP

TQGSWMYKDASEETAQSTNSELVYSPSRALPGAASSGAEQTATHILEPNISSASTPLVSK

YLEPLPTSPNPRSLPQTKQRTQATPRALHTDPPQDEIPVLLLDDDSEEEETRDQVAAPPQ

DVSCEYHPCKHLQTPCAELQRRFRCRCPGLSGEDTTPDPPTLQGVSEVTDTSVLVHWCAP

NSVVLWYQIHYVAEGRSGNQSVVDIYATARQHPLYKLTPGTTYHVCVLAANRAGLSQSQT

SGWRRSCATFTTKPSSVVIFWGLCTASGLLLVSTLVLSVCLWRQRWKPHRQFYDTHLVAF

KNPARAEEVTQWE

>ENSP00000246074;CT075_HUMAN

MRQTLPLLLLTVLRPSWADPPQEKVPLFRVTQQGPWGSSGSNATDSPCEGLPAADATALT

LANRNLERLPGCLPRTLRSLDASHNLLRALSTSELGHLEQLQVLTLRHNRIAALRWGPGG

PAGLHTLDLSYNQLAALPPCTGPALSSLRALALAGNPLRALQPRAFACFPALQLLNLSCT

ALGRGAQGGIAEAAFAGEDGAPLVTLEVLDLSGTFLERVESGWIRDLPKLTSLYLRKMPR

LTTLEGDIFKMTPNLQQLDCQDSPALASVATHIFQDTPHLQVLLFQNCNLSSFPPWTLDS

SQVLSINLFGNPLTCSCDLSWLLTDAKRTVLSRAADTMCAPAAGSSGPFSASLSLSQLPG

VCQSDQSTTLGASHPPCFNRSTYAQGTTVAPSAAPATRPAGDQQSVSKAPNVGSRTIAAW

PHSDAREGTAPSTTNSVAGHSNSSVFPRAASTTRTQHRGEHAPELVLEPDISAASTPLAS

KLLGPFPTSWDRSISSPQPGQRTHATPQAPNPSLSEGEIPVLLLDDYSEEEEGRKEEVGT

PHQDVPCDYHPCKHLQTPCAELQRRWRCRCPGLSGEDTIPDPPRLQGVTETTDTSALVHW

CAPNSVVHGYQIRYSAEGWAGNQSVVGVIYATARQHPLYGLSPGTTYRVCVLAANRAGLS

QPRSSGWRSPCAAFTTKPSFALLLSGLCAASGLLLASTVVLSACLCRRGQTLGLQRCDTH

LVAYKNPAFDDYPLGLQTVS

>CG16974-PA; CG16974-PA

MEAISKISLILCALFVGLKAAAAISGDSTHCLATYSSAEAYLAQIPQQHRPQIRPRIRTW

QEHEFSLLGYKFHLPFVGHAVDSDLDDSDSDEGLWLDAADAGSESVEVEEHELPSVGHVD

PTGNVFKLNCEHVDLRRVNQELLSQRSSHINYNQLMLAHVPADRSNPLKLPQLESLREFS

WQSSELKDETLMELFTRQPRSFEYMERLNLAENRLECLHWAIPLAVRRVKVLEMSGNRLS

NCSLLNLQYMKQLQELHLDRSELTYLPQRFLGELSELRMLNLSQNLLTELPRDIFVGALK

LERLYLSGNRLSVLPFMLFQTAADLQVLDLSDNRLLSFPDNFFARNGQLRQLHLQRNQLK

SIGKHSLYSLRELRQLDLSQNSLSVIDRKAFESLDHLLALNVSGNNLTLLSSIIFQSLHA

LRQLDLSRNQFKQLPSGLFQRQRSLVLLRIDETPIEQFSNWISRYDESLVDPQVLHRLRY

LSVQQNRKLTYLPATLFANTPNIRELLLAENGLLQLPTQISGLSRLQRLSVRGNSLGSLP

ENIKELRQLHYLNILGNEYQCDCSMYWLTAWLANTSTSLRHQMPQAQNHSNGSTNQTPLD

SYESIDHQIDALKCQYGYRGDMLRVLSKLNCSVPTVVQFSEPKMHKLLSTAKLECQISGS

PVPDIIWVTPRNKILRHHADPDKRPIIIDSKEDAHQPPSAQELAALMDESYIQSLNWTRQ

NSLVGRRVVLVENGSLLVHNISRIDSGLYTCYAFNVMGKASAGLRLYIDPIVFYRVKIGS

ILFGTALATAFLLLTLIVQGLRSCLSRWGICNRFYCCVNRKKKSPRKHQIYAMLDSIETY

KSQQLERLRENYTQQVHRIRENCAQQVEWIQSSYTSQAKHIREFRDIGSNHLTTLKDQYY

DQVKKVRDYSTGQLSWVRENYVFQRNKIRKFSAHQVLRLREGYKYQQQTLNKVLENLPSF

YFENCRGRCEEDIAEDIDCYFKGQMDFGDSKELHIQKIKARLSANYSASKASLYYTPPDD

DLLHSSQLNLQNSPIHINYIDENLDQQKQLEHDFKMEPQLLLYNASMLYMNPEGASSSGQ

AAALAAAGALSQFISVEDNNQEQEMQPLRKISGKPLGMPELKDLNDVKTSKSCPAIYKVS

KQRDGSTLHELQKEGEAPYQMLRINPVETTSLTSTVAPVMQARTEKLNIILDECGTASLC

KAEQEGDAGNSETPPSESSCESNSLAASCGDVCQVSSKLANDASLPSTPPKPDHAST

>F20D1.7;F20D1.7

MVLQIFVLSIFFISLFNKAIACPLGCSCNESSISCTFLTKPELYVFLEVLKDSPFNETTD

LSLASIDNFWLRMLPDMPKLRVLKVQSSPSLDDSDWFLRRNQFPELQTLHFKNCSLKTFP

KALMALTSLLELDLSDNMLENLGADSIHIRSIQRVILRNNQIKSIGVHVFRYMPTLKMLD

LSGNNMTRLVTSDFTSAVSLRELILRENKIELIETDTTEPMQQLETLDLSGNLLSEVRLE

AQQNFRHLFSLNLSCNPLQIIREGFLQLPDLQVLQLDNCNISVVEAGAFVSLPRLHSMDI

KDNPNLAYFSPYAFSNNTAFYRMNIQNSGFKRIPLTILEKISQLYIKGTQLDCSCTSRDM

QDYGAITIVDWNDATCKTKIGAVQKLSHLEKTGEPCRDNLLTPFGIRQTATVGHSYRIYC

ANDGTNSKLLWISPNKTTIEASHPKLRKSSDKRTDYFTTTLLDPSFSRNHEERIHISNEY

YGFDVVLENDAGHYECVSKSDMKTITRKIELEVVKPNIYLNASHVATTSVHLNWNRNLKI

EAVDRVALRITASSEKMFKRQVQLSLYNMFRSYNLVNLSADKEYLICLEWYLTDNDAVIY

SSCISQKTKPFKTVMQSLNAKLAIALIIILIIVVVFCCDTCIHQKVAYFSRIKKNAKMQQ

SVSGQSMLTQSSSADATTYENFQLSVTSASEHPLL

>Y37E3.13;Y37E3.13

MREAALLLLFLPIISCSNAPKTCKSFWISNRPSQDCASLTLQEPPQLLPNVLSLSLSNNS

IFRITTFPSEYRRLQSLRLDQCQLEKLDFDALSVFEQLRELDVSRNSLSKLIIPRNLASL

RVLNLAFNAFTYVPDMSHLESLRLVDLSHNRLISVRPRMLPFNLEVVRLAANRFQHLSPW

PFLHKLQELDVTFNDLECDCSLWHFVTWAEKLALFDRRFQTVCGPTVVTSSPESAVVSLD

DAHVMCCTALATPSPQLYWQFNGKNISSGLSQKHLSESGKLEFCLEIPKVRLKDMGKYKC

VASLAGLNSSKEFHVERDKIPIVLNSAEGIMIYCQFTICTFIGVCCVISCCVLRTGGGGR

GKTRPNREYLHTKVLEIPHDSCEYCEVDDDDVDDTCEDWGDAETAAVRQYLQWRQMQMQQ

EQMQKCPMLPKIRHESLCRLSNESGSSNRSIPRAPLARFDEPSCVSFELTHL

>XP_991236; Lrrc37a

MTPLGFCFCAPSLLLTGQVLLVVIQADPIAEWVQNPVRLTSEPW

VRTKPWSWHPSDQAPKSPKAHPSEAGEGALDYLGSSAPSQMFSPPRELKDSFLSFQAM

DTSVASHPESDQFTVPHQHLAKQMTPPRKPLESVPKLNGDQNQSPQFKSISSLDQAAD

DQLFEIPDPPLDSENSSITKFIASPQSLQKELVQHRPLAKEVVGTTKQFAKSQLQKKT

VVDEYRDPNMNEAYSKSLPLQSQANREGPSEKIEHPEYPLEAQTQESENLDMVHESPE

YLPVLPEEDEPSVPKENPVQHHFASGKAAALKHPDRNAPSPKRNEARHSKLPNVTVKP

EDLEVTVMSEADKETQQTLSQQQAPGHLPESPKEVEPSSTPQDTLEQFSGGPEEIETL

LTHQEAAAPNPELPEELGPSLVQQEVLSESLELPKDLEASGSQLEVPARRTKPPEEVN

PPTEQEARIPTPEPSMPNIIEIPAATVSHHNQDQVHHYTLPTITVQPADVEVTITSKP

FKEAEISPVQEETQTPGPPRKVGHYSLEQQPAVSSESSGEVGSLERYLGFLTQPPEEE

EEEEEEEDEESSLTQEDIPSQHPSPILEGEPSPIELQQPIRPSESPEEVGLGPKTQSE

VLVKPAELPEEVKSPVEQEAPLQAPESHFETIVDTPPIHEVQPAPNKAHHHHWPNVTV

RPVDLELAITSEPTRETESSLAQQESSVHPSEYTEERDSFRYKQEQLAQPSKLEILIH

TSEHHHLTSSPSAHHRTHHSSSPTTMVRPPDVQLTIAQNPTAEVEPPPALHEVVGKPI

AISDKGVNTSTHHTHPAVTPEPSEEVELLSNQEEAPVQSAEPVEYEKSSLSQQKSTDK

NPELLEEGGLSSAQQETLVNPPVFPNEMIVQPSAHYEATGPPLIQTQLHPPASYNVTA

KPPELTNEVETSSQQGVEPSNDQQEEVTSQYPMPPETHQDSPVYQWVLTQPAKLPEEV

SSGQQGNLSPPLKHPPGVQFIPFQQELPDQHLEPPKEGFFLSPVADSVLFSPEDLEAM

FRYETSVLHKPTVIHLNQAVSSMEVESLKVPTEQVEYSQVHFEYPSYTPAFPETQFSQ

EKSITQEADLHEDIYPFPTQHRGLYLPPDSFMGAEPSVTQHLSLSQLEDLAENVGPSL

VLQLTPAQASEPPKEIVFSPTQQVVPNQLPESLNNIVTQLLTQQIIGPTPGQIQEEYP

TEHTVSFQALDLEFTITSQYTPEANHTTEPKRTPPPTYPQVTFSYPSEVTVQPLDLGL

TISPQPTPEELPQAIPEITTQITEPPREVVAPAPLYQKMTVPTPGQDQVEYPIPPAIS

FQPLDLELTKNSEPTRETQQPTATMKTIVPSPEHLQDYISYVAEVTVQPLNLGLTISP

QSAPEELPQPMPEITTQITEPPREVVAPAPLYQKMTIPTPGQDQDAHSTTLQPVGLEL

TETSDSTREAEHYTTLVMTTVSSPKYSLPEQVLLQHLNPAEVTVQALDLGLTITPQPD

TEKELSQTTQESTTQLIDPPKEAVAQAPVYQEVTLATPSQDAFENPTLPIITFRLLDP

DPEPAREADNLATLKETTIPPPKYPPVTLPEQVHTQHPHPSEVTFQPLDMELSMSPQP

TPEVELSQAEQETKTQPSYSAKEVVTPKPGYPEVTVPTPDQDKTEDQTLTAALFQPLD

LELTRSSEPTTPHKSTVVSLPQYYQTTLSGEVYTQHPNLTEVTDQPLNLELTVIPSPN

LNVETSPHIQEMLPEPERPHEEVTAKAPMFYEITPIQDQTPHLVSPKVTDQHIKVEHD

IPTISSVENGLLAATYSSTALPLTHHEMVLLSEDQVQVLHGNPTQVTIHPSHPEFSLA

SPPATMVKRSAPVQSTESSKETAAQSPVHLKETVLMIAHGQSQHLNSETTQPEVPATT

HPETTQSTASPTTHSETTHSTASPTTHPDTTQSTASPTTHPETTQSTASPTTHSETTQ

STTSPTTHPETTQSTASPTTHPDTTQSTASPTIHPDTTQYTTSPTTHPETTQYTDLTT

MPPPSTHYEVVLSQSSQVQTQQPKPSEVTMWPSPTSQQSSTTPQSVILASPFATMLET

TAQQIPSGIALEPMDLEPTITPYAGNFNTEKDLIFQMKPNVSTSTNICDLCLCENHTL

LCSHLSPKRRLHQVPVLRPGTHKGTLTILNFHGNVISYIDKNVWKAYRWAEKLILSEN

RLTELHKESFEGLLTLQVLDLSCNKIHYIERRTFESLPFLKYINLGCNLLTELNFGTF

QAWHGMQFLQQLILNHNPLTVVEDPFLFKLPTLKYLDLGATQVQLTMVENILMMMLEL

EHRILPSRMACCLCKFKADIEIICKTIKLRCHTGCLTNTTHCLKASIGDPEGAFMKAL

LARKENSRAELTIEPEKSYADQTNLGSLGLMNEQLDFNDESDVISALNYILPYFSEGN

LEDVVSTLLPFIKLLFSNIQNGDNSLGPFQNDTKSLTLKSVPKASKLAYKNKVNKLYF

LENLLDEEINEVKEKEKTAMLVHHSGRLDPKFKRQIFEKRWEPARTGKDSLAEIEKAE

RQLHSMSRVPKETGSIQKRHFKDVSGKSLWSKQSVQTPVESISKDRQLGSPPSMELQQ

LGLEQKPRELVGYSFPSEPLLPKEHRGELSSSPDLPLLDKAPTTNSLPDFIDRRKDLS

YTIYVLESANANVKRAKGSNPSLQPEARHRNLRKKKSHFQLIAKRPAASSAVRSLISS

SARGVFSSLGDLRYPERPFSELYVAPEPSTKKPLEENRAATDNVEENNLKQIVTTPEE

TTSENKPPENPTADSNVSTTSNLISTVQQTSKPQSVFTVGADTHADLTDVAYPSLMSP

GEQFESHLNQQLLPLIPNKDVRRLISHVIRTLKMDCSDTRVQMSCAKLISRTGLLMKL

LSEQQDFKLSRADWDTDQWKTENYINESTETQGEQRSLEPSQLTKAVPGYGYNNKVIL

AISVTVIVTVLIIIFCLIEVRTSVHAWVQNVPCLCSRFRTWKLETSVTYPLCLQVMSP

CVHRYKKNYYKISETQDGFFWLRCPLWLRDMYRPLHDTRKKNMAQDLQDKESSGEEEI

FNKDVPREFKRRSSVKSPAEESVVEAPR

>ENSP00000335617; LRRC37A

MEMYGKHTVGPRNCEILRENNLTELHKDSFEGLLSLQYLDLSCNKIQSIERHTFEPLPFL

KFINLSCNVITELSFGTFQAWHGMQFLHKLILNHNPLTTVEDPYLFKLPALKYLDMGTTL

VPLTTLKNILMMTVELEKLILPSHMACCLCQFKNSIEAVCKTVKLHCNSACLTNTTHCPE

EASVGNPEGAFMKVLQARKNYTSTELIVEPEEPSDSSGINLSGFGSEQLDTNDESDFIST

LSYILPYFSAVNLDVKSLLLPFIKLPTTGNSLAKIQTVGQNRQRVKRVLMGPRSIQKRHF

KEVGRQSIRREQGAQASVENAAEEKRLGSPAPREVEQPHTQQGPEKLAGNAVYTKPSFTQ

EHKAAVSVLKPFSKGAPSTSSPAKALPQVRDRWKDLTHAISILESAKARVTNTKTSKPIV

HARKKYRFHKTRSHVTHRTPKVKKSPKVRKKSYLSRLMLANRLPFSAAKSLINSPSQGAF

SSLGDLSPQENPFLEVSAPSEHFIENNNTKHTTARNAFEENDFMENTNMPEGTISENTNY

NHPHEADSAGTAFNLGPTVKQTETKWEYNNVGTDLSPEPKSFNYPLLSSPGDQFEIQLTQ

QLQSLIPNNNVRRLIAHVIRTLKMDCSGAHVQVTCAKLISRTGHLMKLLSGQQEVKASKI

EWDTDQWKIENYINESTEAQSEQKEKSLELKKEVPGYGYTDKLILALIVTGILTILIILF

CLIVICCHRRSLQEDEEGFSRGIFRFLPWRGCSSRRESQDGLSSFGQPLWFKDMYKPLSA

TRINNHAWKLHKKSSNEDKILNRDPGDSEAPTEEEESEALP

>ENSP00000366062; LRRC37A2

MSSAQCPALVCVMSRLRFWGPWPLLMWQLLWLLVKEAQPLEWVKDPLQLTSNPLGPPESW

SSHSSHFPRESPHAPTLPADPWDFDHLGPSASSEMPAPPQESTENLVPFLDTWDSAGEQP

LEPEQFLASQQDLKDKLSPQERLPVSPKKLKKDPAQRWSLAEIIGITRQLSTPQSQKQTL

QNEYSSTDTPYPGSLPPELRVKSDEPPGPSEQVGPSQFHLEPETQNPETLEDIQSSSLQQ

EAPAQLPQLLEEEPSSMQQEAPALPPESSMESLTLPNHEVSVQPPGEDQAYYHLPNITVK

PADVEVTITSEPTNETESSQAQQETPIQFPEEVEPSATQQEAPIEPPVPPMEHELSISEQ

QQPVQPSESPREVESSPTQQETPGQPPEHHEVTVSPPGHHQTHHLASPSVSVKPPDVQLT

IAAEPSAEVGTSLVHQEATTRLSGSGNDVEPPAIQHGGPPLLPESSEEAGPLAVQQETSF

QSPEPINNENPSPTQQEAAAEHPQTAEEGESSLTHQEAPAQTPEFPNVVVAQPPEHSHLT

QATVQPLDLGFTITPESKTEVELSPTMKETPTQPPKKVVPQLRVYQGVTNPTPGQDQAQH

PVSPSVTVQLLDLGLTITPEPTTEVGHSTPPKRTIVSPKHPEVTLPHPDQVQTQHSHLTR

ATVQPLDLGFTITPKSMTEVEPSTALMTTAPPPGHPEVTLPPSDKGQAQHSHLTQATVQP

LDLELTITTKPTTEVKPSPTTEETSTQPPDLGLAIIPEPTTETGHSTALEKTTAPRPDRV

QTLHRSLTEVTGPPTELEPAQDSLVQSESYTQNKALTAPEEHKASTSTNICELCTCGDEM

LSCIDLNPEQRLRQVPVPEPNTHNGTFTILNFQGNYISYIDGNVWKAYSWTEKLILRENN

LTELHKDSFEGLLSLQYLDLSCNKIQSIERHTFEPLPFLKFINLSCNVITELSFGTFQAW

HGMQFLHKLILNHNPLTTVEDPYLFKLPALKYLDMGTTLVPLTTLKNILMMTVELEKLIL

PSHMACCLCQFKNSIEAVCKTVKLHCNSACLTNTTHCPEEASVGNPEGAFMKVLQARKNY

TSTELIVEPEEPSDSSGINLSGFGSEQLDTNDESDFISTLSYILPYFSAVNLDVKSLLLP

FIKLPTTGNSLAKIQTVGQNRQRVKRVLMGPRSIQKRHFKEVGRQSIRREQGAQASVENA

AEEKRLTSPAPREVEQPHTQQGPEKLAGNAVYTKPSFTQEHKAAVSVLKPFSKGAPSTSS

PAKALPQVRDRSKDLTHAISILESAKARVTNTKTSKPIVHARKKYRFHKTRSHVTHRTTK

VKKSPKVRKKSYLSRLMLANRLPFSAAKSLINSPSQGAFSSLGDLSPQENPFLEVSALSE

HFIEKNNTKHTTARNAFEENDFMENTNMPEGTISENTNYNHPPEADSAGTAFNLGPTVKQ

TETKWEYNNVGTDLSPEPKSFNYPLLSSPGDQFEIQLTQQLQSLIPNNNVRRLIAHVIRT

LKMDCSGAHVQVTCAKLISRTGHLMKLLSGQQEVKASKIEWDTDQWKIENYINESTEAQS

EQKEKSLELKKEVPGYGYTDKLILALIVTGILTILIILFCLIVICCHRRSLQEDEEGFSR

GIFRFLPWRGCSSRRESQDGLSSFGQPLWFKDLYKPLSATRINNHAWKLHKKSSNEDKIL

NRDPGDSEAPTEEEESEALP

>ENSP00000325713; LRRC37A3

MTSAQCPALACVMSPLRFWGPWPLLMWQLLWLLVKEAQPLEWVKDPLQLTSNPLGPPEPW

SSHSSHFPRESPHAPTLPADPWDFDHLGPSASSEMPAPPQESTENLVPFLDTWDSAGELP

LEPEQFLASQQDLKDKLSPQERLPVSPKKLKKDPAQRWSLAEIIGIIHQLSTPQSQKQTL

QNEYSSTDTPYPGSLPPELRVKSDEPPGPSEQVGPSQFHLEPETQNPETLEDIQSSSLQQ

EAPAQLPQLLEEEPSSMQQEAPALPPESSMESLTLPNHEVSVQPPGEDQAYYHLPNITVK

PADVEVTITSEPTNETESSQAQQETPIQFPEEVEPSATQQEAPIEPPVPPMEHELSISEQ

QQPVQPSESSREVESSPTQQETPGQPPEHHEVTVSPPGHHQTHHLASPSVSVKPPDVQLT

IAAEPSAEVGTSLVHQEATTRLSGSGNDVEPPAIQHGGPPLLPESSEEAGPLAVQQETSF

QSPEPINNENPSPTQQEAAAEHPQTAEEGESSLTHQEAPAQTPEFPNVVVAQPPEHSHLT

QATVQPLDLGFTITPESMTEVELSPTMKETPTQPPKKVVPQLRVYQGVTNPTPGQDQAQH

PVSPSVTVQLLDLGLTITPEPTTEVGHSTPPKRTIVSPKHPEVTLPHPDQVQTQHSHLTR

ATVQPLDLGFTITPKSMTEVEPSTALMTTAPPPGHPEVTLPPSDKGQAQHSHLTQATVQP

LDLELTITTKPTTEVKPSPTTEETSTQLPDLGLAIIPEPTTETGHSTALEKTTAPRPDRV

QTLHRSLTEVTGPPTELEPAQDSLVQSESYTQNKALTAPEEHKASTSTNICELCTCGDEM

LSCIDLNPEQRLRQVPVPEPNTHNGTFTILNFQGNYISYIDGNVWKAYSWTEKLILRENN

LTELHKDSFEGLLSLQYLDLSCNKIQSIERHTFEPLPFLKFINLSCNVITELSFGTFQAW

HGMQFLHKLILNHNPLTTVEDPYLFKLPALKYLDMGTTLVPLTTLKNILMMTVELEKLIV

PSHMACCLCQFKNSIEAVCKTVKLHCNSACLTNTTHCPEEASVGNPEGAFMKVLQARKNY

TSTELIIEPEEPSDSSGINLSGFGSEQLDTNDESDVTSTLSYILPYFSAVNLDVKSLLLP

FIKLPTTGNSLAKIQTVGKNRQRLNRVLMGPRSIQKRHFKEVGRQSIRREQGAQASVENT

AEEKRLGSPAPRELKQPHTQQGPEKLAGNAVYTKPSFTQEHKAAVSVLKPFSKGAPSTSS

PAKALPQVRDRWKDLTHAISILESAKARVTNMKTSKPIVHSRKKYRFHKTRSRMTHRTPK

VKKSPKVRKKSYLSRLMLSNRLPFSAAKSLINSPSQGAFSSLRDLSPQENPFLEVSAPSE

HFIENNNTKDTTARNAFEENVFMENTNMPEGTISENTNYNHPPEADSAGTAFNLGPTVKQ

TETKWEYNNVGTDLSPEPKSFNYPLLSSPGDQFEIQLTQQLQSVIPNNNVRRLIAHVIRT

LKMDCSGAHVQVTCAKLVSRTGHLMKLLSGQQEVKASKIEWDTDQWKTENYINESTEAQS

EQKEKSLEFTKELPGYGYTKKLILALIVTGILTILIILLCLIEICCHRRSLQEDEEGFSR

DSEAPTEEESEALP

>ENSP00000332536; LRRC37B NM_052888

MSWLRFPMAPPYVATIVFTSQGQPLVWVKDPLQLTSNPLGPPEPWSSRSSHLPWESPHAP

APPAAPGDFDYLGPSASSQMSALPQEPTENLAPFLKELDSAGELPLGPEPFLAAHQDLND

KRTPEERLPEVVPLLNRDQNQALVQLPRLKWVQTTDLDRAAGHQADEILVPLDSKVSRPT

KFVVSPKNLKKDLAERWSLPEIVGIPHQLSKPQRQKQTLPDDYLSMDTLYPGSLPPELRV

NADEPPGPPEQVGLSQFHLEPKSQNPETLEDIQSSSLQEEAPAQLLQLPQEVEPSTQQEA

PALPPESSMESLAQTPLNHEVTVQPPGEDQAHYNLPKFTVKPADVEVTMTSEPKNETEST

QAQQEAPIQPPEEAEPSSTALRTTDPPPEHPEVTLPPSDKGQAQHSHLTEATVQPLDLEL

SITTEPTTEVKPSPTTEETSAQPPDPGLAITPEPTTEIGHSTALEKTRAPHPDQVQTLHR

SLTEVTGPPTKLESSQDSLVQSETAPEEQKASTSTNICELCTCGDETLSCVGLSPKQRLR

QVPVPEPDTYNGIFTTLNFQGNYISYLDGNVWKAYSWTEKLILSENYLTELPKDSFEGLL

YLQYLDLSCNKIRYIERQTFESLPFLQYINLGCNLITKLSLGTFQAWHGMQFLHNLILNR

NPLTTVEDPYLFELPALKYLDMGTTHITLTTLKNILTMTVELEKLILPSHMACCLCQFKN

SIEAVCKTVKLHCNTACLTNSIHCPEEASVGNPEGAFMKMLQARKQHMSTQLTIESEAPS

DSSGINLSGFGGDQLEIQLTEQLRSLIPNEDVRKFMSHVIRTLKMECSETHVQGSCAKLM

LRTGLLMKLLSEQQEAKALNVEWDTDQQKTNYINENMEQNEQKEQKSSELMKEVPGDDYK

NKLIFAISVTVILIILIIIFCLIEVNSHKRASEKYKDNPSISGA

>ENSMUSP00000020087; Dcn

MKATLIFFLLAQVSWAGPFEQRGLFDFMLEDEASGIIPYDPDNPLISMCPYRCQCHLRVV

QCSDLGLDKVPWDFPPDTTLLDLQNNKITEIKEGAFKNLKDLHTLILVNNKISKISPEAF

KPLVKLERLYLSKNQLKELPEKMPRTLQELRVHENEITKLRKSDFNGLNNVLVIELGGNP

LKNSGIENGAFQGLKSLSYIRISDTNITAIPQGLPTSLTEVHLDGNKITKVDAPSLKGLI

NLSKLGLSFNSITVMENGSLANVPHLRELHLDNNKLLRVPAGLAQHKYIQVVYLHNNNIS

AVGQNDFCRAGHPSRKASYSAVSLYGNPVRYWEIFPNTFRCVYVRSAIQLGNYK

>ENSMUSP00000021820; Aspn

MKEYVMLLLLAVCSAKPFFSPSHTALKNMMLKDMEDTDDDDNDDDDNSLFPTKEPVNPFF

PFDLFPTCPFGCQCYSRVVHCSDLGLTSVPNNIPFDTRMVDLQNNKIKEIKENDFKGLTS

LYALILNNNKLTKIHPKTFLTTKKLRRLYLSHNQLSEIPLNLPKSLAELRIHDNKVKKIQ

KDTFKGMNALHVLEMSANPLENNGIEPGAFEGVTVFHIRIAEAKLTSIPKGLPPTLLELH

LDFNKISTVELEDLKRYRELQRLGLGNNRITDIENGTFANIPRVREIHLEHNKLKKIPSG

LQELKYLQIIFLHYNSIAKVGVNDFCPTVPKMKKSLYSAISLFNNPMKYWEIQPATFRCV

LGRMSVQLGNVGK

>ENSMUSP00000033741; Bgn

MCPLWLLTLLLALSQALPFEQKGFWDFTLDDGLLMMNDEEASGSDTTSGVPDLDSVTPTF

SAMCPFGCHCHLRVVQCSDLGLKTVPKEISPDTTLLDLQNNDISELRKDDFKGLQHLYAL

VLVNNKISKIHEKAFSPLRKLQKLYISKNHLVEIPPNLPSSLVELRIHDNRIRKVPKGVF

SGLRNMNCIEMGGNPLENSGFEPGAFDGLKLNYLRISEAKLTGIPKDLPETLNELHLDHN

KIQAIELEDLLRYSKLYRLGLGHNQIRMIENGSLSFLPTLRELHLDNNKLSRVPAGLPDL

KLLQVVYLHSNNITKVGINDFCPMGFGVKRAYYNGISLFNNPVPYWEVQPATFRCVTDRL

AIQFGNYKK

>ENSP00000052754; DCN

MKATIILLLLAQVSWAGPFQQRGLFDFMLEDEASGIGPEVPDDRDFEPSLGPVCPFRCQC

HLRVVQCSDLGLDKVPKDLPPDTTLLDLQNNKITEIKDGDFKNLKNLHALILVNNKISKV

SPGAFTPLVKLERLYLSKNQLKELPEKMPKTLQELRAHENEITKVRKVTFNGLNQMIVIE

LGTNPLKSSGIENGAFQGMKKLSYIRIADTNITSIPQGLPPSLTELHLDGNKISRVDAAS

LKGLNNLAKLGLSFNSISAVDNGSLANTPHLRELHLDNNKLTRVPGGLAEHKYIQVVYLH

NNNISVVGSSDFCPPGHNTKKASYSGVSLFSNPVQYWEIQPSTFRCVYVRSAIQLGNYK

>ENSP00000223657; ASPN

MKEYVLLLFLALCSAKPFFSPSHIALKNMMLKDMEDTDDDDDDDDDDDDDDEDNSLFPTR

EPRSHFFPFDLFPMCPFGCQCYSRVVHCSDLGLTSVPTNIPFDTRMLDLQNNKIKEIKEN

DFKGLTSLYGLILNNNKLTKIHPKAFLTTKKLRRLYLSHNQLSEIPLNLPKSLAELRIHE

NKVKKIQKDTFKGMNALHVLEMSANPLDNNGIEPGAFEGVTVFHIRIAEAKLTSVPKGLP

PTLLELHLDYNKISTVELEDFKRYKELQRLGLGNNKITDIENGSLANIPRVREIHLENNK

LKKIPSGLPELKYLQIIFLHSNSIARVGVNDFCPTVPKMKKSLYSAISLFNNPVKYWEMQ

PATFRCVLSRMSVQLGNFGM

>ENSP00000327336; BGN

MWPLWRLVSLLALSQALPFEQRGFWDFTLDDGPFMMNDEEASGADTSGVLDPDSVTPTYS

AMCPFGCHCHLRVVQCSDLGLKSVPKEISPDTTLLDLQNNDISELRKDDFKGLQHLYALV

LVNNKISKIHEKAFSPLRKLQKLYISKNHLVEIPPNLPSSLVELRIHDNRIRKVPKGVFS

GLRNMNCIEMGGNPLENSGFEPGAFDGLKLNYLRISEAKLTGIPKDLPETLNELHLDHNK

IQAIELEDLLRYSKLYRLGLGHNQIRMIENGSLSFLPTLRELHLDNNKLARVPSGLPDLK

LLQVVYLHSNNITKVGVNDFCPMGFGVKRAYYNGISLFNNPVPYWEVQPATFRCVTDRLA

IQFGNYKK

>ENSMUSP00000035489; Fmod release 40

MQWASVLLLAGLCSLSQGQYDEDSHWWIQYLRNQQSTYYDPYDPYPYEPSEPYPYGVEEG

PAYAYGAPPPPEPRDCPQECDCPPNFPTAMYCDNRNLKYLPFVPSRMKYVYFQNNQISAI

QEGVFDNATGLLWVALHGNQITSDKVGRKVFSKLRHLERLYLDHNNLTRMPGPLPRSLRE

LHLDHNQISRVPNNALEGLENLTALYLHHNEIQEVGSSMRGLRSLILLDLSYNHLRRVPD

GLPSALEQLYLEHNNVYTVPDSYFRGSPKLLYVRLSHNSLTNNGLATNTFNSSSLLELDL

SYNQLQKIPPVNTNLENLYLQGNRINEFSISSFCTVVDVMNFSKLQVLRLDGNEIKRSAM

PVDAPLCLRLANLIEI

>ENSP00000347041; FMOD

MQWTSLLLLAGLFSLSQAQYEDDPHWWFHYLRSQQSTYYDPYDPYPYETYEPYPYGVDEG

PAYTYGSPSPPDPRDCPQECDCPPNFPTAMYCDNRNLKYLPFVPSRMKYVYFQNNQITSI

QEGVFDNATGLLWIALHGNQITSDKVGRKVFSKLRHLERLYLDHNNLTRMPGPLPRSLRE

LHLDHNQISRVPNNALEGLENLTALYLQHNEIQEVGSSMRGLRSLILLDLSYNHLRKVPD

GLPSALEQLYMEHNNVYTVPDSYFRGAPKLLYVRLSHNSLTNNGLASNTFNSSSLLELDL

SYNQLQKIPPVNTNLENLYLQGNRINEFSISSFCTVVDVVNFSKLQVLRLDGNEIKRSAM

PADAPLCLRLASLIEI

>ENSMUSP00000040877; Lum

MNVCAFSLALALVGSVSGQYYDYDIPLFMYGQISPNCAPECNCPHSYPTAMYCDDLKLKS

VPMVPPGIKYLYLRNNQIDHIDEKAFENVTDLQWLILDHNLLENSKIKGKVFSKLKQLKK

LHINYNNLTESVGPLPKSLQDLQLTNNKISKLGSFDGLVNLTFIYLQHNQLKEDAVSASL

KGLKSLEYLDLSFNQMSKLPAGLPTSLLTLYLDNNKISNIPDEYFKRFTGLQYLRLSHNE

LADSGVPGNSFNISSLLELDLSYNKLKSIPTVNENLENYYLEVNELEKFDVKSFCKILGP

LSYSKIKHLRLDGNPLTQSSLPPDMYECLRVANEITVN

>ENSP00000266718; LUM

MSLSAFTLFLALIGGTSGQYYDYDFPLSIYGQSSPNCAPECNCPESYPSAMYCDELKLKS

VPMVPPGIKYLYLRNNQIDHIDEKAFENVTDLQWLILDHNLLENSKIKGRVFSKLKQLKK

LHINHNNLTESVGPLPKSLEDLQLTHNKITKLGSFEGLVNLTFIHLQHNRLKEDAVSAAF

KGLKSLEYLDLSFNQIARLPSGLPVSLLTLYLDNNKISNIPDEYFKRFNALQYLRLSHNE

LADSGIPGNSFNVSSLVELDLSYNKLKNIPTVNENLENYYLEVNQLEKFDIKSFCKILGP

LSYSKIKHLRLDGNRISETSLPPDMYECLRVANEVTLN

>ENSMUSP00000048803; Prelp

MRASFFWLLPLLLILASVAQGQPTRPKPGIRRKPKPRPTPRFPQAPEPAEPTDLPPPLPP

GPPSVFPDCPRECYCPPDFPSALYCDSRNLRRVPVIPPRIHYLYLQNNFITELPLESFQN

ATGLRWVNLDNNRIRKVDQRVLGKLPSLAFLYMEKNQLEEVPSALPRNLEQLRLSQNLIS

RIPPGVFSKLENLLLLDLQHNRLSDGVFKADTFQGLKNLMQLNLAHNILRKMPPKVPQAI

HQLYLDSNKIETIPNGYFKDFPNLAFIRMNYNKLSDRGLPKNSFNISNLLVLHLSHNKIS

NVPAISNKLEHLYLNNNSIEKINGTQICPNNLVAFHDFSSDLENVPHLRYLRLDGNFLKP

PIPLDLMMCFRLLQSVVI

>ENSP00000343924; PRELP

MRSPLCWLLPLLILASVAQGQPTRRPRPGTGPGRRPRPRPRPTPSFPQPDEPAEPTDLPP

PLPPGPPSIFPDCPRECYCPPDFPSALYCDSRNLRKVPVIPPRIHYLYLQNNFITELPVE

SFQNATGLRWINLDNNRIRKIDQRVLEKLPGLVFLYMEKNQLEEVPSALPRNLEQLRLSQ

NHISRIPPGVFSKLENLLLLDLQHNRLSDGVFKPDTFHGLKNLMQLNLAHNILRKMPPRV

PTAIHQLYLDSNKIETIPNGYFKSFPNLAFIRLNYNKLTDRGLPKNSFNISNLLVLHLSH

NRISSVPAINNRLEHLYLNNNSIEKINGTQICPNDLVAFHDFSSDLENVPHLRYLRLDGN

YLKPPIPLDLMMCFRLLQSVVI

>ENSMUSP00000020091; Kera

MATPNCLILWVLLIADTVWTQSVRQAYEIQDPEDWDVHDDFYCPRECFCPPSFPTALYCE

NRGLTEIPPIPSRIWYLYLENNLIESIPEKPFENATQLRWINLNKNKITNYGIEKGALSQ

LKKLLFLFLEDNELEEVPSPLPRSLEQLQLARNKVSRIPQGTFSNLENLTLLDLQHNKLL

DNAFQRDTFKGLKNLMQLNMAKNALRNMPPRLPANTMQLFLDNNSIEGIPENYFNVIPKV

AFLRLNHNKLSDAGLPSRGFDVSSILDLQLSYNQLTNFPRINANLQHLHLDHNKIKNVNM

SVICPTTLRAEQDAFIHGPQLSYLRLDGNEIKPPIPIDLVACFKSSGFHNIRQNPNWFKG

RCMWYEILIVTSRLDHTCNCSYPPFSYVQLVLSV

>ENSP00000266719; KERA

MAGTICFIMWVLFITDTVWSRSVRQVYEVHDSDDWTIHDFECPMECFCPPSFPTALYCEN

RGLKEIPAIPSRIWYLYLQNNLIETIPEKPFENATQLRWINLNKNKITNYGIEKGALSQL

KKLLFLFLEDNELEEVPSPLPRSLEQLQLARNKVSRIPQGTFSNLENLTLLDLQNNKLVD

NAFQRDTFKGLKNLMQLNMAKNALRNMPPRLPANTMQLFLDNNSIEGIPENYFNVIPKVA

FLRLNHNKLSDEGLPSRGFDVSSILDLQLSHNQLTKVPRISAHLQHLHLDHNKIKSVNVS

VICPSPSMLPAERDSFSYGPHLRYLRLDGNEIKPPIPMALMTCFRLLQAVII

>ENSMUSP00000065706; Omd

MGFLSPIYVLFFCFGVRVYCQYEAYRWDDDYDQEPNEDYDPEFQFHQNIEYGVPFYNNIL

GCAKECFCPTNFPTSMYCDNRKLKTIPIIPMHIQQLNLQFNDIEAVTANSFINATHLKEI

NLSHNKIKSQKIDYGVFAKLSNLQQLHLEHNNLEEFPFPLPKSLERLLLGYNEISILPTN

AMDGLVNVTMLDLCYNHLSDSMLKEKTLSKMEKLMQLNLCNNRLESMPLGLPSSLMYLSL

ENNSISSIPDNYFDKLPKLHALRISHNKLEDIPYDIFNLSNLIELNVGHNKLKQAFYIPR

NLEHLYLQNNEIESINVTMICPSPDPVHHHHLTYLRVDQNKLKEPISSYIFFCFPRIHSI

YYGEQRSTNGETIQLKTQVFRSYQEEEEEDDHDSQDNTLEGQEVSDEHYNSHYYEMQEWQ

DTI

>ENSP00000247535; OMD

MGFLSPIYVIFFFFGVKVHCQYETYQWDEDYDQEPDDDYQTGFPFRQNVDYGVPFHQYTL

GCVSECFCPTNFPSSMYCDNRKLKTIPNIPMHIQQLYLQFNEIEAVTANSFINATHLKEI

NLSHNKIKSQKIDYGVFAKLPNLLQLHLEHNNLEEFPFPLPKSLERLLLGYNEISKLQTN

AMDGLVNLTMLDLCYNYLHDSLLKDKIFAKMEKLMQLNLCSNRLESMPPGLPSSLMYLSL

ENNSISSIPEKYFDKLPKLHTLRMSHNKLQDIPYNIFNLPNIVELSVGHNKLKQAFYIPR

NLEHLYLQNNEIEKMNLTVMCPSIDPLHYHHLTYIRVDQNKLKEPISSYIFFCFPHIHTI

YYGEQRSTNGQTIQLKTQVFRRFPDDDDESEDHDDPDNAHESPEQEGAEGHFDLHYYENQ

E

>ENSMUSP00000010455; Optc

MKFLAFLSLLSLVLQKAETASLLGEREREEQSPEEGDTYASLYVGNHTLSIEDYNEVIDL

SNYEELADYGDQIPEAKISNLTLPTRTSPTSTVAQKTLSPNLTMAVPTTTGLLNSQSSHG

LPTCLVCVCLGSSVYCDDADLENIPPLPQMTTYLYARFNHISHIQAGDFKGLTKLRRIDL

SGNSISSIHNDALRLLPALQDLILPENQLAALPVLPSGIEFLDVRLNRLQSSGIQPEAFV

ALKKLQFLYLANNMLDSIPGPLPLSLRSLHLQNNMIETMESDTFCDTGEHRHERRQLEDI

RLDGNPINLSLFPEAYFCLPRLPVGHFT

>ENSP00000343233; OPTC

MRLLAFLSLLALVLQETGTASLPRKERKRREEQMPREGDSFEVLPLRNDVLNPDNYGEVI

DLSNYEELTDYGDQLPEVKVTSLAPATSISPAKSTTAPGTPSSNPTMTRPTTAGLLLSSQ

PNHGLPTCLVCVCLGSSVYCDDIDLEDIPPLPRRTAYLYARFNRISRIRAEDFKGLTKLK

RIDLSNNLISSIDNDAFRLLHALQDLILPENQLEALPVLPSGIEFLDVRLNRLQSSGIQP

AAFRAMEKLQFLYLSDNLLDSIPGPLPLSLRSVHLQNNLIETMQRDVFCDPEEHKHTRRQ

LEDIRLDGNPINLSLFPSAYFCLPRLPIGRFT

>ENSMUSP00000020094; Dspg3

MGMLARVALGLIIIDAVLAAPTTELFNYDSEVYDAILEDTGTFYNYEHIPDNHVENEKVS

ERLSGNRELLTPGPQLGDNQDEDKDEESTPRLIDGSSPQEPEFPGLLGPHTNEDFPTCLL

CTCISTTVYCDDHELDAIPPLPKKTTYFYSRFNRIKKINKNDFASLNDLKRIDLTSNLIS

EIDEDAFRKLPHLQELVLRDNKIKQLPELPNTLTFIDISNNRLGRKGIKQEAFKDMYDLH

HLYITDNSLDHIPLPLPESLRALHLQNNDILEMHEDTFCNVKNLTYVRKALEDIRLDGNP

INLSRTPQAYMCLPRLPIGSFI

>ENSP00000261172; DSPG3

MKTLAGLVLGLVIFDAAVTAPTLESINYDSETYDATLEDLDNLYNYENIPVDKVEIEIAT

VMPSGNRELLTPPPQPEKAQEEEEEEESTPRLIDGSSPQEPEFTGVLGPHTNEDFPTCLL

CTCISTTVYCDDHELDAIPPLPKNTAYFYSRFNRIKKINKNDFASLSDLKRIDLTSNLIS

EIDEDAFRKLPQLRELVLRDNKIRQLPELPTTLTFIDISNNRLGRKGIKQEAFKDMYDLH

HLYLTDNNLDHIPLPLPENLRALHLQNNNILEMHEDTFCNVKNLTYIRKALEDIRLDGNP

INLSKTPQAYMCLPRLPVGSLV

>ENSMUSP00000021822; Ogn

METVHSTFLLLLFVPLTQQAPQSQLDSHVNYEYATGNSEETKFSQDYEDKYLDGKSIKEK

ETMIIPDEKSLQLQKDEVIPSLPTKKENDEMPTCLLCVCLSGSVYCEEVDIDAVPPLPKE

SAYLYARFNKIKKLTAKDFADMPNLRRLDFTGNLIEDIEDGTFSKLSLLEELTLAENQLL

RLPVLPPKLTLLNAKHNKIKSKGIKANTFKKLNKLSFLYLDHNDLESVPPNLPESLRVIH

LQFNSISSLTDDTFCKANDTRYIRERIEEIRLEGNPIALGKHPNSFICLKRLPIGSYF

>ENSP00000262551; OGN

MKTLQSTLLLLLLVPLIKPAPPTQQDSRIIYDYGTDNFEESIFSQDYEDKYLDGKNIKEK

ETVIIPNEKSLQLQKDEAITPLPPKKENDEMPTCLLCVCLSGSVYCEEVDIDAVPPLPKE

SAYLYARFNKIKKLTAKDFADIPNLRRLDFTGNLIEDIEDGTFSKLSLLEELSLAENQLL

KLPVLPPKLTLFNAKYNKIKSRGIKANAFKKLNNLTFLYLDHNALESVPLNLPESLRVIH

LQFNNIASITDDTFCKANDTSYIRDRIEEIRLEGNPIVLGKHPNSFICLKRLPIGSYF

>XP_485967; Lrrc32

MSHQILLLLAMLTLGLAISQRREQVPCRTVNKEALCHGLGLLQVPSVLSLDIQALYLSGN

QLQSILVSPLGFYTALRHLDLSDNQISFLQAGVFQALPYLEHLNLAHNRLATGMALNSGG

LGRLPLLVSLDLSGNSLHGNLVERLLGETPRLRTLSLAENSLTRLARHTFWGMPAVEQLD

LHSNVLMDIEDGAFEALPHLTHLNLSRNSLTCISDFSLQQLQVLDLSCNSIEAFQTAPEP

QAQFQLAWLDLRENKLLHFPDLAVFPRLIYLNVSNNLIQLPAGLPRGSEDLHAPSEGWSA

SPLSNPSRNASTHPLSQLLNLDLSYNEIELVPASFLEHLTSLRFLNLSRNCLRSFEARQV

DSLPCLVLLDLSHNVLEALELGTKVLGSLQTLLLQDNALQELPPYTFASLASLQRLNLQG

NQVSPCGGPAEPGPPGCVDFSGIPTLHVLNMAGNSMGMLRAGSFLHTPLTELDLSTNPGL

DVATGALVGLEASLEVLELQGNGLTVLRVDLPCFLRLKRLNLAENQLSHLPAWTRAVSLE

VLDLRNNSFSLLPGNAMGGLETSLRRLYLQGNPLSCCGNGWLAAQLHQGRVDVDATQDLI

CRFGSQEELSLSLVRPEDCEKGGLKNVNLILLLSFTLVSAIVLTTLATICFLRRQKLSQQ

YKA

>ENSP00000260061; LRRC32

MRPQILLLLALLTLGLAAQHQDKVPCKMVDKKVSCQVLGLLQVPSVLPPDTETLDLSGNQ

LRSILASPLGFYTALRHLDLSTNEISFLQPGAFQALTHLEHLSLAHNRLAMATALSAGGL

GPLPRVTSLDLSGNSLYSGLLERLLGEAPSLHTLSLAENSLTRLTRHTFRDMPALEQLDL

HSNVLMDIEDGAFEGLPRLTHLNLSRNSLTCISDFSLQQLRVLDLSCNSIEAFQTASQPQ

AEFQLTWLDLRENKLLHFPDLAALPRLIYLNLSNNLIRLPTGPPQDSKGIHAPSEGWSAL

PLSAPSGNASGRPLSQLLNLDLSYNEIELIPDSFLEHLTSLCFLNLSRNCLRTFEARRLG

SLPCLMLLDLSHNALETLELGARALGSLRTLLLQGNALRDLPPYTFANLASLQRLNLQGN

RVSPCGGPDEPGPSGCVAFSGITSLRSLSLVDNEIELLRAGAFLHTPLTELDLSSNPGLE

VATGALGGLEASLEVLALQGNGLMVLQVDLPCFICLKRLNLAENRLSHLPAWTQAVSLEV

LDLRNNSFSLLPGSAMGGLETSLRRLYLQGNPLSCCGNGWLAAQLHQGRVDVDATQDLIC

RFSSQEEVSLSHVRPEDCEKGGLKNINLIIILTFILVSAILLTTLAACCCVRRQKFNQQY

KA

>ENSMUSP00000067130; Lrrc33 CURATED

MEFPPLWLCLGFHFLIVEWRSGPGTATAASQG

GCKVVDGVADCRGLNLASVPSSLPPHSRMLILDANPLKDLWNHSLQAYPRLENLSLHSCH

LDRISHYAFREQGHLRNLVLADNRLSENYKESAAALHTLLGLRRLDLSGNSLTEDMAALM

LQNLSSLEVVSLARNTLMRLDDSIFEGLEHLVELDLQRNYIFEIEGGAFDGLTELRRLNL

AYNNLPCIVDFSLTQLRFLNVSYNILEWFLAAREEVAFELEILDLSHNQLLFFPLLPQCG

KLHTLLLQDNNMGFYRELYNTSSPQEMVAQFLLVDGNVTNITTVNLWEEFSSSDLSALRF

LDMSQNQFRHLPDGFLKKTPSLSHLNLNQNCLKMLHIREHEPPGALTELDLSHNQLAELH

LAPGLTGSLRNLRVFNLSSNQLLGVPTGLFDNASSITTIDMSHNQISLCPQMVPVDWEGP

PSCVDFRNMGSLRSLSLDGCGLKALQDCPFQGTSLTHLDLSSNWGVLNGSISPLWAVAPT

LQVLSLRDVGLGSGAAEMDFSAFGNLRALDLSGNSLTSFPKFKGSLALRTLDLRRNSLTA

LPQRVVSEQPLRGLQTIYLSQNPYDCCGVEGWGALQQHFKTVADLSMVTCNLSSKIVRVV

ELPEGLPQGCKWEQVDTGLFYLVLILPSCLTLLVACTVVFLTFKKPLLQVIKSRCHWSSI

Y

>ENSP00000328625; LRRC33

MELLPLWLCLGFHFLTVGWRNRSGTATAASQGVCKLVGGAADCRGQSLASVPSSLPPHAR

MLTLDANPLKTLWNHSLQPYPLLESLSLHSCHLERISRGAFQEQGHLRSLVLGDNCLSEN

YEETAAALHALPGLRRLDLSGNALTEDMAALMLQNLSSLRSVSLAGNTIMRLDDSVFEGL

ERLRELDLQRNYIFEIEGGAFDGLAELRHLNLAFNNLPCIVDFGLTRLRVLNVSYNVLEW

FLATGGEAAFELETLDLSHNQLLFFPLLPQYSKLRTLLLRDNNMGFYRDLYNTSSPREMV

AQFLLVDGNVTNITTVSLWEEFSSSDLADLRFLDMSQNQFQYLPDGFLRKMPSLSHLNLH

QNCLMTLHIREHEPPGALTELDLSHNQLSELHLAPGLASCLGSLRLFNLSSNQLLGVPPG

LFANARNITTLDMSHNQISLCPLPAASDRVGPPSCVDFRNMASLRSLSLEGCGLGALPDC

PFQGTSLTYLDLSSNWGVLNGSLAPLQDVAPMLQVLSLRNMGLHSSFMALDFSGFGNLRD

LDLSGNCLTTFPRFGGSLALETLDLRRNSLTALPQKAVSEQLSRGLRTIYLSQNPYDCCG

VDGWGALQHGQTVADWAMVTCNLSSKIIRVTELPGGVPRDCKWERLDLGLLYLVLILPSC

LTLLVACTVIVLTFKKPLLQVIKSRCHWSSVY

>ENSMUSP00000057725; Rtn4rl2

MLPGLRRLLQGPASACLLLTLLALPSVTPSCPMLCTCYSSPPTVSCQANNFSSVPLSLPP

STQRLFLQNNLIRSLRPGTFGPNLLTLWLFSNNLSTIHPGTFRHLQALEELDLGDNRHLR

SLEPDTFQGLERLQSLHLYRCQLSSLPGNIFRGLVSLQYLYLQENSLLHLQDDLFADLAN

LSHLFLHGNRLRLLTEHVFRGLGSLDRLLLHGNRLQGVHRAAFHGLSRLTILYLFNNSLA

SLPGEALADLPALEFLRLNANPWACDCRARPLWAWFQRARVSSSDVTCATPPERQGRDLR

ALRDSDFQACPPPTPTRPGSRARGNSSSNHLYGVAEAGAPPADPSTLYRDLPAEDSRGRQ

GGDAPTEDDYWGGYGGEDQRGEQTCPGAACQAPADSRGPALSAGLRTPLLCLLPLALHHL

>ENSP00000335397; RTN4RL2

MLPGLRRLLQAPASACLLLMLLALPLAAPSCPMLCTCYSSPPTVSCQANNFSSVPLSLPP

STQRLFLQNNLIRTLRPGTFGSNLLTLWLFSNNLSTIYPGTFRHLQALEELDLGDNRHLR

SLEPDTFQGLERLQSLHLYRCQLSSLPGNIFRGLVSLQYLYLQENSLLHLQDDLFADLAN

LSHLFLHGNRLRLLTEHVFRGLGSLDRLLLHGNRLQGVHRAAFRGLSRLTILYLFNNSLA

SLPGEALADLPSLEFLRLNANPWACDCRARPLWAWFQRARVSSSDVTCATPPERQGRDLR

ALREADFQACPPAAPTRPGSRARGNSSSNHLYGVAEAGAPPADPSTLYRDLPAEDSRGRQ

GGDAPTEDDYWGGYGGEDQRGEQMCPGAACQAPPDSRGPALSAGLPSPLLCLLLLVPHHL

>BC058381; Rtn4r

MKRASSGGSRLLAWVLWLQAWRVATPCPGACVCYNEPKVTTSCP

QQGLQAVPTGIPASSQRIFLHGNRISHVPAASFQSCRNLTILWLHSNALARIDAAAFT

GLTLLEQLDLSDNAQLHVVDPTTFHGLGHLHTLHLDRCGLRELGPGLFRGLAALQYLY

LQDNNLQALPDNTFRDLGNLTHLFLHGNRIPSVPEHAFRGLHSLDRLLLHQNHVARVH

PHAFRDLGRLMTLYLFANNLSMLPAEVLMPLRSLQYLRLNDNPWVCDCRARPLWAWLQ

KFRGSSSEVPCNLPQRLADRDLKRLAASDLEGCAVASGPFRPIQTSQLTDEELLSLPK

CCQPDAADKASVLEPGRPASAGNALKGRVPPGDTPPGNGSGPRHINDSPFGTLPSSAE

PPLTALRPGGSEPPGLPTTGPRRRPGCSRKNRTRSHCRLGQAGSGASGTGDAEGSGAL

PALACSLAPLGLALVLWTVLGPC

>ENSP00000043402; **RTN4R**

MKRASAGGSRLLAWVLWLQAWQVAAPCPGACVCYNEPKVTTSCPQQGLQAVPVGIPAASQ

RIFLHGNRISHVPAASFRACRNLTILWLHSNVLARIDAAAFTGLALLEQLDLSDNAQLRS

VDPATFHGLGRLHTLHLDRCGLQELGPGLFRGLAALQYLYLQDNALQALPDDTFRDLGNL

THLFLHGNRISSVPERAFRGLHSLDRLLLHQNRVAHVHPHAFRDLGRLMTLYLFANNLSA

LPTEALAPLRALQYLRLNDNPWVCDCRARPLWAWLQKFRGSSSEVPCSLPQRLAGRDLKR

LAANDLQGCAVATGPYHPIWTGRATDEEPLGLPKCCQPDAADKASVLEPGRPASAGNALK

GRVPPGDSPPGNGSGPRHINDSPFGTLPGSAEPPLTAVRPEGSEPPGFPTSGPRRRPGCS

RKNRTRSHCRLGQAGSGGGGTGDSEGSGALPSLTCSLTPLGLALVLWTVLGPC

>BC030471; Rtn4rl1

MLRKGCCVELLLLLLAGELPLGGGCPRDCVCYPAPMTVSCQAHN

FAAIPEGIPEDSERIFLQNNRITFLQQGHFSPAMVTLWIYSNNITFIAPNTFEGFVHL

EELDLGDNRQLRTLAPETFQGLVKLHALYLYKCGLSALPAGIFGGLHSLQYLYLQDNH

IEYLQDDIFVDLVNLSHLFLHGNKLWSLGQGIFRGLVNLDRLLLHENQLQWVHHKAFH

DLHRLTTLFLFNNSLTELQGDCLAPLVALEFLRLNGNAWDCGCRARSLWEWLRRFRGS

SSAVPCATPELRQGQDLKLLRVEDFRNCTGPVSPHQIKSHTLTTSDRAARKEHHPSHG

ASRDKGHPHGHPPGSRSGYKKAGKNCTSHRNRNQISKVSSGKELTELQDYAPDYQHKF

SFDIMPTARPKRKGKCARRTPIRAPSGVQQASSGTALGAPLLAWILGLAVTLR

>NM_178568; RTN4RL1

MLRKGCCVELLLLLVAAELPLGGGCPRDCVCYPAPMTVSCQAHN

FAAIPEGIPVDSERVFLQNNRIGLLQPGHFSPAMVTLWIYSNNITYIHPSTFEGFVHL

EELDLGDNRQLRTLAPETFQGLVKLHALYLYKCGLSALPAGVFGGLHSLQYLYLQDNH

IEYLQDDIFVDLVNLSHLFLHGNKLWSLGPGTFRGLVNLDRLLLHENQLQWVHHKAFH

DLRRLTTLFLFNNSLSELQGECLAPLGALEFLRLNGNPWDCGCRARSLWEWLQRFRGS

SSAVPCVSPGLRHGQDLKLLRAEDFRNCTGPASPHQIKSHTLTTTDRAARKEHHSPHG

PTRSKGHPHGPRPGHRKPGKNCTNPRNRNQISKAGAGKQAPELPDYAPDYQHKFSFDI

MPTARPKRKGKCARRTPIRAPSGVQQASSASSLGASLLAWTLGLAVTLR

>XP_984483;

MPLLAPRLLPLLLVIGTGGSVPRPLALPQGCYVAEEAGEQTFRC

SRAGLSAVPNGIPNDTRKLYLDANQLASVPAGAFQHLPALEELDLSHNALVHLSGAAF

QGLEGTLRHLDLSANQLASVPVAAFVGLQIQVNLSANPWRCDCALQEVLRHVRLAPGS

GTGIVCGPEARPDLVGHEFLLLTREEELCGTGRGGTRRSTDVALLVTMGGWLTLVVAY

LIRYVWQNRDETRRPVKRAPPAQPVRSEDSSTLSTVL

>ENSP00000367157; only a partial sequence

LSLIRPLVSITRVPTRPSGAYLAHLAATQVPPRGCYVAKEAGERTFRCSQAGLSAVPSGI

PNDTRKLYLDANQLASVPAGAFQHLPVLEELDLSHNALAHLSGAAFQGLEGTLRHLDLSA

NQLASVPVEAFVGLQIQVNLSANPWHCDCALQEVLRQVRLVPGTGTGIVCGSGARPDLVG

QEFLLLAGEEELCGSGWGGARRSTDVALLVTMGGWLTLMVAYLVHYVWQNRDETRRSLKR

APVLP

>XP_001126626

mtsssfvsyc tpglcqfmam lptaghllpl llvigtggtv pspqvpprgc yvakeagert

frcsqaglsa vpsgipndtr klyldanqla svpagafqhl pvleeldlsh nalahlsgaa

fqglegtlrh ldlsanqlas vpveafvglq iqvnlsanpw hcdcalqevl rqvrlvpgtg

tgivcgsgar pdlvgqefll lageeelcgs gwggarrstd vallvtmggw ltlmvaylvh

yvwqnrdetr rslkrapvlp vrsedssils tvv

>ENSMUSP00000059570; Lrrc3 NM_145152

MGPRGRQSPSATLAPSQGSCFFILFCLRLGASCPQACQCPDHAGAVAVHCSSRGLQEIPR

DIPADTVLLKLDANRISRVPNGAFQHLPQLRELDLSHNAIEAIGPAAFSGLAGGLRLLDL

SHNRIRRIPKDALGKLSAKIRLSHNPLHCECALQEALWELKLDPDSVDEIACHTSAQEQF

VGKPLIQVLDSGASFCSTHRKTTDVAMLVTMFGWFTMVIAYVVYYVRHNQEDARRHLEYL

KSLPSAPVSKEPLSPVP

>ENSP00000291592; LRRC3

MGTVRPPRPSLLLVSTRESCLFLLFCLHLGAACPQPCRCPDHAGAVAVFCSLRGLQEVPE

DIPANTVLLKLDANKISHLPDGAFQHLHRLRELDLSHNAIEAIGSATFAGLAGGLRLLDL

SYNRIQRIPKDALGKLSAKIRLSHNPLHCECALQEALWELKLDPDSVDEIACHTSVQEEF

VGKPLVQALDAGASLCSVPHRTTDVAMLVTMFGWFAMVIAYVVYYVRHNQEDARRHLEYL

KSLPSAPASKDPIGPGP

>ENSMUSP00000059463; Lrrc3b

MNLVDLWLSRSLSMCLLLQSFVLMILCFHSASMCPKGCLCSSSGGLNVTCSNANLKEIPR

DLPPETVLLYLDSNQITSIPNEIFKDLHQLRVLNLSKNGIEFIDEHAFKGVAETLQTLDL

SDNRIQSVHKNAFNNLKARARIANNPWHCDCTLQQVLRSMASNHETAHNVICKTSVLDEH

AGRPFLNAANDADLCNLPKKTTDYAMLVTMFGWFTMVISYVVYYVRQNQEDARRHLEYLK

SLPSRQKKADEPDDISTVV

>ENSP00000326763; LRRC3B

MNLVDLWLTRSLSMCLLLQSFVLMILCFHSASMCPKGCLCSSSGGLNVTCSNANLKEIPR

DLPPETVLLYLDSNQITSIPNEIFKDLHQLRVLNLSKNGIEFIDEHAFKGVAETLQTLDL

SDNRIQSVHKNAFNNLKARARIANNPWHCDCTLQQVLRSMASNHETAHNVICKTSVLDEH

AGRPFLNAANDADLCNLPKKTTDYAMLVTMFGWFTMVISYVVYYVRQNQEDARRHLEYLK

SLPSRQKKADEPDDISTVV

>XP_001001535; XP_001001535

MERPQSSIWVFMLLLFMVLLQSPAWHVAAQRCPQTCVCDNSRRH

VTCRHQNLTEVPNTIPELTQRLDLQGNILKVLPAAAFQDLPHLTHLDLRNCQVEMVAE

GAFRGLGRLLLLNLASNRLSTLPQEALDGLGSLRRLELEGNMLEELRPGTFGALGSLT

TLNLAHNALVYLPAMAFQGLLRTRWLQLSHNALSVLAPEALAGLPALRRLSLHHNELQ

ALPGAALSQARSLARLELGHNPLTYTGEEDGLALPGLRELALDHGSLQALGPRAFAHC

PRLHTLDLRGNQLTTLPPLQVPGQLRRLRLQGNPLWCACHARPLLEWLVRARVRSDGA

CRGPRRLRGEALDTLRPSDLRCPGDAAAGDGDGDEDEDRPAGPRAPPLRSPHGEAAWA

TPCPPACACVAETRHSTCDGRGLQAVPRGFPNDTQLLDLRRNHFPSVPRAAFPGLRHL

VSLHLQHCGVAELEPGALAGLDRLLYLYLSHNQLSGLSAAALEGAPNLGYLYLEHNRF

LRIPGTALRALPTLVSLHLQDNAVDRLAPGDLAGARALRCLYLSGNHITQVSPGALGP

ARELEKLHLDRNRLREVPTGALEGLPALKELQLSGNPLRALPDGAFQPVGRSLQQLFL

NSSDLEQISPRAFSGLGKGLRSLYLHKNQLQSLPAPLGLSGLELVDLSGNPFHCDCQL

LPLHRWLTGLNLRVGATCATPPSVRGQKVKVAAPVFEACPGWTARKAKRTPTSRGSAR

RTPSLSRH

>ENSP00000216241; Q5JY13_HUMAN

MEGPRSSTHVPLVLPLLVLLLLAPARQAAAQRCPQACICDNSRRHVACRYQNLTEVPDAI

PELTQRLDLQGNLLKVIPAAAFQGVPHLTHLDLRHCEVELVAEGAFRGLGRLLLLNLASN

HLRELPQEALDGLGSLRRLELEGNALEELRPGTFGALGALATLNLAHNALVYLPA

MAFQG

LLRVRWLRLSHNALSVLAPEALAGLPALRRLSLHHNELQALPGPVLSQARGLARLELGHN

PLTYAGEEDGLALPGLRELLLDGGALQALGPRAFAHCPRLHTLDLRGNQLDTLPPLQGPG

QLRRLRLQGNPLWCGCQARPLLEWLARARVRSDGACQGPRRLRGEALDALRPWDLRCPGD

AAQEEEELEERAVAGPRAPPRGPPRGPGEERAVAPCPRACVCVPESRHSSCEGCGLQAVP

RGFPSDTQLLDLRRNHFPSVPRAAFPGLGHLVSLHLQHCGIAELEAGALAGLGRLIYLYL

SDNQLAGLSAAALEGAPRLGYLYLERNRFLQVPGAALRALPSLFSLHLQDNAVDRLAPGD

LGRTRALRWVYLSGNRITEVSLGALGPARELEKLHLDRNQLREVPTGALEGLPALLELQL

SGNPLRALRDGAFQPVGRSLQHLFLNSSGLEQICPGAFSGLGPGLQSLHLQKNQLRALPA

LPSLSQLELIDLSSNPFHCDCQLLPLHRWLTGLNLRVGATCATPPNARGQRVKAAAAVFE

DCPGWAARKAKRTPASRPSARRTPIKGRQCGADKVGHGAGGV

>ENSMUSP00000047844; Chad

MARALLFSLVFLAILLPALAACPQNCHCHGDLQHVICDKVGLQKIPKVSETTKLLNLQRN

NFPVLAANSFRTMPNLVSLHLQHCNIREVAAGAFRGLKQLIYLYLSHNDIRVLRAGAFDD

LTELTYLYLDHNKVSELPRGLLSPLVNLFILQLNNNKIRELRAGAFQGAKDLRWLYLSEN

ALSSLQPGSLDDVENLAKFHLDKNQLSSYPSAALSKLRVVEELKLSHNPLKSIPDNAFQS

FGRYLETLWLDNTNLEKFSDAAFSGVTTLKHVHLDNNRLNQLPSSFPFDNLETLTLTNNP

WKCTCQLRGLRRWLEAKASRPDATCSSPAKFKGQRIRDTDALRSCKSPTKRSKKAGRH

>ENSP00000258969; CHAD

MVRPMLLLSLGLLAGLLPALAACPQNCHCHSDLQHVICDKVGLQKIPKVSEKTKLLNLQR

NNFPVLAANSFRAMPNLVSLHLQHCQIREVAAGAFRGLKQLIYLYLSHNDIRVLRAGAFD

DLTELTYLYLDHNKVTELPRGLLSPLVNLFILQLNNNKIRELRAGAFQGAKDLRWLYLSE

NALSSLQPGALDDVENLAKFHVDRNQLSSYPSAALSKLRVVEELKLSHNPLKSIPDNAFQ

SFGRYLETLWLDNTNLEKFSDGAFLGVTTLKHVHLENNRLNQLPSNFPFDSLETLALTNN

PWKCTCQLRGLRRWLEAKASRPDATCASPAKFKGQHIRDTDAFRSCKFPTKRSKKAGRH

>ENSMUSP00000006559; Tpbg

MPGAGSRGPSAGDGRLRLARLALVLLGWVSASAPSSSVPSSSTSPAAFLASGSAQPPPAE

RCPAACECSEAARTVKCVNRNLLEVPADLPPYVRNLFLTGNQMTVLPAGAFARQPPLADL

EALNLSGNHLKEVCAGAFEHLPGLRRLDLSHNPLTNLSAFAFAGSNASVSAPSPLEELIL

NHIVPPEDQRQNGSFEGMVAFEGMVAAALRSGLALRGLTRLELASNHFLFLPRDLLAQLP

SLRYLDLRNNSLVSLTYASFRNLTHLESLHLEDNALKVLHNSTLAEWHGLAHVKVFLDNN

PWVCDCYMADMVAWLKETEVVPDKARLTCAFPEKMRNRGLLDLNSSDLDCDAVLPQSLQT

SYVFLGIVLALIGAIFLLVLYLNRKGIKKWMHNIRDACRDHMEGYHYRYEINADPRLTNL

SSNSDV

>ENSP00000275030; TPBG

MPGGCSRGPAAGDGRLRLARLALVLLGWVSSSSPTSSASSFSSSAPFLASAVSAQPPLPD

QCPALCECSEAARTVKCVNRNLTEVPTDLPAYVRNLFLTGNQLAVLPAGAFARRPPLAEL

AALNLSGSRLDEVRAGAFEHLPSLRQLDLSHNPLADLSPFAFSGSNASVSAPSPLVELIL

NHIVPPEDERQNRSFEGMVVAALLAGRALQGLRRLELASNHFLYLPRDVLAQLPSLRHLD

LSNNSLVSLTYVSFRNLTHLESLHLEDNALKVLHNGTLAELQGLPHIRVFLDNNPWVCDC

HMADMVTWLKETEVVQGKDRLTCAYPEKMRNRVLLELNSADLDCDPILPPSLQTSYVFLG

IVLALIGAIFLLVLYLNRKGIKKWMHNIRDACRDHMEGYHYRYEINADPRLTNLSSNSDV

>XP_001005865; XP_001005865

MAPRAGQRGLWSPLPGLLLLAAALSRPAAPCPFQCYCFGSPRLM

LRCASGAELRQPPRDVPPDARNLTIVGANLTVLRAAAFAGGGEGATDGVRLPLLTALR

LTHNNIEVVEDGAFDGLPSLAALDLSHNPLRALGYRAFRGLPALRSLQLNHALARGSP

GMLDALDAALAPLAELRLLGLVGNALSRLPLAALRLPRLEQLDARVNALAGLGPDELS

ALERDGDLPQPRLLLADNPLSCGCTSRPLLAWLHNATERVPDARRLRCASPRVLLDRP

LIDLDEARLGCSDGDAHESGEGIDVAGPELEASYVFFGLVLALIGLIFLMVLYLNRRG

IQRWMHNLREACRDQMEGYHYRYEQDADPRRAPAPAAPAGSRATSPGSGL

>XP_945324; XP_945324

MAPRAGQPGLQGLLLVAAALSQPAAPCPFQCYCFGGPKLLLRCA

SGAELRQPPRDVPPDARNLTIVGANLTVLRAAAFAGGDGDGDQAAGVRLPLLSALRLT

HNHIEVVEDGAFDGLPSLAALDLSHNPLRALGGGAFRGLPALRSLQLNHALVRGGPAL

LAALDAALAPLAELRLLGLAGNALSRLPPAALRLARLEQLDVRLNALAGLDPDELRAL

ERDGGLPGPRLLLADNPLRCGCAARPLLAWLRNATERVPDSRRLRCAAPRALLDRPLL

DLDGARLRCADSGADARGEEAEAAGPELEASYVFFGLVLALIGLIFLMVLYLNRRGIQ

RWMRNLREACRDQMEGYHYRYEQDADPRRAPAPAAPAGSRATSPGSGL

>NM_173415; Nyx

MLILLLHAVVFSLPYTRATEACLRACPAACTCSHVERGCSVRCD

RAGLQRVPQEFPCEAASIDLDRNGLRILGERAFGTLPSLRRLSLRHNNLSFITPGAFK

GLPRLAELRLAHNGELRYLHVRTFAALGRLRRLDLAACRLFSVPERLLAELPALRELT

AFDNLFRRVPGALRGLANLTHAHFERSRIEAVASGSLLGMRRLRSLSLQANRVRAVHA

GAFGDCGALEDLLLNDNLLATLPAAAFRGLRRLRTLNLGGNALGSVARAWFSDLAELE

LLYLDRNSITFVEEGAFQNLSGLLALHLNGNRLTVLSWAAFQPGFFLGRLFLFRNPWR

CDCQLEWLRDWMEGSGRVADVACASPGSVAGQDLSQVVFERSSDGLCVDPDELNFTTS

SPGPSPEPVATTVSRFSSLLSKLLAPRAPVEEVANTTWELVNVSLNDSFRSHAVMVFC

YKATFLFTSCVLLSLAQYVVVGLQRE

>ENSP00000340328; NYX

MKGRGMLVLLLHAVVLGLPSAWAVGACARACPAACACSTVERGCSVRCDRAGLLRVPAEL

PCEAVSIDLDRNGLRFLGERAFGTLPSLRRLSLRHNNLSFITPGAFKGLPRLAELRLAHN

GDLRYLHARTFAALSRLRRLDLAACRLFSVPERLLAELPALRELAAFDNLFRRVPGALRG

LANLTHAHLERGRIEAVASSSLQGLRRLRSLSLQANRVRAVHAGAFGDCGVLEHLLLNDN

LLAELPADAFRGLRRLRTLNLGGNALDRVARAWFADLAELELLYLDRNSIAFVEEGAFQN

LSGLLALHLNGNRLTVLAWVAFQPGFFLGRLFLFRNPWCCDCRLEWLRDWMEGSGRVTDV

PCASPGSVAGLDLSQVTFGRSSDGLCVDPEELNLTTSSPGPSPEPAATTVSRFSSLLSKL

LAPRVPVEEAANTTGGLANASLSDSLSSRGVGGAGRQPWFLLASCLLPSVAQHVVFGLQM

D

>ENSMUSP00000053597; Lrrc38

MSLCVAPRHPTGAAAALGLGSLLVLLGPGRACPAGCACTDPHTVDCRDRGLPSVPDPFPL

DVRKLLVAGNRIQQIPEDFFIFHGDLVYLDFRNNSLRSLEEGTFSGSGKLAFLDLSYNNL

TQLGAGAFRSAGRLVKLSLANNHLAGVHEAAFESLESLQVLELNDNNLRSLNVAALDALP

ALRTVRLDGNPWLCDCDFAHLFSWIQENTSKLPKGLDAIQCSLPMEDRRVALRELSEASF

SECKFSLSLTDLFIIIFSGVAVSIAAIISSFFLATVVQCFQRCAPNKDTEDEDDDEDD

>OTTHUMP00000009643; LRRC38

MRPRAPACAAAALGLCSLLLLLAPGHACPAGCACTDPHTVDCRDRGLPSVPDPFPLDVRK

LLVAGNRIQRIPEDFFIFYGDLVYLDFRNNSLRSLEEGTFSGSAKLVFLDLSYNNLTQLG

AGAFRSAGRLVKLSLANNNLVGVHEDAFETLESLQVLELNDNNLRSLSVAALAALPALRS

LRLDGNPWLCDCDFAHLFSWIQENASKLPKGLDEIQCSLPMESRRISLRELSEASFSECR

FSLSLTDLCIIIFSGVAVSIAAIISSFFLATVVQCLQRCAPNKDAEDEDEDKDD

>ENSMUSP00000041417; Lrrc55 Release 40

MGSLQHCCCQLPKMGDTWAQLPWPGPPHSALLLVFFLLAAGVMHSDAGTSCPVLCTCRNQ

VVDCSNQRLFSVPPDLPMDTRNLSLAHNRIAAVPPGYLTCYMELRVLDLRNNSLMELPPG

LFLHAKRLAHLDLSYNNLSHVPADMFREAHGLVHIDLSHNPWLRRVHPQAFQGLVHLRDL

DLSYGGLAFLSLEALEGLPGLVTLQIGGNPWVCGCTMEPLLKWLRNRIQRCTADSQLAEC

RGPPEVEGAPLFSLTEESFKACHLTLTLDDYLFIAFVGFVVSIASVATNFLLGITANCCH

RWSKANEEEEI

>ENSP00000330864; LRRC55

MLLISLLLAAGLMHSDAGTSCPVLCTCRNQVVDCSSQRLFSVPPDLPMDTRNLSLAHNRI

TAVPPGYLTCYMELQVLDLHNNSLMELPRGLFLHAKRLAHLDLSYNNFSHVPADMFQEAH

GLVHIDLSHNPWLRRVHPQAFQGLMQLRDLDLSYGGLAFLSLEALEGLPGLVTLQIGGNP

WVCGCTMEPLLKWLRNRIQRCTADSQLAECRGPPEVEGAPLFSLTEESFKACHLTLTLDD

YLFIAFVGFVVSIASVATNFLLGITANCCHRWSKASEEEEI

>ENSMUSP00000061828; Lrtm1

MLNEGLCCGAWAMKGTLLLVSSVGLLLPGVGSCPMKCLCHPSSNSVDCSGQGLSKVPRDL

PPWTVTLLLQDNRIHWLPALAFQSVSLLSTLNLSNNSLSNLAAEAFYGLPHLRVLNVTQN

SLLSIESSFAHALPGLRELDLSSNSLRILPTSLGKPWENLTVFAVQQNHLLHLDRELLEA

MPKVRLVLLKDNPWICDCHLLGLKLWLERFTFQGGETDGAICRLPEPWQGKALLSIPHEL

YQPCSLPSQDLAPSLVQQPGSAPQDAQKSHENSSGQQDPLECEAKPKPKPTNLRHAVATV

VITGVVCGIVCLMMLAAAIYGCTYAAITAQYQGRPLASARKSEKMGSKELMDSSSA

>ENSP00000273286; LRTM1

MKGELLLFSSVIVLLQVVCSCPDKCYCQSSTNFVDCSQQGLAEIPSHLPPQTRTLHLQDN

QIHHLPAFAFRSVPWLMTLNLSNNSLSNLAPGAFHGLQHLQVLNLTQNSLLSLESRLFHS

LPQLRELDLSSNNISHLPTSLGETWENLTILAVQQNQLQQLDRALLESMPSVRLLLLKDN

LWKCNCHLLGLKLWLEKFVYKGGLTDGIICESPDTWKGKDLLRIPHELYQPCPLPAPDPV

SSQAQWPGSAHGVVLRPPENHNAGERELLECELKPKPRPANLRHAIATVIITGVVCGIVC

LMMLAAAIYGCTYAAITAQYHGGPLAQTNDPGKVEEKERFDSSPA

>ENSMUSP00000063882; Lrtm2 AI841794

MLAPGGGPEQRSKLVLQWRQVSWITCWIALCAVEVIPACPFSCTCDSRSLEVDCSGLGLT

TVPPDVPAATQSLLLLNNKLSALPSWAFANLSNLQRLDLSNNFLDQLPRSIFEDLVNLTE

LQLRNNSIRTLDRDLLQHSPLLRHLDLSINGLAQLPPGLFDGLLALRSLSLRSNRLQSLD

RLTFEPLASLQLLQVGDNPWECDCNLREFKHWLEWFSYRGGRLDQLACTLPKELRGKDMR

AVPMEMFNYCSQLEDENNSAGLDAPGPPCTKASPEPPKPKPGAEPEPEPSTACPQKQRYR

PVSVRRAIGTVIIAGVVCGIVCIMMVVAAAYGCIYASLMAKYHRELKKRQPLMGDPEGEH

EDQKQISSVA

>ENSP00000299194; LRTM2

MLAPGSSPGQRGRLALQWRQVSWITCWIALYAVEALPTCPFSCKCDSRSLEVDCSGLGLT

TVPPDVPAATRTLLLLNNKLSALPSWAFANLSSLQRLDLSNNFLDRLPRSIFGDLTNLTE

LQLRNNSIRTLDRDLLRHSPLLRHLDLSINGLAQLPPGLFDGLLALRSLSLRSNRLQNLD

RLTFEPLANLQLLQVGDNPWECDCNLREFKHWMEWFSYRGGRLDQLACTLPKELRGKDMR

MVPMEMFNYCSQLEDENSSAGLDIPGPPCTKASPEPAKPKPGAEPEPEPSTACPQKQRHR

PASVRRAMGTVIIAGVVCGVVCIMMVVAAAYGCIYASLMAKYHRELKKRQPLMGDPEGEH

EDQKQISSVA

>ENSMUSP00000028337; BC004853

MRGSFFSRLPPQLSLLLLLSLRRVWTQEDIGTAPSKSPVAPECPEACSCSLGGKANCSAL

ALPAVPADLSWQVRSLLLDHNRVSALPPGAFANAGALLYLDLRENRLRSVHARAFWGLGV

LQWLDLSSNQLETLPPGTFAPLRALSFLSLAGNRLALLEPSILGPLPLLRVLSLQDNSLS

AIEAGLLNNLPALDVLRLHGNPWTCNCALRPLCTWLRKHPRPASETETLLCVSPRLQTLS

LLTAFPDAAFKQCTQSLAARDLAVVYALGPVSFLASLAICLALGSVLTACGARRRRRRRT

TVRHLLRRQLDPEGPPSLEDAGSPVTAAIQA

>ENSP00000360597; NP_001013675.1

MRGPSWSRPRPLLLLLLLLSPWPVWAQVSATASPSGSLGAPDCPEVCTCVPGGLASCSAL

SLPAVPPGLSLRLRALLLDHNRVRALPPGAFAGAGALQRLDLRENGLHSVHVRAFWGLGA

LQLLDLSANQLEALAPGTFAPLRALRNLSLAGNRLARLEPAALGALPLLRSLSLQDNELA

ALAPGLLGRLPALDALHLRGNPWGCGCALRPLCAWLRRHPLPASEAETVLCVWPGRLTLS

PLTAFSDAAFSHCAQPLALRDLAVVYTLGPASFLVSLASCLALGSGLTACRARRRRLRTA

ALRPPRPPDPNPDPDPHGCASPADPGSPAAAAQA

>ENSMUSP00000047213; Lrrc52

MSLASGPSSKLLLFSLGMGLVSGSKCPNKCVCQDQEVACIDLHLTEYPADIPLNTRRLYL

NNNKITSLPALQLGFLSDLVYLDCQNNRIREVMDYTFIGIFRLIYLDLSSNNLTSISPFS

FSVLTNLVRLNISHNPHLLYLDKYVFANTTSLRYLDLRNTGLHIIDHNGFHHLVVLQTLY

LSGNPWICNCSFLDFTIHLLVSHMDHPDAQNATCTEPAELKGWPITKVGNPLQYMCITHL

DQQDYIFLLLIGFCIFAAGTVAAWLTGVCAVLYQNALRTSSGDDTEDETGSRFANQIFRS

NTHLGPIRRFPELI

>ENSP00000294818; LRRC52

MSLASGPGPGWLLFSFGMGLVSGSKCPNNCLCQAQEVICTGKQLTEYPLDIPLNTRRLFL

NENRITSLPAMHLGLLSDLVYLDCQNNRIREVMDYTFIGVFKLIYLDLSSNNLTSISPFT

FSVLSNLVQLNIANNPHLLSLHKFTFANTTSLRYLDLRNTGLQTLDSAALYHLTTLETLF

LSGNPWKCNCSFLDFAIFLIVFHMDPSDDLNATCVEPTELTGWPITRVGNPLRYMCITHL

DHKDYIFLLLIGFCIFAAGTVAAWLTGVCAVLYQNTRHKSSEEDEDEAGTRVEVSRRIFQ

TQTSSVQEFPQLI

>ENSP00000339075; SYNLEURIN

MCGLQFSLPCLRLFLVVTCYLLLLLHKEILGCSSVCQLCTGRQINCRNLGLSSIPKNFPE

STVFLYLTGNNISYINESELTGLHSLVALYLDNSNILYVYPKAFVQLRHLYFLFLNNNFI

KRLDPGIFKGLLNLRNLYLQYNQVSFVPRVFNDLVSVQYLNLQRNRLTVLGSGTFVGMVA

LRILDLSNNNILRISESGFQHLENLACLYLGSNNLTKVPSNAFEVLKSLRRLSLSHNPIE

AIQPFAFKGLANLEYLLLKNSRIRNVTRDGFSGINNLKHLILSHNDLENLNSDTFSLLKN

LIYLKLDRNRIISIDNDTFENMGASLKILNLSFNNLTALHPRVLKPLSSLIHLQANSNPW

ECNCKLLGLRDWLASSAITLNIYCQNPPSMRGRALRYINITNCVTSSINVSRAWAVVKSP

HIHHKTTALMMAWHKVTTNGSPLENTETENITFWERIPTSPAGRFFQENAFGNPLETTAV

LPVQIQLTTSVTLNLEKNSALPNDAASMSGKTSLICTQEVEKLNEAFDILLAFFILACVL

IIFLIYKVVQFKQKLKASENSRENRLEYYSFYQSARYNVTASICNTSPNSLESPGLEQIR

LHKQIVPENEAQVILFEHSAL

>CG5820-PA; CG5820-PA

MSRQSTCRLVLLLAFTLALIHASHSNTVGPKIHFIHDDLTENELNSGEEVSPTESLPKIS

TPKVKDLPVETTTRSPTKKTTSSGTEKSLSEEAWNDLMEAAKHSQALADDPHFNKMLNTT

VAAGEKNSTDTDDSEYYDYDDDYNYFDEETVTISPSPKKAKSNPKLQPIVDATITKSKSK

SNDQKTKESANDIDSDSQYDYDEGLDDDDDEDDAADDLLTDDEQVVFSEDVPCPRFCQCA

RNVNSYLVATCSRLDMGIQKFGSDITDLVVTNVGPKYPILMGPNFFQNLGLKNVASIKIA

NCTLEYLHAEAFHGLNELYAVNLTDVGLAIINPDTFVGNKKLRMLTISGNDLSVMSSIHY

LLKSSSIEELDFSRNNLMELNPKAFSHLSNVVYINLSQNSLKKLPEKAFEKVTLLEELDL

SYNSLTELPRDIFNGTTLSILHLKYNTFNGDLHFGTKDLQQLDLSFNSIVQVHHSMFDKM

PGLTNLNLKGNGIKKIQPDSFLTLKNLRHIDLSINDLDQISGMLFFKNSELDVIRLNDNP

RLSQLPTDGFLSYSGEFTVYYLDISNCAIGPLGHKAFSTMPHLTTLKLAWNNINHLPREI

FTGLHKLIDLDLSNNLITRMDDLIFMDNGELTKLSLAGNPISRLSVRLFLPLHQLRCLDV

NDCELTTLLSDRDLGAGYKIFDSLRSFNASGNLIKKISSEDVKSFKNLRSLDITNNPLKC

TPDFQEFISYVTLQMQMTPKRLPVLANLEDDATIVQLETLAQAGWSSLAHEVCKHAEGSD

LLDEKKADSAEAKLEKRLKESVKKLNEDEAKLLSVLDKSVLDKSRLSSMQKIMKGSVNTE

TAKPEEDADEENLNNGGEDKEDDSSDYDSEEDDDDDDEDDDDDNNDDDRFEEAKKNAKAK

ADANKAFDTFVNKELKPVLKTDGIQAEEVDMSQETRDKFLLEKLGFDSESEESDEYTEKM

IFHGELSYDLVVPLIVVSFCLLMIVLSIARVIYVVLRKRGERYRMALLASKNSFVYQKLS

EDIVKPTSKEQKEPKHLKQPKVHRYAPINQV

>ENSMUSP00000020400; Lrrtm1

MDFLLLGLCLHWLLRRPSGVVLCLLGACFQMLPAAPSGCPGQCRCEGRLLYCEALNLTEA

PHNLSGLLGLSLRYNSLSELRAGQFTGLMQLTWLYLDHNHICSVQGDAFQKLRRVKELTL

SSNQITELANTTFRPMPNLRSVDLSYNKLQALAPDLFHGLRKLTTLHMRANAIQFVPVRI

FQDCRSLKFLDIGYNQLKSLARNSFAGLFKLTELHLEHNDLIKVNFAHFPRLISLHSLCL

RRNKVAIVVSSLDWVWNLEKMDLSGNEIEYMEPHVFETVPYLQTLQLDSNRLTYIEPRIL

NSWKSLTSITLAGNLWDCGRNVCALASWLSNFQGRYDANLQCASPEYAQGEDVLDAVYAF

HLCEDGAEPTSGHLLSVAVTNRSDLTPPESSATTLVDGGEGHDGTFEPITVALPGGEHAE

NAVQIHKVVTGTMALIFSFLIVVLVLYVSWKCFPASLRQLRQCFVTQRRKQKQKQTMHQM

AAMSAQEYYVDYKPNHIEGALVIINEYGSCTCHQQPARECEV

>ENSP00000295057; LRRTM1

MDFLLLGLCLYWLLRRPSGVVLCLLGACFQMLPAAPSGCPQLCRCEGRLLYCEALNLTEA

PHNLSGLLGLSLRYNSLSELRAGQFTGLMQLTWLYLDHNHICSVQGDAFQKLRRVKELTL

SSNQITQLPNTTFRPMPNLRSVDLSYNKLQALAPDLFHGLRKLTTLHMRANAIQFVPVRI

FQDCRSLKFLDIGYNQLKSLARNSFAGLFKLTELHLEHNDLVKVNFAHFPRLISLHSLCL

RRNKVAIVVSSLDWVWNLEKMDLSGNEIEYMEPHVFETVPHLQSLQLDSNRLTYIEPRIL

NSWKSLTSITLAGNLWDCGRNVCALASWLNNFQGRYDGNLQCASPEYAQGEDVLDAVYAF

HLCEDGAEPTSGHLLSAVTNRSDLGPPASSATTLADGGEGQHDGTFEPATVALPGGEHAE

NAVQIHKVVTGTMALIFSFLIVVLVLYVSWKCFPASLRQLRQCFVTQRRKQKQKQTMHQM

AAMSAQEYYVDYKPNHIEGALVIINEYGSCTCHQQPARECEV

>AY182027; Lrrtm2

MGLHFKWPLGAPMLAAIYAMSVVLKMLPALGMACPPKCRCEKLL

FYCDSQGFHSVPNATDKGSLGLSLRHNHITALERDQFASFSQLTWLHLDHNQISTVKE

DAFQGLYKLKELILSSNKIFYLPNTTFTQLINLQNLDLSFNQLSSLHPELFYGLRKLQ

TLHLRSNSLRTIPVRLFWDCRSLEFLDLSTNRLRSLARNGFAGLIKLRELHLEHNQLT

KINFAHFLRLSSLHTLFLQWNKISNLTCGMDWTWSTLEKLDLTGNEIKAIDLTVFETM

PNLKILLMDNNKLNSLDSKILNSLKSLTTVGLSGNLWECSPRVCALASWLGSFQGRWE

HSILCHSPDHTQGEDILDAVHGFQLCWNLSTTVTAMATTYRDPTTEYTKISSSSYHVG

DKEIPTTAGIAVTTEEHFPEPDNAIFTQRVITGTMALLFSFFFIIFIVFISRKCCPPT

LRRIRQCSMIQNHRQLRSQTRLHMSNMSDQGPYNEYEPTHEGPFIIINGYGQCKCQQL

PYKECEV

>AY182026; LRRTM2

MGLHFKWPLGAPMLAAIYAMSMVLKMLPALGMACPPKCRCEKLLFYCDSQGFHSVPNATDK

GSLGLSLRHNHITELERDQFASFSQLTWLHLDHNQISTVKEDAFQGLYKLKELILSSNKI

FYLPNTTFTQLINLQNLDLSFNQLSSLHPELFYGLRKLQTLHLRSNSLRTIPVRLFWDCR

SLEFLDLSTNRLRSLARNGFAGLIKLRELHLEHNQLTKINFAHFLRLSSLHTLFLQWNKI

SNLTCGMEWTWGTLEKLDLTGNEIKAIDLTVFETMPNLKILLMDNNKLNSLDSKILNSLR

SLTTVGLSGNLWECSARICALASWLGSFQGRWEHSILCHSPDHTQGEDILDAVHGFQLCW

NLSTTVTVMATTYRDPTTEYTKRISSSSYHVGDKEIPTTAGIAVTTEEHFPEPDNAIFTQ

RVITGTMALLFSFFFIIFIVFISRKCCPPTLRRIRQCSMVQNHRQLRSQTRLHMSNMSDQ

GPYNEYEPTHEGPFIIINGYGQCKCQQLPYKECEV

>AY182029; Lrrtm3

MGFNVIRLLRGSAVAVVLAPTVLLTMLSSAERGCPKGCRCEGKMVYCESQKLQEIPSSISA

GCLGLSLRYNSLQKLKYNQFKGLNQLTWLYLDHNHISNIDENAFNGIRRLKELILSSNRI

SYFLNNTFRPVTNLRNLDLSYNQLHSLGSEQFRGLRKLLSLHLRSNSLRTIPVRIFQDCR

NLELLDLGYNRIRSLARNVFAGMIRLKELHLEHNQFSKLNLALFPRLVSLQNLYMQWNKI

SVIGQTMSWTWSSLQRLDLSGNEIEAFSGPSVFQCVPNLQRLNLDSNKLTFIGQEILDSW

ISLNDISLAGNIWECSRNICSLVNWLRSFKGLRENTIICASPKELQGVNVIDAVKNYSIC

GKSTTTERFDLARALPKPTFKPKLPRPKHESKPPLPPTVGATEPSPETDVDTEHISFHKI

IAGSVALFLSVLVILLVMYVSWKRYPASMKQLQQRSLMRRHRKKKRQSLKQMTPGTQEFY

VDYKPTNTETSEMLLNGTGPCTYSKSGSRECEIPLSMNVSTFLAYDQPTISYCGVHHELL

SHKSFETNAQEDTMESHLETELDLSTITSAGRISDHKPQLA

>AY182028; LRRTM3

MGFNVIRLLSGSAVALVIAPTVLLTMLSSAERGCPKGCRCEGKM

VYCESQKLQEIPSSISAGCLGLSLRYNSLQKLKYNQFKGLNQLTWLYLDHNHISNIDE

NAFNGIRRLKELILSSNRISYFLNNTFRPVTNLRNLDLSYNQLHSLGSEQFRGLRKLL

SLHLRSNSLRTIPVRIFQDCRNLELLDLGYNRIRSLARNVFAGMIRLKELHLEHNQFS

KLNLALFPRLVSLQNLYLQWNKISVIGQTMSWTWSSLQRLDLSGNEIEAFSGPSVFQC

VPNLQRLNLDSNKLTFIGQEILDSWISLNDISLAGNIWECSRNICSLVNWLKSFKGLR

ENTIICASPKELQGVNVIDAVKNYSICGKSTTERFDLARALPKPTFKPKLPRPKHESK

PPLPPTVGATEPGPETDADAEHISFHKIIAGSVALFLSVLVILLVIYVSWKRYPASMK

QLQQRSLMRRHRKKKRQSLKQMTPSTQEFYVDYKPTNTETSEMLLNGTGPCTYNKSGS

RECEV

>AY182031; Lrrtm4

MGFRLITQLKGMSVFLVLFPTLLLVMLTGAQRACPKNCRCDGKI

VYCESHAFADIPENISGGSQGLSLRFNSIQKLKSNQFAGLNQLIWLYLDHNYISSVDE

DAFQGIRRLKELILSSNKITYLHNKTFHPVPNLRNLDLSYNKLQTLQSEQFKGLRKLI

ILHLRSNSLKTVPIRVFQDCRNLDFLDLGYNRLRSLSRNAFAGLLKLKELHLEHNQFS

KINFAHFPRLFNLRSIYLQWNRIRSVSQGLTWTWSSLHTLDLSGNDIQAIEPGTFKCL

PNLQKLNLDSNKLTNVSQETVNAWISLISITLSGNMWECSRSICPLFYWLKNFKGNKE

STMICAGPKHIQGEKVSDAVETYNICSDVQVVNTERSHLAPQTPQKPPFIPKPTIFKP

DAVPATLEAVSPSPGFQIPGTDHEYEHVSFHKIIAGSVALFLSVAMILLVIYVSWKRY

PASMKQLQQHSLMKRRRKKARESERQMNSPLQEYYVDYKPTNSETMDISVNGSGPCTY

TISGSRECEV

>AY182030; LRRTM4

MGFHLITQLKGMSVVLVLLPTLLLVMLTGAQRACPKNCRCDGKI

VYCESHAFADIPENISGGSQGLSLRFNSIQKLKSNQFAGLNQLIWLYLDHNYISSVDE

DAFQGIRRLKELILSSNKITYLHNKTFHPVPNLRNLDLSYNKLQTLQSEQFKGLRKLI

ILHLRSNSLKTVPIRVFQDCRNLDFLDLGYNRLRSLSRNAFAGLLKLKELHLEHNQFS

KINFAHFPRLFNLRSIYLQWNRIRSISQGLTWTWSSLHNLDLSGNDIQGIEPGTFKCL

PNLQKLNLDSNKLTNISQETVNAWISLISITLSGNMWECSRSICPLFYWLKNFKGNKE

STMICAGPKHIQGEKVSDAVETYNICSEVQVVNTERSHLVPQTPQKPLIIPRPTIFKP

DVTQSTFETPSPSPGFQIPGAEQEYEHVSFHKIIAGSVALFLSVAMILLVIYVSWKRY

PASMKQLQQHSLMKRRRKKARESERQMNSPLQEYYVDYKPTNSETMDISVNGSGPCTY

TISGSRECEV

>NM_001013384; Podnl1

MRPQELLLLLLMLKWSLAHTEDPAFPHLGDSSQPLPRPCPWRCS

CPRDDTVDCAGLDLRIFPDNITRAARHLSLQNNQLRELPYNELSRLSGLRTLDLHSNL

ITSEGLPDEAFESLNQLENFYVAHNKLSVAPQFLPRSLRVADLAANEVVEIFPLTFGE

KPALRSVYLHNNRLRNTGLPPNTFHGSEAITTLSLSSNQLSYLPPSLPASLERLHLQN

NLISKVPRGALSLQTHLRELYLQHNQLTDSGLDATTFSKLSSLEYLDLSHNQLATVPE

GLPGTLTILHLGRNCIRHVEAVRLHKARGLRYLLLQHNKLGASALPKGTLRPLRALHT

LHLYGNKLERVPPALPRHLQALVMPHNHVAALGARDLVSARALAELNLAYNSLASARV

HPSAFRRLRALRSLDLAGNQLTRLPEGLPASLRSLRLQRNQLRTLEPEQLAGLNKLRE

LNLTHNRLRVGDIGPGTWHELQALKVLDLSHNELSFVPPDLPEALEELHLQANRISHV

GPEAFLSTPHLRALFLRANRLHMTSIAAEALQGLTHLRVVDTAENPEQVLVQLPRKGK

EP

>ENSP00000254320; PODNL1

MAESGLAMWPSLLLLLLLPGPPPVAGLEDAAFPHLGESLQPLPRACPLRCSCPRVDTVDC

DGLDLRVFPDNITRAAQHLSLQNNQLQELPYNELSRLSGLRTLNLHNNLISSEGLPDEAF

ESLTQLQHLCVAHNKLSVAPQFLPRSLRVADLAANQVMEIFPLTFGEKPALRSVYLHNNQ

LSNAGLPPDAFRGSEAIATLSLSNNQLSYLPPSLPPSLERLHLQNNLISKVPRGALSRQT

QLRELYLQHNQLTDSGLDATTFSKLHSLEYLDLSHNQLTTVPAGLPRTLAILHLGRNRIR

QVEAARLHGARGLRYLLLQHNQLGSSGLPAGALRPLRGLHTLHLYGNGLDRVPPALPRRL

RALVLPHNHVAALGARDLVATPGLTELNLAYNRLASARVHHRAFRRLRALRSLDLAGNQL

TRLPMGLPTGLRTLQLQRNQLRMLEPEPLAGLDQLRELSLAHNRLRVGDIGPGTWHELQA

LQVRHRLVSHTVPRAPPSPCLPCHVPNILVSW

>ENSMUSP00000048962; Podn

MAGSRGLPLLLLVLQLFLGPVLPVRAPVFGRSDTPTLSPEENEFVEEENQPVLVLSSEEP

EPGPATVDCPRDCACSQEGVVDCGGIDLREFPGDLPEHTNHLSLQNNQLEKIYPEELSRL

QRLETLNLQNNRLTSRGLPEEAFEHLTSLNYLYLANNKLTLAPRFLPNALISVDFAANYL

TKIYGLTFGQKPNLRSVYLHNNKLADAGLPDHMFNGSSNVEILILSSNFLRHVPKHLPPA

LYKLHLKNNKLEKIPPGAFSELSNLRELYLQNNYLTDEGLDNETFWKLSSLEYLDLSSNN

LSRVPAGLPRSLVLLHLEKNAIQSVEADVLTPIRNLEYLLLHSNQLQAKGIHPLAFQGLK

KLHTVHLYNNALERVPSGLPRRVRTLMILHNQITGIGREDFATTYFLEELNLSYNRITSP

QMHRDAFRKLRLLRSLDLSGNRLQTLPPGLPKNVHVLKVKRNELAALARGALAGMAQLRE

LYLTGNRLRSRALGPRAWVDLAGLQLLDIAGNQLTEVPEGLPPSLEYLYLQNNKISAVPA

NAFDSTPNLKGIFLRFNKLAVGSVVESAFRRLKHLQVLDIEGNFEFGNGSKDKDEEEEEE

EEEEDEEEETR

>ENSP00000308315; PODN curated

MAQSRVLLLLLL

LPPQLHLGPVLAVRAPGFGRSGGHSLSPEENEFAEEEPVLVLSPEEPGPGPAAVSCPRDC

ACSQEGVVDCGGIDLREFPGDLPEHTNHLSLQNNQLEKIYPEELSRLHRLETLNLQNNRL

TSRGLPEKAFEHLTNLNYLYLANNKLTLAPRFLPNALISVDFAANYLTKIYGLTFGQKPN

LRSVYLHNNKLADAGLPDNMFNGSSNVEVLILSSNFLRHVPKHLPPALYKLHLKNNKLEK

IPPGAFSELSSLRELYLQNNYLTDEGLDNETFWKLSSLEYLDLSSNNLSRVPAGLPRSLV

LLHLEKNAIRSVDANVLTPIRSLEYLLLHSNQLREQGIHPLAFQGLKRLHTVHLYNNALE

RVPSGLPRRVRTLMILHNQITGIGREDFATTYFLEELNLSYNRITSPQVHRDAFRKLRLL

RSLDLSGNRLHTLPPGLPRNVHVLKVKRNELAALARGALVGMAQLRELYLTSNRLRSRAL

GPRAWVDLAHLQLLDIAGNQLTEIPEGLPESLEYLYLQNNKISAVPANAFDSTPNLKGIF

LRFNKLAVGSVVDSAFRRLKHLQVLDIEGNLEFGDISKDRGRLGKEKEEEEEEEEEEEET

R

>ENSMUSP00000057563; Gp1ba

MALLILLFLLPSPLHSQHTCSISKVTSLLEVNCENKKLTALPADLPADTGILHLGENQLG

TFSTASLVHFTHLTYLYLDRCELTSLQTNGKLIKLENLDLSHNNLKSLPSLGWALPALTT

LDVSFNKLGSLSPGVLDGLSQLQELYLQNNDLKSLPPGLLLPTTKLKKLNLANNKLRELP

SGLLDGLEDLDTLYLQRNWLRTIPKGFFGTLLLPFVFLHANSWYCDCEILYFRHWLQENA

NNVYLWKQGVDVKDTTPNVASVRCANLDNAPVYSYPGKGCPTSSGDTDYDDYDDIPDVPA

TRTEVKFSTNTKVHTTHWSLLAAAPSTSQDSQMISLPPTHKPTKKQSTFIHTQSPGFTTL

PETMESNPTFYSLKLNTVLIPSPTTLEPTSTQATPEPNIQPMLTTSTLTTPEHSTTPVPT

TTILTTPEHSTIPVPTTAILTTPKPSTIPVPTTATLTTLEPSTTPVPTTATLTTPEPSTT

LVPTTATLTTPEHSTTPVPTTATLTTPEHSTTPVPTTATLTTPEPSTTLTNLVSTISPVL

TTTLTTPESTPIETILEQFFTTELTLLPTLESTTTIIPEQNSFLNLPEVALVSSDTSESS

PFLNSDFCCFLPLGFYVLGLLWLLFASVVLILLLTWTWHVTPHSLDMEQSAALATSTHTT

SLEVQRARQVTMPRAWLLFLQGSLPTFRSSLFLWVRPNGRVGPLVAGRRPSALSQGRGQD

LLGTVGIRYSGHSL

>ENSP00000329380; GP1BA RELEASE 40

MPLLLLLLLLPSPLHPHPICEVSKVASHLEVNCDKRNLTALPPDLPKDTTILHLSENLLY

TFSLATLMPYTRLTQLNLDRCELTKLQVDGTLPVLGTLDLSHNQLQSLPLLGQTLPALTV

LDVSFNRLTSLPLGALRGLGELQELYLKGNELKTLPPGLLTPTPKLEKLSLANNNLTELP

AGLLNGLENLDTLLLQENSLYTIPKGFFGSHLLPFAFLHGNPWLCNCEILYFRRWLQDNA

ENVYVWKQGVDVKAMTSNVASVQCDNSDKFPVYKYPGKGCPTLGDEGDTDLYDYYPEEDT

EGDKVRATRTVVKFPTKAHTTPWGLFYSWSTASLDSQMPSSLHPTQESTKEQTTFPPRWT

PNFTLHMESITFSKTPKSTTEPTPSPTTSEPPTPSPTTPEPTSEPAPSPTTPEPTSEPAP

SPTTPEPTPIPTIATSPTILVSATSLITPKSTFLTTTKPVSLLESTKKTIPELDQPPKLR

GVLQGHLESSRNDPFLHPDFCCLLPLGFYVLGLFWLLFASVVLILLLSWVGHVKPQALDS

GQGAALTTATQTTHLELQRGRQVTVPRAWLLFLRGSLPTFRSSLFLWVRPNGRVGPLVAG

RRPSALSQGRGQDLLSTVSIRYSGHSL

>ENSMUSP00000057071; Lrrc54 RELEASE 40

MLCSLFLLLLAVGRVQTTRPCFPGCQCEEETFGLFDSFSLIRVDCSSLGPHIVPVPIPLD

TAHLDLSSNRLETVNESVLAGPGYTTLAGLDLSYNLLTSIMPSAFSRLRYLESLDLSHNG

LAALPAEIFTSSPLSDINLSHNRLREVSISAFTTHSQGRALHVDLSHNLIHRLLPHPARA

SLPAPTIQSLNLSWNRFRAVPDLRDLPLRYLSLDGNPLATINPDAFMGLAGLTHLSLASL

QGILHLPPHGFRELPGLQVLDLSGNPKLKWAGAEVFSGLGLLQELDLSGSSLVPLPEMLL

HHLPALQSVSVGQDVQCRRLVREGAYHRQPGSSPKVVLHCGDTQESAARGPDIL

>ENSP00000332668; LRRC54

MPWPLLLLLAVSGAQTTRPCFPGCQCEVETFGLFDSFSLTRVDCSGLGPHIMPVPIPLDT

AHLDLSSNRLEMVNESVLAGPGYTTLAGLDLSHNLLTSISPTAFSRLRYLESLDLSHNGL

TALPAESFTSSPLSDVNLSHNQLREVSVSAFTTHSQGRALHVDLSHNLIHRLVPHPTRAG

LPAPTIQSLNLAWNRLHAVPNLRDLPLRYLSLDGNPLAVIGPGAFAGLGGLTHLSLASLQ

RLPELAPSGFRELPGLQVLDLSGNPKLNWAGAEVFSGLSSLQELDLSGTNLVPLPEALLL

HLPALQSVSVGQDVRCRRLVREGTYPRRPGSSPKVALHCVDTRDSAARGPTIL

>ENSMUSP00000065874; Lrrc9

MIESENLNRGEIIKELCLCNGLTYEIVGQEGSDTSKLEMFFSGYPRIVGLSLFHNLSSLT

IVAQDIREISGLETCLQLKELWIAECCIEKIEGLQGCRNLEKLYLYYNKISKIENLEKLI

KLEVLWLNHNMIKNIEGLQTLKNLKDLNLAGNLVSSIGRCLDPNEQLEKLNLSGNQITSF

KDLTNLTKLTRLKDLCLNDPQYKSNPVCQLCNYSTHVLYHLPSLQRLDTFDVSAKQIKEL

ADSTAMKKIMYYNMRIKTVQRHLNEELEKLNDRKCKLQKLPEERIKLFNFAKKTLERELA

ELKISSKGQSDTTPEAEKPRNSEVVTQESVLQQKILTKLSALDDRVTFWNKKLHEIEAIY

RTEVKQKKKTHGLLTPFLLTELETVGNIHFEEGTQADDWFNSCCELILSRFCTWDFRAYG

ITGVKVKRVIKVNNRILRLKFEEKFQKCLDLEDTQDPDYRKMLECLFYVFDPEVTVKKKH

LLQILERGFKDSDTSKPSLKKEAVTLVNSLSMCECPRIEFLQQKYKEEKKGPSESELYRH

GTILIAKVFLGQSIQARDQEPINKANYPMVNSVFVPQRHVLRQRTCDCGYRQYKWFVFDH

DLVLPEYIVEFEYTTVVKVHSLFSTSNNVILEEGKKYSEGLVFSQDLKFDDEVLKMEPRI

KPRPKLISLDEKTIISLAKTNIYSHIVNLNLHGNSLSKLRDLAKLTGLRKLNISFNEFTC

LDDVYHLYNLEYLDASHNHVITLEGFRGLMKLKHLDLSWNQLKKTGEEINVLCKHTTSLL

TLDIQHNPWQKPATLRLSVIGRLKTLTHLDGLVISEEETRAALKFISGTKITQLTLLQHS

SSKEERPRMLSTWPSAKILTQISKLGPHFHLTGNWYSKITALNLDGQHLFEITNLEKLEN

LKWASFSNNNLSKMEGLESCVNLEELTLDGNCISKIEGITRLTKLSRLSMNNNLLTGLEK

HTFDNLLHSLPLSGEQQDHISERVTEDLHSDRVVHKQQLHSCEPGDLQLKGFMQLGHSRH

VWKHYYMEPRKLPVLCNISSSRTESLGWSLN

>ENSP00000254271; NP_940901.1 curated

MFFLGYPRIVGLSLFPNLTSLT

IVAQDIKEISGLEPCLQLKELWIAECCIEKIEGLQECRNLEKLYLYFNKISKIENLEKLI

KLKVLWLNHNTIKNIEGLQTLKNLKDLNLAGNLINSIGRCLDSNEQLERLNLSGNQICSF

KELTNLTRLPCLKDLCLNDPQYTTNPVCLLCNYSTHVLYHLPCLQRFDTLDVSAKQIKEL

ADTTAMKKIMYYNMRIKTLQRHLKEDLEKLNDQKCKLQKLPEERVKLFSFVKKTLERELA

ELKGSGKGHSDGSNNSKVTDPETLKSCETVTEEPSLQQKILAKLNALNERVTFWNKKLDE

IEAIYHIEVKQKKKSHGLLIPLLLIELETVGNFHFEEGTRSDDWFNFCYELILSRFCAWD

FRTYGITGVKVKRIIKVNNRILRLKFEEKFQKFLENEDMHDSESYRRMLECLFYVFDPEV

SVKKKHLLQILEKGFKDSETSKLPLKKEAIIVSNSLSISECPRIEFLQQKHKDEKKISLK

HELFRHGILLITKVFLGQSVQAHEKESISQSNYPMVNSVFIPRKYLLNSVMGQRNCDCSV

RQCKWFVFDHDLVLPEYVVEFEYITMVKAPSLFSVFNNVILEESKKNPEVSVFSKDLKFD

DEVIKMEPRIKARPKLISLDDKTILSLAKTSVYSHIVSLNLHGNSLSKLRDLSKLTGLRK

LNISFNEFTCLDDVYHLYNLEYLDASHNHVITLEGFRGLMKLKHLDLSWNQLKKSGNEIN

MLCKHTTSLLTLDIQHNPWQKPATLRLSVIGRLKTLTHLNGVFISEEEATAAMKFIAGTR

ITQLSLLRHSSTKEERPRILSIWPSAKILTQVSKLGPHLHLSGNCYLKITALNLDGQHLF

EITNLEKLENLKWASFSNNNLTKMEGLESCINLEELTLDGNCISKIEGISKMTKLTRLSI

NNNLLTGWEEHTFDNMLHLHSLSLENNRITSLSGLQKSFTLVELYISNNYIAVNQEMHNL

KGLCNLVILDMCGNIIIWNQENYRLFVIFHLPELKALDGIPIEPSETDSAKDLFGGRLTS

DMIAERQGHSNFKQMQELNWTSSSISCIYFV

>ENSMUSP00000032133; Gp9

MTTWGLLFLLWPATTDTQACPRPCTCQSLETMGLKVNCEGQGLTALPVIPAHTRQLLLAN

NSLRSVPPGAFDHLPQLWDLDVTHNPWHCDCSLTYLRLWLEDHMPEALMHVYCASPDLAT

RRPLGQLTGYELGSCGWKLPPSWAYPGVWWDVSLVAVAVLGLILLAGLLNTFTESRN

>ENSP00000303942; GP9

MPAWGALFLLWATAEATKDCPSPCTCRALETMGLWVDCRGHGLTALPALPARTRHLLLAN

NSLQSVPPGAFDHLPQLQTLDVTQNPWHCDCSLTYLRLWLEDRTPEALLQVRCASPSLAA

HGPLGRLTGYQLGSCGWQLQASWVRPGVLWDVALVAVAALGLALLAGLLCATTEALD

>ENSMUSP00000038048; Lrg1

MVSWQHQGSLQDLKTCLARTLFLLALLGRVSSLKECLILQSAEGSTVSCHGPTEFPSSLP

ADTVHLSVEFSNLTQLPAAALQGCPGLRELHLSSNRLQALSPELLAPVPRLRALDLTRNA

LRSLPPGLFSTSANLSTLVLRENQLREVSAQWLQGLDALGHLDLAENQLSSLPSGLLASL

GALHTLDLGYNLLESLPEGLLRGPRRLQRLHLEGNRLQRLEDSLLAPQPFLRVLFLNDNQ

LVGVATGSFQGLQHLDMLDLSNNSLSSTPPGLWAFLGRPTRDMQDGFDISHNPWICDKNL

ADLCRWLVANRNKMFSQNDTRCAGPEAMKGQRLLDVAELGSL

>ENSP00000302621; LRG1

MSSWSRQRPKSPGGIQPHVSRTLFLLLLLAASAWGVTLSPKDCQVFRSDHGSSISCQPPA

EIPGYLPADTVHLAVEFFNLTHLPANLLQGASKLQELHLSSNGLESLSPEFLRPVPQLRV

LDLTRNALTGLPPGLFQASATLDTLVLKENQLEVLEVSWLHGLKALGHLDLSGNRLRKLP

PGLLANFTLLRTLDLGENQLETLPPDLLRGPLQLERLHLEGNKLQVLGKDLLLPQPDLRY

LFLNGNKLARVAAGAFQGLRQLDMLDLSNNSLASVPEGLWASLGQPNWDMRDGFDISGNP

WICDQNLSDLYRWLQAQKDKMFSQNDTRCAGPEAVKGQTLLAVAKSQ

>ENSMUSP00000056094; Lrrc19

MKVTRFMFWLFSMLLPSVKSQASETEVPCNFSRRNYTLIPEGISTNVTILDLSYNRITLN

AADSRVLQMYSLLTELYLMENNIIALYNSSFRNLLNLEILNICGNSISVIQQGSFVGLNE

LKQLFLCQNKILQLNPDTFVPLNNLKVLNLQGNLIRLFDAPQLPHLEILTLDGNPWNCTC

GLLELHNWLNTSNVTLENENMTMCSYPDELKHDSIKSAPFTTECHSTFISTITEDFQSTR

NSSFNSSSHNLTWTSEHEPLGKSWAFLVGVVATVLLTSLLIFIAIKCPVWYNILLSYNHH

RLEEHEAETYENGLTRNPSSLSQITDTNSEDTTVIFEQLHAFVVDDDGFIEDRYIDINEV

HEEK

>ENSP00000330698; LRRC19

MKVTGITILFWPLSMILLSDKIQSSKREVQCNFTEKNYTLIPADIKKDVTILDLSYNQIT

LNGTDTRVLQTYFLLTELYLIENKVTILHNNGFGNLSSLEILNICRNSIYVIQQGAFLGL

NKLKQLYLCQNKIEQLNADVFVPLRSLKLLNLQGNLISYLDVPPLFHLELITLYGNLWNC

SCSLFNLQNWLNTSNVTLENENITMCSYPNSLQSYNIKTVPHKAECHSKFPSSVTEDLYI

HFQPISNSIFNSSSNNLTRNSEHEPLGKSWAFLVGVVVTVLTTSLLIFIAIKCPIWYNIL

LSYNHHRLEEHEAETYEDGFTGNPSSLSQIPETNSEETTVIFEQLHSFVVDDDGFIEDKY

IDIHELCEEN

>ENSMUSP00000049686; Lrrc25

MGSIRTRLLWLCLLMLLALLHKSGSQDLTCMVHPSRVDWTQTFNGTCLNFSGLGLSLPRS

PLQASHAQVLDLSKNGLQVLPGAFFDKLEKLQTLIVTHNQLDSVDRSLALRCDLELKADC

SCGLASWYALRQNCSGQQQLLCLHPATEAPRNLSTFLQVSCPPSWGPGTIGALVAGTISL

AVAVSGSVLAWRLLRRRRRASEHSLSKAQMSPHDIPKPVTDFLPRYSSRRPGPKAPDSPP

SRFTMDYENVFIGQPAEDCSWSAARNSPSGDSDCYMNYRSVDQDSQPVYCNLESLGR

>ENSP00000340983; LRRC25

MGGTLAWTLLLPLLLRESDSLEPSCTVSSADVDWNAEFSATCLNFSGLSLSLPHNQSLRA

SNVILLDLSGNGLRELPVTFFAHLQKLEVLNVLRNPLSRVDGALAARCDLDLQADCNCAL

ESWHDIRRDNCSGQKPLLCWDTTSSQHNLSAFLEVSCAPGLASATIGAVVVSGCLLLGLA

IAGPVLAWRLWRCRVARSRELNKPWAAQDGPKPGLGLQPRYGSRSAPKPQVAVPSCPSTP

DYENMFVGQPAAEHQWDEQGAHPSEDNDFYINYKDIDLASQPVYCNLQSLGQAPMDEEEY

VIPGH

>ENSMUSP00000038569; Lrrc17

MRIVAILLLFCLCRAAEPRKSSPGVLRSQGNPSRSHGRGGRRGSSPVKRYAPGLPCDVYT

YLHEKYLDCQERKLVYVLPDWPQDLLHMLLARNKIRVLKNNMFAKFKRLKSLDLQQNEIS

KIESEAFFGLNKLTTLLLQHNQIKVLTEEAFIYTPLLSYLRLYDNPWHCTCELETLISML

QIPRNRNLGNYAKCGSPPALRNKKLLQLKPQELCDEEEKEQLDPKPQVSGIPAVIRPEAD

STLCHNYVFPIQTLDCKRKELKKVPSNIPPDIVKLDLSSNKIRQLRPKEFEDVHELKKLN

LSSNGIEFIDPAAFLGLIHLEELDLSNNSLQNFDYGVLEDLYFLKLLWLRDNPWRCDYSI

HYLYYWLKHHYNVHYNGLECKTPEEYKGWSVGKYVRSYYEECPKDKLPAYPETFDQDTED

DEWQKIHRDHPAKKHRVRITIVG

>ENSP00000344242; LRRC17

MRVVTIVILLCFCKAAELRKASPGSVRSRVNHGRAGGGRRGSNPVKRYAPGLPCDVYTYL

HEKYLDCQERKLVYVLPGWPQDLLHMLLARNKIRTLKNNMFSKFKKLKSLDLQQNEISKI

ESEAFFGLNKLTTLLLQHNQIKVLTEEVFIYTPLLSYLRLYDNPWHCTCEIETLISMLQI

PRNRNLGNYAKCESPQEQKNKKLRQIKSEQLCNEEEKEQLDPKPQVSGRPPVIKPEVDST

FCHNYVFPIQTLDCKRKELKKVPNNIPPDIVKLDLSYNKINQLRPKEFEDVHELKKLNLS

SNGIEFIDPAAFLGLTHLEELDLSNNSLQNFDYGVLEDLYFLKLLWLRDNPWRCDYNIHY

LYYWLKHHYNVHFNGLECKTPEEYKGWSVGKYIRSYYEECPKDKLPAYPESFDQDTEDDE

WEKKHRDHTAKKQSVIITIVG

>ENSMUSP00000084423; BC031901

MRDFYVRVTILVTGLCFVETVTTPSRKSSVSFNPEYQRNGDLLVNWSSIRHVSQNTDAMD

RSFYFFRVLFQPHTQKERHIKPPDRTHHRISKVTLDPLAHLHALEILNLSNKAIHYFSLD

QPLPPSSHQKRHGGHSHSRLPRLQVLILQRNQLSGTPKGLWKLKSLRSLDLSFNRIVHIG

LSDFHGCLQLESIYLKSNKICTIHPKAFKGLKKLQVVDLRSNALTTLVPIVTIALELPHL

ELGLADNQWQCSESNVNFQNITSSSWREIWKAICNMSVENKRPNAETHQIRKSRDTHLLL

SPPSDLKSLIQSKAERPQAGMDMHLSALGKEAKDGYGDLRGMWPQSPVELRDSQDEQVTD

RKDDKPPALELAICLSVFITFVVAFCLGAFARPYIDRLRQQRCSNKRPGSDNAYSNKGFH

GDIEGAQHMEYQGTDLHQTTHHLHLSENQNPSWVAEPIPHSAVQSEQMLGSNGTDPGHQQ

SPEQLKDSNESRSGDSIVLPSGPVAHLALHGLPNADAHKAISPVQHHHDFLEEAHYDTVA

QEYSLIDDVMDRSSITGPLGTFPSSVESRRDDLHPSQPRDVVASFSKTLAHANTREAEGS

METGCPEPLGAMDSQMGSSEERQVSNSIRELATQQPSFQGVDAEERLSHVYSEVLHNDPP

SLRPRWGSGHYVIPATGEPVERDAPFDPHYDLVTNYESDSDEGSLFTLSSEGSEDTRSLA

EEQASVENDGTSQPLPSRNLGEYKDSVTSAESVEDLTSQRIPEKCEAQEAHLRNTLISGP

DSCVCETNQENDSSSLDPENRSTWPQLPGHKLSHHETLGTYGDIEPQSEAVDWHYSLRDL

ESPNVDSSPSPPYSDEDLSGPEDRARKRSKHW

>ENSP00000341944; NP_001019782.1

MKNLYFRVITIVIGLYFTGIMTNASRKSNILFNSECQWNEYILTNCSFTGKCDIPVDISQ

TAATVDVSFNFFRVLLQSHTKKEEWKIKHLDLSNNLISKITLSPFAYLHALEVLNLSNNA

IHSLSLDLLSPKSSWVKRHRSSFRNRFPLLKVLILQRNKLSDTPKGLWKLKSLQSLDLSF

NGILQIGWSDFHNCLQLENLCLKSNKIFKIPPQAFKDLKKLQVIDLSNNALITILPMMII

ALEFPHLVVDLADNNWQCDDSVAVFQNFISESWRKKWNVICNRSIGNEEANGGTPQSRIS

RETRLPPIHLHRMKSLIRSKAERPQGGRHTGISTLGKKAKAGSGLRKKQRRLPRSVRSTR

DVQAAGKKEDAPQDLALAVCLSVFITFLVAFSLGAFTRPYVDRLWQKKCQSKSPGLDNAY

SNEGFYDDMEAAGHTPHPETHLRQVFPHLSLYENQTPFWVTQPHPHATVIPDRTLGRSRK

DPGSSQSPGQCGDNTGAGSGNDGAVYSILQRHPHAGNRELMSAAQDHIHRNDILGEWTYE

TVAQEEPLSAHSVGVSSVAGTSHAVSGSSRYDSNELDPSLSGEITASLCKMLTHAEAQRT

GDSKERGGTEQSLWDSQMEFSKERQVSSSIDLLSIQQPRLSGARAEEALSAHYSEVPYGD

PRDTGPSVFPPRWDSGLDVTPANKEPVQKSTPSDTCCELESDCDSDEGSLFTLSSISSES

ARSKTEEAVPDEESLQDESSGASKDNVTAVDSLEENVTFQTIPGKCKNQEDPFEKPLISA

PDSGMYKTHLENASDTDRSEGLSPWPRSPGNSPLGDEFPGMFTYDYDTALQSKAAEWHCS

LRDLEFSNVDVLQQTPPCSAEVPSDPDKAAFHERDSDILK

>ENSMUSP00000054140; Omg

MEYQILKMSSCLFILLFLTPGILCICPLQCTCTERHRHVDCSGRNLTTLPPGLQENIIHL

NLSYNHFTDLHNQLTPYTNLRTLDISNNRLESLPAQLPRSLWNMSAANNNIKLLDKSDTA

YQWNLKYLDVSKNMLEKVVLIKNTLRSLEVLNLSSNKLWTVPTNMPSKLHIVDLSNNSLT

QILPGTLINLTNLTHLYLHNNKFTFIPEQSFDQLLQLQEITLHNNRWSCDHKQNITYLLK

WVMETKAHVIGTPCSKQVSSLKEQSMYPTPPGFTSSLFTMSEMQTVDTINSLSMVTQPKV

TKTPKQYRGKETTFGVTLSKDTTFSSTDRAVVAYPEDTPTEMTNSHEAAAATLTIHLQDG

MSSNASLTSATKSPPSPVTLSIARGMPNNFSEMPRQSTTLNLRREETTANGNTRPPSAAS

AWKVNASLLLMLNAVVMLAG

>ENSP00000247271; OMG

MEYQILKMSLCLFILLFLTPGILCICPLQCICTERHRHVDCSGRNLSTLPSGLQENIIHL

NLSYNHFTDLHNQLTQYTNLRTLDISNNRLESLPAHLPRSLWNMSAANNNIKLLDKSDTA

YQWNLKYLDVSKNMLEKVVLIKNTLRSLEVLNLSSNKLWTVPTNMPSKLHIVDLSNNSLT

QILPGTLINLTNLTHLYLHNNKFTFIPDQSFDQLFQLQEITLYNNRWSCDHKQNITYLLK

WMMETKAHVIGTPCSTQISSLKEHNMYPTPSGFTSSLFTVSGMQTVDTINSLSVVTQPKV

TKIPKQYRTKETTFGATLSKDTTFTSTDKAFVPYPEDTSTETINSHEAAAATLTIHLQDG

MVTNTSLTSSTKSSPTPMTLSITSGMPNNFSEMPQQSTTLNLWREETTTNVKTPLPSVAN

AWKVNASFLLLLNVVVMLAV

>ENSMUSP00000056669; Cd14

MERVLGLLLLLLVHASPAPPEPCELDEESCSCNFSDPKPDWSSAFNCLGAADVELYGGGR

SLEYLLKRVDTEADLGQFTDIIKSLSLKRLTVRAARIPSRILFGALRVLGISGLQELTLE

NLEVTGTAPPPLLEATGPDLNILNLRNVSWATRDAWLAELQQWLKPGLKVLSIAQAHSLN

FSCEQVRVFPALSTLDLSDNPELGERGLISALCPLKFPTLQVLALRNAGMETPSGVCSAL

AAARVQLQGLDLSHNSLRDAAGAPSCDWPSQLNSLNLSFTGLKQVPKGLPAKLSVLDLSY

NRLDRNPSPDELPQVGNLSLKGNPFLDSESHSEKFNSGVVTAGAPSSQAVALSGTLALLL

GDRLFV

> ENSP00000304236; CD14

MERASCLLLLLLPLVHVSATTPEPCELDDEDFRCVCNFSEPQPDWSEAFQCVSAVEVEIH

AGGLNLEPFLKRVDADADPRQYADTVKALRVRRLTVGAAQVPAQLLVGALRVLAYSRLKE

LTLEDLKITGTMPPLPLEATGLALSSLRLRNVSWATGRSWLAELQQWLKPGLKVLSIAQA

HSPAFSCEQVRAFPALTSLDLSDNPGLGERGLMAALCPHKFPAIQNLALRNTGMETPTGV

CAALAAAGVQPHSLDLSHNSLRATVNPSAPRCMWSSALNSLNLSFAGLEQVPKGLPAKLR

VLDLSCNRLNRAPQPDELPEVDNLTLDGNPFLVPGTALPHEGSMNSGVVPACARSTLSVG

VSGTLVLLQGARGFA

> ENSMUSP00000070130; Nepn

MHPLWAFLLGLSLTNGLSANCPGRCSCDSMQSVQCYRLMELPSGIPSTTKRLYISHSRIQ

HLQLSNFTGLLALEDFILLASGTESIENDTFKTLSTLKTLELWKNKLRQVPSALPANLEV

LKLNDNAICALRGSEFEGLKNLKVLELKNNLISSLSPSMLSPLASLQSLMVDGNNIESVV

GPLSLPHLKYMSMENNQLHLIPGNVFTSLQNLQFLSFSGNFLTKIPINLPKSLLSLKMER

NQLKVVRFRDMKHLENLSHLYLSENFLSSIDGAQQLTNLTTLEVSQNQLQMLPPRLPSRL

QKLDCSSNFIQRVTAPEFQDLRDLKHLFLDNNVVSLFEAGALQRCSQLSNLALEQNLLLS

IPLRLPKTLARLDLKGNAIQDMAERELRDLKQLQVLNLRNNRISALDFKALEGLPRLRHL

YLDGNPWNCTCSLLRAREVLKAKGTDVKGGQCAAPAERQGESWMSSKKILRQCEHHLQQS

EKSKETKKKPKPEDSSSIRLNMDDDDDDYEID

>GENSCAN00000039828; translated only partial sequence

MIKWLMTAKT HKHVESVRDL QILPFRLPVK LQKLDCSNNL IQRVTAQDFQ DLQDLKHLIL

DNNNASFFEA GALQRCSQLS NLALEQNLLL SIPLRLPGTL TRLDLKSNVI QNIAEREIKD

LKQLHVLNLR NNKISALDLK ALEGLPHLRH LYLDGNPWNC TFSLLKAREV LMAKGTDVRG

GQCAAPTEQH GESWMSSKEI MRQCKHHFHL TEKSKETKKK SKPEDPSSIR INMDDG

>ENSMUSP00000059270; Gp1bb

MLPPHPSASLSGPRGALSLLLLLLALLSRPASGCPAPCSCAGTLVDCGRRGLTWASLPAA

FPPDTTELVLTGNNLTALPPGLLDALPALRAAHLGANPWRCDCRLLPLRAWLAGRPERAP

YRDLRCVAPPALRGRLLPYVAEDELRAACAPGLLCWGALVAQLALLVLGLLHALLLALLL

GRLRRLRARARARSIQEFSLTAPLVAESARGGAS

>OTTHUMP00000028578; NM_000407 Gp1BB

MGSGPRGALSLLLLLLAPPSRPAAGCPAPCSCAGTLVDCGRRGL

TWASLPTAFPVDTTELVLTGNNLTALPPGLLDALPALRTAHLGANPWRCDCRLVPLRA

WLAGRPERAPYRDLRCVAPPALRGRLLPYLAEDELRAACAPGPLCWGALAAQLALLGL

GLLHALLLVLLLCRLRRLRARARARAAARLSLTDPLVAERAGTDES

>ENSMUSP00000097897; Slitrk1

MLLWILLLETSLCFAAGNVTGDVCKEKICSCNEIEGDLHVDCEKKGFTSLQRFTAPTSQF

YHLFLHGNSLTRLFPNEFANFYNAVSLHMENNGLHEIVPGAFLGLQLVKRLHINNNKIKS

FRKQTFLGLDDLEYLQADFNLLRDIDPGAFQDLNKLEVLILNDNLISTLPANVFQYVPIT

HLDLRGNRLKTLPYEEVLEQIPGIAEILLEDNPWDCTCDLLSLKEWLENIPKNALIGRVV

CEAPTRLQGKDLNETTEQDLCPLKNRVDSSLPAPPAQEETFAPGPLPTPFKTNGQDEHAT

PGAVPNGGTKIPGNWQLKIKPTPPIATGSARNKPPVHGLPCPGGCSCDHIPGSGLKMNCN

NRNVSSLADLKPKLSNVQELFLRDNKIHSIRKSHFVDYKNLILLDLGNNNIANIENNTFK

NLLDLRWLYMDSNYLDTLSREKFAGLQNLEYLNVEYNAIQLILPGTFNAMPKLRILILNN

NLLRSLPVDVFAGVSLSKLSLHNNYFMYLPVAGVLDQLTSIIQIDLHGNPWECSCTIVPF

KQWAERLGSEVLMSDLKCETPVNFFRKDFMLLSNEEICPQLYARISPTLTSHSKNSTGLA

ETGTHSNSYLDTSRVSISVLVPGLLLVFVTSAFTVVGMLVFILRNRKRSKRRDANSSASE

INSLQTVCDSSYWHNGPYNADGSHRVYDCGSHSLSD

>ENSP00000320697; SLITRK1

MLLWILLLETSLCFAAGNVTGDVCKEKICSCNEIEGDLHVDCEKKGFTSLQRFTAPTSQF

YHLFLHGNSLTRLFPNEFANFYNAVSLHMENNGLHEIVPGAFLGLQLVKRLHINNNKIKS

FRKQTFLGLDDLEYLQADFNLLRDIDPGAFQDLNKLEVLILNDNLISTLPANVFQYVPIT

HLDLRGNRLKTLPYEEVLEQIPGIAEILLEDNPWDCTCDLLSLKEWLENIPKNALIGRVV

CEAPTRLQGKDLNETTEQDLCPLKNRVDSSLPAPPAQEETFAPGPLPTPFKTNGQEDHAT

PGSAPNGGTKIPGNWQIKIRPTAAIATGSSRNKPLANSLPCPGGCSCDHIPGSGLKMNCN

NRNVSSLADLKPKLSNVQELFLRDNKIHSIRKSHFVDYKNLILLDLGNNNIATVENNTFK

NLLDLRWLYMDSNYLDTLSREKFAGLQNLEYLNVEYNAIQLILPGTFNAMPKLRILILNN

NLLRSLPVDVFAGVSLSKLSLHNNYFMYLPVAGVLDQLTSIIQIDLHGNPWECSCTIVPF

KQWAERLGSEVLMSDLKCETPVNFFRKDFMLLSNDEICPQLYARISPTLTSHSKNSTGLA

ETGTHSNSYLDTSRVSISVLVPGLLLVFVTSAFTVVGMLVFILRNRKRSKRRDANSSASE

INSLQTVCDSSYWHNGPYNADGAHRVYDCGSHSLSD

>ENSMUSP00000044094; Slitrk2

MLSGVWFLSVLTVAGILQTESRKTAKDICKIRCLCEEKENVLNINCENKGFTTVSLLQPP

QYRIYQLFLNGNLLTRLYPNEFVNYSNAVTLHLGNNGLQEIRPGAFSGLKTLKRLHLNNN

KLEVLREDTFLGLESLEYLQADYNYISTIEAGAFSKLNKLKVLILNDNLLLSLPSNVFRF

VLLTHLDLRGNRLKVMPFAGVLEHIGGIMEIQLEENPWNCTCDLLPLKAWLDTITVFVGE

IVCETPFRLHGKDVTQLTRQDLCPRKSASGDSSQRSSHSDTHVQRLTPTTNPALNPTRAP

KASRPPKMRNRPTPRVTVSKDRQSFGPIMVYQTKSPVALTCPSSCVCTSQSSDNGLNVNC

QERKFTNISDLQPKPTSPKKLYLTGNYLQTVYKNDLLEYSSLDLLHLGNNRIAVIQEGAF

TNLTSLRRLYLNGNYLEVLYPSMFDGLQSLQYLYLEYNVIKEIKPLTFDALINLQLLFLN

NNLLRSLPDNIFGGTALTRLNLRNNHFSHLPVKGVLDQLPAFIQIDLQENPWDCTCDIMG

LKDWTEHANSPVIINEVTCESPAKHAGEILKFLGREAICPENPNLSDGTILSMNHNTDTP

RSLSVSPSSYPELHTEVPLSVLILGLLVVFILSVCFGAGLFVFVLKRRKGVPNVPRNATN

LDVSSFQLQYGSYNTETNDKADGHVYNYIPPPVGQMCQNPIYMQKEGDPVAYYRNLQDFS

YGNLEEKKEEPATLAYTISATELLEKQATPREPELLYQNIAERVKELPSAGLVHYNFCTL

PKRQFAPSYESRRQNQDRINKTVLYGTPRKCFVGQSKPDHPLLQAKPQSEPDYLEVLEKQ

TAISQL

>ENSP00000334374; SLITRK2

MLSGVWFLSVLTVAGILQTESRKTAKDICKIRCLCEEKENVLNINCENKGFTTVSLLQPP

QYRIYQLFLNGNLLTRLYPNEFVNYSNAVTLHLGNNGLQEIRTGAFSGLKTLKRLHLNNN

KLEILREDTFLGLESLEYLQADYNYISAIEAGAFSKLNKLKVLILNDNLLLSLPSNVFRF

VLLTHLDLRGNRLKVMPFAGVLEHIGGIMEIQLEENPWNCTCDLLPLKAWLDTITVFVGE

IVCETPFRLHGKDVTQLTRQDLCPRKSASDSSQRGSHADTHVQRLSPTMNPALNPTRAPK

ASRPPKMRNRPTPRVTVSKDRQSFGPIMVYQTKSPVPLTCPSSCVCTSQSSDNGLNVNCQ

ERKFTNISDLQPKPTSPKKLYLTGNYLQTVYKNDLLEYSSLDLLHLGNNRIAVIQEGAFT

NLTSLRRLYLNGNYLEVLYPSMFDGLQSLQYLYLEYNVIKEIKPLTFDALINLQLLFLNN

NLLRSLPDNIFGGTALTRLNLRNNHFSHLPVKGVLDQLPAFIQIDLQENPWDCTCDIMGL

KDWTEHANSPVIINEVTCESPAKHAGEILKFLGREAICPDSPNLSDGTVLSMNHNTDTPR

SLSVSPSSYPELHTEVPLSVLILGLLVVFILSVCFGAGLFVFVLKRRKGVPSVPRNTNNL

DVSSFQLQYGSYNTETHDKTDGHVYNYIPPPVGQMCQNPIYMQKEGDPVAYYRNLQEFSY

SNLEEKKEEPATPAYTISATELLEKQATPREPELLYQNIAERVKELPSAGLVHYNFCTLP

KRQFAPSYESRRQNQDRINKTVLYGTPRKCFVGQSKPNHPLLQAKPQSEPDYLEVLEKQT

AISQL

>ENSMUSP00000088561; Slitrk3

MMKPSIAEMLHRGRMLWIILLSTIALGWTTPIPLIEDSEEIDEPCFDPCYCEVKESLFHI

HCDSKGFTNISQITEFWSRPFKLYLQRNSMRRLYTNSFLHLNNAVSINLGNNALQDIQTG

AFNGLKILKRLYLHENKLDVFRNDTFLGLESLEYLQADYNVIKRIESGAFRNLSKLRVLI

LNDNLIPVLPTNLFKAVSLTHLDLRGNRLKVLFYRGMLDHIGRSLMELQLEENPWNCTCE

IVQLKSWLERIPYTALVGDITCETPFHFHGKDLREIKKTELCPLLSDSEVEASLGIPHLS

SSKENAWPTKPSSMLSSVHFTASSVEYKSSNKQPKPTKQPRTPRPPSTSQALYPGPNQPP

IAPYQTRPPIPIICPTGCTCNLHINDLGLTVNCKERGFNNISELLPRPLNAKKLYLSSNL

IQKIYRSDFWNFSSLDLLHLGNNRISYVQDGAFINLPNLKSLFLNGNDIEKLTPGMFRGL

QSLHYLYFEFNVIREIQPAAFSLMPNLKLLFLNNNLLRTLPTDAFAGTSLARLNLRKNYF

LYLPVAGVLEHLNAIVQIDLNENPWDCTCDLVPFKQWIETISSVSVVGDVLCRTPENLTH

RDVRTIELEVLCPEMLHIAQPGPSPPQPGDYHPNGGPTSASPYEFSPPGGPVPLSVLILS

LLVLFFSAVFVAAGLFAYVLRRRRKKLPFRSKRQEGVDLTGIQMQCHRLFEDSGGNSGGS

GGGGRPTLSSPEKAPPVGHVYEYIPHPVTQMCNNPIYKPREEEEVAASAAQDTGATDRGG

PGTQPTGMAEVLLGSEQFAETPKENHSNYRTLLEKEKEWALAVSNSQLNTIVTVNHHHPH

PHHSAVGGVSGVGGGTGGDLAGFRHHEKNGGVVLFPPGGGCGGGSLLLDRERPQPAPCTV

GFVDCLYGTVPKLKELHVHPPGMQYPDLQQDARLKETLLFSAGKGFTDHQTPKSDYLDLR

AKLQTKPDYLEVLEKTAYRF

>ENSP00000241274; SLITRK3

MKPSIAEMLHRGRMLWIILLSTIALGWTTPIPLIEDSEEIDEPCFDPCYCEVKESLFHIH

CDSKGFTNISQITEFWSRPFKLYLQRNSMRKLYTNSFLHLNNAVSINLGNNALQDIQTGA

FNGLKILKRLYLHENKLDVFRNDTFLGLESLEYLQADYNVIKRIESGAFRNLSKLRVLIL

NDNLIPMLPTNLFKAVSLTHLDLRGNRLKVLFYRGMLDHIGRSLMELQLEENPWNCTCEI

VQLKSWLERIPYTALVGDITCETPFHFHGKDLREIRKTELCPLLSDSEVEASLGIPHSSS

SKENAWPTKPSSMLSSVHFTASSVEYKSSNKQPKPTKQPRTPRPPSTSQALYPGPNQPPI

APYQTRPPIPIICPTGCTCNLHINDLGLTVNCKERGFNNISELLPRPLNAKKLYLSSNLI

QKIYRSDFWNFSSLDLLHLGNNRISYVQDGAFINLPNLKSLFLNGNDIEKLTPGMFRGLQ

SLHYLYFEFNVIREIQPAAFSLMPNLKLLFLNNNLLRTLPTDAFAGTSLARLNLRKNYFL

YLPVAGVLEHLNAIVQIDLNENPWDCTCDLVPFKQWIETISSVSVVGDVLCRSPENLTHR

DVRTIELEVLCPEMLHVAPAGESPAQPGDSHLIGAPTSASPYEFSPPGGPVPLSVLILSL

LVLFFSAVFVAAGLFAYVLRRRRKKLPFRSKRQEGVDLTGIQMQCHRLFEDGGGGGGGSG

GGGRPTLSSPEKAPPVGHVYEYIPHPVTQMCNNPIYKPREEEEVAVSSAQEAGSAERGGP

GTQPPGMGEALLGSEQFAETPKENHSNYRTLLEKEKEWALAVSSSQLNTIVTVNHHHPHH

PAVGGVSGVVGGTGGDLAGFRHHEKNGGVVLFPPGGGCGSGSMLLDRERPQPAPCTVGFV

DCLYGTVPKLKELHVHPPGMQYPDLQQDARLKETLLFSAGKGFTDHQTQKSDYLELRAKL

QTKPDYLEVLEKTTYRF

>ENSMUSP00000064443; Slitrk4

MFLWLFLIVSALISSTNADSDISVEICNVCSCVSVENVLYVNCEKVSVYRPNQLKPPWSN

FYHLNFQNNFLNILYPNTFVNFSHAVSLHLGNNKLQNIEGGAFLGLSALKQLHLNNNELK

ILRADTFLGIENLEYLQADYNLIKYIERGAFNKLHKLKVLILNDNLISFLPDNIFRFASL

THLDIRGNRIQKLPYIGVLEHIGRVVELQLEDNPWNCSCDLLPLKAWLENMPYNIYIGEA

ICETPSDLYGRLLKETNKQELCPMGTGSDFDVRILPPSQQENGFTTPNGHTTQTTLHRLV

TKPPKTTNPSKISGIVAGKALSNRNLSQIVSYQTRVPPLTPCPVPCFCKTHPSDLGLSVN

CQEKNIQSMSELTPKPLNAKKLHVNGNNIKDVDISDFTEFEGLDLLHLGSNQITLIKGEV

FHNLTNLRRLYLNGNQIERLYPEIFSGLHNLQYLYLEYNLIKEILAGTFDSMPNLQLLYL

NNNLLKSLPVYIFSGAPLARLNLRNNKFMYLPVSGVLDQLQSLTQIDLEGNPWDCTCDLV

ALKLWLEKLNDGIVVKELKCETPVQFANIELKSLKNEILCPKLLNKPSATFTSPAPAITF

TTPLGPIRSPPGGPVPLSILILSILVVLILTVFVAFCLLVFVLRRNKKPTVKHEGLGNSE

CGSMQLQLRKHDHKTNKKDGLSTEAFIPQTIEQMSKSHTCGLKESETGFMFSDPPGQKVM

MRNAADKDKDLLHVDTRKRLSTIDELDELFPSRDSNVFIQNFLESKKEYNSIGVSGFEIR

YPEKQQDKKNKKSLIGGNHSKIVVEQRKSEYFELKAKLQSSPDYLQVLEEQTALNKI

>ENSP00000336627; SLITRK4

MFLWLFLILSALISSTNADSDISVEICNVCSCVSVENVLYVNCEKVSVYRPNQLKPPWSN

FYHLNFQNNFLNILYPNTFLNFSHAVSLHLGNNKLQNIEGGAFLGLSALKQLHLNNNELK

ILRADTFLGIENLEYLQADYNLIKYIERGAFNKLHKLKVLILNDNLISFLPDNIFRFASL

THLDIRGNRIQKLPYIGVLEHIGRVVELQLEDNPWNCSCDLLPLKAWLENMPYNIYIGEA

ICETPSDLYGRLLKETNKQELCPMGTGSDFDVRILPPSQLENGYTTPNGHTTQTSLHRLV

TKPPKTTNPSKISGIVAGKALSNRNLSQIVSYQTRVPPLTPCPAPCFCKTHPSDLGLSVN

CQEKNIQSMSELIPKPLNAKKLHVNGNSIKDVDVSDFTDFEGLDLLHLGSNQITVIKGDV

FHNLTNLRRLYLNGNQIERLYPEIFSGLHNLQYLYLEYNLIKEISAGTFDSMPNLQLLYL

NNNLLKSLPVYIFSGAPLARLNLRNNKFMYLPVSGVLDQLQSLTQIDLEGNPWDCTCDLV

ALKLWVEKLSDGIVVKELKCETPVQFANIELKSLKNEILCPKLLNKPSAPFTSPAPAITF

TTPLGPIRSPPGGPVPLSILILSILVVLILTVFVAFCLLVFVLRRNKKPTVKHEGLGNPD

CGSMQLQLRKHDHKTNKKDGLSTEAFIPQTIEQMSKSHTCGLKESETGFMFSDPPGQKVV

MRNVADKEKDLLHVDTRKRLSTIDELDELFPSRDSNVFIQNFLESKKEYNSIGVSGFEIR

YPEKQPDKKSKKSLIGGNHSKIVVEQRKSEYFELKAKLQSSPDYLQVLEEQTALNKI

>ENSMUSP00000041499; Slitrk5

MHVCCPPVTLEQDLHRKMHSWMLQTLAFAVTSLVLSCAETIDYYGEICDNACPCEEKDGI

LTVSCENRGIISLSEISPPRFPIYHLLLSGNLLSRLYPNEFVNYTGASILHLGSNVIQDI

ETGAFHGLRGLRRLHLNNNKLELLRDDTFLGLENLEYLQVDYNYISVIEPNAFGKLHMLQ

VLILNDNLLSGLPNNLFRFVPLTHLDLRGNRLKLLPYVGLLQHMDKVVELQLEENPWNCS

CELISLKDWLDSISYSALVGDVVCETPFRLHGRDLDEVSKQELCPRKLISDYEMRPQTPL

STTGYLHTTPASVNSVATSSSAVYKPPLKPPKGTRQPNKPRVRPTSRQPSKDLGYSNYGP

SIAYQTKSPVPLECPTACTCNLQISDLGLNVNCQERKIESIAELQPKPYNPKKMYLTENY

ITVVRRTDFLEATGLDLLHLGNNRISMIQDRAFGDLGNLRRLYLNGNRIERLSPELFYGL

QSLQYLFLQYNLIREIQAGTFDPVPNLQLLFLNNNQLQAMPSGVFSGLTLLRLNLRGNSF

TSLPVSGVLDQLTSLIQIDLHDNPWDCTCDVVGMKLWIEQLKVGVLVDEVICKAPKKFAE

TYMRSIKSELLCPDYSDVVVSTPTPSSIQVPSRTNAATPAVRLNSTGTPAGLGAGTGASS

VPLSVLILSLLLVFIMSVFVAAGLFVLVMKRRKKNQSDHTSTNNSDVSSFNMQYSVYGGG

GGGGGGHPHAHVHHRGPALPKVKTPAGHVYEYIPHPLGHMCKNPIYRSREGNSVEDYKDL

HELKVTYSSNHHLQQQPPPPPQQPQQQPPPQMQMQPGEEERRESHHLRSPAYSVSTIEPR

EDLLSPVQDADRFYRGILEPDKHCSTTPAGSSLPEYPKFPCSPAAYTFSPNYDLRRPHQY

LHPGAGESRLREPVLYSPPGAVFVEPNRNEYLELKAKLNVEPDYLEVLEKQTTFSQF

>ENSP00000298025; SLITRK5

MHTCCPPVTLEQDLHRKMHSWMLQTLAFAVTSLVLSCAETIDYYGEICDNACPCEEKDGI

LTVSCENRGIISLSEISPPRFPIYHLLLSGNLLNRLYPNEFVNYTGASILHLGSNVIQDI

ETGAFHGLRGLRRLHLNNNKLELLRDDTFLGLENLEYLQVDYNYISVIEPNAFGKLHLLQ

VLILNDNLLSSLPNNLFRFVPLTHLDLRGNRLKLLPYVGLLQHMDKVVELQLEENPWNCS

CELISLKDWLDSISYSALVGDVVCETPFRLHGRDLDEVSKQELCPRRLISDYEMRPQTPL

STTGYLHTTPASVNSVATSSSAVYKPPLKPPKGTRQPNKPRVRPTSRQPSKDLGYSNYGP

SIAYQTKSPVPLECPTACSCNLQISDLGLNVNCQERKIESIAELQPKPYNPKKMYLTENY

IAVVRRTDFLEATGLDLLHLGNNRISMIQDRAFGDLTNLRRLYLNGNRIERLSPELFYGL

QSLQYLFLQYNLIREIQSGTFDPVPNLQLLFLNNNLLQAMPSGVFSGLTLLRLNLRSNHF

TSLPVSGVLDQLKSLIQIDLHDNPWDCTCDIVGMKLWVEQLKVGVLVDEVICKAPKKFAE

TDMRSIKSELLCPDYSDVVVSTPTPSSIQVPARTSAVTPAVRLNSTGAPASLGAGGGASS

VPLSVLILSLLLVFIMSVFVAAGLFVLVMKRRKKNQSDHTSTNNSDVSSFNMQYSVYGGG

GGTGGHPHAHVHHRGPALPKVKTPAGHVYEYIPHPLGHMCKNPIYRSREGNSVEDYKDLH

ELKVTYSSNHHLQQQQQPPPPPQQPQQQPPPQLQLQPGEEERRESHHLRSPAYSVSTIEP

REDLLSPVQDADRFYRGILEPDKHCSTTPAGNSLPEYPKFPCSPAAYTFSPNYDLRRPHQ

YLHPGAGDSRLREPVLYSPPSAVFVEPNRNEYLELKAKLNVEPDYLEVLEKQTTFSQF

>ENSMUSP00000077492; Slitrk6

MKLWTYLLYPSLLACLSLQSQSPMPSVRGSCDTLCNCEEKDGIMIINCEEKGINKLSQIS

VPPSRPFHLSLLNNGLTMLHTNDFSGLTNALSIHLGFNNIADIETGAFNGLGLLKQLHIN

HNSLEILKEDTFHGLENLEFLQADNNFITIIEPSAFSKLNRLKVLILNDNAIESLPPNIF

RFVPLTHLDLRGNQLQTLPYVGFLEHIGRILDLQLEDNKWACNCELLQLKNWLENMPPQS

IIGDVICYSPPPFKGSVLSRLKKESFCPTPPVYEEHEDPSGSLLAITSSTSDSRLSSKNT

SILKQPTKAPGLIPYLTKPSTQLPVPYCPIPCNCKVLSPSGLLIHCQERNIESLSDLQPP

PHNPRKLILAGNIIHTLMKSDLTDYFTLEMLHLGNNRIEVLEEGSFMNLTRLQKLYLNGN

HLTKLNKGMFLGLHSLEYLYLEYNAVKEILPGTFNPMPKLKVLYLNNNLLQVLPAHIFLG

IPLTRVNLKTNQFTHLPVSNILDDLDFLIQIDLEDNPWDCSCDLVGLQQWIHKLGKGTMT

DDILCTSPGHLDKKELKALNSDLLCPGLVNNPSMPTQTTYVIVTSPTVAADTASTLFSSL

TDAVPLSVLILGLLIVFITIVFCAAGIVVFVLHRRRRYKKKKVEEQLRDNSPVHLQYSMY

GHKTTHHTTERPSSSLYEQHMVSPMVHVYRSPSFGPKHLEEVEERNDKEGNDAKHLQRSL

LERENHSPLTGSNMKYKTTDQSTDFISFQDASLLYRNILEKERELQQLGITEYLRKNLAQ

LQPEVEVNYPGAHEELKLMETLMYSRPRKVLVEQTKNEYFELKANLHAEPDYLEVLEQQT

>ENSP00000327635; SLITRK6

MKLWIHLFYSSLLACISLHSQTPVLSSRGSCDSLCNCEEKDGTMLINCEAKGIKMVSEIS

VPPSRPFQLSLLNNGLTMLHTNDFSGLTNAISIHLGFNNIADIEIGAFNGLGLLKQLHIN

HNSLEILKEDTFHGLENLEFLQADNNFITVIEPSAFSKLNRLKVLILNDNAIESLPPNIF

RFVPLTHLDLRGNQLQTLPYVGFLEHIGRILDLQLEDNKWACNCDLLQLKTWLENMPPQS

IIGDVVCNSPPFFKGSILSRLKKESICPTPPVYEEHEDPSGSLHLAATSSINDSRMSTKT

TSILKLPTKAPGLIPYITKPSTQLPGPYCPIPCNCKVLSPSGLLIHCQERNIESLSDLRP

PPQNPRKLILAGNIIHSLMKSDLVEYFTLEMLHLGNNRIEVLEEGSFMNLTRLQKLYLNG

NHLTKLSKGMFLGLHNLEYLYLEYNAIKEILPGTFNPMPKLKVLYLNNNLLQVLPPHIFS

GVPLTKVNLKTNQFTHLPVSNILDDLDLLTQIDLEDNPWDCSCDLVGLQQWIQKLSKNTV

TDDILCTSPGHLDKKELKALNSEILCPGLVNNPSMPTQTSYLMVTTPATTTNTADTILRS

LTDAVPLSVLILGLLIMFITIVFCAAGIVVLVLHRRRRYKKKQVDEQMRDNSPVHLQYSM

YGHKTTHHTTERPSASLYEQHMVSPMVHVYRSPSFGPKHLEEEEERNEKEGSDAKHLQRS

LLEQENHSPLTGSNMKYKTTNQSTEFLSFQDASSLYRNILEKERELQQLGITEYLRKNIA

QLQPDMEAHYPGAHEELKLMETLMYSRPRKVLVEQTKNEYFELKANLHAEPDYLEVLEQQ

T

>C44H4.2; Sym-5

MLVLESLLKPASVVRFTVELFWTVQIVLPVSLTMNQAELVELPPNFFSGLFIRRLDLSQN

KIKKIDDAAFAGINPVLEEVVLNHNLIEKVPAAALAGLPNLLRLDLSNNSIVEIQEQEIF

PNLNKLYDINLGSNKIFSIHTSTFQNVKNSIQTINLGHNNMTAVPSSAIRGLKQLQSLHL

HKNRIEQLDALNFLNLPVLNLLNLAGNQIHELNRQAFLNVPSLRYLYLSGNKITKLTAYQ

FQTFEQLEMLDLTNNEIGAIPANSLSGLKQLRQLYLAHNKISNISSNAFTNSSIVVLVLS

SNELKTLTAGIISGLPNLQQVSFRDNQIKTINRNAFYDAASLVMLDLAKNQLTEIAPTTF

LAQLNLLLVDLSENKLPKTPYSAFNSRVGTVLLKENPLVCTENLHMLQQGTGVYVRDSPD

IICGRKPTPKPEPVLVPIVTDSLISTQRPALVQIPKMQIHRNVHTTTGDQAPQIPSGAFQ

QIDLGKSRSLPRGHSRFILDKPSTREQSVEPTEELTPIQPIILPSREDEIRQSSMEAGTS

QESVEATSQKIPSTTDIIDRPNVVLPFPVPFLKRGPNLSESKKVESTDMPSTSQVFHTLP

PSILIEPGSTPKVAQPSTEANIKSEHIDEFALASSNSNEPTLQPRLEKSFFTTTIIFICV

GTAVIVLVVVIAGLCISKHRQLQFENTYSDSSAARTSEYISTQYRQNSLRGTGGRVGRFE

ESPAWIYNPGSSYCNYYK

>C44H4.1; C44H4.1

MRLLLFNLLIAGWVYSQCPTLQLQEPCTCTSTRYEAVSINCDGGSSLDAVLESLSNSPQA

IDSLTISNTPIEKMPGYAFQGFQIKKLFLRNNGLRSFHPNTFTGNLENSLEELEIRGNYI

DGIPQSGVSILKQLKILSLPDNLIEYVQDNAFLSYHSRDSLLKLDLSANNLTAIHPTGLL

GLENLSQLSLDKNLLSEIPSQALENIPSLEDLSLGVNRIHTISRNSLPLPNLKSLSLEVN

QIRLIPSDSFSETPLLSYLYLGNNLLTSIDASMFLHIGGLKVLSMSNNKDITSIQANGKL

SVQQFKLCICILAFQHAPSLIRLELFDCSISRIEPKSLQKVQHIQVILLSRNQITQINAV

DDFAFSQLPMLTSLDLSSNRLESLPSNVIYDSLMQKKTSPVQRKLSIQNNPWRCDKDLMW

LRKWLRDNGDVTTAASNSPPAKCWTPSNLSGLDLRQTDSKLPHKTTKPPVDQFQYKNQQV

TNSNGTSQLGYQNPHTEVNGLALVGLILGIVLVVFVSCIILGYLMRFIFLSYEAKTKPNV

FGSTISSSGCIRNMYGGDGTLVSEDHGHPSSANNNVYLNRPRHWWF

>C44H4.3; Sym-1

MLLRLCVALLVLPACLAFCPKLFQNQTACSCDSTVEGPVIKCSGHDGLRMVEKLSTTTTM

EVRELALENADIIEVGPKAFKTLRIKKLILSNNRIEKIHDHAFTGLENVMQELSLSENNL

KEVPTSALAGLRVLNILSLKCNKIENITTKAFVNMTSLIDVNLGCNQICSMAADTFANVK

MSLQNLILDNNCMTEFPSKAVRNMNNLIALHIKYNKINAIRQNDFVNLTSLSMLSLNGNN

ISEIKGGALQNTPNLHYLYLNENNLQTLDNGVLEQFKQLQVLDLSFNNFTDITKEMFEGL

ESIQHLNLDSNRISAVAPGAFAGTPLLLLWLPNNCLTEVSQQTLKGAPFLRMVSLSNNNI

REVHELSFDHLPNLHTLDLANNKIMSLQNKSLSGPENLAVRLQENPVVCVKNGFHVLNSG

EAIWLTNEANTICKGEWQAQTADLCPKAQPRPIRPVCCSNEITTTTTTTTTTTTLAPTTT

TEEEKEETTETTEKVTTVGKSKKTKATTTTEEPEDDEDVYVDDDEEGTEEEEEEVSTTEK

STTTTTTTTEASKSKKVNMERFWRLSNKPSGKSPFLRHSQGNKPRYLQTTTEAPEEEDEA

EYISDDGGAEESEEAETTVATTTTTEPSVKVPARIRERQRMFASNIPWMAPRTKTDSVEE

EFSKDDEVETVETAEKPARH

>ZK682.5; ZK682.5

MGLRAIILATLVVTVYGQCPALSGACRCAPSVYEPVAIICQNAGSLQNAIQAIQAARDIP

IDSLTILDTAIPTIPANAFQSFTILRLVLNRNTLQNIDDQAFNGPLLDSLIELDLNDNNL

GQIPQTGIPRLRNLRKLYLNRNRINQLSSTAFNAFESRDLLLKLELAGNRLTDATLGDAT

VFRPLTLLQELSLETNSLTSIPSSALVNQRNTLTNLNLGLNSINEVPVGALDFPVLSSLS

LEFNGITVIPPQAFQGVPNLQFLYMTGNKFPSWAPEMFRYITQLKTLGIGETPISVIPNN

AFMHIPNLIRLEMSEAAVDTIERGAFQRTPQIQAIVLNKNRLSQVRADFFEGLNDLYSID

LQGNRIDNVQPLGFANLPAISHLDISYNLLQTMPSNVFQNSFLPQPNDRRVIYACGNPWY

CNSELEWFRTLLRDNLDIDVEKPGCTAVCTSSPNGCPVEGTPLRSVDFCQNNEEAQPLVG

RALSMVGWIILAVIMTILLISICLLAMVRYGMSHQRKKQKDAEVAAEEYHHQTTATSIYN

APISVVDRPYSTVPPVNLDLPAAYTLDDRPNNYLY

> T01G9.3; T01G9.3

MISYILLLFLSFLVLCSIALPSSCPNLCECDQNDSSWSVYCRKAIINDTIYAEILNQLPL

TLRSLHIQPPSNRIGSNKLRWNDNINRFAQLRVLRLINCQIPAMSRSIRLPSLEVLDLHS

NNIEHATMSNFGGMPKLRVLDLSSNHLNILPTGVFTYLRALRSLSLSNNTISDLSTNLLR

GLNSLRVLRLDRNPIPIEHINELFTDVSQLDELYLNHCNLSSIYSLALDRIPQLRQLGIG

GNNLKMVPTKELRSLPQLSVLDLSHNSIQEITACAFCNTNISKLDLSHNLLGISKDSPFN

EDAFRTMPLRHLDLSFNHMNDFDSKWLGWAQEELTSIALSGNFLKNFEESWTYTLKSLIH

LELAYNHIKFIPVQLPSRYYHLISLNISGNELTYLPDNINTLLPNVKTFDITANRFHTFS

HTDLAFLNNVEQVYVDGNPWDCSCAIQGLQVHMRDRYAMRHILNYDNVRCATPSLVEGHS

VLAITDVNDCAVLFGARYGLTQTSEMLILLAGVLLFAALLLMILGCIYFLRERQYKGSYV

TREHSRTPLTMANTHSCSSSTNDTHGPLSPPFDPFLVSTETFKATPPLIPPAPPKPGSSY

FGI

>K07A12.2; K07A12.2

MRWLTLIAVAHLIAFLSSAEITCPRIPEKCDCKISKSMIILSCNGEDVKTIAQTVGTSQI

DELHILNGTDVKIESLPFNGLRTIAILNSTLQSFSPTAWRHVEATIEHITINGNELKTVP

VFGNLSTLMSMNLNSNQISSIPDKAFNGLSALTQLRLENNAICDFPPKSLDAVKASLVLL

DVSGNCLDAIPAQILRNAANLMYLDLGSNNISEINNFELMNLPFLRELRVQNNTLRRIHP

MAFMNVPQLQYLYLQDNIISTLDGNRLQGFKNLEVLDVSNNALYALPSLKDLPNLKQVRV

DGNLITKIETLAFSNNPNLQLISVQNNNIVQISRNSFESLDKLVVLLVGNNSLAKIERGM

FDGMKNLQQLSIRNNTLTALDASSFAQLAHLTTLDLGHNKIHDIEEGTFDKLSKLFWLDL

SNNKISGFKTSVFKKKISNILLDGNQLICDESFNEFLTYLIANKVRTFLPFQQEIMCHGP

EKYAGVRLKDLMMKKANETLSEGSRLLGVPQGSNQHSLLSSFLPSLGPLGTLNGAGGAAI

PLVNTLTNTIPALRSIPGFGGNIPVGTGASSVPNKNLNDAIEGFTGPLVRFATGGQPVAS

DIEQLIRSIPNMVVNVPGFGDIDLSKMDPTMIQYVLNGGQIPGIDKATLDKIVKQTMNKM

HTAAAANLAGNPVEGQEKVLPPLDKLPSGLVTQVMSGEPLPGLNENQTKIIMEYYTHQMP

GMDGIPARPVESQGNTTANNMFNPAMFDLLKMLPPGYNLSKIPMEVIAAVTRGEVPDMRL

LPEDLLEHFKQHTTSLTSMFAGATAKNISIEEILEKLPVFVRPELSTFVPYDINELTSEM

VLEQEQNERHRNIRIITAIALAFVGAVTVVVIIFFVNYTKKQRRLRKSLVYRSSPSSSGS

SGQNAANESGRSSAAPSPIRPPLMNIPKTPNNRTMESTFGQPQLCSTLLENPQAVSHRSR

H

>T23G11.6; T23G11.6

MRPLVAIFLVQLCFVAVGAAIPNCPDLDVLENQSDLSQDDLNNLILCFCKPDDKEKVVIS

CLYGSNLDHLMKATEAVKNASLITSSISIQHMEFPDGGLPEFGKLAPDLKSLEIKECSGQ

DELKVGDDSFKGLEQTLRNLTIHACNLQTIPPSVDSLENLETVVFSNNKLDSLGVDQFKN

KKQLSYLDVSGNFITSIEEKAFEPLTSLETLVIGEHNFINETVVEEIGRLKALKTLDLSR

ADGIFQPPETLFKEIPQIEVLKLSGCSIPTLEPGQFATLKKLKELDLRVNLIENITAYAF

DGLESLTRLSLAGNFISKLEPDVFFGLSSLEELDLGWNEIKTIPTDVFKPLTDKLKTISL

RNNPISELPSTGLGMLEKLSLAECGFTSISADQLKDYPKLEELDLSKCNISNIVENTFEN

QKDSLKKLNLQKNKLKSLPNLIKNLPAIESLDVSSNPYRCDGELVNFVFGVEDRVKKAEE

NGNSFFVANTNETVCDRPYTLRGEQILQVEVEKFQPYDEKSDTTTAPSTTTTSTVEETTT

ELKIPDLLVGSRTNDTLFKEEPRRDVYDLSKTDDAKGVYNQKGTYAVPITIGAIGLVTVI

VIVAVVLFIKKNKKVGTETAKGGKSDKVVKMEDGMVEIELDSQPGTQHATPRR

>C02C6.3; C02C6.3

MPQRNNQIHSYNIPRRSGLISITYLLISSTVWVVQAGNRQEYQYSDDAIENKLICAGKLA

DYAACQCDPSAGEYSCINAQFVDANVFLDIATNYKYIKSVTFHGNNFQDLTSTPLFGADS

QTSLIKLNLSANYIVNLNSNALRNMPNLQVLDISNNEIVFRPRDVDFLKHTPHLTQLYMR

RAFTVTINRTQQFELMLEMFRQAKLEYLQVLDLSYNFIHNVPFEIACPFPSLHTLDLRQN

FLKNFIVNETCIKEVNTINLSRNQFYVIPKDFRALADKAQPETFLLRNQFYCDCNSKEFI

TWLRSTKSVREKNSLVCDRASPKMYVGARIAEVPLNKLTCDEPLFTSMSSFKFNFYSMII

AFIVSKMMF

> C41C4.3; C41C4.3

MRSTITFFESLETHMKFQIYFFLLISCVLAKKPVIFCDDAYGRYTAVAGNLDLSTFEFPI

MSKISSANDASGSKMLHPCYSLSNTNNYKNQLFDIFSVGEFAPNICERQCAHFVDVFRFE

CGDRRPPPSWLAGTVIGAKRKINCSDWAIPLTEKGKMDGISLTFTYGTYQSSNGNDLSPP

YTLDNKSIEYLDSMVIKGLHIKPVVDPTKKDTAMSEMESMFEQFVARHPEIIHIAFGNPI

TFFGDTFRNDSKLYAPRFIPTSKFWATLSKIPSLKSLSLSVIEITGKEDIPEGMTRNLRS

IGFYDVKMNSIPNWIKTDQIQFLEFQSSISDNVDMSALDELPTLEHFLLTESSLTSIKSP

FLSKSSKLISLTLQCNSISSIAAGAFDHFTELQFLNLAGNRIASLPSNLLLNLNNLIVLD

LKALDNSSNTIGTYNELSMQCQINQKASTVPVILDKMPDVSKPEKLVALEIRGQENIMKN

DKSFLKNFKNLEILNLGNLGLLSAQNLSLETLCNLIDLNLVGNPLSDKDWISEDLFVNLN

LQRLRMGQPSSMTSIPNSLVAFMRTASQIMYSTPISLNSVDIYKRKIGYRISSATMFGYS

LRNASCESNVKSAIENIRTMELQKMETQCQ

> F10F2.4; F10F2.4

MHILIILLFTSIISSIQADLCAHCDCDFISHTVVCNRPSLLIRTVSMLPTIRQLHLNSLN

LPQPPHFLFHPNLRVLRMSRCGMHEIPGSTFLPLPGLEVIDLSNNHLETLPPTVLRSLKF

LRVLILSNNRLSNLDQLTWILAPGVVLEQLDLSGNPIAIATSMTVFPPVRQLFLSDTRME

SVNETAIMFKKLPGKCDRDVCRHIPIHNLNVSIITTIDFSSNRDLEIDSGALDVFSNATY

VDLSNTRLPVGFEEWLERKSRVKSLNISHCQLPLHEDTWTACGQFLHSLDISGIGAKRLR

FSRFCPIRSVFARDNLISSVYIDAVSMESLHLERNMFSEFPIPPPGVELTELHTLGLSHN

LMTSLPPHALQSYPNLQHFDVSSNQLSEIDPQAFPSIGLGLISLDLSSNQLSSLPHPILP

SLLLLDLSFNTISHLDPHFFTGLPMLQQLRIASNPTLFSRCPNRDSPCWSDHLDELTSLV

DLDISNSGLEFSLHWRHLRTLKSLILRGNEIRIIDSKSLPENLRTLDLGENRVQFTSNFS

KLEHLRDLRVDQNPLRCDCSLYDIVPHLLNQSQISDPLLYYCFSGSWQYPLLPYLASVKP

CVDTTRNFYSILITTFIIAFAIVAGLIGGFLVYRKYADRANFVYKRISLMESPVRL

> F37E3.2; F37E3.2

MWELSLAIFGLFGLARSCDPLHAQAHGINLVCCSLNHSLTPSCEFSSLCQETCSCFLTIT

RDLDCKQPNLPNPDAPYTIVIENSNESLTHSFLLGSCSKTVTSLTFQHYHKLYDSIDFGE

CFPRLASFQVVLLQRTKANFHGSLRNLKNLKVLSLVNVDFDFWLQPSPFINTITYIHIEN

SSITELPKWLSTSKSLSTVYLKGTSISSLTPVAQLPAVKSLKLSHNLIENLHRLLFVSPF

LIHVDLSYNQITSFASHTFSKCIDLRVLDLTGNPIKMLPYKPFAKNIKLKWLKLSRTNIT

TLTPDHFYGLNALKTLSLSRMPIQSISPYSFVPLKSLRYLDMDSCNLTKIPQAVTANCHL

ARLNVANNMLHRSSSMPPEVMAMLSGLSQLRIEGNPLTEFPASFLLISRENFRLLRQLLH

STMTLPVWLREPCTPYYWSMHLANRTNNLKSFVNQYSEMRMLKSGLGYCKEQYDWMLEQM

EVYRELEKNSGCYSLRRLRSSLSSKPVPKISKNSTNSLILSIPHDLSSPAFHIPILLLIS

LCANFLLLFSILMCSIMACRMEKGVDSV

> K03A1.2.1; K03A1.2.1

MRLKFALLFLILVVYSFAYNPVKSNDKDKALADNSKKVKVKKAELTAAQDEDDEGSKSLD

EVEEDAGIDDDEQDDDEDVKEVPKKKSHVKQKKSKKPPAKKDKTRKSKKNDQEITEELKD

EIEEEDDSNEYQACNADTSPDNELCHCDVHEIDCSEITMESGDPYLRTLDVAIMKKDFEP

ITAKFTKNKISRLQNDKVLPKFEKFVSILDVSYNEIRFIDNDVFKPFTNLTKLYLSHNVL

QTVKKDVFDAAKNTLHRLDLGYNRIKVVSDNSFDTLSKLKVLSLDGNPIKAWRKEMFKGL

DSLEELSLDNCNIENLPADIFEYLPKLVKLSLRENPLEEIPAVVAHLKSLKDIDLSVTNL

TEIRDHAFAGDSDLEEIILEKMPFLTVVRDCGFCGLPQLKTLILNDNKYLQELHPNAFGY

IKSQPGHKSAAITSLQIHNSNISTISEHMVDYDNLKTFQVGGNPWNCNCDTQFMLEEKFA

FKQDSVAPKCTSPAGLNGRLLVTVRASDACEDARFLGRSGRFSSVLGLALLVGFIAIGTY

YMVSSGKLERLVRRVRKEPEVTYTNLQNAGEDFALETDFQPRPAEV

> Y75B8A.5; Y75B8A.5

MILLLGTTYVQKFNIFIWKLLILFHFLKYSNALRDTCPMGCQCDEDKQQVTCEGQQVVTL

PDRLPSGYESLVIRNSSVRTIEKNSFRKMEKLMQIEFENNPNLGTIEKLAFKGLKKIRLI

KFTTCPGLTELQKNAFSGIQNQMGLKIIFEKTPIHRIDGHTFRNAQNIRELTISGEELAL

SRHCFANINQLDFLTVSGVVLIEPEIFTNSTRFHVVHFKNSQFDIPPSTFSTLSHTSHLL

IEHSKVPSIAPDAFSGLTTIQVIELHACQLGTISARAFANVENLGELKILRNTIGDLDTS

ESIMSRALKTRIEENTLECSCGMKWMTSVEEMSDINFCSTTASFRSIRSFIKAKCLNPSQ

EISRKTNHLPSISSFSSSSIVNFYFLSLVWYFSKTDINNLQNRFDKNLMPCRNTFALLPW

QNLHQISESLIDFTVKFSRKRARSRSFA

> Y76A2B.2; Y76A2B.2

MVFRSSIIPLIRLLLLPYIISGLTTCPRQCICHEHSIACSCETSEKPELIISSLGSTYIT

SLVVHTCDKVTVQNGSFAGVVLVERLSFIAIGRLYFEPHAFKDILQSPRQLVIDECTISS

LAPVSFAGLSHIDHLWFRNSRIDVIATEAFHYLTNIDYIYFHKTKIGRIERKAFSKMYQI

DHLYFKDSIEIATIESEAFSGSQVDEMIFDGVTVETAHDTFLLNVESDMAILKNCSIYLI

PRKEDDMVVFDEKQKIIERCLIESSSFNIMSPYKLGCNVLEVTSSVIQRIGPVSRDIDTP

VFRPEPFIISSLNSVIFSNCSIGSVDSFAFTNYTLSILSFNHSRIGEMKTRTIEKSKISN

FEFGGSTVKLCNSSSIDKSKIQNFEVSGAEIDRINSDWIQNSMIRKLKIRSSKIGKSEKN

IFKDSVFEEVEIDGNRILMMDSETLHGDNQISTLRITGNDITTSRPSPSFLSSSPQSHPS

NFILANNTFDCLPNDCSTNSFLLNSPKHSPLLYKISSNRCRPPLENPCVEPRSISLEDHG

ITCRISSLVADCACLTEKSSIPSKFPLNYNISVVILGDCEHLTIDQKLADFTQIYIFRTT

KLIVQRLPKSLKVLKIFHSTVTLEPSSFLNFPQDWEVSNSKVEHLGLSNLNISSLHLKFS

RISHISAAKNSKIKNLHIENCHLESTQHLFEISQTLQMHNSIIFSSPRGLGTISSAQLQN

NTLLECCNLIELDSRCDLHFFGSRCVEEDDVSTNHLPITSGSNITSFDVYIAIYLFVSRI

LL

> ZK994.3; ZK994.3

MNLYLLLLVIATSSWQFVAGLECPVECTCDKKGLVVDCSSSGLTRIPKNISRNVRSLVIR

NNRIHKLKRSDLEGFNQLETLVLTHNKIKIIEENVLDHLPELKRLSLAHNELVYIPPLCS

DSRPLASLNLKRNHIQFIDEQVLRYFPDLTQLDFSHNRIQSLRTKLFDNLPALSHAHLHS

NPWHCDCRASKVKALLQKVKWEKKVYCTNPVELRHQALDEVDDSALTCARPAEESWTGEE

IKLTCAKNSSSKLVVWMYENVEVDSSSLDGYEIHDTVITVPRKTNVNFMTCTYDFDHIPH

HRRLRQSQHQGNGSPQFTYKPRDNSFREGSEVKVNCEVMGNPKPTINWYHNGKRFISSRK

RQLGLSNNVLRIYPFLEEDSGRYTCEAVNSVGKVRHAFSLDLISSVPPNIYEGPQSVSQN

LGGSVVFVCKANGNPVPDYTWSFDGSTIGHIKGRFMVSDDGTELRISNIEKKDEGYYSCM

AGNPVGAMSADAKLTVIGGETRKAAAPQIDEELLRAIAQKARQNVENAVEKTRKQLTQDK

VTNTNDLKRLFRFSTPKQAVELSKAREIYEESVRLVREHVEKGLILNVDELHPKNVSYES

VLHVTHVQALMGLSGCHTGQYKNPCTDTCFHHRYRSFDGQCNNKNKPMTGVSLMPLRRLL

KPVYENGFNTPVGWEKGRLYNGYPLPNVREVSRQLVATENITPHSKLSSMVMQWGQFVDH

DLTHTVTALSRHSYATGAFCNRTCENLDPCFNIPLSPNDPRVKSGSAKYPCIEFERSAAV

CGSGETSLVFNRVTYREQMNALTSFLDASNVYGSNEVQAQELRDTYNNNGMLRFDITSEA

GKEYLPFEKDSNMDCRRNFSEENPIRCFLAGDLRANEQLALAATHTIFIREHNRIAKKLK

SMNGNWDGEIIYHETRKIVGAMMQHITYKHWMPIIFGGQAQMNKFVGTYQGYDPDVDASV

TNAFATAAFRFGHTIINPSLFRLGNDFMPIKEGHIALHKAFFTPELVLTQGGVDPLLRGL

FASPLKHPMPTQLLNMELIEKLFMKGHEVSLDLAVMNIQRSRDHGLPSYTEYRKFCNLPV

PVQWEDMKGYIKDDMIIQKLRGLYGVPQNIDLWVGGIVEEKLENGLFGPTFACIIGEQFR

KIRDGDRFWYEKDGVFTPEQLREIKKITLARLFCDNGDNIDRIQKDVFMYPGMDKENYGT

CQETEMMNLRAWSKCCDNVCPTMLDRILRSRHRGSRLHGCNQNGIWRPEGAKWIPQNEIC

TECVCQGSRVWCSTKEDCSDNRSPF

>CG11280-PA;trn

MQRNMMIAFVGIWCILASIGVEPAAGLANCPPGCQCDDNTLVVQCGEGQLDVLPIALNPS

IQRLVIKSNKIKTIDSSIQFYAELTFLDLSSNHLMTIPQRTFAYQKKLQEVHLNHNKIGQ

ISNKTFIGLSAVTVLNLRGNQISELHQGTFTPLLKIEELNLGENRIGYLDPKAFDGLSQL

RILYLDDNALTTVPDPVIFQAMPSLAELFLGMNTLQSIQADAFQDLKGLTRLELKGASLR

NISHDSFLGLQELRILDLSDNRLDRIPSVGLSKLVRLEQLSLGQNDFEVISEGAFMGLKQ

LKRLEVNGALRLKRVMTGAFSDNGNLEYLNLSSNKMLLEVQEGALSGLSQLKHVVLKANA

LTSLAEGLFPWKDLQTLDLSENPLSCDCRVMWLHNLLVAKNASQDDVSELLCEFPERLRG

ESLRHLNPAMMGCTHADPRKQALIGALLVGSAATITALALVLYRCRHKIRETIKGGLWGN

SALGRKEREYQKTFCDEDYMSRHQHHPCSLGIHSTFPNTYTAPHHPGATHHYGMCPMPVN

DLGAIDPQQKFQQLVVPTATMISEKKLNNNKALVSQGAIDDSASFVLHMKSATMGRDVHQ

QNPQLNHYTKPQFLSATATVGDSCYSYADVPMVHGAPLGGPNQPQLRLTQEHFKQRELYD

QEMGSEILDHNYIYSNTHYSMPLEQLGRSKTPTPPPMPPALPLRNGLCATTGRRSFQQKS

ASQKQQQNNNTLRQFTH

>CG11282-PA;Caps

MSRRRAITMSLAPHLGQAFSLCLCLCLCLVLATLPVALGLANCPNGCECDDDTLMVNCGE

GTLDVLPIALNPAIQRLVIKNNKLKTIDSSMQFYAQLTFLDLSFNDMLTIPERSFAYHAK

LQELHLDHNKIGQVSNKTFLGLSTISVLNLRGNLIAELEYRTFSPMVKLAELNLGQNRIS

HIDPHALDGLDNLRVLYLDDNTLTTVPGELTFQALHSLAELYLGTNSFMTIPGGAFQDLK

GLTRLDLRGAGLHNISGDALKGLVSLRFLDLSDNRLPAIPTAAFQRLGRLEQLNIGQNDF

EVISSGAFSGLRELRHLELTGAQRLRRVESGAFSGNTNLEHLNLSSNKQLNELSSIALGG

LPHLSTVVLKANQLSSLDEGLVPWADLQTLDLSENPFECDCRLLWLRHLLVSRNASGQYA

PVICAYPTALRDLPLAHLAEPLLGCAHGAASKQAIIGILVVACAALITTLALVLYTCRHR

IREMLKGHSALGRKEREYQKTFSDEEYMSRPPPGGGGVHPAAGGYPYIAGNSRMIPVTEL

>CG5195-PA; CG5195-PA

MMLLPIFLLLCIGINLIRAESCPPSQAILPCRCSLRGKEIQIWCSHSNLPQIMDGLKAVE

RNIKGRIDELVLENNQLPALPGRFFGSLQIVRLMLRHNSIERVSNGWLNELENGLVEIFV

VEPQLRSIPAESLNGMINMLAITIQSEELKHLPDFSGLLSLTYLSVQTGALQELPSHLFR

HLPKLQHIHITGGSGLTRLEAGLFDGLISLKNLDLSHNGLNWIHLRALSRLPNLVSLKLS

HNQISDVGMVGRIVKDLEHLKKLRLDNNLITVIEDGSFVDLPNLSELHLNDNRITELQYG

AFLRTPQLKTIYLQNNLIRRIHPESLLQASGSGVEAVHMYNNEIGHVEALRALLDALPRL

RYLDMSGNLLSELPYGALRGHGTLEQLHLNHNHLRLIERDALMAMPALRELRMRNNSLSS

DLPLPFWNLPGLKGLDLAQNQFARVDSQLLAGLPSLRRLDLSENGLIELAPNSFRHNPLL

ETLNISSNELTKIHSSTLIHLERLFEVDASYNQLKSVIAGLPRIVERISLKGNQITSLPA

AASKDLQLPNLRMLDLSQNRIEQLPRHGFQGAMELRVLSLAQNELRQLKDTSFIGIQRLE

LLHLQENQLGEADERALLPLAELRNLNLQSNKLEAITDNFFSNNSRLEQLDLSRNLIRSI

SPTAFDTQRSLEYLDLSGNALLDISVGLGNLNNLRDIDLSYNQISRIQSDVIGGWRNVVE

IRLSNNLIVELQQGTFRNLPKLQYLDLSSNEIRNVEPGALKGLDELQEFVLADNKLVELK

DHVFEELPSLLASHFQYNKLRYISPESFHNANSLVFLNLSNNHFRNMENIGLRSMRNLEV

LDLSTNGVKLVSTMPLKALNWLVELKMDNNQICRIQGSPFETMPRLRVLSMRNNQLRSIK

ERTFRNVRGNIAILDVDGNPIDCNCEMQWLSVWLQETNFPYPGPKCQDGRLLRSARMERS

LCVGADIYGNERTDGNQLPLLNEHGDVFQRDLPDDFNDECEAGEGTRLPGDRPLVGESEY

FYDQYVDATEAPDTTHSAISTSQRPKPTPTINSNIDLNNTILHTKYFNRKPQPGSGSPFT

FFGYPLPSVSLGRFFGFGDRGRKQRTDSNDDMPATHRMAHISLPSGRGKTRMYQPNSAEF

EKYLKDQQKQEKLNIARNRYVDTDSTTSSMEDALSNESGSAATTVGVFRTTFREPSSIER

GGFRPIVPAHVGGFMPVHDPQQRRGLVEVVNITGNPSEIVQGIGQRKFIPISTQARPKPT

KSSGESSETATYEVTETTPDITTTTPLMQRIATSTTKASTTTTRSTTTTSTTQVTPAENN

ASSSTELDSQYDDEDLEAQSVTLLRPPPLVQTTTAETILLIPPAEEHVAQIKSRSWVTTT

TPQSPSDNQVTLAGPTSTVPPPPPASPPLRGGGRSTITKVYTPYQQQVAQPTAEEYQRTT

PSGDNEGVASEHQLTANAQKRTELELLQVDRVDRKDGMDWYYESFKKKRDFNGGAAVRKT

AHKEVFYDGVAASSSWNRLHKEILFISLLLLWRAC

>CG6749-PA; CG6749-PA

MSQTANWVLPLLLWLLCMGFPILHGADLCQPIGWRNFQCSEVASLQDLVDLGAENWHTLA

IRNVQTELEVGSGENADHLANLLDLDLTGAAPINVHTNGFSILPNLRQLNLSGCGLVDIR

GNHFAPESALQRIDFSHNQMELLDRDFFGNLRKLIYANFSHNALKQCDLPHMPLLNRLEL

GHNRLVNATFGVCPQLQELILNDNQLIQLDVNAFRGLHGLLELQLSGNRLSSIGLETFQP

LAQLRKLNLSQNALDALRPNVFGAVQNFVLHLQQLDLSGNRIRLLFDNQFRVLARLQMLD

VSRNSIASLSPAHFVGLGSLRKLYLQYNAILEIKPATFAALLNLDTLDLSYNNLEFLEEQ

IFGGNTLPRMRRLNLNGNRMKHLHPLAFSSLPFLEYLKLGHNELKSLDVRMFAPMRRLQK

LHLGHNLLEEINLDVLESLSSVQEILVDNNRLTFLAKVNVSFPNLKRVAIEGNPWQCPCF

VKLQHWLATRDVVYLRDNTGYYKGERPLCIVTNVDYCIQNLQAVRRLGILGDFQGEQVEA

DAFDDDASAAQD

>CG18095-PA; CG18095-PA

MYLLPGSLLLLIFSFSPRLSCLQLSKCNNTEVTLIRKTELLTSLTLSNCTLPHVENGFFV

RFDHLLHLELQHSGLSDLDDFSLNGLTKLQYLSLSHNNLSSLRSWSSEPLGALTNLDLSH

NMLSKLSVKSFEQYPQLQQLDLRYNRISQIENDSFDGLSHLKHLYLNGNQLAHIDGSFFR

GLHRLSSLSLQHNRIEFIEMDSFESNTHLRSLRLDQNLLSSLQFLSQRGLARLVHLNLSS

NLLQKLEPFVFSKNFELQDLDLSYNNITKLNKEALSGLDSLERLNISHNYVDKIYDESLD

SLIALLQLDISFNLLTTLPDNLFHFNTQLEEIILANNKIEEISSQMMFNQNHLRYIKLSG

NAISDAAFLDRLSPSVNRFTLYVDLSSNRLKSLNLSSLLHFRYINLADNNWSCNWLVANL

VQKLPNSVNFARPWTVINNLSENTTNVEGIDCIEGGTNRSIILLDVSGVPQPKSDNCDCV

VAYDETNPSPPPLTWPKIPTDRFDSRSVIIWMLVAIAIAFSGLRWLRRFVDRNEKRKLGQ

KMLHNVVC

>CG4054-PA; CG4054-PA

MPDHTRVTATTRSRPWLCGAIPVLLLLLLTLVILPPETTAFCPSKCQCLGGEANSRALCV

DAALEDVPIQLNPETKYINLTVNRIRTLEFSLPFYMKLEILDLSQNIIETLGSKNFEYQS

ELRTLNLSRNLVSSLHKHAFKGLTNLLLLDLSFNRIETVHPTALSDLASLVELDLTNNNI

VSLEDNCFKGMNTLEVLVFRNNRLLDVPASNLWHLHALKSLDMSLNLVEFVRNDSFEGLK

ELLALSVQGNVMSELDLSAFEGLISLKHLDLSDNNLTRISHPLERLV

>CG4950-PA; CG4950-PA curated

MNRTRRILEVIVILILAISSCDGSGGDIPLRCDEYDS

MGYMDVNEAFCTVTGFTVTHSQNVVIKNIPPGNMVKFMKFYESTLLYMPFHLFETFPYLK

TLDVSNTNILELTRNAFSAASNLTYLNLAYNNLTSIQTSVFIGANVLMRLDLSYNEISSL

SVNAFCGLHTISQIYLTGNLLKELHNDIFKDNEYLEKVSFEGNLLTSIQPEVFRNMRRIK

EVNLSNNRLIFIHPDTFADAASLENLVLSYNELKNFQLTEKNIVHQLHLDNNYLTNLTIN

ATRFVRASHNQISELFLHQSLHIETLDLSANKLSSISNITNITHMLYLDVSDNPIGPLNI

STFSQLKRLRGLNLRGTGIRELKFGMFSKQKYLEELDLSFNNLTILNLDMFVPYLTNLKK

FLIDGNGLTELQGNRTFSEAFPQLQKLGVSRNRFNCSYLHHLLIPPSLPESVVLNIEPDT

NLDETPHIRDVSCISQSQEVVNASFTAEESKLESQIQMLSKQLDIVGSHSKNLELHLAFM

QIFAYVMGGLVLVGVVSLITLRYLKNQRSGRYDRSSIVFRSNATMEANLTLGSEDLQ

>CG15151-PA;PFE

MLFKWLLFSLCIMPAMFSNTKRKCPTECQCSMDDLDRYQAICTKGGLNSLLSPNELDVDV

KVIIIRGPRNSITIGPALRQFMKLEILRITDSNLPAIGAESFWGLKYLRILDLSKNNITN

ITENNFRGQDNLLELDLSKNKVLRMASSTFRHLTDLRRLNLADNSIVELVQRNFFMLSRL

KYLDLSGNPLQDLQPDVFRDVPELKVLKCRNCQLKKINPQMYNLLPLLSELDLGRNEFKF

LDKDEFRDVKRLTKVLLDGNQLSVVVDQLFRMQKSLNHLDLSYNRLAKVPNDSFLQLTNL

TFLDLSYNKLVRLEPQSIRSLSNLLTLNISGNVLMDLREMRETFELIPQLTHLAIADMGT

MPVGLLHPFKQLRYLNISGNSLNNTALEVIDPCRELEFLDLSRNQLHGISEDTVLRIQGI

RNVRLDNNPLICDECHMGKLINVVRQLQWKWDTYPICFLPKSLRGAEINNLDINGLHTCL

TFITDEEQNAASTSYNFLEHGGLNTLAILGGIIFVLIAVIILSLVACFSKNRARYYTRED

HLNGSESKCLEKNLEATTITTLGNGSSPTTTTTLTLATSPAAPNGPKTNGQGLSLSPANG

STPVHGISDDKGKEINFTFPVDDRVCTIDELMPSPPPPPQQHPHPNMGSLMYSVSHSPNS

ATTLAAVAAAATVIPPGAPSHSSMPTPTPTPAEAVVVVLPPPPLPPQQHHHQMDGSLASL

REVQQQLVHLSSTLEPLVSVN

>CG18249-PA; CG18249-PA

MHFYKYILLGICGLTVSDALAVYDASGRFNLSASCPDSFCSLSDRPVAYSATPALQLREL

HLRNCSRQSITWLVLQLTPGLRTLVIRNCATYHISKESLRPVENLTSLQMQGTSLGVLRD

QIFNAVPRLEILQLSQNFIHTVHVAAFQGLSKLRLLGLQGNAIAEILSSTLDPLMELVHL

DLSRNELTTLPQNIFAKNKKLQTLLLNGNPLRILMPDVLGSLPNLRLLDLGHAAELEVMT

LNLTNVQNLVLEGSSLSSLVINGGFIKLQAGNNELNHLQVGNKSSVIEMDLHGNLLNGND

TAALLRGMWNLQRLDLSKNRIEALPQHGSGLDASGTQELLILPSLKFLNLANNQLVRLPP

ESPILSSRLSYLDLSHNLMLTLDVAILRSLSVLKGLYVEGNRLNTINYQKLHEEHPDLSE

LGLHDNPWSSGLYRKMFLYFTDRGVHLQARPQNRVLNKSSRVDIDWPPNEGQAEAQKMDP

PGVTGIHPYWTLRDILAFVTLLVVMIILLMNLYHILEEEGCLRRFRHWRRSRTLGQNTST

TRNSGRRLDEQDSEV

>CG7800-PA; CG7800-PAv

MYLIIIALLASVSALGRPDLEDYCNDSYCHLLGRSRTFSDKKATKLTEFHMDSCEKKVLK

LMPNLRTLELENCDSPDFTMNDLNQLPYLTSLQLRRGNLLGLHDEHFSKWPNMKILMLGG

NNITRLSNECFKGLAQLWLLSLPGNGIQGLPWDVFQNLPELLHLDLSGNRIETLHENIFT

GVPKLEMLLLNGNPLTWIAPTSLKSLSNLRLLDMSNCGPLPDLSLPGAHTLILDNSGVQR

LDILGSVHKLQARKNHITEIKLPDKSSVIELDLHSNLLTATDIPKLLTGMWRLQRLDLSE

NIIGIYAAAGSDNTSELFILPNLMYMNLSANRLTRLHFDSPIPWERLTHLDASYNRIYAP

AKVGIDEAFNLQSLHLEGNYINNFELTPWKPHPSLKEVALYDNKFQPKGYKNITKFFNEI

GVNVLEKTQYSQSNNTTPTCKPCIPDARDFPTSISADTNQTIDTKVNPLSNDTYQWNVWN

VLMLVSLIVSLFVNSFLIVKLIRLRGRNQFTQSSSVPAIIEMFSNESDDEIIL

>CG5819-PA; CG5819-PA

MRIVLELALLVLGVISSCRAVNCPWPCRCTWVVDSLYADCSRRSLQTYPNFDGIPVEHLD

LSGNKFLEFPTLYADIDSLIYLDLSSNYISSIGAKTLIGFTSLRTLLLANNSIDSWESLS

PNEAFKYAPSLKRLGLDGNRLGSFGNGESFELLTSSSLTDLGLSSCGISSIGGDQMVNQL

PNLERLNLANNQLAQIAALPSRTLRVLDLSNCSIKNLSGFFLDAMQNLEALNLSRNTELQ

FDSLSEDPILTYMLRKLDVSYCNLDSIELSGLPQLTEVRLQGNLLRVVDVNTFANNSMLE

VVDLSQNVLRHIGQDAFAKLKRLKELNLAFNEIARLDRNFIRNNDVLVELNLSRNVLQKL

TKIVSNSVRTINMSWCEITSIESTALSSLSVIQKLDLSNNLITDMPTFMRSETLQQLNLA

NCRLTTVRNNTFREFPELADLHLNGNRLTSPIPPNYFDGNKFLDQLWLGDNPWICDCHSP

LFVDFYDYLTAKPAKIKDRNHLRCAAPAVFYGKLWEFACADVWILNARTSSTGEKAWSII

MLTLLGLGALVLGYACLQKYLRKRKVRQSDREYEENDDELRRIRDLNERILREEATPSLQ

HTQEISLLPSYEDALRMPKLVRPVKSMMDLSGPERARNSRKLRRSQTHADGDGSQSEAED

GLQLDSRQRFRSVEMLSNRDKERTAQYGPYRRTGYMEYNQSGSRRFSIEDSRFPAAHLKT

QNLQSAEQIGNFQSYENSPYTKRKPKIAEIPPFKRVNMMADSVEFLTDPEYDEVGSKHGS

PFAKRKPKPPVLPPPTMKVSAQVYTLQQSPVLELDPAIEDYFSAARKQPSSSTIASDFQE

LDEPELKSLPDDNRSSTISRSDVEQDLERGKRKKRKNSTSRRVSGSFTAANAAESSSSDS

ELNALVHKPMRETLF

>CG14351-PA; CG14351-PA

MRPGRTTTALPAATTPLLLLLPLILGRLHVAHAQCPWQRDVPDLQTSCICAYNLGRELSV

QCDQVDFSQLLAAMNTHARLKPVDLLYVNNSTISELPDAVFSNLSLHNLQLSSCGIQRIA

TGAFKGQESVLRNLNLQDNLLADVPVEALKVLGKLNLLDLSKNQLSHIPDDAFVGLTKLS

TLKLNDNNVTLASNAFRGLEQSLKNLNLKGTKQRKVPESIRGLKSLAFLDLSQNGIKELP

GAGGIRVFDGLDALTALNLERNLIQSIGETAFAGVRKTLSSLSLLNNLLAEFPIGAVHSL

KELRVLDIGFNLLTSLPEAAFRGNPGITLLALDGNPLSSVPEGAFAHLNATLRGLSLGGR

FLHCDCKLRWVAEWIRNGDLQVTSRERNPQFCGTPPRFRDRGFYSIQPEELSCPDIADAA

LRGPVGLADNLKPTLPSSPDSVEYETGTGTGTVGTGTAASAPVTSSVSSSTSTTTPTTTT

TTTTAEPTTRRSTTRPPTKSTTTISATTASPASNPPVNGTGTVQATSITTSSSSSSSSST

STGHGNGKQAPGWRQGVTNGNGNSGVGGAGGLHKPQRPPLVLGYPPQRGTRIDDANEVQV

KHAFRQDSSVIIQWDSDTANILGFRVVYRLFGEKAFKQGPPLESSEREFKIKNVPAQECI

IVCVISLEELHVTPETVPYQQCREVRTVASQASNMDKITIAASAAICGTIIVAVIVFIAA

SRRSRKLQSSQQKSPLPIGGLPVNCCGPTGSPGPLGSIATLSAFNNHKEWDQVSAYSGRS

IPRPRIYPVEQPDDMRSHFSGMPGKVGKSSRSLADGQSQHSFSNNSHRGYLGSAFPSNLV

NSRPELRQSRQSLAAASERMSRASYAGSIHGGNGGGGGLGGNGGGGGNGLPVSSLMAMSG

MVGGGGPASIASSARRSRPRSRSREQLNTTHIHNHRPGSRYSQAGSTHTLNNYCDTSDNW

TDHDMDIYMARNPTTRNGLGTEGRGRGRRQQQQQQQQQQQQQQQHQQLQHQQQQQHQHQL

QQLRNERHQQRHRNDQQWEQPYQPAQQQHQRLQYYMPQTERCTSPPDVVPRAANFHNSNT

TSTGHNDNSNSHGSSSSSSSNIAQQQQAPLAAMTAALGDATAAATAAAAAAASASGIVVE

NYFNEYERQEIIYHHPTSRRSSSSSSSCSAAATRHVPRSNAFRLQLPAVATATTNNILYN

ISNKHGATGHNGSIGNYKAHTFSDSCNCTCNCSLSEEEEISQETAAVTAAAGEDGDQAGS

SLSSTSVSSIEQVQQGQQEQQGQEVYLSLNLTLADAAAGADAAADAADASADRAEL

>CG15658-PA; CG15658-PA

MELHWQLLTFIVCLQLLHSAGFIIQSEVRKCTYGHIDKLLRIRCYDLDLKEVPQNLKSSV

EVLDLSHNRIRKLKTSSFQRYTDIKFLMLYDNMILSVEVGTFEPLTSLQEIDLSNNGLTT

IPLELFQLPRLRNLYIDSNELTSLNLQALEKPIRAPLEYLNVAGCELQELPDLGILPKLW

QLNASMNPLQNFRIDSLANMCHLQVIDLTKSQLSQCGCQQVTNHLMMLGASPKFVPVCLE

ALDIRECPLPYNRTIHSPTFASCQTTLQFAETRSFWLFGAGCFGGVCFVLLIVIFCVIHC

RRKRAQRRKRNAQKRKPFVISPRNAINNRQPEDEPLHCDIARK

>CG18480-PA; CG18480-PA

MALQCVTKPKMWLGLKQILLYVGLLLCVLLQVCRSKTQSQMFCPTVCHCDLHAQRNRAVC

SAKRLISANIEIPTTVELLDLSYNDITTIDDDSFKTTIHLLNLTLAHNAIHTLYGDAFVE

LTRLRYLDLSYNRLEQIDEHILESNNQLIHLNLEGNKLSTLGKGPILRSPSLRSLNLRNS

QVNQLGTQLLSALPQLRQLDLAQNLLLTLSPGDFHAPRNLASLNVEENPFNCDRALAKVA

TGLRQRGVAIFMSNCMEEEVQDHQDDAEAVNFPNKNEKFESMEYLEPSTRGPQSVLSVWR

DLDSSEEQNSSQDDEQMSSLSDVCEGSREKLCLRYRICLERVSHELLAGGNSQLEDEIIR

THTYDEDDLKLAFVVGGATGVCMVIFIITFALCLKSCCEMRKKKSNPEVATGVSAPLNPS

QDSLPTIHTWSPQRTRNPRRSNPQRPRSTQSHAVVRQPYGPQDNFVSRLFGRPARSQYYR

TINQNTATLIRRLSRSNLFTSRDREPSSPAEPPTSTARFYTDVIESGAIRPETPPPNYGD

VVVIENCDNK

>CG4781-PA; CG4781-PA

MARLEVIRGLTFALLLTQLLAAHVARAEVIDAEETDRFCYPESSKNSRRSCECSNVSASP

WGNRALRIDCSYKDYKVADLSLLLPLYIDSLDLSWNALDSVPIFTSDSLHQLNLRHNNIS

QLVSGNFKQLTSLRELYLGWNSIGKLESGSFDGLPHLQVLDLAHNNLHLLPGHLFAPLLV

LGTLDISWNRRFNESGGDLYTGLGVNWKLSTLRLDACSLNDLHLPVNAPLKELSLRRNQL

KRIPTQLPETLLRLDISDNLLEELLPEDTANLTQVRQLFIEDMPVLQRVVANSLTHVDVL

ETLSFQNSRQLSHLDAEAFGPIMTTPTKKRALRSLSFRGTMLRTFNSTLAPIFTQLAELD

LNGLPLQCDCELVWLKQLPVQTNGRCYKPARIRGMLVTSARGDAFSCDTWPRWAYGLVVL

SLIALSAAGIYLIVMGLRPHRGVTMRRKVGAGSPYARVTIEPNRQENPH

>CG5096-PA; CG5096-PA

MNLTKSATLAAAIFCIVLSQVSGQTKDETTTTETPKEKIKVIDSKLCKKCNCYIDTNLLD

CSEKLQDWLSAEDWEDLTNGNVVFKTINLEHNNLTSVPILPKYDVENLYLANNQIDSISV

GAFQNLTELVTLDLSHNRLTSKVLVPDVFKGPFTVQDFESLENLKTLNLGYNDLHSLDAD

LFEHIPHIEELVLCSNSFHVIDQLSETAISGLQSLKILDVSYMEIDDLPDTILHGPRDLE

IFIAAGNLFNQLPKALKYATNLTSLVLNENPIENLIGDNVFPPLTKLTHLSMTFMSKLYK

IGPGAFSELQSLTELILSDNKLLNEIDEEALSKNVTGGQYLDYPPLEKVYLNNCNVSTLP

KELLVRWDKLKALDLRFNPWNCDESNDFLINVLIDRINKTTPVLAKDVKCGGPNKLNDVT

LLRVANEHMIESSTGSLIWVGLLVVLLIAVPTIIGAYVMKRRGCFGVFRRHDSGASSALY

NRTSFNEDFHI

>CG7503-PA;Con

MLPHHQRHKMATLADSAICFLLLSLLLIGACLVTPTEGRAKDDRRTRGRGSSSGVLSSSS

SSSNNMNNGYYSGSSSTAGSSSGYVFTSSSAVNSGSTGYSGPMDSTGFCTRRRDMKLMCY

CTPDENHVPVQKAECWVFSEGLHQNDTTWTRFYQQKRLRELKFVIQNNARLDYIPTMIIE

PLKNLSSIVIEYSQVEIVKSYAFANLPFLERIILNNNHIMALDQDAFANHIRLRELNLEH

NQIFEMDRYAFRNLPLCERLFLNNNNISTLHEGLFADMARLTFLNLAHNQINVLTSEIFR

GLGNLNVLKLTRNNLNFIGDTVFAELWSLSELELDDNRIERISERALDGLNTLKTLNLRN

NLLKKIDNGLLRGTPALLSINVQANKLETLTFYTFQPIMDNLVNSTSELLVSDNKFICDC

RLQWIFELKNRTRHLQLRDSLEDLHCTLQEPKLSHFVDPVPPTILDVLNIGGFTAIGSNS

ASMGGVGNSVVGSSYSGLTMDDSRKHLGSRSRQALRGQRQFASSAENVVESKMRRRRKRQ

EEVKEKDLAAVAPAHKRYDYYDDNNGGMSLGHGLDLDDNLSLHKQGFYGAGSPVVGHNDV

DVLMTSHSASGDALDILTTKNAIYIKLFLLKPEMLPCHDELSDPTELPLSRDLMDVRSNV

GQDMSTAGANSLAQGMTIIVSLVALMMISRG

>CG7702-PA; CG7702-PA

MKHKLLLLFLAGAALLLATEVRSQHEDIPYQPVSNICQTCLCLSTQDVDHRTHFNLDCSV

RNFEHILARWPEQFGSQAIASGAASEIVVSYSGNRIKLLQQLPATNASLTLSCRHCGLQD

LQAPLFMDVPNVQALYISWNDITDDALVPDLFRGPFRNTRYEPIGLRDLDLSHNRIVRLD

RRLFEHTPHLTKLNLAYNKLSSLDEATTASIGSVATLQRLDLSHNGLMTLPAQLFSKLTS

LRFLDVSGNEFSTMPASLQLLGKSLVQLNLAGNAFLSLKENSLQGLVSLKRLNISSMPSL

RSLEKGALNLPALEHLDCSRNSKLERLELADLLSSRNLSQLDLSWNALTTLVINATGSSN

NSSNSTNETWPRLRRMSISGNPWYCSCELFKALELIGLNHIDREWDGTEARCETPYLLAG

SPLSNLTAERICKMVIPKKYREVDEEPPRFLRRHYIILTAIIASIVLVIGLVIGFVVVCV

RRRLKGSDYGVQPIRYTSVRGSNLSQFSQLQPVSVASKFNNVHAGSSSGVTTGAANA

>ZC262.3 (773aa)

MRKFVFFVVAILIQIHTTTSQRNRSSSPSGFLDLQLEKCPQVLGCRCVRDSTRNIQCFSI

DESKLLEIQKIYGSNIQRLELHNWQHDQLNFDIFAPFPQLEHIILRDGDLESLNGTVIHP

TLKVLSIENSELTSSSEVCRLLSIFPKIQSLSLSKNYFEKFECDTSNTKLKILDLSQNRI

SHLEVPNTLRVLNVSRNRLTSFENISTKLTDLDISFNKLSLWPSFDDWKFPNLRSLSAIK

LDLQTGFQLDAPLLNSLNIDGASLRYLNFHQILTPKLKKFSARYLTELRNIAGRLPSTVT

DVAFTDTMLRTLPADFIPMSSTNHMQKVSFDFSTNQLLCDKCLLQWSLPVYAQTSIRKDC

NLTREEIESASCKIGVVANDTGIQYGKYEKPTAISCFSYGVPSPKISWWRFRPAEKLGSY

DPITDEISYTNVSETMKESYEIQSGGSLLIRSPNRSHVERYVCVVENEYGKDYGIYHFRL

DYLDWYSYDVFNSVFWGGLATSLIVCLISFLLNITWILTRKSALWWIQRAERLSRVRKMV

EAMEKYRVRQMESLHEKYTKRVQIVRDNYHQQVEALRVSYASQQEKFQNYKAAQVDAVHS

HLDAMRDGYNNQLGRVREYGSKRAEQLWESYERQVNRMRTFSLQHRLKMMRQYKVKQRYV

NKLLESLQATSPEVQLENEEKVRAALEIPDDLATIDGSMDTPSRLSRSSSFHSLPEYVID

EQGNVRPGIIPTNAPSIRFTTKPTTSSISNEASTSSPSSSGAHRSPDSPPEKR

>CG7665; Fsh

MEKHPSLSQRMGTTYRPRKGLKCLSFEFQCRLLLHHLLLTSLSGRHFVYATSAVGGALSA

NNCHDIHHGFDVYPNLTAVSLAQSTDTPLTATMPRSAWKCCCWNASNQAEEVECRCEGDG

LNRVPQTLTLPIQRLTIASAGLPRLRHTGLKVYGSTLLDVAFTDCLQLELIQDGAFANLT

LLRTIYITNAPKLTFLSKDVFLGISDTVDIIRIINSGLTRVPDLGHLPPHNILQMIDLDN

NQITRIDSKSIKVKTAQLILTNNEISYVDDSAFFGSKIAKLSLKENKKLQMMHPNAFDGI

IDITELDLSSTSLVGLPSAGLQNIEALYIQNTHTLKTIPSIYNFRNLQRAYLTHSFHCCA

FQFPSRHDPQRHAQRMLEIEKWRKQCKSDSGTRKERSTLDNPFNMPEDFGSFGGTDDSAT

DITPITFASFDYMADDTMNKGTFHEKIILNPGDDSSAELCGNFTFRKPNIECYPMPNDLN

PCEDVMGYQWLRISVWIVVALAVVGNVAVLTVILSIRPESTPVPRFLMCHLAFADLCLGL

YLLLVACIDAHSMGEYFNFAYDWQYGLGCKVAGFLTVFASHLSVFTLTVITIERWLAITQ

AMYLNHRIKLRPAALIMLGGWIYSMLMSSLPLFGISNYSSTSICLPMENRDVYDTIYLIA

ILGSNGVAFSIIAVCYAQIYLSLGRETRQAHQNSPGELSVAKKMALLVFTNFACWSPIAF

FGLTALAGYPLINVTKSKILLVFFYPLNSCADPYLYAILTSQYRQDLFTLLSKLGLCQQS

ALKYKDSLSGQATTRFTIHGSIQRHSSLTCKMQTVMGAETQKMLKNSEDYV

>C50H2.1; Fshr-1

MTCSTYRSIQLFSNLLIFILIILYLQTIECQNALSLAQTKHSCMALKAEDGEGCTCKQTK

RYGQPECCCMGLIVSQLPTNLTADVGYLYLHNTSISVITPNFFDKFPSIRELEIDNSAHL

EHIDGSSLSVLSKLRKLSVVKCPNLREISGKLLVNNTRIQNVILKNNGLATMPSLRMTDA

HHVLLDRIDLSGNKIKFISDSKVRNVKARTVVLSENKLIEISGYAFTESQFLKLKLNNNP

DLRSLSVDAFKNMAGLQTLDLSHTSIDTLPINGLKKLKTLILNDVPTLKSLPSVLSFTDL

ETAHFTYPHHCCLFKYVDDVTMNDNGKYQRNAKEIHKRICDKREQQKVARRRKRETSGID

FLDMLLKEWTDNSTYTGPDDADDDELPPFVEIGAEPCQSIGEEVQKYYSNITCYPQPDAL

NPCENIVGYPFLRIAVWVVCLAAIVGNIIVWALLGIVYEKRMRMHYLYMINMSVADMVTG

IYLAVLAIADAKMSDEYYRHAVWWQTGWGCRAAGFLAVFASELGIISMFLIAFEMSYNTR

QSFRGRRLSPKVGVLLMIGGWLFAIIMAILPWFDVSSYSESSVCLPLRAATIFDKSYLIF

GLSFNFLAFAAMALSYGFIVKMLKENETREEDRALITKMTVLVVTDLICWFPTLFFGFTA

TIGFPLLSLSSAKFVLVFFFPINAFANPFLYVFFTEVIQHRVRSKTLPVIRRITTGPLNA

ASSLSNFYHSQPPGAHRRSRDEPTSPSGMHLAVTQTTSLNSTPRGSDVSRASDGMLFDYD

RRASASPRVSFDIPISPTTPRSDRKNRISLLKRIVSSIPEVSDLSEHSSESHHEHVPHPR

RKLRSSLNRILALGRRQEGSADSGRGSIASSAGSRDQNERVSLTSNASSILSPLLSPTNS

NILILPPEPSKNRRKSTPAIPLLVVSDCS

>ABI34171

mciahlpitf tlaillaias negaqgvesa trtaieairt gigtkpetei adateaeapv

61 revisllgii dgaesdilvp daddkcpggy fhcnttaqcv pqrancdgsv dcddasdevn

121 cvnevdakyw dhlyrkqpfg rhdnlrigec lwpnenfscp crgdeilcrf qqltdiperl

181 pqhdlatldl tgnnfetihe tffselpdvd slvlkfcsir eiashafdrl adnplrtlym

241 ddnklphlpe hffpegnqls ililarnhlh hlkrsdflnl qklqeldlrg nrignfeaev

301 farlpnlevl ylnenhlkrl dpdrfprtll nlhtlslayn qiediaantf pfprlrylfl

361 agnrlshird etfcnlsnlq glhlnenrie gfdleafacl knlssllltg nrfqtldsrv

421 lknltsldyi yfswfhlcsa amnvrvcdph gdgissklhl ldnqilrgsv wvmasiavvg

481 nllvllgryf yksrsnvehs lylrhlaasd flmgiyltli acadisfrge yikyeetwrh

541 sgvcafagfl stfscqsstl lltlvtwdrl msvtrplkpr dtekvrivlr llllwgisfg

601 laaapllpnp yfgshfygnn gvclslhihd pyakgweysa llfilvntls lifilfsyir

661 mlqairdsgg gmrsthsgre nvvatrfaii vttdcacwlp iivvklaals gceispdlya

721 wlavlvlpvn salnpvlytl ttaafkqqlr rychtlpscs lvnnetrsqt qtayesglsv

781 slahlgggvg ggsgrkrmsh rqmsyl

>CG31096; lgr3

MVYGRSIAVGFCLMTVVLLLAAVIFYLSLGPCPAASFACDNGTLCVPRRQMCDSRNDCAD

SSDENPVECGLLYGSKEIADKIVRNAIEKKQQRLISAVSNASGADSTTSMVPRNQSLTLN

MTCDIVTYPKACQCGQGTILYCGRYAKLRRFPRLSSEVTNLIIIRNNLTLRDNIFANFTR

LQKLTLKYNNISRVPLGSFSGLFHLERLELSHNNVSHLPHGVFLGLHSLQWLFLVNNHLH

HLPVEQLRFFRRLEWLVLSRNRLTLRNVQLPKIPTLYEVYLDFNRIEYIGEETFSQLDNL

HLLDLQHNLITHIHGRAFANLTNMRDIRLVGNPIKELSGETFLHNTRLEALSLALMPIHI

SSSLMEPLNISFLNLTGIRYDHIDFEAINSMRNLTYIIYDRFFYCSMTPRVRMCKPSTDG

VSSFQDLLSKPVLRYSAWVMATLTIAGNVLVLWGRFIYRDENVAVTMVIRNLALADMLMG

FYLVTIGVQDYRYRNEYYKVVLDWITSWQCTLIGTLAVSSSEVSMLILAFMSLERFLLIA

DPFRGHRSIGNRVMWLALICIWITGVGLAVAPVLLWRTSTLPYYGSYSGTCFPLHIHEAF

PMGWLYSAFVFLGVNLLLLVMIAMLYTALLISIWRTRSATPLTLLDCEFAVRFFFIVLTD

FLCWVPIIVMKIWVFFNYNISDDIYAWLVVFVLPLNSAVNPLLYTFTTPKYRNQIFLRGW

KKITSRKRAEAGNGNVATTTTGTATGSSQHPDDFTIFAKAAMRCH

>CG8355-PC; sli

MAAPSRTTLMPPPFRLQLRLLILPILLLLRHDAVHAEPYSGGFGSSAVSSGGLGSVGIHI

PGGGVGVITEARCPRVCSCTGLNVDCSHRGLTSVPRKISADVERLELQGNNLTVIYETDF

QRLTKLRMLQLTDNQIHTIERNSFQDLVSLERLRLNNNRLKAIPENFVTSSASLLRLDIS

NNVITTVGRRVFKGAQSLRSLQLDNNQITCLDEHAFKGLVELEILTLNNNNLTSLPHNIF

GGLGRLRALRLSDNPFACDCHLSWLSRFLRSATRLAPYTRCQSPSQLKGQNVADLHDQEF

KCSGLTEHAPMECGAENSCPHPCRCADGIVDCREKSLTSVPVTLPDDTTELRLEQNFITE

LPPKSFSSFRRLRRIDLSNNNISRIAHDALSGLKQLTTLVLYGNKIKDLPSGVFKGLGSL

QLLLLNANEISCIRKDAFRDLHSLSLLSLYDNNIQSLANGTFDAMKSIKTVHLAKNPFIC

DCNLRWLADYLHKNPIETSGARCESPKRMHRRRIESLREEKFKCSWDELRMKLSGECRMD

SDCPAMCHCEGTTVDCTGRGLKEIPRDIPLHTTELLLNDNELGRISSDGLFGRLPHLVKL

ELKRNQLTGIEPNAFEGASHIQELQLGENKIKEISNKMFLGLHQLKTLNLYDNQISCVMP

GSFEHLNSLTSLNLASNPFNCNCHLAWFAEWLRKKSLNGGAARCGAPSKVRDVQIKDLPH

SEFKCSSENSEGCLGDGYCPPSCTCTGTVVRCSRNQLKEIPRGIPAETSELYLESNEIEQ

IHYERIRHLRSLTRLDLSNNQITILSNYTFANLTKLSTLIISYNKLQCLQRHALSGLNNL

RVLSLHGNRISMLPEGSFEDLKSLTHIALGSNPLYCDCGLKWFSDWIKLDYVEPGIARCA

EPEQMKDKLILSTPSSSFVCRGRVRNDILAKCNACFEQPCQNQAQCVALPQREYQCLCQP

GYHGKHCEFMIDACYGNPCRNNATCTVLEEGRFSCQCAPGYTGARCETNIDDCLGEIKCQ

NNATCIDGVESYKCECQPGFSGEFCDTKIQFCSPEFNPCANGAKCMDHFTHYSCDCQAGF

HGTNCTDNIDDCQNHMCQNGGTCVDGINDYQCRCPDDYTGKYCEGHNMISMMYPQTSPCQ

NHECKHGVCFQPNAQGSDYLCRCHPGYTGKWCEYLTSISFVHNNSFVELEPLRTRPEANV

TIVFSSAEQNGILMYDGQDAHLAVELFNGRIRVSYDVGNHPVSTMYSFEMVADGKYHAVE

LLAIKKNFTLRVDRGLARSIINEGSNDYLKLTTPMFLGGLPVDPAQQAYKNWQIRNLTSF

KGCMKEVWINHKLVDFGNAQRQQKITPGCALLEGEQQEEEDDEQDFMDETPHIKEEPVDP

CLENKCRRGSRCVPNSNARDGYQCKCKHGQRGRYCDQGEGSTEPPTVTAASTCRKEQVRE

YYTENDCRSRQPLKYAKCVGGCGNQCCAAKIVRRRKVRMVCSNNRKYIKNLDIVRKCGCT

KKCY

>F40E10.4; Slt-1

MLICFIFILLIPESATCPAECVCVDRTVSCVGQQLTEVPQNIPNDTIRLDLQDNEITKIG

PNDFSSLMNLKALQLMDNQIVTIHNQSFSSLVFLQKLRLSRNRIRHLPDNVFQNNLKLTH

LDLSENDITVVSDAQLQGPEFLEVLNLDKNHIFCLENNVISSWVSLEVLTLNGNRLTTFE

EPSNARFRQLDLFNNPWNCDCRLRWMRKWLEKAEGQNKTVCATPLNLQGSSIEILQDKFM

TCSGNRKRRYKKTCETAEICPLPCTCTGTTVDCRDSGLTYVPTNLPPSTTEIRLEQNQIS

SIPSHSFKNLKNLTRLDLSKNIITEIQPKAFLGLHNLHTLVLYGNNITDLKSDTFEGLGS

LQLLLLNANQLTCIRRGTFDHVPKLSMLSLYDNDIKSISEVTFQNLTSLSTLHLAKNPLI

CDCNLQWLAQINLQKNIETSGARCEQPKRLRKKKFATLPPNKFKCKGSESFVSMYADSCF

IDSICPTQCDCYGTTVDCNKRGLNTIPTSIPRFATQLLLSGNNISTVDLNSNIHVLENLE

VLDLSNNHITFINDKSFEKLSKLRELRLNDNKLHHFSSMVLDEQSNLEILDLSGNNIQCF

SSIFFNKATRIREIKVIGNDLLCDCRILPLMSWLRSNSSHSIDIPPCQQFQYSDNESDKQ

RCAAFPEETCSDDSNLCPPKCSCLDRVVRCSNKNLTSFPSRIPFDTTELYLDANYINEIP

AHDLNRLYSLTKLDLSHNRLISLENNTFSNLTRLSTLIISYNKLRCLQPLAFNGLNALRI

LSLHGNDISFLPQSAFSNLTSITHIAVGSNSLYCDCNMAWFSKWIKSKFIEAGIARCEYP

NTVSNQLLLTAQPYQFTCDSKVPTKLATKCDLCLNSPCKNNAICETTSSRKYTCNCTPGF

YGVHCENQIDACYGSPCLNNATCKVAQAGRFNCYCNKGFEGDYCEKNIDDCVNSKCENGG

KCVDLINSYRCDCPMEYEGKHCEDKLEYCTKKLNPCENNGKCIPINGSYSCMCSPGFTGN

NCETNIDDCKNVECQNGGSCVDGILSYDCLCRPGYAGQYCEIPPMMDMEYQKTDACQQSA

CGQGECVASQNSSDFTCKCHEGFSGPSCDRQMSVGFKNPGAYLALDPLASDGTITMTLRT

TSKIGILLYYGDDHFVSAELYDGRVKLVYYIGNFPASHMYSSVKVNDGLPHRISIRTSER

KCFLQIDKNPVQIVENSGKSDQLITKGKEMLYIGGLPIEKSQDAKRRFHVKNSESLKGCI

SSITINEVPINLQQALENVNTEQSCSATVNFCAGIDCGNGKCTNNALSPKGYMCQCDSHF

SGEHCDEKRIKCDKQKFRRHHIENECRSVDRIKIAECNGYCGGEQNCCTAVKKKQRKVKM

ICKNGTTKISTVHIIRQCQCEPTKSVLSEK

>CG7509-PA;

MSLGVWQLVSLVLLMDPAEVITGPSQAKEPPIQSLDEFRQRYLLPLISSDTRNCSLNACE

SLGIVSQLLMLINSMPNGTQSAANDKKPPKSKANGHNDGDVNGGEINAMSHLANFDLVKR

VRQIESRLRSVEQPVWHLATGSQIEWNHCTSGVCRCNPDTKSFTCWNTNLKSVPVTQVIP

MNMVNIDLSRNILSTLHKDTFRGLTVLKELDISHNVLDFLPFDLFQDLDSLLVLRIQNNQ

LEDIDHRTFWKLRNLNILDLSKNEIGMLPESIFYHAQRLTVINMCDNQIQNFPPNLLRDQ

LMLEELDMSRNKISELSSGSIRYLTKLKTLDFGWNQIAKIDDDFFAGLRSLRTLSLHNNR

ISSLSGTIFNNLANLVTLDLTTNRISHIDGNAFVELNNLNELFLGQNSMSSIPADLFLNV

SALTRLTLFSNNLTTLEADDFQGLNNLKILLLNNNILKNFDARAFEPLSQLEKLRIDSNK

LMFLPHGALHGLKNLVAVKLDKNPWHCDCRALYLARWIREFVLKLWDGQQPMCRGPGDLG

GHEVGLLRYDDLCDGQWASMLSLSPRLPVRKHQISTPMNYTDYFNLYLKHIYNGTTDEEL

KEADITSVSIKKVHN

>CG10824-PA;

MYVRNCLPFLCSYLLISLAATATTDEDADADVDSGSSRIVLVSSLEGHCQTEGKVTSCRG

FEFAGEDEKATFDLPTEVRIAEDGTTYEVGDEEHSRTLIFENCTFTNFPLRLFYTLEVSE

LDMRGCGIRFIYWENFSIGADKLVILLLSDNHIEVLPTKTFRGAGNLEFIFLNRNKLGKL

QAGAFDNLLKLQYLDLTENRLEALAADVFAGLKSLRHVGLAGNQLTTIESDLFAHNPDLL

SVAMQNNRLREVGEYAFRSRGRHHQMQYVDLSNNPELVVLLLNINATNLTARNCSLDRVN

LYGSVTNVDLSDNRVRELYFPASEALEHLVLRNNSLVQLASLSRVPRLRHLDVADNPNLG

QLPDGWRTPHLEMLVLRNTGQMELPLEALQGMQNLQKLDISGNNLTEIDPSAFPTLTQLT

HFYIHGNNWNCFSLRNIMDVLIRANGIAYTVDNYDPDFPGEYFHGIACMYRLPEKEGVDS

SSSSEISASVESSPITSSSDPSEVDKLRDELKAVVQHFDSKFDLIFSKLAQLNEQIQAFE

VLNKTVWSQVTLSV

>CG6959;

MILVVAIAAIILAIGAGGVLGENSSSCGPNFPAACSCGQEMYESEMQYVVNCTNAGLVNT

SVLEFMPEQVEVLIFTGNRITELPWNVFGSINNYKQLRIVDMSSNHIREIRGKSYHHVPR

VERLILNHNNLSISRYEDEVNHHHPRVFSNFINLQSLHLTDAFEDNSSPQLSEDLHDIFV

NSQLVKLQKLHLEQNEITHFKDRNVFCDLPSLRDLHLGDNDLRDLNFEVRCLNNLRFLDL

ERNKFSFVKPTDLRVLNELEERPNRTANLIVDFNLNPFGCDCRTAPFRAWITTTRVTVRN

KDSLMCFHSTGHVDAEPAHLLQMDMGQCADAIAAAATILNTAEDEPHYGDPEASHLQPQA

HISGHTATLIFLLIVLTMILLGLLVALVYVSRDKLKYMITPVFDNVAKKVQYTSIKDEDC

PEVHV

>CG11910-PA;

MAVLWWLLSLQVVLILPPSYHLATPSSGSGQLRRLWLQDHCSAGICTNVVIGRSDYVILS

QAPIGGTTMLTFLNSSIAKIPHLLFDTFPDLQVLRMENCSLETFEKPQFEGASNLMSLFL

GYNRLKDIPKNIFLGADNLATLHLQGNQLKQLGNHSFHALKEVKELSLAENQLEQISLGV

FSGMRKLMDLNLAGNRLDALPRGVFDRNLNLTKLNLARNRFTAFESELLKLQPVFTQLDI

SGNIFQELTLNFTMLDVAIAHSCDLRRLTVYGVIHELDLHNNSLREMPHIPLAANVSSLD

LSHNPLGNLQGNPLRRFTSLLRLNLSATGAHELPEGLFKKQSHLQMLDISGNSIYSLKIT

IFDSLKALQYFYFQQNNWNCDFLQLLMSSFVKRKDISFMEDITAPELVDDYVDGIACWYE

SDKQSKKCESGGSDAAMELSVVRNEIKTFTELVEKKFVKVYRMLEEMKMKL

>CG5810-PA;

MRMLNYLNYILIAVIAFATCESQKLEEIAIDSLDCRENTCTNLKYPSASAVAYFSENVTK

HLRKYETLVLHSSDLANLPRKIFLNLPQLVEFHVLECELQQIESVCFDGAKNLKRLNFGG

NALRVLDSNTFELATQLEELNLSDNQLEDLPTTIFRPLKNLQKINLSNNRLITLSQHIFS

QLGSLKSINVDSNQLVELPGELFRDQRKHLSEFSAQSNQLVRIPFNIFREIDHLSLSFNP

QLRRLHLSAKINELEATNCDLESVELDGRVIGVQLEANPKLHELKISQPQDLEHLYLANT

NLYRLDFLSKASKLVDLDVTDIVNLADLPKITSAKGLERLSFTYDNLTSNHMDMLPHLKD

LNYLEISHEKGKEIFIKDLDEDFFVEEAELNCGQLADLLEFVELPKDTTILEDRLVGDPR

GPMRCGMA

>AAV36870; Fili

1 mpdhtrvtat trsrpwlcga ipvllllllt lvilppetta fcpskcqclg geansralcv

61 daaledvpiq lnpetkyinl tvnrirtlef slpfymklei ldlsqniiet lgsknfeyqs

121 elrtlnlsrn lvsslhkhaf kgltnlllld lsfnrietvh ptalsdlasl veldltnnni

181 vsledncfkg mntlevlvfr nnrlldvpas nlwhlhalks ldmslnlvef vrndsfeglk

241 ellalsvqgn vmseldlsaf eglislkhld lsdnnltmvp tqqlsklsnl tylnlggnrf

301 sqlpavafln lfhlrelhls rldflqrids rafvdnthlq tlhlnnnpql sdipmrlfqg

361 npnilevymq snslqtlysa qfpvdqlqkl ylgdnplqcn csllwlwrlv tgnfegvdpg

421 mehaaggava alakeaddee ladeatavas tddgvaalaa yiaeqhivna lhttepsaye

481 latsssnrns gilrmdrqqi gcdiwrdkvr trrklltmse geitcpahiv tvvcavitcl

541 lvamigisvl yylrfvkrrr kllhergpmr tsksiinvhd rilqghnpgg lglgmsmtlg

601 ggnhvnglgm tlnyphhaqt lqahhhyhqa mplqshggng nheyqqttlp qldklelery

661 laaqtianey ralkpwelpv keaddepehl yerfdhyeyp dthtmsklkq aaslnhsnss

721 agpspvppss gkphvvyv

>CG5541;

MRNIRNLKDPTTWTRILLPFLSCLLLAVQADLVNVTCGEYGNTITCDCNNSDQTMALPSL

RGNVYMIEIRNCRDLLVEANRLANTMGLHKVVFRQVGQLVLREHALSVPRYASNKALIVE

FEQTNLKLIESHAINGNIEEISFVGGRIELMKPFGFTTTKDSAILLKLDGVTIQRIESQA

FKKFAVEQMSIANCQFLGDLPTRAFYELEVTNELSLRGNQFQEVHSHAFSFKLVSKLSLS

DNHFLSVDGEWLEAQIRDAATIRGNEFGATSEIAFRSLTVHRSYQLSERLELRFHNNSLR

SSRPGADPRVDGTAENVAPQPLQFDNSFALNVRDIRYNNAWSCEQLDRNVEPPLPRAEFF

RLHSDQLMFHPPDFGGQGQIQPYIPLRSLITDECRERSYMAFIISGSVLLGLLLLLLILL

LWWRVVQKRRRRKLDVVQPEPRTYKETQIVYQIENAGLLKTDL

>CG10148-PA;

MVKIVNQHCDLGLALLLAWTWLTRLVVAAHLVDIPTSSRLAAEREEQQLSRQDVGRLSYQ

SIHRMLRDENEPDSFRGELRYQQKRHKRELELNAPANKLNLTHRDLRTFNSTGGQWKGDF

QVITAMDLSSNQLESLSLDNFNQLRQLDLGNNSLEVIPLSLADTNMSLPFVTLDLSCNKF

SQISTSFFAQRLPQLKNLNLAHNELLNISRESFYNLLELQTLVLSHNNISDIDYETFLAL

PNLQYLDLSHNRLSGSAIRALQGIPDLVSLSIAYNPDVGVAMQEFVASWSLKELDASGTG

LCQVPAALAQSVRTLKLSDNWLKVKVIQK

>CG11136-PA;

MLLLLCTLAAANDLKDLLEDSTTSSSTSTTSSTIPSSVTSPTTPTAYVVSAAAVPALVAG

RGKSKSTSGKYVNKSSPRKRKAEQVSSLPLPVDDALMEWKCPNITGTRNAELECGCDLPH

TLRCNIDLHGMMLLADRLRTSPYSISLLDCSLRNVTFLSDAKIFDNVSLHGLVISSGEIK

RVHKSAFLGIRGPLQALGLPGNALMSVPWNALSTLSALERLDLANNKIKALGTADFVGLT

SLVYLELSNNQISSISQRTFVNLRKLEVLKLGGNRLGDYAQSLRSLSQCLSLRQLDLQAN

NLNGPLSEQTLPGMRNLESLNLNRNLIKSIQNKALANFSRLVSLSLRHNQIDVLQDHAFF

GLGALDSLDLSYNGIVAISSASLQHLSRLTVLDLTHNFLRALTSDLIAPLPSLRELRLAG

NDISIVARNAMDGARELESLQMQENPLSCDCSIRPFAEWLQESQLHSSLSASCVTPPRLE

GAPLLQVPVETLSCDMDNVEKDNANIMQHLETLAKPNQTSPIKDLSEEIILHELHFSTDY

GLILTWLLNLSKKDYMCDAIFVYKEEHINEILIDNSPIHCESKVVNGQNTVSVIVPDSSS

LEIGESYRFCLVMIQEQKPDSELNIGCSNITRLERSSPGAVPVSRQYQRRPYYNANELKP

EVVHDAGEDYQVNQRRFNSVVGSQPQQTQQSTLLHSYTVIDSLNKSFLPGLGLGVLVTSV

LVLIWGATRIRQTGGSSGGGGRNGDSIDSNSSSMHNNNNNSRPGTPTATTCYAASDHIAR

LADAENGTRYLKLQATTSL

>CG14662-PA;

MFKSKSFDLVIEEKTKKPERLYQPRRMRWLKYIILPAVFSFALLLILVNVDFSDNSEDST

HLGNDTSLIISGYGFENNTLRRGFFSGGIALHSLVIENCTIVHINDAAFNQESTVNITSL

QLINVQLENLTESALEGLQKLQNFTLVNENNHFRPFGFLSAVAESLVSAEIHQSLAAAIS

YSVCDFLGSRNFPQLKYLDLSGTHLDKSLIKESFDNLPALEQLLLRNCGLGNIEWEIVRP

RLKLLHYLDLGGAQKTGNYEHQLDVSAFSPETTTNAEEISTILAKRAMAPEVVGTTTLGP

TTSIEISPPSTQSTTTPKEESTSMTETTILTTPSPKCEEELCQDLECSRITTDTVASADL

GKSSCQDGLLVEICESTCTTPTFFCVILGENFTSASNCCSHHTMRCVVSAQVSWFEDHSG

LVIGLGVGLLFIGSFLGMLIVFGTLRLNPSWLRGNKRRESNTIGLIQGRFEKDPYEQVGG

PIATIDNNEYVTAYHRYLEQANHRPTESNKYIRPPRDRAPSVPPSSDYPLPLPARNCNVY

ESCELYEELP

>CG14762-PA;

MAGIQLQSGCTMVVTVVLLLQVLVMDQVLGQGPPQTQVCPEQSEIAPCICTVKKNGLDIL

CETTDLAHITKSMGTLKGKSPIIFYLKLRHNNLPKLQGFVFLALDIRHLTIHNSSLAAIE

ENALSSLGAGLTQLDVSLNQMKTVPSQALQHLFHLLILNLNHNKITVIHNNAFEGLETLE

ILTLYENKITQIDPEAFRGLEDHIKRLNLGGNDLTNIPQKALSILSTLKKLEIQENKIRT

ISEGDFEGLQSLDSLILAHNMITTVPANVFSHLTLLNSLELEGNKISVIDKDAFKGLEEN

LQYLRLGDNQIHTIPSEALRPLHRLRHLDLRNNNINVLAEDAFTGFGDSLTFLNLQKNDI

KVLPSLLFENLNSLETLNLQNNKLQRIPQDIMEPVIDTLRIIDITDNPLNCSCELTWFPK

LLEDLKNKDDEMSQKKKPLCHMSLDNREYFVQAMPTEKMHCAGLNIIGNP

>CG17667-PA;

MHVYYKFGLLLIFLLSVSISHTNAAPAGSEEANPLEDFNYGDDDYSETATGEESPETAEN

RLMPQSSSTSTTTTFRPIFNIPRRSNHVLEPSCPRNCLCLEDFKFVQCANAHLTHVPLDM

PKTAAIIDLSHNVIAELRPEDFANLSRAVEINLNHNLISSIDKDVFQGSERLKRLRLANN

RLTKIDPDTFAAAKELTLLDLSNNTITQRLDGSFLNQPDLVEFSCVNCSWTELPEQTFQN

MSGLEVLRLNKNDFKQQINTKAFSPLTKIIKLKLPELEQQNIEELCSLLTSIDTISFLNY

DISCYEFVLGTPFNGSLIYPTEPPLKGITNPPIVASITSTAKPVTATPAPPRSANRNRGK

MDNSTELVKAGILSSETSTSGVSVEPPAESQTNQVQISQEAINTLLICIMVLAVIGIVIG

LICRQDIGGIKTKCCRTSKPEPKDQVHPTEEIPLNKLA

>CG3095-PA;hfw

MLAYTHGTWLLLLLLLVAGACARPEIAHTPDPAALGEESTPHAHAHPQARHHHHAHPHAP

LKEDEQGTAIPAPILTTDNVGIGETTTASSLAETQSMSDPGSVTDTTSTSTSHSTSTTST

TSPAPLPPAAPEQPEYLKHCFYAEEQLCGHTFDGRAETSEGQGSTVAQSEAQNRGGQGNS

QCQCREHPTRPNSWYCCNISQLTMISSCSNISKWTNLHVRNMTVEDMDLSNPIFRSLQSL

AVTDGNITRLVNAFPRLSALKCLNISNNNISEIHSRAVKDVPHLEFFGMSNNNLSLVPHR

NQNKNITLDISGNMRMLCTPLNEIIYTESINFLNPKHSYCQYNATHTWFQSTDKVSVEQL

ENRKRCVTNCPVIPNYGSCNCTLENIMIIQDNQSKPQCHVDCSNLGLVELPQRLPDNTFM

LNITNNKITSLGDYFHTNPTYHNINRLLADNNQISSIYEFEGTKFIETFQRIYMRNNSLS

KIPEYFLNNALMDSGLGRRIYLAGNKLQCDCNSAKTLQNWLKERSSDIPDYMEIRCRNMP

QRVIELQEAKLCQSPPDWTDYIYYLIAAEVLLLLALITKVSYDYWVFKTAGYLPWPASKM

PKLPCDWLCES

>CG32372-PA;

MTSLVYTVFHLASLQCSVRPEISPCTCETGKAWNHVELSCEKLESFNAVVDSLANKLNAD

TNIDLKITHSQLDDLEMRSFTDMNFNLYKLRMQWNSLKSLPEVPFRGLSNVTYLSIGDND

LDEIPKHALSHMPSLLTLDIGRCKIRAVQQEDFRGIQRVTNLILVSNIITRLDRGSFPKS

LLILHLGRNQLESLNGSLHDLHNLESLFINANNITSLDDELPDGGQLRLLMAHNNRLERL

PANMAGMHSLETVHIHCNQLRSFDRVLRNAVNLSEVMADNNELEYLAQDEFASCSKVETL

QMGCNHIKSLNSSLLPILKLKNANFSFNDIEEFSMAELHGLRSLKTLQLSSNRIQRLLPD

PRGVQELMLVNLDLDNNRIDSLNGALAGLGNLRILNLAGNRLEHLQVGDFDGMIRLDILD

LTGNQLAELKPLEMTLLPSLKILKVAYNNITKLEQDFKGLPVLCQANLTNNQISTISSEL

VTNTRCKNHNVPGKLEIHLDDNPIMCDVGLNELCRLMAVQEARIRGRSQCFENDQEVCTV

LPMLYNVNLPIMVTNLKLTGREVPKPMVRVIVPSLIKANNELLPPLIATLGNPVLISTDL

VNPVISPLPPPLLLATTTPPPPLPLPVESEVEKNESTTANPVPTNYTQETTTTTSTTTTT

TTTETSSQVAPIEIITTTAPTTTTTSTTTPKPNETLPVIVELQDPNPPDTTVNQTALVPP

VVLDPLQQDLERERERERERELERDLDHEAVAKTEYETVEYIPNLVQPPVGSDALTPPPS

KANALEEDSESVHSNSVHAEYPHAAQSLQIPEEPPEE

>CG3413-PA;wdp

MERVHLTAWLALFLIVVANATPTPARTPTGCPADCTCSLSQHTHKPLYHLKCNSTRGLRL

TEKTFQSTVPVHSIDLSHLNLTRLSHLLDKLPELTSADLSHNQLKDLGHLGKGLKRLNLK

HNQLTSDKLRKLPQHLQVLNLQHNNITHLPLELTHMHQLHQLELSHNAINCSCQTLEVRN

WLVERIVYMEHPVVCSYPLEFRGRSWLQLKQDEICKKEKYQWFDTEENELMMGDQPAAVS

AEREDEEELGKDFLPIVGNPAATAKKVRSPQIPLPSDQVEGSGDLSETNMELKLPEETVA

EPEAAESQLVDAAASPSVLEEHIVKDEDEDDEGSGSGGGLLIIPDPSKVKITSEDDIDSD

GKPEESDVRPLENPENSENPDTVFSNKIGIYEGDQEEKKPVEEDNIVPVVMTNLDTGLES

DVVTDGPLDSSKESEDILTAKIGKPKDDSSAIYYLLAVIGLIVVGLVLFVAIKRCKYDSN

AAARDAEAQRQTELLDMDKKQLGKPLHKNGHGNGQEHSPLIGEKTKLDEAQIVKKPYENG

EAKDGAGQQPLLNGNGSANGGTKEAPETGEPAAHEYYPITPRYPTPQSPRASKYAQQQQL

AEQNNNEPDGAYLPSSPKSGRYSPVYSPETGRVKIKLTETPKPKTPMLVTRSKSNAGDII

TTPVRPIEPTHQVINGH

>CG5888-PA

MWWLLLLLSLIAPSWQENATVINPSYVTKAGCEALVKEPNLCECQEQEIKCENLQLHSDN

LQLYGISCSIFDARGFGYIPPLKVGNIGSLIVQNCAIPNAESIKYLLSKLGVSNYTELEI

FNYFDPKKTDRGEVLQQHYTDHESLKKVSIIGFKSTLPENFLENLPALQQLSLKGSSDLP

GNILHPLKNLTHLEIVVKNLGKVSGAIFAKQSKLKHLMIDCDAKNSSVEMSAFGPQELWH

MTELQSIELFNCGDNVPTELFWMSEQLAYIGIRSNISYLSKDFLKVQKKLLTLRLERNNI

ARLPDQLFRNTPLILEIHLAFNNLDRIQSGLFDKLKNLQVLNLEHNPITTIALNAFTPIP

TAHIYVGKLFKAAKNADWARSTNATICEEEYIYGVCIYCKRDEYLDHFADSENCNKPNPK

AKDVLAKKAYENQLKAMPTHSWKKAKEYPVEEEQP

>CG8852-PA;

MGSSHFSVAMTQCVFLVVVSQILSHVRAAGDAMSSANESISLPPACVIRATLYKTSSSER

SQNDFGLPLAIDCSENSSMGMNYFDLGAQRVAGKRHVSLDGFQTPPLGISSYGLEYLDNC

VDLESVEIQRFVGDATLKLSCGGALIPNLTAVSFRYNELGTLSGETIQDLPHLKILHLQQ

NSLRYLEYFKEHSDLEELLIQDERDLVLRFNEILNQLPELRRLSLRNVEKIEDQFLRILP

ENLTDLIIENTPIQPGVLYLTEGVAKLVNVTITNCQLKGFALQPAHNLDIMYLNLSGNAI

SSLNISFESGPSTLLTLDLSRNRLEHLNFTWFYRTTSLRSLHLQENHFHSLSLFQLGFIS

SASIHHIDLRSNELMSFRDTENADLPSWNPQLRISIDDNPWSCQWMLNFSHMQPQLFRLF

QYSKYISHININGLSCTPQEPPSEEPPKVRRIMHVRINGTDSSIPVPHPHGNVSSFTVLY

GNPVELHRSQRSLALIIVFMLPLGIAFLFLLLYLYLHCERLFHLSYYASGLPCFGGEKSS

SGPRFVDHVDIVRYPIANGGSSCPVDVETELPDGYETPVSGGTSICNCTGHRESTCSRTH

HVTYESLPAELPYQLYAEIKEQADENGDADEVLAVTTGPTAPIYDHLSFVEEMPKLDELR

DIS

>CG1504;

MQYKVPVWVLCNTGGLEQIPLRQLPATVENLALTKNNFPIIKPDSFAGLRALKKLSLDGN

NITRIKQFAFRGLPRLKELSIQYTPLQMVAQFAFAGLQNLSTILLSHNQIQRIEGNAFAG

TSNIKLILLTNNPLIRIDSSAFSSLTNVGHLILPSGIRSIEQDAFFGMDTVGLLKLAYMD

LKEVAPFTFRGLSNVLLLTLQESDLGVICADAFTGLTQVETLQILNNKIDSIEELNFTST

AAIKHLKFFGNHVLETPDPNSIIVDGVEHLQLVSNHFPCGCHIHTLLDGPLAEGAHNLTD

FLQKNYCISPLEVNGRVMSELDIDSIGRCSDQLTKGNLGSSAASLALGINSMLLIAVASC

AEWRHVRLLANRLAQLLGHVAWRRRRKRRKGN

>T22E7.1a;

MIEKVLVVVIIGVVGAYAQGSCRTDQHEMTCRGKNSLHDLKKDQDYLEVDTLNVHQAKLD

LTDDLVPDGINLSHLTLLNATDNQITRIGRRGFDKIRNMQFLYLSINQISHSQPDPFQAL

EKLKRLEMNDALDGNMEEKSDMLRNFFHSKNSFVHLAQIELNKNKIEGIYPKTFCGIQGL

QRLELSNNRIPSFDFARTCLKELKALMLAGNLIQKIPADIWDFLPSLSSLDISNNPIDCD

CATIRLLSGDDVVFLNQADTKCASPPELEGKRIFELSRDYCKTTRNPRGKASFFQFLVLF

VIAVGILWLYKKYRERTRHMSSVPVGYTNLQHEQAVEPEFV

>Y71F9B.8;

MRPPVLILLVLVSGVISCQSGCKCPTKTTAVCKGSSLRSIPILLDPRTTVLDLSNNRISR

LSADELSLYPNLEQLILHNNSITHLSADVFSTLPSLRVLDLSSNSLLSLPNEVFSKLKNL

KTLIISSNDVQLGPECFAGLSQLQTLSIADNRLSFLPPSVLKPLSGLRNLDLSANKLLSM

PASVMNNLGGLETLKLKQNLLSSLETGMFLSQKELKHLDVSENLIGDIEEGALYGLEKLE

TLNLTNNQLVRLPGNTWSLPALKTLDLSSNLFVSLETASFDGLPALQYLNISHSRNLKTI

QMATFVQLSSLHWLSISSSALTHIHPSAFNPIPPLSHLDLSNNELRYVAPGMLQWPNIRN

LHLANNDWHCSCDLRVSNLNPRDDAKCSGPENLAGAPINELSSCSILGGLLIPFLLVLFI

LLLALVILALACKKSKPVSLKNRAFYNDQLIAALNSHKEYSFDCHSPYTMSSEDSRDSAY

ESPTSALMPRRPPPSCPPPPRLLTLPRAGPTHVAPPMVPVPNFRNSNDPYLIPKSQVPIT

RL

>Y39A1A.7

MTSITSAFLLLLLFVAVACRSVPCLDGCTCELSEHDPVIRCDGVGLNRFPLPHSSPLRGF

HFLALTCNDIDTIPAISMIKASFPDLQGIDVQGNTRLNCSDLVHLHKDIPVLSDCDNEKP

LQCDNLDKNCDWKCRTLTKLKEMWAQFKDLVNRKAKEWQAEETIEAAKSWFSAQFKKFKI

AIGELSD
