# Supplementary material for: The extracellular Leucine-Rich Repeat superfamily; a comparative survey and analysis of evolutionary relationships and expression patterns
Source: BMC Genomics. 2007 Sep 14;8:320. doi: 10.1186/1471-2164-8-320 (PMC2235866; doi:10.1186/1471-2164-8-320)
Supplement: Additional file 2 — LRRscan_out.html. Graphical comparison of HMMpfam and LRRscan results. A compressed archive (lrr_plots.tar.gz) containing 372 images in Portable Network Graphics (PNG) format, an information file (00README.txt) and two HTML-formatted pages, one with output from LRRscan (LRRscan_out.html) and one that links all the images together (00plots.html). After downloading, the archive must be to uncompressed and unpacked. Most modern operating systems (e.g. Windows XP, Mac OS X) will do this automatically when double-clicking on the file. Alternatively, you can use the free tool 'Stuffit Expander' () or your favourite unpacker. On Linux or Unix systems apply the following command: tar zxf lrr_plots.tar.gz. Please note that some browsers might uncompress the file during download without changing the file ending. If you have trouble unpacking the file try renaming it to lrr_plots.tar and double-click on it again. Unpacking the archive creates a new folder (lrr_plots) in which you can find a file called '00plots.html'. Open this file in a web-browser, either by double-clicking onto it or by using the 'File->Open File' menu (or equivalent) of your browser. This will bring up a web-page with plots of LRR motifs for 372 proteins. If you click on an image you can see the text output from LRRscan in a new window. [file 1471-2164-8-320-S2.gz › lrr_plots/00plots.html]

# LRR plots

Graphical presentation of LRR motif predictions generated by LRRscan and hmmpfam (using Pfam and Smart databases).

Each box represents a motif, yellow for LRRs and red for LRRNT or LRRCT. Within each box the name of the motif is printed followed by the score. The second line contains the start and end position of the motif within the sequence.

The images are presented in alphabetical order of the sequence IDs.

Please note: Overlapping motifs are plotted on top of each other, which in some cases leads to cluttered output. A click on the image opens the according LRRscan output in a different browser window.

1. AAH11057  
  
2. AAV36870  
  
3. AAZ20639  
  
4. ABI34171  
  
5. AK004926.1  
  
6. AY182026  
  
7. AY182027  
  
8. AY182028  
  
9. AY182029  
  
10. AY182030  
  
11. AY182031  
  
12. BC030471  
  
13. BC036337  
  
14. BC043099  
  
15. BC058381  
  
16. BC104037.1  
  
17. C02C6.3  
  
18. C07F11.1  
  
19. C41C4.3  
  
20. C44H4.1  
  
21. C44H4.2  
  
22. C44H4.3  
  
23. C50H2.1  
  
24. C56E6.6  
  
25. CG10148-PA  
  
26. CG10824-PA  
  
27. CG11136-PA  
  
28. CG11280-PA  
  
29. CG11282-PA  
  
30. CG1149-PA  
  
31. CG11910-PA  
  
32. CG12002-PA  
  
33. CG12199-PB  
  
34. CG12283-PA  
  
35. CG13487-PA  
  
36. CG14351-PA  
  
37. CG14662-PA  
  
38. CG14762-PA  
  
39. CG1504-PA  
  
40. CG15151-PA  
  
41. CG15658-PA  
  
42. CG15744-PA  
  
43. CG16974-PA  
  
44. CG1744-PA  
  
45. CG17667-PA  
  
46. CG1804-PA  
  
47. CG18095-PA  
  
48. CG18241-PA  
  
49. CG18249-PA  
  
50. CG18480-PA  
  
51. CG3095-PA  
  
52. CG31096-PA  
  
53. CG32055-PA  
  
54. CG32372-PA  
  
55. CG3413-PA  
  
56. CG40500-PD  
  
57. CG4054-PA  
  
58. CG4168-PA  
  
59. CG4192-PA  
  
60. CG4781-PA  
  
61. CG4950-PA  
  
62. CG4977-PA  
  
63. CG5096-PA  
  
64. CG5195-PA  
  
65. CG5490-PA  
  
66. CG5528-PA  
  
67. CG5541-PA  
  
68. CG5810-PA  
  
69. CG5819-PA  
  
70. CG5820-PA  
  
71. CG5888-PA  
  
72. CG6749-PA  
  
73. CG6890-PA  
  
74. CG6959-PA  
  
75. CG7121-PA  
  
76. CG7250-PA  
  
77. CG7503-PA  
  
78. CG7509-PA  
  
79. CG7665-PA  
  
80. CG7702-PA  
  
81. CG7800-PA  
  
82. CG7896-PA  
  
83. CG8355-PC  
  
84. CG8434-PA  
  
85. CG8561-PA  
  
86. CG8595-PA  
  
87. CG8852-PA  
  
88. CG8896-PA  
  
89. CG8930-PA  
  
90. CG9431-PA  
  
91. ENSMUSP00000006559  
  
92. ENSMUSP00000007620  
  
93. ENSMUSP00000010455  
  
94. ENSMUSP00000020087  
  
95. ENSMUSP00000020091  
  
96. ENSMUSP00000020094  
  
97. ENSMUSP00000020350  
  
98. ENSMUSP00000020400  
  
99. ENSMUSP00000021008  
  
100. ENSMUSP00000021346  
  
101. ENSMUSP00000021820  
  
102. ENSMUSP00000021822  
  
103. ENSMUSP00000022124  
  
104. ENSMUSP00000024916  
  
105. ENSMUSP00000025993  
  
106. ENSMUSP00000027706  
  
107. ENSMUSP00000028337  
  
108. ENSMUSP00000029623  
  
109. ENSMUSP00000030971  
  
110. ENSMUSP00000032105  
  
111. ENSMUSP00000032133  
  
112. ENSMUSP00000033741  
  
113. ENSMUSP00000033876  
  
114. ENSMUSP00000033967  
  
115. ENSMUSP00000034056  
  
116. ENSMUSP00000035444  
  
117. ENSMUSP00000035489  
  
118. ENSMUSP00000035831  
  
119. ENSMUSP00000035999  
  
120. ENSMUSP00000036762  
  
121. ENSMUSP00000037096  
  
122. ENSMUSP00000037616  
  
123. ENSMUSP00000037909  
  
124. ENSMUSP00000038048  
  
125. ENSMUSP00000038569  
  
126. ENSMUSP00000040436  
  
127. ENSMUSP00000040477  
  
128. ENSMUSP00000040877  
  
129. ENSMUSP00000041417  
  
130. ENSMUSP00000041499  
  
131. ENSMUSP00000041579  
  
132. ENSMUSP00000043101  
  
133. ENSMUSP00000043818  
  
134. ENSMUSP00000044094  
  
135. ENSMUSP00000045142  
  
136. ENSMUSP00000045162  
  
137. ENSMUSP00000045770  
  
138. ENSMUSP00000046705  
  
139. ENSMUSP00000047213  
  
140. ENSMUSP00000047573  
  
141. ENSMUSP00000047844  
  
142. ENSMUSP00000048803  
  
143. ENSMUSP00000048962  
  
144. ENSMUSP00000049296  
  
145. ENSMUSP00000049686  
  
146. ENSMUSP00000050039  
  
147. ENSMUSP00000051546  
  
148. ENSMUSP00000051895  
  
149. ENSMUSP00000052055  
  
150. ENSMUSP00000053123  
  
151. ENSMUSP00000053399  
  
152. ENSMUSP00000053573  
  
153. ENSMUSP00000053597  
  
154. ENSMUSP00000053840  
  
155. ENSMUSP00000053869  
  
156. ENSMUSP00000054140  
  
157. ENSMUSP00000054960  
  
158. ENSMUSP00000055604  
  
159. ENSMUSP00000056094  
  
160. ENSMUSP00000056642  
  
161. ENSMUSP00000056669  
  
162. ENSMUSP00000057005  
  
163. ENSMUSP00000057071  
  
164. ENSMUSP00000057293  
  
165. ENSMUSP00000057529  
  
166. ENSMUSP00000057563  
  
167. ENSMUSP00000057725  
  
168. ENSMUSP00000058050  
  
169. ENSMUSP00000059050  
  
170. ENSMUSP00000059270  
  
171. ENSMUSP00000059463  
  
172. ENSMUSP00000059570  
  
173. ENSMUSP00000059913  
  
174. ENSMUSP00000060210  
  
175. ENSMUSP00000060402  
  
176. ENSMUSP00000060793  
  
177. ENSMUSP00000061244  
  
178. ENSMUSP00000061828  
  
179. ENSMUSP00000061853  
  
180. ENSMUSP00000061906  
  
181. ENSMUSP00000062096  
  
182. ENSMUSP00000062158  
  
183. ENSMUSP00000062171  
  
184. ENSMUSP00000063882  
  
185. ENSMUSP00000064443  
  
186. ENSMUSP00000065706  
  
187. ENSMUSP00000066015  
  
188. ENSMUSP00000066777  
  
189. ENSMUSP00000066857  
  
190. ENSMUSP00000067130  
  
191. ENSMUSP00000067897  
  
192. ENSMUSP00000068906  
  
193. ENSMUSP00000069318  
  
194. ENSMUSP00000069772  
  
195. ENSMUSP00000070130  
  
196. ENSMUSP00000074360  
  
197. ENSMUSP00000074381  
  
198. ENSMUSP00000077492  
  
199. ENSMUSP00000077611  
  
200. ENSMUSP00000078757  
  
201. ENSMUSP00000082137  
  
202. ENSMUSP00000082207  
  
203. ENSMUSP00000084423  
  
204. ENSMUSP00000084507  
  
205. ENSMUSP00000088561  
  
206. ENSMUSP00000092127  
  
207. ENSMUSP00000097897  
  
208. ENSP00000021763  
  
209. ENSP00000043402  
  
210. ENSP00000052754  
  
211. ENSP00000215539  
  
212. ENSP00000216241  
  
213. ENSP00000217939  
  
214. ENSP00000223657  
  
215. ENSP00000223661  
  
216. ENSP00000241274  
  
217. ENSP00000246047  
  
218. ENSP00000246074  
  
219. ENSP00000246529  
  
220. ENSP00000246841  
  
221. ENSP00000247271  
  
222. ENSP00000247535  
  
223. ENSP00000249363  
  
224. ENSP00000249842  
  
225. ENSP00000252804  
  
226. ENSP00000254320  
  
227. ENSP00000256447  
  
228. ENSP00000258969  
  
229. ENSP00000259324  
  
230. ENSP00000260010  
  
231. ENSP00000260061  
  
232. ENSP00000261172  
  
233. ENSP00000262304  
  
234. ENSP00000262551  
  
235. ENSP00000265991  
  
236. ENSP00000266058  
  
237. ENSP00000266581  
  
238. ENSP00000266674  
  
239. ENSP00000266718  
  
240. ENSP00000266719  
  
241. ENSP00000273261  
  
242. ENSP00000273286  
  
243. ENSP00000273739  
  
244. ENSP00000275030  
  
245. ENSP00000277120  
  
246. ENSP00000277778  
  
247. ENSP00000278198  
  
248. ENSP00000282466  
  
249. ENSP00000282970  
  
250. ENSP00000291592  
  
251. ENSP00000294818  
  
252. ENSP00000294954  
  
253. ENSP00000295057  
  
254. ENSP00000296795  
  
255. ENSP00000298025  
  
256. ENSP00000298119  
  
257. ENSP00000298171  
  
258. ENSP00000298386  
  
259. ENSP00000299194  
  
260. ENSP00000300147  
  
261. ENSP00000302297  
  
262. ENSP00000302621  
  
263. ENSP00000303942  
  
264. ENSP00000304236  
  
265. ENSP00000304477  
  
266. ENSP00000306276  
  
267. ENSP00000306524  
  
268. ENSP00000306780  
  
269. ENSP00000306864  
  
270. ENSP00000308315  
  
271. ENSP00000309253  
  
272. ENSP00000310126  
  
273. ENSP00000311174  
  
274. ENSP00000312001  
  
275. ENSP00000312082  
  
276. ENSP00000312273  
  
277. ENSP00000312535  
  
278. ENSP00000314901  
  
279. ENSP00000316119  
  
280. ENSP00000319223  
  
281. ENSP00000319286  
  
282. ENSP00000319464  
  
283. ENSP00000320697  
  
284. ENSP00000323096  
  
285. ENSP00000325713  
  
286. ENSP00000326759  
  
287. ENSP00000326763  
  
288. ENSP00000327336  
  
289. ENSP00000327635  
  
290. ENSP00000328625  
  
291. ENSP00000329380  
  
292. ENSP00000330631  
  
293. ENSP00000330698  
  
294. ENSP00000330864  
  
295. ENSP00000332164  
  
296. ENSP00000332536  
  
297. ENSP00000332668  
  
298. ENSP00000332879  
  
299. ENSP00000333227  
  
300. ENSP00000333767  
  
301. ENSP00000334374  
  
302. ENSP00000334952  
  
303. ENSP00000335397  
  
304. ENSP00000335617  
  
305. ENSP00000336627  
  
306. ENSP00000338887  
  
307. ENSP00000339075  
  
308. ENSP00000339255  
  
309. ENSP00000340089  
  
310. ENSP00000340328  
  
311. ENSP00000340983  
  
312. ENSP00000341944  
  
313. ENSP00000343233  
  
314. ENSP00000343924  
  
315. ENSP00000344242  
  
316. ENSP00000345985  
  
317. ENSP00000346893  
  
318. ENSP00000347041  
  
319. ENSP00000349736  
  
320. ENSP00000350933  
  
321. ENSP00000351486  
  
322. ENSP00000354207  
  
323. ENSP00000354333  
  
324. ENSP00000354459  
  
325. ENSP00000354932  
  
326. ENSP00000355396  
  
327. ENSP00000360597  
  
328. ENSP00000361183  
  
329. ENSP00000366062  
  
330. ENSP00000367157  
  
331. ENSP00000373853  
  
332. ENSP00000374508  
  
333. F10F2.4  
  
334. F20D1.7  
  
335. F37E3.2  
  
336. F40E10.4  
  
337. GENSCAN00000039828  
  
338. IPI00662472.2  
  
339. K03A1.2.1  
  
340. K07A12.2  
  
341. K09C8.5  
  
342. M88.6a  
  
343. NM\_001013384  
  
344. NM\_173415  
  
345. NP\_065902  
  
346. NP\_067647  
  
347. OTTHUMP00000009643  
  
348. OTTHUMP00000028578  
  
349. OTTMUSP00000016116  
  
350. T01G9.3  
  
351. T05A1.3  
  
352. T21D12.9  
  
353. T22E7.1a  
  
354. T23G11.6  
  
355. XP\_001001535  
  
356. XP\_001005865  
  
357. XP\_143529  
  
358. XP\_485967  
  
359. XP\_892248  
  
360. XP\_944870  
  
361. XP\_945324  
  
362. XP\_945666  
  
363. XP\_984483  
  
364. XP\_991236  
  
365. Y37E3.13  
  
366. Y39A1A.7  
  
367. Y71F9B.8  
  
368. Y75B8A.5  
  
369. Y76A2B.2  
  
370. ZC262.3a  
  
371. ZK682.5  
  
372. ZK994.4
