# Supplementary figures and images for: The extracellular Leucine-Rich Repeat superfamily; a comparative survey and analysis of evolutionary relationships and expression patterns (part 2 of 2)
Source: BMC Genomics. 2007 Sep 14;8:320. doi: 10.1186/1471-2164-8-320 (PMC2235866; doi:10.1186/1471-2164-8-320)

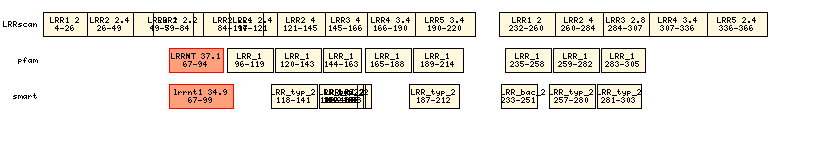

Supplement: Additional file 2 — LRRscan_out.html. Graphical comparison of HMMpfam and LRRscan results. A compressed archive (lrr_plots.tar.gz) containing 372 images in Portable Network Graphics (PNG) format, an information file (00README.txt) and two HTML-formatted pages, one with output from LRRscan (LRRscan_out.html) and one that links all the images together (00plots.html). After downloading, the archive must be to uncompressed and unpacked. Most modern operating systems (e.g. Windows XP, Mac OS X) will do this automatically when double-clicking on the file. Alternatively, you can use the free tool 'Stuffit Expander' () or your favourite unpacker. On Linux or Unix systems apply the following command: tar zxf lrr_plots.tar.gz. Please note that some browsers might uncompress the file during download without changing the file ending. If you have trouble unpacking the file try renaming it to lrr_plots.tar and double-click on it again. Unpacking the archive creates a new folder (lrr_plots) in which you can find a file called '00plots.html'. Open this file in a web-browser, either by double-clicking onto it or by using the 'File->Open File' menu (or equivalent) of your browser. This will bring up a web-page with plots of LRR motifs for 372 proteins. If you click on an image you can see the text output from LRRscan in a new window. [file 1471-2164-8-320-S2.gz › lrr_plots/ENSMUSP00000021820.png]

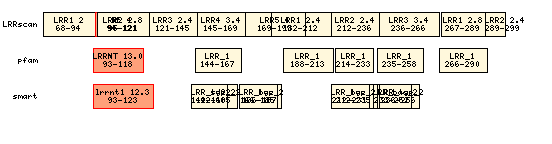

Supplement: Additional file 2 — LRRscan_out.html. Graphical comparison of HMMpfam and LRRscan results. A compressed archive (lrr_plots.tar.gz) containing 372 images in Portable Network Graphics (PNG) format, an information file (00README.txt) and two HTML-formatted pages, one with output from LRRscan (LRRscan_out.html) and one that links all the images together (00plots.html). After downloading, the archive must be to uncompressed and unpacked. Most modern operating systems (e.g. Windows XP, Mac OS X) will do this automatically when double-clicking on the file. Alternatively, you can use the free tool 'Stuffit Expander' () or your favourite unpacker. On Linux or Unix systems apply the following command: tar zxf lrr_plots.tar.gz. Please note that some browsers might uncompress the file during download without changing the file ending. If you have trouble unpacking the file try renaming it to lrr_plots.tar and double-click on it again. Unpacking the archive creates a new folder (lrr_plots) in which you can find a file called '00plots.html'. Open this file in a web-browser, either by double-clicking onto it or by using the 'File->Open File' menu (or equivalent) of your browser. This will bring up a web-page with plots of LRR motifs for 372 proteins. If you click on an image you can see the text output from LRRscan in a new window. [file 1471-2164-8-320-S2.gz › lrr_plots/ENSMUSP00000021822.png]

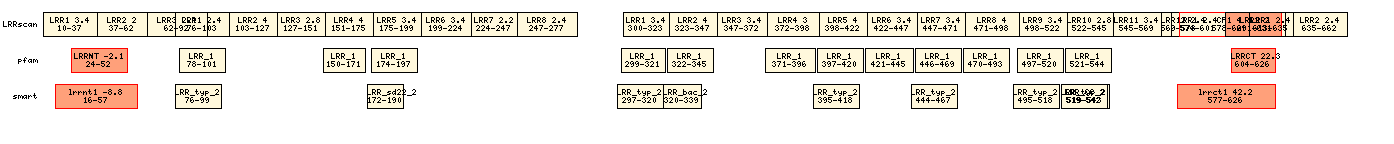

Supplement: Additional file 2 — LRRscan_out.html. Graphical comparison of HMMpfam and LRRscan results. A compressed archive (lrr_plots.tar.gz) containing 372 images in Portable Network Graphics (PNG) format, an information file (00README.txt) and two HTML-formatted pages, one with output from LRRscan (LRRscan_out.html) and one that links all the images together (00plots.html). After downloading, the archive must be to uncompressed and unpacked. Most modern operating systems (e.g. Windows XP, Mac OS X) will do this automatically when double-clicking on the file. Alternatively, you can use the free tool 'Stuffit Expander' () or your favourite unpacker. On Linux or Unix systems apply the following command: tar zxf lrr_plots.tar.gz. Please note that some browsers might uncompress the file during download without changing the file ending. If you have trouble unpacking the file try renaming it to lrr_plots.tar and double-click on it again. Unpacking the archive creates a new folder (lrr_plots) in which you can find a file called '00plots.html'. Open this file in a web-browser, either by double-clicking onto it or by using the 'File->Open File' menu (or equivalent) of your browser. This will bring up a web-page with plots of LRR motifs for 372 proteins. If you click on an image you can see the text output from LRRscan in a new window. [file 1471-2164-8-320-S2.gz › lrr_plots/ENSMUSP00000022124.png]

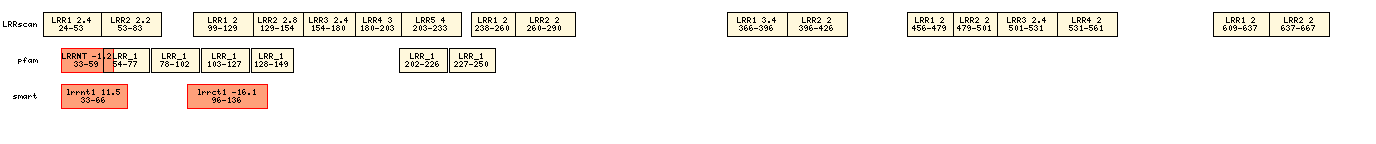

Supplement: Additional file 2 — LRRscan_out.html. Graphical comparison of HMMpfam and LRRscan results. A compressed archive (lrr_plots.tar.gz) containing 372 images in Portable Network Graphics (PNG) format, an information file (00README.txt) and two HTML-formatted pages, one with output from LRRscan (LRRscan_out.html) and one that links all the images together (00plots.html). After downloading, the archive must be to uncompressed and unpacked. Most modern operating systems (e.g. Windows XP, Mac OS X) will do this automatically when double-clicking on the file. Alternatively, you can use the free tool 'Stuffit Expander' () or your favourite unpacker. On Linux or Unix systems apply the following command: tar zxf lrr_plots.tar.gz. Please note that some browsers might uncompress the file during download without changing the file ending. If you have trouble unpacking the file try renaming it to lrr_plots.tar and double-click on it again. Unpacking the archive creates a new folder (lrr_plots) in which you can find a file called '00plots.html'. Open this file in a web-browser, either by double-clicking onto it or by using the 'File->Open File' menu (or equivalent) of your browser. This will bring up a web-page with plots of LRR motifs for 372 proteins. If you click on an image you can see the text output from LRRscan in a new window. [file 1471-2164-8-320-S2.gz › lrr_plots/ENSMUSP00000024916.png]

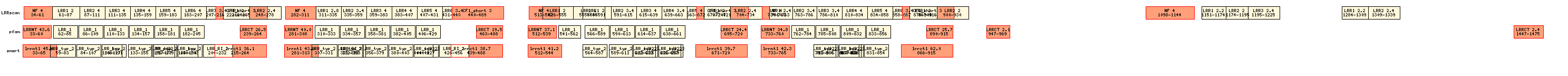

Supplement: Additional file 2 — LRRscan_out.html. Graphical comparison of HMMpfam and LRRscan results. A compressed archive (lrr_plots.tar.gz) containing 372 images in Portable Network Graphics (PNG) format, an information file (00README.txt) and two HTML-formatted pages, one with output from LRRscan (LRRscan_out.html) and one that links all the images together (00plots.html). After downloading, the archive must be to uncompressed and unpacked. Most modern operating systems (e.g. Windows XP, Mac OS X) will do this automatically when double-clicking on the file. Alternatively, you can use the free tool 'Stuffit Expander' () or your favourite unpacker. On Linux or Unix systems apply the following command: tar zxf lrr_plots.tar.gz. Please note that some browsers might uncompress the file during download without changing the file ending. If you have trouble unpacking the file try renaming it to lrr_plots.tar and double-click on it again. Unpacking the archive creates a new folder (lrr_plots) in which you can find a file called '00plots.html'. Open this file in a web-browser, either by double-clicking onto it or by using the 'File->Open File' menu (or equivalent) of your browser. This will bring up a web-page with plots of LRR motifs for 372 proteins. If you click on an image you can see the text output from LRRscan in a new window. [file 1471-2164-8-320-S2.gz › lrr_plots/ENSMUSP00000025993.png]

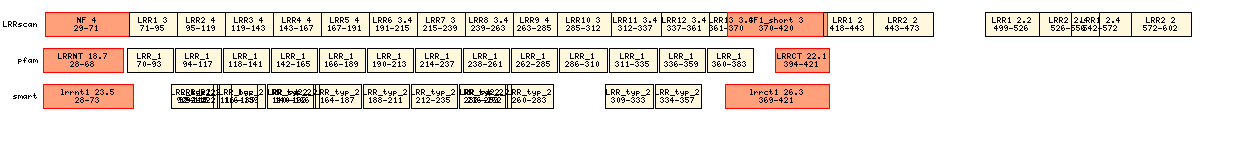

Supplement: Additional file 2 — LRRscan_out.html. Graphical comparison of HMMpfam and LRRscan results. A compressed archive (lrr_plots.tar.gz) containing 372 images in Portable Network Graphics (PNG) format, an information file (00README.txt) and two HTML-formatted pages, one with output from LRRscan (LRRscan_out.html) and one that links all the images together (00plots.html). After downloading, the archive must be to uncompressed and unpacked. Most modern operating systems (e.g. Windows XP, Mac OS X) will do this automatically when double-clicking on the file. Alternatively, you can use the free tool 'Stuffit Expander' () or your favourite unpacker. On Linux or Unix systems apply the following command: tar zxf lrr_plots.tar.gz. Please note that some browsers might uncompress the file during download without changing the file ending. If you have trouble unpacking the file try renaming it to lrr_plots.tar and double-click on it again. Unpacking the archive creates a new folder (lrr_plots) in which you can find a file called '00plots.html'. Open this file in a web-browser, either by double-clicking onto it or by using the 'File->Open File' menu (or equivalent) of your browser. This will bring up a web-page with plots of LRR motifs for 372 proteins. If you click on an image you can see the text output from LRRscan in a new window. [file 1471-2164-8-320-S2.gz › lrr_plots/ENSMUSP00000027706.png]

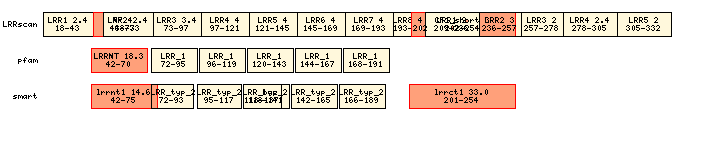

Supplement: Additional file 2 — LRRscan_out.html. Graphical comparison of HMMpfam and LRRscan results. A compressed archive (lrr_plots.tar.gz) containing 372 images in Portable Network Graphics (PNG) format, an information file (00README.txt) and two HTML-formatted pages, one with output from LRRscan (LRRscan_out.html) and one that links all the images together (00plots.html). After downloading, the archive must be to uncompressed and unpacked. Most modern operating systems (e.g. Windows XP, Mac OS X) will do this automatically when double-clicking on the file. Alternatively, you can use the free tool 'Stuffit Expander' () or your favourite unpacker. On Linux or Unix systems apply the following command: tar zxf lrr_plots.tar.gz. Please note that some browsers might uncompress the file during download without changing the file ending. If you have trouble unpacking the file try renaming it to lrr_plots.tar and double-click on it again. Unpacking the archive creates a new folder (lrr_plots) in which you can find a file called '00plots.html'. Open this file in a web-browser, either by double-clicking onto it or by using the 'File->Open File' menu (or equivalent) of your browser. This will bring up a web-page with plots of LRR motifs for 372 proteins. If you click on an image you can see the text output from LRRscan in a new window. [file 1471-2164-8-320-S2.gz › lrr_plots/ENSMUSP00000028337.png]

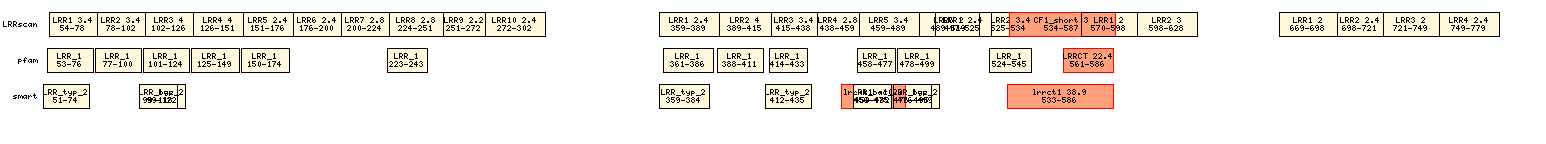

Supplement: Additional file 2 — LRRscan_out.html. Graphical comparison of HMMpfam and LRRscan results. A compressed archive (lrr_plots.tar.gz) containing 372 images in Portable Network Graphics (PNG) format, an information file (00README.txt) and two HTML-formatted pages, one with output from LRRscan (LRRscan_out.html) and one that links all the images together (00plots.html). After downloading, the archive must be to uncompressed and unpacked. Most modern operating systems (e.g. Windows XP, Mac OS X) will do this automatically when double-clicking on the file. Alternatively, you can use the free tool 'Stuffit Expander' () or your favourite unpacker. On Linux or Unix systems apply the following command: tar zxf lrr_plots.tar.gz. Please note that some browsers might uncompress the file during download without changing the file ending. If you have trouble unpacking the file try renaming it to lrr_plots.tar and double-click on it again. Unpacking the archive creates a new folder (lrr_plots) in which you can find a file called '00plots.html'. Open this file in a web-browser, either by double-clicking onto it or by using the 'File->Open File' menu (or equivalent) of your browser. This will bring up a web-page with plots of LRR motifs for 372 proteins. If you click on an image you can see the text output from LRRscan in a new window. [file 1471-2164-8-320-S2.gz › lrr_plots/ENSMUSP00000029623.png]

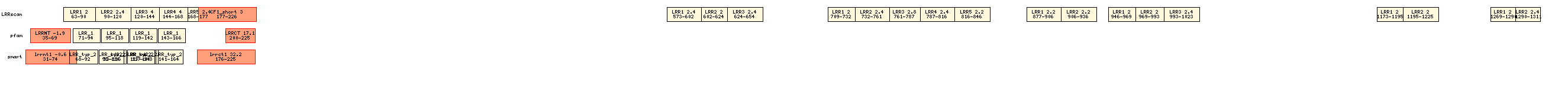

Supplement: Additional file 2 — LRRscan_out.html. Graphical comparison of HMMpfam and LRRscan results. A compressed archive (lrr_plots.tar.gz) containing 372 images in Portable Network Graphics (PNG) format, an information file (00README.txt) and two HTML-formatted pages, one with output from LRRscan (LRRscan_out.html) and one that links all the images together (00plots.html). After downloading, the archive must be to uncompressed and unpacked. Most modern operating systems (e.g. Windows XP, Mac OS X) will do this automatically when double-clicking on the file. Alternatively, you can use the free tool 'Stuffit Expander' () or your favourite unpacker. On Linux or Unix systems apply the following command: tar zxf lrr_plots.tar.gz. Please note that some browsers might uncompress the file during download without changing the file ending. If you have trouble unpacking the file try renaming it to lrr_plots.tar and double-click on it again. Unpacking the archive creates a new folder (lrr_plots) in which you can find a file called '00plots.html'. Open this file in a web-browser, either by double-clicking onto it or by using the 'File->Open File' menu (or equivalent) of your browser. This will bring up a web-page with plots of LRR motifs for 372 proteins. If you click on an image you can see the text output from LRRscan in a new window. [file 1471-2164-8-320-S2.gz › lrr_plots/ENSMUSP00000030971.png]

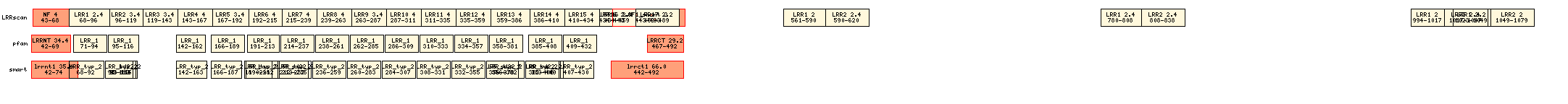

Supplement: Additional file 2 — LRRscan_out.html. Graphical comparison of HMMpfam and LRRscan results. A compressed archive (lrr_plots.tar.gz) containing 372 images in Portable Network Graphics (PNG) format, an information file (00README.txt) and two HTML-formatted pages, one with output from LRRscan (LRRscan_out.html) and one that links all the images together (00plots.html). After downloading, the archive must be to uncompressed and unpacked. Most modern operating systems (e.g. Windows XP, Mac OS X) will do this automatically when double-clicking on the file. Alternatively, you can use the free tool 'Stuffit Expander' () or your favourite unpacker. On Linux or Unix systems apply the following command: tar zxf lrr_plots.tar.gz. Please note that some browsers might uncompress the file during download without changing the file ending. If you have trouble unpacking the file try renaming it to lrr_plots.tar and double-click on it again. Unpacking the archive creates a new folder (lrr_plots) in which you can find a file called '00plots.html'. Open this file in a web-browser, either by double-clicking onto it or by using the 'File->Open File' menu (or equivalent) of your browser. This will bring up a web-page with plots of LRR motifs for 372 proteins. If you click on an image you can see the text output from LRRscan in a new window. [file 1471-2164-8-320-S2.gz › lrr_plots/ENSMUSP00000032105.png]

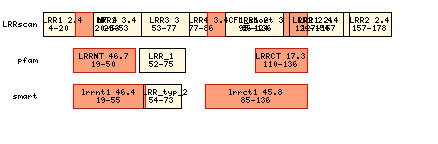

Supplement: Additional file 2 — LRRscan_out.html. Graphical comparison of HMMpfam and LRRscan results. A compressed archive (lrr_plots.tar.gz) containing 372 images in Portable Network Graphics (PNG) format, an information file (00README.txt) and two HTML-formatted pages, one with output from LRRscan (LRRscan_out.html) and one that links all the images together (00plots.html). After downloading, the archive must be to uncompressed and unpacked. Most modern operating systems (e.g. Windows XP, Mac OS X) will do this automatically when double-clicking on the file. Alternatively, you can use the free tool 'Stuffit Expander' () or your favourite unpacker. On Linux or Unix systems apply the following command: tar zxf lrr_plots.tar.gz. Please note that some browsers might uncompress the file during download without changing the file ending. If you have trouble unpacking the file try renaming it to lrr_plots.tar and double-click on it again. Unpacking the archive creates a new folder (lrr_plots) in which you can find a file called '00plots.html'. Open this file in a web-browser, either by double-clicking onto it or by using the 'File->Open File' menu (or equivalent) of your browser. This will bring up a web-page with plots of LRR motifs for 372 proteins. If you click on an image you can see the text output from LRRscan in a new window. [file 1471-2164-8-320-S2.gz › lrr_plots/ENSMUSP00000032133.png]

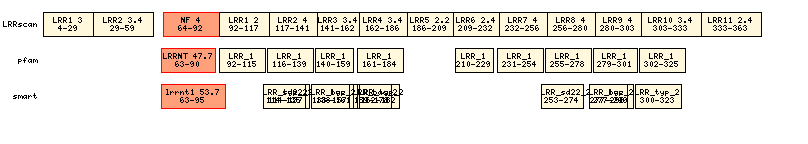

Supplement: Additional file 2 — LRRscan_out.html. Graphical comparison of HMMpfam and LRRscan results. A compressed archive (lrr_plots.tar.gz) containing 372 images in Portable Network Graphics (PNG) format, an information file (00README.txt) and two HTML-formatted pages, one with output from LRRscan (LRRscan_out.html) and one that links all the images together (00plots.html). After downloading, the archive must be to uncompressed and unpacked. Most modern operating systems (e.g. Windows XP, Mac OS X) will do this automatically when double-clicking on the file. Alternatively, you can use the free tool 'Stuffit Expander' () or your favourite unpacker. On Linux or Unix systems apply the following command: tar zxf lrr_plots.tar.gz. Please note that some browsers might uncompress the file during download without changing the file ending. If you have trouble unpacking the file try renaming it to lrr_plots.tar and double-click on it again. Unpacking the archive creates a new folder (lrr_plots) in which you can find a file called '00plots.html'. Open this file in a web-browser, either by double-clicking onto it or by using the 'File->Open File' menu (or equivalent) of your browser. This will bring up a web-page with plots of LRR motifs for 372 proteins. If you click on an image you can see the text output from LRRscan in a new window. [file 1471-2164-8-320-S2.gz › lrr_plots/ENSMUSP00000033741.png]

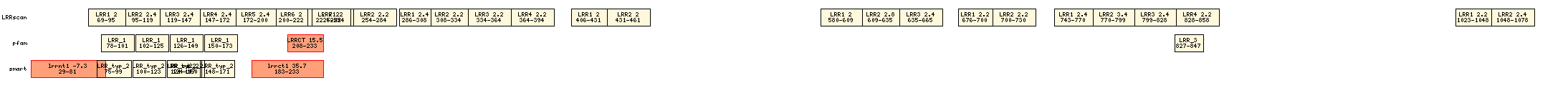

Supplement: Additional file 2 — LRRscan_out.html. Graphical comparison of HMMpfam and LRRscan results. A compressed archive (lrr_plots.tar.gz) containing 372 images in Portable Network Graphics (PNG) format, an information file (00README.txt) and two HTML-formatted pages, one with output from LRRscan (LRRscan_out.html) and one that links all the images together (00plots.html). After downloading, the archive must be to uncompressed and unpacked. Most modern operating systems (e.g. Windows XP, Mac OS X) will do this automatically when double-clicking on the file. Alternatively, you can use the free tool 'Stuffit Expander' () or your favourite unpacker. On Linux or Unix systems apply the following command: tar zxf lrr_plots.tar.gz. Please note that some browsers might uncompress the file during download without changing the file ending. If you have trouble unpacking the file try renaming it to lrr_plots.tar and double-click on it again. Unpacking the archive creates a new folder (lrr_plots) in which you can find a file called '00plots.html'. Open this file in a web-browser, either by double-clicking onto it or by using the 'File->Open File' menu (or equivalent) of your browser. This will bring up a web-page with plots of LRR motifs for 372 proteins. If you click on an image you can see the text output from LRRscan in a new window. [file 1471-2164-8-320-S2.gz › lrr_plots/ENSMUSP00000033876.png]

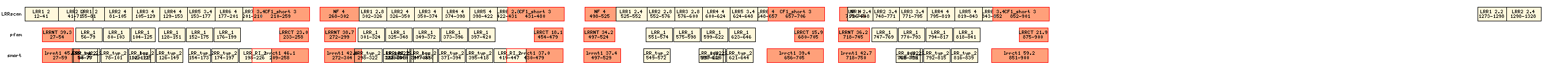

Supplement: Additional file 2 — LRRscan_out.html. Graphical comparison of HMMpfam and LRRscan results. A compressed archive (lrr_plots.tar.gz) containing 372 images in Portable Network Graphics (PNG) format, an information file (00README.txt) and two HTML-formatted pages, one with output from LRRscan (LRRscan_out.html) and one that links all the images together (00plots.html). After downloading, the archive must be to uncompressed and unpacked. Most modern operating systems (e.g. Windows XP, Mac OS X) will do this automatically when double-clicking on the file. Alternatively, you can use the free tool 'Stuffit Expander' () or your favourite unpacker. On Linux or Unix systems apply the following command: tar zxf lrr_plots.tar.gz. Please note that some browsers might uncompress the file during download without changing the file ending. If you have trouble unpacking the file try renaming it to lrr_plots.tar and double-click on it again. Unpacking the archive creates a new folder (lrr_plots) in which you can find a file called '00plots.html'. Open this file in a web-browser, either by double-clicking onto it or by using the 'File->Open File' menu (or equivalent) of your browser. This will bring up a web-page with plots of LRR motifs for 372 proteins. If you click on an image you can see the text output from LRRscan in a new window. [file 1471-2164-8-320-S2.gz › lrr_plots/ENSMUSP00000033967.png]

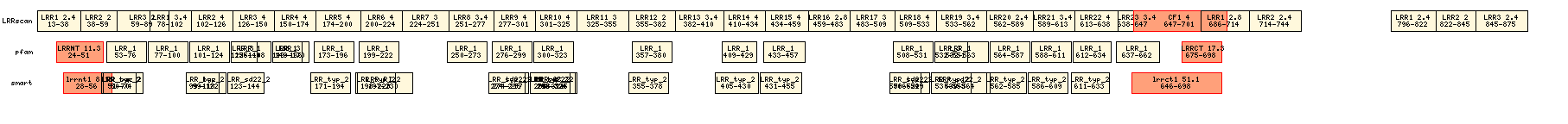

Supplement: Additional file 2 — LRRscan_out.html. Graphical comparison of HMMpfam and LRRscan results. A compressed archive (lrr_plots.tar.gz) containing 372 images in Portable Network Graphics (PNG) format, an information file (00README.txt) and two HTML-formatted pages, one with output from LRRscan (LRRscan_out.html) and one that links all the images together (00plots.html). After downloading, the archive must be to uncompressed and unpacked. Most modern operating systems (e.g. Windows XP, Mac OS X) will do this automatically when double-clicking on the file. Alternatively, you can use the free tool 'Stuffit Expander' () or your favourite unpacker. On Linux or Unix systems apply the following command: tar zxf lrr_plots.tar.gz. Please note that some browsers might uncompress the file during download without changing the file ending. If you have trouble unpacking the file try renaming it to lrr_plots.tar and double-click on it again. Unpacking the archive creates a new folder (lrr_plots) in which you can find a file called '00plots.html'. Open this file in a web-browser, either by double-clicking onto it or by using the 'File->Open File' menu (or equivalent) of your browser. This will bring up a web-page with plots of LRR motifs for 372 proteins. If you click on an image you can see the text output from LRRscan in a new window. [file 1471-2164-8-320-S2.gz › lrr_plots/ENSMUSP00000034056.png]

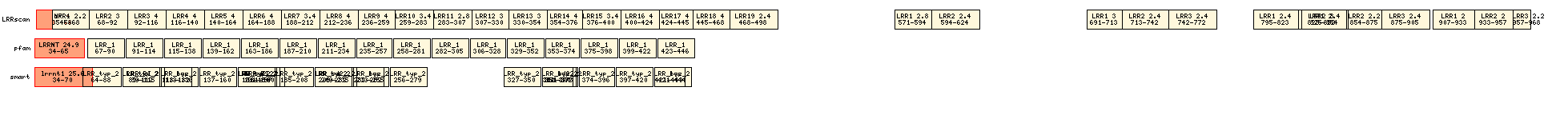

Supplement: Additional file 2 — LRRscan_out.html. Graphical comparison of HMMpfam and LRRscan results. A compressed archive (lrr_plots.tar.gz) containing 372 images in Portable Network Graphics (PNG) format, an information file (00README.txt) and two HTML-formatted pages, one with output from LRRscan (LRRscan_out.html) and one that links all the images together (00plots.html). After downloading, the archive must be to uncompressed and unpacked. Most modern operating systems (e.g. Windows XP, Mac OS X) will do this automatically when double-clicking on the file. Alternatively, you can use the free tool 'Stuffit Expander' () or your favourite unpacker. On Linux or Unix systems apply the following command: tar zxf lrr_plots.tar.gz. Please note that some browsers might uncompress the file during download without changing the file ending. If you have trouble unpacking the file try renaming it to lrr_plots.tar and double-click on it again. Unpacking the archive creates a new folder (lrr_plots) in which you can find a file called '00plots.html'. Open this file in a web-browser, either by double-clicking onto it or by using the 'File->Open File' menu (or equivalent) of your browser. This will bring up a web-page with plots of LRR motifs for 372 proteins. If you click on an image you can see the text output from LRRscan in a new window. [file 1471-2164-8-320-S2.gz › lrr_plots/ENSMUSP00000035444.png]

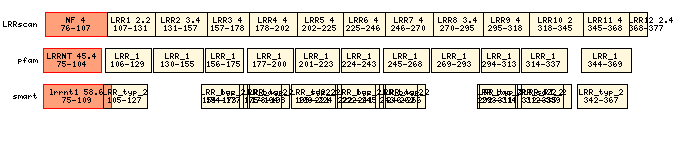

Supplement: Additional file 2 — LRRscan_out.html. Graphical comparison of HMMpfam and LRRscan results. A compressed archive (lrr_plots.tar.gz) containing 372 images in Portable Network Graphics (PNG) format, an information file (00README.txt) and two HTML-formatted pages, one with output from LRRscan (LRRscan_out.html) and one that links all the images together (00plots.html). After downloading, the archive must be to uncompressed and unpacked. Most modern operating systems (e.g. Windows XP, Mac OS X) will do this automatically when double-clicking on the file. Alternatively, you can use the free tool 'Stuffit Expander' () or your favourite unpacker. On Linux or Unix systems apply the following command: tar zxf lrr_plots.tar.gz. Please note that some browsers might uncompress the file during download without changing the file ending. If you have trouble unpacking the file try renaming it to lrr_plots.tar and double-click on it again. Unpacking the archive creates a new folder (lrr_plots) in which you can find a file called '00plots.html'. Open this file in a web-browser, either by double-clicking onto it or by using the 'File->Open File' menu (or equivalent) of your browser. This will bring up a web-page with plots of LRR motifs for 372 proteins. If you click on an image you can see the text output from LRRscan in a new window. [file 1471-2164-8-320-S2.gz › lrr_plots/ENSMUSP00000035489.png]

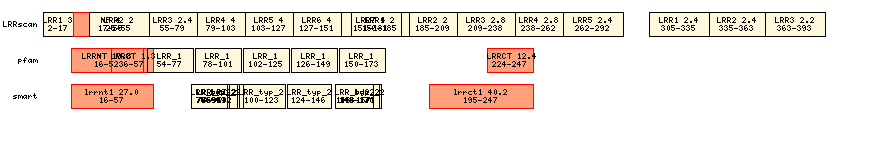

Supplement: Additional file 2 — LRRscan_out.html. Graphical comparison of HMMpfam and LRRscan results. A compressed archive (lrr_plots.tar.gz) containing 372 images in Portable Network Graphics (PNG) format, an information file (00README.txt) and two HTML-formatted pages, one with output from LRRscan (LRRscan_out.html) and one that links all the images together (00plots.html). After downloading, the archive must be to uncompressed and unpacked. Most modern operating systems (e.g. Windows XP, Mac OS X) will do this automatically when double-clicking on the file. Alternatively, you can use the free tool 'Stuffit Expander' () or your favourite unpacker. On Linux or Unix systems apply the following command: tar zxf lrr_plots.tar.gz. Please note that some browsers might uncompress the file during download without changing the file ending. If you have trouble unpacking the file try renaming it to lrr_plots.tar and double-click on it again. Unpacking the archive creates a new folder (lrr_plots) in which you can find a file called '00plots.html'. Open this file in a web-browser, either by double-clicking onto it or by using the 'File->Open File' menu (or equivalent) of your browser. This will bring up a web-page with plots of LRR motifs for 372 proteins. If you click on an image you can see the text output from LRRscan in a new window. [file 1471-2164-8-320-S2.gz › lrr_plots/ENSMUSP00000035831.png]

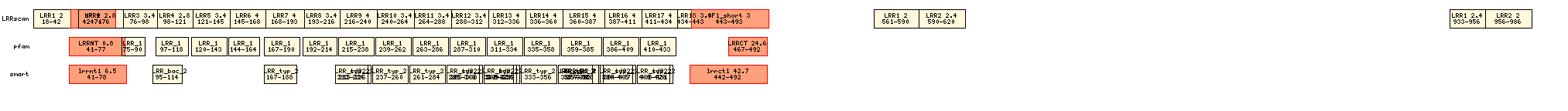

Supplement: Additional file 2 — LRRscan_out.html. Graphical comparison of HMMpfam and LRRscan results. A compressed archive (lrr_plots.tar.gz) containing 372 images in Portable Network Graphics (PNG) format, an information file (00README.txt) and two HTML-formatted pages, one with output from LRRscan (LRRscan_out.html) and one that links all the images together (00plots.html). After downloading, the archive must be to uncompressed and unpacked. Most modern operating systems (e.g. Windows XP, Mac OS X) will do this automatically when double-clicking on the file. Alternatively, you can use the free tool 'Stuffit Expander' () or your favourite unpacker. On Linux or Unix systems apply the following command: tar zxf lrr_plots.tar.gz. Please note that some browsers might uncompress the file during download without changing the file ending. If you have trouble unpacking the file try renaming it to lrr_plots.tar and double-click on it again. Unpacking the archive creates a new folder (lrr_plots) in which you can find a file called '00plots.html'. Open this file in a web-browser, either by double-clicking onto it or by using the 'File->Open File' menu (or equivalent) of your browser. This will bring up a web-page with plots of LRR motifs for 372 proteins. If you click on an image you can see the text output from LRRscan in a new window. [file 1471-2164-8-320-S2.gz › lrr_plots/ENSMUSP00000035999.png]

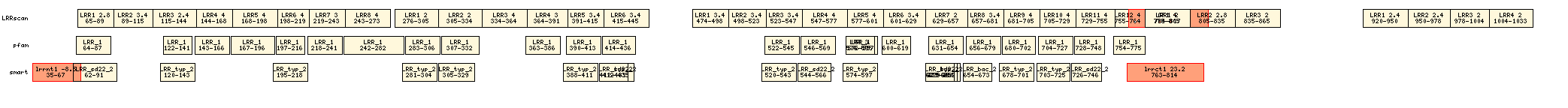

Supplement: Additional file 2 — LRRscan_out.html. Graphical comparison of HMMpfam and LRRscan results. A compressed archive (lrr_plots.tar.gz) containing 372 images in Portable Network Graphics (PNG) format, an information file (00README.txt) and two HTML-formatted pages, one with output from LRRscan (LRRscan_out.html) and one that links all the images together (00plots.html). After downloading, the archive must be to uncompressed and unpacked. Most modern operating systems (e.g. Windows XP, Mac OS X) will do this automatically when double-clicking on the file. Alternatively, you can use the free tool 'Stuffit Expander' () or your favourite unpacker. On Linux or Unix systems apply the following command: tar zxf lrr_plots.tar.gz. Please note that some browsers might uncompress the file during download without changing the file ending. If you have trouble unpacking the file try renaming it to lrr_plots.tar and double-click on it again. Unpacking the archive creates a new folder (lrr_plots) in which you can find a file called '00plots.html'. Open this file in a web-browser, either by double-clicking onto it or by using the 'File->Open File' menu (or equivalent) of your browser. This will bring up a web-page with plots of LRR motifs for 372 proteins. If you click on an image you can see the text output from LRRscan in a new window. [file 1471-2164-8-320-S2.gz › lrr_plots/ENSMUSP00000036762.png]

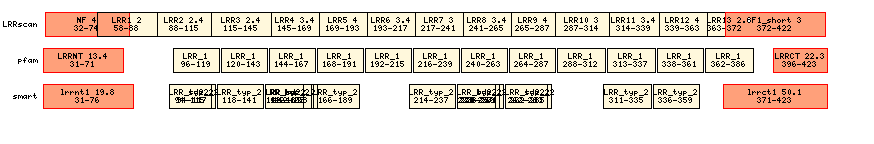

Supplement: Additional file 2 — LRRscan_out.html. Graphical comparison of HMMpfam and LRRscan results. A compressed archive (lrr_plots.tar.gz) containing 372 images in Portable Network Graphics (PNG) format, an information file (00README.txt) and two HTML-formatted pages, one with output from LRRscan (LRRscan_out.html) and one that links all the images together (00plots.html). After downloading, the archive must be to uncompressed and unpacked. Most modern operating systems (e.g. Windows XP, Mac OS X) will do this automatically when double-clicking on the file. Alternatively, you can use the free tool 'Stuffit Expander' () or your favourite unpacker. On Linux or Unix systems apply the following command: tar zxf lrr_plots.tar.gz. Please note that some browsers might uncompress the file during download without changing the file ending. If you have trouble unpacking the file try renaming it to lrr_plots.tar and double-click on it again. Unpacking the archive creates a new folder (lrr_plots) in which you can find a file called '00plots.html'. Open this file in a web-browser, either by double-clicking onto it or by using the 'File->Open File' menu (or equivalent) of your browser. This will bring up a web-page with plots of LRR motifs for 372 proteins. If you click on an image you can see the text output from LRRscan in a new window. [file 1471-2164-8-320-S2.gz › lrr_plots/ENSMUSP00000037096.png]

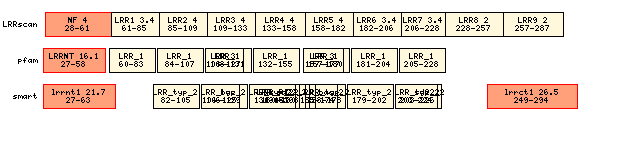

Supplement: Additional file 2 — LRRscan_out.html. Graphical comparison of HMMpfam and LRRscan results. A compressed archive (lrr_plots.tar.gz) containing 372 images in Portable Network Graphics (PNG) format, an information file (00README.txt) and two HTML-formatted pages, one with output from LRRscan (LRRscan_out.html) and one that links all the images together (00plots.html). After downloading, the archive must be to uncompressed and unpacked. Most modern operating systems (e.g. Windows XP, Mac OS X) will do this automatically when double-clicking on the file. Alternatively, you can use the free tool 'Stuffit Expander' () or your favourite unpacker. On Linux or Unix systems apply the following command: tar zxf lrr_plots.tar.gz. Please note that some browsers might uncompress the file during download without changing the file ending. If you have trouble unpacking the file try renaming it to lrr_plots.tar and double-click on it again. Unpacking the archive creates a new folder (lrr_plots) in which you can find a file called '00plots.html'. Open this file in a web-browser, either by double-clicking onto it or by using the 'File->Open File' menu (or equivalent) of your browser. This will bring up a web-page with plots of LRR motifs for 372 proteins. If you click on an image you can see the text output from LRRscan in a new window. [file 1471-2164-8-320-S2.gz › lrr_plots/ENSMUSP00000037616.png]

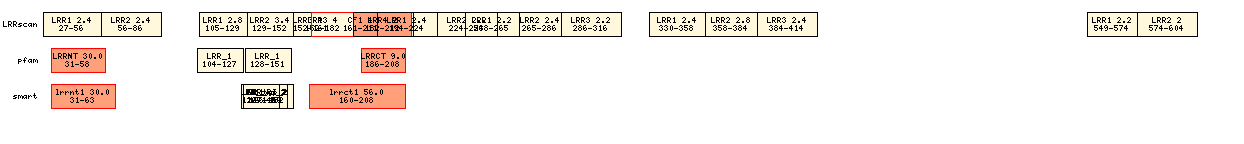

Supplement: Additional file 2 — LRRscan_out.html. Graphical comparison of HMMpfam and LRRscan results. A compressed archive (lrr_plots.tar.gz) containing 372 images in Portable Network Graphics (PNG) format, an information file (00README.txt) and two HTML-formatted pages, one with output from LRRscan (LRRscan_out.html) and one that links all the images together (00plots.html). After downloading, the archive must be to uncompressed and unpacked. Most modern operating systems (e.g. Windows XP, Mac OS X) will do this automatically when double-clicking on the file. Alternatively, you can use the free tool 'Stuffit Expander' () or your favourite unpacker. On Linux or Unix systems apply the following command: tar zxf lrr_plots.tar.gz. Please note that some browsers might uncompress the file during download without changing the file ending. If you have trouble unpacking the file try renaming it to lrr_plots.tar and double-click on it again. Unpacking the archive creates a new folder (lrr_plots) in which you can find a file called '00plots.html'. Open this file in a web-browser, either by double-clicking onto it or by using the 'File->Open File' menu (or equivalent) of your browser. This will bring up a web-page with plots of LRR motifs for 372 proteins. If you click on an image you can see the text output from LRRscan in a new window. [file 1471-2164-8-320-S2.gz › lrr_plots/ENSMUSP00000037909.png]

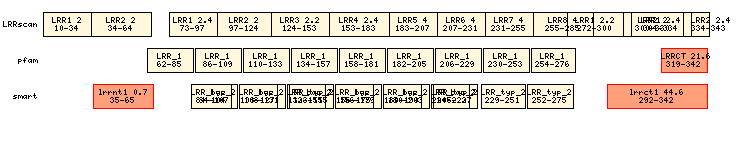

Supplement: Additional file 2 — LRRscan_out.html. Graphical comparison of HMMpfam and LRRscan results. A compressed archive (lrr_plots.tar.gz) containing 372 images in Portable Network Graphics (PNG) format, an information file (00README.txt) and two HTML-formatted pages, one with output from LRRscan (LRRscan_out.html) and one that links all the images together (00plots.html). After downloading, the archive must be to uncompressed and unpacked. Most modern operating systems (e.g. Windows XP, Mac OS X) will do this automatically when double-clicking on the file. Alternatively, you can use the free tool 'Stuffit Expander' () or your favourite unpacker. On Linux or Unix systems apply the following command: tar zxf lrr_plots.tar.gz. Please note that some browsers might uncompress the file during download without changing the file ending. If you have trouble unpacking the file try renaming it to lrr_plots.tar and double-click on it again. Unpacking the archive creates a new folder (lrr_plots) in which you can find a file called '00plots.html'. Open this file in a web-browser, either by double-clicking onto it or by using the 'File->Open File' menu (or equivalent) of your browser. This will bring up a web-page with plots of LRR motifs for 372 proteins. If you click on an image you can see the text output from LRRscan in a new window. [file 1471-2164-8-320-S2.gz › lrr_plots/ENSMUSP00000038048.png]

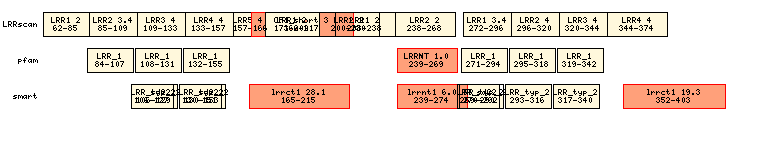

Supplement: Additional file 2 — LRRscan_out.html. Graphical comparison of HMMpfam and LRRscan results. A compressed archive (lrr_plots.tar.gz) containing 372 images in Portable Network Graphics (PNG) format, an information file (00README.txt) and two HTML-formatted pages, one with output from LRRscan (LRRscan_out.html) and one that links all the images together (00plots.html). After downloading, the archive must be to uncompressed and unpacked. Most modern operating systems (e.g. Windows XP, Mac OS X) will do this automatically when double-clicking on the file. Alternatively, you can use the free tool 'Stuffit Expander' () or your favourite unpacker. On Linux or Unix systems apply the following command: tar zxf lrr_plots.tar.gz. Please note that some browsers might uncompress the file during download without changing the file ending. If you have trouble unpacking the file try renaming it to lrr_plots.tar and double-click on it again. Unpacking the archive creates a new folder (lrr_plots) in which you can find a file called '00plots.html'. Open this file in a web-browser, either by double-clicking onto it or by using the 'File->Open File' menu (or equivalent) of your browser. This will bring up a web-page with plots of LRR motifs for 372 proteins. If you click on an image you can see the text output from LRRscan in a new window. [file 1471-2164-8-320-S2.gz › lrr_plots/ENSMUSP00000038569.png]

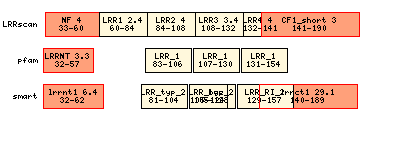

Supplement: Additional file 2 — LRRscan_out.html. Graphical comparison of HMMpfam and LRRscan results. A compressed archive (lrr_plots.tar.gz) containing 372 images in Portable Network Graphics (PNG) format, an information file (00README.txt) and two HTML-formatted pages, one with output from LRRscan (LRRscan_out.html) and one that links all the images together (00plots.html). After downloading, the archive must be to uncompressed and unpacked. Most modern operating systems (e.g. Windows XP, Mac OS X) will do this automatically when double-clicking on the file. Alternatively, you can use the free tool 'Stuffit Expander' () or your favourite unpacker. On Linux or Unix systems apply the following command: tar zxf lrr_plots.tar.gz. Please note that some browsers might uncompress the file during download without changing the file ending. If you have trouble unpacking the file try renaming it to lrr_plots.tar and double-click on it again. Unpacking the archive creates a new folder (lrr_plots) in which you can find a file called '00plots.html'. Open this file in a web-browser, either by double-clicking onto it or by using the 'File->Open File' menu (or equivalent) of your browser. This will bring up a web-page with plots of LRR motifs for 372 proteins. If you click on an image you can see the text output from LRRscan in a new window. [file 1471-2164-8-320-S2.gz › lrr_plots/ENSMUSP00000040436.png]

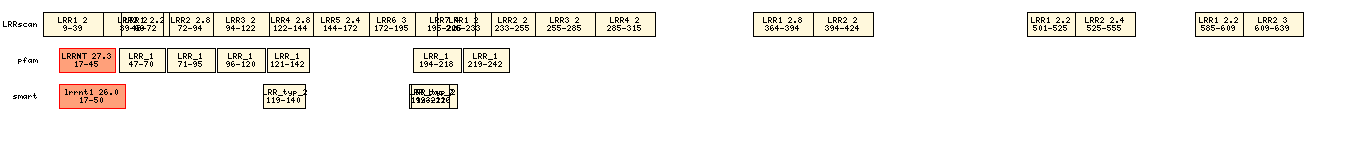

Supplement: Additional file 2 — LRRscan_out.html. Graphical comparison of HMMpfam and LRRscan results. A compressed archive (lrr_plots.tar.gz) containing 372 images in Portable Network Graphics (PNG) format, an information file (00README.txt) and two HTML-formatted pages, one with output from LRRscan (LRRscan_out.html) and one that links all the images together (00plots.html). After downloading, the archive must be to uncompressed and unpacked. Most modern operating systems (e.g. Windows XP, Mac OS X) will do this automatically when double-clicking on the file. Alternatively, you can use the free tool 'Stuffit Expander' () or your favourite unpacker. On Linux or Unix systems apply the following command: tar zxf lrr_plots.tar.gz. Please note that some browsers might uncompress the file during download without changing the file ending. If you have trouble unpacking the file try renaming it to lrr_plots.tar and double-click on it again. Unpacking the archive creates a new folder (lrr_plots) in which you can find a file called '00plots.html'. Open this file in a web-browser, either by double-clicking onto it or by using the 'File->Open File' menu (or equivalent) of your browser. This will bring up a web-page with plots of LRR motifs for 372 proteins. If you click on an image you can see the text output from LRRscan in a new window. [file 1471-2164-8-320-S2.gz › lrr_plots/ENSMUSP00000040477.png]

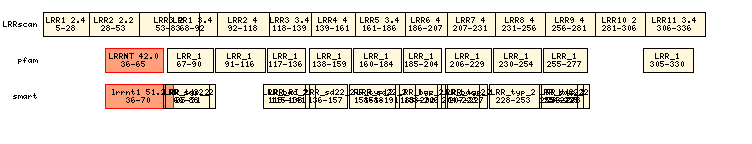

Supplement: Additional file 2 — LRRscan_out.html. Graphical comparison of HMMpfam and LRRscan results. A compressed archive (lrr_plots.tar.gz) containing 372 images in Portable Network Graphics (PNG) format, an information file (00README.txt) and two HTML-formatted pages, one with output from LRRscan (LRRscan_out.html) and one that links all the images together (00plots.html). After downloading, the archive must be to uncompressed and unpacked. Most modern operating systems (e.g. Windows XP, Mac OS X) will do this automatically when double-clicking on the file. Alternatively, you can use the free tool 'Stuffit Expander' () or your favourite unpacker. On Linux or Unix systems apply the following command: tar zxf lrr_plots.tar.gz. Please note that some browsers might uncompress the file during download without changing the file ending. If you have trouble unpacking the file try renaming it to lrr_plots.tar and double-click on it again. Unpacking the archive creates a new folder (lrr_plots) in which you can find a file called '00plots.html'. Open this file in a web-browser, either by double-clicking onto it or by using the 'File->Open File' menu (or equivalent) of your browser. This will bring up a web-page with plots of LRR motifs for 372 proteins. If you click on an image you can see the text output from LRRscan in a new window. [file 1471-2164-8-320-S2.gz › lrr_plots/ENSMUSP00000040877.png]

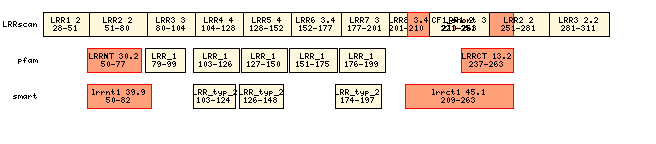

Supplement: Additional file 2 — LRRscan_out.html. Graphical comparison of HMMpfam and LRRscan results. A compressed archive (lrr_plots.tar.gz) containing 372 images in Portable Network Graphics (PNG) format, an information file (00README.txt) and two HTML-formatted pages, one with output from LRRscan (LRRscan_out.html) and one that links all the images together (00plots.html). After downloading, the archive must be to uncompressed and unpacked. Most modern operating systems (e.g. Windows XP, Mac OS X) will do this automatically when double-clicking on the file. Alternatively, you can use the free tool 'Stuffit Expander' () or your favourite unpacker. On Linux or Unix systems apply the following command: tar zxf lrr_plots.tar.gz. Please note that some browsers might uncompress the file during download without changing the file ending. If you have trouble unpacking the file try renaming it to lrr_plots.tar and double-click on it again. Unpacking the archive creates a new folder (lrr_plots) in which you can find a file called '00plots.html'. Open this file in a web-browser, either by double-clicking onto it or by using the 'File->Open File' menu (or equivalent) of your browser. This will bring up a web-page with plots of LRR motifs for 372 proteins. If you click on an image you can see the text output from LRRscan in a new window. [file 1471-2164-8-320-S2.gz › lrr_plots/ENSMUSP00000041417.png]

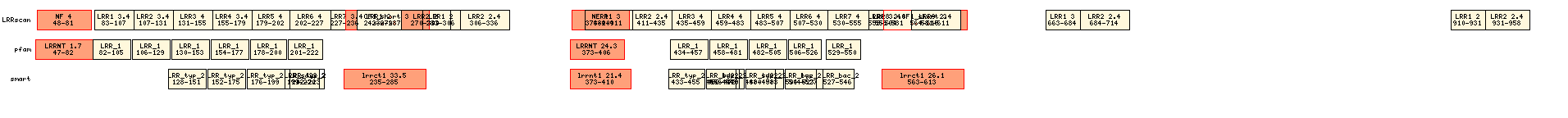

Supplement: Additional file 2 — LRRscan_out.html. Graphical comparison of HMMpfam and LRRscan results. A compressed archive (lrr_plots.tar.gz) containing 372 images in Portable Network Graphics (PNG) format, an information file (00README.txt) and two HTML-formatted pages, one with output from LRRscan (LRRscan_out.html) and one that links all the images together (00plots.html). After downloading, the archive must be to uncompressed and unpacked. Most modern operating systems (e.g. Windows XP, Mac OS X) will do this automatically when double-clicking on the file. Alternatively, you can use the free tool 'Stuffit Expander' () or your favourite unpacker. On Linux or Unix systems apply the following command: tar zxf lrr_plots.tar.gz. Please note that some browsers might uncompress the file during download without changing the file ending. If you have trouble unpacking the file try renaming it to lrr_plots.tar and double-click on it again. Unpacking the archive creates a new folder (lrr_plots) in which you can find a file called '00plots.html'. Open this file in a web-browser, either by double-clicking onto it or by using the 'File->Open File' menu (or equivalent) of your browser. This will bring up a web-page with plots of LRR motifs for 372 proteins. If you click on an image you can see the text output from LRRscan in a new window. [file 1471-2164-8-320-S2.gz › lrr_plots/ENSMUSP00000041499.png]

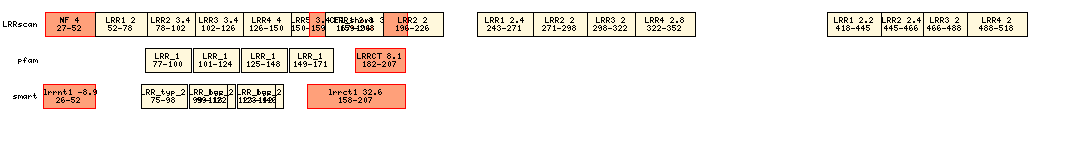

Supplement: Additional file 2 — LRRscan_out.html. Graphical comparison of HMMpfam and LRRscan results. A compressed archive (lrr_plots.tar.gz) containing 372 images in Portable Network Graphics (PNG) format, an information file (00README.txt) and two HTML-formatted pages, one with output from LRRscan (LRRscan_out.html) and one that links all the images together (00plots.html). After downloading, the archive must be to uncompressed and unpacked. Most modern operating systems (e.g. Windows XP, Mac OS X) will do this automatically when double-clicking on the file. Alternatively, you can use the free tool 'Stuffit Expander' () or your favourite unpacker. On Linux or Unix systems apply the following command: tar zxf lrr_plots.tar.gz. Please note that some browsers might uncompress the file during download without changing the file ending. If you have trouble unpacking the file try renaming it to lrr_plots.tar and double-click on it again. Unpacking the archive creates a new folder (lrr_plots) in which you can find a file called '00plots.html'. Open this file in a web-browser, either by double-clicking onto it or by using the 'File->Open File' menu (or equivalent) of your browser. This will bring up a web-page with plots of LRR motifs for 372 proteins. If you click on an image you can see the text output from LRRscan in a new window. [file 1471-2164-8-320-S2.gz › lrr_plots/ENSMUSP00000041579.png]

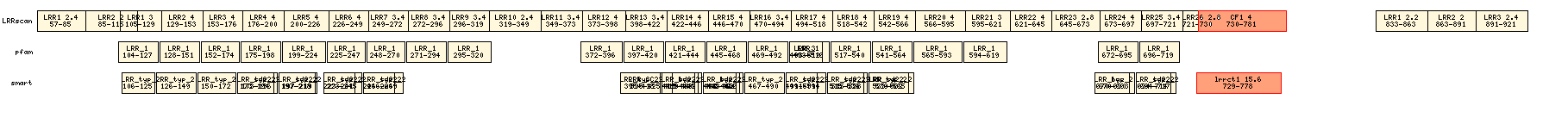

Supplement: Additional file 2 — LRRscan_out.html. Graphical comparison of HMMpfam and LRRscan results. A compressed archive (lrr_plots.tar.gz) containing 372 images in Portable Network Graphics (PNG) format, an information file (00README.txt) and two HTML-formatted pages, one with output from LRRscan (LRRscan_out.html) and one that links all the images together (00plots.html). After downloading, the archive must be to uncompressed and unpacked. Most modern operating systems (e.g. Windows XP, Mac OS X) will do this automatically when double-clicking on the file. Alternatively, you can use the free tool 'Stuffit Expander' () or your favourite unpacker. On Linux or Unix systems apply the following command: tar zxf lrr_plots.tar.gz. Please note that some browsers might uncompress the file during download without changing the file ending. If you have trouble unpacking the file try renaming it to lrr_plots.tar and double-click on it again. Unpacking the archive creates a new folder (lrr_plots) in which you can find a file called '00plots.html'. Open this file in a web-browser, either by double-clicking onto it or by using the 'File->Open File' menu (or equivalent) of your browser. This will bring up a web-page with plots of LRR motifs for 372 proteins. If you click on an image you can see the text output from LRRscan in a new window. [file 1471-2164-8-320-S2.gz › lrr_plots/ENSMUSP00000043101.png]

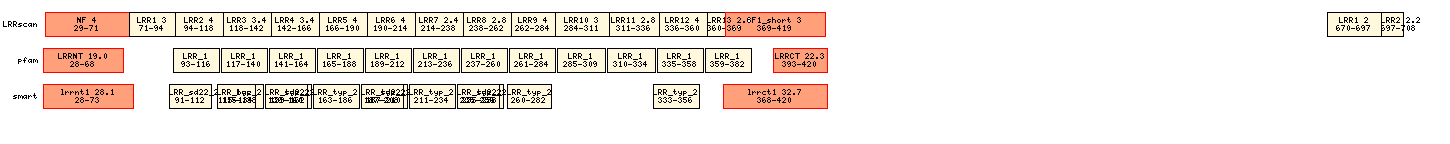

Supplement: Additional file 2 — LRRscan_out.html. Graphical comparison of HMMpfam and LRRscan results. A compressed archive (lrr_plots.tar.gz) containing 372 images in Portable Network Graphics (PNG) format, an information file (00README.txt) and two HTML-formatted pages, one with output from LRRscan (LRRscan_out.html) and one that links all the images together (00plots.html). After downloading, the archive must be to uncompressed and unpacked. Most modern operating systems (e.g. Windows XP, Mac OS X) will do this automatically when double-clicking on the file. Alternatively, you can use the free tool 'Stuffit Expander' () or your favourite unpacker. On Linux or Unix systems apply the following command: tar zxf lrr_plots.tar.gz. Please note that some browsers might uncompress the file during download without changing the file ending. If you have trouble unpacking the file try renaming it to lrr_plots.tar and double-click on it again. Unpacking the archive creates a new folder (lrr_plots) in which you can find a file called '00plots.html'. Open this file in a web-browser, either by double-clicking onto it or by using the 'File->Open File' menu (or equivalent) of your browser. This will bring up a web-page with plots of LRR motifs for 372 proteins. If you click on an image you can see the text output from LRRscan in a new window. [file 1471-2164-8-320-S2.gz › lrr_plots/ENSMUSP00000043818.png]

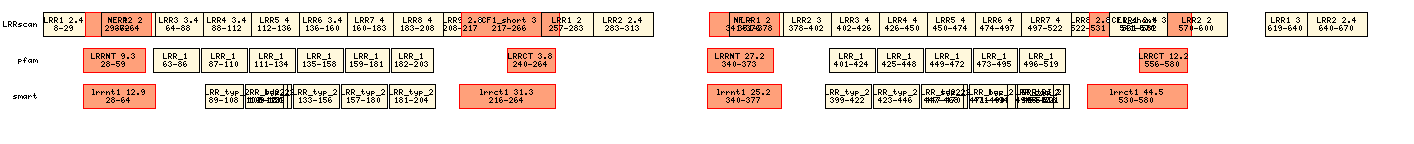

Supplement: Additional file 2 — LRRscan_out.html. Graphical comparison of HMMpfam and LRRscan results. A compressed archive (lrr_plots.tar.gz) containing 372 images in Portable Network Graphics (PNG) format, an information file (00README.txt) and two HTML-formatted pages, one with output from LRRscan (LRRscan_out.html) and one that links all the images together (00plots.html). After downloading, the archive must be to uncompressed and unpacked. Most modern operating systems (e.g. Windows XP, Mac OS X) will do this automatically when double-clicking on the file. Alternatively, you can use the free tool 'Stuffit Expander' () or your favourite unpacker. On Linux or Unix systems apply the following command: tar zxf lrr_plots.tar.gz. Please note that some browsers might uncompress the file during download without changing the file ending. If you have trouble unpacking the file try renaming it to lrr_plots.tar and double-click on it again. Unpacking the archive creates a new folder (lrr_plots) in which you can find a file called '00plots.html'. Open this file in a web-browser, either by double-clicking onto it or by using the 'File->Open File' menu (or equivalent) of your browser. This will bring up a web-page with plots of LRR motifs for 372 proteins. If you click on an image you can see the text output from LRRscan in a new window. [file 1471-2164-8-320-S2.gz › lrr_plots/ENSMUSP00000044094.png]

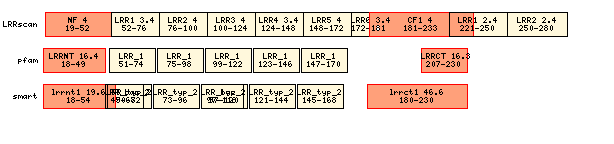

Supplement: Additional file 2 — LRRscan_out.html. Graphical comparison of HMMpfam and LRRscan results. A compressed archive (lrr_plots.tar.gz) containing 372 images in Portable Network Graphics (PNG) format, an information file (00README.txt) and two HTML-formatted pages, one with output from LRRscan (LRRscan_out.html) and one that links all the images together (00plots.html). After downloading, the archive must be to uncompressed and unpacked. Most modern operating systems (e.g. Windows XP, Mac OS X) will do this automatically when double-clicking on the file. Alternatively, you can use the free tool 'Stuffit Expander' () or your favourite unpacker. On Linux or Unix systems apply the following command: tar zxf lrr_plots.tar.gz. Please note that some browsers might uncompress the file during download without changing the file ending. If you have trouble unpacking the file try renaming it to lrr_plots.tar and double-click on it again. Unpacking the archive creates a new folder (lrr_plots) in which you can find a file called '00plots.html'. Open this file in a web-browser, either by double-clicking onto it or by using the 'File->Open File' menu (or equivalent) of your browser. This will bring up a web-page with plots of LRR motifs for 372 proteins. If you click on an image you can see the text output from LRRscan in a new window. [file 1471-2164-8-320-S2.gz › lrr_plots/ENSMUSP00000045142.png]

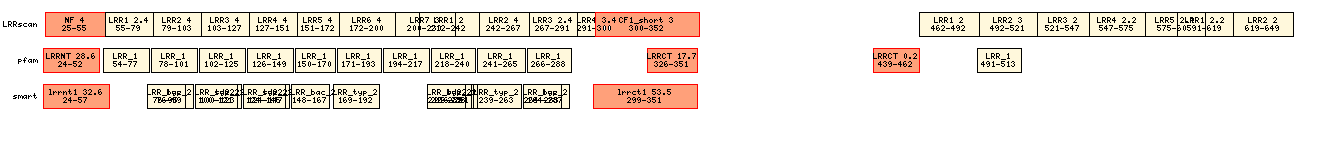

Supplement: Additional file 2 — LRRscan_out.html. Graphical comparison of HMMpfam and LRRscan results. A compressed archive (lrr_plots.tar.gz) containing 372 images in Portable Network Graphics (PNG) format, an information file (00README.txt) and two HTML-formatted pages, one with output from LRRscan (LRRscan_out.html) and one that links all the images together (00plots.html). After downloading, the archive must be to uncompressed and unpacked. Most modern operating systems (e.g. Windows XP, Mac OS X) will do this automatically when double-clicking on the file. Alternatively, you can use the free tool 'Stuffit Expander' () or your favourite unpacker. On Linux or Unix systems apply the following command: tar zxf lrr_plots.tar.gz. Please note that some browsers might uncompress the file during download without changing the file ending. If you have trouble unpacking the file try renaming it to lrr_plots.tar and double-click on it again. Unpacking the archive creates a new folder (lrr_plots) in which you can find a file called '00plots.html'. Open this file in a web-browser, either by double-clicking onto it or by using the 'File->Open File' menu (or equivalent) of your browser. This will bring up a web-page with plots of LRR motifs for 372 proteins. If you click on an image you can see the text output from LRRscan in a new window. [file 1471-2164-8-320-S2.gz › lrr_plots/ENSMUSP00000045162.png]

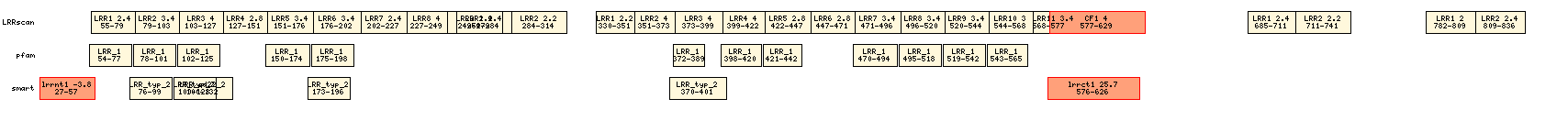

Supplement: Additional file 2 — LRRscan_out.html. Graphical comparison of HMMpfam and LRRscan results. A compressed archive (lrr_plots.tar.gz) containing 372 images in Portable Network Graphics (PNG) format, an information file (00README.txt) and two HTML-formatted pages, one with output from LRRscan (LRRscan_out.html) and one that links all the images together (00plots.html). After downloading, the archive must be to uncompressed and unpacked. Most modern operating systems (e.g. Windows XP, Mac OS X) will do this automatically when double-clicking on the file. Alternatively, you can use the free tool 'Stuffit Expander' () or your favourite unpacker. On Linux or Unix systems apply the following command: tar zxf lrr_plots.tar.gz. Please note that some browsers might uncompress the file during download without changing the file ending. If you have trouble unpacking the file try renaming it to lrr_plots.tar and double-click on it again. Unpacking the archive creates a new folder (lrr_plots) in which you can find a file called '00plots.html'. Open this file in a web-browser, either by double-clicking onto it or by using the 'File->Open File' menu (or equivalent) of your browser. This will bring up a web-page with plots of LRR motifs for 372 proteins. If you click on an image you can see the text output from LRRscan in a new window. [file 1471-2164-8-320-S2.gz › lrr_plots/ENSMUSP00000045770.png]

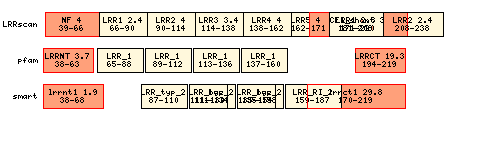

Supplement: Additional file 2 — LRRscan_out.html. Graphical comparison of HMMpfam and LRRscan results. A compressed archive (lrr_plots.tar.gz) containing 372 images in Portable Network Graphics (PNG) format, an information file (00README.txt) and two HTML-formatted pages, one with output from LRRscan (LRRscan_out.html) and one that links all the images together (00plots.html). After downloading, the archive must be to uncompressed and unpacked. Most modern operating systems (e.g. Windows XP, Mac OS X) will do this automatically when double-clicking on the file. Alternatively, you can use the free tool 'Stuffit Expander' () or your favourite unpacker. On Linux or Unix systems apply the following command: tar zxf lrr_plots.tar.gz. Please note that some browsers might uncompress the file during download without changing the file ending. If you have trouble unpacking the file try renaming it to lrr_plots.tar and double-click on it again. Unpacking the archive creates a new folder (lrr_plots) in which you can find a file called '00plots.html'. Open this file in a web-browser, either by double-clicking onto it or by using the 'File->Open File' menu (or equivalent) of your browser. This will bring up a web-page with plots of LRR motifs for 372 proteins. If you click on an image you can see the text output from LRRscan in a new window. [file 1471-2164-8-320-S2.gz › lrr_plots/ENSMUSP00000046705.png]

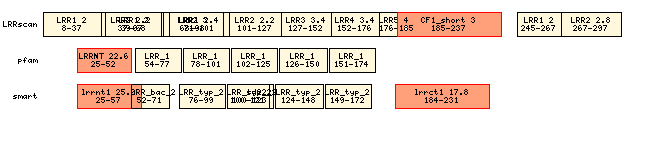

Supplement: Additional file 2 — LRRscan_out.html. Graphical comparison of HMMpfam and LRRscan results. A compressed archive (lrr_plots.tar.gz) containing 372 images in Portable Network Graphics (PNG) format, an information file (00README.txt) and two HTML-formatted pages, one with output from LRRscan (LRRscan_out.html) and one that links all the images together (00plots.html). After downloading, the archive must be to uncompressed and unpacked. Most modern operating systems (e.g. Windows XP, Mac OS X) will do this automatically when double-clicking on the file. Alternatively, you can use the free tool 'Stuffit Expander' () or your favourite unpacker. On Linux or Unix systems apply the following command: tar zxf lrr_plots.tar.gz. Please note that some browsers might uncompress the file during download without changing the file ending. If you have trouble unpacking the file try renaming it to lrr_plots.tar and double-click on it again. Unpacking the archive creates a new folder (lrr_plots) in which you can find a file called '00plots.html'. Open this file in a web-browser, either by double-clicking onto it or by using the 'File->Open File' menu (or equivalent) of your browser. This will bring up a web-page with plots of LRR motifs for 372 proteins. If you click on an image you can see the text output from LRRscan in a new window. [file 1471-2164-8-320-S2.gz › lrr_plots/ENSMUSP00000047213.png]

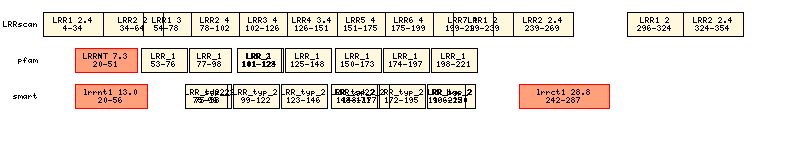

Supplement: Additional file 2 — LRRscan_out.html. Graphical comparison of HMMpfam and LRRscan results. A compressed archive (lrr_plots.tar.gz) containing 372 images in Portable Network Graphics (PNG) format, an information file (00README.txt) and two HTML-formatted pages, one with output from LRRscan (LRRscan_out.html) and one that links all the images together (00plots.html). After downloading, the archive must be to uncompressed and unpacked. Most modern operating systems (e.g. Windows XP, Mac OS X) will do this automatically when double-clicking on the file. Alternatively, you can use the free tool 'Stuffit Expander' () or your favourite unpacker. On Linux or Unix systems apply the following command: tar zxf lrr_plots.tar.gz. Please note that some browsers might uncompress the file during download without changing the file ending. If you have trouble unpacking the file try renaming it to lrr_plots.tar and double-click on it again. Unpacking the archive creates a new folder (lrr_plots) in which you can find a file called '00plots.html'. Open this file in a web-browser, either by double-clicking onto it or by using the 'File->Open File' menu (or equivalent) of your browser. This will bring up a web-page with plots of LRR motifs for 372 proteins. If you click on an image you can see the text output from LRRscan in a new window. [file 1471-2164-8-320-S2.gz › lrr_plots/ENSMUSP00000047573.png]

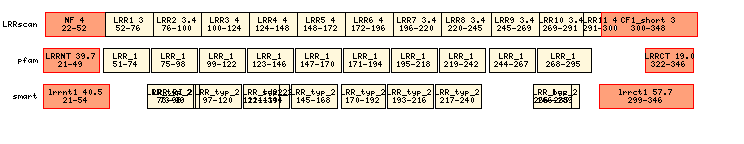

Supplement: Additional file 2 — LRRscan_out.html. Graphical comparison of HMMpfam and LRRscan results. A compressed archive (lrr_plots.tar.gz) containing 372 images in Portable Network Graphics (PNG) format, an information file (00README.txt) and two HTML-formatted pages, one with output from LRRscan (LRRscan_out.html) and one that links all the images together (00plots.html). After downloading, the archive must be to uncompressed and unpacked. Most modern operating systems (e.g. Windows XP, Mac OS X) will do this automatically when double-clicking on the file. Alternatively, you can use the free tool 'Stuffit Expander' () or your favourite unpacker. On Linux or Unix systems apply the following command: tar zxf lrr_plots.tar.gz. Please note that some browsers might uncompress the file during download without changing the file ending. If you have trouble unpacking the file try renaming it to lrr_plots.tar and double-click on it again. Unpacking the archive creates a new folder (lrr_plots) in which you can find a file called '00plots.html'. Open this file in a web-browser, either by double-clicking onto it or by using the 'File->Open File' menu (or equivalent) of your browser. This will bring up a web-page with plots of LRR motifs for 372 proteins. If you click on an image you can see the text output from LRRscan in a new window. [file 1471-2164-8-320-S2.gz › lrr_plots/ENSMUSP00000047844.png]

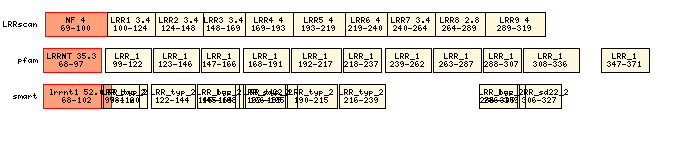

Supplement: Additional file 2 — LRRscan_out.html. Graphical comparison of HMMpfam and LRRscan results. A compressed archive (lrr_plots.tar.gz) containing 372 images in Portable Network Graphics (PNG) format, an information file (00README.txt) and two HTML-formatted pages, one with output from LRRscan (LRRscan_out.html) and one that links all the images together (00plots.html). After downloading, the archive must be to uncompressed and unpacked. Most modern operating systems (e.g. Windows XP, Mac OS X) will do this automatically when double-clicking on the file. Alternatively, you can use the free tool 'Stuffit Expander' () or your favourite unpacker. On Linux or Unix systems apply the following command: tar zxf lrr_plots.tar.gz. Please note that some browsers might uncompress the file during download without changing the file ending. If you have trouble unpacking the file try renaming it to lrr_plots.tar and double-click on it again. Unpacking the archive creates a new folder (lrr_plots) in which you can find a file called '00plots.html'. Open this file in a web-browser, either by double-clicking onto it or by using the 'File->Open File' menu (or equivalent) of your browser. This will bring up a web-page with plots of LRR motifs for 372 proteins. If you click on an image you can see the text output from LRRscan in a new window. [file 1471-2164-8-320-S2.gz › lrr_plots/ENSMUSP00000048803.png]

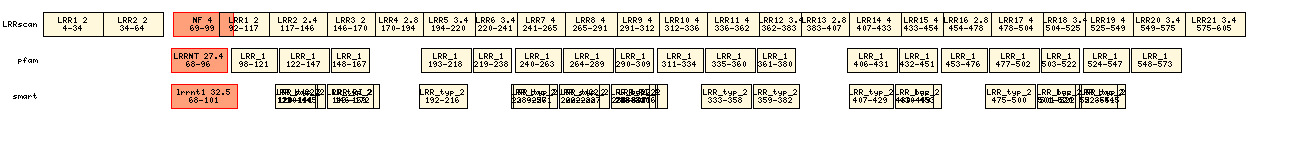

Supplement: Additional file 2 — LRRscan_out.html. Graphical comparison of HMMpfam and LRRscan results. A compressed archive (lrr_plots.tar.gz) containing 372 images in Portable Network Graphics (PNG) format, an information file (00README.txt) and two HTML-formatted pages, one with output from LRRscan (LRRscan_out.html) and one that links all the images together (00plots.html). After downloading, the archive must be to uncompressed and unpacked. Most modern operating systems (e.g. Windows XP, Mac OS X) will do this automatically when double-clicking on the file. Alternatively, you can use the free tool 'Stuffit Expander' () or your favourite unpacker. On Linux or Unix systems apply the following command: tar zxf lrr_plots.tar.gz. Please note that some browsers might uncompress the file during download without changing the file ending. If you have trouble unpacking the file try renaming it to lrr_plots.tar and double-click on it again. Unpacking the archive creates a new folder (lrr_plots) in which you can find a file called '00plots.html'. Open this file in a web-browser, either by double-clicking onto it or by using the 'File->Open File' menu (or equivalent) of your browser. This will bring up a web-page with plots of LRR motifs for 372 proteins. If you click on an image you can see the text output from LRRscan in a new window. [file 1471-2164-8-320-S2.gz › lrr_plots/ENSMUSP00000048962.png]

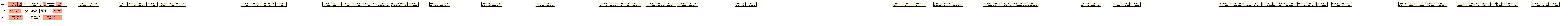

Supplement: Additional file 2 — LRRscan_out.html. Graphical comparison of HMMpfam and LRRscan results. A compressed archive (lrr_plots.tar.gz) containing 372 images in Portable Network Graphics (PNG) format, an information file (00README.txt) and two HTML-formatted pages, one with output from LRRscan (LRRscan_out.html) and one that links all the images together (00plots.html). After downloading, the archive must be to uncompressed and unpacked. Most modern operating systems (e.g. Windows XP, Mac OS X) will do this automatically when double-clicking on the file. Alternatively, you can use the free tool 'Stuffit Expander' () or your favourite unpacker. On Linux or Unix systems apply the following command: tar zxf lrr_plots.tar.gz. Please note that some browsers might uncompress the file during download without changing the file ending. If you have trouble unpacking the file try renaming it to lrr_plots.tar and double-click on it again. Unpacking the archive creates a new folder (lrr_plots) in which you can find a file called '00plots.html'. Open this file in a web-browser, either by double-clicking onto it or by using the 'File->Open File' menu (or equivalent) of your browser. This will bring up a web-page with plots of LRR motifs for 372 proteins. If you click on an image you can see the text output from LRRscan in a new window. [file 1471-2164-8-320-S2.gz › lrr_plots/ENSMUSP00000049296.png]

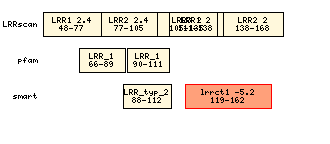

Supplement: Additional file 2 — LRRscan_out.html. Graphical comparison of HMMpfam and LRRscan results. A compressed archive (lrr_plots.tar.gz) containing 372 images in Portable Network Graphics (PNG) format, an information file (00README.txt) and two HTML-formatted pages, one with output from LRRscan (LRRscan_out.html) and one that links all the images together (00plots.html). After downloading, the archive must be to uncompressed and unpacked. Most modern operating systems (e.g. Windows XP, Mac OS X) will do this automatically when double-clicking on the file. Alternatively, you can use the free tool 'Stuffit Expander' () or your favourite unpacker. On Linux or Unix systems apply the following command: tar zxf lrr_plots.tar.gz. Please note that some browsers might uncompress the file during download without changing the file ending. If you have trouble unpacking the file try renaming it to lrr_plots.tar and double-click on it again. Unpacking the archive creates a new folder (lrr_plots) in which you can find a file called '00plots.html'. Open this file in a web-browser, either by double-clicking onto it or by using the 'File->Open File' menu (or equivalent) of your browser. This will bring up a web-page with plots of LRR motifs for 372 proteins. If you click on an image you can see the text output from LRRscan in a new window. [file 1471-2164-8-320-S2.gz › lrr_plots/ENSMUSP00000049686.png]

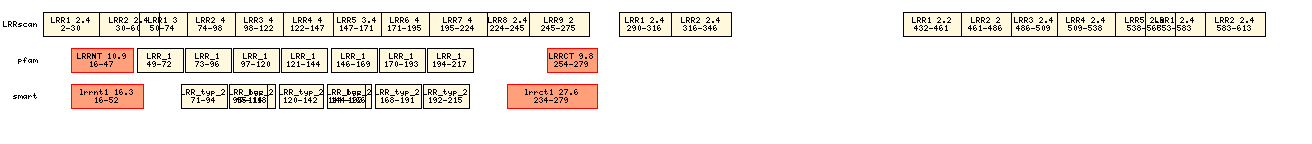

Supplement: Additional file 2 — LRRscan_out.html. Graphical comparison of HMMpfam and LRRscan results. A compressed archive (lrr_plots.tar.gz) containing 372 images in Portable Network Graphics (PNG) format, an information file (00README.txt) and two HTML-formatted pages, one with output from LRRscan (LRRscan_out.html) and one that links all the images together (00plots.html). After downloading, the archive must be to uncompressed and unpacked. Most modern operating systems (e.g. Windows XP, Mac OS X) will do this automatically when double-clicking on the file. Alternatively, you can use the free tool 'Stuffit Expander' () or your favourite unpacker. On Linux or Unix systems apply the following command: tar zxf lrr_plots.tar.gz. Please note that some browsers might uncompress the file during download without changing the file ending. If you have trouble unpacking the file try renaming it to lrr_plots.tar and double-click on it again. Unpacking the archive creates a new folder (lrr_plots) in which you can find a file called '00plots.html'. Open this file in a web-browser, either by double-clicking onto it or by using the 'File->Open File' menu (or equivalent) of your browser. This will bring up a web-page with plots of LRR motifs for 372 proteins. If you click on an image you can see the text output from LRRscan in a new window. [file 1471-2164-8-320-S2.gz › lrr_plots/ENSMUSP00000050039.png]

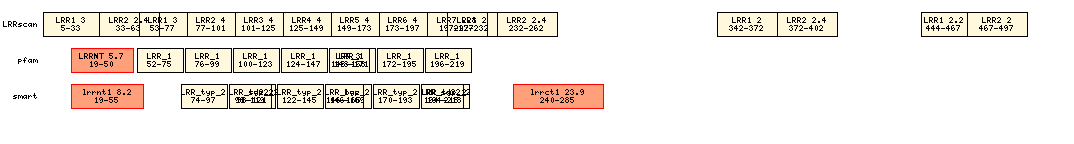

Supplement: Additional file 2 — LRRscan_out.html. Graphical comparison of HMMpfam and LRRscan results. A compressed archive (lrr_plots.tar.gz) containing 372 images in Portable Network Graphics (PNG) format, an information file (00README.txt) and two HTML-formatted pages, one with output from LRRscan (LRRscan_out.html) and one that links all the images together (00plots.html). After downloading, the archive must be to uncompressed and unpacked. Most modern operating systems (e.g. Windows XP, Mac OS X) will do this automatically when double-clicking on the file. Alternatively, you can use the free tool 'Stuffit Expander' () or your favourite unpacker. On Linux or Unix systems apply the following command: tar zxf lrr_plots.tar.gz. Please note that some browsers might uncompress the file during download without changing the file ending. If you have trouble unpacking the file try renaming it to lrr_plots.tar and double-click on it again. Unpacking the archive creates a new folder (lrr_plots) in which you can find a file called '00plots.html'. Open this file in a web-browser, either by double-clicking onto it or by using the 'File->Open File' menu (or equivalent) of your browser. This will bring up a web-page with plots of LRR motifs for 372 proteins. If you click on an image you can see the text output from LRRscan in a new window. [file 1471-2164-8-320-S2.gz › lrr_plots/ENSMUSP00000051546.png]

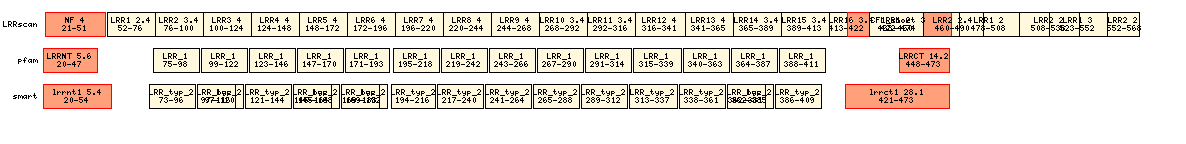

Supplement: Additional file 2 — LRRscan_out.html. Graphical comparison of HMMpfam and LRRscan results. A compressed archive (lrr_plots.tar.gz) containing 372 images in Portable Network Graphics (PNG) format, an information file (00README.txt) and two HTML-formatted pages, one with output from LRRscan (LRRscan_out.html) and one that links all the images together (00plots.html). After downloading, the archive must be to uncompressed and unpacked. Most modern operating systems (e.g. Windows XP, Mac OS X) will do this automatically when double-clicking on the file. Alternatively, you can use the free tool 'Stuffit Expander' () or your favourite unpacker. On Linux or Unix systems apply the following command: tar zxf lrr_plots.tar.gz. Please note that some browsers might uncompress the file during download without changing the file ending. If you have trouble unpacking the file try renaming it to lrr_plots.tar and double-click on it again. Unpacking the archive creates a new folder (lrr_plots) in which you can find a file called '00plots.html'. Open this file in a web-browser, either by double-clicking onto it or by using the 'File->Open File' menu (or equivalent) of your browser. This will bring up a web-page with plots of LRR motifs for 372 proteins. If you click on an image you can see the text output from LRRscan in a new window. [file 1471-2164-8-320-S2.gz › lrr_plots/ENSMUSP00000051895.png]

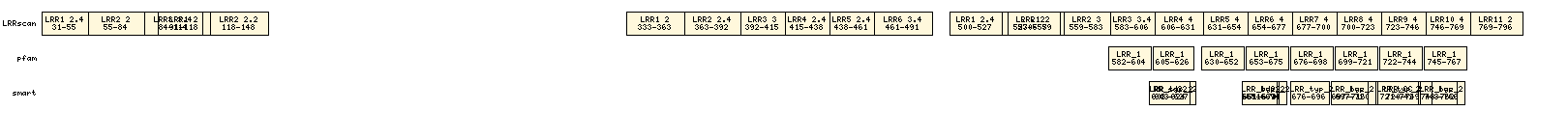

Supplement: Additional file 2 — LRRscan_out.html. Graphical comparison of HMMpfam and LRRscan results. A compressed archive (lrr_plots.tar.gz) containing 372 images in Portable Network Graphics (PNG) format, an information file (00README.txt) and two HTML-formatted pages, one with output from LRRscan (LRRscan_out.html) and one that links all the images together (00plots.html). After downloading, the archive must be to uncompressed and unpacked. Most modern operating systems (e.g. Windows XP, Mac OS X) will do this automatically when double-clicking on the file. Alternatively, you can use the free tool 'Stuffit Expander' () or your favourite unpacker. On Linux or Unix systems apply the following command: tar zxf lrr_plots.tar.gz. Please note that some browsers might uncompress the file during download without changing the file ending. If you have trouble unpacking the file try renaming it to lrr_plots.tar and double-click on it again. Unpacking the archive creates a new folder (lrr_plots) in which you can find a file called '00plots.html'. Open this file in a web-browser, either by double-clicking onto it or by using the 'File->Open File' menu (or equivalent) of your browser. This will bring up a web-page with plots of LRR motifs for 372 proteins. If you click on an image you can see the text output from LRRscan in a new window. [file 1471-2164-8-320-S2.gz › lrr_plots/ENSMUSP00000052055.png]

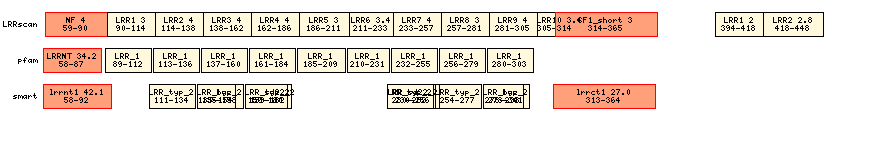

Supplement: Additional file 2 — LRRscan_out.html. Graphical comparison of HMMpfam and LRRscan results. A compressed archive (lrr_plots.tar.gz) containing 372 images in Portable Network Graphics (PNG) format, an information file (00README.txt) and two HTML-formatted pages, one with output from LRRscan (LRRscan_out.html) and one that links all the images together (00plots.html). After downloading, the archive must be to uncompressed and unpacked. Most modern operating systems (e.g. Windows XP, Mac OS X) will do this automatically when double-clicking on the file. Alternatively, you can use the free tool 'Stuffit Expander' () or your favourite unpacker. On Linux or Unix systems apply the following command: tar zxf lrr_plots.tar.gz. Please note that some browsers might uncompress the file during download without changing the file ending. If you have trouble unpacking the file try renaming it to lrr_plots.tar and double-click on it again. Unpacking the archive creates a new folder (lrr_plots) in which you can find a file called '00plots.html'. Open this file in a web-browser, either by double-clicking onto it or by using the 'File->Open File' menu (or equivalent) of your browser. This will bring up a web-page with plots of LRR motifs for 372 proteins. If you click on an image you can see the text output from LRRscan in a new window. [file 1471-2164-8-320-S2.gz › lrr_plots/ENSMUSP00000053123.png]

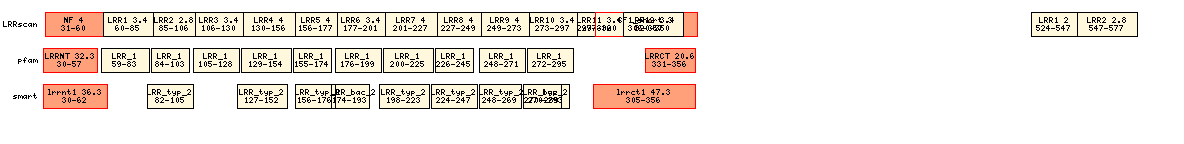

Supplement: Additional file 2 — LRRscan_out.html. Graphical comparison of HMMpfam and LRRscan results. A compressed archive (lrr_plots.tar.gz) containing 372 images in Portable Network Graphics (PNG) format, an information file (00README.txt) and two HTML-formatted pages, one with output from LRRscan (LRRscan_out.html) and one that links all the images together (00plots.html). After downloading, the archive must be to uncompressed and unpacked. Most modern operating systems (e.g. Windows XP, Mac OS X) will do this automatically when double-clicking on the file. Alternatively, you can use the free tool 'Stuffit Expander' () or your favourite unpacker. On Linux or Unix systems apply the following command: tar zxf lrr_plots.tar.gz. Please note that some browsers might uncompress the file during download without changing the file ending. If you have trouble unpacking the file try renaming it to lrr_plots.tar and double-click on it again. Unpacking the archive creates a new folder (lrr_plots) in which you can find a file called '00plots.html'. Open this file in a web-browser, either by double-clicking onto it or by using the 'File->Open File' menu (or equivalent) of your browser. This will bring up a web-page with plots of LRR motifs for 372 proteins. If you click on an image you can see the text output from LRRscan in a new window. [file 1471-2164-8-320-S2.gz › lrr_plots/ENSMUSP00000053399.png]

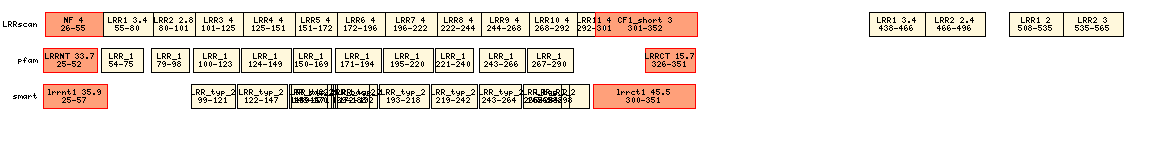

Supplement: Additional file 2 — LRRscan_out.html. Graphical comparison of HMMpfam and LRRscan results. A compressed archive (lrr_plots.tar.gz) containing 372 images in Portable Network Graphics (PNG) format, an information file (00README.txt) and two HTML-formatted pages, one with output from LRRscan (LRRscan_out.html) and one that links all the images together (00plots.html). After downloading, the archive must be to uncompressed and unpacked. Most modern operating systems (e.g. Windows XP, Mac OS X) will do this automatically when double-clicking on the file. Alternatively, you can use the free tool 'Stuffit Expander' () or your favourite unpacker. On Linux or Unix systems apply the following command: tar zxf lrr_plots.tar.gz. Please note that some browsers might uncompress the file during download without changing the file ending. If you have trouble unpacking the file try renaming it to lrr_plots.tar and double-click on it again. Unpacking the archive creates a new folder (lrr_plots) in which you can find a file called '00plots.html'. Open this file in a web-browser, either by double-clicking onto it or by using the 'File->Open File' menu (or equivalent) of your browser. This will bring up a web-page with plots of LRR motifs for 372 proteins. If you click on an image you can see the text output from LRRscan in a new window. [file 1471-2164-8-320-S2.gz › lrr_plots/ENSMUSP00000053573.png]

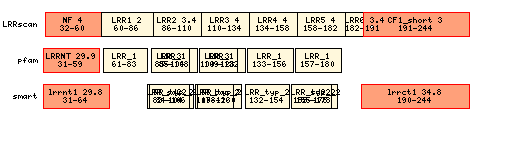

Supplement: Additional file 2 — LRRscan_out.html. Graphical comparison of HMMpfam and LRRscan results. A compressed archive (lrr_plots.tar.gz) containing 372 images in Portable Network Graphics (PNG) format, an information file (00README.txt) and two HTML-formatted pages, one with output from LRRscan (LRRscan_out.html) and one that links all the images together (00plots.html). After downloading, the archive must be to uncompressed and unpacked. Most modern operating systems (e.g. Windows XP, Mac OS X) will do this automatically when double-clicking on the file. Alternatively, you can use the free tool 'Stuffit Expander' () or your favourite unpacker. On Linux or Unix systems apply the following command: tar zxf lrr_plots.tar.gz. Please note that some browsers might uncompress the file during download without changing the file ending. If you have trouble unpacking the file try renaming it to lrr_plots.tar and double-click on it again. Unpacking the archive creates a new folder (lrr_plots) in which you can find a file called '00plots.html'. Open this file in a web-browser, either by double-clicking onto it or by using the 'File->Open File' menu (or equivalent) of your browser. This will bring up a web-page with plots of LRR motifs for 372 proteins. If you click on an image you can see the text output from LRRscan in a new window. [file 1471-2164-8-320-S2.gz › lrr_plots/ENSMUSP00000053597.png]

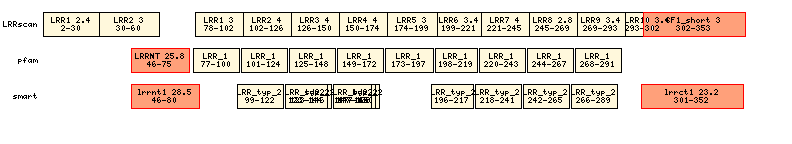

Supplement: Additional file 2 — LRRscan_out.html. Graphical comparison of HMMpfam and LRRscan results. A compressed archive (lrr_plots.tar.gz) containing 372 images in Portable Network Graphics (PNG) format, an information file (00README.txt) and two HTML-formatted pages, one with output from LRRscan (LRRscan_out.html) and one that links all the images together (00plots.html). After downloading, the archive must be to uncompressed and unpacked. Most modern operating systems (e.g. Windows XP, Mac OS X) will do this automatically when double-clicking on the file. Alternatively, you can use the free tool 'Stuffit Expander' () or your favourite unpacker. On Linux or Unix systems apply the following command: tar zxf lrr_plots.tar.gz. Please note that some browsers might uncompress the file during download without changing the file ending. If you have trouble unpacking the file try renaming it to lrr_plots.tar and double-click on it again. Unpacking the archive creates a new folder (lrr_plots) in which you can find a file called '00plots.html'. Open this file in a web-browser, either by double-clicking onto it or by using the 'File->Open File' menu (or equivalent) of your browser. This will bring up a web-page with plots of LRR motifs for 372 proteins. If you click on an image you can see the text output from LRRscan in a new window. [file 1471-2164-8-320-S2.gz › lrr_plots/ENSMUSP00000053840.png]

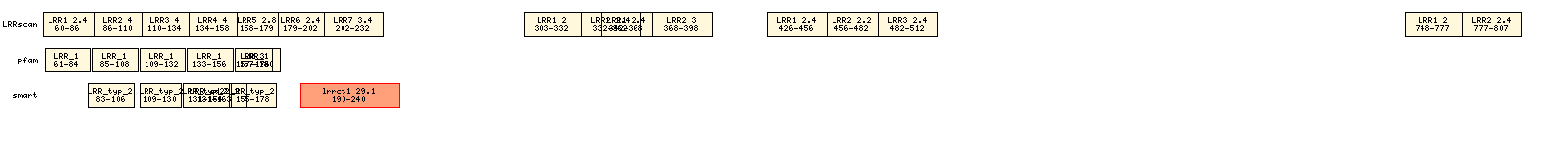

Supplement: Additional file 2 — LRRscan_out.html. Graphical comparison of HMMpfam and LRRscan results. A compressed archive (lrr_plots.tar.gz) containing 372 images in Portable Network Graphics (PNG) format, an information file (00README.txt) and two HTML-formatted pages, one with output from LRRscan (LRRscan_out.html) and one that links all the images together (00plots.html). After downloading, the archive must be to uncompressed and unpacked. Most modern operating systems (e.g. Windows XP, Mac OS X) will do this automatically when double-clicking on the file. Alternatively, you can use the free tool 'Stuffit Expander' () or your favourite unpacker. On Linux or Unix systems apply the following command: tar zxf lrr_plots.tar.gz. Please note that some browsers might uncompress the file during download without changing the file ending. If you have trouble unpacking the file try renaming it to lrr_plots.tar and double-click on it again. Unpacking the archive creates a new folder (lrr_plots) in which you can find a file called '00plots.html'. Open this file in a web-browser, either by double-clicking onto it or by using the 'File->Open File' menu (or equivalent) of your browser. This will bring up a web-page with plots of LRR motifs for 372 proteins. If you click on an image you can see the text output from LRRscan in a new window. [file 1471-2164-8-320-S2.gz › lrr_plots/ENSMUSP00000053869.png]

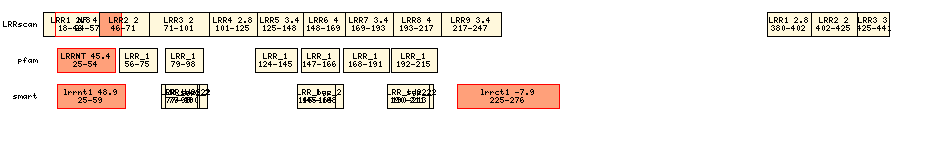

Supplement: Additional file 2 — LRRscan_out.html. Graphical comparison of HMMpfam and LRRscan results. A compressed archive (lrr_plots.tar.gz) containing 372 images in Portable Network Graphics (PNG) format, an information file (00README.txt) and two HTML-formatted pages, one with output from LRRscan (LRRscan_out.html) and one that links all the images together (00plots.html). After downloading, the archive must be to uncompressed and unpacked. Most modern operating systems (e.g. Windows XP, Mac OS X) will do this automatically when double-clicking on the file. Alternatively, you can use the free tool 'Stuffit Expander' () or your favourite unpacker. On Linux or Unix systems apply the following command: tar zxf lrr_plots.tar.gz. Please note that some browsers might uncompress the file during download without changing the file ending. If you have trouble unpacking the file try renaming it to lrr_plots.tar and double-click on it again. Unpacking the archive creates a new folder (lrr_plots) in which you can find a file called '00plots.html'. Open this file in a web-browser, either by double-clicking onto it or by using the 'File->Open File' menu (or equivalent) of your browser. This will bring up a web-page with plots of LRR motifs for 372 proteins. If you click on an image you can see the text output from LRRscan in a new window. [file 1471-2164-8-320-S2.gz › lrr_plots/ENSMUSP00000054140.png]

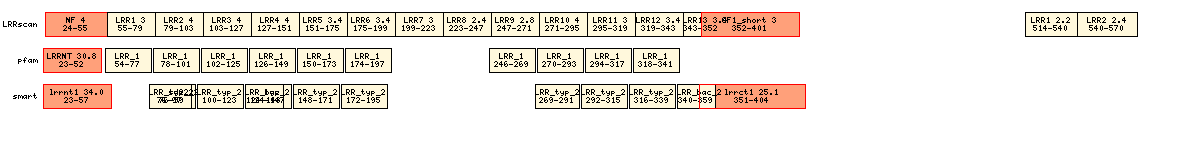

Supplement: Additional file 2 — LRRscan_out.html. Graphical comparison of HMMpfam and LRRscan results. A compressed archive (lrr_plots.tar.gz) containing 372 images in Portable Network Graphics (PNG) format, an information file (00README.txt) and two HTML-formatted pages, one with output from LRRscan (LRRscan_out.html) and one that links all the images together (00plots.html). After downloading, the archive must be to uncompressed and unpacked. Most modern operating systems (e.g. Windows XP, Mac OS X) will do this automatically when double-clicking on the file. Alternatively, you can use the free tool 'Stuffit Expander' () or your favourite unpacker. On Linux or Unix systems apply the following command: tar zxf lrr_plots.tar.gz. Please note that some browsers might uncompress the file during download without changing the file ending. If you have trouble unpacking the file try renaming it to lrr_plots.tar and double-click on it again. Unpacking the archive creates a new folder (lrr_plots) in which you can find a file called '00plots.html'. Open this file in a web-browser, either by double-clicking onto it or by using the 'File->Open File' menu (or equivalent) of your browser. This will bring up a web-page with plots of LRR motifs for 372 proteins. If you click on an image you can see the text output from LRRscan in a new window. [file 1471-2164-8-320-S2.gz › lrr_plots/ENSMUSP00000054960.png]

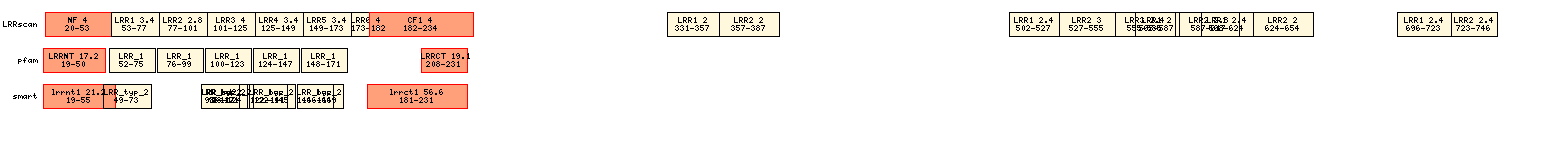

Supplement: Additional file 2 — LRRscan_out.html. Graphical comparison of HMMpfam and LRRscan results. A compressed archive (lrr_plots.tar.gz) containing 372 images in Portable Network Graphics (PNG) format, an information file (00README.txt) and two HTML-formatted pages, one with output from LRRscan (LRRscan_out.html) and one that links all the images together (00plots.html). After downloading, the archive must be to uncompressed and unpacked. Most modern operating systems (e.g. Windows XP, Mac OS X) will do this automatically when double-clicking on the file. Alternatively, you can use the free tool 'Stuffit Expander' () or your favourite unpacker. On Linux or Unix systems apply the following command: tar zxf lrr_plots.tar.gz. Please note that some browsers might uncompress the file during download without changing the file ending. If you have trouble unpacking the file try renaming it to lrr_plots.tar and double-click on it again. Unpacking the archive creates a new folder (lrr_plots) in which you can find a file called '00plots.html'. Open this file in a web-browser, either by double-clicking onto it or by using the 'File->Open File' menu (or equivalent) of your browser. This will bring up a web-page with plots of LRR motifs for 372 proteins. If you click on an image you can see the text output from LRRscan in a new window. [file 1471-2164-8-320-S2.gz › lrr_plots/ENSMUSP00000055604.png]

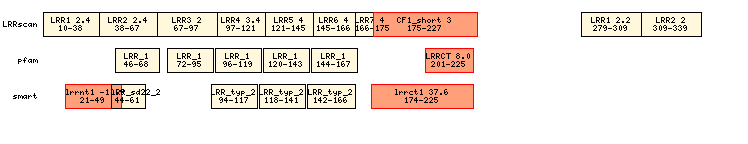

Supplement: Additional file 2 — LRRscan_out.html. Graphical comparison of HMMpfam and LRRscan results. A compressed archive (lrr_plots.tar.gz) containing 372 images in Portable Network Graphics (PNG) format, an information file (00README.txt) and two HTML-formatted pages, one with output from LRRscan (LRRscan_out.html) and one that links all the images together (00plots.html). After downloading, the archive must be to uncompressed and unpacked. Most modern operating systems (e.g. Windows XP, Mac OS X) will do this automatically when double-clicking on the file. Alternatively, you can use the free tool 'Stuffit Expander' () or your favourite unpacker. On Linux or Unix systems apply the following command: tar zxf lrr_plots.tar.gz. Please note that some browsers might uncompress the file during download without changing the file ending. If you have trouble unpacking the file try renaming it to lrr_plots.tar and double-click on it again. Unpacking the archive creates a new folder (lrr_plots) in which you can find a file called '00plots.html'. Open this file in a web-browser, either by double-clicking onto it or by using the 'File->Open File' menu (or equivalent) of your browser. This will bring up a web-page with plots of LRR motifs for 372 proteins. If you click on an image you can see the text output from LRRscan in a new window. [file 1471-2164-8-320-S2.gz › lrr_plots/ENSMUSP00000056094.png]

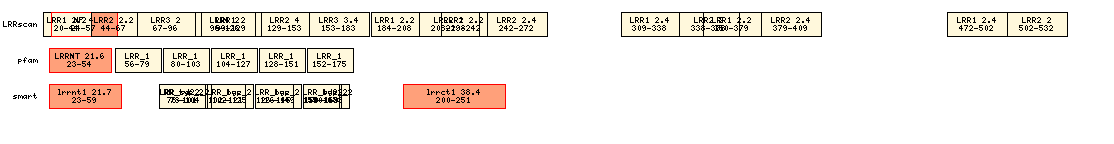

Supplement: Additional file 2 — LRRscan_out.html. Graphical comparison of HMMpfam and LRRscan results. A compressed archive (lrr_plots.tar.gz) containing 372 images in Portable Network Graphics (PNG) format, an information file (00README.txt) and two HTML-formatted pages, one with output from LRRscan (LRRscan_out.html) and one that links all the images together (00plots.html). After downloading, the archive must be to uncompressed and unpacked. Most modern operating systems (e.g. Windows XP, Mac OS X) will do this automatically when double-clicking on the file. Alternatively, you can use the free tool 'Stuffit Expander' () or your favourite unpacker. On Linux or Unix systems apply the following command: tar zxf lrr_plots.tar.gz. Please note that some browsers might uncompress the file during download without changing the file ending. If you have trouble unpacking the file try renaming it to lrr_plots.tar and double-click on it again. Unpacking the archive creates a new folder (lrr_plots) in which you can find a file called '00plots.html'. Open this file in a web-browser, either by double-clicking onto it or by using the 'File->Open File' menu (or equivalent) of your browser. This will bring up a web-page with plots of LRR motifs for 372 proteins. If you click on an image you can see the text output from LRRscan in a new window. [file 1471-2164-8-320-S2.gz › lrr_plots/ENSMUSP00000056642.png]

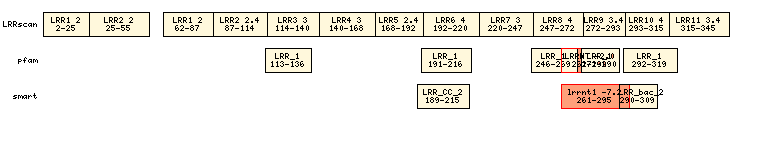

Supplement: Additional file 2 — LRRscan_out.html. Graphical comparison of HMMpfam and LRRscan results. A compressed archive (lrr_plots.tar.gz) containing 372 images in Portable Network Graphics (PNG) format, an information file (00README.txt) and two HTML-formatted pages, one with output from LRRscan (LRRscan_out.html) and one that links all the images together (00plots.html). After downloading, the archive must be to uncompressed and unpacked. Most modern operating systems (e.g. Windows XP, Mac OS X) will do this automatically when double-clicking on the file. Alternatively, you can use the free tool 'Stuffit Expander' () or your favourite unpacker. On Linux or Unix systems apply the following command: tar zxf lrr_plots.tar.gz. Please note that some browsers might uncompress the file during download without changing the file ending. If you have trouble unpacking the file try renaming it to lrr_plots.tar and double-click on it again. Unpacking the archive creates a new folder (lrr_plots) in which you can find a file called '00plots.html'. Open this file in a web-browser, either by double-clicking onto it or by using the 'File->Open File' menu (or equivalent) of your browser. This will bring up a web-page with plots of LRR motifs for 372 proteins. If you click on an image you can see the text output from LRRscan in a new window. [file 1471-2164-8-320-S2.gz › lrr_plots/ENSMUSP00000056669.png]

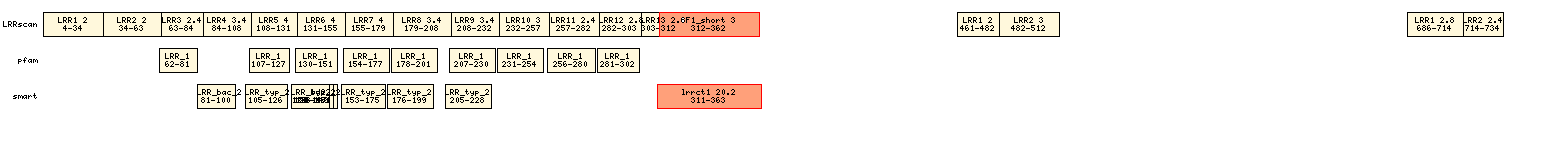

Supplement: Additional file 2 — LRRscan_out.html. Graphical comparison of HMMpfam and LRRscan results. A compressed archive (lrr_plots.tar.gz) containing 372 images in Portable Network Graphics (PNG) format, an information file (00README.txt) and two HTML-formatted pages, one with output from LRRscan (LRRscan_out.html) and one that links all the images together (00plots.html). After downloading, the archive must be to uncompressed and unpacked. Most modern operating systems (e.g. Windows XP, Mac OS X) will do this automatically when double-clicking on the file. Alternatively, you can use the free tool 'Stuffit Expander' () or your favourite unpacker. On Linux or Unix systems apply the following command: tar zxf lrr_plots.tar.gz. Please note that some browsers might uncompress the file during download without changing the file ending. If you have trouble unpacking the file try renaming it to lrr_plots.tar and double-click on it again. Unpacking the archive creates a new folder (lrr_plots) in which you can find a file called '00plots.html'. Open this file in a web-browser, either by double-clicking onto it or by using the 'File->Open File' menu (or equivalent) of your browser. This will bring up a web-page with plots of LRR motifs for 372 proteins. If you click on an image you can see the text output from LRRscan in a new window. [file 1471-2164-8-320-S2.gz › lrr_plots/ENSMUSP00000057005.png]

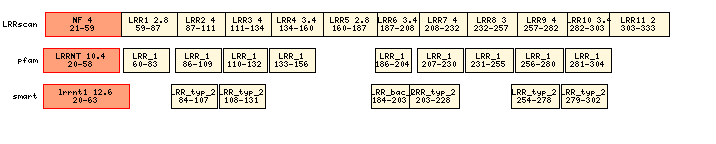

Supplement: Additional file 2 — LRRscan_out.html. Graphical comparison of HMMpfam and LRRscan results. A compressed archive (lrr_plots.tar.gz) containing 372 images in Portable Network Graphics (PNG) format, an information file (00README.txt) and two HTML-formatted pages, one with output from LRRscan (LRRscan_out.html) and one that links all the images together (00plots.html). After downloading, the archive must be to uncompressed and unpacked. Most modern operating systems (e.g. Windows XP, Mac OS X) will do this automatically when double-clicking on the file. Alternatively, you can use the free tool 'Stuffit Expander' () or your favourite unpacker. On Linux or Unix systems apply the following command: tar zxf lrr_plots.tar.gz. Please note that some browsers might uncompress the file during download without changing the file ending. If you have trouble unpacking the file try renaming it to lrr_plots.tar and double-click on it again. Unpacking the archive creates a new folder (lrr_plots) in which you can find a file called '00plots.html'. Open this file in a web-browser, either by double-clicking onto it or by using the 'File->Open File' menu (or equivalent) of your browser. This will bring up a web-page with plots of LRR motifs for 372 proteins. If you click on an image you can see the text output from LRRscan in a new window. [file 1471-2164-8-320-S2.gz › lrr_plots/ENSMUSP00000057071.png]

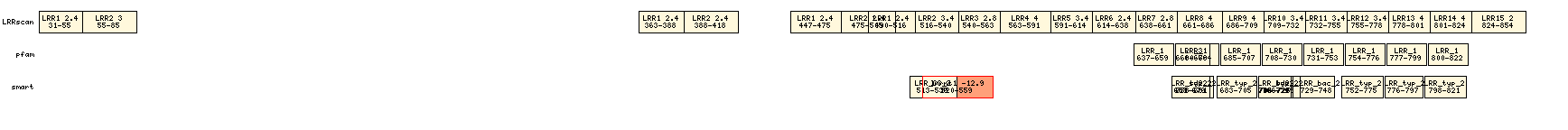

Supplement: Additional file 2 — LRRscan_out.html. Graphical comparison of HMMpfam and LRRscan results. A compressed archive (lrr_plots.tar.gz) containing 372 images in Portable Network Graphics (PNG) format, an information file (00README.txt) and two HTML-formatted pages, one with output from LRRscan (LRRscan_out.html) and one that links all the images together (00plots.html). After downloading, the archive must be to uncompressed and unpacked. Most modern operating systems (e.g. Windows XP, Mac OS X) will do this automatically when double-clicking on the file. Alternatively, you can use the free tool 'Stuffit Expander' () or your favourite unpacker. On Linux or Unix systems apply the following command: tar zxf lrr_plots.tar.gz. Please note that some browsers might uncompress the file during download without changing the file ending. If you have trouble unpacking the file try renaming it to lrr_plots.tar and double-click on it again. Unpacking the archive creates a new folder (lrr_plots) in which you can find a file called '00plots.html'. Open this file in a web-browser, either by double-clicking onto it or by using the 'File->Open File' menu (or equivalent) of your browser. This will bring up a web-page with plots of LRR motifs for 372 proteins. If you click on an image you can see the text output from LRRscan in a new window. [file 1471-2164-8-320-S2.gz › lrr_plots/ENSMUSP00000057293.png]

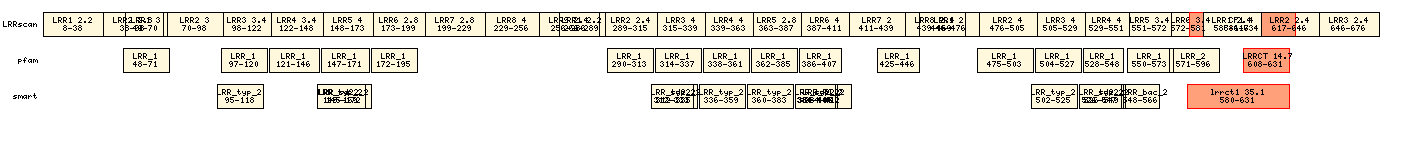

Supplement: Additional file 2 — LRRscan_out.html. Graphical comparison of HMMpfam and LRRscan results. A compressed archive (lrr_plots.tar.gz) containing 372 images in Portable Network Graphics (PNG) format, an information file (00README.txt) and two HTML-formatted pages, one with output from LRRscan (LRRscan_out.html) and one that links all the images together (00plots.html). After downloading, the archive must be to uncompressed and unpacked. Most modern operating systems (e.g. Windows XP, Mac OS X) will do this automatically when double-clicking on the file. Alternatively, you can use the free tool 'Stuffit Expander' () or your favourite unpacker. On Linux or Unix systems apply the following command: tar zxf lrr_plots.tar.gz. Please note that some browsers might uncompress the file during download without changing the file ending. If you have trouble unpacking the file try renaming it to lrr_plots.tar and double-click on it again. Unpacking the archive creates a new folder (lrr_plots) in which you can find a file called '00plots.html'. Open this file in a web-browser, either by double-clicking onto it or by using the 'File->Open File' menu (or equivalent) of your browser. This will bring up a web-page with plots of LRR motifs for 372 proteins. If you click on an image you can see the text output from LRRscan in a new window. [file 1471-2164-8-320-S2.gz › lrr_plots/ENSMUSP00000057529.png]

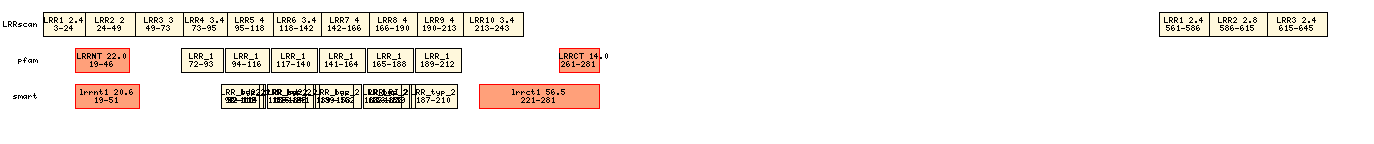

Supplement: Additional file 2 — LRRscan_out.html. Graphical comparison of HMMpfam and LRRscan results. A compressed archive (lrr_plots.tar.gz) containing 372 images in Portable Network Graphics (PNG) format, an information file (00README.txt) and two HTML-formatted pages, one with output from LRRscan (LRRscan_out.html) and one that links all the images together (00plots.html). After downloading, the archive must be to uncompressed and unpacked. Most modern operating systems (e.g. Windows XP, Mac OS X) will do this automatically when double-clicking on the file. Alternatively, you can use the free tool 'Stuffit Expander' () or your favourite unpacker. On Linux or Unix systems apply the following command: tar zxf lrr_plots.tar.gz. Please note that some browsers might uncompress the file during download without changing the file ending. If you have trouble unpacking the file try renaming it to lrr_plots.tar and double-click on it again. Unpacking the archive creates a new folder (lrr_plots) in which you can find a file called '00plots.html'. Open this file in a web-browser, either by double-clicking onto it or by using the 'File->Open File' menu (or equivalent) of your browser. This will bring up a web-page with plots of LRR motifs for 372 proteins. If you click on an image you can see the text output from LRRscan in a new window. [file 1471-2164-8-320-S2.gz › lrr_plots/ENSMUSP00000057563.png]

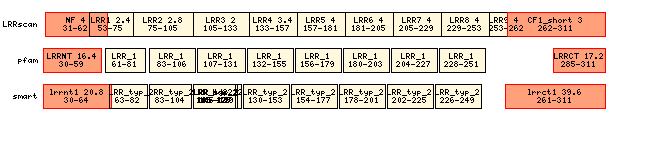

Supplement: Additional file 2 — LRRscan_out.html. Graphical comparison of HMMpfam and LRRscan results. A compressed archive (lrr_plots.tar.gz) containing 372 images in Portable Network Graphics (PNG) format, an information file (00README.txt) and two HTML-formatted pages, one with output from LRRscan (LRRscan_out.html) and one that links all the images together (00plots.html). After downloading, the archive must be to uncompressed and unpacked. Most modern operating systems (e.g. Windows XP, Mac OS X) will do this automatically when double-clicking on the file. Alternatively, you can use the free tool 'Stuffit Expander' () or your favourite unpacker. On Linux or Unix systems apply the following command: tar zxf lrr_plots.tar.gz. Please note that some browsers might uncompress the file during download without changing the file ending. If you have trouble unpacking the file try renaming it to lrr_plots.tar and double-click on it again. Unpacking the archive creates a new folder (lrr_plots) in which you can find a file called '00plots.html'. Open this file in a web-browser, either by double-clicking onto it or by using the 'File->Open File' menu (or equivalent) of your browser. This will bring up a web-page with plots of LRR motifs for 372 proteins. If you click on an image you can see the text output from LRRscan in a new window. [file 1471-2164-8-320-S2.gz › lrr_plots/ENSMUSP00000057725.png]

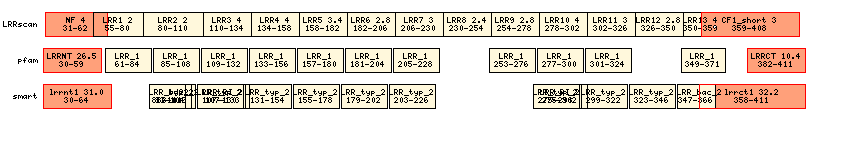

Supplement: Additional file 2 — LRRscan_out.html. Graphical comparison of HMMpfam and LRRscan results. A compressed archive (lrr_plots.tar.gz) containing 372 images in Portable Network Graphics (PNG) format, an information file (00README.txt) and two HTML-formatted pages, one with output from LRRscan (LRRscan_out.html) and one that links all the images together (00plots.html). After downloading, the archive must be to uncompressed and unpacked. Most modern operating systems (e.g. Windows XP, Mac OS X) will do this automatically when double-clicking on the file. Alternatively, you can use the free tool 'Stuffit Expander' () or your favourite unpacker. On Linux or Unix systems apply the following command: tar zxf lrr_plots.tar.gz. Please note that some browsers might uncompress the file during download without changing the file ending. If you have trouble unpacking the file try renaming it to lrr_plots.tar and double-click on it again. Unpacking the archive creates a new folder (lrr_plots) in which you can find a file called '00plots.html'. Open this file in a web-browser, either by double-clicking onto it or by using the 'File->Open File' menu (or equivalent) of your browser. This will bring up a web-page with plots of LRR motifs for 372 proteins. If you click on an image you can see the text output from LRRscan in a new window. [file 1471-2164-8-320-S2.gz › lrr_plots/ENSMUSP00000058050.png]

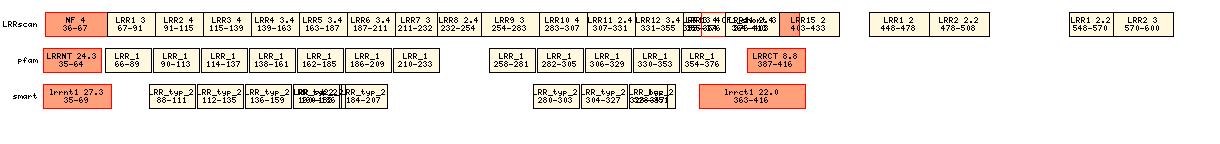

Supplement: Additional file 2 — LRRscan_out.html. Graphical comparison of HMMpfam and LRRscan results. A compressed archive (lrr_plots.tar.gz) containing 372 images in Portable Network Graphics (PNG) format, an information file (00README.txt) and two HTML-formatted pages, one with output from LRRscan (LRRscan_out.html) and one that links all the images together (00plots.html). After downloading, the archive must be to uncompressed and unpacked. Most modern operating systems (e.g. Windows XP, Mac OS X) will do this automatically when double-clicking on the file. Alternatively, you can use the free tool 'Stuffit Expander' () or your favourite unpacker. On Linux or Unix systems apply the following command: tar zxf lrr_plots.tar.gz. Please note that some browsers might uncompress the file during download without changing the file ending. If you have trouble unpacking the file try renaming it to lrr_plots.tar and double-click on it again. Unpacking the archive creates a new folder (lrr_plots) in which you can find a file called '00plots.html'. Open this file in a web-browser, either by double-clicking onto it or by using the 'File->Open File' menu (or equivalent) of your browser. This will bring up a web-page with plots of LRR motifs for 372 proteins. If you click on an image you can see the text output from LRRscan in a new window. [file 1471-2164-8-320-S2.gz › lrr_plots/ENSMUSP00000059050.png]

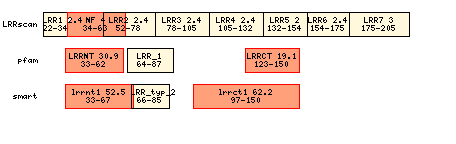

Supplement: Additional file 2 — LRRscan_out.html. Graphical comparison of HMMpfam and LRRscan results. A compressed archive (lrr_plots.tar.gz) containing 372 images in Portable Network Graphics (PNG) format, an information file (00README.txt) and two HTML-formatted pages, one with output from LRRscan (LRRscan_out.html) and one that links all the images together (00plots.html). After downloading, the archive must be to uncompressed and unpacked. Most modern operating systems (e.g. Windows XP, Mac OS X) will do this automatically when double-clicking on the file. Alternatively, you can use the free tool 'Stuffit Expander' () or your favourite unpacker. On Linux or Unix systems apply the following command: tar zxf lrr_plots.tar.gz. Please note that some browsers might uncompress the file during download without changing the file ending. If you have trouble unpacking the file try renaming it to lrr_plots.tar and double-click on it again. Unpacking the archive creates a new folder (lrr_plots) in which you can find a file called '00plots.html'. Open this file in a web-browser, either by double-clicking onto it or by using the 'File->Open File' menu (or equivalent) of your browser. This will bring up a web-page with plots of LRR motifs for 372 proteins. If you click on an image you can see the text output from LRRscan in a new window. [file 1471-2164-8-320-S2.gz › lrr_plots/ENSMUSP00000059270.png]

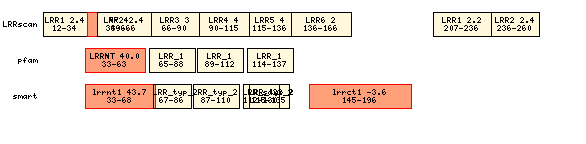

Supplement: Additional file 2 — LRRscan_out.html. Graphical comparison of HMMpfam and LRRscan results. A compressed archive (lrr_plots.tar.gz) containing 372 images in Portable Network Graphics (PNG) format, an information file (00README.txt) and two HTML-formatted pages, one with output from LRRscan (LRRscan_out.html) and one that links all the images together (00plots.html). After downloading, the archive must be to uncompressed and unpacked. Most modern operating systems (e.g. Windows XP, Mac OS X) will do this automatically when double-clicking on the file. Alternatively, you can use the free tool 'Stuffit Expander' () or your favourite unpacker. On Linux or Unix systems apply the following command: tar zxf lrr_plots.tar.gz. Please note that some browsers might uncompress the file during download without changing the file ending. If you have trouble unpacking the file try renaming it to lrr_plots.tar and double-click on it again. Unpacking the archive creates a new folder (lrr_plots) in which you can find a file called '00plots.html'. Open this file in a web-browser, either by double-clicking onto it or by using the 'File->Open File' menu (or equivalent) of your browser. This will bring up a web-page with plots of LRR motifs for 372 proteins. If you click on an image you can see the text output from LRRscan in a new window. [file 1471-2164-8-320-S2.gz › lrr_plots/ENSMUSP00000059463.png]

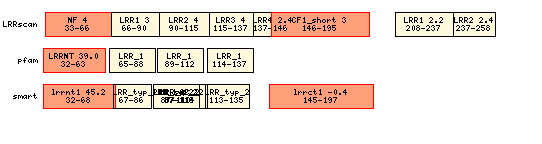

Supplement: Additional file 2 — LRRscan_out.html. Graphical comparison of HMMpfam and LRRscan results. A compressed archive (lrr_plots.tar.gz) containing 372 images in Portable Network Graphics (PNG) format, an information file (00README.txt) and two HTML-formatted pages, one with output from LRRscan (LRRscan_out.html) and one that links all the images together (00plots.html). After downloading, the archive must be to uncompressed and unpacked. Most modern operating systems (e.g. Windows XP, Mac OS X) will do this automatically when double-clicking on the file. Alternatively, you can use the free tool 'Stuffit Expander' () or your favourite unpacker. On Linux or Unix systems apply the following command: tar zxf lrr_plots.tar.gz. Please note that some browsers might uncompress the file during download without changing the file ending. If you have trouble unpacking the file try renaming it to lrr_plots.tar and double-click on it again. Unpacking the archive creates a new folder (lrr_plots) in which you can find a file called '00plots.html'. Open this file in a web-browser, either by double-clicking onto it or by using the 'File->Open File' menu (or equivalent) of your browser. This will bring up a web-page with plots of LRR motifs for 372 proteins. If you click on an image you can see the text output from LRRscan in a new window. [file 1471-2164-8-320-S2.gz › lrr_plots/ENSMUSP00000059570.png]

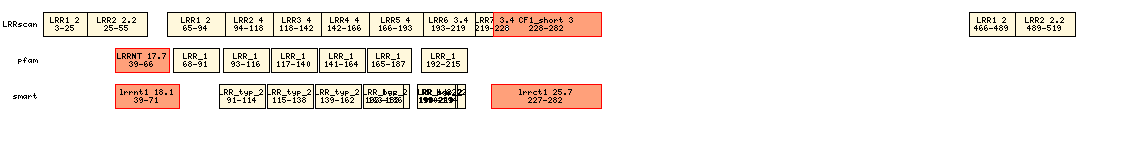

Supplement: Additional file 2 — LRRscan_out.html. Graphical comparison of HMMpfam and LRRscan results. A compressed archive (lrr_plots.tar.gz) containing 372 images in Portable Network Graphics (PNG) format, an information file (00README.txt) and two HTML-formatted pages, one with output from LRRscan (LRRscan_out.html) and one that links all the images together (00plots.html). After downloading, the archive must be to uncompressed and unpacked. Most modern operating systems (e.g. Windows XP, Mac OS X) will do this automatically when double-clicking on the file. Alternatively, you can use the free tool 'Stuffit Expander' () or your favourite unpacker. On Linux or Unix systems apply the following command: tar zxf lrr_plots.tar.gz. Please note that some browsers might uncompress the file during download without changing the file ending. If you have trouble unpacking the file try renaming it to lrr_plots.tar and double-click on it again. Unpacking the archive creates a new folder (lrr_plots) in which you can find a file called '00plots.html'. Open this file in a web-browser, either by double-clicking onto it or by using the 'File->Open File' menu (or equivalent) of your browser. This will bring up a web-page with plots of LRR motifs for 372 proteins. If you click on an image you can see the text output from LRRscan in a new window. [file 1471-2164-8-320-S2.gz › lrr_plots/ENSMUSP00000059913.png]

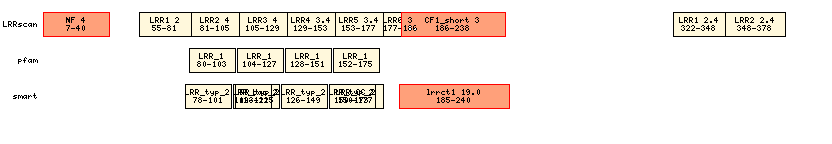

Supplement: Additional file 2 — LRRscan_out.html. Graphical comparison of HMMpfam and LRRscan results. A compressed archive (lrr_plots.tar.gz) containing 372 images in Portable Network Graphics (PNG) format, an information file (00README.txt) and two HTML-formatted pages, one with output from LRRscan (LRRscan_out.html) and one that links all the images together (00plots.html). After downloading, the archive must be to uncompressed and unpacked. Most modern operating systems (e.g. Windows XP, Mac OS X) will do this automatically when double-clicking on the file. Alternatively, you can use the free tool 'Stuffit Expander' () or your favourite unpacker. On Linux or Unix systems apply the following command: tar zxf lrr_plots.tar.gz. Please note that some browsers might uncompress the file during download without changing the file ending. If you have trouble unpacking the file try renaming it to lrr_plots.tar and double-click on it again. Unpacking the archive creates a new folder (lrr_plots) in which you can find a file called '00plots.html'. Open this file in a web-browser, either by double-clicking onto it or by using the 'File->Open File' menu (or equivalent) of your browser. This will bring up a web-page with plots of LRR motifs for 372 proteins. If you click on an image you can see the text output from LRRscan in a new window. [file 1471-2164-8-320-S2.gz › lrr_plots/ENSMUSP00000060210.png]

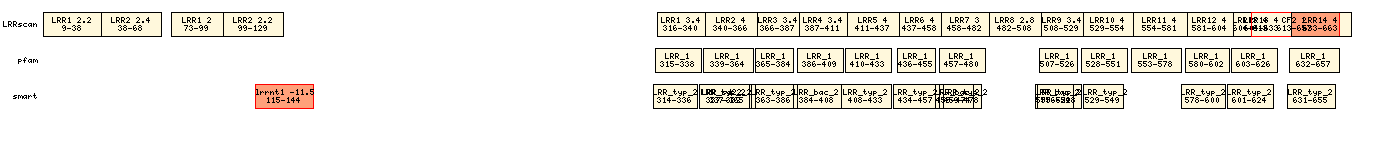

Supplement: Additional file 2 — LRRscan_out.html. Graphical comparison of HMMpfam and LRRscan results. A compressed archive (lrr_plots.tar.gz) containing 372 images in Portable Network Graphics (PNG) format, an information file (00README.txt) and two HTML-formatted pages, one with output from LRRscan (LRRscan_out.html) and one that links all the images together (00plots.html). After downloading, the archive must be to uncompressed and unpacked. Most modern operating systems (e.g. Windows XP, Mac OS X) will do this automatically when double-clicking on the file. Alternatively, you can use the free tool 'Stuffit Expander' () or your favourite unpacker. On Linux or Unix systems apply the following command: tar zxf lrr_plots.tar.gz. Please note that some browsers might uncompress the file during download without changing the file ending. If you have trouble unpacking the file try renaming it to lrr_plots.tar and double-click on it again. Unpacking the archive creates a new folder (lrr_plots) in which you can find a file called '00plots.html'. Open this file in a web-browser, either by double-clicking onto it or by using the 'File->Open File' menu (or equivalent) of your browser. This will bring up a web-page with plots of LRR motifs for 372 proteins. If you click on an image you can see the text output from LRRscan in a new window. [file 1471-2164-8-320-S2.gz › lrr_plots/ENSMUSP00000060402.png]

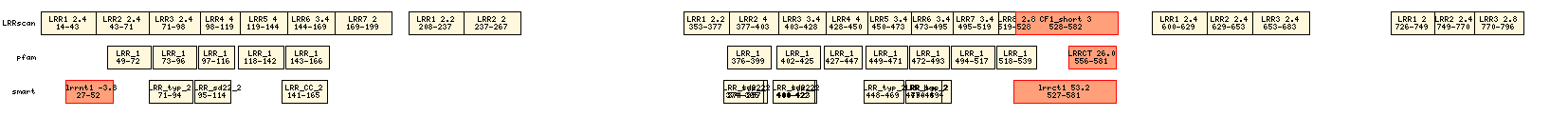

Supplement: Additional file 2 — LRRscan_out.html. Graphical comparison of HMMpfam and LRRscan results. A compressed archive (lrr_plots.tar.gz) containing 372 images in Portable Network Graphics (PNG) format, an information file (00README.txt) and two HTML-formatted pages, one with output from LRRscan (LRRscan_out.html) and one that links all the images together (00plots.html). After downloading, the archive must be to uncompressed and unpacked. Most modern operating systems (e.g. Windows XP, Mac OS X) will do this automatically when double-clicking on the file. Alternatively, you can use the free tool 'Stuffit Expander' () or your favourite unpacker. On Linux or Unix systems apply the following command: tar zxf lrr_plots.tar.gz. Please note that some browsers might uncompress the file during download without changing the file ending. If you have trouble unpacking the file try renaming it to lrr_plots.tar and double-click on it again. Unpacking the archive creates a new folder (lrr_plots) in which you can find a file called '00plots.html'. Open this file in a web-browser, either by double-clicking onto it or by using the 'File->Open File' menu (or equivalent) of your browser. This will bring up a web-page with plots of LRR motifs for 372 proteins. If you click on an image you can see the text output from LRRscan in a new window. [file 1471-2164-8-320-S2.gz › lrr_plots/ENSMUSP00000060793.png]

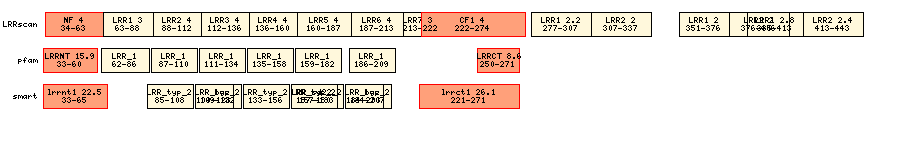

Supplement: Additional file 2 — LRRscan_out.html. Graphical comparison of HMMpfam and LRRscan results. A compressed archive (lrr_plots.tar.gz) containing 372 images in Portable Network Graphics (PNG) format, an information file (00README.txt) and two HTML-formatted pages, one with output from LRRscan (LRRscan_out.html) and one that links all the images together (00plots.html). After downloading, the archive must be to uncompressed and unpacked. Most modern operating systems (e.g. Windows XP, Mac OS X) will do this automatically when double-clicking on the file. Alternatively, you can use the free tool 'Stuffit Expander' () or your favourite unpacker. On Linux or Unix systems apply the following command: tar zxf lrr_plots.tar.gz. Please note that some browsers might uncompress the file during download without changing the file ending. If you have trouble unpacking the file try renaming it to lrr_plots.tar and double-click on it again. Unpacking the archive creates a new folder (lrr_plots) in which you can find a file called '00plots.html'. Open this file in a web-browser, either by double-clicking onto it or by using the 'File->Open File' menu (or equivalent) of your browser. This will bring up a web-page with plots of LRR motifs for 372 proteins. If you click on an image you can see the text output from LRRscan in a new window. [file 1471-2164-8-320-S2.gz › lrr_plots/ENSMUSP00000061244.png]

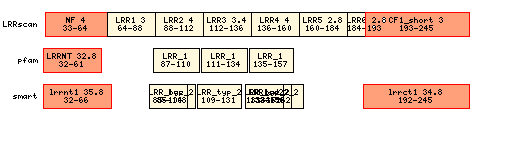

Supplement: Additional file 2 — LRRscan_out.html. Graphical comparison of HMMpfam and LRRscan results. A compressed archive (lrr_plots.tar.gz) containing 372 images in Portable Network Graphics (PNG) format, an information file (00README.txt) and two HTML-formatted pages, one with output from LRRscan (LRRscan_out.html) and one that links all the images together (00plots.html). After downloading, the archive must be to uncompressed and unpacked. Most modern operating systems (e.g. Windows XP, Mac OS X) will do this automatically when double-clicking on the file. Alternatively, you can use the free tool 'Stuffit Expander' () or your favourite unpacker. On Linux or Unix systems apply the following command: tar zxf lrr_plots.tar.gz. Please note that some browsers might uncompress the file during download without changing the file ending. If you have trouble unpacking the file try renaming it to lrr_plots.tar and double-click on it again. Unpacking the archive creates a new folder (lrr_plots) in which you can find a file called '00plots.html'. Open this file in a web-browser, either by double-clicking onto it or by using the 'File->Open File' menu (or equivalent) of your browser. This will bring up a web-page with plots of LRR motifs for 372 proteins. If you click on an image you can see the text output from LRRscan in a new window. [file 1471-2164-8-320-S2.gz › lrr_plots/ENSMUSP00000061828.png]

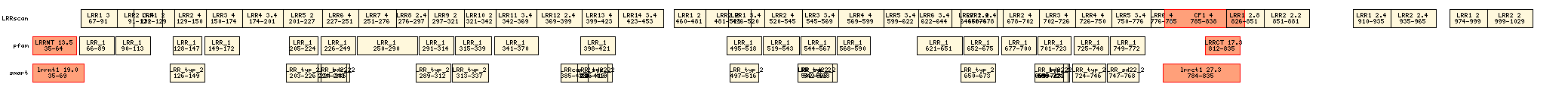

Supplement: Additional file 2 — LRRscan_out.html. Graphical comparison of HMMpfam and LRRscan results. A compressed archive (lrr_plots.tar.gz) containing 372 images in Portable Network Graphics (PNG) format, an information file (00README.txt) and two HTML-formatted pages, one with output from LRRscan (LRRscan_out.html) and one that links all the images together (00plots.html). After downloading, the archive must be to uncompressed and unpacked. Most modern operating systems (e.g. Windows XP, Mac OS X) will do this automatically when double-clicking on the file. Alternatively, you can use the free tool 'Stuffit Expander' () or your favourite unpacker. On Linux or Unix systems apply the following command: tar zxf lrr_plots.tar.gz. Please note that some browsers might uncompress the file during download without changing the file ending. If you have trouble unpacking the file try renaming it to lrr_plots.tar and double-click on it again. Unpacking the archive creates a new folder (lrr_plots) in which you can find a file called '00plots.html'. Open this file in a web-browser, either by double-clicking onto it or by using the 'File->Open File' menu (or equivalent) of your browser. This will bring up a web-page with plots of LRR motifs for 372 proteins. If you click on an image you can see the text output from LRRscan in a new window. [file 1471-2164-8-320-S2.gz › lrr_plots/ENSMUSP00000061853.png]

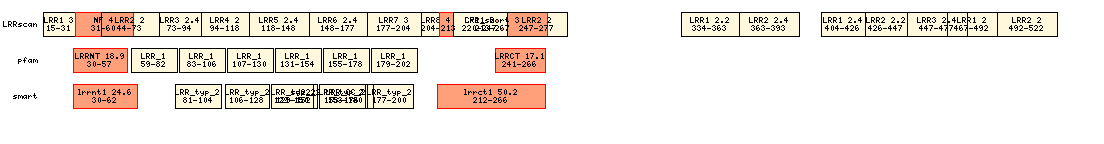

Supplement: Additional file 2 — LRRscan_out.html. Graphical comparison of HMMpfam and LRRscan results. A compressed archive (lrr_plots.tar.gz) containing 372 images in Portable Network Graphics (PNG) format, an information file (00README.txt) and two HTML-formatted pages, one with output from LRRscan (LRRscan_out.html) and one that links all the images together (00plots.html). After downloading, the archive must be to uncompressed and unpacked. Most modern operating systems (e.g. Windows XP, Mac OS X) will do this automatically when double-clicking on the file. Alternatively, you can use the free tool 'Stuffit Expander' () or your favourite unpacker. On Linux or Unix systems apply the following command: tar zxf lrr_plots.tar.gz. Please note that some browsers might uncompress the file during download without changing the file ending. If you have trouble unpacking the file try renaming it to lrr_plots.tar and double-click on it again. Unpacking the archive creates a new folder (lrr_plots) in which you can find a file called '00plots.html'. Open this file in a web-browser, either by double-clicking onto it or by using the 'File->Open File' menu (or equivalent) of your browser. This will bring up a web-page with plots of LRR motifs for 372 proteins. If you click on an image you can see the text output from LRRscan in a new window. [file 1471-2164-8-320-S2.gz › lrr_plots/ENSMUSP00000061906.png]

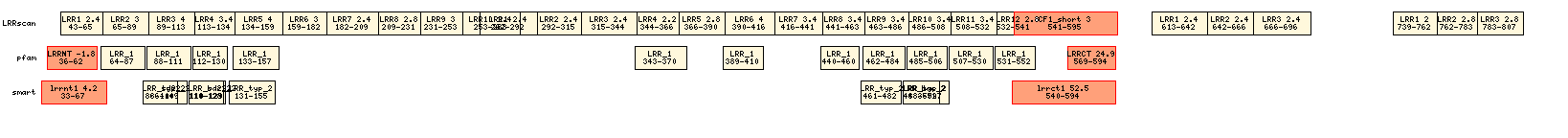

Supplement: Additional file 2 — LRRscan_out.html. Graphical comparison of HMMpfam and LRRscan results. A compressed archive (lrr_plots.tar.gz) containing 372 images in Portable Network Graphics (PNG) format, an information file (00README.txt) and two HTML-formatted pages, one with output from LRRscan (LRRscan_out.html) and one that links all the images together (00plots.html). After downloading, the archive must be to uncompressed and unpacked. Most modern operating systems (e.g. Windows XP, Mac OS X) will do this automatically when double-clicking on the file. Alternatively, you can use the free tool 'Stuffit Expander' () or your favourite unpacker. On Linux or Unix systems apply the following command: tar zxf lrr_plots.tar.gz. Please note that some browsers might uncompress the file during download without changing the file ending. If you have trouble unpacking the file try renaming it to lrr_plots.tar and double-click on it again. Unpacking the archive creates a new folder (lrr_plots) in which you can find a file called '00plots.html'. Open this file in a web-browser, either by double-clicking onto it or by using the 'File->Open File' menu (or equivalent) of your browser. This will bring up a web-page with plots of LRR motifs for 372 proteins. If you click on an image you can see the text output from LRRscan in a new window. [file 1471-2164-8-320-S2.gz › lrr_plots/ENSMUSP00000062096.png]

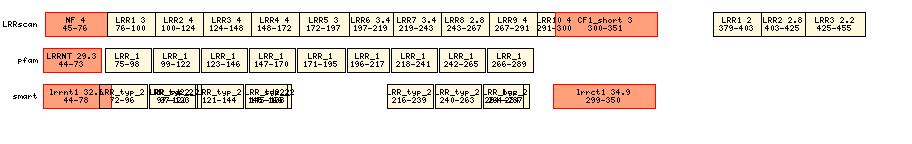

Supplement: Additional file 2 — LRRscan_out.html. Graphical comparison of HMMpfam and LRRscan results. A compressed archive (lrr_plots.tar.gz) containing 372 images in Portable Network Graphics (PNG) format, an information file (00README.txt) and two HTML-formatted pages, one with output from LRRscan (LRRscan_out.html) and one that links all the images together (00plots.html). After downloading, the archive must be to uncompressed and unpacked. Most modern operating systems (e.g. Windows XP, Mac OS X) will do this automatically when double-clicking on the file. Alternatively, you can use the free tool 'Stuffit Expander' () or your favourite unpacker. On Linux or Unix systems apply the following command: tar zxf lrr_plots.tar.gz. Please note that some browsers might uncompress the file during download without changing the file ending. If you have trouble unpacking the file try renaming it to lrr_plots.tar and double-click on it again. Unpacking the archive creates a new folder (lrr_plots) in which you can find a file called '00plots.html'. Open this file in a web-browser, either by double-clicking onto it or by using the 'File->Open File' menu (or equivalent) of your browser. This will bring up a web-page with plots of LRR motifs for 372 proteins. If you click on an image you can see the text output from LRRscan in a new window. [file 1471-2164-8-320-S2.gz › lrr_plots/ENSMUSP00000062158.png]

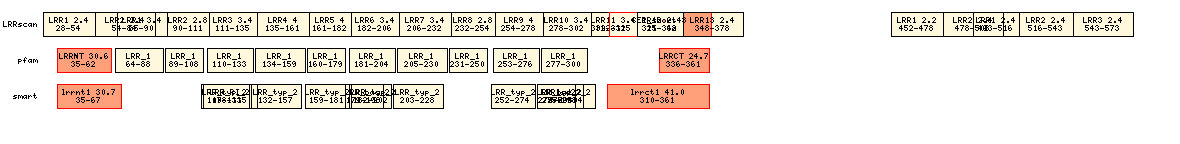

Supplement: Additional file 2 — LRRscan_out.html. Graphical comparison of HMMpfam and LRRscan results. A compressed archive (lrr_plots.tar.gz) containing 372 images in Portable Network Graphics (PNG) format, an information file (00README.txt) and two HTML-formatted pages, one with output from LRRscan (LRRscan_out.html) and one that links all the images together (00plots.html). After downloading, the archive must be to uncompressed and unpacked. Most modern operating systems (e.g. Windows XP, Mac OS X) will do this automatically when double-clicking on the file. Alternatively, you can use the free tool 'Stuffit Expander' () or your favourite unpacker. On Linux or Unix systems apply the following command: tar zxf lrr_plots.tar.gz. Please note that some browsers might uncompress the file during download without changing the file ending. If you have trouble unpacking the file try renaming it to lrr_plots.tar and double-click on it again. Unpacking the archive creates a new folder (lrr_plots) in which you can find a file called '00plots.html'. Open this file in a web-browser, either by double-clicking onto it or by using the 'File->Open File' menu (or equivalent) of your browser. This will bring up a web-page with plots of LRR motifs for 372 proteins. If you click on an image you can see the text output from LRRscan in a new window. [file 1471-2164-8-320-S2.gz › lrr_plots/ENSMUSP00000062171.png]

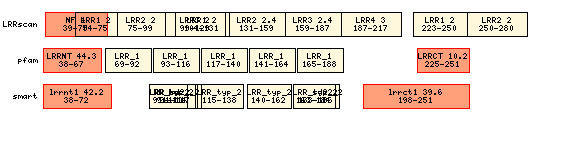

Supplement: Additional file 2 — LRRscan_out.html. Graphical comparison of HMMpfam and LRRscan results. A compressed archive (lrr_plots.tar.gz) containing 372 images in Portable Network Graphics (PNG) format, an information file (00README.txt) and two HTML-formatted pages, one with output from LRRscan (LRRscan_out.html) and one that links all the images together (00plots.html). After downloading, the archive must be to uncompressed and unpacked. Most modern operating systems (e.g. Windows XP, Mac OS X) will do this automatically when double-clicking on the file. Alternatively, you can use the free tool 'Stuffit Expander' () or your favourite unpacker. On Linux or Unix systems apply the following command: tar zxf lrr_plots.tar.gz. Please note that some browsers might uncompress the file during download without changing the file ending. If you have trouble unpacking the file try renaming it to lrr_plots.tar and double-click on it again. Unpacking the archive creates a new folder (lrr_plots) in which you can find a file called '00plots.html'. Open this file in a web-browser, either by double-clicking onto it or by using the 'File->Open File' menu (or equivalent) of your browser. This will bring up a web-page with plots of LRR motifs for 372 proteins. If you click on an image you can see the text output from LRRscan in a new window. [file 1471-2164-8-320-S2.gz › lrr_plots/ENSMUSP00000063882.png]

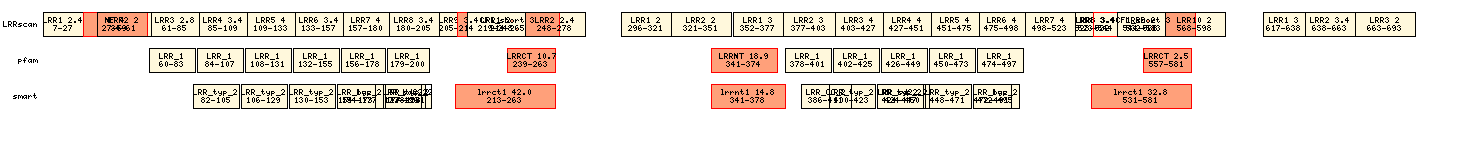

Supplement: Additional file 2 — LRRscan_out.html. Graphical comparison of HMMpfam and LRRscan results. A compressed archive (lrr_plots.tar.gz) containing 372 images in Portable Network Graphics (PNG) format, an information file (00README.txt) and two HTML-formatted pages, one with output from LRRscan (LRRscan_out.html) and one that links all the images together (00plots.html). After downloading, the archive must be to uncompressed and unpacked. Most modern operating systems (e.g. Windows XP, Mac OS X) will do this automatically when double-clicking on the file. Alternatively, you can use the free tool 'Stuffit Expander' () or your favourite unpacker. On Linux or Unix systems apply the following command: tar zxf lrr_plots.tar.gz. Please note that some browsers might uncompress the file during download without changing the file ending. If you have trouble unpacking the file try renaming it to lrr_plots.tar and double-click on it again. Unpacking the archive creates a new folder (lrr_plots) in which you can find a file called '00plots.html'. Open this file in a web-browser, either by double-clicking onto it or by using the 'File->Open File' menu (or equivalent) of your browser. This will bring up a web-page with plots of LRR motifs for 372 proteins. If you click on an image you can see the text output from LRRscan in a new window. [file 1471-2164-8-320-S2.gz › lrr_plots/ENSMUSP00000064443.png]

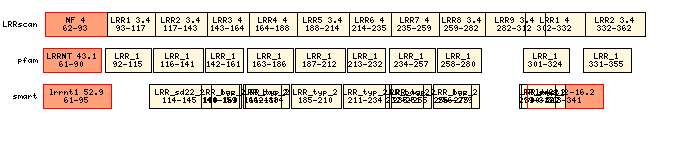

Supplement: Additional file 2 — LRRscan_out.html. Graphical comparison of HMMpfam and LRRscan results. A compressed archive (lrr_plots.tar.gz) containing 372 images in Portable Network Graphics (PNG) format, an information file (00README.txt) and two HTML-formatted pages, one with output from LRRscan (LRRscan_out.html) and one that links all the images together (00plots.html). After downloading, the archive must be to uncompressed and unpacked. Most modern operating systems (e.g. Windows XP, Mac OS X) will do this automatically when double-clicking on the file. Alternatively, you can use the free tool 'Stuffit Expander' () or your favourite unpacker. On Linux or Unix systems apply the following command: tar zxf lrr_plots.tar.gz. Please note that some browsers might uncompress the file during download without changing the file ending. If you have trouble unpacking the file try renaming it to lrr_plots.tar and double-click on it again. Unpacking the archive creates a new folder (lrr_plots) in which you can find a file called '00plots.html'. Open this file in a web-browser, either by double-clicking onto it or by using the 'File->Open File' menu (or equivalent) of your browser. This will bring up a web-page with plots of LRR motifs for 372 proteins. If you click on an image you can see the text output from LRRscan in a new window. [file 1471-2164-8-320-S2.gz › lrr_plots/ENSMUSP00000065706.png]

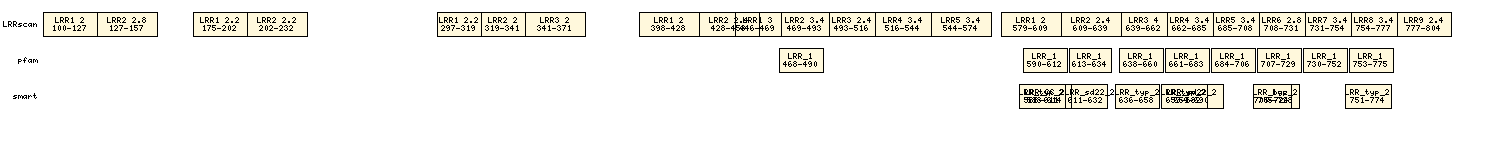

Supplement: Additional file 2 — LRRscan_out.html. Graphical comparison of HMMpfam and LRRscan results. A compressed archive (lrr_plots.tar.gz) containing 372 images in Portable Network Graphics (PNG) format, an information file (00README.txt) and two HTML-formatted pages, one with output from LRRscan (LRRscan_out.html) and one that links all the images together (00plots.html). After downloading, the archive must be to uncompressed and unpacked. Most modern operating systems (e.g. Windows XP, Mac OS X) will do this automatically when double-clicking on the file. Alternatively, you can use the free tool 'Stuffit Expander' () or your favourite unpacker. On Linux or Unix systems apply the following command: tar zxf lrr_plots.tar.gz. Please note that some browsers might uncompress the file during download without changing the file ending. If you have trouble unpacking the file try renaming it to lrr_plots.tar and double-click on it again. Unpacking the archive creates a new folder (lrr_plots) in which you can find a file called '00plots.html'. Open this file in a web-browser, either by double-clicking onto it or by using the 'File->Open File' menu (or equivalent) of your browser. This will bring up a web-page with plots of LRR motifs for 372 proteins. If you click on an image you can see the text output from LRRscan in a new window. [file 1471-2164-8-320-S2.gz › lrr_plots/ENSMUSP00000066015.png]

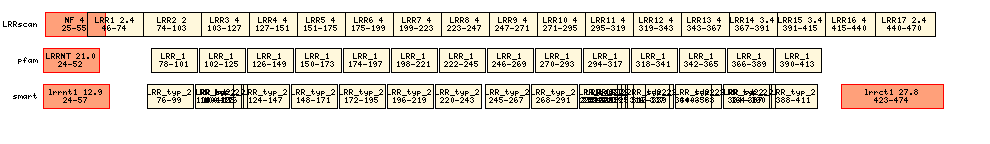

Supplement: Additional file 2 — LRRscan_out.html. Graphical comparison of HMMpfam and LRRscan results. A compressed archive (lrr_plots.tar.gz) containing 372 images in Portable Network Graphics (PNG) format, an information file (00README.txt) and two HTML-formatted pages, one with output from LRRscan (LRRscan_out.html) and one that links all the images together (00plots.html). After downloading, the archive must be to uncompressed and unpacked. Most modern operating systems (e.g. Windows XP, Mac OS X) will do this automatically when double-clicking on the file. Alternatively, you can use the free tool 'Stuffit Expander' () or your favourite unpacker. On Linux or Unix systems apply the following command: tar zxf lrr_plots.tar.gz. Please note that some browsers might uncompress the file during download without changing the file ending. If you have trouble unpacking the file try renaming it to lrr_plots.tar and double-click on it again. Unpacking the archive creates a new folder (lrr_plots) in which you can find a file called '00plots.html'. Open this file in a web-browser, either by double-clicking onto it or by using the 'File->Open File' menu (or equivalent) of your browser. This will bring up a web-page with plots of LRR motifs for 372 proteins. If you click on an image you can see the text output from LRRscan in a new window. [file 1471-2164-8-320-S2.gz › lrr_plots/ENSMUSP00000066777.png]

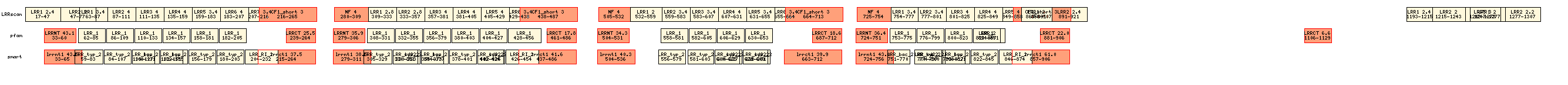

Supplement: Additional file 2 — LRRscan_out.html. Graphical comparison of HMMpfam and LRRscan results. A compressed archive (lrr_plots.tar.gz) containing 372 images in Portable Network Graphics (PNG) format, an information file (00README.txt) and two HTML-formatted pages, one with output from LRRscan (LRRscan_out.html) and one that links all the images together (00plots.html). After downloading, the archive must be to uncompressed and unpacked. Most modern operating systems (e.g. Windows XP, Mac OS X) will do this automatically when double-clicking on the file. Alternatively, you can use the free tool 'Stuffit Expander' () or your favourite unpacker. On Linux or Unix systems apply the following command: tar zxf lrr_plots.tar.gz. Please note that some browsers might uncompress the file during download without changing the file ending. If you have trouble unpacking the file try renaming it to lrr_plots.tar and double-click on it again. Unpacking the archive creates a new folder (lrr_plots) in which you can find a file called '00plots.html'. Open this file in a web-browser, either by double-clicking onto it or by using the 'File->Open File' menu (or equivalent) of your browser. This will bring up a web-page with plots of LRR motifs for 372 proteins. If you click on an image you can see the text output from LRRscan in a new window. [file 1471-2164-8-320-S2.gz › lrr_plots/ENSMUSP00000066857.png]

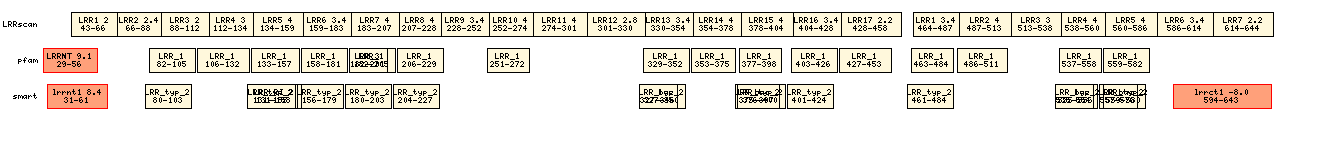

Supplement: Additional file 2 — LRRscan_out.html. Graphical comparison of HMMpfam and LRRscan results. A compressed archive (lrr_plots.tar.gz) containing 372 images in Portable Network Graphics (PNG) format, an information file (00README.txt) and two HTML-formatted pages, one with output from LRRscan (LRRscan_out.html) and one that links all the images together (00plots.html). After downloading, the archive must be to uncompressed and unpacked. Most modern operating systems (e.g. Windows XP, Mac OS X) will do this automatically when double-clicking on the file. Alternatively, you can use the free tool 'Stuffit Expander' () or your favourite unpacker. On Linux or Unix systems apply the following command: tar zxf lrr_plots.tar.gz. Please note that some browsers might uncompress the file during download without changing the file ending. If you have trouble unpacking the file try renaming it to lrr_plots.tar and double-click on it again. Unpacking the archive creates a new folder (lrr_plots) in which you can find a file called '00plots.html'. Open this file in a web-browser, either by double-clicking onto it or by using the 'File->Open File' menu (or equivalent) of your browser. This will bring up a web-page with plots of LRR motifs for 372 proteins. If you click on an image you can see the text output from LRRscan in a new window. [file 1471-2164-8-320-S2.gz › lrr_plots/ENSMUSP00000067130.png]

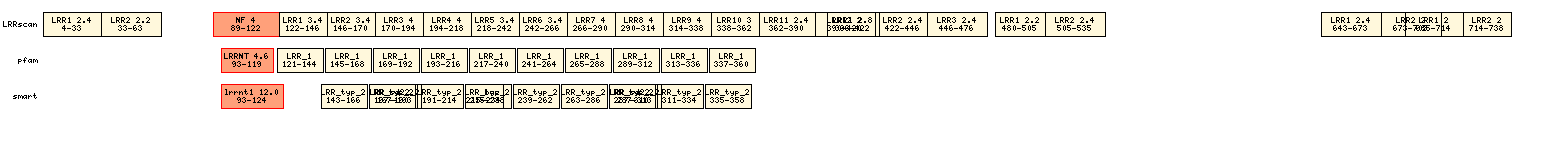

Supplement: Additional file 2 — LRRscan_out.html. Graphical comparison of HMMpfam and LRRscan results. A compressed archive (lrr_plots.tar.gz) containing 372 images in Portable Network Graphics (PNG) format, an information file (00README.txt) and two HTML-formatted pages, one with output from LRRscan (LRRscan_out.html) and one that links all the images together (00plots.html). After downloading, the archive must be to uncompressed and unpacked. Most modern operating systems (e.g. Windows XP, Mac OS X) will do this automatically when double-clicking on the file. Alternatively, you can use the free tool 'Stuffit Expander' () or your favourite unpacker. On Linux or Unix systems apply the following command: tar zxf lrr_plots.tar.gz. Please note that some browsers might uncompress the file during download without changing the file ending. If you have trouble unpacking the file try renaming it to lrr_plots.tar and double-click on it again. Unpacking the archive creates a new folder (lrr_plots) in which you can find a file called '00plots.html'. Open this file in a web-browser, either by double-clicking onto it or by using the 'File->Open File' menu (or equivalent) of your browser. This will bring up a web-page with plots of LRR motifs for 372 proteins. If you click on an image you can see the text output from LRRscan in a new window. [file 1471-2164-8-320-S2.gz › lrr_plots/ENSMUSP00000067897.png]

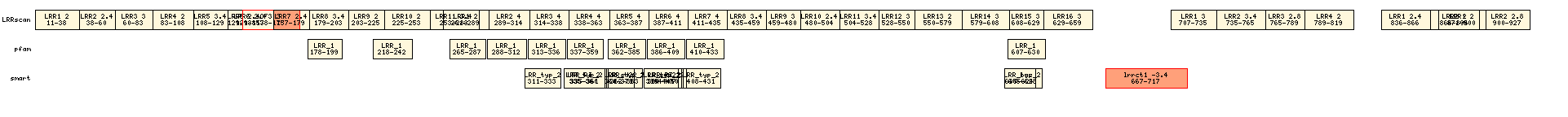

Supplement: Additional file 2 — LRRscan_out.html. Graphical comparison of HMMpfam and LRRscan results. A compressed archive (lrr_plots.tar.gz) containing 372 images in Portable Network Graphics (PNG) format, an information file (00README.txt) and two HTML-formatted pages, one with output from LRRscan (LRRscan_out.html) and one that links all the images together (00plots.html). After downloading, the archive must be to uncompressed and unpacked. Most modern operating systems (e.g. Windows XP, Mac OS X) will do this automatically when double-clicking on the file. Alternatively, you can use the free tool 'Stuffit Expander' () or your favourite unpacker. On Linux or Unix systems apply the following command: tar zxf lrr_plots.tar.gz. Please note that some browsers might uncompress the file during download without changing the file ending. If you have trouble unpacking the file try renaming it to lrr_plots.tar and double-click on it again. Unpacking the archive creates a new folder (lrr_plots) in which you can find a file called '00plots.html'. Open this file in a web-browser, either by double-clicking onto it or by using the 'File->Open File' menu (or equivalent) of your browser. This will bring up a web-page with plots of LRR motifs for 372 proteins. If you click on an image you can see the text output from LRRscan in a new window. [file 1471-2164-8-320-S2.gz › lrr_plots/ENSMUSP00000068906.png]

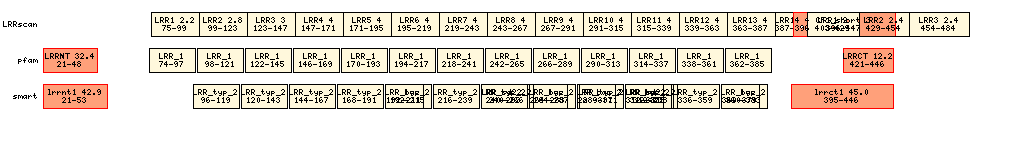

Supplement: Additional file 2 — LRRscan_out.html. Graphical comparison of HMMpfam and LRRscan results. A compressed archive (lrr_plots.tar.gz) containing 372 images in Portable Network Graphics (PNG) format, an information file (00README.txt) and two HTML-formatted pages, one with output from LRRscan (LRRscan_out.html) and one that links all the images together (00plots.html). After downloading, the archive must be to uncompressed and unpacked. Most modern operating systems (e.g. Windows XP, Mac OS X) will do this automatically when double-clicking on the file. Alternatively, you can use the free tool 'Stuffit Expander' () or your favourite unpacker. On Linux or Unix systems apply the following command: tar zxf lrr_plots.tar.gz. Please note that some browsers might uncompress the file during download without changing the file ending. If you have trouble unpacking the file try renaming it to lrr_plots.tar and double-click on it again. Unpacking the archive creates a new folder (lrr_plots) in which you can find a file called '00plots.html'. Open this file in a web-browser, either by double-clicking onto it or by using the 'File->Open File' menu (or equivalent) of your browser. This will bring up a web-page with plots of LRR motifs for 372 proteins. If you click on an image you can see the text output from LRRscan in a new window. [file 1471-2164-8-320-S2.gz › lrr_plots/ENSMUSP00000069318.png]

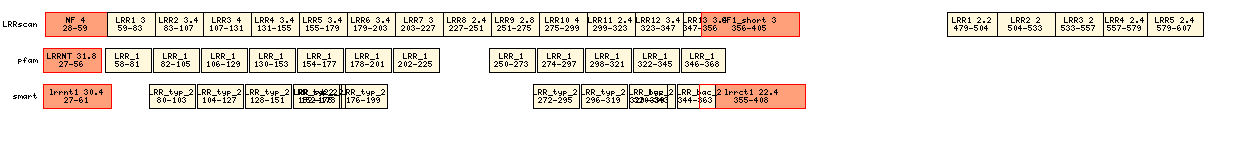

Supplement: Additional file 2 — LRRscan_out.html. Graphical comparison of HMMpfam and LRRscan results. A compressed archive (lrr_plots.tar.gz) containing 372 images in Portable Network Graphics (PNG) format, an information file (00README.txt) and two HTML-formatted pages, one with output from LRRscan (LRRscan_out.html) and one that links all the images together (00plots.html). After downloading, the archive must be to uncompressed and unpacked. Most modern operating systems (e.g. Windows XP, Mac OS X) will do this automatically when double-clicking on the file. Alternatively, you can use the free tool 'Stuffit Expander' () or your favourite unpacker. On Linux or Unix systems apply the following command: tar zxf lrr_plots.tar.gz. Please note that some browsers might uncompress the file during download without changing the file ending. If you have trouble unpacking the file try renaming it to lrr_plots.tar and double-click on it again. Unpacking the archive creates a new folder (lrr_plots) in which you can find a file called '00plots.html'. Open this file in a web-browser, either by double-clicking onto it or by using the 'File->Open File' menu (or equivalent) of your browser. This will bring up a web-page with plots of LRR motifs for 372 proteins. If you click on an image you can see the text output from LRRscan in a new window. [file 1471-2164-8-320-S2.gz › lrr_plots/ENSMUSP00000069772.png]

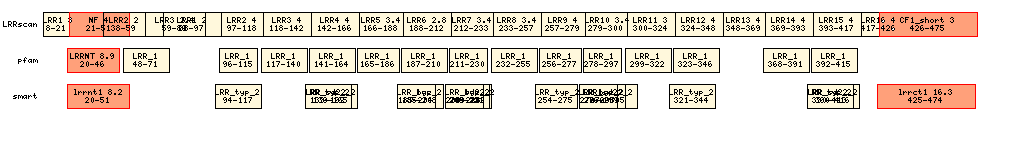

Supplement: Additional file 2 — LRRscan_out.html. Graphical comparison of HMMpfam and LRRscan results. A compressed archive (lrr_plots.tar.gz) containing 372 images in Portable Network Graphics (PNG) format, an information file (00README.txt) and two HTML-formatted pages, one with output from LRRscan (LRRscan_out.html) and one that links all the images together (00plots.html). After downloading, the archive must be to uncompressed and unpacked. Most modern operating systems (e.g. Windows XP, Mac OS X) will do this automatically when double-clicking on the file. Alternatively, you can use the free tool 'Stuffit Expander' () or your favourite unpacker. On Linux or Unix systems apply the following command: tar zxf lrr_plots.tar.gz. Please note that some browsers might uncompress the file during download without changing the file ending. If you have trouble unpacking the file try renaming it to lrr_plots.tar and double-click on it again. Unpacking the archive creates a new folder (lrr_plots) in which you can find a file called '00plots.html'. Open this file in a web-browser, either by double-clicking onto it or by using the 'File->Open File' menu (or equivalent) of your browser. This will bring up a web-page with plots of LRR motifs for 372 proteins. If you click on an image you can see the text output from LRRscan in a new window. [file 1471-2164-8-320-S2.gz › lrr_plots/ENSMUSP00000070130.png]

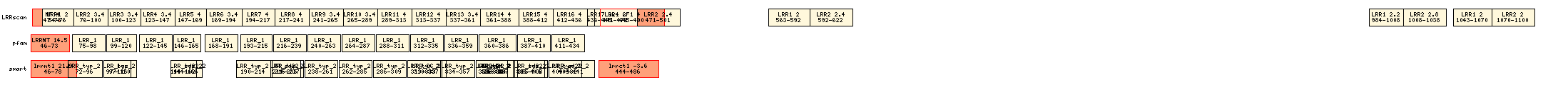

Supplement: Additional file 2 — LRRscan_out.html. Graphical comparison of HMMpfam and LRRscan results. A compressed archive (lrr_plots.tar.gz) containing 372 images in Portable Network Graphics (PNG) format, an information file (00README.txt) and two HTML-formatted pages, one with output from LRRscan (LRRscan_out.html) and one that links all the images together (00plots.html). After downloading, the archive must be to uncompressed and unpacked. Most modern operating systems (e.g. Windows XP, Mac OS X) will do this automatically when double-clicking on the file. Alternatively, you can use the free tool 'Stuffit Expander' () or your favourite unpacker. On Linux or Unix systems apply the following command: tar zxf lrr_plots.tar.gz. Please note that some browsers might uncompress the file during download without changing the file ending. If you have trouble unpacking the file try renaming it to lrr_plots.tar and double-click on it again. Unpacking the archive creates a new folder (lrr_plots) in which you can find a file called '00plots.html'. Open this file in a web-browser, either by double-clicking onto it or by using the 'File->Open File' menu (or equivalent) of your browser. This will bring up a web-page with plots of LRR motifs for 372 proteins. If you click on an image you can see the text output from LRRscan in a new window. [file 1471-2164-8-320-S2.gz › lrr_plots/ENSMUSP00000074360.png]

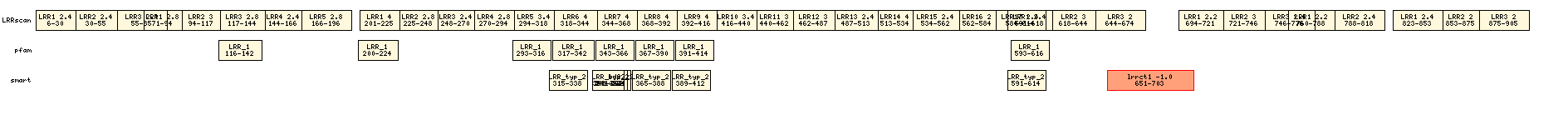

Supplement: Additional file 2 — LRRscan_out.html. Graphical comparison of HMMpfam and LRRscan results. A compressed archive (lrr_plots.tar.gz) containing 372 images in Portable Network Graphics (PNG) format, an information file (00README.txt) and two HTML-formatted pages, one with output from LRRscan (LRRscan_out.html) and one that links all the images together (00plots.html). After downloading, the archive must be to uncompressed and unpacked. Most modern operating systems (e.g. Windows XP, Mac OS X) will do this automatically when double-clicking on the file. Alternatively, you can use the free tool 'Stuffit Expander' () or your favourite unpacker. On Linux or Unix systems apply the following command: tar zxf lrr_plots.tar.gz. Please note that some browsers might uncompress the file during download without changing the file ending. If you have trouble unpacking the file try renaming it to lrr_plots.tar and double-click on it again. Unpacking the archive creates a new folder (lrr_plots) in which you can find a file called '00plots.html'. Open this file in a web-browser, either by double-clicking onto it or by using the 'File->Open File' menu (or equivalent) of your browser. This will bring up a web-page with plots of LRR motifs for 372 proteins. If you click on an image you can see the text output from LRRscan in a new window. [file 1471-2164-8-320-S2.gz › lrr_plots/ENSMUSP00000074381.png]

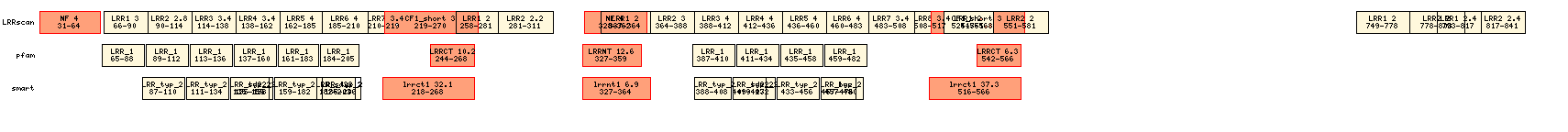

Supplement: Additional file 2 — LRRscan_out.html. Graphical comparison of HMMpfam and LRRscan results. A compressed archive (lrr_plots.tar.gz) containing 372 images in Portable Network Graphics (PNG) format, an information file (00README.txt) and two HTML-formatted pages, one with output from LRRscan (LRRscan_out.html) and one that links all the images together (00plots.html). After downloading, the archive must be to uncompressed and unpacked. Most modern operating systems (e.g. Windows XP, Mac OS X) will do this automatically when double-clicking on the file. Alternatively, you can use the free tool 'Stuffit Expander' () or your favourite unpacker. On Linux or Unix systems apply the following command: tar zxf lrr_plots.tar.gz. Please note that some browsers might uncompress the file during download without changing the file ending. If you have trouble unpacking the file try renaming it to lrr_plots.tar and double-click on it again. Unpacking the archive creates a new folder (lrr_plots) in which you can find a file called '00plots.html'. Open this file in a web-browser, either by double-clicking onto it or by using the 'File->Open File' menu (or equivalent) of your browser. This will bring up a web-page with plots of LRR motifs for 372 proteins. If you click on an image you can see the text output from LRRscan in a new window. [file 1471-2164-8-320-S2.gz › lrr_plots/ENSMUSP00000077492.png]
