# Supplementary material for: The extracellular Leucine-Rich Repeat superfamily; a comparative survey and analysis of evolutionary relationships and expression patterns
Source: BMC Genomics. 2007 Sep 14;8:320. doi: 10.1186/1471-2164-8-320 (PMC2235866; doi:10.1186/1471-2164-8-320)
Supplement: Additional file 8 — Additional methods. In situ hybridisation protocols [file 1471-2164-8-320-S8.doc]

**Additional File 9**

**Supplemental methods**

**RNA in situ hybridization of *Drosophila* embryos**

Wildtype *Drosophila* embryos were collected for 12 hours at room temperature and then aged for an additional 3 hours to attain a range of stages from stage 5 to stage 17, all staging according to Campos-Ortega and Hartenstein (1985). Digoxigenin labelled RNA probes were prepared according to the manufacturers instructions (Roche diagnostics). PCR products were amplified from a cDNA library created by M. Georgiou, a T7 RNA polymerase consensus site was added to the reverse primer. In situ hybridizations were performed according to the Berkley Drosophila Genome Project (BDGP) 96-well plate protocol (http://www.fruitfly.org/about/methods/RNAinsitu.html) (adapted from Tautz and Pfeifle 1989) with a few minor modifications. The embryos were post fixed in 4% paraformaldehyde, pre-hybridization was carried out at 54ºC and the wash buffers contained 0.2% Tween 20. The embryos were cleared in 70% glycerol, mounted on slides and photographed at x200 magnification using a Zeiss Axioscope microscope, Axiocam camera and the accompanying Axiovision software.

# Mouse in situ hybridization

Digoxigenin-labelled antisense cRNA probes for in situ hybridization were designed to encompass a section of coding sequence and 3’UTR >500bp in length. Briefly this involved TA cloning of PCR products into the TOPO vector (Invitrogen) and simultaneous synthesis and Dig-labelling of RNA transcripts from linearised vector using T7- or Sp6- RNA polymerase. Detailed information on probes is available upon request.

In situ hybridization was carried out on vibratome-sectioned C57Bl6 mouse brains (Jackson Laboratories) as described (Riddle et al 1993) with modifications. To obtain brains at E15, timed pregnancy mice were killed by cervical dislocation and embryos were fixed and stored in 4% paraformaldehyde-PBS at 4°C until use. For P0, brains were dissected out prior to immersion in 4% paraformaldehyde-PBS (PFA) at 4°C. P9 brains were fixed by perfusion followed by immersion in 4% paraformaldehyde-PBS at 4°C. Fixed brains were embedded in 3% agarose and 70μm sections were obtained on a vibratome (VT1000S Leica). Sections were washed twice in PBST (PBS containing 0.1% Tween-20), permeabilised in RIPA buffer (150mM NaCl, 50mM Tris-HCl pH 8.0, 1mM EDTA, 1% Nonidet-P40, 0.5% sodium deoxycholate, 0.1% SDS) and postfixed in 4% PFA and 0.2% gluteraldehyde. Hybridisation was performed in a humidified environment overnight at 65°C with 1μg/ml labeled probe in hybridisation buffer (50% formamide, 5X SSC pH4.5, 1% SDS, 50μg/ml yeast tRNA, 50μg/ml heparin). Posthybridisation washes were completed at 65°C using solution I (50% formamide, 5X SSC pH4.5, 1% SDS) and solution III (50% formamide, 2X SSC pH4.5, 0.1% Tween-20) and at room temperature using TBST (TBS containing 1% Tween-20). Brain sections were incubated for >1hr in blocking buffer (TBST, 10% heat-inactivated sheep serum). Immunodetection was carried out in blocking buffer at 4°C overnight using a phosphatase conjugated anti-digoxigenin antibody (Roche) at a 1:2000 dilution. Following antibody incubation extensive TBST washes were performed. Sections were equilibrated in NTMT (100mM Tris-HCl pH 9.5, 100mM NaCl, 50mM MgCl_2_, 1% Tween-20) prior to colourimetric detection using 2μl/ml NBT/BCIP (Roche) in NTMT. Sections were mounted on Superfrost glass slides (VWR international) and analysed with an Olympus IX51 microscope.
