# Supplementary material for: The extracellular Leucine-Rich Repeat superfamily; a comparative survey and analysis of evolutionary relationships and expression patterns
Source: BMC Genomics. 2007 Sep 14;8:320. doi: 10.1186/1471-2164-8-320 (PMC2235866; doi:10.1186/1471-2164-8-320)
Supplement: Additional file 2 — LRRscan_out.html. Graphical comparison of HMMpfam and LRRscan results. A compressed archive (lrr_plots.tar.gz) containing 372 images in Portable Network Graphics (PNG) format, an information file (00README.txt) and two HTML-formatted pages, one with output from LRRscan (LRRscan_out.html) and one that links all the images together (00plots.html). After downloading, the archive must be to uncompressed and unpacked. Most modern operating systems (e.g. Windows XP, Mac OS X) will do this automatically when double-clicking on the file. Alternatively, you can use the free tool 'Stuffit Expander' () or your favourite unpacker. On Linux or Unix systems apply the following command: tar zxf lrr_plots.tar.gz. Please note that some browsers might uncompress the file during download without changing the file ending. If you have trouble unpacking the file try renaming it to lrr_plots.tar and double-click on it again. Unpacking the archive creates a new folder (lrr_plots) in which you can find a file called '00plots.html'. Open this file in a web-browser, either by double-clicking onto it or by using the 'File->Open File' menu (or equivalent) of your browser. This will bring up a web-page with plots of LRR motifs for 372 proteins. If you click on an image you can see the text output from LRRscan in a new window. [file 1471-2164-8-320-S2.gz › lrr_plots/LRRscan_out.html]

## Output produced by LRRscan

Shown are the protein IDs in blue followed by the complete sequence.   
Where LRR/NT/CF motifs were found these are listed together with their starting position, name, and score.  
All cysteines are coloured in red to facilitate identification of additional motifs.

The program was run with the following command line options options:

```
-pattern 'LxxLxLxxN'
-alternative '0=A=0.4|0=I=0.4|0=V=0.4|0=F=0.4|0=G=0.4|0=M=0.4|0=W=0.4|3=A=0.4|3=I=0.4|3=V=0.4|3=F=0.4|3=G=0.4|3=M=0.4|3=W=0.4|5=A=0.4|5=I=0.4|5=V=0.4|5=F=0.4|5=G=0.4|5=M=0.4|5=W=0.4|8=C=0.4|8=S=0.4|8=T=0.4'
-lower 21
-upper 30
-score 1
-min_score 2.4
-add_score
```

<back to main page>

```
>CG32055-PA

MQALQVLALLLLSGVFGKDLQECEDLGNGSYLCREIESFEQLNRYVGKNWKSVKVVNEHT
GIERAEDGE

70	LPGLSTLLQ LDLSESGGVTLGEKGLQDFKA		LRR1	2
100	LQKLNLTHA QLDELKSEQFPNPSE		LRR2	3
124	MINFDVSYN DILAITTKLMSGFGN		LRR3	2.2
148	LVYANFSEN LIAVIEPNAFRHMKN		LRR4	2.8
172	LRFLDLTTN YQENITLGENAN			LRR5	4
193	LRFLSISNN NLRDFQWCHLRVLPK		LRR6	3.4
217	LEELHLHSN WLEHLDMGIFYALPN		LRR7	4
241	LRVLNVSNN NLFEIKRTLFMAPGEIAP		LRR8	3.4
268	LELLDYSSN IVKVLDDSVFCRLKK		LRR9	3
292	LRTLNLWLN QINRIHPRAFLGLSS		LRR10	4
316	LQTLHLQGN KISILPDDVFANLTA		LRR11	4
340	LEKLDLSKN NIQKLGLRVFGERILRK		LRR12	4
366	LIYLDLSNN YIADLHPLALSSMPF		LRR13	4
390	IKELRLRRN RLVSLDLRMFAPLRQ		LRR14	3.4
414	LQLLTINEN RLEEIDGEILDTLDR		LRR15	3.4
438	LNHLELNNN RLTFLPDLKSSQNLLQ		LRR16	4
463	LRNITLEGN				LRR17	3.4
472	PWQCLCLDEITSWLNGRHVSYARPSSAYFSGRKPLCVVTPMDKCLRDLQETKAQGIVE		CF1_short	3

SYEKI

>CG1149-PA

MKLLLTIPDDYCETYCGGICNDTVASKTCDREAYDYIATNEYHLI

46	MRDGHLEVN WKIPDPNIFIISPISKENRLK		LRR1	2.8
76	LNELIVSDT SYPIRAVDYLRQLGVETVTRF		LRR2	2.8

ENQINSFKVIERDVHYINGPKSLKIIIQSNLY

138	LNEIIEYIN KTTDNVNEIIINAYKTVENQQ		LRR1	2.4
168	IALDNLIFN GKSHLRSLTFIGFQIENLSTK		LRR2	2.4

SHIFT -17!

181	LRSLTFIGF QIENLSTKPFAQFIN		LRR1	2.4
205	LKRMVLTNC TVRNLTFLRTLQKS		LRR2	2.8
228	LEHLELDID NEVDLKYFTNFSSLKFMKVRN		LRR3	3

SHIFT -20!

238	EVDLKYFTN FSSLKFMKVRNYIPNKNFTA		LRR1	2
267	LICTHKNCN FIRGINGLE		LRR2	2
285	CPKLCQCLYIIDDLELNIDCSNLGLLQIPPLPIPS		NF	4

SHIFT -25!

295	IDDLELNID CSNLGLLQIPPLPIPSYG		LRR3	2.4
322	GVKLNFSNN SLSQLPTMTLPGYKL		LRR4	2.8
346	VKRLDVSRN RLTNLSINHLPAK			LRR5	2.8
368	LDYLDVSFN EIINMGNDVIKYLRTVPIFKQ		LRR6	3.4

TGNQWTIHCDDKPLLNF

415	FRHLKLIIR MKSAEMKPMFLHSLTE		LRR1	2.4
440	LPKGFLKFL GKHFIWLGVRKQEYYLINEEQ		LRR2	2.4

SHIFT -25!

445	LKFLGKHFI WLGVRKQEYYLINEEQL		LRR1	2
471	LQSMHRKLN NLNTIMSIYKYMEW		LRR2	2.4
494	LHRKLIFVN REYDLFYIRQMAAPCPHKCEC		LRR3	2.4

CYS

527	RDSLILKID CRNKFVYNFPDIVAR		LRR1	2
551	NSRLMUKQN MSSPMELHLSKNNISNITIAM		LRR2	2

SHIFT -18!

563	PMELHLSKN NISNITIAMLPKE			LRR1	3
585	LRFLDLRFN NLVTLDDKVLSYLKKN		LRR2	4
610	SIKTKLSGN				LRR3	2
619	PWNCDCKSRSVLSILRDHEPLEYDVTLKRCNISPTDCPDVCVCCLDNLTWPSFIVDCRGE		CF1	4

SHIFT -16!

663	LDNLTWPSF IVDCRGEGLLQMPSLSSR		LRR1	2.4
690	VTYVDLRNN NLTALSQKNRSSIENR		LRR2	2.8
715	SLKLHLLDN				LRR3	3
724	PWSCSCNDIEKINFMKSVSSSIVDFTEIKCSNGEKLVSINQHIVCPSD		CF1	4

LFYYL

777	ALAISLVAT IIALNFLIWFRQPVLVWFYEH		LRR1	2.2
807	GVCLSLSAK RELDKDKRFDAFLAFTHKDEA		LRR2	2.4

LLEEFVDRLERGRPRFQLCFYLRDWLAGESIPDCIGQSIKDSRRIIVLMTENFMNSTWGR
LEFRLALHATSRDRCKRLIVVLYPNVKNFDSLDSELRTYMAFNTYLERSHPNFWNKLIYS
MPLLPSYVDUD

>CG7665-PA

MEKHPSLSQRMGTTYRPRKGLKCLSFEF

29	QCRLLLHHL LLTSLSGRHFVYATS		LRR1	2
53	AVGGALSAN NCHDIHHGFDVYPN		LRR2	2.8
76	LTAVSLAQS TDTPLTATMPRSAWKCCCWNA		LRR3	2.8

SNQAEEVECRCEGDGLNRVPQTLT

130	LPIQRLTIA SAGLPRLRHTGLKVYGST		LRR1	2
157	LLDVAFTDC LQLELIQDGAFAN			LRR2	2.2
179	LTLLRTIYI TNAPKLTFLSKDVFLGISDTV		LRR3	2

DIIRIINSGLTRVPD

224	LGHLPPHNI LQMIDLDNNQITRIDSKSIK		LRR1	2
253	VKTAQLILT NNEISYVDDSAFFGSK		LRR2	2.2
278	IAKLSLKEN KKLQMMHPNAFDGIID		LRR3	3.4
303	ITELDLSST SLVGLPSAGLQN			LRR4	2.8
324	IEALYIQNT HTLKTIPSIYNFRN		LRR5	2.2
347	LQRAYLTHS				LRR6	2.8
356	FHCCAFQFPSRHDPQRHAQRMLEIEKWRKQCKSDSGT		CF3	3

RKERSTLDNPFNMPEDFGSFGGTDDSATDITPITFASFDYMADDTMNKGTFHEKIILNPG
DDSSAELCGNFTFRKPNIECYPMPNDLNPCEDVMGYQWLRISVWIVVALAVVGN

507	VAVLTVILS IRPESTPVPRFLMCH		LRR1	2.2
531	LAFADLCLG LYLLLVACIDAHSMGEYFNFA		LRR2	2.4

YDWQYGLGCKVAGFLTVFASH

582	LSVFTLTVI TIERWLAITQAMYLNHRIK		LRR1	2.4
610	LRPAALIML GGWIYSMLMSSLPLFGISNYS		LRR2	2.4

SHIFT -13!

627	MSSLPLFGI SNYSSTSICLPMENRDVYDTI		LRR1	2.4
657	YLIAILGSN GVAFSIIAVCYA			LRR2	2.4
678	QIYLSLGRE TRQAHQNSPGELSVAK		LRR3	2
703	KMALLVFTN FACWSPIAFFGLTA		LRR4	2.4
726	LAGYPLINV TKSKILLVFFYPLNSCADPYL		LRR5	2

SHIFT -16!

740	LLVFFYPLN SCADPYLYAILTSQYRQD		LRR1	2.4
767	LFTLLSKLG LCQQSALKYKDSLSGQATTRF		LRR2	2

TIHGSIQRHSSLTCKMQTVMGAETQKMLKNSEDYV

>CG18249-PA

MHFYKYILLGICGLTVSDALA

22	VYDASGRFN LSASCPDSFCSLSDRPVAYSA		LRR1	2.2
52	TPALQLREL HLRNCSRQSITWLVLQLTPG		LRR2	2
81	LRTLVIRNC ATYHISKESLRPVEN		LRR3	2.8
105	LTSLQMQGT SLGVLRDQIFNAVPR		LRR4	2.8
129	LEILQLSQN FIHTVHVAAFQGLSK		LRR5	4
153	LRLLGLQGN AIAEILSSTLDPLME		LRR6	4
177	LVHLDLSRN ELTTLPQNIFAKNKK		LRR7	4
201	LQTLLLNGN PLRILMPDVLGS			LRR8	4
222	LPNLRLLDL GHAAELEVMTLNLTN		LRR9	3
246	VQNLVLEGS SLSSLVINGGFIK			LRR10	2.8
268	LQAGNNELN HLQVGNKSSVIEMD		LRR11	2.4
291	LHGNLLNGN DTAALLRGMWNLQRLDLSKNR		LRR12	3

SHIFT -19!

302	AALLRGMWN LQRLDLSKNRIEALPQH		LRR1	2.8
328	GSGLDASGT QELLILPSLKFLN			LRR2	2.2
350	LANNQLVRL PPESPILSSRLSYLDL		LRR3	2
375	SHNLMLTLD VAILRSLSVLKG			LRR4	2
396	LYVEGNRLN TINYQKLHEEHPD			LRR5	2
418	LSELGLHDN PWSSGLYRKMFLYFTDRGVHL		LRR6	4

QARPQNRVLNKSSRVDIDWPPNEGQAEAQKMDPPGVTGIHPYWTLRDILAFVTLL

503	VVMIILLMN LYHILEEEGCLRRFRHW		LRR1	2.8
529	RRSRTLGQN TSTTRNSGRRLDEQDSEV		LRR2	2


>CG10824-PA

MYVRNCLPFLCSYLLISLAATATTDEDADADVDSGSSRIVLVSSLEGHCQTEGKVTSCRG
FEFAGEDEKATFDLPTEVRIAEDGTTYEVGDEEHSRTLIFENCTFTNFPLRLFYT

116	LEVSELDMR GCGIRFIYWENFSIGADK		LRR1	2
143	LVILLLSDN HIEVLPTKTFRGAGN		LRR2	4
167	LEFIFLNRN KLGKLQAGAFDNLLK		LRR3	3.4
191	LQYLDLTEN RLEALAADVFAGLKS		LRR4	4
215	LRHVGLAGN QLTTIESDLFAHNPD		LRR5	3.4
239	LLSVAMQNN RLREVGEYAFRSRGRHHQ		LRR6	2.8
266	MQYVDLSNN PELVVLLLNINATNLTARNCS		LRR7	2.8
296	LDRVNLYGS VTNVDLSDNRVRELYFPASEA		LRR8	2.8
326	LEHLVLRNN SLVQLASLSRVPR			LRR9	4
348	LRHLDVADN PNLGQLPDGWRTPH		LRR10	3.4
371	LEMLVLRNT GQMELPLEALQGMQN		LRR11	3.4
395	LQKLDISGN NLTEIDPSAFPTLTQ		LRR12	3.4
419	LTHFYIHGN NWNCFSLRNIMDVLIRANGIA		LRR13	2.8

YTVDNYDPDFPGEYFHGIACMYRLPEKEGVDSSSSSEISASVESSPITSSSDPSEVDKLR
DELKAVVQHFDSKFDLIFSKLAQLNEQIQAFEVLNKTVWSQVTLSV

>CG15658-PA

MELHWQLLTFIVC

14	LQLLHSAGF IIQSEVRKCTYGHIDKLLRIR		LRR1	2
44	CYDLDLKEV PQNLKSSVEVLDLSHNRIRKL		LRR2	2

SHIFT -14!

60	VEVLDLSHN RIRKLKTSSFQRYTD		LRR1	3.4
84	IKFLMLYDN MILSVEVGTFEPLTS		LRR2	3.4
108	LQEIDLSNN GLTTIPLELFQLPR		LRR3	3.4
131	LRNLYIDSN ELTSLNLQALEKPIRAP		LRR4	3.4
157	LEYLNVAGC ELQELPDLGILPK			LRR5	2.8
179	LWQLNASMN PLQNFRIDSLANMCH		LRR6	3.4
203	LQVIDLTKS QLSQCGCQQVTNH			LRR7	2.8
225	LMMLGASPK FVPVCLEALDIRECPLPYNRT		LRR8	2.4

IHSPTFASCQTTLQFAETRSFWLFGAGCFGGVCFVLLIVIFCVIHCRRKRAQRRKRNAQK
RKPFVISPRNAINNRQPEDEPLHCDIARK

>CG5490-PA

MSRLKAASELALLVIILQLLQWPGSEASFGRDACSEMSIDGLCQCAPIMSEYEIICPANA
ENPTFRLTIQPKDYVQIMCNLTDTTDYQQLPKKLRIGEVDRVQMRRCMLPGHTPIASI

119	LDYLGIVSP TTLIFESDNLGMNITRQH		LRR1	2.4
146	LDRLHGLKR FRFTTRRLTHIPANLLTDMRN		LRR2	2.4
176	LSHLELRAN IEEMPSHLFDDLEN		LRR3	4
199	LESIEFGSN KLRQMPRGIFGKMPK		LRR4	2.8
223	LKQLNLWSN QLHNLTKHDFEGATS		LRR5	4
247	VLGIDIHDN GIEQLPHDVFAHLTN		LRR6	2.2
271	VTDINLSAN LFRSLPQGLFDHNKH		LRR7	2.8
295	LNEVRLMNN RVPLATLPSRLFANQPE		LRR8	3.4
321	LQILRLRAE LQSLPGDLFEHSTQ		LRR9	3
344	ITNISLGDN LLKTLPATLLEHQVN		LRR10	2.8
368	LLSLDLSNN RLTHLPDSLFAHTTN		LRR11	4
392	LTDLRLEDN LLTGISGDIFSNLGN		LRR12	4
416	LVTLVMSRN RLRTIDSRAFVSTNG		LRR13	3.4
440	LRHLHLDHN DIDLQQPLLDIMLQTQINSPF		LRR14	4

SHIFT -18!

452	LQQPLLDIM LQTQINSPFGYMHG		LRR1	2
475	LLTLNLRNN SIIFVYNDWKNTMLQ		LRR2	4
499	LRELDLSYN NISSLGYEDLAF			LRR3	4
520	LSQNRLHVN MTHNKIRRIALPED		LRR4	3
543	VHLGEGYNN NLVHVDLNDNPLVCDCTI		LRR5	2.2
570	LWFIQLVRG VHKPQYSRQFKLRTDRLVCSQ		LRR6	2.4

PNVLEGTPVRQIEPQTLICPLDFSDDPRERK

631	CPRGCNCHVRTYDKALVINCHSGNLTHVPRLPNLH		NF	4

SHIFT -25!

641	TYDKALVIN CHSGNLTHVPRLPNLHKNMQ		LRR1	2
670	LMELHLENN TLLRLPSANTPGYES		LRR2	4
694	VTSLHLAGN NLTSIDVDQLPTN			LRR3	3.4
716	LTHLDISWN HLQMLNATVLGFLNRTMK		LRR4	3.4
743	WRSVKLSGN				LRR5	2.8
752	PWMCDCTAKPLLLFTQDNFERIGDRNEMMCVNAEMPTRMVELSTNDICPAE		CF1	4

SHIFT -44!

759	AKPLLLFTQ DNFERIGDRNEMMCVNAEMP		LRR1	2.4
788	TRMVELSTN DICPAEKGVFIALAVVIA		LRR2	2.4
815	LTGLLAGFT AALYYKFQTEIKIWLY		LRR3	2.8
840	AHNLLLWFV TEEDLDKDKKFDAFISYSHKD		LRR4	2.4

QSFIEDYLVPQLEHGPQKFQLCVHERDWLVGGHIPENIMRSVADSRRTIIVLSQNFIKSE
WARLEFRAAHRSALNEGRSRIIVIIYSDIGDVEKLDE

967	ELKAYLKMN TYLKWGDPWFWDKLRFA		LRR1	2.4
993	LPHRRPVGN IGNGALIKTALKGST		LRR2	2
1017	DDKLELIKP SPVTPPLTTPPAEAT		LRR3	2
1041	KNPLVAQLN GVTPHQAIMIANGKNG		LRR4	2.4
1066	LTNLYTPNG KSHGNGHINGAFIINTNAKQS		LRR5	2

DV

>CG4977-PA

MSGLPIWIPLLALLAITAA

20	CPPEVCVCKWKGGKQTVECGGQQLSNLPEGMDPG		NF	4

SHIFT -11!

43	LSNLPEGMD PGTQVLNFSGNA			LRR1	2
64	LQVLQSERF LRMDLLNLQKIY			LRR2	2
85	LSRNQLIRI HEKAFRGLTNLVELD		LRR3	2
109	LSENALQNV PSETFQDYSSLMRLSLSGNPI		LRR4	2

SHIFT -11!

128	LMRLSLSGN PIRELKTSAFRHLSF		LRR1	4
152	LTTLELSNC QVERIENEAFVGMDN		LRR2	3.4
176	LEWLRLDGN RIGFIQGTHILPKS		LRR3	4
199	LHGISLHSN				LRR4	3.4
208	RWNCDCRLLDIHFWLVNYNTPLAEEPKCMEPARLKGQVIKSLQREQLACL		CF1_short	3

PEVSPQSSYTEVSEGRNMSITCLVRAIPEPKVLWLFNGQVMSNDSLMDNLHMYYYIDETI
GVSGAEEKRSEIFIYNVGAEDNGTFSCVGQNIAGTTFSNYTLRVIIKEPPVVNEVSFPRD
YMNYIVASSAGGGIIFVVLLCTIVVKCKKTSEPAKQRKKCDQVTSIAGGTDSSTGSTQDT
GMGMMKCASI

448	LNDGGDSMN GNPGLLLGDTLTPTKAANGA		LRR1	2.4
477	AGGGIILGN QMKQNLLLYATPNSA		LRR2	2.2
501	QQQLQLNVN LMGTGPGSPPLLLSN		LRR3	3
525	GHGLAAAYC SPPASLRNYQEKNPDLVNDAE		LRR4	2.2

SVKHKLKTAVSLDGAGEYETQSDCGQYEGCYQLAAAPHPHQGHQHPHPGHPLMGRFAQAM
TTLPRGMQLKPAPHQVDVHLNPVCFLGQDGSFAYDYSSAHMVQQPPQQQQQQQQVQPAN

674	NFYRTLPHN RLHKQQQFQAAAAA		LRR1	2
697	GGNVGVGGN PTLRYSLEAEFIQRGPTVSYE		LRR2	2.2

KYQLPNVRFTAEGYPQQQQQQQQQLQQQQQLQLQQQHQFPSPPEGYKSDLAVMPAPFQQW
PSCLPGYRFAQSPTSLPAVATPPPAAVVATPPPPTSAVSTQSTATSTIPELDESEASSPR
LEEAAGSAAPPAGEEESSDTAKLKQLNGPLADSPDEGYVGDGQETSDI

>CG13487-PA

MLMHRAVAFVDN

13	THLQTLHLN NNPQLSDIPMRLFQGNPN		LRR1	2
40	ILEVYMQSN SLQTLYSAQFPVDQ		LRR2	2.2
63	LQKLYLGDN PLQCNCSLLWLWRLVTGNFEG		LRR3	4

VDPGMEHAAGGAVAALAKEADDEELADEATAVASTDDGVAALAAYIAEQHIVNALHTTEP
SAYELATSSSNRNSGILRMDRQQIGCDIWRDKVRTRRKLLTMSEGEITCPAHIVTVVCAV
ITCLLVAMIGISVLYYLRFVKRRRKLLHERGPMRTSKSIINVHDRILQGHNPGGLGLGMS
MTLGGGNH

281	VNGLGMTLN YPHHAQTLQAHHHYHQAMP		LRR1	2.8
309	LQSHGGNGN HEYQQTTLPQLDKLELERYLA		LRR2	2.4

SHIFT -14!

325	LPQLDKLEL ERYLAAQTIANEYRA		LRR1	2
349	LKPWELPVK EADDEPEHLYERFDHYEYPDT		LRR2	2.4

HTMSKLKQAASLNHSNSSAGPSPVPPSSGKPHVVYV

>CG5195-PA

MMLLPIFLLLCIGINLIRAES

22	CPPSQAILPCRCSLRGKEIQIWCSHSNLPQIMDGLKAV		NF	4

ERNIKGR

67	IDELVLENN QLPALPGRFFGSLQ		LRR1	3.4
90	IVRLMLRHN SIERVSNGWLNELENGLVEIF		LRR2	3.4

SHIFT -15!

105	NGWLNELEN GLVEIFVVEPQLRSIP		LRR1	2
130	AESLNGMIN MLAITIQSEELKH			LRR2	2.8
152	LPDFSGLLS LTYLSVQTGALQELPSHLFRH		LRR3	2.2
182	LPKLQHIHI TGGSGLTRLEAGLFDGLIS		LRR4	2
210	LKNLDLSHN GLNWIHLRALSRLPN		LRR5	4
234	LVSLKLSHN QISDVGMVGRIVKDLEH		LRR6	4
260	LKKLRLDNN LITVIEDGSFVDLPN		LRR7	4
284	LSELHLNDN RITELQYGAFLRTPQ		LRR8	4
308	LKTIYLQNN LIRRIHPESLLQASGSG		LRR9	3.4
334	VEAVHMYNN EIGHVEALRALLDALPR		LRR10	2.2
360	LRYLDMSGN LLSELPYGALRGHGT		LRR11	3.4
384	LEQLHLNHN HLRLIERDALMAMPA		LRR12	4
408	LRELRMRNN SLSSDLPLPFWNLPG		LRR13	3.4
432	LKGLDLAQN QFARVDSQLLAGLPS		LRR14	4
456	LRRLDLSEN GLIELAPNSFRHNPL		LRR15	4
480	LETLNISSN ELTKIHSSTLIHLER		LRR16	3.4
504	LFEVDASYN QLKSVIAGLPRI			LRR17	2.8
525	VERISLKGN QITSLPAAASKDLQLPN		LRR18	2.8
551	LRMLDLSQN RIEQLPRHGFQGAME		LRR19	4
575	LRVLSLAQN ELRQLKDTSFIGIQR		LRR20	4
599	LELLHLQEN QLGEADERALLPLAE		LRR21	4
623	LRNLNLQSN KLEAITDNFFSNNSR		LRR22	4
647	LEQLDLSRN LIRSISPTAFDTQRS		LRR23	4
671	LEYLDLSGN ALLDISVGLGNLNN		LRR24	4
694	LRDIDLSYN QISRIQSDVIGGWRN		LRR25	3.4
718	VVEIRLSNN LIVELQQGTFRNLPK		LRR26	2.8
742	LQYLDLSSN EIRNVEPGALKGLDE		LRR27	4
766	LQEFVLADN KLVELKDHVFEE			LRR28	3.4
787	LPSLLASHF QYNKLRYISPESFHNANS		LRR29	2.4
814	LVFLNLSNN HFRNMENIGLRSMRN		LRR30	4
838	LEVLDLSTN GVKLVSTMPLKALNW		LRR31	4
862	LVELKMDNN QICRIQGSPFETMPR		LRR32	3.4
886	LRVLSMRNN QLRSIKERTFRNVRGN		LRR33	3.4
911	IAILDVDGN				LRR34	2.8
920	PIDCNCEMQWLSVWLQETNFPYPGPKCQDGRLLRSARMERSLCVGA		CF1	4

SHIFT -37!

929	WLSVWLQET NFPYPGPKCQDGRL		LRR1	2.2
952	LRSARMERS LCVGADIYGNERTD		LRR2	2.2
975	GNQLPLLNE HGDVFQRDLPDDFNDECEAGE		LRR3	2.4

GTRLPGDRPLVGESEYFYDQYVDATEAPDTTHSAISTSQRPKPTPTINSNID

1057	LNNTILHTK YFNRKPQPGSGSPFTFFGYP		LRR1	2
1086	LPSVSLGRF FGFGDRGRKQRTDSNDDMPAT		LRR2	2.4

HRMAHISLPSGRGKTRMYQPNSAEFEKYLKDQQKQEKLNIARNRYVDTDSTTSSMEDALS
NESGSAATTVGVFRTTFREPSSIERGGFRPIVPAHVGGFMPVHDPQQRRGLVEVVNITGN
PSEIVQGIGQRKFIPISTQARPKPTKSSGESSETATYEVTETTPDITTTTPLMQRIATST
TKASTTTTRSTTTTSTTQVTPAENNASSSTELDSQYDDEDLEAQSVTLLRPPPLVQTTTA
ETILLIPPAEEHVAQIKSRSWVTTTTPQSPSDNQVTLAGPTSTVPPPPPASPPLRGGGRS
TITKVYTPYQQQVAQPTAEEYQRTTPSGDNEGVASEHQLTANAQKRTELELLQVDRVDRK
DGMDWYYESFKKKRDFNGGAAVRKTAHKEVFYDGVAASSSWNRLHKEILFISLLLLWRAC

>CG11280-PA

MQRNMMIAFVGIWCILASIG

21	VEPAAGLAN CPPGCQCDDNTLVVQCGEGQ		LRR1	2.2
50	LDVLPIALN PSIQRLVIKSNKIKTIDSSIQ		LRR2	3.4

SHIFT -19!

61	IQRLVIKSN KIKTIDSSIQFYAE		LRR1	2.8
84	LTFLDLSSN HLMTIPQRTFAYQKK		LRR2	4
108	LQEVHLNHN KIGQISNKTFIGLSA		LRR3	3.4
132	VTVLNLRGN QISELHQGTFTPLLK		LRR4	3.4
156	IEELNLGEN RIGYLDPKAFDGLSQ		LRR5	3.4
180	LRILYLDDN ALTTVPDPVIFQAMPS		LRR6	4
205	LAELFLGMN TLQSIQADAFQDLKG		LRR7	4
229	LTRLELKGA SLRNISHDSFLGLQE		LRR8	3
253	LRILDLSDN RLDRIPSVGLSKLVR		LRR9	4
277	LEQLSLGQN DFEVISEGAFMGLKQ		LRR10	4
301	LKRLEVNGA LRLKRVMTGAFSDNGN		LRR11	2.4
326	LEYLNLSSN KMLLEVQEGALSGLSQ		LRR12	4
351	LKHVVLKAN ALTSLAEGLFPWKD		LRR13	3.4
374	LQTLDLSEN				LRR14	4
383	PLSCDCRVMWLHNLLVAKNASQDDVSELLCEFPERLRGESLRHLNPAMMGCT		CF1_short	3

SHIFT -42!

393	LHNLLVAKN ASQDDVSELLCEFPER		LRR1	3.4
418	LRGESLRHL NPAMMGCTHADPRKQA		LRR2	2
443	LIGALLVGS AATITALALVLYRCRHKIRE		LRR3	2.8
472	TIKGGLWGN SALGRKEREYQKTFCDEDYMS		LRR4	2.4

RHQHHPCSLGIHSTFPNTYTAPHHPGATHHYGMCPMPVNDLGAIDPQQKFQQLVVPTATM
ISEKKLNNNKALVSQGAIDDSASFVLHMKSATMGRDVHQQNPQLNHYTKPQFLSATATVG
DSCYSYADVPMVHGAPLGGPN

643	QPQLRLTQE HFKQRELYDQEMGSEI		LRR1	2
668	LDHNYIYSN THYSMPLEQLGRSKTPTPPPM		LRR2	2.4
698	PPALPLRNG LCATTGRRSFQQKSASQKQQQ		LRR3	2

NNNTLRQFTH

>CG15151-PA

MLFKWLLFSLCIMPAMFSNTKRK

24	CPTECQCSMDDLDRYQAICTKGGLNSLLSPNELD		NF	4

VDVK

62	VIIIRGPRN SITIGPALRQFMK			LRR1	2.2
84	LEILRITDS NLPAIGAESFWGLKY		LRR2	2.8
108	LRILDLSKN NITNITENNFRGQDN		LRR3	4
132	LLELDLSKN KVLRMASSTFRHLTD		LRR4	4
156	LRRLNLADN SIVELVQRNFFMLSR		LRR5	4
180	LKYLDLSGN PLQDLQPDVFRDVPE		LRR6	4
204	LKVLKCRNC QLKKINPQMYNLLPL		LRR7	2.4
228	LSELDLGRN EFKFLDKDEFRDVKR		LRR8	4
252	LTKVLLDGN QLSVVVDQLFRMQKS		LRR9	3.4
276	LNHLDLSYN RLAKVPNDSFLQLTN		LRR10	4
300	LTFLDLSYN KLVRLEPQSIRSLSN		LRR11	4
324	LLTLNISGN VLMDLREMRETFELIPQ		LRR12	3.4
350	LTHLAIADM GTMPVGLLHPFKQ			LRR13	2.4
372	LRYLNISGN SLNNTALEVIDPCRE		LRR14	3.4
396	LEFLDLSRN QLHGISEDTVLRIQG		LRR15	4
420	IRNVRLDNN PLICDECHMGKLINVVRQLQW		LRR16	2.8

KWDTYPICFLPKSLRGAEINN

471	LDINGLHTC LTFITDEEQNAASTSYNF		LRR1	2.4
498	LEHGGLNTL AILGGIIFVLIA			LRR2	2.4
519	VIILSLVAC FSKNRARYYTREDHLNG		LRR3	2.8
545	SESKCLEKN LEATTITTLGNGSSPTT		LRR4	2
571	TTTLTLATS PAAPNGPKTNGQGLSLSPANG		LRR5	2.4

STPVHGISDDKGKEINFTFPVDDRVCTIDELMPSPPPPPQQHPHPNMGSLMYSVSHSPNS
ATTLAAVAAAATVIPPGAPSHSSMPTPTPTPAEAVVVV

699	LPPPPLPPQ QHHHQMDGSLASLREVQQQ		LRR1	2
727	LVHLSSTLE PLVSVN			LRR2	2


>CG5541-PA

MRNIRNLKDPTTWTRILLPFLSCLL

26	LAVQADLVN VTCGEYGNTITCDCNNSDQT		LRR1	2
55	MALPSLRGN VYMIEIRNCRDL			LRR2	2.4
76	LVEANRLAN TMGLHKVVFRQVGQLV		LRR3	2.4
101	LREHALSVP RYASNKALIVEFE			LRR4	2
123	QTNLKLIES HAINGNIEEISFVGGRIELMK		LRR5	2.4

PFGFTTTKDSAILLKLDGVTIQRIESQAFKKFAVEQMSIANCQFLGDLPTRAFYELEV

211	TNELSLRGN QFQEVHSHAFSFKL		LRR1	3
234	VSKLSLSDN HFLSVDGEWLEAQIRDAATIR		LRR2	3.4

GNEFGATSEIAFRSLTVHRSYQLSERLELRFHNNSLRSSRPGADPRVDGTAENVAPQPLQ
FDNSFALNVRDIRYNNA

341	WSCEQLDRN VEPPLPRAEFFR			LRR1	2.4
362	LHSDQLMFH PPDFGGQGQIQPYIP		LRR2	2
386	LRSLITDEC RERSYMAFIISGSV		LRR3	2.4
409	LLGLLLLLL ILLLWWRVVQKRRRRKLDVVQ		LRR4	3

PEPRTYKETQIVYQIENAGLLKTDL

>CG14662-PA

MFKSKSFDLVIEEKTKKPERLYQPRRMRWLKYIILPAVF

40	SFALLLILV NVDFSDNSEDSTHLGNDTSL		LRR1	2
69	IISGYGFEN NTLRRGFFSGGIA			LRR2	2.2
91	LHSLVIENC TIVHINDAAFNQESTVN		LRR3	2.8
117	ITSLQLINV QLENLTESALEGLQK		LRR4	2.4
141	LQNFTLVNE NNHFRPFGFLSAVAES		LRR5	2.4
166	LVSAEIHQS LAAAISYSVCDFLGSRNFPQ		LRR6	2.2
195	LKYLDLSGT HLDKSLIKESFDNLPA		LRR7	3.4
220	LEQLLLRNC GLGNIEWEIVRPRLKL		LRR8	3.4
245	LHYLDLGGA QKTGNYEHQLDVSAFSPETTT		LRR9	3

NAEEISTILAKRAMAPEVVGTTTLGPTTSIEISPPSTQSTTTPKEESTSMTETTILTTPS
PKCEEELCQDLECSRITTDT

355	VASADLGKS SCQDGLLVEICESTCTTPT		LRR1	2.2
383	FFCVILGEN FTSASNCCSHHTMRCVVSAQV		LRR2	2.8

SWFEDHSGLVIG

425	LGVGLLFIG SFLGMLIVFGTLR			LRR1	2.4
447	LNPSWLRGN KRRESNTIGLIQGRFEKDPYE		LRR2	3

QVGGPIATIDNNEYVTAYHRYLEQANHRPTESNKYIRPPRDRAPSVPPSSDYPLPLPARN
CNVYESCELYEELP

>CG4054-PA

MPDHTRVTATTRSRPWLCGA

21	IPVLLLLLL TLVILPPETTAF		LRR1	2.4
42	CPSKCQCLGGEANSRALCVDAALEDVPIQLNPE		NF	4

SHIFT -29!

46	CQCLGGEAN SRALCVDAALEDVPIQLNPE		LRR2	2.4
75	TKYINLTVN RIRTLEFSLPFYMK		LRR3	2.4
98	LEILDLSQN IIETLGSKNFEYQSE		LRR4	4
122	LRTLNLSRN LVSSLHKHAFKGLTN		LRR5	4
146	LLLLDLSFN RIETVHPTALSDLAS		LRR6	4
170	LVELDLTNN NIVSLEDNCFKGMNT		LRR7	4
194	LEVLVFRNN RLLDVPASNLWHLHA		LRR8	3.4
218	LKSLDMSLN LVEFVRNDSFEGLKE		LRR9	3.4
242	LLALSVQGN VMSELDLSAFEGLIS		LRR10	3.4
266	LKHLDLSDN NLTRISHPLERLV		LRR11	4


>CG18241-PA

MEHSK

6	LWDLRPEVR ERRFKWTSDGQQQQ		LRR1	2
29	QQQLGWCNN KDDPPNSHQKSKSNNDARNLN		LRR2	2.4

SRVRVRARVRVVPGEMRMGDINCSNGLGNIREDYCE

95	IYLDELGEN GTCSIANNEVTTEDYQMK		LRR1	2.4
122	LVFLKLEIN WTSPVFHGWNIFKICNETDYE		LRR2	4

LVIISVLGIRSEVDMRISPAVQYLSLLGIREISGYDIYLPSVLITEMDVHHANGPKMVTF
KYLYDSTVNSVITNNYIRKTMNNTEKIKIYYHNTFE

248	KTTLTMEKN IFHGKNKMSALIFNG		LRR1	2.4
272	LKIKGLTNN TFENLTSLNTLIFDNVF		LRR2	3
298	LKDLSFLRS STLQSSLTYCIMKVDNMVD		LRR3	2.8
326	LKSFEKFTN LEIIEVSQYKGFKNFTAFICE		LRR4	2.4

PYKS

360	HCKFTLGIN EVA		LRR1	2.4
372	CPLKCNCSYNRDKSQLEIDCWQKNLTTIPSLPVPK		NF	4

SHIFT -20!

387	LEIDCWQKN LTTIPSLPVPKKG			LRR2	2.4
409	SSALVFQSN LLAELPDNSLEGYHN		LRR3	2.4
433	LKSLDVSYN QLTSLSVSQLPES			LRR4	3.4
455	LHYLDIRHN KITTLSPQVVEYLYSVNVFNQ		LRR5	3.4

YGNKWSIYCDEYHLQEFFWYKAKLLRIKTSKFQTIMEYIELSSKGSFVENFFVQN

540	IDQLYLEAN EDEIIDAFGPSDK			LRR1	3.4
562	YFNLKLMEA LNHAIWLFSGEFDEII		LRR2	2
587	LHHLNSPCP YRCSCCFEWHTGEFLIN		LRR3	2
613	CRNLSLDIY PRLPNSIPYKTTLYLDRNEIR		LRR4	2

SHIFT -12!

631	KTTLYLDRN EIRKLTNTESLVVAGHAS		LRR1	3
658	IHKLHMSQN LLRELPLHLLPEN			LRR2	2.8
680	ITYLDVRNN LLKYLDDGVIAFLEYREN		LRR3	2.8
707	ITKIELSGN				LRR4	2.8
716	PWECNCKAKAFLSFLRRHEPMEYETVLRRVEITDDKCPEDCICCVDTSNSDSLAYVVDCSGK		CF1	4

ELSEIPQLPTPTYG

792	QTTLVFERN SLKKWPSSLLPGYSS		LRR1	2.4
816	VTRFYLAHN RLSDIDQLPDKLEYLDISNNN		LRR2	2.8

SHIFT -10!

836	LEYLDISNN NFSALDDRVRGFLQKRMNSS		LRR1	3.4
865	QLQLSLFGN				LRR2	3
874	PWTCRCEDKDFLVFVKEQAKNIANASAIQCIDTGRSLIEVEETDICPSV		CF1	4

LIYYTS

929	LAVSLLIIA LSINVFICFRQPIMIWFYEH		LRR1	2
958	EICLSLAAR RELDEDKKYDAFLSFTHKDED		LRR2	2

LIEEFVDRLENGRHKFRLCFYLRDWLVGESIPDCINQSVKGSRRIIILMTKNFLKSTWGR
LEFRLALHATSRDRCKRLIVVLYPDVEHFDDLDSELRAYMVLNTYLDRNNPNFWNKLMYS
MPHASHLKRSRSDAETKV

>CG6749-PA

MSQTA

6	NWVLPLLLW LLCMGFPILHGAD			LRR1	2
28	LCQPIGWRN FQCSEVASLQDLVDLGAENWH		LRR2	2.4

TLAIRNVQTELEVGSGENADH

79	LANLLDLDL TGAAPINVHTNGFSILPN		LRR1	2
106	LRQLNLSGC GLVDIRGNHFAPESA		LRR2	3.4
130	LQRIDFSHN QMELLDRDFFGNLRK		LRR3	2.8
154	LIYANFSHN ALKQCDLPHMPL			LRR4	2.8
175	LNRLELGHN RLVNATFGVCPQ			LRR5	4
196	LQELILNDN QLIQLDVNAFRGLHG		LRR6	4
220	LLELQLSGN RLSSIGLETFQPLAQ		LRR7	4
244	LRKLNLSQN ALDALRPNVFGAVQNFVLH		LRR8	4
272	LQQLDLSGN RIRLLFDNQFRVLAR		LRR9	4
296	LQMLDVSRN SIASLSPAHFVGLGS		LRR10	3.4
320	LRKLYLQYN AILEIKPATFAALLN		LRR11	4
344	LDTLDLSYN NLEFLEEQIFGGNTLPR		LRR12	4
370	MRRLNLNGN RMKHLHPLAFSSLPF		LRR13	3.4
394	LEYLKLGHN ELKSLDVRMFAPMRR		LRR14	4
418	LQKLHLGHN LLEEINLDVLESLSS		LRR15	4
442	VQEILVDNN RLTFLAKVNVSFPN		LRR16	2.2
465	LKRVAIEGN				LRR17	2.8
474	PWQCPCFVKLQHWLATRDVVYLRDNTGYYKGERPLCIVTNVDYCIQNLQAVRRLGILG		CF1_short	3

SHIFT -67!

465	LKRVAIEGN PWQCPCFVKLQHWLAT		LRR17	2.8
490	RDVVYLRDN TGYYKGERPLCIVTNVDYCIQ		LRR18	2.4

SHIFT -15!

505	ERPLCIVTN VDYCIQNLQAVRR			LRR1	2.4
527	LGILGDFQG EQVEADAFDDDASAAQD		LRR2	2


>CG18480-PA

MALQCVTKPKMWLGLKQI

19	LLYVGLLLC VLLQVCRSKTQSQMF		LRR1	2.8
43	CPTVCHCDLHAQRNRAVCSAKRLISANIEIPTT		NF	4

SHIFT -28!

48	HCDLHAQRN RAVCSAKRLISANIEIPTT		LRR2	2.4
76	VELLDLSYN DITTIDDDSFKTTIH		LRR3	3.4
100	LLNLTLAHN AIHTLYGDAFVELTR		LRR4	4
124	LRYLDLSYN RLEQIDEHILESNNQ		LRR5	4
148	LIHLNLEGN KLSTLGKGPILRSPS		LRR6	4
172	LRSLNLRNS QVNQLGTQLLSALPQ		LRR7	3.4
196	LRQLDLAQN LLLTLSPGDFHAPRN		LRR8	4
220	LASLNVEEN PFNCDRALAKVATGLRQRGVA		LRR9	3.4

IFMSNCMEEEVQDHQDDAEAVNFPNKNEKFESMEYLEPSTRGPQSVLSVWRDLDSSEEQN
SSQDDEQMSSLSDVCEGSREKLCLRYRICLERV

343	SHELLAGGN SQLEDEIIRTHTYDEDDLK		LRR1	2.4
371	LAFVVGGAT GVCMVIFIITFALCLKSCCEM		LRR2	2.2

SHIFT -12!

389	TFALCLKSC CEMRKKKSNPEV			LRR1	2.4
410	ATGVSAPLN PSQDSLPTIHTWSPQRTRNPR		LRR2	2.2

RSNPQRPRSTQSHAVVRQPYGPQDNFVSRLFGRPARSQYYRTINQNTATLIRRLSRSNLF
TSRDREPSSPAEPPTSTARFYTDVIESGAIRPETPPPNYGDVVVIENCDNK

>CG4950-PA

MAVCEIPLMQFTKLFGGERFVKKMNRTRRILEVIVILILAISSCDGSGGDIPLRCDEYDS
MGYMDVNEAFCTVTGFTVTHSQNVVIKNIPPGNMVKFMKFYESTLLYMPFHLFETFPY

119	LKTLDVSNT NILELTRNAFSAASN		LRR1	2.8
143	LTYLNLAYN NLTSIQTSVFIGANV		LRR2	4
167	LMRLDLSYN EISSLSVNAFCGLHT		LRR3	4
191	ISQIYLTGN LLKELHNDIFKDNEY		LRR4	2.8
215	LEKVSFEGN LLTSIQPEVFRNMRR		LRR5	2.8
239	IKEVNLSNN RLIFIHPDTFADAAS		LRR6	2.8
263	LENLVLSYN ELKNFQLTEKNI			LRR7	4
284	VHQLHLDNN YLTNLTINATRFVRASHNQ		LRR8	3.4
312	ISELFLHQS LHIETLDLSANK			LRR9	2.8
333	LSSISNITN ITHMLYLDVSDNPIGPLNIST		LRR10	2.4

SHIFT -20!

343	THMLYLDVS DNPIGPLNISTFSQLKR		LRR1	2.4
369	LRGLNLRGT GIRELKFGMFSKQKY		LRR2	3.4
393	LEELDLSFN NLTILNLDMFVPYLTN		LRR3	4
418	LKKFLIDGN GLTELQGNRTFSEAFPQ		LRR4	2.8
444	LQKLGVSRN RFNCSYLHHLLIPPSLPESVV		LRR5	3.4

LNIEPDTNLDETPHIRDVSCISQSQEVVNASFTAEESKLESQIQMLSKQLDIVGSH

530	SKNLELHLA FMQIFAYVMGGLVLVGVVS		LRR1	2
558	LITLRYLKN QRSGRYDRSSIVFRSNATM		LRR2	3
586	EANLTLGSE DLQ			LRR3	2


>CG5819-PA

MRI

4	VLELALLVL GVISS		LRR1	2.4
18	CRAVNCPWPCRCTWVVDSLYADCSRRSLQTYPNFDGIP		NF	4

SHIFT -23!

33	VDSLYADCS RRSLQTYPNFDGIP		LRR2	2.2
56	VEHLDLSGN KFLEFPTLYADIDS		LRR3	3.4
79	LIYLDLSSN YISSIGAKTLIGFTS		LRR4	4
103	LRTLLLANN SIDSWESLSPNEAFKYAPS		LRR5	4
131	LKRLGLDGN RLGSFGNGESFELLTSSS		LRR6	4
158	LTDLGLSSC GISSIGGDQMVNQLPN		LRR7	3.4
183	LERLNLANN QLAQIAALPSRT			LRR8	4
204	LRVLDLSNC SIKNLSGFFLDAMQN		LRR9	3.4
228	LEALNLSRN TELQFDSLSEDPILTYM		LRR10	4
254	LRKLDVSYC NLDSIELSGLPQ			LRR11	2.8
275	LTEVRLQGN LLRVVDVNTFANNSM		LRR12	3.4
299	LEVVDLSQN VLRHIGQDAFAKLKR		LRR13	3.4
323	LKELNLAFN EIARLDRNFIRNNDV		LRR14	4
347	LVELNLSRN VLQKLTKIVSNSVRTINMSWC		LRR15	4

SHIFT -20!

357	LQKLTKIVS NSVRTINMSWCEITSIESTA		LRR1	2.4
386	LSSLSVIQK LDLSNNLITDMPTFMRSET		LRR2	2.4
414	LQQLNLANC RLTTVRNNTFREFPE		LRR3	3.4
438	LADLHLNGN RLTSPIPPNYFDGNKF		LRR4	4
463	LDQLWLGDN				LRR5	4
472	PWICDCHSPLFVDFYDYLTAKPAKIKDRNHLRCAAPAVFYGKLWEFACADV		CF1	4

SHIFT -1!

522	VWILNARTS STGEKAWSIIMLTLLG		LRR1	2.2
547	LGALVLGYA CLQKYLRKRKVRQSDREYEEN		LRR2	3

DDELRRIRD

586	LNERILREE ATPSLQHTQEISLLPSYEDA		LRR1	2
615	LRMPKLVRP VKSMMDLSGPERARNSRKLRR		LRR2	2

SHIFT -15!

630	LSGPERARN SRKLRRSQTHADGDGSQSEA		LRR1	2
659	EDGLQLDSR QRFRSVEMLSNRDKERTAQYG		LRR2	2

PYRRTGYMEYNQSGSRRFSIEDSRFPAAHLKTQNLQSAEQIGNFQSYENSPYTKRKPKIA
EIPPFKRVNMMADSVEFLTDPEYDEVGSKHGSPFAKRKPKPPVLPPPTMKVSAQVYTLQQ
SPVLELDPAIEDYFSAARKQPSSSTIASDFQELDEPELKSLPDDNRSSTISRSDVEQDLE
RGKRKKRKNSTSRRVSGSFTAANAAESSSSDSELNALVHKPMRETLF

>CG5096-PA

MNLTKSATLAAAIFCIVLSQVSGQTKDETTTTETPKEKIKVIDSKL

47	CKKCNCYIDTNLLDCSEKLQDWLSAEDWED		NF	4

LTNGNVV

84	FKTINLEHN NLTSVPILPKYD			LRR1	2.8
105	VENLYLANN QIDSISVGAFQNLTE		LRR2	3.4
129	LVTLDLSHN RLTSKVLVPDVFKGPFTVQDF		LRR3	4

SHIFT -25!

134	LSHNRLTSK VLVPDVFKGPFTVQDFESLEN		LRR1	2
164	LKTLNLGYN DLHSLDADLFEHIPH		LRR2	4
188	IEELVLCSN SFHVIDQLSETAISG		LRR3	3.4
212	LQSLKILDV SYMEIDDLPDTILHGPRD		LRR4	2.4
239	LEIFIAAGN LFNQLPKALKYATN		LRR5	2.8
262	LTSLVLNEN PIENLIGDNVFPPLTK		LRR6	4
287	LTHLSMTFM SKLYKIGPGAFSELQS		LRR7	2.4
312	LTELILSDN KLLNEIDEEALSKNVTGGQY		LRR8	4
341	LDYPPLEKV YLNNCNVSTLPKELLVRWDK		LRR9	2
370	LKALDLRFN PWNCDESNDFLINVLIDRINK		LRR10	4

SHIFT -10!

390	INVLIDRIN KTTPVLAKDVKCGGPNK		LRR1	2.4
416	LNDVTLLRV ANEHMIESSTGS			LRR2	2.4
437	LIWVGLLVV LLIAVPTIIGAYVMKRRGCFG		LRR3	2.4

VFRRHDSGASSALYNRTSFNEDFHI

>CG5528-PA

MCPKYIWDVIVLVCLFLGNVREAYTEFSIQDGLIIEPDSATTSSEEAEEVSKERTD

57	LKSLMLKYE SDDGNSCLLDLIKDEVIWWQ		LRR1	3
86	FPNGTLRDS TKKYAHKLYLDLSHGNLKDDS		LRR2	2.2

DLFREAKLSRKVTIWRTEVFSAAFNTLTAAPFRTLYSMRESLKLLSLRGNNFAELIPDAE
DFARFVNESRLEASNSVPHHCELLLLHNTTDLYDRECYLYFNNNTNMGQSITTGRNYTNF
IKVLKDRFDQHGSSSQSIAWATFPKMPR

264	LVELDISNC SIEYVSKEAFRNVSN		LRR1	2.8
288	LRRLFMSDN KIMTISHDTFYYVQG		LRR2	3.4
312	VQYLDLSFT NFLTYSYQLQLPTLEMALSL		LRR3	2.8
341	IYGLKIQQN VFKYLPELIYLD			LRR4	2.8
362	LSHSKMTRN SAVAFAHLGDKLKFLSLCYTA		LRR5	2.4

SHIFT -10!

382	LKFLSLCYT AIPMVSSTIFKNTV		LRR1	3.4
405	LEGLDLSGN PYLSYNIIDDAFDGIANT		LRR2	4
432	LKYLYFERS NIKDLEWSKSLKN			LRR3	2.8
454	LQVLGLAGN NINALTPAMFQSLES		LRR4	4
478	LEILDLSSN HVGNWYRSAFHNNSA		LRR5	4
502	LRVLNLRSN TINMLSNEMLKDFER		LRR6	4
526	LDYLSLGDN DFICDCHLRAVVEVAAANNKD		LRR7	4

ADCSYRLLNYSQNAVGEEVISLAESLIIDRKLWQSRYIPWLQRSYSNIREFNRANHIIKL
RFSSEDYMVAKCSAAQPYH

635	LGDLDGDLT LKFQLLDYEASQYYCFNNTDQ		LRR1	2.8
665	LQVDELNCQ IRSMSDLAEELHHVTNTVIAV		LRR2	2

MGSLVGACILGFIIYLKRWHIHYYYSSLKSAALLSSASKESVNKFTNISQRDPSAVYDIF
ISYCQNDRTWVLNELLPNVEETGDVSICLHERDFQIGVTILDNIISCMDRSYSLMLIISS
KFLLSHWCQFEMYLAQHRIFEVSKEHLILVFLEDIPRRKRPKT

858	LQYLMDVKT YIKWPTAKEDRK			LRR1	2.4
879	LFWKRLKRS LEVIGINSREISV		LRR2	2.4


>CG7896-PA

MEAWKQLPNG

11	GRFLLLLLA CIAHLGHPILAWRPCPELSP		LRR1	2.4
40	ALRLPCRCN VVPFAATGQLGAVAMDCDRVV		LRR2	2.4

FHGDAPQLPYGAPIVAYTQRHSGQQVLPAQT

101	FGQLKLTIE ELDLSYNLIRRIPEKA		LRR1	2.4
126	FDGLKDSLN ELRLANNLLGDNLNPIFS		LRR2	2.4
153	TAELHVLKN LRLLDLSGNKIKL			LRR3	2.4
175	IEEGLLKGC MDLKEFYIDRNSLTSVPTNSL		LRR4	2.2

SHIFT -19!

186	LKEFYIDRN SLTSVPTNSLNGPSA		LRR1	2.8
210	LRHLSLRQN QIGSLLADSFNAQRQ		LRR2	4
234	LEIIDLRHN VIRSIDSLAFKGLQK		LRR3	3.4
258	IREIKLAGN RISHLNSDVFEKLQS		LRR4	2.8
282	LQKLDLSEN FFGQFPTVALAAVPG		LRR5	4
306	LKHLNLSSN MLQQLDYTHMQVVRS		LRR6	4
330	LESLDISRN TITTITPGTFREMGA		LRR7	3.4
354	LKYLDLSLN SLRTIEDDALEGLDS		LRR8	4
378	LQTLIIKDN NILLVPGSALGRLPQ		LRR9	3.4
402	LTSLQLDYN RVAALSAEILGSLQAGD		LRR10	4
428	ITTLSLSRN VIRELPPGSFQMFSS		LRR11	3.4
452	LHTLDLSGN SLAVINADTFAGLEST		LRR12	4
477	LMALKLSQN RLTGLGGAPWVLPE		LRR13	4
500	LRSLDLSGN TLTELPSTIFEELEN		LRR14	4
524	VQSLNLSGN HLTPLTGALFKPLDR		LRR15	3.4
548	LQVIDLSGC NIRQISGDLLAGLQD		LRR16	2.8
572	LKHIYLNDN QLQELQDGSFVNLWN		LRR17	3.4
596	ISSIDLSNN RIGSIRSGAFVNVMK		LRR18	2.8
620	LQKLDLHGN QLSAFKGEYFNTGTG		LRR19	4
644	IEELDISDN QLSYLFPSSFRIHPR		LRR20	2.8
668	LREIRAANN KFSFFPAELISTLQY		LRR21	2.8
692	LEHIDLSHN QLKTIEELDFARLPR		LRR22	3.4
716	LRVLLVANN QLDMVSEMAFHNSTQ		LRR23	3.4
740	LQILDLAHN NLDRIGERTFEGLVR		LRR24	4
764	LEQLNLEGN RLSELSDGVFERTKLQM		LRR25	4
790	LENINLAHN RFEYAPLNALQRQFFF		LRR26	3.4
815	VSSVDLSHN KIKELPGDDSIMVN		LRR27	2.8
838	IKRIDLSFN PLSSKAVHNVLNEPKT		LRR28	2.8
863	VRELSLAGT GIENLELLETPF			LRR29	2.8
884	LQFLNLSHN KLKNVKPEVFQRVTL		LRR30	4
908	LETLDLSSN QLESLEDLSMAWPQLQV		LRR31	4
934	LQSLDVSNN SFEIVSQSNFGKLEM		LRR32	3.4
958	LRSLRLSHL PQCTRIEKNAFKQLPN		LRR33	3
983	LVSLEAYDL PLLGYLDLQGILEL		LRR34	2.4
1006	LPGLEVLDI EVKDSSIGSEQIQPLKHPR		LRR35	2.4
1034	LKSLGIRGD RLKSISSGTLAG			LRR36	2.4
1055	LKSNDLSVQ LRNTSLNALPPALLFPV		LRR37	2
1081	PRSSHLSLN VEGSKITVLVPQF			LRR38	2
1103	LNALEDRRA SLQLQGLASNPIVCDCNARAL		LRR39	2

RRWLPSSGMPDVTCASPAYLLNRKLIEVGDDELTCDARRMTSSTSRPTASVPHLLKTSSQ
LVTRSSSTTEEPLIIWSLEPTQPPSLKKMKTKAPLMKAQSPIISNDDTLIIGIVGGVVAF
IAILIIIICIIRLRMSNAEYQQNATMIGIPAGMQMGAHNAAYNYKNGAGAALYAVPPYHA
TLPHKAASIHQSSQNLSQRQQQQQQQQQVAAAAAAYSTMSRMSYFSGAGGGNGDGAESLT
HQHPHQHQPYIIYSDDKAYR

>CG7503-PA

MLPHHQRHKMATLADSAICFLLLSLL

27	LIGACLVTP TEGRAKDDRRTRGRGSSSGV		LRR1	2.4
56	LSSSSSSSN NMNNGYYSGSSSTAGSSSGYV		LRR2	2

FTSSSAVNSGSTGYSGPMDSTGFCTRRRDMKLMCYCTPDENHVPVQKAECW

137	VFSEGLHQN DTTWTRFYQQKR			LRR1	2.4
158	LRELKFVIQ NNARLDYIPTMIIEPLKN		LRR2	2.4
185	LSSIVIEYS QVEIVKSYAFANLPF		LRR3	2.2
209	LERIILNNN HIMALDQDAFANHIR		LRR4	3.4
233	LRELNLEHN QIFEMDRYAFRNLPL		LRR5	4
257	CERLFLNNN NISTLHEGLFADMAR		LRR6	3
281	LTFLNLAHN QINVLTSEIFRGLGN		LRR7	4
305	LNVLKLTRN NLNFIGDTVFAELWS		LRR8	4
329	LSELELDDN RIERISERALDGLNT		LRR9	4
353	LKTLNLRNN LLKKIDNGLLRGTPA		LRR10	4
377	LLSINVQAN KLETLTFYTFQPIMDNLVNS		LRR11	2.8
406	TSELLVSDN				LRR12	2.4
415	KFICDCRLQWIFELKNRTRHLQLRDSLEDLHCTLQEPKLSHFVDPVPPTILDVL		CF1_short	3

SHIFT -63!

406	TSELLVSDN KFICDCRLQWIFELKNR		LRR12	2.4
432	TRHLQLRDS LEDLHCTLQEPKLSHFVDPVP		LRR13	2.4

SHIFT -21!

441	LEDLHCTLQ EPKLSHFVDPVPPTI		LRR1	2
465	LDVLNIGGF TAIGSNSASMGGVGNSVVGSS		LRR2	2.4

YSGLTMDDSRKHLGSRSRQALRGQRQFASSAENVVESKMRRRRKRQEEVKEKDLAAVAPA
HKRYDYYDDNNGGMSLGHGLDLDDNLSLHKQGFYGAGSPVVGHNDVDVLMTSHSASGDA

614	LDILTTKNA IYIKLFLLKPEMLPCHDELSD		LRR1	2
644	PTELPLSRD LMDVRSNVGQDMSTAGANSLA		LRR2	2

QGMTIIVSLVALMMISRG

>CG8852-PA

MGSSHFSVAMTQCVFLVVVSQILSHVRAAGDAMSSANESISLPPACVIRATLYKTSSSER
SQNDFGLPLAIDCSENSSMGMNYFDLGAQRVAGKRHVSLDGFQTPPLGIS

111	SYGLEYLDN CVDLESVEIQRFVGDATLKL		LRR1	2
140	SCGGALIPN LTAVSFRYNELGTLSGETIQD		LRR2	2.4
170	LPHLKILHL QQNSLRYLEYFKEHSD		LRR3	2.4
195	LEELLIQDE RDLVLRFNEILNQLPE		LRR4	2.4
220	LRRLSLRNV EKIEDQFLRILPEN		LRR5	3
243	LTDLIIENT PIQPGVLYLTEGVAK		LRR6	2.8
267	LVNVTITNC QLKGFALQPAHNLD		LRR7	2.2
290	IMYLNLSGN AISSLNISFESGPST		LRR8	3.4
314	LLTLDLSRN RLEHLNFTWFYRTTS		LRR9	4
338	LRSLHLQEN HFHSLSLFQLGFISSAS		LRR10	4
364	IHHIDLRSN ELMSFRDTENADLPSWNPQLR		LRR11	2.8

ISIDDNPWSCQWMLNFSHMQPQLFRLFQYSKYISHININGLSCTPQEPPSEEPPKVRRIM
HVRINGTDSSIPVPHPHGNV

474	SSFTVLYGN PVELHRSQRSLALII		LRR1	2
498	VFMLPLGIA FLFLLLYLYLHCER		LRR2	2.4
521	LFHLSYYAS GLPCFGGEKSSSGPRFVDHVD		LRR3	2.4

IVRYPIANGGSSCPVDVETELPDGYETPVSGGTSICNCTGHRESTCSRTHHVTYESLPAE
LPYQLYAEIKEQADENGDADEVLAVTTGPTAPIYDHLSFVEEMPKLDELRDIS

>CG1744-PA

MGLEFFFKFGYAFLTITLMIMIWMSLARASMFDREMEETHYPP

44	CTYNVMCTCSKSSTDLGIVHCKNVPFPALPRMVNQS		NF	4

K

81	VFMLHMENT GLREIEPYFLQSTG		LRR1	2.2
104	MYRLKISGN HLTEIPDDAFTGLERS		LRR2	2.8
129	LWELILPQN DLVEIPSKSLRHLQK		LRR3	4
153	LRHLDLGYN HITHIQHDSFRGLEDS		LRR4	4
178	LQTLILREN CISQLMSHSFSGLLI		LRR5	4
202	LETLDLSGN NLFEIDPNVFVDGMPR		LRR6	4
227	LTRLLLTDN ILSEIPYDALGPLKS		LRR7	4
251	LRTLDISHN VIWSLSGNETYEIKASTKLN		LRR8	3.4
280	LDNLHLEYN HIEVLPPNSFKYFDTVNRTFF		LRR9	4

DGNPIHTLREDAFKPARIREIYMRYCGLTNISPVAFDSLVNS

352	LQILDLSGN NLTKLHHKLFNNFDV		LRR1	4
376	LRVISMRDN KIKIQKPTETFNAVHYT		LRR2	2.8
402	LLKLDLSGD RNDPTNLQTLRNMTRMRNMRS		LRR3	3
432	LSISRLGSS SVGPEDFKDFGVE			LRR4	2.4
454	LEDLQITRA SLSGIQSHAFKHVRG		LRR5	2.4
478	LKRLDFSEN GISSIENDAFHEIGHS		LRR6	3.4
503	LISLKMSHG YSGSALPAEPLRHLTS		LRR7	2.4
528	LQELDFSNN HISSMSDTSFHFLKN		LRR8	3.4
552	LRLLELHDN RIEQVLKGTFQGDIHSK		LRR9	4
578	LEEISLRFN HLTSISQHTFFDLEA		LRR10	3.4
602	LRKLHLDDN KIDKIERRAFMNLDE		LRR11	4
626	LEYLSLRGN KINNLADESFQNLPK		LRR12	4
650	LEILDMAFN QLPNFNFDYFDQVGT		LRR13	3.4
674	LSNLNVNVS HNQIRQLMYNSSWSGRNEHGG		LRR14	2.8

SHIFT -20!

684	NQIRQLMYN SSWSGRNEHGGMYHSN		LRR1	2
709	IKILDLSHN NISIIHPGYFRPAEIS		LRR2	3.4
734	LTHLHLGYN SLMNTTRDVFGNMPH		LRR3	4
758	LQWLDLSYN WIHELDFDAFKNTKQ		LRR4	4
782	LQLVFFGHN YLSDIPQDIFKPVQG		LRR5	2.8
806	LRIVDFSHN HLRGLPDNLFYNGG		LRR6	2.8
829	MEKLDVSHN MMLKIPSSSLSSLAALT		LRR7	2.8
855	LCELHLSNN FISTIHSMDLSNKFRS		LRR8	4
880	LRYLDISYN YLLRIDDAVFATMPK		LRR9	3.4
904	LAVLDLSHN RDLKVMDKSFMGLENS		LRR10	4
929	LIKLGLENI SLSTVPEIRLKY			LRR11	3
950	LREFRLGYN ELPSIPQELAHNMSN		LRR12	3.4
974	LRMLDLSNN DLTNVPLMTQALPH		LRR13	4
997	LRRLMLSGN PITSLNNNSFDGVNED		LRR14	4
1022	LEMLDISNF RLHYFEYGCLDSLPH		LRR15	2.4
1046	LRSLKLTAY SHLEHFNIPHLLRHHYNIRQL		LRR16	3

WIEAPQPFTRIVKKGSGPTQEMQTLQLGNPTDLQREMEGHLPSKLTNITFSGPQFTN

1133	LNERILRGM RSPYLYMQLFNTS			LRR1	2
1155	LQALPPNFF KYMGRVRNISLDIRYH		LRR2	2
1180	NRNLKKIPN PNTGAVPYLPNSVF		LRR3	2
1203	LTDLKMSHT				LRR4	2.8
1212	DLNCDCDLGWVEFWQRKRRQYICSSQTWTDTVFRTFMNSPCQVYGRHNCDEHDDDLRETRCEN		CF1_short	3

KGGQQLMEALKFDLECGWDNANCREAAFVVVMVCVAMVFWM

>CG12002-PA

MRFMLLMLQLLGLLLLLAGGVQSVY

26	CPAGCTCLERTVRCIRAKLSAVPKLPQD		NF	4
54	TQTLDLRFN HIEELPANAFSGLAQ		LRR1	3
78	LTTLFLNDN ELAYLQDGALNGLTA		LRR2	4
102	LRFVYLNNN RLSRLPATIFQRMPR		LRR3	3.4
126	LEAIFLENN DIWQLPAGLFDNLPR		LRR4	3.4
150	LNRLIMYNN KLTQLPVDGFNRLNN		LRR5	3.4
174	LKRLRLDGN				LRR6	4
183	AIDCNCGVYSLWRRWHLDVQRQLVSISLTCAAPQMLQNQGFSSLGEHHFKCA		CF1_short	3

SHIFT -56!

179	LDGNAIDCN CGVYSLWRRWHLDVQRQ		LRR1	2.4
205	LVSISLTCA APQMLQNQGFSSLGEHHFKCA		LRR2	2.4

KPQFLVAPQDAQVAAGEQVELSCEVTGLPRPQITWMHNTQELGLEEQAQAEILPSGSLLI
RSADTSDMGIYQCIARNEMGALRSQPVRLVVNGGNHPLDSPIDARSNQVWADAGTPMHGA
TPLPSPLPSPPHFTHQPHDQIVALHGSGHVLLDCAASGWPQPDIQWFVNGRQLLQSTPSL
QLQANGSLIL

425	LQPNQLSAG TYRCEARNSLGSVQATARIE		LRR1	2
454	LKELPEILT APQSQTIKLGKAFVLECDADG		LRR2	2.4

SHIFT -13!

471	LGKAFVLEC DADGNPLPTIDWQ			LRR1	2.2
493	LNGVPLPGN TPDLQLENENTELVVGAARQE		LRR2	3.4

HAGVYRCTAHNENGETSVEATIKVERSQSPPQLAIEPSNLVAITGTTIELPCQADQPEDG
LQISWRHDGRLIDPNVQLAEKYQISGAGSLFVKNVTIPDGGRYECQLKNQFGRAS

638	ASALVTIRN NVDLAPGDRYVRIAFAEA		LRR1	2.4
665	AKEIDLAIN NTLDMLFSNRSDKAPPNYGEL		LRR2	2.8

LRVFRFPTGEARQLARAAEIYERTLVNIRKHVQEGDNLTMKSEEYEFRDLLSREHLHLVA
ELSGCMEHREMPNCTDMCFHSRYRSIDGTCNNLQHPTWGASLTAFRRLAPPIYENGFSMP
VGWTKGMLYSGHAKPSARLVSTSLVATKEITPDARITHMVMQWGQFLDHDLDHAIPSVSS
ESWDGIDCKKSCEMAPPCYPIEVPPNDPRVRNRRCIDVVRSSAICGSGMTSLFFDSVQHR
EQINQLTSYIDASQVYGYSTAFAQELRNLTSQEGLLRVGVHFPRQKDMLPFAAPQDGMDC
RRNLDENTMSCFVSGDIRVNEQVGLLAMHTIWMREHNRIASKLKQINSHWDGDTLYQEAR
KIVGAQMQHITFKQWLPLIIGESGMEMMGEYQGYNPQLNPSIANEFATAA

1105	LRFGHTIIN PILHRLNETFQPIP		LRR1	2.4
1128	QGHLLLHKA FFAPWRLAYEGGVDP		LRR2	2
1152	LMRGFLAVP AKLKTPDQNLNTELTEKLFQT		LRR3	2.4

SHIFT -19!

1163	LKTPDQNLN TELTEKLFQTAH			LRR1	2
1184	AVALDLAAI NIQRGRDHGMPGYNVYRK		LRR2	2.4
1211	LCNLTVAQD FEDLAGEISSAEIRQKMKELY		LRR3	2.4

GHPDNVDVWLGGILEDQVEGGKVGPLFQCLLVEQFRRLRDGDRLYYENPGVFSPEQLTQI
KQANFGRVLCDVGDNFDQVTENVFILAKHQGGYKKCEDIIGINLYLWQECGRCNSPPAIF
DSYIPQTYTKRSNRQKRDLGKENDEVATAESYDSP

1396	LESLYDVNE ERVSGLEELIGSFQKE		LRR1	2
1421	LKKLHKKLR KLEDSCNSADSEPVAQVVQLA		LRR2	2

AAPPQLVSKPKRSHCVDDKGTTRLNNEVWSPDVCTKCNCFHGQVNCLRERCGEVSCPPGV
DPLTPPEACCPHCPMVK

>CG4781-PA

MARLEVIRGLTFALLLTQLLAAHVARAEVIDAEETDRFCYPESSKNSRRSCECSNVSASP
WGNRALRIDCSYKDYK

77	VADLSLLLP LYIDSLDLSWNA			LRR1	2.4
98	LDSVPIFTS DSLHQLNLRHNNISQLVSGNF		LRR2	2.2

SHIFT -19!

109	LHQLNLRHN NISQLVSGNFKQLTS		LRR1	4
133	LRELYLGWN SIGKLESGSFDGLPH		LRR2	4
157	LQVLDLAHN NLHLLPGHLFAPLLV		LRR3	4
181	LGTLDISWN RRFNESGGDLYTGLGVNWK		LRR4	3.4
209	LSTLRLDAC SLNDLHLPVNAP			LRR5	3.4
230	LKELSLRRN QLKRIPTQLPET			LRR6	4
251	LLRLDISDN LLEELLPEDTANLTQVRQLFI		LRR7	3.4

EDMPVLQRVVANSLTHVDVLETLSFQNSRQ

311	LSHLDAEAF GPIMTTPTKKRA			LRR1	2.4
332	LRSLSFRGT MLRTFNSTLAPIFTQ		LRR2	2.8
356	LAELDLNGL PLQCDCELVWLKQLPVQTNGR		LRR3	3

CYKPARIRGMLVTSARGDAFSCDTWPRWAYGLVVLSLIALSAAGIYLIVMGLRPHRGVTM
RRKVGAGSPYARVTIEPNRQENPH

>CG1504-PA

MQYK

5	VPVWVLCNT GGLEQIPLRQLPAT		LRR1	2.2
28	VENLALTKN NFPIIKPDSFAGLRA		LRR2	3.4
52	LKKLSLDGN NITRIKQFAFRGLPR		LRR3	4
76	LKELSIQYT PLQMVAQFAFAGLQN		LRR4	2.8
100	LSTILLSHN QIQRIEGNAFAGTS		LRR5	3.4
123	NIKLILLTN NPLIRIDSSAFSSLTN		LRR6	3
148	VGHLILPSG IRSIEQDAFFGMDT		LRR7	2.4
171	VGLLKLAYM DLKEVAPFTFRGLSN		LRR8	2.4
195	VLLLTLQES DLGVICADAFTGLTQ		LRR9	2.8
219	VETLQILNN KIDSIEELNFTSTAA		LRR10	2.8
243	IKHLKFFGN HVLETPDPNSIIVDG		LRR11	2.8
267	VEHLQLVSN				LRR12	3.4
276	HFPCGCHIHTLLDGPLAEGAHNLTDFLQKNYCISPLEVNGRVMSELDIDSIGRC		CF1_short	3

SHIFT -63!

267	VEHLQLVSN HFPCGCHIHTLLD			LRR12	3.4
289	GPLAEGAHN LTDFLQKNYCISPLEVNGRVM		LRR13	2.2

SHIFT -13!

306	YCISPLEVN GRVMSELDIDSIGRCSDQ		LRR1	2
333	LTKGNLGSS AASLALGINSMLLIAVASCAE		LRR2	2.8
363	WRHVRLLAN RLAQLLGHVAWRRRRKRRKGN		LRR3	2.8


>CG14351-PA

MRPGRTTTALPAATTPLLLLLPLILGRLHVAHAQCPWQRDVPDLQTSCICAYNLGRELSV
QCDQVDFSQLLAAMNTHAR

80	LKPVDLLYV NNSTISELPDAVFSNLS		LRR1	2.4
106	LHNLQLSSC GIQRIATGAFKGQESV		LRR2	3.4
131	LRNLNLQDN LLADVPVEALKVLGK		LRR3	4
155	LNLLDLSKN QLSHIPDDAFVGLTK		LRR4	4
179	LSTLKLNDN NVTLASNAFRGLEQS		LRR5	4
203	LKNLNLKGT KQRKVPESIRGLKS		LRR6	3.4
226	LAFLDLSQN GIKELPGAGGIRVFDGLDA		LRR7	4
254	LTALNLERN LIQSIGETAFAGVRKT		LRR8	4
279	LSSLSLLNN LLAEFPIGAVHSLKE		LRR9	4
303	LRVLDIGFN LLTSLPEAAFRGNPG		LRR10	3.4
327	ITLLALDGN PLSSVPEGAFAHLNAT		LRR11	3.4
352	LRGLSLGGR				LRR12	3
361	FLHCDCKLRWVAEWIRNGDLQVTSRERNPQFCGTPPRFRDRGFYSIQPEELSCP		CF1_short	3

SHIFT -63!

352	LRGLSLGGR FLHCDCKLRWVAEW		LRR12	3
375	IRNGDLQVT SRERNPQFCGTPPRFRDRGFY		LRR13	2.2

SIQPEELSCPDIADAALRGPVGLADNLKPTLPSSPDSVEYETGTGTGTVGTGTAASAPVT
SSVSSSTSTTTPTTTTTTTTAEPTTRRSTTRPPTKSTTTISATTASPASNPPVNGTGTVQ
ATSITTSSSSSSSSSTSTGHGNGKQAPGWRQGVTNGNGNSGVGGAGGLHKPQRPPLVLGY
PPQRGTRIDDANEVQVKHAFRQDSSVIIQWDSDTANILGFRVVYRLFGEKAFKQGPPLES
SEREFKIKNVPAQECIIVCVISLEELHVTPETVPYQQCREVRTVASQASNMDKITIAASA
AICGTIIVAVIVFIAASRRSRKLQSSQQKSP

736	LPIGGLPVN CCGPTGSPGPLGS			LRR1	3.4
758	IATLSAFNN HKEWDQVSAYSGRSIPRPRIY		LRR2	2.8

PVEQPDDMRSHFSGMPGKVGKSSRSLADGQSQHSFSNNSHRGYLGSAFPSNLVNSRPELR
QSRQSLAAASERMSRASYAGSIHGGNGGGGGLGGNGGGGGNGLPVSSLMAMSGMVGGGGP
ASIASSARRSRPRSRSREQLNTTHIHNHRPGSRYSQAGSTHTLNNYCDTSDNWTDHDMDI
YMARNPTTRNGLGTEGRGRGRRQQQQQQQQQQQQQQQHQQLQHQQQQQHQHQLQQLRNER
HQQRHRNDQQWEQPYQPAQQQHQRLQYYMPQTERCTSPPDVVPRAANFHNSNTTSTGHND
NSNSHGSSSSSSSNIAQQQQAPLAAMTAALGDATAAATAAAAAAASASGIVVENYFNEYE
RQEIIYHHPTSRRSSSSSSSCSAAATRHVPRSNAFRLQLPAVATATTNNILYNISNKHGA
TGHNGSIGNYKAHTFSDSCNCTCNCSLSEEEEISQETAAVTAAAGEDGDQAGSSLSSTSV
SSIEQVQQGQQEQQGQEVYLSLNLTLADAAAGADAAADAADASADRAEL

>CG18095-PA

MYLLPGSLLLLIFSFSPR

19	LSCLQLSKC NNTEVTLIRKTEL			LRR1	3.4
41	LTSLTLSNC TLPHVENGFFVRFDH		LRR2	3.4
65	LLHLELQHS GLSDLDDFSLNGLTK		LRR3	3.4
89	LQYLSLSHN NLSSLRSWSSEPLGA		LRR4	4
113	LTNLDLSHN MLSKLSVKSFEQYPQ		LRR5	4
137	LQQLDLRYN RISQIENDSFDGLSH		LRR6	4
161	LKHLYLNGN QLAHIDGSFFRGLHR		LRR7	4
185	LSSLSLQHN RIEFIEMDSFESNTH		LRR8	4
209	LRSLRLDQN LLSSLQFLSQRGLAR		LRR9	4
233	LVHLNLSSN LLQKLEPFVFSKNFE		LRR10	4
257	LQDLDLSYN NITKLNKEALSGLDS		LRR11	4
281	LERLNISHN YVDKIYDESLDSLIA		LRR12	3.4
305	LLQLDISFN LLTTLPDNLFHFNTQ		LRR13	3.4
329	LEEIILANN KIEEISSQMMFNQNH		LRR14	3.4
353	LRYIKLSGN AISDAAFLDRLSPSVNRF		LRR15	3.4
380	TLYVDLSSN RLKSLNLSSLLH			LRR16	2.4
401	FRYINLADN NWSCNWLVANLVQKLPNSVNF		LRR17	2.8
431	ARPWTVINN LSENTTNVEGIDCIEGGTNRS		LRR18	2.2

IILLDVSGVPQPKSDNCDCVVAYDETNPSPPPLTWPKIPTDRFDSRSVIIWM

513	LVAIAIAFS GLRWLRRFVDRNEKRK		LRR1	2.2
538	LGQKMLHNV VC			LRR2	2


>CG11282-PA

MSRRRAITMSLAPHL

16	GQAFSLCLC LCLCLVLATLPV			LRR1	2.2
22	CLCLCLCLVLATLPVALGLANCPNGCECDDDTLMVNCGEGT		NF	4

SHIFT -26!

37	ALGLANCPN GCECDDDTLMVNCGEGT		LRR2	2.4
63	LDVLPIALN PAIQRLVIKNNKLKTIDSSMQ		LRR3	3.4

SHIFT -19!

74	IQRLVIKNN KLKTIDSSMQFYAQ		LRR1	2.8
97	LTFLDLSFN DMLTIPERSFAYHAK		LRR2	4
121	LQELHLDHN KIGQVSNKTFLGLST		LRR3	4
145	ISVLNLRGN LIAELEYRTFSPMVK		LRR4	3.4
169	LAELNLGQN RISHIDPHALDGLDN		LRR5	4
193	LRVLYLDDN TLTTVPGELTFQALHS		LRR6	4
218	LAELYLGTN SFMTIPGGAFQDLKG		LRR7	4
242	LTRLDLRGA GLHNISGDALKGLVS		LRR8	3
266	LRFLDLSDN RLPAIPTAAFQRLGR		LRR9	4
290	LEQLNIGQN DFEVISSGAFSGLRE		LRR10	3.4
314	LRHLELTGA QRLRRVESGAFSGNTN		LRR11	3
339	LEHLNLSSN KQLNELSSIALGGLPH		LRR12	4
364	LSTVVLKAN QLSSLDEGLVPWAD		LRR13	3.4
387	LQTLDLSEN				LRR14	4
396	PFECDCRLLWLRHLLVSRNASGQYAPVICAYPTALRDLPLAHLAEPLLGCA		CF1_short	3

SHIFT -46!

401	CRLLWLRHL LVSRNASGQYAPVICAYPTA		LRR1	2
430	LRDLPLAHL AEPLLGCAHGAASKQAIIGIL		LRR2	3

SHIFT -27!

433	LPLAHLAEP LLGCAHGAASKQAIIGILV		LRR1	2.4
461	VACAALITT LALVLYTCRHRIREMLKGHSA		LRR2	2.2

LGRKEREYQKTFSDEEYMSRPPPGGGGVHPAAGGYPYIAGNSRMIPVTEL

>CG7121-PA

M

2	LTYLPVVWL FFALLVLRSATGQIIP		LRR1	2.4
27	LPTFCLGLS PQCTCAAEGNVVRFHCPDEYA		LRR2	2.8

MLLEVSEPGASLYMSYYASTE

78	LQWLPRFNI SSLVKIEFDAYIFWPEKF		LRR1	2
105	LSDLLKTLG VQTVKTIIFRDRTLETVVTRD		LRR2	2

VLNSGNGYMETSQPENITTWHFGSVPGLKKFKFFSHVPELQESIFHGFDT

185	LRDLHLSVN VTTLPGNMLSTVNGT		LRR1	4
209	LKTLTIESP GIVSFGNPLLRELQQ		LRR2	2.4
233	LRNLSLALI HPFHERDKQLQPHFFGSMTN		LRR3	3
262	LEEVRLASA TSSVNRSMFKGTNK		LRR4	2.4
285	LQLIKMNGN DDLMELPGEIFLDQVN		LRR5	2.8
310	LKTLDLSCN AIVTLHEDVFKGLGN		LRR6	4
334	LTLLDLSKN RLTNLSSTIFAPLTS		LRR7	4
358	LNVLRLNKN SLTAMSPSVFQDVVS		LRR8	4
382	LNYIEMVNT QFYGATLLMNYEAVVCTNDEA		LRR9	2.2

CQYKSAEWQ

421	CDPRCICWVQRSVGSLIVDCRGTSLEELPDLPRTT		NF	4

SHIFT -11!

445	LEELPDLPR TTLLSTVLKVGNNS		LRR1	2
468	LTSLPTVSE HSGYANVSGLFLSDNNLTSL		LRR2	2
497	GSGDQLPDN LTHLDVRGNQIQSLSDEF		LRR3	2.4
524	LLFLQEPNN TMTLSLSGNPITCGCES		LRR4	3
550	LSLLFFVRT NPQRVRDIADIVCTKQKKSFQ		LRR5	2.8

Q

581	MEAFELCPS YVLLISCVVGGLVIVICL		LRR1	2.2
608	LTVFYLMFQ QELKIWLYNNNLCLWWVSEEE		LRR2	2.4

LDKDKTYDAFISYSHKDEELISKLLPKLESGPHPFRLCLHDRDWLVGDCIPEQIVRTVDD
SKRVIIVLSQHFIDSVWARMEFRIAYQATLQDKRKRIIII

738	LYRELEHMN GIDSELRAYLKLNTYLKWGDP		LRR1	2
768	LFWSKLYYA MPHNRRVLKGQKKHAGPLI		LRR2	2


>CG11136-PA

MLLLLCTLAAANDLKDLLEDSTTSSSTSTTSSTIPSSVTSPTTPTAYVVSAAAVPALVAG
RGKSKSTSGKYVNKSSPRKRKAEQVSSLPLPVDDALMEWKCPNITGTRNAELECGC

117	DLPHTLRCN IDLHGMMLLADRLRTSPYSIS		LRR1	2
147	LLDCSLRNV TFLSDAKIFDNVS			LRR2	2
169	LHGLVISSG EIKRVHKSAFLGIRGP		LRR3	2.4
194	LQALGLPGN ALMSVPWNALSTLSA		LRR4	4
218	LERLDLANN KIKALGTADFVGLTS		LRR5	4
242	LVYLELSNN QISSISQRTFVNLRK		LRR6	4
266	LEVLKLGGN RLGDYAQSLRSLSQCLS		LRR7	4
292	LRQLDLQAN NLNGPLSEQTLPGMRN		LRR8	4
317	LESLNLNRN LIKSIQNKALANFSR		LRR9	4
341	LVSLSLRHN QIDVLQDHAFFGLGA		LRR10	4
365	LDSLDLSYN GIVAISSASLQHLSR		LRR11	4
389	LTVLDLTHN FLRALTSDLIAPLPS		LRR12	4
413	LRELRLAGN DISIVARNAMDGARE		LRR13	4
437	LESLQMQEN				LRR14	3.4
446	PLSCDCSIRPFAEWLQESQLHSSLSASCVTPPRLEGAPLLQVPVETLSCD		CF1_short	3

SHIFT -59!

437	LESLQMQEN PLSCDCSIRPFAEW		LRR14	3.4
460	LQESQLHSS LSASCVTPPRLEGAPLLQVPV		LRR15	2.4

ETLSCDMDNVEKDNANIMQH

510	LETLAKPNQ TSPIKDLSEEII			LRR1	2
531	LHELHFSTD YGLILTWLLNLSKKDYMCDAI		LRR2	2.4

SHIFT -22!

539	DYGLILTWL LNLSKKDYMCDAIFVYKEE		LRR1	2
567	HINEILIDN SPIHCESKVVNGQNTVSVIVP		LRR2	2

DSSSLEIGESYRFCLVMIQEQKPDSELNIGCSNITRLERSSPGAVPVSRQYQRRPYYNAN
ELKPEVVHDAGEDYQVNQRRFNSVVGSQPQQTQQSTLLHSYTVIDSLNKSF

708	LPGLGLGVL VTSVLVLIWGATRIRQTG		LRR1	3
735	GSSGGGGRN GDSIDSNSSSMHNNNNNSRPG		LRR2	2.2

TPTATTCYAASDHIARLADAENGTRYLKLQATTSL

>CG16974-PA

MEAISKISLILCALFVGLKAAAAISGDSTHCLATYSSAEAYLAQIPQQHRPQIRPRIRTW
QEHEFSLLGYKFHLPFVGHAVDSDLDDSDSDEGLWLDAADAGSESVEVEEHELPSVGHVD
PTGNVFKLNCEHVDLRRVNQEL

143	LSQRSSHIN YNQLMLAHVPADR			LRR1	2
165	SNPLKLPQL ESLREFSWQSSE			LRR2	2
186	LKDETLMEL FTRQPRSFEYMERLNLAENR		LRR3	2
215	LECLHWAIP LAVRRVKVLEMSGNR		LRR4	2.4
239	LSNCSLLNL QYMKQLQELHLDRSE		LRR5	2
263	LTYLPQRFL GELSELRMLNLSQNL		LRR6	2
287	LTELPRDIF VGALKLERLYLSGNR		LRR7	2
311	LSVLPFMLF QTAADLQVLDLSD			LRR8	2.4
333	NRLLSFPDN FFARNGQLRQLH			LRR9	2.4
354	LQRNQLKSI GKHSLYSLRELRQLD		LRR10	2
378	LSQNSLSVI DRKAFESLDHLLALNVS		LRR11	2
404	GNNLTLLSS IIFQSLHALRQLDLSRNQFKQ		LRR12	2.8

SHIFT -16!

418	LHALRQLDL SRNQFKQLPSGLFQRQRS		LRR1	2
445	LVLLRIDET PIEQFSNWISRYDESLVDPQV		LRR2	2.8
475	LHRLRYLSV QQNRKLTYLPATLFANTPN		LRR3	2
503	IRELLLAEN GLLQLPTQISGLSR		LRR4	3.4
526	LQRLSVRGN SLGSLPENIKELRQ		LRR5	3.4
549	LHYLNILGN EYQCDCSMYWLTAWLANTSTS		LRR6	3.4

LRHQMPQAQNHSNGSTNQTPLDSYESIDHQIDALKCQYGYRGDM

623	LRVLSKLNC SVPTVVQFSEPKMHKL		LRR1	2.4
648	LSTAKLECQ ISGSPVPDIIWVTPRNKILRH		LRR2	2.4

HADPDKRPIIIDSKEDAHQPPSAQE

703	LAALMDESY IQSLNWTRQNSLV			LRR1	2
725	GRRVVLVEN GSLLVHNISRIDSGLYTCYAF		LRR2	2.8

SHIFT -23!

732	ENGSLLVHN ISRIDSGLYTCYAFNVMGKA		LRR1	2
761	SAGLRLYID PIVFYRVKIGSILFGTA		LRR2	2
787	LATAFLLLT LIVQGLRSCLSRWGICNRFYC		LRR3	2.8

CVNRKKKSPRKHQIYAMLDSIETYKSQQLERLRENYTQQVHRIRENCAQQVEWIQSSYTS
QAKHIREFRDIGSNHLTTLKDQYYDQVKKVRDYSTGQLSWVRENYVFQRNKIRKFSA

934	HQVLRLREG YKYQQQTLNKVLEN		LRR1	2
957	LPSFYFENC RGRCEEDIAEDIDCYFKGQMD		LRR2	2.2

FGDSKELHIQ

997	KIKARLSAN YSASKASLYYTPPDDDLLH		LRR1	2.4
1025	SSQLNLQNS PIHINYIDENLDQQKQLEHDF		LRR2	2.4

SHIFT -25!

1030	LQNSPIHIN YIDENLDQQKQLEHDFK		LRR1	2.4
1056	MEPQLLLYN ASMLYMNPEGASSSGQAAALA		LRR2	2.4

AAGALSQFISVEDNNQEQEMQPLRKISGKPLGMPE

1121	LKDLNDVKT SKSCPAIYKVSKQRDGST		LRR1	2.4
1148	LHELQKEGE APYQMLRINPVETTSLTSTVA		LRR2	2

PVMQARTEKLNIILDECGTASLCKAEQEGDAGNSETPPSESSCESNSLAASCGDVCQVSS
KLANDASLPSTPPKPDHAST

>CG12283-PA

MHIREAVFLVLTLLPGMILGTRYNQLHLYANGGASSSGPGGYRPAPSSQNEVYSIADSQP
MTEDGYMPPSQHFPPTHSDLDPPAQQQST

90	CQTVCACKWKGGKQTVECIDRHLIQIPEHIDPN		NF	4
123	TQVLDMSGN KLQTLSNEQFIRANLLN		LRR1	2.4
149	LQKLYLRNC KIGEIERETFKGLTN		LRR2	3.4
173	LVELDLSHN LLVTVPSLALGHIPS		LRR3	4
197	LRELTLASN HIHKIESQAFGNTPS		LRR4	4
221	LHKLDLSHC DIQTISAQAFGGLQG		LRR5	3.4
245	LTLLRLNGN KLSELLPKTIETLSR		LRR6	4
269	LHGIELHDN				LRR7	3.4
278	PWLCDCRLRDTKLWLMKRNIPYPVAPVCSGGPERIIDRSFADLHVDEFAC		CF1_short	3

RPEMLPISHYVEAAMGENASITCRARAVPAANINWY

364	WNGRLLANN SAFTAYQRIHMLEQVEGGFEK		LRR1	2.4
394	RSKLVLTNA QETDSSEFYCVAENRAGMAEA		LRR2	2

NFTLHVSMRAAGMASLGSGQIVGLSAALVALIVFALGVIMCLLLRVKRQPYVDSKTPNHM
EVITSVNHQNSITNKTQPATGNGSIGGVVIANGAVANIIDGGVVQGGTLERKSSGRGGVP
HGVHDQRSANPVQKPPR

561	LTDLPYSTQ GYDNNGSVLSTASCFISPS		LRR1	2
589	GSTGNGGNN PDLINDTKRFGSDEFADLKIP		LRR2	2.2

PISGVGVGGSGEYSRANGCDSLYPSGLWEHGAPVGTTSADDLFMKRYTDKTPIIDSTQLY
DLHERTAATDYFSKTFPRSHLQQGMMTGGGGGTSTASTVTTNLSGGSSSGYPN

732	DYGLPLVPG AEHQHNHQLQMHP			LRR1	2
754	LQQLQQQLT STLNHQKQEGSSTGSSPHFSS		LRR2	2.4

SHIFT -26!

758	QQQLTSTLN HQKQEGSSTGSSPHFSSRT		LRR1	2
786	LPRLHEGSG GGGSSRSNGEETKPRTD		LRR2	2


>CG31096-PA

MVYGRSIAVGFCLMTVVLLLAAVIFYLSLGPCPAASFACDNGTLCVPRRQMCDSRNDCAD
SSDENPVECGLLYGSKEIADKIVRNAIEKKQQRLISAVSNASGADSTTSMVPRNQSLTLN
MT

123	CDIVTYPKACQCGQGTILYCGRYAKLRRFPRLSSE		NF	4
158	VTNLIIIRN NLTLRDNIFANFTR		LRR1	2.8
181	LQKLTLKYN NISRVPLGSFSGLFH		LRR2	4
205	LERLELSHN NVSHLPHGVFLGLHS		LRR3	4
229	LQWLFLVNN HLHHLPVEQLRFFRR		LRR4	4
253	LEWLVLSRN RLTLRNVQLPKIPT		LRR5	4
276	LYEVYLDFN RIEYIGEETFSQLDN		LRR6	3.4
300	LHLLDLQHN LITHIHGRAFANLTN		LRR7	4
324	MRDIRLVGN PIKELSGETFLHNTR		LRR8	2.8
348	LEALSLALM PIHISSSLMEPLN			LRR9	3
370	ISFLNLTGI RYDHIDFEAINSMRNLTYIIY		LRR10	2.4

DRFFYCSMTPRVRMCKPSTDGVSSFQDLLSKPVLRYSAWV

440	MATLTIAGN VLVLWGRFIYRDENVAVTMV		LRR1	2.8
469	IRNLALADM LMGFYLVTIGVQDYRYRNEYY		LRR2	2.4

KVVLDWITSWQCTLIGTLAVSSSE

523	VSMLILAFM SLERFLLIADPFRGHRSIGNR		LRR1	2.4
553	VMWLALICI WITGVGLAVAPVLLWRTSTLP		LRR2	2.4

YYGSYSGTCFPLHIHEAFPMGWLYSAFVF

612	LGVNLLLLV MIAMLYTALLISIWRTRS		LRR1	2
639	ATPLTLLDC EFAVRFFFIVLTDFLCWVPII		LRR2	2.8
669	VMKIWVFFN YNISDDIYAWLV			LRR3	2.2
690	VFVLPLNSA VNPLLYTFTTPKYRNQIFLRG		LRR4	2.4

WKKITSRKRAEAGNGNVATTTTGTATGSSQHPDDFTIFAKAAMRCH

>CG7250-PA

MIYYMLLILPVVLAQDQQHTTESLSTKHHQQQQLSHSNAIMGEAGVSNSQLMQPSTPART

61	LRPLTAGAG GDPSLYDAPDDCHFMPAAGLD		LRR1	2.4
91	QPEIALTCN LRTVNSEFDTTNFSVIPAEH		LRR2	2.4
120	TIALHILCN DEIMAKSRLEAQSFAHLVR		LRR3	2.4
148	LQQLSIQYC KLGRLGRQVLDGLEQ		LRR4	2.8
172	LRNLTLRTH NILWPALNFEIEADAFSVTRR		LRR5	3
202	LERLDLSSN NIWSLPDNIFCTLSE		LRR6	4
226	LSALNMSEN RLQDVNELGFRDRSKEPTNGS		LRR7	3.4

TESTSTTESAKKSSSSST

274	SCSLDLEYL DVSHNDFVVLPANGFGTLRR		LRR1	2
303	LRVLSVNNN GISMIADKALSGLKN		LRR2	3.4
327	LQILNLSSN KIVALPTELFAEQAKI		LRR3	4
352	IQEVYLQNN SISVLNPQLFSNLDQ		LRR4	2.8
376	LQALDLSMN QITSTWIDKNTFVGLIR		LRR5	4
402	LVLLNLSHN KLTKLEPEIFSDLYT		LRR6	4
426	LQILNLRHN QLENIAADTFAPMNN		LRR7	4
450	LHTLLLSHN KLKYLDAYALNGLYV		LRR8	4
474	LSLLSLDNN ALIGVHPDAFRNCSA		LRR9	4
498	LQDLNLNGN QLKTVPLALRNMRH		LRR10	4
521	LRTVDLGEN MITVMEDSAFKGLGN		LRR11	3.4
545	LYGLRLIGN YLENITMHTFRDLPN		LRR12	4
569	LQILNLARN RIAVVEPGAFEMTSS		LRR13	4
593	IQAVRLDGN ELNDINGLFSNMPS		LRR14	2.8
616	LLWLNISDN RLESFDYGHVPST			LRR15	3.4
638	LQWLDLHKN RLSSLSNRFGLDSELK		LRR16	4
663	LQTLDVSFN QLQRIGPSSIPNS			LRR17	3.4
685	IELLFLNDN LITTVDPDTFMHKTN		LRR18	3.4
709	LTRVDLYAN QITTLDIKSLRILPVWEHRA		LRR19	3.4
738	LPEFYIGGN				LRR20	2.8
747	PFTCDCNIDWLQKINHITSRQYPRIMDLETIYCKLLNNRERAYIPLIEAEPKHFL		CF1_short	3

CTYKTH

808	CFAVCHCCEFDACDCEMTCPTNCTCFHDQTWSTNIVECSG		NF	4

AAYSEMPRRVPMD

861	TSELYIDGN NFVELAGHSFLGRKN		LRR1	2.4
885	LAVLYANNS NVAHIYNTTFSGLKR		LRR2	2.8
909	LLILHLEDN HIISLEGNEFHNLEN		LRR3	4
933	LRELYLQSN KIASIANGSFQMLRK		LRR4	4
957	LEVLRLDGN RLMHFEVWQLSANPY		LRR5	4
981	LVEISLADN				LRR6	3.4
990	QWSCECGYLARFRNYLGQSSEKIIDASRVSCIYNNATSVLREKNGTKCTLR		CF1	4

DGVAHYMHTNEI

1053	EGLLPLLLV ATCAFVAFFGLIFGLFCYRHE		LRR1	2
1083	LKIWAHSTN CLMNFCYKSPRFVDQLDKERP		LRR2	2.4

NDAYFAYSLQDEHFVNQILAQTLENDI

1140	GYRLCLHYR DVNINAYITDALIEAAESAK		LRR1	2.4
1169	QFVLVLSKN FLYNEWSRFEYKSA		LRR2	3
1192	LHELVKRRK RVVFILYGDLPQRDIDMDMRH		LRR3	2

YLRTSTCIEWDDKKF

1237	WQKLRLALP LPNGRGNNNKRVVSGC		LRR1	2.4
1262	LSGRTPSVN MYATSHEYQAGNGGVIPPPSA		LRR2	2

RYADCGSNNYATINECAAAGGGRGYKPIPTSASAAAAACKFNTMNQLSKKQQRDLSVAGM
AKTLEHQHHHNHQANRRSQHEYAVPSYLPSAAPAYDSVDYAKQQIRNNANCECVNLGTAK
RAAGKNPASGLPSSFSSNF

1431	VPPGGASYN CKKSCSCIGDDELLCSC		LRR1	2.2
1457	GGGGGIGVN LLESGTQSSVTMSSSSNNSRQ		LRR2	2.2

SHIFT -26!

1461	GIGVNLLES GTQSSVTMSSSSNNSRQPELT		LRR1	2.2
1491	HYESNLSLN DDEDEDHDQQKNLWA		LRR2	2


>CG5888-PA

M

2	WWLLLLLSL IAPSWQENATVINPSYVTKAG		LRR1	2.4
32	CEALVKEPN LCECQEQEIKCENLQ		LRR2	2
56	LHSDNLQLY GISCSIFDARGFGYIPPLKVG		LRR3	2

SHIFT -28!

58	SDNLQLYGI SCSIFDARGFGYIPPLKVGN		LRR1	2
87	IGSLIVQNC AIPNAESIKYLLSKLGVSNYT		LRR2	2.2

ELEIFNYFDPKKTDRGEVLQQHYTDHESLKKVSII

152	GFKSTLPEN FLENLPALQQLSLKGSSDLP		LRR1	2.4
181	GNILHPLKN LTHLEIVVKNLGKVSGAIFAK		LRR2	2.4

SHIFT -27!

184	LHPLKNLTH LEIVVKNLGKVSGAIFAKQSK		LRR1	2
214	LKHLMIDCD AKNSSVEMSAFGPQELWHMTE		LRR2	2.4
244	LQSIELFNC GDNVPTELFWMSEQ		LRR3	2.8
267	LAYIGIRSN ISYLSKDFLKVQKK		LRR4	2.8
290	LLTLRLERN NIARLPDQLFRNTPL		LRR5	4
314	ILEIHLAFN NLDRIQSGLFDKLKN		LRR6	2.8
338	LQVLNLEHN PITTIALNAFTPIPTAHIYV		LRR7	4
367	GKLFKAAKN ADWARSTNATICEEEYIYGVC		LRR8	2.2

IYCKRDEYLDHFADSENCNKPNPKAKDVLAKKAYENQLKAMPTHSWKKAKEYPVEEEQP

>CG7800-PA

MYLIIIALLASVSALG

17	RPDLEDYCN DSYCHLLGRSRTFSDKKATK		LRR1	2
46	LTEFHMDSC EKKVLKLMPNLRTLELENCDS		LRR2	2.2

SHIFT -20!

56	KKVLKLMPN LRTLELENCDSPDFTMND		LRR1	3
83	LNQLPYLTS LQLRRGNLLGLHDEHFSKWPN		LRR2	2.4
113	MKILMLGGN NITRLSNECFKGLAQ		LRR3	3.4
137	LWLLSLPGN GIQGLPWDVFQNLPE		LRR4	4
161	LLHLDLSGN RIETLHENIFTGVPK		LRR5	4
185	LEMLLLNGN PLTWIAPTSLKS			LRR6	4
206	LSNLRLLDM SNCGPLPDLSLPG			LRR7	3
228	AHTLILDNS GVQRLDILGSVHKLQARKNHI		LRR8	2.8

SHIFT -20!

238	VQRLDILGS VHKLQARKNHITEIKLPDKSS		LRR1	2.2
268	VIELDLHSN LLTATDIPKLLTGMWR		LRR2	3.4
293	LQRLDLSEN IIGIYAAAGSDNTSELFILPN		LRR3	4
323	LMYMNLSAN RLTRLHFDSPIPWER		LRR4	3.4
347	LTHLDASYN RIYAPAKVGIDEAFN		LRR5	3.4
371	LQSLHLEGN YINNFELTPWKPHPS		LRR6	4
395	LKEVALYDN KFQPKGYKNITKFFNEIGVNV		LRR7	3.4

LEKTQYSQSNNTTPTCKPCIPDARDFPTSISADTNQTIDTKVNPLSNDTYQWNV

479	WNVLMLVSL IVSLFVNSFLIVK			LRR1	2.4
501	LIRLRGRNQ FTQSSSVPAIIEMFSNESDDE		LRR2	2.4

IIL

>CG8434-PA

MSWSRRRRRNNNNNNNSSSNNNNAASESRSNDNCNYSTTNDFNKINKLLLITYMLLITAA
TICQSAAQSPSSSNGNGVGGSGSTGGSPLSALLKSGGASYNQQLPQQQLFAMLTRNGGGS
SGPRSSGGNRGYTSARQHFMALSEDYSDADVDEEDMAALHYPLSASHPPAGVQGSSSSSG
ISIQSGSLETNGFVADDGGQYEHAQKALEAAAAAAKASNKYNID

225	CPKDCKCLNVLFDCDKLHLERVPVLPSY		NF	4

SHIFT -21!

232	LNVLFDCDK LHLERVPVLPSY			LRR1	2
253	VQTLHLANN KLNDTTVLEIRNLLN		LRR2	3.4
277	LTKVSLKRN LLEVIPKFIGLSG			LRR3	3.4
299	LKHLVLANN HITSISSESLAALPL		LRR4	4
323	LRTLDLSRN KLHTIELNSFPKSNN		LRR5	4
347	LVHLILSFN EITNVNEHSFATLNN		LRR6	4
371	LTDLELSNN RLSTLPIRVFKNLNQ		LRR7	4
395	LKKLALNFN QLEINWSTFRGLES		LRR8	4
418	MKNLQLKSN KIRALQDGVFYVMHK		LRR9	3.4
442	IETIDLAMN QISSLSRQGLFNLTK		LRR10	2.8
466	LRHLNLSFN AISRIEVDTWEFTQS		LRR11	4
490	LEVLDLSNN AINEFKPQHLDCLHR		LRR12	4
514	LKTLNLAHN RLQYLQENTFDCVKN		LRR13	4
538	LEELNLRRN RLSWIIEDQSAAAPFKGLRK		LRR14	4
567	LRRLDLHGN NLKQISTKAMSGLNN		LRR15	4
591	LEILNLGSN ALASIQVNAFEHMLR		LRR16	4
615	LNKLVFKSL				LRR17	2.4
624	NFICDCDLVWFQQWLKNRFPQQAEHAVCGYPEHLLDRHLKSLSSSELVCV		CF1_short	3

SHIFT -17!

657	LLDRHLKSL SSSELVCVDSPKPRVEQ		LRR1	2
683	EPDDMLAVN AANITLECIASSPTAASLAAA		LRR2	2

DE

715	LKIKWRHDN QHVQERPAVHDGASTET		LRR1	2
741	QIRHDLSTN QTSIYGYLRLTNVTYESAGRY		LRR2	2

QCVVSNAFGTTYAQKFKISIGIHPTFLQVPSNLTLDAGEMARLVCSASGDPTPEIALQKF
GGSEFPAATERRLQVIR

848	EENAFLITN AKPSDSGIYTCTALSAAGEI		LRR1	2.4
877	KVNATLVVN DKPQPSIPLVHQEVVVGRTCV		LRR2	2.4
907	LQCLSETAN ADFELEHPHREWFKENKPIHI		LRR3	3

SPTAPDGDQLVVVKENLNWVVLLVGIITVTVICVVVDCCIIWCTLRYQKKKLRMSLAAER
HSTQLRPRS

1006	LADLGCSYD EANHRRLTVMSTPPSEQRCL		LRR1	2
1035	EQGLTLSYL RQTDLEAQQDHLSSKDSGTGS		LRR2	2

DAAVKRTLDDFEVAMPSHKHHTDDEEEEEVEEVYEENEPPYIKHTYEVNYAEP

1118	EQHLFLQNN NHNYDGGGIGVAGGVNKM		LRR1	3
1145	VPNALLRKC SAGASGSNYSSIQQCTTVDI		LRR2	2.2


>CG7702-PA

MKHKLLLLF

10	LAGAALLLA TEVRSQHEDIPYQPVSNIC		LRR1	2.4
38	QTCLCLSTQ DVDHRTHFNLDCSVRNFEHIL		LRR2	2

ARWPEQFGSQAIASGAASEIVVSYSGNRIKLLQQLP

104	ATNASLTLS CRHCGLQDLQAPLFMDVPN		LRR1	2.2
132	VQALYISWN DITDDALVPDLFRGPFRNTRY		LRR2	2.8

EPIG

166	LRDLDLSHN RIVRLDRRLFEHTPH		LRR1	4
190	LTKLNLAYN KLSSLDEATTASIGSVAT		LRR2	4
217	LQRLDLSHN GLMTLPAQLFSKLTS		LRR3	4
241	LRFLDVSGN EFSTMPASLQLLGKS		LRR4	3.4
265	LVQLNLAGN AFLSLKENSLQGLVS		LRR5	4
289	LKRLNISSM PSLRSLEKGALNLPA		LRR6	2.4
313	LEHLDCSRN SKLERLELADLLSSRN		LRR7	3
338	LSQLDLSWN ALTTLVINATGSSNNSSNSTN		LRR8	4

SHIFT -25!

343	LSWNALTTL VINATGSSNNSSNSTNETWPR		LRR1	2
373	LRRMSISGN				LRR2	2.8
382	PWYCSCELFKALELIGLNHIDREWDGTEARCETPYLLAGSPLSNLTAERICKM		CF1_short	3

SHIFT -62!

373	LRRMSISGN PWYCSCELFKALE			LRR2	2.8
395	LIGLNHIDR EWDGTEARCETPY			LRR3	2
417	LLAGSPLSN LTAERICKMVIPKKYREVDEE		LRR4	2.4

PPRFLRRHYIILTAIIASIVLVIGLVIGFVVVCVRRRLKGSDYGVQPIRYTSVRGSNLSQ
FSQLQPVSVASKFNNVHAGSSSGVTTGAANA

>CG8930-PA

MAARCRWSWRLALCPLLLQLLLQLLLLPPSAMGHDETKENPAPDMQNSQEQEPYVHLQHL
QQQQQQNPQTVQQLSQITVNRTSKSASVTPTGIRENVMLPSADPEKEAQILYEKSLQEYH
GSQLSTASTATDVIAGKRTLHSI

144	CERWLQKHCHCTGSLEVLRLSCRGIGILAVPVNLPNE		NF	4

SHIFT -23!

158	LEVLRLSCR GIGILAVPVNLPNE		LRR1	3
181	VVVLDLGNN NLTKLEANSFFMAPN		LRR2	3.4
205	LEELTLSDN SIINMDPNAFYGLAK		LRR3	4
229	LKRLSLQNC GLKSLPPQSFQGLAQ		LRR4	3.4
253	LTSLQLNGN ALVSLDGDCLGHLQK		LRR5	4
277	LRTLRLEGN LFYRIPTNALAGLRT		LRR6	4
301	LEALNLGSN LLTIINDEDFPRMPN		LRR7	4
325	LIVLLLKRN QIMKISAGALKNLTA		LRR8	4
349	LKVLELDDN LISSLPEGLSKLSQ		LRR9	4
372	LQELSITSN RLRWINDTELPRS			LRR10	3.4
394	MQMLDMRAN PLSTISPGAFRGMSK		LRR11	2.8
418	LRKLILSDV RTLRSFPELEACHA		LRR12	3
441	LEILKLDRA GIQEVPANLCRQTPR		LRR13	3
465	LKSLELKTN SLKRIPNLSSCRD			LRR14	4
487	LRLLDLSSN QIEKIQGKPFNGLKQ		LRR15	4
511	LNDLLLSYN RIKALPQDAFQGIPK		LRR16	4
535	LQLLDLEGN EISYIHKEAFSGFTA		LRR17	4
559	LEDLNLGNN IFPELPESGLRA			LRR18	4
580	LLHLKTFNN PKLREFPPPDTFPR		LRR19	3
603	IQTLILSYA YHCCAFLPLVAMSSQKKTSQ		LRR20	2.4
632	VQEAVLFPS DAEFDMTLWNNSMMNIWPQMH		LRR21	2.2

NLSKQLGASMHDPWETAINFNEEQLQTQTGGQIATSYMEEYFEEHDVSGPATGYGFGTGL
FSGMSTEDFQPGSVQCLPMPGPF

745	LPCADLFDW WTLRCGVWVVFL			LRR1	2.4
766	LSLLGNGTV VFVLLCSRSKMDVPRFLVCNL		LRR2	2

AAADFFMGIY

806	LGILAIVDA ATLGEFRMFAIPWQMSV		LRR1	2.4
832	LCQLSGFLA VLSSELSVYTLAVITLERNYA		LRR2	2.4

SHIFT -23!

839	LAVLSSELS VYTLAVITLERNYAITHAIHL		LRR1	2.4
869	NKRLSLKQA GYIMSVGWVFALIMA		LRR2	2
893	LMPLVGVSD YRKFAVCLPFETTTGPASLTY		LRR3	2.4

SHIFT -17!

906	AVCLPFETT TGPASLTYVISLMFING		LRR1	2.2
932	CAFLTLMGC YLKMYWAIRGSQAWNTNDSRI		LRR2	2.4

AKRMALLVFTDFLCWSPIAFFSITAIFGLQLISLEQAKIFTVFVLPLNSCCNPFLYAIMT
KQFKKDCVTLCKHFEESRVVGGGGPGGRGAVARTKRGDLPPPLLPAAAVAHPPGCRC

1079	LRMLPSEMP NWHKMEQTPSMWQR		LRR1	2
1102	LRTFCCGEN RRRRKQRRQPQQRRQRAYTAA		LRR2	2.4

AANPYQYQFAELRQQRQNRASSISSENFCSSRSSSWRHGPPSSAPVPPGNCSMP

1186	LKMLEPHAH PHGHGRRRHSAWLITRKTSQ		LRR1	2
1215	DSNLSSSRN DSSASATTASTSTFRLSRSSA		LRR2	2

GSSTPLPSIIAHNGKAQLDAVKPRLVRQEAVQEEEDSSPPRLGVRFLPTIPSAADSSVVM
EDGDSANTGVASFLGMPLPGASSGFLIAPTTAATTSPPPVVLQPAKPPPDPNDAPL

>CG12199-PB

MMGNRTERSGRRLGMILLLLGVLVVLMALPPPTAGTTDWMQS

43	CGTCHCQWNSGKKSADCKNKALTKIPQDMSNE		NF	4
75	MQVLDFAHN QIPELRREEFLLAGLPN		LRR1	2.8
101	VHKIFLRNC TIQEVHREAFKGLHI		LRR2	2.2
125	LIELDLSGN RIRELHPGTFAGLEK		LRR3	4
149	LRNVIINNN EIEVLPNHLFVNLSF		LRR4	2.8
173	LSRIEFRNN RLRQVQLHVFAGTMA		LRR5	2.8
197	LSAISLEQN RLSHLHKETFKDLQK		LRR6	3.4
221	LMHLSLQGN				LRR7	4
230	AWNCSCELQDFRDFAISKRLYTPPTDCQEPPQLRGKLWSEVPSENFACR		CF1_short	3

PRILGSVRSFIEANHDNISLPCRIVGSPRPNVTWVYNKRPLQQYDPRVRVLTSVEQMPEQ
PSQVLTSELRIVGVRASDKGAYTCVADNRGGRAEAEFQLLVSGDYAGAVSASDGMGMGAI
GAPTIDPQTNMFLIICLIITTLLLLLLVAVLTLFWYCRRIKTYQKDTTMMSGDGLISSKM
DKTHNGSMLEGSVIMEMQKSLLNEVNPVEKPPRRTDIESVDGGDDVLEIKKTLLDDTVYV
ANHSRDEEAVSVAMSDTTTTPRSRHTYVDDAYANSLPPDLLAFPARVPPTSPSMQSSQSN
IPDQVIYGIRSPPSLTSPVYTHMTPHGIYGTKTMTAPHNGFMTLQHPKSRNLALIATTNS
SRQHQHHHQLQQQQQHHHHHQQQQQQQQQQQHPLATTSPFLPAPVVYSPATGVVMKQGYM
TIPRKPRAPSWAPSTSGAAGHGSIQLSEFQSPTSPNPSETGTATTAELQAEPVYDNLGLR
TTAGGNSTLNLTKIAGSQGGAGQQYSMRDRPLPATPSLTSVSSATNASKIYEPIHELIQQ
QQQLQQQQQQQQQRLGSMDTEPLYGVRQQGITILPGSSISGAGLGHAAYLSPGSGAAVSP
SHASSSGDSPKAAKIPPRPPPKPKKKMSVTTTRSGQGSTSQLFDDEGEDGTEV

>Y39A1A.7

MTSITSAFLLLLLFVAVACRS

22	VPCLDGCTC ELSEHDPVIRCDGVG		LRR1	2.2
46	LNRFPLPHS SPLRGFHFLALTCNDIDTIPA		LRR2	2.8

SHIFT -16!

60	FHFLALTCN DIDTIPAISMIKASFPD		LRR1	3.4
86	LQGIDVQGN TRLNCSDLVHLHKD		LRR2	2.8
109	IPVLSDCDN EKPLQCDNLDKNCDWKCRT		LRR3	2.4
137	LTKLKEMWA QFKDLVNRKAKEWQAEETIEA		LRR4	2

AKSWFSAQFKKFKIAIGELSD

>CG3413-PA

MERVHLTAWLALFLIVVANATPTPARTPTG

31	CPADCTCSLSQHTHKPLYHLKCNSTRGLRLTEKTFQS		NF	4

SHIFT -21!

47	LYHLKCNST RGLRLTEKTFQSTVPVHSID		LRR1	2.4
76	LSHLNLTRL SHLLDKLPELTSAD		LRR2	3
99	LSHNQLKDL GHLGKGLKRLNLKHNQ		LRR3	2
124	LTSDKLRKL PQHLQVLNLQHNN			LRR4	2
146	ITHLPLELT HMHQLHQLELSHNAINCSCQT		LRR5	2.8

LEVRNWLVERIVYMEHPVVCSYPLEFRGRSWLQLKQDEICKKEKYQWFDTEENELMMGDQ
PAAVSAEREDEEELGKDFLPIVGNPAATAKKVRSPQIPLPSDQVEGSGDLSETNMELKLP
EETVAEPEAAESQLVDAAASPSVLEEHIVKDEDEDDEGSGSGGGLLIIPDPSKVKITSED
DIDSDGKPEESDVRPLENPENSENPDTVFSNKIGIYEGDQEEKKPVEEDNIVPVVMTNLD
TGLESDVVTDGPLDSSKESEDILTAKIGKPKDDSSAIYYLLAVIGLIVVGLVLFVAIKRC
KYDSNAAARDAEAQRQTELLDMDKKQLGKPLHKNGHGNGQEHSPLIGEKTKLDEAQIVKK
PYENGEAKDGAGQQPLLNGNGSANGGTKEAPETGEPAAHEYYPITPRYPTPQSPRASKYA
QQQQLAEQNNNEPDGAYLPSSPKSGRYSPVYSPETGRVKIKLTETPKPKTPMLVTRSKSN
AGDIITTPVRPIEPTHQVINGH

>CG17667-PA

MHVYYKFGLLLIFLLSVSISHTNAAPAGSEEANPLEDFNYGDDDYSETATGEESPETAEN
RLMPQSSSTSTTTTFRPIFNIPRRSNHVLEPS

93	CPRNCLCLEDFKFVQCANAHLTHVPLDMPKT		NF	4

SHIFT -29!

95	RNCLCLEDF KFVQCANAHLTHVPLDMPKT		LRR1	2
124	AAIIDLSHN VIAELRPEDFANLSR		LRR2	2.8
148	AVEINLNHN LISSIDKDVFQGSER		LRR3	2.8
172	LKRLRLANN RLTKIDPDTFAAAKE		LRR4	4
196	LTLLDLSNN TITQRLDGSFLNQPDLVEFSC		LRR5	4

SHIFT -11!

215	LNQPDLVEF SCVNCSWTELPEQTFQNMSG		LRR1	2
244	LEVLRLNKN DFKQQINTKAFSPLTK		LRR2	4
269	IIKLKLPEL EQQNIEELCSLLTSIDTISFL		LRR3	2.4

SHIFT -14!

285	LCSLLTSID TISFLNYDISCY			LRR1	2
306	EFVLGTPFN GSLIYPTEPPLKGITNPPIVA		LRR2	2

SITSTAKPVTATPAPPRSANRNRGKMDNSTELVKAGILSSETSTSGVSVEPPAESQTNQV
QISQEAINTLLICIMVLAVIGIVIGLICRQDIGGIKTKCCRTSKPEPKDQVHPTEEIPLN
KLA

>CG10148-PA

MVKIVNQHCDLGLALLLAWTWLTRLVVAAHLVDIPTSSRLAAEREEQQLSRQDVGRLSYQ
SIHRMLRDENEPDSFRGELRYQQKRHKRELELNAP

96	ANKLNLTHR DLRTFNSTGGQWKGDFQV		LRR1	2.4
123	ITAMDLSSN QLESLSLDNFNQ			LRR2	2.8
144	LRQLDLGNN SLEVIPLSLADTNMSLP		LRR3	4
170	FVTLDLSCN KFSQISTSFFAQRLPQ		LRR4	3.4
195	LKNLNLAHN ELLNISRESFYNLLE		LRR5	4
219	LQTLVLSHN NISDIDYETFLALPN		LRR6	4
243	LQYLDLSHN RLSGSAIRALQGIPD		LRR7	4
267	LVSLSIAYN PDVGVAMQEFVASWS		LRR8	3.4
291	LKELDASGT GLCQVPAALAQS			LRR9	2.8
312	VRTLKLSDN WLKVKVIQK			LRR10	3.4


>CG32372-PA

MTSLVYTV

9	FHLASLQCS VRPEISPCTCETGKAWNHVE		LRR1	2.2
38	LSCEKLESF NAVVDSLANKLNADTNIDLKI		LRR2	2

SHIFT -18!

50	VDSLANKLN ADTNIDLKITHSQ			LRR1	2.4
72	LDDLEMRSF TDMNFNLYKLRMQWNS		LRR2	2.4
97	LKSLPEVPF RGLSNVTYLSIGDNDLDEIPK		LRR3	2

SHIFT -16!

111	VTYLSIGDN DLDEIPKHALSHMPS		LRR1	2.8
135	LLTLDIGRC KIRAVQQEDFRGIQR		LRR2	2.8
159	VTNLILVSN IITRLDRGSFPKS			LRR3	3.4
181	LLILHLGRN QLESLNGSLHDLHN		LRR4	4
204	LESLFINAN NITSLDDELPDGGQ		LRR5	3.4
227	LRLLMAHNN RLERLPANMAGMHS		LRR6	3.4
250	LETVHIHCN QLRSFDRVLRNAVN		LRR7	2.8
273	LSEVMADNN ELEYLAQDEFASCSK		LRR8	2.8
297	VETLQMGCN HIKSLNSSLLPILK		LRR9	2.8
320	LKNANFSFN DIEEFSMAELHGLRS		LRR10	2.8
344	LKTLQLSSN RIQRLLPDPRGVQELM		LRR11	4
369	LVNLDLDNN RIDSLNGALAGLGN		LRR12	4
392	LRILNLAGN RLEHLQVGDFDGMIR		LRR13	4
416	LDILDLTGN QLAELKPLEMTLLPS		LRR14	4
440	LKILKVAYN NITKLEQDFKGLPV		LRR15	3.4
463	LCQANLTNN QISTISSELVTNTRCKNHNVP		LRR16	3.4

SHIFT -13!

480	LVTNTRCKN HNVPGKLEIHLDDNPIMCDVG		LRR1	2
510	LNELCRLMA VQEARIRGRSQCFENDQEV		LRR2	2
538	CTVLPMLYN VNLPIMVTNLKLTGREVPKPM		LRR3	2.4

SHIFT -24!

544	LYNVNLPIM VTNLKLTGREVPKPMVRVI		LRR1	2.4
572	VPSLIKANN ELLPPLIATLGNPV		LRR2	2.4
595	LISTDLVNP VISPLPPPLLLATTTP		LRR3	2
620	PPPLPLPVE SEVEKNESTTANPVPTNYTQE		LRR4	2

TTTTTSTTTTTTTTETSSQVAPIEIITTTAPTTTTTSTTTPKPNETLPVIVELQDPNPPD
TTVNQTALVPPVVLDPLQQDLERERERERERELERDLDHEAVAKTEYETVEYIPNLVQPP
VGSDALTPPPSKANALEEDSESVHSNSVHAEYPHAAQSLQIPEEPPEE

>CG8561-PA

MLIIEETPLATIEDYVFYGVNNTLEQLHLLRTNLSHVGL

40	LGFGILGKA KELVIDGHAFQQLPKD		LRR1	2.4
65	LFAGQEIAN SLGIIRVTNGNLSDLPIETFQ		LRR2	2.4

SHIFT -10!

85	LSDLPIETF QPLRKLKTLDLHG			LRR1	2.4
107	NQLENLKRN QFKNLRELEVLDISHNQIKKL		LRR2	2

SHIFT -17!

120	LRELEVLDI SHNQIKKLEAQHIAD		LRR1	2.4
144	LTKLGWCNV SHNALSELSRGTFARNSV		LRR2	2.4
171	LKVLHLSHN QIARLDANSFRGMRF		LRR3	4
195	LRRLFLSDN VLTDIGRGTFGSIAR		LRR4	4
219	IGTIDLARN RLKKIEFQMFTQMNY		LRR5	2.8
243	VELLDLAEN NITKIEKNSFKDIYQAIINVS		LRR6	3.4
273	HNALELIET AAFENCVNITVLD			LRR7	2.4
295	LSHNRLANF SRRSFDETTFATYFQ		LRR8	2
319	LSYNNLTNL AQIPIQNMTGLKVLNASYNSI		LRR9	2

SHIFT -11!

338	LKVLNASYN SITEIPKNCFPKLYE		LRR1	3.4
362	LHTIDVSHN NISSIFNGVFQTLFS		LRR2	2.8
386	LRSIDLSHN SMREIKSSTFGTLPT		LRR3	3.4
410	LLEMDLSHN ELVSVVRGSLAKLTS		LRR4	3.4
434	LRQLYLNNN QLEKLFQLPISLNELYFSHNR		LRR5	4

SHIFT -10!

454	LNELYFSHN RLTNIPSGTWPVMNS		LRR1	3.4
478	LIYLDLSHN QLGDTLNGESFTGLLV		LRR2	4
503	VQRLKLQNN GISQPPKDAVAVMST		LRR3	3.4
527	LQYLHLENN NITTLERSAFGKLPV		LRR4	4
551	LFELNLYGN QVKDISKRAFEGLLQ		LRR5	4
575	LLTLNLSSN GIQTLQNDIFVGLPS		LRR6	4
599	LRNLDLSFN SLTKLDNKTNGVLDDLLS		LRR7	4
626	LETLDLSHN RISFVTKKTFPSHQYIPYN		LRR8	4
654	LRNLNLSYN LMPILTYDITFGTKKLSE		LRR9	4
681	EHIFDLPQN LSWLDLSHNKIYH			LRR10	2.4
703	LPFANLVKV KSLKYVDLTNNSLEDVPASIV		LRR11	2.4

SHIFT -19!

714	LKYVDLTNN SLEDVPASIVGSMRN		LRR1	3.4
738	GSQVLLAGN				LRR2	2.8
747	PLHCGCNARPLKYFMLQQTIAGEDLKSIYCGTPALIKDKQLISLDDEYLHCA		CF1_short	3

SHIFT -42!

757	LKYFMLQQT IAGEDLKSIYCGTPALIKDKQ		LRR1	2.8
787	LISLDDEYL HCAEDEQEMYKGIDYEQLTDV		LRR2	2

RFREVKTNK

826	AGMLTLCWY VSGFSDVSDFHVYIRSSSNDV		LRR1	2.4
856	LHQSDYAYN NRTAQIPVEELST			LRR2	2
878	LGEEKLRGA EICIVSRDSDGNTRKWFSGQC		LRR3	2

QQ

910	LPSLKRPRT FFFGNFQVRGGVSRTSSS		LRR1	2.4
937	LPRCLLYII LVLFVARL			LRR2	2


>CG6959-PA

MILVVAIAAIILAIGAGGVLGENSSS

27	CGPNFPAACSCGQEMYESEMQYVVNCTNAGLVNTSVLEFMP		NF	4

EQ

70	VEVLIFTGN RITELPWNVFGSINNYKQ		LRR1	2.8
97	LRIVDMSSN HIREIRGKSYHHVPR		LRR2	2.8
121	VERLILNHN NLSISRYEDEVNHHHPRVFSN		LRR3	3.4

SHIFT -25!

126	LNHNNLSIS RYEDEVNHHHPRVFSNFIN		LRR1	2.4
154	LQSLHLTDA FEDNSSPQLSEDLHDIFVNSQ		LRR2	3
184	LVKLQKLHL EQNEITHFKDRNVFCDLPS		LRR3	2
212	LRDLHLGDN DLRDLNFEVRCLNN		LRR4	4
235	LRFLDLERN KFSFVKPTDLRV			LRR5	4
256	LNELEERPN RTANLIVDFNLNPFGCDCRTA		LRR6	3

PFRAWITTTRVTVRNKDSLMCFHSTGHVDAEPAHLLQMDMGQCADAIAAAATILNTAEDE
PHYGDPEASHLQPQAHISGHTATLIFLLIVLTMILLGLLVALVYVSRDKLKYMITPVFDN
VAKKVQYTSIKDEDCPEVHV

>CG4192-PA

MAAGRAAATLEAPGPPSGQDIASDNSAQRRTLATKVRRKGPRPQRRLHPPLRPRLPLHL

60	HLLLWLLCC CSQLGQLRAE		LRR1	2.4
79	CPAVCECKWKSGKESVLCLNANLTHIPQPLDAG		NF	4

SHIFT -22!

90	GKESVLCLN ANLTHIPQPLDAG			LRR2	2.4
112	TQLLDLSGN EIQLIPDDSFATAQLLN		LRR3	3
138	LQKVYLARC HLRLIERHAFRKLIN		LRR4	2.8
162	LVELDLSQN LLSAIPSLALYHVSE		LRR5	4
186	LRELRLSGN PILRVPDDAFGHVPQ		LRR6	4
210	LVKLELSDC RLSHIAVRAFAGLESS		LRR7	3.4
235	LEWLKLDGN RLSEVRSGTITSLAS		LRR8	4
259	LHGLELARN				LRR9	4
268	TWNCSCSLRPLRAWMLQQNIPSGIPPTCESPPRLSGRAWDKLDVDDFACV		CF1_short	3

PQIVATDTTAHGVEGRNITMSCYVEGVPQPAVKWLLKNRLIANLSAGGDGDSDSEPRTAA
ATQGRKTYVVNMLRNASNLTILTADMQDAGIYTCAAENKAGKVEASVTLAVSRRPPEAPW
GVRIILLGA

447	VAALLLVGG SSFAAICLCSLQR			LRR1	2.4
469	RRKLRLWNS VPPVRRSESYEKIEMTARTRP		LRR2	2.4

DLGGGASCGGGSATGAGLFHDAEEQGYLRAAHTPLNDNDAGQAAAIVNPSAGSAQRRNGD
YLHVSTHCDDEEEDQQLHHHPQQQPASQHHPHPNQQQHQQRKGSQGH

606	VVSASGANN SAPLEETDLHIPR			LRR1	2.2
628	LIDIGGTDS ASSSISSQVDAAARLAGYAGH		LRR2	2.2

TWKTTPIATTKINSPHSKPVTSAAPSSLNTQATPYAHYGNHPADEMATSVFCSEGQESDL
FDSNYPDLLDIAKYAVAQAQQEGRGQGYAQATTTPNGGLCTLPRKLKTSGKYFRNSSDSQ
SPLLADNSSKYGSSTLGDGSFLNEAMGLGRRYSAESSYANYSSTATYTGGGQRANSFLNL
VQSGAHQGK

847	LLPSHLGQK PSLPSSPVQHQRSLSSAATP		LRR1	2
876	LLDFSALAS RAAGAANTSVAAYDYHAAQL		LRR2	2.2
905	ERFLEEYRN LQDQLCKMKETCDTIRKKETP		LRR3	2

LRVAIGQSAAQLADPVMYSAASHSPKPPATSN

967	LKTKTLLPG QPPDPPPYWLHRNAM		LRR1	2
991	LKRLNGDGS AGTNGSGGSPASPQPRQDIFK		LRR2	2.8

S

>CG3095-PA

MLAYTHGTWLLLLLLLVAGACARPEIAHTPDPAALGEESTPHAHAHPQARHHHHAHPHAP
LKEDEQGTAIPAPILTTDNVGIGETTTASSLAETQSMSDPGSVTDTTSTSTSHSTSTTST
TSPAPLPPAAPEQPEYLKHCFYAEEQLCGHTFDGRAETSEGQGSTVAQSEAQNRGGQGNS
QCQCREHPTRPNSWYCCNISQLTMISSCSNISKWTNLHVRNMTVEDMDLSNPIFRS

237	LQSLAVTDG NITRLVNAFPRLSA		LRR1	2.4
260	LKCLNISNN NISEIHSRAVKDVPH		LRR2	3.4
284	LEFFGMSNN NLSLVPHRNQNK			LRR3	2.8
305	NITLDISGN MRMLCTPLNEIIYTESINFLN		LRR4	2.4

PKHSYCQYNATHTWFQSTDKVSVEQLENRKRCVTN

370	CPVIPNYGSCNCTLENIMIIQDNQSKPQCHVDCSNLGLVELPQR		NF	4
414	LPDNTFMLN ITNNKITSLGDYFHTNPTYHN		LRR1	2.4
444	INRLLADNN QISSIYEFEGTKFIET		LRR2	2.8
469	FQRIYMRNN SLSKIPEYFLNNALMDSGL		LRR3	2.2
497	GRRIYLAGN				LRR4	2.8
506	KLQCDCNSAKTLQNWLKERSSDIPDYMEIRCRNMPQRVIELQEAKLCQSP		CF1	4

SHIFT -10!

546	LQEAKLCQS PPDWTDYIYYLIAAEV		LRR1	2.8
571	LLLLALITK VSYDYWVFKTAGYLPWPASKM		LRR2	3

SHIFT -28!

573	LLALITKVS YDYWVFKTAGYLPWPASKMPK		LRR1	2.4
603	LPCDWLCES			LRR2	2.4


>AAV36870

MPDHTRVTATTRSRPWLCGA

21	IPVLLLLLL TLVILPPETTAF		LRR1	2.4
42	CPSKCQCLGGEANSRALCVDAALEDVPIQLNPE		NF	4

SHIFT -29!

46	CQCLGGEAN SRALCVDAALEDVPIQLNPE		LRR2	2.4
75	TKYINLTVN RIRTLEFSLPFYMK		LRR3	2.4
98	LEILDLSQN IIETLGSKNFEYQSE		LRR4	4
122	LRTLNLSRN LVSSLHKHAFKGLTN		LRR5	4
146	LLLLDLSFN RIETVHPTALSDLAS		LRR6	4
170	LVELDLTNN NIVSLEDNCFKGMNT		LRR7	4
194	LEVLVFRNN RLLDVPASNLWHLHA		LRR8	3.4
218	LKSLDMSLN LVEFVRNDSFEGLKE		LRR9	3.4
242	LLALSVQGN VMSELDLSAFEGLIS		LRR10	3.4
266	LKHLDLSDN NLTMVPTQQLSKLSN		LRR11	4
290	LTYLNLGGN RFSQLPAVAFLNLFH		LRR12	4
314	LRELHLSRL DFLQRIDSRAFVDNTH		LRR13	3
339	LQTLHLNNN PQLSDIPMRLFQGNPN		LRR14	4
364	ILEVYMQSN SLQTLYSAQFPVDQ		LRR15	2.2
387	LQKLYLGDN PLQCNCSLLWLWRLVTGNFEG		LRR16	4

VDPGMEHAAGGAVAALAKEADDEELADEATAVASTDDGVAALAAYIAEQHIVNALHTTEP
SAYELATSSSNRNSGILRMDRQQIGCDIWRDKVRTRRKLLTMSEGEITCPAHIVTVVCAV
ITCLLVAMIGISVLYYLRFVKRRRKLLHERGPMRTSKSIINVHDRILQGHNPGGLGLGMS
MTLGGGNH

605	VNGLGMTLN YPHHAQTLQAHHHYHQAMP		LRR1	2.8
633	LQSHGGNGN HEYQQTTLPQLDKLELERYLA		LRR2	2.4

SHIFT -14!

649	LPQLDKLEL ERYLAAQTIANEYRA		LRR1	2
673	LKPWELPVK EADDEPEHLYERFDHYEYPDT		LRR2	2.4

HTMSKLKQAASLNHSNSSAGPSPVPPSSGKPHVVYV

>CG5820-PA

MSRQS

6	TCRLVLLLA FTLALIHASHSNTVGPKIH		LRR1	2
34	FIHDDLTEN ELNSGEEVSPTESLPKISTPK		LRR2	2.4
64	VKDLPVETT TRSPTKKTTSSGTEKSLSEEA		LRR3	2.2

WNDLMEAAKHSQALADDPHFNKMLNTTVAAGEKNSTDTDDSEYYDYDDDYNYFDEETVTI
SPSPKKAKSNPKLQPIVDATITKSKSKSNDQKTKESANDIDSDSQYDYDEGLDDDDDEDD
AADDLLTDDEQVVFSEDVP

233	CPRFCQCARNVNSYLVATCSRLDMGIQKFGSDIT		NF	4

DLVVTNVGPKYPI

280	LMGPNFFQN LGLKNVASIKIANCT		LRR1	2.4
304	LEYLHAEAF HGLNELYAVNLTDVGLAIINP		LRR2	2.4

SHIFT -19!

315	LNELYAVNL TDVGLAIINPDTFVGNKK		LRR1	2.4
342	LRMLTISGN DLSVMSSIHYLLKSSS		LRR2	3.4
367	IEELDFSRN NLMELNPKAFSHLSN		LRR3	2.8
391	VVYINLSQN SLKKLPEKAFEKVTL		LRR4	2.8
415	LEELDLSYN SLTELPRDIFNGTT		LRR5	4
438	LSILHLKYN TFNGDLHFGTKD			LRR6	4
459	LQQLDLSFN SIVQVHHSMFDKMPG		LRR7	4
483	LTNLNLKGN GIKKIQPDSFLTLKN		LRR8	4
507	LRHIDLSIN DLDQISGMLFFKNSE		LRR9	3.4
531	LDVIRLNDN PRLSQLPTDGFLSYSGEFT		LRR10	3.4
559	VYYLDISNC AIGPLGHKAFSTMPH		LRR11	2.2
583	LTTLKLAWN NINHLPREIFTGLHK		LRR12	4
607	LIDLDLSNN LITRMDDLIFMDNGE		LRR13	4
631	LTKLSLAGN PISRLSVRLFLPLHQ		LRR14	4
655	LRCLDVNDC ELTTLLSDRDLGAGYKIFDS		LRR15	2.8
684	LRSFNASGN LIKKISSEDVKSFKN		LRR16	2.8
708	LRSLDITNN PLKCTPDFQEFISYVTLQMQM		LRR17	3.4

TPKRLPVLANLEDDATIVQLETLAQAGWSSLAHEVCKHAEGSDLLDEKKADSAEAKLEKR

798	LKESVKKLN EDEAKLLSVLDKSV		LRR1	2
821	LDKSRLSSM QKIMKGSVNTETAKPEEDADE		LRR2	2

ENLNNGGEDKEDDSSDYDSEEDDDDDDEDDDDDNNDDDRFEEAKKNAKAKADANKAFDTF
VNKELKPVLKTDGIQAEEVDMSQETRDKFLLEKLGFDSESEESDEYTEKMIFHGELSYDL
VVPLIVVS

979	FCLLMIVLS IARVIYVVLRKRGERY		LRR1	2.2
1004	RMALLASKN SFVYQKLSEDIVKPTSKEQKE		LRR2	2.4

PKHLKQPKVHRYAPINQV

>CG15744-PA

MPTATATSTAAEGGQAVQVQTHQDTELPQNPETGIATGQGTASS

45	CPRKCSCRSTAENIHSLKIRCDEQQITNWRELDFGE		NF	4

DVTS

85	IVSINASKN SIALITAEDFRNFTE		LRR1	2.2
109	LKRLDLSFN LLTELDKDTFGDSLAH		LRR2	4
134	LEKLKLAGN AISHIYEGTFDQMPK		LRR3	4
158	LKLLDLSGN				LRR4	4
167	PLACDCGLIWLIAWSSSREVRLQPPPKCESPGNFRGMPLKKLRVGKDFHC		CF1_short	3

ETLLQPLLELIPSQNQVAFEGDELQLKCHAPRVAIGVPRESEDLPTKAYVFWGWSEKIRA
KNSTEDIIYQDPTKVFGDVNLETRHSTDSGILQSILRIASLTQNHTGMWDCTLRSQQANL
SQAIVLHVVAKGTLYCEARVVHTNKGTYHWPRTMRGETVLQECVEEPSDATQARRASHEC
GPS

400	GEWLNLDTE SCVYVSETTRILEQ		LRR1	2.4
423	FAKVNLTLT KGQNALEIARRLHNFTQAQTQ		LRR2	2.2

LNRIRDPMDLEYIARTLVKY

473	LDQLEQPQQ QQEISHLLMDIVSQ		LRR1	2
496	LLNLPAHLF RAAQSEQGTGQRLLHVVESS		LRR2	2.4
525	AMRLALAST QAEPLPAEMIPW			LRR3	2.8
546	RGSLAQQRN LFVEFFNISLDAFVS		LRR4	2
570	LSCVWLEQS PRGFQCNSANDTIPMYEHGDI		LRR5	2.8

DAAIQLPYSVIGNSSTTLPATTT

623	IRSLRLMIS LHRNGKLLPNLRGSHNESLSS		LRR1	2.8
653	AIIGILAYS SDGEALQFRADNELDPEEDVY		LRR2	2.2

QQRVTVMLRAHPYHNPLSAPQPAWWDADEQRWETSVCQQHYQHRTLVMFSCSRTGYYG

741	LLQRSQYLN DFRSEESGARFRHPPAAVY		LRR1	2
769	AGCGLLFAC CAFNAVTFAVFGRAVRINRVQ		LRR2	2.2

SHIFT -26!

773	LLFACCAFN AVTFAVFGRAVRINRVQRHA		LRR1	2.4
802	LVNTWLALG ALALAFSLGIYQTASQPQCR		LRR2	2
831	LLGLLMHYL GLCVLLWVCVSLSSMYKRLTK		LRR3	2.4

TTTSGQGQCPGQDMEPQRERERKPILGIYLVGWGIALLICGISSAVNLAEYATYDYCFLH
SSTT

925	LNALLVPAV ILVIFCGILALCIYYQLSQQA		LRR1	2.4
955	VNVLQLQMQ HQQNRQYSDNNTQATEHID		LRR2	2.4
983	LDWLDANGS ATTAAIGGGSGNHGGRKEQDH		LRR3	2.8

MQEQYSTLSNPLSSIVDDFERSN

1036	LSHLRGHFI FLVLYAGAWLSAAAYVNGGQE		LRR1	2.4
1066	LYVLSFAGC CSVLGIFLLIFYNLSRNDARQ		LRR2	2.8

SHIFT -18!

1078	LGIFLLIFY NLSRNDARQAWSQGRDGRSI		LRR1	2.4
1107	PAKLVTYNN GSQARGAHPSSMMPGPGTAMI		LRR2	2

SNSIVAYKANPGPGSLYEANNSAGSRSNSQCSRSMRSQTRSQTRSQQEQLLQANGAGGVT
IINNTSGQPGAGGGPAAGGVSGSTGGAPVPPHSLNALLHGSSHDL

1242	IPSAEIFYN PNQINVARKFFKK			LRR1	2.2
1264	QKRLAKRNN FELQRQTMPQMQLQMQMQMQL		LRR2	2

HSQHSLSDASSEQLYSRHHNAMTMLAGGSKINNTNLHYKNQGSPMAGAPKEHGNMGAMSS
GGDSMQFKRFVPASASSASKIMQANIYTNIPETLTPQ

1391	HEVIKLRAN GRTRTPSLLDET			LRR1	2.4
1412	LHELDDDEE DEEEEEHGQTPATMEEPEHEL		LRR2	2

EEEDASSLDEHAPLYANTLPPA

1464	SGMSSLFRN RGSSHQPMSSTPVKQPSLEA		LRR1	2
1493	MNSLGLPEV SLEEPLLQKHEIYVSNSLQVT		LRR2	2.4

TSNSIQLDDDFPSVLIRFSQQQQSKSLNNISEMLAGGGGGGGNAPGLDSLGDQ

1576	QESSQLSVN EGSTLEEQQLRQIYSCSSSN		LRR1	2
1605	LSQLKGHHP TATVDTEDDGRLLSGSPTNES		LRR2	2.4

DLNYQNSEISIRSHGLYAPQADNDLNLTLTDDFRCYQSSNASDADVDVLNEFDDEFVAAT
GGERVVGDAEQDPHHDHDQDTSIDELYEAIKCRSPLRNKQEAVERFERERERDREKEMEM
EAKPLSNSHNENLNETIEDDSSQSSVISYIDPRAANEPRPFPS

>CG9431-PA

M

2	AIKLSFDPC SISLKHLSLFLFK			LRR1	2.2
24	IYCLALIFR SASADWLLDCGNCHCKWNSGK		LRR2	2.4

SHIFT -18!

36	ADWLLDCGN CHCKWNSGKKTAD			LRR1	2.4
42	CGNCHCKWNSGKKTADCRNLSLSGVPEYLSPE		NF	4

SHIFT -16!

58	CRNLSLSGV PEYLSPEVQVLDLSH		LRR2	2
82	NHIFYLEEN AFLTTHLQNLQKLLIRNGT		LRR3	2.4
110	LKYLNQRSF TQLQILIELDLSN			LRR4	2
132	NLLVDLLPN VFDCLSKVRAIF			LRR5	2.4
153	LNGNLLQAL RHGVFRNLKYLHKIE		LRR6	2
177	LKRNRLVSI DAKAFVGVPLLSQIYLDNNE		LRR7	2
206	LTKLRVESF QDLTKLTALSLVENPWNCTCD		LRR8	2.4

SHIFT -19!

217	LTKLTALSL VENPWNCTCDLQM			LRR1	2.4
239	FRDFVIGMN LYTPPTSCHYPLQLRGRLWIE		LRR2	2.2

SHIFT -13!

256	HYPLQLRGR LWIEDQPEAFACKPKIV		LRR1	2
282	YPTLSTSIN TSKENVTLICRVHGSPNTVIA		LRR2	2

WDYTNQVYESRSKPVKSLQKQRIYIELLREDESKIRKFGHDVFVSRLTIVNARKSDE

369	GVYTCLAEN PGGKDSVHISVVVQKD		LRR1	2.4
394	MERISLIDS NFFAIVCLIAMGF			LRR2	2.2
416	LSMSILFSL VTCLIFKRFKQFHPGQHTY		LRR3	2
444	LQPTSLPVQ SPGSEEATAISA			LRR4	2
465	LSSGVIRES KIVLDPLSAINEPSNK		LRR5	2.2
490	NYTLFKTSN SNGSEYMHTRNYKDVR		LRR6	2
515	LNSNTYTEN LDNQAESISSRNRELYSNIAG		LRR7	2

DREKEELKQKDELDKDSRQSSLQSTGCSRKKGQIDELQPDLLPSTQPTALKNINETFGPS
AKKAEVNPRSKYNTNVQKYLKEKYGSVRIKNISTKEPITGVDISI

>CG14762-PA

MAGIQLQSGCTMVVTVVLLLQVLVMDQVLGQGPPQTQV

39	CPEQSEIAPCICTVKKNGLDILCETTDLAHITKSMGTL		NF	4

SHIFT -20!

57	LDILCETTD LAHITKSMGTLKGKSPI		LRR1	2
83	IFYLKLRHN NLPKLQGFVFLALD		LRR2	3.4
106	IRHLTIHNS SLAAIEENALSSLGAG		LRR3	2.2
131	LTQLDVSLN QMKTVPSQALQHLFH		LRR4	3.4
155	LLILNLNHN KITVIHNNAFEGLET		LRR5	4
179	LEILTLYEN KITQIDPEAFRGLEDH		LRR6	4
204	IKRLNLGGN DLTNIPQKALSILST		LRR7	3.4
228	LKKLEIQEN KIRTISEGDFEGLQS		LRR8	3.4
252	LDSLILAHN MITTVPANVFSHLTL		LRR9	4
276	LNSLELEGN KISVIDKDAFKGLEEN		LRR10	4
301	LQYLRLGDN QIHTIPSEALRPLHR		LRR11	4
325	LRHLDLRNN NINVLAEDAFTGFGDS		LRR12	4
350	LTFLNLQKN DIKVLPSLLFENLNS		LRR13	4
374	LETLNLQNN KLQRIPQDIMEPVIDT		LRR14	4
399	LRIIDITDN				LRR15	2.8
408	PLNCSCELTWFPKLLEDLKNKDDEMSQKKKPLCHMSLDNREYFVQAMPTEKMHCA		CF1_short	3

SHIFT -64!

399	LRIIDITDN PLNCSCELTWFPKL		LRR15	2.8
422	LEDLKNKDD EMSQKKKPLCHMSLDNREYFV		LRR16	2

SHIFT -13!

439	LCHMSLDNR EYFVQAMPTEKMH			LRR1	2.4
461	CAGLNIIGN P			LRR2	2.4


>CG4168-PA

MHLYLVLISCLVSSSHCWQFDGGGGHRRPRNSGRGPPSSSGGRGSAALLGSQQQDILYAC
PLNSMCQCAGLPNETSTLIEINCNEVALYKFPAWSPHRTDFLPGMSPCGREARKNSQNYA
PRNFKRIKMGACDGGRGFDNCNWGHKLEIHLISQNFQQCPDPVPLARPENEHRIESVASS
PAGNWFGLRAMDDVPWTWSSLVHRFEFMHSSVRYIEMSNTHLQSVDDET

230	FQGLRLKTL KLIDNELQDISERSFSTMTHS		LRR1	2.4
260	LMTLDISGN KMQHLPLDALQRLHS		LRR2	3.4
284	LSRLVAQRN HITTLDGNWDAQHDT		LRR3	3.4
308	LRSLHLSDN DITEVAPGGGSIEVVSQNDNS		LRR4	4

SQPSSLAAVQTRLETSNSIYPPSPSSGDRTTGGRPFEQ

376	LQKLLWLDL SNNRIYHVAGNYLPRS		LRR1	2.4
401	LVTMDLSSN LLTVFPQQLFEQLPE		LRR2	3.4
425	LRIVSLRDN LIRSVQWKELQVRPLRMH		LRR3	3.4
452	LERLDLGQN CIENLESDYFQQNYSDVH		LRR4	4
479	LRALNLEQN FVTQLPEAVFKATG		LRR5	4
502	IAHLVLAFN AISRVHPSAFEGLTET		LRR6	3.4
527	LEYLDLERN RLTTVPVALSSLHH		LRR7	4
550	LKYLYLTSN QISQLNNLPSFTEN		LRR8	4
573	LRVLSLSGN NFSMIPVLGLKNYTQ		LRR9	4
597	LSYLNMGYN SITDIPEGIFAVDSWGSN		LRR10	3.4
624	LQTILLRNN KITHLHLGSFAGLEQ		LRR11	3.4
648	IQEISLSFN DITIHHPLVFENVSRT		LRR12	2.8
673	LKILELSFA VFPARSLESLDPLDALLPLSQ		LRR13	3
703	LIWLGLDNN NLKQVSNESFAQMRE		LRR14	4
727	LSYINLSFN QLKTLPRGLFQSDAHSH		LRR15	3.4
753	LVEIDLSYN GLERLEAQTFHSLGD		LRR16	3.4
777	LQTLNLQSN RLRTIARHAFHNLEF		LRR17	4
801	LRYLDLSYN RLVNISHGAFTVLPN		LRR18	4
825	LAALDLMHN QLCSLSLKSFLYVSNT		LRR19	4
850	TTPLRLNVS HNHIASFYDELSSYMY		LRR20	2.4
875	IYQLDISHN HVTKSDSFTNLANT		LRR21	2.8
898	LRFLNLAHN QLGSLQSHAFGDLEF		LRR22	4
922	LEILNVAHN NLTSLRRRSFQGLNS		LRR23	3.4
946	LQELDLSHN QLDQLQVEQFSNLRK		LRR24	4
970	LRILRINSN RLRALPREVFMNTR		LRR25	3.4
993	LEFLDIAEN QLSVWPVPAFTDIGFT		LRR26	3.4
1018	LRSIQMSHN NLEYLDASMFINSQF		LRR27	2.8
1042	LYDISLARN RITILPDNTFSFLNN		LRR28	3.4
1066	LTNLDLSQN PLVTTNLREVFVHTPR		LRR29	4
1091	LRKLSLHHM GLYVLPPLKLPL			LRR30	3
1112	LSYLDVSGN YLQELSPLGSLRH			LRR31	3.4
1134	LRHVNVSHN KLTNASCAAEHLPPS		LRR32	2.8
1158	VRVLDLAHN PLRRITLHDLASLRHLAE		LRR33	3.4
1185	LNILDVKVT NPQAFSKLRSLRKLHASSHAN		LRR34	2.8

SHIFT -14!

1201	LRSLRKLHA SSHANLGEIVARIPGLQQ		LRR1	2
1228	LRVHCLEPN IGQQLFAKLANNTK		LRR2	3
1251	IRLLELYGS NVQTIAPDVFTGLSRSQRLQV		LRR3	2.8

KISHTRISDLPPGIFYALREVPHLSIDISHNRINA

1316	LAADSFYPN KSYWDAVGTRSI			LRR1	2.4
1337	MGGLITSHN PLECECGLVWFGHWLRRWLRE		LRR2	2.4

SAQIKVIQKDDLKRMVQRARANTCHDPTSGRHLPILEIFPEDLLCQASALSSSGQRIFLL
SFAMALLIPIVMTTMTL

>CG8595-PA

MAAILLLLLGFSWSLAVESALAPKESESSASAMLGAGTGAAATVSLSGDYSSLLSNVPAA
SPVPANPSQPSGPANQCSWSYNGTSSV

88	HCALRLIER QPGLDLQGADGSSQLTIQ		LRR1	2
115	CSELYLFES TLPVAVFARLQT			LRR2	2.4
136	LEALRLDSC KLLQLPNNAFEGLAT		LRR3	3.4
160	LKSLRLSTH NSEWGPTRTLELFPDS		LRR4	3
185	LGGLKQLTD LDLGDNNLRQLPSGFLCPVGN		LRR5	2
215	LQVLNLTRN RIRTAEQMGFADMNCGAGSGS		LRR6	4

AGSE

249	LQVLDASHN ELRSISESWGISRLRR		LRR1	3.4
274	LQHLNLAYN NLSELSGEALAGLAS		LRR2	4
298	LRIVNLSNN HLETLPEGLFAGSKE		LRR3	3.4
322	LREIHLQQN ELYELPKGLFHRLEQ		LRR4	3.4
346	LLVVDLSGN QLTSNHVDNTTFAGLIR		LRR5	3.4
372	LIVLNLAHN ALTRIDYRTFKELYF		LRR6	4
396	LQILNLRNN SIGHIEDNAFLPLYN		LRR7	4
420	LHTLNLAEN RLHTLDDKLFNGLYV		LRR8	4
444	LSKLTLNNN LISVVEPAVFKNCSD		LRR9	4
468	LKELDLSSN QLNEVPRALQDLAM		LRR10	4
491	LRTLDLGEN QIRTFDNQSFKNLHQ		LRR11	4
515	LTGLRLIDN QIGNITVGMFQDLPR		LRR12	4
539	LSVLNLAKN RIQSIERGSFDKNFE		LRR13	4
563	LEAIRLDRN FLADINGVFATLVS		LRR14	3.4
586	LLWLNLSEN HLVWFDYAFIPSN			LRR15	4
608	LKWLDIHGN YIEALGNYYKLQEEIR		LRR16	3.4
633	VKTLDASHN RITEIGPMSIPNT			LRR17	2.8
655	IELLFINNN LIGNVQPNAFVDKAN		LRR18	2.8
679	LARVDLYAN QLSKLQLQQLRVAPVVAPKP		LRR19	3.4
708	LPEFYLGGN				LRR20	3.4
717	PFECDCTMDWLQRINNLTTRQHPRVMDMANIECVMPHARGAAVRPLSGLRPQDFL		CF1_short	3

CRYESH

778	CFALCHCCDFDACDCEMTCPSNCTCYHDQIWSTNVVDCGG		NF	4

QQTTELPRRVPMD

831	SSVVYLDGN NFPVLKNHAFIGRKN		LRR1	2.4
855	LRALYVNGS QVAAIQNRTFASLAS		LRR2	2.8
879	LQLLHLADN KLRTLHGYEFEQLSA		LRR3	4
903	LRELYLQNN QLTTIENATLAP			LRR4	4
924	LAALELIRI DGNRLVTLPIWQMHATHFGTR		LRR5	3
954	LKSISLGRN				LRR6	3.4
963	QWSCRCQFLQALTSYVADNALIVQDAQDIYCMAASSGTGSAALEDSSSNSGSL		CF1_short	3

EKRELDFNATGAACTDYYSGGSMLQHGIPESYIPLLAAALALLFLLVVIAMVFAFRESLR
IWLFAHYGVRVFGPRCEESEKLYDAVLLHSAKDSEFVCQHLAAQLETGRPPLRVC

1131	LQHRDLAHD ATHYQLLEATRVSR		LRR1	2
1154	RVVILLTRN FLQTEWARCELRRSVHDALRG		LRR2	2.4

RPQKLVIIEEPEVAFEAESDIELLPYLKTSAVHRIRRSDRHFWEKLRYALPVDYPTFRGN
NYTLELDHHNHERVKQPASPGLLYRQAPPPAYCGPADAVGIGAVPQVVPVNASVPAEQNY
STATTATPSPRPQRRGEQPGSGSGGNHHLHAQYYQHHGMRPPSEHIYSSIDSDYSTLDNE
QHMLMMPGAPGGLAMEAAQRAQTWRPKREQLHLQQAQAGTLGSKASQAAHQQQQQQQQQQ
QQQPNPTAVSGQQQGPHVQAYLV

>CG1804-PA

MHRSMDRRRSRTPRTLPVCWILLCLVAWTVAD

33	DWSLSCASN CTCKWTNGKKSAI			LRR1	2
55	CSSLQLTTI PNTLSTELQVLVLNDNHIPYL		LRR2	2

SHIFT -16!

69	TELQVLVLN DNHIPYLNREEFST		LRR1	2
92	LGLLNLQRI YLKKSEVQYIHKESFRNLKI		LRR2	3
121	LVEIDLSDN KLEMLDKDTFMGNDR		LRR3	3.4
145	LRILYLNGN PLKRLAAYQFPILPH		LRR4	4
169	LRTLDMHDC LISYIDPMSLANLNL		LRR5	2.8
193	LEFLNLKNN LLESLSEYVFQHMAN		LRR6	4
217	LKTLSLEEN				LRR7	4
226	PWQCNCKLRKFRGWYVNSRLSSVSLVCKGPPAQKDRTWDSVDDELFGCP		CF1_short	3

SHIFT -58!

217	LKTLSLEEN PWQCNCKLRKFRGWYVNSR		LRR7	4
245	LSSVSLVCK GPPAQKDRTWDSVDDELFGCP		LRR8	2.4

PRVEIFNNEE

285	VQNIDIGSN TTFSCLVYGDPLPEVAWE		LRR1	2.2
312	LNGKILDND NVLFESESIASDK			LRR2	2
334	LWSNLTVFN VTSLDAGTYACTGSNSIGSMT		LRR3	2

QNISIYLSEIVQHVLEKTPETFWYFGLIMGIFGTVFLLISISFVVCLCKRTTRQHRHANK
AGVKSSVSFNDQEKKLLDSSVTTTTNDRGDSYGIDNQPTSIGMNKGDSAGMGFNQIEIHA
VESHRHGSMLVQQQPQQQQVAGGGGMRQQLMQVKDSTCGMMSVPTSMAGHAHSHPAQISE
EFPLNVGVFPPPPEFCSNIVPNPAFGGNIFIRVSVTQDMLDGADLNMYPDLLNIPKRMQD
VQESGAGAVAVPEGQFATLPRHTARRGILKKDTSLQQQQQQHQQQHQHQQQQQQQQIQQQ
QHQQLQQQHQPSGLYTHDEIVTYNLEASGYDPHQSGYHSNAMELPPPPPPPAVTAVVQCH
HPSPNNCASCINNAPPPPSACQSPPVEVTPMRPLDSSAYPKYDNMGRRITASGGLGGSNL
SLHDEERYENETLFGQAESQTKGMPEQSQDLHQPQEVTQGQDKGGGPGEFVSL

>CG7509-PA

MSLGVWQLVS

11	LVLLMDPAE VITGPSQAKEPPIQSLDEFRQ		LRR1	2
41	RYLLPLISS DTRNCSLNACESLGI		LRR2	2.4
65	VSQLLMLIN SMPNGTQSAANDKKPPKSKAN		LRR3	2.8

GHNDGDVNGGEINAMSHLANFDLVKRVRQIESRLRSVEQPVWHLATGSQIEWNH

149	CTSGVCRCNPDTKSFTCWNTNLKSVPVTQVIP		NF	4

MN

183	MVNIDLSRN ILSTLHKDTFRGLTV		LRR1	2.8
207	LKELDISHN VLDFLPFDLFQDLDS		LRR2	3.4
231	LLVLRIQNN QLEDIDHRTFWKLRN		LRR3	3.4
255	LNILDLSKN EIGMLPESIFYHAQR		LRR4	4
279	LTVINMCDN QIQNFPPNLLRDQLM		LRR5	2.8
303	LEELDMSRN KISELSSGSIRYLTK		LRR6	3.4
327	LKTLDFGWN QIAKIDDDFFAGLRS		LRR7	3.4
351	LRTLSLHNN RISSLSGTIFNNLAN		LRR8	4
375	LVTLDLTTN RISHIDGNAFVELNN		LRR9	4
399	LNELFLGQN SMSSIPADLFLNVSA		LRR10	4
423	LTRLTLFSN NLTTLEADDFQGLNN		LRR11	4
447	LKILLLNNN ILKNFDARAFEPLSQ		LRR12	4
471	LEKLRIDSN KLMFLPHGALHGLKN		LRR13	3.4
495	LVAVKLDKN				LRR14	3.4
504	PWHCDCRALYLARWIREFVLKLWDGQQPMCRGPGDLGGHEVGLLRYDDLCDG		CF1_short	3

SHIFT -61!

495	LVAVKLDKN PWHCDCRALYLARWIR		LRR14	3.4
520	EFVLKLWDG QQPMCRGPGDLGGHEVGL		LRR15	2
547	LRYDDLCDG QWASMLSLSPRLPVRKHQIST		LRR16	2

SHIFT -19!

558	ASMLSLSPR LPVRKHQISTPMNYTD		LRR1	2.4
583	YFNLYLKHI YNGTTDEELKEADITSVSIKK		LRR2	2

VHN

>CG8896-PA

MPATSSIITIIAV

14	AACLLLLVA DAHAQQQCNWQYG			LRR1	2.4
36	LTTMDIRCS VRALESGTGTPLDLQVAEA		LRR2	2.2
64	AGRLDLQCS QELLHASELAPGLFRQLQK		LRR3	2.8
92	LSELRIDAC KLQRVPPNAFEGLMS		LRR4	2.8
116	LKRLTLESH NAVWGPGKTLELHGQSFQG		LRR5	3
144	LKELSELHL GDNNIRQLPEGVWCSMPS		LRR6	2
171	LQLLNLTQN RIRSAEFLGFSEK			LRR7	4
193	LCAGSALSN ANGAVSGGSELQTLDVSFNE		LRR8	2.8
222	LRSLPDAWG ASRLRRLQTLSLQH		LRR9	2
245	NNISTLAPN ALAGLSSLRVLNISYNHLVS		LRR10	2
274	LPSEAFAGN KELRELHLQGNDLYE		LRR11	2.4
298	LPKGLLHRL EQLLVLDLSGNQLTSHHVDNS		LRR12	2.4

SHIFT -25!

303	LHRLEQLLV LDLSGNQLTSHHVDNSTFAG		LRR1	2
332	LIRLIVLNL SNNALTRIGSKTFKELYF		LRR2	2.4
359	LQILDMRNN SIGHIEEGAFLPLYN		LRR3	3.4
383	LHTLNLAEN RLHTLDNRIFNGLYV		LRR4	4
407	LTKLTLNNN LVSIVESQAFRNCSD		LRR5	4
431	LKELDLSSN QLTEVPEAVQDLSM		LRR6	4
454	LKTLDLGEN QISEFKNNTFRNLNQ		LRR7	4
478	LTGLRLIDN RIGNITVGMFQDLPR		LRR8	4
502	LSVLNLAKN RIQSIERGAFDKNTE		LRR9	4
526	IEAIRLDKN FLTDINGIFATLAS		LRR10	2.8
549	LLWLNLSEN HLVWFDYAFIPSN			LRR11	4
571	LKWLDIHGN YIEALGNYYKLQEEIR		LRR12	3.4
596	VTTLDASHN RITEIGAMSVPNS			LRR13	2.8
618	IELLFINNN IIGQIQANTFVDKTR		LRR14	2.8
642	LARVDLYAN VLSKISLNALRVAPVSAEKP		LRR15	3.4
671	VPEFYLGGN				LRR16	2.8
680	PFECDCSMEWLQRINNLTTRQHPHVVDLGNIECLMPHSRSAPLRPLASLSASDFV		CF1_short	3

CKYESH

741	CPPTCHCCEYEQCECEVICPGNCSCFHDATWATNIVDCGR		NF	4

SHIFT -15!

766	FHDATWATN IVDCGRQDLAALPNRIPQD		LRR1	2.2
794	VSDLYLDGN NMPELEVGHLTGRRN		LRR2	3.4
818	LRALYLNAS NLMTLQNGSLAQLVN		LRR3	3.4
842	LRVLHLENN KLTALEGTEFRSLGL		LRR4	4
866	LRELYLHNN MLTHISNATFEPLVS		LRR5	4
890	LEVLRLDNN RLSSLPHLQYRHS			LRR6	4
912	LQGLTLGRN				LRR7	4
921	AWSCRCQQLRELAQFVSDNAMVVRDAHDIYCLDAGIKRELELIGNLANGPDCS		CF1_short	3

SHIFT -45!

929	LRELAQFVS DNAMVVRDAHDIYCLDAGI		LRR1	2.4
957	KRELELIGN LANGPDCSDLLDASASNISSS		LRR2	3

QDLAGGYRLPLLAAVLVLIFLVVVLIIVFVFRESVRMWLFAHYGVRVCEPRFEDAGKLYD
AIILHSEKDYEFVCRNIAAELEHGRPPFRLCIQQRDLPPQ

1087	ASHLQLVEG ARASRKIILVLT			LRR1	2.4
1108	RNLLATEWN RIEFRNAFHESLRGLAQKLVI		LRR2	2

IEETSVSAEAEDVAELSPYLKSVPSNRLLTCDRYFWEKLRYAIPIELSPRGNNYTLDHHE
RFKQPVSPGMIFRQAPPPPAYYCTEEMEANYSSATTATPSPRPTRPGGAARIVDSMPMPM
RPPSEHIYHSIESEYSAYDQHEALSMIPTGLMHQHQQQQLRLHQQQQQQQQQRLLQPQFR
AMPQQAIPAPSAPVHLRSGSGLSQASTSTQSTAQASTSAAAAQQQQQQQQQQAAGSEAAN
KNGQAFLV

>CG11910-PA

MAV

4	LWWLLSLQV VLILPPSYHLATPSSGSGQ		LRR1	2
32	LRRLWLQDH CSAGICTNVVIGRSDYVI		LRR2	3
59	LSQAPIGGT TMLTFLNSSIAKIPHLLFDTF		LRR3	2.2

SHIFT -19!

70	LTFLNSSIA KIPHLLFDTFPD			LRR1	2
91	LQVLRMENC SLETFEKPQFEGASN		LRR2	2.8
115	LMSLFLGYN RLKDIPKNIFLGADN		LRR3	4
139	LATLHLQGN QLKQLGNHSFHALKE		LRR4	4
163	VKELSLAEN QLEQISLGVFSGMRK		LRR5	3.4
187	LMDLNLAGN RLDALPRGVFDRNLN		LRR6	4
211	LTKLNLARN RFTAFESELLKLQPV		LRR7	4
235	FTQLDISGN IFQELTLNFTMLDVAIAHSCD		LRR8	2.8
265	LRRLTVYGV IHELDLHNNSLRE			LRR9	2.4
287	MPHIPLAAN VSSLDLSHNPLGN			LRR10	2.8
309	LQGNPLRRF TSLLRLNLSATGAHELPEGLF		LRR11	2

SHIFT -23!

316	RFTSLLRLN LSATGAHELPEGLFKKQSH		LRR1	2
344	LQMLDISGN SIYSLKITIFDSLKA		LRR2	3.4
368	LQYFYFQQN NWNCDFLQLLMSSFVKRKDIS		LRR3	2.8

FMEDITAPELVDDYVDGIACWYESDKQSKKCESGGSDAAMELSVVRNEIKTFTELVEKKF
VKVYRMLEEMKMKL

>CG6890-PA

MLATTHMLYVLIATCVIP

19	IFGAALSKT VLYQAPDECRWSGGGEHDIT		LRR1	2.2
48	LVCHLRTIN SELENTNFSVIQPQN		LRR2	2
72	TVRLRLECN DALFFQSSLSPDSFRSLVE		LRR3	3
100	LRDLTIEYC KLGNLTDGSFRGLQE		LRR4	2.8
124	LRNLTIRTH NGDWSTMSLEMASNSFVEFRQ		LRR5	2.4
154	LERLDLSLN NIWLIPDGMVCPLKS		LRR6	4
178	LQHLNASYN KIQDISNFYFSASLSSRKARV		LRR7	3.4

CGST

212	LQSLDLSAN KMVSLPTAMLSALGR		LRR1	4
236	LTHLNMAKN SMSFLADRAFEGLLS		LRR2	3.4
260	LRVVDLSAN RLTSLPPELFAETKQ		LRR3	3.4
284	LQEIYLRNN SINVLAPGIFGELAE		LRR4	3.4
308	LLVLDLASN ELNSQWINAATFVGLKR		LRR5	4
334	LMMLDLSAN KISRLEAHIFRPLAS		LRR6	4
358	LQILKLEDN YIDQLPGGIFADLTN		LRR7	4
382	LHTLILSRN RISVIEQRTLQGLKN		LRR8	4
406	LLVLSLDFN RISRMDQRSLVNCSQ		LRR9	4
430	LQDLHLNDN KLQAVPEALAHVQL		LRR10	4
453	LKTLDVGEN MISQIENTSITQLES		LRR11	3.4
477	LYGLRMTEN SLTHIRRGVFDRMSS		LRR12	3.4
501	LQILNLSQN KLKSIEAGSLQRNSQ		LRR13	4
525	LQAIRLDGN QLKSIAGLFTELPN		LRR14	3.4
548	LVWLNISGN RLEKFDYSHIPIG			LRR15	3.4
570	LQWLDVRAN RITQLGNYFEIESELS		LRR16	3.4
595	LSTFDASYN LLTEITASSIPNS			LRR17	2.8
617	VEVLYLNDN QISKIQPYTFFKKPN		LRR18	3.4
641	LTRVDLVRN RLTTLEPNALRLSPIAEDRE		LRR19	3.4
670	IPEFYIGHN				LRR20	2.2
679	AYECDCNLDWLQKVNRESRTQPQLMDLDQIHCRLAYARGSSHVSLIEAKSDDFL		CF1_short	3

CKYASH

739	CFALCHCCDFQACDCKMECPDRCSCYHDQSWTSNVVDCSR		NF	4

ASYEQTLPSHIPMD

793	STQLYLDGN NFRELQSHAFIGRKR		LRR1	3
817	LKVLHLNHS RIEVLHNRTFYGLLE		LRR2	3.4
841	LEVLQLQSN QLKALNGNEFQGLDN		LRR3	4
865	LQELYLQHN AIATIDTLTFTHLYH		LRR4	4
889	LKILRLDHN AITSFAVWNFLPSY		LRR5	4
912	LNELRLASN				LRR6	4
921	PWTCSCEFIDKLRDYINRHEYVVDKLKMKCDVISGNSTQQMVIYPGSGEPAS		CF1_short	3

LPVVQCSQTLPLGLDNNFNYAEQAGGENASNATSTKMILNQPPKLDYIPILVAILTAFIF
VMICISLVFIFRQEMRVWCHS

1054	RFGVRLFYN AQKDVDKNEREK			LRR1	2.4
1075	LFDAFVSYS SKDELFVNEELAPMLEMGEH		LRR2	2.2
1104	RYKLCLHQR DFPVGGYLPETIVQAIDSSRR		LRR3	2

TIMVVSENFIKSEWCRFEFKSAHQSVLRDRRRRLIVIVLGEVPQKELDPDLRLYLKTNTY
LQWGDKLFWQKLRFALPDVSSSQRSNVAGQSCHVPINHASYHHHHHVHQQAMPLPHSVHH
HQQQFMLPPPPQQPGSFRRQPSLHQQQQQQQQIRGNNNTTQQQQQQQ

1301	AALLMGGGS VGGPAPQMIPLAGGIQ		LRR1	2.2
1326	QQSLPLPPN QQPTPASRNLHM			LRR2	3


>CG5810-PA

MRM

4	LNYLNYILI AVIAFATCESQKLEEIA		LRR1	2
30	IDSLDCREN TCTNLKYPSASAVAYFSENVT		LRR2	2.4

KHLRK

65	YETLVLHSS DLANLPRKIFLNLPQ		LRR1	2.4
89	LVEFHVLEC ELQQIESVCFDGAKN		LRR2	2.2
113	LKRLNFGGN ALRVLDSNTFELATQ		LRR3	3.4
137	LEELNLSDN QLEDLPTTIFRPLKN		LRR4	4
161	LQKINLSNN RLITLSQHIFSQLGS		LRR5	3.4
185	LKSINVDSN QLVELPGELFRDQRKH		LRR6	2.8
210	LSEFSAQSN QLVRIPFNIFRE			LRR7	2.8
231	IDHLSLSFN PQLRRLHLSAKINELEATNCD		LRR8	3.4
261	LESVELDGR VIGVQLEANPKLHELKISQPQ		LRR9	2.4

SHIFT -21!

270	VIGVQLEAN PKLHELKISQPQD			LRR1	2.8
292	LEHLYLANT NLYRLDFLSKASK			LRR2	3.4
314	LVDLDVTDI VNLADLPKITSAKG		LRR3	2.4
337	LERLSFTYD NLTSNHMDMLPHLKD		LRR4	2.4
361	LNYLEISHE KGKEIFIKDLDEDFFVEEAE		LRR5	2.4
390	LNCGQLADL LEFVELPKDTTILEDRLVGDP		LRR6	2.4

RGPMRCGMA

>CG40500-PD

MQK

4	LTALDFDYN EIVRVDDYSFYGLR		LRR1	3.4
27	ISKLNMKGN RLQGMPEHAFAGLEEC		LRR2	2.8
52	MQEIDVSEN GLRTFPLMALRKLDH		LRR3	2.2
76	LRILRLSNN RIPTFYGDIQLATNNASAAAA		LRR4	4

SHIFT -16!

90	YGDIQLATN NASAAAAAVGAFQLPS		LRR1	2.4
115	LIFLDLSSN QFAEIGPDCFRAFPQ		LRR2	4
139	LKTLSFYAN QIELVQPEAFKSLRE		LRR3	3.4
163	LMSLDMSHN RIIGLDPKVFEKNKR		LRR4	3.4
187	LQTVDLSHN HIHTIGGVFSNLPQ		LRR5	3.4
210	LREVFLSEN NILELPADAFTNSTN		LRR6	3.4
234	VDVIYLESN AIAHIDPNVFSTLVN		LRR7	2.8
258	LDHLYLRSN FIPLLPVTLFDKSTK		LRR8	4
282	LTSLSLDNN EIQDLEIGMFRKLEH		LRR9	4
306	LREVRLHNN RIRRVRRGVFEPLPS		LRR10	3.4
330	LQELHIQKN SIEDIEPQAFHTLEN		LRR11	3.4
354	MQHINLQDN QLTVLEDIFPDENSS		LRR12	2.8
378	LLSVQLEAN YLHKVHPRTFSRQQK		LRR13	3.4
402	VQIMWLKDN QLTRVERSFFADTPQ		LRR14	2.8
426	LGRLYLSDN KIRDIEKDTFVNLLL		LRR15	4
450	LQFLDLSGN QLRQLRRDYFAPLQD		LRR16	4
474	LEELSLARN HIEAIEGYAFAKLKN		LRR17	4
498	LKSLDLSHN PLVQLTRDIFSNEFP		LRR18	4
522	LNSLNLGNC SLRKLEQHAFKSLTN		LRR19	3.4
546	LNELNLERN QLNPADIQTLDIPN		LRR20	4
569	LRRLLLSHN NFSYAGSVGIMAGMLDRLRS		LRR21	4
598	LQQLSMSNC SLGQIPDLLFAKNTN		LRR22	2.8
622	LVRLDLCDN RLTQINRNIFSGLNV		LRR23	4
646	FKELRLCRN ELSDFPHIALYNLST		LRR24	3.4
670	LESLDLARN QLASIDFFKLSGTLN		LRR25	4
694	LRQLILRDN KITALSGFNAVNLTQ		LRR26	4
718	LDSVDLSGN LLLSLPANFLRHSIN		LRR27	3.4
742	LQKVHLSNN RFLQIPSSALSDVSIPR		LRR28	3.4
768	LSWLNLTGN PINRIYTVKEERYPY		LRR29	4
792	LKELYICQT NLSILTSKDFEAFQA		LRR30	2.8
816	LQHLHLVNN RITRISPGAFKSLTN		LRR31	4
840	LLTLDLSVN ELEMLPKERLQGLRL		LRR32	4
864	LRFLNISHN TLKDLEEFSVDLLE		LRR33	3.4
887	MQTLDLSFN QLDRISKKTFRNLHG		LRR34	3.4
911	LLELFLMGN RMTVLSNDAFRFLRK		LRR35	4
935	LHVLDLRKN YFELVPLEPLRPLETN		LRR36	4
960	LRTLRLEEN PLHCSCDAQKLWEWLRDHRKW		LRR37	4

SLSMGSGAGRGIGGLTGGLGGDSINYLRCEHPTELRGKVFGRMEPQQFCDAPLIPKMAIQ
DIQPYSVVVSWQSRDHLGLNGFEIVYHATGDGLVTAGSPTSRERDSLRDIHDPERQRTRD
QARERSRERDRDRERDRERFSIATDEIHGKRLNGTASS

1148	TKLTKLSPN TRYHICVIGSGNWLSEPLANA		LRR1	2
1178	LQRLHTNES LREEEAVMMSAPLPESFSPYQ		LRR2	2.4

HQHNRNSNGNANPAMVQDHNKKQNPDQDLDNVVGQDNALMEVLKNSHISACTDVQTLDS

1267	TPSLVTDEN GLSSNGFIHSIL			LRR1	2
1288	TRRLGLIVG CCLGIIVFIVMISV		LRR2	2
1311	LSYVKLKKK RIENAKRQAALPPEYISYRHF		LRR3	2.4

SIPNEELTRTAANAGTVAAAAAANNSHNSNNSTDEGSSPHMSSVPSGISAHISGTSLSTG
GTGTGSTTTTATTPIGGVPLGKAGST

1427	LVGVGLVVA DSSVDVGYAHNHAHAHAHRHM		LRR1	2.4
1457	AAGVVLNTN HLSVC			LRR2	2.8


>ABI34171

MCIAHLPITFTLAILLAIASNEGAQGVESATRTAIEAIRTGIGTKPETEIADATEAEAPV
REVISLLGIIDGAESDILVPDADDKCPGGYFHCNTTAQCVPQRANCDGSVDCDDASDEVN
CVNEVDAKYWDHLYRKQPFGRHDNLRIGE

150	CLWPNENFSCPCRGDEILCRFQQLTDIPER		NF	4
180	LPQHDLATL DLTGNNFETIHETFFSELPD		LRR1	2
209	VDSLVLKFC SIREIASHAFDRLADNP		LRR2	2.8
235	LRTLYMDDN KLPHLPEHFFPEGNQ		LRR3	3.4
259	LSILILARN HLHHLKRSDFLNLQK		LRR4	4
283	LQELDLRGN RIGNFEAEVFARLPN		LRR5	4
307	LEVLYLNEN HLKRLDPDRFPRTLLN		LRR6	4
332	LHTLSLAYN QIEDIAANTFPFPR		LRR7	4
355	LRYLFLAGN RLSHIRDETFCNLSN		LRR8	4
379	LQGLHLNEN RIEGFDLEAFACLKN		LRR9	4
403	LSSLLLTGN RFQTLDSRVLKN			LRR10	4
424	LTSLDYIYF SWFHLCSAAMNVRVCDPHGDG		LRR11	2

I

455	SSKLHLLDN QILRGSVWVMASIAVV		LRR1	3
480	GNLLVLLGR YFYKSRSNVEHS			LRR2	2.4
501	LYLRHLAAS DFLMGIYLTLIACADISFRGE		LRR3	2.4

YIKYEETWRHSGVCAFAGFLSTFSCQ

557	SSTLLLTLV TWDRLMSVTRPLKPRDTEKV		LRR1	2
586	RIVLRLLLL WGISFGLAAAPLLPNPYFGSH		LRR2	2

SHIFT -25!

591	LLLLWGISF GLAAAPLLPNPYFGSHFYGNN		LRR1	2.4
621	GVCLSLHIH DPYAKGWEYSAL			LRR2	2.4
642	LFILVNTLS LIFILFSYIRMLQAIRDSGGG		LRR3	2.4

MRSTHSGRENVVATRFAIIVTTDCACWLPIIVVKLAALSGCEISPDLYAWLAVLVLPVNS
ALNPVLYTLTTAAFKQQLRRYCHT

756	LPSCSLVNN ETRSQTQTAYESGLSVS		LRR1	3
782	LAHLGGGVG GGSGRKRMSHRQMSYL		LRR2	2.4


>CG8355-PC

MAAPSRTTLMPPPFRLQLRLLILPILLLLRHDAVHAEPYSGGFGSSAVSSGGLGSVGIHI
PGGGVGVITEAR

73	CPRVCSCTGLNVDCSHRGLTSVPRKISAD		NF	4
102	VERLELQGN NLTVIYETDFQRLTK		LRR1	3.4
126	LRMLQLTDN QIHTIERNSFQDLVS		LRR2	4
150	LERLRLNNN RLKAIPENFVTSSAS		LRR3	4
174	LLRLDISNN VITTVGRRVFKGAQS		LRR4	3.4
198	LRSLQLDNN QITCLDEHAFKGLVE		LRR5	4
222	LEILTLNNN NLTSLPHNIFGGLGR		LRR6	4
246	LRALRLSDN				LRR7	4
255	PFACDCHLSWLSRFLRSATRLAPYTRCQSPSQLKGQNVADLHDQEFKCS		CF1_short	3

GLTEHAPMECGAENS

319	CPHPCRCADGIVDCREKSLTSVPVTLPDD		NF	4
348	TTELRLEQN FITELPPKSFSSFRR		LRR1	3
372	LRRIDLSNN NISRIAHDALSGLKQ		LRR2	3.4
396	LTTLVLYGN KIKDLPSGVFKGLGS		LRR3	4
420	LQLLLLNAN EISCIRKDAFRDLHS		LRR4	4
444	LSLLSLYDN NIQSLANGTFDAMKS		LRR5	4
468	IKTVHLAKN				LRR6	2.8
477	PFICDCNLRWLADYLHKNPIETSGARCESPKRMHRRRIESLREEKFKCS		CF1_short	3

WDELRMKLSGECRMDSD

543	CPAMCHCEGTTVDCTGRGLKEIPRDIP		NF	4
570	LHTTELLLN DNELGRISSDGLFGRLPH		LRR1	3
597	LVKLELKRN QLTGIEPNAFEGASH		LRR2	4
621	IQELQLGEN KIKEISNKMFLGLHQ		LRR3	3.4
645	LKTLNLYDN QISCVMPGSFEHLNS		LRR4	4
669	LTSLNLASN				LRR5	4
678	PFNCNCHLAWFAEWLRKKSLNGGAARCGAPSKVRDVQIKDLPHSEFKCS		CF1_short	3

SHIFT -58!

669	LTSLNLASN PFNCNCHLAWFAEW		LRR5	4
692	LRKKSLNGG AARCGAPSKVRDVQIKDLPHS		LRR6	2

EFKCSSENSEGCLGDGY

739	CPPSCTCTGTVVRCSRNQLKEIPRGIPAE		NF	4
768	TSELYLESN EIEQIHYERIRHLRS		LRR1	3
792	LTRLDLSNN QITILSNYTFANLTK		LRR2	4
816	LSTLIISYN KLQCLQRHALSGLNN		LRR3	3.4
840	LRVLSLHGN RISMLPEGSFEDLKS		LRR4	4
864	LTHIALGSN				LRR5	3.4
873	PLYCDCGLKWFSDWIKLDYVEPGIARCAEPEQMKDKLILSTPSSSFVCR		CF1_short	3

GRVRNDILAKCNACFEQPCQNQAQCVALPQREYQCLCQPGYHGKHCEFMIDACYGNPCRN
NATCTVLEEGRFSCQCAPGYTGARCETNIDDCLGEIKCQNNATCIDGVESYKCECQPGFS
GEFCDTKIQFCSPEFNPCANGAKCMDHFTHYSCDCQAGFHGTNCTDNIDDCQNHMCQNGG
TCVDGINDYQCRCPDDYTGKYCEGHNMISMMYPQTSPCQNHECKHGVCFQPNAQGSDYLC
RCHPGYTGKWCEYLTSISFVHNNSFVELEPLRTRPEANVTIVFSSAEQNGILMYDGQDAH
LAVELFNGRIRVSYDVGNHPVSTMYSFEMVADGKYHA

1259	VELLAIKKN FTLRVDRGLARSIINEGS		LRR1	2.8
1286	NDYLKLTTP MFLGGLPVDPAQQAYKNWQIR		LRR2	2

NLTSFKGCMKEVWINHKLVDFGNAQRQQKITPGCALLEGEQQEEEDDEQDFMDETPHIKE
EPVDPCLENKCRRGSRCVPNSNARDGYQCKCKHGQRGRYCDQGEGSTEPPTVTAASTCRK
EQVREYYTENDCRSRQPLKYAKCVGGCGNQCCAAKIVRRRKVRMVCSNNRKYIKNLDIVR
KCGCTKKCY

>ENSP00000260010

MPHTLWMVWVLGVIISLSKEESSN

25	QASLSCDRN GICKGSSGSLNSIPSGLTEA		LRR1	2
54	VKSLDLSNN RITYISNSDLQRCVN		LRR2	3.4
78	LQALVLTSN GINTIEEDSFSSLGS		LRR3	4
102	LEHLDLSYN YLSNLSSSWFKPLSS		LRR4	4
126	LTFLNLLGN PYKTLGETSLFSH			LRR5	4
148	LTKLQILRV GNMDTFTKIQRKDFAGLTF		LRR6	2.4
176	LEELEIDAS DLQSYEPKSLKSIQN		LRR7	2.8
200	VSHLILHMK QHILLLEIFVDVTSS		LRR8	2.4
224	VECLELRDT DLDTFHFSELSTGETNS		LRR9	2.8
250	LIKKFTFRN VKITDESLFQVMKLLNQISG		LRR10	2
279	LLELEFDDC TLNGVGNFRASDNDRVIDPGK		LRR11	2.8

SHIFT -27!

282	LEFDDCTLN GVGNFRASDNDRVIDPGKVET		LRR1	2
312	LTIRRLHIP RFYLFYDLSTLYSLTERVKRI		LRR2	2

SHIFT -14!

328	LSTLYSLTE RVKRITVENSKVF			LRR1	2
350	LVPCLLSQH LKSLEYLDLSEN			LRR2	2
371	LMVEEYLKN SACEDAWPSLQTLI		LRR3	2
394	LRQNHLASL EKTGETLLTLKN			LRR4	2
415	LTNIDISKN SFHSMPETCQWPEK		LRR5	2.8
438	MKYLNLSST RIHSVTGCIPKT			LRR6	2.8
459	LEILDVSNN NLNLFSLNLPQLKELYISRNK		LRR7	3.4
489	LMTLPDASL LPMLLVLKISRNAITTFSKEQ		LRR8	2

SHIFT -22!

497	LLPMLLVLK ISRNAITTFSKEQLDSFHT		LRR1	2.4
525	LKTLEAGGN				LRR2	3.4
534	NFICSCEFLSFTQEQQALAKVLIDWPANYLCDSPSHVRGQQVQDVRLSVSECH		CF1_short	3

SHIFT -12!

575	VQDVRLSVS ECHRTALVSGMCCA		LRR1	2.2
598	LFLLILLTG VLCHRFHGLWYMKMMWAWLQA		LRR2	3

KRKPRKAPSRNICYDAFVSYSERDAYWVENLMVQELENFNP

669	PFKLCLHKR DFIPGKWIIDNIIDSIEKSH		LRR1	2
698	KTVFVLSEN FVKSEWCKYELDFS		LRR2	2.4
721	HFRLFDENN DAAILILLEPIEKKAIPQR		LRR3	2
749	FCKLRKIMN TKTYLEWPMDEAQREGFWVNL		LRR4	2.4

RAAIKS

>ENSP00000343924

MRSPLCWLLPLLILASVAQGQPTRRPRPGTGPGRRPRPRPRPTPSFPQPDEPAEPTDLPP
PLPPGPPSIFPD

73	CPRECYCPPDFPSALYCDSRNLRKVPVIPPR		NF	4
104	IHYLYLQNN FITELPVESFQNATG		LRR1	3.4
128	LRWINLDNN RIRKIDQRVLEKLPG		LRR2	3.4
152	LVFLYMEKN QLEEVPSALPRN			LRR3	3.4
173	LEQLRLSQN HISRIPPGVFSKLEN		LRR4	4
197	LLLLDLQHN RLSDGVFKPDTFHGLKN		LRR5	4
223	LMQLNLAHN ILRKMPPRVPTA			LRR6	4
244	IHQLYLDSN KIETIPNGYFKSFPN		LRR7	3.4
268	LAFIRLNYN KLTDRGLPKNSFNISN		LRR8	3.4
293	LLVLHLSHN RISSVPAINNRLEHLYLNNNS		LRR9	4

IEKINGTQICPNDLVAFHDFSSDLENVPHLRYLRLDGNYLKPPIPLDLMMCFRLLQSVVI

>ENSP00000265991

MESERSKRMGNACIPLKRI

20	AYFLCLLSA LLLTEGKKPAKPKCPAVCTC		LRR1	2.4
49	TKDNALCEN ARSIPRTVPPDVISLSFVRSG		LRR2	2

SHIFT -10!

69	VISLSFVRS GFTEISEGSFLFTPS		LRR1	2.2
93	LQLLLFTSN SFDVISDDAFIGLPH		LRR2	3.4
117	LEYLFIENN NIKSISRHTFRGLKS		LRR3	3.4
141	LIHLSLANN NLQTLPKDIFKGLDS		LRR4	4
165	LTNVDLRGN				LRR5	3.4
174	SFNCDCKLKWLVEWLGHTNATVEDIYCEGPPEYKKRKINSLSSKDFDCI		CF1_short	3

ITEFAKSQDLPYQSLSIDTFSYLNDEYVVIAQPFTGKCIFLEWDHVEKTFRNYDNITGTS
TVVCKPIVIETQLYVIVAQLFGGSHIYKRDSFANKFIKIQDIEILKIRKPNDIETFKIEN
NWYFVVADSSKAGFTTIYKWNGNGFYSHQSLHAWYRDTDVEYLEIVRTPQTLRTPHLILS
SSSQRPVIYQWNKATQLFTNQTDIPNMEDVYAVKHFSVKGDVYICLTRFIGDSKVMKWGG
SSFQDIQRMPSRGSMVFQPLQINNYQYAILGSDYSFTQVYNWDAEKAKFVKFQELNVQAP
RSFTHVSINKRNFLFASSFKGNTQIYKHVIVDLSA

>ENSP00000258969

MVRPMLLLSLGLLAGLLPALAA

23	CPQNCHCHSDLQHVICDKVGLQKIPKVSEK		NF	4
53	TKLLNLQRN NFPVLAANSFRAMPN		LRR1	3
77	LVSLHLQHC QIREVAAGAFRGLKQ		LRR2	3.4
101	LIYLYLSHN DIRVLRAGAFDDLTE		LRR3	4
125	LTYLYLDHN KVTELPRGLLSPLVN		LRR4	4
149	LFILQLNNN KIRELRAGAFQGAKD		LRR5	4
173	LRWLYLSEN ALSSLQPGALDDVEN		LRR6	4
197	LAKFHVDRN QLSSYPSAALSKLRV		LRR7	2.8
221	VEELKLSHN PLKSIPDNAFQSFGRY		LRR8	3.4
246	LETLWLDNT NLEKFSDGAFLGVTT		LRR9	3.4
270	LKHVHLENN RLNQLPSNFPFDS			LRR10	3.4
292	LETLALTNN				LRR11	4
301	PWKCTCQLRGLRRWLEAKASRPDATCASPAKFKGQHIRDTDAFRSCKF		CF1_short	3

PTKRSKKAGRH

>ENSP00000330631

LSLPGCCVELLLLLVAAELPLGGG

25	CPRDCVCYPAPMTVSCQAHNFAAIPEGIPVD		NF	4
56	SERVFLQNN RIGLLQPGHFSPA			LRR1	2.4
78	MVTLWIYSN NITYIHPSTFEGFVH		LRR2	2.8
102	LEELDLGDN RQLRTLAPETFQGLVK		LRR3	4
127	LHALYLYKC GLSALPAGVFGGLHS		LRR4	3.4
151	LQYLYLQDN HIEYLQDDIFVDLVN		LRR5	4
175	LSHLFLHGN KLWSLGPGTFRGLVN		LRR6	4
199	LDRLLLHEN QLQWVHHKAFHDLRR		LRR7	4
223	LTTLFLFNN SLSELQGECLAPLGA		LRR8	4
247	LEFLRLNGN				LRR9	4
256	PWDCGCRARSLWEWLQRFRGSSSAVPCVSPGLRHGQDLKLLRAEDFRNC		CF1_short	3

SHIFT -58!

247	LEFLRLNGN PWDCGCRARSLWEW		LRR9	4
270	LQRFRGSSS AVPCVSPGLRHGQD		LRR10	2.2
293	LKLLRAEDF RNCTGPASPHQIKSHTLTTTD		LRR11	2.4

RAARKEHHSPHGPTRSKGHPHGPRPGHRKPGKNCTNPRNRNQISKAGAGKQAPELPDYAP
DYQHKFSFDIMPTARPKRKGKCARRTPIRAPSGVQQASSASSLGASLLAWTLGLAVTLR

>ENSP00000347041

MQWTSLLLLAGLFSLSQAQYEDDPHWWFHYLRSQQSTYYDPYDPYPYETYEPYPYGVDEG
PAYTYGSPSPPDPRD

76	CPQECDCPPNFPTAMYCDNRNLKYLPFVPSR		NF	4
107	MKYVYFQNN QITSIQEGVFDNATG		LRR1	2.2
131	LLWIALHGN QITSDKVGRKVFSKLRH		LRR2	3.4
157	LERLYLDHN NLTRMPGPLPRS			LRR3	4
178	LRELHLDHN QISRVPNNALEGLEN		LRR4	4
202	LTALYLQHN EIQEVGSSMRGLRS		LRR5	4
225	LILLDLSYN HLRKVPDGLPSA			LRR6	4
246	LEQLYMEHN NVYTVPDSYFRGAPK		LRR7	3.4
270	LLYVRLSHN SLTNNGLASNTFNSSS		LRR8	3.4
295	LLELDLSYN QLQKIPPVNTNLEN		LRR9	4
318	LYLQGNRIN EFSISSFCTVVDVVNFSK		LRR10	2
345	LQVLRLDGN EIKRSAMPADAPLC		LRR11	4
368	LRLASLIEI			LRR12	2.4


>ENSP00000021763

MRGA

5	PARLLLPLL PWLLLLLAPEARGAP		LRR1	2
29	GCPLSIRSC KCSGERPKGLSGGVPGPARRR		LRR2	2.2

VVCSGGDLPE

69	PPEPGLLPN GTVTLLLSNNKITGLRN		LRR1	2
95	GSFLGLSLL EKLDLRNNIISTVQP		LRR2	2.4
119	GAFLGLGEL KRLDLSNNRIGCLTSETFQ		LRR3	2.4
147	GLPRLLRLN ISGNIFSSLQPGVFDE		LRR4	2.4
172	LPALKVVDL GTEFLTCDCHLRWLLPWAQ		LRR5	2.4
200	NRSLQLSEH TLCAYPSALHAQALGS		LRR6	2
225	LQEAQLCCE GALELHTHHLIPSLRQVVFQ		LRR7	2.4
254	GDRLPFQCS ASYLGNDTRIRWYHNRAPVEG		LRR8	2.2

DEQAGILLAESLIHDCTFITSELT

308	LSHIGVWAS GEWECTVSMAQGNASKK		LRR1	2.2
334	VEIVVLETS ASYCPAERVANNRGDFRWPRT		LRR2	2.2
364	LAGITAYQS CLQYPFTSVPLGGGAPGTRAS		LRR3	2.2

RRCDRAGRWEPG

406	DYSHCLYTN DITRVLYTFVLMPINA		LRR1	2
431	SNALTLAHQ LRVYTAEAASFSDMMDVVYVA		LRR2	2

QMIQKFLGYVDQIKELVEVMVDMASNLMLVDEHLLWLAQREDKACSRIVGALERIGGAAL
SPHAQHISVNARNVALEAYLIKPHSYVGLTCTAFQRREGGVPGTRPGSPGQNPPPEPEPP
ADQQLRFRCTTGRPNVSLSSFHIKNSVA

609	LASIQLPPS LFSSLPAALAPPVPPDC		LRR1	2.8
635	TLQLLVFRN GRLFHSHSNTSRPGAAGPGKR		LRR2	2.4

RGVATPVIFAGTSGCGVGNLTEPVAVSLRHWAEGAEPVAAWWSQEGPGEAGGWTSEGCQL
RSSQPNVSALHCQHLGNVAV

745	LMELSAFPR EVGGAGAGLHPVVYPCTA		LRR1	2.4
772	LLLLCLFAT IITYILNHSSIRVSRKGWHM		LRR2	3.4
801	LLNLCFHIA MTSAVFAGGITLTNYQMVCQ		LRR3	2.4
830	AVGITLHYS SLSTLLWMGVKARVLHKELTW		LRR4	2.2

RAPPPQEGDPALPTPSPMLRFYLIAGGIPLIICGITAAVNIHNYRDHSPYCWLVWRPSLG
AFYI

924	PVALILLIT WIYFLCAGLRLRGP		LRR1	2.4
947	LAQNPKAGN SRASLEAGEELRGSTRLRGSG		LRR2	2

PLLSDSGSLLATGSARVGTPGPPEDGDSLYSPGVQLGALVTTHFLYLAM

1026	WACGALAVS QRWLPRVVCSCLYGVA		LRR1	2.2
1051	ASALGLFVF THHCARRRDVRASWRACCPPA		LRR2	2.4

SPAAPHAPPRALPAAAEDGSPVFGEGPPSLKSSPSGSSGHPLALGPCKLTNLQLAQSQVC
EAGAAAGGEGEPEPAGTRGNLAHRHPNNVHHGRRAHKSRAKGHRAGEACGKNR

1194	LKALRGGAA GALELLSSESGSLHNSPT		LRR1	2.4
1221	DSYLGSSRN SPGAGLQLEGEPMLTPSEGSD		LRR2	2

TSAAPLSEAGRAGQRRSASRDSLKGGGALEKESHRRSYPLNAASLNGAPKGGKYDDVTLM
GAEVASGGCMKTGLWKSETTV

>OTTHUMP00000009643

MRPRAPACAAAALGLCSLLLLLAPGHA

28	CPAGCACTDPHTVDCRDRGLPSVPDPFP		NF	4
56	LDVRKLLVA GNRIQRIPEDFFIFYGD		LRR1	2
82	LVYLDFRNN SLRSLEEGTFSGSAK		LRR2	3.4
106	LVFLDLSYN NLTQLGAGAFRSAGR		LRR3	4
130	LVKLSLANN NLVGVHEDAFETLES		LRR4	4
154	LQVLELNDN NLRSLSVAALAALPA		LRR5	4
178	LRSLRLDGN				LRR6	4
187	PWLCDCDFAHLFSWIQENASKLPKGLDEIQCSLPMESRRISLRELSEASFSEC		CF1_short	3

RFSLSLTDLCIIIFSGVAVSIAAIISSFFLATVVQCLQRCAPNKDAEDEDEDKDD

>ENSP00000291592

MGTVRPPRPSLLLVSTRESCLFL

24	LFCLHLGAA CPQPCRCPDHAGAVAVFCS		LRR1	3
52	LRGLQEVPE DIPANTVLLKLDANKISHLPD		LRR2	2

SHIFT -16!

66	TVLLKLDAN KISHLPDGAFQHLHR		LRR1	3
90	LRELDLSHN AIEAIGSATFAGLAGG		LRR2	4
115	LRLLDLSYN RIQRIPKDALGKL			LRR3	4
137	SAKIRLSHN				LRR4	2.4
146	PLHCECALQEALWELKLDPDSVDEIACHTSVQEEFVGKPLVQALDAGAS		CF1_short	3

SHIFT -38!

157	LWELKLDPD SVDEIACHTSVQEEFVGKPL		LRR1	3
186	VQALDAGAS LCSVPHRTTDVAMLVTMFGWF		LRR2	2.2

AMVIAYVVYYVRHNQEDARRHLEYLKSLPSAPASKDPIGPGP

>ENSP00000282970

MALRRGGCGALGLLLLLLGAACLIPRSAQVRRLAR

36	CPATCSCTKESIICVGSSWVPRIVPGD		NF	4
63	ISSLSLVNG TFSEIKDRMFSHLPS		LRR1	2.4
87	LQLLLLNSN SFTIIRDDAFAGLFH		LRR2	4
111	LEYLFIEGN KIETISRNAFRGLRD		LRR3	3.4
135	LTHLSLANN HIKALPRDVFSDLDS		LRR4	4
159	LIELDLRGN				LRR5	4
168	KFECDCKAKWLYLWLKMTNSTVSDVLCIGPPEYQEKKLNDVTSFDYECT		CF1_short	3

TTDFVVHQTLPYQSVSVDTFNSKNDVYVAIAQPSMENCMVLEWDHIEMNFRSYDNITGQS
IVGCKAILIDDQVFVVVAQLFGGSHIYKYDESWTKFVKFQDIEVSRISKPNDIELFQIDD
ETFFVIADSSKAGLSTVYKWNSKGFYSYQSLHEWFRDTDAEFVDIDGKSHLILSSRSQVP
IILQWNKSSKKFVPHGDIPNMEDVLAVKSFRMQNTLYLSLTRFIGDSRVMRWNSKQFVEI
QALPSRGAMTLQPFSFKDNHYLALGSDYTFSQIYQWDKEKQLFKKFKEIYVQAPRSFTAV
STDRRDFFFASSFKGKTKIFEHIIVDLSL

>ENSP00000260061

MRPQ

5	ILLLLALLT LGLAAQHQDKVPCKMVDKKVS		LRR1	2.2
35	CQVLGLLQV PSVLPPDTETLD			LRR2	2
56	LSGNQLRSI LASPLGFYTALRHLDLSTNEI		LRR3	2

SHIFT -11!

75	LRHLDLSTN EISFLQPGAFQALTH		LRR1	4
99	LEHLSLAHN RLAMATALSAGGLGPLPR		LRR2	4
126	VTSLDLSGN SLYSGLLERLLGEAPS		LRR3	3.4
151	LHTLSLAEN SLTRLTRHTFRDMPA		LRR4	4
175	LEQLDLHSN VLMDIEDGAFEGLPR		LRR5	4
199	LTHLNLSRN SLTCISDFSLQQ			LRR6	4
220	LRVLDLSCN SIEAFQTASQPQAEFQ		LRR7	4
245	LTWLDLREN KLLHFPDLAALPR			LRR8	4
267	LIYLNLSNN LIRLPTGPPQDSKGIHAPSEG		LRR9	4
297	WSALPLSAP SGNASGRPLSQLLNLDLSYNE		LRR10	2.4

SHIFT -16!

311	GRPLSQLLN LDLSYNEIELIPDSFLEHLTS		LRR1	2.4
341	LCFLNLSRN CLRTFEARRLGSLPC		LRR2	4
365	LMLLDLSHN ALETLELGARALGS		LRR3	4
388	LRTLLLQGN ALRDLPPYTFANLAS		LRR4	4
412	LQRLNLQGN RVSPCGGPDEPGPSGCVAFSG		LRR5	4

ITS

445	LRSLSLVDN EIELLRAGAFLHTP		LRR1	4
468	LTELDLSSN PGLEVATGALGGLEAS		LRR2	4
493	LEVLALQGN GLMVLQVDLPCFIC		LRR3	4
516	LKRLNLAEN RLSHLPAWTQAVS			LRR4	4
538	LEVLDLRNN SFSLLPGSAMGGLETS		LRR5	4
563	LRRLYLQGN PLSCCGNGWLAAQLHQGRVDV		LRR6	4

DATQDLICRFSSQEEVSLSHVRPEDCEKGGLKNINLIIILTFILVSAILLTTLAACCCVR
RQKFNQQYKA

>ENSP00000320697

MLLWILLLETSLCFAAGNVTGDV

24	CKEKICSCNEIEGDLHVDCEKKGFTSLQRFTAPT		NF	4

SQ

60	FYHLFLHGN SLTRLFPNEFANFYN		LRR1	3.4
84	AVSLHMENN GLHEIVPGAFLGLQL		LRR2	2.8
108	VKRLHINNN KIKSFRKQTFLGLDD		LRR3	2.8
132	LEYLQADFN LLRDIDPGAFQDLNK		LRR4	3.4
156	LEVLILNDN LISTLPANVFQYVP		LRR5	4
179	ITHLDLRGN RLKTLPYEEVLEQIPG		LRR6	3.4
204	IAEILLEDN				LRR7	2.8
213	PWDCTCDLLSLKEWLENIPKNALIGRVVCEAPTRLQGKDLNETTEQDLCPL		CF1_short	3

SHIFT -46!

218	CDLLSLKEW LENIPKNALIGRVVCEAPTR		LRR1	2
247	LQGKDLNET TEQDLCPLKNRVDSSLPAPPA		LRR2	2.4

QEETFAPGPLPTPFKTNGQEDHATPGSAPNGGTKIPGNWQIKIRPTAAIATGSSRNKPLA
NSLP

341	CPGGCSCDHIPGSGLKMNCNNRNVSSLADLKPKL		NF	4

SHIFT -25!

350	IPGSGLKMN CNNRNVSSLADLKPKLSN		LRR1	2.4
377	VQELFLRDN KIHSIRKSHFVDYKN		LRR2	3.4
401	LILLDLGNN NIATVENNTFKNLLD		LRR3	4
425	LRWLYMDSN YLDTLSREKFAGLQN		LRR4	3.4
449	LEYLNVEYN AIQLILPGTFNAMPK		LRR5	3.4
473	LRILILNNN LLRSLPVDVFAGVS		LRR6	4
496	LSKLSLHNN YFMYLPVAGVLDQLTS		LRR7	4
521	IIQIDLHGN				LRR8	2.8
530	PWECSCTIVPFKQWAERLGSEVLMSDLKCETPVNFFRKDFMLLSNDEICPQ		CF1_short	3

SHIFT -60!

521	IIQIDLHGN PWECSCTIVPFKQWAER		LRR8	2.8
547	LGSEVLMSD LKCETPVNFFRKDFMLLSNDE		LRR9	2

ICPQLYARISPTLTSHSKNSTG

599	LAETGTHSN SYLDTSRVSISV			LRR1	2
620	LVPGLLLVF VTSAFTVVGMLVFILRNRKRS		LRR2	2.4

KRRDANSSASEINSLQTVCDSSYWHNGPYNADGAHRVYDCGSHSLSD

>ENSP00000262304

MPPAAPARLALALGLGLWLGALAGGPGRG

30	CGPCEPPCLCGPAPGAACRVNCSGRGLRTLGPALRIP		NF	4

SHIFT -11!

56	LRTLGPALR IPADATALDVSHNLLR		LRR1	2
81	ALDVGLLAN LSALAELDISNNKISTLEEGI		LRR2	2.8

SHIFT -24!

87	LANLSALAE LDISNNKISTLEEGIFANLFN		LRR1	2.4
117	LSEINLSGN				LRR2	3.4
126	PFECDCGLAWLPRWAEEQQVRVVQPEAATCAGPGSLAGQPLLGIPLLDSGCG		CF1_short	3

SHIFT -45!

133	LAWLPRWAE EQQVRVVQPEAATCAGPGS		LRR1	2
161	LAGQPLLGI PLLDSGCGEEYVACLPDNSSG		LRR2	2

TVAAVSFSAAHEGLLQPEACSAFCFSTGQG

221	LAALSEQGW CLCGAAQPSSAS			LRR1	2
242	FACLSLCSG PPPPPAPTCRGPTLLQHVFPA		LRR2	2.4

SPGATLVGPHGPLASGQLAAFHIAAPLPVTATRWDFGDGSAEVDAAGPAASHRYVLPGRY
HV

334	TAVLALGAG SALLGTDVQVEAA			LRR1	2
356	PAALELVCP SSVQSDESLDLSIQNRG		LRR2	2
382	GSGLEAAYS IVALGEEPARAVHPLCPSDTE		LRR3	2.2

IFPGNGHCYRLVVEKAAWLQAQEQCQAWA

441	GAALAMVDS PAVQRFLVSRVTRS		LRR1	2.2
464	LDVWIGFST VQGVEVGPAPQGE			LRR2	2.2
486	AFSLESCQN WLPGEPHPATAEHCVRLGPTG		LRR3	2.4

WCNTDLCSAPHSYVCELQPGGPVQDAENLLVGAPSGDLQGP

557	LTPLAQQDG LSAPHEPVEVMV			LRR1	2
578	FPGLRLSRE AFLTTAEFGTQELRRPAQLR		LRR2	2.4
607	LQVYRLLST AGTPENGSEPESRSPDNRTQL		LRR3	2.4

APACMPGGRWCPGANICLPLDASCHPQACANGCTSGPGLPGAPYA

682	LWREFLFSV PAGPPAQYSVTLHG		LRR1	2
705	QDVLMLPGD LVGLQHDAGPGALLHCSPAPG		LRR2	2

SHIFT -21!

714	LVGLQHDAG PGALLHCSPAPGHPGPR		LRR1	2
740	APYLSANAS SWLPHLPAQLEGTWACP		LRR2	2.2
766	ACALRLLAA TEQLTVLLGLRP			LRR3	2.4
787	NPGLRLPGR YEVRAEVGNGVSRHNLSCSFD		LRR4	2

VVSPVAGLRVIYPAPRDGRLYVPTNGSALVLQVDSGANATATARWPGGSVSARFENVCPA
LVATFVPGCPWETNDTLFSVVALPWLSEGEHVVDVVVENSAS

919	RANLSLRVT AEEPICGLRATPSPEARV		LRR1	2.4
946	LQGVLVRYS PVVEAGSDMVFRWTINDKQS		LRR2	2.2
975	LTFQNVVFN VIYQSAAVFKLSLTASNH		LRR3	2.4
1002	VSNVTVNYN VTVERMNRMQGLQVS		LRR4	2.2
1026	TVPAVLSPN ATLALTAGVLVDSAVEVAFLW		LRR5	2.4

SHIFT -22!

1034	NATLALTAG VLVDSAVEVAFLWTFGDGEQA		LRR1	2
1064	LHQFQPPYN ESFPVPDPSVAQVLVEHNVMH		LRR2	2.4

SHIFT -12!

1082	VAQVLVEHN VMHTYAAPGEYL			LRR1	2.2
1103	LTVLASNAF ENLTQQVPVSVRAS		LRR2	2
1126	LPSVAVGVS DGVLVAGRPVTFYPHPLPSPG		LRR3	2.2

GVLYTWDFGDGSPVLTQSQPAANHTYASRG

1186	TYHVRLEVN NTVSGAAAQADVRVFEE		LRR1	2.4
1212	LRGLSVDMS LAVEQGAPVVVSAAVQTGDNI		LRR2	2.8

TWTFDMGDGTVLSGPEATVEHVYLRAQNCTVTVGAASPAGHLARSLHVLVFVLEVLRVEP
AACIPTQPDARLTAYVTGNPAHYLFD

1328	WTFGDGSSN TTVRGCPTVTHNFTRSG		LRR1	2.2
1354	TFPLALVLS SRVNRAHYFTSICVEPEVGNV		LRR2	2.4

TLQPERQFVQLGDEAWLVACAWPPFPYRYTWDFGTEEAAPTRARGPEVTFIYRDPGSYLV
TVTASNNISAANDSALVEVQEPVLVTSIKV

1474	NGSLGLELQ QPYLFSAVGRGRPASY		LRR1	2
1499	LWDLGDGGW LEGPEVTHAYNSTGDFTVRVA		LRR2	2

GWNEVSRSEAWLNVTVKRRVRGLVVNASRTVVPLNGSVSFSTSLEAGSDVRYSWVLCDRC
TPIPGGPTISYTFRSVGTFNIIVTAENEVGSAQDSIFVYVLQLIEGLQVVGGGRYFPTNH
TVQLQAVVRDGTNVSYSWTAWRDRGPALAGSGKGFSLTVLEAGTYHVQLRATNMLGSAWA
DCTMDFVEPVGWLMVAASPNPAAVNTSVTLSAELAGGSGVVYTWSLEEGLSWETSEPFTT
HSFP

1773	TPGLHLVTM TAGNPLGSANATVEVDVQVP		LRR1	2
1802	VSGLSIRAS EPGGSFVAAGSSVPF		LRR2	2.2
1826	WGQLATGTN VSWCWAVPGGSSKRGPHVTMV		LRR3	2.4

FPD

1859	AGTFSIRLN ASNAVSWVSATYNLTAEEP		LRR1	2.2
1887	IVGLVLWAS SKVVAPGQLVHFQILLAAGSA		LRR2	2.8

VTFRLQVGGANPEVLPGPRFSHSFPRVGDHVVSVRGKNHVSWAQAQVRIVVLEAVSGLQV
PNCCEPGIATGTERNFTARVQRGSRVAYAWYFSLQKVQGDS

2018	LVILSGRDV TYTPVAAGLLEIQVR		LRR1	2.4
2042	AFNALGSEN RTLVLEVQDAVQYVA		LRR2	2.2
2066	LQSGPCFTN RSAQFEAATSPSPRRVAYHWD		LRR3	2.4

FGDGSPGQDTDEPRAEHSYLRPGDYRVQVNASNLVSFFVAQATVTVQVLACREPEVDVVL
PLQVLMRRSQRNYLEAHVDLRDCVTYQTEYRWEVYRTASCQRPGRPARVALPGVDVSRPR
LVLPRLALPVGHYCFVFVVSFGDTPLTQSIQANVTVAPERLVPIIEGGSYRVWSDTRDLV
LDGSESYDPNLEDGDQTPLSFHWACVASTQREAGGCALNFGPRGSSTVTIPRERLAAGVE
YTFSLTVWKAGRKEEATNQTVLIRSGRVPIVSLECVSCKAQAVYEVSRSSYVY

2389	LEGRCLNCS SGSKRGRWAARTFS		LRR1	2.4
2412	NKTLVLDET TTSTGSAGMRLV			LRR2	2.4
2433	LRRGVLRDG EGYTFTLTVLGRSGEEEG		LRR3	2.4
2460	CASIRLSPN RPPLGGSCRLFP			LRR4	2.4
2481	LGAVHALTT KVHFECTGWHDAEDAGAPL		LRR5	2.2
2509	VYALLLRRC RQGHCEEFCVYKGSLSSYGAV		LRR6	2.8

LPPGFRPHFEVGLAVVVQDQ

2559	LGAAVVALN RSLAITLPEPNGSATGLTVW		LRR1	2.8
2588	LHGLTASVL PGLLRQADPQHVI			LRR2	2.4
2610	EYSLALVTV LNEYERALDVAAEPKHERQHR		LRR3	2

AQIRKNITETLVSLRVHTVDDIQQIAAALAQCMGPSRELVCRSCLKQTLHK

2691	LEAMMLILQ AETTAGTVTPTAIGDSILNIT		LRR1	2.4
2721	GDLIHLASS DVRAPQPSELGAESPSRMVAS		LRR2	2.2

QAYNLTSALMRILMRSRVLNEEPLTLAGEEIVAQGKRSDPRSLLCYGGAPGPGCHFSIPE
AFSGALANLSDVVQLIFLVDSNPFPFGYISNYTVSTKVASMAFQTQAGAQIPIERLASER
AITVKVPNNSDWAARGHRSSANSANSVVVQPQASV

2906	GAVVTLDSS NPAAGLHLQLNYTL		LRR1	2.2
2929	LDGHYLSEE PEPYLAVYLHSEPRPNEHNCS		LRR2	2

ASRRIRPESLQGADHRPYTFFISPGSRDPAGSYHLNLSSHFRWSALQVSVGLYTSLCQYF
SEEDMVWRT

3028	EGLLPLEET SPRQAVCLTRHLTAF		LRR1	2.4
3052	GASLFVPPS HVRFVFPEPTADVNYIVMLTC		LRR2	2.2

AVCLVTYMVMAAILHKLDQLDASRGRAIPFCGQRGRFKYEILVKTGWGRGSGTTAHVGIM
LYGVDSRSGHRHLDGDRAFHRNSLDIFRIATPHSLGSVWKIRVWHDNKGLSPAWFLQHVI
VRDLQTARSAFF

3214	LVNDWLSVE TEANGGLVEKEVLAASDAAL		LRR1	2
3243	LRFRRLLVA ELQRGFFDKHIWLSIWDRPPR		LRR2	2

SRFTRIQRATCCVL

3287	LICLFLGAN AVWYGAVGDSAYSTGHVSR		LRR1	4
3315	LSPLSVDTV AVGLVSSVVVYPVY		LRR2	2.4
3338	LAILFLFRM SRSKVAGSPSPTPAGQQVLDI		LRR3	3

SHIFT -27!

3341	LFLFRMSRS KVAGSPSPTPAGQQVLDIDSC		LRR1	2.2
3371	LDSSVLDSS FLTFSGLHAEQAFVGQM		LRR2	2.4
3397	KSDLFLDDS KSLVCWPSGEGT			LRR3	2.4
3418	LSWPDLLSD PSIVGSNLRQLARGQAGHGLG		LRR4	2

PEEDGFSLASPYSPAKSFSASDED

3472	LIQQVLAEG VSSPAPTQDTHMETDL		LRR1	2
3497	LSSLSSTPG EKTETLALQRLGE			LRR2	2
3519	LGPPSPGLN WEQPQAARLSRTGLVEG		LRR3	2
3545	LRKRLLPAW CASLAHGLSLLLVAVAVAVSG		LRR4	2

SHIFT -17!

3558	AHGLSLLLV AVAVAVSGWVGASFPPG		LRR1	2.4
3584	VSVAWLLSS SASFLASFLGWEPLKVL		LRR2	2.2
3610	LEALYFSLV AKRLHPDEDDTLVESPAVTPV		LRR3	2.4

SARVPRVRPPH

3651	GFALFLAKE EARKVKRLHGMLRSLL		LRR1	2.4
3676	VYMLFLLVT LLASYGDASCHGHAYRLQSAI		LRR2	2.8

KQELHSRAFLAITRSEELWPWMAHVLLPYVHGNQSSPELGPPRLRQVRLQEALYPDPPGP
RVHTCSAAGGFSTSDYDVGWESPHNGSGTWAYS

3799	APDLLGAWS WGSCAVYDSGGY			LRR1	2.2
3820	VQELGLSLE ESRDRLRFLQLHNWLDNRSR		LRR2	2.4
3849	AVFLELTRY SPAVGLHAAVTLRLEFPAAGR		LRR3	2.4

SHIFT -19!

3860	AVGLHAAVT LRLEFPAAGRALAALSVRPFA		LRR1	2.2
3890	LRRLSAGLS LPLLTSVCLLLFAVHFAVAEA		LRR2	2.8

SHIFT -21!

3899	LPLLTSVCL LLFAVHFAVAEARTWHREGR		LRR1	2
3928	WRVLRLGAW ARWLLVALTAATALVR		LRR2	2.4
3953	LAQLGAADR QWTRFVRGRPRRFTSFDQVAQ		LRR3	2.4

LSSAARGLAAS

3994	LLFLLLVKA AQQLRFVRQWSVFGKTLCRA		LRR1	3
4023	LPELLGVTL GLVVLGVAYAQLAILLVSSCV		LRR2	2.4

SHIFT -25!

4028	GVTLGLVVL GVAYAQLAILLVSSCVDSLWS		LRR1	2.4
4058	VAQALLVLC PGTGLSTLCPAESWHLSP		LRR2	2.2
4085	LLCVGLWAL RLWGALRLGAVILRWRYHALR		LRR3	2.4

SHIFT -14!

4101	LGAVILRWR YHALRGELYRPAWEPQDYE		LRR1	2.4
4129	MVELFLRRL RLWMGLSKVKEFRHKVRFEGM		LRR2	2.4

EPLPSRSSRGSKVSPDVPPPSAGSDASHPSTSSSQ

4194	LDGLSVSLG RLGTRCEPEPSRLQAVFEA		LRR1	2.4
4222	LLTQFDRLN QATEDVYQLEQQ			LRR2	2
4243	LHSLQGRRS SRAPAGSSRGPSPGLRPALPS		LRR3	2.8

RLARASRGVDLATGPSRTPLRAKNKVHPSST

>ENSP00000302297

MAGLRARGGPGPGLLALSALGFCLMLQVSAKRPPKTPP

39	CPPSCSCTRDTAFCVDSKAVPRNLPSE		NF	4
66	VISLTLVNA AFSEIQDGAFSHLPL		LRR1	2.4
90	LQFLLLNSN KFTLIGDNAFTGLSH		LRR2	4
114	LQYLFIENN DIWALSKFTFRGLKS		LRR3	3.4
138	LTHLSLANN NLQTLPRDIFRPLDI		LRR4	4
162	LNDLDLRGN				LRR5	4
171	SLNCDCKVKWLVEWLAHTNTTVAPIYCASPPRFQEHKVQDLPLREFDCI		CF1_short	3

SHIFT -39!

181	LVEWLAHTN TTVAPIYCASPPRFQEHK		LRR1	2.8
208	VQDLPLREF DCITTDFVLYQTLAFPAVSAE		LRR2	2.4

PFLYSSDLYLALAQPGVSACTILKWDYVERQLRDYDRIPAPSAVHCKPMVVDSQLYVVVA
QLFGGSYIYHWDPNTTRFTRLQDIDPQRVRKPNDLEAFRIDGDWYFAVADSSKAGATSLY
RWHQNGFYSHQALHPWHRDTDLEFVDGEGKPRLIVSSSSQAPVIYQWSRTQKQFVAQGEV
TQVPDAQAVKHFRAGRDSYLCLSRYIGDSKILRWEGTRFSEVQALPSRGSLALQPFLVGG
RRYLALGSDFSFTQIYQWDEGRQKFVRFQELAVQAPRAFCYMPAGDAQLLLAPSFKGQTL
VYRHIVVDLSA

>ENSP00000349736

MVFPMWT

8	LKRQILILF NIILISKLLGARWFPKTLPC		LRR1	2
37	DVTLDVPKN HVIVDCTDKHLTEIPGGIPTN		LRR2	2.4
67	TTNLTLTIN HIPDISPASFHRLDH		LRR3	3
91	LVEIDFRCN CVPIPLGSKNNMCIKRLQIKP		LRR4	2.8

SHIFT -19!

102	PIPLGSKNN MCIKRLQIKPRSFSGLTY		LRR1	2
129	LKSLYLDGN QLLEIPQGLPPS			LRR2	4
150	LQLLSLEAN NIFSIRKENLTELAN		LRR3	4
174	IEILYLGQN CYYRNPCYVSYSIEKDAFLN		LRR4	3.4
203	LTKLKVLSL KDNNVTAVPTVLPST		LRR5	2.4
227	LTELYLYNN MIAKIQEDDFNNLNQ		LRR6	4
251	LQILDLSGN CPRCYNAPFPCAPCKN		LRR7	4
276	NSPLQIPVN AFDALTELKVLR			LRR8	2.4
297	LHSNSLQHV PPRWFKNINKLQELD		LRR9	2
321	LSQNFLAKE IGDAKFLHFLPS			LRR10	2
342	LIQLDLSFN FELQVYRASMNLSQAFSS		LRR11	4
369	LKSLKILRI RGYVFKELKSFNLSPLHNLQN		LRR12	2.4
399	LEVLDLGTN FIKIANLSMFKQFKR		LRR13	4
423	LKVIDLSVN KISPSGDSSEVGFCSNARTSV		LRR14	3.4

ESYEPQVLEQLHYFRYDKYARSCRFKNKEASFMSVNESCYKY

495	GQTLDLSKN SIFFVKSSDFQHLSF		LRR1	3.4
519	LKCLNLSGN LISQTLNGSEFQPLAE		LRR2	4
544	LRYLDFSNN RLDLLHSTAFEELHK		LRR3	3.4
568	LEVLDISSN SHYFQSEGITHMLNFTKNLKV		LRR4	3.4
598	LQKLMMNDN DISSSTSRTMESES		LRR5	3.4
621	LRTLEFRGN HLDVLWREGDNRYLQLFKNL		LRR6	3.4
650	LKLEELDIS KNSLSFLPSGVFDGMPPN		LRR7	2.4
677	LKNLSLAKN GLKSFSWKKLQCLKN		LRR8	4
701	LETLDLSHN QLTTVPERLSNCSRS		LRR9	4
725	LKNLILKNN QIRSLTKYFLQDAFQ		LRR10	4
749	LRYLDLSSN KIQMIQKTSFPENVLNN		LRR11	4
775	LKMLLLHHN				LRR12	4
784	RFLCTCDAVWFVWWVNHTEVTIPYLATDVTCVGPGAHKGQSVISLDLYTCELD		CF1	4

SHIFT -12!

825	VISLDLYTC ELDLTNLILFSLSI		LRR1	2.8
848	SVSLFLMVM MTASHLYFWDVWYIYHFCKAK		LRR2	2

IKGYQRLISPDCCYDAFIVYDTKDPAVTEWV

909	LAELVAKLE DPREKHFNLCLEERDW		LRR1	2.4
934	LPGQPVLEN LSQSIQLSKKTVFVMTDKYAK		LRR2	2.4

TENFKIAFY

973	LSHQRLMDE KVDVIILIFLEKPFQK		LRR1	2
998	SKFLQLRKR LCGSSVLEWPTNPQAHPYFWQ		LRR2	2

CLKNALATDNHVAYSQVFKETV

>ENSP00000241274

MKPSIAEMLHRGRMLWIILLSTIALGWTTPIPLIEDSEEIDEP

44	CFDPCYCEVKESLFHIHCDSKGFTNISQITEFW		NF	4

SR

79	PFKLYLQRN SMRKLYTNSFLHLNN		LRR1	3
103	AVSINLGNN ALQDIQTGAFNGLKI		LRR2	2.8
127	LKRLYLHEN KLDVFRNDTFLGLES		LRR3	4
151	LEYLQADYN VIKRIESGAFRNLSK		LRR4	3.4
175	LRVLILNDN LIPMLPTNLFKAVS		LRR5	4
198	LTHLDLRGN RLKVLFYRGMLDHIGRS		LRR6	4
224	LMELQLEEN				LRR7	4
233	PWNCTCEIVQLKSWLERIPYTALVGDITCETPFHFHGKDLREIRKTELCPL		CF1_short	3

LSDSEVEASLGIPHSSSSKENAWPTKPSSMLSSVHFTASSVEYKSSNKQPKPTKQPRTPR
PPST

348	SQALYPGPN QPPIAPYQTRPPIPIICPT		LRR1	2
376	GCTCNLHIN DLGLTVNCKERGFNNI		LRR2	2.4
401	SELLPRPLN AKKLYLSSNLIQKIYRSDFWN		LRR3	2
431	FSSLDLLHL GNNRISYVQDGAFINLPN		LRR4	2.4
458	LKSLFLNGN DIEKLTPGMFRGLQS		LRR5	4
482	LHYLYFEFN VIREIQPAAFSLMPN		LRR6	3.4
506	LKLLFLNNN LLRTLPTDAFAGTS		LRR7	4
529	LARLNLRKN YFLYLPVAGVLEHLNA		LRR8	4
554	IVQIDLNEN				LRR9	2.8
563	PWDCTCDLVPFKQWIETISSVSVVGDVLCRSPENLTHRDVRTIELEVLCPE		CF1_short	3

MLHVAPAGESPAQPGDSHLIGAPTSASPYEFSPPGGPVP

653	LSVLILSLL VLFFSAVFVAAG			LRR1	3
674	LFAYVLRRR RKKLPFRSKRQEGVDLTGIQM		LRR2	2

QCHRLFEDGGGGGGGSGGGGRPTLSSPEKAPPVGHVYEYIPHPVTQMCNNPIYKPREEEE
VAVSSAQEAGSAERGGPGTQPPGMGEALLGSEQFAETPKENHSNYRTLLEKEKEWALAVS
SSQLNTIVTVNHHHPHHPAVGGVSGVVGGTGGDLAGFRHHEKNGGVVLFPPGGGCGSGSM
LLDRERPQPAPCTVGFVDCLYGTVPK

910	LKELHVHPP GMQYPDLQQDAR			LRR1	2.4
931	LKETLLFSA GKGFTDHQTQKSDYLELRAKL		LRR2	2

QTKPDYLEVLEKTTYRF

>XP_945666

MAPGPFSSALLSPPPAA

18	LPFLLLLWA GASRGQPCPGRCICQNVAPT		LRR1	3
47	LTMLCAKTG LLFVPPAIDRRVVELRLTDNF		LRR2	2.4

SHIFT -10!

67	VVELRLTDN FIAAVRRRDFANMTS		LRR1	3.4
91	LVHLTLSRN TIGQVAAGAFADLRA		LRR2	4
115	LRALHLDSN RLAEVRGDQLRGLGN		LRR3	4
139	LRHLILGNN QIRRVESAAFDAFLST		LRR4	4
164	VEDLDLSYN NLEALPWEAVGQMVN		LRR5	3.4
188	LNTLTLDHN LIDHIAEGTFVQLHK		LRR6	4
212	LVRLDMTSN RLHKLPPDGLFLRSQGTGPKP		LRR7	3.4

SHIFT -20!

222	LHKLPPDGL FLRSQGTGPKPPTPLTVS		LRR1	2
249	FGGNPLHCN CELLWLRRLTREDDLETCATP		LRR2	2.4

EHLTDRYFWSIPEEEFLCEPPLITRQAGGRALVVEGQAVSLRCRAVGDPEPVVHWVAPDG
RLLGNSSRTRVRGDGTLDVTITTLRDSGTFTCIASNAAGEATAPVEVCVVPLPLMAPPPA
APPPLTEPGSSDIATPGRPGANDSAAERRLVAAELTSNSVLIRWPAQRPVPGIRMYQVQY
NSSVDDSLVYRMIPSTSQTFLVNDLAAGRAYDLCVLAVYDDGATALPATRVVGCVQFTTA
GDPAPCRP

527	LRAHFLGGT MIIAIGGVIVASV			LRR1	2.4
549	LVFIVLLMI RYKVYGDGDSRRVKGSRSLPR		LRR2	2.4

VSHVCSQTNGAGTGAAQAPALPAQDHYEALREVESQAAPAVAVEAKAMEAETASAEPEVV
LGRSLGGSATSLCLLPSEETSGEESRAAVGPRRSRSGALEPPTSA

684	PPTLALVPG GAAARPRPQQRYSFD		LRR1	2
708	GDYGALFQS HSYPRRARRTKRHRSTPHLDG		LRR2	2.2

AGGGAAGEDGDLGLGSARACLAFTSTEWMLESTV

>ENSP00000319223

MRLLVAPLLLAWVAGATAAVPVVPWHVP

29	CPPQCACQIRPWYTPRSSYREATTVDCNDLFLTAVPPALPAG		NF	4
71	TQTLLLQSN SIVRVDQSELGYLAN		LRR1	3
95	LTELDLSQN SFSDARDCDFHALPQ		LRR2	4
119	LLSLHLEEN QLTRLEDHSFAGLAS		LRR3	4
143	LQELYLNHN QLYRIAPRAFSGLSN		LRR4	4
167	LLRLHLNSN LLRAIDSRWFEMLPN		LRR5	4
191	LEILMIGGN KVDAILDMNFRPLAN		LRR6	3.4
215	LRSLVLAGM NLREISDYALEGLQS		LRR7	3
239	LESLSFYDN QLARVPRRALEQVPG		LRR8	3.4
263	LKFLDLNKN PLQRVGPGDFANM			LRR9	4
285	LHLKELGLN NMEELVSIDKFALVNLPE		LRR10	3
312	LTKLDITNN PRLSFIHPRAFHHLPQ		LRR11	3.4
337	METLMLNNN ALSALHQQTVESLPN		LRR12	3.4
361	LQEVGLHGN				LRR13	3.4
370	PIRCDCVIRWANATGTRVRFIEPQSTLCAEPPDLQRLPVREVPFREMTDH		CF1_short	3

SHIFT -2!

418	DHCLPLISP RSFPPSLQVASGESMV		LRR1	2
443	LHCRALAEP EPEIYWVTPAGLRLTPAHAGR		LRR2	2

SHIFT -15!

458	VTPAGLRLT PAHAGRRYRVYP			LRR1	2.2
479	EGTLELRRV TAEEAGLYTCVAQNLVGADTK		LRR2	2

SHIFT -10!

499	AQNLVGADT KTVSVVVGRALLQPGRDE		LRR1	2.2
526	GQGLELRVQ ETHPYHILLSWVTPPNTVSTN		LRR2	2.4

SHIFT -14!

542	LLSWVTPPN TVSTNLTWSSASSLRGQGATA		LRR1	2.4
572	LARLPRGTH SYNITRLLQATEYWACLQVAF		LRR2	2

ADAHTQLACVWARTKEATSCHRALGDRPGLIAILALAVLLLAAGLAAHLGTGQPRKGVGG
RRPLPPAWAFWGWSAPSVRVV

683	SAPLVLPWN PGRKLPRSSEGE			LRR1	3
704	TLLPPLSQN S			LRR2	2


>ENSP00000246047

MISAAWSIFLIGTKIGLFLQVAPLSVMAKS

31	CPSVCRCDAGFIYCNDRFLTSIPTGIPED		NF	4
60	ATTLYLQNN QINNAGIPSDLKNLLK		LRR1	3.4
85	VERIYLYHN SLDEFPTNLPKY			LRR2	2.8
106	VKELHLQEN NIRTITYDSLSKIPY		LRR3	3.4
130	LEELHLDDN SVSAVSIEEGAFRDSNY		LRR4	4
156	LRLLFLSRN HLSTIPWGLPRT			LRR5	4
177	IEELRLDDN RISTISSPSLQGLTS		LRR6	3.4
201	LKRLVLDGN LLNNHGLGDKVFFNLVN		LRR7	4
227	LTELSLVRN SLTAAPVNLPGTN			LRR8	4
249	LRKLYLQDN HINRVPPNAFSYLRQ		LRR9	4
273	LYRLDMSNN NLSNLPQGIFDDLDN		LRR10	3.4
297	ITQLILRNN				LRR11	3.4
306	PWYCGCKMKWVRDWLQSLPVKVNVRGLMCQAPEKVRGMAIKDLNAELFDCK		CF1_short	3

SHIFT -60!

297	ITQLILRNN PWYCGCKMKWVRDW		LRR11	3.4
320	LQSLPVKVN VRGLMCQAPEKVRGMAIKDLN		LRR12	3.4

AELFDCKDSGIVSTIQITTAIPNTVYPAQGQWPAPVTKQPDIKNPKLTKDHQTTGSPSRK
TITITVKSVTSDTIHISWKLALPMTALRLSWLKLGHSPAFGSITETIVTGERSEYLVTAL
EPDSPYKVCMVPMETSNLYLFDETPVCIETETAPLRMYNPTTTLNREQEKEPYK

524	NPNLPLAAI IGGAVALVTIALLA		LRR1	2
547	LVCWYVHRN GSLFSRNCAYSKGRRRKDDYA		LRR2	2.8

EAGTKKDNSILEIRETSFQMLPISNEPISKEEFVIHTIFPPNGMNLYKNNHSESSSNRSY
RDSGIPDSDHSHS

>ENSP00000266719

MAGTICFIMWVLFITDTVWSRSVRQVYEVHDSDDWTIHDFE

42	CPMECFCPPSFPTALYCENRGLKEIPAIPSR		NF	4
73	IWYLYLQNN LIETIPEKPFENATQ		LRR1	3.4
97	LRWINLNKN KITNYGIEKGALSQLKK		LRR2	3.4
123	LLFLFLEDN ELEEVPSPLPRS			LRR3	4
144	LEQLQLARN KVSRIPQGTFSNLEN		LRR4	4
168	LTLLDLQNN KLVDNAFQRDTFKGLKN		LRR5	4
194	LMQLNMAKN ALRNMPPRLPAN			LRR6	3.4
215	TMQLFLDNN SIEGIPENYFNVIPK		LRR7	3
239	VAFLRLNHN KLSDEGLPSRGFDVSS		LRR8	3.4
264	ILDLQLSHN QLTKVPRISAHLQHLHLDHNK		LRR9	3.4

IKSVNVSVICPSPSMLPAERDSFSYGPHLRYLRLDGNEIKPPIPMALMTCFRLLQAVII

>ENSP00000266718

MSLSAFTLFLALIGGTSGQYYDYDFPLSIYGQSSPN

37	CAPECNCPESYPSAMYCDELKLKSVPMVPPG		NF	4
68	IKYLYLRNN QIDHIDEKAFENVTD		LRR1	3.4
92	LQWLILDHN LLENSKIKGRVFSKLKQ		LRR2	4
118	LKKLHINHN NLTESVGPLPKS			LRR3	3.4
139	LEDLQLTHN KITKLGSFEGLVN			LRR4	4
161	LTFIHLQHN RLKEDAVSAAFKGLKS		LRR5	3.4
186	LEYLDLSFN QIARLPSGLPVS			LRR6	4
207	LLTLYLDNN KISNIPDEYFKRFNA		LRR7	4
231	LQYLRLSHN ELADSGIPGNSFNVSS		LRR8	4
256	LVELDLSYN KLKNIPTVNENLENYY		LRR9	4
281	LEVNQLEKF DIKSFCKILGPLSYSK		LRR10	2
306	IKHLRLDGN RISETSLPPDMYECLRVANEV		LRR11	3.4

TLN

>ENSP00000302621

MSSWSRQRPKSPGGIQPHVSRTLFLLLLLAASAWGVTLSPKDCQVFRSDHGSSISCQPPA
EIPGYLPADTVHLAVEF

78	FNLTHLPAN LLQGASKLQELH			LRR1	2.4
99	LSSNGLESL SPEFLRPVPQLRVLD		LRR2	2
123	LTRNALTGL PPGLFQASATLDTLVLKENQ		LRR3	2
152	LEVLEVSWL HGLKALGHLDLSGNRLRK		LRR4	2.4
179	LPPGLLANF TLLRTLDLGENQLETLPPD		LRR5	2.4
207	LLRGPLQLE RLHLEGNKLQVL			LRR6	2.4
228	GKDLLLPQP DLRYLFLNGNKLARVAAGAFQ		LRR7	2.4

SHIFT -25!

233	LPQPDLRYL FLNGNKLARVAAGAFQGLRQ		LRR1	2
262	LDMLDLSNN SLASVPEGLWASLGQPNWDMR		LRR2	4

SHIFT -13!

279	LWASLGQPN WDMRDGFDISGNPWICDQN		LRR1	2.4
307	LSDLYRWLQ AQKDKMFSQNDTRCAGPEAVK		LRR2	2

GQTLLAVAKSQ

>XP_944870

MTCWLCVLSLPLLLLPAAPPPAGG

25	CPARCECTVQTRAVACTRRRLTAVPDGIPAE		NF	4
56	TRLLELSRN RIRCLNPGDLAALPA		LRR1	3
80	LEELDLSEN AIAHVEPGAFANLPR		LRR2	4
104	LRVLRLRGN QLKLIPPGVFTRLDN		LRR3	4
128	LTLLDLSEN KLVILLDYTFQDLHS		LRR4	4
152	LRRLEVGDN DLVFVSRRAFAGLLA		LRR5	3.4
176	LEELTLERC NLTALSGESLGHLRS		LRR6	3.4
200	LGALRLRHL AIASLEDQNFRRLPG		LRR7	3
224	LLHLEIDNW PLLEEVAAGSLRGLN		LRR8	2.4
248	LTSLSVTHT NITAVPAAALRHQAH		LRR9	2.8
272	LTCLNLSHN PISTVPRGSFRDLVR		LRR10	4
296	LRELHLAGA LLAVVEPQAFLGLRQ		LRR11	3
320	IRLLNLSNN LLSTLEESTFHSVNT		LRR12	3.4
344	LETLRVDGN				LRR13	3.4
353	PLACDCRLLWIVQRRKTLNFDGRLPACATPAEVRGDALRNLPDSVLFEY		CF1_short	3

FVCRKPKIRERRLQRVTATAGEDVRFLCRAEGEPAPTVAWVTPQHRPVTATSAGRARVLP
GGTLEIQDARPQDSGTYTCVASNAGGNDTYFATLTVRPEPAANRTPGEAHNET

515	LAALRAPLD LTTILVSTAMGCITFLGVV		LRR1	2.4
543	LFCFVLLFV WSRGRGQHKNNFSVEYSFRKV		LRR2	2.4

DGPAAAAGQGGARKFNMKMI

>ENSP00000319464

MLPGAWLLW

10	TSLLLLARP AQP		LRR1	2
22	CPMGCDCFVQEVFCSDEELATVPLDIPPY		NF	4

SHIFT -11!

40	LATVPLDIP PYTKNIIFVETSFTT		LRR2	2.4
64	LETRAFGSN PNLTKVVFLNTQLCQFRPDAF		LRR3	2.4

SHIFT -19!

75	LTKVVFLNT QLCQFRPDAFGGLPR		LRR1	2.2
99	LEDLEVTGS SFLNLSTNIFSNLTS		LRR2	2.8
123	LGKLTLNFN MLEALPEGLFQHLAA		LRR3	4
147	LESLHLQGN QLQALPRRLFQPLTH		LRR4	4
171	LKTLNLAQN LLAQLPEELFHPLTS		LRR5	4
195	LQTLKLSNN ALSGLPQGVFGKLGS		LRR6	4
219	LQELFLDSN NISELPPQVFSQLFC		LRR7	4
243	LERLWLQRN AITHLPLSIFASLGN		LRR8	4
267	LTFLSLQWN MLRVLPAGLFAHTPC		LRR9	4
291	LVGLSLTHN QLETVAEGTFAHLSN		LRR10	4
315	LRSLMLSYN AITHLPAGIFRDLEE		LRR11	4
339	LVKLYLGSN NLTALHPALFQNLSK		LRR12	4
363	LELLSLSKN QLTTLPEGIFDTNYN		LRR13	4
387	LFNLALHGN				LRR14	4
396	PWQCDCHLAYLFNWLQQYTDRLLNIQTYCAGPAYLKGQVVPALNEKQLVCP		CF1_short	3

VTRDHLGFQVTWPDESKAGGSWDLAVQERAARSQCTYSNPEGTVVLACDQAQCRWLNVQL
SPWQGSLGLQYNASQEWDLRSSCGSLRLTVSIEARAAGP

>ENSP00000266058

MALTPGWGSSAGPVRPELWLLLWAAAWRLGASA

34	CPALCTCTGTTVDCHGTGLQAIPKNIP		NF	4
61	RNTERLELN GNNITRIHKNDFAGLKQ		LRR1	2
87	LRVLQLMEN QIGAVERGAFDDMKE		LRR2	4
111	LERLRLNRN QLHMLPELLFQNNQA		LRR3	4
135	LSRLDLSEN AIQAIPRKAFRGATD		LRR4	4
159	LKNLQLDKN QISCIEEGAFRALRG		LRR5	4
183	LEVLTLNNN NITTIPVSSFNHMPK		LRR6	4
207	LRTFRLHSN				LRR7	3.4
216	HLFCDCHLAWLSQWLRQRPTIGLFTQCSGPASLRGLNVAEVQKSEFSCS		CF1_short	3

SHIFT -42!

223	LAWLSQWLR QRPTIGLFTQCSGPAS		LRR1	2
248	LRGLNVAEV QKSEFSCSGQGEAGRVPTCTL		LRR2	2.4

SSGS

282	CPAMCTCSNGIVDCRGKGLTAIPANLPET		NF	4
311	MTEIRLELN GIKSIPPGAFSPYRK		LRR1	2.8
335	LRRIDLSNN QIAEIAPDAFQGLRS		LRR2	3.4
359	LNSLVLYGN KITDLPRGVFGGLYT		LRR3	4
383	LQLLLLNAN KINCIRPDAFQDLQN		LRR4	4
407	LSLLSLYDN KIQSLAKGTFTSLRA		LRR5	4
431	IQTLHLAQN				LRR6	3.4
440	PFICDCNLKWLADFLRTNPIETSGARCASPRRLANKRIGQIKSKKFRCS		CF1_short	3

AKEQYFIPGTE

500	DYQLNSECN SDVVCPHKCRCEANVVE		LRR1	2
526	CSSLKLTKI PERIPQSTAELRLNNNEISIL		LRR2	2

SHIFT -16!

540	QSTAELRLN NNEISILEATGMFKKLTH		LRR1	2.4
567	LKKINLSNN KVSEIEDGAFEGAAS		LRR2	3.4
591	VSELHLTAN QLESIRSGMFRGLDG		LRR3	3.4
615	LRTLMLRNN RISCIHNDSFTGLRN		LRR4	4
639	VRLLSLYDN QITTVSPGAFDTLQS		LRR5	3.4
663	LSTLNLLAN				LRR6	4
672	PFNCNCQLAWLGGWLRKRKIVTGNPRCQNPDFLRQIPLQDVAFPDFRCE		CF1_short	3

SHIFT -42!

679	LAWLGGWLR KRKIVTGNPRCQNPDF		LRR1	2.4
704	LRQIPLQDV AFPDFRCEEGQEEGGCLPRPQ		LRR2	2.4


734	CPQECACLDTVVRCSNKHLRALPKGIPKN		NF	4

SHIFT -22!

741	LDTVVRCSN KHLRALPKGIPKN			LRR1	2.4
763	VTELYLDGN QFTLVPGQLSTFKY		LRR2	3.4
786	LQLVDLSNN KISSLSNSSFTNMSQ		LRR3	3.4
810	LTTLILSYN ALQCIPPLAFQGLRS		LRR4	4
834	LRLLSLHGN DISTLQEGIFADVTS		LRR5	4
858	LSHLAIGAN				LRR6	3.4
867	PLYCDCHLRWLSSWVKTGYKEPGIARCAGPQDMEGKLLLTTPAKKFECQ		CF1_short	3

SHIFT -42!

874	LRWLSSWVK TGYKEPGIARCAGPQDM		LRR1	2
900	EGKLLLTTP AKKFECQGPPTLAVQA		LRR2	2
925	KCDLCLSSP CQNQGTCHNDPLEVYRCACPS		LRR3	2

GYKGRDCEVSLDSCSSGPCENGGTCHAQEGEDAPFTCSCPTGFEGPTCGVNTDDCVDHAC
ANG

1018	GVCVDGVGN YTCQCPLQYEGKACEQLVD		LRR1	2.2
1046	LCSPDLNPC QHEAQCVGTPDGPRCECMPGY		LRR2	2.4

AGDNCSENQDDCRDHRCQNGAQ

1098	CMDEVNSYSCLCAEGYSGQLCEIPPHLPAPKSPCEG		NF	4

TECQNGANCVDQGNRPVCQC

1154	LPGFGGPEC EKLLSVNFVDRDTY		LRR1	2.2
1177	LQFTDLQNW PRANITLQVSTA			LRR2	2
1198	EDNGILLYN GDNDHIAVELYQGHVRVSYDP		LRR3	2.4

GSYPSSAIYSAETINDGQFHTVELVAFDQMVNLSIDGGSPMTMDNFGKHYTLNSEAPLY

1287	VGGMPVDVN SAAFRLWQILNGTGFH		LRR1	2.2
1312	GCIRNLYIN NELQDFTKTQMKPGVVPGCEP		LRR2	2.4

CRKLYCLHGICQPNATPGPMCHCEAGWVGLHCDQPADGPCHGHKCVHGQCVPLDALSYSC
QCQDGYSGALCNQAGALAEPCRGLQCLHGHCQASGTKGAHCVCDPGFSGELCEQESECRG
DPVRDFHQVQRGYAICQTTRPLSWVECRGSCPGQGCCQGLRLKRRKFTFECSDGTSFAEE
VEKPTKCGCALCA

>GENSCAN00000039828

MIKWLMTAKTHKHVESVRDLQILPFRLPVK

31	LQKLDCSNN LIQRVTAQDFQDLQD		LRR1	3
55	LKHLILDNN NASFFEAGALQRCSQ		LRR2	4
79	LSNLALEQN LLLSIPLRLPGT			LRR3	4
100	LTRLDLKSN VIQNIAEREIKDLKQ		LRR4	4
124	LHVLNLRNN KISALDLKALEGLPH		LRR5	4
148	LRHLYLDGN PWNCTFSLLKAREVLMAKGTD		LRR6	4

VRGGQCAAPTEQHGESWMSSKEIMRQCKHHFHLTEKSKETKKKSKPEDPSSIRINMDDG

>ENSP00000275030

MPGGCSRGPAAGDGRLRLARLALVLLGWVSSSSPTSSASSFSSSAPFLASAVSAQPPLPD
Q

62	CPALCECSEAARTVKCVNRNLTEVPTDLPAY		NF	4
93	VRNLFLTGN QLAVLPAGAFARRPPLAE		LRR1	3.4
120	LAALNLSGS RLDEVRAGAFEHLPS		LRR2	3.4
144	LRQLDLSHN PLADLSPFAFSGSNASVSAP		LRR3	4
173	SPLVELILN HIVPPEDERQNRSFEGMVVAA		LRR4	2.4

SHIFT -28!

175	LVELILNHI VPPEDERQNRSFEGMVVAAL		LRR1	3
204	LAGRALQGL RRLELASNHFLY			LRR2	2
225	LPRDVLAQL PSLRHLDLSNNS			LRR3	2
246	LVSLTYVSF RNLTHLESLHLE			LRR4	2
267	DNALKVLHN GTLAELQGLPHIRVFLDNNPW		LRR5	2.4

VCDCHMADMVTWLKETEVVQGKDRLTCAYPEKM

330	RNRVLLELN SADLDCDPILPPSLQTSYVFL		LRR1	2.4
360	GIVLALIGA IFLLVLYLNRKGIKKWMHNIR		LRR2	2.4

DACRDHMEGYHYRYEINADPRLTNLSSNSDV

>ENSP00000310126

MLHTAISCWQPFLGLAVVLIFMGSTIG

28	CPARCECSAQNKSVSCHRRRLIAIPEGIPIE		NF	4
59	TKILDLSKN RLKSVNPEEFISYPL		LRR1	3
83	LEEIDLSDN IIANVEPGAFNNLFN		LRR2	3.4
107	LRSLRLKGN RLKLVPLGVFTGLSN		LRR3	4
131	LTKLDISEN KIVILLDYMFQDLHN		LRR4	3.4
155	LKSLEVGDN DLVYISHRAFSGLLS		LRR5	3.4
179	LEQLTLEKC NLTAVPTEALSHLRS		LRR6	3.4
203	LISLHLKHL NINNMPVYAFKRLFH		LRR7	3
227	LKHLEIDYW PLLDMMPANSLYGLN		LRR8	2.4
251	LTSLSVTNT NLSTVPFLAFKHLVY		LRR9	2.8
275	LTHLNLSYN PISTIEAGMFSDLIR		LRR10	4
299	LQELHIVGA QLRTIEPHSFQGLRF		LRR11	2.4
323	LRVLNVSQN LLETLEENVFSSPRA		LRR12	3.4
347	LEVLSINNN				LRR13	3.4
356	PLACDCRLLWILQRQPTLQFGGQQPMCAGPDTIRERSFKDFHSTALSFY		CF1_short	3

FTCKKPKIREKKLQHLLVDEGQTVQLECSADGDPQPVISWVTPRRRFITTKSNGRATVLG
DGTLEIRFAQDQDS

479	GMYVCIASN AAGNDTFTASLTVKGF		LRR1	2.2
504	ASDRFLYAN RTPMYMTDSNDTISNGTNAN		LRR2	2.4
533	TFSLDLKTI LVSTAMGCFTFLGVV		LRR3	2
557	LFCFLLLFV WSRGKGKHKNSID			LRR4	2.4
579	LEYVPRKNN GAVVEGEVAGPRRFNMKMI		LRR5	2.4


>BC104037.1

MP

3	LLRTLDLHN NKITSVPNEALRYLKN		LRR1	2
28	LAYLDLSSN RLTTLTPDFLENWTHLVSTP		LRR2	4
57	SGVLDLSPS RIILGLQDNPWFCDCHISKMI		LRR3	2.4

ELSKVVDPAIVLLDPLMTCSEPERLTGIL

116	FQRAELEHC LKPSVMTSATKIMSA		LRR1	2.2
140	LGSNVLLRC DATGFPTPQITWTRSDSSPVN		LRR2	2.4

YTVIQESPEEGVRWSIMSLTGISSKDAGDYKCKAKNLAGMSEAVVTVTVLGITTTPIPPD
TSERTGDHPEWDVQPGSGRSTSVSSASSYLWSSSFSPTSSFSASTLSPPSTASFSLSPFS
SSTVSSTTTLSTSISASTTMANKRSFQLHQGG

322	KRNLKVAKN GSKLPPASTSKKEELAL		LRR1	2.4
348	LDQTMLTET NATIENLRVVSET			LRR2	2.4
370	KESVTLMWN MINTTHNSAVTVLYSKYGGKD		LRR3	2.4
400	LLLLNADSS KNQVTIDGLEPGGQYMACVCP		LRR4	2.8

KGVPPQKDQCITFSTERVEGDDSQWSLLLVVTSTACVVILPLICFLLYKVCKLQCKSEPF
WEDDLAKETYIQFETLFPRSQSVGELWTRSHRDDSEKLLLCSRSSVESQVTFKSEGSRPE
YYC

>ENSP00000043402

MKRASAGGSRLLAWVLWLQAWQVAAP

27	CPGACVCYNEPKVTTSCPQQGLQAVPVGIPAA		NF	4
59	SQRIFLHGN RISHVPAASFRACRN		LRR1	2.4
83	LTILWLHSN VLARIDAAAFTGLAL		LRR2	4
107	LEQLDLSDN AQLRSVDPATFHGLGR		LRR3	4
132	LHTLHLDRC GLQELGPGLFRGLAA		LRR4	3.4
156	LQYLYLQDN ALQALPDDTFRDLGN		LRR5	4
180	LTHLFLHGN RISSVPERAFRGLHS		LRR6	4
204	LDRLLLHQN RVAHVHPHAFRDLGR		LRR7	4
228	LMTLYLFAN NLSALPTEALAPLRA		LRR8	4
252	LQYLRLNDN				LRR9	4
261	PWVCDCRARPLWAWLQKFRGSSSEVPCSLPQRLAGRDLKRLAANDLQGC		CF1_short	3

SHIFT -58!

252	LQYLRLNDN PWVCDCRARPLWAW		LRR9	4
275	LQKFRGSSS EVPCSLPQRLAGRD		LRR10	2.2
298	LKRLAANDL QGCAVATGPYHPIWTGRATD		LRR11	2.4
327	EEPLGLPKC CQPDAADKASVLEPGRPASAG		LRR12	2.4

NALKGRVPPGDSPPGNGSGPRHINDSPFGTLPGSAEPPLTAVRPEGSEPPGFPTSGPRRR
PGCSRKNRTRSHCRLGQAGSGGGGTGDSEGSGALPSLTCSLTPLGLALVLWTVLGPC

>ENSP00000335397

M

2	LPGLRRLLQ APASACLLLMLL			LRR1	2
23	ALPLAAPSC PMLCTCYSSPPTVSCQANNFS		LRR2	2.2
31	CPMLCTCYSSPPTVSCQANNFSSVPLSLPPS		NF	4

SHIFT -9!

53	SVPLSLPPS TQRLFLQNNLIRT			LRR3	2.4
75	LRPGTFGSN LLTLWLFSNNLSTIYPGTFRH		LRR4	2.8
105	LQALEELDL GDNRHLRSLEPDTFQGLER		LRR5	2
133	LQSLHLYRC QLSSLPGNIFRGLVS		LRR6	3.4
157	LQYLYLQEN SLLHLQDDLFADLAN		LRR7	4
181	LSHLFLHGN RLRLLTEHVFRGLGS		LRR8	4
205	LDRLLLHGN RLQGVHRAAFRGLSR		LRR9	4
229	LTILYLFNN SLASLPGEALADLPS		LRR10	4
253	LEFLRLNAN				LRR11	4
262	PWACDCRARPLWAWFQRARVSSSDVTCATPPERQGRDLRALREADFQAC		CF1_short	3

PPAAPTRPGSRARGNSSSNHLYGVAEAGAPPADPSTLYRDLPAEDSRGRQGGDAPTEDDY
WGGYGGEDQRGEQMCPGAACQAPPDSRGPALSAGLPSPLLCLLLLVPHHL

>ENSP00000355396

MAPAPLGVPEEQLLGCRSRVLSRLLFIAQTALLLLPAAGAGL

43	CPAPCSCRIPLLDCSRRKLPAPSWRA		NF	4
69	LSGLLPPDT AILDFSHNRLSNWNISLESQT		LRR1	2.4
99	LQEVKMNYN ELTEIPYFGEPTSN		LRR2	2.8
122	ITLLSLVHN IIPEINAQALQFYPA		LRR3	3.4
146	LESLDLSSN IISEIKTSSFPRMQ		LRR4	4
169	LKYLNLSNN RITTLEAGCFDNLSSS		LRR5	4
194	LLVVKLNRN RMSMIPPKIFKLPH		LRR6	3.4
217	LQFLELKRN RIKIVEGLTFQGLDS		LRR7	4
241	LRSLKMQRN GISKLKDGAFFGLNN		LRR8	3.4
265	MEELELEHN NLTRVNKGWLYGLRM		LRR9	3.4
289	LQQLYVSQN AIERISPDAWEFCQR		LRR10	3.4
313	LSELDLSYN QLTRLDESAFVGLSL		LRR11	4
337	LERLNLGDN RVTHIADGVFRFLSN		LRR12	4
361	LQTLDLRNN EISWAIEDASEAFAGLTS		LRR13	4
388	LTKLILQGN QIKSITKKAFIGLES		LRR14	4
412	LEHLDLNNN AIMSIQENAFSQTH		LRR15	4
435	LKELILNTS				LRR16	3.4
444	SLLCDCHLKWLLQWLVDNNFQHSVNVSCAHPEWLAGQSILNVDLKDFVCD		CF1_short	3

DFLKPQIRTHPETIIALRGMNVTLTCTAVSSSDSPMSTVWRKDSEILYDVDTENFVRYWQ
QAGEALEY

562	TSILHLFNV NFTDEGKYQCIVTNHFGSNY		LRR1	2
591	SQKAKLTVN EMPSFLKTPMDLTIRTGAMAR		LRR2	2.4

LECAAEGHPAPQISWQKDGGTDFPAARERRMHVMPEDDVFFIANVKIEDMGIYSCMAQNT
AGGLSANASLTVLETPSFIRPLEDKTVTRGETAVLQCIAGGSPAPRLNWTKDDGPLLVTE
RHFFAAANQLLIIVDAGLEDAGKYTCIMSNTLGTERGHIYLNVISSPNCDSSQSSIGHED
DGWTTVGIVIIVVVCCVVGTSLIWVIVIYHMRRKNEDYSITNTEELNLPADIPSYLSSQG
TLSEPQEGYSNSEAGSHQQLMPPANGYIHKGTDGGTGTRVICSDCYDNANIYSRTREYCP
YTYIAEEDVLDQT

934	LSSLMVQMP KETYLVHPPQDTTA		LRR1	2.4
957	LESLIPSAN REPSAFPTNHERISEKKLPST		LRR2	3

QMSGET

993	LQRPVWNIN RELGLPHPPFSQQPV		LRR1	2.4
1017	HESPQLHQN EGLAGREPDCSASSMSCHRLQ		LRR2	2

DHAFDFSRTRNIQDGSEGT

>ENSP00000334374

MLSGVWF

8	LSVLTVAGI LQTESRKTAKDI		LRR1	2.4
29	CKIRCLCEEKENVLNINCENKGFTTVSLLQPPQ		NF	4

SHIFT -25!

37	EKENVLNIN CENKGFTTVSLLQPPQYR		LRR2	2
64	IYQLFLNGN LLTRLYPNEFVNYSN		LRR3	3.4
88	AVTLHLGNN GLQEIRTGAFSGLKT		LRR4	3.4
112	LKRLHLNNN KLEILREDTFLGLES		LRR5	4
136	LEYLQADYN YISAIEAGAFSKLNK		LRR6	3.4
160	LKVLILNDN LLLSLPSNVFRFVL		LRR7	4
183	LTHLDLRGN RLKVMPFAGVLEHIGG		LRR8	4
208	IMEIQLEEN				LRR9	2.8
217	PWNCTCDLLPLKAWLDTITVFVGEIVCETPFRLHGKDVTQLTRQDLCPR		CF1_short	3

SHIFT -9!

257	LTRQDLCPR KSASDSSQRGSHADTH		LRR1	2
282	VQRLSPTMN PALNPTRAPKASRPPKMRNRP		LRR2	2.4

TPRVTVSKDRQSFGPIMVYQTKSPVPLT

340	CPSSCVCTSQSSDNGLNVNCQERKFTNISDLQPKP		NF	4

SHIFT -25!

350	SSDNGLNVN CQERKFTNISDLQPKPTS		LRR1	2
377	PKKLYLTGN YLQTVYKNDLLEYSS		LRR2	3
401	LDLLHLGNN RIAVIQEGAFTNLTS		LRR3	4
425	LRRLYLNGN YLEVLYPSMFDGLQS		LRR4	4
449	LQYLYLEYN VIKEIKPLTFDALIN		LRR5	4
473	LQLLFLNNN LLRSLPDNIFGGTA		LRR6	4
496	LTRLNLRNN HFSHLPVKGVLDQLPA		LRR7	4
521	FIQIDLQEN				LRR8	2.8
530	PWDCTCDIMGLKDWTEHANSPVIINEVTCESPAKHAGEILKFLGREAICPD		CF1_short	3

SHIFT -41!

540	LKDWTEHAN SPVIINEVTCESPAKHAGEI		LRR1	2.4
569	LKFLGREAI CPDSPNLSDGTVLSMNHNTDT		LRR2	2

PRSLSVSPSSYPELHTEVP

618	LSVLILGLL VVFILSVCFGAG			LRR1	3
639	LFVFVLKRR KGVPSVPRNTNNLDVSSFQLQ		LRR2	2.4

YGSYNTETHDKTDGHVYNYIPPPVGQMCQNPIYMQKEGDPVAYYRNLQEFSYSNLEEKKE
EPATPAYTISATELLEKQATPREPELLYQNIAERVKELPSAGLVHYNFCTLPKRQFAPSY
ESRRQNQDRINKTVLYGTPRKCFVGQSKPNHPLLQAKPQSEPDYLEVLEKQTAISQL

>ENSP00000217939

MPKRAHWGALSVVLILLWGHPRVALA

27	CPHPCACYVPSEVHCTFRSLASVPAGIAKH		NF	4
57	VERINLGFN SIQALSETSFAGLTK		LRR1	2.8
81	LELLMIHGN EIPSIPDGALRDLSS		LRR2	3.4
105	LQVFKFSYN KLRVITGQTLQGLSN		LRR3	2.8
129	LMRLHIDHN KIEFIHPQAFNGLTS		LRR4	3.4
153	LRLLHLEGN LLHQLHPSTFSTFTF		LRR5	4
177	LDYFRLSTI RHLYLAENMVRTLPASM		LRR6	2.4
203	LRNMPLLEN LYLQGNPWTCDCEMRWFLEWD		LRR7	3.4

AKSRGILKCKKDKAYEGGQLCAMCFSPKKLYKHEIHKLKDMTCLKP

279	SIESPLRQN RSRSIEEEQEQEEDG		LRR1	2
303	GSQLILEKF QLPQWSISLNMTDEHGNMVNL		LRR2	2.4

SHIFT -20!

313	LPQWSISLN MTDEHGNMVNLVCDIKKPMD		LRR1	2.8
342	VYKIHLNQT DPPDIDINATVALDFECPMTR		LRR2	2.2

ENYEKLWKLIAYYSEVPVKLHRELMLSKDPRVSYQYRQDADEEALYYTGVRAQILAEPEW
VMQPSIDIQLNRRQSTAKKVLLSYYTQYSQTISTKDTRQARGRSWVMIEPSGAVQRDQTV
LEGGPCQLSCNVKASESPSIFWVLPDGSILKAPMDDPDSKFSILSSGWLRIKSMEPSDSG
LYQCIAQVRDEMDRMVYRVLVQSPSTQPAEKDTVTIGKNPGESVTLPCNALAIPEAHLSW
ILPNRRIINDLANTSHVYMLPNGTLSIPKVQVSDSGYYRCVAVNQQGADHFTVGITVTKK
GSGLPSKRGRRPGAKALSRVREDIVEDEGGSGMGDEENTSRRLLHPKDQEVFLKTKDDAI
NGDKKAKKGRRKLKLWKHSEKEPETNVAEGRRVFESRRRINMANKQINPERWADILAKVR
GKNLPKGTEVPPLIKTTSPPSLSLEVTPPFPAISPPSASPVQTVTSAEESSADVPLLGEE
EHVLGTISSASMGLEHNHNGVILVEPEVTSTPLEEVVDDLSEKTEEITSTEGDLKGTAAP
TLISEPYEPSPTLHTLDTVYEKPTHEETATEGWSAADVGSSPEPTSSEYEPPLDAVSLAE
SEPMQYFDPDLETKSQPDEDKMKEDTFAHLTPTPTIWVNDSSTSQLFEDSTIGEPGVPGQ
SHLQGLTDNIH

1043	LVKSSLSTQ DTLLIKKGMKEMSQTLQG		LRR1	2
1070	GNMLEGDPT HSRSSESEGQESKSIT		LRR2	2.2
1095	LPDSTLGIM SSMSPVKKPAETTVGTLLDKD		LRR3	2

TTTATTTPRQKVAPSSTMSTHPSRRRPNGRRRLRPNKFRHRHKQTPPTTFAPSETFSTQP
TQAPDIKISSQVESSLVPTAWVDNTVNTPKQLEMEKNAEPTSKGTPRRKHGKRPNKHRYT
PSTVSSRASGSKPSPSPENKHRNIVTPSSETILLPRTVSLKTEGPYDSLDYMTTTRKIYS
SYPKVQETLPVTYKPTSDGKEIKDDVATNVDKHKSDILVTGESITNAIPTSRSLVSTMGE
FKEESSPVGFPGTPTWNPSRTAQPGRLQTGIPVTTSGENLTDPPLLKELEDVDFTSEFLS
SLTVSTPFHQEEAGSSTTLSSIKVEVASSQAETTTLDQDHLETTVAI

1472	LLSETRPQN HTPTAARMKEPASSSPSTI		LRR1	2
1500	LMSLGQTTT TKPALPSPRISQASRDSKE		LRR2	2.4
1528	NVFLNYVGN PETEATPVNNEGTQHMSGPNE		LRR3	2

LSTPSSDQDAFNLSTKLELEKQVFGSRSLPRGPDSQRQDGRVHASHQLTRVPAKPILPTA
TVRLPEMSTQSASRYFVTSQSPRHWTNKPEITTYPSGALPENKQFTTPRLSSTTIPLPLH
MSKPSIPSKFTDRRTDQFNGYSKVFGNNNIPEARNPVGKPPSPRIPHYSNGRLPFFTNKT
LSFPQLGVTRRPQIPTSPAPVMRERKVIPGSYNRIHSHSTFHLDFGPPAPPLLHTPQTTG
SPSTNLQNIPMVSSTQSSISFITSSVQSSGSFHQSSSKFFAGGPPASKFWSLGEKPQILT
KSPQTVSVTAETDTVFPCEATGKPKPFVTWTKVSTGALMTPNTRIQRFEV

1908	LKNGTLVIR KVQVQDRGQYMCTASNLHG		LRR1	2.4
1936	LDRMVVLLS VTVQQPQILASHYQDVTVYLG		LRR2	2.2

DTIAMECLAKGTPAPQISWIFPDRRVWQTVSPVEGRITLHENRTLSIKEASFSDRGVYKC
VASNAAGADSLAIRLHVAALPPVIHQEKLENISLPPGLSIHIHCTAKAAPLPSVRWVLGD
GTQIRPSQFLHGNLFVFPNGT

2107	LYIRNLAPK DSGRYECVAANLVGSARRTVQ		LRR1	2
2137	LNVQRAAAN ARITGTSPRRTDVRY		LRR2	2.4
2161	GGTLKLDCS ASGDPWPRILWRLPSKRMIDA		LRR3	2.8

LFSFDSRIKVFANGTLVVKSVTDKDAGDYLCVARNKVGDDYVVLKVDVVMKPAKIEHKEE
NDHKVFYGGDLKVDCVATGLPNPEISWS

2279	LPDGSLVNS FMQSDDSGGRTKRYVV		LRR1	2.8
2304	FNNGTLYFN EVGMREEGDYTCFAENQVGKD		LRR2	2.8

EMRVRVKVVTAPATIRNKTYLAVQVPYGDVVTVACEAKGEPMPKVTWLSPTNKVIPTSSE
KYQIYQDGTLLIQKAQRSDSGNYTCLVRNSAGEDRKTVWIHVNVQPPKINGNPNPITTVR
EIAAGGSRKLIDCKAEGIPTPRVLWAFPEGVVLPAPYY

2492	GNRITVHGN GSLDIRSLRKSDS			LRR1	2.2
2514	VQLVCMARN EGGEARLILQLTVLEPMEKPI		LRR2	2.2

SHIFT -16!

2528	RLILQLTVL EPMEKPIFHDPISEKITAMA		LRR1	2
2557	GHTISLNCS AAGTPTPSLVWVLPNGTDLQS		LRR2	2.2

GQQLQRFYHKADGM

2601	LHISGLSSV DAGAYRCVARNAAGHTER		LRR1	2
2628	LVSLKVGLK PEANKQYHNLVSIIN		LRR2	2.4
2652	GETLKLPCT PPGAGQGRFSWTLPN		LRR3	2.8
2676	GMHLEGPQT LGRVSLLDNGTLTVREASVFD		LRR4	2.2

RGTYVCRMETEYGPSVTSIPVIVIAYPPRITSEPTPVIYTRPGNTVKLNCMAMGIPKADI
TWELPDKSHLKAGVQARLYGNRFLHPQGSLTIQHATQRDAGFYKCMAKNILGSDSKTTYI
HVF

>ENSP00000312273

MGGAGILLLLLAGAGVVVAWRPPKGK

27	CPLRCSCSKDSALCEGSPDLPVSFS		NF	4
52	PTLLSLSLV RTGVTQLKAGSFLRIPS		LRR1	2
78	LHLLLFTSN SFSVIEDDAFAGLSH		LRR2	3.4
102	LQYLFIEDN EIGSISKNALRGLRS		LRR3	3.4
126	LTHLSLANN HLETLPRFLFRGLDT		LRR4	4
150	LTHVDLRGN				LRR5	3.4
159	PFQCDCRVLWLLQWMPTVNASVGTGACAGPASLSHMQLHHLDPKTFKCR		CF1_short	3

SHIFT -44!

164	CRVLWLLQW MPTVNASVGTGACAGPAS		LRR1	2
191	LSHMQLHHL DPKTFKCRAIELSWFQTVGES		LRR2	2.4

ALSVEPFSYQGEPHIVLAQPFA

243	GRCLILSWD YSLQRFRPEEELPAASVVS		LRR1	2.4
271	CKPLVLGPS LFVLAARLWGGSQLWARP		LRR2	2.4
298	SPGLRLAPT QTLAPRRLLRPNDAE		LRR3	2.4
322	LLWLEGQPC FVVADASKAGSTTLLCRDGPG		LRR4	2.8

FYPHQSLHAWHRDTDAEALELDGRPHLLLASASQRPVLFHWTGGRFERRTDIPEAEDVYA
TRHFQA

418	GGDVFLCLT RYIGDSMVMRWDGSMFRL		LRR1	2.2
445	LQQLPSRGA HVFQPLLIARDQ			LRR2	2
466	LAILGSDFA FSQVLRLEPDKGL			LRR3	2
488	LEPLQELGP PALVAPRAFAHITMAGRRFLF		LRR4	2

AACFKGPTQIYQHHEIDLSA

>ENSP00000309253

MTKDKEPIVKSFHFVCLMIIIVGTRIQFSDGNEFAVDKSKRGLIHV

47	PKDLPLKTK VLDMSQNYIAELQVSDMSF		LRR1	2
75	LSELTVLRL SHNRIQLLDLSVFKFNQD		LRR2	2.4
102	LEYLDLSHN QLQKISCHPIVS			LRR3	4
123	FRHLDLSFN DFKALPICKEFGNLSQ		LRR4	3.4
148	LNFLGLSAM KLQKLDLLPIAHLH		LRR5	3
171	LSYILLDLR NYYIKENETESLQILN		LRR6	2.4
196	AKTLHLVFH PTSLFAIQVNISVNT		LRR7	2.4
220	LGCLQLTNI KLNDDNCQVFIKF			LRR8	3
242	LSELTRGST LLNFTLNHIETTWKCLVRVFQ		LRR9	2.4

SHIFT -21!

251	LLNFTLNHI ETTWKCLVRVFQFLWPKPVEY		LRR1	2.4
281	LNIYNLTII ESIREEDFTYSKTT		LRR2	2
304	LKALTIEHI TNQVFLFSQTALYTVFSE		LRR3	2.4
331	MNIMMLTIS DTPFIHMLCPHAPST		LRR4	2.2
355	FKFLNFTQN VFTDSIFEKCSTLVK		LRR5	2.8
379	LETLILQKN GLKDLFKVGLMTKDMPS		LRR6	4
405	LEILDVSWN SLESGRHKENCTWVES		LRR7	3.4
430	IVVLNLSSN MLTDSVFRCLPPR			LRR8	3.4
452	IKVLDLHSN KIKSVPKQVVKLEA		LRR9	3.4
475	LQELNVAFN SLTDLPGCGSFSS			LRR10	3.4
497	LSVLIIDHN SVSHPSADFFQSCQK		LRR11	3.4
521	MRSIKAGDN				LRR12	2.2
530	PFQCTCELREFVKNIDQVSSEVLEGWPDSYKCDYPESYRGSPLKDFHMSELSCN		CF1_short	3

ITLLIVTIGATMLVLAVTVTSL

606	CIYLDLPWY LRMVCQWTQTRRRARN		LRR1	2
631	IPLEELQRN LQFHAFISYSEHDSAWVKSEL		LRR2	2.4

VPYLEKEDIQICLHERNFVPGKSIVENIINCIEKSYKSIFVLSPNFVQSEWCHYELYFAH
HNLFHEG

728	SNNLILILL EPIPQNSIPNKYHK		LRR1	2
751	LKALMTQRT YLQWPKEKSKRGLF		LRR2	2.4
774	WANIRAAFN MKLTLVTENNDVKS		LRR3	2.2


>ENSP00000298119

MEKI

5	LFYLFLIGI AVKAQICPKRCVCQILSPN		LRR1	3
33	LATLCAKKG LLFVPPNIDRRTVELRLADNF		LRR2	2.4

SHIFT -10!

53	TVELRLADN FVTNIKRKDFANMTS		LRR1	3
77	LVDLTLSRN TISFITPHAFADLRN		LRR2	4
101	LRALHLNSN RLTKITNDMFSGLSN		LRR3	4
125	LHHLILNNN QLTLISSTAFDDVFA		LRR4	4
149	LEELDLSYN NLETIPWDAVEKMVS		LRR5	4
173	LHTLSLDHN MIDNIPKGTFSHLHK		LRR6	4
197	MTRLDVTSN KLQKLPPDPLFQRAQVLATSG		LRR7	2.8

SHIFT -20!

207	LQKLPPDPL FQRAQVLATSGIISPS		LRR1	2
232	TFALSFGGN PLHCNCELLWLRRLSREDDLE		LRR2	2.4

TCASPPLLTGRYFWSIPEEEFLCEPPLITRHTHEMRVLEGQRATLRCKARGDPEPAIHWI
SPEGKLISNATRSLVYDNGT

342	LDILITTVK DTGAFTCIASNPAGEATQIVD		LRR1	2
372	LHIIKLPHL LNSTNHIHEPDPGSSDISTST		LRR2	2.4

KSGSNTSSSNGDTKLSQDKIVVAEATSSTALLKFNFQRNIPG

444	IRMFQIQYN GTYDDTLVYRMIPP		LRR1	2.2
467	TSKTFLVNN LAAGTMYDLCVLAIYDDGITS		LRR2	2

LTATRVVGCIQFTTEQDYVRCHFMQSQFLGGTMIIIIGGIIVASVLVFIIILMIRYKVCN
NNGQHKVTKVSNVYSQTNGAQIQGCSVTLPQSVSKQAVGHEENAQCCKATSDNVIQSSET
CSSQDSSTTTSALPPSWTSSTSVSQKQKRKTGTKPSTEPQNEAVTNVESQNTNRNN

673	STALQLASR PPDSVTEGPTSKRAH		LRR1	2
697	IKPNALLTN VDQIVQETQRLELI		LRR2	2.4


>ENSP00000273739

MRGVGWQMLSL

12	SLGLVLAIL NKVAPQACPAQCSCSGSTVD		LRR1	2
41	CHGLALRSV PRNIPRNTERLDLNGNNITRI		LRR2	2

SHIFT -16!

55	RNTERLDLN GNNITRITKTDFAGLRH		LRR1	2
81	LRVLQLMEN KISTIERGAFQDLKE		LRR2	4
105	LERLRLNRN HLQLFPELLFLGTAK		LRR3	4
129	LYRLDLSEN QIQAIPRKAFRGAVD		LRR4	4
153	IKNLQLDYN QISCIEDGAFRALRD		LRR5	3.4
177	LEVLTLNNN NITRLSVASFNHMPK		LRR6	4
201	LRTFRLHSN				LRR7	3.4
210	NLYCDCHLAWLSDWLRQRPRVGLYTQCMGPSHLRGHNVAEVQKREFVCS		CF1_short	3

GHQSFMAPS

268	CSVLHCPAACTCSNNIVDCRGKGLTEIPTNLPET		NF	4
302	ITEIRLEQN TIKVIPPGAFSPYKK		LRR1	2.8
326	LRRIDLSNN QISELAPDAFQGLRS		LRR2	3.4
350	LNSLVLYGN KITELPKSLFEGLFS		LRR3	4
374	LQLLLLNAN KINCLRVDAFQDLHN		LRR4	4
398	LNLLSLYDN KLQTIAKGTFSPLRA		LRR5	4
422	IQTMHLAQN				LRR6	2.8
431	PFICDCHLKWLADYLHTNPIETSGARCTSPRRLANKRIGQIKSKKFRCS		CF1_short	3

AKEQYFIPGTEDYRSKLSGDCFADLA

506	CPEKCRCEGTTVDCSNQKLNKIPEHIP		NF	4
533	QYTAELRLN NNEFTVLEATGIFKKLPQ		LRR1	2.4
560	LRKINFSNN KITDIEEGAFEGASG		LRR2	2.8
584	VNEILLTSN RLENVQHKMFKGLES		LRR3	2.8
608	LKTLMLRSN RITCVGNDSFIGLSS		LRR4	4
632	VRLLSLYDN QITTVAPGAFDTLHS		LRR5	3.4
656	LSTLNLLAN				LRR6	4
665	PFNCNCYLAWLGEWLRKKRIVTGNPRCQKPYFLKEIPIQDVAIQDFTCD		CF1_short	3

DGNDDNSCSPLSR

727	CPTECTCLDTVVRCSNKGLKVLPKGIPRD		NF	4

SHIFT -22!

734	LDTVVRCSN KGLKVLPKGIPRD			LRR1	2.4
756	VTELYLDGN QFTLVPKELSNYKH		LRR2	3.4
779	LTLIDLSNN RISTLSNQSFSNMTQ		LRR3	3.4
803	LLTLILSYN RLRCIPPRTFDGLKS		LRR4	4
827	LRLLSLHGN DISVVPEGAFNDLSA		LRR5	4
851	LSHLAIGAN				LRR6	3.4
860	PLYCDCNMQWLSDWVKSEYKEPGIARCAGPGEMADKLLLTTPSKKFTCQ		CF1_short	3

GPVDVNILAKCNPCLSNPCKNDGTCNSDPVDFYRCTCPYGFKGQDCDVPIHACISNPCKH
GGTCHLKEGEEDGFWCICADGFEGENCEVNVDDCEDNDCENNSTCVDGINNYTCLCPPEY
TGELCEEKLDFCAQDLNPCQHDSKCILTPKGFKCDCTPGYVGEHCDIDFDDCQDNKCKNG
AHCTDAVNGYTCICPEGYSGLFCEFSPPMVLPRTSPCDNFDCQNGAQCIVRINEPICQCL
PGYQGEKCEKLVSVNFINKESYLQIPSAKVRPQTNITLQIATDEDSGILLYKGDKDHIAV
ELYRGRVRASYDTGSHPASAIYSVETINDGNFHIVELLALDQS

1252	LSLSVDGGN PKIITNLSKQSTLNFDSPLY		LRR1	2
1281	VGGMPGKSN VASLRQAPGQNGTSFH		LRR2	2.2
1306	GCIRNLYIN SELQDFQKVPMQTGILPGCEP		LRR3	2.4

CHKKVCAHGTCQPSSQAGFTCECQEGWMGPLCDQRTNDPCLGNKCVHGTCLPINAFSYSC
KCLEGHGGVLCDEEEDLFNPCQAIKCKHGKCRLSGLGQPYCECSSGYTGDSCDREISCRG
ERIRDYYQKQQGYAACQTTKKVSRLECRGGCAGGQCCGPLRSKRRKYSFECTDGSSFVDE
VEKVVKCGCTRCVS

>ENSP00000340983

MGGT

5	LAWTLLLPL LLRESDSLEPSCT			LRR1	2
27	VSSADVDWN AEFSATCLNFSGLSLSLPHNQ		LRR2	2.2

SHIFT -14!

43	LNFSGLSLS LPHNQSLRASNVI			LRR1	2.4
65	LLDLSGNGL RELPVTFFAHLQK			LRR2	2.4
87	LEVLNVLRN PLSRVDGALAARC			LRR3	3.4
109	DLDLQADCN CALESWHDIRRDNCSGQKPLL		LRR4	2.4

CWDTTSSQHNLSAFLEVSCAPGLASATIGAVVVSGCLLLGLAIAGP

185	VLAWRLWRC RVARSRELNKPWAAQDGP		LRR1	2.2
212	KPGLGLQPR YGSRSAPKPQVAVPSCPSTPD		LRR2	2

YENMFVGQPAAEHQWDEQGAHPSEDNDFYINYKDIDLASQPVYCNLQSLGQAPMDEEEYV
IPGH

>ENSP00000354333

MPSPPGLRALWLCAALCASRRAGGAPQPGPGPTA

35	CPAPCHCQEDGIMLSADCSELGLSAVPGDLDPL		NF	4

SHIFT -23!

45	GIMLSADCS ELGLSAVPGDLDPL		LRR1	2.2
68	TAYLDLSMN NLTELQPGLFHHLRF		LRR2	3
92	LEELRLSGN HLSHIPGQAFSGLYS		LRR3	4
116	LKILMLQNN QLGGIPAEALWELPS		LRR4	4
140	LQSLRLDAN LISLVPERSFEGLSS		LRR5	4
164	LRHLWLDDN ALTEIPVRALNNLPA		LRR6	4
188	LQAMTLALN RISHIPDYAFQNLTS		LRR7	3.4
212	LVVLHLHNN RIQHLGTHSFEGLHN		LRR8	4
236	LETLDLNYN KLQEFPVAIRTLGR		LRR9	4
259	LQELGFHNN NIKAIPEKAFMGNPL		LRR10	3.4
283	LQTIHFYDN PIQFVGRSAFQYLPK		LRR11	2.8
307	LHTLSLNGA MDIQEFPDLKGTTS		LRR12	3
330	LEILTLTRA GIRLLPSGMCQQLPR		LRR13	3
354	LRVLELSHN QIEELPSLHRCQK			LRR14	4
376	LEEIGLQHN RIWEIGADTFSQLSS		LRR15	3.4
400	LQALDLSWN AIRSIHPEAFSTLHS		LRR16	4
424	LVKLDLTDN QLTTLPLAGLGG			LRR17	4
445	LMHLKLKGN LALSQAFSKDSFPK		LRR18	4
468	LRILEVPYA YQCCPYGMCASFFKASGQWE		LRR19	2.4
497	AEDLHLDDE ESSKRPLGLLARQAENHYDQD		LRR20	2.4
527	LDELQLEME DSKPHPSVQCSPTPGPFKPCE		LRR21	3

YLFESWGIRLAVWA

571	IVLLSVLCN GLVLLTVFAGGPVPLPPVKF		LRR1	2.8
600	VVGAIAGAN TLTGISCGLLASVDALTFGQF		LRR2	2.2

SEYGARWETGLGCRATGFLAVLGSEASVLLLTLAAVQCSVSVSCVRAYGKSPSLGSVRAG
V

691	LGCLALAGL AAALPLASVGEYG			LRR1	3
713	ASPLCLPYA PPEGQPAALGFTVALVMMNSF		LRR2	2.4

CFLVVAGAYIKLYCDLPRGDFEAVWDCAMVRHVAWLIFADGLLYCPVAFLSF

795	ASMLGLFPV TPEAVKSVLLVVLPLPACL		LRR1	2.4
823	NPLLYLLFN PHFRDDLRRLRPRAGDSGPLA		LRR2	3

SHIFT -28!

825	LLYLLFNPH FRDDLRRLRPRAGDSGPLAY		LRR1	2.4
854	AAAGELEKS SCDSTQALVAFS			LRR2	2.2
875	DVDLILEAS EAGRPPGLETYG			LRR3	2.4
896	FPSVTLISC QQPGAPRLEGSHCVEPEGNHF		LRR4	2.2

SHIFT -17!

909	APRLEGSHC VEPEGNHFGNPQPSM		LRR1	2.2
933	DGELLLRAE GSTPAGGGLSGGGGFQPSGLA		LRR2	2

FASHV

>ENSP00000254320

MAESGLAM

9	WPSLLLLLL LPGPPPVAGLEDAAFPHLGES		LRR1	2.4
39	LQPLPRACP LRCSCPRVDTVDCDG		LRR2	2
46	CPLRCSCPRVDTVDCDGLDLRVFPDNITRA		NF	4

SHIFT -13!

63	LDLRVFPDN ITRAAQHLSLQN			LRR3	2.4
84	NQLQELPYN ELSRLSGLRTLNLHNNLISSE		LRR4	2

SHIFT -20!

94	LSRLSGLRT LNLHNNLISSEGLPDEAFES		LRR1	2.8
123	LTQLQHLCV AHNKLSVAPQFLPRS		LRR2	2
147	LRVADLAAN QVMEIFPLTFGEKPA		LRR3	3.4
171	LRSVYLHNN QLSNAGLPPDAFRGSEA		LRR4	3.4
197	IATLSLSNN QLSYLPPSLPPS			LRR5	3.4
218	LERLHLQNN LISKVPRGALSRQTQ		LRR6	4
242	LRELYLQHN QLTDSGLDATTFSKLHS		LRR7	4
268	LEYLDLSHN QLTTVPAGLPRT			LRR8	4
289	LAILHLGRN RIRQVEAARLHGARG		LRR9	4
313	LRYLLLQHN QLGSSGLPAGALRPLRG		LRR10	4
339	LHTLHLYGN GLDRVPPALPRR			LRR11	4
360	LRALVLPHN HVAALGARDLVATPG		LRR12	4
384	LTELNLAYN RLASARVHHRAFRRLRA		LRR13	4
410	LRSLDLAGN QLTRLPMGLPTG			LRR14	4
431	LRTLQLQRN QLRMLEPEPLAGLDQ		LRR15	4
455	LRELSLAHN RLRVGDIGPGTWHE		LRR16	4
478	LQALQVRHR LVSHTVPRAPPSPCLPCHVPN		LRR17	2.4

ILVSW

>ENSP00000314901

MARMSFVIAACQLVLGLLMTSLTESSIQNSE

32	CPQLCVCEIRPWFTPQSTYREATTVDCNDLRLTRIPSNLSSD		NF	4

SHIFT -16!

58	CNDLRLTRI PSNLSSDTQVLLLQSNNIAKT		LRR1	2
88	VDELQQLFN LTELDFSQNNFTNIKEVG		LRR2	2.4
115	LANLTQLTT LHLEENQITEMTDYCLQDLSN		LRR3	2.4
145	LQELYINHN QISTISAHAFAGLKN		LRR4	3.4
169	LLRLHLNSN KLKVIDSRWFDSTPN		LRR5	4
193	LEILMIGEN PVIGILDMNFKPLAN		LRR6	3.4
217	LRSLVLAGM YLTDIPGNALVGLDS		LRR7	3
241	LESLSFYDN KLVKVPQLALQKVPN		LRR8	3.4
265	LKFLDLNKN PIHKIQEGDFKNM			LRR9	4
287	LRLKELGIN NMGELVSVDRYALDNLPE		LRR10	3
314	LTKLEATNN PKLSYIHRLAFRSVPA		LRR11	3.4
339	LESLMLNNN ALNAIYQKTVESLPN		LRR12	4
363	LREISIHSN				LRR13	2.8
372	PLRCDCVIHWINSNKTNIRFMEPLSMFCAMPPEYKGHQVKEVLIQDSSEQ		CF1_short	3

CLPMISHDSFPNRLNVDIGTTVFLDCRAMAEPEPEIYWVTPIGNKITVETLSDKYKLSSE
GTLEISNIQIEDSGRYTCVAQNVQGADTRVATIKVNGTLLDGTQVLKIYVKQTESHSILV
SWKVNSNVMTSNLKWSSATMKIDNPHITYTARVPVDVHEYNLTHLQPSTDYEVCLTVSNI
HQQTQKSCVNVTTKNAAFAVDISDQETSTALAAVMGSMFAVISLASIAVYFAKRFKRKNY
HHSLKKYMQKTSSIPLNELYPPLINLWEGDSEKDKDGSADTKPTQVDTSRSYYMW

>ENSP00000340089

MGDHLDL

8	LLGVVLMAG PVFGIPSCSFDGRIAFYRFCN		LRR1	2.4
38	LTQVPQVLN TTERLLLSFNYIRTVTASSFP		LRR2	2.4

SHIFT -20!

48	TERLLLSFN YIRTVTASSFPF			LRR1	3
69	LEQLQLLEL GSQYTPLTIDKEAFRNLPN		LRR2	3
97	LRILDLGSS KIYFLHPDAFQGLFH		LRR3	3.4
121	LFELRLYFC GLSDAVLKDGYFRNLKA		LRR4	3.4
147	LTRLDLSKN QIRSLYLHPSFGKLNS		LRR5	4
172	LKSIDFSSN QIFLVCEHELEPLQGKT		LRR6	2.8
198	LSFFSLAAN SLYSRVSVDWGKCMNPFRNMV		LRR7	3.4
228	LEILDVSGN GWTVDITGNFSNAISKSQ		LRR8	3.4
255	AFSLILAHH IMGAGFGFHNIKDPDQNTFAG		LRR9	2.4

SHIFT -20!

265	MGAGFGFHN IKDPDQNTFAGLARSS		LRR1	2.2
290	VRHLDLSHG FVFSLNSRVFETLKD		LRR2	2.4
314	LKVLNLAYN KINKIADEAFYGLDN		LRR3	4
338	LQVLNLSYN LLGELYSSNFYGLPK		LRR4	4
362	VAYIDLQKN HIAIIQDQTFKFLEK		LRR5	2.8
386	LQTLDLRDN ALTTIHFIPSIPDIF		LRR6	4
410	LSGNKLVTL PKINLTANLIHLSENR		LRR7	2
435	LENLDILYF LLRVPHLQILILNQNRFSSCS		LRR8	2.4

SHIFT -20!

445	LRVPHLQIL ILNQNRFSSCSGDQTPSENPS		LRR1	2
475	LEQLFLGEN MLQLAWETELCWDVFEGLSH		LRR2	4
504	LQVLYLNHN YLNSLPPGVFSHLTA		LRR3	4
528	LRGLSLNSN RLTVLSHNDLPAN			LRR4	4
550	LEILDISRN QLLAPNPDVFVS			LRR5	3.4
571	LSVLDITHN				LRR6	3.4
580	KFICECELSTFINWLNHTNVTIAGPPADIYCVYPDSFSGVSLFSLSTEGCDEE		CF1	4

SHIFT -12!

621	LFSLSTEGC DEEEVLKSLKFSLFIVC		LRR1	2.4
647	TVTLTLFLM TILTVTKFRGFCFICYKTAQR		LRR2	2

LVFKDHPQGTEPDMYKYDAYLCFSSKDFTWVQNALLKHLDTQYSDQNRFNLCFEERDFVP
GENRIANIQDAIWNSRKIVCLVSRHFLRDGWCLEAFSYAQGRCLSDLNSALIMVVVGSLS
QYQLMKHQSIRGFVQKQQYLRWPEDFQDVGWFLHKLSQQILKKEKEKKKDNNIPLQTVAT
IS

>ENSP00000278198

M

2	LNKMTLHPQ QIMIGPRFNRALFDPLLVV		LRR1	2.4
30	LLALQLLVV AGLVRAQTCPSVCSCSNQFSK		LRR2	3

VICVRKNLREVPDGISTN

78	TRLLNLHEN QIQIIKVNSFKHLRH		LRR1	3
102	LEILQLSRN HIRTIEIGAFNGLAN		LRR2	4
126	LNTLELFDN RLTTIPNGAFVYLSK		LRR3	4
150	LKELWLRNN PIESIPSYAFNRIPS		LRR4	4
174	LRRLDLGEL KRLSYISEGAFEGLSN		LRR5	3
199	LRYLNLAMC NLREIPNLTPLIK			LRR6	3.4
221	LDELDLSGN HLSAIRPGSFQGLMH		LRR7	4
245	LQKLWMIQS QIQVIERNAFDNLQS		LRR8	2.8
269	LVEINLAHN NLTLLPHDLFTPLHH		LRR9	3.4
293	LERIHLHHN				LRR10	3.4
302	PWNCNCDILWLSWWIKDMAPSNTACCARCNTPPNLKGRYIGELDQNYFTCY		CF1_short	3

APVIVEPPADLNVTEGMAAELKCRASTSLTSVSWITPNGTVMTHGAYKVRIAVLSDGTLN
FTNVTVQDTGMYTCMVSNSVGNTTASATLNVTAATTTPFSYFSTVTVETMEPSQDEARTT
DNNVGPTPVVDWETTNVTTSLTPQSTRSTEKTFTIPVTDINSGIPGIDEVMKTTKIIIGC
FVAITLMAAVMLVIFYKMRKQHHRQNHHAPTRTVEIINVDDEITGDTPMESHLPMPAIEH
EHLNHYNSYKSPFNHTTTVNTINSIHSSVHEPLLIRMNSKDNVQETQI

>ENSP00000367157

LSLIRPLVSITRVPTRPSGAYLAHLAATQVPPRGCYVAKEAGERTFRCSQAGLSAVPSGI
PND

64	TRKLYLDAN QLASVPAGAFQHLPV		LRR1	3
88	LEELDLSHN ALAHLSGAAFQGLEGT		LRR2	4
113	LRHLDLSAN QLASVPVEAFVGL			LRR3	4
135	QIQVNLSAN				LRR4	2.4
144	PWHCDCALQEVLRQVRLVPGTGTGIVCGSGARPDLVGQEFLLLAGEEEL		CF1_short	3

SHIFT -38!

155	LRQVRLVPG TGTGIVCGSGARPDLVG		LRR1	2.4
181	QEFLLLAGE EELCGSGWGGARRSTDVAL		LRR2	2
209	LVTMGGWLT LMVAYLVHYVWQNRDETRRSL		LRR3	2.2

KRAPVLP

>ENSP00000346893

MMSASRLAGTLIPAMAFLSCVRPESWEPCVEVVPNITYQCME

43	LNFYKIPDN LPFSTKNLDLSFNP		LRR1	2.4
66	LRHLGSYSF FSFPELQVLDLSRCEIQTIED		LRR2	2

SHIFT -16!

80	LQVLDLSRC EIQTIEDGAYQSLSH		LRR1	3.4
104	LSTLILTGN PIQSLALGAFSGLSS		LRR2	4
128	LQKLVAVET NLASLENFPIGHLKT		LRR3	2.8
152	LKELNVAHN LIQSFKLPEYFSNLTN		LRR4	3.4
177	LEHLDLSSN KIQSIYCTDLRVLHQMP		LRR5	4
203	LLNLSLDLS LNPMNFIQPGAFKEIR		LRR6	3.4
228	LHKLTLRNN FDSLNVMKTCIQG			LRR7	4
250	LAGLEVHRL VLGEFRNEGNLEKFD		LRR8	2.4
274	KSALEGLCN LTIEEFRLAYLDYY		LRR9	2.4
297	LDDIIDLFN CLTNVSSFSLVSVTIERVKDF		LRR10	2.4

SYNFG

332	WQHLELVNC KFGQFPTLKLKS			LRR1	2.8
353	LKRLTFTSN KGGNAFSEVDLPS			LRR2	3.4
375	LEFLDLSRN GLSFKGCCSQSDFGTTS		LRR3	4
401	LKYLDLSFN GVITMSSNFLGLEQ		LRR4	4
424	LEHLDFQHS NLKQMSEFSVFLSLRN		LRR5	2.8
449	LIYLDISHT HTRVAFNGIFNGLSS		LRR6	2.8
473	LEVLKMAGN SFQENFLPDIFTELRN		LRR7	3.4
498	LTFLDLSQC QLEQLSPTAFNSLSS		LRR8	3.4
522	LQVLNMSHN NFFSLDTFPYKCLNS		LRR9	3.4
546	LQVLDYSLN HIMTSKKQELQHFPSS		LRR10	3
571	LAFLNLTQN				LRR11	4
580	DFACTCEHQSFLQWIKDQRQLLVEVERMECATPSDKQGMPVLSLNITCQMN		CF1	4

SHIFT -15!

616	QGMPVLSLN ITCQMNKTIIGVSV		LRR1	2
639	LSVLVVSVV AVLVYKFYFHLMLLAGCIKYG		LRR2	2.4

SHIFT -11!

658	LMLLAGCIK YGRGENIYDAFVIYSSQDED		LRR1	2.4
687	WVRNELVKN LEEGVPPFQLCLHYRDF		LRR2	2.4
713	IPGVAIAAN IIHEGFHKSRKVIVVVSQHFI		LRR3	2.2

QSRWCIFEYEIAQTWQFLSSRAGIIFIVLQKVEKTLLRQQV

784	ELYRLLSRN TYLEWEDSVLGRHIFWRR		LRR1	2
811	LRKALLDGK SWNPEGTVGTGCNWQEATSI		LRR2	2.4


>ENSP00000306524

MIPVAEFKQFTEQQPAFKVLKPWWDVLAEY

31	LTVAMLMIG VFGCTLQVTQDKIIC		LRR1	2.4
55	LPNHELQEN LSEAPCQQLLPRGIPEQIGA		LRR2	3
84	LQEVKGLKN NLDLQQYSFINQLCYETALHW		LRR3	2.8

SHIFT -23!

91	KNNLDLQQY SFINQLCYETALHWYAKY		LRR1	2
118	FPYLVVIHT LIFMVCTSFWFKFPGTSSKIE		LRR2	2.2

HFISILGKCFDSPWTTRALSEVSGENQKGPAATERAAATIVAMAGTGPGKAGEGEKEKVL
AEPEKVVTEPPVVTLLDKKEGEQAKALFEKVKKFRMHVEEGDILYTMYIRQTVLKVCKFL
AILVYNLVYVEKISFLVACRVETSEVTGYASFCCNHTKAHLFSKLAFCYISFVCIYGLTC
IYTLYW

334	LFHRPLKEY SFRSVREETGMGDIPDVKNDF		LRR1	2
364	AFMLHLIDQ YDSLYSKRFAVFLSEVSESR		LRR2	2.4
393	LKQLNLNHE WTPEKLRQKLQRNA		LRR3	3
416	AGRLELALC MLPGLPDTVFELSE		LRR4	2.8
439	VESLRLEAI CDITFPPGLSQLVH		LRR5	2.4
462	LQELSLLHS PARLPFSLQVFLRDHLKVMRV		LRR6	3.4

KCEE

496	LREVPLWVF GLRGLEELHLEG			LRR1	2.4
517	LFPQELARA ATLESLRELKQLKVLSLRSNA		LRR2	2

SHIFT -16!

531	LRELKQLKV LSLRSNAGKVPASVTDVAGH		LRR1	2
560	LQRLSLHND GARLVALNSLKKLAA		LRR2	3
584	LRELELVAC GLERIPHAVFSLGA		LRR3	3.4
607	LQELDLKDN HLRSIEEILSFQHCRK		LRR4	4
632	LVTLRLWHN QIAYVPEHVRKLRS		LRR5	4
655	LEQLYLSYN KLETLPSQLGLCSG		LRR6	4
678	LRLLDVSHN GLHSLPPEVGLLQN		LRR7	3.4
701	LQHLALSYN ALEALPEELFFCRK		LRR8	4
724	LRTLLLGDN QLSQLSPHVGALRA		LRR9	4
747	LSRLELKGN RLEALPEELGNCGG		LRR10	4
770	LKKAGLLVE DTLYQGLPAEVRDKMEEE		LRR11	2.4


>ENSP00000354932

MTSIFHFAIIFMLILQIRIQLSEESEFLVDRSKNGLIHVPKD

43	LSQKTTILN ISQNYISELWTSDILSLSK		LRR1	2
71	LRILIISHN RIQYLDISVFKFNQE		LRR2	3.4
95	LEYLDLSHN KLVKISCHPTVN			LRR3	4
116	LKHLDLSFN AFDALPICKEFGNMSQ		LRR4	4
141	LKFLGLSTT HLEKSSVLPIAHLNIS		LRR5	3.4
166	KVLLVLGET YGEKEDPEGLQDFNTESLHIV		LRR6	2.4

FPTNKEFHFILDVSVKT

213	VANLELSNI KCVLEDNKCSYF			LRR1	2.4
234	LSILAKLQT NPKLSNLTLNNIETTWNSF		LRR2	2.4
262	IRILQLVWH TTVWYFSISNVKLQGQLDFRD		LRR3	2.4

FDYSGTSLKALSIHQVVSDVFGFPQSYIYEIFSNMNIKNFTVSGTRMVHMLCPSKI

348	SPFLHLDFS NNLLTDTVFENCGHLTE		LRR1	2.4
374	LETLILQMN QLKELSKIAEMTTQMKS		LRR2	4
400	LQQLDISQN SVSYDEKKGDCSWTKS		LRR3	3.4
425	LLSLNMSSN ILTDTIFRCLPPR			LRR4	3.4
447	IKVLDLHSN KIKSIPKQVVKLEA		LRR5	3.4
470	LQELNVAFN SLTDLPGCGSFSS			LRR6	3.4
492	LSVLIIDHN SVSHPSADFFQSCQK		LRR7	3.4
516	MRSIKAGDN				LRR8	2.2
525	PFQCTCELGEFVKNIDQVSSEVLEGWPDSYKCDYPESYRGTLLKDFHMSELSCN		CF1_short	3

ITLLIVTIVATMLVLAVTVTSL

601	CSYLDLPWY LRMVCQWTQTRRRARN		LRR1	2
626	IPLEELQRN LQFHAFISYSGHDSF		LRR2	2.4
650	WVKNELLPN LEKEGMQICLHERNFVPGKSI		LRR3	2.4

VENIITCIEKSYKSIFVLSPNFVQSEWCHYELYFAHHNLFHEG

723	SNSLILILL EPIPQYSIPSSYHK		LRR1	2
746	LKSLMARRT YLEWPKEKSKRGLF		LRR2	2.8
769	WANLRAAIN IKLTEQAKK			LRR3	2.8


>ENSP00000312082

MKESSLQNSSCS

13	LGKETKKEN MFLQSSMLTCIFLLISG		LRR1	2
39	SCELCAEEN FSRSYPCDEKKQNDSVIAECS		LRR2	2.4

NRRLQEVPQTVGKY

83	VTELDLSDN FITHITNESFQGLQN		LRR1	3.4
107	LTKINLNHN PNVQHQNGNPGIQSNGLNITD		LRR2	3.4
137	GAFLNLKNL RELLLEDNQLPQIPSGLPES		LRR3	2.4
166	LTELSLIQN NIYNITKEGISRLIN		LRR4	4
190	LKNLYLAWN CYFNKVCEKTNIEDGVFET		LRR5	4
218	LTNLELLSL SFNSLSHVPPKLPSS		LRR6	3
242	LRKLFLSNT QIKYISEEDFKGLIN		LRR7	3.4
266	LTLLDLSGN CPRCFNAPFPCVPCDGGASIN		LRR8	4

IDRFAFQN

304	LTQLRYLNL SSTSLRKINAAWFKNMPH		LRR1	2
331	LKVLDLEFN YLVGEIASGAFLTMLPR		LRR2	4
357	LEILDLSFN YIKGSYPQHINISRNFSKLLS		LRR3	4
387	LRALHLRGY VFQELREDDFQPLMQLPN		LRR4	3
414	LSTINLGIN FIKQIDFKLFQNFSN		LRR5	3.4
438	LEIIYLSEN RISPLVKDTRQSYANSSSFQR		LRR6	3.4

HIRKRRSTDFEFDPHSNFYHFTRPLIKPQCAAY

501	GKALDLSLN SIFFIGPNQFENLPD		LRR1	3.4
525	IACLNLSAN SNAQVLSGTEFSAIPH		LRR2	3.4
550	VKYLDLTNN RLDFDNASALTELSD		LRR3	3.4
574	LEVLDLSYN SHYFRIAGVTHHLEFIQNFTN		LRR4	4
604	LKVLNLSHN NIYTLTDKYNLESKS		LRR5	4
628	LVELVFSGN RLDILWNDDDNRY			LRR6	3.4
650	ISIFKGLKN LTRLDLSLNRLKHIPN		LRR7	2.2
675	EAFLNLPAS LTELHINDNMLKFFNWTLLQQ		LRR8	2.4
705	FPRLELLDL RGNKLLFLTDSLSDFTSS		LRR9	2.4
732	LRTLLLSHN RISHLPSGFLSEVSS		LRR10	4
756	LKHLDLSSN LLKTINKSALETKTTTK		LRR11	4
782	LSMLELHGN				LRR12	4
791	PFECTCDIGDFRRWMDEHLNVKIPRLVDVICASPGDQRGKSIVSLELTTCVSD		CF1	4

SHIFT -12!

832	IVSLELTTC VSDVTAVILFFFTFFITTMVM		LRR1	2.8
862	LAALAHHLF YWDVWFIYNVCLAKVKGYRSL		LRR2	2

STSQTFYDAYISYDTKDASVTDWVINELRYHLEESRDKNVLLCLEERDWDPGLAI

947	IDNLMQSIN QSKKTVFVLTKKYAKSWNFKT		LRR1	2.4
977	AFYLALQRL MDENMDVIIFILLEPVLQH		LRR2	2.4
1005	SQYLRLRQR ICKSSILQWPDNPKAEG		LRR3	2
1031	LFWQTLRNV VLTENDSRYNNMYVDSIKQY		LRR4	2


>ENSP00000341944

MKNLYFRVITIVIGLYFTGIMTNASRKSNILFNSECQWNEYILTNCSFTGKCDIPVDISQ
T

62	AATVDVSFN FFRVLLQSHTKKEEWK		LRR1	2.2
87	IKHLDLSNN LISKITLSPFAYLHA		LRR2	3.4
111	LEVLNLSNN AIHSLSLDLLSPKSSWVKRHR		LRR3	4

SHIFT -20!

121	IHSLSLDLL SPKSSWVKRHRSSFRNRFPL		LRR1	2.4
150	LKVLILQRN KLSDTPKGLWKLKS		LRR2	4
173	LQSLDLSFN GILQIGWSDFHNCLQ		LRR3	4
197	LENLCLKSN KIFKIPPQAFKDLKK		LRR4	4
221	LQVIDLSNN ALITILPMMIIALEFP		LRR5	3.4
246	HLVVDLADN NWQCDDSVAVFQNFISESWRK		LRR6	2.4

KWNVICNRSIGNEEANGGTPQSRISRETRLPPIHLHRMKSLIRSKAERPQGGRHTGISTL
GKKAKAGSGLRKKQRRLPRSVRSTRDVQAAGKKEDAPQDLALAVCLSVFITFLVAFSLGA
FTRPYVDRLWQKKCQSKSPGLDNAYSNEGFYDDMEAAGHTPHPETHLRQVFPHLSLYENQ
TPFWVTQPHPHATVIPDRTLGRSRKDPGSSQSPGQCGDNTGAGSGNDGAVYSILQRHPHA
GNRELMSAAQDHIHRNDILGEWTYETVAQEEPLSAHSVGVSSVAGTSHAVSGSSRYDSNE
LDPSLSGEITASLCKMLTHAEAQRTGDSKERGGTEQSLWDSQMEFSKERQVSSSIDLLSI
QQPRLSGARAEEALSAHYSEVPYGDPRDTGPSVFPPRWDSGLDVTPANKEPVQKSTPSDT
CCELESDCDSDEGSLFTLSSISSESARSKTEEAVPDEESLQDESSGASKDNVTAVDSLEE
NVTFQTIPGKCKNQEDPFEKPLISAPDSGMYKTHLENASDTDRSEGLSPWPRSPGNSPLG
DEFPGMFTYDYDTALQSKAAEWHCSLRDLEFSNVDVLQQTPPCSAEVPSDPDKAAFHERD
SDILK

>ENSP00000328625

MELLPLWLCLGFHFLTVGWRNRSGTATAASQGVCKLVGGAADCRGQSLASVPSSLPPH

59	ARMLTLDAN PLKTLWNHSLQPYPL		LRR1	3.4
83	LESLSLHSC HLERISRGAFQEQGH		LRR2	3.4
107	LRSLVLGDN CLSENYEETAAALHALPG		LRR3	4
134	LRRLDLSGN ALTEDMAALMLQNLSS		LRR4	4
159	LRSVSLAGN TIMRLDDSVFEGLER		LRR5	3.4
183	LRELDLQRN YIFEIEGGAFDGLAE		LRR6	4
207	LRHLNLAFN NLPCIVDFGLTR			LRR7	4
228	LRVLNVSYN VLEWFLATGGEAAFE		LRR8	3.4
252	LETLDLSHN QLLFFPLLPQYSK			LRR9	4
274	LRTLLLRDN NMGFYRDLYNTSSPREMV		LRR10	4
301	AQFLLVDGN VTNITTVSLWEEFSSSDLAD		LRR11	2.8
330	LRFLDMSQN QFQYLPDGFLRKMPS		LRR12	3.4
354	LSHLNLHQN CLMTLHIREHEPPGA		LRR13	4
378	LTELDLSHN QLSELHLAPGLASCLGS		LRR14	4
404	LRLFNLSSN QLLGVPPGLFANARN		LRR15	3.4
428	ITTLDMSHN QISLCPLPAASDRVGPPSCVD		LRR16	2.8

SHIFT -18!

440	LCPLPAASD RVGPPSCVDFRNMAS		LRR1	2.4
464	LRSLSLEGC GLGALPDCPFQGTS		LRR2	3.4
487	LTYLDLSSN WGVLNGSLAPLQDVAPM		LRR3	4
513	LQVLSLRNM GLHSSFMALDFSGFGN		LRR4	3
538	LRDLDLSGN CLTTFPRFGGSLA			LRR5	4
560	LETLDLRRN SLTALPQKAVSEQLSRG		LRR6	4
586	LRTIYLSQN PYDCCGVDGWGALQHGQTV		LRR7	3.4
614	ADWAMVTCN LSSKIIRVTELPGGVPRDCK		LRR8	2.2
643	WERLDLGLL YLVLILPSCLTLLVACTVIVL		LRR9	2.4

TFKKPLLQVIKSRCHWSSVY

>ENSP00000273261

MARPVRGGLGAPRRSPCLLLLWLLLLRLEPVTAAAGPRAP

41	CAAACTCAGDSLDCGGRGLAALPGD		NF	4
66	LPSWTRSLN LSYNKLSEIDPAGFEDLPN		LRR1	2.4
94	LQEVYLNNN ELTAVPSLGAASSH		LRR2	3.4
117	VVSLFLQHN KIRSVEGSQLKAYLS		LRR3	3.4
141	LEVLDLSLN NITEVRNTCFPHGPP		LRR4	4
165	IKELNLAGN RIGTLELGAFDGLSRS		LRR5	3.4
190	LLTLRLSKN RITQLPVRAFKLPR		LRR6	4
213	LTQLDLNRN RIRLIEGLTFQGLNS		LRR7	4
237	LEVLKLQRN NISKLTDGAFWGLSK		LRR8	4
261	MHVLHLEYN SLVEVNSGSLYGLTA		LRR9	3.4
285	LHQLHLSNN SIARIHRKGWSFCQK		LRR10	4
309	LHELVLSFN NLTRLDEESLAELSS		LRR11	4
333	LSVLRLSHN SISHIAEGAFKGLRS		LRR12	4
357	LRVLDLDHN EISGTIEDTSGAFSGLDS		LRR13	4
384	LSKLTLFGN KIKSVAKRAFSGLEG		LRR14	4
408	LEHLNLGGN AIRSVQFDAFVKMKN		LRR15	4
432	LKELHISSD				LRR16	2.4
441	SFLCDCQLKWLPPWLIGRMLQAFVTATCAHPESLKGQSIFSVPPESFVCD		CF1_short	3

SHIFT -59!

432	LKELHISSD SFLCDCQLKWLPPWLI		LRR16	2.4
457	GRMLQAFVT ATCAHPESLKGQSIFSVPPES		LRR17	2.2

FVCDDFLKPQIITQPETTMAMVGKDIRFTCSAASSSSSPMTFAWKKDNEVLTNADMENFV
HVHAQDGEVMEY

559	TTILHLRQV TFGHEGRYQCVITNHFGSTY		LRR1	2
588	SHKARLTVN VLPSFTKTPHDITIRTTTMAR		LRR2	2.4

LECAATGHPNPQIAWQKDGGTDFPAARERRMHVMPDDDVFFITDVKIDDAGVYSCTAQNS
AGSISANATLTVLETPSLVVPLEDRVVSVGETVALQCKATGNPPPRITWFKGDRPLSLTE
RHHLTPDNQLLVVQNVVAEDAGRYTCEMSNTLGTERAHSQ

778	LSVLPAAGC RKDGTTVGIFTIAVVSSIV		LRR1	2.8
806	LTSLVWVCI IYQTRKKSEEYSVTNTDETVV		LRR2	2.4

PPDVPSYLSSQGTLSDRQETVVRTEGGPQANGHIESNGVCPRDASHFPEPDTHSVACRQP
KLCAGSAYHKEPWKAMEKAEGTPGPHKMEHGGRVVCSDCNTEVDCYSRGQAFHPQPVSRD
SAQPSAPNGPEPGGSDQEHSPHHQCSRTAAGSCPECQGSLYPSNHDRMLTAVKKKPMASL
DGKGDSSWT

1025	LARLYHPDS TELQPASSLTSGSPERA		LRR1	2.4
1051	EAQYLLVSN GHLPKACDASPESTPLTGQLP		LRR2	2

GKQRVPLLLAPKS

>ENSP00000306864

MCSRVPLLLPLLLLLALGPGVQG

24	CPSGCQCSQPQTVFCTARQGTTVPRDVPPD		NF	4
54	TVGLYVFEN GITMLDAGSFAGLPG		LRR1	2.4
78	LQLLDLSQN QIASLPSGVFQPLAN		LRR2	4
102	LSNLDLTAN RLHEITNETFRGLRR		LRR3	4
126	LERLYLGKN RIRHIQPGAFDTLDR		LRR4	4
150	LLELKLQDN ELRALPPLRLPR			LRR5	4
171	LLLLDLSHN SLLALEPGILDTANVEALR		LRR6	4
199	LAGLGLQQL DEGLFSRLRNLHDLDVSDNQL		LRR7	3

SHIFT -14!

215	LRNLHDLDV SDNQLERVPPVIRGLRG		LRR1	2
241	LTRLRLAGN TRIAQLRPEDLAGLAA		LRR2	4
266	LQELDVSNL SLQALPGDLSGLFPR		LRR3	2.4
290	LRLLAAARN				LRR4	3.4
299	PFNCVCPLSWFGPWVRESHVTLASPEETRCHFPPKNAGRLLLELDYADFGCP		CF1_short	3

ATTTTATVPTTRPVVREPTALSSSLAPTWLSPTEPATEAPSPPSTAPPTVGPVPQPQDCP
PSTCLNGGTCHLGTRHHLACLCPEGFTGLYCESQMGQGTRPSPTPVTPRP

461	PRSLTLGIE PVSPTSLRVGLQRYLQGSSVQ		LRR1	2
491	LRSLRLTYR NLSGPDKRLVTLRLPASLAE		LRR2	3
520	YTVTQLRPN ATYSVCVMPLGPGRVPEGEEA		LRR3	2

CGEAHTPPAVHSNHAPVTQAREGNLPLLIAPALAAVLLAA

590	LAAVGAAYC VRRGRAMAAAAQDKGQVGPG		LRR1	2.2
619	AGPLELEGV KVPLEPGPKATEGGGEALPSG		LRR2	2.4

SECEVPLMGFPGPGLQSPLHAKPYI

>ENSP00000336627

MFL

4	WLFLILSAL ISSTNADSDISVEI		LRR1	2.4
27	CNVCSCVSVENVLYVNCEKVSVYRPNQLKPPW		NF	4

SHIFT -25!

34	SVENVLYVN CEKVSVYRPNQLKPPWSN		LRR2	2
61	FYHLNFQNN FLNILYPNTFLNFSH		LRR3	2.8
85	AVSLHLGNN KLQNIEGGAFLGLSA		LRR4	3.4
109	LKQLHLNNN ELKILRADTFLGIEN		LRR5	4
133	LEYLQADYN LIKYIERGAFNKLHK		LRR6	3.4
157	LKVLILNDN LISFLPDNIFRFAS		LRR7	4
180	LTHLDIRGN RIQKLPYIGVLEHIGR		LRR8	3.4
205	VVELQLEDN				LRR9	3.4
214	PWNCSCDLLPLKAWLENMPYNIYIGEAICETPSDLYGRLLKETNKQELCPM		CF1_short	3

SHIFT -46!

219	CDLLPLKAW LENMPYNIYIGEAICETPSD		LRR1	2
248	LYGRLLKET NKQELCPMGTGSDFDVRI		LRR2	2.4
275	LPPSQLENG YTTPNGHTTQTS			LRR3	2
296	LHRLVTKPP KTTNPSKISGIVAGKA		LRR4	2
321	LSNRNLSQI VSYQTRVPPLTPCPAPCFCKT		LRR5	2

H

352	PSDLGLSVN CQEKNIQSMSELIPKP		LRR1	3
377	LNAKKLHVN GNSIKDVDVSDFTDFEG		LRR2	3
403	LDLLHLGSN QITVIKGDVFHNLTN		LRR3	4
427	LRRLYLNGN QIERLYPEIFSGLHN		LRR4	4
451	LQYLYLEYN LIKEISAGTFDSMPN		LRR5	4
475	LQLLYLNNN LLKSLPVYIFSGAP		LRR6	4
498	LARLNLRNN KFMYLPVSGVLDQLQS		LRR7	4
523	LTQIDLEGN				LRR8	3.4
532	PWDCTCDLVALKLWVEKLSDGIVVKELKCETPVQFANIELKSLKNEILCPK		CF1_short	3

SHIFT -60!

523	LTQIDLEGN PWDCTCDLVALK			LRR8	3.4
544	LWVEKLSDG IVVKELKCETPVQFA		LRR9	2
568	NIELKSLKN EILCPKLLNKPSAPFTSPAPA		LRR10	2

ITFTTPLGPIRSPPGGPVP

617	LSILILSIL VVLILTVFVAFC			LRR1	3
638	LLVFVLRRN KKPTVKHEGLGNPDCG		LRR2	3.4
663	SMQLQLRKH DHKTNKKDGLSTEAFIPQTIE		LRR3	2

QMSKSHTCGLKESETGFMFSDPPGQKVVMRNVADKEKDLLHVDTRKRLSTIDELDELFPS
RDSNVFIQNFLESKKEYNSIGVSGFEIRYPEKQPDKKSKKSLIGGNHSKIVVEQRKSEYF
ELKAKLQSSPDYLQVLEEQTALNKI

>ENSP00000339075

MCGLQFSLPCLRLFLVV

18	TCYLLLLLH KEILGCSSVCQLCTGRQIN		LRR1	2
46	CRNLGLSSI PKNFPESTVFLYLTGNNISYI		LRR2	2

SHIFT -14!

62	TVFLYLTGN NISYINESELTGLHS		LRR1	3
86	LVALYLDNS NILYVYPKAFVQLRH		LRR2	3.4
110	LYFLFLNNN FIKRLDPGIFKGLLN		LRR3	4
134	LRNLYLQYN QVSFVPRVFNDLVS		LRR4	4
157	VQYLNLQRN RLTVLGSGTFVGMVA		LRR5	3.4
181	LRILDLSNN NILRISESGFQHLEN		LRR6	4
205	LACLYLGSN NLTKVPSNAFEVLKS		LRR7	4
229	LRRLSLSHN PIEAIQPFAFKGLAN		LRR8	4
253	LEYLLLKNS RIRNVTRDGFSGINN		LRR9	3.4
277	LKHLILSHN DLENLNSDTFSLLKN		LRR10	4
301	LIYLKLDRN RIISIDNDTFENMGAS		LRR11	4
326	LKILNLSFN NLTALHPRVLKPLSS		LRR12	4
350	LIHLQANSN				LRR13	3.4
359	PWECNCKLLGLRDWLASSAITLNIYCQNPPSMRGRALRYINITNCVTS		CF1	4

SHIFT -57!

350	LIHLQANSN PWECNCKLLGLRDW		LRR13	3.4
373	LASSAITLN IYCQNPPSMRGRA			LRR14	2.4
395	LRYINITNC VTSSINVSRAWAVVKSPHIHH		LRR15	2.2

KTTALMMAWHKVTTNGSPLENTETENITFWERIPTSPAGRFFQENAFGNPLETTAVLPVQ
IQLTTSVTLNLEKNSALPNDAASMSGKTSLICTQEVEKLNEAFDI

530	LLAFFILAC VLIIFLIYKVVQF			LRR1	2.2
552	KQKLKASEN SRENRLEYYSFYQSARYNVTA		LRR2	2.4

SICNTSPNSLESPGLEQIRLHKQIVPENEAQVILFEHSAL

>ENSP00000246074

MRQTLPLLLLTVLRPSWADPPQEKVPLFRVTQQGPWGSSGSNATDSPCEGLPAAD

56	ATALTLANR NLERLPGCLPRT			LRR1	2.4
77	LRSLDASHN LLRALSTSELGHLEQ		LRR2	3.4
101	LQVLTLRHN RIAALRWGPGGPAG		LRR3	4
124	LHTLDLSYN QLAALPPCTGPALSS		LRR4	4
148	LRALALAGN PLRALQPRAFACFPA		LRR5	4
172	LQLLNLSCT ALGRGAQGGIAEAAFAGEDGA		LRR6	3.4

SHIFT -28!

174	LLNLSCTAL GRGAQGGIAEAAFAGEDGAP		LRR1	2
203	LVTLEVLDL SGTFLERVESGWIRDLPK		LRR2	2.4
230	LTSLYLRKM PRLTTLEGDIFKMTPN		LRR3	3
255	LQQLDCQDS PALASVATHIFQDTPH		LRR4	2.4
280	LQVLLFQNC NLSSFPPWTLDSSQ		LRR5	2.8
303	VLSINLFGN				LRR6	2.8
312	PLTCSCDLSWLLTDAKRTVLSRAADTMCAPAAGSSGPFSASLSLSQLPGV		CF1_short	3

CQSDQSTTLGASHPPCFNRSTYAQGTTVAPSAAPATRPAGDQQSVSKAPNVGSRTIAAWP
HSDAREGTAPSTTNSVAGHSNSSVFPRAASTTRTQHRGEHAPELVLEPDISAASTPLASK
LLGPFPTSWDRSISSPQPGQRTHATPQAPNPSLSEGEIPVLLLDDYSEEEEGRKEEVGTP
HQDVPCDYHPCKHLQTPCAELQRRWRCRCPGLSGEDTIPDPPRLQGVTETTDTSALVHWC
APNSVVHGYQIRYSAEGWAGNQSVVGVIYATARQHPLYGLSPGTTYRVCVLAANRAGLSQ
PRSSGWRSPCAAFTTKP

679	SFALLLSGL CAASGLLLASTVVLSACLCRR		LRR1	2
709	GQTLGLQRC DTHLVAYKNPAFD			LRR2	2.8
731	DYPLGLQTV S			LRR3	2


>ENSP00000052754

MKATIILLLLAQVSWAGPFQQRGLFDFMLEDEASGIGPEVPDDRDFEPSLGPV

54	CPFRCQCHLRVVQCSDLGLDKVPKDLPP		NF	4
82	DTTLLDLQN NKITEIKDGDFKNLKN		LRR1	2
107	LHALILVNN KISKVSPGAFTPLVK		LRR2	4
131	LERLYLSKN QLKELPEKMPKT			LRR3	4
152	LQELRAHEN EITKVRKVTFNGLNQ		LRR4	3.4
176	MIVIELGTN PLKSSGIENGAFQGMKK		LRR5	2.8
202	LSYIRIADT NITSIPQGLPPS			LRR6	2.2
223	LTELHLDGN KISRVDAASLKGLNN		LRR7	4
247	LAKLGLSFN SISAVDNGSLANTPH		LRR8	4
271	LRELHLDNN KLTRVPGGLAEHKY		LRR9	4
294	IQVVYLHNN NISVVGSSDFCPPGHNTKKAS		LRR10	2.8
324	YSGVSLFSN PVQYWEIQPSTFRCVYVRSAI		LRR11	2.4

QLGNYK

>ENSP00000311174

MLYSSCKSRLLDSVEQDFHLEIAKKGFCRSA

32	LHPLSLLVQ AIMLAMTLALGTLPAFLPCE		LRR1	3
61	LQPHGLVNC NWLFLKSVPHFSMAAPRGN		LRR2	2.4
89	VTSLSLSSN RIHHLHDSDFAHLPS		LRR3	3.4
113	LRHLNLKWN CPPVGLSPMHFPCHMTIEPST		LRR4	4

F

144	LAVPTLEEL NLSYNNIMTVPALPKS		LRR1	2
169	LISLSLSHT NILMLDSASLAGLHA		LRR2	3.4
193	LRFLFMDGN CYYKNPCRQALEVA		LRR3	3.4
216	PGALLGLGN LTHLSLKYNNLTVVPRNLPSS		LRR4	2.4
246	LEYLLLSYN RIVKLAPEDLANLTA		LRR5	4
270	LRVLDVGGN CRRCDHAPNPCMECPRHFPQL		LRR6	3.4

HPDTFSH

307	LSRLEGLVL KDSSLSWLNASWFRGLGN		LRR1	2.4
334	LRVLDLSEN FLYKCITKTKAFQGLTQ		LRR2	4
360	LRKLNLSFN YQKRVSFAHLSLAPSFGSLVA		LRR3	4
390	LKELDMHGI FFRSLDETTLRPLARLPM		LRR4	2.4
417	LQTLRLQMN FINQAQLGIFRAFPG		LRR5	4
441	LRYVDLSDN RISGASELTATMGEADGGEKV		LRR6	3.4

W

472	LQPGDLAPA PVDTPSSEDFRPNCSTL		LRR1	2.4
498	NFTLDLSRN NLVTVQPEMFAQLSH		LRR2	3
522	LQCLRLSHN CISQAVNGSQFLPLTG		LRR3	4
547	LQVLDLSHN KLDLYHEHSFTELPR		LRR4	4
571	LEALDLSYN SQPFGMQGVGHNFSFVAHLRT		LRR5	4
601	LRHLSLAHN NIHSQVSQQLCSTS		LRR6	4
624	LRALDFSGN ALGHMWAEGDLYLHFFQGLSG		LRR7	3.4
654	LIWLDLSQN RLHTLLPQTLRNLPKS		LRR8	4
679	LQVLRLRDN YLAFFKWWSLHFLPK		LRR9	4
703	LEVLDLAGN QLKALTNGSLPAGTR		LRR10	4
727	LRRLDVSCN SISFVAPGFFSKAKE		LRR11	3.4
751	LRELNLSAN ALKTVDHSWFGPLASA		LRR12	4
776	LQILDVSAN				LRR13	3.4
785	PLHCACGAAFMDFLLEVQAAVPGLPSRVKCGSPGQLQGLSIFAQDLRLCLDE		CF1	4

SHIFT -17!

820	LQGLSIFAQ DLRLCLDEALSWDCFA		LRR1	2.4
845	LSLLAVALG LGVPMLHHLCGWDLWYCFH		LRR2	2.4
873	LCLAWLPWR GRQSGRDEDALPYDAFVVFDK		LRR3	2.4

TQSAVADWVYNELRGQLEECRGRWALRLCLEERDWLPGKTLFENLWASVYGSRKTLFVLA
HTDRVSGL

971	LRASFLLAQ QRLLEDRKDVVV			LRR1	2
992	LVILSPDGR RSRYVRLRQRLCRQSVLLWPH		LRR2	2

QPSGQRSFWAQLGMALTRDNHHFYNRNFCQGPTAE

>ENSP00000323096

MTWLVLLGTL

11	LCMLRVGLG TPDSEGFPPRALHN		LRR1	2.4
34	CPYKCICAADLLSCTGLGLQDVPAELPAA		NF	4

SHIFT -24!

39	ICAADLLSC TGLGLQDVPAELPAA		LRR2	2.2
63	TADLDLSHN ALQRLRPGWLAPLFQ		LRR3	3
87	LRALHLDHN ELDALGRGVFVNASG		LRR4	4
111	LRLLDLSSN TLRALGRHDLDGLGA		LRR5	4
135	LEKLLLFNN RLVHLDEHAFHGLRA		LRR6	4
159	LSHLYLGCN ELASFSFDHLHGLSATH		LRR7	4
185	LLTLDLSSN RLGHISVPELAALPAFL		LRR8	4
211	KNGLYLHNN				LRR9	3
220	PLPCDCRLYHLLQRWHQRGLSAVRDFAREYVCLAFKVPASRVRFFQHSRVFENC		CF1_short	3

SSAPALGLERPEEHLYALVGRSLRLYCNTSVPAMRIAWVSPQQELLRAPGSRDGSIAV

332	LADGSLAIG NVQEQHAGLFVCL			LRR1	2.4
354	ATGPRLHHN QTHEYNVSVHFPRPEPEAFNT		LRR2	2.4

GFTTLLGCAVGLV

397	LVLLYLFAP PCRCCRRACRCRRWPQTPSP		LRR1	3
426	LQELSAQSS VLSTTPPDAPSRKASVHKHVV		LRR2	2.8

F

457	LEPGRRGLN GRVQLAVAEEFDLYN		LRR1	2.4
481	PGGLQLKAG SESASSIGSEGPMTT		LRR2	2


>ENSP00000299194

MLAPGSSPGQRGRLALQWRQVSWITCWIA

30	LYAVEALPT 		LRR1	2.2
39	CPFSCKCDSRSLEVDCSGLGLTTVPPDVPAA		NF	4

SHIFT -16!

54	CSGLGLTTV PPDVPAATRTLL			LRR2	2
75	LLNNKLSAL PSWAFANLSSLQRLD		LRR3	2
99	LSNNFLDRL PRSIFGDLTNLTELQLRNNSI		LRR4	2

SHIFT -25!

104	LDRLPRSIF GDLTNLTELQLRNNSIRT		LRR1	2
131	LDRDLLRHS PLLRHLDLSINGLAQLPPG		LRR2	2.4
159	LFDGLLALR SLSLRSNRLQNLDRLTFEP		LRR3	2.4
187	LANLQLLQV GDNPWECDCNLREFKHWMEWF		LRR4	3

SYRGGRLDQLACTLPKELRGKDMRMVPMEMFNYCSQLEDENSSAGLDIPGPPCTKASPEP
AKPKPGAEPEPEPSTACPQKQRHRPASVRRAMGTVIIAGVVCGVVCIMMVVAAAYGCIYA
SLMAKYHRELKKRQPLMGDPEGEHEDQKQISSVA

>ENSP00000295057

MD

3	FLLLGLCLY WLLRRPSGVVLCLLGAC		LRR1	2.4
29	FQMLPAAPS G			LRR2	2.2
39	CPQLCRCEGRLLYCEALNLTEAPHNLSGL		NF	4

SHIFT -18!

50	LYCEALNLT EAPHNLSGLLGLSLRYNS		LRR3	2.4
77	LSELRAGQF TGLMQLTWLYLDHNHICSVQG		LRR4	2.4

SHIFT -19!

88	LMQLTWLYL DHNHICSVQGDAFQKLRR		LRR1	2.4
115	VKELTLSSN QITQLPNTTFRPMPN		LRR2	3.4
139	LRSVDLSYN KLQALAPDLFHGLRK		LRR3	3.4
163	LTTLHMRAN AIQFVPVRIFQDCRS		LRR4	3.4
187	LKFLDIGYN QLKSLARNSFAGLFK		LRR5	3.4
211	LTELHLEHN DLVKVNFAHFPRLIS		LRR6	4
235	LHSLCLRRN KVAIVVSSLDWVWN		LRR7	4
258	LEKMDLSGN EIEYMEPHVFETVPH		LRR8	3.4
282	LQSLQLDSN RLTYIEPRILNSWKS		LRR9	4
306	LTSITLAGN LWDCGRNVCALASWLNNFQGR		LRR10	3.4

YDGNLQCASPEYAQGEDVLDAVYAFHLCEDGAEPTSGHLLSAVTNRSDLGPPASSATTLA
DGGEGQHDGTFEPATVA

413	LPGGEHAEN AVQIHKVVTGTMALIFSFL		LRR1	2.4
441	IVVLVLYVS WKCFPASLRQLRQCFVTQRRK		LRR2	2.8

QKQKQTMHQMAAMSAQEYYVDYKPNHIEGALVIINEYGSCTCHQQPARECEV

>ENSP00000329380

M

2	PLLLLLLLL PSPLHPHPICEVS			LRR1	2
24	KVASHLEVN CDKRNLTALPPDLPKD		LRR2	2
49	TTILHLSEN LLYTFSLATLMPYTR		LRR3	3
73	LTQLNLDRC ELTKLQVDGTLPV			LRR4	3.4
95	LGTLDLSHN QLQSLPLLGQTLPA		LRR5	4
118	LTVLDVSFN RLTSLPLGALRGLGE		LRR6	3.4
142	LQELYLKGN ELKTLPPGLLTPTPK		LRR7	4
166	LEKLSLANN NLTELPAGLLNGLEN		LRR8	4
190	LDTLLLQEN SLYTIPKGFFGSHL		LRR9	4
213	LPFAFLHGN PWLCNCEILYFRRWLQDNAEN		LRR10	3.4

VYVWKQGVDVKAMTSNVASVQCDNSDKFPVYKYPGKGCPTLGDEGDTDLYDYYPEEDTEG
DKVRATRTVVKFPTKAHTTPWGLFYSWSTASLDSQMPSSLHPTQESTKEQTTFPPRWTPN
FTLHMESITFSKTPKSTTEPTPSPTTSEPPTPSPTTPEPTSEPAPSPTTPEPTSEPAPSP
TTPEPTPIPTIATSPTILVSATSLITPKSTFLTTTKPVSLLESTKKTIPELDQPPKLRG

482	VLQGHLESS RNDPFLHPDFCC			LRR1	2.2
503	LLPLGFYVL GLFWLLFASVVLILLLSWVGH		LRR2	2.4

SHIFT -11!

522	VLILLLSWV GHVKPQALDSGQGAALTTATQ		LRR1	2.4
552	TTHLELQRG RQVTVPRAWLLFLRGSLPTF		LRR2	2
581	RSSLFLWVR PNGRVGPLVAGRRPSALSQGR		LRR3	2

GQDLLSTVSIRYSGHSL

>XP_945324

MAPRAGQPG

10	LQGLLLVAA ALSQPAAP		LRR1	3
27	CPFQCYCFGGPKLLLRCASGAELRQPPRDVPP		NF	4

SHIFT -23!

36	GPKLLLRCA SGAELRQPPRDVPPDARN		LRR2	2.4
63	LTIVGANLT VLRAAAFAGGDGDGDQAA		LRR3	2.2
90	GVRLPLLSA LRLTHNHIEVVEDGAFDGLPS		LRR4	2.4
120	LAALDLSHN PLRALGGGAFRGLPA		LRR5	4
144	LRSLQLNHA LVRGGPALLAALDAALAPLAE		LRR6	3
174	LRLLGLAGN ALSRLPPAALRLAR		LRR7	4
197	LEQLDVRLN ALAGLDPDELRALERDGGLP		LRR8	3.4
226	GPRLLLADN				LRR9	3.4
235	PLRCGCAARPLLAWLRNATERVPDSRRLRCAAPRALLDRPLLDLDGARLRCA		CF1_short	3

SHIFT -44!

243	RPLLAWLRN ATERVPDSRRLRCAAPRA		LRR1	2.4
270	LLDRPLLDL DGARLRCADSGADARGEEAEA		LRR2	2

AGPELEASYVFFGLVLALIGLIFLMVLYLNRRGIQRWMRNLREACRDQMEGYHYRYEQDA
DPRRAPAPAAPAGSRATSPGSGL

>ENSP00000246841

MDLRDWLFLCYGLIAFLTEVIDSTT

26	CPSVCRCDNGFIYCNDRGLTSIPADIPDD		NF	4
55	ATTLYLQNN QINNAGIPQDLKTKVN		LRR1	3.4
80	VQVIYLYEN DLDEFPINLPRS			LRR2	2.8
101	LRELHLQDN NVRTIARDSLARIPL		LRR3	4
125	LEKLHLDDN SVSTVSIEEDAFADSKQ		LRR4	4
151	LKLLFLSRN HLSSIPSGLPHT			LRR5	4
172	LEELRLDDN RISTIPLHAFKGLNS		LRR6	4
196	LRRLVLDGN LLANQRIADDTFSRLQN		LRR7	4
222	LTELSLVRN SLAAPPLNLPSAH			LRR8	4
244	LQKLYLQDN AISHIPYNTLAKMRE		LRR9	4
268	LERLDLSNN NLTTLPRGLFDDLGN		LRR10	4
292	LAQLLLRNN				LRR11	4
301	PWFCGCNLMWLRDWVKARAAVVNVRGLMCQGPEKVRGMAIKDITSEMDECF		CF1_short	3

ETGPQGGVANAAAKTTASNHASATTPQGSLFTLKAKRPGLRLPDSNIDYPMATGDGAKTL
AIHVKALTADSIRITWKATLPASSFR

438	LSWLRLGHS PAVGSITETLVQGDKTEYL		LRR1	3.4
466	LTALEPKST YIICMVTMETSNAYVADETPV		LRR2	2.4

CAKAETADSYGP

508	TTTLNQEQN AGPMASLPLAGIIGGAVA		LRR1	2
535	LVFLFLVLG AICWYVHQAGELLTRERAYNR		LRR2	3

GSRKKDDYMESGTKKDNSILEIRGPGLQMLPINPYRAKEEYVVHTIFPSNGSSLCKATHT
IGYGTTRGYRDGGIPDIDYSYT

>ENSP00000261172

MKT

4	LAGLVLGLV IFDAAVTAPTLESINYDSETY		LRR1	3
34	DATLEDLDN LYNYENIPVDKVEIEIATVMP		LRR2	2

SGNRELLTPPPQPEKAQEEEEEEESTPRLIDGSSPQEPEFTGVLGPHTNEDFPTCLLCTC
ISTTVYCDDHEL

118	CLLCTCISTTVYCDDHELDAIPPLPKNT		NF	4

SHIFT -10!

136	DAIPPLPKN TAYFYSRFNRIKKINKNDFAS		LRR1	2
166	LSDLKRIDL TSNLISEIDEDAFRKLPQ		LRR2	2
193	LRELVLRDN KIRQLPELPTTLTFIDISNNR		LRR3	4

SHIFT -10!

213	LTFIDISNN RLGRKGIKQEAFKDMYD		LRR1	2.8
239	LHHLYLTDN NLDHIPLPLPEN			LRR2	4
260	LRALHLQNN NILEMHEDTFCNVKNLTYIRK		LRR3	4

A

291	LEDIRLDGN PINLSKTPQAYMC			LRR1	3.4
313	LPRLPVGSL V			LRR2	2.4


>ENSP00000330864

MLRSPTFTDAGPRCSCLPVSQTLDSMDTVLMGSLQHCCCLLPKMGDTWAQLPWPGPPHPA
MLLISLLLAAGLMHSDAGTS

81	CPVLCTCRNQVVDCSSQRLFSVPPDLPMD		NF	4

SHIFT -29!

81	CPVLCTCRN QVVDCSSQRLFSVPPDLPMD		LRR1	2
110	TRNLSLAHN RITAVPPGYLTCYME		LRR2	3
134	LQVLDLHNN SLMELPRGLFLHAKR		LRR3	4
158	LAHLDLSYN NFSHVPADMFQEAHG		LRR4	4
182	LVHIDLSHN PWLRRVHPQAFQGLMQ		LRR5	3.4
207	LRDLDLSYG GLAFLSLEALEGLPG		LRR6	3
231	LVTLQIGGN				LRR7	3.4
240	PWVCGCTMEPLLKWLRNRIQRCTADSQLAECRGPPEVEGAPLFSLTEESFKAC		CF1_short	3

SHIFT -42!

251	LKWLRNRIQ RCTADSQLAECRGPPEVEGAP		LRR1	2
281	LFSLTEESF KACHLTLTLDDYLFIAFVGFV		LRR2	2
311	VSIASVATN FLLGITANCCHRWSKASEEEE		LRR3	2.2

I

>ENSP00000354207

MDVSLCPAKCSFWRIFLLGSVWLDYV

27	GSVLACPAN CVCSKTEINCRRPDDGNLFP		LRR1	2.4
56	LLEGQDSGN SNGNASINITDISRNITSIHI		LRR2	2.4

ENWRSLHTLNAVDMELYTG

105	LQKLTIKNS GLRSIQPRAFAKNPH		LRR1	2.8
129	LRYINLSSN RLTTLSWQLFQTLS		LRR2	3.4
152	LRELQLEQN				LRR3	4
161	FFNCSCDIRWMQLWQEQGEAKLNSQNLYCINADGSQLPLFRMNISQCDLP		CF1	4

SHIFT -59!

152	LRELQLEQN FFNCSCDIRWMQLWQEQGEAK		LRR3	4
182	LNSQNLYCI NADGSQLPLFRMNISQCDLPE		LRR4	2

SHIFT -18!

194	GSQLPLFRM NISQCDLPEISVSHVNLTVRE		LRR1	2.4
224	GDNAVITCN GSGSPLPDVDWIVTGLQSINT		LRR2	2.2

SHIFT -16!

238	LPDVDWIVT GLQSINTHQTNLNWTNVH		LRR1	2.2
265	AINLTLVNV TSEDNGFTLTCI			LRR2	2.4
286	AENVVGMSN ASVALTVYYPPRVVSLE		LRR3	2.2
312	EPELRLEHC IEFVVRGNPPPT			LRR4	2.4
333	LHWLHNGQP LRESKIIHVEYYQEGE		LRR5	2
358	ISEGCLLFN KPTHYNNGNYTLIAKNP		LRR6	2.8
384	LGTANQTIN GHFLKEPFPESTDNFILFDEV		LRR7	2.4

SPTPPITVTHKPEEDTFGVSIAVGLAAFACVLLVVLFVMINKYGRRSKFGMKGPVAVISG
EEDSASPLHHINHGITTPSSLDAGPDTVVIGMTRIPVIENPQYFRQGHNCHKPDTYVQHI
KRRDIVLKRELGEGA

549	FGKVFLAEC YNLSPTKDKMLVAVKA		LRR1	2.2
574	LKDPTLAAR KDFQREAELLTNLQHEHIVKF		LRR2	2

YGVCGDGDPLIMVFEYMKHGDLNKFLRAHGPDAMILVDGQPRQAKGELGLSQMLHIASQI
ASGMVYLASQHFVHRDLATRNCLVGANLLVKIGDFGMSRDVYSTDYYRLFNPSGNDFCIW
CEVGGHTMLPIRWMPPESIMYRKFTTESDVWSFGVILWEIFTYGKQPWFQLSNTEVIECI
TQGRVLERPRVCPKEVYDVMLGCWQREPQQRLNIKEIYKILHALGKATPIYLDILG

>ENSP00000298386

MIVFLVFKHLFSLRLITMFF

21	LLHFIVLIN VKDFALTQGSMITPSCQKGY		LRR1	2.8
50	FPCGNLTKC LPRAFHCDGKDDCGNGADEEN		LRR2	2.2

CGDTSGWATIFGTVHGNANSVALTQE

106	CFLKQYPQCCDCKETELECVNGDLKSVPMISNN		NF	4
139	VTLLSLKKN KIHSLPDKVFIKYTK		LRR1	3.4
163	LKKIFLQHN CIRHISRKAFFGLCN		LRR2	3.4
187	LQILYLNHN CITTLRPGIFKDLHQ		LRR3	4
211	LTWLILDDN PITRISQRLFTGLNS		LRR4	4
235	LFFLSMVNN YLEALPKQMCAQMPQ		LRR5	3.4
259	LNWVDLEGN RIKYLTNSTFLSCDS		LRR6	3.4
283	LTVLFLPRN QIGFVPEKTFSSLKN		LRR7	4
307	LGELDLSSN TITELSPHLFKDLKL		LRR8	4
331	LQKLNLSSN PLMYLHKNQFESLKQ		LRR9	4
355	LQSLDLERI EIPNINTRMFQPMKNLSHIYF		LRR10	3

KNFRYCSYAPHVRICMPLTDGISSFEDLLANNILRIFVWVIAFITCFGNLFVIGMRSFIK
AENTTHAMSIKILCCADCLMGVYLFFVGIFDIKYRGQYQKYALLWMESVQCR

497	LMGFLAMLS TEVSVLLLTYLTLEKF		LRR1	2.2
522	LVIVFPFSN IRPGKRQTSVILICIWMAGF		LRR2	2.4
551	LIAVIPFWN KDYFGNFYGKNGVCFPLYYDQ		LRR3	2.4

TEDIGSKGYSLGIFLGVNLLAFLIIVFSYITMFCSIQKTALQTTEVRNCFGREVAVANRF
FFIVFSDAICWIPVFVVKILS

662	LFRVEIPDT MTSWIVIFFLPVNSALN		LRR1	2.2
688	PILYTLTTN FFKDKLKQLLHKHQRKSIFKI		LRR2	2

SHIFT -16!

702	LKQLLHKHQ RKSIFKIKKKSLSTSIVWIED		LRR1	2
732	SSSLKLGVL NKITLGDSIMKPVS		LRR2	2


>ENSP00000252804

SRPWWLRASERPSAPSAMAKRSRGPGRRCLLALVLFCAWGTLAVVAQKPGAG

53	CPSRCLCFRTTVRCMHLLLEAVPAVAPQ		NF	4
81	TSILDLRFN RIREIQPGAFRRLRN		LRR1	3
105	LNTLLLNNN QIKRIPSGAFEDLEN		LRR2	4
129	LKYLYLYKN EIQSIDRQAFKGLAS		LRR3	4
153	LEQLYLHFN QIETLDPDSFQHLPK		LRR4	4
177	LERLFLHNN RITHLVPGTFNHLES		LRR5	4
201	MKRLRLDSN				LRR6	3.4
210	TLHCDCEILWLADLLKTYAESGNAQAAAICEYPRRIQGRSVATITPEELNCE		CF1_short	3

SHIFT -61!

201	MKRLRLDSN TLHCDCEILWLADL		LRR6	3.4
224	LKTYAESGN AQAAAICEYPRRIQGRSVATI		LRR7	2

TPEELNCERPRITSEPQDADVTSGNTVYFTCRAEGNPK

292	PEIIWLRNN NELSMKTDSRLNL			LRR1	2.4
314	LDDGTLMIQ NTQETDQGIYQCMAKNVAGEV		LRR2	2.4

KTQEVTLRYFGSPARPTFVIQPQNTEVLVGESVTLECSATGHPPPRISWTRGDRTPLPVD
PRVNITPSGGLYIQNVVQGDSGEYACSATNNIDSVHATAFIIVQALPQFTVTPQDRVVIE
GQTVDFQCEAKGNPPPVIAWTKGGSQLSVDRRHLVLSSGTLRISGVALHDQGQYECQAVN
IIGSQKVVAHLTVQPRVTPVFASIPSDTTVEVGANVQLPCSSQGEPEPAITWNKDGVQVT
ESGKFHI

591	SPEGFLTIN DVGPADAGRYECVARNTIGSA		LRR1	2.4
621	SVSMVLSVN VPDVSRNGDPFVATSIVEAIA		LRR2	2.4

TVDRAINSTRTHLFDSRPRSP

672	NDLLALFRY PRDPYTVEQARAGEIF		LRR1	2
697	ERTLQLIQE HVQHGLMVDLNGTSYHYNDLV		LRR2	2

SPQYLNLIANLSGCTAHRRVNNCSDMCFHQKYRTHDGTCNNLQHPMWGASLTAFERLLKS
VYENGFNTPRGINPHRLYNGHALPMPRLVSTTLIGTETVTPDEQFTHMLMQWGQFLDHDL
DSTVVALSQARFSDGQHCSNVCSNDPPCFSVMIPPNDSRARSGARCMFFVRSSPVCGSGM
TSLLMNSVYPREQINQ

923	LTSYIDASN VYGSTEHEARSIRDLASHRGL		LRR1	2
953	LRQGIVQRS GKPLLPFATGPPTECMRDENE		LRR2	2.2

SHIFT -17!

966	LPFATGPPT ECMRDENESPIPCFLAGDHRA		LRR1	2.2
996	NEQLGLTSM HTLWFREHNRIA			LRR2	2
1017	TELLKLNPH WDGDTIYYETRKIVGAEIQHI		LRR3	2

TYQHWLPKILGEVGMRTLGEYHGYDPGINAGIFNAFATAAFRFGHTLVNPLLYRLDENFQ
PIA

1110	QDHLPLHKA FFSPFRIVNEGGIDPL		LRR1	2
1135	LRGLFGVAG KMRVPSQLLNTELTERLFSMA		LRR2	2.4

HTVALDLAAINIQRGRDHGIPPYHDYRVYCNLSAAHTFEDLKNEIKNPEIREKLKR

1221	LYGSTLNID LFPALVVEDLVPGSRLGPT		LRR1	2
1249	LMCLLSTQF KRLRDGDRLWYENPGVFSPAQ		LRR2	2

LTQIKQTSLARILCDNADNITRVQSDVFRVAEFPHGYGSCDEIPRVDLRVWQDCCEDCRT
RGQFNAFSYHFRGRRSLEFSYQEDKPTKKTRPRKIPSVGRQGEHLSNSTSAFSTRSDASG
TNDFREFVLEMQKTITDLRTQIKKLESRLSTTECVDAGGESHANNTKWKKDACTICECKD
GQVTCFVEACPPATCAVPVNIPGACCPVCLQKRAEEKP

>ENSP00000282466

MKVKGRGITCLLVSFAVICLVATPGGKA

29	CPRRCACYMPTEVHCTFRYLTSIPDSIPPN		NF	4
59	VERINLGYN SLVRLMETDFSGLTK		LRR1	2.8
83	LELLMLHSN GIHTIPDKTFSDLQA		LRR2	4
107	LQVLKMSYN KVRKLQKDTFYGLRS		LRR3	3.4
131	LTRLHMDHN NIEFINPEVFYGLNF		LRR4	3.4
155	LRLVHLEGN QLTKLHPDTFVS			LRR5	3.4
176	LSYLQIFKI SFIKFLYLSDNF			LRR6	2.4
197	LTSLPQEMV SYMPDLDSLYLHGNPWTCDCH		LRR7	2
227	LKWLSDWIQ EKPDVIKCKKDRSPSS		LRR8	2
252	AQQCPLCMN PRTSKGKPLAMVSAAAFQCAK		LRR9	2.4

PTIDSS

288	LKSKSLTIL EDSSSAFISPQGFM		LRR1	2
311	APFGSLTLN MTDQSGNEANMVCSIQKPSRT		LRR2	2.8

SPIAFTEENDYIVLNT

357	SFSTFLVCN IDYGHIQPVWQILALYS		LRR1	2
383	DSPLILERS HLLSETPQLYYKYKQVAPKPE		LRR2	2.4

DIFTNIEADLRADPSWLMQDQISLQLNRTATTFSTLQIQYSSDAQITLPRAEMRPVKHKW
TMISRDNNTK

483	LEHTVLVGG TVGLNCPGQGDPTPH		LRR1	2
507	VDWLLADGS KVRAPYVSEDGRILIDK		LRR2	2.2
533	SGKLELQMA DSFDTGVYHCISSNYDDADIL		LRR3	2

TYRITVVEPLVEAYQENGIHHTVFIGETLDLPCHSTGIPDASISWVIPGNNVLYQSSRDK
KV

625	LNNGTLRIL QVTPKDQGYYRCVAANPSG		LRR1	2.4
653	VDFLIFQVS VKMKGQRPLEHDGETEGSGLD		LRR2	2.2

ESNPIAHLKEPPGAQ

698	LRTSALMEA EVGKHTSSTSKRHN		LRR1	2
721	YRELTLQRR GDSTHRRFRENRRHFPPSARR		LRR2	2

IDPQHWAALLEKAKKNAMPDKRENTTVSPPPVVTQLPNIPGEEDDSSGMLALHEEFMVPA
TKALNLPARTVTADSRTISDSPMTNINYGTEFSPVVNSQILPPEEPTDFKLSTAIKTTAM
SKNINPTMSSQIQGTTNQHSSTVFPLLLGATEFQDSDQMGRGREHFQSRPPITVRTMIKD
VNVKMLSSTTNKLLLESVNTTNSHQTSVREVSEPRHNHFYSHTTQILSTSTFPSDPHTAA
HSQFPIPRNSTVNIPLFRRFGRQRKIGGRGRIISPYRTPVLRRHRYSIFRSTTRGSSEKS
TTAFSATVLNVTCLSCLPRER

1072	LTTATAALS FPSAAPITFPKADIARVPS		LRR1	2.2
1100	EESTTLVQN PLLLLENKPSVEKTTPTIKYF		LRR2	2

RTEISQVTPTGAVMTYAPTSIPMEKTHKVNASYPRVSSTNEAKRDSVITSSLSGAITKPP
MTIIAITRFSRRKIPWQQNFVNNHNPKGRLRNQHKVSLQKSTAVMLPKTSPALPRDKVSP
FHFTTLSTSVMQIPSNTLTTAHHTTTKTHNPGSLPTKKELPFPPLNPMLPSIISKDSSTK
SIISTQTAIPATTPTFPASVITYETQTERSRAQTIQREQEPQKKNRTDPNISPDQSSGFT
TPTAMTPPVLTTAETSVKPSVSAFTHSPPENTTGISSTISFHSRTLNLTDVIEELAQAST
QTLKSTIASETTLSSKSHQSTTTRKAIIRHSTIPPFLSSSATLMPVPISPPFTQRAVTDN
VATPISGLMTNTVVKLHESSRHNAKPQQLVAEVATSPKVHPNAKFTIGTTHFIYSNLLHS
TPMPALTTVKSQNSK

1565	LTPSPWAEN QFWHKPYSEIAEKGKKPEVSM		LRR1	2.4
1595	LATTGLSEA TTLVSDWDGQKNTKKSDFDKK		LRR2	2

SHIFT -18!

1607	VSDWDGQKN TKKSDFDKKPVQEATTSKL		LRR1	2.2
1635	LPFDSLSRY IFEKPRIVGGKAASFTIPANS		LRR2	2

DAFLPCEAVGNPLPTIHWTRVPSG

1689	LDLSKRKQN SRVQVLPNGTLSIQRVEIQDR		LRR1	2
1719	GQYLCSASN LFGTDHLHVTLSVVSYPPRIL		LRR2	2.4

ERRTKEITVHSGSTVELKCRAEGRPSPTVTWILANQTVVSESSQGSRQAVVTVDGTLVLH
NLSIYDRGFYKCVASNPGGQDSLLVKIQVIAAPPVILEQRRQVIVGTW

1857	GESLKLPCT AKGTPQPSVYWVLSDGTE		LRR1	2.8
1884	VKPLQFTNS KLFLFSNGTLYIRNLASSDRG		LRR2	2.2

TYECIATSSTGSERRVVMLTMEERVTSPRIEAASQKRTEVNFGDKLLLNCSATGEPKPQI
MWRLPSKAVVDQQHRV

1990	GSWIHVYPN GSLFIGSVTEKDS			LRR1	2.2
2012	GVYLCVARN KMGDDLILMHVSLRLKPAKID		LRR2	2.8

HKQYFRKQVLHGKDFQVDCKASGSPVPEISWS

2074	LPDGTMINN AMQADDSGHRTRRYTL		LRR1	2.8
2099	FNNGTLYFN KVGVAEEGDYTCYAQNTLGKD		LRR2	2.8

EMKVHLTVITAAPRIRQSNKTNKRIKAGDTAVLDCEVTGDPKP

2172	KIFWLLPSN DMISFSIDRYTF			LRR1	2.4
2193	HANGSLTIN KVKLLDSGEYVCVARNPSGDD		LRR2	2.4

SHIFT -14!

2209	GEYVCVARN PSGDDTKMYKLDVVSKP		LRR1	2.2
2235	PLINGLYTN RTVIKATAVRHSKKHFDCRAE		LRR2	2

GTPSPEVMWIMPDNIFLTAPYY

2287	GSRITVHKN GTLEIRNVRLSDS			LRR1	2.2
2309	ADFICVARN EGGESVLVVQLEV			LRR2	2.2
2331	LEMLRRPTF RNPFNEKIVAQLGKSTALNCS		LRR3	2

VDGNPPPEIIWILPNGTRFSNGPQSYQYLIASNGSFIISKTTREDAGKYRCAARNKVGYI
EK

2423	LVILEIGQK PVILTYAPGTVKGIS		LRR1	2.4
2447	GESLSLHCV SDGIPKPNIKWTMPSGYVVDR		LRR2	2.4

PQINGKYILHDNGTLVIKEATAYDRGNYICKAQNSVGHTLITVPVMIVAYPPRITNRPPR
SIVTRTGAAFQLHCVALGVPKPEITWEMPDHSLLSTASKERTHGSEQLHLQGTLVIQNPQ
TSDSGIYKCTAKNPLGSDYAATYIQVI

>ENSP00000335617

MEMYGKHTVGP

12	RNCEILREN NLTELHKDSFEGLLS		LRR1	2
36	LQYLDLSCN KIQSIERHTFEPLPF		LRR2	4
60	LKFINLSCN VITELSFGTFQAWHGMQF		LRR3	3.4
87	LHKLILNHN PLTTVEDPYLFKLPA		LRR4	4
111	LKYLDMGTT LVPLTTLKNILMMTVE		LRR5	2.8
136	LEKLILPSH				LRR6	3
145	MACCLCQFKNSIEAVCKTVKLHCNSACLTNTTH		CF3	3

SHIFT -42!

136	LEKLILPSH MACCLCQFKNSIEAV		LRR6	3
160	CKTVKLHCN SACLTNTTHCPEEASVGNPEG		LRR7	2.4

AF

192	MKVLQARKN YTSTELIVEPEEPSDSS		LRR1	2.8
218	GINLSGFGS EQLDTNDESDFISTLSYILPY		LRR2	2.2

SHIFT -24!

224	FGSEQLDTN DESDFISTLSYILPYFSAVN		LRR1	2.4
253	LDVKSLLLP FIKLPTTGNSLAKIQTVGQNR		LRR2	2

QRVKRVLMGPRSIQKRHFKEVGRQSIRREQGAQASVENAAEEKRLGSPAPREVEQPHTQQ
GPEKLAGNAVYTKPSFTQEHKAAVSVLKPFSKGAPSTSSPAKALPQVRDRWKDLTHAISI
LESAKARVTNTKTSKPIVHARKKYRFHKTRSHVTHRTPKVKKSPKVRKKSY

454	LSRLMLANR LPFSAAKSLINSPSQGAFSS		LRR1	3
483	LGDLSPQEN PFLEVSAPSEHFIENNNTKHT		LRR2	3

TARNAFEENDFMENTN

529	MPEGTISEN TNYNHPHEADSA			LRR1	2.2
550	GTAFNLGPT VKQTETKWEYNNVGTD		LRR2	2.2
575	LSPEPKSFN YPLLSSPGDQFEIQLTQQ		LRR3	2
602	LQSLIPNNN VRRLIAHVIRTLKMDCSGAHV		LRR4	3

SHIFT -13!

619	IRTLKMDCS GAHVQVTCAKLISRTGH		LRR1	2.2
645	LMKLLSGQQ EVKASKIEWDTDQWKIENYIN		LRR2	2

ESTEAQSEQK

685	EKSLELKKE VPGYGYTDKLILALIVTGI		LRR1	2
713	LTILIILFC LIVICCHRRSLQEDEEGFSRG		LRR2	2.8

SHIFT -27!

716	LIILFCLIV ICCHRRSLQEDEEGFSRGI		LRR1	2
744	FRFLPWRGC SSRRESQDGLSSFGQPLWFKD		LRR2	2.2

MYKPLSATRINNHAWKLHKKSSNEDKILNRDPGDSEAPTEEEESEALP

>ENSP00000308315

MEGARARGAQLRLGERVRPVGRRSAPGRSRFRQPWRPGASDSAPPAGTMAQS

53	RVLLLLLLL PPQLHLGPVLAVRAPGFGRSG		LRR1	2
83	GHSLSPEEN EFAEEEPVLVLSPEEPGPGPA		LRR2	2.4

AVS

116	CPRDCACSQEGVVDCGGIDLREFPGDLPEH		NF	4

SHIFT -7!

139	PGDLPEHTN HLSLQNNQLEKIYPEE		LRR1	2
164	LSRLHRLET LNLQNNRLTSRGLPEKAFEH		LRR2	2.4
193	LTNLNYLYL ANNKLTLAPRFLPNA		LRR3	2
217	LISVDFAAN YLTKIYGLTFGQKPN		LRR4	2.8
241	LRSVYLHNN KLADAGLPDNMFNGSSN		LRR5	3.4
267	VEVLILSSN FLRHVPKHLPPA			LRR6	3.4
288	LYKLHLKNN KLEKIPPGAFSELSS		LRR7	4
312	LRELYLQNN YLTDEGLDNETFWKLSS		LRR8	4
338	LEYLDLSSN NLSRVPAGLPRS			LRR9	4
359	LVLLHLEKN AIRSVDANVLTPIRS		LRR10	4
383	LEYLLLHSN QLREQGIHPLAFQGLKR		LRR11	4
409	LHTVHLYNN ALERVPSGLPRR			LRR12	3.4
430	VRTLMILHN QITGIGREDFATTYF		LRR13	2.8
454	LEELNLSYN RITSPQVHRDAFRKLRL		LRR14	4
480	LRSLDLSGN RLHTLPPGLPRN			LRR15	4
501	VHVLKVKRN ELAALARGALVGMAQ		LRR16	2.8
525	LRELYLTSN RLRSRALGPRAWVDLAH		LRR17	4
551	LQLLDIAGN QLTEIPEGLPES			LRR18	3.4
572	LEYLYLQNN KISAVPANAFDSTPN		LRR19	4
596	LKGIFLRFN KLAVGSVVDSAFRRLKH		LRR20	3.4
622	LQVLDIEGN LEFGDISKDRGRLGKEKEEEE		LRR21	3.4

EEEEEEEETR

>ENSP00000223657

MK

3	EYVLLLFLA LCSAKPFFSPSHIA		LRR1	2
26	LKNMMLKDM EDTDDDDDDDDDDDDDDEDNS		LRR2	2.4

LFPTREPRSHFFPFDLFPM

75	CPFGCQCYSRVVHCSDLGLTSVPTNIPFD		NF	4
104	TRMLDLQNN KIKEIKENDFKGLTS		LRR1	3
128	LYGLILNNN KLTKIHPKAFLTTKK		LRR2	4
152	LRRLYLSHN QLSEIPLNLPKS			LRR3	4
173	LAELRIHEN KVKKIQKDTFKGMNA		LRR4	3.4
197	LHVLEMSAN PLDNNGIEPGAFEGVTVFHIR		LRR5	3.4

IAEAKLTSVPKG

239	LPPTLLELH LDYNKISTVELEDFKRYKE		LRR1	2
267	LQRLGLGNN KITDIENGSLANIPR		LRR2	4
291	VREIHLENN KLKKIPSGLPELKY		LRR3	2.8
314	LQIIFLHSN SIARVGVNDFCPTVPKMKKS		LRR4	3.4
343	LYSAISLFN NPVKYWEMQPATFRCVLSRMS		LRR5	2.4

VQLGNFGM

>ENSP00000273286

MKGELLLFSSVIVLLQVV

19	CSCPDKCYCQSSTNFVDCSQQGLAEIPSHLPPQ		NF	4
52	TRTLHLQDN QIHHLPAFAFRSVPW		LRR1	3
76	LMTLNLSNN SLSNLAPGAFHGLQH		LRR2	4
100	LQVLNLTQN SLLSLESRLFHSLPQ		LRR3	4
124	LRELDLSSN NISHLPTSLGETWEN		LRR4	4
148	LTILAVQQN QLQQLDRALLESMPS		LRR5	3.4
172	VRLLLLKDN				LRR6	3.4
181	LWKCNCHLLGLKLWLEKFVYKGGLTDGIICESPDTWKGKDLLRIPHELYQPC		CF1_short	3

SHIFT -59!

174	LLLLKDNLW KCNCHLLGLKLWLEKFVYKGG		LRR1	2
204	LTDGIICES PDTWKGKDLLRIPHELYQPCP		LRR2	2.2

LPAPDPVSSQAQWPGSAH

252	GVVLRPPEN HNAGERELLECE			LRR1	2.4
273	LKPKPRPAN LRHAIATVIITGVVCGIVC		LRR2	2
301	LMMLAAAIY GCTYAAITAQYHGGPLAQTND		LRR3	2.4

PGKVEEKERFDSSPA

>OTTHUMP00000028578

MGSGPRGALSLLLLLLAPPSRPAAG

26	CPAPCSCAGTLVDCGRRGLTWASLPTAFP		NF	4

SHIFT -11!

44	LTWASLPTA FPVDTTELVLTGNNLTA		LRR1	2.4
70	LPPGLLDAL PALRTAHLGANPWRCDCR		LRR2	2.4
97	LVPLRAWLA GRPERAPYRDLRCVAPPA		LRR3	2.4
124	LRGRLLPYL AEDELRAACAPGP			LRR4	2
146	LCWGALAAQ LALLGLGLLHAL			LRR5	2.4
167	LLVLLLCRL RRLRARARARAAARLSLTDPL		LRR6	3

VAERAGTDES

>ENSP00000343233

MRLLAFLSLLALVLQETGTASLPRKERKRREEQMPREGDSFEVLPLRNDVLNPDNYGEVI
DLSNYEELTDYGDQLPEVKVTSLAPATSISPAKSTTAPGTPSSNPTMTRPTTAGLLLSSQ
PNHGLPT

128	CLVCVCLGSSVYCDDIDLEDIPPLPRR		NF	4

SHIFT -26!

129	LVCVCLGSS VYCDDIDLEDIPPLPRR		LRR1	2.8
155	TAYLYARFN RISRIRAEDFKGLTK		LRR2	2.4
179	LKRIDLSNN LISSIDNDAFRLLHA		LRR3	3.4
203	LQDLILPEN QLEALPVLPSGIEFLDVR		LRR4	4
230	LNRLQSSGI QPAAFRAMEKLQFLY		LRR5	2
254	LSDNLLDSI PGPLPLSLRSVHLQNNLIETM		LRR6	2

QRDVFCDPEEHKHTRRQ

301	LEDIRLDGN PINLSLFPSAYFC			LRR1	3.4
323	LPRLPIGRF T			LRR2	2.4


>Y39A1A.7

MTSITSAFLLLLLFVAVACRS

22	VPCLDGCTC ELSEHDPVIRCDGVG		LRR1	2.2
46	LNRFPLPHS SPLRGFHFLALTCNDIDTIPA		LRR2	2.8

SHIFT -16!

60	FHFLALTCN DIDTIPAISMIKASFPD		LRR1	3.4
86	LQGIDVQGN TRLNCSDLVHLHKD		LRR2	2.8
109	IPVLSDCDN EKPLQCDNLDKNCDWKCRT		LRR3	2.4
137	LTKLKEMWA QFKDLVNRKAKEWQAEETIEA		LRR4	2

AKSWFSAQFKKFKIAIGELSD

>ENSP00000330698

MKVTGITILFWPLSMILLSDKIQSSKREVQCNFTEKNYTLIPADIKKD

49	VTILDLSYN QITLNGTDTRVLQTYFL		LRR1	3.4
75	LTELYLIEN KVTILHNNGFGNLSS		LRR2	4
99	LEILNICRN SIYVIQQGAFLGLNK		LRR3	3.4
123	LKQLYLCQN KIEQLNADVFVPLRS		LRR4	4
147	LKLLNLQGN LISYLDVPPLFH			LRR5	4
168	LELITLYGN				LRR6	3.4
177	LWNCSCSLFNLQNWLNTSNVTLENENITMCSYPNSLQSYNIKTVPHKAECHS		CF1_short	3

KFPSSVTEDLYIHFQPISNSIFN

252	SSSNNLTRN SEHEPLGKSWAFLVGVVVTV		LRR1	2
281	LTTSLLIFI AIKCPIWYNILLSYNHHRLEE		LRR2	2

HEAETYEDGFTGNPSSLSQIPETNSEETTVIFEQLHSFVVDDDGFIEDKYIDIHELCEEN

>ENSP00000344242

MRVVTIVILLCFCKAAELRKASPGSVRSRVNHGRAGGGRRGSNPVKRYAPGLPCDVYTY

60	LHEKYLDCQ ERKLVYVLPGWPQD		LRR1	2
83	LLHMLLARN KIRTLKNNMFSKFKK		LRR2	3.4
107	LKSLDLQQN EISKIESEAFFGLNK		LRR3	4
131	LTTLLLQHN QIKVLTEEVFIYTPL		LRR4	4
155	LSYLRLYDN				LRR5	4
164	PWHCTCEIETLISMLQIPRNRNLGNYAKCESPQEQKNKKLRQIKSEQLCNE		CF1_short	3

EEKEQLDPKPQVSGRPPVIKPEVDSTFCHNYVFPIQTLDCKRKELKKVPNNIPPD

270	IVKLDLSYN KINQLRPKEFEDVHE		LRR1	3.4
294	LKKLNLSSN GIEFIDPAAFLGLTH		LRR2	4
318	LEELDLSNN SLQNFDYGVLEDLYF		LRR3	4
342	LKLLWLRDN PWRCDYNIHYLYYWLKHHYNV		LRR4	4

HFNGLECKTPEEYKGWSVGKYIRSYYEECPKDKLPAYPESFDQDTEDDEWEKKHRDHTAK
KQSVIITIVG

>ENSP00000339255

MALRA

6	PALLPLLLL LLPLRAAG		LRR1	2
23	CPAACRCYSATVECGALRLRVVPLGIPPG		NF	4

SHIFT -16!

36	CGALRLRVV PLGIPPGTQTLFLQDNNIAR		LRR2	2
65	LEPGALAPL AALRRLYLHNNS			LRR3	2.4
86	LRALEAGAF RAQPRLLELALTSNR		LRR4	2.4
110	LRGLRSGAF VGLAQLRVLYLAGNQLARLLD		LRR5	2
140	FTFLHLPRL QELHLQENSIELLEDQALAG		LRR6	2.4
169	LSSLALLDL SRNQLGTISREALQPLAS		LRR7	3
196	LQVLRLTEN				LRR8	4
205	PWRCDCALHWLGAWIKEGGQRLLTSRDRKIMCAEPPRLALQSLLDVSHSSLICI		CF1_short	3

SHIFT -47!

212	LHWLGAWIK EGGQRLLTSRDRKIMCAE		LRR1	2.4
239	PPRLALQSL LDVSHSSLICIPPSVH		LRR2	2
264	VQPLELTAN LGEDLRVACQASGYPQPLVTW		LRR3	3.4

RKVPQPREGRPRAQAQ

310	LEGGLLGLG GHSASDTGSGMLF			LRR1	2.4
332	LSNITLAHA GKYECEASNAGGAA		LRR2	2.4
355	RVPFRLLVN ASRQQPQQPAQPPPPAARPAG		LRR3	2.4

SEPRPEAGSMAFRALGVATQTAIA

409	AAIALLALT ALLLVAMICRRRRRRKKARGP		LRR1	2.2
439	PGEGALFVN DYLDGPCTFAQLEELRDERGH		LRR2	2.4

EMFVINRSKPLFAEGPAEAPADCGPEQGAGPGLRVPPPVAYEIHC

>ENSP00000306276

MPLKHYLLLLVGCQAWGAGLAYHG

25	CPSECTCSRASQVECTGARIVAVPTPLPWN		NF	4

SHIFT -9!

46	AVPTPLPWN AMSLQILNTHITELNESPF		LRR1	2.4
74	LNISALIAL RIEKNELSRITPGAFRNLGS		LRR2	2
103	LRYLSLANN KLQVLPIGLFQGLDS		LRR3	4
127	LESLLLSSN QLLQIQPAHFSQCSN		LRR4	4
151	LKELQLHGN HLEYIPDGAFDHLVG		LRR5	4
175	LTKLNLGKN SLTHISPRVFQHLGN		LRR6	4
199	LQVLRLYEN RLTDIPMGTFDGLVN		LRR7	4
223	LQELALQQN QIGLLSPGLFHNNHN		LRR8	4
247	LQRLYLSNN HISQLPPSIFMQLPQ		LRR9	4
271	LNRLTLFGN SLKELSLGIFGPMPN		LRR10	4
295	LRELWLYDN HISSLPDNVFSNLRQ		LRR11	4
319	LQVLILSRN QISFISPGAFNGLTE		LRR12	4
343	LRELSLHTN ALQDLDGNVFRMLAN		LRR13	4
367	LQNISLQNN RLRQLPGNIFANVNG		LRR14	3.4
391	LMAIQLQNN QLENLPLGIFDHLGK		LRR15	3.4
415	LCELRLYDN PWRCDSDILPLRNWLL		LRR16	4
440	LNQPRLGTD TVPVCFSPANVRGQSLIIINV		LRR17	2

NVAVPSVHVPEVPSYPETPWYPDTPSYPDTTSVSSTTELTSPVEDYTDLTTIQVTDDRSV
WGMTQAQSGLAIAAIVIGIVALACSLAACVGCCCCKKRSQAVLMQMKAPNEC

>ENSP00000366062

MSSAQCPALVCVMSRLRFWGPWP

24	LLMWQLLWL LVKEAQPLEWVKDPLQ		LRR1	2.4
49	LTSNPLGPP ESWSSHSSHFPRESPHAPTLP		LRR2	2

ADPWDFDHLGPSASSEMPAPPQESTENLVPFLDTWDSAGEQPLEPEQFLASQQDLKDKLS
PQERLPVSPKKLKKDPAQRWSLAEIIGITRQLSTPQSQKQTLQNEYSSTDTPYPGSLPPE
LRVKSDEPPGPSEQVGPSQFHLEPETQNPETLEDIQSSSLQQEAPAQ

246	LPQLLEEEP SSMQQEAPALPPESS		LRR1	2
270	MESLTLPNH EVSVQPPGEDQAYYHLPNITV		LRR2	2.4

KPADVEVTITSEPTNETESSQAQQETPIQFPEEVEPSATQQEAPIEPPVPPMEHELSISE
QQQPVQPSESPREVESSPTQQETPGQPPEHHEVTVSPPGHHQTHHLASPSVSVKPPDVQL
TIAAEPSAEVGTSLVHQEATTRLSGSGNDVEPPAIQHGGPPLLPESSEEAGPLAVQQETS
FQSPEPINNENPSPTQQEAAAEHPQTAEEGESSLTHQEAPAQTPEFPNVVVAQPPEHSHL
TQATVQPLDLGFTITPESKTEVELSPTMKETPTQPPKKVVPQ

582	LRVYQGVTN PTPGQDQAQHPVSPSVTVQ		LRR1	2.4
610	LLDLGLTIT PEPTTEVGHSTPPKRTIVSPK		LRR2	3.4

HPEVTLPHPDQVQTQHSHLTRATVQPLDLGFTITPKSMTEVEPSTALMTTAPPPGHPEVT
LPPSDKGQAQHSHLTQAT

718	VQPLDLELT ITTKPTTEVKPSPTTEETSTQ		LRR1	2.8
748	PPDLGLAII PEPTTETGHSTALEKTTAPRP		LRR2	2

DRVQTLHRSLTEVTGPPTELEPAQDSLVQSESYTQNKALTAPEEHKASTSTNICELCTCG
DEM

831	CELCTCGDEMLSCIDLNPEQRLRQVPVP		NF	4

SHIFT -18!

841	LSCIDLNPE QRLRQVPVPEPNTHNGT		LRR1	2.4
867	FTILNFQGN YISYIDGNVWKAYSW		LRR2	2.8
891	TEKLILREN NLTELHKDSFEGLLS		LRR3	3
915	LQYLDLSCN KIQSIERHTFEPLPF		LRR4	4
939	LKFINLSCN VITELSFGTFQAWHGMQF		LRR5	3.4
966	LHKLILNHN PLTTVEDPYLFKLPA		LRR6	4
990	LKYLDMGTT LVPLTTLKNILMMTVE		LRR7	2.8
1015	LEKLILPSH				LRR8	3
1024	MACCLCQFKNSIEAVCKTVKLHCNSACLTNTTH		CF3	3

SHIFT -42!

1015	LEKLILPSH MACCLCQFKNSIEAV		LRR8	3
1039	CKTVKLHCN SACLTNTTHCPEEASVGNPEG		LRR9	2.4

AF

1071	MKVLQARKN YTSTELIVEPEEPSDSS		LRR1	2.8
1097	GINLSGFGS EQLDTNDESDFISTLSYILPY		LRR2	2.2

SHIFT -24!

1103	FGSEQLDTN DESDFISTLSYILPYFSAVN		LRR1	2.4
1132	LDVKSLLLP FIKLPTTGNSLAKIQTVGQNR		LRR2	2

QRVKRVLMGPRSIQKRHFKEVGRQSIRREQGAQASVENAAEEKRLTSPAPREVEQPHTQQ
GPEKLAGNAVYTKPSFTQEHKAAVSVLKPFSKGAPSTSSPAKALPQVRDRSKDLTHAISI
LESAKARVTNTKTSKPIVHARKKYRFHKTRSHVTHRTTKVKKSPKVRKKSY

1333	LSRLMLANR LPFSAAKSLINSPSQGAFSS		LRR1	3
1362	LGDLSPQEN PFLEVSALSEHFIEKNNTKHT		LRR2	3

SHIFT -14!

1378	LSEHFIEKN NTKHTTARNAFEENDFMENTN		LRR1	2.4
1408	MPEGTISEN TNYNHPPEADSA			LRR2	2.2
1429	GTAFNLGPT VKQTETKWEYNNVGTD		LRR3	2.2
1454	LSPEPKSFN YPLLSSPGDQFEIQLTQQ		LRR4	2
1481	LQSLIPNNN VRRLIAHVIRTLKMDCSGAHV		LRR5	3

SHIFT -13!

1498	IRTLKMDCS GAHVQVTCAKLISRTGH		LRR1	2.2
1524	LMKLLSGQQ EVKASKIEWDTDQWKIENYIN		LRR2	2

ESTEAQSEQK

1564	EKSLELKKE VPGYGYTDKLILALIVTGI		LRR1	2
1592	LTILIILFC LIVICCHRRSLQEDEEGFSRG		LRR2	2.8

SHIFT -27!

1595	LIILFCLIV ICCHRRSLQEDEEGFSRGI		LRR1	2
1623	FRFLPWRGC SSRRESQDGLSSFGQP		LRR2	2.2
1648	LWFKDLYKP LSATRINNHAWKLHKKSSNED		LRR3	2

KILNRDPGDSEAPTEEEESEALP

>ENSP00000277120

MSSWIRWHGPAMARLWGFCWLVVGFWRAAFACPTSCKCSASRIWCSDPSPGIVAFPRLEP
NSVDPENITEIFIANQKRLEIINEDDVEAYVG

93	LRNLTIVDS GLKFVAHKAFLKNSN		LRR1	2.8
117	LQHINFTRN KLTSLSRKHFRHLD		LRR2	2.8
140	LSELILVGN				LRR3	4
149	PFTCSCDIMWIKTLQEAKSSPDTQDLYCLNESSKNIPLANLQIPNCGLP		CF1	4

SHIFT -24!

174	LYCLNESSK NIPLANLQIPNCG			LRR1	2
196	LPSANLAAP NLTVEEGKSITLSCSVAGDPV		LRR2	2.4

PNMYWDVGNLVSKHMNETSHTQGSLRITNISSDDSGKQISCVAENLVGEDQDSVNLTVHF
APTITFLESPTSDHHWCIPFTVKGNPKPALQWFYNGAILNESKYICTKIHVTNHTEYHGC
LQLDNPTHMNNGDYTLIAKNEYGKDEKQISAHFMGWPGIDDGANPNYPDVIYEDYGTAAN
DIGDTTNRSNEIPSTDVTDKTGREHLSVYAVVVIASVVGFCLLVM

451	LFLLKLARH SKFGMKDFSWFGFGKVKSRQG		LRR1	3
481	VGPASVISN DDDSASPLHHISNGSNTPSSS		LRR2	2.2

SHIFT -17!

494	ASPLHHISN GSNTPSSSEGGPDAVIIG		LRR1	2.4
521	MTKIPVIEN PQYFGITNSQLKPDTFVQHIK		LRR2	2.2

RHNIVLKRELGEGAFGKVFLAECYNLCPEQDKILVAVKTLKDASDNARKDFHREAELLTN
LQHEHIVKFYGVCVEGDPLIMVFEYMKHGDLNKFLRAHGPDAVLMAEGNPPTELTQSQML
HIAQQIAAGMVYLASQHFVHRDLATRNCLVGENLLVKIGDFGMSRDVYSTDYYRVGGHTM
LPIRWMPPESIMYRKFTTESDVWSLGVVLWEIFTYGK

768	QPWYQLSNN EVIECITQGRVLQRPRTCPQE		LRR1	2
798	VYELMLGCW QREPHMRKNIKGIHTL		LRR2	2.4
823	LQNLAKASP VYLDILG			LRR3	2


>AY182028

MGFNVIRLLSGSAVALVIAPTVLLTMLSSAERG

34	CPKGCRCEGKMVYCESQKLQEIPSS		NF	4
59	ISAGCLGLS LRYNSLQKLKYNQFKGLNQ		LRR1	2.2
87	LTWLYLDHN HISNIDENAFNGIRR		LRR2	4
111	LKELILSSN RISYFLNNTFRPVTN		LRR3	4
135	LRNLDLSYN QLHSLGSEQFRGLRK		LRR4	4
159	LLSLHLRSN SLRTIPVRIFQDCRN		LRR5	4
183	LELLDLGYN RIRSLARNVFAGMIR		LRR6	4
207	LKELHLEHN QFSKLNLALFPRLVS		LRR7	4
231	LQNLYLQWN KISVIGQTMSWTWSS		LRR8	4
255	LQRLDLSGN EIEAFSGPSVFQCVPN		LRR9	4
280	LQRLNLDSN KLTFIGQEILDSWIS		LRR10	4
304	LNDISLAGN IWECSRNICSLVNWL		LRR11	3.4
328	KSFKGLREN TIICASPKELQG			LRR12	2
349	VNVIDAVKN YSICGKSTTERFDLARALPKP		LRR13	2.2

TFKPKLPRPKHESKPPLPPTVGATEPGPETDADAEHISFHKII

422	AGSVALFLS VLVILLVIYVSWKRYPASMKQ		LRR1	2.2
452	LQQRSLMRR HRKKKRQSLKQMTPSTQEFYV		LRR2	2

DYKPTNTETSEMLLNGTGPCTYNKSGSRECEV

>AAH11057

MQVSKRMLAGGVRSMPSPLLACWQPILLLVLGSVLSGSATG

42	CPPRCECSAQDRAVLCHRKRFVAVPEGIPTE		NF	4
73	TRLLDLGKN RIKTLNQDEFASFPH		LRR1	3
97	LEELELNEN IVSAVEPGAFNNLFN		LRR2	4
121	LRTLGLRSN RLKLIPLGVFTGLSN		LRR3	4
145	LTKLDISEN KIVILLDYMFQDLYN		LRR4	3.4
169	LKSLEVGDN DLVYISHRAFSGLNS		LRR5	3.4
193	LEQLTLEKC NLTSIPTEALSHLHG		LRR6	3.4
217	LIVLRLRHL NINAIRDYSFKR			LRR7	3
238	LYRLKVLEI SHWPYLDTMTPNC			LRR8	2.4
260	LYGLNLTSL SITHCNLTAVPYLAVRHLVY		LRR9	3
289	LRFLNLSYN PISTIEGSMLHELLR		LRR10	4
313	LQEIQLVGG QLAVVEPYAFRGLNY		LRR11	2.4
337	LRVLNVSGN QLTTLEESVFHSVGN		LRR12	3.4
361	LETLILDSN				LRR13	4
370	PLACDCRLLWVFRRRWRLNFNRQQPTCATPEFVQGKEFKDFPDVLLPNY		CF1_short	3

SHIFT -58!

361	LETLILDSN PLACDCRLLWVF			LRR13	4
382	RRRWRLNFN RQQPTCATPEFVQGKEFK		LRR14	2.4
409	DFPDVLLPN YFTCRRARIRDRKAQQVFVDE		LRR15	2

GHTVQFVCRADGDPP

454	PAILWLSPR KHLVSAKSNGRLTVFPDGTLE		LRR1	2
484	VRYAQVQDN GTYLCIAANAGGNDSMPAHLH		LRR2	2.2

VRSYSPDWPHQPNKTFAFISNQPGEGEANSTRATVPFPFD

554	IKTLIIATT MGFISFLGVVLFC			LRR1	2.2
576	LVLLFLWSR GKGNTKHNIEIEYVPRKSDAG		LRR2	3

ISSADAPRKFNMKMI

>ENSP00000266674

MDTSR

6	LGVLLSLPV LLQLATGGSSPRS			LRR1	2
28	GVLLRGCPT HCHCEPDGRMLLRVDCSD		LRR2	2.2
34	CPTHCHCEPDGRMLLRVDCSDLGLSELPSNLSVF		NF	4

SHIFT -13!

55	LGLSELPSN LSVFTSYLDLSM			LRR3	3
76	NNISQLLPN PLPSLRFLEELR			LRR4	2
97	LAGNALTYI PKGAFTGLYSLKVLM		LRR5	2
121	LQNNQLRHV PTEALQNLRSLQSLRLDANHI		LRR6	2

SHIFT -17!

134	LQNLRSLQS LRLDANHISYVPPSCFSGLHS		LRR1	2.4
164	LRHLWLDDN ALTEIPVQAFRSLSA		LRR2	4
188	LQAMTLALN KIHHIPDYAFGNLSS		LRR3	3.4
212	LVVLHLHNN RIHSLGKKCFDGLHS		LRR4	4
236	LETLDLNYN NLDEFPTAIRTLSN		LRR5	4
259	LKELGFHSN NIRSIPEKAFVGNPS		LRR6	3.4
283	LITIHFYDN PIQFVGRSAFQHLPE		LRR7	2.8
307	LRTLTLNGA SQITEFPDLTGTAN		LRR8	3
330	LESLTLTGA QISSLPQTVCNQLPN		LRR9	3
354	LQVLDLSYN LLEDLPSFSVCQK			LRR10	4
376	LQKIDLRHN EIYEIKVDTFQQLLS		LRR11	3.4
400	LRSLNLAWN KIAIIHPNAFSTLPS		LRR12	4
424	LIKLDLSSN LLSSFPITGLHG			LRR13	4
445	LTHLKLTGN HALQSLISSENFPELKVIEMP		LRR14	4

YAYQCCAFGVCENAYKISNQWNKGDNSSMDDLHKKDAGMFQAQDERDLEDFLLDFEED

533	LKALHSVQC SPSPGPFKPCEH			LRR1	2.4
554	LLDGWLIRI GVWTIAVLALTCNALVTSTVF		LRR2	2.4

SHIFT -19!

565	WTIAVLALT CNALVTSTVFRSPLYISPIKL		LRR1	2.2
595	LIGVIAAVN MLTGVSSAVLAGVDAFTFGSF		LRR2	2.8
625	ARHGAWWEN GVGCHVIGFLSIFASESS		LRR3	2.2
652	VFLLTLAAL ERGFSVKYSAKFETKAPFSS		LRR4	2.4
681	LKVIILLCA LLALTMAAVPLLGGSKYG		LRR5	2.4
708	ASPLCLPLP FGEPSTMGYMVALIL		LRR6	2.4
732	LNSLCFLMM TIAYTKLYCNLDKGDLENIWD		LRR7	2.4

SHIFT -20!

742	IAYTKLYCN LDKGDLENIWDCSMVK		LRR1	2.4
767	HIALLLFTN CILNCPVAFLSFSS		LRR2	3
790	LINLTFISP EVIKFILLVVVPLPACL		LRR3	2.4
816	NPLLYILFN PHFKEDLVSLRKQTYVWTRSK		LRR4	2.4

HPSLMSINSDDVEKQSCDSTQALVTFTSSSITYDLPPSSVPSPAYPVTESCHLSSVAFVP
CL

>ENSP00000334952

MEPPGRRRGRAQPPLLLPLSLLALLALLGGGGGGGAAALPAGCKHDGRPRGAGRAAGAAE
GKVVCSSLELAQV

74	LPPDTLPNR TVTLILSNNKISELKNGS		LRR1	2
101	FSGLSLLER LDLRNNLISSIDPGAFWGLSS		LRR2	2.4
131	LKRLDLTNN RIGCLNADIFRGLTN		LRR3	4
155	LVRLNLSGN LFSSLSQGTFDYLAS		LRR4	4
179	LRSLEFQTE				LRR5	2.4
188	YLLCDCNILWMHRWVKEKNITVRDTRCVYPKSLQAQPVTGVKQELLTCD		CF1_short	3

PPLELPSFYMTPSHRQVVFEGDSLPFQCMASYIDQDMQVLWYQDGRIVETDESQGIFVEK
NMIHNCSLIASALTISNIQAGSTGNWGCHVQTKRGNNTRTVDIVVLESSAQYCPPERVVN
NKGDFRWPRTLAGITAYLQCTRNTHGSGIYPGNPQDERKAWRRCDRGGFWADDDYSRCQY
ANDVTRVLYMFNQMPLNLTNAVATARQLLAYTVEAANFSDKMDVIFVAEMIEKFGRFTKE
EKSKELGDVMVDIASNIMLADERVLWLAQREAKACSRIVQCLQRIATYRLAGGAHVYSTY
SPNIALEAYVIKSTGFTGMTCTVFQKVAASDRTGLSDYGRRDPEGNLDKQLSFKCNVSNT
FSSLALKNTIVEASIQLPPSLFSPKQKRELRPTDDSLYKLQLIAFRNGKLFPATGNSTNL
ADDGKRRTVVTPVILTKIDGVNVDTHHIPVNVTLRRIAHGADAVAARWDFDLLNGQGGWK
SDG

720	CHILYSDEN ITTIQCYSLSNYAV		LRR1	2
743	LMDLTGSEL YTQAASLLHPVVYTTAIILL		LRR2	2.4
772	LCLLAVIVS YIYHHSLIRISLKSWHM		LRR3	2.8
798	LVNLCFHIF LTCVVFVGGITQTRNASICQ		LRR4	2.4
827	AVGIILHYS TLATVLWVGVTARNIYKQVTK		LRR5	2.2

KAKRCQDPDEPPPPPRPMLRFYLIGGGIPII

888	VCGITAAAN IKNYGSRPNAPYCWMAWEPS		LRR1	2.2
917	LGAFYGPAS FITFVNCMYFLSIFIQLKRHP		LRR2	2.2

ERKYELKEPT

957	EEQQRLAAN ENGEINHQDSMSLS		LRR1	2
980	LISTSALEN EHTFHSQLLGASLTL		LRR2	2.4
1004	LLYVALWMF GALAVSLYYPLDLVFSFVFGA		LRR3	2.4

TSLSFSAFFVVHHCVNREDVRLAWIMTCCPGRSSYSVQVNVQPPNSNGTNGEAPKCPNSS
AESSCTNKSASSFKNSSQGCKLTNLQAAAAQCHANSLPLNSTPQLDNSLTEHSMDNDIKM
HVAPLEVQFRTNVHSSRHHKNRSKGHRASR

1184	LTVLREYAY DVPTSVEGSVQNG			LRR1	2
1206	LPKSRLGNN EGHSRSRRAYLAYRERQYNPP		LRR2	3

QQDSSDACSTLPKSSRNFEKPVSTTSKKDALRKPAVVELENQQK

1280	SYGLNLAIQ NGPIKSNGQEGP			LRR1	2
1301	LLGTDSTGN VRTGLWKHETTV			LRR2	2


>ENSP00000340328

MKGRGMLVLLLHAVVLGLPSAWAVGA

27	CARACPAACACSTVERGCSVRCDRAGLLRVPAELPCE		NF	4
64	AVSIDLDRN GLRFLGERAFGTLPS		LRR1	2.8
88	LRRLSLRHN NLSFITPGAFKGLPR		LRR2	4
112	LAELRLAHN GDLRYLHARTFAALSR		LRR3	4
137	LRRLDLAAC RLFSVPERLLAELPA		LRR4	3.4
161	LRELAAFDN LFRRVPGALRGLAN		LRR5	3.4
184	LTHAHLERG RIEAVASSSLQGLRR		LRR6	2.4
208	LRSLSLQAN RVRAVHAGAFGDCGV		LRR7	4
232	LEHLLLNDN LLAELPADAFRGLRR		LRR8	4
256	LRTLNLGGN ALDRVARAWFADLAE		LRR9	4
280	LELLYLDRN SIAFVEEGAFQNLSG		LRR10	4
304	LLALHLNGN RLTVLAWVAFQPGFF		LRR11	4
328	LGRLFLFRN				LRR12	4
337	PWCCDCRLEWLRDWMEGSGRVTDVPCASPGSV		CF3	3

SHIFT -25!

344	LEWLRDWME GSGRVTDVPCASPGS		LRR1	2
368	VAGLDLSQV TFGRSSDGLCVD			LRR2	2.4
389	PEELNLTTS SPGPSPEPAATTVSRFSSL		LRR3	2.4
417	LSKLLAPRV PVEEAANTTGGLANASLSDSL		LRR4	2.4

SHIFT -10!

437	LANASLSDS LSSRGVGGAGRQPWF		LRR1	2.8
461	LLASCLLPS VAQHVVFGLQMD			LRR2	2.4


>ENSP00000312535

M

2	APPLLLLLL ASGAAACPLPCVCQNLSES		LRR1	2.4
30	LSTLCAHRG LLFVPPNVDRRTVELRLADNF		LRR2	2.4

SHIFT -10!

50	TVELRLADN FIQALGPPDFRNMTG		LRR1	3
74	LVDLTLSRN AITRIGARAFGDLES		LRR2	4
98	LRSLHLDGN RLVELGTGSLRGPVN		LRR3	4
122	LQHLILSGN QLGRIAPGAFDDFLES		LRR4	4
147	LEDLDLSYN NLRQVPWAGIGAMPA		LRR5	4
171	LHTLNLDHN LIDALPPGAFAQLGQ		LRR6	4
195	LSRLDLTSN RLATLAPDPLFSRGRDAEAS		LRR7	4
224	PAPLVLSFS GNPLHCNCELLW			LRR8	2.4
245	LRRLARPDD LETCASPPGLAGRYFWAVPEG		LRR9	2

EFSCEPPLIARHTQR

290	LWVLEGQRA TLRCRALGDPAPTMHWV		LRR1	2.4
316	GPDDRLVGN SSRARAFPNGTLEIGVTGAGD		LRR2	2.4

AGGYTCIATNPAGEATARVELRVLALPHGGNSSAEGGRPGPSDIAASARTAAEGEGTLES
EPAVQVTEVTATSGLVSWGPGRPADP

432	VWMFQIQYN SSEDETLIYRIVPASSHHFL		LRR1	2.2
461	LKHLVPGAD YDLCLLALSPAAGPSD		LRR2	2
486	LTATRLLGC AHFSTLPASPLCHA		LRR3	2.4
509	LQAHVLGGT LTVAVGGVLVAALLVFTVAL		LRR4	2.4
538	LVRGRGAGN GRLPLKLSHVQSQTNGGPSPT		LRR5	2.8

SHIFT -15!

553	LSHVQSQTN GGPSPTPKAHPPRSPPPRPQR		LRR1	2.4
583	SCSLDLGDA GCYGYARRLGGAWARRSHSVH		LRR2	2

GGLLGAGCRGVGGSAERLEESVV

>ENSP00000262551

MKTLQSTLLLLLLVPLIKPAPPTQQDSRIIYDYGTDNFEESIFSQDYEDKYLDGKNIKEK
ETVIIPN

68	EKSLQLQKD EAITPLPPKKENDEMPT		LRR1	2
94	CLLCVCLSGSVYCEEVDIDAVPPLPKE		NF	4

SHIFT -26!

95	LLCVCLSGS VYCEEVDIDAVPPLPKE		LRR2	2.8
121	SAYLYARFN KIKKLTAKDFADIPN		LRR3	2.4
145	LRRLDFTGN LIEDIEDGTFSKLSL		LRR4	3.4
169	LEELSLAEN QLLKLPVLPPKLTLFNAKYNK		LRR5	4

SHIFT -17!

182	LPVLPPKLT LFNAKYNKIKSRGIKANAFKK		LRR1	2.4
212	LNNLTFLYL DHNALESVPLNLPES		LRR2	2.4
236	LRVIHLQFN NIASITDDTFCKANDTSYIRD		LRR3	3.4

R

267	IEEIRLEGN PIVLGKHPNSFIC			LRR1	2.8
289	LKRLPIGSY F			LRR2	2.4


>ENSP00000249363

MKLLWQVTVHHHTWNAILLPFVYLTAQVWILCAAIAAAASAGPQN

46	CPSVCSCSNQFSKVVCTRRGLSEVPQGIPSN		NF	4
77	TRYLNLMEN NIQMIQADTFRHLHH		LRR1	3
101	LEVLQLGRN SIRQIEVGAFNGLAS		LRR2	4
125	LNTLELFDN WLTVIPSGAFEYLSK		LRR3	4
149	LRELWLRNN PIESIPSYAFNRVPS		LRR4	4
173	LMRLDLGEL KKLEYISEGAFEGLFN		LRR5	3
198	LKYLNLGMC NIKDMPNLTPLVG			LRR6	3.4
220	LEELEMSGN HFPEIRPGSFHGLSS		LRR7	3.4
244	LKKLWVMNS QVSLIERNAFDGLAS		LRR8	2.8
268	LVELNLAHN NLSSLPHDLFTPLRY		LRR9	4
292	LVELHLHHN				LRR10	4
301	PWNCDCDILWLAWWLREYIPTNSTCCGRCHAPMHMRGRYLVEVDQASFQCS		CF1_short	3

APFIMDAPRDLNISEGRMAELKCRTPPM

380	SSVKWLLPN GTVLSHASRHPRISV		LRR1	2
404	LNDGTLNFS HVLLSDTGVYTCM			LRR2	2.8
426	VTNVAGNSN ASAYLNVSTAELNTSNYSFFT		LRR3	2.2

TVTVETTEISPEDTTRKYKPVPTTSTGYQPAYTTSTTVLIQTTRVPKQVAVPATDTTDKM
QTSLDEVMKTTKIIIGCFVAVTLLAAAMLIVFYKLRKRHQQRSTVTAARTVEIIQVDEDI
PAATSAAATAAPSGVSGEGAVVLPTIHDHINYNTYKPAHGAHWTENSLGNSLHPTVTTIS
EPYIIQTHTKDKVQETQI

>ENSP00000215539

MALRKGGLALALLLLSWVALGPRSLEGADPGTPGEAEGPA

41	CPAACVCSYDDDADELSVFCSSRNLTRLPDGVPGG		NF	4

SHIFT -23!

53	ADELSVFCS SRNLTRLPDGVPGG		LRR1	2.2
76	TQALWLDGN NLSSVPPAAFQNLSS		LRR2	3
100	LGFLNLQGG QLGSLEPQALLGLEN		LRR3	3
124	LCHLHLERN QLRSLALGTFAHTPA		LRR4	4
148	LASLGLSNN RLSRLEDGLFEGLGS		LRR5	4
172	LWDLNLGWN SLAVLPDAAFRGLGS		LRR6	4
196	LRELVLAGN RLAYLQPALFSGLAE		LRR7	4
220	LRELDLSRN ALRAIKANVFVQLPR		LRR8	4
244	LQKLYLDRN LIAAVAPGAFLGLKA		LRR9	4
268	LRWLDLSHN RVAGLLEDTFPGLLG		LRR10	4
292	LRVLRLSHN AIASLRPRTFKDLHF		LRR11	4
316	LEELQLGHN RIRQLAERSFEGLGQ		LRR12	4
340	LEVLTLDHN QLQEVKAGAFLGLTN		LRR13	4
364	VAVMNLSGN CLRNLPEQVFRGLGK		LRR14	2.8
388	LHSLHLEGS CLGRIRPHTFTGLSG		LRR15	3.4
412	LRRLFLKDN GLVGIEEQSLWGLAE		LRR16	4
436	LLELDLTSN QLTHLPHRLFQGLGK		LRR17	4
460	LEYLLLSRN RLAELPADALGPLQR		LRR18	4
484	AFWLDVSHN RLEALPNSLLAPLGR		LRR19	2.8
508	LRYLSLRNN SLRTFTPQPPGLERLWLEGNP		LRR20	4

WDCGCPLKALRDFALQNPSAVPRFVQAICEGDDCQPPAYTYNNITCASPPEVVGLDLRDL
SEAHFAPC

>ENSP00000298171

MRPADLLQLVLLLDLPRDLGGMG

24	CSSPPCECHQEEDFRVTCKDIQRIPSLPPSTQT		NF	4

LK

59	LIETHLRTI PSHAFSNLPNISRIYVSIDVT		LRR1	2
89	LQQLESHSF YNLSKVTHIEIRNTRNLTYID		LRR2	2

PDALKE

125	LPLLKFLGI FNTGLKMFPDLTKVYSTDI		LRR1	2.4
153	FFILEITDN PYMTSIPVNAFQGLCNE		LRR2	2.8
179	TLTLKLYNN GFTSVQGYAFNGTK		LRR3	3
202	LDAVYLNKN KYLTVIDKDAFGGVYSGPSLL		LRR4	3.4

DVSQTSVTA

241	LPSKGLEHL KELIARNTWTLKKLPLS		LRR1	2
267	LSFLHLTRA DLSYPSHCCAFKNQKKIR		LRR2	3
294	GILESLMCN ESSMQSLRQRKSVNALNSPLH		LRR3	2.4

QEYEENLGDSIVGYKEKSKFQDTHNNAHYYVFFEEQEDEIIGFGQELKNPQEETLQAFDS
HYDYTICGDSEDMVCTPKSDEFNPCEDIMGYKF

417	LRIVVWFVS LLALLGNVFVLLI			LRR1	2.2
439	LLTSHYKLN VPRFLMCNLAFADFCM		LRR2	2
464	GMYLLLIAS VDLYTHSEYYNHAIDWQTGPG		LRR3	2.8

CNTAGFFTVFASELSVYTLTVITLERWYAITFAMRLDRKIRLRHACAIMVGGWVC

549	CFLLALLPL VGISSYAKVSICLPMDT		LRR1	2
575	ETPLALAYI VFVLTLNIVAFVIVCCCYVKI		LRR2	2

YITVRNPQYNPGDKDTKIAKRMAVLIFTDFICMAPIS

642	FYALSAILN KPLITVSNSKILLVL		LRR1	2.8
666	FYPLNSCAN PFLYAIFTKAFQRDVFILLSK		LRR2	2.4

FGICKRQAQAYRGQRVPPKNSTDIQVQKVTHEMRQGLHNMEDVYELIENSHLTPKKQGQI
SEEYMQTVL

>ENSP00000360597

MRGPSWSRPRPLLLLLLLLSPWPVWAQVSATASPSGSLGAPDCPEVCTCVPGGLASCSAL
SLPAVPP

68	GLSLRLRAL LLDHNRVRALPPGAFAGAGA		LRR1	2.4
97	LQRLDLREN GLHSVHVRAFWGLGA		LRR2	4
121	LQLLDLSAN QLEALAPGTFAPLRA		LRR3	4
145	LRNLSLAGN RLARLEPAALGALPL		LRR4	4
169	LRSLSLQDN ELAALAPGLLGRLPA		LRR5	4
193	LDALHLRGN				LRR6	4
202	PWGCGCALRPLCAWLRRHPLPASEAETVLCVWPGRLTLSPLTAFSDAAFSHC		CF1_short	3

SHIFT -61!

193	LDALHLRGN PWGCGCALRPLCAW		LRR6	4
216	LRRHPLPAS EAETVLCVWPGRLT		LRR7	2.4
239	LSPLTAFSD AAFSHCAQPLALRDLAVVYT		LRR8	2.4
268	LGPASFLVS LASCLALGSGLTACRARRRR		LRR9	2.2
297	LRTAALRPP RPPDPNPDPDPHGCASPADPG		LRR10	2.4

SPAAAAQA

>ENSP00000327635

MKLWIHLFYSSLLACISLHSQTPVLSSRGS

31	CDSLCNCEEKDGTMLINCEAKGIKMVSEISVPP		NF	4

SR

66	PFQLSLLNN GLTMLHTNDFSGLTN		LRR1	3
90	AISIHLGFN NIADIEIGAFNGLGL		LRR2	2.8
114	LKQLHINHN SLEILKEDTFHGLEN		LRR3	3.4
138	LEFLQADNN FITVIEPSAFSKLNR		LRR4	3.4
162	LKVLILNDN AIESLPPNIFRFVP		LRR5	4
185	LTHLDLRGN QLQTLPYVGFLEHIGR		LRR6	4
210	ILDLQLEDN				LRR7	3.4
219	KWACNCDLLQLKTWLENMPPQSIIGDVVCNSPPFFKGSILSRLKKESICPT		CF1_short	3

SHIFT -12!

258	LSRLKKESI CPTPPVYEEHEDP			LRR1	2
280	SGSLHLAAT SSINDSRMSTKT			LRR2	2.4
301	TSILKLPTK APGLIPYITKPSTQLPGPYCP		LRR3	2

IPCNCKV

338	LSPSGLLIH CQERNIESLSDLRPPPQN		LRR1	2
365	PRKLILAGN IIHSLMKSDLVEYFT		LRR2	3
389	LEMLHLGNN RIEVLEEGSFMNLTR		LRR3	4
413	LQKLYLNGN HLTKLSKGMFLGLHN		LRR4	4
437	LEYLYLEYN AIKEILPGTFNPMPK		LRR5	4
461	LKVLYLNNN LLQVLPPHIFSGVP		LRR6	4
484	LTKVNLKTN QFTHLPVSNILDDLDL		LRR7	3.4
509	LTQIDLEDN				LRR8	3.4
518	PWDCSCDLVGLQQWIQKLSKNTVTDDILCTSPGHLDKKELKALNSEILCPG		CF1_short	3

SHIFT -60!

509	LTQIDLEDN PWDCSCDLVGLQ			LRR8	3.4
530	QWIQKLSKN TVTDDILCTSPGH			LRR9	2
552	LDKKELKAL NSEILCPGLVNNPSMPTQTSY		LRR10	2

LMVTTPATTTNTADTI

598	LRSLTDAVP LSVLILGLLIMFITIVFCAAG		LRR1	2
628	IVVLVLHRR RRYKKKQVDEQMRDNSPVHLQ		LRR2	2.4

YSMYGHKTTHHTTERPSASLYEQHMVSPMVHVYRSPSFGPKHLEEEEERNEKEGSDAKHL
QRSLLEQENHSPLTGSNMKYKTTNQSTEFLSFQDASSLYRNILEKERE

766	LQQLGITEY LRKNIAQLQPDMEAHYPGA		LRR1	2.4
794	HEELKLMET LMYSRPRKVLVEQTK		LRR2	2.4
818	NEYFELKAN LHAEPDYLEVLEQQT		LRR3	2.4


>ENSP00000326763

MNLVDLWLTRS

12	LSMCLLLQS FVLMILCFHSASM		LRR1	2.4
34	CPKGCLCSSSGGLNVTCSNANLKEIPRDLPPE		NF	4

SHIFT -27!

39	LCSSSGGLN VTCSNANLKEIPRDLPPE		LRR2	2.4
66	TVLLYLDSN QITSIPNEIFKDLHQ		LRR3	3
90	LRVLNLSKN GIEFIDEHAFKGVAET		LRR4	4
115	LQTLDLSDN RIQSVHKNAFNN			LRR5	4
136	LKARARIAN NPWHCDCTLQQVLRSMASNHE		LRR6	2

TAHNVICKTSVLDEHAGRPFLNAANDADLCNLPKKTTDYAM

207	LVTMFGWFT MVISYVVYYVRQNQEDARRH		LRR1	2.2
236	LEYLKSLPS RQKKADEPDDISTVV		LRR2	2.4


>AY182030

MGFHLITQLKGMSVVLVLLPTLLLVMLTGAQRA

34	CPKNCRCDGKIVYCESHAFADIPENISGG		NF	4
63	SQGLSLRFN SIQKLKSNQFAGLNQ		LRR1	3
87	LIWLYLDHN YISSVDEDAFQGIRR		LRR2	4
111	LKELILSSN KITYLHNKTFHPVPN		LRR3	4
135	LRNLDLSYN KLQTLQSEQFKGLRK		LRR4	4
159	LIILHLRSN SLKTVPIRVFQDCRN		LRR5	4
183	LDFLDLGYN RLRSLSRNAFAGLLK		LRR6	4
207	LKELHLEHN QFSKINFAHFPRLFN		LRR7	4
231	LRSIYLQWN RIRSISQGLTWTWSS		LRR8	3.4
255	LHNLDLSGN DIQGIEPGTFKCLPN		LRR9	4
279	LQKLNLDSN KLTNISQETVNAWIS		LRR10	4
303	LISITLSGN MWECSRSICPLFYWLKNFKGN		LRR11	3.4

KESTMICAGPKHIQGEKVSDAVETYNICSEVQVVNTERSHLVPQTPQKPLIIPRPTIFKP
DVTQSTFETPSPSPGFQIPGAEQEYEHVSFHKII

427	AGSVALFLS VAMILLVIYVSWKRYPASMKQ		LRR1	2.2
457	LQQHSLMKR RRKKARESERQMNSPLQEYYV		LRR2	2

DYKPTNSETMDISVNGSGPCTYTISGSRECEV

>ENSP00000374508

M

2	PGPLGLLCF LALGLLGSAGPSGA		LRR1	2
25	APPLCAAPC SCDGDRRVDCSGKGLTAVPEG		LRR2	2.2

LSAF

59	TQALDISMN NITQLPEDAFKNFPF		LRR1	2.4
83	LEELQLAGN DLSFIHPKALSGLKE		LRR2	4
107	LKVLTLQNN QLKTVPSEAIRGLSA		LRR3	4
131	LQSLRLDAN HITSVPEDSFEGLVQ		LRR4	4
155	LRHLWLDDN SLTEVPVHPLSNLPT		LRR5	4
179	LQALTLALN KISSIPDFAFTNLSS		LRR6	4
203	LVVLHLHNN KIRGLSQHCFDGLDN		LRR7	4
227	LETLDLSYN NLGEFPQAIKARPS		LRR8	4
250	LKELGFHSN SISVIPDGAFDGNPL		LRR9	3.4
274	LRTIHLYDN PLSFVGNSASHNLSD		LRR10	3.4
298	LHSLVIRGA SMVQQFPNLTGTVH		LRR11	2.4
321	LESLTLTGT KISSIPNNLCQEQKM		LRR12	3.4
345	LRTLDLSYN NIRDLPSFNGCHA			LRR13	4
367	LEEISLQRN QIYQIKEGTFQGLIS		LRR14	3.4
391	LRILDLSRN LIHEIHSRAFATLGP		LRR15	4
415	ITNLDVSFN ELTSFPTEGPNG			LRR16	2.8
436	LNQLKLVGN FKLKEALAAKDFVN		LRR17	4
459	LRSLSVPYA YQCCAFWGCDSYANLNTEDNS		LRR18	2.4

LQDHSVAQEKGTADAANVTSTLENEEHSQIIIHCTPSTGAFKPCEYL

536	LGSWMIRLT VWFIFLVALFFNL			LRR1	2.2
558	LVILTTFAS CTSLPSSKLFIGLISVSNLFM		LRR2	2.4

SHIFT -18!

570	LPSSKLFIG LISVSNLFMGIYTGI		LRR1	2
594	LTFLDAVSW GRFAEFGIWWETGSGCKV		LRR2	2.4
621	AGFLAVFSS ESAIFLLMLATVERS		LRR3	2.2
645	LSAKDIMKN GKSNHLKQFRVA			LRR4	2.4
666	ALSAFLGAT VAGCFPLFHRGEYS		LRR5	2.2
689	ASPLCLPFP TGETPSLGFTVTLVL		LRR6	2.4
713	LNSLAFLLM AVIYTKLYCNLEKEDLSENSQ		LRR7	2.4

SHIFT -20!

723	VIYTKLYCN LEKEDLSENSQSSMIKH		LRR1	2.4
749	VAWLIFTNC IFFCPVAFFSFAPLITAISIS		LRR2	2.2

PEIMKSVTLIFFPLPACLNPVLYVFFNPKFKEDWKLLKRRVTKKSGSVSVSISSQGGCLE
QDFYYDCGMYSHLQGNLTVCDCCESFLLTKPVSCKHLIKSHSCPALAVASCQRPEGYWSD
CGTQSAHSDYADEEDSFVSDSSDQVQACGRACFYQSRGFPLVRYAYNLPRVKD

>ENSP00000296795

MRQTLPCIYFWGGL

15	LPFGMLCAS STTKCTVSHEVAD			LRR1	2.8
37	CSHLKLTQV PDDLPTNITVLNLTHNQ		LRR2	2
63	LRRLPAANF TRYSQLTSLDVGFNTISKLEP		LRR3	2.4

SHIFT -16!

77	LTSLDVGFN TISKLEPELCQKLPM		LRR1	3.4
101	LKVLNLQHN ELSQLSDKTFAFCTN		LRR2	4
125	LTELHLMSN SIQKIKNNPFVKQKN		LRR3	4
149	LITLDLSHN GLSSTKLGTQVQLEN		LRR4	4
173	LQELLLSNN KIQALKSEELDIFANSS		LRR5	4
199	LKKLELSSN QIKEFSPGCFHAIGR		LRR6	4
223	LFGLFLNNV QLGPSLTEKLCLELANTS		LRR7	3
250	IRNLSLSNS QLSTTSNTTFLGLKWTN		LRR8	2.8
276	LTMLDLSYN NLNVVGNDSFAWLPQ		LRR9	4
300	LEYFFLEYN NIQHLFSHSLHGLFN		LRR10	3.4
324	VRYLNLKRS FTKQSISLASLPKIDDFSFQW		LRR11	2.8
354	LKCLEHLNM EDNDIPGIKSNMFTGLIN		LRR12	2
381	LKYLSLSNS FTSLRTLTNETFVSLAHSP		LRR13	3.4
409	LHILNLTKN KISKIESDAFSWLGH		LRR14	4
433	LEVLDLGLN EIGQELTGQEWRGLEN		LRR15	4
458	IFEIYLSYN KYLQLTRNSFALVPS		LRR16	2.8
482	LQRLMLRRV ALKNVDSSPSPFQPLRN		LRR17	3
508	LTILDLSNN NIANINDDMLEGLEK		LRR18	4
532	LEILDLQHN NLARLWKHANPGGPIYFLKG		LRR19	4
561	LSHLHILNL ESNGFDEIPVEVFKDLFE		LRR20	2.4
588	LKIIDLGLN NLNTLPASVFNNQVS		LRR21	3.4
612	LKSLNLQKN LITSVEKKVFGPAFRN		LRR22	4
637	LTELDMRFN				LRR23	3.4
646	PFDCTCESIAWFVNWINETHTNIPELSSHYLCNTPPHYHGFPVRLFDTSSCKDS		CF1	4

SHIFT -29!

671	LSSHYLCNT PPHYHGFPVRLFDTSSCKDSA		LRR1	2.4
701	PFELFFMIN TSILLIFIFIVLLIHFE		LRR2	2.4
727	GWRISFYWN VSVHRVLGFKEIDRQTEQFEY		LRR3	2.2

AAYIIHAYKDKDWVWEHFSSMEKEDQSLKFCLEERDFEAG

797	VFELEAIVN SIKRSRKIIFVITHH		LRR1	2.8
821	LLKDPLCKR FKVHHAVQQAIEQN		LRR2	2
844	LDSIILVFL EEIPDYKLNHALCLRRGMFKS		LRR3	2.4

HCILNWPVQKERIGAFRHKLQVALGSKNSVH

>ENSP00000325713

MTSAQCPALACVMSPLRFWGPWP

24	LLMWQLLWL LVKEAQPLEWVKDPLQ		LRR1	2.4
49	LTSNPLGPP EPWSSHSSHFPRESPHAPTLP		LRR2	2

ADPWDFDHLGPSASSEMPAPPQESTENLVPFLDTWDSAGELPLEPEQFLASQQDLKDKLS
PQERLPVSPKKLKKDPAQRWSLAEIIGIIHQLSTPQSQKQTLQNEYSSTDTPYPGSLPPE
LRVKSDEPPGPSEQVGPSQFHLEPETQNPETLEDIQSSSLQQEAPAQ

246	LPQLLEEEP SSMQQEAPALPPESS		LRR1	2
270	MESLTLPNH EVSVQPPGEDQAYYHLPNITV		LRR2	2.4

KPADVEVTITSEPTNETESSQAQQETPIQFPEEVEPSATQQEAPIEPPVPPMEHELSISE
QQQPVQPSESSREVESSPTQQETPGQPPEHHEVTVSPPGHHQTHHLASPSVSVKPPDVQL
TIAAEPSAEVGTSLVHQEATTRLSGSGNDVEPPAIQHGGPPLLPESSEEAGPLAVQQETS
FQSPEPINNENPSPTQQEAAAEHPQTAEEGESSLTHQEAPAQTPEFPNVVVAQPPEHSHL
TQATVQPLDLGFTITPES

558	MTEVELSPT MKETPTQPPKKVVPQ		LRR1	2.2
582	LRVYQGVTN PTPGQDQAQHPVSPSVTVQ		LRR2	2.4
610	LLDLGLTIT PEPTTEVGHSTPPKRTIVSPK		LRR3	3.4

HPEVTLPHPDQVQTQHSHLTRATVQPLDLGFTITPKSMTEVEPSTALMTTAPPPGHPEVT
LPPSDKGQAQHSHLTQAT

718	VQPLDLELT ITTKPTTEVKPSPTTEETSTQ		LRR1	2.8
748	LPDLGLAII PEPTTETGHSTALEKTTAPRP		LRR2	3

DRVQTLHRSLTEVTGPPTELEPAQDSLVQSESYTQNKALTAPEEHKASTSTNICELCTCG
DEM

831	CELCTCGDEMLSCIDLNPEQRLRQVPVP		NF	4

SHIFT -18!

841	LSCIDLNPE QRLRQVPVPEPNTHNGT		LRR1	2.4
867	FTILNFQGN YISYIDGNVWKAYSW		LRR2	2.8
891	TEKLILREN NLTELHKDSFEGLLS		LRR3	3
915	LQYLDLSCN KIQSIERHTFEPLPF		LRR4	4
939	LKFINLSCN VITELSFGTFQAWHGMQF		LRR5	3.4
966	LHKLILNHN PLTTVEDPYLFKLPA		LRR6	4
990	LKYLDMGTT LVPLTTLKNILMMTVE		LRR7	2.8
1015	LEKLIVPSH				LRR8	2.4
1024	MACCLCQFKNSIEAVCKTVKLHCNSACLTNTTH		CF3	3

SHIFT -42!

1015	LEKLIVPSH MACCLCQFKNSIEAV		LRR8	2.4
1039	CKTVKLHCN SACLTNTTHCPEEASVGNPEG		LRR9	2.4

AF

1071	MKVLQARKN YTSTELIIEPEEPSDSS		LRR1	2.8
1097	GINLSGFGS EQLDTNDESDVTSTLSYILPY		LRR2	2.2

SHIFT -24!

1103	FGSEQLDTN DESDVTSTLSYILPYFSAVN		LRR1	2.4
1132	LDVKSLLLP FIKLPTTGNSLAKIQTVGKNR		LRR2	2

QRLNRVLMGPRSIQKRHFKEVGRQSIRREQGAQASVENTAEEKRLGSPAPRELKQPHTQQ
GPEKLAGNAVYTKPSFTQEHKAAVSVLKPFSKGAPSTSSPAKALPQVRDRWKDLTHAISI
LESAKARVTNMKTSKPIVHSRKKYRFHKTRSRMTHRTPKVKKSPKVRKKSY

1333	LSRLMLSNR LPFSAAKSLINSPSQGAFSS		LRR1	3
1362	LRDLSPQEN PFLEVSAPSEHFIENNNTKDT		LRR2	3

TARNAFEENVFMENTN

1408	MPEGTISEN TNYNHPPEADSA			LRR1	2.2
1429	GTAFNLGPT VKQTETKWEYNNVGTD		LRR2	2.2
1454	LSPEPKSFN YPLLSSPGDQFEIQLTQQ		LRR3	2
1481	LQSVIPNNN VRRLIAHVIRTLKMDCSGAHV		LRR4	2.4

SHIFT -13!

1498	IRTLKMDCS GAHVQVTCAKLVSRTGH		LRR1	2.2
1524	LMKLLSGQQ EVKASKIEWDTDQWKTENYIN		LRR2	2

ESTEAQSEQKEKSLEFTKELPGYGYTKKLILALIVTGILTILIILLCLIEICCHRRSLQE
DEEGFSRDSEAPTEEESEALP

>ENSP00000216241

MEGPRSSTHVPLVLPLLVLLLLAPARQAAAQR

33	CPQACICDNSRRHVACRYQNLTEVPDAIPEL		NF	4
64	TQRLDLQGN LLKVIPAAAFQGVPH		LRR1	3
88	LTHLDLRHC EVELVAEGAFRGLGR		LRR2	3.4
112	LLLLNLASN HLRELPQEALDGLGS		LRR3	4
136	LRRLELEGN ALEELRPGTFGALGA		LRR4	4
160	LATLNLAHN ALVYLPAMAFQGLLR		LRR5	4
184	VRWLRLSHN ALSVLAPEALAGLPA		LRR6	3.4
208	LRRLSLHHN ELQALPGPVLSQARG		LRR7	4
232	LARLELGHN PLTYAGEEDGLALPG		LRR8	4
256	LRELLLDGG ALQALGPRAFAHCPR		LRR9	3
280	LHTLDLRGN QLDTLPPLQGPGQ			LRR10	4
302	LRRLRLQGN				LRR11	4
311	PLWCGCQARPLLEWLARARVRSDGACQGPRRLRGEALDALRPWDLRCP		CF1_short	3

SHIFT -37!

322	LEWLARARV RSDGACQGPRRLRGEALDA		LRR1	2
350	LRPWDLRCP GDAAQEEEELEERAVAGPRAP		LRR2	2.4

PRGPPRGPGEERAVAP

396	CPRACVCVPESRHSSCEGCGLQAVPRGFPSD		NF	4
427	TQLLDLRRN HFPSVPRAAFPGLGH		LRR1	3
451	LVSLHLQHC GIAELEAGALAGLGR		LRR2	3.4
475	LIYLYLSDN QLAGLSAAALEGAPR		LRR3	4
499	LGYLYLERN RFLQVPGAALRALPS		LRR4	4
523	LFSLHLQDN AVDRLAPGDLGRTRA		LRR5	4
547	LRWVYLSGN RITEVSLGALGPARE		LRR6	3.4
571	LEKLHLDRN QLREVPTGALEGLPA		LRR7	4
595	LLELQLSGN PLRALRDGAFQPVGRS		LRR8	4
620	LQHLFLNSS GLEQICPGAFSGLGPG		LRR9	3.4
645	LQSLHLQKN QLRALPALPSLSQ			LRR10	4
667	LELIDLSSN				LRR11	3.4
676	PFHCDCQLLPLHRWLTGLNLRVGATCATPPNARGQRVKAAAAVFEDCP		CF1_short	3

SHIFT -57!

667	LELIDLSSN PFHCDCQLLPLHRW		LRR11	3.4
690	LTGLNLRVG ATCATPPNARGQRVKAAAAVF		LRR12	3

EDCPGWAARKAKRTPASRPSARRTPIKGRQCGADKVGHGAGGV

>ENSP00000256447

MAFDVSCFFWVVLFSAGCKVITSWDQMCIEKEANKTYN

39	CENLGLSEI PDTLPNTTEFLEFS		LRR1	2
62	FNFLPTIHN RTFSRLMNLTFLDLTRCQINW		LRR2	2.4

SHIFT -16!

76	LMNLTFLDL TRCQINWIHEDTFQSHHQ		LRR1	2.4
103	LSTLVLTGN PLIFMAETSLNGPKS		LRR2	4
127	LKHLFLIQT GISNLEFIPVHNLEN		LRR3	3.4
151	LESLYLGSN HISSIKFPKDFPARN		LRR4	4
175	LKVLDFQNN AIHYISREDMRSLEQ		LRR5	3.4
199	AINLSLNFN GNNVKGIELGAFDSTI		LRR6	3.4
224	FQSLNFGGT PNLSVIFNGLQNST		LRR7	2.2
247	TQSLWLGTF EDIDDEDISSAMLKGLCEMS		LRR8	2
276	VESLNLQEH RFSDISSTTFQCFTQ		LRR9	2.4
300	LQELDLTAT HLKGLPSGMKGLNL		LRR10	3.4
323	LKKLVLSVN HFDQLCQISAANFPS		LRR11	4
347	LTHLYIRGN VKKLHLGVGCLEKLGN		LRR12	3.4
372	LQTLDLSHN DIEASDCCSLQLKNLSH		LRR13	4
398	LQTLNLSHN EPLGLQSQAFKECPQ		LRR14	4
422	LELLDLAFT RLHINAPQSPFQNLHF		LRR15	3.4
447	LQVLNLTYC FLDTSNQHLLAGLPV		LRR16	3.4
471	LRHLNLKGN HFQDGTITKTNLLQTVGS		LRR17	4
498	LEVLILSSC GLLSIDQQAFHSLGK		LRR18	3.4
522	MSHVDLSHN SLTCDSIDSLSHLK		LRR19	2.8
545	GIYLNLAAN SINIISPRLLPILSQ		LRR20	3.4
569	QSTINLSHN				LRR21	2.4
578	PLDCTCSNIHFLTWYKENLHKLEGSEETTCANPPSLRGVKLSDVKLSCGIT		CF1	4

SHIFT -60!

569	QSTINLSHN PLDCTCSNIHFLTWYKEN		LRR21	2.4
596	LHKLEGSEE TTCANPPSLRGVK			LRR22	2.4
618	LSDVKLSCG ITAIGIFFLIVF			LRR23	2.4
639	LLLLAILLF FAVKYLLRWKYQHI		LRR24	2.4


>ENSP00000361183

MASVFHYFLLVLVFLDTHA

20	AQPFCLPGC TCSEESFGRTLQCTS		LRR1	2.2
44	VSLGKIPGN LSEEFKQVRIENSPLFEMPQG		LRR2	2.2

SFINM

79	STLEYLWLN FNNISVIHLGALEHLPE		LRR1	2
105	LRELRLEGN KLCSVPWTAFRATPL		LRR2	4
129	LRVLDLKRN KIDALPELALQFLVS		LRR3	4
153	LTYLDLSSN RLTVVSKSVFLNWPAYQKCRQ		LRR4	4

PDCGAEILSSLVVALHDNPWVCDCRLRGLVQFVKSITLPVILVNSYLICQGPLSKAGQLF
HETELSACMKPQISTPSANITIRAGQNVTLRCLAQASPSPSIAWTYPLSMWREFDVLTSS
TGEDTALSELAIPAAHLVDSGNYTCMASNSIGKSNLVISLHVQPAQALHAPDSLSIPSEG
NAYIDLRVVKQTVHGILLEWLAVADTSKEEWFTLYIASDEAFRKEVVHIGPGINTYAVDD
LLPGTKYEACLSLEGQPPHQGQCVAFVTGRDAGGLEAREHLLHVTVVLCVVLLAVPVGAY
AWAAQGPCSCSKWVLRGCLHRRKAPSCTPAAPQSKDGSFREHPAVCDDGEGHIDTEGDKE
KGGTEDNS

>ENSP00000373853

MARARGSPCPPLPPGRMSWPHGALLFLWLFSPPLGAGGGGVAVTSAAGGGSPPATS

57	CPVACSCSNQASRVICTRRDLAEVPASIPVN		NF	4
88	TRYLNLQEN GIQVIRTDTFKHLRH		LRR1	3
112	LEILQLSKN LVRKIEVGAFNGLPS		LRR2	4
136	LNTLELFDN RLTTVPTQAFEYLSK		LRR3	4
160	LRELWLRNN PIESIPSYAFNRVPS		LRR4	4
184	LRRLDLGEL KRLEYISEAAFEGLVN		LRR5	3
209	LRYLNLGMC NLKDIPNLTALVR			LRR6	3.4
231	LEELELSGN RLDLIRPGSFQGLTS		LRR7	4
255	LRKLWLMHA QVATIERNAFDDLKS		LRR8	3
279	LEELNLSHN NLMSLPHDLFTPLHR		LRR9	4
303	LERVHLNHN				LRR10	3.4
312	PWHCNCDVLWLSWWLKETVPSNTTCCARCHAPAGLKGRYIGELDQSHFTCY		CF1_short	3

APVIVEPPTDLNVTEGMAAELKCRTGTSM

392	TSVNWLTPN GTLMTHGSYRVRISV		LRR1	2
416	LHDGTLNFT NVTVQDTGQYTCMVTNSAGNT		LRR2	2.8

TASATLNVSAVDPVAAGGTGSGGGGPGGSGGVGGGSGGYTYFTTVTVETLETQPGEEALQ
PRGTEKEPPGPTTDGVWGGGRPGDAAGPASSSTTAPAPRSSRPTEKAFTVPITDVTENAL
KDLDDVMKTTKIIIGCFVAITFMAAVMLVAFYKLRKQHQLHKHHGPTRTVEIINVEDELP
AASAVSVAAAAAVASGGGVGGDSHLALPALERDHLNHHHYVAAAFKAHYSSNPSGGGCGG
KGPPGLNSIHEPLLFKSGSKENVQETQI

>ENSP00000319286

MLRGTLLCAVLGLLRAQPFP

21	CPPACKCVFRDAAQCSGGDVARISALGLPT		NF	4

N

52	LTHILLFGM GRGVLQSQSFSGMTV		LRR1	2.4
76	LQRLMISDS HISAVAPGTFSDLIK		LRR2	2.8
100	LKTLRLSRN KITHLPGALLDKMVL		LRR3	4
124	LEQLFLDHN ALRGIDQNMFQKLVN		LRR4	4
148	LQELALNQN QLDFLPASLFTNLEN		LRR5	4
172	LKLLDLSGN NLTHLPKGLLGAQAK		LRR6	4
196	LERLLLHSN RLVSLDSGLLNSLGA		LRR7	4
220	LTELQFHRN HIRSIAPGAFDRLPN		LRR8	3.4
244	LSSLTLSRN HLAFLPSALFLHSHN		LRR9	4
268	LTLLTLFEN PLAELPGVLFGEMGG		LRR10	4
292	LQELWLNRT QLRTLPAAAFRNLSR		LRR11	3.4
316	LRYLGVTLS PRLSALPQGAFQGLGE		LRR12	2.8
341	LQVLALHSN GLTALPDGLLRGLGK		LRR13	4
365	LRQVSLRRN RLRALPRALFRNLSS		LRR14	3.4
389	LESVQLDHN QLETLPGDVFGALPR		LRR15	3.4
413	LTEVLLGHN				LRR16	3.4
422	SWRCDCGLGPFLGWLRQHLGLVGGEEPPRCAGPGAHAGLPLWALPGGDAECP		CF1_short	3

SHIFT -61!

413	LTEVLLGHN SWRCDCGLGPFLGWL		LRR16	3.4
437	RQHLGLVGG EEPPRCAGPGAHAG		LRR17	2
460	LPLWALPGG DAECPGPRGPPPRPAADSSSE		LRR18	2.4

SHIFT -28!

462	LWALPGGDA ECPGPRGPPPRPAADSSSEA		LRR1	2.4
491	PVHPALAPN SSEPWVWAQPVTTGKGQDHSP		LRR2	2

FWGFYFLLLAVQAMITVIIVFAMIKIGQLFRKLIRERALG

>ENSP00000327336

MWP

4	LWRLVSLLA LSQALPFEQRGFWDFT		LRR1	2
29	LDDGPFMMN DEEASGADTSGVLDPDSVTPT		LRR2	2.8

YSAM

63	CPFGCHCHLRVVQCSDLGLKSVPKEISP		NF	4
91	DTTLLDLQN NDISELRKDDFKGLQH		LRR1	2
116	LYALVLVNN KISKIHEKAFSPLRK		LRR2	4
140	LQKLYISKN HLVEIPPNLPSS			LRR3	3.4
161	LVELRIHDN RIRKVPKGVFSGLRN		LRR4	3.4
185	MNCIEMGGN PLENSGFEPGAFDG		LRR5	2.2
208	LKLNYLRIS EAKLTGIPKDLPET		LRR6	2.4
231	LNELHLDHN KIQAIELEDLLRYSK		LRR7	4
255	LYRLGLGHN QIRMIENGSLSFLPT		LRR8	4
279	LRELHLDNN KLARVPSGLPDLKL		LRR9	4
302	LQVVYLHSN NITKVGVNDFCPMGFGVKRAY		LRR10	3.4
332	YNGISLFNN PVPYWEVQPATFRCVTDRLAI		LRR11	2.4

QFGNYKK

>ENSP00000247271

MEYQILKMSLCLFILLF

18	LTPGILCIC PLQCICTERHRHVDCSGRN		LRR1	2.8
24	CICPLQCICTERHRHVDCSGRNLSTLPSGLQEN		NF	4

SHIFT -11!

46	LSTLPSGLQ ENIIHLNLSYNHFTDL		LRR2	2
71	HNQLTQYTN LRTLDISNNRLESLPAHLPRS		LRR3	2
101	LWNMSAANN NIKLLDKSDTAYQWN		LRR4	2.8
125	LKYLDVSKN MLEKVVLIKNTLRS		LRR5	3.4
148	LEVLNLSSN KLWTVPTNMPSK			LRR6	4
169	LHIVDLSNN SLTQILPGTLINLTN		LRR7	3.4
193	LTHLYLHNN KFTFIPDQSFDQLFQ		LRR8	4
217	LQEITLYNN RWSCDHKQNITYLLKWMMETK		LRR9	3.4

AHVIGTPCSTQISSLKEHNMYPTPSGFTSSLFTVSGMQTVDTINSLSVVTQPKVTKIPKQ
YRTKETTFGATLSKDTTFTSTDKAFVPYPEDTSTETINSHEAAAATLTIH

357	LQDGMVTNT SLTSSTKSSPTPMT		LRR1	2.2
380	LSITSGMPN NFSEMPQQSTTLN			LRR2	2.4
402	LWREETTTN VKTPLPSVANAWKV		LRR3	2
425	NASFLLLLN VVVMLAV			LRR4	2.4


>ENSP00000338887

MFTLAEVASLNDIQPTYRILKPWWDVFMDY

31	LAVVMLMVA IFAGTMQLTKDQVVC		LRR1	2.4
55	LPVLPSPVN SKAHTPPGNAEVTTN		LRR2	3
79	IPKMEAATN QDQDGRTTNDISFGTSAVTPD		LRR3	2.2

IPLRATYPRTDFALPNQEAKKEKKDPTGRKTNLDFQQYVFINQMCYHLALPWYSKYFPYL
ALIHTIILMVSSNFWFKYPKTCSKVEHFVSILGKCFESPWTTKALSETACEDSEENKQRI
TGAQTLPKHVSTSSDEGSPSASTPMINKTGFKFSAEKPVIEVPSMTILDKKDGEQAKALF
EKVRKFRAHVEDSDLIYKLYVVQTVIKTAKFIFILCYTANFVNAISFEHVCKPKVEHLIG
YEVFECTHNMAYM

362	LKKLLISYI SIICVYGFICLYTLFW		LRR1	2.4
387	LFRIPLKEY SFEKVREESSFSDIPDVKNDF		LRR2	2.4

AFLLHMVDQYDQLYSKRFGVFLSEVSENK

446	LREISLNHE WTFEKLRQHISRNAQDKQE		LRR1	2.4
474	LHLFMLSGV PDAVFDLTDLDVLKLELIPEA		LRR2	2.4

SHIFT -15!

489	LTDLDVLKL ELIPEAKIPAKISQMTN		LRR1	2.4
515	LQELHLCHC PAKVEQTAFSFLRDH		LRR2	3.4
539	LRCLHVKFT DVAEIPAWVYLLKN		LRR3	2.8
562	LRELYLIGN LNSENNKMIGLESLRELRH		LRR4	4
590	LKILHVKSN LTKVPSNITDVAPH		LRR5	3.4
613	LTKLVIHND GTKLLVLNSLKKMMN		LRR6	2.4
637	VAELELQNC ELERIPHAIFSLSN		LRR7	2.8
660	LQELDLKSN NIRTIEEIISFQHLKR		LRR8	4
685	LTCLKLWHN KIVTIPPSITHVKN		LRR9	4
708	LESLYFSNN KLESLPVAVFSLQK		LRR10	3.4
731	LRCLDVSYN NISMIPIEIGLLQN		LRR11	3.4
754	LQHLHITGN KVDILPKQLFKCIK		LRR12	3.4
777	LRTLNLGQN CITSLPEKVGQLSQ		LRR13	4
800	LTQLELKGN CLDRLPAQLGQCRM		LRR14	4
823	LKKSGLVVE DHLFDTLPLEVKEALNQDINI		LRR15	2

PFANGI

>ENSP00000332879

MGLQTTKWPSHGAFFLKSWLIISLGLYSQ

30	VSKLLACPS VCRCDRNFVYCNERS		LRR1	2.2
54	LTSVPLGIP EGVTVLYLHNNQINNAGFPAE		LRR2	2.4

SHIFT -19!

65	VTVLYLHNN QINNAGFPAELHNVQS		LRR1	3.4
90	VHTVYLYGN QLDEFPMNLPKN			LRR2	2.8
111	VRVLHLQEN NIQTISRAALAQLLK		LRR3	3.4
135	LEELHLDDN SISTVGVEDGAFREAIS		LRR4	4
161	LKLLFLSKN HLSSVPVGLPVD			LRR5	4
182	LQELRVDEN RIAVISDMAFQNLTS		LRR6	3.4
206	LERLIVDGN LLTNKGIAEGTFSHLTK		LRR7	3.4
232	LKEFSIVRN SLSHPPPDLPGTH			LRR8	2.8
254	LIRLYLQDN QINHIPLTAFSNLRK		LRR9	4
278	LERLDISNN QLRMLTQGVFDNLSN		LRR10	3.4
302	LKQLTARNN				LRR11	3.4
311	PWFCDCSIKWVTEWLKYIPSSLNVRGFMCQGPEQVRGMAVRELNMNLLSCP		CF1_short	3

SHIFT -60!

302	LKQLTARNN PWFCDCSIKWVTEW		LRR11	3.4
325	LKYIPSSLN VRGFMCQGPEQVRG		LRR12	2.4
348	MAVRELNMN LLSCPTTTPGLPLFTPAPSTA		LRR13	2.4

SPTTQPPTLSIPNPSRSYTPPTPTTSKLPTIPDWDGRERVTPPISERIQLSIHFVNDTSI
QVSWLSLFTVMAYK

452	LTWVKMGHS LVGGIVQERIVSGEKQH		LRR1	2.2
478	LSLVNLEPR STYRICLVPLDAFNYRAVEDT		LRR2	2.4

SHIFT -15!

493	LVPLDAFNY RAVEDTICSEATTH		LRR1	2.4
516	ASYLNNGSN TASSHEQTTSHSMGSPFL		LRR2	2.4
543	LAGLIGGAV IFVLVVLLSVFCWHMHKKGRY		LRR3	2.4

TSQKWKYNRGRRKDDYCEAGTKKDNSILEMTETSFQIVSLNNDQLLKGDFRLQPIYTPNG
GINYTDCHIPNNMRYCNSSVPDLEHCHT

>ENSP00000306780

MALLLVSL

9	LAFLSLGSG CHHRICHCSNRVFLCQESKVT		LRR1	3
39	EIPSDLPRN AIELRFVLTKLRVIQKGAFSG		LRR2	2

SHIFT -21!

48	AIELRFVLT KLRVIQKGAFSGFGD		LRR1	2.2
72	LEKIEISQN DVLEVIEADVFSN			LRR2	2.8
94	LPKLHEIRI EKANNLLYINPEAFQNLPN		LRR3	2
122	LQYLLISNT GIKHLPDVHKIHS			LRR4	2.8
144	LQKVLLDIQ DNINIHTIERNSFVGLSFE		LRR5	2.4
172	SVILWLNKN GIQEIHNCAFNGTQ		LRR6	3
195	LDELNLSDN NNLEELPNDVFHGASGPVILD		LRR7	4

SHIFT -22!

203	NNNLEELPN DVFHGASGPVILDISRTRIHS		LRR1	2
233	LPSYGLENL KKLRARSTYNLKKLPT		LRR2	2
258	LEKLVALME ASLTYPSHCCAFANWRRQ		LRR3	2.4
285	ISELHPICN KSILRQEVDYMTQARGQRSSL		LRR4	2.4

AEDNESSYSRGFDMTYTEFDYDLCNEVVDVTCSPKPDAFNPCEDIMGYNI

365	LRVLIWFIS ILAITGNIIVLVILTTSQYKL		LRR1	2.8
395	TVPRFLMCN LAFADLCIGIYLLLIASVDIH		LRR2	2

TKSQYHNYAIDWQTGAGCDAAGFFTVFASELSVYTLTAITLERWHTITHAMQLDCKVQLR
HAASVMVMGWIFAFAAA

502	LFPIFGISS YMKVSICLPMDIDSP		LRR1	2.2
526	LSQLYVMSL LVLNVLAFVVICGCYIHIYLT		LRR2	2.4

VRNPNIVSSSSDTRIAKRMAMLIFTDFLCMAPISFFAISASLKVPLITVSKAKILLVLFH
PINSCANPFLYAIFTKNFRRDFFILLSKCGCYEMQAQIYRTETSSTVHNTHPRNGHCSSA
PRVTSGSTYILVPLSHLAQN

>NP_067647

MTSGSVFFYILIFGKYFSHGGGQDVKCSLGYFPCGNITKCLPQLLHCNGVDDCGNQADED
NCGDNNGWSLQFDKYFASYYKMTSQYPFEAETPE

95	CLVGSVPVQCLCQGLELDCDETNLRAVPSVSSN		NF	4

SHIFT -22!

106	CQGLELDCD ETNLRAVPSVSSN			LRR1	2
128	VTAMSLQWN LIRKLPPDCFKNYHD		LRR2	2.8
152	LQKLYLQNN KITSISIYAFRGLNS		LRR3	4
176	LTKLYLSHN RITFLKPGVFEDLHR		LRR4	4
200	LEWLIIEDN HLSRISPPTFYGLNS		LRR5	3.4
224	LILLVLMNN VLTRLPDKPLCQHMPR		LRR6	4
249	LHWLDLEGN HIHNLRNLTFISCSN		LRR7	4
273	LTVLVMRKN KINHLNENTFAPLQK		LRR8	3.4
297	LDELDLGSN KIENLPPLIFKDLKE		LRR9	4
321	LSQLNLSYN PIQKIQANQFDYLVK		LRR10	4
345	LKSLSLEGI EISNIQQRMFRP			LRR11	3
366	LMNLSHIYF KKFQYCGYAPHVRSCKPNTDG		LRR12	2

ISSLENLLASIIQRVFVWVVSAVTCFGNIFVICMRPYIRSENKLYAMSIISLCCADCLMG
IYLFVIGGFDLKFRGEYNKHAQLWMESTHCQLVGSLAILSTEVSV

501	LLLTFLTLE KYICIVYPFRCVRPGKCRT		LRR1	2
529	ITVLILIWI TGFIVAFIPLSNKEFFKNYYG		LRR2	2.4

TNGVCFPLHSEDTESIGAQIYSVAIFLGINLAAFIIIVFSYGSMFYSVHQSAITATEIRN
QVKKEMILAKRFFFIVFTDALCWIPIFVVKF

650	LSLLQVEIP GTITSWVVIFILPINSALNPI		LRR1	2.4
680	LYTLTTRPF KEMIHRFWYNYRQRKSMDSKG		LRR2	2

QKTYAPSFIWVEMWPLQEMPPELMKPDLFTYPCEMSLISQSTRLNSYS

>ENSP00000312001

MKDMPLRIHVLLGLAITTLVQAVDKKVD

29	CPRLCTCEIRPWFTPRSIYMEASTVDCNDLGLLTFPARLPAN		NF	4
71	TQILLLQTN NIAKIEYSTDFPVN		LRR1	3
94	LTGLDLSQN NLSSVTNINVKKMPQ		LRR2	4
118	LLSVYLEEN KLTELPEKCLSELSN		LRR3	3.4
142	LQELYINHN LLSTISPGAFIGLHN		LRR4	3.4
166	LLRLHLNSN RLQMINSKWFDALPN		LRR5	4
190	LEILMIGEN PIIRIKDMNFKPLIN		LRR6	3.4
214	LRSLVIAGI NLTEIPDNALVGLEN		LRR7	2.4
238	LESISFYDN RLIKVPHVALQKVVN		LRR8	2.8
262	LKFLDLNKN PINRIRRGDFSNM			LRR9	4
284	LHLKELGIN NMPELISIDSLAVDNLPD		LRR10	3
311	LRKIEATNN PRLSYIHPNAFFRLPK		LRR11	2.8
336	LESLMLNSN ALSALYHGTIESLPN		LRR12	4
360	LKEISIHSN				LRR13	2.8
369	PIRCDCVIRWMNMNKTNIRFMEPDSLFCVDPPEFQGQNVRQVHFRDMMEI		CF1_short	3

CLPLIAPESFPSNLNVEAGSYVSFHCRATAEPQPEIYWITPSGQKLLPNTLTDKFYV

476	HSEGTLDIN GVTPKEGGLYTCIATN		LRR1	2.4
501	LVGADLKSV MIKVDGSFPQDNNGSLNIKIR		LRR2	2.4

DIQANSVLVSWKASSKILKSSVKWTAFVKTENSHAAQSARIPSDVKVYNLTHLNPSTEYK
ICIDIPTIYQKNRKKCVNVTTKGLHPDQKEYEKNNTTTLMACLGGLLGIIGVICL

646	ISCLSPEMN CDGGHSYVRNYLQKPTFA		LRR1	2.4
673	LGELYPPLI NLWEAGKEKSTSLKVK		LRR2	2
698	ATVIGLPTN MS			LRR3	2.8


>ENSP00000247535

MGFLSPIYVIFFFFGVKVHCQYETYQWDEDYDQEPDDDYQTGFPFRQNVDYGVPFHQYTL
G

62	CVSECFCPTNFPSSMYCDNRKLKTIPNIPMH		NF	4
93	IQQLYLQFN EIEAVTANSFINATH		LRR1	3.4
117	LKEINLSHN KIKSQKIDYGVFAKLPN		LRR2	3.4
143	LLQLHLEHN NLEEFPFPLPKS			LRR3	4
164	LERLLLGYN EISKLQTNAMDGLVN		LRR4	4
188	LTMLDLCYN YLHDSLLKDKIFAKMEK		LRR5	4
214	LMQLNLCSN RLESMPPGLPSS			LRR6	4
235	LMYLSLENN SISSIPEKYFDKLPK		LRR7	4
259	LHTLRMSHN KLQDIPYNIFNLPN		LRR8	3.4
282	IVELSVGHN KLKQAFYIPRNLEHLYLQNNE		LRR9	2.8

SHIFT -10!

302	LEHLYLQNN EIEKMNLTVMCPSIDPLHYHH		LRR1	4
332	LTYIRVDQN KLKEPISSYIFFCFPHIHTIY		LRR2	2.8

YGEQRSTNGQTIQLKTQVFRRFPDDDDESEDHDDPDNAHESPEQEGAEGHFDLHYYENQE

>ENSP00000266581

MS

3	LRVHTLPTL LGAVVRPGCREL			LRR1	2
24	LCLLMITVT VGPGASGVCPTACICATDIVS		LRR2	2.8

CTNKNLSKVPGN

66	LFRLIKRLD LSYNRIGLLDSEWIPVSFAK		LRR1	2
95	LNTLILRHN NITSISTGSFSTTPN		LRR2	4
119	LKCLDLSSN KLKTVKNAVFQELKV		LRR3	4
143	LEVLLLYNN HISYLDPSAFGGLSQ		LRR4	4
167	LQKLYLSGN FLTQFPMDLYVGRFKLAE		LRR5	4
194	LMFLDVSYN RIPSMPMHHINLVPGKQ		LRR6	3.4
220	LRGIYLHGN				LRR7	3.4
229	PFVCDCSLYSLLVFWYRRHFSSVMDFKNDYTCRLWSDSRHSRQVLLLQDSFMNC		CF1_short	3

SDSIINGSFRALGFIHEAQVGERLMVHCDSKTGNANTDFIWVGPDNRLLEPDKEMENFYV
FHNGSLVIESPRFEDAGVYSCIAMNKQRLLNETVDVTINVSNFTVSRSHAHEAFNTAFTT
LAACVASIVLVLLYLYLTPCPCKCKTKRQKNMLHQSNAHSSILSPGPASDASADERKAGA
GKRVVF

469	LEPLKDTAA GQNGKVRLFPSEAV		LRR1	2
492	IAEGILKST RGKSDSDSVNSVFSDTPFVAS		LRR2	2.2

T

>ENSP00000259324

MIPVTELRYFADTQPAYRILKPWWDVFTDYISIVMLMIAVFGGTLQVTQDKMICLPCKWV
TKDSCNDSFRGWAAPGPEPTYPNSTILPTPDTGPTGIKYDLDRHQYNYVDAVCYENRLHW
FAKYFPYLVLLHTLIFLACSNFWFKFPRTSSKLEHFVSILLKCFDSPWTTRALSETVVEE
SDPKPAFSKMNGSMDKKSSTVSEDVEATVPMLQRTKSRIEQGIVDRSETGVLDKKEGEQA
KALFEKVKKFRTHVEEGDIVYRLYMRQTIIK

272	VIKFILIIC YTVYYVHNIKFDVDCTVD		LRR1	2.2
299	IESLTGYRT YRCAHPLATLFKILASFYIS		LRR2	2.2
328	LVIFYGLIC MYTLWWMLRRSLKKYSFESIR		LRR3	2.2

EESSYSDIPDVKNDF

373	AFMLHLIDQ YDPLYSKRFAVFLSEVSENK		LRR1	2.4
402	LRQLNLNNE WTLDKLRQRLTKNAQDKLE		LRR2	3
430	LHLFMLSGI PDTVFDLVELEVLKLELIPDV		LRR3	2.4

SHIFT -15!

445	LVELEVLKL ELIPDVTIPPSIAQLTG		LRR1	2.4
471	LKELWLYHT AAKIEAPALAFLREN		LRR2	3.4
495	LRALHIKFT DIKEIPLWIYSLKT		LRR3	2.8
518	LEELHLTGN LSAENNRYIVIDGLRELKR		LRR4	4
546	LKVLRLKSN LSKLPQVVTDVG			LRR5	4
567	VHLQKLSIN NEGTKLIVLNSLKKMAN		LRR6	2.4
593	LTELELIRC DLERIPHSIFSLHN		LRR7	3.4
616	LQEIDLKDN NLKTIEEIISFQHLHR		LRR8	3.4
641	LTCLKLWYN HIAYIPIQIGNLTN		LRR9	4
664	LERLYLNRN KIEKIPTQLFYCRK		LRR10	4
687	LRYLDLSHN NLTFLPADIGLLQN		LRR11	4
710	LQNLAITAN RIETLPPELFQCRK		LRR12	3.4
733	LRALHLGNN VLQSLPSRVGELTN		LRR13	4
756	LTQIELRGN RLECLPVELGECPL		LRR14	3.4
779	LKRSGLVVE EDLFNTLPPEVKERLWRADKE		LRR15	2

QA

>ENSP00000223661

MKIAVLFCFFLLIIFQTDFGKNEEIPRKQRRKIYHRRLRKSSTSHKHRSNRQLGIQQTTV
FTPVARLPIVNFDYSMEEKFESFSSFPGVESSYNVLPGKKGHCLVKGITMYNKAVWSPEP

121	CTTCLCSDGRVLCDETMCHPQRCPQTVIPEGECCPVCS		NF	4

ATVSYSL

166	LSGIALNDR NEFSGDSSEQREPTN		LRR1	2.4
190	LLHKQLPPP QVGMDRIVRKEALQSEEDEEV		LRR2	2

KEEDTEQKRETPESRNQGQLYSEGDSRGGDRKQRPGEERRLAHQQQRQGREEEEDEEEEG
EEGEEDEEDEEDPVRGDMFRMPSRSPLPAPPRGTLRLPSGCSLSYRTISCINAMLTQIPP
LTAPQ

345	ITSLELTGN SIASIPDEAFNGLPN		LRR1	3.4
369	LERLDLSKN NITSSGIGPKAFKLLKK		LRR2	4
395	LMRLNMDGN NLIQIPSQLPST			LRR3	3.4
416	LEELKVNEN NLQAIDEESLSDLNQ		LRR4	3.4
440	LVTLELEGN NLSEANVNPLAFKPLKS		LRR5	4
466	LAYLRLGKN KFRIIPQGLPGS			LRR6	4
487	IEELYLENN QIEEITEICFNHTRK		LRR7	3.4
511	INVIVLRYN KIEENRIAPLAWINQEN		LRR8	2.8
537	LESIDLSYN KLYHVPSYLPKS			LRR9	3.4
558	LLHLVLLGN QIERIPGYVFGHMEPG		LRR10	4
583	LEYLYLSFN KLADDGMDRVSFYGAYHS		LRR11	4
610	LRELFLDHN DLKSIPPGIQEMKA		LRR12	4
633	LHFLRLNNN				LRR13	4
642	KIRNILPEEICNAEEDDDSNLEHLHLENNYIKIREIPSYTFSCI		CF2	2

SHIFT -53!

633	LHFLRLNNN KIRNILPEEICNAEEDDDSN		LRR13	4
662	LEHLHLENN YIKIREIPSYTFSCIRSYSSI		LRR14	4

VLKPQNIK

>ENSP00000246529

MAILP

6	LLLCLLPLA PASSPPQSATPSP		LRR1	2
28	CPRRCRCQTQSLPLSVLCPGAGLLFVPPSLDRR		NF	4

SHIFT -25!

36	TQSLPLSVL CPGAGLLFVPPSLDRR		LRR2	2
61	AAELRLADN FIASVRRRDLANMTG		LRR3	3.4
85	LLHLSLSRN TIRHVAAGAFADLRA		LRR4	4
109	LRALHLDGN RLTSLGEGQLRGLVN		LRR5	4
133	LRHLILSNN QLAALAAGALDDCAET		LRR6	4
158	LEDLDLSYN NLEQLPWEALGRLGN		LRR7	4
182	VNTLGLDHN LLASVPAGAFSRLHK		LRR8	3.4
206	LARLDMTSN RLTTIPPDPLFSR			LRR9	3.4
228	LPLLARPRG SPASALVLAFGGNPLHCNCE		LRR10	2
257	LVWLRRLAR EDDLEACASPPALGGRYFWAV		LRR11	2

GEEEFVCEPPVVTHRSPPLAVPAGRPAALRCRAVGDPEPRVRWVSPQGRLLGNSSRARAF
PNGTLELLVTEPGDGGIFTCIAANAAGEATAAVELTVGPPPPPQLANSTSCDPPRDGDPD
ALTPPSAASASAKVADTGPPTDRGVQVTEHGATAALVQWPDQRPIPGIRMYQIQYNSSAD
DILVYRMIPAESRSFLLTDLASGRTYDLCVLAVYEDSATGLTATRPVGCARFSTEPALRP
CGAPHAPFLGGTMIIALGGVIVASVLVFIFVLLMRYKVHGGQPPGKAKIPAPVSSVCSQT
NGALGPTPTPAPPAPEPAALRAHTVVQLDCEPWGPGHEPVGP

>ENSP00000277778

MRVALGMLWLLALAWPPQARGF

23	CPSQCSCSLHIMGDGSKARTVVCNDPDMTLPPASIPPD		NF	4
61	TSRLRLERT AIRRVPGEAFRPLGR		LRR1	2.4
85	LEQLWLPYN ALSELNALMLRGLRR		LRR2	4
109	LRELRLPGN RLAAFPWAALRDAPK		LRR3	4
133	LRLLDLQAN RLSAVPAEAARFLEN		LRR4	4
157	LTFLDLSSN QLMRLPQELIVSWAHLETGIF		LRR5	4

SHIFT -20!

167	LMRLPQELI VSWAHLETGIFPPGHHP		LRR1	2
193	RRVLGLQDN				LRR2	3
202	PWACDCRLYDLVHLLDGWAPNLAFIETELRCASPRSLAGVAFSQLELRKCQGP		CF1	4

SHIFT -62!

193	RRVLGLQDN PWACDCRLYDLVHLLDGW		LRR2	3
220	APNLAFIET ELRCASPRSLAGVA		LRR3	2.2
243	FSQLELRKC QGPELHPGVASIRSL		LRR4	2.8
267	LGGTALLRC GATGVPGPEMSWRRANGRPLN		LRR5	2.4

GTVHQEVSSDGTSWTLLGLPAVSHLDSGDYICQAKNFLGASETVISLIVTEPPTSTEHSG
SPGALWARTGGGGEAAAYNNKLVARHVPQIPKPAVLATGPSVPSTKEELTLEHFQMDALG
ELSDGRAGPSEARMVRSVKVVGDTYHSVSLVWKAPQAKNTTAFSVLYAVFGQHSMRRVIV
QPGKTRVTITGLLPKTKYVACVCVQGLVPRKEQCVIFSTNEVVDAENTQQLINVVVISVA
IVIALPLTLLVCCSALQKRCRKCFNKDSTEATVTYVNLERLGYSEDGLEELSRHSVSEAD
RLLSARSSVDFQAFGVKGGRRINEYFC

>NP_065902

MFPLRALWLVWALLGVAGS

20	CPEPCACVDKYAHQFADCAYKELREVPEGLPAN		NF	4
53	VTTLSLSAN KITVLRRGAFADVTQ		LRR1	3.4
77	VTSLWLAHN EVRTVEPGALAVLSQ		LRR2	3.4
101	LKNLDLSHN FISSFPWSDLRNLSA		LRR3	4
125	LQLLKMNHN RLGSLPRDALGALPD		LRR4	3.4
149	LRSLRINNN RLRTLAPGTFDALSA		LRR5	3.4
173	LSHLQLYHN				LRR6	4
182	PFHCGCGLVWLQAWAASTRVSLPEPDSIACASPPALQGVPVYRLPALPCAPP		CF1	4

SVHLSAEPPLEAPGTPLRAGLAFVLHCIADGHPTPRLQWQLQIPGGTVVLEPPVLSGEDD
GVGAEEGEGEGDGDLLTQTQAQTPTPAPAWPAPPAT

330	PRFLALANG SLLVPLLSAKEAGVYTC		LRR1	2
356	RAHNELGAN STSIRVAVAATGPPKHAPGAG		LRR2	2

GEPDGQAPTSERKSTAKGRGNSVLPSKPEGKIKGQGLAKVSILGETETEPEEDTSEGEEA
EDQILADPAEEQRCGNGDPSRYVSNHAFNQSAELKPHVFELGVIALDVAEREARVQ

502	LTPLAARWG PGPGGAGGAPRPGRRP		LRR1	2.4
527	LRLLYLCPA GGGAAVQWSRVEEGVNAYW		LRR2	3
555	FRGLRPGTN YSVCLALAGEACHVQVVFSTK		LRR3	2.4

SHIFT -20!

565	SVCLALAGE ACHVQVVFSTKKE			LRR1	2
587	LPSLLVIVA VSVFLLVLATVPLLGAACCHL		LRR2	2.4

SHIFT -19!

598	VFLLVLATV PLLGAACCHLLAKHPGK		LRR1	2.4
624	PYRLILRPQ APDPMEKRIAADFDPRASYLE		LRR2	2

SEKSYPAGGEAGGEEPEDVQGEGLDEDAEQGDPSGDLQREES

696	LAACSLVES QSKANQEEFEAGSEYSDR		LRR1	2.4
723	LPLGAEAVN IAQEINGNYRQTAG		LRR2	2.4


>ENSP00000294954

MKQRFSALQLLKLLLLLQPPLPRALREALCPEPCNCVPDGALRCPGPTAGLTRLSLAYLP
VKVIPSQAFRGLNEVIKIEISQIDSLERIEANAF

95	DNLLNLSEI LIQNTKNLRYIEPGAFINLPR		LRR1	2
125	LKYLSICNT GIRKFPDVTKVFSSES		LRR2	2.8
150	NFILEICDN LHITTIPGNAFQGMNNE		LRR3	2.4
176	SVTLKLYGN GFEEVQSHAFNGTT		LRR4	3
199	LTSLELKEN VHLEKMHNGAFRGATGPKTLD		LRR5	4

SHIFT -22!

207	NVHLEKMHN GAFRGATGPKTLDISSTK		LRR1	2
234	LQALPSYGL ESIQRLIATSSYS			LRR2	2
256	LKKLPSRET FVNLLEATLTYPSHCCAFRNL		LRR3	2.4

PTKEQNFSHSISENFSKQCESTVRKVNNKTLYSSMLAESELSGWDYEYGFCLPKTPRCAP
EPDAFNPCEDIMGYDF

362	LRVLIWLIN ILAIMGNMTVLFVLLTSRYKL		LRR1	3.4
392	TVPRFLMCN LSFADFCMGLYLLLIASVDSQ		LRR2	2

TKGQYYNHAIDWQTGSGCSTAGFFTVFASE

452	LSVYTLTVI TLERWHTITYAIHLDQKLR		LRR1	2
480	LRHAILIML GGWLFSSLIAMLPLVGVSNYM		LRR2	2.4

SHIFT -13!

497	IAMLPLVGV SNYMKVSICFPMDVETTLSQV		LRR1	2.4
527	YILTILILN VVAFFIICACYIKIYFAVRNP		LRR2	2

ELMATNKDTKIAKKMAILIFTDFTCMAPISFFAISAAFKVPLITVTNS

605	KVLLVLFYP INSCANPFLYAIFTKTFQR		LRR1	2
633	DFFLLLSKF GCCKRRAELYRRKDFSAYTSN		LRR2	2

CKNGFTGSNKPSQSTLKLSTLHCQGTALLDKTRYTEC

>ENSP00000351486

MLRGGRRGQLGWHSWAAGPGSLLAWLI

28	LASAGAAPC PDACCPHGSSGLRCTRDGA		LRR1	2.2
56	LDSLHHLPG AENLTELYIENQQH		LRR2	2
79	LQHLELRDL RGLGELRNLTIVKSGLRFVAP		LRR3	3

SHIFT -22!

87	LRGLGELRN LTIVKSGLRFVAPDAFHFTPR		LRR1	3
117	LSRLNLSFN ALESLSWKTVQGLS		LRR2	4
140	LQELVLSGN				LRR3	4
149	PLHCSCALRWLQRWEEEGLGGVPEQKLQCHGQGPLAHMPNASCGVP		CF1	4

SHIFT -39!

156	LRWLQRWEE EGLGGVPEQKLQCHG		LRR1	2
180	QGPLAHMPN ASCGVPTLKVQVPNASVDVGD		LRR2	2

DVLLRCQVEGRG

222	LEQAGWILT ELEQSATVMKSGG			LRR1	2.2
244	LPSLGLTLA NVTSDLNRKNVTCWAENDVGR		LRR2	3

AEVSVQVNVSFPASVQLHTAVEMHHWCIPFSVDGQPAPS

313	LRWLFNGSV LNETSFIFTEFLEPAANET		LRR1	2
341	VRHGCLRLN QPTHVNNGNYTLLAANPFGQA		LRR2	2.8

SASIMAAFMDNPFEFNPEDPIPVSFSPVDTNSTSGDPVEKKDETPFGVSVAVGLAVFACL
FLSTL

436	LLVLNKCGR RNKFGINRPAVLAPEDGLAMS		LRR1	2
466	LHFMTLGGS SLSPTEGKGSGLQGHIIENPQ		LRR2	2.8

YFSDACVHHIKRRDIVLKWELGEGAFGKVFLAECHNLLPEQDKMLVAVKALKEASESARQ
DFQREAELLTMLQHQHIVRFFGVCTEGRPLLMVFEYMRHGDLNRFLRSHGPDAKLLAGGE
DVA

619	PGPLGLGQL LAVASQVAAGMVY			LRR1	2
641	LAGLHFVHR DLATRNCLVGQGLVVKIGDFG		LRR2	2.4

MSRDIYSTDYYRVGGRTMLPIRWMPPESILYRKFTTESDVWSFGVVLWEIFTYGKQPWYQ
LSNTEAIDCITQGRELERPRACPPEVYAIMRGCWQREPQQRHSIKDVHARLQALAQAPPV
YLDVLG

>ENSP00000294818

MSLASGPGPGWLLFSFGMG

20	LVSGSKCPN NCLCQAQEVICTGKQLT		LRR1	2.4
26	CPNNCLCQAQEVICTGKQLTEYPLDIPLN		NF	4

SHIFT -9!

46	EYPLDIPLN TRRLFLNENRITS			LRR2	2.4
68	LPAMHLGLL SDLVYLDCQNNRIREVMDYTF		LRR3	2.4

SHIFT -25!

73	LGLLSDLVY LDCQNNRIREVMDYTFIGVFK		LRR1	2
103	LIYLDLSSN NLTSISPFTFSVLSN		LRR2	4
127	LVQLNIANN PHLLSLHKFTFANTTS		LRR3	3.4
152	LRYLDLRNT GLQTLDSAALYHLTT		LRR4	3.4
176	LETLFLSGN				LRR5	4
185	PWKCNCSFLDFAIFLIVFHMDPSDDLNATCVEPTELTGWPITRVGNPLRYMC		CF1_short	3

SHIFT -61!

176	LETLFLSGN PWKCNCSFLDFAIF		LRR5	4
199	LIVFHMDPS DDLNATCVEPTELTGWPITRV		LRR6	2.2

GNPLRYMCITHLDHKD

245	YIFLLLIGF CIFAAGTVAAWLT			LRR1	2
267	GVCAVLYQN TRHKSSEEDEDEAGTRVEVSR		LRR2	2.8

RIFQTQTSSVQEFPQLI

>ENSP00000249842

MQELHLLWWALLLGLAQA

19	CPEPCDCGEKYGFQIADCAYRDLESVPPGFPAN		NF	4
52	VTTLSLSAN RLPGLPEGAFREVPL		LRR1	3.4
76	LQSLWLAHN EIRTVAAGALASLSH		LRR2	4
100	LKSLDLSHN LISDFAWSDLHNLSA		LRR3	4
124	LQLLKMDSN ELTFIPRDAFRSLRA		LRR4	3.4
148	LRSLQLNHN RLHTLAEGTFTPLTA		LRR5	4
172	LSHLQINEN				LRR6	3.4
181	PFDCTCGIVWLKTWALTTAVSIPEQDNIACTSPHVLKGTPLSRLPPLPCSAP		CF1	4

SHIFT -42!

191	LKTWALTTA VSIPEQDNIACTSPHVLKGTP		LRR1	2.4
221	LSRLPPLPC SAPSVQLSYQPSQDGAELRP		LRR2	2.4
250	GFVLALHCD VDGQPAPQLHWHIQIPSGIVE		LRR3	2.4

ITSPNVGTDGRALPGTPVASSQPRFQAFANGSLLIPDFGKLEEGTYSCLATNELGSAESS
VDVALATPGEGGEDTLGRRFHGKAVEGKGCYTVDNEVQPSGPEDNVV

387	IIYLSRAGN PEAAVAEGVPGQ			LRR1	2.4
408	LPPGLLLLG QSLLLFFFLTSF			LRR2	2.4


>ENSP00000298025

MHTCCPPVTLEQDLHRKMHSWMLQTLAFAVTSLVLSCAETIDYYGEI

48	CDNACPCEEKDGILTVSCENRGIISLSEISPPR		NF	4

FP

83	IYHLLLSGN LLNRLYPNEFVNYTG		LRR1	3.4
107	ASILHLGSN VIQDIETGAFHGLRG		LRR2	3.4
131	LRRLHLNNN KLELLRDDTFLGLEN		LRR3	4
155	LEYLQVDYN YISVIEPNAFGKLHL		LRR4	3.4
179	LQVLILNDN LLSSLPNNLFRFVP		LRR5	4
202	LTHLDLRGN RLKLLPYVGLLQHMDK		LRR6	4
227	VVELQLEEN				LRR7	3.4
236	PWNCSCELISLKDWLDSISYSALVGDVVCETPFRLHGRDLDEVSKQELCPR		CF1_short	3

SHIFT -44!

243	LISLKDWLD SISYSALVGDVVCETPFR		LRR1	2
270	LHGRDLDEV SKQELCPRRLISDYEMRPQTP		LRR2	2

SHIFT -17!

283	LCPRRLISD YEMRPQTPLSTTGY		LRR1	2
306	LHTTPASVN SVATSSSAVYKPPLKPPKGTR		LRR2	2.4

QPNKPRVRPTSRQPSKDLGYSNYGPSIAYQTKSPVPLE

374	CPTACSCNLQISDLGLNVNCQERKIESIAELQPKP		NF	4

SHIFT -27!

382	LQISDLGLN VNCQERKIESIAELQPKPYN		LRR1	3
411	PKKMYLTEN YIAVVRRTDFLEATG		LRR2	2.4
435	LDLLHLGNN RISMIQDRAFGDLTN		LRR3	4
459	LRRLYLNGN RIERLSPELFYGLQS		LRR4	4
483	LQYLFLQYN LIREIQSGTFDPVPN		LRR5	4
507	LQLLFLNNN LLQAMPSGVFSGLT		LRR6	4
530	LLRLNLRSN HFTSLPVSGVLDQLKS		LRR7	4
555	LIQIDLHDN				LRR8	3.4
564	PWDCTCDIVGMKLWVEQLKVGVLVDEVICKAPKKFAETDMRSIKSELLCPD		CF1_short	3

SHIFT -60!

555	LIQIDLHDN PWDCTCDIVGMKLWVEQ		LRR8	3.4
581	LKVGVLVDE VICKAPKKFAETDMRSIKSEL		LRR9	2.4

LCPDYSDVVVSTPTPSSIQVPARTSAVTPAVRLNSTGAPASLGAGGGASSVP

663	LSVLILSLL LVFIMSVFVAAG			LRR1	3
684	LFVLVMKRR KKNQSDHTSTNNSDVSSFNMQ		LRR2	2.4

YSVYGGGGGTGGHPHAHVHHRGPALPKVKTPAGHVYEYIPHPLGHMCKNPIYRSREGNSV
EDYKDLHELKVTYSSNHHLQQQQQPPPPPQQPQQQPPPQLQLQPGEEERRESHHLRSPAY
SVSTIEPREDLLSPVQDADRFYRGILEPDKHCSTTPAGNSLPEYPKFPCSPAAYTFSPNY
DLRRPHQYLHPGAGDSR

911	LREPVLYSP PSAVFVEPNRNE			LRR1	2
932	YLELKAKLN VEPDYLEVLEKQTTFSQF		LRR2	2.4


>ENSP00000333767

MDAATAPKQAWLPWSPLLFLLLLPGGSISS

31	CPTVCDCTSQTRAVFCAHRRLDTIPGGLPLD		NF	4

SHIFT -7!

55	PGGLPLDTE LLDLSGNRLWGLQRGM		LRR1	2
80	LSRLGQLQE LDLSYNQLSTLEPGAFHGLQS		LRR2	2
110	LLTLRLQGN RLRIVGPGIFSGLTA		LRR3	4
134	LTLLDLRLN QIVLFLDGAFSELGS		LRR4	4
158	LQQLEVGDN HLVFVAPGAFAGLAK		LRR5	3.4
182	LSTITLERC NLSTVPGLALAQLPA		LRR6	2.8
206	LVALRLREL DIERLPAGALRGLGQ		LRR7	3
230	LKELEIHHW PSLEALDPGSLVGLN		LRR8	2.4
254	LSSLAITRC NLSSVPFQALHHLSF		LRR9	2.8
278	LRILDLSQN PISAIPARRLSPLVR		LRR10	4
302	LQELRLSGA CLTSIAAHAFHGLTA		LRR11	3
326	FHLLDVADN ALQTLEETAFPSPDK		LRR12	2.8
350	LVTLRLSGN				LRR13	4
359	PLTCDCRLLWLLRLRRRLDFGTSPPACAGPQHVQGKSLREFSDILPPGH		CF1_short	3

FTCKPALIRKSGPRWVIAEEGGHAVFSCSGDGDPAPTVSWMRPQGAWLGRVGRVRVLEDG
TLEIRSVQLRDRGAYVCVVSNVAGNDSLRTWLEVIQVEPPNGTLSDPNITMPGIPGPFFL
DSRGVAMVLAVGFLPFLTSVTLCFGLIALWSKGKGRVKHHMTFDFVAPRPSGDKNSGGNR
VTAKLF

>ENSP00000333227

MAGRGWGALWVCVAAATLLHAGGLARADCWLIEGDKGFVWLAICSQNQPPYEAIPQQIN

60	STIVDLRLN ENRIRSVQYASLSRFGN		LRR1	2.4
86	LTYLNLTKN EIGYIEDGAFSGQFN		LRR2	4
110	LQVLQLGYN RLRNLTEGMLRGLGK		LRR3	4
134	LEYLYLQAN LIEVVMASSFWECPN		LRR4	4
158	IVNIDLSMN RIQQLNSGTFAGLAK		LRR5	2.8
182	LSVCELYSN				LRR6	3
191	PFYCSCELLGFLRWLAAFTNATQTYDRMQCESPPVYSGYYLLGQGRRGHRSI		CF1_short	3

LSKLQSVCTEDSYAAEVVGPPRPASGRSQPGRSPPPPPPPEPSDMPCADDECFSGDGTTP
LVALPTLATQAEARPL

319	IKVKQLTQN SATITVQLPSPFHR		LRR1	2.4
342	MYTLEHFNN SKASTVSRLTKAQEEIR		LRR2	2.4
368	LTNLFTLTN YTYCVVSTSAGLRHNHTCLTI		LRR3	3

SHIFT -27!

371	LFTLTNYTY CVVSTSAGLRHNHTCLTIC		LRR1	2
399	LPRLPSPPG PVPSPSTATHYIMTI		LRR2	2
423	LGCLFGMVL VLGAVYYCLRRRRRQEEKHKK		LRR3	2.4

SHIFT -25!

428	GMVLVLGAV YYCLRRRRRQEEKHKKAASA		LRR1	2.4
457	AAAGSLKKT IIELKYGPELEAPGLAP		LRR2	2.2
483	LSQGPLLGP EAVTRIPYLPAAGEVEQYKLV		LRR3	2.4

ESADTPKASKGSYMEVRTGDPPERRDCELGRPGPDSQSSVAEISTIAKEVDKVNQIINNC
IDALKSESTSFQGVKSGPVSVAEPPLVLLSEPLAAKHGFLAPGYKDAFGHSLQRHHSVEA
AGPPRASTSSSGSVRSPRAFRAEAVGVHKAAAAEAKYIEKGSPAADAILTVTPAAAVLRA
EAEKGRQYGEHRHSYPGSHPAEPPAPPGPPPPPPHEGLGRKASILEPLTRPRPRDLAYSQ
LSPQYHSLSYSSSPEYTCRASQSIWERFRLSRRRHKEEEEFMAAGHALRKKVQFAKDEDL
HDILDYWKGVSAQHKS

>ENSP00000326759

MSAPSLRARAAGLGLLLCAVLGRAGRSDSGGRGELGQPSGVAAERP

47	CPTTCRCLGDLLDCSRKRLARLPEPLPSW		NF	4

SHIFT -22!

54	LGDLLDCSR KRLARLPEPLPSW			LRR1	2
76	VARLDLSHN RLSFIKASSMSHLQS		LRR2	3.4
100	LREVKLNNN ELETIPNLGPVSAN		LRR3	3.4
123	ITLLSLAGN RIVEILPEHLKEFQS		LRR4	3.4
147	LETLDLSSN NISELQTAFPALQ			LRR5	4
169	LKYLYLNSN RVTSMEPGYFDNLANT		LRR6	4
194	LLVLKLNRN RISAIPPKMFKLPQ		LRR7	4
217	LQHLELNRN KIKNVDGLTFQGLGA		LRR8	4
241	LKSLKMQRN GVTKLMDGAFWGLSN		LRR9	3.4
265	MEILQLDHN NLTEITKGWLYGLLM		LRR10	3.4
289	LQELHLSQN AINRISPDAWEFCQK		LRR11	4
313	LSELDLTFN HLSRLDDSSFLGLSL		LRR12	4
337	LNTLHIGNN RVSYIADCAFRGLSS		LRR13	3.4
361	LKTLDLKNN EISWTIEDMNGAFSGLDK		LRR14	4
388	LRRLILQGN RIRSITKKAFTGLDA		LRR15	4
412	LEHLDLSDN AIMSLQGNAFSQMKK		LRR16	4
436	LQQLHLNTS				LRR17	3.4
445	SLLCDCQLKWLPQWVAENNFQSFVNASCAHPQLLKGRSIFAVSPDGFVCD		CF1_short	3

DFPKPQITVQPETQSAIK

513	GSNLSFICS AASSSDSPMTFAWKKDNE		LRR1	2.2
540	LLHDAEMEN YAHLRAQGGEVMEY		LRR2	2
563	TTILRLREV EFASEGKYQCVISNHFGSSY		LRR3	2
592	SVKAKLTVN MLPSFTKTPMDLTIRAGAMAR		LRR4	2.4

LECAAVGHPAPQIAWQKDGGTDFPAARERRMHVMPEDDVFFIVDVKIEDIGVYSCTAQNS
AGSISANATLTVLETPSFLRPLLDRTVTKGETAVLQCIAGGSPPPKLNWTKDDSPLVVTE
RHFFAAGNQLLIIVDSDVSDAGKYTCEMSNTLGTERGNVRLSVIPTPTCDSPQMTAPSLD
DDGWATVGVVIIAVVCCVVGTSLVWVVIIYHTRRRNEDCSITNTDETNLPADIPSYLSSQ
GTLADRQDGYVSSESGSHHQFVTSSGAGFFLPQHDSSGTCHIDNSSEADVEAATDLFLCP
FLGSTGPMYLKGNVYGSDPFETYHTGCSPDPRTVLMDHYEPSYIKKKECYPCSHPSEESC
ERSFSNISWPSHVRKLLNTSYSHNEGPGMKNLC

1015	LNKSSLDFS ANPEPASVASSNSFMGTFGKA		LRR1	2.4
1045	LRRPHLDAY SSFGQPSDCQPR			LRR2	2
1066	AFYLKAHSS PDLDSGSEEDGKERTDFQEEN		LRR3	2.2

HICTFKQTLENYRTPNFQSYDLDT

>ENSP00000300147

MLRLGL

7	CAAALLCVCRPGAVRADCWLIEGDKGYVWLAIC		NF	4

SQNQPPYETIPQHIN

55	STVHDLRLN ENKLKAVLYSSLNRFGN		LRR1	2
81	LTDLNLTKN EISYIEDGAFLGQSS		LRR2	4
105	LQVLQLGYN KLSNLTEGMLRGMSR		LRR3	4
129	LQFLFVQHN LIEVVTPTAFSECPS		LRR4	3.4
153	LISIDLSSN RLSRLDGATFASLAS		LRR5	3.4
177	LMVCELAGN				LRR6	3
186	PFNCECDLFGFLAWLVVFNNVTKNYDRLQCESPREFAGYPLLVPRPYHSLNA		CF1_short	3

ITVLQAKCRNGSLPARPVSHPTPYSTDAQREPDENSGFNPDEILSVEPPASSTTDASAGP
AIKLHHVTFTSATLVVIIPHPYSK

322	MYILVQYNN SYFSDVMTLKNKKEIVT		LRR1	2.4
348	LDKLRAHTE YTFCVTSLRNSRRFNHTCLTF		LRR2	2.4

TTRDPVPGDLAPSTSTTTHYIMTILGCLFGMVIVLGAVYYCLRKRRMQEEKQKSVNVKKT
ILEMRYGADVDAGSIVHAAQKLGEPPVLPVSRMASIPSMIGEKLPTAKGLEAGLDTPKVA
TKGNYIEVRTGAGGDGLARPEDDLPDLENGQGSAAEISTIAKEVDKVNQIINNC

552	IDALKLDSA SFLGGGSSSGDPELAFECQS		LRR1	2.4
581	LPAAAAASS ATGPGALERPSFLSPPYKESS		LRR2	2.2

HHPLQRQLSADAAVTRKTCSVSSSGSIKSAKVFSLDVPDHPAATGLAKGDSKYIEKGSPL
NSPLDRLPLVPAGSGGGSGGGGGIHHLEVKPAYHCSEHRHSFPALYYEEGADSLSQRVSF
LKPLTRSKRDSTYSQLSPRHYYSGYSSSPEYSSESTHKIWERFRPYKKHHREEVYMAAGH
ALRKKVQFAKDEDLHDILDYWKGVSAQQKL

>ENSP00000332164

MAPGWAGVGAAVRARL

17	ALALALASV LSGPPAVACPTKCTCSAASVD		LRR1	2.4
47	CHGLGLRAV PRGIPRNAERLDLDRNNITRI		LRR2	2

SHIFT -14!

63	AERLDLDRN NITRITKMDFAGLKN		LRR1	3.4
87	LRVLHLEDN QVSVIERGAFQDLKQ		LRR2	4
111	LERLRLNKN KLQVLPELLFQSTPK		LRR3	4
135	LTRLDLSEN QIQGIPRKAFRGITD		LRR4	4
159	VKNLQLDNN HISCIEDGAFRALRD		LRR5	3.4
183	LEILTLNNN NISRILVTSFNHMPK		LRR6	4
207	IRTLRLHSN				LRR7	3.4
216	HLYCDCHLAWLSDWLRQRRTVGQFTLCMAPVHLRGFNVADVQKKEYVCP		CF1_short	3

APHSEPPSCNANSIS

280	CPSPCTCSNNIVDCRGKGLMEIPANLPEG		NF	4
309	IVEIRLEQN SIKAIPAGAFTQYKK		LRR1	2.8
333	LKRIDISKN QISDIAPDAFQGLKS		LRR2	2.8
357	LTSLVLYGN KITEIVKGLFDGLVS		LRR3	4
381	LQLLLLNAN KINCLRVNTFQDLQN		LRR4	4
405	LNLLSLYDN KLQTISKGLFAPLQS		LRR5	4
429	IQTLHLAQN				LRR6	3.4
438	PFVCDCHLKWLADYLQDNPIETSGARCSSPRRLANKRISQIKSKKFRCS		CF1_short	3

GSEDYRSRFSSECFMDLV

505	CPEKCRCEGTIVDCSNQKLVRIPSHLP		NF	4
532	EYVTDLRLN DNEVSVLEATGIFKKLPN		LRR1	2
559	LRKINLSNN KIKEVREGAFDGAAS		LRR2	3.4
583	VQELMLTGN QLETVHGRVFRGLSG		LRR3	3.4
607	LKTLMLRSN LIGCVSNDTFAGLSS		LRR4	4
631	VRLLSLYDN RITTITPGAFTTLVS		LRR5	3.4
655	LSTINLLSN				LRR6	3.4
664	PFNCNCHLAWLGKWLRKRRIVSGNPRCQKPFFLKEIPIQDVAIQDFTCD		CF1_short	3

GNEESSCQLSPR

725	CPEQCTCMETVVRCSNKGLRALPRGMPKD		NF	4

SHIFT -11!

743	LRALPRGMP KDVTELYLEGNHLTAVPRE		LRR1	2
771	LSALRHLTL IDLSNNSISMLTNYTFSNMSH		LRR2	2
801	LSTLILSYN RLRCIPVHAFNGLRS		LRR3	4
825	LRVLTLHGN DISSVPEGSFNDLTS		LRR4	4
849	LSHLALGTN				LRR5	4
858	PLHCDCSLRWLSEWVKAGYKEPGIARCSSPEPMADRLLLTTPTHRFQCK		CF1_short	3

SHIFT -42!

865	LRWLSEWVK AGYKEPGIARCSSPEPM		LRR1	2
891	ADRLLLTTP THRFQCKGPVDINIVAKCNAC		LRR2	2.4

LSSPCKNNGTCTQDPVELYRCACPYSYKGKDCTVPINTCIQNPCQHGGTCHLSDSHKDGF
SCSCPLGFEGQRCEINPDDCEDNDCENN

1009	ATCVDGINN YVCICPPNYTGELCDEVIDHC		LRR1	2.2
1011	CVDGINNYVCICPPNYTGELCDEVIDHCVPELNLCQHEAKCIP		NF	4

SHIFT -15!

1039	VPELNLCQH EAKCIPLDKGFSCECVP		LRR2	2.4
1065	GYSGKLCET DNDDCVAHKCRHGAQCVDTIN		LRR3	2.2

GYTCTCPQGFSGPFCEHPPPMVLLQTSPCDQYECQNGAQCIVVQQEPTCRCPPGFAGPRC
EKLITVNFVGKDSYVELASAKVRPQANISLQVATDKDN

1193	GILLYKGDN DPLALELYQGHVR			LRR1	2.4
1215	LVYDSLSSP PTTVYSVETVNDGQFHSVE		LRR2	2
1243	LVTLNQTLN LVVDKGTPKSLGKLQKQPAVG		LRR3	3

SHIFT -26!

1247	NQTLNLVVD KGTPKSLGKLQKQPAVGINSP		LRR1	2
1277	LYLGGIPTS TGLSALRQGTDRPLGGFHGCI		LRR2	2.2

HEVRINNELQDFKALPPQSLGVSPGCKSCTVCKHGLCRSVEKDSVVCECRPGWTGPLCDQ
EARDPCLGHRCHHGKCVATGTSYMCKCAEGYGGDLCDNKNDSANACSAFKCHHGQCHISD
QGEPYCLCQPGFSGEHCQQENPCLGQVVREVIRRQKGYASCATASKVPIMECRGGCGPQC
CQPTRSKRRKYVFQCTDGSSFVEEVERHLECGCLACS

>ENSP00000354459

MRLIRNIYIFCSIVMTAEGDAPEL

25	PEERELMTN CSNMSLRKVPADLTPA		LRR1	2
50	TTTLDLSYN LLFQLQSSDFHSVSK		LRR2	3
74	LRVLILCHN RIQQLDLKTFEFNKE		LRR3	4
98	LRYLDLSNN RLKSVTWYLLAG			LRR4	4
119	LRYLDLSFN DFDTMPICEEAGNMSH		LRR5	4
144	LEILGLSGA KIQKSDFQKIAHLH		LRR6	3
167	LNTVFLGFR TLPHYEEGSLPILNTTKL		LRR7	2.4
194	HIVLPMDTN FWVLLRDGIKTSKILEMTNID		LRR8	2.4

GKSQFVSYEM

234	QRNLSLENA KTSVLLLNKVDLL			LRR1	2
256	WDDLFLILQ FVWHTSVEHFQIRNVTF		LRR2	2.4
282	GGKAYLDHN SFDYSNTVMRTIKLEHVHFRV		LRR3	2.8

FYIQQDKIYL

322	LLTKMDIEN LTISNAQMPHMLFPNYPTK		LRR1	2
350	FQYLNFANN ILTDELFKRTIQLPH		LRR2	2.8
374	LKTLILNGN KLETLSLVSCFANNTP		LRR3	4
399	LEHLDLSQN LLQHKNDENCSWPET		LRR4	4
423	VVNMNLSYN KLSDSVFRCLPKS			LRR5	2.8
445	IQILDLNNN QIQTVPKETIHLMA		LRR6	3.4
468	LRELNIAFN FLTDLPGCSHFSR			LRR7	3.4
490	LSVLNIEMN FILSPSLDFVQSCQE		LRR8	3.4
514	VKTLNAGRN				LRR9	2.8
523	PFRCTCELKNFIQLETYSEVMMVGWSDSYTCEYPLNLRGTRLKDVHLHELSCN		CF1_short	3

SHIFT -17!

559	LRGTRLKDV HLHELSCNTALLIVTIV		LRR1	2
585	VIMLVLGLA VAFCCLHFDLPWY			LRR2	2.4
607	LRMLGQCTQ TWHRVRKTTQEQLKRNVRFHA		LRR3	2

SHIFT -14!

623	TTQEQLKRN VRFHAFISYSEHDSL		LRR1	2
647	WVKNELIPN LEKEDGSILICLYESYFDPGK		LRR2	2.4

SHIFT -13!

664	LICLYESYF DPGKSISENIVSFIEKSY		LRR1	2
691	KSIFVLSPN FVQNEWCHYEFYFAHHNLFHE		LRR2	2.4

NSD

724	HIILILLEP IPFYCIPTRYHK			LRR1	2
745	LKALLEKKA YLEWPKDRRKCGLF		LRR2	2
768	WANLRAAIN VNVLATREMYELQTFTELNEE		LRR3	2.8

SRGSTISLMRTDCL

>ENSP00000332536

MSWLRFPMAPPYVATIVFTSQGQPLVWVKDPLQLTSNPLGPPEPWSSRSSHLPWESPHAP
APPAAPGDFDYLGPSASSQMSALPQEPTENLAPFLKELDS

101	AGELPLGPE PFLAAHQDLNDKRTPEER		LRR1	2.4
128	LPEVVPLLN RDQNQALVQLPRLKWVQTTDL		LRR2	2.4

SHIFT -15!

143	LVQLPRLKW VQTTDLDRAAGHQADEI		LRR1	2
169	LVPLDSKVS RPTKFVVSPKNLKKDLAERWS		LRR2	2.4

LPEIVGIPHQLSKPQRQKQTLPDDY

224	LSMDTLYPG SLPPELRVNADEPPGPPEQVG		LRR1	2
254	LSQFHLEPK SQNPETLEDIQSSSLQEEAP		LRR2	2.4
283	AQLLQLPQE VEPSTQQEAPALPPESSMES		LRR3	2.4
312	LAQTPLNHE VTVQPPGEDQAHYNLPKFTVK		LRR4	2

PADVEVTMTSEPKNETESTQAQQEAPIQPPEEAEPSSTALRTTDPPPEHPEVTLPPSDKG
QAQHSHLTEATVQPLDLELSITTEPTTEVKPSPTTEETSAQPPDPGLAITPEPTTEIGHS
TALEKTRAPHPDQVQTLHRSLTEVTGPPTKLESSQDSLVQSETAPEEQKASTSTNICELC
TCGDET

518	CELCTCGDETLSCVGLSPKQRLRQVPVP		NF	4

SHIFT -18!

528	LSCVGLSPK QRLRQVPVPEPDTYNGI		LRR1	2.4
554	FTTLNFQGN YISYLDGNVWKAYSW		LRR2	2.8
578	TEKLILSEN YLTELPKDSFEGLLY		LRR3	3
602	LQYLDLSCN KIRYIERQTFESLPF		LRR4	4
626	LQYINLGCN LITKLSLGTFQAWHGMQF		LRR5	3.4
653	LHNLILNRN PLTTVEDPYLFELPA		LRR6	4
677	LKYLDMGTT HITLTTLKNILTMTVE		LRR7	2.8
702	LEKLILPSH				LRR8	3
711	MACCLCQFKNSIEAVCKTVKLHCNTACLTNSIH		CF3	3

SHIFT -42!

702	LEKLILPSH MACCLCQFKNSIEAV		LRR8	3
726	CKTVKLHCN TACLTNSIHCPEEASVGNPEG		LRR9	2.4

AFMKMLQARKQHMSTQLTIESEAPSDSSGINLSGFG

792	GDQLEIQLT EQLRSLIPNEDVRKFMSHV		LRR1	2.2
820	IRTLKMECS ETHVQGSCAKLM			LRR2	2.2
841	LRTGLLMKL LSEQQEAKALNVEWDTDQQKT		LRR3	2.4

NYINENMEQNEQKEQKSSELMKEVPGDDYKNKLIFAISVTVILIILIIIFCLIEVNSHKR
ASEKYKDNPSISGA

>ENSP00000345985

MET

4	LLGGLLAFG MAFAVVDACPKYCVCQNLSES		LRR1	2.4
34	LGTLCPSKG LLFVPPDIDRRTVELRLGGNF		LRR2	2

SHIFT -10!

54	TVELRLGGN FIIHISRQDFANMTG		LRR1	3
78	LVDLTLSRN TISHIQPFSFLDLES		LRR2	4
102	LRSLHLDSN RLPSLGEDTLRGLVN		LRR3	4
126	LQHLIVNNN QLGGIADEAFEDFLLT		LRR4	3.4
151	LEDLDLSYN NLHGLPWDSVRRMVN		LRR5	4
175	LHQLSLDHN LLDHIAEGTFADLQK		LRR6	4
199	LARLDLTSN RLQKLPPDPIFARSQASALTA		LRR7	4

SHIFT -20!

209	LQKLPPDPI FARSQASALTATPFAPPLSFS		LRR1	2
239	FGGNPLHCN CELLWLRRLERDDDLETCGSP		LRR2	2.4

GGLKGRYFWHVREEEFVCEPPLITQHT

296	HKLLVLEGQ AATLKCKAIGDPSPLIHWV		LRR1	2
324	APDDRLVGN SSRTAVYDNGTLDIFITTSQD		LRR2	2.4

SGAFTCIAANAAGEATAMVEVSIVQLPHLSNSTSRTAPPKSRLSDITGSSKTSRGGGGSG
GGEPPKSPPERAVLVSEVTTTSALVKWSVSKSAPRVKMYQLQYNCSDDEVLIYRMIPASN
KAFVVNNLVSGTGYDLCVLAMWDDTATTLTATNIVGCAQFFTKADYPQCQSMHSQILGGT
MILVIGGIIVATLLVFIVILMVRYKVCNHEAPSKMAAAVSNVYSQTNGAQPPPPSSAPAG
APPQGPPKVVVRNELLDFTASLARASDSSSSSSLGSGEAAGLGRAPWRIPPSAPRPKPSL
DRLMGAFASLDLKSQRKEELLDSRTPAGRGAGTSARGHHSDREPLLGPPAARARSLLPLP
LEGKAKRSHSFDMGDFAAAAAGGVVPGGYSPPRKVSNIWTKRSLSVNGMLLPFEESDLVG
ARGTFGSSEWVMESTV

>ENSP00000316119

MHPHRDPRGLWLLLPSLSLLLFEVARAGRAVVS

34	CPAACLCASNILSCSKQQLPNVPHSLPSY		NF	4
63	TALLDLSHN NLSRLRAEWTPTRLTQ		LRR1	3
88	LHSLLLSHN HLNFISSEAFSPVPN		LRR2	4
112	LRYLDLSSN QLRTLDEFLFSDLQV		LRR3	4
136	LEVLLLYNN HIMAVDRCAFDDMAQ		LRR4	4
160	LQKLYLSQN QISRFPLELVKEGAKLPK		LRR5	4
187	LTLLDLSSN KLKNLPLPDLQKLPAWI		LRR6	4
213	KNGLYLHNN				LRR7	3
222	PLNCDCELYQLFSHWQYRQLSSVMDFQEDLYCMNSKKLHNVFNLSFLNCGEY		CF1	4

SHIFT -45!

229	LYQLFSHWQ YRQLSSVMDFQEDLYCMN		LRR1	2
256	SKKLHNVFN LSFLNCGEYKER			LRR2	2
277	AWEAHLGDT LIIKCDTKQQGMTKVWVTPSN		LRR3	2.2
307	ERVLDEVTN GTVSVSKDGSLLFQQVQVEDG		LRR4	2

GVYTCYAMGETFNET

352	LSVELKVHN FTLHGHHDTLNTAYTT		LRR1	2
377	LVGCILSVV LVLIYLYLTPCRCWCRGVEKP		LRR2	2

SHIFT -21!

386	LVLIYLYLT PCRCWCRGVEKPSSHQGDS		LRR1	2.8
414	LSSSMLSTT PNHDPMAGGDKDDGFDRRVAF		LRR2	2.4

LEPAGPGQGQNGKLKPGNTLPVPEATGKGQRRMSDPESVSSVFSDTPIVV

>AY182026

MGLHFKWPLGAPMLAAIYAMSMVLKMLPALGMA

34	CPPKCRCEKLLFYCDSQGFHSVPNATD		NF	4
61	KGSLGLSLR HNHITELERDQFASFSQ		LRR1	2
87	LTWLHLDHN QISTVKEDAFQGLYK		LRR2	4
111	LKELILSSN KIFYLPNTTFTQLIN		LRR3	4
135	LQNLDLSFN QLSSLHPELFYGLRK		LRR4	4
159	LQTLHLRSN SLRTIPVRLFWDCRS		LRR5	4
183	LEFLDLSTN RLRSLARNGFAGLIK		LRR6	4
207	LRELHLEHN QLTKINFAHFLRLSS		LRR7	4
231	LHTLFLQWN KISNLTCGMEWTWGT		LRR8	4
255	LEKLDLTGN EIKAIDLTVFETMPN		LRR9	4
279	LKILLMDNN KLNSLDSKILNSLRS		LRR10	3.4
303	LTTVGLSGN LWECSARICALASWLGSFQGR		LRR11	3.4

WEHSILCHSPDHTQGEDILDAVHGFQLCWNLSTTVTVMATTYRDPTTEYTKRISSSSYHV
GDKEIPTTAGIAVTTEEHFPEPDNAIFTQRVITGTMALLFSFFFIIFIVFISRKCCPPTL
RRIRQCSMVQNHRQLRSQTRLHMSNMSDQGPYNEYEPTHEGPFIIINGYGQCKCQQLPYK
ECEV

>ENSP00000332668

M

2	PWPLLLLLA VSGAQTTRP		LRR1	2
20	CFPGCQCEVETFGLFDSFSLTRVDCSGLGPHIMPVPIP		NF	4

SHIFT -30!

28	VETFGLFDS FSLTRVDCSGLGPHIMPVPIP		LRR2	2.2
58	LDTAHLDLS SNRLEMVNESVLAGPGYTT		LRR3	2.8
86	LAGLDLSHN LLTSISPTAFSRLRY		LRR4	4
110	LESLDLSHN GLTALPAESFTSSP		LRR5	4
133	LSDVNLSHN QLREVSVSAFTTHSQGR		LRR6	3.4
159	ALHVDLSHN LIHRLVPHPTRAGLPAPT		LRR7	2.8
186	IQSLNLAWN RLHAVPNLRDLP			LRR8	3.4
207	LRYLSLDGN PLAVIGPGAFAGLGG		LRR9	4
231	LTHLSLASL QRLPELAPSGFRELPG		LRR10	3
256	LQVLDLSGN PKLNWAGAEVFSGLSS		LRR11	4
281	LQELDLSGT NLVPLPEALLLH			LRR12	3.4
302	LPALQSVSV GQDVRCRRLVREGTYPRRPGS		LRR13	2

SPKVALHCVDTRDSAARGPTIL

>ENSP00000304236

MERASCLLLLLLPLVHVSATTPEP

25	CELDDEDFRCVCNFSEPQPDWSEAFQCVSAVEVEIHAGGLNL		NF	4

SHIFT -6!

61	AGGLNLEPF LKRVDADADPRQYADTVKA		LRR1	2.4
89	LRVRRLTVG AAQVPAQLLVGALRVLAYSR		LRR2	2
118	LKELTLEDL KITGTMPPLPLEATGLA		LRR3	3
144	LSSLRLRNV SWATGRSWLAELQQWLKPG		LRR4	3
172	LKVLSIAQA HSPAFSCEQVRAFPA		LRR5	2.4
196	LTSLDLSDN PGLGERGLMAALCPHKFPA		LRR6	4
224	IQNLALRNT GMETPTGVCAALAAAGVQ		LRR7	2.8
251	PHSLDLSHN SLRATVNPSAPRCMWSSA		LRR8	3
278	LNSLNLSFA GLEQVPKGLPAK			LRR9	3
299	LRVLDLSCN RLNRAPQPDELPE			LRR10	4
321	VDNLTLDGN PFLVPGTALPHEGSMNSGVVP		LRR11	3.4

ACARSTLSVGVSGTLVLLQGARGFA

>ENSP00000303942

MPA

4	WGALFLLWA TAEATKD		LRR1	2.4
20	CPSPCTCRALETMGLWVDCRGHGLTALPALPAR		NF	4

SHIFT -24!

29	LETMGLWVD CRGHGLTALPALPAR		LRR2	2.4
53	TRHLLLANN SLQSVPPGAFDHLPQ		LRR3	3
77	LQTLDVTQN				LRR4	3.4
86	PWHCDCSLTYLRLWLEDRTPEALLQVRCASPSLAAHGPLGRLTGYQLGSC		CF1_short	3

SHIFT -41!

95	YLRLWLEDR TPEALLQVRCASPSLAAHGP		LRR1	2
124	LGRLTGYQL GSCGWQLQASWVRPGV		LRR2	2.4
149	LWDVALVAV AALGLALLAGLLCATTEALD		LRR3	2.4


>BC036337

MEAAR

6	ALRLLLVVC GCLALPPLAEPV		LRR1	2.8
27	CPERCDCQHPQHLLCTNRGLRVVPKTSSLP		NF	4

SHIFT -22!

35	HPQHLLCTN RGLRVVPKTSSLPSPHD		LRR2	2
61	VLTYSLGGN FITNITAFDFHRLGQ		LRR3	2.4
85	LRRLDLQYN QIRSLHPKTFEKLSR		LRR4	4
109	LEELYLGNN LLQALAPGTLAPLRK		LRR5	4
133	LRILYANGN EISRLSRGSFEGLES		LRR6	3.4
157	LVKLRLDGN ALGALPDAVFAPLGN		LRR7	4
181	LLYLHLESN RIRFLGKNAFAQLGK		LRR8	4
205	LRFLNLSAN ELQPSLRHAATFAPLRS		LRR9	4
231	LSSLILSAN SLQHLGPRIFQHLPR		LRR10	4
255	LGLLSLRGN QLTHLAPEAFWGLEA		LRR11	4
279	LRELRLEGN RLSQLPTALLEPLHS		LRR12	4
303	LEALDLSGN ELSALHPATFGHLGR		LRR13	4
327	LRELSLRNN ALSALSGDIFAASPA		LRR14	4
351	LYRLDLDGN				LRR15	4
360	GWTCDCRLRGLKRWMGDWHSQGRLLTVFVQCRHPPALRGKYLDYLDDQQLQNG		CF1_short	3

SHIFT -46!

367	LRGLKRWMG DWHSQGRLLTVFVQCRHPPA		LRR1	2
396	LRGKYLDYL DDQQLQNGSCADPSPSASLTA		LRR2	2

DRRRQPLPTAAGEEMTPPAGLAEELPPQPQLQQQGRFLAGVAWDGAARELVGN

479	RSALRLSRR GPGLQQPSPSVAAAAGPA		LRR1	2
506	PQSLDLHKK PQRGRPTRADPALAEPTPTAS		LRR2	2

PGSAPSPAGDPWQRATKHRLGTEHQERAAQSDGGAGLPPLVSDPCDFNKFILCNLTVEAV
GADSASVRWAVREHRSPRPLGGARFRLLFDRFGQQPKFHRFVYLPESSDSATLRELRGDT
PYLVCVEGVLGGRVCPVAPRDHCAG

681	LVTLPEAGS RGGVDYQLLTLALLTVNAL		LRR1	2.4
709	LVLLALAAW ASRWLRRKLRARRKGGAPVHV		LRR2	3

RHMYSTRRPLRSMGTGVSADFSGFQSHRPRTTVCALSEADLIEFPCDRFMDSAGGGAGGS
LRREDRLLQRFAD

>ENSP00000350933

MIT

4	LTELKCLAD AQSSYHILKPWWDVFWYYIT		LRR1	2
33	LIMLLVAVL AGALQLTQSRVLCC		LRR2	2.4
56	LPCKVEFDN HCAVPWDILKASMNTSSNP		LRR3	2
84	GTPLPLPLR IQNDLHRQQYSYIDAVCYEKQ		LRR4	2.4

LHWFAKFFPYLVLLHTLIFAACSNFWLHYPSTSSRLEHFVAILHKCFDSPWTTRALSETV
AEQSVRPLKLSKSKILLSSSGCSADIDSGKQSLPYPQPGLESAGIESPTSSVLDKKEGEQ
AKAIFEKVKRFRMHVEQKDIIYRVYLKQIIVKVILFVLIITYVPYFLTHITLEIDCSVDV
QAFTGYKRYQCVYSLAEIFKVLASFYVI

322	LVILYGLTS SYSLWWMLRSSLKQYS		LRR1	2.8
347	FEALREKSN YSDIPDVKNDFAFILHLADQY		LRR2	2.4

SHIFT -10!

367	AFILHLADQ YDPLYSKRFSIFLSEVSENK		LRR1	2.4
396	LKQINLNNE WTVEKLKSKLVKNAQDKIE		LRR2	2.4
424	LHLFMLNGL PDNVFELTEMEVLSLELIPEV		LRR3	2.4

SHIFT -12!

442	MEVLSLELI PEVKLPSAVSQLVN		LRR1	2.4
465	LKELRVYHS SLVVDHPALAFLEEN		LRR2	2.8
489	LKILRLKFT EMGKIPRWVFHLKN		LRR3	3.4
512	LKELYLSGC VLPEQLSTMQLEGFQDLKN		LRR4	3.4
540	LRTLYLKSS LSRIPQVVTDLLPS		LRR5	3.4
563	LQKLSLDNE GSKLVVLNNLKKMVN		LRR6	3
587	LKSLELISC DLERIPHSIFSLNN		LRR7	3.4
610	LHELDLREN NLKTVEEIISFQHLQN		LRR8	4
635	LSCLKLWHN NIAYIPAQIGALSN		LRR9	4
658	LEQLSLDHN NIENLPLQLFLCTK		LRR10	4
681	LHYLDLSYN HLTFIPEEIQYLSN		LRR11	4
704	LQYFAVTNN NIEMLPDGLFQCKK		LRR12	2.8
727	LQCLLLGKN SLMNLSPHVGELSN		LRR13	4
750	LTHLELIGN YLETLPPELEGCQSLK		LRR14	4
775	RNCLIVEEN LLNTLPLPVTERLQTCLDKC		LRR15	2.4


>ENSP00000304477

MIPVTEFRQFSEQQPAFRVLKPWWDVFTDYLSVAMLMIGVFGCTLQVMQDKIICLPKRVQ
PAQNHSSLSNVSQAVASTTPLPPPKPSPANPITVEMKGL

100	KTDLDLQQY SFINQMCYERALHWYAKY		LRR1	2
127	FPYLVLIHT LVFMLCSNFWFKFPGSSSKIE		LRR2	2.8

HFISILGKCFDSPWTTRALSEVSGEDSEEKDNRKNNMNRSNTIQSGPEDSLVNSQSLKSI
PEKFVVDKSTAGALDKKEGEQAKALFEKVKKFRLHVEEGDILYAMYVRQTVLKVIKFLII
IAYNSALVSKVQFTVDCNVD

297	IQDMTGYKN FSCNHTMAHLFSK			LRR1	2.2
319	LSFCYLCFV SIYGLTCLYTLYW			LRR2	2
341	LFYRSLREY SFEYVRQETGIDDIPDVKNDF		LRR3	2

AFMLHMIDQYDPLYSKRFAVFLSEVSE

398	NKLKQLNLN NEWTPDKLRQKLQTNAHNRLE		LRR1	2
428	LPLIMLSGL PDTVFEITELQSLKLEIIKNV		LRR2	2.4

SHIFT -12!

446	LQSLKLEII KNVMIPATIAQLDN		LRR1	3
469	LQELSLHQC SVKIHSAALSFLKEN		LRR2	3.4
493	LKVLSVKFD DMRELPPWMYGLRN		LRR3	2.4
516	LEELYLVGS LSHDISRNVTLESLRDLKS		LRR4	3.4
544	LKILSIKSN VSKIPQAVVDVSSHLQKMCIH		LRR5	3.4

NDGTK

579	LVMLNNLKK MTNLTELELVHCDLERIPHAV		LRR1	2
609	FSLLSLQEL DLKENNLKSIEEIVSFQHLRK		LRR2	2.4
639	LTVLKLWHN SITYIPEHIKKLTS		LRR3	4
662	LERLSFSHN KIEVLPSHLFLCNK		LRR4	3.4
685	IRYLDLSYN DIRFIPPEIGVLQS		LRR5	3.4
708	LQYFSITCN KVESLPDELYFCKK		LRR6	2.8
731	LKTLKIGKN SLSVLSPKIGNLLF		LRR7	3.4
754	LSYLDVKGN HFEILPPELGDCRA		LRR8	3.4
777	LKRAGLVVE DALFETLPSDVREQMKTE		LRR9	2.4


>XP_143529

MTAQDQGEAPASSCDSA

18	LKCLPEEWT LPSFWCESTRRL			LRR1	2.4
39	VLCNDLDMN EVPANFPVDTSKLRIEKTVVR		LRR2	2.4

RLPAEAFYY

78	LVELQYLWL AYNSVASIETSSFYNLRQ		LRR1	2
105	LHELRLDGN SLTAFPWVSLLDMPH		LRR2	4
129	LRTLDLHNN RIASVPNEAVRYLRN		LRR3	4
153	LTCLDLSSN RLTTLPPDFLDS			LRR4	4
174	WSHLAVTPS RSPDFPPRRIILGLQDNPWFC		LRR5	2.2

DCHISKVIELSKVTDHAVVLLDPLMVCSEPERFQGIL

241	FQRVELEKC LKPSVMMSATKITSA		LRR1	2.2
265	LGSNVLLRC DAKGHPTPQLTWTRSDGSTVN		LRR2	2.4

YTVIQESPGEGIRWSIISLTSISHKDAGDYRCKAKNLAGISEAVVTVTVVGGVTTTLSPD
SSERSPGEPPEQHPQPGLGGSTPPSKSWLSPGLTSAPSYPTPSAALYTSTWSPPPSSLPP
IFSAASATTSVQTSISGRTARTSHQPP

442	LLHPGGKSN AKIEKNGRKFPPLSASK		LRR1	2.4
468	KEELALLDQ AAPMETNVTIKDLRVARET		LRR2	2
496	GVSVTLMWN SSSSTQESSVTVLYSKY		LRR3	2.8
522	GEKDLLLVN ADDYGKNQATINGLEPGSQYV		LRR4	2.4

ACVCPKGVGPREDLCITFSTNRVEGRGSQWSLLLVVTSTACVIVVPLICFLLYKVCKLQC
TSDPFWEEDLSKETYIQFETLSPRSQSIGELWTRRHRDDGERLLLCSQSSVDSQMNLKSD
GCRTEYYG

>ENSMUSP00000065706

MGFLSPIYVLFFCFGVRVYCQYEAYRWDDDYDQEPNEDYDPEFQFHQNIEYGVPFYNNIL
G

62	CAKECFCPTNFPTSMYCDNRKLKTIPIIPMH		NF	4
93	IQQLNLQFN DIEAVTANSFINATH		LRR1	3.4
117	LKEINLSHN KIKSQKIDYGVFAKLSN		LRR2	3.4
143	LQQLHLEHN NLEEFPFPLPKS			LRR3	4
164	LERLLLGYN EISILPTNAMDGLVN		LRR4	4
188	VTMLDLCYN HLSDSMLKEKTLSKMEK		LRR5	3.4
214	LMQLNLCNN RLESMPLGLPSS			LRR6	4
235	LMYLSLENN SISSIPDNYFDKLPK		LRR7	4
259	LHALRISHN KLEDIPYDIFNLSN		LRR8	3.4
282	LIELNVGHN KLKQAFYIPRNLEHLYLQNNE		LRR9	3.4

SHIFT -10!

302	LEHLYLQNN EIESINVTMICPSPDPVHHHH		LRR1	4
332	LTYLRVDQN KLKEPISSYIFFCFPRIHSIY		LRR2	3.4

YGEQRSTNGETIQLKTQVFRSYQEEEEEDDHDSQDNTLEGQEVSDEHYNSHYYEMQEWQD
TI

>XP_892248

MQKRGREVSCLLISLTAICLVVTPGSRV

29	CPRRCACYVPTEVHCTFRYLTSIPDGIPAN		NF	4
59	VERVNLGYN SLTRLTENDFSGLSR		LRR1	2.8
83	LELLMLHSN GIHRVSDKTFSGLQS		LRR2	4
107	LQVLKMSYN KVQIIEKDTLYGLRS		LRR3	3.4
131	LTRLHLDHN NIEFINPEAFYGLTL		LRR4	4
155	LRLVHLEGN RLTKLHPDTFVS			LRR5	3.4
176	LSYLQIFKT SFIKYLYLSDNF			LRR6	2.8
197	LTSLPKEMV SSMPNLESLYLHGNPWTCDCH		LRR7	2
227	LKWLSEWMQ GNPDIIKCKKERIPSS		LRR8	2
252	PQQCPLCMN PRISKGRSIAMVPSGSFLCTK		LRR9	2

PTIDPS

288	LKSKSLGIQ EDNGSASVSPQDFIEPFGSLS		LRR1	2
318	LNMTDLSGN KANVICSIQKPSRT		LRR2	3
341	LPIAFTEEN DYIMLNMSFSTNLVCSVNYNH		LRR3	2.4

SHIFT -18!

353	MLNMSFSTN LVCSVNYNHIQPV			LRR1	2.2
375	WQLLALYSD SPLILERKPQHTETPLLSPKY		LRR2	2.4

SHIFT -22!

383	DSPLILERK PQHTETPLLSPKYQQVA		LRR1	2
409	LRPEDTFTN IEADFKADPFWFQ			LRR2	2
431	QEKISLQLN RTATTLSTLQIQFSTDAQITL		LRR3	2.4

SHIFT -16!

445	LSTLQIQFS TDAQITLPKAEMRPVKR		LRR1	2.8
471	KWTMILMMN NTRLEHTVLVGGTIALDCPGK		LRR2	2.4

SHIFT -18!

483	LEHTVLVGG TIALDCPGKGDPSPH		LRR1	2
507	LEWVLADGS KVRAPYVSEDGRILIDK		LRR2	2.2
533	KGKLELQMA DTFDAGLYHCISTNDADADIL		LRR3	2

TYRITVVEPYVENKHENGALHTVIMGEILDLPCLSTGIPDA

604	SISWILPRN TVFSQSSRDMQI			LRR1	2.4
625	LNNGTLRIL QATPKDQGHYRCVAANPSGAD		LRR2	2.4

FSSFQVSVQMKGQRTIEHDRDIDGSGLEEPKPSVLLKQPPSLKLPASSLTGTEAGKQVSG
IHKKNKHRDLTHRRRGDSTLRRFREH

741	RRQLPLSAR RIDPQHWAALLEKAKK		LRR1	2
766	NSVLRKQEN TTVKPTPLAIPLVELAGEEKD		LRR2	2

ASGLTPPDEEFTVLKTKAFGVPERSPTADSRPVNHGFVTSSASGTEVSSTVNPQTLLPTH
LPDFKLFNVVDSAAVSKSMNRPVTSKIEDTTHQNPIIIFPSVAEIQDSAQVGRTSSQSAH
PATGGAMATYGYTTMLSSFTNKANTVLQSANPTESYGPQIPLTEVSRVSSNNSLAHTTKD
PGFSKRPSDSHTTAPSLFQTPRNNSTGNVGRERTIWSRGRAISPYRTPVLRRHRHRIVRP

1036	ALKGPANRN ISQVSATEPPGMCRTCSSTER		LRR1	2.2
1066	LTMATAALS VTGSSHTTLPKANNVGIISEE		LRR2	2.2

SHIFT -16!

1080	HTTLPKANN VGIISEESTTVV			LRR1	2
1101	KKPSLLLKN KQDVDIETITTTINYFRSEST		LRR2	2

HMTPTEASMISAPTSISLGKTPIDTSGHLSMPRTIQAGTDLVVTPPLSSPLSQPSIPTKA
TSTKLSRRKIPWHPIFANNHNKEGMLKNLHQFGLQKNTATKPPEKAPLLPTDHGSSSPST
TLLASLTPAQSATMAATRRNGTEVQGARSLSAGKEQPFINSFLVLPSTTRKRSSTLSFLS
VETPTVTTPPVIASAIISETQEVRSKKAKDQTKGSLKNRKGPTITPRQISGYSTYSVPTT
TDTPLAFSHSPGKVTGRTVSTAAPHSAASLLGITELPQKCTHTSGNITASETTLLSKSQE
STAMKRASATPPLLSSGAPRMPTPSPPPFTKVVVTDSEVPAVFKMMSNRMVTIYESSRHD
IDLQQPSAEASPNPEILTGSTDFPLSSLLTSTPMPAPRVDKPQDSQWKPSPWPENKFQLR
SYSETIEKGKRPEISLSPHLSFPEASTHALHWNAQRHAEKSVFDKKPAQNPTSKHLPYDS
LPKTILKKPRIIGGKAASFTVPTNSDVLLPCEAVGDPKPTIHWTRVSSGREISRGIQKTR
FHV

1674	LPNGTLSIQ RVSIQDRGQYLCAASNP		LRR1	2.4
1700	LGVDHLHVT LSVVSYPARILESHVKEITAH		LRR2	2.4

SGSTVKLKCRVEGMPRPTISWILANQTVVSETPEGSRKVWVTPDGTLIIHNLSLYDRGFY
KCVANNPSGQDSLLVKIQVITAPPVIIEQKRQAIVGVL

1828	GESLKLPCT AKGTPQPSVHWVLYDGTE		LRR1	2.8
1855	LKPLQLTHS RFFLYPNGTLYIRNIVSSVRG		LRR2	3.4

TYECIATSSSGSERRVVILRVEEQETVPRIETASQKWTEVNLGEKLLLNCSATGDPKPTI
IWKLPSKVVIDQWHRM

1961	GSRIHVYPN GSLVIGSVTEKDG			LRR1	2.2
1983	GDYLCVARN KMGDDLVLMHVRLRLTPAKIE		LRR2	2.8

HKQHFKKQVLHGKDFQVDCKASGSPVPEVSWS

2045	LPDGTVVNN VAQADDSGYRTKRYTL		LRR1	2.8
2070	FHNGTLYFN KVGMAEEGDYICSAQNTLGKD		LRR2	2.8

EMKVHLTVLTAIPRIRQNYRSNVRIKAGDTAVLDCEVTGEPKP

2143	NVFWLLPSN NVISFSNDRFIF			LRR1	2.4
2164	HANGTLSIN KVKPLDSGKYVCVAQNPSGDD		LRR2	2.4

SHIFT -14!

2180	GKYVCVAQN PSGDDTKTYKLDIVSRPPL		LRR1	2.2
2208	INGLYANKT VIKATAIQHSKKHLDCRADGV		LRR2	2.2

PPPQITWIMPDNIFLTAPYYGGRITVHQNGTLE

2271	IRNIRLSDS ADFTCVVRSEGGESVLVVQ		LRR1	2.2
2299	LKVLEMLRR PTFRNPFNEKVVAQVGKPVAM		LRR2	2.4

NCSVDGNPTPEIIWI

2344	LPDGTQFAN GPQNSPYLMASNGS		LRR1	2.4
2367	LIVYKATRN KSGKYRCTARNKVGY		LRR2	2.4
2391	IEKLILLEI GQKPVILTYEPGMIKSAG		LRR3	2.4
2418	GESLSLHCV SDGIPKPNVKWTTPGGLVIDR		LRR4	2.4

PQVGGKYILHENGTLVIKETTAHDRGNYICKAQNSVGQAVISVPVTIVAYPPRIINYLPR
SMLRRTGEAMQLHCVALGVPKPQITWETPGYSLLSTATERRPHRSEMLPLQGTLVIQNLR
ASDSGVYKCRAQNVLGADYATTYIQVL

>ENSMUSP00000060210

MLRLGL

7	CAAALLCVCQPGAVRADCWLIEGDKGYVWLAIC		NF	4

SQNQPPYETIPQHIN

55	STVHDLRLN ENKLKAVLYSSLNRFGN		LRR1	2
81	LTDLNLTKN EISYIEDGAFLGQTS		LRR2	4
105	LQVLQLGYN RLSNLTEGMLRGMSR		LRR3	4
129	LQFLFVQHN LIEVVTPTAFSECPS		LRR4	3.4
153	LISIDLSSN RLSRLDGATFASLAS		LRR5	3.4
177	LMVCELAGN				LRR6	3
186	PFNCECDLFGFLAWLVVFNNVTKNYDRLQCESPREFAGYPLLVPRPYHSLNA		CF1_short	3

ITVLQAKCRNGSMPARPVSHPTPYSTDAQREPDENSGFNPDEILSVEPPASSTTDASAGP
AIKLHQVTFTSATLVVIIPHPYSK

322	MYVLVQYNN SYFSDVMTLKNKKEIVT		LRR1	2.4
348	LDKLRAHTE YTFCVTSLRNSRRFNHTCLTF		LRR2	2.4

TTRDLVPGDLAPSTSTTTHYIMTILGCLFGMVIVLGAVYYCLRKRRMQEEKQKSVNVKKT
ILEMRYGADVDAGSIVHAAQKLGEPPVLPVARMSSIPSMVGEKLPASKGLEAGLDTPKVA
TKGNYIEVRTGAAGDSLARPEEELPEIENGQGSAAEISTIAKEVDKVNQIINNCIDALKL
DSASFLGGGGGGGGGGDSDLAFECQSLPAAPAASSAATPGALERPSFLSPPYKESSHHPL
QRQLSADAAVSRKTCSVSSSGSIKSAKVFSLDVPDHPTPTGLAKSDSKYIEKGSPLNSPL
DRLPLVPTGSSGSSGGGGGIHHLEVKPAYHCSEHRHSFPALYYEEGADSLSQRVSFLKPL
TRSKRDSTYSQLSPRHYYSGYSSSPEYSSESTHKIWERFRPYKKHHREEVYMAAGHALRK
KVQFAKDEDLHDILDYWKGVSAQQKL

>ENSMUSP00000057529

MACQLDL

8	LIGVIFMAS PVLVISPCSSDGRIAFFRGCN		LRR1	2.2
38	LTQIPWILN TTTERLLLSFNYISMVVATSF		LRR2	2.8

SHIFT -19!

49	TERLLLSFN YISMVVATSFPL			LRR1	3
70	LERLQLLEL GTQYANLTIGPGAFRNLPN		LRR2	3
98	LRILDLGQS QIEVLNRDAFQGLPH		LRR3	3.4
122	LLELRLFSC GLSSAVLSDGYFRNLYS		LRR4	3.4
148	LARLDLSGN QIHSLRLHSSFRELNS		LRR5	4
173	LSDVNFAFN QIFTICEDELEPLQGKT		LRR6	2.8
199	LSFFGLKLT KLFSRVSVGWETCRNPFRGVR		LRR7	2.8
229	LETLDLSEN GWTVDITRNFSNIIQGSQ		LRR8	4
256	ISSLILKHH IMGPGFGFQNIRDPDQSTFAS		LRR9	2.4

SHIFT -20!

266	MGPGFGFQN IRDPDQSTFASLAR		LRR1	2.2
289	SSVLQLDLS HGFIFSLNPRLFGTLKD		LRR2	2.4
315	LKMLNLAFN KINKIGENAFYGLDS		LRR3	4
339	LQVLNLSYN LLGELYNSNFYGLPR		LRR4	4
363	VAYVDLQRN HIGIIQDQTFRLLKT		LRR5	2.8
387	LQTLDLRDN ALKAIGFIPSIQMVL		LRR6	4
411	LGGNKLVHL PHIHFTANFLELSENRLEN		LRR7	2
439	LSDLYFLLR VPQLQFLILNQNRLSSCKAAH		LRR8	2.4

SHIFT -23!

446	LRVPQLQFL ILNQNRLSSCKAAHTPSENPS		LRR1	2
476	LEQLFLTEN MLQLAWETGLCWDVFQGLSR		LRR2	4
505	LQILYLSNN YLNFLPPGIFNDLVA		LRR3	4
529	LRMLSLSAN KLTVLSPGSLPAN			LRR4	4
551	LEILDISRN QLFSPDPALFSS			LRR5	3.4
572	LRVLDITHN				LRR6	3.4
581	EFVCNCELSTFISWLNQTNVTLFGSPADVYCMYPNSLLGGSLYNISTEDCDEE		CF1	4

SHIFT -46!

588	LSTFISWLN QTNVTLFGSPADVYCMYPNS		LRR1	2.4
617	LLGGSLYNI STEDCDEEEAMRSLKFSLFI		LRR2	2.4
646	LCTVTLTLF LVITLVVIKFRGICFLCYKTI		LRR3	2.4

QKLVFKDKVWSLEPGAYRYDAYFCFSSKDFEWAQNALLKHLDAHYSSRNRLRLCFEERDF
IPGENHISNIQAAVWGSRKTVCLVSRHFLKDGWCLEAFRYAQSRSLSDLKSILIVVVVGS
LSQYQLMRHETIRGFLQKQQYLRWPEDLQDVGWFLDKLSGCILKEEKGKKRSSSIQLRTI
ATIS

>ENSMUSP00000057071

MLCSLFLLLLAVGRVQTTRP

21	CFPGCQCEEETFGLFDSFSLIRVDCSSLGPHIVPVPIP		NF	4
59	LDTAHLDLS SNRLETVNESVLAGPGYTT		LRR1	2.8
87	LAGLDLSYN LLTSIMPSAFSRLRY		LRR2	4
111	LESLDLSHN GLAALPAEIFTSSP		LRR3	4
134	LSDINLSHN RLREVSISAFTTHSQGR		LRR4	3.4
160	ALHVDLSHN LIHRLLPHPARASLPAPT		LRR5	2.8
187	IQSLNLSWN RFRAVPDLRDLP			LRR6	3.4
208	LRYLSLDGN PLATINPDAFMGLAG		LRR7	4
232	LTHLSLASL QGILHLPPHGFRELPG		LRR8	3
257	LQVLDLSGN PKLKWAGAEVFSGLGL		LRR9	4
282	LQELDLSGS SLVPLPEMLLHH			LRR10	3.4
303	LPALQSVSV GQDVQCRRLVREGAYHRQPGS		LRR11	2

SPKVVLHCGDTQESAARGPDIL

>ENSMUSP00000048962

MAG

4	SRGLPLLLL VLQLFLGPVLPVRAPVFGRSD		LRR1	2
34	TPTLSPEEN EFVEEENQPVLVLSSEEPEPG		LRR2	2

PATVD

69	CPRDCACSQEGVVDCGGIDLREFPGDLPEH		NF	4

SHIFT -7!

92	PGDLPEHTN HLSLQNNQLEKIYPEE		LRR1	2
117	LSRLQRLET LNLQNNRLTSRGLPEEAFEH		LRR2	2.4
146	LTSLNYLYL ANNKLTLAPRFLPNA		LRR3	2
170	LISVDFAAN YLTKIYGLTFGQKPN		LRR4	2.8
194	LRSVYLHNN KLADAGLPDHMFNGSSN		LRR5	3.4
220	VEILILSSN FLRHVPKHLPPA			LRR6	3.4
241	LYKLHLKNN KLEKIPPGAFSELSN		LRR7	4
265	LRELYLQNN YLTDEGLDNETFWKLSS		LRR8	4
291	LEYLDLSSN NLSRVPAGLPRS			LRR9	4
312	LVLLHLEKN AIQSVEADVLTPIRN		LRR10	4
336	LEYLLLHSN QLQAKGIHPLAFQGLKK		LRR11	4
362	LHTVHLYNN ALERVPSGLPRR			LRR12	3.4
383	VRTLMILHN QITGIGREDFATTYF		LRR13	2.8
407	LEELNLSYN RITSPQMHRDAFRKLRL		LRR14	4
433	LRSLDLSGN RLQTLPPGLPKN			LRR15	4
454	VHVLKVKRN ELAALARGALAGMAQ		LRR16	2.8
478	LRELYLTGN RLRSRALGPRAWVDLAG		LRR17	4
504	LQLLDIAGN QLTEVPEGLPPS			LRR18	3.4
525	LEYLYLQNN KISAVPANAFDSTPN		LRR19	4
549	LKGIFLRFN KLAVGSVVESAFRRLKH		LRR20	3.4
575	LQVLDIEGN FEFGNGSKDKDEEEEEEEEEE		LRR21	3.4

DEEEETR

>ENSMUSP00000088561

MMKPSIAEMLHRGRMLWIILLSTIALGWTTPIPLIEDSEEIDEP

45	CFDPCYCEVKESLFHIHCDSKGFTNISQITEFW		NF	4

SR

80	PFKLYLQRN SMRRLYTNSFLHLNN		LRR1	3
104	AVSINLGNN ALQDIQTGAFNGLKI		LRR2	2.8
128	LKRLYLHEN KLDVFRNDTFLGLES		LRR3	4
152	LEYLQADYN VIKRIESGAFRNLSK		LRR4	3.4
176	LRVLILNDN LIPVLPTNLFKAVS		LRR5	4
199	LTHLDLRGN RLKVLFYRGMLDHIGRS		LRR6	4
225	LMELQLEEN				LRR7	4
234	PWNCTCEIVQLKSWLERIPYTALVGDITCETPFHFHGKDLREIKKTELCPL		CF1_short	3

LSDSEVEAS

294	LGIPHLSSS KENAWPTKPSSM			LRR1	2.4
315	LSSVHFTAS SVEYKSSNKQPKPTKQPRTPR		LRR2	2.2

PPST

349	SQALYPGPN QPPIAPYQTRPPIPIICPT		LRR1	2
377	GCTCNLHIN DLGLTVNCKERGFNNI		LRR2	2.4
402	SELLPRPLN AKKLYLSSNLIQKIYRSDFWN		LRR3	2
432	FSSLDLLHL GNNRISYVQDGAFINLPN		LRR4	2.4
459	LKSLFLNGN DIEKLTPGMFRGLQS		LRR5	4
483	LHYLYFEFN VIREIQPAAFSLMPN		LRR6	3.4
507	LKLLFLNNN LLRTLPTDAFAGTS		LRR7	4
530	LARLNLRKN YFLYLPVAGVLEHLNA		LRR8	4
555	IVQIDLNEN				LRR9	2.8
564	PWDCTCDLVPFKQWIETISSVSVVGDVLCRTPENLTHRDVRTIELEVLCPE		CF1_short	3

MLHIAQPGPSPPQPGDYHPNGGPTSASPYEFSPPGGPVP

654	LSVLILSLL VLFFSAVFVAAG			LRR1	3
675	LFAYVLRRR RKKLPFRSKRQEGVDLTGIQM		LRR2	2

QCHRLFEDSGGNSGGSGGGGRPTLSSPEKAPPVGHVYEYIPHPVTQMCNNPIYKPREEEE
VAASAAQDTGATDRGGPGTQPTGMAEVLLGSEQFAETPKENHSNYRTLLEKEKEWALAVS
NSQLNTIVTVNHHHPHPHHSAVGGVSGVGGGTGGDLAGFRHHEKNGGVVLFPPGGGCG

883	GGSLLLDRE RPQPAPCTVGFVDCLYGTVPK		LRR1	2.4
913	LKELHVHPP GMQYPDLQQDAR			LRR2	2.4
934	LKETLLFSA GKGFTDHQTPKSDYLDLRAKL		LRR3	2

QTKPDYLEVLEKTAYRF

>ENSMUSP00000060402

MKLAVLFC

9	FILLIVLQT DCERGTRRQRRRMHQRRLRK		LRR1	2.2
38	SSSFHLRAN RQLEVQQTTAAPDARLPTANS		LRR2	2.4

DYSVE

73	ENIESLLSN LGVESSYSVLPGKKGYC		LRR1	2
99	FVKGMIMYN KAVWSPEPCTTCLCSNGRVLC		LRR2	2.2

DETECHPKACPYTIKPEGECCPICSDAEQESINKLHKQVPPPQMEMDQVAIKEALQSEED
EEIAEGHKEHKKETSVPTKIHGDGERTERKLRPEKEGRSAHQPLYHGRREEEESKEETER
EGEEEEEEEEEEEEDAIRGDVFRMSSRVIPGTPRGRPRLPRSCSLSYRTISCVHADFTEI
PPITAPE

316	VTNLELVGN SIISIPDEAFNGLPN		LRR1	3.4
340	LERLDLSRN NITSSGIGPKAFKSLKK		LRR2	4
366	LMRLNMDGN NLVHIPSDLPST			LRR3	3.4
387	LEELKINDN NLQAIDEKSLSDLNQ		LRR4	3.4
411	LVTLELEGN NLSEINVDPLAFQSLES		LRR5	4
437	LSYLRLGRN KFRIIPQGLPAS			LRR6	4
458	TEELYLENN QIEEITEICFNHTRK		LRR7	3
482	ITMIILRYN KIEESRIAPLAWINQEN		LRR8	2.8
508	LESIDLSYN KLYHVPSYLPKS			LRR9	3.4
529	LLHLVLIGN QIDRIPGYVFGHMQPG		LRR10	4
554	LEYLYLSFN RLSDDGVDLVSFYGAYHS		LRR11	4
581	LRELFLDHN DFKSIPPGIQDMKA		LRR12	4
604	LHFLRLNNN				LRR13	4
613	KIRNIHPEQICNAEEDEDSALEHLHLENNYIRTREISSYAFSCI		CF2	2

SHIFT -53!

604	LHFLRLNNN KIRNIHPEQICNAEEDEDSA		LRR13	4
633	LEHLHLENN YIRTREISSYAFSCIRLYSSI		LRR14	4

VLKPQHIK

>ENSMUSP00000066015

MIPVTEFRQFSEQQPAFRVLKPWWDVFTDYLSVAMLMIGVFGCTLQVMQDKIICLPKRVQ
PAQNHSSVPNVSQAVISTTPLPPPKPSPTNPATVEMKGL

100	KTDLDLQQY SFINQMCYERALHWYAKY		LRR1	2
127	FPYLVLIHT LVFMLCSNFWFKFPGSSSKIE		LRR2	2.8

HFISILGKCFDSPWTTRA

175	LSEVSGEDS EEKDNRKNNMNRSGTIQS		LRR1	2.2
202	GPEGNLVRS QSLKSIPEKFVVDKSAAGALD		LRR2	2.2

KKEGEQAKALFEKVKKFRLHVEEGDILYAMYVRQTVLKVIKFLIIIAYNSALVSKVQFTV
DCNVD

297	IQDMTGYKN FSCNHTMAHLFSK			LRR1	2.2
319	LSFCYLCFV SIYGLTCLYTLYW			LRR2	2
341	LFYRSLREY SFEYVRQETGIDDIPDVKNDF		LRR3	2

AFMLHMIDQYDPLYSKRFAVFLSEVSE

398	NKLKQLNLN NEWTPDKLRQKLQTNAHNRLE		LRR1	2
428	LPLIMLSGL PDTVFEITELQSLKLEIIKNV		LRR2	2.4

SHIFT -12!

446	LQSLKLEII KNVMIPATIAQLDN		LRR1	3
469	LQELCLHQC SVKIHSAALSFLKEN		LRR2	3.4
493	LKVLSVKFD DMRELPPWMYGLRN		LRR3	2.4
516	LEELYLVGS LSHDISKNVTLESLRDLKS		LRR4	3.4
544	LKILSIKSN VSKIPQAVVDVSSHLQKMCVH		LRR5	3.4

NDGTK

579	LVMLNNLKK MTNLTELELVHCDLERIPHAV		LRR1	2
609	FSLLSLQEL DLKENNLKSIEEIVSFQHLRK		LRR2	2.4
639	LTVLKLWYN SIAYIPEHIKKLTS		LRR3	4
662	LERLFFSHN KVEVLPSHLFLCNK		LRR4	3.4
685	IRYLDLSYN DIRFIPPEIGVLQS		LRR5	3.4
708	LQYFSITCN KVESLPDELYFCKK		LRR6	2.8
731	LKTLKIGKN SLSVLSPKIGNLLF		LRR7	3.4
754	LSYLDIKGN HFEVLPPELGDCRA		LRR8	3.4
777	LKRAGLVVE DALFETLPSDVREQMKAD		LRR9	2.4


>ENSMUSP00000055604

MGPFGALCLAWALLGVVRA

20	CPEPCACVDKYAHQFADCAYKELREVPEGLPAN		NF	4
53	VTTLSLSAN KITVLRRGAFVNVTQ		LRR1	3.4
77	VTSLWLAHS EVRTVESGALAVLSQ		LRR2	2.8
101	LKNLDLSHN LISNFPWSDLRNLSA		LRR3	4
125	LQLLKMNHN RLGSLPRDALGALPD		LRR4	3.4
149	LRSLRINNN RLRTLEPGTFDALSA		LRR5	3.4
173	LSHLQLYHN				LRR6	4
182	PFHCSCGLVWLQAWAASTRVSLPEPDSIACASPPELQGVPVHRLPALPCAPP		CF1	4

SVRLSAEPPPEAPGTPLRAGLAFMLHCVAEGHPTPRLQWQLQIPGGTVVLVPPVLSKEED
GGDKVEDGEGDGDEDLPTQTEAPTPTPAPAWPAPPAT

331	PRFLALANG SLLVPLLSAKEAGIYTC		LRR1	2
357	RAHNELGTN STSLRVTVAAAGPPKHAPGTG		LRR2	2

EEPDAQVPTSERKATTKGRSNSVLPFKPEGKTKGQGLARVSVLGEIEAELEETDEGEQME
GQIPADPMGEKHCGHGDPSRYVSNHAFNQSSDLKPHVFELGVIALDVAEREARVQ

502	LTPLAARWG PGPDGASGARRPGRRP		LRR1	2.4
527	LRLLYLCPA GGGTAVQWSRVEEGVNAYW		LRR2	3
555	FRGLRPGTN YSVCLALAGEACHVQVVFSTK		LRR3	2.4

SHIFT -20!

565	SVCLALAGE ACHVQVVFSTKKE			LRR1	2
587	LPSLLVIVT VSVFLLVLATVPLLGAACCHL		LRR2	2.8

SHIFT -19!

598	VFLLVLATV PLLGAACCHLLAKHPGK		LRR1	2.4
624	PYRLILRPQ APDPMEKRIAADFDPRASYLE		LRR2	2

SEKSYPARGEAGGEEPEEVPEEGLDEDVEQGDPSGDLQREES

696	LAGCSLVES QSKANQEEFEAGSEYSDR		LRR1	2.4
723	LPLGAEAVN IAQEINGNYRQTAG		LRR2	2.4


>ENSMUSP00000043818

MKDTPLQVHVLLGLAITTLVQAIDKKVD

29	CPQLCTCEIRPWFTPRSIYMEASTVDCNDLGLLNFPARLPAD		NF	4
71	TQILLLQTN NIARIEHSTDFPVN		LRR1	3
94	LTGLDLSQN NLSSVTNINVQKMSQ		LRR2	4
118	LLSVYLEEN KLTELPEKCLYGLSN		LRR3	3.4
142	LQELYVNHN LLSTISPGAFIGLHN		LRR4	3.4
166	LLRLHLNSN RLQMINSQWFDALPN		LRR5	4
190	LEILMLGDN PIIRIKDMNFQPLVK		LRR6	4
214	LRSLVIAGI NLTEIPDDALAGLEN		LRR7	2.4
238	LESISFYDN RLSKVPQVALQKAVN		LRR8	2.8
262	LKFLDLNKN PINRIRRGDFSNM			LRR9	4
284	LHLKELGIN NMPELVSIDSLAVDNLPD		LRR10	3
311	LRKIEATNN PRLSYIHPNAFFRLPK		LRR11	2.8
336	LESLMLNTN ALSALYHGTIESLPN		LRR12	4
360	LKEISIHSN				LRR13	2.8
369	PIRCDCVIRWINMNKTNIRFMEPDSLFCVDPPEFQGQNVRQVHFRDMMEI		CF1_short	3

CLPLIAPESFPSDLDVEADSYVSLHCRATAEPQPEIYWITPSGKKLLPNTMREKFYVHSE
GTLEIRGITPKEGGLYTCIATNLVGADLKSIMIKVGGSVPQDNNGSLNIKIRDIRANSVL
VSWKASSKILKSSVKWTAFVKTEDSHAAQSARIPFDVKVYNLTHLKPSTEYKICIDIPTV
YQKSRKQCVNVTTKSLEHDGKEYGKNHTVFVACVGGLLGIIGVMCLFSCVSQEGSSEGEH
SYAVNHCHKPA

670	LAFSELYPP LINLWESSKEKRATLEVK		LRR1	2
697	ATAIGVPTN MS			LRR2	2.2


>ENSMUSP00000097897

MLLWILLLETSLCFAAGNVTGDV

24	CKEKICSCNEIEGDLHVDCEKKGFTSLQRFTAPT		NF	4

SQ

60	FYHLFLHGN SLTRLFPNEFANFYN		LRR1	3.4
84	AVSLHMENN GLHEIVPGAFLGLQL		LRR2	2.8
108	VKRLHINNN KIKSFRKQTFLGLDD		LRR3	2.8
132	LEYLQADFN LLRDIDPGAFQDLNK		LRR4	3.4
156	LEVLILNDN LISTLPANVFQYVP		LRR5	4
179	ITHLDLRGN RLKTLPYEEVLEQIPG		LRR6	3.4
204	IAEILLEDN				LRR7	2.8
213	PWDCTCDLLSLKEWLENIPKNALIGRVVCEAPTRLQGKDLNETTEQDLCPL		CF1_short	3

SHIFT -46!

218	CDLLSLKEW LENIPKNALIGRVVCEAPTR		LRR1	2
247	LQGKDLNET TEQDLCPLKNRVDSSLPAPPA		LRR2	2.4

QEETFAPGPLPTPFKTNGQDEHATPGAVPNGGTKIPGNWQLKIKPTPPIATGSARNKPPV
HGLP

341	CPGGCSCDHIPGSGLKMNCNNRNVSSLADLKPKL		NF	4

SHIFT -25!

350	IPGSGLKMN CNNRNVSSLADLKPKLSN		LRR1	2.4
377	VQELFLRDN KIHSIRKSHFVDYKN		LRR2	3.4
401	LILLDLGNN NIANIENNTFKNLLD		LRR3	4
425	LRWLYMDSN YLDTLSREKFAGLQN		LRR4	3.4
449	LEYLNVEYN AIQLILPGTFNAMPK		LRR5	3.4
473	LRILILNNN LLRSLPVDVFAGVS		LRR6	4
496	LSKLSLHNN YFMYLPVAGVLDQLTS		LRR7	4
521	IIQIDLHGN				LRR8	2.8
530	PWECSCTIVPFKQWAERLGSEVLMSDLKCETPVNFFRKDFMLLSNEEICPQ		CF1_short	3

SHIFT -60!

521	IIQIDLHGN PWECSCTIVPFKQWAER		LRR8	2.8
547	LGSEVLMSD LKCETPVNFFRKDFMLLSNEE		LRR9	2

ICPQLYARISPTLTSHSKNSTG

599	LAETGTHSN SYLDTSRVSISV			LRR1	2
620	LVPGLLLVF VTSAFTVVGMLVFILRNRKRS		LRR2	2.4

KRRDANSSASEINSLQTVCDSSYWHNGPYNADGSHRVYDCGSHSLSD

>ENSMUSP00000047213

MSLASGP

8	SSKLLLFSL GMGLVSGSKCPNKCVCQDQE		LRR1	2
37	VACIDLHLT EYPADIPLNTRRLYLNNNKIT		LRR2	2.2

SHIFT -28!

39	CIDLHLTEY PADIPLNTRRLYLNNNKITS		LRR1	2
68	LPALQLGFL SDLVYLDCQNNRIREVMDYTF		LRR2	3

SHIFT -27!

71	LQLGFLSDL VYLDCQNNRIREVMDYTFIGI		LRR1	2.4
101	FRLIYLDLS SNNLTSISPFSFSVLTN		LRR2	2.2
127	LVRLNISHN PHLLYLDKYVFANTTS		LRR3	3.4
152	LRYLDLRNT GLHIIDHNGFHHLVV		LRR4	3.4
176	LQTLYLSGN				LRR5	4
185	PWICNCSFLDFTIHLLVSHMDHPDAQNATCTEPAELKGWPITKVGNPLQYMC		CF1_short	3

ITHLDQQD

245	YIFLLLIGF CIFAAGTVAAWLT			LRR1	2
267	GVCAVLYQN ALRTSSGDDTEDETGSRFANQ		LRR2	2.8

IFRSNTHLGPIRRFPELI

>ENSMUSP00000051895

MLRSALLSAVLALLRAQPFP

21	CPKTCKCVVRDAAQCSGGSVAHIAELGLPT		NF	4

N

52	LTHILLFRM DQGILRNHSFSGMTV		LRR1	2.4
76	LQRLMLSDS HISAIDPGTFNDLVK		LRR2	3.4
100	LKTLRLTRN KISRLPRAILDKMVL		LRR3	4
124	LEQLFLDHN ALRDLDQNLFQQLRN		LRR4	4
148	LQELGLNQN QLSFLPANLFSSLRE		LRR5	4
172	LKLLDLSRN NLTHLPKGLLGAQVK		LRR6	4
196	LEKLLLYSN QLTSVDSGLLSNLGA		LRR7	4
220	LTELRLERN HLRSVAPGAFDRLGN		LRR8	4
244	LSSLTLSGN LLESLPPALFLHVSS		LRR9	4
268	VSRLTLFEN PLEELPDVLFGEMAG		LRR10	3.4
292	LRELWLNGT HLSTLPAAAFRNLSG		LRR11	3.4
316	LQTLGLTRN PRLSALPRGVFQGLRE		LRR12	4
341	LRVLALHTN ALAELRDDALRGLGH		LRR13	4
365	LRQVSLRHN RLRALPRTLFRNLSS		LRR14	3.4
389	LESVQLEHN QLETLPGDVFAALPQ		LRR15	3.4
413	LTQVLLGHN				LRR16	3.4
422	PWLCDCGLWPFLQWLRHHPDILGRDEPPQCRGPEPRASLSFWELLQGDPWCP		CF1_short	3

SHIFT -41!

433	LQWLRHHPD ILGRDEPPQCRGPEPRAS		LRR1	2
460	LSFWELLQG DPWCPDPRSLPLDPPTENALE		LRR2	2.4

SHIFT -12!

478	LPLDPPTEN ALEAPVPSWLPNSWQSQTWAQ		LRR1	2
508	LVARGESPN NRLYWGLYILLLVAQAIIAAF		LRR2	2

SHIFT -15!

523	LYILLLVAQ AIIAAFIVFAMIKIGQLFRT		LRR1	3
552	LIREKLLLE AMGKSCN			LRR2	2


>ENSMUSP00000074381

MGRYW

6	LLPGLLLSL PLVTGWSTSNCLVTE		LRR1	2.4
30	GSRLPLVSR YFTFCRHSKLSFLAAC		LRR2	2.4
55	LSVSNLTQT LEVVPRTVEGLCLGGTVSTLL		LRR3	2.4

SHIFT -14!

71	VEGLCLGGT VSTLLPDAFSAFPG		LRR1	2.8
94	LKVLALSLH LTQLLPGALRGLGQ		LRR2	3
117	LQSLSFFDS PLRRSLFLPPDAFSDLIS		LRR3	2.8
144	LQRLHISGP CLDKKAGIRLPPG			LRR4	2.4
166	LQWLGVTLS CIQDVGELAGMFPDLVQGSSS		LRR5	2.8

RVSWT

201	LQKLDLSSN WKLKMASPGSLQGLQ		LRR1	4
225	VEILDLTRT PLDAVWLKGLGLQK		LRR2	2.8
248	LDVLYAQTA TAELAAEAVAHFE			LRR3	2.4
270	LQGLIVKES KIGSISQEALASCHS		LRR4	2.8
294	LKTLGLSST GLTKLPPGFLTAMPR		LRR5	3.4
318	LQRLELSGN QLQSAVLCMNETGDVSG		LRR6	4
344	LTTLDLSGN RLRILPPAAFSCLPH		LRR7	4
368	LRELLLRYN QLLSLEGYLFQELQQ		LRR8	4
392	LETLKLDGN PLLHLGKNWLAALPA		LRR9	4
416	LTTLSLLDT QIRMSPEPGFWGAKN		LRR10	3.4
440	LHTLSLKLP ALPAPAVLFLPMY			LRR11	3
462	LTSLELHIA SGTTEHWTLSPAIFPS		LRR12	3
487	LETLTISGG GLKLKLGSQNASGVFPA		LRR13	2.4
513	LQKLSLLKN SLDAFCSQGTSN			LRR14	4
534	LFLWQLPKL QSLRVWGAGNSSRPCLITG		LRR15	2.4
562	LPSLRELKL ASLQSITQPRSVQ			LRR16	2
584	LEELVGDLP QLQALVLSSTGLKSLSAAAFQ		LRR17	2.4

SHIFT -23!

591	LPQLQALVL SSTGLKSLSAAAFQRLHS		LRR1	2.4
618	LQVLVLEYE KDLMLQDSLREYSPQMP		LRR2	3
644	HYIYILESN LACHCANAWMEPWVKRSTKTY		LRR3	2

IYIRDNRLCPGQDRLSARGS

694	LPSFLWDHC PQTLELKLFLASSALVFM		LRR1	2.2
721	LIALPLLQE ARNSWIPYLQALFRVW		LRR2	3
746	LQGLRGKGD KGKRFLFDVFVSHCRQDQGWV		LRR3	2.4

SHIFT -16!

760	LFDVFVSHC RQDQGWVIEELLPALEGFL		LRR1	2.2
788	PAGLGLRLC LPERDFEPGKDVVDNVVDSML		LRR2	2.4

SSRTT

823	LCVLSGQAL CNPRCRLELRLATSLLLAAPS		LRR1	2.4
853	PPVLLLVFL EPISRHQLPGYHR			LRR2	2
875	LARLLRRGD YCLWPEEEERKSGFWTWLRSR		LRR3	2

LG

>ENSMUSP00000084507

MESESSRRMGNACIPLKRI

20	AYFLCLFSV VLLTEGKKPAKPKCPAVCTC		LRR1	2.4
49	SKDNALCEN ARSIPRTVPPDVISLSFVRSG		LRR2	2

SHIFT -10!

69	VISLSFVRS GFTEISEGSFLFTPS		LRR1	2.2
93	LQLLLFTSN SFDVISDDAFIGLPH		LRR2	3.4
117	LEYLFIENN NIKSISRHTFRGLKS		LRR3	3.4
141	LIHLSLANN NLQTLPKDIFKGLDS		LRR4	4
165	LTNVDLRGN				LRR5	3.4
174	AFNCDCKLKWLVEWLGHTNATVEDIYCEGPPEYKKRKINSLSPKDFDCI		CF1_short	3

ITEFAKSQDLPYQSLSIDTFSYLNDEYVVIAQPFTGKCIFLEWDHVEKTFRNYDNITGTS
TVVCKPIVIDTQLYVIVAQLFGGSHIYKRDGFANKFIKIQDIEVLKIRKPNDIETFKIED
NWYFVVADSSKAGFTTIYKWNGNGFYSHQSLHAWYRDTDVEYLEIA

389	RPPLALRTP HLILSSSSQRPVIYQW		LRR1	2
414	SKATQLFTN QTDIPNMEDVYAVKHFSVKGD		LRR2	2

VYICLTRFIGDSKVMKWGGSSFQDIQRMPSRGSMVFQPLQINNYQYAILGSDYSFTQVYN
WDAEKAKFVKFQELNVQAPRSFTHVSINKRNFLFASSFKGNTQIYKHVIVDLSA

>ENSMUSP00000022124

MAPDISCFF

10	LVALFLASC RATTSSDQKCIEKEVNKT		LRR1	3.4
37	YNCENLGLN EIPGTLPNSTECLEFS		LRR2	2
62	FNVLPTIQN TTFSRLINLTFLDLTRCQIYW		LRR3	2.4

SHIFT -16!

76	LINLTFLDL TRCQIYWIHEDTFQSQHR		LRR1	2.4
103	LDTLVLTAN PLIFMAETALSGPKA		LRR2	4
127	LKHLFFIQT GISSIDFIPLHNQKT		LRR3	2.8
151	LESLYLGSN HISSIKLPKGFPTEK		LRR4	4
175	LKVLDFQNN AIHYLSKEDMSSLQQ		LRR5	3.4
199	ATNLSLNLN GNDIAGIELGAFDSAV		LRR6	3.4
224	FQSLNFGGT QNLLVIFKGLKNST		LRR7	2.2
247	IQSLWLGTF EDMDDEDISPAVFEGLCEMSV		LRR8	2.4

ESINLQKHYFFNISSNTFHCFSG

300	LQELDLTAT HLSELPSGLVGLST		LRR1	3.4
323	LKKLVLSAN KFENLCQISASNFPS		LRR2	4
347	LTHLSIKGN TKRLELGTGCLENLEN		LRR3	3.4
372	LRELDLSHD DIETSDCCNLQLRNLSH		LRR4	3
398	LQSLNLSYN EPLSLKTEAFKECPQ		LRR5	4
422	LELLDLAFT RLKVKDAQSPFQNLHL		LRR6	3.4
447	LKVLNLSHS LLDISSEQLFDGLPA		LRR7	3.4
471	LQHLNLQGN HFPKGNIQKTNSLQTLGR		LRR8	4
498	LEILVLSFC DLSSIDQHAFTSLKM		LRR9	3.4
522	MNHVDLSHN RLTSSSIEALSHLK		LRR10	2.8
545	GIYLNLASN RISIILPSLLPILSQ		LRR11	3.4
569	QRTINLRQN				LRR12	2.4
578	PLDCTCSNIYFLEWYKENMQKLEDTEDTLCENPPLLRGVRLSDVTLSCSMA		CF1	4

SHIFT -55!

574	LRQNPLDCT CSNIYFLEWYKENMQKLE		LRR1	2.4
601	DTEDTLCEN PPLLRGVRLSDVTLSCSMAAV		LRR2	2

SHIFT -18!

613	LRGVRLSDV TLSCSMAAVGIFF			LRR1	2.4
635	LIVFLLVFA ILLIFAVKYFLRWKYQHI		LRR2	2.4


>ENSMUSP00000057725

MLPGLRRLLQGPASACLLLTLLALPSVTPS

31	CPMLCTCYSSPPTVSCQANNFSSVPLSLPPS		NF	4

SHIFT -9!

53	SVPLSLPPS TQRLFLQNNLIRS			LRR1	2.4
75	LRPGTFGPN LLTLWLFSNNLSTIHPGTFRH		LRR2	2.8
105	LQALEELDL GDNRHLRSLEPDTFQGLER		LRR3	2
133	LQSLHLYRC QLSSLPGNIFRGLVS		LRR4	3.4
157	LQYLYLQEN SLLHLQDDLFADLAN		LRR5	4
181	LSHLFLHGN RLRLLTEHVFRGLGS		LRR6	4
205	LDRLLLHGN RLQGVHRAAFHGLSR		LRR7	4
229	LTILYLFNN SLASLPGEALADLPA		LRR8	4
253	LEFLRLNAN				LRR9	4
262	PWACDCRARPLWAWFQRARVSSSDVTCATPPERQGRDLRALRDSDFQAC		CF1_short	3

PPPTPTRPGSRARGNSSSNHLYGVAEAGAPPADPSTLYRDLPAEDSRGRQGGDAPTEDDY
WGGYGGEDQRGEQTCPGAACQAPADSRGPALSAGLRTPLLCLLPLALHHL

>ENSMUSP00000054140

MEYQILKMSSCLFILLF

18	LTPGILCIC PLQCTCTERHRHVDCSGRN		LRR1	2.8
24	CICPLQCTCTERHRHVDCSGRNLTTLPPGLQEN		NF	4

SHIFT -11!

46	LTTLPPGLQ ENIIHLNLSYNHFTDL		LRR2	2
71	HNQLTPYTN LRTLDISNNRLESLPAQLPRS		LRR3	2
101	LWNMSAANN NIKLLDKSDTAYQWN		LRR4	2.8
125	LKYLDVSKN MLEKVVLIKNTLRS		LRR5	3.4
148	LEVLNLSSN KLWTVPTNMPSK			LRR6	4
169	LHIVDLSNN SLTQILPGTLINLTN		LRR7	3.4
193	LTHLYLHNN KFTFIPEQSFDQLLQ		LRR8	4
217	LQEITLHNN RWSCDHKQNITYLLKWVMETK		LRR9	3.4

AHVIGTPCSKQVSSLKEQSMYPTPPGFTSSLFTMSEMQTVDTINSLSMVTQPKVTKTPKQ
YRGKETTFGVTLSKDTTFSSTDRAVVAYPEDTPTEMTNSHEAAAATLTIHLQDGMSSNAS
LTSATKSPPSPVT

380	LSIARGMPN NFSEMPRQSTTLN			LRR1	2.8
402	LRREETTAN GNTRPPSAASAWKV		LRR2	2
425	NASLLLMLN AVVMLAG			LRR3	3


>ENSMUSP00000037096

MARLSTGKAACQVVLGLLITSLTESSILTSE

32	CPQLCVCEIRPWFTPQSTYREATTVDCNDLRLTRIPGNLSSD		NF	4

SHIFT -16!

58	CNDLRLTRI PGNLSSDTQVLLLQSNNIAKT		LRR1	2
88	VDELQQLFN LTELDFSQNNFTNIKEVG		LRR2	2.4
115	LANLTQLTT LHLEENQISEMTDYCLQDLSN		LRR3	2.4
145	LQELYINHN QISTISANAFSGLKN		LRR4	3.4
169	LLRLHLNSN KLKVIDSRWFDSTPN		LRR5	4
193	LEILMIGEN PVIGILDMNFRPLSN		LRR6	3.4
217	LRSLVLAGM YLTDVPGNALVGLDS		LRR7	3
241	LESLSFYDN KLIKVPQLALQKVPN		LRR8	3.4
265	LKFLDLNKN PIHKIQEGDFKNM			LRR9	4
287	LRLKELGIN NMGELVSVDRYALDNLPE		LRR10	3
314	LTKLEATNN PKLSYIHRLAFRSVPA		LRR11	3.4
339	LESLMLNNN ALNAVYQKTVESLPN		LRR12	4
363	LREISIHSN				LRR13	2.8
372	PLRCDCVIHWINSNKTNIRFMEPLSMFCAMPPEYRGQQVKEVLIQDSSEQ		CF1_short	3

CLPMISHDTFPNHLNMDIGTTLFLDCRAMAEPEPEIYWVTPIGNKITVETLSDKYKLSSE
GTLEIANIQIEDSGRYTCVAQNVQGADTRVATIKVNGTLLDGAQVLKIYVKQTESHSILV
SWKVNSNVMTSNLKWSSATMKIDNPHITYTARVPVDVHEYNLTHLQPSTDYEVCLTVSNI
HQQTQKSCVNVTTKTAAFALDISDHETSTALAAVMGSMFAVISLASIAIYIAKRFKRKNY
HHSLKKYMQKTSSIPLNELYPPLINLWEADSDKDKDGSADTKPTQVDTSRSYYMW

>ENSMUSP00000010455

MKFLAFLSLLSLVLQKAETASLLGEREREEQSPEEGDTYASLYVGNHTLSIEDYNEVIDL
SNYEELADYGDQIPEAKISNLTLPTRTSPTSTVAQKTLSPNLTMAVPTTTGLLNSQSSHG
LPT

124	CLVCVCLGSSVYCDDADLENIPPLPQM		NF	4

SHIFT -26!

125	LVCVCLGSS VYCDDADLENIPPLPQM		LRR1	2.8
151	TTYLYARFN HISHIQAGDFKGLTK		LRR2	2.4
175	LRRIDLSGN SISSIHNDALRLLPA		LRR3	3.4
199	LQDLILPEN QLAALPVLPSGIEFLDVR		LRR4	4
226	LNRLQSSGI QPEAFVALKKLQFLY		LRR5	2
250	LANNMLDSI PGPLPLSLRSLHLQNNMIETM		LRR6	2

ESDTFCDTGEHRHERRQ

297	LEDIRLDGN PINLSLFPEAYFC			LRR1	3.4
319	LPRLPVGHF T			LRR2	2.4


>ENSMUSP00000054960

MTCWLHMLGLHLLLLPTAPLAAG

24	CPARCECSASTRTVACGRRRLTAIPEGIPAE		NF	4
55	TRMLELSRN RIRCLNPGDLASLPT		LRR1	3
79	LEELDLNHN VIAHVEPGAFANLPR		LRR2	4
103	LRVLRLRGN QLKLIPPGVFTHLDS		LRR3	4
127	LTLLDLSEN KLVILLDFSFQDLRS		LRR4	4
151	LQRLEVGDN DLVFISRRAFAGLLG		LRR5	3.4
175	LAELTLERC NLTSLSPESLGHLRG		LRR6	3.4
199	LGALRLRHL AIAALEDQNFQKLPG		LRR7	3
223	LSHLEIDNW PLLEEVAPGSLRGLN		LRR8	2.4
247	LTSLSITHT NITAVPAAALRQQAH		LRR9	2.8
271	LTCLNLSHN PISMVPRGSFRDLVR		LRR10	4
295	LRELHLAGA LLAVIEPQAFVGLRQ		LRR11	3
319	IRLLNLSDN LLSTLEENTFHSVNT		LRR12	3.4
343	LETLRVDGN				LRR13	3.4
352	PLACDCRLLWIVQRRKTLNFDGRLPACATPAEVRGDALHNLPDSVLFEY		CF1_short	3

FVCRKPKIRERRLQHVTATEGDDVRFLCRAEGEPAPTVAWVTPQHHSVTAASRGRARVLP
GGTLTIADTRPQDSGTYTCVASNAGGNDTYFATLTVQPAANRTQGDGHNETQV

514	GVRFPLDLT TILVSTAMGCITFLGVV		LRR1	2.2
540	LFCFLLLFV WSRGRGQHKNNFSVEYSFRKV		LRR2	2.4

DGPAAAAGQGGARKFNMKMI

>XP_984483

MPLLAPRLLPLLLVIGTGGSV

22	PRPLALPQG CYVAEEAGEQTFRCS		LRR1	2
46	RAGLSAVPN GIPNDTRKLYLDANQLASVPA		LRR2	2.4

SHIFT -16!

60	TRKLYLDAN QLASVPAGAFQHLPA		LRR1	3
84	LEELDLSHN ALVHLSGAAFQGLEGT		LRR2	4
109	LRHLDLSAN QLASVPVAAFVGL			LRR3	4
131	QIQVNLSAN				LRR4	2.4
140	PWRCDCALQEVLRHVRLAPGSGTGIVCGPEARPDLVGHEFLLLTREEEL		CF1_short	3

SHIFT -38!

151	LRHVRLAPG SGTGIVCGPEARPDLVG		LRR1	2.4
177	HEFLLLTRE EELCGTGRGGTRRSTDVAL		LRR2	2
205	LVTMGGWLT LVVAYLIRYVWQNRDETRRPV		LRR3	2.2

KRAPPAQPVRSEDSSTLSTVL

>ENSMUSP00000037616

MAVLPLLLCLLPLAPASSPPQPAISSP

28	CPRRCRCQTQSMPLSVLCPGAGLLFVPPSLDRR		NF	4
61	AAELRLADN FIAAVRRRDLANMTG		LRR1	3.4
85	LLHLSLSRN TIRHVAAGAFADLRA		LRR2	4
109	LRALHLDGN RLTSLGEGQLRGLVN		LRR3	4
133	LRHLILSNN QLAALAAGALDDCAET		LRR4	4
158	LEDLDLSYN NLEQLPWEALGRLGN		LRR5	4
182	VNTLGLDHN LLASVPAGAFSRLHK		LRR6	3.4
206	LARLDMTSN RLTTIPPDPLFSR			LRR7	3.4
228	LPLLARPRG SPASALVLAFGGNPLHCNCE		LRR8	2
257	LVWLRRLAR EDDLEACASPPALGGRYFWAV		LRR9	2

GEEEFVCEPPVVTHRSPPLAVPAGRPAALRCRAVGDPEPRVRWVSPQGRLLGNSSRARAF
PNGTLELLVTEPEDGGTFTCIAANAAGEATAAVELTVGPPPPPQLANSTSCDPPRDGEPD
ALTPPSAASASAKVADTVAPTDRGVQVTEHGATAALVQWPDQRPVPGIRMYQIQYNSSAD
DILVYRMIPADSRSFLLTDLASGRTYDLCVLAVYEDSATGLTATRPVGCARFSTEPALRP
CAAPHAPFLGGTMIIALGGVIVASVLVFIFVLLLRYKVHGGQPPGKAKATAPVSSVCSQT
NGALGPVPSAPAPEPAAPRAHTVVQLDCEPWGPSHEPAGP

>OTTMUSP00000016116

M

2	PGPLRLLCF FALGLLGSAGPSGA		LRR1	2
25	APPLCAAPC SCDGDRRVDCSGKGLTAVPEG		LRR2	2.2

LSAF

59	TQALDISMN NITQLPEDAFKNFPF		LRR1	2.4
83	LEELQLAGN DLSFIHPKALSGLKE		LRR2	4
107	LKVLTLQNN QLKTVPSEAIRGLSA		LRR3	4
131	LQSLRLDAN HITSVPEDSFEGLVQ		LRR4	4
155	LRHLWLDDN ILTEVPVRPLSNLPT		LRR5	4
179	LQALTLALN NISSIPDFAFTNLSS		LRR6	4
203	LVVLHLHNN KIKSLSQHCFDGLDN		LRR7	4
227	LETLDLNYN NLDEFPQAIKALPS		LRR8	4
250	LKELGFHSN SISVIPDGAFAGNPL		LRR9	3.4
274	LRTIHLYDN PLSFVGNSAFHNLSD		LRR10	3.4
298	LHSLVIRGA SLVQWFPNLAGTVH		LRR11	2.4
321	LESLTLTGT KISSIPDDLCQNQKM		LRR12	3.4
345	LRTLDLSYN DIRDLPSFNGCRA			LRR13	4
367	LEEISLQRN QISLIKETTFQGLTS		LRR14	3.4
391	LRILDLSRN LIREIHSGAFAKLGT		LRR15	4
415	ITNLDVSFN ELTSFPTEGLNG			LRR16	2.8
436	LNQLKLVGN FQLKDALAARDFAN		LRR17	4
459	LRSLSVPYA YQCCAFWGCDSYANLNTEDNS		LRR18	2.4

PQDHSVTKEKGATDAANATSTAESEEHSQIIIHCTPSTGAFKPCEYL

536	LGSWMIRLT VWFIFLVALLFNL			LRR1	2.2
558	LVILTVFAS CSSLPASKLFIGLISVSNLLM		LRR2	2.8

SHIFT -18!

570	LPASKLFIG LISVSNLLMGIYTGI		LRR1	2
594	LTFLDAVSW GRFAEFGIWWETGSGCKV		LRR2	2.4
621	AGSLAVFSS ESAVFLLTLAAVERSVFAKDV		LRR3	2.2

MKNGKSSHLRQFQVAA

667	LVALLGAAI AGCFPLFHGGQYS			LRR1	2.4
689	ASPLCLPFP TGETPSLGFTVTLVL		LRR2	2.4
713	LNSLAFLLM AIIYTKLYCNLEKEDPSENSQ		LRR3	2.4

SHIFT -20!

723	IIYTKLYCN LEKEDPSENSQSSMIKH		LRR1	2.4
749	VAWLIFTNC IFFCPVAFFSFAPLITAISIS		LRR2	2.2

PEIMKSVTLIFFPLPACLNPVLYVFFNPKFKDDWKLLKRRVTRKHGSVSVSISSQGGCGE
QDFYYDCGMYSHLQGNLTVCDCCESFLLTKPVSCKHLIKSHSCPVLTVASCQRPEAYWSD
CGTQSAHSDYADEEDSFVSDSSDQVQACGRACFYQSRGFPLVRYAYNLPRVRD

>ENSMUSP00000040477

MALLLVSL

9	LAFLGSGSG CHHWLCHCSNRVFLCQDSKVT		LRR1	2
39	EIPPDLPRN AIELRFVLTKLRVIPKGSFSG		LRR2	2

SHIFT -21!

48	AIELRFVLT KLRVIPKGSFSGFGD		LRR1	2.2
72	LEKIEISQN DVLEVIEADVFSN			LRR2	2.8
94	LPNLHEIRI EKANNLLYINPEAFQNLPS		LRR3	2
122	LRYLLISNT GIKHLPAFHKIQS			LRR4	2.8
144	LQKVLLDIQ DNINIHIIARNSFMGLSFE		LRR5	2.4
172	SVILWLNKN GIQEIHNCAFNGTQ		LRR6	3
195	LDELNLSDN NNLEELPDDVFQGASGPVVLD		LRR7	4

SHIFT -19!

206	LEELPDDVF QGASGPVVLDISRTKVYS		LRR1	2
233	LPNHGLENL KKLRARSTYRLKK			LRR2	2
255	LPSLDKFVM LIEASLTYPSHCCAFANWRRQ		LRR3	2
285	TSELHPICN KSISRQDIDDMTQPGDQRVSL		LRR4	2

VDDEPSYGKGSDMLYSEFDYDLCNEFVDVTCSPKPDAFNPCEDIMGYNI

364	LRVLIWFIS ILAITGNTTVLVVLTTSQYKL		LRR1	2.8
394	TVPRFLMCN LAFADLCIGIYLLLIASVDIH		LRR2	2

TKSQYHNYAIDWQTGAGCDAAGFFTVFASELSVYTLAAITLERWHTITHAMQLECKVQLC
HAASIMVLGWAFAFAAA

501	LFPIFGISS YMKVSICLPMDIDSP		LRR1	2.2
525	LSQLYVMAL LVLNALAFVVICGCYTHIYLT		LRR2	2.4

VRNPNIVSSSRDTKIAKRMATLIFTDFLCM

585	APILFFAIS ASLKVPLITVSKAKI		LRR1	2.2
609	LLVLFYPIN SCANPFLYAIFTKNFRRDFFV		LRR2	3

LMSKFGCYEVQAQIYKTETSSITHNFHSRKNPCSSAPRVTNSYVLVPLNHSVQN

>ENSMUSP00000069318

MFPGAWLCWVSLLLLARLTQPCPVGCDCFGREVFCSDEQLADIPPDIPPHITDIVFVETA
FTTVRTRAFSGSPN

75	LTKVVFLNT QVRHLEPDAFGGLPR		LRR1	2.2
99	LQDLEITGS PVSNLSAHIFSNLSS		LRR2	2.8
123	LEKLTLDFD RLAGLPEDLFCHMDI		LRR3	3
147	LESLQLQGN QLRTLPGRLFQSLRD		LRR4	4
171	LRTLNLAQN LLTQLPKGAFQSLTG		LRR5	4
195	LQMLKLSNN MLARLPEGALGSLSS		LRR6	4
219	LQELFLDGN AITELSPHLFSQLFS		LRR7	4
243	LEMLWLQHN AICHLPVSLFSSLHN		LRR8	4
267	LTFLSLKDN ALRTLPEGLFAHNQG		LRR9	4
291	LLHLSLSYN QLETIPEGAFTNLSR		LRR10	4
315	LVSLTLSHN AITDLPEHVFRNLEQ		LRR11	4
339	LVKLSLDSN NLTALHPALFHNLSR		LRR12	4
363	LQLLNLSRN QLTTLPGGIFDTNYD		LRR13	4
387	LFNLALLGN				LRR14	4
396	PWQCDCHLSYLTSWLRLYNNQISNTHTFCAGPAYLKGQLVPNLKQEQLICP		CF1_short	3

SHIFT -44!

403	LSYLTSWLR LYNNQISNTHTFCAGPA		LRR1	2
429	YLKGQLVPN LKQEQLICPVNPGHLS		LRR2	2.4
454	FRALGLDEG EPAGSWDLTVEGRAAHSQCAY		LRR3	2.4

SNPEGTVLLACEESRCRWLNIQLSSRDGSDSAAMVYNSSQEWGLRSSCGLLRVTVSIEAP
AAGP

>ENSMUSP00000056642

MAFVFYCFLQVLVSWVIHA

20	VQPFCLPEC TCSEESFGRSLQCMS		LRR1	2.2
24	CLPECTCSEESFGRSLQCMSMSLGKIPDNFPEE		NF	4

SHIFT -13!

44	MSLGKIPDN FPEELKQVRIENSP		LRR2	2.2
67	LFELSQGFF TNMSSLEYLWLNFNNVTVIH		LRR3	2
96	LGALEDLPE LRELRLEGNKLRSVPWTAFRA		LRR4	2

SHIFT -27!

99	LEDLPELRE LRLEGNKLRSVPWTAFRATPL		LRR1	2
129	LRVLDLKHN RIDSVPELALQFLTN		LRR2	4
153	LIYLDISSN RLTVVSKGVFLNWPAYQKRQQ		LRR3	3.4

L

184	GCGAEFLSN MVLSLHNNPWLCDCR		LRR1	2.2
208	LRGLAQFVK SVGPPFILVNSYLVCQGPVSK		LRR2	2

SHIFT -19!

219	GPPFILVNS YLVCQGPVSKAGQL		LRR1	2.2
242	LHETELGVC MKPTISTPSVNVTIQVGKNVT		LRR2	2.4

LQCFAQASPSPTIAWKYPLSTWREFDVLASPIAEGII

309	LSQLVIPAA QLVDGGNYTCMAFNSIGRSS		LRR1	2.4
338	LVILLYVQP AQAMPGLHFLSTSSEVSAYVD		LRR2	2

SHIFT -18!

350	MPGLHFLST SSEVSAYVDLRVVKQTVHGI		LRR1	2.2
379	LLQWLTVTN LAEEQWFTLYITSDEALRKKV		LRR2	2.4

VHIGPGINTYAVDDLLPATKYKACLSLRNQPPSQGQCVVFVTGKDSGGLEGREHLLHVTV
VLC

472	AVLLALPVG AYVWVSQGPYNFSEWCWRRCP		LRR1	2.4
502	LHRKTLRCP QAVPQCKDNSFKDPSGVYEDG		LRR2	2

ESHRVMEEDEEVEKEGIS

>ENSMUSP00000067897

MWL

4	LLHVILLTE VKDFALADSSMVAPLCPKGY		LRR1	2.4
33	FPCGNLTKC LPRAFHCDGVDDCGNGADEDN		LRR2	2.2

CGDTSGWTTIFGTVHGNVNKVTLTQE

89	CFLSQYPQHCYCRENELECVKADLKAVPKVSSN		NF	4
122	VTLLSLKKN KIHRLPVKVFSRYTE		LRR1	3.4
146	LRKIYLQHN CITHISRRAFLGLHN		LRR2	3.4
170	LQILYLSHN CITSLRPGIFKDLHQ		LRR3	4
194	LAWLILDDN PITRISQKSFMGLNS		LRR4	4
218	LFFLSMVGN RLEALPETLCAQMPQ		LRR5	3.4
242	LNWVDLANN GIKYITNSTFLTCDS		LRR6	3.4
266	LTVLFLPRN QIGFVPEKTFSSLKN		LRR7	4
290	LGELDLSSN MITKLPVHLFSDLHL		LRR8	4
314	LQKLNLSSN PLLYVHKNQFGSLKQ		LRR9	4
338	LQSLDLERI EIPNISTGMFQPMKN		LRR10	3
362	LSHIYLKTF RYCSYVPHVRICMPSTDGI		LRR11	2.4
390	SSSEDLLAN GILRVSVWVIAFITCVGNFLV		LRR12	2

SHIFT -24!

396	LANGILRVS VWVIAFITCVGNFLVIA		LRR1	2.8
422	VRSLIKAEN TTHAMSIKILCCADC		LRR2	2.4
446	LMGVYLFSV GVFDIKYRGQYQKYALLWMES		LRR3	2.4

VPCR

480	LLGFLATLS TEVSVLLLTFLTLEKF		LRR1	2.2
505	LVIVFPFSN LRLGKRQTAVALASIWVVGFL		LRR2	2.4

IAAVPFTREDYFGNFYGKNGVCFPLHYDQAEDFGSRGYSLGIFLGVNLLAFLVIVISYVT
MFCSIHKTALQTAEVRSHIGKEVAVANRFFFIVFSDAICWIPVFVVKI

643	LSLLQVEIP GTITSWIVVFFLPVNSALNPI		LRR1	2.4
673	LYTLTTSFF KDKLKQLLHKHRRKPIFKVKK		LRR2	2

SHIFT -18!

685	LKQLLHKHR RKPIFKVKKKSLSASIVWTD		LRR1	2
714	ESSLKLGVL SKIALGDSIMKPVSP		LRR2	2


>ENSMUSP00000040436

MALWRGGGALGLLLLSAACLIPPSAQVRRLAR

33	CPATCSCTKESIICVGSSWVPRIVPGD		NF	4
60	ISSLSLVNG TFLEIKDRMFSHLPS		LRR1	2.4
84	LQLLLLNSN SFTVIRDDAFAGLFH		LRR2	4
108	LEYLFIEGN KIETISRNAFRGLRD		LRR3	3.4
132	LTHLDLRGN				LRR4	4
141	KFECDCKAKWLYLWLKMTNSTVSDVLCIGPPEYQEKKLNEVTSFDYECT		CF1_short	3

TTGPQTDEAKQRGWQLELSLGFCELIFVFQHPLSDFVVHQTLPYQSVSVDTFNSKNDVYV
AIAQPSMENCMVLEWDHIEMNFRSYDNITGQSIVGCKAILIDDQVFVVVAQLFGGSHIYK
YDESWTKFVKFQDIEVSRISKPNDIELFEIDDETFFIIADSSKAGLSTVYKWNSKGFYSY
QSLHEWFRDTDAEFVDIDGKSHLILSSRSQVPIILQWNKSSKKFVPHGDIPNMEDVLAVK
SFRMQNTLYLSLTRFIGDSRVMRWNSKQFVEVQALPSRGAMTLQPFSFKDNHYLALGSDY
TFSQIYQWDKEKQQFKKFKEIYVQAPRSFTAVSTDRRDFFFASSFKGKTKIFEHIIVDLS
L

>ENSMUSP00000059913

MS

3	LRFHTLPTL PRAVKPGCRELLC			LRR1	2
25	LLVIAVMVS PSASGMCPTACICATDIVSCT		LRR2	2.2

NKNLSKVPGN

65	LFRLIKRLD LSYNRIGLLDADWIPVSFVK		LRR1	2
94	LSTLILRHN NITSISTGSFSTTPN		LRR2	4
118	LKCLDLSSN RLKSVKSATFQELKA		LRR3	4
142	LEVLLLYNN HISYLDPAAFGGLSH		LRR4	4
166	LQKLYLSGN FLTQFPMDLYTGRFKLAD		LRR5	4
193	LTFLDVSYN RIPSIPMHHINLVPGRQ		LRR6	3.4
219	LRGIYLHGN				LRR7	3.4
228	PFVCDCSLYSLLIFWYRRHFSSVMDFKNDYTCRLWSDSRHSHQLQLLQESFLNC		CF1_short	3

SYSVINGSFHALGFIHEAQVGERAIVHCDSKTGNGNTDFIWVGPDNRLLEPDKDMGNFRV
FYNGSLVIENPGFEDAGVYSCIAMNRQRLLNETVDIMINVSNFTINRSHAHEAFNTAFTT
LAACVASIVLVLLYLYLTPCPCKCKAKRQKNTLSQSSAHSSILSPGPTGDASADDRKAGK
RVVF

466	LEPLKDTAA GQNGKVKLFPSETV		LRR1	2
489	IAEGILKST RAKSDSDSVNSVFSDTPFVAS		LRR2	2.2

T

>ENSMUSP00000053123

MAQAHIRGSPCPLLPPGRMSWPHGALLLLWLFSPPLRAGGGGVAVTSAAGGGSPPATS

59	CPAACSCSNQASRVICTRRELAEVPASIPVN		NF	4
90	TRYLNLQEN SIQVIRTDTFKHLRH		LRR1	3
114	LEILQLSKN LVRKIEVGAFNGLPS		LRR2	4
138	LNTLELFDN RLTTVPTQAFEYLSK		LRR3	4
162	LRELWLRNN PIESIPSYAFNRVPS		LRR4	4
186	LRRLDLGEL KRLEYISEAAFEGLVN		LRR5	3
211	LRYLNLGMC NLKDIPNLTALVR			LRR6	3.4
233	LEELELSGN RLDLIRPGSFQGLTS		LRR7	4
257	LRKLWLMHA QVATIERNAFDDLKS		LRR8	3
281	LEELNLSHN NLMSLPHDLFTPLHR		LRR9	4
305	LERVHLNHN				LRR10	3.4
314	PWHCNCDVLWLSWWLKETVPSNTTCCARCHAPAGLKGRYIGELDQSHFTCY		CF1_short	3

APVIVEPPTDLNVTEGMAAELKCRTGTSM

394	TSVNWLTPN GTLMTHGSYRVRISV		LRR1	2
418	LHDGTLNFT NVTVQDTGQYTCMVTNSAGNT		LRR2	2.8

TASATLNVSAVDPVAAGGPGGGGPGGGGGAGGAGGYTYFTTVTVETLETQPGEEAQQPRG
TEKEPPGPTTDGAWGGGRPDAAAPASASTTAPAPRSSRPTEKAFTVPITDVTENALKDLD
DVMKTTKIIIGCFVAITFMAAVMLVAFYKLRKQHQLHKHHGPTRTVEIINVEDELPAASA
VSVAAAAAVAGGAGVGGDSHLALPALERDHLNHHHYVAAAFKAHYGGNPGGGCGAKGPGL
NSIHEPLLFKSGSKENVQETQI

>ENSMUSP00000062171

MGLQTTKWPGRGAFILKFWLIISLGLY

28	LQVSKLLAC PSVCRCDRNFVYCNERS		LRR1	2.4
54	LTSVPLGIP EGVTVLYLHNNQINNAGFPAE		LRR2	2.4

SHIFT -19!

65	VTVLYLHNN QINNAGFPAELHNVQS		LRR1	3.4
90	VHTVYLYGN QLDEFPMNLPKN			LRR2	2.8
111	VRVLHLQEN NIQTISRAALAQLLK		LRR3	3.4
135	LEELHLDDN SISTVGVEDGAFREAIS		LRR4	4
161	LKLLFLSKN HLSSVPVGLPVD			LRR5	4
182	LQELRVDEN RIAVISDMAFQNLTS		LRR6	3.4
206	LERLIVDGN LLTNKGIAEGTFSHLTK		LRR7	3.4
232	LKEFSIVRN SLSHPPPDLPGTH			LRR8	2.8
254	LIRLYLQDN QINHIPLTAFANLRK		LRR9	4
278	LERLDISNN QLRMLTQGVFDHLSN		LRR10	3.4
302	LKQLTARNN				LRR11	3.4
311	PWFCDCSIKWVTEWLKYIPSSLNVRGFMCQGPEQVRGMAVRELNMNLLSCP		CF1_short	3

SHIFT -60!

302	LKQLTARNN PWFCDCSIKWVTEW		LRR11	3.4
325	LKYIPSSLN VRGFMCQGPEQVRG		LRR12	2.4
348	MAVRELNMN LLSCPTTTPGLPVFTPAPSTV		LRR13	2.4

SPTTQSPTLSVPSPSRGSVPPAPTPSKLPTIPDWDGRERVTPPISERIQLSIHFVNDTSI
QVSWLSLFTVMAYK

452	LTWVKMGHS LVGGIVQERIVSGEKQH		LRR1	2.2
478	LSLVNLEPR STYRICLVPLDAFNYRTVEDT		LRR2	2.4

SHIFT -15!

493	LVPLDAFNY RTVEDTICSEATTH		LRR1	2.4
516	ASYLNNGSN TASSHEQTTSHSMGSPFL		LRR2	2.4
543	LAGLIGGAV IFVLVVLLSVFCWHMHKKGRY		LRR3	2.4

TSQKWKYNRGRRKDDYCEAGTKKDNSILEMTETSFQIVSLNNDQLLKGDFRLQPIYTPNG
GINYTDCHIPNNMRYCNSSVPDLEHCHT

>ENSMUSP00000077492

MKLWTYLLYPSLLACLSLQSQSPMPSVRGS

31	CDTLCNCEEKDGIMIINCEEKGINKLSQISVPP		NF	4

SR

66	PFHLSLLNN GLTMLHTNDFSGLTN		LRR1	3
90	ALSIHLGFN NIADIETGAFNGLGL		LRR2	2.8
114	LKQLHINHN SLEILKEDTFHGLEN		LRR3	3.4
138	LEFLQADNN FITIIEPSAFSKLNR		LRR4	3.4
162	LKVLILNDN AIESLPPNIFRFVP		LRR5	4
185	LTHLDLRGN QLQTLPYVGFLEHIGR		LRR6	4
210	ILDLQLEDN				LRR7	3.4
219	KWACNCELLQLKNWLENMPPQSIIGDVICYSPPPFKGSVLSRLKKESFCPT		CF1_short	3

SHIFT -12!

258	LSRLKKESF CPTPPVYEEHEDPS		LRR1	2
281	GSLLAITSS TSDSRLSSKNTSILKQPTKAP		LRR2	2.2

GLIPYLTKPSTQLPVPY

328	CPIPCNCKVLSPSGLLIHCQERNIESLSDLQPPP		NF	4

SHIFT -25!

337	LSPSGLLIH CQERNIESLSDLQPPPHN		LRR1	2
364	PRKLILAGN IIHTLMKSDLTDYFT		LRR2	3
388	LEMLHLGNN RIEVLEEGSFMNLTR		LRR3	4
412	LQKLYLNGN HLTKLNKGMFLGLHS		LRR4	4
436	LEYLYLEYN AVKEILPGTFNPMPK		LRR5	4
460	LKVLYLNNN LLQVLPAHIFLGIP		LRR6	4
483	LTRVNLKTN QFTHLPVSNILDDLDF		LRR7	3.4
508	LIQIDLEDN				LRR8	3.4
517	PWDCSCDLVGLQQWIHKLGKGTMTDDILCTSPGHLDKKELKALNSDLLCPG		CF1_short	3

SHIFT -44!

524	LVGLQQWIH KLGKGTMTDDILCTSPGH		LRR1	2
551	LDKKELKAL NSDLLCPGLVNNPSMPTQTTY		LRR2	2

VIVTSPTVAADTASTLFSSLTDAVPLSVLILGLLIVFITIVFCAAGIVVFVLHRRRRYKK
KKVEEQLRDNSPVHLQYSMYGHKTTHHTTERPSSSLYEQHMVSPMVHVYRSPSFGPKHLE
EVEERNDKEGNDAKHLQRSLLERENHSPLTGSNMKYKTTDQSTDFISF

749	QDASLLYRN ILEKERELQQLGITEYLRKN		LRR1	2
778	LAQLQPEVE VNYPGAHEELKLMETLMYSRP		LRR2	2

SHIFT -15!

793	HEELKLMET LMYSRPRKVLVEQTK		LRR1	2.4
817	NEYFELKAN LHAEPDYLEVLEQQT		LRR2	2.4


>ENSMUSP00000045162

MHSRSCLPPLLLLLLVLLGSGVQG

25	CPSGCQCNQPQTVFCTARQGTTVPRDVPPD		NF	4
55	TVGLYIFEN GITTLDVGCFAGLPG		LRR1	2.4
79	LQLLDLSQN QITSLPGGIFQPLVN		LRR2	4
103	LSNLDLTAN KLHEISNETFRGLRR		LRR3	4
127	LERLYLGKN RIRHIQPGAFDALDR		LRR4	4
151	LLELKLPDN ELRVLPPLHLPR			LRR5	4
172	LLLLDLSHN SIPALEAGILDTANVEALR		LRR6	4
200	LAGLGLRQL DEGLFGRLLNLHDLDVSDNQL		LRR7	3

SHIFT -18!

212	LFGRLLNLH DLDVSDNQLEHMPSVIQGLRG		LRR1	2
242	LTRLRLAGN TRIAQIRPEDLAGLTA		LRR2	4
267	LQELDVSNL SLQALPSDLSSLFPR		LRR3	2.4
291	LRLLAAARN				LRR4	3.4
300	PFNCLCPLSWFGPWVRENHVVLASPEETRCHFPPKNAGRLLLDLDYADFGCP		CF1_short	3

VTTTTATVPTIRSTIREPTLSTSSQAPTWPSLTEPTTQASTVLSTAPPTMRPAPQPQDCP
ASICLNGGSCRLGARHHWECLCPEGFIGLYCESPVEQGMKPSSIPDTPRP

462	PPLLPLSIE PVSPTSLRVKLQRYLQGNTVQ		LRR1	2
492	LRSLRLTYR NLSGPDKRLVTLRLPASLAE		LRR2	3
521	YTVTQLRPN ATYSICVTPLGAGRTPE		LRR3	2
547	GEEACGEAN TSQAVRSNHAPVTQAREGN		LRR4	2.2
575	LPLLIAPAL AAVLLAVLAAAGAAYCVRRAR		LRR5	2.4

SHIFT -14!

591	LAAAGAAYC VRRARATSTAQDKGQVGPG		LRR1	2.2
619	TGPLELEGV KAPLEPGSKATEGGGEALSGG		LRR2	2

PECEVPLMGYPGPSLQGVLPAKHYI

>ENSMUSP00000046705

MAGLRARRGPGRRLLVLSTLGFCLMLQVSAKRPPKTPP

39	CPPSCSCTRDTAFCVDSKSVPKNLPSE		NF	4
66	VISLTLVNA AFSEIQDGAFSHLPL		LRR1	2.4
90	LQFLLLNSN KFTLIGDNAFIGLSH		LRR2	4
114	LQYLFIENN DIWALSKFTFRGLKS		LRR3	3.4
138	LTHLSLANN NLQTLPRDIFRPLDI		LRR4	4
162	LSDLDLRGN				LRR5	4
171	ALNCDCKVKWLVEWLAHTNTTVAPIYCASPPRFQEHKVQDLPLREFDCI		CF1_short	3

SHIFT -39!

181	LVEWLAHTN TTVAPIYCASPPRFQEHK		LRR1	2.8
208	VQDLPLREF DCITTDFVLYQTLSFPAVSAE		LRR2	2.4

PFLYSSDLYLALAQPGASACTILKWDYVERQLRDYDRIPAPSAVHCKPMVVDGQLYVVVA
QLFGGSYIYHWDPNTTRFTKLQDIDPQRVRKPNDLEAFRIDGDWFFAVADSSKAGATSLY
RWHQNGFYSHQALHAWHRDTDLEFVDGEGKPRLIVSSSSQAPVIYQWSRSQKQFVAQGEV
TQVPDAQAVKHFRAGRDSYLCLSRYIGDSKILRWEGTRFSEVQALPSRGSLALQPFLVGG
HRYLALGSDFSFTQIYQWDEGRQKFVRFQELAVQAPRAFCYMPAGDAQLLLAPSFKGQTL
VYRHVVVDLSA

>ENSMUSP00000077611

MTSGPFFFCIFIIGKYFTLGSAQDVSCPLGSFPCGNMSR

40	CLPQLLHCN GVDDCGNRADEDHCGDNN		LRR1	2
67	GWSLQLDKY FANYYKLASTNSFEAETSECL		LRR2	2.4

VGSVPMHCL

106	CRDLELDCD EANLRAVPSVSSN			LRR1	2
128	VTVMSLQRN FIRTLPPNGFRKYHE		LRR2	2.8
152	LQKLCLQNN RIHSVSVSAFRGLRS		LRR3	4
176	LTKLYLSHN RITFLKPGVFEDLHR		LRR4	4
200	LEWLIIEDN HLSRISPLTFYGLNS		LRR5	3.4
224	LILLVLMNN ALTRLPDKPLCQHMPR		LRR6	4
249	LHWLDFEGN RIHNLRNLTFISCNN		LRR7	3.4
273	LTVLVMRKN KINYLNEHAFTHLQK		LRR8	3.4
297	LDELDLGSN KIENLPPNIFKDLKE		LRR9	4
321	LSQLNISYN PIQKIEVNQFDCLAK		LRR10	3.4
345	LKSLSLEGI EISNIQQRMFRP			LRR11	3
366	LINLSHIYF KKFQYCGYAPHVRSCKPNTDG		LRR12	2

ISSLENLLASIIQRVFVWVVSAITCFGNIFVICMRPYIRSENKLHAMSIISLCCADCLMG
VYLFVIGAFDLKFRGEYNKHAQPWMESVHCQFMGSLAILSTEVSVLLLTFLTLEKYICIV
YPFRCLRPRKCRTITVLIFIWIIGFIVAFAPLGNKEFFKNYYGTNGVCFPLHSEDTGSTG
AQIYSVVIFLGINLVAFIIIVFSYGSMFYSVHQSSVTVTEIQKQVKKEVVLAKRFFFIVF
TDALCWIPIFILKFLSLLQVEIPDSITSW

665	VVIFILPIN SALNPIIYTLTTRPFK		LRR1	2.8
690	EMIHQLWHN YRQRRSVDRKETQKAYAPSFI		LRR2	2

WVEMWPLQEMSSGFMKPGAFTDPCDLSLVSQSSRLNSYS

>ENSMUSP00000064443

MFLWLF

7	LIVSALISS TNADSDISVEI		LRR1	2.4
27	CNVCSCVSVENVLYVNCEKVSVYRPNQLKPPW		NF	4

SHIFT -25!

34	SVENVLYVN CEKVSVYRPNQLKPPWSN		LRR2	2
61	FYHLNFQNN FLNILYPNTFVNFSH		LRR3	2.8
85	AVSLHLGNN KLQNIEGGAFLGLSA		LRR4	3.4
109	LKQLHLNNN ELKILRADTFLGIEN		LRR5	4
133	LEYLQADYN LIKYIERGAFNKLHK		LRR6	3.4
157	LKVLILNDN LISFLPDNIFRFAS		LRR7	4
180	LTHLDIRGN RIQKLPYIGVLEHIGR		LRR8	3.4
205	VVELQLEDN				LRR9	3.4
214	PWNCSCDLLPLKAWLENMPYNIYIGEAICETPSDLYGRLLKETNKQELCPM		CF1_short	3

SHIFT -46!

219	CDLLPLKAW LENMPYNIYIGEAICETPSD		LRR1	2
248	LYGRLLKET NKQELCPMGTGSDFDVRILPP		LRR2	2.4

SQQENGFTTPNGHTTQTT

296	LHRLVTKPP KTTNPSKISGIVAGKA		LRR1	2
321	LSNRNLSQI VSYQTRVPPLTPCPVPCFCKT		LRR2	2

H

352	PSDLGLSVN CQEKNIQSMSELTPKP		LRR1	3
377	LNAKKLHVN GNNIKDVDISDFTEFEG		LRR2	3
403	LDLLHLGSN QITLIKGEVFHNLTN		LRR3	4
427	LRRLYLNGN QIERLYPEIFSGLHN		LRR4	4
451	LQYLYLEYN LIKEILAGTFDSMPN		LRR5	4
475	LQLLYLNNN LLKSLPVYIFSGAP		LRR6	4
498	LARLNLRNN KFMYLPVSGVLDQLQS		LRR7	4
523	LTQIDLEGN				LRR8	3.4
532	PWDCTCDLVALKLWLEKLNDGIVVKELKCETPVQFANIELKSLKNEILCPK		CF1_short	3

SHIFT -60!

523	LTQIDLEGN PWDCTCDLVALK			LRR8	3.4
544	LWLEKLNDG IVVKELKCETPVQFA		LRR9	2
568	NIELKSLKN EILCPKLLNKPSATFTSPAPA		LRR10	2

ITFTTPLGPIRSPPGGPVP

617	LSILILSIL VVLILTVFVAFC			LRR1	3
638	LLVFVLRRN KKPTVKHEGLGNSECG		LRR2	3.4
663	SMQLQLRKH DHKTNKKDGLSTEAFIPQTIE		LRR3	2

QMSKSHTCGLKESETGFMFSDPPGQKVMMRNAADKDKDLLHVDTRKRLSTIDELDELFPS
RDSNVFIQNFLESKKEYNSIGVSGFEIRYPEKQQDKKNKKSLIGGNHSKIVVEQRKSEYF
ELKAKLQSSPDYLQVLEEQTALNKI

>ENSMUSP00000082207

MV

3	LRRRTLHPL SLLVQAAVLAET			LRR1	2
24	LALGTLPAF LPCELKPHGLVD			LRR2	2.4
45	CNWLFLKSV PRFSAAASCSNITRLSLISNR		LRR3	2

SHIFT -10!

65	ITRLSLISN RIHHLHNSDFVHLSN		LRR1	3.4
89	LRQLNLKWN CPPTGLSPLHFSCHMTIEPRT		LRR2	4

SHIFT -16!

103	LSPLHFSCH MTIEPRTFLAMRT			LRR1	2.4
125	LEELNLSYN GITTVPRLPSSLVNLSLSH		LRR2	4
153	TNILVLDAN SLAGLYSLRVLFMDGNCYYKN		LRR3	3

SHIFT -20!

163	LAGLYSLRV LFMDGNCYYKNPCTGAVKVT		LRR1	2
192	PGALLGLSN LTHLSLKYNNLTKVPRQLPPS		LRR2	2.4
222	LEYLLVSYN LIVKLGPEDLANLTS		LRR3	3.4
246	LRVLDVGGN CRRCDHAPNPCIECG		LRR4	3.4
270	QKSLHLHPE TFHHLSHLEGLV			LRR5	2
291	LKDSSLHTL NSSWFQGLVNLSVLD		LRR6	2
315	LSENFLYES ITHTNAFQNLTR			LRR7	2.4
336	LRKLNLSFN YRKKVSFARLHLASSFKNL		LRR8	4
364	VSLQELNMN GIFFRLLNKYTLRWLADLPK		LRR9	2.4
393	LHTLHLQMN FINQAQLSIFGTFRA		LRR10	4
417	LRFVDLSDN RISGPSTLSEATPEEADDAEQ		LRR11	3.4

EELLSADPHPAPLSTPASKNFMDRCKNF

475	KFTMDLSRN NLVTIKPEMFVNLSR		LRR1	2.4
499	LQCLSLSHN SIAQAVNGSQFLPLTN		LRR2	4
524	LQVLDLSHN KLDLYHWKSFSELPQ		LRR3	4
548	LQALDLSYN SQPFSMKGIGHNFSFVTHLSM		LRR4	4
578	LQSLSLAHN DIHTRVSSHLNSNS		LRR5	4
601	VRFLDFSGN GMGRMWDEGGLYLHFFQGLSG		LRR6	2.8
631	LLKLDLSQN NLHILRPQNLDNLPKS		LRR7	4
656	LKLLSLRDN YLSFFNWTSLSFLPN		LRR8	4
680	LEVLDLAGN QLKALTNGTLPNGTL		LRR9	4
704	LQKLDVSSN SIVSVVPAFFALAVE		LRR10	3.4
728	LKEVNLSHN ILKTVDRSWFGPIVMN		LRR11	3.4
753	LTVLDVRSN				LRR12	3.4
762	PLHCACGAAFVDLLLEVQTKVPGLANGVKCGSPGQLQGRSIFAQDLRLCLDE		CF1	4

SHIFT -8!

806	DLRLCLDEV LSWDCFGLSLLAVAVGMVVPI		LRR1	2
836	LHHLCGWDV WYCFHLCLAWLPLLARSRRSA		LRR2	2.4

QTLPYDAFVVFDKAQSAVADWVYNELRVRLEERRGRRALRLCLEDRDWLPGQTLFENLWA
SIYGSRKTLFVLAHTDRVSGL

947	LRTSFLLAQ QRLLEDRKDVVV			LRR1	2
968	LVILRPDAH RSRYVRLRQRLCRQSVLFWPQ		LRR2	2

QPNGQGGFWAQLSTALTRDNRHFYNQNFCRGPTAE

>ENSMUSP00000021008

MAVRPTRRCLLALLLCFAWWAMAVVASKQGAG

33	CPSRCLCFRTTVRCMHLLLEAVPAVAPQ		NF	4
61	TSILDLRFN RIREIQPGAFRRLRS		LRR1	3
85	LNTLLLNNN QIKKIPNGAFEDLEN		LRR2	4
109	LKYLYLYKN EIQSIDRQAFKGLAS		LRR3	4
133	LEQLYLHFN QIETLDPESFQHLPK		LRR4	4
157	LERLFLHNN RITHLVPGTFSQLES		LRR5	4
181	MKRLRLDSN				LRR6	3.4
190	ALHCDCEILWLADLLKTYAQSGNAQAAATCEYPRRIQGRSVATITPEELNCE		CF1_short	3

SHIFT -61!

181	MKRLRLDSN ALHCDCEILWLADL		LRR6	3.4
204	LKTYAQSGN AQAAATCEYPRRIQGRSVATI		LRR7	2

TPEELNCERPRITSEPQDADVTSGNTVYFTCRAEGNPK

272	PEIIWLRNN NELSMKTDSRLNL			LRR1	2.4
294	LDDGTLMIQ NTQEADEGVYQCMAKNVAGEA		LRR2	2.4

KTQEVT

330	LRYLGSPAR PTFVIQPQNTEVLV		LRR1	2
353	GESVTLECS ATGHPLPQITWTRGDRTPLPI		LRR2	2.2

DPRVNITPSGGLYIQNVAQSDSGEYTCFASNSVDSIHATAFIIVQALPQFTVTPQSRVVI
EGQTVDFQCAAKGHPQPVIAWTKGGSQLSVDRRHLVLSSGTLRISGVALHDQGQYECQAV
NIIGSQKVVAHLTVQPRVTPVFASIPSDMTVEVGTNVQLPCSSQGEPEPAITWNKDGVQV
TESGKFHI

571	SPEGFLTIN DVGTADAGRYECVARNTIGYA		LRR1	2.4
601	SVSMVLSVN VPDVSRNGDPYVATSIVEAIA		LRR2	2.4

TVDRAINSTRTHLFDSRPRSP

652	NDLLALFRY PRDPYTVGQARAGEIF		LRR1	2
677	ERTLQLIQE HVQHGLMVDLNGTSYHYNDLV		LRR2	2

SPQYLSLIANLSGCTAHRRVNNCSDMCFHQKYRTHDGTCNNLQHPMWGASLTAFERLLKA
VYENGFNTPRGINSQRQYNGHVLPMPRLVSTTLIGTEVITPDEQFTHMLMQWGQFLDHDL
DSTVVALSQARFSDGQHCSSVCSNDPPCFSVMIPPNDPRVRSGARCMFFVRSSPVCGSGM
TSLLMNSVYPREQINQ

903	LTSYIDASN VYGSTDHEARSIRDLASHRGL		LRR1	2
933	LRQGIVQRS GKPLLPFATGPPTECMRDENE		LRR2	2.2

SHIFT -17!

946	LPFATGPPT ECMRDENESPIPCFLAGDHRA		LRR1	2.2
976	NEQLGLTSM HTLWFREHNRIA			LRR2	2
997	AELLKLNPH WDGDTVYHETRKIVGAEIQHI		LRR3	2.4

TYRHWLPKILGEVGMKMLGEYRGYDPSVNAGIFNAFATAAFRFGHTLINPLLYRLDENFE
PIPQGHVPLHKAFFSPFRIVNEGGIDPLLRGLFGVAGKMRIPSQLLNTELTERLFSMAHT
VALDLAAINIQRGRDHGIPPYHDYRVYCNLSAAYTFEDLKNEIKSPVIREKLQR

1201	LYGSTLNID LFPALMVEDLVPGSRLGPT		LRR1	2
1229	LMCLLSTQF RRLRDGDRLWYENPGVFSPAQ		LRR2	2
1259	LTQLKQTSL ARILCDNSDNITRVQQDVFRV		LRR3	2

AEFPHGYSSCEDIPRVDLRVWQDCCEDCRTRGQFNAFSYHFRGRRSLEFSYEDDKPTKRA
RWRKALSVKHGKHLSNATSATHEHLEGPATNDLKEFVLEMQKIITDLRKQINSLESRLST
TECVDDSGESHGGNTKWKKDPCTVCECKNGQITCFVEACQPAACPQPVKVEGACCPVCLK
NTAEEKP

>ENSMUSP00000074360

MGAPGLRAATAALGLLLCAGLGRAGPAGSGGHGAPGQLLDDDAQRP

47	CPAACHCLGDLLDCSRRRLVRLPDPLPAW		NF	4

SHIFT -22!

54	LGDLLDCSR RRLVRLPDPLPAW			LRR1	2
76	VTRLDLSHN RLSFIQTSSLSHLQS		LRR2	3.4
100	LQEVKLNNN ELETIPNLGSISAN		LRR3	3.4
123	IRQLSLAGN AIDKILPEQLEAFQS		LRR4	3.4
147	LETLDLSNN NISELRTAFPPLQ			LRR5	4
169	LKYLYINNN RVSSMEPGYFDNLAST		LRR6	3.4
194	LLVLKLNRN RISAIPPKMFKLPQ		LRR7	4
217	LQHLELNRN KIKNVDGLTFQGLGA		LRR8	4
241	LKSLKMQRN GVTKLMDGAFWGLSN		LRR9	3.4
265	MEVLQLDHN NLTEITKGWLYGLLM		LRR10	3.4
289	LRELHLSQN AINRISPDAWEFCQK		LRR11	4
313	LSELDLTFN HLSRLDDSSFLGLSL		LRR12	4
337	LNALHIGNN KVSYIADCAFRGLTS		LRR13	3.4
361	LKTLDLRNN EISWTIEDMSGAFSGLDR		LRR14	4
388	LRQLILQGN RIRSITKKAFAGLDT		LRR15	4
412	LEHLDLSGN AIMSLQSNAFSQMKK		LRR16	4
436	LQQLHLNTS				LRR17	3.4
445	SLLCDCQLRWLPQWVAENNFQSLTKWICCLPLFTYAQSFGCCSSL		CF1	4

SHIFT -49!

441	LNTSSLLCD CQLRWLPQWVAENNFQSLTKW		LRR1	2
471	ICCLPLFTY AQSFGCCSSLRQLLYDFPKPQ		LRR2	2.4

ITVQPETQSAIKGSDVSFTCSAASSSDSPMTFAWKKDNEALQDAEMENYAHLRAQGGELM
EY

563	TTILRLRNV EFTSEGKYQCVISNHFGSSY		LRR1	2
592	SVKAKLTIN MLPSFTKTPMDLTIRAGAMAR		LRR2	2.4

LECAAVGHPAPQIAWQKDGGTDFPAARERRMHVMPEDDVFFIVDVKIEDIGVYSCTAQNS
AGSVSANATLTVLETPSFLRPLLDRTVTKGETAVLQCIAGGSPPPRLNWTKDDSPLVVTE
RHFFAAGNQLLIIVDSDVSDAGKYTCEMSNTLGTERGNVRLSVIPTPTCDSPHMTAPSLD
GDGWATVGVVIIAVVCCVVGTSLVWVVIIYHTRRRNEDCSITNTDETNLPADIPSYLSSQ
GTLADRQDGYISSESGSHHQFVTSSGGGFFLPQHDGAGTCHFDDSSEADVEAASDPFLCP
FVGSTGPVYLQGNLYSPDPFEVYLPGCSSDPRTALMDHCESSYVKQDRFSCARPSEEPCE
RS

984	LKSIPWPHS RKLTDSTYPPNEGHT		LRR1	2.2
1008	VQTLCLNKS SVDFSTGPEPGSATSSNSFMG		LRR2	2.8

TFGKP

1043	LRRPHLDAF SSSAQPPDCQPRPCHGKS		LRR1	2
1070	LSSPELDSE SEENDKERTDFREENHRCTYQ		LRR2	2

QIFHTYRTPDCQPCDSDT

>ENSMUSP00000020091

MATPNCLILWVLLIADTVWTQSVRQAYEIQDPEDWDVHDDFY

43	CPRECFCPPSFPTALYCENRGLTEIPPIPSR		NF	4
74	IWYLYLENN LIESIPEKPFENATQ		LRR1	3.4
98	LRWINLNKN KITNYGIEKGALSQLKK		LRR2	3.4
124	LLFLFLEDN ELEEVPSPLPRS			LRR3	4
145	LEQLQLARN KVSRIPQGTFSNLEN		LRR4	4
169	LTLLDLQHN KLLDNAFQRDTFKGLKN		LRR5	4
195	LMQLNMAKN ALRNMPPRLPAN			LRR6	3.4
216	TMQLFLDNN SIEGIPENYFNVIPK		LRR7	3
240	VAFLRLNHN KLSDAGLPSRGFDVSS		LRR8	3.4
265	ILDLQLSYN QLTNFPRINANLQHLHLDHNK		LRR9	3.4

IKNVNMSVICPTTLRAEQDAFIHGPQLSYLRLDGNEIKPPIPIDLVACFKSSGFHNIRQN
PNWFKGRCMWYEILIVTSRLDHTCNCSYPPFSYVQLVLSV

>ENSMUSP00000060793

MTKPNSLIFYCII

14	VLGLTLMKI QLSEECELIIKRPNANLTRV		LRR1	2.4
43	PKDLPLQTT TLDLSQNNISELQTSDILS		LRR2	2.4
71	LSKLRVLIM SYNRLQYLNISVFKFNTE		LRR3	2.4
98	LEYLDLSHN ELKVILCHPTVS			LRR4	4
119	LKHLDLSFN AFDALPICKEFGNMSQ		LRR5	4
144	LQFLGLSGS RVQSSSVQLIAHLNIS		LRR6	3.4
169	KVLLVLGDA YGEKEDPESLRHVSTETLHIV		LRR7	2

FPSKREFRF

208	LLDVSVSTT IGLELSNIKCVLEDQGCSYF		LRR1	2.2
237	LRALSKLGK NLKLSNLTLNNVETTWNSFIN		LRR2	2

ILQIVWHTPVKYFSISNVKLQGQLAFRMFNYSDTSLKALSIHQVVTDVFSFPQSYIYSIF
ANMNIQNFTMSGTHMVHMLCPSQVSP

353	FLHVDFTDN LLTDMVFKDCRNLVR		LRR1	2.2
377	LKTLSLQKN QLKNLENIILTSAKMTS		LRR2	4
403	LQKLDISQN SLRYSDGGIPCAWTQS		LRR3	3.4
428	LLVLNLSSN MLTGSVFRCLPPK			LRR4	4
450	VKVLDLHNN RIMSIPKDVTHLQA		LRR5	3.4
473	LQELNVASN SLTDLPGCGAFSS			LRR6	3.4
495	LSVLVIDHN SVSHPSEDFFQSCQN		LRR7	3.4
519	IRSLTAGNN				LRR8	2.8
528	PFQCTCELRDFVKNIGWVAREVVEGWPDSYRCDYPESSRGTALRDFHMSPLSCD		CF1_short	3

TVLLTVTIGATMLVLAVT

600	GAFLCLYFD LPWYVRMLCQWTQTRHRARH		LRR1	2.4
629	IPLEELQRN LQFHAFVSYSGHDSA		LRR2	2.4
653	WVKNELLPN LEKDDIQICLHERNFVPGKSI		LRR3	2.4

VENIINFIEKSYKSIFVLSPHFIQSEWCHYELYFAHHNLFHEG

726	SDNLILILL APIPQYSIPTNYHK		LRR1	2
749	LKTLMSRRT YLEWPTEKNKHG			LRR2	2.4
770	LFWANLRAS INVKLVNQAEGTCYTQQ		LRR3	2.8


>ENSMUSP00000021822

METVHSTFLLLLFVPLTQQAPQSQLDSHVNYEYATGNSEETKFSQDYEDKYLDGKSIKEK
ETMIIPD

68	EKSLQLQKD EVIPSLPTKKENDEMPT		LRR1	2
94	CLLCVCLSGSVYCEEVDIDAVPPLPKE		NF	4

SHIFT -26!

95	LLCVCLSGS VYCEEVDIDAVPPLPKE		LRR2	2.8
121	SAYLYARFN KIKKLTAKDFADMPN		LRR3	2.4
145	LRRLDFTGN LIEDIEDGTFSKLSL		LRR4	3.4
169	LEELTLAEN QLLRLPVLPPKLTLLNAKHNK		LRR5	4

SHIFT -17!

182	LPVLPPKLT LLNAKHNKIKSKGIKANTFKK		LRR1	2.4
212	LNKLSFLYL DHNDLESVPPNLPES		LRR2	2.4
236	LRVIHLQFN SISSLTDDTFCKANDTRYIRE		LRR3	3.4

R

267	IEEIRLEGN PIALGKHPNSFIC			LRR1	2.8
289	LKRLPIGSY F			LRR2	2.4


>BC043099

MEGVG

6	AVRFWLVVC GCLAFPPRAESV		LRR1	2.2
27	CPERCDCQHPQHLLCTNRGLRAVPKTSSLP		NF	4

SHIFT -22!

35	HPQHLLCTN RGLRAVPKTSSLPSPQD		LRR2	2
61	VLTYSLGGN FITNITAFDFHRLGQ		LRR3	2.4
85	LRRLDLQYN QIRSLHPKTFEKLSR		LRR4	4
109	LEELYLGNN LLQALVPGTLAPLRK		LRR5	4
133	LRILYANGN EIGRLSRGSFEGLES		LRR6	3.4
157	LVKLRLDGN VLGALPDAVFAPLGN		LRR7	4
181	LLYLHLESN RIRFLGKNAFSQLGK		LRR8	4
205	LRFLNLSAN ELQPSLRHAATFVPLRS		LRR9	4
231	LSTLILSAN SLQHLGPRVFQHLPR		LRR10	4
255	LGLLSLSGN QLTHLAPEAFWGLEA		LRR11	4
279	LRELRLEGN RLNQLPLTLLEPLHS		LRR12	4
303	LEALDLSGN ELSALHPATFGHQGR		LRR13	4
327	LRELSLRDN ALSALSGDIFAASPA		LRR14	4
351	LYRLDLDGN				LRR15	4
360	GWTCDCRLRGLKRWMGNWHSQGRLLTVFVQCRHPPALRGKYLDYLDDQLLQNG		CF1_short	3

SHIFT -46!

367	LRGLKRWMG NWHSQGRLLTVFVQCRHPPA		LRR1	2
396	LRGKYLDYL DDQLLQNGSCVDPSPSPTAGS		LRR2	2

RQWPLPTSSEEGMTPPAGL

445	SQELPLQPQ PQPQQRGRLLPGVAWG		LRR1	2
470	GAAKELVGN RSALRLSRRGPGPHQGPSAAA		LRR2	2.4

SHIFT -21!

479	RSALRLSRR GPGPHQGPSAAAPGSA		LRR1	2
504	PQSLDLHEK PGRGRHTRANLSQTEPTPTSE		LRR2	2

PASGTPSARDSWQRAAKQRLASEQQESAVQSVSGVGLPPLVSDPCDFNKFILCNLTVEAV
SANSASVRWAVREHRSPRPQGGARFRLLFDRFGQQPKFQRFVYLPERSDSATLHELRGDT
PYLVCVEGVLGGRVCPVAPRDHCAG

679	LVTLPEAGG RGGVDYQLLTLVLLAVNAL		LRR1	2
707	LVLLALAAW GSRWLRRKLRARRKGGAPVHV		LRR2	3

RHMYSTRRPLRSMGTGVSADFSGFQSHRPRTTVCALSEADLIEFPCDRFMDSTGGGTSGS
LRREDHLLQRFAD

>ENSMUSP00000049686

MGSIRTRLLWLCLLMLLALLHKSGSQDLTCMVHPSRVDWTQTFNGTC

48	LNFSGLGLS LPRSPLQASHAQVLDLSKNG		LRR1	2.4
77	LQVLPGAFF DKLEKLQTLIVTHNQLDSV		LRR2	2.4
105	DRSLALRCD LELKADCSCGLASWYALRQNC		LRR3	2

SHIFT -24!

111	RCDLELKAD CSCGLASWYALRQNCSGQ		LRR1	2
138	QQLLCLHPA TEAPRNLSTFLQVSCPPSWGP		LRR2	2

GTIGALVAGTISLAVAVSGSVLAWRLLRRRRRASEHSLSKAQMSPHDIPKPVTDFLPRYS
SRRPGPKAPDSPPSRFTMDYENVFIGQPAEDCSWSAARNSPSGDSDCYMNYRSVDQDSQP
VYCNLESLGR

>ENSMUSP00000084423

MRDFYVRVTIL

12	VTGLCFVET VTTPSRKSSVSFNPEY		LRR1	2.2
37	QRNGDLLVN WSSIRHVSQNTDAMDRSFYFF		LRR2	2.4

RVLFQPHTQKERHIKPPDRTHHRISKVTLDPLAHLHALEILNLSNKAIHYFSLDQPLPPS
SHQKRHGGHSHSR

140	LPRLQVLIL QRNQLSGTPKGLWKLKS		LRR1	2.4
166	LRSLDLSFN RIVHIGLSDFHGCLQ		LRR2	4
190	LESIYLKSN KICTIHPKAFKGLKK		LRR3	3.4
214	LQVVDLRSN ALTTLVPIVTIALE		LRR4	3.4
237	LPHLELGLA DNQWQCSESNVNFQNITSSSW		LRR5	3

REIWKAICNMSVENKRPNAETHQIRKSR

295	DTHLLLSPP SDLKSLIQSKAERPQAGMDMH		LRR1	2
325	LSALGKEAK DGYGDLRGMWPQSPVELRDSQ		LRR2	2

DEQVTDRKDDKPPALELAICLSVFITFVVAFCLGAFARPYIDRLRQQRCSNKRPGSDNAY
SNKGFHGDIEGAQHMEYQGTDLHQT

440	THHLHLSEN QNPSWVAEPIPHSAV		LRR1	3
464	QSEQMLGSN GTDPGHQQSPEQLKDSNESRS		LRR2	2

GDSIVLPSGPVAHLALHGLPNADAHKAISPVQHHHDFLEEAHYDTVAQEYSLIDDVMDRS
SITGPLGTFPSSVESRRDDLHPSQPRDVVASFSKTLAHANTREAEGSMETGCPEPLGAMD
SQMGSSEERQVSNSIRELATQQPSFQGVDAEERLSHVYSEVLHNDPPSLRPRWGSGHYVI
PATGEPVERDAPFDPHYDLVTNYESDSDEGS

705	LFTLSSEGS EDTRSLAEEQASVENDGTSQP		LRR1	2.4
735	LPSRNLGEY KDSVTSAESVEDLTSQRIPEK		LRR2	2

CEAQEAHLRNTLISGPDSCVCETNQENDSSSLDPENRSTWPQ

807	LPGHKLSHH ETLGTYGDIEPQSEAVDWHYS		LRR1	2
837	LRDLESPNV DSSPSPPYSDEDLSGPEDRAR		LRR2	2

KRSKHW

>ENSMUSP00000020400

MD

3	FLLLGLCLH WLLRRPSGVVLCLLGAC		LRR1	2.4
29	FQMLPAAPS G			LRR2	2.2
39	CPGQCRCEGRLLYCEALNLTEAPHNLSGL		NF	4

SHIFT -18!

50	LYCEALNLT EAPHNLSGLLGLSLRYNS		LRR3	2.4
77	LSELRAGQF TGLMQLTWLYLDHNHICSVQG		LRR4	2.4

SHIFT -19!

88	LMQLTWLYL DHNHICSVQGDAFQKLRR		LRR1	2.4
115	VKELTLSSN QITELANTTFRPMPN		LRR2	3.4
139	LRSVDLSYN KLQALAPDLFHGLRK		LRR3	3.4
163	LTTLHMRAN AIQFVPVRIFQDCRS		LRR4	3.4
187	LKFLDIGYN QLKSLARNSFAGLFK		LRR5	3.4
211	LTELHLEHN DLIKVNFAHFPRLIS		LRR6	4
235	LHSLCLRRN KVAIVVSSLDWVWN		LRR7	4
258	LEKMDLSGN EIEYMEPHVFETVPY		LRR8	3.4
282	LQTLQLDSN RLTYIEPRILNSWKS		LRR9	4
306	LTSITLAGN LWDCGRNVCALASWLSNFQGR		LRR10	3.4

YDANLQCASPEYAQGEDVLDAVYAFHLCEDGAEPTSGHLLSVAVTNRSDLTPPESSATTL
VDGGEGHDGTFEPITVA

413	LPGGEHAEN AVQIHKVVTGTMALIFSFL		LRR1	2.4
441	IVVLVLYVS WKCFPASLRQLRQCFVTQRRK		LRR2	2.8

QKQKQTMHQMAAMSAQEYYVDYKPNHIEGALVIINEYGSCTCHQQPARECEV

>ENSMUSP00000033741

MCP

4	LWLLTLLLA LSQALPFEQKGFWDFT		LRR1	3
29	LDDGLLMMN DEEASGSDTTSGVPDLDSVTP		LRR2	3.4

TFSAM

64	CPFGCHCHLRVVQCSDLGLKTVPKEISP		NF	4
92	DTTLLDLQN NDISELRKDDFKGLQH		LRR1	2
117	LYALVLVNN KISKIHEKAFSPLRK		LRR2	4
141	LQKLYISKN HLVEIPPNLPSS			LRR3	3.4
162	LVELRIHDN RIRKVPKGVFSGLRN		LRR4	3.4
186	MNCIEMGGN PLENSGFEPGAFDG		LRR5	2.2
209	LKLNYLRIS EAKLTGIPKDLPET		LRR6	2.4
232	LNELHLDHN KIQAIELEDLLRYSK		LRR7	4
256	LYRLGLGHN QIRMIENGSLSFLPT		LRR8	4
280	LRELHLDNN KLSRVPAGLPDLKL		LRR9	4
303	LQVVYLHSN NITKVGINDFCPMGFGVKRAY		LRR10	3.4
333	YNGISLFNN PVPYWEVQPATFRCVTDRLAI		LRR11	2.4

QFGNYKK

>ENSMUSP00000037909

MDVSLCPAKCSFWRIFLLGSVWLDYV

27	GSVLACPAN CVCSKTEINCRRPDDGNLFP		LRR1	2.4
56	LLEGQDSGN SNGNASINITDISRNITSIHI		LRR2	2.4

ENWRGLHTLNAVDMELYTG

105	LQKLTIKNS GLRNIQPRAFAKNPH		LRR1	2.8
129	LRYINLSSN RLTTLSWQLFQTLS		LRR2	3.4
152	LRELRLEQN				LRR3	4
161	FFNCSCDIRWMQLWQEQGEARLDSQSLYCISADGSQLPLFRMNISQCDLP		CF1	4

SHIFT -59!

152	LRELRLEQN FFNCSCDIRWMQLWQEQGEAR		LRR3	4
182	LDSQSLYCI SADGSQLPLFRMNISQCDLPE		LRR4	2

SHIFT -18!

194	GSQLPLFRM NISQCDLPEISVSHVNLTVRE		LRR1	2.4
224	GDNAVITCN GSGSPLPDVDWIVTGLQSINT		LRR2	2.2

SHIFT -16!

238	LPDVDWIVT GLQSINTHQTNLNWTNVH		LRR1	2.2
265	AINLTLVNV TSEDNGFTLTCI			LRR2	2.4
286	AENVVGMSN ASVALTVYYPPRVVSLVEPEV		LRR3	2.2

RLEHCIEFVVRGNP

330	TPTLHWLYN GQPLRESKIIHMDYYQEGE		LRR1	2.4
358	VSEGCLLFN KPTHYNNGNYTLIAKNA		LRR2	2.8
384	LGTANQTIN GHFLKEPFPESTDFFDFESDA		LRR3	2.4

SPTPPITVTHKPEEDTFGVSIAVGLAAFACVLLVVLFIMINKYGRRSKFGMKGPVAVISG
EEDSASPLHHINHGITTPSSLDAGPDTVVIGMTRIPVIENPQYFRQGHNCHKPDTYVQHI
KRRDIVLKRELGEGA

549	FGKVFLAEC YNLSPTKDKMLVAVKA		LRR1	2.2
574	LKDPTLAAR KDFQREAELLTNLQHEHIVKF		LRR2	2

YGVCGDGDPLIMVFEYMKHGDLNKFLRAHGPDAMILVDGQPRQAKGELGLSQMLHIASQI
ASGMVYLASQHFVHRDLATRNCLVGANLLVKIGDFGMSRDVYSTDYYRVGGHTMLPIRWM
PPESIMYRKFTTESDVWSFGVILWEIFTYGKQPWFQLSNTEVIECITQGRVLERPRVCPK
EVYDVMLGCWQREPQQRLNIKEIYKILHALGKATPIYLDILG

>ENSMUSP00000027706

MRLLVAALLLSWVAGTTAAAPVVPWRVP

29	CPPQCACQIRPWYTPRSSYREATTVDCNDLFLTAVPPRLPAG		NF	4
71	TQTLLLQSN SISRIDQTELAYLAN		LRR1	3
95	LTELDLSQN SFSDARDCDFQALPQ		LRR2	4
119	LLSLHLEEN RLNRLEDHSFAGLTS		LRR3	4
143	LQELYLNHN QLCRISPRAFAGLGN		LRR4	4
167	LLRLHLNSN LLRTIDSRWFEMLPN		LRR5	4
191	LEILMIGGN KVDAILDMNFRPLAN		LRR6	3.4
215	LRSLVLAGM SLREISDYALEGLQS		LRR7	3
239	LESLSFYDN QLAQVPKRALEQVPG		LRR8	3.4
263	LKFLDLNKN PLQRVGPGDFANM			LRR9	4
285	LHLKELGLN NMEELVSIDKFALVNLPE		LRR10	3
312	LTKLDITNN PRLSFIHPRAFHHLPQ		LRR11	3.4
337	METLMLNNN ALSALHQQTVESLPN		LRR12	3.4
361	LQEVGLHGN				LRR13	3.4
370	PIRCDCVIRWANATGTHVRFIEPQSTLCAEPPDLQRRPVREVPFREMTDH		CF1_short	3

SHIFT -2!

418	DHCLPLISP RSFPSSLQIASGESTV		LRR1	2
443	LHCRALAEP EPEIYWVTPAGVRLTPARSGR		LRR2	2

RYRVFPEGTLELRRVTAEEAGLYTCV

499	AQNLVGADT KTVSVVVGHAPFQPGRDK		LRR1	2.2
526	GPGLKLHVQ ETHPYHILLFWAPPPNIVSTN		LRR2	2.4

SHIFT -14!

542	LLFWAPPPN IVSTNLTWSSASSLRDHEAPA		LRR1	2.4
572	LARLPRGTH RYNITRLLPATEYWACLQVAF		LRR2	2

ADAHTQLACVWARTKEASPCHRALGDRPGLIAILALAVLLLAAGLAAHLGRGQSKQGVVG
EKPLLPVWAFWGWSAPSVRVVSAPLVLPWNPGRKQPRCQMGRGCHHHCLNIAEVPPGLSS
REITRTTLH

>ENSMUSP00000043101

MSGLYRILVQLEQSPYVKTVPLNMRRDFFFLVVTWMPKTVKMNGSSFVPSLQLLLM

57	LVGFSLPPV AETYGFNKCTQYEFDIHHV		LRR1	2.4
85	LCIRKKITN LTEAISDIPRYTTHLNLTHNE		LRR2	2

SHIFT -10!

105	TTHLNLTHN EIQVLPPWSFTNLSA		LRR1	3
129	LVDLRLEWN SIWKIDEGAFRGLEN		LRR2	4
153	LTLLNLVEN KIQSVNNSFEGLSS		LRR3	4
176	LKTLLLSHN QITHIHKDAFTPLIK		LRR4	4
200	LKYLSLSRN NISDFSGILEAVQHLPC		LRR5	4
226	LERLDLTNN SIMYLDHSPRSLVS		LRR6	4
249	LTHLSFEGN KLRELNFSALSLPN		LRR7	3.4
272	LTNLSASRN GNKVIQNVYLKTLPQ		LRR8	3.4
296	LKSLNLSGT VIKLENLSAKHLQN		LRR9	3.4
319	LRAMDLSNW ELRHGHLDMKTVCHLLGNLPK		LRR10	2.4
349	LETLVFQKN VTNAEGIKQLAKCTR		LRR11	3.4
373	LLFLDLGQN SDLIYLNDSEFNALPS		LRR12	4
398	LQKLNLNKC QLSFINNRTWSSLQN		LRR13	3.4
422	LTSLDLSHN KFKSFPDFAFSPLKH		LRR14	4
446	LEFLSLSRN PITELNNLAFSGLFA		LRR15	4
470	LKELNLAAC WIVTIDRYSFTQFPN		LRR16	3.4
494	LEVLDLGDN NIRTLNHGTFRPLKK		LRR17	4
518	LQSLILSHN CLKILEPNSFSGLTN		LRR18	4
542	LRSLDLMYN SLSYFHEHLFSGLEK		LRR19	4
566	LLILKLGFN KITYETTRTLQYPPFIKLKS		LRR20	4
595	LKQLNLEGQ RHGIQVVPSNFFQGLGS		LRR21	3
621	LQELLLGKN PSVFLDHHQFDPLIN		LRR22	4
645	LTKLDISGT KDGDRSLYLNASLFQNLKR		LRR23	2.8
673	LKILRLENN NLESLVPDMFSSLQS		LRR24	4
697	LQVFSLRFN NLKVINQSHLKNLKS		LRR25	3.4
721	LMFFDVYGN				LRR26	2.8
730	KLQCTCDNLWFKNWSMNTEEVHIPFLRSYPCQQPGSQSLLIDFDDAMCNFD		CF1	4

LGKVYFLCSFSMVLSTMVFSWFSTKMIASLWYGLYICRAWYLTKWHKTEKKF

833	LYDAFVSFS ATDEAWVYKELVPALEQGSQT		LRR1	2.2
863	TFKLCLHQR DFEPGIDIFENIQNAINTS		LRR2	2
891	RKTLCVVSN HYLHSEWCRLEVQLASMKMFY		LRR3	2.4

EHKDVIILIFLEEIPNYKLSSYHRLRKLINKQTFITWPDSVHQQPLFWARIRNALGKETV
EKENTHLIVVE

>AAZ20639

MAPGPFSSGLFSPPPAA

18	LPFLLLLWA GASRGQPCPGRCICQNVAPT		LRR1	3
47	LTMLCAKTG LLFVPPAIDRRVVELRLTDNF		LRR2	2.4

SHIFT -10!

67	VVELRLTDN FIAAVRRRDFADMTS		LRR1	3.4
91	LVHLTLSRN TIGQVAAGAFADLRA		LRR2	4
115	LRALHLDSN RLAEVRGDQLRGLGN		LRR3	4
139	LRHLILGNN QIRKVESAAFDAFLST		LRR4	4
164	VEDLDLSYN NLEALPWEAVGQMVN		LRR5	3.4
188	LNTLTLDHN LIDHIAEGTFVQLHK		LRR6	4
212	LVRLDMTSN RLHKLPPDGLFLRSQGGGPKP		LRR7	3.4

SHIFT -20!

222	LHKLPPDGL FLRSQGGGPKPPTPLTVS		LRR1	2
249	FGGNPLHCN CELLWLRRLTREDDLETCATP		LRR2	2.4

EHLTDRYFWSIPEEEFLCEPPLITRQAGGRALVVEGQAVSLRCRAVGDPEPVVHWVAPDG
RLLGNSSRTRVRGDGTLDVTITTLRDSGTFTCIASNAAGEATAPVEVCVVPLPLMAPPPA
APPPLTEPGSSDIATPGRPGANDSTSERRLVAAELTSSSVLIRWPAQRPVPGIRMYQVQY
NSSADDSLVYRMIPSTSQTFLVNDLAAGRAYDLCVLAVYDDGATALPATRVVGCVQFTTA
GDPAPCRP

527	LRAHFLGGT MIIAIGGVIVASV			LRR1	2.4
549	LVFIVLLMI RYKVYGDGDSRRIKGTSRTPP		LRR2	2.4

RVSHVCSQTNGAGAQQASAPPAPDRYEALREVAVPAAIEAKAMEAEATSTELEVVLGRSL
GGSATSLCLLPSEETSGEESRAMTGPRRSRSGALGPPTSA

679	PPTLALVPG GAPARPRPQQRYSFD		LRR1	2
703	GDYGALFQS HSYPRRARRTKRHRSTPHLDG		LRR2	2.2

AGGGAAGEDGDLGLGSARARLAFTSTEWMLESTV

>BC030471

MLRKGCCVELLLLLLAGELPLGGG

25	CPRDCVCYPAPMTVSCQAHNFAAIPEGIPED		NF	4
56	SERIFLQNN RITFLQQGHFSPA			LRR1	2.4
78	MVTLWIYSN NITFIAPNTFEGFVH		LRR2	2.8
102	LEELDLGDN RQLRTLAPETFQGLVK		LRR3	4
127	LHALYLYKC GLSALPAGIFGGLHS		LRR4	3.4
151	LQYLYLQDN HIEYLQDDIFVDLVN		LRR5	4
175	LSHLFLHGN KLWSLGQGIFRGLVN		LRR6	4
199	LDRLLLHEN QLQWVHHKAFHDLHR		LRR7	4
223	LTTLFLFNN SLTELQGDCLAPLVA		LRR8	4
247	LEFLRLNGN				LRR9	4
256	AWDCGCRARSLWEWLRRFRGSSSAVPCATPELRQGQDLKLLRVEDFRNC		CF1_short	3

SHIFT -58!

247	LEFLRLNGN AWDCGCRARSLWEW		LRR9	4
270	LRRFRGSSS AVPCATPELRQGQD		LRR10	2.2
293	LKLLRVEDF RNCTGPVSPHQIKSHTLTTSD		LRR11	2.4

RAARKEHHPSHGASRDKGHPHGHPPGSRSGYKKAGKNCTSHRNRNQISKVSSGKELTELQ
DYAPDYQHKFSFDIMPTARPKRKGKCARRTPIRAPSGVQQASSGTALGAPLLAWILGLAV
TLR

>AY182029

MGFNVIRLLRGSAVAVVLAPTVLLTMLSSAERG

34	CPKGCRCEGKMVYCESQKLQEIPSS		NF	4
59	ISAGCLGLS LRYNSLQKLKYNQFKGLNQ		LRR1	2.2
87	LTWLYLDHN HISNIDENAFNGIRR		LRR2	4
111	LKELILSSN RISYFLNNTFRPVTN		LRR3	4
135	LRNLDLSYN QLHSLGSEQFRGLRK		LRR4	4
159	LLSLHLRSN SLRTIPVRIFQDCRN		LRR5	4
183	LELLDLGYN RIRSLARNVFAGMIR		LRR6	4
207	LKELHLEHN QFSKLNLALFPRLVS		LRR7	4
231	LQNLYMQWN KISVIGQTMSWTWSS		LRR8	3.4
255	LQRLDLSGN EIEAFSGPSVFQCVPN		LRR9	4
280	LQRLNLDSN KLTFIGQEILDSWIS		LRR10	4
304	LNDISLAGN IWECSRNICSLVNWL		LRR11	3.4
328	RSFKGLREN TIICASPKELQG			LRR12	2
349	VNVIDAVKN YSICGKSTTTERFDLARALPK		LRR13	2.2

PTFKPKLPRPKHESKPPLPPTVGATEPSPETDVDTEHISFHKII

423	AGSVALFLS VLVILLVMYVSWKRYPASMKQ		LRR1	2.2
453	LQQRSLMRR HRKKKRQSLKQMTPGTQEFYV		LRR2	2

DYKPTNTETSEMLLNGTGPCTYSKSGSR

511	ECEIPLSMN VSTFLAYDQPTISYCGVHHEL		LRR1	2.4
541	LSHKSFETN AQEDTMESHLETELDLSTITS		LRR2	2.4

AGRISDHKPQLA

>ENSMUSP00000047573

MET

4	LLGGLLAFG MAFAVVDACPKYCVCQNLSES		LRR1	2.4
34	LGTLCPSKG LLFVPPDIDRRTVELRLGGNF		LRR2	2

SHIFT -10!

54	TVELRLGGN FIIHIGRQDFANMTG		LRR1	3
78	LVDLTLSRN TISHIQPFSFLDLES		LRR2	4
102	LRSLHLDSN RLPSLGEDTLRGLVN		LRR3	4
126	LQHLIVNNN QLGGIADDAFEDFLLT		LRR4	3.4
151	LEDLDLSYN NLHGLPWDSVRRMVN		LRR5	4
175	LHQLSLDHN LLDHIAEGTFADLQK		LRR6	4
199	LARLDLTSN RLQKLPPDPIFARSQASLLTA		LRR7	4

SHIFT -20!

209	LQKLPPDPI FARSQASLLTATPFAPPLSFS		LRR1	2
239	FGGNPLHCN CELLWLRRLERDDDLETCGSP		LRR2	2.4

GSLKGRYFWHIREEEFVCEPPLITQHT

296	HKLLVLEGQ AATLKCKAIGDPSPLIHWV		LRR1	2
324	APDDRLVGN SSRTAVYDNGTLDILITTSQD		LRR2	2.4

SGPFTCIAANAAGEATATVEVSIVQLPHLSNSTSRMAPPKSRLSDITGSSKTSRGGGGSG
AGEPPKSTPERAVLVSDVTTTSALVKWSVSKSAPRVKMYQLQYNCSDDEVLIYRMIPASN
KAFVVNNLVSGTGYDLCVLAMWDDTATTLTATNIVGCAQFFTKADYPQCQSMHSQILGGT
MILVIGGIIVATLLVFIVILMVRYKVCNHDTPGKMAAATVSNVYSQTNGSQPPPLGGIPV
GQLPQAPPKVVVRNELMDFSTSLARACDSSSSSSLGSGEAAGLGRGPWRLPPPAPRPKPS
LDRLMGAFASLDLKSQRKEELLDSRTPAGRGAGTSSRGHHSDREPLLGPPATRARSLLPL
PLEGKAKRSHSFDMGDFAAAAAAVPGGYSPPRRVSNIWTKRSLSVNGMLLPFEESDLVGA
RGTFGSSEWVMESTV

>ENSMUSP00000034056

MKGCSSYLMYSF

13	GGLLSLWIL LVSSTNQCTVRYNVAD		LRR1	2.4
38	CSHLKLTHI PDDLPSNITVLN			LRR2	2
59	LTHNQLRRL PPTNFTRYSQLAILDAGFNSI		LRR3	2

SHIFT -11!

78	LAILDAGFN SISKLEPELCQILPL		LRR1	3.4
102	LKVLNLQHN ELSQISDQTFVFCTN		LRR2	4
126	LTELDLMSN SIHKIKSNPFKNQKN		LRR3	4
150	LIKLDLSHN GLSSTKLGTGVQLEN		LRR4	4
174	LQELLLAKN KILALRSEELEFLGNSS		LRR5	4
200	LRKLDLSSN PLKEFSPGCFQTIGK		LRR6	4
224	LFALLLNNA QLNPHLTEKLCWELSNTS		LRR7	3
251	IQNLSLANN QLLATSESTFSGLKWTN		LRR8	3.4
277	LTQLDLSYN NLHDVGNGSFSYLPS		LRR9	4
301	LRYLSLEYN NIQRLSPRSFYGLSN		LRR10	4
325	LRYLSLKRA FTKQSVSLASHPNIDDFSFQW		LRR11	3
355	LKYLEYLNM DDNNIPSTKSNTFTGLVS		LRR12	2
382	LKYLSLSKT FTSLQTLTNETFVSLAHSP		LRR13	3.4
410	LLTLNLTKN HISKIANGTFSWLGQ		LRR14	4
434	LRILDLGLN EIEQKLSGQEWRGLRN		LRR15	4
459	IFEIYLSYN KYLQLSTSSFALVPS		LRR16	2.8
483	LQRLMLRRV ALKNVDISPSPFRPLRN		LRR17	3
509	LTILDLSNN NIANINEDLLEGLEN		LRR18	4
533	LEILDFQHN NLARLWKRANPGGPVNFLKG		LRR19	3.4
562	LSHLHILNL ESNGLDEIPVGVFKNLFE		LRR20	2.4
589	LKSINLGLN NLNKLEPFIFDDQTS		LRR21	3.4
613	LRSLNLQKN LITSVEKDVFGPPFQN		LRR22	4
638	LNSLDMRFN				LRR23	3.4
647	PFDCTCESISWFVNWINQTHTNISELSTHYLCNTPHHYYGFPLKLFDTSSCKDS		CF1	4

SHIFT -15!

686	GFPLKLFDT SSCKDSAPFELLFIISTSM		LRR1	2.8
714	LLVFILVVL LIHIEGWRISFYWNVSVHRIL		LRR2	2.4

GFKEIDTQAEQFEYTAYIIHAHKDRDWVWEHFSPMEEQDQSLKFCLEERDFE

796	AGVLGLEAI VNSIKRSRKIIFVITHH		LRR1	2.4
822	LLKDPLCRR FKVHHAVQQAIEQN		LRR2	2
845	LDSIILIFL QNIPDYKLNHALCLRRGMFKS		LRR3	2.4

HCILNWPVQKERINAFHHKLQVALGSRNSAH

>ENSMUSP00000052055

MIPVAEFKQFTEQQPAFKVLKPWWDVLAEY

31	LTVAMLMIG VFGCTLQVTQDKIIC		LRR1	2.4
55	LPSHESREN ISGAPCQQLLPQGISEQMGG		LRR2	2
84	LRELSGLKN NLDLQQYSFINQLCYETALHW		LRR3	3.4

SHIFT -23!

91	KNNLDLQQY SFINQLCYETALHWYAKY		LRR1	2
118	FPYLVVIHT LIFMVCTSFWFKFPGTSSKIE		LRR2	2.2

HFISILGKCFDSPWTTRALSEVSGENHKGPASGRAMVTTVTTTGAGSGKVGEGEKEKVLI
EPEKVVSEPPVVTLLDKKEGEQAKALFEKVKKFRVHVEEGDILYSMYIRQTVLKVCKFFA
ILVYNLIYVEKISFLVACRVETSEITGYASFCCNHTKAHLFSKLAFCYISFVCVYGITCL
YTLYW

333	LFHRPLKEY SFRSVREETGMNDIPDVKNDF		LRR1	2
363	AFMLHLIDQ YDSLYSKRFAVFLSEVSESR		LRR2	2.4
392	LKQLNLNHE WTPEKLRQKLQRNM		LRR3	3
415	RGRLELSLC MLPGLPDTVFELSE		LRR4	2.4
438	VEALRLEAI CDISFPPGLSQLVN		LRR5	2.4
461	LQELSLLHS PARLPFSSQIFLRDRLKVICV		LRR6	3.4

KFEELREVP

500	LWVFGLRGL EELHLEGLFPPEMARGAT		LRR1	2.4
527	LESLRELKQ LKVLSLRSNAGKVPASVTDVA		LRR2	2

SHIFT -27!

530	LRELKQLKV LSLRSNAGKVPASVTDVAGH		LRR1	2
559	LQRLSLHND GARLLALNSLKKLAV		LRR2	3
583	LRELELVAC GLERIPHAIFSLGA		LRR3	3.4
606	LQELDLKDN HLRSIEEILSFQHCRK		LRR4	4
631	LVTLRLWHN QIAYVPEHVRKLRS		LRR5	4
654	LEQLYLSHN KLETLPTQLGQCFG		LRR6	4
677	LRLLDLSHN GLRSLPPELGLLQS		LRR7	4
700	LQHLALSYN ALESLPDELFFCHK		LRR8	4
723	LRTLLLGYN HLTQLSPDVAALQA		LRR9	4
746	LSRLELKGN RLETLPEELGDCKG		LRR10	4
769	LKKSGLLVE DTLYEGLPAEVREKMEEE		LRR11	2


>ENSMUSP00000049296

MPLGAPALLALALGLGLWLGALAGDPGRG

30	CGPCPLPCFCGPAPDAACRVNCSGRWLQTLGPSLRIP		NF	4

SHIFT -11!

56	LQTLGPSLR IPADATALDLSHNL		LRR1	2
79	LQTLDIGLL VNLSALVELDLSNNRISTLEE		LRR2	2.4

SHIFT -22!

87	LVNLSALVE LDLSNNRISTLEEGVFANLFN		LRR1	2.4
117	LSEINLSGN				LRR2	3.4
126	PFECNCGLAWLPRWAKEHQVHVVQSEATTCRGPIPLAGQPLLSIPLLDNACG		CF1_short	3

SHIFT -45!

133	LAWLPRWAK EHQVHVVQSEATTCRGPIP		LRR1	2
161	LAGQPLLSI PLLDNACGEEYVACLPDNSSG		LRR2	2

AVAAVPFYFAHEGPLETEACSAFCFSAGEG

221	LAALSEQNQ CLCGAGQASNSSAACSSW		LRR1	2
248	CSSISLSLN SACGGPTLLQHTFPASPGATL		LRR2	2.4

VGPHGPLASGQPADFHITSSLPISSTRWNFGDGSPEVDMASPAATHFYVLPGSYHM

334	TVVLALGAG SALLETEVQVEAT			LRR1	2
356	PTVLELVCP SFVHSNESLELGIRHRG		LRR2	2
382	GSALEVTYS ILALDKEPAQVVHPLCP		LRR3	2.2
408	LDTEIFPGN GHCYRLVAEKAPWLQAQEQCR		LRR4	2.4

TWA

441	GAALAMVDS PAIQHFLVSKVTRS		LRR1	2.2
464	LDVWIGFSS VEGTEGLDPRGE			LRR2	2.2
485	AFSLESCQN WLPGEPHPATAEHCVRLGPAG		LRR3	2.4

QCNTDLCSAPHSYVCELRPGGPVWDTENFVMGMSGGGLSGPLHPLAQQETVQGPLRPVEV
MVFPGLSPSREAFLTAAEFSTQKLEEPAQMRLQVYRPSGGAAAVPEGSSEPDNRTEPAPK
CVPEELWCPGANVCIPFDASCNSHVCINGS

665	VSRLGLSRA SYTLWKEFFFSVPAGPPTQY		LRR1	2.4
694	LVTLHSQDV PMLPGDLIGLQHDAGPGT		LRR2	2
721	LLQCPLASS CPGQALYLSTNASDWMTNLPV		LRR3	2.4

SHIFT -19!

732	GQALYLSTN ASDWMTNLPVHLEEAWAGP		LRR1	3.4
760	VCSLQLLLV TERLTPLLGLGPNPGLQHPGH		LRR2	2.4

YEVRATVGNSVSRQNLSCSFSVVSPIAGLRVIHPIPLDGHIYVPTNGSVLVLQVDSGANA
TATAQWFGGNISAPFEDACPPEVDFLKQDCTEEANGTL

888	FSVLMLPRL KEGDHTVEIVAQNGAS		LRR1	2.4
913	QANLSLRVT AEEPICGLRAVPSPEARV		LRR2	2.4
940	LQGILVRYS PMVEAGSDVAFRWTIDDKQS		LRR3	2.2
969	LTFHNTVFN VIYQSAAIFKLSLTASNH		LRR4	2
996	VSNITVNYN VTVERMNKMHGLWVS		LRR5	2.2
1020	AVPTVLPPN ATLALTGGVLVDS			LRR6	2.4
1042	AVEVAFLWN FGDGEQVLRQFKPPYDESFQV		LRR7	2.2

PDPT

1076	VAQVLVEHN TTHIYTTPGEYN			LRR1	2.2
1097	LTVLVSNTY ENLTQQVTVSVRTV		LRR2	2
1120	LPNVAIGMS SNVLVAGQPITFSPYPLPSTD		LRR3	2.2

GVLYTWDFGDGSPVLIQSQPVLNHTYSMTG

1180	AYRITLEVN NTVSSVTAHADIRVFQE		LRR1	2.8
1206	LHGLTVYLS PSVEQGAPMVVSASVESGDNI		LRR2	2.8

TWTFDMGDGTVFTGPEATVQHVYLRAQNFTVTVEAANPAGHLSQSLHVQVFVLEVLHIEP
STCIPTQPSAQLMAHVTGDPVHYLFD

1322	WTFGDGSSN VTVHGHPSVTHNFTRSG		LRR1	2.2
1348	IFPLALVLS SHVNKAHYFTSICVEPEIRNI		LRR2	2.8

TLQPERQFVKLGDEARLVAYSWPPFPYRYTWDFGTEDTTHTQTGGSEVKFIYREPGSYLV
IVTVSNNISSTNDSAFVEVQEPVLVTGIRING

1470	SHVLELQQP YLLSAMGSGSPATY		LRR1	2
1493	LWELGDGSQ SEGPEVTHIYSSTGDFTVRVS		LRR2	2

GWNEVSRSEAQLNITVKQRVRGLTINASRTVVPLNGSVSFSTLLEVGSDVHYSWVLCDRC
TPIPGGPTISYTFRSVGTFNIIVTAENEVGSAQDSIFIYVLQFIEGLQVAGGDNGCCFPT

1643	NYTLQLQAA VRDGTNISYSWTAQQEGS		LRR1	2
1670	LITLFGSGK CFSLTSLKASTYYVHLRATNM		LRR2	2.4
1700	LGSAAANRT IDFVEPVESLILSASPNPA		LRR3	2.2
1728	AVNMSLTLC AELAGGSGVVYTWYLEEGLSW		LRR4	2.2

KTSMPSTTHTFA

1770	APGLHLVRV TAENQLGSVNATVEVAIQVP		LRR1	2.4
1799	VGGLSIRTS EPDSIFVAAGSTLPF		LRR2	2.2
1823	WGQLAEGTN VTWCWTLPGGSKDSQYIAVRF		LRR3	2.4

ST

1855	AGSFSLQLN ASNAVSWVSAMYNLTVEEP		LRR1	2.8
1883	IVNLMLWAS SKVVAPGQPVHFEILLAAGSA		LRR2	2.8

LTFRLQVGGSVPEVLPSPHFSHSFFRVGDHLVNVQAENHVSHAQAQVRILVLEAVVGLQV
PNCCEPGMATGTEKNFTARVQRGSRVAYAWYFSLQKVQGDS

2014	LVILSGRDV TYTPVAAGLLEIHVRAFNE		LRR1	2.4
2042	LGGVNLTLM VEVQDIIQYVTLQSGRCFTNR		LRR2	2.4

SARFEAATSPSPRRVTYHWDFGDGTPVQKTEEFWADHYYLRPGDYHVEVNATNLVSFFVA
QATVTVQVLACREPEVEVALPLQVLMRRSQRNYLEAHVDLRNCVSYQTEYRWEIYRTASC
QRPGRMAQMVLPGVDVSRPQLVVPRLALPVGHYCFVFVVSFGDTPLARSIQANVTVAAER
LVPIIEGGSYRVWSDTQDLVLDGSKSYDPNLEDGDQTPLNFHWACVASTQSETGGCVLNF
GPRGSSVVTIPLERLEAGVEYTFNLIVWKAGRKEEATNQTVLIRSGRVPIVSLECVSCKA
QAVYEVSRSSYVYLEGHCHNCSRGYKQGCWAARTFSNKTLVLNETTTSTGSTGMNLVVRP
GALRDGEGYIFT

2444	LTVLGHSGE EEGCASIRLSPNRPPLGGSCR		LRR1	2
2474	LFPLDSVRG LTTKVHFECTGWRDAEDGGAP		LRR2	2
2504	LVYALLLKR CRQSYCENFCIYKGSLSTYGA		LRR3	2.4

VLPPGFQPLFVVSLAVVVQDQ

2555	LGAAVVALN RSLTIVLPEPSGNPADLVPW		LRR1	2.8
2584	LHSLTASVL PGLLKQADPQHVI			LRR2	2.4
2606	EYSLALITV LNEYEQAPDVSEPNVEQQLRA		LRR3	2

QMRKNITETLISLRVNTVDDIQQITAALAQCMVSSRELMCRSCLKKMLQKLEGM

2690	MRILQAETT EGTLTPTTIADSILNITGD		LRR1	2.2
2718	LIHLASLDM QGPQPLELGVEPPS		LRR2	2
2741	LMVASKAYN LSSALMRILMRSRV		LRR3	2.4
2764	LNEEPLTLA GEEIVALGKRSDP			LRR4	2
2786	LSLLCYGKA LGPSCHFSIPEAFSGA		LRR5	2
2811	LSNLSDVVQ LIFLVDSNPFPFGYISNYTVS		LRR6	2

TKVASMAFQTQTGTQIPIEQLAAERAITVKVPNNSDQAAQSSHNPVGSTIVQPQTSVSAV
VTADNSNPQAGLHLRITYTVLNERYLSAEPEPYLAVYLHSVSQPNEYNCSASRRIS

2957	LEVLEGADH RLYTFFIAPGTGTLDR		LRR1	2.4
2982	SYYLNLTSH FHWSALEVSVGLYTSLCQYFS		LRR2	2

EEMMMWRTEGIVPLEETSPSQAVCLTRHLTAF

3044	GASLFVPPS HVQFIFPEPSAS			LRR1	2.2
3065	INYIVLLTC VICLVTYVVMAMILRK		LRR2	2.2
3090	LDQLDVSRV RVIPFCGKGGRFKYEILVKTG		LRR3	2.4

WSRGSGTTAHVGIMLYGEDNRSGHRHLDGDRAFHRNSLDIFQIATPHSLGSVWKIRVWHD
NKGLSPAWFLQHIIVRDLQSARSTFFLVNDWLSVETEANGGLVEKEVLAANEAALWQFQR
LLVAELQRGFFDKHIWLSIWDRPPRSRFTRVQRVTCCVLLLCLFLAANAVWYGVVRDTTY
SMGPVSSLISPGVDTVAIGLVSSVVVYPVYLAV

3333	LFLFRMSRS KVSGDQNPTPTGQQALDVDSY		LRR1	2.2
3363	LDPSVLDSS LLTLSGLTEAFAGQV		LRR2	2.4
3387	KNDLFLEDA KSLVCWPSSEGT			LRR3	2
3408	LSWPDLLSD PSVVSSTLQRLTQGRPGCMLG		LRR4	2

SHIFT -14!

3424	LQRLTQGRP GCMLGSEEDGASLVSPS		LRR1	2
3450	LPAKYLSAS DEDLIHQVLADGANNLVPTQD		LRR2	2.4

SHIFT -18!

3462	LIHQVLADG ANNLVPTQDTLLETDLLTS		LRR1	2
3490	LSSVPGEKT ETLILQTVGEERPASMGLSWE		LRR2	2.2

SHIFT -22!

3498	TETLILQTV GEERPASMGLSWEQSPVTR		LRR1	2
3526	LSRTGLVEG FQKRLLPAWCAPL			LRR2	2
3548	AHGLSLLLV AVAVAVSGWIGASFPPS		LRR3	2.4
3574	VSVMWLLSS SSSFLASFLGWEPLKVL		LRR4	2.2
3600	LEALYFSLV AKRLHPDEDDTLVESPAVTPV		LRR5	2.4

SERVPRVRPPH

3641	GFALFLAKE EARKVKRLHDMLKRLL		LRR1	2.4
3666	VYMLFLLVT LLANYGDASCHGHAYRLQSAI		LRR2	2.8

KQELDSQAFLAITRSDEFWPWMSHVFLPYVHGNQSSPELGPPRLRQVRLQEAFCPDPSSS
EHMCSAAGSLSTSDYGIGWQSVVQNGSETWAYSAPDLLGAWYWGYCAVYDSGGYIQELGL
SLEESRAR

3824	LGFLQLHNW LDSRSRAVFVELTRYSP		LRR1	3
3850	AVGLHAAVT LRLEFPVAGHALAAFSVRPFA		LRR2	2.2
3880	LRRLSTGLS LPLLTSVCLLLFALYFSMAEV		LRR3	2.4

SHIFT -13!

3897	LLLFALYFS MAEVQTWRKDGCACTARPDTW		LRR1	2.8
3927	ARCLLVILT AATGLVRLAQLGIADRQWTHF		LRR2	2.2

VQDHPRHFTSFDQVAQLGSVARGLAAS

3984	LLFLLLVKA AQQLRFVRQWSVFGKTLCRA		LRR1	3
4013	LPELMGATL GLVLLGVAYAQMAILLISSGA		LRR2	2.4

SHIFT -25!

4018	GATLGLVLL GVAYAQMAILLISSGADTLYN		LRR1	2.4
4048	MARAFLVLC PGARVPTLCPSESWYLSP		LRR2	2.2
4075	LLCVGLWAL RVWGALRLGAILLRWRYHALR		LRR3	2.4

SHIFT -14!

4091	LGAILLRWR YHALRGELYRPAWEPQDYE		LRR1	2.4
4119	MVELFLRRL RLWMGFSKVKEFRHKVRFEGM		LRR2	2.4

DPLPSRSSRGSKSSPVVLPPSSGSEASHPSTSSSQPD

4186	GPSASLSRS TLKLEPEPSRLHAVFES		LRR1	2.2
4212	LLVQFDRLN QATEDVYQLEQQ			LRR2	2
4233	LQSLQGHGH NGPPSSPSPGCFPGSQPALPS		LRR3	2.4

RLSRASQGLDQTVGPNRVSLWPNNKVHPSST

>BC058381

MKRASSGGSRLLAWVLWLQAWRVATP

27	CPGACVCYNEPKVTTSCPQQGLQAVPTGIPAS		NF	4
59	SQRIFLHGN RISHVPAASFQSCRN		LRR1	2.4
83	LTILWLHSN ALARIDAAAFTGLTL		LRR2	4
107	LEQLDLSDN AQLHVVDPTTFHGLGH		LRR3	4
132	LHTLHLDRC GLRELGPGLFRGLAA		LRR4	3.4
156	LQYLYLQDN NLQALPDNTFRDLGN		LRR5	4
180	LTHLFLHGN RIPSVPEHAFRGLHS		LRR6	4
204	LDRLLLHQN HVARVHPHAFRDLGR		LRR7	4
228	LMTLYLFAN NLSMLPAEVLMPLRS		LRR8	4
252	LQYLRLNDN				LRR9	4
261	PWVCDCRARPLWAWLQKFRGSSSEVPCNLPQRLADRDLKRLAASDLEGC		CF1_short	3

SHIFT -58!

252	LQYLRLNDN PWVCDCRARPLWAW		LRR9	4
275	LQKFRGSSS EVPCNLPQRLADRD		LRR10	2.2
298	LKRLAASDL EGCAVASGPFRPIQTSQLTD		LRR11	2.4
327	EELLSLPKC CQPDAADKASVLEPGRPASAG		LRR12	2.4

NALKGRVPPGDTPPGNGSGPRHINDSPFGTLPSSAEPPLTALRPGGSEPPGLPTTGPRRR
PGCSRKNRTRSHCRLGQAGSGASGTGDAEGSGALPALACSLAPLGLALVLWTVLGPC

>ENSMUSP00000061853

MVFSMWTRKRQILIFLNMLLVSRVFGFRWFPKTLPCEVKVNIPEAHVIVDCTDKHLTEIP
EGIPTN

67	TTNLTLTIN HIPSISPDSFRRLNH		LRR1	3
91	LEEIDLRCN CVPVLLGSKANVCTKRLQIRP		LRR2	3.4

SHIFT -19!

102	PVLLGSKAN VCTKRLQIRPGSFSGLSD		LRR1	2
129	LKALYLDGN QLLEIPQDLPSS			LRR2	4
150	LHLLSLEAN NIFSITKENLTELVN		LRR3	4
174	IETLYLGQN CYYRNPCNVSYSIEKDAF		LRR4	3.4
201	LVMRNLKVL SLKDNNVTAVPTTLPPN		LRR5	2
227	LLELYLYNN IIKKIQENDFNNLNE		LRR6	4
251	LQVLDLSGN CPRCYNVPYPCTPCEN		LRR7	4
276	NSPLQIHDN AFNSLTELKVLR			LRR8	2.4
297	LHSNSLQHV PPTWFKNMRNLQELD		LRR9	2
321	LSQNYLARE IEEAKFLHFLPN			LRR10	2
342	LVELDFSFN YELQVYHASITLPHSLSS		LRR11	3.4
369	LENLKILRV KGYVFKELKNSSLSVLHKLPR		LRR12	2.4
399	LEVLDLGTN FIKIADLNIFKHFEN		LRR13	4
423	LKLIDLSVN KISPSEESREVGFCPNAQTSV		LRR14	3.4

DRHGPQV

460	LEALHYFRY DEYARSCRFKNK			LRR1	2
481	EPPSFLPLN ADCHIYGQTLDLSRNNIFFIK		LRR2	2

SHIFT -15!

496	GQTLDLSRN NIFFIKPSDFQHLSF		LRR1	3.4
520	LKCLNLSGN TIGQTLNGSELWPLRE		LRR2	4
545	LRYLDFSNN RLDLLYSTAFEELQS		LRR3	3.4
569	LEVLDLSSN SHYFQAEGITHMLNFTKKLRL		LRR4	4
599	LDKLMMNDN DISTSASRTMESDS		LRR5	3.4
622	LRILEFRGN HLDVLWRAGDNRY			LRR6	3.4
644	LDFFKNLFN LEVLDISRNSLNSLPPEVFEG		LRR7	2.4

SHIFT -24!

650	LFNLEVLDI SRNSLNSLPPEVFEGMPPN		LRR1	2.4
678	LKNLSLAKN GLKSFFWDRLQLLKH		LRR2	4
702	LEILDLSHN QLTKVPERLANCSKS		LRR3	4
726	LTTLILKHN QIRQLTKYFLEDALQ		LRR4	4
750	LRYLDISSN KIQVIQKTSFPENVLNN		LRR5	3.4
776	LEMLVLHHN				LRR6	4
785	RFLCNCDAVWFVWWVNHTDVTIPYLATDVTCVGPGAHKGQSVISLDLYTCELD		CF1	4

SHIFT -12!

826	VISLDLYTC ELDLTNLILFSVSISS		LRR1	2.8
851	VLFLMVVMT TSHLFFWDMWYIYYFWKAKIK		LRR2	2.2

GYQHLQSMESCYDAFIVYDTKNSAVTEWV

910	LQELVAKLE DPREKHFNLCLEERDW		LRR1	2.4
935	LPGQPVLEN LSQSIQLSKKTVFVMTQKYAK		LRR2	2.4

TESFKMAFY

974	LSHQRLLDE KVDVIILIFLEKPLQK		LRR1	2
999	SKFLQLRKR LCRSSVLEWPANPQAHPYFWQ		LRR2	2

CLKNALTTDNHVAYSQMFKETV

>ENSMUSP00000051546

MEKF

5	LFYLFLIGI AVRAQICPKRCVCQILSPN		LRR1	3
33	LATLCAKKG LLFVPPNIDRRTVELRLADNF		LRR2	2.4

SHIFT -10!

53	TVELRLADN FVTNIKRKDFANMTS		LRR1	3
77	LVDLTLSRN TISFITPHAFADLRN		LRR2	4
101	LRALHLNSN RLTKITNDMFSGLSN		LRR3	4
125	LHHLILNNN QLTLISSTAFDDVFA		LRR4	4
149	LEELDLSYN NLETIPWDAVEKMVS		LRR5	4
173	LHTLSLDHN MIDNIPKGTFSHLHK		LRR6	4
197	MTRLDVTSN KLQKLPPDPLFQRAQVLATSG		LRR7	2.8

SHIFT -20!

207	LQKLPPDPL FQRAQVLATSGIISPS		LRR1	2
232	TFALSFGGN PLHCNCELLWLRRLSREDDLE		LRR2	2.4

TCASPALLTGRYFWSIPEEEFLCEPPLITRHTHEMRVLEGQRATLRCKARGDPEPAIHWI
SPEGKLISNATRSLVYDNGT

342	LDILITTVK DTGAFTCIASNPAGEATQTVD		LRR1	2
372	LHIIKLPHL LNSTNHIHEPDPGSSDISTST		LRR2	2.4

KSGSNASSSNGDTKMSQDKIVVAEATSSTALLKFNFQRNIPG

444	IRMFQIQYN GTYDDTLVYRMIPP		LRR1	2.2
467	TSKTFLVNN LASGTMYDLCVLAIYDDGITS		LRR2	2

LTATRVVGCIQFTTEQDYVRCHFMQSQFLGGTMIIIIGGIIVASVLVFIIILMIRYKVCN
NNGQHKVTKVSNVYSQTNGAQMQGCSVTLPQSMSKQAMGHEENAQCCKVASDNAIQSSET
CSSQDSSTTTSALPPTWTSSAPVSQKQKRKTGTKPSAEPQSEAVTNVESQNTNRNNSTAL
QLASCPPDSVTEGPTSQRAHTKPSKFLTVPAEGSRARHRASLSGGLKDSFHYGNSQLSLK
RSMSMNAMWT

>ENSMUSP00000025993

MALTPQRGSSSGLSRPELWLLLWAAAWRLGATA

34	CPALCTCTGTTVDCHGTGLQAIPKNIP		NF	4
61	RNTERLELN GNNITRIHKNDFAGLKQ		LRR1	2
87	LRVLQLMEN QIGAVERGAFDDMKE		LRR2	4
111	LERLRLNRN QLQVLPELLFQNNQA		LRR3	4
135	LSRLDLSEN FLQAVPRKAFRGATD		LRR4	4
159	LKNLQLDKN RISCIEEGAFRALRG		LRR5	4
183	LEVLTLNNN NITTIPVSSFNHMPK		LRR6	4
207	LRTFRLHSN				LRR7	3.4
216	HLFCDCHLAWLSQWLRQRPTIGLFTQCSGPASLRGLNVAEVQKGEFSCS		CF1_short	3

SHIFT -42!

223	LAWLSQWLR QRPTIGLFTQCSGPAS		LRR1	2
248	LRGLNVAEV QKGEFSCSGQGEAAGAPACTL		LRR2	2.4

SSGS

282	CPAMCSCSSGIVDCRGKGLTAIPANLPET		NF	4
311	MTEIRLELN GIKSIPPGAFSPYRK		LRR1	2.8
335	LRRIDLSNN QIAEIAPDAFQGLRS		LRR2	3.4
359	LNSLVLYGN KITDLPRGVFGGLYT		LRR3	4
383	LQLLLLNAN KINCIRPDAFQDLQN		LRR4	4
407	LSLLSLYDN KIQSLAKGTFTSLRA		LRR5	4
431	IQTLHLAQN				LRR6	3.4
440	PFICDCNLKWLADFLRTNPIETTGARCASPRRLANKRIGQIKSKKFRCS		CF1_short	3

AKEQYFIPGTEDYHLNSECTSDVA

513	CPHKCRCEASVVECSSLKLSKIPERIPQS		NF	4

SHIFT -16!

526	CSSLKLSKI PERIPQSTTELRLNNNEISI		LRR1	2
555	LEATGLFKK LSHLKKINLSNNKVSEIEDGT		LRR2	2

SHIFT -21!

564	LSHLKKINL SNNKVSEIEDGTFEGAAS		LRR1	2
591	VSELHLTAN QLESIRSGMFRGLDG		LRR2	3.4
615	LRTLMLRNN RISCIHNDSFTGLRN		LRR3	4
639	VRLLSLYDN HITTISPGAFDTLQA		LRR4	3.4
663	LSTLNLLAN				LRR5	4
672	PFNCNCHLSWLGDWLRKRKIVTGNPRCQNPDFLRQIPLQDVAFPDFRCE		CF1_short	3

SHIFT -42!

679	LSWLGDWLR KRKIVTGNPRCQNPDF		LRR1	2
704	LRQIPLQDV AFPDFRCEEGQEEVGCLPRPQ		LRR2	2.4


734	CPQECACLDTVVRCSNKHLQALPKGIPKN		NF	4

SHIFT -22!

741	LDTVVRCSN KHLQALPKGIPKN			LRR1	2.4
763	VTELYLDGN QFTLVPGQLSTFKY		LRR2	3.4
786	LQLVDLSNN KISSLSNSSFTNMSQ		LRR3	3.4
810	LTTLILSYN ALQCIPPLAFQGLRS		LRR4	4
834	LRLLSLHGN DVSTLQEGIFADVTS		LRR5	4
858	LSHLAIGAN				LRR6	3.4
867	PLYCDCRLRWLSSWVKTGYKEPGIARCAGPPEMEGKLLLTTPAKKFECQ		CF1_short	3

SHIFT -42!

874	LRWLSSWVK TGYKEPGIARCAGPPEM		LRR1	2
900	EGKLLLTTP AKKFECQGPPSLAVQAKCDPC		LRR2	2

LSSPCQNQGTCHNDPLEVYRCTCPSGYKGRHCEVSLDGCSSNPCGNGGTCHAQEGEDAGF
TCSCPSGFEGPTCGVDTDDCVKHACVNGGVCVDGVGNYTCQCPLQYTGRACEQLVDFCSP
DMNPCQHEAQCVGTPDGPRCECMLGYTGDNCSENQDDCKDHKCQNGAQ

1098	CVDEVNSYACLCVEGYSGQLCEIPPAPRSSCEGTECQNGANCVDQG		NF	4

SRPVCQC

1151	LPGFGGPEC EKLLSVNFVDRDTY		LRR1	2.2
1174	LQFTDLQNW PRANITLQVSTA			LRR2	2
1195	EDNGILLYN GDNDHIAVELYQGHVRVSYDP		LRR3	2.4

GSYPSSAIYSAETINDGQFHTVELVTFDQMVNLSIDGGSPMTMDNFGKHYTLNSEAPLY

1284	VGGMPVDVN SAAFRLWQILNGTSFH		LRR1	2.2
1309	GCIRNLYIN NELQDFTKTQMKPGVVPGCEP		LRR2	2.4

CRKLYCLHGICQPNATPGPVCHCEAGWGGLHCDQPVDGPCHGHKCVHGKCVPLDALAYSC
QCQDGYSGALCNQVGAVAEPCGGLQCLHGHCQASATKGAHCVCSPGFSGELCEQESECRG
DPVRDFHRVQRGYAICQTTRPLSWVECRGACPGQGCCQGLRLKRRKLTFECSDGTSFAEE
VEKPTKCGCAQCA

>ENSMUSP00000035489

MQWASVLLLAGLCSLSQGQYDEDSHWWIQYLRNQQSTYYDPYDPYPYEPSEPYPYGVEEG
PAYAYGAPPPPEPRD

76	CPQECDCPPNFPTAMYCDNRNLKYLPFVPSR		NF	4
107	MKYVYFQNN QISAIQEGVFDNATG		LRR1	2.2
131	LLWVALHGN QITSDKVGRKVFSKLRH		LRR2	3.4
157	LERLYLDHN NLTRMPGPLPRS			LRR3	4
178	LRELHLDHN QISRVPNNALEGLEN		LRR4	4
202	LTALYLHHN EIQEVGSSMRGLRS		LRR5	4
225	LILLDLSYN HLRRVPDGLPSA			LRR6	4
246	LEQLYLEHN NVYTVPDSYFRGSPK		LRR7	4
270	LLYVRLSHN SLTNNGLATNTFNSSS		LRR8	3.4
295	LLELDLSYN QLQKIPPVNTNLEN		LRR9	4
318	LYLQGNRIN EFSISSFCTVVDVMNFSK		LRR10	2
345	LQVLRLDGN EIKRSAMPVDAPLC		LRR11	4
368	LRLANLIEI			LRR12	2.4


>ENSMUSP00000053399

MISPAWSLFLIGTKIGLFFQVAPLSVVAKS

31	CPSVCRCDAGFIYCNDRSLTSIPVGIPED		NF	4
60	ATTLYLQNN QINNVGIPSDLKNLLK		LRR1	3.4
85	VQRIYLYHN SLDEFPTNLPKY			LRR2	2.8
106	VKELHLQEN NIRTITYDSLSKIPY		LRR3	3.4
130	LEELHLDDN SVSAVSIEEGAFRDSNY		LRR4	4
156	LRLLFLSRN HLSTIPGGLPRT			LRR5	4
177	IEELRLDDN RISTISSPSLHGLTS		LRR6	3.4
201	LKRLVLDGN LLNNHGLGDKVFFNLVN		LRR7	4
227	LTELSLVRN SLTAAPVNLPGTS			LRR8	4
249	LRKLYLQDN HINRVPPNAFSYLRQ		LRR9	4
273	LYRLDMSNN NLSNLPQGIFDDLDN		LRR10	3.4
297	ITQLILRNN				LRR11	3.4
306	PWYCGCKMKWVRDWLQSLPVKVNVRGLMCQAPEKVRGMAIKDLSAELFDCK		CF1_short	3

SHIFT -60!

297	ITQLILRNN PWYCGCKMKWVRDW		LRR11	3.4
320	LQSLPVKVN VRGLMCQAPEKVRGMAIKDLS		LRR12	3.4

AELFDCKDSGIVSTIQITTAIPNTAYPAQGQWPAPVTKQPDIKNPKLIKDQRTTGSPSRK
TILITVKSVTPDTIHISWRLALPMTALRLSWLKLGHSPAFGSITETIVTGERSEYLVTAL
EPESPYRVCMVPMETSNLYLFDETPVCIETQTAPLRMYNPTTTLNREQEKEPYK

524	NPNLPLAAI IGGAVALVSIALLA		LRR1	2
547	LVCWYVHRN GSLFSRNCAYSKGRRRKDDYA		LRR2	2.8

EAGTKKDNSILEIRETSFQMLPISNEPISKEEFVIHTIFPPNGMNLYKNNLSESSSNRSY
RDSGIPDSDHSHS

>ENSMUSP00000028337

MRGSFFSRLPPQLSLLL

18	LLSLRRVWT QEDIGTAPSKSPVAPE		LRR1	2.4
43	CPEACSCSLGGKANCSALALPAVPADLSWQ		NF	4

SHIFT -25!

48	SCSLGGKAN CSALALPAVPADLSWQ		LRR2	2.4
73	VRSLLLDHN RVSALPPGAFANAGA		LRR3	3.4
97	LLYLDLREN RLRSVHARAFWGLGV		LRR4	4
121	LQWLDLSSN QLETLPPGTFAPLRA		LRR5	4
145	LSFLSLAGN RLALLEPSILGPLPL		LRR6	4
169	LRVLSLQDN SLSAIEAGLLNNLPA		LRR7	4
193	LDVLRLHGN				LRR8	4
202	PWTCNCALRPLCTWLRKHPRPASETETLLCVSPRLQTLSLLTAFPDAAFKQC		CF1_short	3

SHIFT -45!

209	LRPLCTWLR KHPRPASETETLLCVSPR		LRR1	2
236	LQTLSLLTA FPDAAFKQCTQS			LRR2	3
257	LAARDLAVV YALGPVSFLASL			LRR3	2
278	AICLALGSV LTACGARRRRRRRTTVRH		LRR4	2.4
305	LLRRQLDPE GPPSLEDAGSPVTAAIQA		LRR5	2


>ENSMUSP00000061244

MQPQRDLRGLWLLLLSVFLLLFEVARAGRSVVS

34	CPANCLCASNILSCSKQQLPNVPQSLPSY		NF	4
63	TALLDLSHN NLSRLRAEWTPTRLTN		LRR1	3
88	LHSLLLSHN HLNFISSEAFVPVPN		LRR2	4
112	LRYLDLSSN HLHTLDEFLFSDLQA		LRR3	4
136	LEVLLLYNN HIVVVDRNAFEDMAQ		LRR4	4
160	LQKLYLSQN QISRFPVELIKDGNKLPK		LRR5	4
187	LMLLDLSSN KLKKLPLTDLQKLPAWV		LRR6	4
213	KNGLYLHNN				LRR7	3
222	PLECDCKLYQLFSHWQYRQLSSVMDFQEDLYCMHSKKLHNIFSLDFFNCSEY		CF1	4

KES

277	AWEAHLGDT LTIRCDTKQQGMTKVWVSPSN		LRR1	2.2
307	EQVLSQGSN GSVSVRNGDLFFKKVQVEDGG		LRR2	2

VYTCYAMGETFNET

351	LSVELKVYN FTLHGHHDTLNTAYTT		LRR1	2
376	LVGCILSVV LVLIYLYLTPCRCWCRGVEKP		LRR2	2

SHIFT -21!

385	LVLIYLYLT PCRCWCRGVEKPSSHQGDS		LRR1	2.8
413	LSSSMLSTT PNHDPMAGGDKDDGFDRRVAF		LRR2	2.4

LEPAGPGQGQNGKLKPGNTLPVPEATGKGQRRMSDPESVSSVFSDTPIVV

>ENSMUSP00000070130

MHPLWAF

8	LLGLSLTNG LSAN		LRR1	3
21	CPGRCSCDSMQSVQCYRLMELPSGIPSTTK		NF	4

SHIFT -13!

38	LMELPSGIP STTKRLYISHSR			LRR2	2
59	IQHLQLSNF TGLLALEDFILLASGTESIEN		LRR3	2.4

SHIFT -21!

68	TGLLALEDF ILLASGTESIENDTFKTLST		LRR1	2
97	LKTLELWKN KLRQVPSALPAN			LRR2	4
118	LEVLKLNDN AICALRGSEFEGLKN		LRR3	4
142	LKVLELKNN LISSLSPSMLSPLAS		LRR4	4
166	LQSLMVDGN NIESVVGPLSLPH			LRR5	3.4
188	LKYMSMENN QLHLIPGNVFTSLQN		LRR6	2.8
212	LQFLSFSGN FLTKIPINLPKS			LRR7	3.4
233	LLSLKMERN QLKVVRFRDMKHLEN		LRR8	3.4
257	LSHLYLSEN FLSSIDGAQQLTN			LRR9	4
279	LTTLEVSQN QLQMLPPRLPSR			LRR10	3.4
300	LQKLDCSSN FIQRVTAPEFQDLRD		LRR11	3
324	LKHLFLDNN VVSLFEAGALQRCSQ		LRR12	4
348	LSNLALEQN LLLSIPLRLPKT			LRR13	4
369	LARLDLKGN AIQDMAERELRDLKQ		LRR14	4
393	LQVLNLRNN RISALDFKALEGLPR		LRR15	4
417	LRHLYLDGN				LRR16	4
426	PWNCTCSLLRAREVLKAKGTDVKGGQCAAPAERQGESWMSSKKILRQCE		CF1_short	3

HHLQQSEKSKETKKKPKPEDSSSIRLNMDDDDDDYEID

>IPI00662472.2

M

2	LRGQRLGQL GWHRPAAGLGSL			LRR1	2
23	MTSLMLACA SAASCREVCCPVGPSGLRCTR		LRR2	2.4

SHIFT -25!

28	LACASAASC REVCCPVGPSGLRCTRAGS		LRR1	2.2
56	LDTLRGLRG AGNLTELYVENQQH		LRR2	2.4
79	LQRLEFEDL QGLGELRSLTIVKSGLRFVAP		LRR3	2.4

SHIFT -22!

87	LQGLGELRS LTIVKSGLRFVAPDAFRFTPR		LRR1	2.4
117	LSHLNLSSN ALESLSWKTVQGLS		LRR2	4
140	LQDLTLSGN PLHCSCALFWLQRWEQEGLCG		LRR3	4

SHIFT -14!

156	LFWLQRWEQ EGLCGVHTQTLHDSGPG		LRR1	2
182	DQFLPLGHN TSCGVPTVKIQMPNDSVEVGD		LRR2	3

DVFLQCQ

219	VEGLALQQA DWILTELEGAATVKKFGD		LRR1	2.4
246	LPSLGLILV NVTSDLNKKNVTCWAENDVGR		LRR2	3

AEVSVQVSVSFPA

289	SVHLGLAVE QHHWCIPFSVDGQP		LRR1	2
312	APSLRWLFN GSVLNETSFIFTQFLESALTN		LRR2	2.8

SHIFT -27!

315	LRWLFNGSV LNETSFIFTQFLESALTNET		LRR1	2
344	MRHGCLRLN QPTHVNNGNYTLLAANPYGQA		LRR2	2.8

AASVMAAFMDNPFEFNPEDPIPVSFSPVDGNSTSRDPVEKKDETPFGVSVAVGLAVSAAL
FLSAL

439	LLVLNKCGQ RSKFGINRPAVLAPEDGLAMS		LRR1	2
469	LHFMTLGGS SLSPTEGKGSGLQGHIMENPQ		LRR2	2.8

YFSDTCVHHIKRQDIILKWELGEGAFGKVFLAECYNLLNDQDKMLVAVKALKEASENARQ
DFQREAELLTMLQHQHIVRFFGVCTEGGPLLMVFEYMRHGDLNRFLRSHGPDAKLLAGGE
DVA

622	PGPLGLGQL LAVASQVAAGMVY			LRR1	2
644	LASLHFVHR DLATRNCLVGQGLVVKIGDFG		LRR2	2.4

MSRDIYSTDYYRVGGRTMLPIRWMPPESILYRKFSTESDVWSFGVVLWEIFTYGKQPWYQ
LSNTEAIECITQGRELERPRACPPDVYAIMRGCWQREPQQRLSMKDVHARLQALAQAPPS
YLDVLG

>ENSMUSP00000047844

MARALLFSLVFLAILLPALAA

22	CPQNCHCHGDLQHVICDKVGLQKIPKVSET		NF	4
52	TKLLNLQRN NFPVLAANSFRTMPN		LRR1	3
76	LVSLHLQHC NIREVAAGAFRGLKQ		LRR2	3.4
100	LIYLYLSHN DIRVLRAGAFDDLTE		LRR3	4
124	LTYLYLDHN KVSELPRGLLSPLVN		LRR4	4
148	LFILQLNNN KIRELRAGAFQGAKD		LRR5	4
172	LRWLYLSEN ALSSLQPGSLDDVEN		LRR6	4
196	LAKFHLDKN QLSSYPSAALSKLRV		LRR7	3.4
220	VEELKLSHN PLKSIPDNAFQSFGRY		LRR8	3.4
245	LETLWLDNT NLEKFSDAAFSGVTT		LRR9	3.4
269	LKHVHLDNN RLNQLPSSFPFDN			LRR10	3.4
291	LETLTLTNN				LRR11	4
300	PWKCTCQLRGLRRWLEAKASRPDATCSSPAKFKGQRIRDTDALRSCKS		CF1_short	3

PTKRSKKAGRH

>Y39A1A.7

MTSITSAFLLLLLFVAVACRS

22	VPCLDGCTC ELSEHDPVIRCDGVG		LRR1	2.2
46	LNRFPLPHS SPLRGFHFLALTCNDIDTIPA		LRR2	2.8

SHIFT -16!

60	FHFLALTCN DIDTIPAISMIKASFPD		LRR1	3.4
86	LQGIDVQGN TRLNCSDLVHLHKD		LRR2	2.8
109	IPVLSDCDN EKPLQCDNLDKNCDWKCRT		LRR3	2.4
137	LTKLKEMWA QFKDLVNRKAKEWQAEETIEA		LRR4	2

AKSWFSAQFKKFKIAIGELSD

>ENSMUSP00000021820

MKE

4	YVMLLLLAV CSAKPFFSPSHTA			LRR1	2
26	LKNMMLKDM EDTDDDDNDDDDNS		LRR2	2.4
49	LFPTKEPVN PFFPFDLFPTCPFGCQCYSRV		LRR3	2

SHIFT -20!

59	FFPFDLFPT CPFGCQCYSRVVHCSD		LRR1	2.2
84	LGLTSVPNN IPFDTRMVDLQNNKIKEIKEN		LRR2	2.4

SHIFT -17!

97	TRMVDLQNN KIKEIKENDFKGLTS		LRR1	2.4
121	LYALILNNN KLTKIHPKTFLTTKK		LRR2	4
145	LRRLYLSHN QLSEIPLNLPKS			LRR3	4
166	LAELRIHDN KVKKIQKDTFKGMNA		LRR4	3.4
190	LHVLEMSAN PLENNGIEPGAFEGVTVFHIR		LRR5	3.4

IAEAKLTSIPKG

232	LPPTLLELH LDFNKISTVELEDLKRYRE		LRR1	2
260	LQRLGLGNN RITDIENGTFANIPR		LRR2	4
284	VREIHLEHN KLKKIPSGLQELKY		LRR3	2.8
307	LQIIFLHYN SIAKVGVNDFCPTVPKMKKS		LRR4	3.4
336	LYSAISLFN NPMKYWEIQPATFRCVLGRMS		LRR5	2.4

VQLGNVGK

>ENSMUSP00000030971

MEPPPPLLLLPLALLALLWGGERGAAALPAGCKHDGRARGTGRAAAAAEGKVVCSSLELA
QV

63	LPPDTLPNR TVTLILSNNKISELKNGS		LRR1	2
90	FSGLSLLER LDLRNNLISRIAPGAFWGLSS		LRR2	2.4
120	LKRLDLTNN RIGCLNADVFRGLTN		LRR3	4
144	LVRLNLSGN LFTSLSQGTFDYLGS		LRR4	4
168	LRSLEFQTE				LRR5	2.4
177	YLLCDCNILWMHRWVKERNITVRDTRCVYPKSLQAQPVTGVKQELLTCD		CF1_short	3

PPLELPSFYMTPSHRQVVFEGDSLPFQCMASYIDQDMQVLWYQDGRIVETDESQGIFVEK
SMIHNCSLIASALTISNIQAGSTGNWGCHVQTKRGNNTRTVDIVVLESSAQYCPPERVVN
NKGDFRWPRTLAGITAYLQCTRNTHSSGIYPGSAQDERKAWRRCDRGGFWADDDYSRCQY
ANDVTRVLYMFNQMPLNLTNAVATARQLLAYTVEAANFSDKMDVIFVAEMIEKFGRFTRE
EKSKELGDVMVDVASNIMLADERVLWLAQREAKACSRIVQCLQRIATHRLASGAHVYSTY
SPNIALEAYVIKAAGFTGMTCSVFQKVAASDRAGLSDYGRRDPDGNL

573	DKQLSFKCN VSSTFSSLALKNTIMEASIQ		LRR1	2.4
602	LPSSLLSPK HKREARAADDALY			LRR2	2
624	KLQLIAFRN GKLFPATGNSTKLADDGKRRT		LRR3	2.4

VVTPVILTKIDGATVDTHHIPVNVTLRRIAHGADAVAAQWDFDLLNGQGGWKSDG

709	CCILYSDEN ITTIQCGSLGNYAV		LRR1	2
732	LMDLTGTEL YTPAASLLHPVVYTTAITLL		LRR2	2.4
761	LCLLAVIIS YMYHHSLIRISLKSWHM		LRR3	2.8
787	LVNLCFHIL LTCVVFVGGITQTRNASVCQ		LRR4	2.4
816	AVGIILHYS TLATVLWVGVTARNIYKQVTK		LRR5	2.2

KAKRCQDPDEPPAPPRPMLRFYLIGGGIPII

877	VCGITAAAN IKNYGSRPSAPYCWMAWEPS		LRR1	2.2
906	LGAFYGPAS FITFVNCMYFLSIFIQLKRHP		LRR2	2.2

ERKYELKEPT

946	EEQQRLAAN ENGEINHQDSMSLS		LRR1	2
969	LISTSTLEN EHSFQSQLLGASLTL		LRR2	2
993	LLYVILWMF GAMAVSLYYPLDLVFSFFFGA		LRR3	2.4

TCLSFSAFMMVHHCINREDVRLAWIMMCCPGRSSYSVQVNVQPPNSSATNGEAPKCTNSS
AESSCTNKSASSFKNSSQGCKLTNLQAAAAQYHSNALPVNATPQLDNSLTEHSMDNDIKM
HVAPLDVQFRTNVHPSRHHKNRSKGHRASR

1173	LTVLREYAY DVPTSVEGSVQNG			LRR1	2
1195	LPKSRPGSN EGHSRSRRAYLAYRERQYNPP		LRR2	2

QQDSSDACSTLPKSSRNVEKPVSTSSKKDAPRKPAAADLESQQK

1269	SYGLNLAVQ NGPVKSNGQEGP			LRR1	2
1290	LLATDVTGN VRTGLWKHETTV			LRR2	2.4


>ENSMUSP00000033876

MPVPPARLLLLPLLPCLLLLAPGTRGAPGCPVPIRGCKCSGERPKGLSGGAHNPARRRVV
CGGGDLPE

69	PPDPGLLPN GTITLLLSNNKITGLRN		LRR1	2
95	GSFLGLSLL EKLDLRSNVISTVQP		LRR2	2.4
119	GAFLGLGEL KRLDLSNNRIGCLTSETFQ		LRR3	2.4
147	GLPRLLRLN ISGNIYSSLQPGVFDE		LRR4	2.4
172	LPALKIVDF GTEFLTCDCRLRWLLPWAR		LRR5	2.4
200	NHSLQLSER TLCAYPSALHAHA			LRR6	2
222	LSSLQESQL RCEGALELHTHYLIPSLRQVV		LRR7	2

SHIFT -27!

225	LQESQLRCE GALELHTHYLIPSLRQVVFQ		LRR1	2
254	GDRLPFQCS ASYLGNDTRIHWYHNGAPMES		LRR2	2.2

DE

286	QAGIVLAEN LIHDCTFITSELT			LRR1	2.4
308	LSHIGVWAS GEWECSVSTVQGNTSKK		LRR2	2.2
334	VEIVVLETS ASYCPAERVTNNRGDFRWPRT		LRR3	2.2
364	LAGITAYQS CLQYPFTSVPLSGGAPGTRAS		LRR4	2.2

RRCDRAGRWEPG

406	DYSHCLYTN DITRVLYTFVLMPINA		LRR1	2
431	SNALTLAHQ LRVYTAEAASFSDMMDVVYVA		LRR2	2

QMIQKFLGYVDQIKELVEVMVDMASNLMLVDEHLLWLAQREDKACSGIVGALERIGGAAL
SPHAQHISVNSRNVALEAYLIKPHSYVGLTCTAFQRREVGVSGAQPSSVGQDAPVEPEP

580	LADQQLRFR CTTGRPNISLSSFHIKNSVA		LRR1	2
609	LASIQLPPS LFSTLPAALAPPVPPDC		LRR2	2.8
635	TLQLLVFRN GRLFRSHGNNTSRPGAAGPGK		LRR3	2.4

RRGVATPVIFA

676	GTSGCGVGN LTEPVAVSLRHWAEG		LRR1	2.2
700	ADPMAAWWN QDGPGGWSSEGCRLRYSQPNV		LRR2	2.2

SSLYCQHLGNVAV

743	LMELNAFPR EAGGSGAGLHPVVYPCTA		LRR1	2.4
770	LLLLCLFST IITYILNHSSIHVSRKGWHM		LRR2	3.4
799	LLNLCFHMA MTSAVFVGGVTLTNYQMVCQ		LRR3	2.4
828	AVGITLHYS SLSSLLWMGVKARVLHKELSW		LRR4	2.2

RAPPLEEGEAAPPGPRPMLRFYLIAGGIPLIICGITAAVNIHNYRDHSPYCWLVWRPSLG
AFYIPVALILPITWIYFLCAGLHLRSHVAQNPKQGNRISLEPGEELRGSTRLRSSGVLLN
DSGSLLATVSAGVGTPAPPEDGDGVYSPGVQLGALMTTHFLYLAM

1023	WACGALAVS QRWLPRVVCSCLYGVA		LRR1	2.2
1048	ASALGLFVF THHCARRRDVRASWRACCPPA		LRR2	2.4

SPSASHVPARALPTATEDGSPVLGEGPASLKSSPSGSSGRAPPPPCKLTNLQVAQSQVCE
ASVAARGDGEPEPTGSRGSLAPRHHNNLHHGRRVHKSRAKGHRAGETGGKSRLKALRAGT
SPGAPELLSSESGSLHNSPSDSYPGSSRNSPGDGLPLEGEPMLTPSEGSDTSAAPIAETG
RPGQRRSASRDNLKGSGSALERESKRRSYPLNTTSLNGAPKGGKYEDASVTGAEAIAGGS
MKTGLWKSETTV

>ENSMUSP00000062158

MKLLWQVTVHHTWNAVLLPVVYLTAQVWILCAAIAAAASAGPQN

45	CPSVCSCSNQFSKVVCTRRGLSEVPQGIPSN		NF	4
76	TRYLNLMEN NIQMIQADTFRHLHH		LRR1	3
100	LEVLQLGRN SIRQIEVGAFNGLAS		LRR2	4
124	LNTLELFDN WLTVIPSGAFEYLSK		LRR3	4
148	LRELWLRNN PIESIPSYAFNRVPS		LRR4	4
172	LMRLDLGEL KKLEYISEGAFEGLFN		LRR5	3
197	LKYLNLGMC NIKDMPNLTPLVG			LRR6	3.4
219	LEELEMSGN HFPEIRPGSFHGLSS		LRR7	3.4
243	LKKLWVMNS QVSLIERNAFDGLAS		LRR8	2.8
267	LVELNLAHN NLSSLPHDLFTPLRY		LRR9	4
291	LVELHLHHN				LRR10	4
300	PWNCDCDILWLAWWLREYIPTNSTCCGRCHAPMHMRGRYLVEVDQAAFQCS		CF1_short	3

APFIMDAPRDLNISEDRMAELKCRTPPM

379	SSVKWLLPN GTVLSHASRHPRISV		LRR1	2
403	LNDGTLNFS RVLLIDTGVYTCM			LRR2	2.8
425	VTNVAGNSN ASAYLNVSSAELNTPNFSFFT		LRR3	2.2

TVTVETTEISPEDITRKYKPVPTTSTGYQPAYTTSTTVLIQTTRVPKQVPVPSTDTTDKM
QTSLDEVMKTTKIIIGCFVAVTLLAAAMLIVFYKLRKRHQQRSTVTAARTVEIIQVDEDI
PAAAPAAATAAPSGVSGEGAVVLPTIHDHINYNTYKPAHGAHWTENSLGNSLHPTVTTIS
EPYIIQTHTKDKVQETQI

>NM_173415

MLILLLHAVVFSLPYTRATEA

22	CLRACPAACTCSHVERGCSVRCDRAGLQRVPQEFPCE		NF	4
59	AASIDLDRN GLRILGERAFGTLPS		LRR1	2.8
83	LRRLSLRHN NLSFITPGAFKGLPR		LRR2	4
107	LAELRLAHN GELRYLHVRTFAALGR		LRR3	4
132	LRRLDLAAC RLFSVPERLLAELPA		LRR4	3.4
156	LRELTAFDN LFRRVPGALRGLAN		LRR5	3.4
179	LTHAHFERS RIEAVASGSLLGMRR		LRR6	2.2
203	LRSLSLQAN RVRAVHAGAFGDCGA		LRR7	4
227	LEDLLLNDN LLATLPAAAFRGLRR		LRR8	4
251	LRTLNLGGN ALGSVARAWFSDLAE		LRR9	4
275	LELLYLDRN SITFVEEGAFQNLSG		LRR10	4
299	LLALHLNGN RLTVLSWAAFQPGFF		LRR11	4
323	LGRLFLFRN				LRR12	4
332	PWRCDCQLEWLRDWMEGSGRVADVACASPGSVAGQDLSQVVFERSSDG		CF1_short	3

SHIFT -41!

339	LEWLRDWME GSGRVADVACASPGSVAGQD		LRR1	2
368	LSQVVFERS SDGLCVDPDELNFTTSSPGPS		LRR2	2.2

PEPVATTVSRFSS

411	LLSKLLAPR APVEEVANTTWE			LRR1	2
432	LVNVSLNDS FRSHAVMVFCYKATFLFTS		LRR2	2.8
460	CVLLSLAQY VVVGLQRE			LRR3	2


>ENSMUSP00000041579

MGGAGILLFLLAWAGAGVAWSPPKGK

27	CPPHCSCSKENTLCEGSPELPESFS		NF	4
52	TTLLSLSLV RMGVSRLKAGSFLKMPS		LRR1	2
78	LHLLLFTSN TFSVIEGDAFIGLSY		LRR2	3.4
102	LQYLFIEDN KIGSISKNALRGLRS		LRR3	3.4
126	LTHLSLANN HLEALPRFLFRGLET		LRR4	4
150	LTHVDLRGN				LRR5	3.4
159	PFQCDCRVLWLLQWMPTVNASVGTGACAGPPAVAQIQLNHLDPKKFKCR		CF1_short	3

SHIFT -41!

167	LWLLQWMPT VNASVGTGACAGPPAVAQIQ		LRR1	2.8
196	LNHLDPKKF KCRATELSWLQTVGESALSVE		LRR2	2

SFSYQGEPHMVLAQPFA

243	GRCLILVWD YSLQRFRPEEELSAPSVVS		LRR1	2.4
271	CKPLVLGPR LFILAARLWGGSQLWSRS		LRR2	2
298	SPDLRLTPV QVLAPQRLLRPNDAE		LRR3	2
322	LLWLDGQPC FVVADASKAGSTTLLCRDGPG		LRR4	2.8

FYPRQSLHAWHRDTDAEALELDGRPHLLLASASQRPVLFHWVGGRFERRTDIPEAEDVYA
TKHFQA

418	GGDVFLCLT RYIGDSMVMRWDGSMFRL		LRR1	2.2
445	LQQLPSRGS HVFQPLLIARDQ			LRR2	2.4
466	LAILGSDFA FSQVFRFESDKGI			LRR3	2
488	LEPLQELGP PALVAPRAFAQVTVAGRRFLF		LRR4	2

AACFKGPTQIYQHHELDLSA

>XP_001005865

MAPRAGQRGLWSP

14	LPGLLLLAA ALSRPAAP		LRR1	3
31	CPFQCYCFGSPRLMLRCASGAELRQPPRDVPP		NF	4

SHIFT -23!

40	SPRLMLRCA SGAELRQPPRDVPPDARN		LRR2	2
67	LTIVGANLT VLRAAAFAGGGEGATD		LRR3	2.2
92	GVRLPLLTA LRLTHNNIEVVEDGAFDGLPS		LRR4	2.4
122	LAALDLSHN PLRALGYRAFRGLPA		LRR5	4
146	LRSLQLNHA LARGSPGMLDALDAALAPLAE		LRR6	3
176	LRLLGLVGN ALSRLPLAALRLPR		LRR7	4
199	LEQLDARVN ALAGLGPDELSALERDGDLP		LRR8	3.4
228	QPRLLLADN				LRR9	3
237	PLSCGCTSRPLLAWLHNATERVPDARRLRCASPRVLLDRPLIDLDEARLGCS		CF1_short	3

SHIFT -44!

245	RPLLAWLHN ATERVPDARRLRCASPRV		LRR1	2.4
272	LLDRPLIDL DEARLGCSDGDAHESGEGIDV		LRR2	2

AGPELEASYVFFGLVLALIGLIFLMVLYLNRRGIQRWMHNLREACRDQMEGYHYRYEQDA
DPRRAPAPAAPAGSRATSPGSGL

>ENSMUSP00000092127

MIT

4	LTELKCLAD AQSSYHILKPWWDVFWYYIT		LRR1	2
33	LIMLLVAVL AGALQLTQSRVLCC		LRR2	2.4
56	LPCKVEFDN QCAVPWDLLKGSENASSNSGL		LRR3	2
86	LLPLPLRIQ NDLHRQQYSYIDAVCYEKQLH		LRR4	3

WFAKFFPYLVLLHTLIFAACSNFWLHYPSTSSRLEHFVSILHKCFDSPWTTRALPLKLSK
SKTLLSTSGGSADIDASKQSLPYPQPGLESPGIESPTSSVLDKKEGEQAKAIFEKVKRFR
LHVEQRDIIYRVYLKQIIVKVILFVLIITYVPYFLSYITLEIDCSIDVQAFTGYKRYQCV
YSLAEIFKVLASFYVI

312	LVMLYGLTS SYSLWWMLRSSLKQYS		LRR1	2.8
337	FEALREKSN YSDIPDVKNDFAFILHLADQY		LRR2	2.4

SHIFT -10!

357	AFILHLADQ YDPLYSKRFSIFLSEVSENK		LRR1	2.4
386	LKQINLNNE WTVERLKSKLVKNSQDKVE		LRR2	2.4
414	LHLFMLNGL PDNVFELTEMEVLSLELIPEV		LRR3	2.4

SHIFT -12!

432	MEVLSLELI PEVKLPAAVAQLVN		LRR1	2.4
455	LRELHVYHS SLVVDHPALAFLEEN		LRR2	2.8
479	LRILRLKFT EMGKIPRWVFHLKN		LRR3	3.4
502	LKELYLSGC VLPEQLSSLHLEGFQDLKN		LRR4	3.4
530	LRTLYLKSS LSRIPQVVTDLLPS		LRR5	3.4
553	LQKLSLDNE GSKLVVLNNLKKMVN		LRR6	3
577	LKSLELLSC DLERIPHSIFSLNN		LRR7	3.4
600	LHELDLKEN NLKTVEEIISFQHLPS		LRR8	4
625	LSCLKLWHN NIAYIPAQIGALSN		LRR9	4
648	LEQLFLGHN NIESLPLQLFLCTK		LRR10	4
671	LHYLDLSYN HLTFIPEEIQYLTN		LRR11	4
694	LQYFAVTNN NIEMLPDGLFQCKK		LRR12	2.8
717	LQCLLLGRN SLTDLSPLVGELSN		LRR13	4
740	LTHLELTGN YLETLPVELEGCQS		LRR14	4
763	LKRSCLIVE DSLLNSLPLPVAERLQTCLDK		LRR15	2

C

>ENSMUSP00000057563

MA

3	LLILLFLLP SPLHSQHTCSIS			LRR1	2.4
24	KVTSLLEVN CENKKLTALPADLPAD		LRR2	2
49	TGILHLGEN QLGTFSTASLVHFTH		LRR3	3
73	LTYLYLDRC ELTSLQTNGKLIK			LRR4	3.4
95	LENLDLSHN NLKSLPSLGWALPA		LRR5	4
118	LTTLDVSFN KLGSLSPGVLDGLSQ		LRR6	3.4
142	LQELYLQNN DLKSLPPGLLLPTTK		LRR7	4
166	LKKLNLANN KLRELPSGLLDGLED		LRR8	4
190	LDTLYLQRN WLRTIPKGFFGTLL		LRR9	4
213	LPFVFLHAN SWYCDCEILYFRHWLQENANN		LRR10	3.4

VYLWKQGVDVKDTTPNVASVRCANLDNAPVYSYPGKGCPTSSGDTDYDDYDDIPDVPATR
TEVKFSTNTKVHTTHWSLLAAAPSTSQDSQMISLPPTHKPTKKQSTFIHTQSPGFTTLPE
TMESNPTFYSLKLNTVLIPSPTTLEPTSTQATPEPNIQPMLTTSTLTTPEHSTTPVPTTT
ILTTPEHSTIPVPTTAILTTPKPSTIPVPTTATLTTLEPSTTPVPTTATLTTPEPSTTLV
PTTATLTTPEHSTTPVPTTATLTTPEHSTTPVPTTATLTTPEPSTTLTNLVSTISPVLTT
TLTTPESTPIETILEQFF

561	TTELTLLPT LESTTTIIPEQNSFLN		LRR1	2.4
586	LPEVALVSS DTSESSPFLNSDFCCFLPLG		LRR2	2.8
615	FYVLGLLWL LFASVVLILLLTWTWHVTPHS		LRR3	2.4

LDMEQSAALATSTHTTSLEVQRARQVTMPRAWLLFLQGSLPTFRSSLFLWVRPNGRVGPL
VAGRRPSALSQGRGQDLLGTVGIRYSGHSL

>ENSMUSP00000053869

MAGHGWGTAWVLVAAATLLHAGGLAQGDCWLIEGDKGFVWLAICSQNQPPYEAIPQQIN

60	NTIVDLRLN ENRIRSVQYASLSRFGN		LRR1	2.4
86	LTYLNLTKN EIGYIEDGAFSGQFN		LRR2	4
110	LQVLQLGYN RLRNLTEGMLRGLSK		LRR3	4
134	LEYLYLQAN LIEVVMASAFWECPN		LRR4	4
158	IVNIDLSMN RIQQLGSGTFAG			LRR5	2.8
179	LTKLSVCEI YSNPFYCSCELLGF		LRR6	2.4
202	LRWLAAFTN ATQTHDRVQCESPPVYAGYFL		LRR7	3.4

LGQGRHGHQRSILSKLQSVCTEGSYTAEVLGPPRPVPGRSQPGHSPPPPPPEPSDMPCAD
DECFSGDGTTP

303	LVILTTLVP QTEARPSMKVKQLTQNSATI		LRR1	2
332	MVQLPSPFN RMYTLEQYNNSKSFTVSKLTQ		LRR2	2.4

SHIFT -20!

342	MYTLEQYNN SKSFTVSKLTQPQEEIR		LRR1	2.4
368	LTNLYTLTN YTYCVVSTSSGTHHNHTCLTI		LRR2	3

CLPKPPSPPGPVPSPSTATHYIMTILGC

426	LFGMVLVLG AVYYCLRKRRRQEEKHKKAVA		LRR1	2.4
456	AAAGSLKKT IIELKYGPEIEAPGLAP		LRR2	2.2
482	LTQGPLLGP EAVTRIPYLPAATSDVEQYKL		LRR3	2.4

VESSETPKATKGNYIEVRTGEPQERRGCELSRPGEPQSSVAEISTIAKEVDRVNQIINNC
IDALKSESTSFQGAKSGAVSAAEPQLVLLSEPLASKHSFLSPVYKDAFGHGGLQRHHSVE
AAPGPPRASTSSSGSARSPRTFRAEATGTHKAPATETKYIEKSSPVPETILTVTPAATVL
RAEADKSRQYGEHRHSYPGSHPAEPPAPPPPPPTHEGLGGRKASILEPLTRPRPRD

748	LVYSQLSPQ YHNLSYSSSPEYTCRASPSI		LRR1	2
777	WERLRLSRR RHKDDAEFMAAGHALRKKVQF		LRR2	2.4

AKDEDLHDILDYWKGVSAQHKS

>ENSMUSP00000033967

MSGIGWQTLSL

12	SLGLVLSIL NKVAPQACPAQCSCSGSTVD		LRR1	2
41	CHGLALRSV PRNIPRNTERLDLNGNNITRI		LRR2	2

SHIFT -16!

55	RNTERLDLN GNNITRITKIDFAGLRH		LRR1	2
81	LRVLQLMEN RISTIERGAFQDLKE		LRR2	4
105	LERLRLNRN NLQLFPELLFLGTAK		LRR3	4
129	LYRLDLSEN QIQAIPRKAFRGAVD		LRR4	4
153	IKNLQLDYN QISCIEDGAFRALRD		LRR5	3.4
177	LEVLTLNNN NITRLSVASFNHMPK		LRR6	4
201	LRTFRLHSN				LRR7	3.4
210	NLYCDCHLAWLSDWLRQRPRVGLYTQCMGPSHLRGHNVAEVQKREFVCS		CF1_short	3

GHQSFMAPS

268	CSVLHCPAACTCSNNIVDCRGKGLTEIPTNLPET		NF	4
302	ITEIRLEQN SIRVIPPGAFSPYKK		LRR1	2.8
326	LRRLDLSNN QISELAPDAFQGLRS		LRR2	4
350	LNSLVLYGN KITELPKSLFEGLFS		LRR3	4
374	LQLLLLNAN KINCLRVDAFQDLHN		LRR4	4
398	LNLLSLYDN KLQTVAKGTFSALRA		LRR5	4
422	IQTMHLAQN				LRR6	2.8
431	PFICDCHLKWLADYLHTNPIETSGARCTSPRRLANKRIGQIKSKKFRCS		CF1_short	3

GTEDYRSKLSGDCFADLA

498	CPEKCRCEGTTVDCSNQRLNKIPDHIP		NF	4
525	QYTAELRLN NNEFTVLEATGIFKKLPQ		LRR1	2.4
552	LRKINFSNN KITDIEEGAFEGASG		LRR2	2.8
576	VNEILLTSN RLENVQHKMFKGLES		LRR3	2.8
600	LKTLMLRSN RISCVGNDSFIGLGS		LRR4	4
624	VRLLSLYDN QITTVAPGAFDSLHS		LRR5	3.4
648	LSTLNLLAN				LRR6	4
657	PFNCNCHLAWLGEWLRRKRIVTGNPRCQKPYFLKEIPIQDVAIQDFTCD		CF1_short	3

DGNDDNSCSPLSR

719	CPSECTCLDTVVRCSNKGLKVLPKGIPKD		NF	4

SHIFT -22!

726	LDTVVRCSN KGLKVLPKGIPKD			LRR1	2.4
748	VTELYLDGN QFTLVPKELSNYKH		LRR2	3.4
771	LTLIDLSNN RISTLSNQSFSNMTQ		LRR3	3.4
795	LLTLILSYN RLRCIPPRTFDGLKS		LRR4	4
819	LRLLSLHGN DISVVPEGAFNDLSA		LRR5	4
843	LSHLAIGAN				LRR6	3.4
852	PLYCDCNMQWLSDWVKSEYKEPGIARCAGPGEMADKLLLTTPSKKFTCQ		CF1_short	3

GPVDITIQAKCNPCLSNPCKNDGTCNNDPVDFYRCTCPYGFKGQDCDVPIHACISNPCKH
GGTCHLKEGENAGFWCTCADGFEGENCEVNIDDCEDNDCENNSTCVDGINNYTCLCPPEY
TGELCEEKLDFCAQDLNPCQHDSKCILTPKGFKCDCTPGYIGEHCDIDFDDCQDNKCKNG
AHCTDAVNGYTCVCPEGYSGLFCEFSPPMVLPRTSPCDNFDCQNGAQCIIRINEPICQCL
PGYLGEKCEKLVSVNFVNKESYLQIPSAKVRPQTNITLQIATDEDSGILLYKGDKDHIAV
ELYRGRVRASYDTGSHPASAIYSVETINDGNFHIVELLTLDSSLSLSVDGGSPKVITNLS
KQSTLNFDSPLY

1273	VGGMPGKNN VASLRQAPGQNGTSFH		LRR1	2.2
1298	GCIRNLYIN SELQDFRKMPMQTGILPGCEP		LRR2	2.4

CHKKVCAHGMCQPSSQSGFTCECEEGWMGPLCDQRTNDPCLGNKCVHGTCLPINAFSYSC
KCLEGHGGVLCDEEEDLFNPCQMIKCKHGKCRLSGVGQPYCECNSGFTGDSCDREISCRG
ERIRDYYQKQQGYAACQTTKKVSRLECRGGCAGGQCCGPLRSKRRKYSFECTDGSSFVDE
VEKVVKCGCARCAS

>ENSMUSP00000063882

MLAPGGGPEQRSKLVLQWRQVSWITCWIALCAVEVIPA

39	CPFSCTCDSRSLEVDCSGLGLTTVPPDVPAA		NF	4

SHIFT -16!

54	CSGLGLTTV PPDVPAATQSLL			LRR1	2
75	LLNNKLSAL PSWAFANLSNLQRLD		LRR2	2
99	LSNNFLDQL PRSIFEDLVNLTELQLRNNSI		LRR3	2

SHIFT -25!

104	LDQLPRSIF EDLVNLTELQLRNNSIRT		LRR1	2
131	LDRDLLQHS PLLRHLDLSINGLAQLPPG		LRR2	2.4
159	LFDGLLALR SLSLRSNRLQSLDRLTFEP		LRR3	2.4
187	LASLQLLQV GDNPWECDCNLREFKHWLEWF		LRR4	3

SYRGGR

223	LDQLACTLP KELRGKDMRAVPMEMFNY		LRR1	2
250	CSQLEDENN SAGLDAPGPPCTKASPEPPKP		LRR2	2

KPGAEPEPEPSTACPQKQRYRPVSVRRAIGTVIIAGVVCGIVCIMMVVAAAYGCIYASLM
AKYHRELKKRQPLMGDPEGEHEDQKQISSVA

>AY182027

MGLHFKWPLGAPMLAAIYAMSVVLKMLPALGMA

34	CPPKCRCEKLLFYCDSQGFHSVPNATD		NF	4
61	KGSLGLSLR HNHITALERDQFASFSQ		LRR1	2
87	LTWLHLDHN QISTVKEDAFQGLYK		LRR2	4
111	LKELILSSN KIFYLPNTTFTQLIN		LRR3	4
135	LQNLDLSFN QLSSLHPELFYGLRK		LRR4	4
159	LQTLHLRSN SLRTIPVRLFWDCRS		LRR5	4
183	LEFLDLSTN RLRSLARNGFAGLIK		LRR6	4
207	LRELHLEHN QLTKINFAHFLRLSS		LRR7	4
231	LHTLFLQWN KISNLTCGMDWTWST		LRR8	4
255	LEKLDLTGN EIKAIDLTVFETMPN		LRR9	4
279	LKILLMDNN KLNSLDSKILNSLKS		LRR10	3.4
303	LTTVGLSGN LWECSPRVCALASWLGSFQGR		LRR11	3.4

WEHSILCHSPDHTQGEDILDAVHGFQLCWNLSTTVTAMATTYRDPTTEYTKISSSSYHVG
DKEIPTTAGIAVTTEEHFPEPDNAIFTQRVITGTMALLFSFFFIIFIVFISRKCCPPTLR
RIRQCSMIQNHRQLRSQTRLHMSNMSDQGPYNEYEPTHEGPFIIINGYGQCKCQQLPYKE
CEV

>ENSMUSP00000045770

MMPPWLLARTLIMALFFSCLTPGSLNPCIEVVPNITYQCMDQKLSKVPDDIPSS

55	TKNIDLSFN PLKILKSYSFSNFSE		LRR1	2.4
79	LQWLDLSRC EIETIEDKAWHGLHH		LRR2	3.4
103	LSNLILTGN PIQSFSPGSFSGLTS		LRR3	4
127	LENLVAVET KLASLESFPIGQLIT		LRR4	2.8
151	LKKLNVAHN FIHSCKLPAYFSNLTN		LRR5	3.4
176	LVHVDLSYN YIQTITVNDLQFLRENP		LRR6	3.4
202	QVNLSLDMS LNPIDFIQDQAFQGIK		LRR7	2.4
227	LHELTLRGN FNSSNIMKTCLQN			LRR8	4
249	LAGLHVHRL ILGEFKDERNLEIFEPSIMEG		LRR9	2.4

SHIFT -25!

254	VHRLILGEF KDERNLEIFEPSIMEGLCDVT		LRR1	2.4
284	IDEFRLTYT NDFSDDIVKFHCLANVSAMSL		LRR2	2.2

AGVSIKYLEDVPKHFK

330	WQSLSIIRC QLKQFPTLDLPF			LRR1	2.2
351	LKSLTLTMN KGSISFKKVALPS			LRR2	4
373	LSYLDLSRN ALSFSGCCSYSDLGTNS		LRR3	4
399	LRHLDLSFN GAIIMSANFMGLEE		LRR4	4
422	LQHLDFQHS TLKRVTEFSAFLSLEK		LRR5	2.8
447	LLYLDISYT NTKIDFDGIFLGLTS		LRR6	2.8
471	LNTLKMAGN SFKDNTLSNVFANTTN		LRR7	3.4
496	LTFLDLSKC QLEQISWGVFDTLHR		LRR8	3.4
520	LQLLNMSHN NLLFLDSSHYNQLYS		LRR9	3.4
544	LSTLDCSFN RIETSKGILQHFPKS		LRR10	3
568	LAFFNLTNN				LRR11	3.4
577	SVACICEHQKFLQWVKEQKQFLVNVEQMTCATPVEMNTSLVLDFNNSTCYMY		CF1	4

KTIISVSVVSVIVVSTVAFLIYHFYFHLILIAGCKKYSRGESIYDAFVIYSSQNED

685	WVRNELVKN LEEGVPRFHLCLHYRDF		LRR1	2.4
711	IPGVAIAAN IIQEGFHKSRKVIVVVSRHFI		LRR2	2.2

QSRWCIFEYEIAQTWQFLSSRSGIIFIVLEKVEKSLLRQQV

782	ELYRLLSRN TYLEWEDNPLGRHIFWRR		LRR1	2
809	LKNALLDGK ASNPEQTAEEEQETATWT		LRR2	2.4


>ENSMUSP00000045142

MRALCLLCWAVLLNLVRA

19	CPEPCDCGEKYGFQIADCAYRDLEGVPPGFPAN		NF	4
52	VTTLSLSAN RLPGLPEGAFREVPL		LRR1	3.4
76	LQSLWLAHN EIRSVAIGALAPLSH		LRR2	4
100	LKSLDLSHN LLSEFAWSDLHNLSA		LRR3	4
124	LQLLKMDSN ELAFIPRDAFSSLSA		LRR4	3.4
148	LRSLQLNHN RLHALAEGTFAPLTA		LRR5	4
172	LSHLQINDN				LRR6	3.4
181	PFDCTCGIVWFKTWALASAVSIPEQDNIACTTPHVLKGIPLGRLPPLPCSAP		CF1	4

SHIFT -12!

221	LGRLPPLPC SAPSVQLSYQPSQDGAELRP		LRR1	2.4
250	GFVLALHCD VDGQPVPQLHWHIHTPGGTVE		LRR2	2.4

IASPNVGTDGRALPGALATSGQPRFQAFANGSLLIPDFGKLEEGTYSCLATNELGSAESS
VNVALATPGEGGEDAVGHKFHGKAVEGKGCYTVDNEVQPSGPEDNVVIIYLSRAGPPEAA
IAADGRPAQQFSGILLLGQSLLVLSFFYF

>ENSMUSP00000066777

MPLKHYLLLLVSCQAWAAGLAYYG

25	CPSECTCSRASQVECTGAQIVAMPSPLPWN		NF	4

SHIFT -9!

46	AMPSPLPWN AMSLQILNTHITELPEDKF		LRR1	2.4
74	LNISALIAL KMEKNELANIMPGAFRNLGS		LRR2	2
103	LRHLSLANN KLKNLPVRLFQDVNN		LRR3	4
127	LETLLLSNN QLVQIQPAQFSQFSN		LRR4	4
151	LKELQLYGN NLEYIPEGVFDHLVG		LRR5	4
175	LTKLNLGNN GFTHLSPRVFQHLGN		LRR6	4
199	LQVLRLYEN RLSDIPMGTFDALGN		LRR7	4
223	LQELALQEN QIGTLSPGLFHNNRN		LRR8	4
247	LQRLYLSNN HISHLPPGIFMQLPH		LRR9	4
271	LNKLTLFGN SLKELSPGVFGPMPN		LRR10	4
295	LRELWLYNN HITSLPDNAFSHLNQ		LRR11	4
319	LQVLILSHN QLSYISPGAFNGLTN		LRR12	4
343	LRELSLHTN ALQDLDGNVFRSLAN		LRR13	4
367	LRNVSLQNN RLRQLPGSIFANVNG		LRR14	3.4
391	LMTIQLQNN NLENLPLGIFDHLGN		LRR15	3.4
415	LCELRLYDN PWRCDSNILPLHDWLI		LRR16	4
440	LNRARLGTD TLPVCSSPASVRGQSLVIINV		LRR17	2.4

NFPGPSVQGPETPEVSSYPDTSSYPDSTSISSTTEITRSTDDDYTDLNTIEPIDDRNTWG
MTDAQSGLAIAAIVIGIIALACSLAACICCCCCKKRSQAVLMQMKAPNEC

>ENSMUSP00000059570

MGPRGRQSPSATLAPSQGSCFFILFCLRLGAS

33	CPQACQCPDHAGAVAVHCSSRGLQEIPRDIPAD		NF	4
66	TVLLKLDAN RISRVPNGAFQHLPQ		LRR1	3
90	LRELDLSHN AIEAIGPAAFSGLAGG		LRR2	4
115	LRLLDLSHN RIRRIPKDALGKL			LRR3	4
137	SAKIRLSHN				LRR4	2.4
146	PLHCECALQEALWELKLDPDSVDEIACHTSAQEQFVGKPLIQVLDSGAS		CF1_short	3

FCSTHRKTTDVAM

208	LVTMFGWFT MVIAYVVYYVRHNQEDARRH		LRR1	2.2
237	LEYLKSLPS APVSKEPLSPVP			LRR2	2.4


>ENSMUSP00000029623

MLRALWLFWILVAITVLFSKRCSAQESLSCDASGVCDGRSRSFTSIPSGLTAA

54	MKSLDLSFN KITYIGHGDLRACAN		LRR1	3.4
78	LQVLMLKSS RINTIEGDAFYSLGS		LRR2	3.4
102	LEHLDLSDN HLSSLSSSWFGPLSS		LRR3	4
126	LKYLNLMGN PYQTLGVTSLFPNLTN		LRR4	4
151	LQTLRIGNV ETFSEIRRIDFAGLTS		LRR5	2.4
176	LNELEIKAL SLRNYQSQSLKSIRD		LRR6	2.4
200	IHHLTLHLS ESAFLLEIFADILSS		LRR7	2.8
224	VRYLELRDT NLARFQFSPLPVDEVSSP		LRR8	2.8
251	MKKLAFRGS VLTDESFNELLK			LRR9	2.2
272	LLRYILELS EVEFDDCTLNGLGDFNPSESD		LRR10	2.4

VVSELGKVETVTIRRLHIPQFYLFYDLSTVYSLLEKVKRITVENSKVFLVPCSFSQH

359	LKSLEFLDL SENLMVEEYLKNSACKGAWPS		LRR1	2.4
389	LQTLVLSQN HLRSMQKTGEILLTLKN		LRR2	4
415	LTSLDISRN TFHPMPDSCQWPEK		LRR3	3.4
438	MRFLNLSST GIRVVKTCIPQT			LRR4	2.8
459	LEVLDVSNN NLDSFSLFLPRLQELYISRNK		LRR5	3.4
489	LKTLPDASL FPVLLVMKIRENAVSTFSKDQ		LRR6	2

SHIFT -22!

497	LFPVLLVMK IRENAVSTFSKDQLGSFPK		LRR1	2.4
525	LETLEAGDN				LRR2	3.4
534	HFVCSCELLSFTMETPALAQILVDWPDSYLCDSPPRLHGHRLQDARPSVLECH		CF1_short	3

SHIFT -17!

570	LHGHRLQDA RPSVLECHQAALVSGVCCA		LRR1	2
598	LLLLILLVG ALCHHFHGLWYLRMMWAWLQA		LRR2	3

KRKPKKAPCRDVCYDAFVSYSEQDSHWVENLMVQQLENSDP

669	PFKLCLHKR DFVPGKWIIDNIIDSIEKSH		LRR1	2
698	KTVFVLSEN FVRSEWCKYELDFS		LRR2	2.4
721	HFRLFDENN DAAILVLLEPIERKAIPQR		LRR3	2
749	FCKLRKIMN TKTYLEWPLDEGQQEVFWVNL		LRR4	2.4

RTAIKS

>AY182031

MGFRLITQLKGMSVFLVLFPTLLLVMLTGAQRA

34	CPKNCRCDGKIVYCESHAFADIPENISGG		NF	4
63	SQGLSLRFN SIQKLKSNQFAGLNQ		LRR1	3
87	LIWLYLDHN YISSVDEDAFQGIRR		LRR2	4
111	LKELILSSN KITYLHNKTFHPVPN		LRR3	4
135	LRNLDLSYN KLQTLQSEQFKGLRK		LRR4	4
159	LIILHLRSN SLKTVPIRVFQDCRN		LRR5	4
183	LDFLDLGYN RLRSLSRNAFAGLLK		LRR6	4
207	LKELHLEHN QFSKINFAHFPRLFN		LRR7	4
231	LRSIYLQWN RIRSVSQGLTWTWSS		LRR8	3.4
255	LHTLDLSGN DIQAIEPGTFKCLPN		LRR9	4
279	LQKLNLDSN KLTNVSQETVNAWIS		LRR10	4
303	LISITLSGN MWECSRSICPLFYWLKNFKGN		LRR11	3.4

KESTMICAGPKHIQGEKVSDAVETYNICSDVQVVNTERSHLAPQTPQKPPFIPKPTIFKP
DAVPATLEAVSPSPGFQIPGTDHEYEHVSFHKII

427	AGSVALFLS VAMILLVIYVSWKRYPASMKQ		LRR1	2.2
457	LQQHSLMKR RRKKARESERQMNSPLQEYYV		LRR2	2

DYKPTNSETMDISVNGSGPCTYTISGSRECEV

>ENSMUSP00000067130

MEFPPLWLCLGFHFLIVEWRSGPGTATAASQGGCKVVDGVAD

43	CRGLNLASV PSSLPPHSRMLILD		LRR1	2
66	ANPLKDLWN HSLQAYPRLENLS			LRR2	2.4
88	LHSCHLDRI SHYAFREQGHLRNLV		LRR3	2
112	LADNRLSEN YKESAAALHTLLG			LRR4	3
134	LRRLDLSGN SLTEDMAALMLQNLSS		LRR5	4
159	LEVVSLARN TLMRLDDSIFEGLEH		LRR6	3.4
183	LVELDLQRN YIFEIEGGAFDGLTE		LRR7	4
207	LRRLNLAYN NLPCIVDFSLTQ			LRR8	4
228	LRFLNVSYN ILEWFLAAREEVAFE		LRR9	3.4
252	LEILDLSHN QLLFFPLLPQCGK			LRR10	4
274	LHTLLLQDN NMGFYRELYNTSSPQEMV		LRR11	4
301	AQFLLVDGN VTNITTVNLWEEFSSSDLSA		LRR12	2.8
330	LRFLDMSQN QFRHLPDGFLKKTPS		LRR13	3.4
354	LSHLNLNQN CLKMLHIREHEPPGA		LRR14	4
378	LTELDLSHN QLAELHLAPGLTGSLRN		LRR15	4
404	LRVFNLSSN QLLGVPTGLFDNASS		LRR16	3.4
428	ITTIDMSHN QISLCPQMVPVDWEGPPSCVD		LRR17	2.2

FRNMGS

464	LRSLSLDGC GLKALQDCPFQGTS		LRR1	3.4
487	LTHLDLSSN WGVLNGSISPLWAVAPT		LRR2	4
513	LQVLSLRDV GLGSGAAEMDFSAFGN		LRR3	3
538	LRALDLSGN SLTSFPKFKGSLA			LRR4	4
560	LRTLDLRRN SLTALPQRVVSEQPLRG		LRR5	4
586	LQTIYLSQN PYDCCGVEGWGALQQHFKT		LRR6	3.4
614	VADLSMVTC NLSSKIVRVVELPEGLPQGCK		LRR7	2.2

WEQVDTGLFYLVLILPSCLTLLVACTVVFLTFKKPLLQVIKSRCHWSSIY

>ENSMUSP00000035831

M

2	LWLLALGGP HQAWGF		LRR1	3
17	CPSECSCSLRILSDGSKARTVVCSDPDLTLPPASIPPD		NF	4

SHIFT -30!

25	LRILSDGSK ARTVVCSDPDLTLPPASIPPD		LRR2	2
55	TCKLRLERT AIRRVPGETFRPLSR		LRR3	2.4
79	LEQLWLPYN ALSELSALMLRGLRR		LRR4	4
103	LRELRLPGN RLVTFPWAALRDTPQ		LRR5	4
127	LQLLDLQAN RLSTLPPEAAHFLEN		LRR6	4
151	LTFLDLSNN QLMRLPEELLDVWAHLKTGPF		LRR7	4

SHIFT -25!

156	LSNNQLMRL PEELLDVWAHLKTGPFLSGH		LRR1	2
185	HARLILGLQ DNPWVCDCRLYDLVH		LRR2	2
209	LLDGWVSSN LIFIEARLRCASPRSLAGVA		LRR3	2.8
238	FSQLELRKC QSPELRPGVTSIISP		LRR4	2.8
262	LGSTVLLRC GATGIPGPEMSWRRANGRPLN		LRR5	2.4

GTVHQEVSSDGSS

305	WTLLDLPVV SLFDSGDYICQAKNFLGASET		LRR1	2.4
335	LISLIVTEP QTSTGYSGIPGVLWARTGE		LRR2	2.4
363	GAEAAAYNN KLVARHVPHMPEHVALATKPS		LRR3	2.2

MPSIKEELALQNFQMDVPGEFSREPSEHQEAQMVRSLKVVGDTYHSVSLVWKAPQAGNTT
AFSVLYAVFGHRDMRRMTVEPGKTSVTIEGLAPKTKYVACVCVRGLVPTKEQCVIFSTDE
VVDAEGTQRLINMVVISVAAIIALPPTLLVCCGALRRRCHKCRTGGSAEASGAYVNLERL
GHSEDSSEVLSRSSLSEGDRLLSARSSLDSQVLGVRGGRRINEYFC

>ENSMUSP00000050039

M

2	APPLLLLLL ASGAAACPLPCVCQNLSES		LRR1	2.4
30	LSTLCAHRG LLFVPPNVDRRTVELRLADNF		LRR2	2.4

SHIFT -10!

50	TVELRLADN FIQALGPPDFRNMTG		LRR1	3
74	LVDLTLSRN AITRIGARSFGDLES		LRR2	4
98	LRSLHLDGN RLVELGSSSLRGPVN		LRR3	4
122	LQHLILSGN QLGRIAPGAFDDFLDS		LRR4	4
147	LEDLDVSYN NLRQVPWAGIGSMPA		LRR5	3.4
171	LHTLNLDHN LIDALPPGVFAQLSQ		LRR6	4
195	LSRLDLTSN RLATLAPDPLFSRGRDAEAS		LRR7	4
224	PSPLVLSFS GNPLHCNCELLW			LRR8	2.4
245	LRRLARPDD LETCASPPTLAGRYFWAVPEG		LRR9	2

EFSCEPPLIARHTQR

290	LWVLEGQRA TLRCRALGDPVPTMHWV		LRR1	2.4
316	GPDDRLVGN SSRAWAFPNGTLEIGVTGAGD		LRR2	2.4

AGAYTCIATNPAGEATARVELRVLALPHGGNTSAEGGRPGPSDIAASARTAAEGEGTLES
EPAVQVTEVTATSGLVSWGLGRPADP

432	VWMFQIQYN SSEDETLIYRIVPASSHHFL		LRR1	2.2
461	LKHLVPGAD YDLCLLALSPAAGPSD		LRR2	2
486	LTATRLLGC AHFSTLPATPLCHA		LRR3	2.4
509	LQAHVLGGT LTVAVGGVLVAALLVFTVAL		LRR4	2.4
538	LVRGRGAGN GRLPLKLSHVQSQTNGGTSPM		LRR5	2.8

SHIFT -15!

553	LSHVQSQTN GGTSPMPKSHPPRSPPPRPQR		LRR1	2.4
583	SCSLDLGDT GGCYGYARRLGGAWARRSHSV		LRR2	2.4

HGGLLGAGCRGVGGSAERLEESVV

>ENSMUSP00000035999

MAAAPRGIWEQRRLGCG

18	LGPLARLLI LAQALRLLPAARAGL		LRR1	2
42	CPAPCACRLPLLDCSRRKLPAPSWRALSG		NF	4

SHIFT -24!

47	ACRLPLLDC SRRKLPAPSWRALSGPLPSD		LRR2	2.8
76	ISSLDLSHN RLSNWNNTLESQT			LRR3	3.4
98	LQEVKMNYN ELTEIPYFGEPTPN		LRR4	2.8
121	ITLLSLVHN LIPEINAEAFELYSA		LRR5	3.4
145	LESLDLSSN IISEIKTSSFPRMS		LRR6	4
168	LKYLNLSNN RISTLEAGCFDNLSDS		LRR7	4
193	LLVVKLNRN RISMIPPKVFKLPH		LRR8	3.4
216	LQFLELKRN RIKIVEGLTFQGLDS		LRR9	4
240	LRSLKMQRN GISKLKDGAFFGLNN		LRR10	3.4
264	MEELELEHN NLTGVNKGWLYGLRM		LRR11	3.4
288	LQQLYMSQN AIEKISPDAWEFCQR		LRR12	3.4
312	LSELDLSYN QLTRLDESAFVGLSL		LRR13	4
336	LERLNLGDN RVTHIADGVFRFLSN		LRR14	4
360	LQTLDLRNN DISWAIEDASEAFSGLKS		LRR15	4
387	LTKLILQGN RIKSVTQKAFIGLES		LRR16	4
411	LEYLDLNNN AIMSIQENAFSQTH		LRR17	4
434	LKGLVLNTS				LRR18	3.4
443	SLLCDCHLKWLLQWLVDNNFHHSVNVSCAHPEWLAGQSILNVDLKDFVCD		CF1_short	3

DFLKPQIRTHPESTIALRGVNVTLTCTAVSSSDSPMSTIWRKDSEILYDVDIENFVRYRQ
QDGEALEY

561	TSVLRLFSV NFTDEGKYQCIVTNHFGSNY		LRR1	2
590	SQKAKLTVN EMPSFLKTPMDLTIRTGAMAR		LRR2	2.4

LECAAEGHPTPQISWQKDGGTDFPAARERRMHVMPEDDVFFIANVKIEDMGIYSCMAQNI
AGGLSANASLTVLETPSFIRPLEDKTVTRGETAVLQCIAGGSPAPRLNWTKDDGPLLVTE
RHFFAAANQLLIIVDAGLEDAGKYTCLMSNTLGTERGHIYLNVISSPNCDSSQSSIGHED
DGWTTVGIVIIVVVCCVVGTSLIWVIVIYHMRRKNEDYSITNTEELNLPADIPSYLSSQG
TLSEPQEGYSNSEAGSHQQLMPPANGYTHRGTDGGAGTRVICSDCYDNANIYSRTREYCP
YTYIAEEDVLDQA

933	LSSLMVQMP KETFLSHPPQDAAN		LRR1	2.4
956	LESLIPSAE REPAAFPTNHERMTENLPFSQ		LRR2	2

RSSEIFQRPLWNMNRELGLLPFSQQPVLESPELTERDPNCSSPVTCRRLHDHAFDFSRTR
IIQDGTEGT

>ENSMUSP00000041499

MHVCCPPVTLEQDLHRKMHSWMLQTLAFAVTSLVLSCAETIDYYGEI

48	CDNACPCEEKDGILTVSCENRGIISLSEISPPR		NF	4

FP

83	IYHLLLSGN LLSRLYPNEFVNYTG		LRR1	3.4
107	ASILHLGSN VIQDIETGAFHGLRG		LRR2	3.4
131	LRRLHLNNN KLELLRDDTFLGLEN		LRR3	4
155	LEYLQVDYN YISVIEPNAFGKLHM		LRR4	3.4
179	LQVLILNDN LLSGLPNNLFRFVP		LRR5	4
202	LTHLDLRGN RLKLLPYVGLLQHMDK		LRR6	4
227	VVELQLEEN				LRR7	3.4
236	PWNCSCELISLKDWLDSISYSALVGDVVCETPFRLHGRDLDEVSKQELCPR		CF1_short	3

SHIFT -44!

243	LISLKDWLD SISYSALVGDVVCETPFR		LRR1	2
270	LHGRDLDEV SKQELCPRKLISDYEMRPQTP		LRR2	2

SHIFT -17!

283	LCPRKLISD YEMRPQTPLSTTGY		LRR1	2
306	LHTTPASVN SVATSSSAVYKPPLKPPKGTR		LRR2	2.4

QPNKPRVRPTSRQPSKDLGYSNYGPSIAYQTKSPVPLE

374	CPTACTCNLQISDLGLNVNCQERKIESIAELQPKP		NF	4

SHIFT -27!

382	LQISDLGLN VNCQERKIESIAELQPKPYN		LRR1	3
411	PKKMYLTEN YITVVRRTDFLEATG		LRR2	2.4
435	LDLLHLGNN RISMIQDRAFGDLGN		LRR3	4
459	LRRLYLNGN RIERLSPELFYGLQS		LRR4	4
483	LQYLFLQYN LIREIQAGTFDPVPN		LRR5	4
507	LQLLFLNNN QLQAMPSGVFSGLT		LRR6	4
530	LLRLNLRGN SFTSLPVSGVLDQLTS		LRR7	4
555	LIQIDLHDN				LRR8	3.4
564	PWDCTCDVVGMKLWIEQLKVGVLVDEVICKAPKKFAETYMRSIKSELLCPD		CF1_short	3

SHIFT -60!

555	LIQIDLHDN PWDCTCDVVGMKLWIEQ		LRR8	3.4
581	LKVGVLVDE VICKAPKKFAETYMRSIKSEL		LRR9	2.4

LCPDYSDVVVSTPTPSSIQVPSRTNAATPAVRLNSTGTPAGLGAGTGASSVP

663	LSVLILSLL LVFIMSVFVAAG			LRR1	3
684	LFVLVMKRR KKNQSDHTSTNNSDVSSFNMQ		LRR2	2.4

YSVYGGGGGGGGGHPHAHVHHRGPALPKVKTPAGHVYEYIPHPLGHMCKNPIYRSREGNS
VEDYKDLHELKVTYSSNHHLQQQPPPPPQQPQQQPPPQMQMQPGEEERRESHHLRSPAYS
VSTIEPREDLLSPVQDADRFYRGILEPDKHCSTTPAGSSLPEYPKFPCSPAAYTFSPNYD
LRRPHQYLHPGAGESR

910	LREPVLYSP PGAVFVEPNRNE			LRR1	2
931	YLELKAKLN VEPDYLEVLEKQTTFSQF		LRR2	2.4


>ENSMUSP00000044094

MLSGVWF

8	LSVLTVAGI LQTESRKTAKDI		LRR1	2.4
29	CKIRCLCEEKENVLNINCENKGFTTVSLLQPPQ		NF	4

SHIFT -25!

37	EKENVLNIN CENKGFTTVSLLQPPQYR		LRR2	2
64	IYQLFLNGN LLTRLYPNEFVNYSN		LRR3	3.4
88	AVTLHLGNN GLQEIRPGAFSGLKT		LRR4	3.4
112	LKRLHLNNN KLEVLREDTFLGLES		LRR5	4
136	LEYLQADYN YISTIEAGAFSKLNK		LRR6	3.4
160	LKVLILNDN LLLSLPSNVFRFVL		LRR7	4
183	LTHLDLRGN RLKVMPFAGVLEHIGG		LRR8	4
208	IMEIQLEEN				LRR9	2.8
217	PWNCTCDLLPLKAWLDTITVFVGEIVCETPFRLHGKDVTQLTRQDLCPR		CF1_short	3

SHIFT -9!

257	LTRQDLCPR KSASGDSSQRSSHSDTH		LRR1	2
283	VQRLTPTTN PALNPTRAPKASRPPKMRNRP		LRR2	2.4

TPRVTVSKDRQSFGPIMVYQTKSPVALT

341	CPSSCVCTSQSSDNGLNVNCQERKFTNISDLQPKP		NF	4

SHIFT -25!

351	SSDNGLNVN CQERKFTNISDLQPKPTS		LRR1	2
378	PKKLYLTGN YLQTVYKNDLLEYSS		LRR2	3
402	LDLLHLGNN RIAVIQEGAFTNLTS		LRR3	4
426	LRRLYLNGN YLEVLYPSMFDGLQS		LRR4	4
450	LQYLYLEYN VIKEIKPLTFDALIN		LRR5	4
474	LQLLFLNNN LLRSLPDNIFGGTA		LRR6	4
497	LTRLNLRNN HFSHLPVKGVLDQLPA		LRR7	4
522	FIQIDLQEN				LRR8	2.8
531	PWDCTCDIMGLKDWTEHANSPVIINEVTCESPAKHAGEILKFLGREAICPE		CF1_short	3

SHIFT -41!

541	LKDWTEHAN SPVIINEVTCESPAKHAGEI		LRR1	2.4
570	LKFLGREAI CPENPNLSDGTILSMNHNTDT		LRR2	2

PRSLSVSPSSYPELHTEVP

619	LSVLILGLL VVFILSVCFGAG			LRR1	3
640	LFVFVLKRR KGVPNVPRNATNLDVSSFQLQ		LRR2	2.4

YGSYNTETNDKADGHVYNYIPPPVGQMCQNPIYMQKEGDPVAYYRNLQDFSYGNLEEKKE
EPATLAYTISATELLEKQATPREPELLYQNIAERVKELPSAGLVHYNFCTLPKRQFAPSY
ESRRQNQDRINKTVLYGTPRKCFVGQSKPDHPLLQAKPQSEPDYLEVLEKQTAISQL

>XP_001001535

MERPQSSIWVFMLLLFMVLLQSPAWHVAAQR

32	CPQTCVCDNSRRHVTCRHQNLTEVPNTIPEL		NF	4

SHIFT -14!

49	HQNLTEVPN TIPELTQRLDLQGNI		LRR1	2
73	LKVLPAAAF QDLPHLTHLDLRNCQVEMVAE		LRR2	2.4

SHIFT -19!

84	LPHLTHLDL RNCQVEMVAEGAFRGLGR		LRR1	2
111	LLLLNLASN RLSTLPQEALDGLGS		LRR2	4
135	LRRLELEGN MLEELRPGTFGALGS		LRR3	4
159	LTTLNLAHN ALVYLPAMAFQGLLR		LRR4	4
183	TRWLQLSHN ALSVLAPEALAGLPA		LRR5	3
207	LRRLSLHHN ELQALPGAALSQARS		LRR6	4
231	LARLELGHN PLTYTGEEDGLALPG		LRR7	4
255	LRELALDHG SLQALGPRAFAHCPR		LRR8	3
279	LHTLDLRGN QLTTLPPLQVPGQ			LRR9	4
301	LRRLRLQGN				LRR10	4
310	PLWCACHARPLLEWLVRARVRSDGACRGPRRLRGEALDTLRPSDLRCP		CF1_short	3

SHIFT -37!

321	LEWLVRARV RSDGACRGPRRLRGEA		LRR1	2
346	LDTLRPSDL RCPGDAAAGDGDGDEDEDRPA		LRR2	2

GPRAPPLRSPHGEAAWATP

395	CPPACACVAETRHSTCDGRGLQAVPRGFPND		NF	4
426	TQLLDLRRN HFPSVPRAAFPGLRH		LRR1	3
450	LVSLHLQHC GVAELEPGALAGLDR		LRR2	3.4
474	LLYLYLSHN QLSGLSAAALEGAPN		LRR3	4
498	LGYLYLEHN RFLRIPGTALRALPT		LRR4	4
522	LVSLHLQDN AVDRLAPGDLAGARA		LRR5	4
546	LRCLYLSGN HITQVSPGALGPARE		LRR6	4
570	LEKLHLDRN RLREVPTGALEGLPA		LRR7	4
594	LKELQLSGN PLRALPDGAFQPVGRS		LRR8	4
619	LQQLFLNSS DLEQISPRAFSGLGKG		LRR9	3.4
644	LRSLYLHKN QLQSLPAPLGLSG			LRR10	4
666	LELVDLSGN				LRR11	3.4
675	PFHCDCQLLPLHRWLTGLNLRVGATCATPPSVRGQKVKVAAPVFEACP		CF1_short	3

SHIFT -57!

666	LELVDLSGN PFHCDCQLLPLHRW		LRR11	3.4
689	LTGLNLRVG ATCATPPSVRGQKVKVAAPVF		LRR12	3

EACPGWTARKAKRTPTSRGSARRTPSLSRH

>ENSMUSP00000057005

MRW

4	TLMLQLLQL LLQLLMAQSQSLERISQDRIP		LRR1	2
34	LFRLTQQGD WDSLDRHPTDSLCVGLPAAG		LRR2	2
63	VTTLNLANR SLESLPSCLPRT			LRR3	2.4
84	LRSLDGSHN LLRALSEPVLGRLPE		LRR4	3.4
108	LRVLTLHHN RISVLHWGRDTLAE		LRR5	4
131	LRELDLSHN LLTELPPCAGPSGSS		LRR6	4
155	LRSLALAGN PLRALLPRTFACFPA		LRR7	4
179	LRLLNLSCS ELGHIAQEAFAGVDGGPLAA		LRR8	3.4
208	LELLDLSGT SLERVESGWIRNLPK		LRR9	3.4
232	LKSLFLRKM PRLKTLEGDIFKMTPN		LRR10	3
257	LRQLDCGDS PALTSVHTEIFQDTPN		LRR11	2.4
282	LQVLQFQNC NLSSFGPWNSSQ			LRR12	2.8
303	VLSVSLFGN				LRR13	2.8
312	PLICSCELAWLLVDVNKTVLHRAADTMCEPALGSTGPFSGPLSLSHLSNV		CF1_short	3

CRSDQSTTLLPSNPGRFDHSVFAPRIQGPSIEQSTALSAQPGGSQQNITKVPSLTMTSPT
QGSWMYKDASEETAQSTNSELVYSPSRALPGAASSGAEQ

461	TATHILEPN ISSASTPLVSKY			LRR1	2
482	LEPLPTSPN PRSLPQTKQRTQATPRALHTD		LRR2	3

PPQDEIPVLLLDDDSEEEETRDQVAAPPQDVSCEYHPCKHLQTPCAELQRRFRCRCPGLS
GEDTTPDPPTLQGVSEVTDTSVLVHWCAPNSVVLWYQIHYVAEGRSGNQSVVDIYATARQ
HPLYKLTPGTTYHVCVLAANRAGLSQSQTSGWRRSCATFTTKPSSVVIFWGLCT

686	ASGLLLVST LVLSVCLWRQRWKPHRQFY		LRR1	2.8
714	DTHLVAFKN PARAEEVTQWE			LRR2	2.4


>ENSMUSP00000006559

MPGAGSRGPSAGDGRLRLARLALVLLGWVSASAPSSSVPSSSTSPAAFLASGSAQPPPAE
R

62	CPAACECSEAARTVKCVNRNLLEVPADLPPY		NF	4
93	VRNLFLTGN QMTVLPAGAFARQPPLAD		LRR1	3.4
120	LEALNLSGN HLKEVCAGAFEHLPG		LRR2	4
144	LRRLDLSHN PLTNLSAFAFAGSNASVSAP		LRR3	4
173	SPLEELILN HIVPPEDQRQNGSFEGMVAFE		LRR4	2

GMVAAAL

210	RSGLALRGL TRLELASNHFLF			LRR1	2
231	LPRDLLAQL PSLRYLDLRNNS			LRR2	2
252	LVSLTYASF RNLTHLESLHLE			LRR3	2
273	DNALKVLHN STLAEWHGLAHVKVFLDNNPW		LRR4	2.4

VCDCYMADMVAWLKETEVVPDKARLTCAFPEKM

336	RNRGLLDLN SSDLDCDAVLPQSLQTSYVFL		LRR1	2.4
366	GIVLALIGA IFLLVLYLNRKGIKKWMHNIR		LRR2	2.4

DACRDHMEGYHYRYEINADPRLTNLSSNSDV

>ENSMUSP00000056669

M

2	ERVLGLLLL LLVHASPAPPEPCE		LRR1	2
25	LDEESCSCN FSDPKPDWSSAFNCLGAADVE		LRR2	2

LYGGGRS

62	LEYLLKRVD TEADLGQFTDIIKSLS		LRR1	2
87	LKRLTVRAA RIPSRILFGALRVLGISG		LRR2	2.4
114	LQELTLENL EVTGTAPPPLLEATGPD		LRR3	3
140	LNILNLRNV SWATRDAWLAELQQWLKPG		LRR4	3
168	LKVLSIAQA HSLNFSCEQVRVFPA		LRR5	2.4
192	LSTLDLSDN PELGERGLISALCPLKFPT		LRR6	4
220	LQVLALRNA GMETPSGVCSALAAARVQ		LRR7	3
247	LQGLDLSHN SLRDAAGAPSCDWPSQ		LRR8	4
272	LNSLNLSFT GLKQVPKGLPAK			LRR9	3.4
293	LSVLDLSYN RLDRNPSPDELPQ			LRR10	4
315	VGNLSLKGN PFLDSESHSEKFNSGVVTAGA		LRR11	3.4

PSSQAVALSGTLALLLGDRLFV

>ENSMUSP00000020087

MKATLIFFLLAQVSWAGPFEQRGLFDFMLEDEASGIIPYDPDNPLISM

49	CPYRCQCHLRVVQCSDLGLDKVPWDFPP		NF	4
77	DTTLLDLQN NKITEIKEGAFKNLKD		LRR1	2
102	LHTLILVNN KISKISPEAFKPLVK		LRR2	4
126	LERLYLSKN QLKELPEKMPRT			LRR3	4
147	LQELRVHEN EITKLRKSDFNGLNN		LRR4	3.4
171	VLVIELGGN PLKNSGIENGAFQGLKS		LRR5	2.8
197	LSYIRISDT NITAIPQGLPTS			LRR6	2.2
218	LTEVHLDGN KITKVDAPSLKGLIN		LRR7	3.4
242	LSKLGLSFN SITVMENGSLANVPH		LRR8	4
266	LRELHLDNN KLLRVPAGLAQHKY		LRR9	4
289	IQVVYLHNN NISAVGQNDFCRAGHPSRKAS		LRR10	2.8
319	YSAVSLYGN PVRYWEIFPNTFRCVYVRSAI		LRR11	2.4

QLGNYK

>ENSMUSP00000059270

MLPPHPSASLSGPRGALSLLL

22	LLLALLSRP ASG		LRR1	2.4
34	CPAPCSCAGTLVDCGRRGLTWASLPAAFP		NF	4

SHIFT -11!

52	LTWASLPAA FPPDTTELVLTGNNLTA		LRR2	2.4
78	LPPGLLDAL PALRAAHLGANPWRCDCR		LRR3	2.4
105	LLPLRAWLA GRPERAPYRDLRCVAPPA		LRR4	2.4
132	LRGRLLPYV AEDELRAACAPGL			LRR5	2
154	LCWGALVAQ LALLVLGLLHAL			LRR6	2.4
175	LLALLLGRL RRLRARARARSIQEFSLTAPL		LRR7	3

VAESARGGAS

>ENSMUSP00000048803

MRASFFWLLPLLLILASVAQGQPTRPKPGIRRKPKPRPTPRFPQAPEPAEPTDLPPPLPP
GPPSVFPD

69	CPRECYCPPDFPSALYCDSRNLRRVPVIPPR		NF	4
100	IHYLYLQNN FITELPLESFQNATG		LRR1	3.4
124	LRWVNLDNN RIRKVDQRVLGKLPS		LRR2	3.4
148	LAFLYMEKN QLEEVPSALPRN			LRR3	3.4
169	LEQLRLSQN LISRIPPGVFSKLEN		LRR4	4
193	LLLLDLQHN RLSDGVFKADTFQGLKN		LRR5	4
219	LMQLNLAHN ILRKMPPKVPQA			LRR6	4
240	IHQLYLDSN KIETIPNGYFKDFPN		LRR7	3.4
264	LAFIRMNYN KLSDRGLPKNSFNISN		LRR8	2.8
289	LLVLHLSHN KISNVPAISNKLEHLYLNNNS		LRR9	4

IEKINGTQICPNNLVAFHDFSSDLENVPHLRYLRLDGNFLKPPIPLDLMMCFRLLQSVVI

>ENSMUSP00000032133

MTT

4	WGLLFLLWP ATTDTQA		LRR1	2.4
20	CPRPCTCQSLETMGLKVNCEGQGLTALPVIPAH		NF	4

SHIFT -24!

29	LETMGLKVN CEGQGLTALPVIPAH		LRR2	3.4
53	TRQLLLANN SLRSVPPGAFDHLPQ		LRR3	3
77	LWDLDVTHN				LRR4	3.4
86	PWHCDCSLTYLRLWLEDHMPEALMHVYCASPDLATRRPLGQLTGYELGSC		CF1_short	3

SHIFT -41!

95	YLRLWLEDH MPEALMHVYCASPDLATRRP		LRR1	2
124	LGQLTGYEL GSCGWKLPPSWAYPGVWWDVS		LRR2	2.4

SHIFT -27!

127	LTGYELGSC GWKLPPSWAYPGVWWDVSLVA		LRR1	2.4
157	VAVLGLILL AGLLNTFTESRN			LRR2	2.4


>ENSMUSP00000068906

MPRMERHQFC

11	SVLLILILL TLVSLTLTGWAWTIPDCI		LRR1	2
38	IADSLLFPN LSYYIPFCTSAPG			LRR2	2.4
60	LHLLASCSN VKNLNQTLKRVPRN		LRR3	3
83	TEVLCLQGM VPTLPAKAFIRFHSLQ		LRR4	2
108	LLRLQLRTT SVTSRTFQGLDQ			LRR5	3.4
129	LQYLFFDHH				LRR6	2.4
138	APCCLSLFLSPNCFESLRSLSSLSFQGYCLTYSQS		CF3	3

SHIFT -44!

129	LQYLFFDHH APCCLSLFLSPNCFESLRS		LRR6	2.4
157	LSSLSFQGY CLTYSQSIYLPTS			LRR7	2.4
179	LRHLTLRNS CLTKFQDLQRLFPDL		LRR8	3.4
203	LLSTSSTPN IKPGAPFLETLDL			LRR9	2
225	SYNLQLKQA GVRDLYGLTLHSLILDGTP		LRR10	2
253	LKALDLTDS GLLHLHFLSLVGTGIEKVPAS		LRR11	3.4

SHIFT -22!

261	SGLLHLHFL SLVGTGIEKVPASLTGYSE		LRR1	2
289	LRALDLGKN QIQNILENGEIPGYKA		LRR2	4
314	LEFLSLHDN HLQTLPTRFLHTLPQ		LRR3	4
338	LQKLNLSMN KLGPILELPEGLFSTN		LRR4	4
363	LKVLDLSYN QLCDVPHGALSLLSQ		LRR5	4
387	LQELWLSGN NISSLSNESLQGLRQ		LRR6	4
411	LRTLDLSWN QIKVLKPGWLSHLPA		LRR7	4
435	LTTLNLLGT YLEYILGIQLQGPKM		LRR8	3.4
459	LRHLQLGSY PILDIYPPWPPT			LRR9	3
480	LLSLEIQAE SCIQFMIHSGQPFLF		LRR10	2.4
504	LENLTLETS ILLLKPDNITIHFPS		LRR11	3.4
528	LRRLTLRGY SFIFSTSQLQRFF			LRR12	3
550	PQQLPLLEH FFIWCENSYAVDLYLFGMPR		LRR13	2
579	LRVLELGYL NFFYESSTMKLEMLLKEVPQ		LRR14	3
608	LQVLALSHL NLRNLSVSSFKS			LRR15	3
629	LQDLKLLLF NSERALEMNSNLQEFIPQMPQ		LRR16	3

YVYFSDVTFTCQCEASWLESWATRAPNTFVYGLEKSICIANASDYSKT

707	LLFSFLATN CPHGTEFWGFLTSFILLLL		LRR1	3
735	LIILPLISC PKWSWLHHLWTLFHTCWWKLC		LRR2	3.4
765	GHRLRGQFN YDVFISYCEEDQAWV		LRR3	2.8
789	LEELVPVLE KAPPEGEGLRLCLPARDFGIG		LRR4	2

NDRMESMIASMGKSRAT

836	LCVLTGQAL ASPWCNLELRLATYHLVARPG		LRR1	2.4
866	TTHLLLLFL EPLDRQRLHSYHRLSRWLQKE		LRR2	2

SHIFT -25!

871	LLFLEPLDR QRLHSYHRLSRWLQKEDYFD		LRR1	2
900	LSQGKVEWN SFCEQLKRRLSKAGQERD		LRR2	2.8


>AK004926.1

MALRTGSPALVVLLAFWVALGPCYLQGTDPGASADAEGPQ

41	CPVTCTCSYDDYTDELSVFCSSRNLTQLPDSIPVS		NF	4

SHIFT -20!

56	LSVFCSSRN LTQLPDSIPVSTRALW		LRR1	2.4
81	LDGNNLSSI PSAAFQNLSSLDFLN		LRR2	2
105	LQGSWLRSL EPQALLGLQNLYHLHLERNL		LRR3	2
134	LRSLAAGLF RHTPSLASLSLGNNL		LRR4	2.4
158	LGRLEEGLF RGLSHLWDLNLGWNS		LRR5	2
182	LVVLPDTVF QGLGNLHELVLAGNKLTY		LRR6	2
209	LQPALLCGL GELRELDLSRNALRSVKANVF		LRR7	2.4

SHIFT -25!

214	LCGLGELRE LDLSRNALRSVKANVFIHLPR		LRR1	2
244	LQKLYLDRN LITAVAPRAFLGMKA		LRR2	4
268	LRWLDLSHN RVAGLLEDTFPGLLG		LRR3	4
292	LHVLRLAHN AITSLRPRTFKDLHF		LRR4	4
316	LEELQLGHN RIRQLGEKTFEGLGQ		LRR5	4
340	LEVLTLNDN QIHEVKVGAFFGLFN		LRR6	4
364	VAVMNLSGN CLRSLPEHVFQGLGR		LRR7	2.8
388	LHSLHLEHS CLGRIRLHTFAGLSG		LRR8	3.4
412	LRRLFLRDN SISSIEEQSLAGLSE		LRR9	4
436	LLELDLTAN QLTHLPRQLFQGLGQ		LRR10	4
460	LEYLLLSNN QLTMLSEDVLGPLQR		LRR11	4
484	AFWLDLSHN RLETPAEGLFSSLGR		LRR12	3.4
508	LRYLNLRNN SLQTFVPQPGLERLWLDANPW		LRR13	4

SHIFT -25!

513	LRNNSLQTF VPQPGLERLWLDANPWDCSCP		LRR1	2
543	LKALRDFAL QNPGVVPRFVQTVCEGDDCQP		LRR2	2

VYTYNNITCAGPANVSGLDLRDISETLFVHC

>ENSMUSP00000066857

MALGRTGAGAAVRARL

17	ALGLALASI LSGPPAAACPTKCTCSAASVD		LRR1	2.4
47	CHGLGLRAV PRGIPRNAERLDLDRNNITRI		LRR2	2

SHIFT -14!

63	AERLDLDRN NITRITKMDFAGLKN		LRR1	3.4
87	LRVLHLEDN QVSIIERGAFQDLKQ		LRR2	4
111	LERLRLNKN KLQVLPELLFQSTPK		LRR3	4
135	LTRLDLSEN QIQGIPRKAFRGVTG		LRR4	4
159	VKNLQLDNN HISCIEDGAFRALRD		LRR5	3.4
183	LEILTLNNN NISRILVTSFNHMPK		LRR6	4
207	IRTLRLHSN				LRR7	3.4
216	HLYCDCHLAWLSDWLRQRRTIGQFTLCMAPVHLRGFSVADVQKKEYVCP		CF1_short	3

GPHSEAPACNANSLS

280	CPSACSCSNNIVDCRGKGLTEIPANLPEG		NF	4
309	IVEIRLEQN SIKSIPAGAFTQYKK		LRR1	2.8
333	LKRIDISKN QISDIAPDAFQGLKS		LRR2	2.8
357	LTSLVLYGN KITEIPKGLFDGLVS		LRR3	4
381	LQLLLLNAN KINCLRVNTFQDLQN		LRR4	4
405	LNLLSLYDN KLQTISKGLFVPLQS		LRR5	4
429	IQTLHLAQN				LRR6	3.4
438	PFVCDCHLKWLADYLQDNPIETSGARCSSPRRLANKRISQIKSKKFRCS		CF1_short	3

GSEDYRNRFSSECFMDLV

505	CPEKCRCEGTIVDCSNQKLARIPSHLP		NF	4
532	EYTTDLRLN DNDISVLEATGIFKKLPN		LRR1	2
559	LRKINLSNN RIKEVREGAFDGAAG		LRR2	3.4
583	VQELMLTGN QLETMHGRMFRGLSS		LRR3	3.4
607	LKTLMLRSN LISCVSNDTFAGLSS		LRR4	4
631	VRLLSLYDN RITTITPGAFTTLVS		LRR5	3.4
655	LSTINLLSN				LRR6	3.4
664	PFNCNCHMAWLGRWLRKRRIVSGNPRCQKPFFLKEIPIQDVAIQDFTCD		CF1_short	3

GNEESSCQLSPR

725	CPEQCTCVETVVRCSNRGLHALPKGMPKD		NF	4
754	VTELYLEGN HLTAVPKELSAFRQ		LRR1	3.4
777	LTLIDLSNN SISMLTNHTFSNMSH		LRR2	3.4
801	LSTLILSYN RLRCIPVHAFNGLRS		LRR3	4
825	LRVLTLHGN DISSVPEGSFNDLTS		LRR4	4
849	LSHLALGTN				LRR5	4
858	PLHCDCSLRWLSEWVKAGYKEPGIARCSSPESMADRLLLTTPTHRFQCK		CF1_short	3

SHIFT -42!

865	LRWLSEWVK AGYKEPGIARCSSPESM		LRR1	2
891	ADRLLLTTP THRFQCKGPVDINIVAKCNAC		LRR2	2.4

LSSPCKNNGTCSQDPVEQYRCTCPYSYKGKDCTVPINTCVQNPCEHGGTCHLSENLRDGF
SCSCPLGFEGQRCEINPDDCEDNDCENSATCVDGINNYACLCPPNYTGELCDEVIDYCVP
EMNLCQHEAKCISLDKGFRCECVPGYSGKLCETNNDDCVAHKCRHGAQCVDEVNGYTCIC
PQGFSGLFCEHPPPMVLLQTSPCDQYECQNGAQCIVVQQEPTCRCPPGFAGPRCEKLITV
NFVGKDSYVELASAKVRPQANISLQVATDKDN

1193	GILLYKGDN DPLALELYQGHVR			LRR1	2.4
1215	LVYDSLSSP PTTVYSVETVNDGQFHSVE		LRR2	2
1243	LVMLNQTLN LVVDKGAPKSLGKLQKQPAVG		LRR3	3

SHIFT -26!

1247	NQTLNLVVD KGAPKSLGKLQKQPAVGSNSP		LRR1	2
1277	LYLGGIPTS TGLSALRQGADRPLGGFHGCI		LRR2	2.2

HEVRINNELQDFKALPPQSLGVSPGCKSCTVCRHGLCRSVEKDSVVCECHPGWTGPLCDQ
EARDPCLGHSCRHGTCMATGDSYVCKCAEGYGGALCDQKNDSASACSAFKCHHGQCHISD
RGEPYCLCQPGFSGHHCEQENPCMGEIVREAIRRQKDYASCATASKVPIMECRGGCGSQC
CQPIRSKRRKYVFQCTDGSSFVEEVERHLECGCRACS

>ENSMUSP00000021346

MRPGSLLLLVLLLALSRSLRGKE

24	CASPPCECHQEDDFRVTCKELHRIPSLPPSTQT		NF	4

LK

59	LIETHLKTI PSLAFSSLPNISRIYLSIDAT		LRR1	2
89	LQRLEPHSF YNLSKMTHIEIRNTRSLTYID		LRR2	2

PDALTE

125	LPLLKFLGI FNTGLRIFPDLTKIYSTDI		LRR1	2.4
153	FFILEITDN PYMTSVPENAFQGLCNE		LRR2	2.8
179	TLTLKLYNN GFTSVQGHAFNGTK		LRR3	3
202	LDAVYLNKN KYLTAIDNDAFGGVYSGPTLL		LRR4	3.4

DVSSTSVTA

241	LPSKGLEHL KELIAKDTWTLKKLPLS		LRR1	2
267	LSFLHLTRA DLSYPSHCCAFKNQKKIR		LRR2	3
294	GILESLMCN ESSIRNLRQRKSVNILRGPIY		LRR3	2.4

QEYEEDPGDNSVGYKQNSKFQESPSNSHYYVFFEEQEDEVVGFGQELKNPQEETLQAFES
HYDYTVCGDNEDMVCTPKSDEFNPCEDIMGYRF

417	LRIVVWFVS LLALLGNIFVLLILLTSHYKL		LRR1	2.2
447	TVPRFLMCN LAFADFCMGVYLLLIASVD		LRR2	2
475	LYTHSEYYN HAIDWQTGPGCNTAGFFTVFA		LRR3	2

SELSVYTLTVITLERWYAITFAMRLDRKIRLRHAYTIMAGGWVS

549	CFLLALLPM VGISSYAKVSICLPMDT		LRR1	2
575	DTPLALAYI VLVLLLNVVAFVVVCSCYVKI		LRR2	2

YITVRNPQYNPRDKDTKIAKRMAVLIFTDFMCMAPIS

642	FYALSALMN KPLITVTNSKILLVL		LRR1	2.8
666	FYPLNSCAN PFLYAIFTKAFQRDVFILLSK		LRR2	2.4

FGICKRQAQAYQGQRVCPNNSTGIQIQKIPQDTRQSLPNMQDTYELLGNSQLAPKLQGQI
SEEYKQTAL

>ENSMUSP00000062096

MVKSLWDSLCNMSQDRKPIVGSFHFVCALALIVGSMTPFSNE

43	LESMVDYSN RNLTHVPKDLPPR			LRR1	2.4
65	TKALSLSQN SISELRMPDISFLSE		LRR2	3
89	LRVLRLSHN RIRSLDFHVFLFNQD		LRR3	4
113	LEYLDVSHN RLQNISCCPMAS			LRR4	3.4
134	LRHLDLSFN DFDVLPVCKEFGNLTK		LRR5	4
159	LTFLGLSAA KFRQLDLLPVAHLH		LRR6	3
182	LSCILLDLV SYHIKGGETESLQIPNTT		LRR7	2.4
209	VLHLVFHPN SLFSVQVNMSVNA			LRR8	2.8
231	LGHLQLSNI KLNDENCQRLMTF			LRR9	3
253	LSELTRGPT LLNVTLQHIETTWKCSVKLFQ		LRR10	2.4

SHIFT -21!

262	LLNVTLQHI ETTWKCSVKLFQFFWPRPVEY		LRR1	2.4
292	LNIYNLTIT ERIDREEFTYSETA		LRR2	2.4
315	LKSLMIEHV KNQVFLFSKEALYSVFAEMN		LRR3	2.4
344	IKMLSISDT PFIHMVCPPSPSS			LRR4	2.2
366	FTFLNFTQN VFTDSVFQGCSTLKR		LRR5	2.8
390	LQTLILQRN GLKNFFKVALMTKNMSS		LRR6	4
416	LETLDVSLN SLNSHAYDRTCAWAES		LRR7	3.4
441	ILVLNLSSN MLTGSVFRCLPPK			LRR8	3.4
463	VKVLDLHNN RIMSIPKDVTHLQA		LRR9	3.4
486	LQELNVASN SLTDLPGCGAFSS			LRR10	3.4
508	LSVLVIDHN SVSHPSEDFFQSCQN		LRR11	3.4
532	IRSLTAGNN				LRR12	2.8
541	PFQCTCELRDFVKNIGWVAREVVEGWPDSYRCDYPESSKGTALRDFHMSPLSCD		CF1_short	3

TVLLTVTIGATMLVLAVT

613	GAFLCLYFD LPWYVRMLCQWTQTRHRARH		LRR1	2.4
642	IPLEELQRN LQFHAFVSYSEHDSA		LRR2	2.4
666	WVKNELLPN LEKDDIRVCLHERNFVPGKSI		LRR3	2.4

VENIINFIEKSYKAIFVLSPHFIQSEWCHYELYFAHHNLFHEG

739	SDNLILILL EPILQNNIPSRYHK		LRR1	2
762	LRALMAQRT YLEWPTEKGKRG			LRR2	2.8
783	LFWANLRAS FIMKLALVNEDDVKT		LRR3	2.8


>ENSMUSP00000032105

MARPGPGVLGAPRLAPRLLLWLLLLLLQWPESAGAQAGPRAP

43	CAAACTCAGDSLDCSGRGLATLPRD		NF	4
68	LPSWTRSLN LSYNRLSEIDSAAFEDLTN		LRR1	2.4
96	LQEVYLNSN ELTAIPSLGAASIG		LRR2	3.4
119	VVSLFLQHN KILSVDGSQLKSYLS		LRR3	3.4
143	LEVLDLSSN NITEIRSSCFPNGLR		LRR4	4
167	IRELNLASN RISILESGAFDGLSRS		LRR5	3.4
192	LLTLRLSKN RITQLPVKAFKLPR		LRR6	4
215	LTQLDLNRN RIRLIEGLTFQGLDS		LRR7	4
239	LEVLRLQRN NISRLTDGAFWGLSK		LRR8	4
263	MHVLHLEYN SLVEVNSGSLYGLTA		LRR9	3.4
287	LHQLHLSNN SISRIQRDGWSFCQK		LRR10	4
311	LHELILSFN NLTRLDEESLAELSS		LRR11	4
335	LSILRLSHN AISHIAEGAFKGLKS		LRR12	4
359	LRVLDLDHN EISGTIEDTSGAFTGLDN		LRR13	4
386	LSKLTLFGN KIKSVAKRAFSGLES		LRR14	4
410	LEHLNLGEN AIRSVQFDAFAKMKN		LRR15	4
434	LKELYISSE				LRR16	2.4
443	SFLCDCQLKWLPPWLMGRMLQAFVTATCAHPESLKGQSIFSVLPDSFVCD		CF1_short	3

SHIFT -59!

434	LKELYISSE SFLCDCQLKWLPPWLM		LRR16	2.4
459	GRMLQAFVT ATCAHPESLKGQSIFSVLPDS		LRR17	2.2

FVCDDFPKPQIITQPETTMAVVGKDIRFTCSAASSSSSPMTFAWKKDNEVLANADMENFA
HVRAQDGEVMEY

561	TTILHLRHV TFGHEGRYQCIITNHFGSTY		LRR1	2
590	SHKARLTVN VLPSFTKIPHDIAIRTGTTAR		LRR2	2.4

LECAATGHPNPQIAWQKDGGTDFPAARERRMHVMPDDDVFFITDVKIDDMGVYSCTAQNS
AGSVSANATLTVLETPSLAVPLEDRVVTVGETVAFQCKATGSPTPRITWLKGGRPLSLTE
RHHFTPGNQLLVVQNVMIDDAGRYTCEMSNPLGTERAHSQ

780	LSILPTPGC RKDGTTVGIFTIAVVCSIV		LRR1	2.4
808	LTSLVWVCI IYQTRKKSEEYSVTNTDETIV		LRR2	2.4

PPDVPSYLSSQGTLSDRQETVVRTEGGHQANGHIESNGVCLRDPSLFPEVDIHSTTCRQP
KLCVGYTREPWKVTEKADRTAAPHTTAHSGSAVCSDCSTDTAYHPQPVPRDSGQPGTASS
QELRQHDREYSPHHPYSGTADGSHTLSGGSLYPSNH

994	DRILPSLKN KAASADGNGDSSWT		LRR1	2
1017	LAKLHEADC IDLKPSPTLASGSPELMEDAI		LRR2	2.4

SHIFT -24!

1023	ADCIDLKPS PTLASGSPELMEDAIST		LRR1	2.2
1049	EAQHLLVSN GHLPKACDSSPESVPLKGQIT		LRR2	2

GKRRGPLLLAPRS

>ENSMUSP00000057293

MFTLAEVASLNDIQPTYRILKPWWDVFMDY

31	LAVVMLMVA IFAGTMQLTKDQVVC		LRR1	2.4
55	LPVLPSPAN SKAHTPPGNADITTEVPRMET		LRR2	3

ATHQDQNGQTTTNDVAFGTSAVTPDIPLQATHPHAESTLPNQEAKKEKRDPTGRKTNLDF
QQYVFINQMCYHLALPWYSKYFPYLALIHTIILMVSSNFWFKYPKTCSKVEHFVSILGKC
FESPWTTKALSETACEDSEENKQRITGAQTLPKHVSTSSDEGSPSASTPMINKTGFKFSA
EKPVIEVPSMTILDKKDGEQAKALFEKVRKFRAHVEDSDLIYKLYVVQTLIKTAKFIFIL
CYTANFVNAISFEHVCKPKVEHLTGYEVFECTHNMAYM

363	LKKLLISYI SIICVYGFICLYTLFW		LRR1	2.4
388	LFRIPLKEY SFEKVREESSFSDIPDVKNDF		LRR2	2.4

AFLLHMVDQYDQLYSKRFGVFLSEVSENK

447	LREISLNHE WTFEKLRQHVSRNAQDKQE		LRR1	2.4
475	LHLFMLSGV PDAVFDLTDLDVLKLELIPEA		LRR2	2.4

SHIFT -15!

490	LTDLDVLKL ELIPEAKIPAKISQMTN		LRR1	2.4
516	LQELHLCHC PAKVEQTAFSFLRDH		LRR2	3.4
540	LRCLHVKFT DVAEIPAWVYLLKN		LRR3	2.8
563	LRELYLIGN LNSENNKMIGLESLRELRH		LRR4	4
591	LKILHVKSN LTKVPSNITDVAPH		LRR5	3.4
614	LTKLVIHND GTKLLVLNSLKKMMN		LRR6	2.4
638	VAELELQNC ELERIPHAIFSLSN		LRR7	2.8
661	LQELDLKSN NIRTIEEIISFQHLKR		LRR8	4
686	LTCLKLWHN KIVAIPPSITHVKN		LRR9	4
709	LESLYFSNN KLESLPTAVFSLQK		LRR10	3.4
732	LRCLDVSYN NISTIPIEIGLLQN		LRR11	3.4
755	LQHLHITGN KVDILPKQLFKCVK		LRR12	3.4
778	LRTLNLGQN CIASLPEKISQLTQ		LRR13	4
801	LTQLELKGN CLDRLPAQLGQCRM		LRR14	4
824	LKKSGLVVE DQLFDTLPLEVKEALNQDVNV		LRR15	2

PFANGI

>ENSMUSP00000020350

MDTSCVHMLLS

12	LLALLQLVA AGSSPGPDAIPRG		LRR1	2
34	CPSHCHCELDGRMLLRVDCSDLGLSELPSNLSVF		NF	4

SHIFT -26!

42	LDGRMLLRV DCSDLGLSELPSNLSVF		LRR2	2
68	TSYLDLSMN NISQLPASLLHRLCF		LRR3	3
92	LEELRLAGN ALTHIPKGAFTGLHS		LRR4	4
116	LKVLMLQNN QLRQVPEEALQNLRS		LRR5	4
140	LQSLRLDAN HISYVPPSCFSGLHS		LRR6	4
164	LRHLWLDDN ALTDVPVQAFRSLSA		LRR7	4
188	LQAMTLALN KIHHIADYAFGNLSS		LRR8	3.4
212	LVVLHLHNN RIHSLGKKCFDGLHS		LRR9	4
236	LETLDLNYN NLDEFPTAIKTLSN		LRR10	4
259	LKELGFHSN NIRSIPERAFVGNPS		LRR11	3.4
283	LITIHFYDN PIQFVGVSAFQHLPE		LRR12	2.8
307	LRTLTLNGA SHITEFPHLTGTAT		LRR13	3
330	LESLTLTGA KISSLPQAVCDQLPN		LRR14	3
354	LQVLDLSYN LLEDLPSLSGCQK			LRR15	4
376	LQKIDLRHN EIYEIKGSTFQQLFN		LRR16	3.4
400	LRSLNLAWN KIAIIHPNAFSTLPS		LRR17	4
424	LIKLDLSSN LLSSFPVTGLHG			LRR18	4
445	LTHLKLTGN RALQSLIPSANFPELKIIEMP		LRR19	4

SAYQCCAFGGCENVYKISNQWNKDDGNSVDDLHKKDAGLFQVQDERDLEDFLLDFEED

533	LKALHSVQC SPSPGPFKPCEHLFGSW		LRR1	2.4
559	LIRIGVWTT AVLALSCNALVALTVFRTPLY		LRR2	2.2

SHIFT -17!

572	LSCNALVAL TVFRTPLYISSIKLLIGVIAV		LRR1	2
602	VDILMGVSS AVLAAVDAFTFGRFAQHGAWW		LRR2	2.2

EDGIGCQIVGFLSIFASESS

652	IFLLTLAAL ERGFSVKCSSKFEVKAP		LRR1	2.4
678	LFSLRAIVL LCVLLALTIATIPLLGGSKYN		LRR2	2.4
708	ASPLCLPLP FGEPSTTGYMVALVL		LRR3	2.4
732	LNSLCFLIM TIAYTKLYCSLEKGE		LRR4	2.4
756	LENLWDCSM VKHIALLLFANCILYCPVAF		LRR5	2
785	LSFSSLLNL TFISPDVIKFIL			LRR6	2
806	LVIVPLPSC LNPLLYIVFNPHFKEDMGSLG		LRR7	2.8

SHIFT -21!

815	LNPLLYIVF NPHFKEDMGSLGKHTRFWMRS		LRR1	2
845	KHASLLSIN SDDVEKRSCESTQALVSFTHA		LRR2	2

SIAYDLPSTSGASPAYPMTESCHLSSVAFVPCL

>ENSMUSP00000082137

MAWLVLSGILLCMLGAGLGTSDLEDVLPPAPHN

34	CPDICICAADVLSCAGRGLQDLPVALPTT		NF	4

SHIFT -11!

52	LQDLPVALP TTAAELDLSHNALKR		LRR1	2.4
76	LHPGWLAPL SRLRALHLGYNK			LRR2	2.4
97	LEVLGHGAF TNASGLRTLDLSSNM		LRR3	2
121	LRMLHTHDL DGLEELEKLLLFNNS		LRR4	2
145	LMHLDLDAF QGLRMLSHLYLSCNELSSFSF		LRR5	3

SHIFT -19!

156	LRMLSHLYL SCNELSSFSFNHLHGLGLTR		LRR1	2
185	LRTLDLSSN WLKHISIPELAALPTYL		LRR2	4
211	KNRLYLHNN				LRR3	3
220	PLPCDCSLYHLLRRWHQRGLSALHDFEREYTCLVFKVSESRVRFFEHSRVFKNC		CF1_short	3

SHIFT -63!

211	KNRLYLHNN PLPCDCSLYHLLRRWHQRG		LRR3	3
239	LSALHDFER EYTCLVFKVSESRVRFFEHSR		LRR4	2

VFKNCSVAAAPGLELPEEQLHAQVGQSLRLFCNTSVPATRVAWVSPKNELLVAPASQDGS
IAV

332	LADGSLAIG RVQEQHAGVFVCL			LRR1	2.4
354	ASGPRLHHN QTLEYNVSVQKARPEPETFNT		LRR2	2.4

GFTTLLGCIVGLVLVLLYLFAPPCRGCCHCCQRACRNRCWPRASSPLQELSAQSSMLSTT
PPDAPSRKASVHKHVVF

461	LEPGKKGLN GRVQLAVAEDFDLCN		LRR1	2.4
485	PMGLQLKAG SESASSTGSEGLVMS		LRR2	2


>ENSMUSP00000020094

MGM

4	LARVALGLI IIDAVLAAPTTELFNYDSE		LRR1	2.4
32	VYDAILEDT GTFYNYEHIPDNHVENE		LRR2	2.2
58	KVSERLSGN RELLTPGPQLGDNQDEDKDEE		LRR3	2

STPRLIDGSSPQEPEFPGLLGPHTNEDFPT

118	CLLCTCISTTVYCDDHELDAIPPLPKKT		NF	4

TYFYSRFNRIKKINKNDFAS

166	LNDLKRIDL TSNLISEIDEDAFRKLPH		LRR1	2
193	LQELVLRDN KIKQLPELPNTLTFIDISNNR		LRR2	4

SHIFT -10!

213	LTFIDISNN RLGRKGIKQEAFKDMYD		LRR1	2.8
239	LHHLYITDN SLDHIPLPLPES			LRR2	3.4
260	LRALHLQNN DILEMHEDTFCNVKNLTYVRK		LRR3	4

A

291	LEDIRLDGN PINLSRTPQAYMC			LRR1	3.4
313	LPRLPIGSF I			LRR2	2.4


>ENSMUSP00000053840

M

2	LNKMTLHPQ QIMIGPRFNRALFDPLLVV		LRR1	2.4
30	LLALQLLVV AGLVRAQTCPSVCSCSNQFSK		LRR2	3

VICVRKNLREVPDGISTN

78	TRLLNLHEN QIQIIKVNSFKHLRH		LRR1	3
102	LEILQLSRN HIRTIEIGAFNGLAN		LRR2	4
126	LNTLELFDN RLTTIPNGAFVYLSK		LRR3	4
150	LKELWLRNN PIESIPSYAFNRIPS		LRR4	4
174	LRRLDLGEL KRLSYISEGAFEGLSN		LRR5	3
199	LRYLNLAMC NLREIPNLTPLIK			LRR6	3.4
221	LDELDLSGN HLSAIRPGSFQGLMH		LRR7	4
245	LQKLWMIQS QIQVIERNAFDNLQS		LRR8	2.8
269	LVEINLAHN NLTLLPHDLFTPLHH		LRR9	3.4
293	LERIHLHHN				LRR10	3.4
302	PWNCNCDILWLSWWIRDMAPSNTACCARCNTPPNLKGRYIGELDQNYFTCY		CF1_short	3

APVIVEPPADLNVTEGMAAELKCRASTSLTSVSWITPNGTVMTHGAYKVRIAVLSDGTLN
FTNVTVQDTGMYTCMVSNSVGNTTASATLNVTAATTTPFSYFSTVTVETMEPSQDEARTT
DNNVGPTPVIDWETTNVTTSLTPQSTRSTEKTFTIPVTDINSGIPGIDEVMKTTKIIIGC
FVAITLMAAVMLVIFYKMRKQHHRQNHHAPTRTVEIINVDDEITGDTPMESHLPMPAIEH
EHLNHYNSYKSPFNHTTTVNTINSIHSSVHEPLLIRMNSKDNVQETQI

>ENSMUSP00000061906

MALRAPTLLLLLLG

15	LLLLPLLPG LPPRATG		LRR1	3
31	CPAACRCYSATVECGALRLRVVPPGIPPG		NF	4

SHIFT -16!

44	CGALRLRVV PPGIPPGTQTLFLQDNSIAH		LRR2	2
73	LEQGSLAPL AALRHLYLHNNT			LRR3	2.4
94	LRALESGAF RAQPRLLELALTGNR		LRR4	2
118	LRGLRGGAF VGLVQLRVLYLAGNQLAKLLD		LRR5	2.4
148	FTFLHLPRL QELHLQENSIELLEDQALAG		LRR6	2.4
177	LSSLALLDL SRNQLGTISKEALQPLSS		LRR7	3
204	LQVLRLTEN				LRR8	4
213	PWRCDCALHWLGSWIKEGGRRLLSSRDKKITCAEPPRLALQSLLEVSGGSLICI		CF1_short	3

SHIFT -47!

220	LHWLGSWIK EGGRRLLSSRDKKITCAE		LRR1	2
247	PPRLALQSL LEVSGGSLICIPPSVNVEPPE		LRR2	2

FTANLGEDLQVACQASGYPQPLVVWRKVPQPRDGKPQAQAQLEGGAPGLGGHGTRDT

334	GSGMLFLTN ITLAHAGKYECEAANAGGKA		LRR1	2.2
363	RVPFHLLVN ASRQQSQQLPDPQAPATRPVG		LRR2	2.4

HEPQHEAGSMA

404	FRALGLATQ TAITAAIALLALT			LRR1	2.4
426	ALLLAAMIC RRRRRRKKVPAP			LRR2	2.2
447	SGEGTLFVN DYSDGPCTFAQLEELRDDHGH		LRR3	2.4

SHIFT -10!

467	LEELRDDHG HEMFVIDRSKPLFPEV		LRR1	2
492	LPEEAPEHN PPDGLKSGLRLPTRVAYEIHC		LRR2	2


>NM_001013384

MRPQELLLLLLMLKWSLAHTEDPAFPHLGDSSQPLPRP

39	CPWRCSCPRDDTVDCAGLDLRIFPDNITRA		NF	4

SHIFT -16!

53	CAGLDLRIF PDNITRAARHLS			LRR1	2
74	LQNNQLREL PYNELSRLSGLRTLDLHSNLI		LRR2	2

SHIFT -17!

87	LSRLSGLRT LDLHSNLITSEGLPDEA		LRR1	2.8
113	FESLNQLEN FYVAHNKLSVAPQFLPRS		LRR2	2.4
140	LRVADLAAN EVVEIFPLTFGEKPA		LRR3	3.4
164	LRSVYLHNN RLRNTGLPPNTFHGSEA		LRR4	3.4
190	ITTLSLSSN QLSYLPPSLPAS			LRR5	3.4
211	LERLHLQNN LISKVPRGALSLQTH		LRR6	4
235	LRELYLQHN QLTDSGLDATTFSKLSS		LRR7	4
261	LEYLDLSHN QLATVPEGLPGT			LRR8	4
282	LTILHLGRN CIRHVEAVRLHKARG		LRR9	4
306	LRYLLLQHN KLGASALPKGTLRPLRA		LRR10	4
332	LHTLHLYGN KLERVPPALPRH			LRR11	4
353	LQALVMPHN HVAALGARDLVSARA		LRR12	3.4
377	LAELNLAYN SLASARVHPSAFRRLRA		LRR13	4
403	LRSLDLAGN QLTRLPEGLPAS			LRR14	4
424	LRSLRLQRN QLRTLEPEQLAGLNK		LRR15	4
448	LRELNLTHN RLRVGDIGPGTWHELQA		LRR16	4
474	LKVLDLSHN ELSFVPPDLPEA			LRR17	4
495	LEELHLQAN RISHVGPEAFLSTPH		LRR18	4
519	LRALFLRAN RLHMTSIAAEALQG		LRR19	4
542	LTHLRVVDT AENPEQVLVQLPRKGKEP		LRR20	2.8


>ENSMUSP00000041417

MGSLQHCCCQLPKMGDTWAQLPWPGPP

28	HSALLLVFF LLAAGVMHSDAGTS		LRR1	2
51	CPVLCTCRNQVVDCSNQRLFSVPPDLPMD		NF	4

SHIFT -29!

51	CPVLCTCRN QVVDCSNQRLFSVPPDLPMD		LRR2	2
80	TRNLSLAHN RIAAVPPGYLTCYME		LRR3	3
104	LRVLDLRNN SLMELPPGLFLHAKR		LRR4	4
128	LAHLDLSYN NLSHVPADMFREAHG		LRR5	4
152	LVHIDLSHN PWLRRVHPQAFQGLVH		LRR6	3.4
177	LRDLDLSYG GLAFLSLEALEGLPG		LRR7	3
201	LVTLQIGGN				LRR8	3.4
210	PWVCGCTMEPLLKWLRNRIQRCTADSQLAECRGPPEVEGAPLFSLTEESFKAC		CF1_short	3

SHIFT -42!

221	LKWLRNRIQ RCTADSQLAECRGPPEVEGAP		LRR1	2
251	LFSLTEESF KACHLTLTLDDYLFIAFVGFV		LRR2	2
281	VSIASVATN FLLGITANCCHRWSKANEEEE		LRR3	2.2

I

>ENSMUSP00000040877

MNVC

5	AFSLALALV GSVSGQYYDYDIPL		LRR1	2.4
28	FMYGQISPN CAPECNCPHSYPTAMY		LRR2	2.2
53	CDDLKLKSV PMVPPGIKYLYLRNNQIDHID		LRR3	2

SHIFT -15!

68	IKYLYLRNN QIDHIDEKAFENVTD		LRR1	3.4
92	LQWLILDHN LLENSKIKGKVFSKLKQ		LRR2	4
118	LKKLHINYN NLTESVGPLPKS			LRR3	3.4
139	LQDLQLTNN KISKLGSFDGLVN			LRR4	4
161	LTFIYLQHN QLKEDAVSASLKGLKS		LRR5	3.4
186	LEYLDLSFN QMSKLPAGLPTS			LRR6	4
207	LLTLYLDNN KISNIPDEYFKRFTG		LRR7	4
231	LQYLRLSHN ELADSGVPGNSFNISS		LRR8	4
256	LLELDLSYN KLKSIPTVNENLENYY		LRR9	4
281	LEVNELEKF DVKSFCKILGPLSYSK		LRR10	2
306	IKHLRLDGN PLTQSSLPPDMYECLRVANEI		LRR11	3.4

TVN

>ENSMUSP00000035444

MHSPPGLLALWLCAVLCASARAGSDPQPGPGRPA

35	CPAPCHCQEDGIMLSADCSELGLSVVPADLDPL		NF	4

SHIFT -23!

45	GIMLSADCS ELGLSVVPADLDPL		LRR1	2.2
68	TAYLDLSMN NLTELQPGLFHHLRF		LRR2	3
92	LEELRLSGN HLSHIPGQAFSGLHS		LRR3	4
116	LKILMLQSN QLRGIPAEALWELPS		LRR4	4
140	LQSLRLDAN LISLVPERSFEGLSS		LRR5	4
164	LRHLWLDDN ALTEIPVRALNNLPA		LRR6	4
188	LQAMTLALN HIRHIPDYAFQNLTS		LRR7	3.4
212	LVVLHLHNN RIQHVGTHSFEGLHN		LRR8	4
236	LETLDLNYN ELQEFPLAIRTLGR		LRR9	4
259	LQELGFHNN NIKAIPEKAFMGSPL		LRR10	3.4
283	LQTIHFYDN PIQFVGRSAFQYLSK		LRR11	2.8
307	LHTLSLNGA TDIQEFPDLKGTTS		LRR12	3
330	LEILTLTRA GIRLLPPGVCQQLPR		LRR13	3
354	LRILELSHN QIEELPSLHRCQK			LRR14	4
376	LEEIGLRHN RIKEIGADTFSQLGS		LRR15	3.4
400	LQALDLSWN AIRAIHPEAFSTLRS		LRR16	4
424	LVKLDLTDN QLTTLPLAGLGG			LRR17	4
445	LMHLKLKGN LALSQAFSKDSFPK		LRR18	4
468	LRILEVPYA YQCCAYGICASFFKTSGQWQA		LRR19	2.4

EDFHPEEEEAPKRPLGLLAGQAENHYDLDLDELQMGTEDSKPHPSVQCSPVPGPFKPCEH
LFESWGIRLAVWA

571	IVLLSVLCN GLVLLTVFASGPSP		LRR1	2.8
594	LSPVKLVVG AMAGANALSGISCGLLASVDA		LRR2	2.4

LTYGQFAEYGARWESGLGCQATGFLAVLGSEASVLLLTLAAVQCSISVTCVRAYGKAPSP
GSVRAGA

691	LGCLALAGL AAALPLASVGEYG			LRR1	3
713	ASPLCLPYA PPEGRPAALGFAVALVMMNS		LRR2	2.4
742	LCFLVVAGA YIKLYCDLPRGDFEAVWDCAM		LRR3	2.4

VRHVAWLIFADGLLYCPVAFLSF

795	ASMLGLFPV TPEAVKSVLLVVLPLPACL		LRR1	2.4
823	NPLLYLLFN PHFRDDLRRLWPSPRSPGPLA		LRR2	3

SHIFT -28!

825	LLYLLFNPH FRDDLRRLWPSPRSPGPLAY		LRR1	2.4
854	AAAGELEKS SCDSTQALVAFS			LRR2	2.2
875	DVDLILEAS EAGQPPGLETYGFPSVTLISR		LRR3	2.4

HQ

907	PGATRLEGN HFVESDGTKFGNPQPPM		LRR1	2
933	KGELLLKAE GATLAGCGSSVGGAL		LRR2	2
957	WPSGSLFAS HL			LRR3	2.2


>XP_485967

MSHQI

6	LLLLAMLTL GLAISQRREQVPCRTVNKEAL		LRR1	2.4
36	CHGLGLLQV PSVLSLDIQALY			LRR2	2
57	LSGNQLQSI LVSPLGFYTALRHLDLSDNQI		LRR3	2

SHIFT -11!

76	LRHLDLSDN QISFLQAGVFQALPY		LRR1	4
100	LEHLNLAHN RLATGMALNSGGLGRLPL		LRR2	4
127	LVSLDLSGN SLHGNLVERLLGETPR		LRR3	4
152	LRTLSLAEN SLTRLARHTFWGMPA		LRR4	4
176	VEQLDLHSN VLMDIEDGAFEALPH		LRR5	3.4
200	LTHLNLSRN SLTCISDFSLQQ			LRR6	4
221	LQVLDLSCN SIEAFQTAPEPQAQFQ		LRR7	4
246	LAWLDLREN KLLHFPDLAVFPR			LRR8	4
268	LIYLNVSNN LIQLPAGLPRGSEDLHAPSEG		LRR9	3.4

WSASPLSNPSRNAS

312	THPLSQLLN LDLSYNEIELVPASFLEHLTS		LRR1	2
342	LRFLNLSRN CLRSFEARQVDSLPC		LRR2	4
366	LVLLDLSHN VLEALELGTKVLGS		LRR3	4
389	LQTLLLQDN ALQELPPYTFASLAS		LRR4	4
413	LQRLNLQGN QVSPCGGPAEPGPPGCVDFSG		LRR5	4

IPT

446	LHVLNMAGN SMGMLRAGSFLHTP		LRR1	3.4
469	LTELDLSTN PGLDVATGALVGLEAS		LRR2	4
494	LEVLELQGN GLTVLRVDLPCFLR		LRR3	4
517	LKRLNLAEN QLSHLPAWTRAVS			LRR4	4
539	LEVLDLRNN SFSLLPGNAMGGLETS		LRR5	4
564	LRRLYLQGN PLSCCGNGWLAAQLHQGRVDV		LRR6	4

DATQDLICRFGS

606	QEELSLSLV RPEDCEKGGLKNV			LRR1	2
628	NLILLLSFT LVSAIVLTTLATICF		LRR2	2.4
652	LRRQKLSQQ YKA			LRR3	2


>ENSMUSP00000053597

MSLCVAPRHPTGAAAALGLGSLLVLLGPGRA

32	CPAGCACTDPHTVDCRDRGLPSVPDPFP		NF	4
60	LDVRKLLVA GNRIQQIPEDFFIFHGD		LRR1	2
86	LVYLDFRNN SLRSLEEGTFSGSGK		LRR2	3.4
110	LAFLDLSYN NLTQLGAGAFRSAGR		LRR3	4
134	LVKLSLANN HLAGVHEAAFESLES		LRR4	4
158	LQVLELNDN NLRSLNVAALDALPA		LRR5	4
182	LRTVRLDGN				LRR6	3.4
191	PWLCDCDFAHLFSWIQENTSKLPKGLDAIQCSLPMEDRRVALRELSEASFSEC		CF1_short	3

KFSLSLTDLFIIIFSGVAVSIAAIISSFFLATVVQCFQRCAPNKDTEDEDDDEDD

>ENSMUSP00000038048

MVSWQHQGS

10	LQDLKTCLA RTLFLLALLGRVSSL		LRR1	2
34	KECLILQSA EGSTVSCHGPTEFPSSLPADT		LRR2	2

VHLSVEFSN

73	LTQLPAAAL QGCPGLRELHLSSNR		LRR1	2.4
97	LQALSPELL APVPRLRALDLTRNALRS		LRR2	2
124	LPPGLFSTS ANLSTLVLRENQLREVSAQW		LRR3	2.2
153	LQGLDALGH LDLAENQLSSLPSGLLASLGA		LRR4	2.4
183	LHTLDLGYN LLESLPEGLLRGPRR		LRR5	4
207	LQRLHLEGN RLQRLEDSLLAPQPF		LRR6	4
231	LRVLFLNDN QLVGVATGSFQGLQH		LRR7	4
255	LDMLDLSNN SLSSTPPGLWAFLGRPTRDMQ		LRR8	4

SHIFT -13!

272	LWAFLGRPT RDMQDGFDISHNPWICDKN		LRR1	2.2
300	LADLCRWLV ANRNKMFSQNDTRCAGPEAMK		LRR2	2

SHIFT -26!

304	CRWLVANRN KMFSQNDTRCAGPEAMKGQRL		LRR1	2.4
334	LDVAELGSL			LRR2	2.4


>ENSMUSP00000007620

MIPVTELRYFADTQPAYRILKPWWDVFTDYISIVMLMIAVFGGTLQVTQDKMICLPCKWV
TKDSCNDSFRGWAASSPEPTYPNSTVLPTPDTGPTGIKYDLDRHQYNYVDAVCYENRLHW
FAKYFPYLVLLHTLIFLACSNFWFKFPRTSSKLEHFVSILLKCFDSPWTTRALSETVVEE
SDPKPAFSKMNGSMDKKSSTVSEDVEATVPMLQRTKSRIEQGIVDRSETGVLDKKEGEQA
KALFEKVKKFRTHVEEGDIVYRLYMRQTIIK

272	VIKFALIIC YTVYYVHNIKFDVDCTVD		LRR1	2.2
299	IESLTGYRT YRCAHPLATLFKILASFYIS		LRR2	2.2
328	LVIFYGLIC MYTLWWMLRRSLKKYSFESIR		LRR3	2.2

EESSYSDIPDVKNDF

373	AFMLHLIDQ YDPLYSKRFAVFLSEVSENK		LRR1	2.4
402	LRQLNLNNE WTLDKLRQRLTKNAQDKLE		LRR2	3
430	LHLFMLSGI PDTVFDLVELEVLKLELIPDV		LRR3	2.4

SHIFT -15!

445	LVELEVLKL ELIPDVTIPPSIAQLTG		LRR1	2.4
471	LKELWLYHT AAKIEAPALAFLREN		LRR2	3.4
495	LRALHIKFT DIKEIPLWIYSLKT		LRR3	2.8
518	LEELHLTGN LSAENNRYIVIDGLRELKR		LRR4	4
546	LKVLRLKSN LSKLPQVVTDVG			LRR5	4
567	VHLQKLSIN NEGTKLIVLNSLKKMVN		LRR6	2.4
593	LTELELIRC DLERIPHSIFSLHN		LRR7	3.4
616	LQEIDLKDN NLKTIEEIISFQHLHR		LRR8	3.4
641	LTCLKLWYN HIAYIPIQIGNLTN		LRR9	4
664	LERLYLNRN KIEKIPTQLFYCRK		LRR10	4
687	LRYLDLSHN NLTFLPADIGLLQN		LRR11	4
710	LQNLAVTAN RIEALPPELFQCRK		LRR12	3.4
733	LRALHLGNN VLQSLPSRVGELTN		LRR13	4
756	LTQIELRGN RLECLPVELGECPL		LRR14	3.4
779	LKRSGLVVE EDLFSTLPPEVKERLWRADKE		LRR15	2

QA

>ENSMUSP00000058050

MDAATAPKQAWLPWSPLLFLLLLPGGSISS

31	CPTVCDCTSQTRAVFCAHRRLDTIPGGLPLD		NF	4

SHIFT -7!

55	PGGLPLDTE LLDLSGNRLWGLQRGM		LRR1	2
80	LSRLGQLQE LDLSYNQLSTLEPGAFHGLQS		LRR2	2
110	LLTLRLQGN RLRIVGPGIFSGLTA		LRR3	4
134	LTLLDLRLN QIVLFLDGAFSELGS		LRR4	4
158	LQQLEVGDN HLVFVAPGAFAGLAK		LRR5	3.4
182	LSTITLERC NLSTVPGLALAQLPA		LRR6	2.8
206	LVALRLREL DIERLPAGALRGLGQ		LRR7	3
230	LKELEIHHW PSLEALDPGSLVGLN		LRR8	2.4
254	LSSLAITRC NLSSVPFQALHHLSF		LRR9	2.8
278	LRILDLSQN PISAIPARRLSPLVR		LRR10	4
302	LQELRLSGA CLTSIAAHAFHGLTA		LRR11	3
326	FHLLDVADN ALQTLEETAFPSPDK		LRR12	2.8
350	LVTLRLSGN				LRR13	4
359	PLTCDCRLLWLLRLRRRLDFGTSPPACAGPQHVQGKSLREFSDILPPGH		CF1_short	3

FTCKPALIRKSGPRWVIAEEGGHAVFSCSGDGDPAPTVSWMRPQGAWLGRVGRVRVLEDG
TLEIRSVQLRDRGAYVCVVSNVAGNDSLRTWLEVIQVEPPNGTLSDPNITMPGIPGPFFL
DSRGVAMVLAVGFLPFLTSVTLCFGLIALWSKGKGRVKHHMTFDFVAPRPSGDKNSGGNR
VTAKLF

>ENSMUSP00000059463

MNLVDLWLSRS

12	LSMCLLLQS FVLMILCFHSASM		LRR1	2.4
34	CPKGCLCSSSGGLNVTCSNANLKEIPRDLPPE		NF	4

SHIFT -27!

39	LCSSSGGLN VTCSNANLKEIPRDLPPE		LRR2	2.4
66	TVLLYLDSN QITSIPNEIFKDLHQ		LRR3	3
90	LRVLNLSKN GIEFIDEHAFKGVAET		LRR4	4
115	LQTLDLSDN RIQSVHKNAFNN			LRR5	4
136	LKARARIAN NPWHCDCTLQQVLRSMASNHE		LRR6	2

TAHNVICKTSVLDEHAGRPFLNAANDADLCNLPKKTTDYAM

207	LVTMFGWFT MVISYVVYYVRQNQEDARRH		LRR1	2.2
236	LEYLKSLPS RQKKADEPDDISTVV		LRR2	2.4


>XP_991236

MTPLGFCFCAPSLLLTGQVLLVVIQADPIAEWVQNPVRLTSEPWVRTKPWSWHPSDQAPK
SPKAHPSEAGEGALDYLGSSAPSQMFSPPRELKDSFLSFQAMDTSVASHPESDQFTVPHQ
HLAKQMTPPRKPLESVPKLNGDQNQSPQFKSISSLDQAADDQLFEIPDPPLDSENSSITK
FIASPQSLQKELVQHRPLAKEVVGTTKQFAKSQLQKKTVVDEYRDPNMNEAYSKSLPLQS
QANREGPSEKIEHPEYPLEAQTQESENLDMVHESPEYLPVLPEEDEPSVPKENPVQHHFA
SGKAAALKHPDRNAPSPKRNEARHSKLPNVTVKPEDLEVTVMSEADKETQQTLSQQQAPG
HLPESPKEVEPSSTPQDTLEQFSGGPEEIETLLTHQEAAAPNPELPEELGPSLVQQEVLS
ESLELPKDLEASGSQLEVPARRTKPPEEVNPPTEQEARIPTPEPSMPNIIEIPAATVSHH
NQDQVHHYTLPTITVQPADVEVTITSKPFKEAEISPVQEETQTPGPPRKVGHYSLEQQPA
VSSESSGEVGSLERYLGFLTQPPEEEEEEEEEEDEESSLTQEDIPSQHPSPILEGEPSPI
ELQQPIRPSESPEEVGLGPKTQSEVLVKPAELPEEVKSPVEQEAPLQAPESHFETIVDTP
PIHEVQPAPNKAHHHHWPNVTVRPVDLELAITSEPTRETESSLAQQESSVHPSEYTEERD
SFRYKQEQLAQPSKLEILIHTSEHHHLTSSPSAHHRTHHSSSPTTMVRPPDVQLTIAQNP
TAEVEPPPALHEVVGKPIAISDKGVNTSTHHTHPAVTPEPSEEVELLSNQEEAPVQSAEP
VEYEKSSLSQQKSTDKNPELLEEGGLSSAQQET

874	LVNPPVFPN EMIVQPSAHYEATGPP		LRR1	2.4
899	LIQTQLHPP ASYNVTAKPPELTNEVETSSQ		LRR2	2

QGVEPSNDQQEEVTSQYPMPPETHQDSPVYQWVLTQPAKLPEEVSSGQQGNLSPPLKHPP
GVQFIPFQQELPDQHLEPPKEGFFLSPVADSVLFSPEDLEAMFRYETSVLHKPTVIHLNQ
AVSSMEVESLKVPTEQVEYSQVHFEYPSYTPAFPETQFSQEKSITQEADLHEDIYPFPTQ

1109	HRGLYLPPD SFMGAEPSVTQH			LRR1	2
1130	LSLSQLEDL AENVGPSLVLQLTPAQASEPP		LRR2	2

SHIFT -17!

1143	GPSLVLQLT PAQASEPPKEIVFSPTQQVV		LRR1	2.8
1172	PNQLPESLN NIVTQLLTQQIIGPTPGQIQE		LRR2	2

EYPTEHTVSFQALDLEFTITSQYTPEANHTTEPKRTPPPTYPQVTFSYPSEVTVQPLDLG
LTISPQPTPEELPQAIPEITTQITEPPREVVAPAPLYQKMTVPTPGQDQVEYPIPPAISF
QPLDLELTKNSEPTRETQQPTATMKTIVPSPEHLQDYISYVAEVTVQPLNLGLTISPQSA
PEELPQPMPEITTQITEPPREVVAPAPLYQKMTIPTPGQDQDAHSTTLQPVGLELTETSD
STREAEHYTTLVMTTVSSPKYSLPEQVLLQHLNPAEVTVQALDLGLTITPQPDTEKELSQ
TTQESTTQLIDPPKEAVAQAPVYQEVTLATPSQDAFENPTLPIITFRLLDPDPEPAREAD
NLATLKETTIPPPKYPPVTLPEQVHTQHPHPSEVTFQPLDMELSMSPQPTPEVELSQAEQ
ETKTQPSYSAKEVVTPKPGYPEVTVPTPDQDKTEDQTLTAALFQPLDLELTRSSEPTTPH
KSTVVSLPQYYQTTLSGEVYTQHPNLTEVTDQPLNLELTVIPSPNLNVETSPHIQEMLPE
PERPHEEVTAKAPMFYEITPIQDQTPHLVSPKVTDQHIKVEHDIPTISS

1791	VENGLLAAT YSSTALPLTHHEMVLLSEDQ		LRR1	2.2
1820	VQVLHGNPT QVTIHPSHPEFSLASPPATMV		LRR2	2.2

KRSAPVQSTESSKETAAQSPVHLKETVLMIAHGQSQHLNSETTQPEVPATTHPETTQSTA
SPTTHSETTHSTASPTTHPDTTQSTASPTTHPETTQSTASPTTHSETTQSTTSPTTHPET
TQSTASPTTHPDTTQSTASPTIHPDTTQYTTSPTTHPETTQYTDLTTMPPPSTHYEVVLS
QSSQVQTQQPKPSEVTMWPSPTSQQSSTTPQSVILASPFATMLETTAQQIPSGIALEPMD
LEPTITPYAGNFNTEKDLIFQMKPNVSTSTNI

2122	CDLCLCENHTLLCSHLSPKRRLHQVPVL		NF	4

SHIFT -18!

2132	LLCSHLSPK RRLHQVPVLRPGTHKGT		LRR1	2
2158	LTILNFHGN VISYIDKNVWKAYRW		LRR2	3.4
2182	AEKLILSEN RLTELHKESFEGLLT		LRR3	3.4
2206	LQVLDLSCN KIHYIERRTFESLPF		LRR4	4
2230	LKYINLGCN LLTELNFGTFQAWHGMQF		LRR5	3.4
2257	LQQLILNHN PLTVVEDPFLFKLPT		LRR6	4
2281	LKYLDLGAT QVQLTMVENILMMMLE		LRR7	3.4
2306	LEHRILPSR				LRR8	2
2315	MACCLCKFKADIEIICKTIKLRCHTGCLTNTTH		CF3	3

CLKASIGDPEGAFM

2362	KALLARKEN SRAELTIEPEKSYADQTN		LRR1	2
2389	LGSLGLMNE QLDFNDESDVISALNYILPYF		LRR2	3

SEGNLEDVVSTL

2431	LPFIKLLFS NIQNGDNSLGPFQND		LRR1	2.8
2455	TKSLTLKSV PKASKLAYKNKVNK		LRR2	2
2478	LYFLENLLD EEINEVKEKEKTAMLVHHSGR		LRR3	2

LDPKFKRQIFEKRWEPARTGKDSLAEIEKAERQLHSMSRVPKETGSIQKRHFKDVSGKSL
WSKQSVQTPVESISKDRQLGSPPSMELQQLGLEQKPRELVGYSFPSEPLLPKEHRGELSS
SPDLPLLDKAPTTNSLPDFIDRRKDLSYTIYVLESANANVKRAKGSNPSLQPEARHRNLR
KKKSHFQLIAKRPAASSAVRSLISSSARGVFSSLGDLRYPERPFSELYVAPEPSTKKPLE
ENRAATDNVEENNLKQIVTTPEETTSENKPPENPTADSNVSTTSNLISTVQQTSKPQSVF
TVGADTHADLTDVAYPSLMSPGEQFESH

2836	LNQQLLPLI PNKDVRRLISHV			LRR1	2
2857	IRTLKMDCS DTRVQMSCAKLISRTG		LRR2	2.2
2882	LLMKLLSEQ QDFKLSRADWDTDQWKTENYI		LRR3	2

NESTETQGEQRS

2924	LEPSQLTKA VPGYGYNNKVILAISVTVI		LRR1	2
2952	VTVLIIIFC LIEVRTSVHAWVQNVPCLCSR		LRR2	2.2
2982	FRTWKLETS VTYPLCLQVMSPCVHRYKKNY		LRR3	2.2

YKISETQDGFFWLRCPLWLRDMYRPLHDTRKKNMAQDLQDKESSGEEEIFNKDVPREFKR
RSSVKSPAEESVVEAPR

>ENSMUSP00000059050

MLAGGMRSMPSPLLACWQPILLLVLGSVLSGSATG

36	CPPRCECSAQDRAVLCHRKRFVAVPEGIPTE		NF	4
67	TRLLDLGKN RIKTLNQDEFASFPH		LRR1	3
91	LEELELNEN IVSAVEPGAFNNLFN		LRR2	4
115	LRTLGLRSN RLKLIPLGVFTGLSN		LRR3	4
139	LTKLDISEN KIVILLDYMFQDLYN		LRR4	3.4
163	LKSLEVGDN DLVYISHRAFSGLNS		LRR5	3.4
187	LEQLTLEKC NLTSIPTEALSHLHG		LRR6	3.4
211	LIVLRLRHL NINAIRDYSFKR			LRR7	3
232	LYRLKVLEI SHWPYLDTMTPNC			LRR8	2.4
254	LYGLNLTSL SITHCNLTAVPYLAVRHLVY		LRR9	3
283	LRFLNLSYN PIGTIEGSMLHELLR		LRR10	4
307	LQEIQLVGG QLAVVEPYAFRGLNY		LRR11	2.4
331	LRVLNVSGN QLTTLEESAFHSVGN		LRR12	3.4
355	LETLILDSN				LRR13	4
364	PLACDCRLLWVFRRRWRLNFNRQQPTCATPEFVQGKEFKDFPDVLLPNY		CF1_short	3

SHIFT -58!

355	LETLILDSN PLACDCRLLWVF			LRR13	4
376	RRRWRLNFN RQQPTCATPEFVQGKEFK		LRR14	2.4
403	DFPDVLLPN YFTCRRAHIRDRKAQQVFVDE		LRR15	2

GHTVQFVCRADGDPP

448	PAILWLSPR KHLVSAKSNGRLTVFPDGTLE		LRR1	2
478	VRYAQVQDN GTYLCIAANAGGNDSMPAHLH		LRR2	2.2

VRSYSPDWPHQPNKTFAFISNQPGEGEANSTRATVPFPFD

548	IKTLIIATT MGFISFLGVVLFC			LRR1	2.2
570	LVLLFLWSR GKGNTKHNIEIEYVPRKSDAG		LRR2	3

ISSADAPRKFNMKMI

>ENSMUSP00000053573

MDLRDWLFLCYGLIAFLTEVIDSTT

26	CPSVCRCDNGFIYCNDRGLTSIPSDIPDD		NF	4
55	ATTLYLQNN QINNAGIPQDLKTKVK		LRR1	3.4
80	VQVIYLYEN DLDEFPINLPRS			LRR2	2.8
101	LRELHLQDN NVRTIARDSLARIPL		LRR3	4
125	LEKLHLDDN SVSTVSIEEDAFADSKQ		LRR4	4
151	LKLLFLSRN HLSSIPSGLPHT			LRR5	4
172	LEELRLDDN RISTIPLHAFKGLNS		LRR6	4
196	LRRLVLDGN LLANQRIADDTFSRLQN		LRR7	4
222	LTELSLVRN SLAAPPLNLPSAH			LRR8	4
244	LQKLYLQDN AISHIPYNTLAKMRE		LRR9	4
268	LERLDLSNN NLTTLPRGLFDDLGN		LRR10	4
292	LAQLLLRNN				LRR11	4
301	PWFCGCNLMWLRDWVRARAAVVNVRGLMCQGPEKVRGMAIKDITSEMDECF		CF1_short	3

EAGSQGGAANAAAKTTVSNHASATTPQGSLFTLKAKRPGLRLPDSNIDYPMATGDGAKTL
VIQVKPLTADSIRITWKAMLPASSFR

438	LSWLRLGHS PAVGSITETLVQGDKTEYL		LRR1	3.4
466	LTALEPKST YIICMVTMETGNTYVADETPV		LRR2	2.4

CAKAETADSYGP

508	TTTLNQEQN AGPMAGLPLAGIIGGAVA		LRR1	2
535	LVFLFLVLG AICWYVHRAGELLTRERVYNR		LRR2	3

GSRRKDDYMESGTKKDNSILEIRGPGLQMLPINPYRSKEEYVVHTIFPSNGSSLCKGAHT
IGYGTTRGYREAGIPDVDYSYT

>ENSMUSP00000036762

MENMPPQSWILTCFCLLSSGTSAIFHKANYSRSYPCDEIRHNSLVIAECNHRQLHEVPQT
IGKY

65	VTNIDLSDN AITHITKESFQKLQN		LRR1	2.8
89	LTKIDLNHN AKQQHPNENKNGMNITE		LRR2	3.4
115	GALLSLRNL TVLLLEDNQLYTIPAGLPES		LRR3	2.4
144	LKELSLIQN NIFQVTKNNTFGLRN		LRR4	4
168	LERLYLGWN CYFKCNQTFKVEDGAFKNLIH		LRR5	4
198	LKVLSLSFN NLFYVPPKLPSS			LRR6	4
219	LRKLFLSNA KIMNITQEDFKGLEN		LRR7	3
243	LTLLDLSGN CPRCYNAPFPCTPCKENSSIH		LRR8	4

IHP

276	LAFQSLTQL LYLNLSSTSLRTIPSTWFEN		LRR1	2
305	LSNLKELHL EFNYLVQEIASGAFLTKLPS		LRR2	2
334	LQILDLSFN FQYKEYLQFINISSNFSKLRS		LRR3	4
364	LKKLHLRGY VFRELKKKHFEHLQSLPN		LRR4	3
391	LATINLGIN FIEKIDFKAFQNFSK		LRR5	3.4
415	LDVIYLSGN RIASVLDGTDYSSWRNRLRKP		LRR6	3.4

LSTDDDEFDPHVNFYHSTKPLIKPQCTAY

474	GKALDLSLN NIFIIGKSQFEGFQD		LRR1	3.4
498	IACLNLSFN ANTQVFNGTEFSSMPH		LRR2	3.4
523	IKYLDLTNN RLDFDDNNAFSDLHD		LRR3	3.4
547	LEVLDLSHN AHYFSIAGVTHRLGFIQNLIN		LRR4	4
577	LRVLNLSHN GIYTLTEESELKSIS		LRR5	4
601	LKELVFSGN RLDRLWNANDGKYWSIFKS		LRR6	3.4
629	LQNLIRLDL SYNNLQQIPNGAFLNLPQS		LRR7	2
657	LQELLISGN KLRFFNWTLLQYFPH		LRR8	3.4
681	LHLLDLSRN ELYFLPNCLSKFAHS		LRR9	4
705	LETLLLSHN HFSHLPSGFLSEARN		LRR10	4
729	LVHLDLSFN TIKMINKSSLQTKMKTN		LRR11	4
755	LSILELHGN				LRR12	4
764	YFDCTCDISDFRSWLDENLNITIPKLVNVICSNPGDQKSKSIMSLDLTTCVSD		CF1	4

SHIFT -42!

775	RSWLDENLN ITIPKLVNVICSNPGDQKSKS		LRR1	2
805	IMSLDLTTC VSDTTAAVLFFLTFLTTSMVM		LRR2	2.8
835	LAALVHHLF YWDVWFIYHMCSAKLKGYRTS		LRR3	2

STSQTFYDAYISYDTKDASVTDWVINELRYHLEESEDKSVLLCLEERDWDPGLPI

920	IDNLMQSIN QSKKTIFVLTKKYAKSWNFKT		LRR1	2.4
950	AFYLALQRL MDENMDVIIFILLEPVLQY		LRR2	2.4
978	SQYLRLRQR ICKSSILQWPNNPKAEN		LRR3	2
1004	LFWQSLKNV VLTENDSRYDDLYIDSIRQY		LRR4	2


>ENSMUSP00000024916

MGRRVPALRQLLVLAMLVLKQSQ

24	LHSPELSGS RCPEPCDCAPDGALRCPGPR		LRR1	2.4
53	AGLARLSLT YLPVKVIPSQAFRGLNEVVKI		LRR2	2.2

EISQSDSLERIEANAF

99	DNLLNLSEI LIQNTKNLLYIEPGAFTNLPR		LRR1	2
129	LKYLSICNT GIRTLPDVSKISSSEF		LRR2	2.8
154	NFILEICDN LYITTIPGNAFQGMNNE		LRR3	2.4
180	SITLKLYGN GFEEVQSHAFNGTT		LRR4	3
203	LISLELKEN IYLEKMHSGTFQGATGPSILD		LRR5	4

VSSTK

238	LQALPSHGL ESIQTLIATSSYS			LRR1	2
260	LKTLPSREK FTSLLVATLTYPSHCCAFRNL		LRR2	2

PKKEQNFSFSIFENFSKQCESTVREANNETLYSAIFEENELSGWDYDYDFCSPKTLQCTP
EPDAFNPCEDIMGYAF

366	LRVLIWLIN ILAIFGNLTVLFVLLTSRYKL		LRR1	3.4
396	TVPRFLMCN LSFADFCMGLYLLLIASVDSQ		LRR2	2

TKGQYYNHAIDWQTGSGCSAAGFFTVFASE

456	LSVYTLTVI TLERWHTITYAVQL		LRR1	2
479	DQKLRLRHA IPIMLGGWIFSTL			LRR2	2
501	MATLPLVGV SSYMKVSICLPMDVESTLSQV		LRR3	2.4
531	YILSILLLN AVAFVVICACYVRIYFAVQNP		LRR4	2

ELTAPNKDTKIAKKMAILIFTDFTCMAPISFFAISAAFKVPLITVTNS

609	KVLLVLFYP VNSCANPFLYAVFTKAFQR		LRR1	2
637	DFFLLLSRF GCCKHRAELYRRKEFSACTFN		LRR2	2

SKNGFPRSSKPSQAALKLSIVHCQQPTPPRVLIQ

>ENSMUSP00000069772

MLHTAIPCWQPFLGLAVVLLLMGSTIG

28	CPARCECSAQNKSVSCHRRRLLAIPEGIPIE		NF	4
59	TKILDLSKN RLKSINPEEFISYPL		LRR1	3
83	LEEIDLSDN IIANVEPGAFNNLFN		LRR2	3.4
107	LRSLRLKGN RLKLVPLGVFTGLSN		LRR3	4
131	LTKLDISEN KIVILLDYMFQDLHN		LRR4	3.4
155	LKSLEVGDN DLVYISHRAFSGLLS		LRR5	3.4
179	LEQLTLEKC NLTAVPTEALSHLRS		LRR6	3.4
203	LIALHLKHL NINNMPVYAFKRLFH		LRR7	3
227	LKNLEIDYW PLLDLMPANSLYGLN		LRR8	2.4
251	LTSLSITNT NLSTVPFLAFKHLVY		LRR9	2.8
275	LTHLNLSYN PISTIEAGMFSDLIR		LRR10	4
299	LQELHIVGA QLRTIEPHSFQGLRF		LRR11	2.4
323	LRVLNVSQN LLETLEENVFSSPRA		LRR12	3.4
347	LEVLSINNN				LRR13	3.4
356	PLACDCRLLWLLQRQPNLQFGGQQPMCAGPDTIRERSFKDFHSTALSFY		CF1_short	3

FTCKKPKIREKKLQHLLVDEGQTVQLECNADGDPQPVISWVTPRRRFITTKSNGRATVLG
DGTLEIRFAQDQDS

479	GMYVCIASN AAGNDTFTASLTVKGF		LRR1	2.2
504	TSDRFLYAN RTPMYMTDSNDTVSNGTNAN		LRR2	2
533	TFSLDLKTI LVSTAMGCFTFLGVV		LRR3	2
557	LFCFLLLFV WSRGKGKHKNSID			LRR4	2.4
579	LEYVPRKNN GAVVEGEVAGPRRFNMKMI		LRR5	2.4


>ENSMUSP00000061828

MLNEGLCCGAWAMKGTLLLVSSVGLLLPGVGS

33	CPMKCLCHPSSNSVDCSGQGLSKVPRDLPPW		NF	4
64	TVTLLLQDN RIHWLPALAFQSVSL		LRR1	3
88	LSTLNLSNN SLSNLAAEAFYGLPH		LRR2	4
112	LRVLNVTQN SLLSIESSFAHALPG		LRR3	3.4
136	LRELDLSSN SLRILPTSLGKPWEN		LRR4	4
160	LTVFAVQQN HLLHLDRELLEAMPK		LRR5	2.8
184	VRLVLLKDN				LRR6	2.8
193	PWICDCHLLGLKLWLERFTFQGGETDGAICRLPEPWQGKALLSIPHELYQPC		CF1_short	3

SLPSQDLAPSLVQQPGSAPQDAQKSHENSSGQQDPLECEAKPKPKPTNLRHAVATVVITG
VVCGIVCLMMLAAAIYGCTYAAITAQYQGRPLASARKSEKMGSKELMDSSSA

>ENSMUSP00000038569

MRIVAILLLFCLCRAAEPRKSSPGVLRSQGNPSRSHGRGGRRGSSPVKRYAPGLPCDVYT
Y

62	LHEKYLDCQ ERKLVYVLPDWPQD		LRR1	2
85	LLHMLLARN KIRVLKNNMFAKFKR		LRR2	3.4
109	LKSLDLQQN EISKIESEAFFGLNK		LRR3	4
133	LTTLLLQHN QIKVLTEEAFIYTPL		LRR4	4
157	LSYLRLYDN				LRR5	4
166	PWHCTCELETLISMLQIPRNRNLGNYAKCGSPPALRNKKLLQLKPQELCDE		CF1_short	3

SHIFT -44!

173	LETLISMLQ IPRNRNLGNYAKCGSPPA		LRR1	2
200	LRNKKLLQL KPQELCDEEEKEQLDPKPQVS		LRR2	2

SHIFT -22!

208	LKPQELCDE EEKEQLDPKPQVSGIPAVIRP		LRR1	2
238	EADSTLCHN YVFPIQTLDCKRKELKKVPSN		LRR2	2

IPPD

272	IVKLDLSSN KIRQLRPKEFEDVHE		LRR1	3.4
296	LKKLNLSSN GIEFIDPAAFLGLIH		LRR2	4
320	LEELDLSNN SLQNFDYGVLEDLYF		LRR3	4
344	LKLLWLRDN PWRCDYSIHYLYYWLKHHYNV		LRR4	4

HYNGLECKTPEEYKGWSVGKYVRSYYEECPKDKLPAYPETFDQDTEDDEWQKIHRDHPAK
KHRVRITIVG

>ENSMUSP00000056094

MKVTRFMFW

10	LFSMLLPSV KSQASETEVPCNFSRRNYT		LRR1	2.4
38	LIPEGISTN VTILDLSYNRITLNAADSRV		LRR2	2.4
67	LQMYSLLTE LYLMENNIIALYNSSFRNLLN		LRR3	2
97	LEILNICGN SISVIQQGSFVGLNE		LRR4	3.4
121	LKQLFLCQN KILQLNPDTFVPLNN		LRR5	4
145	LKVLNLQGN LIRLFDAPQLPH			LRR6	4
166	LEILTLDGN				LRR7	4
175	PWNCTCGLLELHNWLNTSNVTLENENMTMCSYPDELKHDSIKSAPFTTECHS		CF1_short	3

TFISTITEDFQSTRNSSFNSSSHNLTWTSEHEPLGKSWAFLVGVVATVLLTS

279	LLIFIAIKC PVWYNILLSYNHHRLEEHEAE		LRR1	2.2
309	TYENGLTRN PSSLSQITDTNSEDTTVIFEQ		LRR2	2

LHAFVVDDDGFIEDRYIDINEVHEEK

>ENSMUSP00000078757

MSPWLKWHGPAMARLWGLCLLVLGFWRASLA

32	CPTSCKCSSARIWCTEPSPGIVAFPRLEP		NF	4

NSVDPE

67	NITEILIAN QKRLEIINEDDVEAYVG		LRR1	2
93	LRNLTIVDS GLKFVAYKAFLKNSN		LRR2	2.8
117	LRHINFTRN KLTSLSRRHFRHLD		LRR3	2.8
140	LSDLILTGN				LRR4	4
149	PFTCSCDIMWLKTLQETKSSPDTQDLYCLNESSKNMPLANLQIPNCGLP		CF1	4

SHIFT -39!

159	LKTLQETKS SPDTQDLYCLNESSKNMP		LRR1	2.4
186	LANLQIPNC GLPSARLAAPNLTVEE		LRR2	2.8
211	GKSVTLSCS VGGDPLPTLYWDVGNLVSKHM		LRR3	2.2

NETSHTQGSLRITNISSDDSGKQISCVAENLVGEDQDSVNLTVHFAPTITFLESPTSDHH
WCIPFTVRGNPKPALQWFYNGAILNESKYICTKIHVTNHTEYHGCLQLDNPTHMNNGDYT
LMAKNEYGKDERQISAHFMGRPGVDYETNPNYPEVLYEDWTTPTDIGDTTNKSNEIPSTD
VADQSNREHLSVYAVVVIASVVGFCL

447	LVMLLLLKL ARHSKFGMKGPASVISNDDDS		LRR1	3
477	ASPLHHISN GSNTPSSSEGGPDAVIIG		LRR2	2.4
504	MTKIPVIEN PQYFGITNSQLKPDTFVQHIK		LRR3	2.2

RHNIVLKRELGEGAFGKVFLAECYNLCPEQDKILVAVKTLKDASDNARKDFHREAELLTN
LQHEHIVKFYGVCVEGDPLIMVFEYMKHGDLNKFLRAHGPDAVLMAEGNPPTELTQSQML
HIAQQIAAGMVYLASQHFVHRDLATRNCLVGENLLVKIGDFGMSRDVYSTDYYRVGGHTM
LPIRWMPPESIMYRKFTTESDVWSLGVVLWEIFTYGK

751	QPWYQLSNN EVIECITQGRVLQRPRTCPQE		LRR1	2
781	VYELMLGCW QREPHTRKNIKSIHTL		LRR2	2.4
806	LQNLAKASP VYLDILG			LRR3	2


>C44H4.3

MLLRLCVALLVLPACLAFCPKLFQNQTACSCDSTVEGPVIKCSGHDGLRMVEKLSTTTTM
E

62	VRELALENA DIIEVGPKAFKTLR		LRR1	2.4
85	IKKLILSNN RIEKIHDHAFTGLENV		LRR2	3.4
110	MQELSLSEN NLKEVPTSALAGLRV		LRR3	3.4
134	LNILSLKCN KIENITTKAFVNMTS		LRR4	4
158	LIDVNLGCN QICSMAADTFANVKMS		LRR5	3.4
183	LQNLILDNN CMTEFPSKAVRNMNN		LRR6	4
207	LIALHIKYN KINAIRQNDFVNLTS		LRR7	3.4
231	LSMLSLNGN NISEIKGGALQNTPN		LRR8	4
255	LHYLYLNEN NLQTLDNGVLEQFKQ		LRR9	4
279	LQVLDLSFN NFTDITKEMFEGLES		LRR10	4
303	IQHLNLDSN RISAVAPGAFAGTP		LRR11	3.4
326	LLLLWLPNN CLTEVSQQTLKGAPF		LRR12	4
350	LRMVSLSNN NIREVHELSFDHLPN		LRR13	3.4
374	LHTLDLANN KIMSLQNKSLSGPE		LRR14	4
397	NLAVRLQEN PVVCVKNGFHVLNSGE		LRR15	2.4
422	AIWLTNEAN TICKGEWQAQTADLCPKAQPR		LRR16	2.4

PIRPVCCSNEITTTTTTTTTTTTLAPTTTTEEEKEETTETTEKVTTVGKSKKTKATTTTE
EPEDDEDVYVDDDEEGTEEEEEEVSTTEKSTTTTTTTTEASKSKKVNMERFWRLSNKPSG
KSPFLRHSQGNKPRYLQTTTEAPEEEDEAEYISDDGGAEESEEAETTVATTTTTEPSVKV
PARIRERQRMFASNIPWMAPRTKTDSVEEEFSKDDEVETVETAEKPARH

>ZC262.3a

MRKFVFFVVAILIQIHTTTSQRNRSSSPSGFLDLQLEK

39	CPQVLGCRCVRDSTRNIQCFSIDESKLLEIQKIY		NF	4

GSN

76	IQRLELHNW QHDQLNFDIFAPFPQ		LRR1	2.4
100	LEHIILRDG DLESLNGTVIHPT			LRR2	2.4
122	LKVLSIENS ELTSSSEVCRLLSIFPK		LRR3	2.8
148	IQSLSLSKN YFEKFECDTSNTK			LRR4	3.4
170	LKILDLSQN RISHLEVPNTLRVLNVS		LRR5	4
196	RNRLTSFEN ISTKLTDLDISFNKLSLWPSF		LRR6	2

SHIFT -17!

209	LTDLDISFN KLSLWPSFDDWKFPN		LRR1	3.4
233	LRSLSAIKL DLQTGFQLDAPL			LRR2	2.4
254	LNSLNIDGA SLRYLNFHQILTPKLKKFS		LRR3	2.4
282	ARYLTELRN IAGRLPSTVTDVAFTDTM		LRR4	2.4
309	LRTLPADFI PMSSTNHMQKVSFDFSTNQL		LRR5	2.4
338	LCDKCLLQW SLPVYAQTSIRKDCNLTREEI		LRR6	2

ESASCKIGVVANDTGIQYGKYEKPTAISCFSYGVPSPKISWWRFRPAEKLGSYDPITDEI
SYTNVSETMKESYEIQSGGSLLIRSPNRSHVERYVCVVENEYGKDYGIYHFR

480	LDYLDWYSY DVFNSVFWGGLATSLIVC		LRR1	2.4
507	LISFLLNIT WILTRKSALWWIQRAERLSRV		LRR2	2.8

RKMVEAMEKYRVRQMESLHEKYTKRVQIVRDNYHQQVEALRVSYASQQEKFQNYKAAQVD
AVHSHLDAMRDGYNNQLGRVREYGSKRAEQLWESYERQVNRMRTFSLQHRLKMMRQYKVK
QRYVNKLLESLQATSPEVQLENEEKVRAALEIPDDLATIDGSMDTPSRLSRSSSFHSLPE
YVIDEQGNVRPGIIPTNAPSIRFTTKPTTSSISNEASTSSPSSSGAHRSPDSPPEKR

>T23G11.6

MRP

4	LVAIFLVQL CFVAVGAAIPNCPD		LRR1	2.4
27	LDVLENQSD LSQDDLNNLILCFCKPDDKEK		LRR2	2

SHIFT -21!

36	LSQDDLNNL ILCFCKPDDKEKVVISC		LRR1	2
62	LYGSNLDHL MKATEAVKNASLITSSISIQH		LRR2	2

MEFPDGGLPEFGKLAPD

109	LKSLEIKEC SGQDELKVGDDSFKGLEQT		LRR1	2.8
137	LRNLTIHAC NLQTIPPSVDSLEN		LRR2	2.8
160	LETVVFSNN KLDSLGVDQFKNKKQ		LRR3	2.8
184	LSYLDVSGN FITSIEEKAFEPLTS		LRR4	3.4
208	LETLVIGEH NFINETVVEEIGRLKA		LRR5	2.4
233	LKTLDLSRA DGIFQPPETLFKEIPQ		LRR6	3
258	IEVLKLSGC SIPTLEPGQFATLKK		LRR7	2.8
282	LKELDLRVN LIENITAYAFDGLES		LRR8	4
306	LTRLSLAGN FISKLEPDVFFGLSS		LRR9	4
330	LEELDLGWN EIKTIPTDVFKPLTDK		LRR10	4
355	LKTISLRNN PISELPSTGLGM			LRR11	3.4
376	LEKLSLAEC GFTSISADQLKDYPK		LRR12	3.4
400	LEELDLSKC NISNIVENTFENQKDS		LRR13	3.4
425	LKKLNLQKN KLKSLPNLIKNLPA		LRR14	4
448	IESLDVSSN PYRCDGELVNFVFGVEDRVKK		LRR15	2.8

AEENGNSFFVANTNETVCDRPYTLRGEQILQVEVEKFQPYDEKSDTTTAPSTTTTSTVEE
TTTELKIPDLLVGSRTNDTLFKEEPRRDVYDLSKTDDAKGVYNQKGTYAVPITIGAIGLV
TVIVIVAVVLFIKKNKKVGTETAKGGKSDKVVKMEDGMVEIELDSQPGTQHATPRR

>F37E3.2

MWELS

6	LAIFGLFGL ARSCDPLHAQAHGIN		LRR1	2.4
30	LVCCSLNHS LTPSCEFSSLCQETCSCFLTI		LRR2	2.4

TRDLDCKQPNLPNPDAPYTIVIENSNESLTHSFLLGSCSKTVTSLTFQHYHKLYDSIDFG
ECFPRLAS

128	FQVVLLQRT KANFHGSLRNLKN			LRR1	2.2
150	LKVLSLVNV DFDFWLQPSPFINTITYIHIE		LRR2	3

NSSITELPKWLSTSKS

196	LSTVYLKGT SISSLTPVAQLPA			LRR1	2.8
218	VKSLKLSHN LIENLHRLLFVSPF		LRR2	3.4
241	LIHVDLSYN QITSFASHTFSKCID		LRR3	3.4
265	LRVLDLTGN PIKMLPYKPFAKNIK		LRR4	4
289	LKWLKLSRT NITTLTPDHFYGLNA		LRR5	3.4
313	LKTLSLSRM PIQSISPYSFVPLKS		LRR6	3
337	LRYLDMDSC NLTKIPQAVTANCH		LRR7	2.8
360	LARLNVANN MLHRSSSMPPEVMAMLSG		LRR8	3.4
387	LSQLRIEGN PLTEFPASFLLISRENFR		LRR9	3.4
414	LLRQLLHST MTLPVWLREPCTPYYW		LRR10	2.4
439	SMHLANRTN NLKSFVNQYSEMRMLKSGLGY		LRR11	2

CKEQYDWMLEQMEVYRELEKNSGCYSLRRLRSSLSSKPVPKISKNS

515	TNSLILSIP HDLSSPAFHIPI			LRR1	2
536	LLLISLCAN FLLLFSILMCSIMACRMEKGV		LRR2	3.4

DSV

>F10F2.4

MHILIILLFTSIISSIQADL

21	CAHCDCDFISHTVVCNRPSLLIRTVSML		NF	4
49	PTIRQLHLN SLNLPQPPHFLFHPN		LRR1	2
73	LRVLRMSRC GMHEIPGSTFLPLPG		LRR2	2.8
97	LEVIDLSNN HLETLPPTVLRSLKF		LRR3	3.4
121	LRVLILSNN RLSNLDQLTWILAPGVV		LRR4	4
147	LEQLDLSGN PIAIATSMTVFPP			LRR5	4
169	VRQLFLSDT RMESVNETAIMFKKLPGKCDR		LRR6	2.8

DVCRHIPIHNLNVSI

214	ITTIDFSSN RDLEIDSGALDVFSN		LRR1	2.2
238	ATYVDLSNT RLPVGFEEWLERKSR		LRR2	2.2
262	VKSLNISHC QLPLHEDTWTACGQF		LRR3	2.2
286	LHSLDISGI GAKRLRFSRFCP			LRR4	2.4
307	IRSVFARDN LISSVYIDAVSMESLHLERNM		LRR5	2.2

SHIFT -10!

327	MESLHLERN MFSEFPIPPPGVELTE		LRR1	3.4
352	LHTLGLSHN LMTSLPPHALQSYPN		LRR2	4
376	LQHFDVSSN QLSEIDPQAFPSIGLG		LRR3	2.8
401	LISLDLSSN QLSSLPHPILPS			LRR4	4
422	LLLLDLSFN TISHLDPHFFTGLPM		LRR5	4
446	LQQLRIASN PTLFSRCPNRDSPCWSDH		LRR6	3.4
473	LDELTSLVD LDISNSGLEFSLHWRHLRT		LRR7	2
501	LKSLILRGN EIRIIDSKSLPEN			LRR8	4
523	LRTLDLGEN RVQFTSNFSKLEH			LRR9	4
545	LRDLRVDQN				LRR10	3.4
554	PLRCDCSLYDIVPHLLNQSQISDPLLYYCFSGSWQYPLLPYLASVKPCVDT		CF1	4

TRNFYSILITTFIIAFAIVAGLIGGFLVYRKYADRANFVYKRISLMESPVRL

>T22E7.1a

MIEKVLVVVIIGVVGAYAQGSCRTDQHEMTCRGKNSLHDLKKDQDY

47	LEVDTLNVH QAKLDLTDDLVPDGINLSH		LRR1	2
75	LTLLNATDN QITRIGRRGFDKIRN		LRR2	3.4
99	MQFLYLSIN QISHSQPDPFQALEK		LRR3	3.4
123	LKRLEMNDA LDGNMEEKSDMLRNFFHSKNS		LRR4	2.4

SHIFT -26!

127	EMNDALDGN MEEKSDMLRNFFHSKNSFVH		LRR1	2
156	LAQIELNKN KIEGIYPKTFCGIQG		LRR2	3.4
180	LQRLELSNN RIPSFDFARTCLKE		LRR3	4
203	LKALMLAGN LIQKIPADIWDFLPS		LRR4	4
227	LSSLDISNN				LRR5	3.4
236	PIDCDCATIRLLSGDDVVFLNQADTKCASPPELEGKRIFELSRDYCKTT		CF1	4

RNPRGKASFFQFLVLFVIAVGILWLYKKYRERTRHMSSVPVGYTNLQHEQAVEPEFV

>ZK682.5

MGLRAIILATLVVTVYGQ

19	CPALSGACRCAPSVYEPVAIICQNAGSLQNAIQAIQA		NF	4

ARDIP

61	IDSLTILDT AIPTIPANAFQS			LRR1	2.2
82	FTILRLVLN RNTLQNIDDQAFNGPLLDS		LRR2	3.4
110	LIELDLNDN NLGQIPQTGIPRLRN		LRR3	4
134	LRKLYLNRN RINQLSSTAFNAFESRDL		LRR4	4
161	LLKLELAGN RLTDATLGDATVFRPLTL		LRR5	4
188	LQELSLETN SLTSIPSSALVNQRNT		LRR6	4
213	LTNLNLGLN SINEVPVGALDFPV		LRR7	4
236	LSSLSLEFN GITVIPPQAFQGVPN		LRR8	4
260	LQFLYMTGN KFPSWAPEMFRYITQ		LRR9	3.4
284	LKTLGIGET PISVIPNNAFMHIPN		LRR10	2.8
308	LIRLEMSEA AVDTIERGAFQRTPQ		LRR11	2.4
332	IQAIVLNKN RLSQVRADFFEGLND		LRR12	2.8
356	LYSIDLQGN RIDNVQPLGFANLPA		LRR13	3.4
380	ISHLDISYN LLQTMPSNVFQNSFLPQPNDR		LRR14	2.8

RVIYACGNPWYCNSELEWFRTLLRDNLDIDVEKPGCTAVCTSSPNGCPVEGTP

463	LRSVDFCQN NEEAQPLVGRALSMVG		LRR1	2.8
488	WIILAVIMT ILLISICLLAMVRYGMSHQRK		LRR2	2.2

KQKDAEVAAEEYHHQTTATSIYNAPISVVDRPYSTVPPVNLDLPAAYTLDDRPNNYLY

>F20D1.7

MVLQIFVLSIFFISLFNKAI

-4	CPLGCSCNESSISCTFLTKPELYVF		NF	4

SHIFT 0!

21	ACPLGCSCN ESSISCTFLTKPELYVF		LRR1	2.4
47	LEVLKDSPF NETTDLSLASIDNFW		LRR2	2
71	LRMLPDMPK LRVLKVQSSPSLD			LRR3	2
93	DSDWFLRRN QFPELQTLHFKNCSLKTFPKA		LRR4	2.4
123	LMALTSLLE LDLSDNMLENLGADSIHIRS		LRR5	2
152	IQRVILRNN QIKSIGVHVFRYMPT		LRR6	2.8
176	LKMLDLSGN NMTRLVTSDFTSAVS		LRR7	4
200	LRELILREN KIELIETDTTEPMQQ		LRR8	4
224	LETLDLSGN LLSEVRLEAQQNFRH		LRR9	4
248	LFSLNLSCN PLQIIREGFLQLPD		LRR10	4
271	LQVLQLDNC NISVVEAGAFVSLPR		LRR11	3.4
295	LHSMDIKDN PNLAYFSPYAFSNNTAFYRMN		LRR12	2.8

IQNSGFKRIPLTILEKISQLYIKGTQLDCSCTSRDMQDYGAITIVDWNDATCKTKIGAVQ
KLSHLEKTGEPCRDNL

401	LTPFGIRQT ATVGHSYRIYCANDGTN		LRR1	2.2
427	SKLLWISPN KTTIEASHPKLRKSSDKRTDY		LRR2	2.4

FTTTLLDPSFSRNHEERIHISNEYYGFDVVLENDAGHYECVSKSDMKTITRKIELEVVKP
NIYLNASHVATTSVHLNWNRNLKIEA

543	VDRVALRIT ASSEKMFKRQVQLS		LRR1	2.2
566	LYNMFRSYN LVNLSADKEYLI			LRR2	2.4
587	CLEWYLTDN DAVIYSSCISQKTKPFKTVMQ		LRR3	2.4

SLNAKLAIALIIILIIVVVFCCDTCIHQKVAYFSRIKKNAKMQQSVSGQSMLTQSSSADA
TTYENFQLSVTSASEHPLL

>F40E10.4

MLICFIFILLIPESAT

17	CPAECVCVDRTVSCVGQQLTEVPQNIPND		NF	4
46	TIRLDLQDN EITKIGPNDFSSLMN		LRR1	3
70	LKALQLMDN QIVTIHNQSFSSLVF		LRR2	4
94	LQKLRLSRN RIRHLPDNVFQNNLK		LRR3	4
118	LTHLDLSEN DITVVSDAQLQGPEF		LRR4	4
142	LEVLNLDKN HIFCLENNVISSWVS		LRR5	4
166	LEVLTLNGN RLTTFEEPSNAR			LRR6	4
187	FRQLDLFNN				LRR7	3.4
196	PWNCDCRLRWMRKWLEKAEGQNKTVCATPLNLQGSSIEILQDKFMTCS		CF1_short	3

GNRKRRYKKTCETAEI

260	CPLPCTCTGTTVDCRDSGLTYVPTNLPPS		NF	4
289	TTEIRLEQN QISSIPSHSFKNLKN		LRR1	2.4
313	LTRLDLSKN IITEIQPKAFLGLHN		LRR2	4
337	LHTLVLYGN NITDLKSDTFEGLGS		LRR3	4
361	LQLLLLNAN QLTCIRRGTFDHVPK		LRR4	4
385	LSMLSLYDN DIKSISEVTFQNLTS		LRR5	4
409	LSTLHLAKN				LRR6	4
418	PLICDCNLQWLAQINLQKNIETSGARCEQPKRLRKKKFATLPPNKFKCK		CF1_short	3

SHIFT -42!

425	LQWLAQINL QKNIETSGARCEQPKRLRK		LRR1	2
453	KKFATLPPN KFKCKGSESFVSMYADSCFID		LRR2	2.4

SI

485	CPTQCDCYGTTVDCNKRGLNTIPTSIPRF		NF	4
514	ATQLLLSGN NISTVDLNSNIHVLEN		LRR1	3.4
539	LEVLDLSNN HITFINDKSFEKLSK		LRR2	4
563	LRELRLNDN KLHHFSSMVLDEQSN		LRR3	4
587	LEILDLSGN NIQCFSSIFFNKATR		LRR4	4
611	IREIKVIGN				LRR5	2.2
620	DLLCDCRILPLMSWLRSNSSHSIDIPPCQQFQYSDNESDKQRCAAF		CF1	4

PEETCSDDSNL

677	CPPKCSCLDRVVRCSNKNLTSFPSRIPFD		NF	4

SHIFT -22!

684	LDRVVRCSN KNLTSFPSRIPFD			LRR1	2.4
706	TTELYLDAN YINEIPAHDLNRLYS		LRR2	3
730	LTKLDLSHN RLISLENNTFSNLTR		LRR3	4
754	LSTLIISYN KLRCLQPLAFNGLNA		LRR4	3.4
778	LRILSLHGN DISFLPQSAFSNLTS		LRR5	4
802	ITHIAVGSN				LRR6	2.2
811	SLYCDCNMAWFSKWIKSKFIEAGIARCEYPNTVSNQLLLTAQPYQFTCD		CF1_short	3

SHIFT -16!

844	SNQLLLTAQ PYQFTCDSKVPTKL		LRR1	2
867	ATKCDLCLN SPCKNNAICETTSSRKYTCNC		LRR2	2.4

TPGFYGVHCENQIDACYGSPCLNNATCKVAQAGRFNCYCNKGFEGDYCEKNIDDCVNSKC
ENG

960	GKCVDLINS YRCDCPMEYEGKHCEDK		LRR1	2.2
986	LEYCTKKLN PCENNGKCIPINGSYSCMCSP		LRR2	2

GFTGNNCETNIDDCKNVECQNGGSCVDGILSYDCLCRPGYAGQYCEIPPMMDMEYQKTDA
CQQSACGQGECVASQNSSDFTCKCHEGFSGPSCDRQMSVGFKNPGAYLALDPLASDGTIT
MTLRTTSKIGILLYYGDDHFVSAELYDGRVKLVYYIGNFPASHMYSSVKVNDGLPHRISI
RTSER

1201	KCFLQIDKN PVQIVENSGKSDQLITKGKEM		LRR1	2.4
1231	LYIGGLPIE KSQDAKRRFHVKNSESLKGCI		LRR2	2.4

SSITINEVPINLQQALENVNTEQSCSATVNFCAGIDCGNGKCTNNALSPKGYMCQCDSHF
SGEHCDEKRIKCDKQKFRRHHIENECRSVDRIKIAECNGYCGGEQNCCTAVKKKQRKVKM
ICKNGTTKISTVHIIRQCQCEPTKSVLSEK

>K03A1.2.1

MR

3	LKFALLFLI LVVYSFAYNPVKSN		LRR1	2.4
26	DKDKALADN SKKVKVKKAELTAAQDEDDEG		LRR2	2

SKSLDEVEEDAGIDDDEQDDDEDVKEVPKKKSHVKQKKSKKPPAKKDKTRKSKKNDQEIT
EELKDEIEEEDDSNEYQACNADTSPDNELCHCDVHEIDCSEITMESGDPYLRTLDVAIMK
KDFEPITAKFTKNKISRLQNDKVLPKFEKF

206	VSILDVSYN EIRFIDNDVFKPFTN		LRR1	2.8
230	LTKLYLSHN VLQTVKKDVFDAAKNT		LRR2	4
255	LHRLDLGYN RIKVVSDNSFDTLSK		LRR3	4
279	LKVLSLDGN PIKAWRKEMFKGLDS		LRR4	4
303	LEELSLDNC NIENLPADIFEYLPK		LRR5	3.4
327	LVKLSLREN PLEEIPAVVAHLKS		LRR6	4
350	LKDIDLSVT NLTEIRDHAFAGDSD		LRR7	2.8
374	LEEIILEKM PFLTVVRDCGFCGLPQ		LRR8	2.4
399	LKTLILNDN KYLQELHPNAFGYIKSQPGHK		LRR9	4

SHIFT -21!

408	KYLQELHPN AFGYIKSQPGHKSAA		LRR1	2
432	ITSLQIHNS NISTISEHMVDYDN		LRR2	2.2
455	LKTFQVGGN				LRR3	2.8
464	PWNCNCDTQFMLEEKFAFKQDSVAPKCTSPAGLNGRLLVTVRASDACED		CF1_short	3

SHIFT -18!

495	GLNGRLLVT VRASDACEDARFLGRSGRFSS		LRR1	2.2
525	VLGLALLVG FIAIGTYYMVSSGK		LRR2	2.4
548	LERLVRRVR KEPEVTYTNLQNAGEDFALET		LRR3	2

DFQPRPAEV

>C41C4.3

MRSTITFFESLETHMKFQ

19	IYFFLLISC VLAKKPVIFCDDAYGRYTAV		LRR1	2.2
48	AGNLDLSTF EFPIMSKISSANDASGSKM		LRR2	2.4
76	LHPCYSLSN TNNYKNQLFDIFSVGEFAPNI		LRR3	2

CERQCAHFVDVFRFECGDRRPPPSWLAGTVIGAKRKINCSDWAIPLTEKGKMDGISLTFT
YGTYQSSNGNDLSPPYTLDNKSIEYLDSMVIKGLHIKPVVDPTKKDTAMSEMESMFEQFV
ARHPEIIHIAFGNPITFFGDTFRNDSKLYAPRFIPTSKFWATLSKIPSLKSLSLSVIEIT
GKEDIPEGMTRNLRSIGFYDVKMNSIPNWIKTDQ

320	IQFLEFQSS ISDNVDMSALDELPT		LRR1	2.2
344	LEHFLLTES SLTSIKSPFLSKSSK		LRR2	2.8
368	LISLTLQCN SISSIAAGAFDHFTE		LRR3	4
392	LQFLNLAGN RIASLPSNLLLNLNN		LRR4	4
416	LIVLDLKAL DNSSNTIGTYNELSMQCQINQ		LRR5	3

KASTVPVILDKMPDVSKPEK

466	LVALEIRGQ ENIMKNDKSFLKNFKN		LRR1	2.4
491	LEILNLGNL GLLSAQNLSLETLCN		LRR2	3
515	LIDLNLVGN PLSDKDWISEDL			LRR3	4
536	FVNLNLQRL RMGQPSSMTSIPNSLVAFMRT		LRR4	2.4

ASQIMYSTPISLNSVDIYKRKIGYRISSATMFGYSLRNASCESNVKSAIENIRTMELQKM
ETQCQ

>T05A1.3

MSVLPLLHLFLISSLQIAPTATSSPSQFPCPTRCQCYADNEQNQQVHLICKWEQLNVTTL
QLARPDLVRTLTIKCPYHSPKVSTPPHSLFQGFRN

96	LDRLELDRC LIDTVPEGLFAGLGQ		LRR1	3.4
120	LYSLIVKNA KITDFPREIFAHVPN		LRR2	2.4
144	LMTLDLSGN RLRIEPYSLRSLQN		LRR3	4
167	LIHLDVSDN DIGFLTNTLISLTK		LRR4	3.4
190	LKVITMNNN KITNIDFRRFPEN			LRR5	2.8
212	LTDLSIRHN LVSTIHYVPASARN		LRR6	3.4
235	LKRLDLAGN RMEFVGGLTTGAVNVLPAE		LRR7	4
263	LKHVDLSNN KLTYLHDHAFEHLTN		LRR8	3.4
287	LILLDLKNN SLKEVKSSSFAGSKF		LRR9	4
311	QVKLFLSEN				LRR10	3
320	PLLCHCNHKWLMDSSAKNISIGDLQTIECLNILKPEKKMLLTLAHSRNQLL		CF1_short	3

CKYSNM

377	CEADCECCQKKECECRFECPPTCKCLRSADVSIVRTSQNI		NF	4

MVCDK

422	LRWDKLGKF PSPLTQLHMNQTDWKIFE		LRR1	2
449	TEKLKELSN LRALKITNSKMTSVE		LRR2	2
473	MEKLTDLAN LTHLEITSSAITSIPETITK		LRR3	2.4
502	LPSLTHLYL SDNPLDQLSSTSLNHLDG		LRR4	2
529	LKKIRLGGN FSRFACDCESPSDF		LRR5	3.4
552	QRWLMRRIN RAKIDDIDDLYCDLNGHGTVW		LRR6	2

MLEALPGTNESVCLDPTEETKQWMTFVENAQKGIMERVYTSTESTTKSEKTMMTTTLKKS
ILDTMDELEGVKSTEKISNKVISTTTVPTTTPRYRKKYKNYDNEPHKFVNVLIFLLFLCV
IILIISIGVTVYLKFWHPEELLMKRRKKERLRPEIQREEEPLQHLD

>ZK994.4

MNLYLLLLVIATSSWQFVAGLE

23	CPVECTCDKKGLVVDCSSSGLTRIPKNISRN		NF	4
54	VRSLVIRNN RIHKLKRSDLEGFNQ		LRR1	2.8
78	LETLVLTHN KIKIIEENVLDHLPE		LRR2	4
102	LKRLSLAHN ELVYIPPLCSDSRP		LRR3	4
125	LASLNLKRN HIQFIDEQVLRYFPD		LRR4	4
149	LTQLDFSHN RIQSLRTKLFDNLPA		LRR5	3.4
173	LSHAHLHSN PWHCDCRASKVKALLQKVKWE		LRR6	3.4

KKVYCTNPVELRHQALDEVCIR

>M88.6a

MRSLLSFVLLALARIAISEETKSCIDIEKGFKEEFNAHKQPVCICADNGIFSTVKGFTIE
CES

64	ASIASVSEN LASLNGTELGRLTIRDSTVN		LRR1	2.2
93	VLPQDLFEN VFAKQVKLERCG			LRR2	2.4
114	LSTLQPNSF QSLGGSAELLSLRENRIKKLE		LRR3	2

SHIFT -15!

129	AELLSLREN RIKKLEKGLFTGLKS		LRR1	3.4
153	LKTLDLAMN KIQEIDVGAFEELKK		LRR2	4
177	VEELLLNEN DIRVLKTGTFDGMKN		LRR3	3.4
201	LKKLTLQNC NLEIIQKGAFRGLNS		LRR4	3.4
225	LEQLILSNN NLENIDWTIFSALKN		LRR5	4
249	LRVLDLGSN KISNVEMKSFPK			LRR6	4
270	LEKLVLNNN TIDSMKSIKLKDLPS		LRR7	4
294	LVVALFDRN KIESIGDMDMFGLTRSDR		LRR8	2.8
321	IETLSLARN NLSQISPKAFQHTPN		LRR9	3.4
345	LITLLLQYN QIEELSSRKLTHFIFKKKLIF		LRR10	4

SHIFT -17!

358	LSSRKLTHF IFKKKLIFFPDSPSQVRTPF		LRR1	2
387	LASLKKLVT LQLSSNNLSVIRSDELPKS		LRR2	2.4
415	LSSLALDHN VISKIEARALEGME		LRR3	4
438	IKRLYLHSN KLNYLYQGTFDSFSPKS		LRR4	3.4
464	VEAVDVSLN				LRR5	2.2
473	PWVCVCNDPKEWLPRWLEASEEADVAEGALGCLAIPNCGQKEGSTVMPEEEEVYRSGWIT		CF1_short	3

VAATILTIVTIVIMVIIAMLYFKDARYQFPLRGRRSDSDLHKLIENDPLNIASDSILVVP
AMPKRNTGPKKTVRFQNF

>Y71F9B.8

MRPPVLILLVLVSGVIS

18	CQSGCKCPTKTTAVCKGSSLRSIPILLDPR		NF	4
48	TTVLDLSNN RISRLSADELSLYPN		LRR1	3
72	LEQLILHNN SITHLSADVFSTLPS		LRR2	4
96	LRVLDLSSN SLLSLPNEVFSKLKN		LRR3	4
120	LKTLIISSN DVQLGPECFAGLSQ		LRR4	3.4
143	LQTLSIADN RLSFLPPSVLKPLSG		LRR5	3.4
167	LRNLDLSAN KLLSMPASVMNNLGG		LRR6	4
191	LETLKLKQN LLSSLETGMFLSQKE		LRR7	4
215	LKHLDVSEN LIGDIEEGALYGLEK		LRR8	3.4
239	LETLNLTNN QLVRLPGNTWSLPA		LRR9	4
262	LKTLDLSSN LFVSLETASFDGLPA		LRR10	4
286	LQYLNISHS RNLKTIQMATFVQLSS		LRR11	2.8
311	LHWLSISSS ALTHIHPSAFNPIPP		LRR12	2.8
335	LSHLDLSNN ELRYVAPGMLQWPN		LRR13	4
358	IRNLHLANN				LRR14	3.4
367	DWHCSCDLRVSNLNPRDDAKCSGPENLAGAPINELSSCSILGGLLIPFLLVLFILLLALV		CF1_short	3

SHIFT -9!

418	LFILLLALV ILALACKKSKPVSLKNRAFY		LRR1	3
447	NDQLIAALN SHKEYSFDCHSPYTMSSEDSR		LRR2	2.4

DSAYESPTSALMPRRPPPSCPPPPRLLTLPRAGPTHVAPPMVPVPNFRNSNDPYLIPKSQ
VPITRL

>Y75B8A.5

MILLLGTTYVQKFNIFIWKLLILFHFLKYSNALRDTCPMGCQCDEDKQQVTCEGQQVVTL
PDRLPSGYESLVIRNSSVRTIEKNSFRKMEK

92	LMQIEFENN PNLGTIEKLAFKG			LRR1	2.8
114	LKKIRLIKF TTCPGLTELQKNAFSGIQNQM		LRR2	2.4

GLKIIFEKTPIHRIDGHTFRNAQNIRELTIS

175	GEELALSRH CFANINQLDFLTVSGVV		LRR1	2.4
201	LIEPEIFTN STRFHVVHFKNSQFDIPPSTF		LRR2	2.4

STLSHTSHLLIEHSKVPSIAPDAFSGLTT

260	IQVIELHAC QLGTISARAFANVEN		LRR1	2.2
284	LGELKILRN TIGDLDTSESIMSRALKTRIE		LRR2	3.4

ENTLECSCGMKWMTSVEEMSDINFCSTTASFRSIRSFIKAKCLNPSQEISRKTNHLPSIS
SFSSSSIVN

383	FYFLSLVWY FSKTDINNLQNRF			LRR1	2.4
405	DKNLMPCRN TFALLPWQNLHQISES		LRR2	2
430	LIDFTVKFS RKRARSRSFA			LRR3	2.2


>C44H4.2

M

2	LVLESLLKP ASVVRFTVELFWTVQI		LRR1	2
27	VLPVSLTMN QAELVELPPNFFSGLF		LRR2	2.8
52	IRRLDLSQN KIKKIDDAAFAGINPV		LRR3	3.4
77	LEEVVLNHN LIEKVPAAALAGLPN		LRR4	3.4
101	LLRLDLSNN SIVEIQEQEIFPNLNK		LRR5	4
126	LYDINLGSN KIFSIHTSTFQNVKNS		LRR6	3.4
151	IQTINLGHN NMTAVPSSAIRGLKQ		LRR7	2.8
175	LQSLHLHKN RIEQLDALNFLNLPV		LRR8	4
199	LNLLNLAGN QIHELNRQAFLNVPS		LRR9	4
223	LRYLYLSGN KITKLTAYQFQTFEQ		LRR10	4
247	LEMLDLTNN EIGAIPANSLSGLKQ		LRR11	4
271	LRQLYLAHN KISNISSNAFTNSS		LRR12	4
294	IVVLVLSSN ELKTLTAGIISGLPN		LRR13	3.4
318	LQQVSFRDN QIKTINRNAFYDAAS		LRR14	2.8
342	LVMLDLAKN QLTEIAPTTFLAQLN		LRR15	4
366	LLLVDLSEN KLPKTPYSAFNSR			LRR16	3.4
388	VGTVLLKEN PLVCTENLHMLQQGTGVYVRD		LRR17	2.8

SPDIICGRKPTPKPEPVLVPIVTDSLISTQRPALVQ

454	IPKMQIHRN VHTTTGDQAPQIPSGA		LRR1	2.2
479	FQQIDLGKS RSLPRGHSRFILDKPSTREQS		LRR2	2.2

VEPTEELTPIQPIILPSREDEIRQSSMEAGTSQESVEATSQKIPSTTDIIDRPNVVLPFP

569	VPFLKRGPN LSESKKVESTDMPSTSQVFHT		LRR1	2.4
599	LPPSILIEP GSTPKVAQPSTEANIKSEH		LRR2	2
627	IDEFALASS NSNEPTLQPRLEKSFFTTTII		LRR3	2.2

FICVGTAVIVLVVVIAGLCISKHRQLQFENTYSDSSAARTSEYISTQYRQNSLRGTGGRV
GRFEESPAWIYNPGSSYCNYYK

>C02C6.3

MPQRNNQIHSYNIPRRSGLISITYLLISSTVWVVQAGNRQEYQYSDDAIENKLICAGKLA
DYAACQCDPSAGEYSCINAQFVDA

85	NVFLDIATN YKYIKSVTFHGNNFQD		LRR1	2.4
110	LTSTPLFGA DSQTSLIKLNLSA			LRR2	2
132	NYIVNLNSN ALRNMPNLQVLDISNNEIVFR		LRR3	2.4

SHIFT -14!

148	LQVLDISNN EIVFRPRDVDFLKHTPH		LRR1	3.4
174	LTQLYMRRA FTVTINRTQQFELMLEMFRQA		LRR2	2.4

K

205	LEYLQVLDL SYNFIHNVPFEIACPFPS		LRR1	2.4
232	LHTLDLRQN FLKNFIVNETCIKE		LRR2	4
255	VNTINLSRN QFYVIPKDFRALADKA		LRR3	2.8
280	QPETFLLRN				LRR4	2
289	QFYCDCNSKEFITWLRSTKSVREKNSLVCDRASPKMYVGARIAEVPLNKLT		CF1_short	3

CDEPLFTSMSSFKFNFYSMIIAFIVSKMMF

>Y39A1A.7

MTSITSAFLLLLLFVAVACRS

22	VPCLDGCTC ELSEHDPVIRCDGVG		LRR1	2.2
46	LNRFPLPHS SPLRGFHFLALTCNDIDTIPA		LRR2	2.8

SHIFT -16!

60	FHFLALTCN DIDTIPAISMIKASFPD		LRR1	3.4
86	LQGIDVQGN TRLNCSDLVHLHKD		LRR2	2.8
109	IPVLSDCDN EKPLQCDNLDKNCDWKCRT		LRR3	2.4
137	LTKLKEMWA QFKDLVNRKAKEWQAEETIEA		LRR4	2

AKSWFSAQFKKFKIAIGELSD

>Y37E3.13

MREAA

6	LLLLFLPII SCSNAPKTCKSFWISNRPSQD		LRR1	3
36	CASLTLQEP PQLLPNVLSLSLSNNSIFRIT		LRR2	2

SHIFT -19!

47	LLPNVLSLS LSNNSIFRITTFPSEYRR		LRR1	2.4
74	LQSLRLDQC QLEKLDFDALSVFEQ		LRR2	3.4
98	LRELDVSRN SLSKLIIPRNLAS			LRR3	3.4
120	LRVLNLAFN AFTYVPDMSHLES			LRR4	4
142	LRLVDLSHN RLISVRPRMLPFN			LRR5	3.4
164	LEVVRLAAN RFQHLSPWPFLHK			LRR6	3.4
186	LQELDVTFN				LRR7	3.4
195	DLECDCSLWHFVTWAEKLALFDRRFQTVCGPTVVTSSPESAVVSLDDAHVM		CF1_short	3

SHIFT -60!

186	LQELDVTFN DLECDCSLWHFVTW		LRR7	3.4
209	AEKLALFDR RFQTVCGPTVVTSSPESAVVS		LRR8	2.4
239	LDDAHVMCC TALATPSPQLYWQFNGKNISS		LRR9	2.2

GLSQKHLSESGKLEFCLEIPKVRLKDMGKYKCVASLAGLNSSKEFHVERDKIPIVLNSAE
GIMIYCQFTICTFIGVCCVISCCVLRTGGGGRGKTRPNREYLHTKVLEIPHDSCEYCEVD
DDDVDDTCEDWGDAETAAVRQYLQWRQMQMQQEQMQKCPMLPKIRHESLCRLSNESGSSN
RSIPRAPLARFDEPSCVSFELTHL

>K09C8.5

M

2	LLEFLLLIG ISLSTA		LRR1	2.4
17	CPSECRCAGLDVHCEGKNLTAIPGHIPIA		NF	4

SHIFT -20!

26	LDVHCEGKN LTAIPGHIPIATTN		LRR2	2
49	LYFSNNLLN SLSKSNFQALPN			LRR3	2
70	LQYLDLSNN SIRDIEETLLDSFPG		LRR4	4
94	LKYLDLSWN KIRYVPKLSTAPNA		LRR5	4
117	LVSLNLVHN EISRLDNDLVSHSPY		LRR6	4
141	MQTFLIQRN RIQSLPHDFFNSRMVPT		LRR7	2.2
167	LKTVKMAGN				LRR8	2.8
176	PWSCDCRMVNVKQFADSLFAHSNQNIFIVGKCFFPKGLRNYVFRNLSIENLECE		CF1_short	3

SHIFT -63!

167	LKTVKMAGN PWSCDCRMVNVKQF		LRR8	2.8
190	ADSLFAHSN QNIFIVGKCFFPKGLRNYVFR		LRR9	2.8

NLSIENLECEKPEYSKTDDGMFKMSCPNNEMEGYH

255	YDSIFLENN KEARHTAHFARDKDGSL		LRR1	2.4
281	LSNGQFTRN YQCAFYRQKQSIHMQKKMQAS		LRR2	2.8

SSTEPPITTTTMEPMTTSTMDSMDTTESVVTMTTMPEIDTKIVFEHKQLDTTSRD

366	GETLELKCE ASGEPTPTITWLFEKQKLTE		LRR1	2.4
395	SRKHKLTKN GSVLKIFPFLNTDIGQYECVA		LRR2	2

SHIFT -18!

407	LKIFPFLNT DIGQYECVASNGEESKSH		LRR1	2.2
434	IFSVSLKES EQPVIIDAPMDTNATI		LRR2	2.2
459	GQQVTLRCN AKGFPVPDVVWLFEGIRIPRR		LRR3	2.8

NTRYTISDNNIELTIEKVTRHDSGVFTCQAVNSVGSAVATANLLVGAELTEKVDKLLDDS
TIEKIAKEAKQKVEKALSSTKDQQRMDKIESPNDLSKLFKFAIN

593	LKKVDLGKA REIYEESIRLVQMHIDNGLA		LRR1	2.4
622	FESAMISPN VSYEAVLPVSYVQTLMEKSGC		LRR2	2.2

QTGQFAESCEDHCFFSKYRSYDGQCNNHEHPWWGVSEMAFMRLLPPRYENGFNTPVGWEK
GKRYNGYEVPNARKVSRVLIGTDETTPHSHLSAMTMQWGQFIDHDLTLTAPALTRHSYKE
GAFCNRTCENADPCFNIQLEADDPKLHTGLYQKHPCMEFERNGAACGSGETSPIFQRVTY

832	RDQLNLLTS YLDASGIYGNSE			LRR1	2.4
853	EQALELRDL YSDHGLLRFDIVSGANKPYMP		LRR2	2

FEKDSDMDCRRNFSRENPIKC

904	FLAGDVRAN EQLGLMSMHTIFLREHNR		LRR1	2.2
931	IASRLLEVN ENWDGETIFQETRK		LRR2	2.4
954	LIGAMLQHI TYNAWLPKILGKATYNTIIGE		LRR3	2.4

YKGYNPDVNPTIANEFATAA

1004	LRFAHTLIN THLFRFDKDFKETK		LRR1	2.4
1027	QGHLPLHNA FFAPERLVSEGGVDPL		LRR2	2
1052	LRGLFAAPI KMPRPDQVLNKELTEKLFNRF		LRR3	2.4

H

1083	EVALDLAAL NIQRGRDHGLPSWTEYRK		LRR1	2
1110	FCNLTVPKT WSDMKNIVQNDTVISK		LRR2	2.2
1135	LQSLYGVTE NIDLWVGGVTEKRTADA		LRR3	2.4
1161	LMGPTLACI IADQFKRLRDGDRFWYENEEM		LRR4	2

FSKAQLRQIKKVTLSKIICTNGDDIDRIQRDIFVYHGNSTQFYEPCESLPEINLNMWTTC
CDAMCSSSSTLARNAIGGDEKAKRRKRRHHHSKKSCHDKGKRRKSGDRWNHSNDICVECM
CHDGEVWCKTNNFCKSQV

>C44H4.1

MRLLLFNLLIAGWVYSQ

18	CPTLQLQEP CTCTSTRYEAVSINCDGGSSL		LRR1	2
48	DAVLESLSN SPQAIDSLTISNTPIEKMPGY		LRR2	2

SHIFT -17!

61	IDSLTISNT PIEKMPGYAFQGFQ		LRR1	2.2
84	IKKLFLRNN GLRSFHPNTFTGNLENS		LRR2	3.4
110	LEELEIRGN YIDGIPQSGVSILKQ		LRR3	3.4
134	LKILSLPDN LIEYVQDNAFLSYHSRDS		LRR4	4
161	LLKLDLSAN NLTAIHPTGLLGLEN		LRR5	4
185	LSQLSLDKN LLSEIPSQALENIPS		LRR6	4
209	LEDLSLGVN RIHTISRNSLPLPN		LRR7	4
232	LKSLSLEVN QIRLIPSDSFSETPL		LRR8	4
256	LSYLYLGNN LLTSIDASMFLHIGG		LRR9	4
280	LKVLSMSNN KDITSIQANGKLS			LRR10	3.4
302	VQQFKLCIC ILAFQHAPSLIRLELFDCSIS		LRR11	2.2

SHIFT -12!

320	LIRLELFDC SISRIEPKSLQKVQH		LRR1	3.4
344	IQVILLSRN QITQINAVDDFAFSQLPM		LRR2	2.8
371	LTSLDLSSN RLESLPSNVIYDSLMQKKTSP		LRR3	4

SHIFT -25!

376	LSSNRLESL PSNVIYDSLMQKKTSPV		LRR1	2
402	QRKLSIQNN PWRCDKDLMWLRKWLRDNGDV		LRR2	2.4

SHIFT -12!

420	WLRKWLRDN GDVTTAASNSPPAKCWTPSN		LRR1	2.4
449	LSGLDLRQT DSKLPHKTTKPPVDQFQYKNQ		LRR2	3.4

QVTNSNGTSQLGYQNPHTE

498	VNGLALVGL ILGIVLVVFVSCII		LRR1	2.4
521	LGYLMRFIF LSYEAKTKPNVFGSTISSSGC		LRR2	2

IRNMYGGDGTLVSEDHGHPSSANNNVYLNRPRHWWF

>C56E6.6

MK

3	LTQLDLSHN NLSVIPTWALTYLHS		LRR1	4
27	LQILHLENN RIDVLRSNTFDETQLNN		LRR2	4
53	LQFLYLDNN QLRIIPNLAFNHLR		LRR3	4
76	LVVLMLANN RITEIQKMSLPQT			LRR4	4
98	LKFLVLRNN LLTQIPYVALNDLKT		LRR5	4
122	LQSIDLEGN NITHLMDTNEVTFES		LRR6	3.4
146	EMKVILRNN KVRRLDKHSFRSFRK		LRR7	2.4
170	IRELDISYN QIQTVEDSSFETVGH		LRR8	2.8
194	MQSLDLSYN RIAYLPRGMLKNFAKT		LRR9	3.4
219	LKTLKLAEN MIHATPEALRDLRN		LRR10	4
242	LTHLNLNGN KLNRIDGDVLKGCTDT		LRR11	4
267	LVELFIANN YLEHIPHGVLSGMKQ		LRR12	3.4
291	LEHLDISKN KIMSLKKPTSLLSITKEETST		LRR13	3.4
321	VRRLNLAGN RINNMSDYLIFEHMPL		LRR14	3.4
346	LTYVDVSFN RIRFISPRVFEKLKN		LRR15	2.8
370	LESLFLQNN QLAHFPSLFRLDK			LRR16	4
392	LRHLMLDNN QIQKIDNFSLADLPK		LRR17	4
416	LQHLSLAGN QLDIITENMFGSSSSSE		LRR18	4
442	LKSLNLAHN KIHSISSRSFSDLDN		LRR19	4
466	LQQLRLSHN NIRTITSMTFSNLRN		LRR20	4
490	LRYLDLSHN RIIKILPSALYQLPA		LRR21	4
514	LDVLHLDHN NLNEIDRDAFRSFSD		LRR22	4
538	LQSLKLSHN AFRRFSCEFLGSISQ		LRR23	4
562	VHQLDLSSN QINEIDIFCIARG			LRR24	3.4
584	IRKLSLASN SVEKINRKLLQDATE		LRR25	3.4
608	LTSIDISHN GIIDVDSDAFCECRK		LRR26	2.8
632	LSHIKLSHN YIRNLWKGTFQYQES		LRR27	3.4
656	LHTLDISFN DILFLHQGTFGK			LRR28	3.4
677	NNILQLHVN NNKLSRIPLEALSSTMAS		LRR29	3
704	LHLLDLAHN NIKIVDSSQLTSFGN		LRR30	4
728	LSILSFANN KVDSIEDGAFENLLS		LRR31	3.4
752	LKILDLSNN PVTSWSPTAFRD			LRR32	4
773	LSHSISSIN MANTGLFSMPKFSHRS		LRR33	2
798	IQSLNISCN KIYELSEKDLAPLTK		LRR34	2.8
822	VVALDISHN NLKQISSMAFEPLIH		LRR35	2.8
846	LKQLNVSAN PITHLTNEHIQQ			LRR36	3.4
867	LYKLYNIPD MARPYQISSILSN			LRR37	2
889	LPPLHTIYV DIKESALDRQFYTADTRL		LRR38	2
916	LRHLVVAGR NLTKIEVGAFATIRGFRVRIE		LRR39	2.4

IHNSSIEEFPSRIFDTLTGISILSLS

972	LTDNKLTTF NPFQSTVAPAVNQHGTI		LRR1	2
998	LHSLELKNN				LRR2	4
1007	PIKCDCQFKWMDDFIRVTKFLSDHHISHDFDKVECADAQSSNLESFASAANELFSYK		CF1_short	3

SHIFT -15!

1049	LESFASAAN ELFSYKTKMTLLSKSEDFG		LRR1	2.4
1077	LECAVKYSN PLKPLIYVLMLVCLSYL		LRR2	2.4


>K07A12.2

MRWLTLIA

9	VAHLIAFLS SAEIT		LRR1	2.2
23	CPRIPEKCDCKISKSMIILSCNGEDVKTIAQTVGTS		NF	4

SHIFT -23!

36	KSMIILSCN GEDVKTIAQTVGTSQIDE		LRR2	2.4
63	LHILNGTDV KIESLPFNGLRTIAILNSTLQ		LRR3	2.4

SHIFT -17!

76	LPFNGLRTI AILNSTLQSFSPTAWRHVEAT		LRR1	2
106	IEHITINGN ELKTVPVFGNLST			LRR2	2.2
128	LMSMNLNSN QISSIPDKAFNGLSA		LRR3	3.4
152	LTQLRLENN AICDFPPKSLDAVKAS		LRR4	4
177	LVLLDVSGN CLDAIPAQILRNAAN		LRR5	3.4
201	LMYLDLGSN NISEINNFELMNLPF		LRR6	4
225	LRELRVQNN TLRRIHPMAFMNVPQ		LRR7	3.4
249	LQYLYLQDN IISTLDGNRLQGFKN		LRR8	4
273	LEVLDVSNN ALYALPSLKDLPN			LRR9	3.4
295	LKQVRVDGN LITKIETLAFSNNPN		LRR10	2.8
319	LQLISVQNN NIVQISRNSFESLDK		LRR11	2.8
343	LVVLLVGNN SLAKIERGMFDGMKN		LRR12	3.4
367	LQQLSIRNN TLTALDASSFAQLAH		LRR13	3.4
391	LTTLDLGHN KIHDIEEGTFDKLSK		LRR14	4
415	LFWLDLSNN KISGFKTSVFKKK			LRR15	4
437	ISNILLDGN QLICDESFNEFLTYLIANKVR		LRR16	2.8

TFLPFQQEIMCHGPEKYAGVR

488	LKDLMMKKA NETLSEGSRLLGVPQGSNQHS		LRR1	2.4
518	LLSSFLPSL GPLGTLNGAGGAAIPLVNTLT		LRR2	2

NTIPALRSIPGFGGNIPVGTGASSVPNKNLNDAIEGFTGPLVRFATGGQPVASDIEQLIR
SIPNMVVNVPGFGDIDLSKMDPTMIQYVLNGGQIPGIDKATLDKIVKQTMNKMHTAAAAN
LAGNPVEGQEKVLPPLDKLPSGLVTQVMSGEPLPGLNENQTKIIMEYYTHQMPGMDGIPA
RPVESQGNTTANNMFNPAMFDL

750	LKMLPPGYN LSKIPMEVIAAVTRGEVPDMR		LRR1	3
780	LLPEDLLEH FKQHTTSLTSMFAGATAKNIS		LRR2	2

IEEI

814	LEKLPVFVR PELSTFVPYDINELTS		LRR1	2.4
839	EMVLEQEQN ERHRNIRIITAIALAFVGAVT		LRR2	2
869	VVVIIFFVN YTKKQRRLRKSLVYRSSPSSS		LRR3	2.2

GSSGQNAANESGRSSAAPSPIRPP

923	LMNIPKTPN NRTMESTFGQPQ			LRR1	2.4
944	LCSTLLENP QAVSHRSRH			LRR2	2


>T01G9.3

MISYILLLFLSFLVLCSIALPSS

24	CPNLCECDQNDSSWSVYCRKAIINDTIYAEI		NF	4
55	LNQLPLTLR SLHIQPPSNRIG			LRR1	3
76	SNKLRWNDN INRFAQLRVLRLINCQIPAMS		LRR2	2.4

SHIFT -15!

91	LRVLRLINC QIPAMSRSIRLPS			LRR1	3.4
113	LEVLDLHSN NIEHATMSNFGGMPK		LRR2	4
137	LRVLDLSSN HLNILPTGVFTYLRA		LRR3	4
161	LRSLSLSNN TISDLSTNLLRGLNS		LRR4	4
185	LRVLRLDRN PIPIEHINELFTDVSQ		LRR5	4
210	LDELYLNHC NLSSIYSLALDRIPQ		LRR6	3.4
234	LRQLGIGGN NLKMVPTKELRSLPQ		LRR7	3.4
258	LSVLDLSHN SIQEITACAFCNTN		LRR8	4
281	ISKLDLSHN LLGISKDSPFNEDAFRTMP		LRR9	3.4
309	LRHLDLSFN HMNDFDSKWLGWAQEE		LRR10	4
334	LTSIALSGN FLKNFEESWTYTLKS		LRR11	3.4
358	LIHLELAYN HIKFIPVQLPSRYYH		LRR12	4
382	LISLNISGN ELTYLPDNINTLLPN		LRR13	3.4
406	VKTFDITAN RFHTFSHTDLAFLNN		LRR14	2.2
430	VEQVYVDGN				LRR15	2.2
439	PWDCSCAIQGLQVHMRDRYAMRHILNYDNVRCATPSLVEGHSVLAITDVNDCAV		CF1_short	3

LFGARYGLTQTSEMLILLAGVLLFAALLLMILGCIYFLRERQYKGSYVTREHSRTPLTMA
NTHSCSSSTNDTHGPLSPPFDPFLVSTETFKATPPLIPPAPPKPGSSYFGI

>T21D12.9

MIVYSLL

8	LLILYDFLP KVVTFDTVCIGLCHCVGNVVD		LRR1	2
38	CSSLDLSEI PTTIPNNTRILLLSDNEIESI		LRR2	2

SHIFT -14!

54	TRILLLSDN EIESIDKSRLKGFYF		LRR1	3
78	LQTLDISNN IIRHIDFEFFYNLPN		LRR2	3.4
102	LKILNIRKN RLARIPRGSHELGH		LRR3	3.4
125	LEKLDLRSN LISTVTSEELSYLAA		LRR4	4
149	VRSVDLSRN LISYLPKPTTSAKVN		LRR5	2.8
173	IEKLDLASN SITDIGTDHFSSFNT		LRR6	3.4
197	LVTLKLARN HITTLNQFSFSRLRK		LRR7	4
221	LESLDLTRN MIREVRFLAFNQLPS		LRR8	4
245	LQNVSLARN DVYRLDDGMFYACEG		LRR9	3.4
269	LKHLNLSTN RVQAVTEGWMFGLTS		LRR10	4
293	LEVLDLSYN QIQSFHISSWSHTPK		LRR11	4
317	LKWLSLHSN RIQSLPSGSFRVLRQ		LRR12	4
341	LEELILSAN SIDSLHKFALVGMSS		LRR13	4
365	LHKLDLSSN TLAVCVEDGAVLYNTSMPF		LRR14	4
393	LRSLRFTNN QLRVIPKRAFERFPA		LRR15	3.4
417	LEELDLTDN PIATIHPEAFEP			LRR16	4
438	LELKRLVMN SSSILCDCQISWLASW		LRR17	3
463	IYRLKLDKS SIIAKCSYPPPLADLYVVAID		LRR18	2.8

TANLTCHNDSPRAKIVRQPVEVSTLIGEKARFTCNVYGASPLSIEWRVMENGQPRVLVQ

552	DSATFLSIN RTAVVNGTFDERELAAAELL		LRR1	2
581	LDNVAMTDN SEYQCVARNRFGSDFSTHVKL		LRR2	2.8

QVYQAPKFTYTPEDMPLLVGQTAKFLCAATGTPRPEIKWAFEQIPFPAAEARRLYVTPND
DHIYIMNVTKEDQGAYTCHATNVAGQTQASANLIVFENFFHYPESPDLSPMLIKKRDALK
IDCSCDLISSRQRMVWKRQQQVILFLKKAKFSDNDQILTLTQTTFSDSGEYSCELWVDDT
ILMRKITMVKVVNEDEFVSSEATLTQRVQRIR

823	AMASKLVDN IKSTGNGIYLMASCSAFG		LRR1	2.4
850	LMILGVITS ICICIAKCSCNQNHVFNVLPV		LRR2	2.8

QV

>Y76A2B.2

MVFRSSIIPLIRLLLLPYIISGLTTCPRQCICHEHSIACSCETSEKPELIISSLGSTYIT
SLVVHTCDKVTVQNGSFAGVVLVERLSFIAIGRLYFEPHAFKDILQSPRQLVIDECTISS
LAPVSFAGLSHIDHLWFRNSRIDVIATEAFHYLTNIDYIYFHKTKIGRIERKAFSKMYQI
DHLYFKDSIEIATIESEAFSGSQVDEMIFDGVTVETAHDTFLLNVESDMAILKNCSIYLI
PRKEDDMVVFDEKQKIIERCLIESSSFNIMSPYKLGCNVLEVTSSVIQRIGPVSRDIDTP
VFRPEPFIISS

312	LNSVIFSNC SIGSVDSFAFTNYT		LRR1	2.2
335	LSILSFNHS RIGEMKTRTIEKSKISNFEFG		LRR2	2.8
365	GSTVKLCNS SSIDKSKIQNFEVSGAEIDRI		LRR3	2.2

NSDWIQNSM

404	IRKLKIRSS KIGKSEKNIFKDSV		LRR1	2.2
427	FEEVEIDGN RILMMDSETLHGDNQ		LRR2	2.2
451	ISTLRITGN DITTSRPSPSFLSSSPQSH		LRR3	2.8
479	PSNFILANN TFDCLPNDCSTNSFLLNSPKH		LRR4	2.4

SPLLYKISSNRCRPPLENPCVEPRSISLEDHGITCRISSLVADCACLTEKSSIPSKFPLN
YNISVVILGDCEHLTIDQKLADFTQIYIFRTTKLIVQRLPKSLKVLKIFHSTVTLEPSSF
LNFPQDWEVSNSKVEH

645	LGLSNLNIS SLHLKFSRISHISAAKNSK		LRR1	2.4
673	IKNLHIENC HLESTQHLFEISQTLQMHNSI		LRR2	2.2

IFSSPRGLGTISSAQLQNNTLLECCNLIELDSRCDLHFFGSRCVEEDDVSTNHLPITSGS
NITSFDVYIAIYLFVSRILL

>C50H2.1

MTCST

6	YRSIQLFSN LLIFILIILYLQTIEC		LRR1	2.4
31	QNALSLAQT KHSCMALKAEDGEGCTCKQTK		LRR2	2.4

RYGQPECCCMGLIVSQLPTNLTAD

85	VGYLYLHNT SISVITPNFFDKFPS		LRR1	2.8
109	IRELEIDNS AHLEHIDGSSLSVLSK		LRR2	2.2
134	LRKLSVVKC PNLREISGKLLVNNTR		LRR3	2.8
159	IQNVILKNN GLATMPSLRMTDAHHVL		LRR4	2.8
185	LDRIDLSGN KIKFISDSKVRNVK		LRR5	3.4
208	ARTVVLSEN KLIEISGYAFTESQ		LRR6	2.8
231	FLKLKLNNN PDLRSLSVDAFKNMAG		LRR7	3.4
256	LQTLDLSHT SIDTLPINGLKK			LRR8	3.4
277	LKTLILNDV PTLKSLPSVLSFTDLETAHFT		LRR9	3

SHIFT -19!

288	LKSLPSVLS FTDLETAHFTYPHHCCLFKY		LRR1	2.4
317	VDDVTMNDN GKYQRNAKEIHKRICDKREQQ		LRR2	2.2

KVARRRKRETSGIDFLDMLLKEWTDNSTYTGPDDADDDELPPFVEIGAEPCQSIGEEVQK
YYSNITCYPQ

417	PDALNPCEN IVGYPFLRIAVWV			LRR1	2
439	VCLAAIVGN IIVWALLGIVYEKRMRMHYLY		LRR2	2.2

MINMSVADMVTGIY

483	LAVLAIADA KMSDEYYRHAVWWQTGWGCRA		LRR1	2.4
513	AGFLAVFAS ELGIISMFLIAFEMSYNTRQS		LRR2	2.2

FRGRRLSPKVGVLLMIGGWLFAIIMAILPWFDVSSYSES

582	SVCLPLRAA TIFDKSYLIFGLSFNF		LRR1	2
607	LAFAAMALS YGFIVKMLKENETREEDRALI		LRR2	2.2

SHIFT -19!

618	FIVKMLKEN ETREEDRALITKMTV		LRR1	2.4
642	LVVTDLICW FPTLFFGFTATIG			LRR2	2
664	FPLLSLSSA KFVLVFFFPINAFANPFLYVF		LRR3	2.4

FTEVIQHRVRSKTLPVIRRITTGP

718	LNAASSLSN FYHSQPPGAHRRSRDEPTSPS		LRR1	2.4
748	GMHLAVTQT TSLNSTPRGSDVSRASDGMLF		LRR2	2.2

DYDRRASASPRVSFDIPISPTTPRSDRKNRISLLKRIVSSIPEVSDLSEHSSESHHEHVP
HPRRKLRSSL

848	NRILALGRR QEGSADSGRGSIASSAGSRDQ		LRR1	2
878	NERVSLTSN ASSILSPLLSPTN			LRR2	2.4
900	SNILILPPE PSKNRRKSTPAIPLLVVSDCS		LRR3	2


>C07F11.1

MRRKMKLFLFL

12	LLVINICRS AAANGDECPKFCKCAPDPVQP		LRR1	2.2
42	TSKLLLCDY SSKNTTITPIASSNYDQVANI		LRR2	2

SHIFT -26!

46	LLCDYSSKN TTITPIASSNYDQVANIRS		LRR1	2
74	LFISCDNNN FQFPDAYFKSLTA			LRR2	2
96	LHHLRIVGC ETTHFSVKLFEDLAA		LRR3	2.8
120	LRRLELDQI STASTSFEMTEDVLMPLAR		LRR4	3
148	LEKFSLTRS RNIELPQRLLCSLPH		LRR5	2.8
172	LQVLNISSN ELPSLRREESCVAQQ		LRR6	3.4
196	LLIVDLSRN RLTNIEQFLRGIPA		LRR7	3.4
219	IRQISVAYN SIAELDLSLATPF			LRR8	2.2
241	LQQLDAEAN RIVDLTSLPGTVVHVN		LRR9	3.4
266	LAGNALKRV PDAVAELASLVALNVSRNEIE		LRR10	2

SHIFT -15!

281	LASLVALNV SRNEIEAGNSSVFSSPE		LRR1	2.4
307	LEMLDASYN KLDSLPVEWLQKCEKR		LRR2	3.4
332	IAHLHLEHN SIEQLTGGVLANATN		LRR3	3.4
356	LQTLDLSSN QLRVFRDEVLPENSK		LRR4	4
380	IGNLRLSNN SLELLEPSSLSGLK		LRR5	3.4
403	LESLDLSHN KLTEVPAAIGKVEQ		LRR6	4
426	LKKVDLSHN RIAKVYQYVLNKIKQ		LRR7	3.4
450	LHTVDLSNN QLQSIGPYIFSDSSE		LRR8	3.4
474	LHSLDVSNN EISLLFKDAFARCPK		LRR9	3.4
498	LRKISMKMN KIKSLDEGLTEASG		LRR10	2.8
521	LRRLDVSHN EILVLKWSALPEN			LRR11	3.4
543	LEILNADNN DINLLTAASMSPSTAN		LRR12	3.4
568	LKSVSLSNN GITIMNADQIPNS			LRR13	3.4
590	LESLDVSNN RLAKLGKTALAAKSQ		LRR14	3.4
614	LRRLNLKGN LLTVVATESMKVVEA		LRR15	4
638	VHPLKVEIS ENPLICDCQMGWMIGGAKPKV		LRR16	2.2

LIQDSETASCSHAVDGHQIQIQSLSKKDLLCPYKSV

704	CEPECICCQYGNCDCKSVCPANCRCFRDDQFNINIVRCHG		NF	4

NSSMVPKREFV

755	VSELPVSAT EIILSGVTLPQLRTHSFIGR		LRR1	2.2
784	LRLQRLHIN GTGLRSIQPKAFHTLPA		LRR2	3
810	LKTLDLSDN SLISLSGEEFLKCGE		LRR3	4
834	VSQLFLNGN RFSTLSRGIFEKLPN		LRR4	3.4
858	LKYLTLHNN SLEDIPQVLHSTA			LRR5	4
880	LSKISLSSN PLRCDCSGGSQQHLHHRRDPK		LRR6	3.4

AHPFWEHNAAEWFSLHRHLVVDFPKVECWENVTKAFLTNDTTVLSAYPPNMGNDVFVMPI
EEFLRDYNSTICVPFSSGFFGQDPQNSILFVIITISIAVLLCVLVILAISFIRKSHDAIN
QRRYKASSLNCSTSAGSSPLPVPLLSYHAFVSYSKKDEKMVIDQLCRPLEDEDYQ

1085	LCLLHRDGP TYCSNLHAISDELIAQMDS		LRR1	2
1113	SQCLILVLT KHFLENEWKTLQIKTSHQLFA		LRR2	2.4

KNRAKRVIAVLGDGVDAN

1161	LLDDELGQI LRKHTRIEMRSH			LRR1	2
1182	LFWTLLHSS LPSRLPLPSNSGDDSSQLYSD		LRR2	2.4

IYGIVPSDVV
```
